# Computational Comparison of Chemical and Isotopic Approaches to Control the Photoisomerization Dynamics of Light-Driven Molecular Motors

**DOI:** 10.1021/acs.joc.1c00063

**Published:** 2021-03-30

**Authors:** Jun Wang, Baswanth Oruganti, Bo Durbeej

**Affiliations:** † Institut de Química Computacional i Catàlisi, Facultat de Ciències, Universitat de Girona, ES-17003 Girona, Spain; ‡ Department of Chemistry and Biomedical Sciences, Faculty of Health and Life Sciences, Linnaeus University, SE-45041 Kalmar, Sweden; § Division of Theoretical Chemistry, IFM, Linköping University, SE-58183 Linköping, Sweden

## Abstract

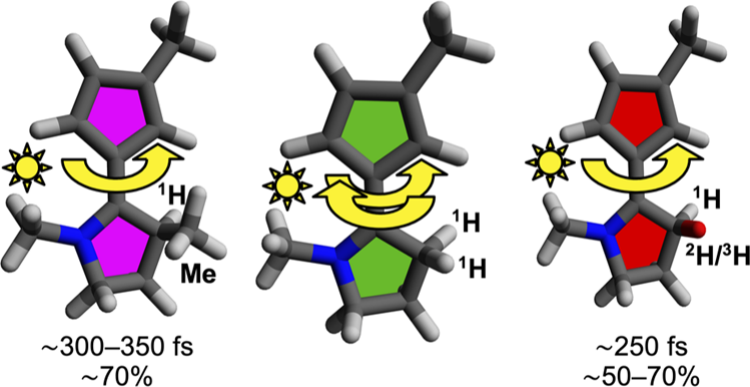

Synthetic molecular motors driven by *E*/*Z* photoisomerization reactions are able to produce unidirectional rotary motion because of a structural asymmetry that makes one direction of rotation more probable than the other. In most such motors, this asymmetry is realized through the incorporation of a chemically asymmetric carbon atom. Here, we present molecular dynamics simulations based on multiconfigurational quantum chemistry to investigate whether the merits of this approach can be equaled by an alternative approach that instead exploits isotopic chirality. By first considering an *N*-methylpyrrolidine–cyclopentadiene motor design, it is shown that isotopically chiral variants of this design undergo faster photoisomerizations than a chemically chiral counterpart, while maintaining rotary photoisomerization quantum yields of similarly high magnitude. However, by subsequently considering a pyrrolinium–cyclopentene design, it is also found that the introduction of isotopic chirality does not provide any control of the directionality of the photoinduced rotations within this framework. Taken together, the results highlight both the potential usefulness of isotopic rather than chemical chirality for the design of light-driven molecular motors, and the need for further studies to establish the exact structural circumstances under which this asymmetry is best exploited.

## Introduction

Molecular motors are molecules that use energy from an external source to produce mechanical motion and furthermore have the ability to control the direction of the resulting motion.^1−3^ Among the different types of synthetic molecular motors available today, those that achieve 360° unidirectional rotary motion through UV- or visible-light-powered photoisomerization reactions around a double bond are commonly referred to as light-driven rotary molecular motors, with examples including overcrowded-alkene,^4,5^ hemithioindigo,^6,7^ dibenzofulvene,^8^ and *N*-alkyl-imine^9,10^ motors. Typically, the required control of the photoinduced rotation of such systems is attained by the introduction of chirality into the motors and the asymmetry between clockwise (CW) and counterclockwise (CCW) photoisomerization directions that chirality imparts on their excited-state dynamics. While most of these motors contain a chiral center, it is not absolutely essential that they do. Indeed, overcrowded alkenes and dibenzofulvenes have proven to be able to sustain unidirectional rotary motion through the presence of a pseudoasymmetric carbon atom^11^ or an axially chiral trityl moiety,^8^ respectively, in place of a chiral center. Furthermore, it has been demonstrated that the CW or CCW rotary direction of motors featuring a ring-puckered cyclohexenylidene motif can be controlled by the intrinsic asymmetry afforded by this motif.^12^ Complementing these structure-based approaches to introduce the desirable asymmetry, it is also possible to steer the rotary motion of light-driven molecular motors by means of the chirality of the radiation supplied to them.^13^

**Scheme 1:**
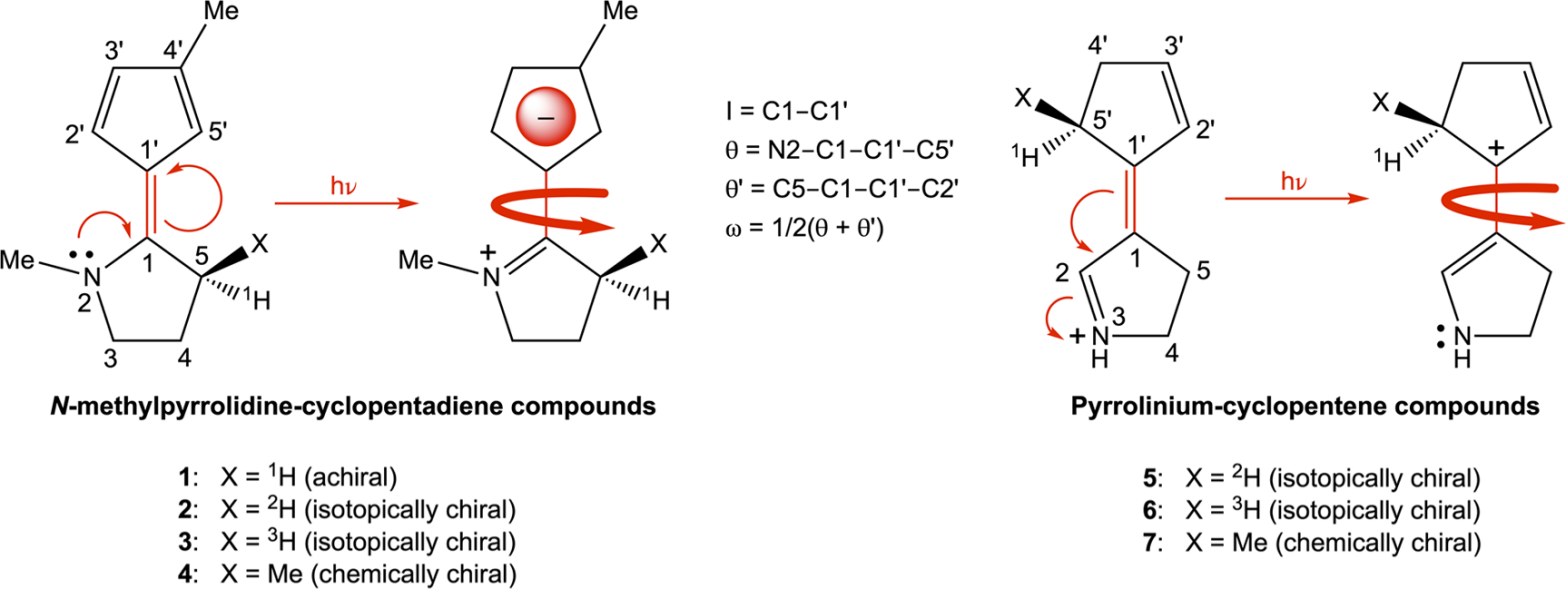
Chemical Structures of Compounds Studied in This Work Shown in Their *E* Isomeric Forms and Definitions of Key Geometric Parameters

Recently, an entirely new approach based on isotopic chirality (chirality resulting from isotopic substitution of an otherwise achiral species^14,15^) to induce unidirectional rotary motion in molecular motors operated through double-bond photoisomerizations was put forth and tested computationally by our group using quantum chemical methods and nonadiabatic molecular dynamics (NAMD) simulations.^16^ Although the study of isotopic chirality has a long history, beginning in 1949 with the first measurement of optical activity of an isotopically chiral molecule (α-deuteroethylbenzene)^17^ and involving both the manifestation of this phenomenon in many different contexts (such as in asymmetric autocatalysis,^18−20^ deracemization processes,^21^ and supramolecular aggregation^22,23^) and its investigation by theoretical methods,^15,24,25^ the consequences of isotopic chirality are generally less readily detectable than those of chemical chirality. Hence, it is noteworthy that our computational study predicted that two isotopically chiral compounds (**2** and **3** in Scheme 1), derived by replacing the methyl substituent at the C5 asymmetric carbon atom of a chemically chiral molecular motor (**4** in Scheme 1) by either ^2^H (in **2**) or ^3^H (in **3**), sustain unidirectional rotary motion almost just as well as the parent motor through their *E* → *Z* and *Z* → *E* photoisomerizations around the central olefinic bond.^16^ This finding shows that isotopic chirality has a possible role to play in the realization of rotary motion by molecular motors. In this connection, recent progress toward asymmetric synthesis of isotopically chiral hydrocarbons is very timely.^26^

Against this background, it is worthwhile to further investigate the possibility to exploit isotopic chirality for the design of molecular motors, especially since this idea is untested beyond the above-described results.^16^ In the present work, we report NAMD simulations that shed new light on this possibility from three different perspectives.

First, we explore a potential advantage that the isotopically chiral compounds **2** and **3** hold over the chemically chiral species **4** in that their rotary motion might be faster, because the moment of inertia with respect to the axis of the central olefinic bond is likely to be decreased when the C5 methyl group of **4** is replaced by ∼7.5-fold (^2^H in **2**) and ∼5-fold (^3^H in **3**) lighter substituents. In order to make a statistically rigorous assessment of this hypothesis, many more NAMD trajectories need to be calculated than what our initial study could afford. Despite the steep computational cost of such an endeavor, mandated both by statistical considerations and the need to use expensive multiconfigurational methods to ensure that the photochemical modeling is reliable, this work aims to test this hypothesis. Second, by performing these NAMD simulations, we are also able to assess how the photoisomerization quantum yields (QYs) of **2** and **3** compare to those of **4**, which have been shown to be high.^27^ This is particularly important because attaining high QYs is one of the main drivers of current research on light-driven molecular motors,^28−31^ stemming from the fact that the most developed class of such motors (the paradigm-shifting overcrowded alkenes^30^) performs rather poorly in this regard^32−34^ and, consequently, convert only a small portion of the absorbed radiation into rotary motion. Third, by running NAMD simulations of isotopically chiral counterparts of not only **4** but also of another type of chemically chiral motors with entirely different excited-state properties (**7** in Scheme 1), we begin to investigate the extent to which the concept of isotopic chirality is broadly applicable in the field of molecular motors.

## Results and Discussion

Before presenting the results from the NAMD simulations, it is appropriate to first briefly describe the key features of the chemically chiral motor designs **4** and **7** shown in Scheme 1, from which the isotopically chiral compounds studied in this work are derived. In **4**, the *E*/*Z*-isomerizable olefinic bond connects a cyclopentadiene motif to a chiral, electron-donating *N*-methylpyrrolidine motif, whereas in **7**, it connects a chiral cyclopentene to a protonated, nitrogen Schiff-base. These designs, which have their origin in computational work^27,29,35^ but for which synthetic routes are available,^36−38^ have been put forth as possible alternatives to overcrowded-alkene motors based on predictions that their photoisomerization QYs are higher.^27,39^ This is because the cleavage of the isomerizing π-bond is facilitated either by the concurrent transformation of the nonaromatic cyclopentadiene into an aromatic cyclopentadienyl anion in the bright second excited singlet state (S_2_) of **4**(27,40,41) or by the electron-withdrawing capacity of the cationic nitrogen center in the bright first excited singlet state (S_1_) of **7**.^29,42^

As for the ability of **4** and **7** to produce unidirectional rotary motion, this stems in part from the asymmetry between CW and CCW photoisomerization directions introduced by the presence of a chiral center in both compounds (C5 in **4** and C5′ in **7**). Furthermore, the steric hindrance toward photoisomerization posed by the methyl substituent at the respective chiral center is different for the two directions, which is also a characteristic of overcrowded-alkene motors.^30^ In this light, investigating how the motor performance is influenced by the reduction of this steric hindrance through the replacement of the methyl by ^2^H or ^3^H is of particular interest.

Deriving from **4**, achiral compound **1** and isotopically chiral compounds **2** and **3** harbor ^1^H, ^2^H or ^3^H in place of the C5 methyl, respectively. Similarly, deriving from **7**, isotopically chiral compounds **5** and **6** contain ^2^H or ^3^H in place of the C5′ methyl, respectively. Importantly, all of these compounds are small enough to be amenable to the NAMD-based modeling required to meet the goals of this work. Indeed, owing to their description of the simultaneous dynamics of electronic (treated quantum mechanically) and nuclear (treated classically) degrees of freedom, NAMD simulations are able to probe the effects of isotopic substitution on chemical reactions in a way that is not feasible by regular quantum chemical calculations performed within the adiabatic Born–Oppenheimer approximation (in this approximation, nuclear masses do not enter the electronic Hamiltonian). The merits of NAMD simulations in this regard have been amply demonstrated in studies of excited-state proton transfer reactions by Thiel and co-workers.^43^

### NAMD Simulations of **1–4**

Since the *E* → *Z* and *Z* → *E* photoisomerizations of **1–4** are triggered through population of the S_2_ state (by absorption of UV photons),^16^ the corresponding NAMD simulations were started in this state, from the respective vertically excited Franck–Condon (FC) point. The simulations were run for maximally 800 fs and, to obtain good statistical coverage, with 100 different initial nuclear configurations and velocities for each of the eight reactions. In order to quantify the results, a few designations and definitions were made as follows.

**Figure 1:**
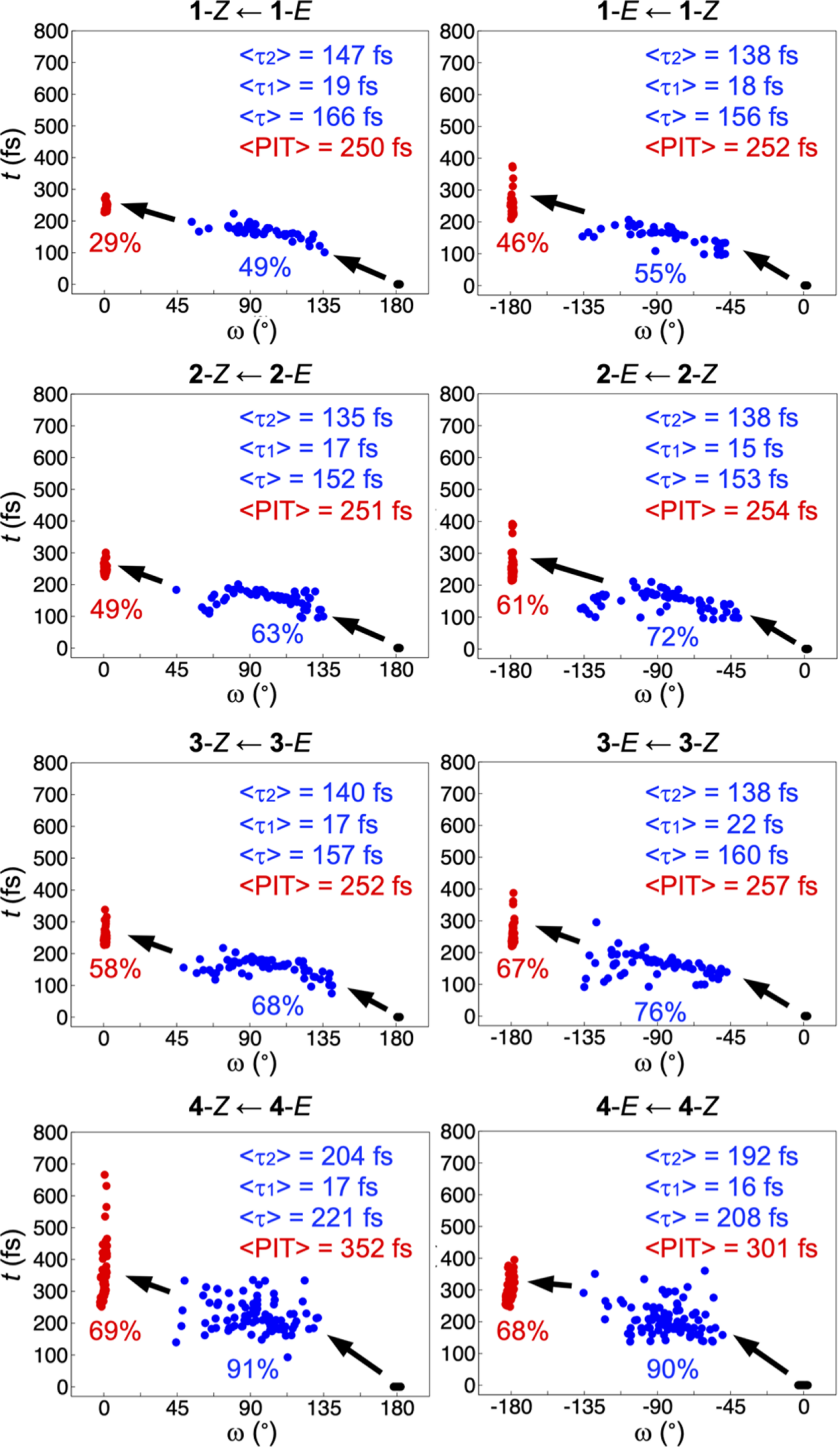
Distributions of τ (full blue circles) and PIT (full red circles) values from the NAMD simulations of the *E* and *Z* isomers of **1–4**, and the corresponding changes in the ω dihedral angle relative to the initial nuclear configuration (full black circles). Also shown are the average τ, τ_1_, τ_2_, and PIT values (⟨τ⟩, ⟨τ_1_⟩, ⟨τ_2_⟩ and ⟨PIT⟩), the percentages of trajectories that rotate in the direction of decreasing ω values and decay to the S_0_ state (in blue font), and the rotary QYs (in red font). The data underlying these results are summarized in Table S1.

First, a trajectory was considered successful if it both reaches the ground state (S_0_) through successive S_2_/S_1_ and S_1_/S_0_ nonadiabatic events and completes a full 180° rotation around the central olefinic bond within the maximum simulation time. Second, the photoisomerization time (PIT) and the total excited-state lifetime (τ) were defined as the time needed for one such rotation and the time needed for a trajectory to decay to the S_0_ state, respectively. Similarly, the S_2_ lifetime (τ_2_) was defined as the time needed for a trajectory to decay to the S_1_ state. Accordingly, it follows that the S_1_ lifetime (τ_1_) was obtained as τ_1_ = τ – τ_2_. Given that not all trajectories underwent decay to S_0_ within 800 fs (see Table S1 of the Supporting Information), average τ, τ_1_, and τ_2_ values were calculated exclusively among those trajectories that really did. Third, the total and rotary QYs of the photoisomerization of a given species were calculated as
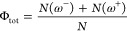
1and
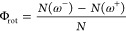
2respectively. Here, *N* is the total number of trajectories run (i.e., 100), and *N*(ω^–^) and *N*(ω^+^) are the number of successful trajectories rotating in the direction of decreasing and increasing values of the ω dihedral angle (defined in Scheme 1), respectively. With this way of calculating the QYs, the key predictor of whether **1–4** are efficient in producing 360° unidirectional rotary motion through consecutive *E* → *Z* and *Z* → *E* photoisomerizations is that the *rotary* QYs (rather than the *total* QYs) are high for both reactions. The main results from all 800 NAMD trajectories are summarized in Figure 1, with complementary data given in Table S1.

Starting with the chemically chiral compound **4**, which serves as the benchmark for the other compounds, it is notable from Figure 1 that the rotary QYs are high for both isomers. Specifically, equaling 69 (*E*) and 68% (*Z*), they reveal a clear propensity for the photoisomerizations to occur in the direction of decreasing ω values (cf. eq 2). In fact, Table S1 shows that out of the 71 (*E*) and 74 (*Z*) trajectories that were successful for **4**, only 1 (*E*) and 3 (*Z*) resulted in a rotation in the opposite direction. Regarding the 29 (*E*) and 26 (*Z*) unsuccessful trajectories, these correspond to situations where the decay to the S_0_ state is followed by regeneration of the parent species from which the reaction started or where the photoexcited system remains in the S_2_ state without isomerizing. In order to clarify which of these bottlenecks is the most significant one, it is observed that 91 (*E*) and 90% (*Z*) of the trajectories reach the S_0_ state after having undergone appreciable torsional motion in the direction of decreasing ω values (indicated by blue circles in Figure 1), typically decaying at ω values around 90 (*E*) or −90° (*Z*). Hence, by comparing these percentages with the rotary QYs of 69 (*E*) and 68% (*Z*), it is clear that regeneration of the parent species is the major bottleneck.

Besides its high rotary QYs, which substantially exceed those that are typical for overcrowded-alkene motors,^32−34^ another feature that makes **4** an attractive motor design is that its average τ and PIT values are small, amounting to no more than 221 and 352 fs for the *E* isomer and 208 and 301 fs for the *Z* isomer. For comparison, overcrowded-alkene motors usually have excited-state lifetimes of at least 1 ps.^34^ Thus, **4** appears capable of producing rather fast rotary motion. It can also be deduced from Figure 1 that most of the excited-state evolution of **4** occurs in the S_2_ state because the average τ_1_ values are much smaller (17 and 16 fs) than the average τ_2_ values (204 and 192 fs).

Continuing with the results for **1**–**3**, it is first noted that the rotary QYs of 29 (*E*) and 46% (*Z*) that the achiral compound **1** achieves are much lower than those of 69 (*E*) and 68% (*Z*) attained by **4**. The importance of chemical chirality for **4** to function as an efficient motor is thereby evident. However, moving to **2** and **3**, it is striking that this gap in the rotary QYs is significantly narrowed (in **2**) or even almost eliminated (in **3**) by the isotopic chirality of these species. More specifically, the rotary QYs are 49 (*E*) and 61% (*Z*) for **2** and 58 (*E*) and 67% (*Z*) for **3**. Emerging from a very large set of calculated NAMD trajectories, these results suggest that at least within certain molecular frameworks, the potency of isotopic chirality in facilitating high-QY rotary motion in light-driven molecular motors is comparable to that of chemical chirality, despite that isotopic chirality affords less steric hindrance to control the directionality of the photoisomerizations. As for the rotary QYs being somewhat higher for **3** than for **2**, this is consistent with the expectation, discussed by Soai and co-workers,^19^ that a larger isotope mass ratio (^3^H/^1^H in **3** vs ^2^H/^1^H in **2**) should give a larger isotope effect also in the context of chirality.

Another observation for **2** and **3** in Figure 1 concerns the differences between the percentages of trajectories that decay to the S_0_ state (63–72% for **2** and 68–76% for **3**) and their rotary QYs (49–61% for **2** and 58–67% for **3**), which show that the decay process is the major bottleneck for the photoisomerizations of these compounds. This is contrary to the aforementioned situation for **4**, for which regeneration of the parent species is the main bottleneck. Through previous minimum energy path (MEP) calculations starting from the S_2_ FC points of the *E* and *Z* isomers of **2** and **3**, it has been found that the decay process is likely to be mediated by S_2_/S_1_ and S_1_/S_0_ conical intersection seams, as evidenced by the localization of structures with very small S_2_/S_1_ (∼1–2 kcal mol^–1^) and S_1_/S_0_ (∼5 kcal mol^–1^) energy gaps along the MEPs of both isomers.^16^ These structures, which are shown in Figure S2 of the Supporting Information, also provide a tentative explanation for the observation that the average τ_1_ values for **2** and **3** are much smaller (15–22 fs) than their average τ_2_ values (135–140 fs). Specifically, for both isomers, it was found that the small-energy-gap S_1_/S_0_ MEP structure lies very close to the small-energy-gap S_2_/S_1_ MEP structure along the ω reaction coordinate, both being substantially rotated relative to the S_2_ FC point (see Figure S2).^16^ Hence, the photoinduced motion takes place predominantly in the S_2_ state, and there is no need for any appreciable further rotation in the S_1_ state to subsequently reach the S_1_/S_0_ decay channel.

**Figure 2:**
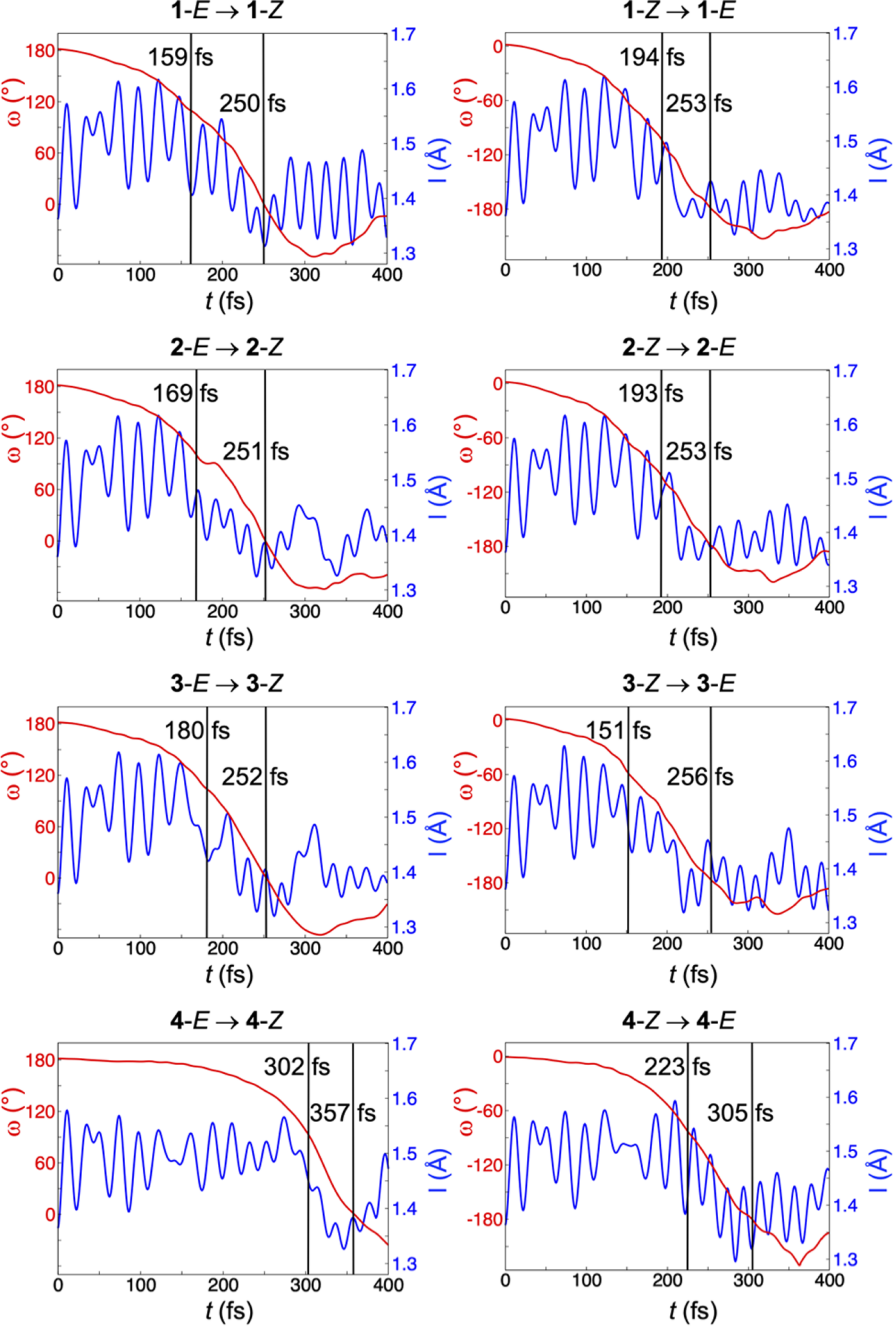
Changes in the ω dihedral angle and the length of the central olefinic bond (I) along typical NAMD trajectories for the *E* and *Z* isomers of **1–4**. τ and PIT values are indicated with vertical lines.

From Figure 1, it can also be inferred that the use of isotopic chirality to control the directionality of the *E*/*Z* photoisomerizations of **2** and **3** holds a potential advantage over the use of chemical chirality by allowing for faster rotary motion. Indeed, although it has already been seen that **4** exhibits average τ and PIT values that are small (208–221 and 301–352 fs), those of **2** and **3** (152–160 and 251–257 fs) are even smaller by ∼15–30% (as for the average τ values, this difference is primarily contained in the average τ_2_ values, which amount to 192–204 fs for **4** and 135–140 fs for **2** and **3**). This is likely a consequence of the decrease in moment of inertia with respect to the axis of rotation that comes from replacing the C5 methyl group of **4** with lighter ^2^H and ^3^H substituents in **2** and **3**. In order to further assess the difference in τ and PIT values between **2**/**3** and **4**, it is of interest to investigate how the ω dihedral angle and the length of the central olefinic bond evolve along individual NAMD trajectories for the *E* and *Z* isomers of **1–4**. This is done in Figure 2, for “typical” trajectories whose PIT values are similar to the average PIT values in Figure 1. As can be seen, for each reaction, the very initial excited-state dynamics is dominated by substantial stretching of the central bond, which changes character from C=C (bond length smaller than 1.4 Å) to C–C (bond length larger than 1.5 Å) to facilitate the subsequent rotation. However, while there is a plateau-like lag for the rotation to begin in the *E* and *Z* isomers of **4**, in the lighter species **2** and **3,** there is no such delay, and the rotation can be completed in shorter time.

### NAMD Simulations of **5–7**

**Figure 3:**
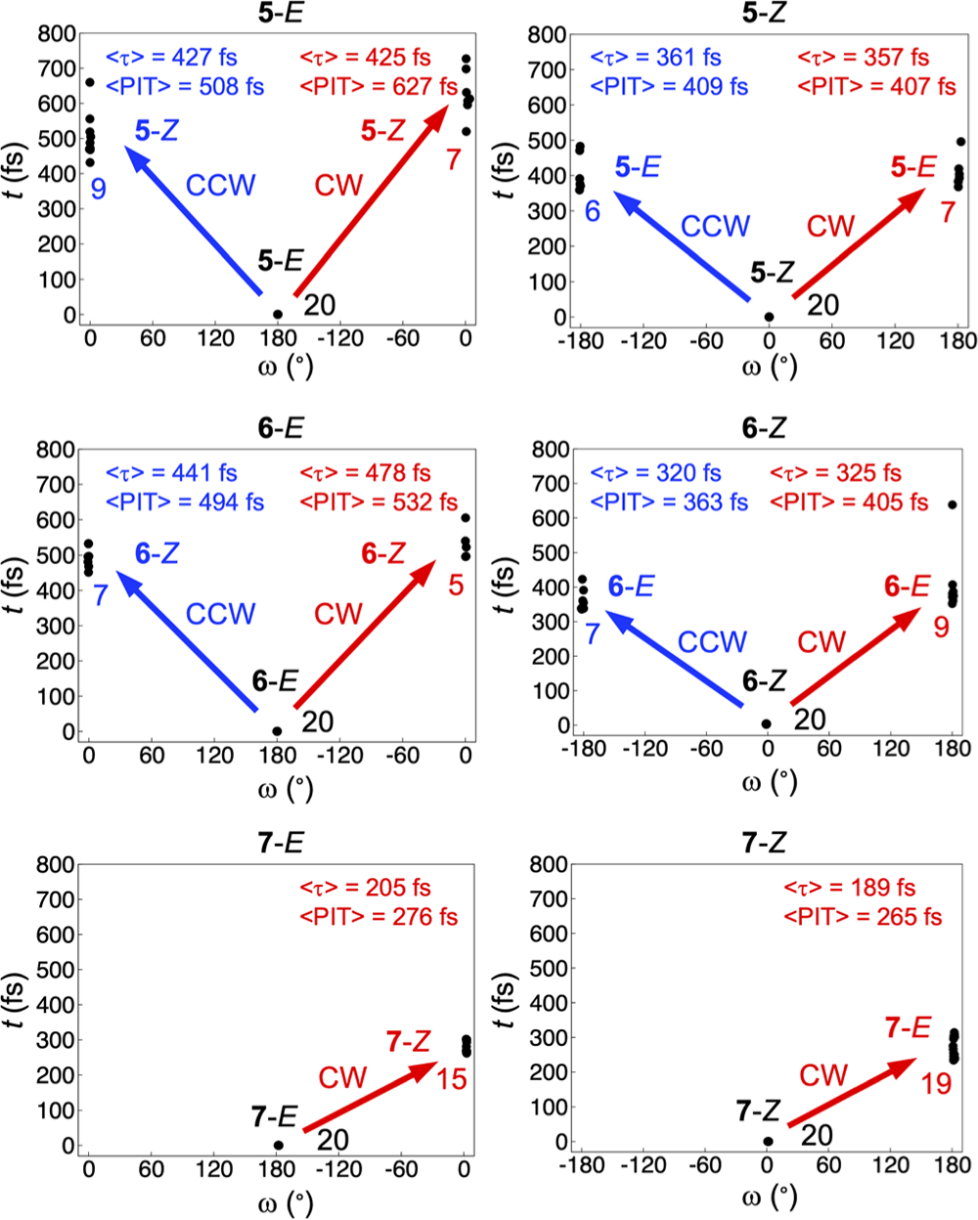
Distributions of PIT values for successful NAMD trajectories for the *E* and *Z* isomers of **5–7** rotating in the direction of either increasing (CW) or decreasing (CCW) ω dihedral angle, the corresponding average τ and PIT values (⟨τ⟩ and ⟨PIT⟩), and the corresponding changes in the ω value relative to the initial nuclear configuration. Also shown are the number of successful trajectories in each category. The data underlying these results are summarized in Table S2.

The NAMD-based modeling of **5–7** was performed similarly to that of **1–4**, starting from the respective FC point in the S_1_ state in which the *E* → *Z* and *Z* → *E* photoisomerizations of small, protonated Schiff-base compounds are known to occur following absorption of UV photons.^44^ However, since the aim of these simulations to investigate *qualitatively* whether isotopic chirality can induce unidirectional rotary motion also in a Schiff-base motor framework is very different from that of the simulations of **1–4** (which was to provide *quantitatively* accurate estimates of rotary QYs, τ and PIT values), a fewer number of different initial nuclear configurations and velocities were considered for each of the six photoisomerizations (20 instead of 100). This, we believe, is a sound and economical strategy because assessing whether the resulting trajectories have a tendency to rotate in one and the same direction requires less statistical coverage than calculating rotary QYs, τ and PIT values. The main results from all 120 trajectories are summarized in Figure 3, with complementary data given in Table S2 of the Supporting Information.

Starting with the chemically chiral compound **7**, Figure 3 shows that the ratios of successful photoisomerization trajectories are high for both isomers, amounting to 15/20 (*E*) and 19/20 (*Z*). Furthermore, all of these rotations are perfectly unidirectional, occurring exclusively in the direction of increasing ω values, which is here defined as a CW rotation. Combined with the observation that the average τ and PIT values are small, equaling 205 and 276 fs for the *E* isomer and 189 and 265 fs for the *Z* isomer, **7** appears to be another motor design alongside **4** capable of achieving both high rotary QYs and fast rotary motion. Continuing with the results for the isotopically chiral compounds **5** and **6**, however, the situation is different. In fact, even though the ratios of successful trajectories remain high (16/20 and 13/20 for the *E* and *Z* isomers of **5**, and 12/20 and 16/20 for the *E* and *Z* isomers of **6**), the frequencies with which the rotations occur in the CW direction are *not* consistently larger than the frequencies with which they occur in the opposite CCW direction, toward decreasing ω values. Contrarily, the two directions appear equally probable. This suggests that **5** and **6** are less able than the chemically chiral compound **7** to utilize consecutive *E* → *Z* and *Z* → *E* photoisomerizations to produce a full 360° rotation around the central olefinic bond. Furthermore, this finding indicates that the overall ability of isotopic chirality to control the directionality of *E*/*Z* photoisomerizations depends on the molecular framework. Indeed, while we have seen that this ability is pronounced within the *N*-methylpyrrolidine–cyclopentadiene framework of **1–4**, it seems poor within the pyrrolinium–cyclopentene framework of **5–7**.

In view of these results, identifying general structural circumstances under which isotopic chirality can contribute to the design of light-driven rotary molecular motors is a natural goal for future research in this field. From the viewpoint of quantum chemical modeling, such endeavors are likely to require nonstandard approaches that go beyond the Born–Oppenheimer approximation and allow for the difference in mass between the relevant isotopes to influence calculated molecular geometries and reaction paths.^45^ Progress along those lines will also stimulate computational studies of the role of isotopic chirality in various other experimentally documented contexts, such as asymmetric autocatalysis^18−20^ and supramolecular aggregation.^22,23^

## Conclusions

In summary, we have performed extensive NAMD simulations to investigate potential merits of using isotopic chirality instead of chemical chirality to induce unidirectional rotary motion in molecular motors operated by *E*/*Z* photoisomerization reactions around an olefinic bond. To this end, we have studied two different types of motors that on the one hand are sufficiently small to enable NAMD simulations with a state-of-the-art electronic-structure method, and, on the other, photoisomerize at a short enough time scale to make extensive simulations tractable. By first considering an *N*-methylpyrrolidine–cyclopentadiene motor, it is found that isotopically chiral variants of this motor show rotary QTs for their photoisomerizations that are quite comparable to the ones of ∼70% achieved by the parent, chemically chiral species. Furthermore, it is noted that the use of isotopic chirality to control the directionality of the photoisomerizations allows for faster rotary motion, which is a clear advantage. However, by then considering a pyrrolinium–cyclopentene motor, no preference for one direction of isomerization (CW or CCW) over the other is observed upon introduction of isotopic chirality, which suggests that only certain molecular frameworks are able to exploit this asymmetry for the production of unidirectional rotary motion. In future research, we will first attempt to identify key characteristics of such frameworks and, if successful, subsequently investigate whether these characteristics are present in or can be introduced into well-established overcrowded-alkene^4,5,30^ or hemithioindigo motors.^6,7^ For example, it will be of interest to assess whether isotopic chirality can help reduce the >1 ps excited-state lifetimes of overcrowded-alkene motors.^34^

## Computational Methods

The NAMD simulations were carried out with Tully’s fewest switches algorithm^46^ and the complete active space self-consistent field (CASSCF) method^47^ in combination with the double-zeta 6-31G(d) basis set, using the OpenMolcas suite of programs.^48^ Electronic wave functions were calculated through a state-average CASSCF procedure with equal weights for the states involved in the photoisomerizations—S_0_, S_1_, and S_2_ for **1–4**, and S_0_ and S_1_ for **5–7**. As for active spaces, the full π-system of the cyclopentadiene motif and the central olefinic bond, as well as the nitrogen lone pair of the *N*-methylpyrrolidine motif, was included in the CASSCF modeling of **1–4** (i.e., CAS(8,7)), and the full π-system of the pyrrolinium–cyclopentene skeleton was included in the CASSCF modeling of **5–7** (i.e., CAS(6,6)). The simulations were started in the S_2_/S_1_ state of **1–4**/**5–7** and were run for maximally 800 fs with 100/20 different initial nuclear configurations and velocities generated by performing S_0_ Born–Oppenheimer MD simulations for 3 ps at the CASSCF/6-31G(d) level of theory. An integration time step of 0.5 fs was used for the classical propagation of the nuclear positions by means of the velocity Verlet algorithm.^49^

As outlined in detail elsewhere,^46,50^ the quantum mechanical description of electrons in Tully’s method makes use of the possibility to express the total wave function Ψ(**r**,*t*;**R**) for a molecular system as a linear combination of adiabatic electronic states {ϕ_*j*_(**r**;**R**)} with time-dependent complex amplitudes {*a*_*j*_(*t*)}
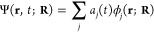
3where **r** denotes electronic coordinates and **R** = **R**(*t*) is the trajectory followed by the nuclei. In the present case, this expansion includes the S_0_, S_1_, and S_2_ states for **1–4**, and the S_0_ and S_1_ states for **5–7**. The requirement that Ψ(**r**,*t*;**R**) satisfies the time-dependent Schrödinger equation then leads to a set of coupled differential equations that can be numerically integrated (herein with a time step of 0.0025 fs) to obtain the amplitudes
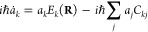
4Here, *E*_*k*_(**R**) is the potential energy of the adiabatic electronic state *k* and {*C*_*kj*_} are the non-adiabatic coupling matrix elements between states *k* and *j*, defined as
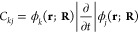
5where the integration is over the electronic coordinates **r**. Throughout the NAMD simulations, the trajectories are free to populate any state included in the expansion 3, with the respective population determined by the value of |*a*_*j*_(*t*)|^2^.

## Supporting Information Available

Molecular orbital plots; key MEP structures; detailed statistics from the NAMD simulations; orbital occupation numbers; energies and Cartesian coordinates of stationary points of **1–7**; and energies and Cartesian coordinates along the **2**-*E* → **2**-*Z* and **2**-*Z* → **2**-*E* photoisomerization trajectories in Figure 2 (PDF)
