# Supplementary material for: Computational Comparison of Chemical and Isotopic Approaches to Control the Photoisomerization Dynamics of Light-Driven Molecular Motors
Source: J Org Chem. 2021 Mar 30;86(8):5552–9. doi: 10.1021/acs.joc.1c00063 (PMC8154570; doi:10.1021/acs.joc.1c00063)
Supplement: Supplementary file 1 — jo1c00063_si_001.pdf [file jo1c00063_si_001.pdf]

Computational Comparison of Chemical and Isotopic Approaches to Control the Photoisomerization Dynamics of Light-Driven Molecular Motors

Jun Wang,*,† Baswanth Oruganti,‡ and Bo Durbeej*,§

†Institut de Química Computacional i Catàlisi, Facultat de Ciències, Universitat de Girona, ES-17003 Girona, Spain
‡Department of Chemistry and Biomedical Sciences, Faculty of Health and Life Sciences, Linnaeus University, SE-45041 Kalmar, Sweden
§Division of Theoretical Chemistry, IFM, Linköping University, SE-58183 Linköping, Sweden

Supporting Information

Corresponding Authors

*E-mail: jun.wang@udg.edu (J.W.)

*E-mail: bodur@ifm.liu.se (B.D.)

Table of contents

Figure S1	page S3
Figure S2	page S4
Table S1	page S5
Table S2	page S6
Table S3	page S8
References for this document	page S9
Total electronic energies and Cartesian coordinates of optimized S_0 geometries	page S10
Total electronic energies of current state and Cartesian coordinates along the **2**-*E* → **2**-*Z* photoisomerization trajectory in Figure 2	page S18
Total electronic energies of current state and Cartesian coordinates along the **2**-*Z* → **2**-*E* photoisomerization trajectory in Figure 2	page S546

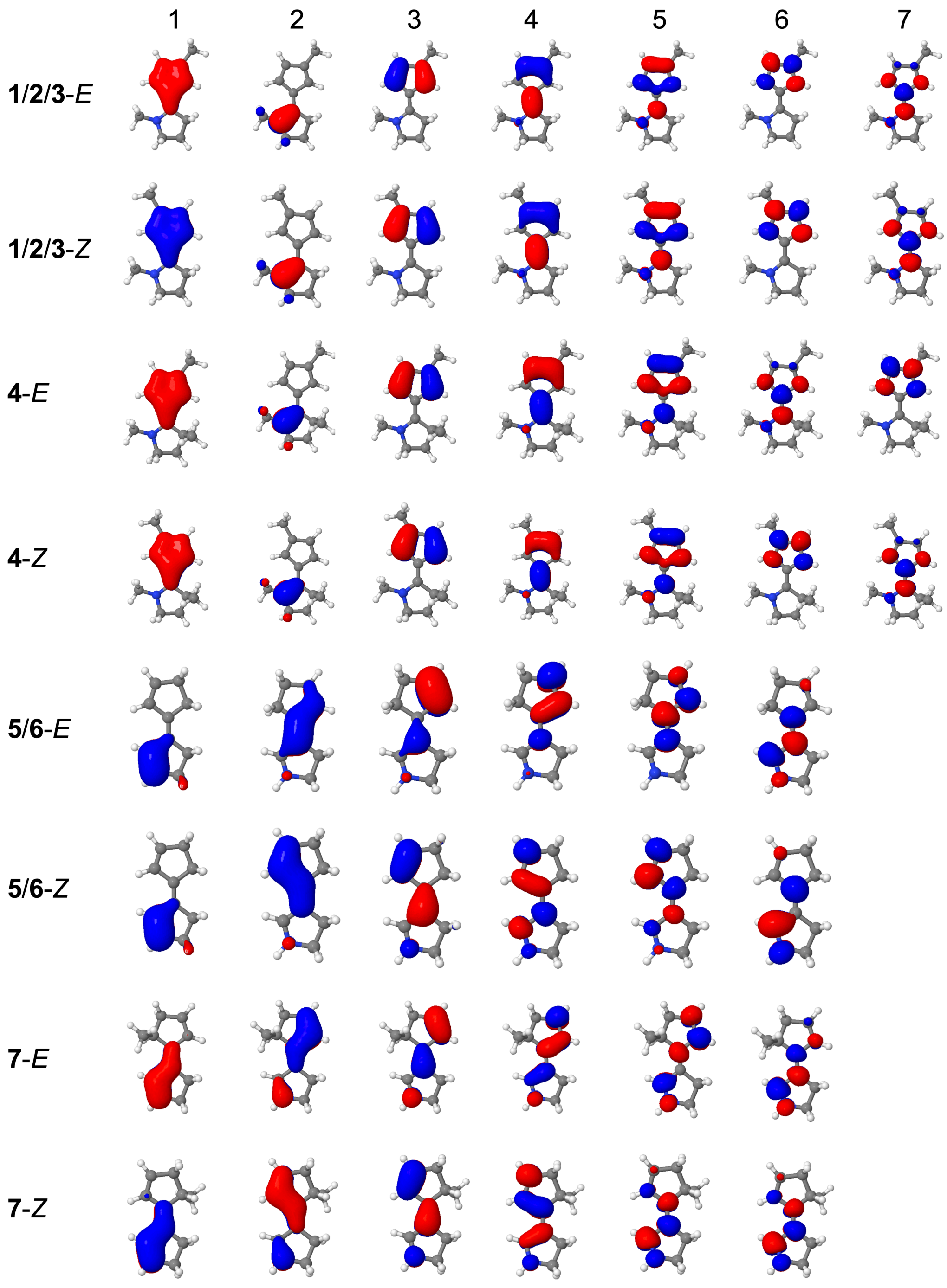

Figure S1. Active molecular orbitals in the CASSCF modeling of the *E* and *Z* isomers of **1**–**7** (at their CASSCF/cc-pVTZ S_0 equilibrium geometries).

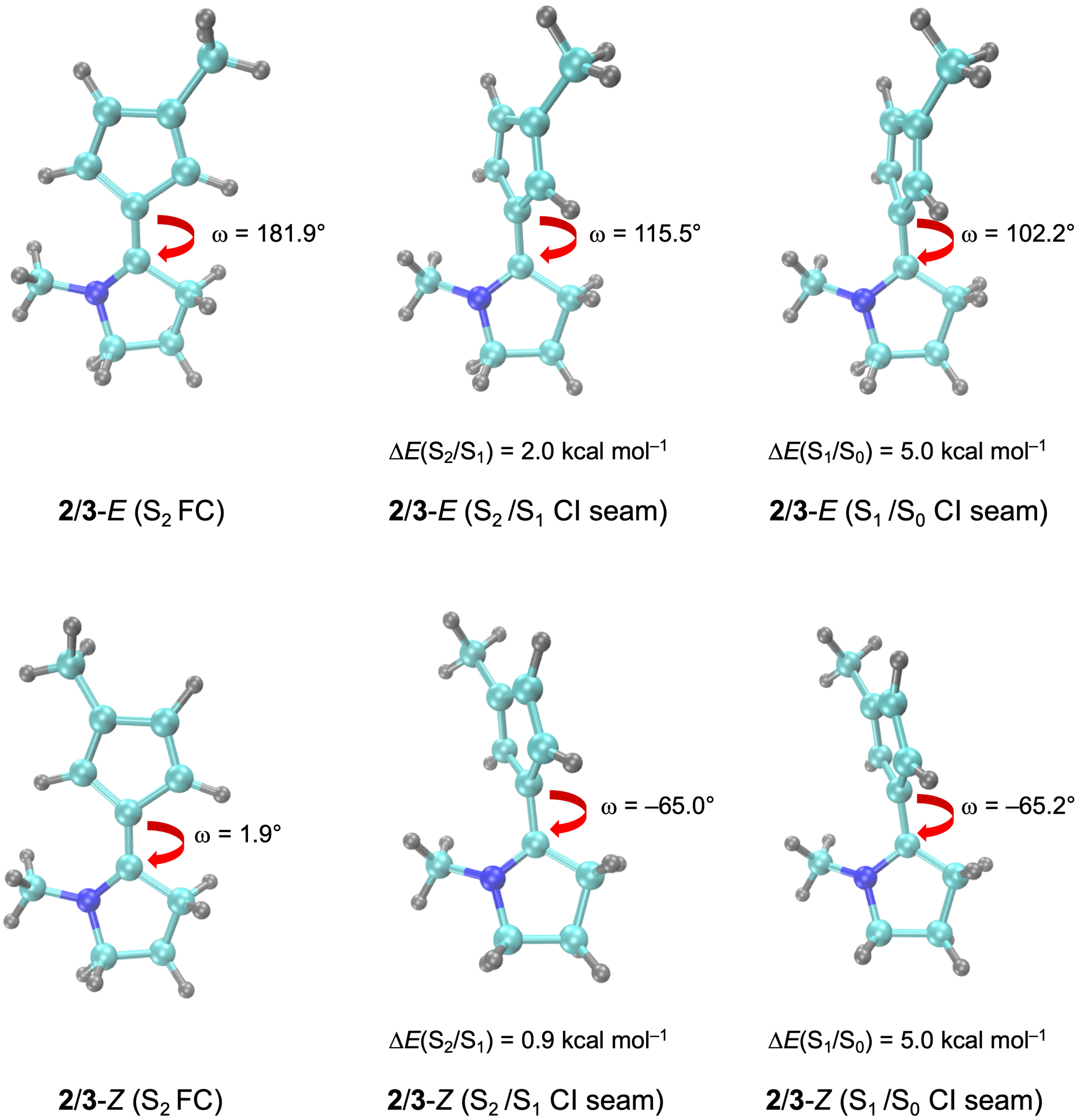

Figure S2. Structures of **2** and **3** with small S_2/S_1 and S_1/S_0 energy gaps ($\Delta E(S_2/S_1)$ and $\Delta E(S_1/S_0)$, respectively) presumably located in the vicinity of conical intersection (CI) seams obtained in a previous study through minimum energy path calculations performed at the CASSCF(8,7)/cc-pVTZ level of theory starting from the S_2 Franck-Condon (FC) points of the *E* and *Z* isomers.[1] Also shown are the corresponding values of the ω dihedral angle.

Table S1. Summary of Results from the NAMD Simulations of the *E* and *Z* Isomers of 1–4

	**1**		**2**		**3**		**4**	
quantity	*E*	*Z*	*E*	*Z*	*E*	*Z*	*E*	*Z*
N^a	100	100	100	100	100	100	100	100
N(fail)[b]	71	54	51	39	42	33	29	26
$N(\omega^-)$[c]	29	46	49	61	58	67	70	71
<PIT>[d]	250	252	251	254	252	257	352	301
N(decay)[e]	49	55	63	72	68	76	91	90
$\langle\tau\rangle$[f]	166	156	152	153	157	160	221	208
$\langle\tau_1\rangle$[g]	19	18	17	15	17	22	17	16
$\langle\tau_2\rangle$[h]	147	138	135	138	140	138	204	192
$N(\omega^+)$[i]	0	0	0	0	0	0	1	3
Φ_{rot}[j]	0.29	0.46	0.49	0.61	0.58	0.67	0.69	0.68
Φ_{tot}[k]	0.29	0.46	0.49	0.61	0.58	0.67	0.71	0.74

[a]Total number of trajectories run for each of the *E* and *Z* isomers of **1**–**4**.

[b]Number of unsuccessful trajectories, i.e., trajectories that fail to complete a photoisomerization within 800 fs by undergoing a full 180° rotation around the central olefinic bond.

[c]Number of successful trajectories that complete a photoisomerization within 800 fs by undergoing a full 180° rotation around the central olefinic bond in the direction of decreasing ω values.

[d]Average photoisomerization time (in fs) for $N(\omega^-)$ trajectories.

[e]Number of trajectories that rotate in the direction of decreasing ω values and decay to the S_0 state.

[f]Average total excited-state lifetime (in fs) for N(decay) trajectories.

[g]Average S_1 lifetime (in fs) for N(decay) trajectories.

[h]Average S_2 lifetime (in fs) for N(decay) trajectories.

[i]Number of successful trajectories that complete a photoisomerization within 800 fs by undergoing a full 180° rotation around the central olefinic bond in the direction of increasing ω values.

[j]Rotary photoisomerization quantum yield calculated as $\Phi_{\mathrm{rot}} = \frac{N(\omega^-) - N(\omega^+)}{N}$.

[k]Total photoisomerization quantum yield calculated as $\Phi_{\mathrm{tot}} = \frac{N(\omega^-) + N(\omega^+)}{N}$.

Table S2. Summary of Results from the NAMD Simulations of the *E* and *Z* Isomers of 5–7

	**5**		**6**		**7**	
	E	*Z*	*E*	*Z*	*E*	*Z*
trajectory	DPI[a]/τ[b]/PIT[c]	DPI[a]/τ[b]/PIT[c]	DPI[a]/τ[b]/PIT[c]	DPI[a]/τ[b]/PIT[c]	DPI[a]/τ[b]/PIT[c]	DPI[a]/τ[b]/PIT[c]
1	+/169/727	+/349/393	+/444/497	+/323/371	+/205/301	+/193/245
2	+/469/520	+/322/368	+/459/523	+/306/352	+/203/266	+/195/247
3	+/646/698	+/340/383	+/489/540	+/315/361	+/204/268	+/198/250
4	–/470/519	+/341/384	–/436/496	+/308/381	+/204/300	+/196/275
5	–/395/660	–/326/376	–/433/481	–/341/391	+/204/267	+/195/266
6	–/379/432	–/324/371	–/397/468	–/309/355	+/207/281	+/190/243
7	–/500/556	–/347/391	–/480/532	–/294/338	+/203/268	+/186/296
8	–/431/488	–/313/359	*/*/*	*/342/*	+/204/302	+/182/234
9	*/453/*	*/*/*	*/453/*	*/*/*	*/*/*	+/180/255
10	*/432/*	*/*/*	*/489/*	*/*/*	*/*/*	+/183/236
11	+/453/613	+/416/496	+/445/496	+/304/372	+/203/265	+/187/239
12	+/431/596	+/372/419	+/554/605	+/326/375	+/204/267	+/182/307
13	+/413/631	+/360/404	–/453/497	+/341/387	+/205/267	+/188/314
14	+/393/607	–/433/483	–/407/451	+/358/407	+/204/269	+/188/302
15	–/416/468	–/423/471	–/482/533	+/342/638	+/198/262	+/186/239
16	–/458/505	*/370/*	*/399/*	–/294/338	+/203/266	+/194/246
17	–/398/472	*/*/*	*/*/*	–/373/423	+/217/294	+/193/303
18	–/395/470	*/*/*	*/*/*	–/292/336	*/*/*	+/184/236
19	*/*/*	*/*/*	*/*/*	–/315/361	*/*/*	+/187/308
20	*/*/*	*/*/*	*/*/*	*/*/*	*/*/*	*/*/*
averages						
$<\tau^{+}>$[d]	425	357	478	325	205	189
$<\tau^{-}>$[e]	427	361	441	320	n/a	n/a
$<\tau>$[f]	428	360	454	323	205	189
$<PIT^{+}>$[g]	627	407	532	405	276	265
$<PIT^{-}>$[h]	508	409	494	363	n/a	n/a
$<PIT>$[i]	560	408	510	387	276	265

[a]Direction toward either increasing (+) or decreasing (–) ω values in which a photoisomerization is completed within 800 fs by undergoing a full 180° rotation around the central olefinic bond. Trajectories that fail to complete one such photoisomerization are indicated with an asterisk (*).

[b]Excited-state lifetime (in fs) for trajectory if it decays to the S_0 state.

[c]Photoisomerization time (in fs) for trajectory if it undergoes a full 180° rotation around the central olefinic bond.

[d]Average excited-state lifetime (in fs) for trajectories that rotate in the direction of increasing ω values and decay to the S_0 state.

[e]Average excited-state lifetime (in fs) for trajectories that rotate in the direction of decreasing ω values and decay to the S_0 state.

[f]Average excited-state lifetime (in fs) for trajectories that rotate in the direction of either increasing or decreasing ω values and decay to the S_0 state.

[g]Average photoisomerization time (in fs) for trajectories that undergo a full 180° rotation around the central olefinic bond in the direction of increasing ω values.

[h]Average photoisomerization time (in fs) for trajectories that undergo a full 180° rotation around the central olefinic bond in the direction of decreasing ω values.

[i]Average photoisomerization time (in fs) for trajectories that undergo a full 180° rotation around the central olefinic bond in the direction of either increasing or decreasing ω values.

Table S3. Occupation Numbers for Active Molecular Orbitals in the CASSCF Modeling of the *E* and *Z* Isomers of 1–7[a]

		active molecular orbital						
compound/isomer	state	1	2	3	4	5	6	7
**1**/**2**/**3**-*E*	S_0	1.96	2.00	1.91	1.91	0.11	0.05	0.06
	S_1	1.95	2.00	1.89	1.04	0.99	0.06	0.08
	S_2	1.95	1.97	1.33	1.85	0.68	0.07	0.15
**1**/**2**/**3**-*Z*	S_0	1.96	2.00	1.91	1.91	0.11	0.05	0.06
	S_1	1.95	2.00	1.89	1.02	1.00	0.06	0.08
	S_2	1.95	1.97	1.33	1.85	0.68	0.07	0.15
**4**-*E*	S_0	1.96	2.00	1.91	1.91	0.11	0.05	0.06
	S_1	1.95	2.00	1.89	1.05	0.97	0.06	0.08
	S_2	1.95	1.96	1.32	1.86	0.69	0.07	0.15
**4**-*Z*	S_0	1.96	1.99	1.91	1.92	0.11	0.05	0.06
	S_1	1.95	2.00	1.89	1.04	0.98	0.06	0.08
	S_2	1.95	1.96	1.32	1.87	0.69	0.07	0.14
**5**/**6**-*E*	S_0	1.98	1.99	1.94	0.04	0.02	0.02	*
	S_1	1.98	1.94	1.12	0.88	0.02	0.06	*
**5**/**6**-*Z*	S_0	1.97	1.94	1.92	0.09	0.05	0.03	*
	S_1	1.97	1.92	1.08	0.92	0.03	0.08	*
**7**-*E*	S_0	1.97	1.94	1.92	0.09	0.05	0.03	*
	S_1	1.97	1.92	1.01	0.99	0.04	0.07	*
**7**-*Z*	S_0	1.97	1.94	1.91	0.09	0.03	0.05	*
	S_1	1.97	1.92	1.11	0.89	0.03	0.08	*

[a]Occupation numbers at CASSCF/cc-pVTZ S_0 equilibrium geometries.

References for this document

(1) Wang, J.; Oruganti, B.; Durbeej, B. Unidirectional Rotary Motion in Isotopically Chiral Molecular Motors: A Computational Analysis. *Org. Lett.* **2020**, *22*, 7113–7117.

Total electronic energies (E, in a.u.) and Cartesian coordinates (in Å) of optimized S_0 geometries

1/**2**/**3**-*E*, CASSCF/cc-pVTZ (all vibrational frequencies real)

$E(S_0)$ = -479.91984687

C	-0.82345788	-0.19105163	-0.07200512
C	0.49443647	0.13263129	-0.05763125
C	1.18611380	1.42845150	-0.11642525
C	1.57199870	-0.86277009	0.02215496
C	2.77102897	-0.23744413	0.01301335
C	2.51907430	1.20581557	-0.07541507
N	-1.92681172	0.62801596	-0.16993252
C	-3.14130340	-0.08139670	0.17895103
H	3.28228516	1.95869640	-0.09999021
C	-1.32999859	-1.61849635	0.04945710
C	-2.82880977	-1.51063168	-0.23432201
H	-3.02008788	-1.63054475	-1.29346959
H	-3.41747576	-2.24318156	0.30031322
H	-3.35098699	-0.01695583	1.24805599
H	-3.98872290	0.33797721	-0.34755990
H	-0.82349804	-2.29163912	-0.62525013
H	0.72946669	2.39031196	-0.17497362
C	-1.89161678	2.05332965	0.01318573
H	-1.46969290	2.33985885	0.97396986
H	-1.32080173	2.53079274	-0.77009700
H	-2.90193585	2.43409031	-0.04009589
H	1.42571498	-1.92162486	0.08208470
C	4.13298530	-0.85595154	0.08042927
H	4.72216124	-0.60321852	-0.79560208
H	4.68147663	-0.50326863	0.94833642
H	4.07067510	-1.93518108	0.14084669
H	-1.15493813	-1.97433495	1.05873732

1/**2**/**3**-*Z*, CASSCF/cc-pVTZ (all vibrational frequencies real)

E(S_0) = -479.91988498

C	-0.88232863	-0.24638718	-0.05543604
C	0.47254241	-0.31825879	-0.02273584
C	1.20820017	-1.58510701	0.07580876
C	1.51583332	0.71699491	-0.07427238
C	2.73298955	0.12736149	-0.01302634
C	2.53321526	-1.32357851	0.08218889
N	-1.69826422	0.85722982	-0.17282078
C	-3.07224912	0.53420846	0.15509062
H	3.32214608	-2.04665230	0.14680605
C	-1.78441622	-1.46370980	0.06412245
C	-3.18172274	-0.92724014	-0.24885554
H	-3.38074579	-0.99459440	-1.31121003
H	-3.96723314	-1.45297500	0.27608156
H	-3.27247718	0.66512447	1.21990726
H	-3.75201023	1.17764664	-0.38815660
H	-1.48539778	-2.26302836	-0.59648529
H	0.76009435	-2.55469024	0.13580046
C	-1.25342630	2.21238843	0.00742030
H	-0.78480272	2.37197911	0.97589393
H	-0.55381921	2.49658267	-0.76507725
H	-2.10833565	2.86975716	-0.06758013
H	1.35895967	1.77036260	-0.14162385
C	4.07316480	0.79523333	-0.03242026
H	4.63586375	0.57084260	0.86838836
H	4.66781466	0.45567565	-0.87473337
H	3.97468742	1.87101959	-0.10544417
H	-1.73842152	-1.84332217	1.07884721

4-*E*, CASSCF/cc-pVTZ (all vibrational frequencies real)

$E(S_0)$ = -518.96617913

C	-0.70961456	0.04540345	-0.14274171
C	0.62712226	0.28222922	-0.08057695
C	1.38251242	1.53490622	0.05712169
C	1.65426367	-0.76385776	-0.17243320
C	2.88322506	-0.20319845	-0.09918737
C	2.70350669	1.24500897	0.04616617
N	-1.74543013	0.94456464	-0.10799482
C	-3.04397005	0.30759076	-0.15942332
H	3.50302370	1.95500305	0.12764112
C	-1.31683805	-1.34601512	-0.26197585
C	-2.73243637	-1.05020177	-0.77160481
H	-2.72846369	-0.96579275	-1.85142101
H	-3.44959174	-1.81146305	-0.49536895
H	-3.48443348	0.21161243	0.83248894
H	-3.73014734	0.88930758	-0.76267552
H	-0.76762375	-1.94209588	-0.97547876
H	0.97318002	2.51529342	0.15077350
C	-1.64277200	2.31511050	0.31122714
H	-1.13636886	2.41068362	1.26654405
H	-1.11698346	2.91969911	-0.41745057
H	-2.63893443	2.72105455	0.41722750
C	-1.33269621	-2.07539507	1.08586928
H	-1.79993794	-3.04850467	0.98021236
H	-0.33107243	-2.21828764	1.46672618
H	-1.88973879	-1.51695086	1.83015341
H	1.45981772	-1.80976446	-0.29051962
C	4.21263251	-0.89010291	-0.15721413
H	4.80474110	-0.52913743	-0.99242338
H	4.78781508	-0.70889259	0.74546960
H	4.09602704	-1.96076307	-0.26903195

4-*Z*, CASSCF/cc-pVTZ (all vibrational frequencies real)

$E(S_0)$ = -518.96623391

C	-0.71781832	-0.01609776	-0.11443201
C	0.63172808	-0.20510083	-0.10359531
C	1.27101618	-1.50702535	-0.25973404
C	1.74119647	0.73908789	0.03535575
C	2.90616095	0.07758366	-0.02945328
C	2.61491717	-1.34155771	-0.21501220
N	-1.42772847	1.15095025	0.00430837
C	-2.86266324	0.96381448	-0.10081397
H	3.35301556	-2.11391681	-0.30540647
C	-1.72912331	-1.14600430	-0.24588423
C	-2.98901612	-0.41754744	-0.72792674
H	-2.97379015	-0.32555304	-1.80686196
H	-3.90369519	-0.92125737	-0.44648503
H	-3.33151013	1.01648935	0.87861064
H	-3.29831215	1.74292300	-0.71347016
H	-1.39819312	-1.86517866	-0.97900861
H	0.76230266	-2.43878718	-0.39394718
C	-0.87769524	2.44026860	0.33253743
H	-0.27703283	2.39117722	1.23118223
H	-0.26706906	2.82682186	-0.47366803
H	-1.69209511	3.12934783	0.50301897
C	-1.95014348	-1.86277690	1.09051064
H	-2.69753788	-2.64061018	0.97853363
H	-1.03589593	-2.31584840	1.44637653
H	-2.29559084	-1.17508229	1.85493132
H	1.66093746	1.79644315	0.16448651
C	4.28551844	0.65298150	0.06279963
H	4.25380672	1.72717416	0.19691377
H	4.83469153	0.22794564	0.89775489
H	4.85986235	0.44509056	-0.83513910

5/**6**-*E*, CASSCF/cc-pVTZ (all vibrational frequencies real)

$E(S_0)$ = -402.23158349

C	-3.00286818	0.58685280	0.00018320
C	-0.64965031	0.04398702	-0.00008282
C	-1.45809807	-1.13121347	-0.00005658
C	0.72053786	0.03502974	-0.00005653
C	1.56551628	1.21921400	-0.00002142
C	1.62389692	-1.18604040	-0.00005294
C	2.86071786	0.86959928	0.00003922
H	1.44233324	-1.79900001	0.87315948
C	3.06035315	-0.61854623	0.00006206
H	3.62166038	-0.93704870	-0.86908749
H	3.62151158	-0.93703110	0.86931497
C	-1.60027460	1.23220086	-0.00012688
H	-3.58343315	0.82478079	0.87782313
H	-1.46285603	1.85245660	-0.87358305
N	-2.70560373	-0.85923442	0.00001155
H	-3.58389829	0.82492178	-0.87710680
H	-1.46260419	1.85277552	0.87305959
H	-1.11569943	-2.14789877	-0.00007870
H	1.19087609	2.22232337	-0.00003496
H	3.67864266	1.56233380	0.00009193
H	-3.41637921	-1.55713600	0.00014806
H	1.44244216	-1.79889447	-0.87336300

5/**6**-*Z*, CASSCF/cc-pVTZ (all vibrational frequencies real)

E(S_0) = -402.22850300

C	-2.97737384	-0.63578294	-0.00061339
C	-0.64620407	-0.00538074	0.00027419
C	-1.49447324	1.13568489	0.00020225
C	0.70782889	0.03729232	0.00013951
C	1.57655715	1.23236465	-0.00000117
C	1.60137054	-1.18102070	0.00006295
C	2.86515730	0.85631010	-0.00016857
H	1.39933887	-1.79336748	0.87023140
C	3.04283265	-0.63407707	-0.00019286
H	3.60085472	-0.95878215	-0.86930350
H	3.60116932	-0.95878179	0.86871463
C	-1.55146213	-1.22650239	0.00054855
H	-3.54738100	-0.89678149	-0.87866340
H	-1.38782077	-1.84192805	0.87355899
N	-2.73372654	0.81904444	-0.00009016
H	-3.54905803	-0.89723199	0.87619782
H	-1.38688069	-1.84324740	-0.87133664
H	-1.19106796	2.16402869	0.00035032
H	1.23156805	2.24660760	0.00005720
H	3.69313069	1.53709960	-0.00027375
H	-3.46857289	1.49115904	-0.00026706
H	1.39906598	-1.79346214	-0.86997432

7-*E*, CASSCF/cc-pVTZ (all vibrational frequencies real)

$E(S_0)$ = -441.27637597

C	2.74205825	-1.94557762	-0.91802380
C	0.72290043	-0.75425453	-0.31733149
C	1.88496938	0.06059280	-0.13536517
C	-0.54519954	-0.36515079	-0.05808741
C	-1.71949201	-1.21745485	-0.17763280
C	-1.00883168	1.00796961	0.41654639
C	-2.79507411	-0.60080528	0.33446406
H	-0.40451679	1.34631372	1.24888695
C	-2.46676041	0.76080897	0.87514257
H	-3.14254697	1.51942497	0.50176729
H	-2.55393309	0.76769409	1.95562613
C	1.21197775	-2.10885478	-0.81401387
H	3.29485437	-2.61898579	-0.28216799
C	-0.94290379	2.04927677	-0.70683864
H	-1.31301895	3.00198551	-0.35172353
H	0.06832086	2.20110220	-1.06603105
H	-1.55067543	1.74553466	-1.55070925
H	0.78808804	-2.35865981	-1.77524791
N	2.96331504	-0.56241999	-0.44511488
H	3.11827363	-2.02707844	-1.92583434
H	0.95808952	-2.89956889	-0.12329365
H	1.92018558	1.07013869	0.22139254
H	-1.70522105	-2.20572528	-0.59005473
H	-3.77667671	-1.02779919	0.38837449
H	3.87015992	-0.15196076	-0.37590339

7-*Z*, CASSCF/cc-pVTZ (all vibrational frequencies real)

$E(S_0)$ = -441.27592504

C	3.12299636	-1.01051337	0.20510736
C	0.72360746	-0.79138892	-0.02333648
C	1.16402791	-2.11477643	-0.34931877
C	-0.57263263	-0.40691852	0.02022299
C	-1.74434241	-1.24208118	-0.22895983
C	-1.06543591	0.98483168	0.38324419
C	-2.86151638	-0.58782231	0.11838335
H	-0.55410433	1.33719300	1.27045259
C	-2.57460024	0.78436197	0.65662461
H	-3.17858557	1.54278198	0.17586792
H	-2.80140160	0.82728278	1.71585891
C	1.97531060	0.01555709	0.29538742
H	3.88074471	-0.75160345	-0.51714090
C	-0.82610683	1.98283995	-0.75652065
H	-1.20949395	2.95557144	-0.47832296
H	0.22498420	2.09692609	-0.98592244
H	-1.33548304	1.66650140	-1.65913710
H	1.92914376	0.45697537	1.27980313
N	2.43649870	-2.23945513	-0.24479301
H	3.59694623	-1.21364231	1.15303259
H	2.12743284	0.81204352	-0.41670757
H	0.55767743	-2.94588570	-0.64780277
H	-1.71491263	-2.24347299	-0.60858548
H	-3.85057481	-0.99607394	0.05574693
H	2.91789256	-3.09507825	-0.42331272

Total electronic energies of current state (E, in a.u.) and Cartesian coordinates (in Å) along the 2-E → 2-Z photoisomerization trajectory in Figure 2

Time = 0.00 fs
E(CASSCF/6-31G(d)) = -479.5474915

C	-0.82657351	-0.19076393	-0.06701285
C	0.49459628	0.13404613	-0.05537721
C	1.18652546	1.43278138	-0.13500064
C	1.57288578	-0.86710907	0.03523735
C	2.77603301	-0.24044191	0.01535347
C	2.52191717	1.20369959	-0.09387095
N	-1.93410213	0.63191495	-0.17382555
C	-3.15266276	-0.08355498	0.17053649
H	3.28723727	1.95141689	-0.13427799
C	-1.33308167	-1.61798837	0.05537951
C	-2.82848298	-1.51342227	-0.24765729
H	-3.00529672	-1.61966494	-1.30996407
H	-3.41733397	-2.25729005	0.27478209
H	-3.37300640	-0.01732594	1.23638049
H	-4.00143792	0.33241562	-0.36893210
H	-0.81670002	-2.29596060	-0.60344863
H	0.73074004	2.39112852	-0.21216847
C	-1.88688477	2.05824427	0.03036219
H	-1.43976265	2.32705589	0.98320731
H	-1.33055230	2.54134884	-0.76052443
H	-2.89625861	2.44771520	0.00327081
H	1.42406961	-1.92899711	0.11219866
C	4.14220132	-0.85614519	0.09519151
H	4.73315757	-0.62049154	-0.78553466
H	4.68903563	-0.48898372	0.95927995
H	4.08558132	-1.93874141	0.17539134
H	-1.17630592	-1.96860955	1.06902896

Time = 0.50 fs
E(CASSCF/6-31G(d)) = -479.5479095

C	-0.82821645	-0.19115427	-0.06707806
C	0.49613988	0.13460150	-0.05533899
C	1.18551356	1.43291332	-0.13508410
C	1.57189672	-0.86707340	0.03529458
C	2.77672494	-0.23973464	0.01530345
C	2.52288915	1.20335676	-0.09389710
N	-1.93380131	0.63164598	-0.17349002
C	-3.15282688	-0.08368687	0.17035428

H	3.28758109	1.95165393	-0.13451632
C	-1.33331757	-1.61777701	0.05535672
C	-2.82855630	-1.51332564	-0.24781216
H	-3.00531571	-1.61992404	-1.30952160
H	-3.41738597	-2.25716556	0.27509993
H	-3.37221128	-0.01713843	1.23641940
H	-4.00084105	0.33253646	-0.36895216
H	-0.81641235	-2.29634454	-0.60371396
H	0.73037852	2.39107367	-0.21236112
C	-1.88682723	2.05814654	0.03029257
H	-1.43959643	2.32609061	0.98301355
H	-1.33034168	2.54022626	-0.75989140
H	-2.89554269	2.44745945	0.00374002
H	1.42361525	-1.92960323	0.11249619
C	4.14234025	-0.85637988	0.09522922
H	4.73369063	-0.62032016	-0.78569760
H	4.68962149	-0.48849080	0.95931031
H	4.08629447	-1.93969722	0.17568000
H	-1.17611768	-1.96802018	1.06924298

Time = 1.00 fs
E(CASSCF/6-31G(d)) = -479.5491956

C	-0.83085001	-0.19177977	-0.06719373
C	0.49871009	0.13541074	-0.05531756
C	1.18380602	1.43291962	-0.13517958
C	1.57026126	-0.86718847	0.03536180
C	2.77782014	-0.23852886	0.01522805
C	2.52447953	1.20269818	-0.09388551
N	-1.93324543	0.63130431	-0.17299758
C	-3.15308188	-0.08386974	0.17007558
H	3.28815852	1.95208889	-0.13475766
C	-1.33356131	-1.61746624	0.05532271
C	-2.82862412	-1.51323110	-0.24795026
H	-3.00538645	-1.62031771	-1.30928605
H	-3.41754072	-2.25686158	0.27538153
H	-3.37094464	-0.01662524	1.23645294
H	-3.99983810	0.33271389	-0.36874797
H	-0.81576670	-2.29688113	-0.60423973
H	0.72977601	2.39143119	-0.21258661
C	-1.88680504	2.05815191	0.03018254
H	-1.43944325	2.32481608	0.98277737
H	-1.33005264	2.53890924	-0.75922272
H	-2.89490224	2.44704152	0.00421368
H	1.42305066	-1.92998610	0.11280131
C	4.14245017	-0.85662405	0.09526414
H	4.73439989	-0.62022399	-0.78588076

H	4.69037849	-0.48806841	0.95938041
H	4.08702330	-1.94052167	0.17595706
H	-1.17552688	-1.96762320	1.06985911

Time = 1.50 fs
E(CASSCF/6-31G(d)) = -479.5512456

C	-0.83441482	-0.19262502	-0.06735931
C	0.50225161	0.13645831	-0.05531279
C	1.18143297	1.43279422	-0.13528578
C	1.56800108	-0.86744239	0.03543839
C	2.77930527	-0.23684821	0.01512870
C	2.52666532	1.20174298	-0.09383813
N	-1.93244839	0.63089107	-0.17235269
C	-3.15341962	-0.08410163	0.16970656
H	3.28897631	1.95270754	-0.13500077
C	-1.33381137	-1.61706666	0.05527661
C	-2.82868928	-1.51314140	-0.24806817
H	-3.00551178	-1.62084578	-1.30928315
H	-3.41779374	-2.25637835	0.27561969
H	-3.36923705	-0.01580529	1.23647077
H	-3.99850110	0.33297009	-0.36835788
H	-0.81481156	-2.29750107	-0.60496331
H	0.72891784	2.39219848	-0.21284417
C	-1.88681381	2.05825087	0.03003767
H	-1.43929417	2.32326490	0.98252295
H	-1.32965495	2.53745056	-0.75858033
H	-2.89439119	2.44648170	0.00468095
H	1.42236314	-1.93012273	0.11311070
C	4.14253799	-0.85687682	0.09529651
H	4.73526248	-0.62022069	-0.78605214
H	4.69128582	-0.48773948	0.95945982
H	4.08775489	-1.94116310	0.17621834
H	-1.17454851	-1.96740511	1.07082778

Time = 2.00 fs
E(CASSCF/6-31G(d)) = -479.5538910

C	-0.83883047	-0.19367014	-0.06757378
C	0.50668905	0.13772318	-0.05532447
C	1.17843561	1.43253252	-0.13540124
C	1.56514782	-0.86781544	0.03552326
C	2.78116171	-0.23472618	0.01500744
C	2.52941476	1.20051869	-0.09375770
N	-1.93143016	0.63040847	-0.17156145
C	-3.15382860	-0.08437991	0.16925565
H	3.29003927	1.95348622	-0.13524386

C	-1.33406604	-1.61659287	0.05521892
C	-2.82875551	-1.51305961	-0.24816439
H	-3.00568994	-1.62150507	-1.30951728
H	-3.41814341	-2.25572403	0.27581117
H	-3.36712911	-0.01470552	1.23646138
H	-3.99692914	0.33333585	-0.36783742
H	-0.81361054	-2.29811561	-0.60580533
H	0.72779630	2.39334022	-0.21313040
C	-1.88684815	2.05843179	0.02986407
H	-1.43914036	2.32148038	0.98227765
H	-1.32912067	2.53590915	-0.75802791
H	-2.89406106	2.44580097	0.00512997
H	1.42152876	-1.93002357	0.11342299
C	4.14261250	-0.85713578	0.09532653
H	4.73625371	-0.62032874	-0.78617689
H	4.69232112	-0.48752825	0.95951517
H	4.08847083	-1.94158395	0.17646052
H	-1.17320614	-1.96733984	1.07206559

Time = 2.50 fs
E(CASSCF/6-31G(d)) = -479.5569300

C	-0.84399902	-0.19489236	-0.06783581
C	0.51192885	0.13917981	-0.05535249
C	1.17486754	1.43213199	-0.13552437
C	1.56174495	-0.86828044	0.03561502
C	2.78336432	-0.23220666	0.01486691
C	2.53268639	1.19906143	-0.09364779
N	-1.93021647	0.62986039	-0.17063081
C	-3.15429481	-0.08470129	0.16873258
H	3.29134990	1.95439314	-0.13548466
C	-1.33432390	-1.61606248	0.05515160
C	-2.82882726	-1.51298914	-0.24823936
H	-3.00591489	-1.62228938	-1.30997138
H	-3.41859178	-2.25491433	0.27595694
H	-3.36466860	-0.01335839	1.23641306
H	-3.99524033	0.33384830	-0.36725426
H	-0.81223659	-2.29862519	-0.60667822
H	0.72641109	2.39478846	-0.21343936
C	-1.88690215	2.05868171	0.02966767
H	-1.43897397	2.31951287	0.98206921
H	-1.32842764	2.53434486	-0.75762479
H	-2.89395551	2.44501889	0.00554832
H	1.42051309	-1.92972892	0.11373877
C	4.14268402	-0.85739709	0.09535436
H	4.73734901	-0.62056644	-0.78622056
H	4.69346212	-0.48745887	0.95951338

H	4.08914748	-1.94176320	0.17668140
H	-1.17153078	-1.96739231	1.07346358

Time = 3.00 fs
E(CASSCF/6-31G(d)) = -479.5601161

C	-0.84980690	-0.19626774	-0.06814375
C	0.51786126	0.14079873	-0.05539677
C	1.17079514	1.43159313	-0.13565362
C	1.55784837	-0.86880427	0.03571209
C	2.78588135	-0.22934364	0.01471036
C	2.53642890	1.19741524	-0.09351268
N	-1.92883901	0.62925308	-0.16956867
C	-3.15480282	-0.08506170	0.16814790
H	3.29290955	1.95539020	-0.13572054
C	-1.33458416	-1.61549507	0.05507797
C	-2.82890964	-1.51293382	-0.24829542
H	-3.00617660	-1.62318961	-1.31060795
H	-3.41914384	-2.25397120	0.27606162
H	-3.36190991	-0.01180141	1.23631451
H	-3.99356249	0.33454677	-0.36668221
H	-0.81076669	-2.29892789	-0.60749393
H	0.72476692	2.39644761	-0.21376320
C	-1.88696946	2.05898732	0.02945410
H	-1.43878931	2.31741873	0.98192304
H	-1.32756291	2.53281471	-0.75742003
H	-2.89410649	2.44415213	0.00592337
H	1.41927175	-1.92930570	0.11406040
C	4.14276386	-0.85765561	0.09538008
H	4.73852608	-0.62095035	-0.78615263
H	4.69468867	-0.48755319	0.95942502
H	4.08975685	-1.94169884	0.17688010
H	-1.16956008	-1.96752190	1.07489617

Time = 3.50 fs
E(CASSCF/6-31G(d)) = -479.5631850

C	-0.85612825	-0.19777223	-0.06849559
C	0.52436335	0.14254735	-0.05545730
C	1.16629714	1.43092005	-0.13578746
C	1.55352625	-0.86934974	0.03581280
C	2.78867453	-0.22620037	0.01454161
C	2.54058160	1.19563118	-0.09335731
N	-1.92733504	0.62859559	-0.16838372
C	-3.15533690	-0.08545616	0.16751221
H	3.29471854	1.95643448	-0.13594859
C	-1.33484721	-1.61491121	0.05500249

C	-2.82900821	-1.51289790	-0.24833643
H	-3.00646198	-1.62419414	-1.31137281
H	-3.41980751	-2.25292189	0.27613320
H	-3.35891260	-0.01007564	1.23615587
H	-3.99202254	0.33546847	-0.36619437
H	-0.80927553	-2.29892887	-0.60817259
H	0.72287196	2.39819963	-0.21409257
C	-1.88704361	2.05933551	0.02922829
H	-1.43858380	2.31525808	0.98185998
H	-1.32652577	2.53136917	-0.75744746
H	-2.89453033	2.44321311	0.00624275
H	1.41775320	-1.92883659	0.11439148
C	4.14286386	-0.85790515	0.09540369
H	4.73976574	-0.62149427	-0.78594884
H	4.69598335	-0.48782932	0.95922666
H	4.09026689	-1.94140697	0.17705701
H	-1.16733646	-1.96768704	1.07623312

Time = 4.00 fs
E(CASSCF/6-31G(d)) = -479.5659014

C	-0.86282955	-0.19938286	-0.06888913
C	0.53130188	0.14439089	-0.05553423
C	1.16146331	1.43012094	-0.13592451
C	1.54885824	-0.86987782	0.03591569
C	2.79169975	-0.22284854	0.01436495
C	2.54507586	1.19376674	-0.09318721
N	-1.92574656	0.62789975	-0.16708496
C	-3.15588187	-0.08587846	0.16683527
H	3.29677601	1.95748086	-0.13616583
C	-1.33511493	-1.61433119	0.05493046
C	-2.82912884	-1.51288593	-0.24836738
H	-3.00675594	-1.62528952	-1.31220045
H	-3.42059287	-2.25179755	0.27618225
H	-3.35573991	-0.00822427	1.23592962
H	-3.99073507	0.33664364	-0.36585608
H	-0.80782934	-2.29854816	-0.60865028
H	0.72073677	2.39991072	-0.21441718
C	-1.88711811	2.05971373	0.02899414
H	-1.43835876	2.31309245	0.98189440
H	-1.32532973	2.53004883	-0.75772250
H	-2.89522474	2.44220899	0.00649460
H	1.41590221	-1.92840900	0.11473623
C	4.14299610	-0.85813905	0.09542513
H	4.74105382	-0.62220889	-0.78559395
H	4.69733336	-0.48830083	0.95890364
H	4.09064267	-1.94092007	0.17721368

H	-1.16490489	-1.96784880	1.07735073

Time = 4.50 fs
E(CASSCF/6-31G(d)) = -479.5680618

C	-0.86977206	-0.20107869	-0.06932198
C	0.53853616	0.14629297	-0.05562778
C	1.15639293	1.42920863	-0.13606358
C	1.54393395	-0.87035018	0.03601953
C	2.79490806	-0.21936636	0.01418509
C	2.54983655	1.19188402	-0.09300836
N	-1.92411972	0.62718046	-0.16568177
C	-3.15642434	-0.08632105	0.16612557
H	3.29908112	1.95848427	-0.13636933
C	-1.33539095	-1.61377420	0.05486763
C	-2.82927764	-1.51290277	-0.24839381
H	-3.00704259	-1.62646109	-1.31301978
H	-3.42151087	-2.25063112	0.27622078
H	-3.35245804	-0.00629188	1.23563080
H	-3.98979225	0.33809084	-0.36571847
H	-0.80648188	-2.29772694	-0.60888434
H	0.71837077	2.40144094	-0.21472661
C	-1.88718663	2.06011047	0.02875459
H	-1.43812002	2.31098335	0.98203234
H	-1.32400327	2.52888259	-0.75824026
H	-2.89616830	2.44114146	0.00666787
H	1.41366232	-1.92810417	0.11509871
C	4.14317236	-0.85835049	0.09544430
H	4.74238207	-0.62310088	-0.78508363
H	4.69873118	-0.48897555	0.95845184
H	4.09084751	-1.94028442	0.17735270
H	-1.16231139	-1.96797445	1.07814149

Time = 5.00 fs
E(CASSCF/6-31G(d)) = -479.5695030

C	-0.87681548	-0.20284118	-0.06979162
C	0.54592179	0.14821644	-0.05573826
C	1.15119191	1.42820040	-0.13620371
C	1.53885023	-0.87073170	0.03612356
C	2.79824740	-0.21583613	0.01400701
C	2.55478445	1.19004779	-0.09282697
N	-1.92250362	0.62645535	-0.16418376
C	-3.15695358	-0.08677495	0.16538976
H	3.30163280	1.95940199	-0.13655643
C	-1.33568076	-1.61325772	0.05481984
C	-2.82946088	-1.51295351	-0.24842114

H	-3.00730679	-1.62769421	-1.31376056
H	-3.42257231	-2.24945571	0.27626138
H	-3.34913576	-0.00432365	1.23525765
H	-3.98925595	0.33981306	-0.36581322
H	-0.80527069	-2.29643276	-0.60885765
H	0.71578077	2.40265336	-0.21501101
C	-1.88724313	2.06051564	0.02851159
H	-1.43787813	2.30899120	0.98227075
H	-1.32258930	2.52788731	-0.75897573
H	-2.89732109	2.44000730	0.00675254
H	1.41098007	-1.92798445	0.11548196
C	4.14340377	-0.85853304	0.09546105
H	4.74374828	-0.62417293	-0.78442501
H	4.70017480	-0.48985539	0.95787829
H	4.09084410	-1.93955590	0.17747737
H	-1.15960117	-1.96804076	1.07852296

Time = 5.50 fs
E(CASSCF/6-31G(d)) = -479.5701424

C	-0.88381984	-0.20465460	-0.07029545
C	0.55331219	0.15012362	-0.05586604
C	1.14597010	1.42711802	-0.13634421
C	1.53370838	-0.87099281	0.03622752
C	2.80166454	-0.21234225	0.01383584
C	2.55983870	1.18832425	-0.09264940
N	-1.92094997	0.62574462	-0.16260098
C	-3.15746179	-0.08722950	0.16463271
H	3.30443102	1.96019529	-0.13672471
C	-1.33599175	-1.61279666	0.05479279
C	-2.82968473	-1.51304340	-0.24845434
H	-3.00753422	-1.62897407	-1.31435936
H	-3.42378602	-2.24830247	0.27631568
H	-3.34584305	-0.00236529	1.23481167
H	-3.98915230	0.34179484	-0.36614993
H	-0.80421649	-2.29466080	-0.60858025
H	0.71296844	2.40342218	-0.21526162
C	-1.88728191	2.06091998	0.02826634
H	-1.43764720	2.30717433	0.98259905
H	-1.32114201	2.52706882	-0.75988762
H	-2.89862833	2.43880038	0.00673996
H	1.40780729	-1.92808501	0.11588738
C	4.14370070	-0.85868127	0.09547525
H	4.74515629	-0.62542291	-0.78363697
H	4.70166762	-0.49093556	0.95720138
H	4.09059662	-1.93879559	0.17759146
H	-1.15681824	-1.96803459	1.07844259

Time = 6.00 fs
E(CASSCF/6-31G(d)) = -479.5699560

C	-0.89064896	-0.20650587	-0.07083087
C	0.56056263	0.15197730	-0.05601150
C	1.14083804	1.42598691	-0.13648465
C	1.52861115	-0.87111114	0.03633173
C	2.80510675	-0.20896843	0.01367673
C	2.56491921	1.18677855	-0.09248189
N	-1.91951127	0.62507044	-0.16094369
C	-3.15794450	-0.08767256	0.16385733
H	3.30747672	1.96083215	-0.13687235
C	-1.33633286	-1.61240325	0.05479164
C	-2.82995527	-1.51317765	-0.24849722
H	-3.00771327	-1.63028693	-1.31476485
H	-3.42515784	-2.24719872	0.27639360
H	-3.34265075	-0.00046205	1.23429661
H	-3.98946965	0.34400145	-0.36671476
H	-0.80332241	-2.29243563	-0.60808924
H	0.70992908	2.40364330	-0.21547166
C	-1.88729780	2.06131529	0.02801953
H	-1.43744533	2.30558766	0.98299803
H	-1.31972471	2.52642278	-0.76092095
H	-2.90002333	2.43751220	0.00662292
H	1.40410492	-1.92840625	0.11631411
C	4.14407232	-0.85879108	0.09548681
H	4.74661533	-0.62684479	-0.78274896
H	4.70321793	-0.49220560	0.95644975
H	4.09007183	-1.93806426	0.17769876
H	-1.15400412	-1.96795538	1.07788211

Time = 6.50 fs
E(CASSCF/6-31G(d)) = -479.5689579

C	-0.89717088	-0.20838408	-0.07139524
C	0.56753161	0.15374096	-0.05617496
C	1.13590422	1.42483532	-0.13662486
C	1.52365999	-0.87107247	0.03643713
C	2.80852361	-0.20579582	0.01353474
C	2.56994877	1.18547343	-0.09233046
N	-1.91824007	0.62445642	-0.15922279
C	-3.15840064	-0.08809068	0.16306492
H	3.31077130	1.96128711	-0.13699801
C	-1.33671443	-1.61208703	0.05482070
C	-2.83027849	-1.51336151	-0.24855208
H	-3.00783577	-1.63162148	-1.31494269

H	-3.42668963	-2.24616767	0.27650259
H	-3.33963142	0.00134045	1.23372020
H	-3.99016257	0.34637957	-0.36747226
H	-0.80257496	-2.28981017	-0.60744705
H	0.70665158	2.40323818	-0.21563642
C	-1.88728622	2.06169420	0.02777174
H	-1.43729258	2.30428384	0.98344341
H	-1.31840461	2.52593893	-0.76201370
H	-2.90143294	2.43613587	0.00639584
H	1.39984507	-1.92890838	0.11675846
C	4.14452647	-0.85886014	0.09549570
H	4.74813831	-0.62842869	-0.78179914
H	4.70483731	-0.49364976	0.95566033
H	4.08924058	-1.93741655	0.17780271
H	-1.15119723	-1.96781553	1.07685882

Time = 7.00 fs
E(CASSCF/6-31G(d)) = -479.5672878

C	-0.90326247	-0.21027998	-0.07198617
C	0.57408459	0.15537993	-0.05635674
C	1.13127272	1.42369304	-0.13676488
C	1.51895278	-0.87087087	0.03654520
C	2.81186751	-0.20290023	0.01341462
C	2.57485452	1.18446682	-0.09220070
N	-1.91718665	0.62392639	-0.15744916
C	-3.15883211	-0.08846964	0.16225542
H	3.31431697	1.96154449	-0.13710114
C	-1.33714765	-1.61185487	0.05488320
C	-2.83066010	-1.51359991	-0.24861944
H	-3.00789742	-1.63296850	-1.31487780
H	-3.42837847	-2.24522629	0.27664684
H	-3.33685646	0.00299809	1.23309139
H	-3.99115560	0.34886050	-0.36836905
H	-0.80194727	-2.28686160	-0.60673685
H	0.70311786	2.40216251	-0.21575400
C	-1.88724339	2.06204999	0.02752357
H	-1.43721066	2.30331060	0.98390595
H	-1.31724906	2.52560184	-0.76310143
H	-2.90278286	2.43466650	0.00605486
H	1.39501301	-1.92951627	0.11721441
C	4.14506970	-0.85888828	0.09550202
H	4.74974082	-0.63016194	-0.78083219
H	4.70653970	-0.49524833	0.95487630
H	4.08807935	-1.93689691	0.17790611
H	-1.14843235	-1.96763991	1.07542397

Time = 7.50 fs
E(CASSCF/6-31G(d)) = -479.5651034

C	-0.90881115	-0.21218503	-0.07260166
C	0.58009624	0.15686207	-0.05655716
C	1.12704073	1.42259000	-0.13690484
C	1.51458132	-0.87050823	0.03665792
C	2.81509456	-0.20035029	0.01332071
C	2.57956944	1.18381047	-0.09209768
N	-1.91639748	0.62350334	-0.15563353
C	-3.15924324	-0.08879488	0.16142803
H	3.31811636	1.96159910	-0.13718196
C	-1.33764389	-1.61171129	0.05498113
C	-2.83110537	-1.51389734	-0.24869793
H	-3.00789830	-1.63432133	-1.31457619
H	-3.43021650	-2.24438501	0.27682707
H	-3.33439545	0.00446881	1.23241984
H	-3.99235162	0.35136482	-0.36933949
H	-0.80140298	-2.28368595	-0.60605664
H	0.69930338	2.40040952	-0.21582542
C	-1.88716633	2.06237633	0.02727604
H	-1.43722173	2.30271029	0.98435338
H	-1.31632104	2.52539334	-0.76412293
H	-2.90400278	2.43310295	0.00559767
H	1.38960779	-1.93012625	0.11767402
C	4.14570746	-0.85887765	0.09550596
H	4.75143927	-0.63202994	-0.77989606
H	4.70833971	-0.49697910	0.95414395
H	4.08657191	-1.93653555	0.17801085
H	-1.14574051	-1.96746416	1.07365900

Time = 8.00 fs
E(CASSCF/6-31G(d)) = -479.5625841

C	-0.91371650	-0.21409020	-0.07324005
C	0.58545212	0.15815838	-0.05677646
C	1.12329673	1.42155500	-0.13704497
C	1.51062971	-0.86999311	0.03677763
C	2.81816521	-0.19820556	0.01325684
C	2.58403327	1.18354823	-0.09202575
N	-1.91591338	0.62320798	-0.15378647
C	-3.15964019	-0.08905228	0.16058176
H	3.32217121	1.96145545	-0.13724145
C	-1.33821427	-1.61165904	0.05511494
C	-2.83161917	-1.51425773	-0.24878436
H	-3.00784267	-1.63567655	-1.31406449
H	-3.43219081	-2.24364836	0.27704070

H	-3.33231560	0.00571251	1.23171606
H	-3.99364308	0.35380743	-0.37031241
H	-0.80090072	-2.28039159	-0.60551209
H	0.69517988	2.39800801	-0.21585440
C	-1.88705315	2.06266731	0.02703072
H	-1.43734619	2.30252001	0.98475396
H	-1.31567393	2.52529632	-0.76502666
H	-2.90503204	2.43145101	0.00502377
H	1.38364323	-1.93061286	0.11812784
C	4.14644406	-0.85883281	0.09550789
H	4.75324860	-0.63401704	-0.77903857
H	4.71025047	-0.49881874	0.95350919
H	4.08471033	-1.93634574	0.17811769
H	-1.14314889	-1.96733247	1.07166964

Time = 8.50 fs
E(CASSCF/6-31G(d)) = -479.5599581

C	-0.91789263	-0.21598501	-0.07390019
C	0.59005096	0.15924375	-0.05701490
C	1.12011874	1.42061431	-0.13718552
C	1.50717268	-0.86933910	0.03690687
C	2.82104431	-0.19651482	0.01322615
C	2.58819326	1.18371463	-0.09198847
N	-1.91576765	0.62305737	-0.15191803
C	-3.16003026	-0.08922881	0.15971611
H	3.32648135	1.96112799	-0.13728134
C	-1.33886881	-1.61169960	0.05528350
C	-2.83220584	-1.51468418	-0.24887395
H	-3.00773863	-1.63703372	-1.31338777
H	-3.43428380	-2.24301520	0.27728215
H	-3.33068016	0.00669207	1.23099011
H	-3.99492201	0.35610349	-0.37121811
H	-0.80039994	-2.27709111	-0.60520785
H	0.69071778	2.39502065	-0.21584720
C	-1.88690310	2.06291746	0.02678992
H	-1.43760145	2.30277051	0.98507826
H	-1.31534802	2.52529617	-0.76577497
H	-2.90582381	2.42972425	0.00433438
H	1.37714790	-1.93084155	0.11856586
C	4.14728290	-0.85876057	0.09550831
H	4.75518046	-0.63610764	-0.77830404
H	4.71228197	-0.50074442	0.95301434
H	4.08249613	-1.93632321	0.17822630
H	-1.14068105	-1.96729440	1.06957783

Time = 9.00 fs

E(CASSCF/6-31G(d)) = -479.5574631

C	-0.92127053	-0.21785629	-0.07458157
C	0.59380679	0.16009768	-0.05727274
C	1.11757291	1.41979059	-0.13732668
C	1.50427411	-0.86856301	0.03704822
C	2.82370119	-0.19531426	0.01323098
C	2.59200470	1.18433330	-0.09198846
N	-1.91598416	0.62306355	-0.15003721
C	-3.16042119	-0.08931324	0.15883148
H	3.33104358	1.96064050	-0.13730413
C	-1.33961577	-1.61183388	0.05548406
C	-2.83286904	-1.51517877	-0.24896066
H	-3.00759776	-1.63839493	-1.31260570
H	-3.43647422	-2.24247923	0.27754351
H	-3.32954635	0.00737474	1.23024966
H	-3.99608935	0.35817454	-0.37199494
H	-0.79986706	-2.27389306	-0.60523862
H	0.68588913	2.39154059	-0.21581230
C	-1.88671669	2.06312185	0.02655663
H	-1.43800130	2.30348451	0.98529996
H	-1.31536849	2.52538109	-0.76634646
H	-2.90634819	2.42794390	0.00353230
H	1.37016472	-1.93068340	0.11897864
C	4.14822661	-0.85866980	0.09550787
H	4.75724146	-0.63828711	-0.77773016
H	4.71443947	-0.50273534	0.95269503
H	4.07994100	-1.93644714	0.17833528
H	-1.13835749	-1.96740165	1.06751249

Time = 9.50 fs
E(CASSCF/6-31G(d)) = -479.5553035

C	-0.92379902	-0.21968757	-0.07528425
C	0.59665048	0.16070473	-0.05755023
C	1.11571232	1.41910194	-0.13746858
C	1.50198604	-0.86768293	0.03720415
C	2.82610961	-0.19462663	0.01327281
C	2.59543126	1.18541634	-0.09202734
N	-1.91657620	0.62323245	-0.14815198
C	-3.16082062	-0.08929683	0.15792969
H	3.33584948	1.96002408	-0.13731295
C	-1.34046093	-1.61206289	0.05571232
C	-2.83361171	-1.51574236	-0.24903777
H	-3.00743426	-1.63976439	-1.31178764
H	-3.43873810	-2.24203054	0.27781546
H	-3.32896458	0.00773250	1.22949995

H	-3.99706395	0.35995346	-0.37259503
H	-0.79928089	-2.27089417	-0.60568079
H	0.68067211	2.38768289	-0.21575968
C	-1.88649574	2.06327629	0.02633452
H	-1.43855462	2.30467680	0.98539811
H	-1.31574356	2.52554319	-0.76673809
H	-2.90659416	2.42613952	0.00262195
H	1.36275048	-1.93002670	0.11935818
C	4.14927722	-0.85857102	0.09550734
H	4.75943162	-0.64054246	-0.77734522
H	4.71672203	-0.50477381	0.95257741
H	4.07706727	-1.93668257	0.17844235
H	-1.13619651	-1.96770392	1.06559936

Time = 10.00 fs
E(CASSCF/6-31G(d)) = -479.5536635

C	-0.92544579	-0.22145848	-0.07600887
C	0.59853097	0.16105492	-0.05784761
C	1.11457602	1.41856152	-0.13761129
C	1.50034766	-0.86671645	0.03737679
C	2.82824784	-0.19446050	0.01335220
C	2.59844524	1.18696377	-0.09210571
N	-1.91754563	0.62356324	-0.14626897
C	-3.16123559	-0.08917373	0.15701428
H	3.34088357	1.95931491	-0.13731145
C	-1.34140702	-1.61238827	0.05596264
C	-2.83443597	-1.51637444	-0.24909841
H	-3.00726406	-1.64114772	-1.31100659
H	-3.44105010	-2.24165685	0.27808823
H	-3.32897704	0.00774297	1.22874276
H	-3.99778887	0.36138881	-0.37298874
H	-0.79863753	-2.26817270	-0.60658518
H	0.67505540	2.38357581	-0.21570011
C	-1.88624352	2.06337749	0.02612779
H	-1.43926497	2.30635323	0.98535770
H	-1.31646458	2.52577796	-0.76696505
H	-2.90656990	2.42434803	0.00160922
H	1.35497475	-1.92878879	0.11969885
C	4.15043620	-0.85847593	0.09550759
H	4.76174334	-0.64286282	-0.77716610
H	4.71912161	-0.50684612	0.95267617
H	4.07390801	-1.93698425	0.17854471
H	-1.13421526	-1.96824525	1.06395075

Time = 10.50 fs
E(CASSCF/6-31G(d)) = -479.5526657

C	-0.92619766	-0.22314479	-0.07675638
C	0.59941621	0.16114387	-0.05816516
C	1.11418830	1.41817737	-0.13775479
C	1.49938475	-0.86567922	0.03756788
C	2.83009869	-0.19481017	0.01346879
C	2.60102759	1.18896362	-0.09222311
N	-1.91888241	0.62404784	-0.14439342
C	-3.16167226	-0.08894149	0.15609065
H	3.34612107	1.95855171	-0.13730354
C	-1.34245333	-1.61281261	0.05622830
C	-2.83534309	-1.51707301	-0.24913620
H	-3.00710379	-1.64255128	-1.31033285
H	-3.44338490	-2.24134528	0.27835280
H	-3.32961685	0.00738998	1.22797675
H	-3.99823634	0.36244778	-0.37316770
H	-0.79795313	-2.26578401	-0.60797202
H	0.66904406	2.37934808	-0.21564408
C	-1.88596469	2.06342339	0.02594096
H	-1.44012987	2.30851073	0.98517088
H	-1.31750664	2.52608383	-0.76705973
H	-2.90630181	2.42261312	0.00050137
H	1.34691883	-1.92692270	0.11999780
C	4.15170453	-0.85839690	0.09550954
H	4.76416097	-0.64523968	-0.77719719
H	4.72162264	-0.50894295	0.95299350
H	4.07050656	-1.93730136	0.17863932
H	-1.13243057	-1.96906016	1.06265619

Time = 11.00 fs
E(CASSCF/6-31G(d)) = -479.5523784

C	-0.92606017	-0.22471872	-0.07752812
C	0.59929328	0.16097283	-0.05850311
C	1.11455818	1.41795287	-0.13789908
C	1.49910933	-0.86458404	0.03777862
C	2.83164962	-0.19565597	0.01362129
C	2.60316798	1.19139237	-0.09237807
N	-1.92056488	0.62467118	-0.14252925
C	-3.16213557	-0.08860115	0.15516608
H	3.35152584	1.95777253	-0.13729341
C	-1.34359545	-1.61333959	0.05650197
C	-2.83633341	-1.51783445	-0.24914587
H	-3.00696966	-1.64398144	-1.30982775
H	-3.44571877	-2.24108396	0.27860192
H	-3.33090747	0.00666388	1.22719763
H	-3.99840982	0.36311780	-0.37314611

H	-0.79726498	-2.26375803	-0.60982872
H	0.66266420	2.37511885	-0.21560098
C	-1.88566542	2.06341325	0.02577857
H	-1.44114066	2.31113721	0.98483716
H	-1.31883069	2.52646094	-0.76706915
H	-2.90583188	2.42098356	-0.00069303
H	1.33867442	-1.92442297	0.12025562
C	4.15308259	-0.85834636	0.09551415
H	4.76666112	-0.64766686	-0.77743029
H	4.72420231	-0.51105939	0.95351895
H	4.06691561	-1.93758250	0.17872332
H	-1.13086007	-1.97017130	1.06177515

Time = 11.50 fs
E(CASSCF/6-31G(d)) = -479.5528017

C	-0.92505705	-0.22614995	-0.07832527
C	0.59816923	0.16054844	-0.05886168
C	1.11567915	1.41788711	-0.13804421
C	1.49951920	-0.86343997	0.03800963
C	2.83289289	-0.19696516	0.01380757
C	2.60486460	1.19421595	-0.09256819
N	-1.92256046	0.62541182	-0.14067896
C	-3.16262943	-0.08815739	0.15424952
H	3.35704882	1.95701333	-0.13728512
C	-1.34482526	-1.61397387	0.05677596
C	-2.83740635	-1.51865364	-0.24912377
H	-3.00687711	-1.64544376	-1.30953818
H	-3.44803068	-2.24086339	0.27883106
H	-3.33286210	0.00556254	1.22639877
H	-3.99834351	0.36340705	-0.37296052
H	-0.79663022	-2.26210134	-0.61211062
H	0.65596833	2.37098643	-0.21557816
C	-1.88535325	2.06334812	0.02564490
H	-1.44228296	2.31421144	0.98436292
H	-1.32038668	2.52690888	-0.76705068
H	-2.90521399	2.41951124	-0.00196428
H	1.33034289	-1.92132633	0.12047615
C	4.15457008	-0.85833646	0.09552237
H	4.76921360	-0.65014007	-0.77784564
H	4.72683157	-0.51319436	0.95423041
H	4.06319641	-1.93778148	0.17879441
H	-1.12952265	-1.97158717	1.06133232

Time = 12.00 fs
E(CASSCF/6-31G(d)) = -479.5538642

C	-0.92322867	-0.22740691	-0.07914893
C	0.59607048	0.15988261	-0.05924098
C	1.11752942	1.41797545	-0.13819028
C	1.50059840	-0.86225238	0.03826089
C	2.83382567	-0.19869301	0.01402474
C	2.60612378	1.19739078	-0.09279019
N	-1.92482683	0.62624285	-0.13884405
C	-3.16315677	-0.08761842	0.15335141
H	3.36262714	1.95630488	-0.13728268
C	-1.34613120	-1.61472057	0.05704290
C	-2.83856039	-1.51952396	-0.24906830
H	-3.00683967	-1.64694231	-1.30949196
H	-3.45030315	-2.24067749	0.27903891
H	-3.33548378	0.00409098	1.22557194
H	-3.99809975	0.36334290	-0.37266775
H	-0.79612268	-2.26079867	-0.61474405
H	0.64903864	2.36702091	-0.21558028
C	-1.88503709	2.06323100	0.02554365
H	-1.44353677	2.31770381	0.98376104
H	-1.32211694	2.52742542	-0.76706766
H	-2.90450952	2.41824919	-0.00330158
H	1.32203368	-1.91770876	0.12066648
C	4.15616571	-0.85837846	0.09553505
H	4.77178274	-0.65265621	-0.77841358
H	4.72947624	-0.51534969	0.95509583
H	4.05941703	-1.93786134	0.17885117
H	-1.12843931	-1.97330153	1.06131549

Time = 12.50 fs
E(CASSCF/6-31G(d)) = -479.5554585

C	-0.92063016	-0.22845821	-0.07999938
C	0.59304169	0.15899183	-0.05964111
C	1.12007245	1.41821053	-0.13833764
C	1.50231767	-0.86102335	0.03853178
C	2.83445041	-0.20078503	0.01426929
C	2.60695971	1.20086584	-0.09304011
N	-1.92731401	0.62713359	-0.13702505
C	-3.16371956	-0.08699556	0.15248317
H	3.36818457	1.95567190	-0.13728960
C	-1.34749873	-1.61558479	0.05729592
C	-2.83979298	-1.52043735	-0.24898016
H	-3.00686861	-1.64847883	-1.30969493
H	-3.45252319	-2.24052384	0.27922781
H	-3.33876530	0.00226169	1.22470774
H	-3.99776273	0.36296951	-0.37234128
H	-0.79582868	-2.25981957	-0.61763280

H	0.64198722	2.36326171	-0.21560926
C	-1.88472703	2.06306661	0.02547762
H	-1.44487790	2.32157692	0.98304945
H	-1.32396113	2.52800337	-0.76718347
H	-2.90378170	2.41724802	-0.00469332
H	1.31386270	-1.91368153	0.12083624
C	4.15786695	-0.85848244	0.09555300
H	4.77432938	-0.65521261	-0.77909721
H	4.73209913	-0.51752899	0.95607572
H	4.05565079	-1.93779878	0.17889333
H	-1.12763306	-1.97529335	1.06167669

Time = 13.00 fs
E(CASSCF/6-31G(d)) = -479.5574138

C	-0.91732984	-0.22927427	-0.08087639
C	0.58914522	0.15789684	-0.06006198
C	1.12325754	1.41858277	-0.13848682
C	1.50463489	-0.85975252	0.03882114
C	2.83477507	-0.20317821	0.01453720
C	2.60739399	1.20458387	-0.09331336
N	-1.92996578	0.62805085	-0.13522175
C	-3.16431935	-0.08630291	0.15165666
H	3.37363184	1.95513198	-0.13730905
C	-1.34891102	-1.61657076	0.05752907
C	-2.84110064	-1.52138450	-0.24886234
H	-3.00697282	-1.65005285	-1.31013057
H	-3.45468243	-2.24040440	0.27940387
H	-3.34268987	0.00009433	1.22379738
H	-3.99743218	0.36234388	-0.37206648
H	-0.79583983	-2.25912412	-0.62066585
H	0.63495677	2.35971621	-0.21566397
C	-1.88443424	2.06286173	0.02544835
H	-1.44627830	2.32578635	0.98225032
H	-1.32585952	2.52862963	-0.76745706
H	-2.90309049	2.41655458	-0.00612707
H	1.30595199	-1.90938062	0.12099716
C	4.15966971	-0.85865702	0.09557688
H	4.77681312	-0.65780622	-0.77985546
H	4.73466204	-0.51973626	0.95712631
H	4.05197412	-1.93758644	0.17892196
H	-1.12712824	-1.97752913	1.06233741

Time = 13.50 fs
E(CASSCF/6-31G(d)) = -479.5595526

C	-0.91340687	-0.22982903	-0.08177882

C	0.58445950	0.15662205	-0.06050334
C	1.12702113	1.41908085	-0.13863860
C	1.50749599	-0.85843823	0.03912741
C	2.83481336	-0.20580336	0.01482410
C	2.60745500	1.20848331	-0.09360488
N	-1.93272203	0.62896073	-0.13343339
C	-3.16495766	-0.08555676	0.15088360
H	3.37886919	1.95469622	-0.13734380
C	-1.35034967	-1.61768110	0.05773752
C	-2.84247897	-1.52235506	-0.24872001
H	-3.00715899	-1.65166139	-1.31076130
H	-3.45677707	-2.24032508	0.27957663
H	-3.34723174	-0.00238447	1.22283343
H	-3.99721390	0.36153093	-0.37193453
H	-0.79624671	-2.25867021	-0.62372611
H	0.62811655	2.35636516	-0.21574073
C	-1.88417077	2.06262500	0.02545599
H	-1.44770743	2.33028175	0.98138813
H	-1.32775717	2.52928329	-0.76793758
H	-2.90248761	2.41620917	-0.00758973
H	1.29842882	-1.90495676	0.12116206
C	4.16156802	-0.85890920	0.09560723
H	4.77919468	-0.66043266	-0.78064640
H	4.73712799	-0.52197441	0.95820278
H	4.04846467	-1.93723436	0.17893951
H	-1.12694975	-1.97996529	1.06319571

Time = 14.00 fs
E(CASSCF/6-31G(d)) = -479.5616890

C	-0.90894919	-0.23010146	-0.08270464
C	0.57907739	0.15519489	-0.06096471
C	1.13128834	1.41969216	-0.13879400
C	1.51083604	-0.85707883	0.03944865
C	2.83458494	-0.20858709	0.01512541
C	2.60717722	1.21250003	-0.09390930
N	-1.93552084	0.62983022	-0.13165892
C	-3.16563652	-0.08477486	0.15017487
H	3.38378980	1.95437051	-0.13739625
C	-1.35179556	-1.61891605	0.05791753
C	-2.84392267	-1.52333787	-0.24856012
H	-3.00743209	-1.65329916	-1.31153178
H	-3.45880736	-2.24029499	0.27975843
H	-3.35235711	-0.00514171	1.22181073
H	-3.99720961	0.36059806	-0.37203564
H	-0.79713218	-2.25842042	-0.62669925
H	0.62165626	2.35317086	-0.21583394

C	-1.88394925	2.06236682	0.02549912
H	-1.44913355	2.33500792	0.98048786
H	-1.32960707	2.52993469	-0.76866036
H	-2.90201232	2.41624355	-0.00906771
H	1.29142499	-1.90056350	0.12134401
C	4.16355379	-0.85924437	0.09564440
H	4.78143820	-0.66308538	-0.78143046
H	4.73946343	-0.52424396	0.95926249
H	4.04519932	-1.93676988	0.17894976
H	-1.12712171	-1.98255166	1.06413532

Time = 14.50 fs
E(CASSCF/6-31G(d)) = -479.5636506

C	-0.90405142	-0.23007679	-0.08365083
C	0.57310426	0.15364523	-0.06144538
C	1.13597475	1.42040297	-0.13895427
C	1.51458050	-0.85567418	0.03978276
C	2.83411548	-0.21145373	0.01543647
C	2.60660050	1.21656879	-0.09422105
N	-1.93830055	0.63062866	-0.12989712
C	-3.16635893	-0.08397570	0.14953990
H	3.38828410	1.95415753	-0.13746847
C	-1.35322970	-1.62027285	0.05806644
C	-2.84542564	-1.52432128	-0.24839084
H	-3.00779592	-1.65495886	-1.31237387
H	-3.46077664	-2.24032536	0.27996346
H	-3.35802517	-0.00813854	1.22072702
H	-3.99750659	0.35960938	-0.37245212
H	-0.79856527	-2.25834797	-0.62948194
H	0.61577755	2.35008779	-0.21593701
C	-1.88378263	2.06209901	0.02557477
H	-1.45052500	2.33990600	0.97957307
H	-1.33137236	2.53054530	-0.76964408
H	-2.90168850	2.41667933	-0.01054702
H	1.28507612	-1.89634568	0.12155538
C	4.16561659	-0.85966634	0.09568860
H	4.78351329	-0.66575504	-0.78217343
H	4.74164017	-0.52654185	0.96026798
H	4.04225227	-1.93623608	0.17895767
H	-1.12766592	-1.98523518	1.06503566

Time = 15.00 fs
E(CASSCF/6-31G(d)) = -479.5652905

C	-0.89881306	-0.22974743	-0.08461348
C	0.56665591	0.15200484	-0.06194436

C	1.14098845	1.42119861	-0.13912085
C	1.51864663	-0.85422691	0.04012760
C	2.83343664	-0.21432705	0.01575265
C	2.60576928	1.22062455	-0.09453447
N	-1.94100163	0.63132893	-0.12814677
C	-3.16712936	-0.08317779	0.14898605
H	3.39224487	1.95405931	-0.13756234
C	-1.35463407	-1.62174526	0.05818249
C	-2.84698107	-1.52529341	-0.24822098
H	-3.00825390	-1.65663171	-1.31321264
H	-3.46269026	-2.24042815	0.28020664
H	-3.36418926	-0.01133109	1.21958336
H	-3.99816814	0.35862046	-0.37325191
H	-0.80059614	-2.25844129	-0.63198883
H	0.61068407	2.34707431	-0.21604334
C	-1.88368380	2.06183447	0.02567849
H	-1.45185151	2.34491463	0.97866415
H	-1.33302741	2.53106846	-0.77088945
H	-2.90152320	2.41752692	-0.01201352
H	1.27952063	-1.89242915	0.12180703
C	4.16774360	-0.86017752	0.09573987
H	4.78539667	-0.66842901	-0.78284882
H	4.74363691	-0.52886056	0.96118935
H	4.03969324	-1.93568907	0.17896917
H	-1.12860013	-1.98796363	1.06578184

Time = 15.50 fs
E(CASSCF/6-31G(d)) = -479.5664962

C	-0.89333679	-0.22911347	-0.08558784
C	0.55985651	0.15030682	-0.06246042
C	1.14623213	1.42206355	-0.13929534
C	1.52294510	-0.85274350	0.04048108
C	2.83258577	-0.21713166	0.01606947
C	2.60473176	1.22460348	-0.09484389
N	-1.94356819	0.63190825	-0.12640662
C	-3.16795405	-0.08239898	0.14851811
H	3.39557255	1.95407991	-0.13767962
C	-1.35599232	-1.62332336	0.05826454
C	-2.84858159	-1.52624249	-0.24805924
H	-3.00880992	-1.65830820	-1.31397323
H	-3.46455437	-2.24061471	0.28050252
H	-3.37079803	-0.01467119	1.21838448
H	-3.99922616	0.35767426	-0.37448335
H	-0.80325202	-2.25870701	-0.63415808
H	0.60657063	2.34410369	-0.21614729
C	-1.88366529	2.06158675	0.02580463

H	-1.45308527	2.34997117	0.97777664
H	-1.33455766	2.53145092	-0.77237948
H	-2.90150668	2.41878505	-0.01345313
H	1.27489805	-1.88891312	0.12210775
C	4.16991970	-0.86077914	0.09579811
H	4.78707328	-0.67109128	-0.78343936
H	4.74544030	-0.53118772	0.96200585
H	4.03758604	-1.93519383	0.17899074
H	-1.12993621	-1.99068904	1.06627404

Time = 16.00 fs
E(CASSCF/6-31G(d)) = -479.5671950

C	-0.88772679	-0.22818288	-0.08656851
C	0.55283598	0.14858489	-0.06299202
C	1.15160561	1.42298155	-0.13947942
C	1.52738202	-0.85123477	0.04084129
C	2.83160540	-0.21979439	0.01638263
C	2.60353890	1.22844376	-0.09514372
N	-1.94594946	0.63234888	-0.12467552
C	-3.16884111	-0.08165597	0.14813807
H	3.39818113	1.95422818	-0.13782215
C	-1.35729039	-1.62499345	0.05831188
C	-2.85021940	-1.52715710	-0.24791358
H	-3.00946880	-1.65997839	-1.31458647
H	-3.46637415	-2.24089362	0.28086369
H	-3.37779627	-0.01810729	1.21713773
H	-4.00067589	0.35679795	-0.37617138
H	-0.80653558	-2.25917106	-0.63595437
H	0.60361207	2.34117422	-0.21624521
C	-1.88373885	2.06136954	0.02594657
H	-1.45420202	2.35501250	0.97691996
H	-1.33595864	2.53163518	-0.77408122
H	-2.90161457	2.42044093	-0.01485202
H	1.27134666	-1.88586613	0.12246388
C	4.17212753	-0.86147150	0.09586308
H	4.78853705	-0.67372237	-0.78393830
H	4.74704563	-0.53350580	0.96270713
H	4.03598719	-1.93482002	0.17902909
H	-1.13167897	-1.99337049	1.06643480

Time = 16.50 fs
E(CASSCF/6-31G(d)) = -479.5673489

C	-0.88208739	-0.22697123	-0.08754954
C	0.54572805	0.14687294	-0.06353738
C	1.15700774	1.42393574	-0.13967480

C	1.53186061	-0.84971621	0.04120654
C	2.83054258	-0.22224495	0.01668808
C	2.60224368	1.23208593	-0.09542848
N	-1.94810066	0.63263825	-0.12295234
C	-3.16980043	-0.08096390	0.14784503
H	3.40000278	1.95451987	-0.13799193
C	-1.35851701	-1.62673829	0.05832382
C	-2.85188645	-1.52802649	-0.24779053
H	-3.01023712	-1.66163269	-1.31499514
H	-3.46815275	-2.24126983	0.28129986
H	-3.38512585	-0.02158521	1.21585290
H	-4.00247516	0.35600208	-0.37831622
H	-0.81042338	-2.25987833	-0.63737061
H	0.60195408	2.33831573	-0.21633597
C	-1.88391510	2.06119620	0.02609717
H	-1.45518158	2.35997603	0.97609665
H	-1.33723373	2.53156268	-0.77594901
H	-2.90181089	2.42247115	-0.01619690
H	1.26900034	-1.88332471	0.12287924
C	4.17434781	-0.86225424	0.09593445
H	4.78979082	-0.67629968	-0.78434918
H	4.74845669	-0.53579249	0.96329310
H	4.03494476	-1.93463661	0.17909069
H	-1.13382462	-1.99597642	1.06621543

Time = 17.00 fs
E(CASSCF/6-31G(d)) = -479.5669677

C	-0.87652188	-0.22550115	-0.08852462
C	0.53866732	0.14520449	-0.06409453
C	1.16233924	1.42490877	-0.13988319
C	1.53628407	-0.84820733	0.04157543
C	2.82944768	-0.22441728	0.01698209
C	2.60089973	1.23547365	-0.09569288
N	-1.94998370	0.63276882	-0.12123584
C	-3.17084317	-0.08033616	0.14763517
H	3.40099163	1.95497873	-0.13819110
C	-1.35966403	-1.62853747	0.05829939
C	-2.85357460	-1.52884080	-0.24769477
H	-3.01112325	-1.66326244	-1.31515843
H	-3.46989006	-2.24174309	0.28181688
H	-3.39272684	-0.02504924	1.21454159
H	-4.00454661	0.35528163	-0.38089431
H	-0.81486746	-2.26088977	-0.63842702
H	0.60170434	2.33559413	-0.21642172
C	-1.88420317	2.06107914	0.02624907
H	-1.45600815	2.36480108	0.97530272

H	-1.33839146	2.53117705	-0.77792882
H	-2.90205237	2.42484306	-0.01747534
H	1.26798382	-1.88129662	0.12335529
C	4.17655982	-0.86312661	0.09601179
H	4.79084567	-0.67879811	-0.78468488
H	4.74968526	-0.53802141	0.96377306
H	4.03449766	-1.93470666	0.17918138
H	-1.13635985	-1.99848532	1.06559924

Time = 17.50 fs
E(CASSCF/6-31G(d)) = -479.5660848

C	-0.87113100	-0.22380154	-0.08948719
C	0.53178689	0.14361208	-0.06466129
C	1.16750416	1.42588289	-0.14010616
C	1.54055714	-0.84673111	0.04194680
C	2.82837360	-0.22625020	0.01726126
C	2.59956066	1.23855413	-0.09593188
N	-1.95156767	0.63273785	-0.11952490
C	-3.17198114	-0.07978431	0.14750230
H	3.40112641	1.95563817	-0.13842212
C	-1.36072645	-1.63036803	0.05823703
C	-2.85527574	-1.52959127	-0.24762864
H	-3.01213761	-1.66486043	-1.31505574
H	-3.47158219	-2.24230821	0.28241632
H	-3.40053797	-0.02844316	1.21321713
H	-4.00678484	0.35461819	-0.38386154
H	-0.81979667	-2.26227872	-0.63916852
H	0.60292879	2.33310990	-0.21650770
C	-1.88461036	2.06102948	0.02639524
H	-1.45666988	2.36942995	0.97452851
H	-1.33944210	2.53042845	-0.77996318
H	-2.90229383	2.42751743	-0.01867603
H	1.26840833	-1.87976388	0.12389131
C	4.17874183	-0.86408769	0.09609464
H	4.79172013	-0.68119097	-0.78496605
H	4.75075018	-0.54016328	0.96416426
H	4.03467461	-1.93508255	0.17930590
H	-1.13926113	-2.00088663	1.06460364

Time = 18.00 fs
E(CASSCF/6-31G(d)) = -479.5648162

C	-0.86601224	-0.22190661	-0.09043073
C	0.52521643	0.14212686	-0.06523539
C	1.17241312	1.42684028	-0.14034519
C	1.54459009	-0.84531224	0.04231979

C	2.82737310	-0.22768844	0.01752254
C	2.59827818	1.24127835	-0.09614070
N	-1.95282897	0.63254702	-0.11781771
C	-3.17322602	-0.07931830	0.14743802
H	3.40041390	1.95654025	-0.13868757
C	-1.36170245	-1.63220518	0.05813433
C	-2.85698202	-1.53027030	-0.24759219
H	-3.01329268	-1.66642020	-1.31468641
H	-3.47322033	-2.24295220	0.28309457
H	-3.40849676	-0.03171000	1.21188996
H	-4.00906084	0.35398478	-0.38715691
H	-0.82512361	-2.26412401	-0.63965807
H	0.60564270	2.33100241	-0.21660304
C	-1.88514187	2.06105638	0.02652902
H	-1.45716078	2.37380706	0.97375661
H	-1.34039723	2.52927327	-0.78199343
H	-2.90249126	2.43044632	-0.01978922
H	1.27036406	-1.87869725	0.12448542
C	4.18087177	-0.86513643	0.09618250
H	4.79243852	-0.68345067	-0.78521858
H	4.75167597	-0.54218698	0.96448930
H	4.03549489	-1.93580281	0.17946769
H	-1.14249601	-2.00317894	1.06327679

Time = 18.50 fs
E(CASSCF/6-31G(d)) = -479.5632508

C	-0.86125836	-0.21985468	-0.09134882
C	0.51907972	0.14077819	-0.06581449
C	1.17698437	1.42776309	-0.14060158
C	1.54830025	-0.84397584	0.04269371
C	2.82649772	-0.22868340	0.01776329
C	2.59710134	1.24360179	-0.09631494
N	-1.95375129	0.63220174	-0.11611217
C	-3.17458848	-0.07894653	0.14743245
H	3.39888660	1.95773438	-0.13899010
C	-1.36259305	-1.63402324	0.05798777
C	-2.85868588	-1.53087168	-0.24758303
H	-3.01460260	-1.66793687	-1.31407132
H	-3.47479187	-2.24365699	0.28384366
H	-3.41654041	-0.03479342	1.21056997
H	-4.01123565	0.35335013	-0.39070951
H	-0.83074765	-2.26650391	-0.63997124
H	0.60981498	2.32943888	-0.21671983
C	-1.88580056	2.06116688	0.02664458
H	-1.45747868	2.37788138	0.97296587
H	-1.34126556	2.52767954	-0.78396580

H	-2.90260800	2.43357814	-0.02080694
H	1.27391624	-1.87805967	0.12513489
C	4.18292801	-0.86627176	0.09627488
H	4.79302902	-0.68555000	-0.78547077
H	4.75249083	-0.54406127	0.96477357
H	4.03696696	-1.93688846	0.17966862
H	-1.14602250	-2.00536965	1.06169607

Time = 19.00 fs
E(CASSCF/6-31G(d)) = -479.5615401

C	-0.85695591	-0.21768717	-0.09223530
C	0.51349206	0.13959320	-0.06639632
C	1.18114592	1.42863391	-0.14087639
C	1.55161509	-0.84274568	0.04306801
C	2.82579565	-0.22919424	0.01798132
C	2.59607498	1.24548510	-0.09645064
N	-1.95432551	0.63171062	-0.11440553
C	-3.17607716	-0.07867617	0.14747482
H	3.39660132	1.95927430	-0.13933214
C	-1.36340171	-1.63579666	0.05779256
C	-2.86038022	-1.53139048	-0.24759661
H	-3.01608257	-1.66940659	-1.31325010
H	-3.47628055	-2.24439938	0.28465161
H	-3.42460642	-0.03763821	1.20926436
H	-4.01316972	0.35268392	-0.39444499
H	-0.83656261	-2.26948772	-0.64018743
H	0.61536935	2.32860682	-0.21687270
C	-1.88658676	2.06136546	0.02673700
H	-1.45762511	2.38160683	0.97213246
H	-1.34205160	2.52562783	-0.78583478
H	-2.90261832	2.43685846	-0.02172323
H	1.27909933	-1.87781666	0.12583679
C	4.18489007	-0.86749254	0.09637127
H	4.79352152	-0.68746295	-0.78574997
H	4.75322473	-0.54575623	0.96504180
H	4.03908900	-1.93834192	0.17990887
H	-1.14979098	-2.00747206	1.05996091

Time = 19.50 fs
E(CASSCF/6-31G(d)) = -479.5598227

C	-0.85318371	-0.21544722	-0.09308434
C	0.50855843	0.13859632	-0.06697864
C	1.18483555	1.42943610	-0.14117046
C	1.55447305	-0.84164293	0.04344219
C	2.82531102	-0.22918866	0.01817496

C	2.59523945	1.24689485	-0.09654436
N	-1.95454948	0.63108475	-0.11269476
C	-3.17769782	-0.07851323	0.14755407
H	3.39363423	1.96121497	-0.13971597
C	-1.36413370	-1.63750109	0.05754263
C	-2.86205840	-1.53182315	-0.24762655
H	-3.01774795	-1.67082650	-1.31227807
H	-3.47766752	-2.24515306	0.28550373
H	-3.43263292	-0.04019191	1.20797837
H	-4.01473435	0.35196043	-0.39829138
H	-0.84246228	-2.27312870	-0.64038173
H	0.62219419	2.32869870	-0.21707753
C	-1.88749827	2.06165393	0.02680247
H	-1.45760411	2.38494403	0.97123283
H	-1.34275402	2.52311411	-0.78756622
H	-2.90251085	2.44023280	-0.02253439
H	1.28591539	-1.87793814	0.12658792
C	4.18673910	-0.86879728	0.09647119
H	4.79394556	-0.68916630	-0.78607945
H	4.75390738	-0.54724531	0.96531511
H	4.04184768	-1.94014629	0.18018682
H	-1.15374576	-2.00950325	1.05818612

Time = 20.00 fs
E(CASSCF/6-31G(d)) = -479.5582598

C	-0.85001029	-0.21317850	-0.09389055
C	0.50437045	0.13780866	-0.06755932
C	1.18800167	1.43015477	-0.14148444
C	1.55682555	-0.84068497	0.04381572
C	2.82508249	-0.22864374	0.01834308
C	2.59462990	1.24780444	-0.09659328
N	-1.95442800	0.63033740	-0.11097647
C	-3.17945268	-0.07846272	0.14765936
H	3.39007578	1.96360706	-0.14014329
C	-1.36479547	-1.63911442	0.05723066
C	-2.86371444	-1.53216741	-0.24766510
H	-3.01961382	-1.67219443	-1.31122184
H	-3.47893302	-2.24589070	0.28638379
H	-3.44055998	-0.04240547	1.20671523
H	-4.01582090	0.35116260	-0.40218506
H	-0.84834678	-2.27745725	-0.64061669
H	0.63015087	2.32989705	-0.21735020
C	-1.88853028	2.06203120	0.02683832
H	-1.45742173	2.38786131	0.97024566
H	-1.34336550	2.52015031	-0.78913920
H	-2.90229010	2.44364798	-0.02323907

H	1.29433146	-1.87840370	0.12738512
C	4.18845851	-0.87018390	0.09657416
H	4.79432814	-0.69064051	-0.78647534
H	4.75456618	-0.54850670	0.96560806
H	4.04521819	-1.94226637	0.18049912
H	-1.15782741	-2.01148130	1.05649269

Time = 20.50 fs
E(CASSCF/6-31G(d)) = -479.5570061

C	-0.84749223	-0.21092410	-0.09464908
C	0.50100439	0.13724764	-0.06813636
C	1.19060350	1.43077745	-0.14181880
C	1.55863800	-0.83988436	0.04418803
C	2.82514219	-0.22754677	0.01848521
C	2.59427571	1.24819508	-0.09659527
N	-1.95397255	0.62948345	-0.10924692
C	-3.18133977	-0.07852871	0.14778063
H	3.38602425	1.96649161	-0.14061497
C	-1.36539407	-1.64061772	0.05684853
C	-2.86534294	-1.53242223	-0.24770384
H	-3.02169365	-1.67350810	-1.31015307
H	-3.48005762	-2.24658558	0.28727532
H	-3.44833031	-0.04423435	1.20547605
H	-4.01634781	0.35028480	-0.40607545
H	-0.85412876	-2.28247430	-0.64093484
H	0.63908507	2.33235713	-0.21770513
C	-1.88967550	2.06249322	0.02684286
H	-1.45708525	2.39033581	0.96915394
H	-1.34387310	2.51676407	-0.79054666
H	-2.90197690	2.44705321	-0.02383834
H	1.30427887	-1.87920486	0.12822538
C	4.19003459	-0.87164937	0.09667971
H	4.79469201	-0.69187059	-0.78694428
H	4.75522456	-0.54952463	0.96592620
H	4.04916431	-1.94465146	0.18084097
H	-1.16197550	-2.01342128	1.05499676

Time = 21.00 fs
E(CASSCF/6-31G(d)) = -479.5561896

C	-0.84567227	-0.20872562	-0.09535565
C	0.49851926	0.13692657	-0.06870799
C	1.19261094	1.43129504	-0.14217395
C	1.55989031	-0.83924820	0.04455846
C	2.82551508	-0.22589582	0.01860154
C	2.59420033	1.24805675	-0.09654899

N	-1.95320106	0.62853909	-0.10750214
C	-3.18335267	-0.07871439	0.14790894
H	3.38157895	1.96989441	-0.14113072
C	-1.36593659	-1.64199602	0.05638771
C	-2.86693916	-1.53258771	-0.24773434
H	-3.02399828	-1.67476448	-1.30914208
H	-3.48102379	-2.24721357	0.28816302
H	-3.45589024	-0.04563933	1.20426007
H	-4.01626548	0.34933446	-0.40992840
H	-0.85973757	-2.28814715	-0.64135341
H	0.64883878	2.33618919	-0.21815378
C	-1.89092425	2.06303302	0.02681532
H	-1.45660266	2.39235425	0.96794668
H	-1.34425942	2.51299788	-0.79179495
H	-2.90160743	2.45040085	-0.02433568
H	1.31565429	-1.88034443	0.12910566
C	4.19145682	-0.87318938	0.09678734
H	4.79505443	-0.69284679	-0.78748189
H	4.75590082	-0.55029033	0.96626469
H	4.05363847	-1.94723893	0.18120637
H	-1.16613135	-2.01533187	1.05379903

Time = 21.50 fs
E(CASSCF/6-31G(d)) = -479.5558980

C	-0.84457804	-0.20662222	-0.09600660
C	0.49695534	0.13685447	-0.06927269
C	1.19400457	1.43170267	-0.14255024
C	1.56057731	-0.83877792	0.04492623
C	2.82621819	-0.22370033	0.01869297
C	2.59442067	1.24738904	-0.09645399
N	-1.95213740	0.62752126	-0.10573788
C	-3.18548048	-0.07902207	0.14803664
H	3.37683339	1.97382050	-0.14168878
C	-1.36642982	-1.64323880	0.05583995
C	-2.86849900	-1.53266502	-0.24774883
H	-3.02653487	-1.67595920	-1.30825154
H	-3.48181727	-2.24775497	0.28903408
H	-3.46319044	-0.04658726	1.20306535
H	-4.01555909	0.34833259	-0.41372829
H	-0.86512113	-2.29440789	-0.64186207
H	0.65926184	2.34144193	-0.21870324
C	-1.89226472	2.06364107	0.02675557
H	-1.45598209	2.39391347	0.96662021
H	-1.34450414	2.50890742	-0.79290235
H	-2.90123089	2.45364703	-0.02473677
H	1.32832250	-1.88183289	0.13002265

C	4.19271821	-0.87479798	0.09689653
H	4.79542649	-0.69356515	-0.78807220
H	4.75660737	-0.55080262	0.96660771
H	4.05858189	-1.94995837	0.18158846
H	-1.17024011	-2.01721219	1.05297537

Time = 22.00 fs
E(CASSCF/6-31G(d)) = -479.5561663

C	-0.84421982	-0.20465003	-0.09659899
C	0.49633236	0.13703550	-0.06982919
C	1.19477492	1.43200045	-0.14294806
C	1.56070796	-0.83846971	0.04529049
C	2.82726119	-0.22098122	0.01876118
C	2.59494767	1.24620207	-0.09631074
N	-1.95081142	0.62644776	-0.10394993
C	-3.18770788	-0.07945300	0.14815765
H	3.37186989	1.97824924	-0.14228565
C	-1.36688003	-1.64434018	0.05519801
C	-2.87001900	-1.53265635	-0.24774084
H	-3.02930609	-1.67708603	-1.30753076
H	-3.48242842	-2.24819620	0.28987929
H	-3.47018736	-0.04705210	1.20188947
H	-4.01424907	0.34731305	-0.41747842
H	-0.87024613	-2.30115512	-0.64242384
H	0.67022044	2.34808973	-0.21935502
C	-1.89368322	2.06430535	0.02666393
H	-1.45523158	2.39502124	0.96517917
H	-1.34458631	2.50455984	-0.79389673
H	-2.90090608	2.45675186	-0.02504934
H	1.34212036	-1.88368255	0.13097223
C	4.19381531	-0.87646729	0.09700666
H	4.79581280	-0.69402753	-0.78868815
H	4.75735043	-0.55106795	0.96692893
H	4.06392500	-1.95273601	0.18197990
H	-1.17425326	-2.01904989	1.05256929

Time = 22.50 fs
E(CASSCF/6-31G(d)) = -479.5569766

C	-0.84459053	-0.20284172	-0.09713059
C	0.49664943	0.13746914	-0.07037654
C	1.19492296	1.43219379	-0.14336786
C	1.56030526	-0.83831489	0.04565037
C	2.82864579	-0.21777131	0.01880864
C	2.59578547	1.24451704	-0.09612074
N	-1.94925819	0.62533674	-0.10213399

C	-3.19001571	-0.08000726	0.14826727
H	3.36675566	1.98313176	-0.14291590
C	-1.36729302	-1.64529875	0.05445633
C	-2.87149635	-1.53256482	-0.24770566
H	-3.03230944	-1.67813643	-1.30701102
H	-3.48285302	-2.24853058	0.29069380
H	-3.47684356	-0.04701504	1.20072990
H	-4.01238848	0.34632097	-0.42119986
H	-0.87509585	-2.30825785	-0.64297879
H	0.68160175	2.35602636	-0.22010456
C	-1.89516452	2.06501203	0.02654089
H	-1.45435912	2.39569560	0.96363607
H	-1.34448696	2.50003053	-0.79481192
H	-2.90069681	2.45967880	-0.02528275
H	1.35686140	-1.88590087	0.13194904
C	4.19474813	-0.87818730	0.09711708
H	4.79621224	-0.69424166	-0.78929333
H	4.75813064	-0.55110004	0.96719332
H	4.06958815	-1.95549947	0.18237326
H	-1.17812958	-2.02081971	1.05258747

Time = 23.00 fs
E(CASSCF/6-31G(d)) = -479.5582505

C	-0.84566507	-0.20122634	-0.09760009
C	0.49788468	0.13815009	-0.07091404
C	1.19446027	1.43229327	-0.14381011
C	1.55940534	-0.83830073	0.04600503
C	2.83036614	-0.21411525	0.01883860
C	2.59693121	1.24236674	-0.09588655
N	-1.94751755	0.62420675	-0.10028570
C	-3.19238172	-0.08068364	0.14836209
H	3.36154189	1.98839026	-0.14357213
C	-1.36767420	-1.64611713	0.05361173
C	-2.87292883	-1.53239458	-0.24764068
H	-3.03553705	-1.67909936	-1.30670254
H	-3.48309285	-2.24875865	0.29147741
H	-3.48312836	-0.04646463	1.19958414
H	-4.01005831	0.34541014	-0.42492868
H	-0.87966672	-2.31556221	-0.64345039
H	0.69331352	2.36506623	-0.22094135
C	-1.89669229	2.06574605	0.02638683
H	-1.45337307	2.39596402	0.96201005
H	-1.34419196	2.49539968	-0.79568355
H	-2.90066682	2.46239387	-0.02544765
H	1.37234036	-1.88848354	0.13294594
C	4.19551999	-0.87994580	0.09722699

H	4.79661927	-0.69422066	-0.78984471
H	4.75894430	-0.55091906	0.96735976
H	4.07548271	-1.95818225	0.18276143
H	-1.18183599	-2.02248399	1.05299883

Time = 23.50 fs
E(CASSCF/6-31G(d)) = -479.5598659

C	-0.84740352	-0.19982935	-0.09800704
C	0.49999743	0.13906891	-0.07144131
C	1.19341029	1.43231398	-0.14427525
C	1.55805758	-0.83841112	0.04635375
C	2.83240813	-0.21007007	0.01885520
C	2.59837357	1.23979572	-0.09561186
N	-1.94563298	0.62307617	-0.09840059
C	-3.19478109	-0.08147941	0.14843966
H	3.35626409	1.99392038	-0.14424505
C	-1.36802903	-1.64680108	0.05266375
C	-2.87431482	-1.53215054	-0.24754555
H	-3.03897479	-1.67996132	-1.30659384
H	-3.48315531	-2.24888819	0.29223468
H	-3.48901693	-0.04539655	1.19845049
H	-4.00736012	0.34463928	-0.42871143
H	-0.88396309	-2.32289876	-0.64375333
H	0.70528084	2.37495046	-0.22184943
C	-1.89824945	2.06649156	0.02620175
H	-1.45228209	2.39586190	0.96032704
H	-1.34369396	2.49074888	-0.79654612
H	-2.90087409	2.46486462	-0.02555537
H	1.38833836	-1.89140543	0.13395338
C	4.19613705	-0.88172829	0.09733550
H	4.79702542	-0.69398293	-0.79029589
H	4.75978466	-0.55055084	0.96738444
H	4.08151212	-1.96072707	0.18313789
H	-1.18534652	-2.02399302	1.05373664

Time = 24.00 fs
E(CASSCF/6-31G(d)) = -479.5616550

C	-0.84975119	-0.19867275	-0.09835214
C	0.50292925	0.14021224	-0.07195818
C	1.19180872	1.43227440	-0.14476361
C	1.55632301	-0.83862755	0.04669605
C	2.83475021	-0.20570446	0.01886335
C	2.60009257	1.23686005	-0.09530150
N	-1.94365088	0.62196330	-0.09647400
C	-3.19718796	-0.08239013	0.14849804

H	3.35094581	1.99959422	-0.14492354
C	-1.36836333	-1.64735864	0.05161492
C	-2.87565321	-1.53183860	-0.24742206
H	-3.04260340	-1.68070659	-1.30665241
H	-3.48305359	-2.24893280	0.29297438
H	-3.49449091	-0.04381319	1.19732726
H	-4.00440759	0.34406815	-0.43259962
H	-0.88799263	-2.33009271	-0.64380263
H	0.71743698	2.38536582	-0.22280909
C	-1.89981861	2.06723283	0.02598508
H	-1.45109665	2.39543084	0.95861563
H	-1.34299563	2.48615726	-0.79742739
H	-2.90136615	2.46705849	-0.02561756
H	1.40462705	-1.89461801	0.13495929
C	4.19660794	-0.88351820	0.09744158
H	4.79742127	-0.69355102	-0.79060080
H	4.76064373	-0.55002521	0.96722437
H	4.08757383	-1.96308897	0.18349703
H	-1.18864221	-2.02528890	1.05470557

Time = 24.50 fs
E(CASSCF/6-31G(d)) = -479.5634351

C	-0.85264289	-0.19777546	-0.09863730
C	0.50660765	0.14156371	-0.07246468
C	1.18970373	1.43219483	-0.14527529
C	1.55427306	-0.83892994	0.04703174
C	2.83736371	-0.20109812	0.01886876
C	2.60205902	1.23362642	-0.09496142
N	-1.94161915	0.62088574	-0.09450098
C	-3.19957626	-0.08340941	0.14853535
H	3.34560259	2.00526753	-0.14559517
C	-1.36868364	-1.64779897	0.05047065
C	-2.87694338	-1.53146544	-0.24727385
H	-3.04639850	-1.68131811	-1.30682828
H	-3.48280544	-2.24891127	0.29370910
H	-3.49953774	-0.04172341	1.19621348
H	-4.00131717	0.34375253	-0.43664356
H	-0.89176090	-2.33697269	-0.64352220
H	0.72971616	2.39596273	-0.22379834
C	-1.90138252	2.06795478	0.02573557
H	-1.44982893	2.39471701	0.95690630
H	-1.34211034	2.48169882	-0.79834667
H	-2.90217544	2.46894271	-0.02564563
H	1.42097323	-1.89804382	0.13594885
C	4.19694322	-0.88529720	0.09754408
H	4.79779827	-0.69295120	-0.79071754

H	4.76151406	-0.54937476	0.96684128
H	4.09356081	-1.96523677	0.18383429
H	-1.19170916	-2.02630838	1.05579021

Time = 25.00 fs
E(CASSCF/6-31G(d)) = -479.5650077

C	-0.85600179	-0.19715355	-0.09886548
C	0.51094659	0.14310378	-0.07296099
C	1.18715556	1.43209619	-0.14581010
C	1.55198784	-0.83929766	0.04736094
C	2.84021433	-0.19634050	0.01887776
C	2.60423528	1.23017112	-0.09459848
N	-1.93958744	0.61986095	-0.09247676
C	-3.20192124	-0.08452853	0.14854940
H	3.34024951	2.01078611	-0.14624645
C	-1.36899751	-1.64813152	0.04923911
C	-2.87818532	-1.53103876	-0.24710591
H	-3.05033199	-1.68177879	-1.30705869
H	-3.48243245	-2.24884595	0.29445357
H	-3.50415279	-0.03914332	1.19510890
H	-3.99819978	0.34373899	-0.44088558
H	-0.89526718	-2.34338137	-0.64285347
H	0.74204300	2.40637773	-0.22479468
C	-1.90292444	2.06864355	0.02545144
H	-1.44849382	2.39377136	0.95522912
H	-1.34106311	2.47744169	-0.79931292
H	-2.90331659	2.47048423	-0.02565038
H	1.43714078	-1.90157728	0.13690468
C	4.19715485	-0.88704554	0.09764175
H	4.79815008	-0.69221193	-0.79061199
H	4.76238988	-0.54863292	0.96620497
H	4.09936409	-1.96715352	0.18414628
H	-1.19453801	-2.02698895	1.05686596

Time = 25.50 fs
E(CASSCF/6-31G(d)) = -479.5662317

C	-0.85974501	-0.19682061	-0.09904092
C	0.51585112	0.14481085	-0.07344734
C	1.18423619	1.43199822	-0.14636745
C	1.54955455	-0.83970989	0.04768419
C	2.84326253	-0.19152924	0.01889729
C	2.60657503	1.22657818	-0.09422044
N	-1.93760532	0.61890553	-0.09039650
C	-3.20420018	-0.08573627	0.14853721
H	3.33490630	2.01599564	-0.14686357

C	-1.36931372	-1.64836489	0.04793074
C	-2.87937948	-1.53056711	-0.24692410
H	-3.05437277	-1.68207212	-1.30727389
H	-3.48195847	-2.24876064	0.29522376
H	-3.50833739	-0.03609515	1.19401329
H	-3.99515086	0.34406061	-0.44535359
H	-0.89850131	-2.34918202	-0.64176113
H	0.75432534	2.41625879	-0.22577711
C	-1.90442842	2.06928677	0.02513037
H	-1.44710967	2.39264646	0.95361121
H	-1.33989061	2.47344517	-0.80032363
H	-2.90478440	2.47164903	-0.02564159
H	1.45289424	-1.90508973	0.13780749
C	4.19725569	-0.88874257	0.09773324
H	4.79847423	-0.69136340	-0.79026081
H	4.76326873	-0.54783291	0.96529638
H	4.10487491	-1.96883630	0.18443076
H	-1.19712193	-2.02727192	1.05780950

Time = 26.00 fs
E(CASSCF/6-31G(d)) = -479.5669845

C	-0.86378538	-0.19678777	-0.09916906
C	0.52121944	0.14666143	-0.07392399
C	1.18102726	1.43191847	-0.14694628
C	1.54706474	-0.84014635	0.04800232
C	2.84646563	-0.18676772	0.01893466
C	2.60902518	1.22293718	-0.09383572
N	-1.93572203	0.61803530	-0.08825564
C	-3.20639311	-0.08701854	0.14849487
H	3.32960333	2.02075123	-0.14743300
C	-1.36964233	-1.64850614	0.04655776
C	-2.88052674	-1.53005975	-0.24673453
H	-3.05848793	-1.68218342	-1.30740372
H	-3.48140828	-2.24867900	0.29603555
H	-3.51209944	-0.03260746	1.19292605
H	-3.99224340	0.34473282	-0.45005600
H	-0.90144188	-2.35426520	-0.64023771
H	0.76644914	2.42528652	-0.22672781
C	-1.90587944	2.06987348	0.02476987
H	-1.44569904	2.39139587	0.95207493
H	-1.33864042	2.46975930	-0.80136537
H	-2.90655368	2.47240306	-0.02562782
H	1.46800145	-1.90843705	0.13863693
C	4.19725913	-0.89036732	0.09781715
H	4.79877292	-0.69043663	-0.78965361
H	4.76415212	-0.54700640	0.96410963

H	4.10998715	-1.97029451	0.18468663
H	-1.19945589	-2.02710701	1.05850946

Time = 26.50 fs
E(CASSCF/6-31G(d)) = -479.5671761

C	-0.86803232	-0.19706369	-0.09925643
C	0.52694450	0.14863018	-0.07439111
C	1.17761765	1.43187146	-0.14754507
C	1.54461170	-0.84058788	0.04831643
C	2.84977969	-0.18216210	0.01899742
C	2.61152783	1.21934040	-0.09345313
N	-1.93398581	0.61726513	-0.08605026
C	-3.20848326	-0.08835832	0.14841729
H	3.32438471	2.02492424	-0.14794221
C	-1.36999468	-1.64856023	0.04513371
C	-2.88162848	-1.52952657	-0.24654290
H	-3.06264326	-1.68210178	-1.30738395
H	-3.48080592	-2.24862313	0.29690340
H	-3.51545457	-0.02871711	1.19184813
H	-3.98952546	0.34575000	-0.45497775
H	-0.90405497	-2.35855335	-0.63830673
H	0.77827757	2.43319014	-0.22763292
C	-1.90726380	2.07039407	0.02436750
H	-1.44428773	2.39007523	0.95063934
H	-1.33736757	2.46642703	-0.80241783
H	-2.90858127	2.47271528	-0.02561616
H	1.48223667	-1.91146633	0.13937243
C	4.19717850	-0.89189907	0.09789209
H	4.79905313	-0.68946277	-0.78879430
H	4.76504574	-0.54618272	0.96265324
H	4.11459918	-1.97154718	0.18491364
H	-1.20153571	-2.02645629	1.05887454

Time = 27.00 fs
E(CASSCF/6-31G(d)) = -479.5668229

C	-0.87239653	-0.19765464	-0.09931073
C	0.53291889	0.15069090	-0.07484879
C	1.17410188	1.43186761	-0.14816179
C	1.54228883	-0.84101586	0.04862790
C	2.85315940	-0.17781844	0.01909307
C	2.61402118	1.21587962	-0.09308160
N	-1.93244242	0.61660850	-0.08377679
C	-3.21045777	-0.08973578	0.14829830
H	3.31931367	2.02841402	-0.14838038
C	-1.37038305	-1.64852960	0.04367270

C	-2.88268645	-1.52897813	-0.24635399
H	-3.06680571	-1.68181986	-1.30716143
H	-3.48017311	-2.24861046	0.29783878
H	-3.51842372	-0.02446596	1.19077712
H	-3.98701494	0.34708563	-0.46007908
H	-0.90629588	-2.36200278	-0.63602105
H	0.78964873	2.43976841	-0.22848441
C	-1.90856909	2.07084059	0.02392125
H	-1.44290695	2.38873774	0.94931420
H	-1.33613424	2.46348077	-0.80345292
H	-2.91080846	2.47255585	-0.02561233
H	1.49538340	-1.91403406	0.13999484
C	4.19702694	-0.89331812	0.09795667
H	4.79932734	-0.68847252	-0.78770198
H	4.76596013	-0.54538804	0.96095094
H	4.11861690	-1.97262052	0.18511236
H	-1.20335791	-2.02529685	1.05884134

Time = 27.50 fs
E(CASSCF/6-31G(d)) = -479.5659423

C	-0.87679059	-0.19856402	-0.09934060
C	0.53903572	0.15281631	-0.07529706
C	1.17057717	1.43191310	-0.14879402
C	1.54018701	-0.84141234	0.04893826
C	2.85655987	-0.17383923	0.01922885
C	2.61644200	1.21264305	-0.09272983
N	-1.93113453	0.61607722	-0.08143240
C	-3.21230754	-0.09112851	0.14813090
H	3.31447229	2.03115184	-0.14873893
C	-1.37082039	-1.64841425	0.04218880
C	-2.88370283	-1.52842545	-0.24617119
H	-3.07094409	-1.68133556	-1.30669933
H	-3.47952835	-2.24865364	0.29884957
H	-3.52103417	-0.01990219	1.18970903
H	-3.98470364	0.34869252	-0.46529728
H	-0.90811019	-2.36460435	-0.63346169
H	0.80038038	2.44489584	-0.22928024
C	-1.90978439	2.07120646	0.02342990
H	-1.44159166	2.38743554	0.94810231
H	-1.33500522	2.46094559	-0.80444054
H	-2.91316584	2.47190055	-0.02562068
H	1.50723831	-1.91601463	0.14048739
C	4.19681710	-0.89460639	0.09800961
H	4.79961200	-0.68749563	-0.78640984
H	4.76690931	-0.54464509	0.95904041
H	4.12195547	-1.97354341	0.18528380

H	-1.20491929	-2.02362329	1.05837934

Time = 28.00 fs
E(CASSCF/6-31G(d)) = -479.5646237

C	-0.88113045	-0.19979180	-0.09935546
C	0.54518993	0.15497805	-0.07573577
C	1.16714117	1.43200984	-0.14943893
C	1.53839275	-0.84175974	0.04924904
C	2.85993739	-0.17032056	0.01941151
C	2.61872764	1.20971263	-0.09240610
N	-1.93010105	0.61568109	-0.07901520
C	-3.21402676	-0.09251177	0.14790764
H	3.30996168	2.03310501	-0.14901184
C	-1.37131996	-1.64821196	0.04069563
C	-2.88468011	-1.52787984	-0.24599620
H	-3.07502967	-1.68065253	-1.30598028
H	-3.47888618	-2.24875923	0.29993907
H	-3.52331879	-0.01508031	1.18863816
H	-3.98256136	0.35050545	-0.47054977
H	-0.90943698	-2.36638058	-0.63073364
H	0.81027328	2.44852892	-0.23002458
C	-1.91090027	2.07148602	0.02289335
H	-1.44038022	2.38621902	0.94699993
H	-1.33404477	2.45883981	-0.80535181
H	-2.91557819	2.47073233	-0.02564436
H	1.51761397	-1.91731282	0.14083693
C	4.19656119	-0.89574796	0.09804977
H	4.79992673	-0.68656065	-0.78496362
H	4.76791001	-0.54397328	0.95697176
H	4.12454140	-1.97434414	0.18542917
H	-1.20621703	-2.02144826	1.05749213

Time = 28.50 fs
E(CASSCF/6-31G(d)) = -479.5629877

C	-0.88533705	-0.20133386	-0.09936523
C	0.55128023	0.15714680	-0.07616462
C	1.16388926	1.43215564	-0.15009339
C	1.53698607	-0.84204055	0.04956168
C	2.86325011	-0.16734878	0.01964705
C	2.62081809	1.20716102	-0.09211793
N	-1.92937600	0.61542733	-0.07652435
C	-3.21561254	-0.09385914	0.14762118
H	3.30589920	2.03427869	-0.14919602
C	-1.37189479	-1.64791883	0.03920575
C	-2.88562104	-1.52735273	-0.24582891

H	-3.07903709	-1.67978059	-1.30500814
H	-3.47825675	-2.24892738	0.30110581
H	-3.52531545	-0.01006054	1.18755663
H	-3.98054257	0.35244529	-0.47574004
H	-0.91021289	-2.36738124	-0.62795945
H	0.81911770	2.45070643	-0.23072764
C	-1.91190883	2.07167443	0.02231279
H	-1.43931313	2.38513612	0.94599732
H	-1.33331328	2.45717593	-0.80616278
H	-2.91796997	2.46904355	-0.02568550
H	1.52634293	-1.91787295	0.14103480
C	4.19627089	-0.89672962	0.09807618
H	4.80029268	-0.68569491	-0.78341916
H	4.76898024	-0.54338903	0.95480501
H	4.12631424	-1.97504668	0.18554962
H	-1.20724896	-2.01880238	1.05621716

Time = 29.00 fs
E(CASSCF/6-31G(d)) = -479.5611824

C	-0.88933784	-0.20318129	-0.09938023
C	0.55721079	0.15929247	-0.07658313
C	1.16091218	1.43234456	-0.15075406
C	1.53603869	-0.84223701	0.04987745
C	2.86645837	-0.16499754	0.01994052
C	2.62265793	1.20504918	-0.09187185
N	-1.92898741	0.61531995	-0.07396008
C	-3.21706431	-0.09514318	0.14726490
H	3.30241405	2.03471483	-0.14929129
C	-1.37255708	-1.64753000	0.03773025
C	-2.88652860	-1.52685540	-0.24566738
H	-3.08294479	-1.67873573	-1.30380799
H	-3.47764593	-2.24915215	0.30234365
H	-3.52706614	-0.00490806	1.18645435
H	-3.97859436	0.35442436	-0.48076495
H	-0.91037699	-2.36767669	-0.62527131
H	0.82670063	2.45154452	-0.23140511
C	-1.91280376	2.07176754	0.02169093
H	-1.43843170	2.38423181	0.94508007
H	-1.33286413	2.45596128	-0.80685756
H	-2.92027063	2.46683771	-0.02574543
H	1.53328125	-1.91768533	0.14107739
C	4.19595746	-0.89754124	0.09808810
H	4.80073080	-0.68492452	-0.78183922
H	4.77013766	-0.54290641	0.95260684
H	4.12722797	-1.97566771	0.18564599
H	-1.20801390	-2.01573300	1.05462270

Time = 29.50 fs
E(CASSCF/6-31G(d)) = -479.5593696

C	-0.89306835	-0.20531969	-0.09941084
C	0.56289291	0.16138441	-0.07699067
C	1.15829387	1.43256757	-0.15141752
C	1.53561257	-0.84233094	0.05019731
C	2.86952493	-0.16332538	0.02029585
C	2.62419793	1.20342441	-0.09167318
N	-1.92895627	0.61535922	-0.07132371
C	-3.21838327	-0.09633627	0.14683346
H	3.29964118	2.03448805	-0.14930030
C	-1.37331757	-1.64704064	0.03627832
C	-2.88740585	-1.52639879	-0.24550804
H	-3.08673515	-1.67753994	-1.30242446
H	-3.47705572	-2.24942214	0.30364234
H	-3.52861599	0.00030791	1.18531921
H	-3.97666463	0.35635227	-0.48552259
H	-0.90987631	-2.36734956	-0.62280179
H	0.83281394	2.45122635	-0.23207721
C	-1.91358042	2.07176183	0.02103196
H	-1.43777629	2.38354737	0.94423051
H	-1.33274116	2.45519846	-0.80743061
H	-2.92241934	2.46413074	-0.02582490
H	1.53831159	-1.91678899	0.14096643
C	4.19563189	-0.89817605	0.09808506
H	4.80125988	-0.68427454	-0.78028985
H	4.77139781	-0.54253795	0.95044701
H	4.12725209	-1.97621441	0.18571869
H	-1.20851205	-2.01230192	1.05280243

Time = 30.00 fs
E(CASSCF/6-31G(d)) = -479.5577157

C	-0.89647379	-0.20772854	-0.09946741
C	0.56824672	0.16339186	-0.07738644
C	1.15610952	1.43281350	-0.15208037
C	1.53575875	-0.84230371	0.05052194
C	2.87241485	-0.16237367	0.02071565
C	2.62539646	1.20231908	-0.09152589
N	-1.92929536	0.61554094	-0.06861749
C	-3.21957185	-0.09741147	0.14632340
H	3.29771391	2.03369912	-0.14922814
C	-1.37418484	-1.64644690	0.03485696
C	-2.88825592	-1.52599319	-0.24534607
H	-3.09039458	-1.67622046	-1.30091818

H	-3.47648499	-2.24972172	0.30498839
H	-3.53001201	0.00551580	1.18413713
H	-3.97470964	0.35814200	-0.48992026
H	-0.90867147	-2.36648551	-0.62067396
H	0.83726278	2.44998623	-0.23276736
C	-1.91423584	2.07165446	0.02034151
H	-1.43738464	2.38311949	0.94342911
H	-1.33297690	2.45488519	-0.80788796
H	-2.92436875	2.46095144	-0.02592429
H	1.54134548	-1.91527058	0.14070870
C	4.19530502	-0.89863077	0.09806684
H	4.80189470	-0.68376931	-0.77883656
H	4.77277244	-0.54229558	0.94839445
H	4.12637240	-1.97668351	0.18576760
H	-1.20874551	-2.00858228	1.05086812

Time = 30.50 fs
E(CASSCF/6-31G(d)) = -479.5563625

C	-0.89951007	-0.21038081	-0.09955992
C	0.57320241	0.16528448	-0.07776955
C	1.15442351	1.43307015	-0.15273940
C	1.53651596	-0.84213670	0.05085164
C	2.87509597	-0.16216553	0.02120120
C	2.62622108	1.20175050	-0.09143258
N	-1.93000868	0.61585618	-0.06584469
C	-3.22063290	-0.09834331	0.14573350
H	3.29675418	2.03246470	-0.14908179
C	-1.37516477	-1.64574704	0.03347092
C	-2.88908182	-1.52564784	-0.24517582
H	-3.09391253	-1.67480914	-1.29936151
H	-3.47593087	-2.25003283	0.30636652
H	-3.53130253	0.01064201	1.18289421
H	-3.97270107	0.35971514	-0.49388152
H	-0.90674184	-2.36516427	-0.61899306
H	0.83987570	2.44808710	-0.23350027
C	-1.91476880	2.07144312	0.01962641
H	-1.43728965	2.38298059	0.94265749
H	-1.33359111	2.45501487	-0.80824830
H	-2.92608792	2.45734244	-0.02604372
H	1.54232540	-1.91325725	0.14031558
C	4.19498779	-0.89890562	0.09803352
H	4.80264413	-0.68343254	-0.77754040
H	4.77426772	-0.54219142	0.94651340
H	4.12459128	-1.97706113	0.18579203
H	-1.20871862	-2.00465486	1.04894079

Time = 31.00 fs
E(CASSCF/6-31G(d)) = -479.5554342

C	-0.90214322	-0.21324290	-0.09969760
C	0.57770101	0.16703230	-0.07813893
C	1.15328913	1.43332583	-0.15339177
C	1.53791093	-0.84181128	0.05118635
C	2.87753815	-0.16270509	0.02175228
C	2.62664877	1.20172070	-0.09139432
N	-1.93109109	0.61629108	-0.06300975
C	-3.22157001	-0.09910859	0.14506508
H	3.29686443	2.03090577	-0.14886924
C	-1.37626002	-1.64494239	0.03212282
C	-2.88988658	-1.52537102	-0.24499139
H	-3.09728134	-1.67334176	-1.29783247
H	-3.47538956	-2.25033591	0.30775991
H	-3.53253620	0.01561269	1.18157689
H	-3.97063047	0.36100678	-0.49735069
H	-0.90409105	-2.36345171	-0.61783749
H	0.84051164	2.44579758	-0.23430007
C	-1.91517990	2.07112680	0.01889455
H	-1.43751833	2.38315769	0.94189837
H	-1.33459100	2.45557559	-0.80854108
H	-2.92756351	2.45335866	-0.02618334
H	1.54122552	-1.91090831	0.13980218
C	4.19469116	-0.89900402	0.09798543
H	4.80350942	-0.68328720	-0.77645461
H	4.77588260	-0.54223833	0.94486008
H	4.12192809	-1.97732381	0.18579083
H	-1.20843898	-2.00060433	1.04713936

Time = 31.50 fs
E(CASSCF/6-31G(d)) = -479.5550396

C	-0.90435215	-0.21627484	-0.09988901
C	0.58169595	0.16860697	-0.07849344
C	1.15274691	1.43357102	-0.15403510
C	1.53995771	-0.84131029	0.05152580
C	2.87971349	-0.16397754	0.02236724
C	2.62666686	1.20221762	-0.09141083
N	-1.93252763	0.61682669	-0.06011783
C	-3.22238671	-0.09968717	0.14432208
H	3.29811774	2.02913675	-0.14859888
C	-1.37746958	-1.64403789	0.03081345
C	-2.89067301	-1.52516948	-0.24478732
H	-3.10049477	-1.67185596	-1.29640794
H	-3.47485774	-2.25061171	0.30915228

H	-3.53376005	0.02035581	1.18017295
H	-3.96851139	0.36196910	-0.50029769
H	-0.90074982	-2.36139326	-0.61725334
H	0.83906996	2.44336373	-0.23518819
C	-1.91547162	2.07070548	0.01815445
H	-1.43809029	2.38367138	0.94113744
H	-1.33597192	2.45654899	-0.80880494
H	-2.92879960	2.44906583	-0.02634318
H	1.53805184	-1.90840057	0.13918618
C	4.19442641	-0.89893244	0.09792316
H	4.80448359	-0.68335564	-0.77562187
H	4.77760833	-0.54245052	0.94347988
H	4.11841884	-1.97744133	0.18576260
H	-1.20791763	-1.99651460	1.04557146

Time = 32.00 fs
E(CASSCF/6-31G(d)) = -479.5552107

C	-0.90612713	-0.21943070	-0.10014159
C	0.58515314	0.16998220	-0.07883194
C	1.15282320	1.43380012	-0.15466777
C	1.54265710	-0.84061951	0.05186952
C	2.88159708	-0.16594993	0.02304304
C	2.62627394	1.20321682	-0.09148051
N	-1.93429409	0.61743943	-0.05717495
C	-3.22308646	-0.10006246	0.14351103
H	3.30054828	2.02725236	-0.14827854
C	-1.37878854	-1.64304266	0.02954207
C	-2.89144375	-1.52504833	-0.24455908
H	-3.10354753	-1.67039077	-1.29515815
H	-3.47433408	-2.25084376	0.31052934
H	-3.53501997	0.02480222	1.17867390
H	-3.96638148	0.36257399	-0.50272045
H	-0.89677702	-2.35901170	-0.61725197
H	0.83549929	2.44098076	-0.23618086
C	-1.91564834	2.07018046	0.01741488
H	-1.43901632	2.38453659	0.94036487
H	-1.33771843	2.45791026	-0.80908564
H	-2.92981625	2.44453933	-0.02652338
H	1.53284222	-1.90590903	0.13848609
C	4.19420503	-0.89870009	0.09784754
H	4.80555054	-0.68365937	-0.77507188
H	4.77942763	-0.54284363	0.94240498
H	4.11411540	-1.97737902	0.18570581
H	-1.20716950	-1.99246500	1.04432394

Time = 32.50 fs

E(CASSCF/6-31G(d)) = -479.5559593

C	-0.90747008	-0.22265974	-0.10046177
C	0.58805159	0.17113453	-0.07915324
C	1.15353006	1.43401260	-0.15528890
C	1.54599706	-0.83972907	0.05221706
C	2.88316683	-0.16857273	0.02377538
C	2.62547956	1.20468352	-0.09160063
N	-1.93635755	0.61810174	-0.05418784
C	-3.22367262	-0.10022188	0.14264086
H	3.30414571	2.02531987	-0.14791499
C	-1.38020801	-1.64196987	0.02830724
C	-2.89220123	-1.52501091	-0.24430381
H	-3.10643373	-1.66898466	-1.29413976
H	-3.47381976	-2.25101870	0.31187939
H	-3.53635892	0.02888752	1.17707501
H	-3.96429989	0.36281384	-0.50464437
H	-0.89225967	-2.35630622	-0.61780951
H	0.82980328	2.43877211	-0.23728751
C	-1.91571639	2.06955453	0.01668452
H	-1.44029815	2.38576119	0.93957506
H	-1.33980665	2.45962635	-0.80943207
H	-2.93064649	2.43986072	-0.02672404
H	1.52566548	-1.90358944	0.13771991
C	4.19403868	-0.89831846	0.09775958
H	4.80668577	-0.68421860	-0.77482073
H	4.78131530	-0.54343444	0.94165371
H	4.10908480	-1.97710213	0.18561922
H	-1.20621423	-1.98852671	1.04345438

Time = 33.00 fs
E(CASSCF/6-31G(d)) = -479.5572234

C	-0.90839371	-0.22590754	-0.10085473
C	0.59038330	0.17204402	-0.07945617
C	1.15486473	1.43421387	-0.15589857
C	1.54995274	-0.83863527	0.05256805
C	2.88440408	-0.17178144	0.02455884
C	2.62430408	1.20657449	-0.09176744
N	-1.93867718	0.61878284	-0.05116388
C	-3.22414860	-0.10015720	0.14172255
H	3.30885002	2.02337223	-0.14751346
C	-1.38171539	-1.64083633	0.02710735
C	-2.89294764	-1.52505861	-0.24402063
H	-3.10914617	-1.66767479	-1.29339133
H	-3.47331935	-2.25112790	0.31319428
H	-3.53781678	0.03255325	1.17537712

H	-3.96234489	0.36270112	-0.50612079
H	-0.88730965	-2.35325633	-0.61887071
H	0.82204684	2.43677341	-0.23850934
C	-1.91568409	2.06883219	0.01597156
H	-1.44192782	2.38734639	0.93876742
H	-1.34220623	2.46165579	-0.80989323
H	-2.93133271	2.43511575	-0.02694523
H	1.51662212	-1.90155706	0.13690350
C	4.19393893	-0.89780096	0.09766042
H	4.80785702	-0.68505160	-0.77487076
H	4.78323894	-0.54424032	0.94123033
H	4.10340779	-1.97657918	0.18550203
H	-1.20507606	-1.98476024	1.04298592

Time = 33.50 fs
E(CASSCF/6-31G(d)) = -479.5588942

C	-0.90892022	-0.22911737	-0.10132442
C	0.59215309	0.17269508	-0.07973963
C	1.15680973	1.43441495	-0.15649773
C	1.55448691	-0.83734186	0.05292235
C	2.88529418	-0.17549896	0.02538701
C	2.62277812	1.20884022	-0.09197636
N	-1.94120538	0.61944987	-0.04811090
C	-3.22451826	-0.09986468	0.14076847
H	3.31455061	2.02140383	-0.14707726
C	-1.38329485	-1.63966200	0.02594124
C	-2.89368503	-1.52519091	-0.24371085
H	-3.11167647	-1.66649628	-1.29293016
H	-3.47284179	-2.25116822	0.31447043
H	-3.53942991	0.03574858	1.17358707
H	-3.96060890	0.36226691	-0.50722299
H	-0.88205865	-2.34982911	-0.62035544
H	0.81235912	2.43492968	-0.23983878
C	-1.91556179	2.06801995	0.01528335
H	-1.44388819	2.38928675	0.93794549
H	-1.34488325	2.46394815	-0.81051391
H	-2.93192241	2.43039109	-0.02718698
H	1.50584524	-1.89987016	0.13604950
C	4.19391680	-0.89716238	0.09755134
H	4.80902518	-0.68617331	-0.77521117
H	4.78515974	-0.54527768	0.94112552
H	4.09717735	-1.97578514	0.18535417
H	-1.20378370	-1.98121497	1.04290592

Time = 34.00 fs
E(CASSCF/6-31G(d)) = -479.5608304

C	-0.90908032	-0.23223192	-0.10187351
C	0.59337795	0.17307687	-0.08000258
C	1.15933337	1.43463178	-0.15708822
C	1.55955011	-0.83586123	0.05328016
C	2.88582710	-0.17963812	0.02625274
C	2.62094136	1.21142682	-0.09222213
N	-1.94388926	0.62006906	-0.04503715
C	-3.22478610	-0.09934500	0.13979194
H	3.32108992	2.01937585	-0.14660816
C	-1.38492781	-1.63846868	0.02480875
C	-2.89441518	-1.52540538	-0.24337816
H	-3.11401408	-1.66548108	-1.29275049
H	-3.47239967	-2.25114169	0.31570839
H	-3.54123029	0.03843127	1.17171794
H	-3.95919138	0.36155744	-0.50804077
H	-0.87665204	-2.34598579	-0.62216619
H	0.80093186	2.43310213	-0.24126004
C	-1.91536177	2.06712619	0.01462612
H	-1.44615327	2.39157000	0.93711657
H	-1.34780224	2.46644358	-0.81133186
H	-2.93246322	2.42577124	-0.02744912
H	1.49350124	-1.89851956	0.13516661
C	4.19398248	-0.89641859	0.09743366
H	4.81014698	-0.68759490	-0.77582029
H	4.78703521	-0.54656125	0.94131859
H	4.09049669	-1.97470518	0.18517650
H	-1.20237005	-1.97792755	1.04316602

Time = 34.50 fs
E(CASSCF/6-31G(d)) = -479.5628556

C	-0.90891171	-0.23519495	-0.10250343
C	0.59408628	0.17318374	-0.08024410
C	1.16239075	1.43488341	-0.15767264
C	1.56508109	-0.83421468	0.05364204
C	2.88599822	-0.18410431	0.02714832
C	2.61884117	1.21427776	-0.09249897
N	-1.94667208	0.62060702	-0.04195106
C	-3.22495776	-0.09860309	0.13880651
H	3.32827034	2.01722246	-0.14610672
C	-1.38659374	-1.63727892	0.02371112
C	-2.89513971	-1.52569776	-0.24302850
H	-3.11614673	-1.66465768	-1.29282435
H	-3.47200902	-2.25105519	0.31691277
H	-3.54324514	0.04056861	1.16978872
H	-3.95819100	0.36062996	-0.50867336

H	-0.87124142	-2.34169086	-0.62419738
H	0.78801500	2.43108642	-0.24275039
C	-1.91509819	2.06616125	0.01400483
H	-1.44868921	2.39417728	0.93629051
H	-1.35092834	2.46907302	-0.81237490
H	-2.93299831	2.42133570	-0.02773129
H	1.47979121	-1.89742543	0.13425960
C	4.19414475	-0.89558614	0.09730876
H	4.81117728	-0.68932265	-0.77666792
H	4.78882128	-0.54810252	0.94177972
H	4.08347716	-1.97333691	0.18497101
H	-1.20087122	-1.97492298	1.04368682

Time = 35.00 fs
E(CASSCF/6-31G(d)) = -479.5647889

C	-0.90845779	-0.23795283	-0.10321464
C	0.59431663	0.17301558	-0.08046335
C	1.16592541	1.43518983	-0.15825412
C	1.57100779	-0.83243189	0.05400890
C	2.88580888	-0.18879848	0.02806571
C	2.61653071	1.21733530	-0.09280074
N	-1.94949481	0.62103203	-0.03886107
C	-3.22504045	-0.09764776	0.13782522
H	3.33586540	2.01486033	-0.14557283
C	-1.38827102	-1.63611483	0.02265107
C	-2.89586005	-1.52606211	-0.24266966
H	-3.11806118	-1.66405089	-1.29310424
H	-3.47168853	-2.25091958	0.31809160
H	-3.54549620	0.04213824	1.16782343
H	-3.95769681	0.35954779	-0.50922141
H	-0.86597661	-2.33692080	-0.62634452
H	0.77390798	2.42863788	-0.24428228
C	-1.91478704	2.06513721	0.01342300
H	-1.45145549	2.39708315	0.93547826
H	-1.35422873	2.47175903	-0.81365910
H	-2.93356205	2.41715611	-0.02803283
H	1.46495079	-1.89644271	0.13333005
C	4.19441045	-0.89468185	0.09717799
H	4.81207158	-0.69135663	-0.77771833
H	4.79047472	-0.54990787	0.94247284
H	4.07623620	-1.97169178	0.18474088
H	-1.19932542	-1.97221678	1.04436558

Time = 35.50 fs
E(CASSCF/6-31G(d)) = -479.5664591

C	-0.90776583	-0.24045627	-0.10400626
C	0.59411721	0.17257777	-0.08065960
C	1.16986993	1.43556941	-0.15883616
C	1.57724659	-0.83055017	0.05438186
C	2.88526756	-0.19361862	0.02899669
C	2.61406766	1.22054102	-0.09312100
N	-1.95229772	0.62131510	-0.03577577
C	-3.22504351	-0.09649135	0.13686006
H	3.34362987	2.01220374	-0.14500671
C	-1.38993763	-1.63499667	0.02163280
C	-2.89657729	-1.52649101	-0.24231086
H	-3.11974371	-1.66368188	-1.29352759
H	-3.47145864	-2.25074841	0.31925543
H	-3.54799964	0.04312814	1.16585020
H	-3.95777988	0.35837536	-0.50977908
H	-0.86099932	-2.33167072	-0.62851282
H	0.75894982	2.42550345	-0.24582571
C	-1.91444571	2.06406782	0.01288287
H	-1.45440655	2.40025668	0.93469042
H	-1.35767367	2.47441687	-0.81518753
H	-2.93417764	2.41329481	-0.02835296
H	1.44925246	-1.89537750	0.13237758
C	4.19478410	-0.89372275	0.09704274
H	4.81278906	-0.69369099	-0.77893330
H	4.79195629	-0.55197854	0.94335871
H	4.06889436	-1.96979612	0.18449047
H	-1.19777138	-1.96981533	1.04508321

Time = 36.00 fs
E(CASSCF/6-31G(d)) = -479.5677305

C	-0.90688586	-0.24266133	-0.10487669
C	0.59354362	0.17188117	-0.08083220
C	1.17414939	1.43603675	-0.15942238
C	1.58370510	-0.82861207	0.05476223
C	2.88438986	-0.19846325	0.02993313
C	2.61151153	1.22383717	-0.09345321
N	-1.95502181	0.62143117	-0.03270335
C	-3.22497875	-0.09514915	0.13592122
H	3.35131702	2.00917355	-0.14440925
C	-1.39157210	-1.63394200	0.02066171
C	-2.89729235	-1.52697582	-0.24196206
H	-3.12118187	-1.66356830	-1.29402247
H	-3.47134001	-2.25055626	0.32041615
H	-3.55076507	0.04353726	1.16389899
H	-3.95848475	0.35717301	-0.51042591
H	-0.85643541	-2.32596112	-0.63062452

H	0.74350294	2.42144912	-0.24735094
C	-1.91409292	2.06296803	0.01238540
H	-1.45749316	2.40366105	0.93393549
H	-1.36123647	2.47695650	-0.81695075
H	-2.93485456	2.40980233	-0.02869058
H	1.43299917	-1.89400653	0.13140158
C	4.19526725	-0.89272559	0.09690429
H	4.81329461	-0.69631204	-0.78027511
H	4.79323248	-0.55430802	0.94439768
H	4.06157363	-1.96769002	0.18422525
H	-1.19624685	-1.96772051	1.04571641

Time = 36.50 fs
E(CASSCF/6-31G(d)) = -479.5685071

C	-0.90586927	-0.24453059	-0.10582368
C	0.59265758	0.17094171	-0.08098063
C	1.17868318	1.43660070	-0.16001626
C	1.59028298	-0.82666335	0.05515138
C	2.88319898	-0.20323301	0.03086709
C	2.60892193	1.22716702	-0.09379074
N	-1.95761013	0.62135986	-0.02965159
C	-3.22486077	-0.09363896	0.13501673
H	3.35869177	2.00571010	-0.14378278
C	-1.39315422	-1.63296477	0.01974409
C	-2.89800590	-1.52750698	-0.24163328
H	-3.12236563	-1.66372458	-1.29451395
H	-3.47135206	-2.25035692	0.32158556
H	-3.55379526	0.04337515	1.16199980
H	-3.95982319	0.35599294	-0.51122065
H	-0.85238983	-2.31984117	-0.63262450
H	0.72793833	2.41628717	-0.24883102
C	-1.91374831	2.06185362	0.01193051
H	-1.46066400	2.40725442	0.93321851
H	-1.36489300	2.47928492	-0.81892802
H	-2.93558846	2.40671680	-0.02904437
H	1.41651975	-1.89210290	0.13040294
C	4.19585824	-0.89170682	0.09676389
H	4.81356121	-0.69919846	-0.78170920
H	4.79427785	-0.55688187	0.94555238
H	4.05439474	-1.96542603	0.18395162
H	-1.19478679	-1.96593161	1.04614677

Time = 37.00 fs
E(CASSCF/6-31G(d)) = -479.5687414

C	-0.90476750	-0.24603391	-0.10684446

C	0.59152517	0.16977993	-0.08110440
C	1.18338744	1.43726303	-0.16062101
C	1.59687377	-0.82475063	0.05555060
C	2.88172576	-0.20783233	0.03179099
C	2.60635678	1.23047516	-0.09412702
N	-1.96000904	0.62108614	-0.02662768
C	-3.22470698	-0.09198059	0.13415218
H	3.36554404	2.00178273	-0.14313150
C	-1.39466565	-1.63207467	0.01888666
C	-2.89871839	-1.52807427	-0.24133392
H	-3.12328868	-1.66416223	-1.29493065
H	-3.47151145	-2.25016159	0.32277404
H	-3.55708575	0.04266163	1.16018044
H	-3.96177028	0.35487648	-0.51219694
H	-0.84894264	-2.31339015	-0.63448399
H	0.71261970	2.40989737	-0.25024393
C	-1.91343202	2.06074073	0.01151737
H	-1.46386712	2.41099065	0.93253990
H	-1.36862061	2.48130900	-0.82108943
H	-2.93636229	2.40406348	-0.02941289
H	1.40016070	-1.88946209	0.12938576
C	4.19655194	-0.89068237	0.09662271
H	4.81357154	-0.70232127	-0.78320631
H	4.79507666	-0.55967726	0.94678981
H	4.04747499	-1.96306670	0.18367673
H	-1.19342172	-1.96444766	1.04626990

Time = 37.50 fs
E(CASSCF/6-31G(d)) = -479.5684296

C	-0.90363068	-0.24714876	-0.10793606
C	0.59021496	0.16842024	-0.08120310
C	1.18817763	1.43801771	-0.16123938
C	1.60336732	-0.82291887	0.05596110
C	2.88000830	-0.21217120	0.03269771
C	2.60387057	1.23370790	-0.09445557
N	-1.96216923	0.62060077	-0.02363806
C	-3.22453749	-0.09019551	0.13333069
H	3.37170053	1.99739558	-0.14246176
C	-1.39609043	-1.63127703	0.01809610
C	-2.89943006	-1.52866718	-0.24107207
H	-3.12394975	-1.66489041	-1.29521103
H	-3.47183075	-2.24997756	0.32398940
H	-3.56062473	0.04142617	1.15846471
H	-3.96426376	0.35385317	-0.51336183
H	-0.84614638	-2.30671638	-0.63620231
H	0.69788734	2.40224184	-0.25157437

C	-1.91316417	2.05964537	0.01114469
H	-1.46705101	2.41482011	0.93189460
H	-1.37239653	2.48293862	-0.82339873
H	-2.93714870	2.40185504	-0.02979465
H	1.38427467	-1.88592568	0.12835876
C	4.19733969	-0.88966758	0.09648178
H	4.81331878	-0.70564378	-0.78474354
H	4.79562345	-0.56266263	0.94808253
H	4.04092631	-1.96068108	0.18340811
H	-1.19217622	-1.96326981	1.04600345

Time = 38.00 fs
E(CASSCF/6-31G(d)) = -479.5676129

C	-0.90250678	-0.24786066	-0.10909511
C	0.58879625	0.16689029	-0.08127639
C	1.19297032	1.43885065	-0.16187356
C	1.60965177	-0.82120972	0.05638379
C	2.87809155	-0.21616564	0.03358066
C	2.60151347	1.23681321	-0.09477009
N	-1.96404663	0.61990045	-0.02068840
C	-3.22437461	-0.08830659	0.13255318
H	3.37703285	1.99259396	-0.14178217
C	-1.39741528	-1.63057248	0.01737850
C	-2.90014090	-1.52927512	-0.24085405
H	-3.12435320	-1.66591568	-1.29530779
H	-3.47231715	-2.24980651	0.32523562
H	-3.56439264	0.03970709	1.15687001
H	-3.96720588	0.35294092	-0.51469670
H	-0.84402620	-2.29995410	-0.63780593
H	0.68404566	2.39337379	-0.25281499
C	-1.91296438	2.05858280	0.01081099
H	-1.47016575	2.41869075	0.93127194
H	-1.37619662	2.48408964	-0.82581650
H	-2.93791368	2.40009233	-0.03018814
H	1.36921024	-1.88140126	0.12733590
C	4.19820943	-0.88867719	0.09634211
H	4.81280769	-0.70912294	-0.78630574
H	4.79592436	-0.56579902	0.94940999
H	4.03485288	-1.95834173	0.18315343
H	-1.19106790	-1.96240207	1.04529194

Time = 38.50 fs
E(CASSCF/6-31G(d)) = -479.5663575

C	-0.90144046	-0.24816300	-0.11031807
C	0.58733718	0.16522020	-0.08132405

C	1.19768551	1.43973997	-0.16252516
C	1.61561651	-0.81965959	0.05681933
C	2.87602612	-0.21973895	0.03443383
C	2.59933013	1.23974110	-0.09506448
N	-1.96560317	0.61898788	-0.01778343
C	-3.22424207	-0.08633786	0.13181871
H	3.38146400	1.98746428	-0.14110350
C	-1.39862978	-1.62995746	0.01673885
C	-2.90085071	-1.52988768	-0.24068392
H	-3.12450971	-1.66724237	-1.29519187
H	-3.47297172	-2.24964401	0.32651225
H	-3.56836237	0.03755064	1.15540610
H	-3.97046896	0.35214836	-0.51616106
H	-0.84258090	-2.29325811	-0.63934592
H	0.67135061	2.38343964	-0.25396711
C	-1.91285111	2.05756706	0.01051488
H	-1.47316369	2.42254924	0.93065579
H	-1.37999379	2.48468691	-0.82830342
H	-2.93862069	2.39876546	-0.03059196
H	1.35529837	-1.87587694	0.12633685
C	4.19914595	-0.88772525	0.09620465
H	4.81205407	-0.71270991	-0.78788506
H	4.79599656	-0.56904057	0.95075821
H	4.02934965	-1.95612065	0.18292014
H	-1.19010635	-1.96185238	1.04411081

Time = 39.00 fs
E(CASSCF/6-31G(d)) = -479.5647521

C	-0.90047204	-0.24805678	-0.11160141
C	0.58590300	0.16344172	-0.08134592
C	1.20224836	1.44065669	-0.16319507
C	1.62115502	-0.81829826	0.05726812
C	2.87386702	-0.22282242	0.03525188
C	2.59735916	1.24244384	-0.09533294
N	-1.96680738	0.61787155	-0.01492694
C	-3.22416417	-0.08431434	0.13112501
H	3.38497081	1.98213190	-0.14043842
C	-1.39972633	-1.62942470	0.01618058
C	-2.90155915	-1.53049485	-0.24056332
H	-3.12443649	-1.66887266	-1.29485472
H	-3.47378910	-2.24947945	0.32781424
H	-3.57249974	0.03500994	1.15407444
H	-3.97390331	0.35147805	-0.51769839
H	-0.84178618	-2.28679647	-0.64089303
H	0.66000040	2.37267620	-0.25504104
C	-1.91284124	2.05661040	0.01025523

H	-1.47599983	2.42634227	0.93002538
H	-1.38375680	2.48466707	-0.83082318
H	-2.93923508	2.39785528	-0.03100487
H	1.34284018	-1.86943028	0.12538709
C	4.20013138	-0.88682507	0.09607026
H	4.81108377	-0.71635112	-0.78948000
H	4.79586709	-0.57233566	0.95211897
H	4.02450080	-1.95408505	0.18271510
H	-1.18929284	-1.96163309	1.04246795

Time = 39.50 fs
E(CASSCF/6-31G(d)) = -479.5628993

C	-0.89963685	-0.24755014	-0.11294160
C	0.58455469	0.16158759	-0.08134204
C	1.20659064	1.44156569	-0.16388352
C	1.62616773	-0.81714789	0.05773030
C	2.87167193	-0.22535594	0.03603014
C	2.59563281	1.24487628	-0.09557002
N	-1.96763484	0.61656534	-0.01212170
C	-3.22416469	-0.08226197	0.13046908
H	3.38758286	1.97675653	-0.13980120
C	-1.40069995	-1.62896400	0.01570503
C	-2.90226575	-1.53108715	-0.24049138
H	-3.12415697	-1.67080652	-1.29430867
H	-3.47475762	-2.24929655	0.32913209
H	-3.57676414	0.03214389	1.15286803
H	-3.97734675	0.35093033	-0.51924333
H	-0.84159952	-2.28074160	-0.64253098
H	0.65012982	2.36140146	-0.25605574
C	-1.91294951	2.05572299	0.01003134
H	-1.47863207	2.43001797	0.92935657
H	-1.38744961	2.48398076	-0.83334502
H	-2.93972828	2.39733493	-0.03142589
H	1.33209574	-1.86223125	0.12451737
C	4.20114572	-0.88598913	0.09593976
H	4.80993137	-0.71998973	-0.79109388
H	4.79557157	-0.57562855	0.95348844
H	4.02037853	-1.95229412	0.18254434
H	-1.18862002	-1.96176031	1.04040280

Time = 40.00 fs
E(CASSCF/6-31G(d)) = -479.5609112

C	-0.89896436	-0.24665793	-0.11433512
C	0.58334755	0.15969076	-0.08131260
C	1.21065182	1.44242715	-0.16459005

C	1.63056479	-0.81622227	0.05820575
C	2.86949926	-0.22728870	0.03676468
C	2.59417699	1.24699655	-0.09577067
N	-1.96806866	0.61508819	-0.00936942
C	-3.22426574	-0.08020738	0.12984775
H	3.38937770	1.97152476	-0.13920709
C	-1.40154788	-1.62856325	0.01531105
C	-2.90296994	-1.53165568	-0.24046486
H	-3.12370016	-1.67304145	-1.29358560
H	-3.47585987	-2.24907443	0.33045253
H	-3.58110963	0.02901589	1.15177213
H	-3.98063488	0.35050722	-0.52072931
H	-0.84196663	-2.27526040	-0.64434809
H	0.64180849	2.35000052	-0.25703789
C	-1.91318804	2.05491243	0.00984290
H	-1.48102124	2.43352751	0.92862335
H	-1.39103140	2.48259424	-0.83584549
H	-2.94008151	2.39717151	-0.03185427
H	1.32327479	-1.85453978	0.12376289
C	4.20216758	-0.88522900	0.09581387
H	4.80863816	-0.72356701	-0.79273269
H	4.79515216	-0.57886112	0.95486511
H	4.01704185	-1.95079615	0.18241278
H	-1.18807246	-1.96225303	1.03798338

Time = 40.50 fs
E(CASSCF/6-31G(d)) = -479.5589035

C	-0.89847764	-0.24540112	-0.11577853
C	0.58233012	0.15778378	-0.08125800
C	1.21437967	1.44319815	-0.16531360
C	1.63426873	-0.81552646	0.05869410
C	2.86740611	-0.22857980	0.03745233
C	2.59301164	1.24876673	-0.09593039
N	-1.96809964	0.61346349	-0.00667081
C	-3.22448660	-0.07817775	0.12925824
H	3.39047206	1.96663944	-0.13867173
C	-1.40226906	-1.62820941	0.01499462
C	-2.90367112	-1.53219223	-0.24047848
H	-3.12309952	-1.67557228	-1.29273400
H	-3.47707381	-2.24878939	0.33175964
H	-3.58548640	0.02569254	1.15076576
H	-3.98361136	0.35021584	-0.52209608
H	-0.84282852	-2.27050402	-0.64642825
H	0.63504399	2.33890652	-0.25802055
C	-1.91356602	2.05418358	0.00968998
H	-1.48313102	2.43682676	0.92779979

H	-1.39445706	2.48049056	-0.83830949
H	-2.94028874	2.39732777	-0.03228955
H	1.31653034	-1.84669731	0.12316224
C	4.20317496	-0.88455518	0.09569329
H	4.80724955	-0.72702378	-0.79440239
H	4.79465504	-0.58197465	0.95624732
H	4.01453570	-1.94962595	0.18232404
H	-1.18762732	-1.96313153	1.03530148

Time = 41.00 fs
E(CASSCF/6-31G(d)) = -479.5570104

C	-0.89819357	-0.24380623	-0.11726825
C	0.58154399	0.15589840	-0.08117898
C	1.21773003	1.44383463	-0.16605267
C	1.63721612	-0.81505711	0.05919486
C	2.86544628	-0.22919823	0.03809067
C	2.59215154	1.25015334	-0.09604517
N	-1.96772630	0.61171840	-0.00402561
C	-3.22484276	-0.07620059	0.12869845
H	3.39100952	1.96230935	-0.13821060
C	-1.40286358	-1.62788957	0.01474871
C	-2.90436862	-1.53268923	-0.24052540
H	-3.12239166	-1.67839031	-1.29181416
H	-3.47837384	-2.24841633	0.33303601
H	-3.58984134	0.02224293	1.14982303
H	-3.98613607	0.35007123	-0.52329566
H	-0.84412893	-2.26659823	-0.64884124
H	0.62979070	2.32857685	-0.25904091
C	-1.91408942	2.05353850	0.00957285
H	-1.48492809	2.43987673	0.92686141
H	-1.39767878	2.47766895	-0.84072969
H	-2.94035783	2.39776293	-0.03273141
H	1.31195578	-1.83911248	0.12275586
C	4.20414592	-0.88397693	0.09557864
H	4.80581333	-0.73030237	-0.79610660
H	4.79412865	-0.58491224	0.95763109
H	4.01289036	-1.94880459	0.18228035
H	-1.18725574	-1.96441480	1.03246531

Time = 41.50 fs
E(CASSCF/6-31G(d)) = -479.5553422

C	-0.89812186	-0.24190469	-0.11880105
C	0.58102253	0.15406484	-0.08107652
C	1.22066738	1.44429369	-0.16680534
C	1.63936036	-0.81480265	0.05970738

C	2.86366777	-0.22912427	0.03867815
C	2.59160668	1.25112908	-0.09611179
N	-1.96695501	0.60988327	-0.00143240
C	-3.22534514	-0.07430344	0.12816729
H	3.39114552	1.95873187	-0.13783781
C	-1.40333216	-1.62759214	0.01456322
C	-2.90506180	-1.53313982	-0.24059777
H	-3.12161483	-1.68148377	-1.29089331
H	-3.47973296	-2.24793187	0.33426467
H	-3.59412169	0.01873679	1.14891699
H	-3.98809453	0.35009849	-0.52429869
H	-0.84582084	-2.26363443	-0.65163490
H	0.62596231	2.31946265	-0.26013768
C	-1.91476076	2.05297658	0.00949182
H	-1.48638171	2.44264659	0.92578750
H	-1.40064750	2.47414567	-0.84310659
H	-2.94031194	2.39843542	-0.03317983
H	1.30958419	-1.83223923	0.12258475
C	4.20505962	-0.88350204	0.09547044
H	4.80437603	-0.73334710	-0.79784371
H	4.79361986	-0.58761950	0.95900739
H	4.01212067	-1.94833816	0.18228257
H	-1.18692383	-1.96611910	1.02959213

Time = 42.00 fs
E(CASSCF/6-31G(d)) = -479.5540362

C	-0.89826543	-0.23973224	-0.12037371
C	0.58079099	0.15231143	-0.08095193
C	1.22316334	1.44453609	-0.16756960
C	1.64067180	-0.81474506	0.06023102
C	2.86211148	-0.22834929	0.03921409
C	2.59138362	1.25167345	-0.09612775
N	-1.96579986	0.60799097	0.00111116
C	-3.22599933	-0.07251345	0.12766478
H	3.39103050	1.95608087	-0.13756568
C	-1.40367559	-1.62730759	0.01442527
C	-2.90575003	-1.53353778	-0.24068741
H	-3.12080711	-1.68483639	-1.29003866
H	-3.48112381	-2.24731566	0.33543033
H	-3.59827545	0.01524368	1.14802113
H	-3.98940196	0.35033317	-0.52509687
H	-0.84787150	-2.26166416	-0.65482816
H	0.62345081	2.31197975	-0.26134791
C	-1.91557912	2.05249452	0.00944693
H	-1.48746414	2.44511323	0.92456202
H	-1.40331553	2.46995188	-0.84544605

H	-2.94018793	2.39930266	-0.03363480
H	1.30939516	-1.82654877	0.12268814
C	4.20589682	-0.88313663	0.09536913
H	4.80298122	-0.73610625	-0.79960483
H	4.79317242	-0.59004699	0.96036024
H	4.01222589	-1.94821871	0.18233018
H	-1.18659529	-1.96825439	1.02679830

Time = 42.50 fs
E(CASSCF/6-31G(d)) = -479.5532049

C	-0.89862032	-0.23732832	-0.12198323
C	0.58086615	0.15066420	-0.08080688
C	1.22519587	1.44452877	-0.16834351
C	1.64113841	-0.81486184	0.06076530
C	2.86080970	-0.22687626	0.03969864
C	2.59148632	1.25177400	-0.09609144
N	-1.96428241	0.60607620	0.00360845
C	-3.22680530	-0.07085704	0.12719199
H	3.39079321	1.95449211	-0.13740391
C	-1.40389443	-1.62702913	0.01431973
C	-2.90643272	-1.53387753	-0.24078648
H	-3.12000484	-1.68842720	-1.28931118
H	-3.48252043	-2.24655263	0.33652095
H	-3.60225384	0.01183173	1.14711228
H	-3.99000713	0.35082111	-0.52570502
H	-0.85026515	-2.26069585	-0.65840743
H	0.62214687	2.30647640	-0.26270353
C	-1.91654021	2.05208670	0.00943771
H	-1.48815081	2.44726239	0.92317473
H	-1.40563919	2.46513236	-0.84775728
H	-2.94003437	2.40032227	-0.03409625
H	1.31132402	-1.82249405	0.12310129
C	4.20664054	-0.88288489	0.09527505
H	4.80166727	-0.73853297	-0.80137236
H	4.79282457	-0.59215126	0.96166539
H	4.01318960	-1.94842561	0.18242138
H	-1.18623332	-1.97082177	1.02419005

Time = 43.00 fs
E(CASSCF/6-31G(d)) = -479.5529486

C	-0.89917578	-0.23473563	-0.12362685
C	0.58125617	0.14914658	-0.08064340
C	1.22674817	1.44424703	-0.16912542
C	1.64076519	-0.81512879	0.06130998
C	2.85978524	-0.22471984	0.04013290

C	2.59191700	1.25142738	-0.09600224
N	-1.96243148	0.60417497	0.00606375
C	-3.22775714	-0.06935935	0.12675092
H	3.39052634	1.95405195	-0.13735892
C	-1.40398887	-1.62675306	0.01422996
C	-2.90710935	-1.53415411	-0.24088809
H	-3.11924107	-1.69222983	-1.28875964
H	-3.48389950	-2.24563438	0.33752886
H	-3.60601295	0.00856660	1.14617287
H	-3.98989331	0.35161703	-0.52616064
H	-0.85300317	-2.26069501	-0.66232595
H	0.62196035	2.30320530	-0.26422814
C	-1.91763651	2.05174544	0.00946293
H	-1.48842079	2.44908888	0.92162178
H	-1.40758151	2.45974346	-0.85005001
H	-2.93990824	2.40145244	-0.03456390
H	1.31527494	-1.82046999	0.12385298
C	4.20727658	-0.88274890	0.09518839
H	4.80046584	-0.74058622	-0.80311933
H	4.79260754	-0.59389589	0.96288983
H	4.01497980	-1.94892737	0.18255330
H	-1.18580288	-1.97381082	1.02185447

Time = 43.50 fs
E(CASSCF/6-31G(d)) = -479.5533237

C	-0.89991427	-0.23199943	-0.12530216
C	0.58196045	0.14777908	-0.08046389
C	1.22780782	1.44367637	-0.16991416
C	1.63957320	-0.81552325	0.06186536
C	2.85905110	-0.22190657	0.04051883
C	2.59267649	1.25064012	-0.09586055
N	-1.96028271	0.60232395	0.00848233
C	-3.22884334	-0.06804380	0.12634418
H	3.39027646	1.95478900	-0.13743340
C	-1.40395880	-1.62647885	0.01413873
C	-2.90777943	-1.53436323	-0.24098683
H	-3.11854438	-1.69621228	-1.28841566
H	-3.48524142	-2.24456026	0.33845164
H	-3.60951555	0.00551032	1.14519277
H	-3.98907691	0.35278212	-0.52652178
H	-0.85610163	-2.26158784	-0.66650665
H	0.62283659	2.30230156	-0.26593428
C	-1.91885753	2.05146147	0.00952035
H	-1.48825741	2.45059671	0.91990561
H	-1.40911484	2.45385102	-0.85233184
H	-2.93987068	2.40265230	-0.03503704

H	1.32113529	-1.82077128	0.12496292
C	4.20779382	-0.88272848	0.09510922
H	4.79940077	-0.74223140	-0.80480974
H	4.79254444	-0.59525198	0.96399219
H	4.01754947	-1.94968418	0.18272219
H	-1.18527289	-1.97719745	1.01985194

Time = 44.00 fs
E(CASSCF/6-31G(d)) = -479.5543343

C	-0.90081172	-0.22916698	-0.12700727
C	0.58296952	0.14657907	-0.08027109
C	1.22836660	1.44281357	-0.17070919
C	1.63759824	-0.81602724	0.06243239
C	2.85861057	-0.21847470	0.04085935
C	2.59376423	1.24942914	-0.09566791
N	-1.95787808	0.60055997	0.01087032
C	-3.23004716	-0.06693147	0.12597469
H	3.39003973	1.95667084	-0.13762610
C	-1.40380403	-1.62620900	0.01402929
C	-2.90844255	-1.53450129	-0.24107908
H	-3.11793806	-1.70033665	-1.28829036
H	-3.48653092	-2.24333783	0.33929255
H	-3.61273252	0.00272029	1.14417045
H	-3.98760382	0.35438062	-0.52686300
H	-0.85958666	-2.26326748	-0.67084797
H	0.62476579	2.30377065	-0.26782177
C	-1.92019018	2.05122436	0.00960661
H	-1.48764903	2.45179865	0.91803447
H	-1.41022314	2.44752811	-0.85460559
H	-2.93998219	2.40388192	-0.03551436
H	1.32878857	-1.82355459	0.12643938
C	4.20818435	-0.88282103	0.09503745
H	4.79848780	-0.74344076	-0.80639988
H	4.79264990	-0.59619842	0.96492419
H	4.02083730	-1.95065061	0.18292366
H	-1.18461805	-1.98094247	1.01821072

Time = 44.50 fs
E(CASSCF/6-31G(d)) = -479.5559187

C	-0.90183776	-0.22628689	-0.12874091
C	0.58426505	0.14556060	-0.08006808
C	1.22842077	1.44166678	-0.17151054
C	1.63488933	-0.81663013	0.06301288
C	2.85845784	-0.21447399	0.04115826
C	2.59517767	1.24782178	-0.09542703

N	-1.95526521	0.59891937	0.01323475
C	-3.23134736	-0.06604057	0.12564521
H	3.38976416	1.95960587	-0.13793187
C	-1.40352475	-1.62594857	0.01388631
C	-2.90909829	-1.53456545	-0.24116320
H	-3.11743976	-1.70455932	-1.28837312
H	-3.48775723	-2.24198261	0.34006041
H	-3.61564392	0.00024834	1.14311343
H	-3.98554361	0.35647555	-0.52726958
H	-0.86348822	-2.26560260	-0.67523204
H	0.62778350	2.30748808	-0.26987731
C	-1.92161920	2.05102301	0.00971714
H	-1.48658997	2.45271552	0.91602134
H	-1.41090365	2.44085305	-0.85686730
H	-2.94029756	2.40510230	-0.03599370
H	1.33812319	-1.82881077	0.12827737
C	4.20844348	-0.88302148	0.09497284
H	4.79773471	-0.74419366	-0.80784061
H	4.79293025	-0.59672167	0.96563291
H	4.02476874	-1.95177833	0.18315291
H	-1.18382010	-1.98499121	1.01692467

Time = 45.00 fs
E(CASSCF/6-31G(d)) = -479.5579483

C	-0.90295623	-0.22340840	-0.13050252
C	0.58581991	0.14473423	-0.07985825
C	1.22797206	1.44025483	-0.17231882
C	1.63150734	-0.81733028	0.06360961
C	2.85857913	-0.20996523	0.04142030
C	2.59691134	1.24585545	-0.09514175
N	-1.95249657	0.59743753	0.01558343
C	-3.23271919	-0.06538584	0.12535786
H	3.38935799	1.96345035	-0.13834198
C	-1.40312198	-1.62570455	0.01369667
C	-2.90974624	-1.53455372	-0.24123944
H	-3.11706161	-1.70883147	-1.28863236
H	-3.48891393	-2.24051738	0.34076920
H	-3.61823969	-0.00186021	1.14203782
H	-3.98298193	0.35912371	-0.52783038
H	-0.86783335	-2.26844706	-0.67953415
H	0.63196218	2.31321057	-0.27207547
C	-1.92312756	2.05084610	0.00984617
H	-1.48508144	2.45337526	0.91388262
H	-1.41116788	2.43390765	-0.85910504
H	-2.94086111	2.40627527	-0.03647182
H	1.34903492	-1.83635334	0.13045776

C	4.20856958	-0.88332229	0.09491494
H	4.79714198	-0.74447666	-0.80908022
H	4.79338419	-0.59681540	0.96606379
H	4.02925749	-1.95301861	0.18340500
H	-1.18286857	-1.98927429	1.01595402

Time = 45.50 fs
E(CASSCF/6-31G(d)) = -479.5602413

C	-0.90412566	-0.22058057	-0.13229241
C	0.58759841	0.14410700	-0.07964529
C	1.22702904	1.43860571	-0.17313502
C	1.62752393	-0.81813515	0.06422634
C	2.85895393	-0.20501960	0.04165104
C	2.59895558	1.24357686	-0.09481711
N	-1.94962849	0.59614817	0.01792487
C	-3.23413548	-0.06497794	0.12511367
H	3.38870292	1.96801932	-0.13884466
C	-1.40259805	-1.62548514	0.01345011
C	-2.91038589	-1.53446498	-0.24130968
H	-3.11681070	-1.71309980	-1.28901828
H	-3.48999828	-2.23897072	0.34143700
H	-3.62051977	-0.00356723	1.14096697
H	-3.98001173	0.36237022	-0.52862966
H	-0.87263966	-2.27164996	-0.68363258
H	0.63739507	2.32059773	-0.27438094
C	-1.92469698	2.05068251	0.00998695
H	-1.48313262	2.45381152	0.91163628
H	-1.41104179	2.42677572	-0.86129849
H	-2.94170269	2.40736337	-0.03694421
H	1.36142231	-1.84582681	0.13294756
C	4.20856375	-0.88371352	0.09486316
H	4.79670379	-0.74428325	-0.81006777
H	4.79400380	-0.59647969	0.96616389
H	4.03420734	-1.95432462	0.18367506
H	-1.18176080	-1.99370948	1.01522942

Time = 46.00 fs
E(CASSCF/6-31G(d)) = -479.5625820

C	-0.90530041	-0.21785145	-0.13411168
C	0.58955673	0.14368231	-0.07943309
C	1.22560860	1.43675453	-0.17396028
C	1.62302041	-0.81905999	0.06486773
C	2.85955654	-0.19971786	0.04185693
C	2.60129516	1.24104091	-0.09445920
N	-1.94671994	0.59508268	0.02026819

C	-3.23556793	-0.06482297	0.12491208
H	3.38766996	1.97310150	-0.13942584
C	-1.40195712	-1.62529891	0.01313946
C	-2.91101668	-1.53429906	-0.24137700
H	-3.11668989	-1.71730786	-1.28946759
H	-3.49101026	-2.23737541	0.34208474
H	-3.62249400	-0.00484255	1.13992964
H	-3.97672416	0.36624289	-0.52973878
H	-0.87790937	-2.27506517	-0.68741804
H	0.64417716	2.32923929	-0.27675149
C	-1.92630835	2.05052173	0.01013194
H	-1.48076131	2.45406257	0.90930041
H	-1.41056493	2.41954218	-0.86342010
H	-2.94283440	2.40833022	-0.03740495
H	1.37517780	-1.85673355	0.13570150
C	4.20842951	-0.88418298	0.09481670
H	4.79640954	-0.74361395	-0.81075692
H	4.79477614	-0.59572049	0.96588580
H	4.03951396	-1.95565342	0.18395843
H	-1.18050119	-1.99820469	1.01465866

Time = 46.50 fs
E(CASSCF/6-31G(d)) = -479.5647583

C	-0.90643162	-0.21526717	-0.13596226
C	0.59164359	0.14345996	-0.07922575
C	1.22373748	1.43474120	-0.17479572
C	1.61808704	-0.82012527	0.06553912
C	2.86035755	-0.19414949	0.04204515
C	2.60390809	1.23830942	-0.09407516
N	-1.94383120	0.59426946	0.02262288
C	-3.23698835	-0.06492193	0.12475066
H	3.38613618	1.97847390	-0.14006990
C	-1.40120548	-1.62515399	0.01276066
C	-2.91163789	-1.53405684	-0.24144506
H	-3.11669895	-1.72139738	-1.28990923
H	-3.49195123	-2.23576619	0.34273455
H	-3.62418125	-0.00566445	1.13895722
H	-3.97320004	0.37074699	-0.53120839
H	-0.88362497	-2.27855982	-0.69080168
H	0.65238385	2.33868685	-0.27914157
C	-1.92794224	2.05035413	0.01027317
H	-1.47799468	2.45416980	0.90689152
H	-1.40978887	2.41229225	-0.86543681
H	-2.94424916	2.40914082	-0.03784663
H	1.39017518	-1.86847602	0.13866464
C	4.20817233	-0.88471643	0.09477460

H	4.79624509	-0.74247564	-0.81110920
H	4.79568455	-0.59454848	0.96519087
H	4.04506750	-1.95696729	0.18425090
H	-1.17910003	-2.00266175	1.01413538

Time = 47.00 fs
E(CASSCF/6-31G(d)) = -479.5665965

C	-0.90746950	-0.21287091	-0.13784715
C	0.59380190	0.14343607	-0.07902736
C	1.22145242	1.43260813	-0.17564213
C	1.61282061	-0.82135365	0.06624630
C	2.86132575	-0.18841057	0.04222363
C	2.60676511	1.23544911	-0.09367301
N	-1.94102207	0.59373291	0.02499880
C	-3.23836982	-0.06527049	0.12462487
H	3.38399904	1.98392096	-0.14076115
C	-1.40035173	-1.62505719	0.01231266
C	-2.91224862	-1.53374020	-0.24151751
H	-3.11683570	-1.72531004	-1.29027099
H	-3.49282301	-2.23417756	0.34340829
H	-3.62560825	-0.00601997	1.13808033
H	-3.96950243	0.37586148	-0.53306202
H	-0.88974651	-2.28202102	-0.69372177
H	0.66205440	2.34848625	-0.28150593
C	-1.92957916	2.05017124	0.01040264
H	-1.47486980	2.45417594	0.90442261
H	-1.40877536	2.40511040	-0.86731236
H	-2.94592007	2.40976203	-0.03826037
H	1.40626211	-1.88040896	0.14177607
C	4.20779939	-0.88529802	0.09473576
H	4.79619487	-0.74088260	-0.81109774
H	4.79671091	-0.59297944	0.96405305
H	4.05075436	-1.95823488	0.18454875
H	-1.17757184	-2.00698093	1.01354913

Time = 47.50 fs
E(CASSCF/6-31G(d)) = -479.5679450

C	-0.90836339	-0.21070197	-0.13976953
C	0.59596866	0.14360304	-0.07884220
C	1.21880196	1.43039808	-0.17649984
C	1.60732524	-0.82276586	0.06699523
C	2.86242850	-0.18260427	0.04240088
C	2.60982832	1.23253091	-0.09326154
N	-1.93835085	0.59349297	0.02740557
C	-3.23968759	-0.06585870	0.12452818

H	3.38119088	1.98924214	-0.14148351
C	-1.39940709	-1.62501363	0.01179682
C	-2.91284771	-1.53335212	-0.24159735
H	-3.11709696	-1.72898894	-1.29048537
H	-3.49362559	-2.23264152	0.34412527
H	-3.62680806	-0.00590553	1.13732606
H	-3.96567327	0.38153517	-0.53529231
H	-0.89621040	-2.28536099	-0.69614736
H	0.67317524	2.35820301	-0.28380272
C	-1.93120000	2.04996589	0.01051271
H	-1.47143306	2.45412469	0.90190375
H	-1.40759267	2.39808138	-0.86901150
H	-2.94780323	2.41016433	-0.03863592
H	1.42324709	-1.89188758	0.14497219
C	4.20731874	-0.88591025	0.09469894
H	4.79624215	-0.73885363	-0.81070894
H	4.79783582	-0.59103136	0.96246020
H	4.05646080	-1.95943182	0.18484887
H	-1.17593388	-2.01106493	1.01279423

Time = 48.00 fs
E(CASSCF/6-31G(d)) = -479.5687481

C	-0.90906509	-0.20879459	-0.14173311
C	0.59807799	0.14394968	-0.07867434
C	1.21584464	1.42815220	-0.17736859
C	1.60170837	-0.82437798	0.06779166
C	2.86363369	-0.17683754	0.04258590
C	2.61305209	1.22962732	-0.09285012
N	-1.93587209	0.59356389	0.02985257
C	-3.24091980	-0.06667112	0.12445227
H	3.37768691	1.99426941	-0.14222224
C	-1.39838515	-1.62502608	0.01121642
C	-2.91343382	-1.53289667	-0.24168633
H	-3.11747995	-1.73238082	-1.29049638
H	-3.49435686	-2.23118583	0.34490144
H	-3.62781829	-0.00532678	1.13671410
H	-3.96173118	0.38768718	-0.53785995
H	-0.90292993	-2.28852021	-0.69808122
H	0.68567320	2.36744944	-0.28599644
C	-1.93278622	2.04973243	0.01059651
H	-1.46774015	2.45405888	0.89934029
H	-1.40631317	2.39128855	-0.87050281
H	-2.94983949	2.41032236	-0.03896193
H	1.44090178	-1.90231789	0.14819082
C	4.20673933	-0.88653465	0.09466277
H	4.79637068	-0.73641372	-0.80994447

H	4.79904053	-0.58872557	0.96041635
H	4.06207498	-1.96054005	0.18514878
H	-1.17420384	-2.01482376	1.01178017

Time = 48.50 fs
E(CASSCF/6-31G(d)) = -479.5689864

C	-0.90953037	-0.20717701	-0.14374166
C	0.60006251	0.14446118	-0.07852767
C	1.21264838	1.42590851	-0.17824744
C	1.59607905	-0.82619849	0.06864086
C	2.86491038	-0.17121969	0.04278803
C	2.61638352	1.22681068	-0.09244845
N	-1.93363505	0.59395360	0.03234850
C	-3.24204789	-0.06768704	0.12438756
H	3.37351097	1.99887540	-0.14296437
C	-1.39730167	-1.62509470	0.01057600
C	-2.91400534	-1.53237891	-0.24178451
H	-3.11798301	-1.73543754	-1.29026409
H	-3.49501138	-2.22983234	0.34574795
H	-3.62867912	-0.00429868	1.13625393
H	-3.95767347	0.39420800	-0.54069582
H	-0.90979767	-2.29146824	-0.69955917
H	0.69941133	2.37590356	-0.28806010
C	-1.93432012	2.04946672	0.01064830
H	-1.46385491	2.45401974	0.89673348
H	-1.40501006	2.38481376	-0.87176221
H	-2.95195911	2.41021650	-0.03922624
H	1.45896240	-1.91119516	0.15137456
C	4.20607055	-0.88715213	0.09462582
H	4.79656529	-0.73359312	-0.80882209
H	4.80030756	-0.58608588	0.95794242
H	4.06748966	-1.96154746	0.18544655
H	-1.17239828	-2.01817843	1.01043878

Time = 49.00 fs
E(CASSCF/6-31G(d)) = -479.5686915

C	-0.90972096	-0.20587051	-0.14579867
C	0.60185540	0.14511910	-0.07840574
C	1.20928874	1.42370058	-0.17913472
C	1.59054532	-0.82822609	0.06954730
C	2.86622945	-0.16586000	0.04301675
C	2.61976353	1.22415112	-0.09206639
N	-1.93168210	0.59466288	0.03490107
C	-3.24305657	-0.06888096	0.12432390
H	3.36873726	2.00297989	-0.14369925

C	-1.39617427	-1.62521695	0.00988065
C	-2.91456035	-1.53180483	-0.24188993
H	-3.11860619	-1.73811740	-1.28976807
H	-3.49557989	-2.22859596	0.34667036
H	-3.62943138	-0.00284534	1.13594249
H	-3.95348085	0.40096322	-0.54370628
H	-0.91668929	-2.29420244	-0.70064744
H	0.71419017	2.38332339	-0.28997678
C	-1.93578492	2.04916624	0.01066369
H	-1.45984794	2.45404624	0.89408129
H	-1.40375441	2.37873653	-0.87277593
H	-2.95408673	2.40983428	-0.03941625
H	1.47713624	-1.91813398	0.15447360
C	4.20532198	-0.88774338	0.09458664
H	4.79681222	-0.73042696	-0.80737550
H	4.80162115	-0.58313824	0.95507596
H	4.07260463	-1.96244700	0.18574083
H	-1.17053125	-2.02106401	1.00873001

Time = 49.50 fs
E(CASSCF/6-31G(d)) = -479.5679286

C	-0.90960626	-0.20488873	-0.14790694
C	0.60339252	0.14590153	-0.07831174
C	1.20584686	1.42155666	-0.18002808
C	1.58521155	-0.83044817	0.07051441
C	2.86756396	-0.16086528	0.04328147
C	2.62312852	1.22171444	-0.09171358
N	-1.93004735	0.59568483	0.03751662
C	-3.24393372	-0.07022332	0.12425139
H	3.36348825	2.00655314	-0.14441905
C	-1.39502189	-1.62538777	0.00913525
C	-2.91509661	-1.53118127	-0.24199854
H	-3.11935152	-1.74038625	-1.28900936
H	-3.49604926	-2.22748425	0.34766837
H	-3.63011484	-0.00099972	1.13576287
H	-3.94912428	0.40779924	-0.54678080
H	-0.92346871	-2.29674448	-0.70143766
H	0.72975297	2.38955584	-0.29174054
C	-1.93716497	2.04883009	0.01063979
H	-1.45579461	2.45417459	0.89137961
H	-1.40261249	2.37313314	-0.87354274
H	-2.95614677	2.40917165	-0.03951932
H	1.49511090	-1.92288927	0.15744814
C	4.20450322	-0.88828931	0.09454377
H	4.79709905	-0.72695491	-0.80565316
H	4.80296731	-0.57991078	0.95186997

H	4.07732896	-1.96323533	0.18603068
H	-1.16861312	-2.02343194	1.00664550

Time = 50.00 fs
E(CASSCF/6-31G(d)) = -479.5667817

C	-0.90916467	-0.20423706	-0.15006805
C	0.60461421	0.14678315	-0.07824838
C	1.20240723	1.41949908	-0.18092454
C	1.58017626	-0.83283966	0.07154435
C	2.86888918	-0.15633744	0.04359132
C	2.62641206	1.21956021	-0.09139923
N	-1.92875557	0.59700451	0.04019976
C	-3.24466996	-0.07168131	0.12416122
H	3.35792977	2.00961470	-0.14511874
C	-1.39386420	-1.62559996	0.00834374
C	-2.91561148	-1.53051572	-0.24210419
H	-3.12022312	-1.74221829	-1.28801057
H	-3.49640284	-2.22649757	0.34873601
H	-3.63076674	0.00119682	1.13568424
H	-3.94457303	0.41455037	-0.54980123
H	-0.92999428	-2.29913493	-0.70203963
H	0.74579371	2.39453999	-0.29335672
C	-1.93844581	2.04845901	0.01057530
H	-1.45177274	2.45443808	0.88862401
H	-1.40164358	2.36807534	-0.87407534
H	-2.95806880	2.40823412	-0.03952325
H	1.51256442	-1.92536887	0.16027009
C	4.20362383	-0.88877146	0.09449579
H	4.79741418	-0.72322048	-0.80371626
H	4.80433333	-0.57643340	0.94839075
H	4.08158311	-1.96391147	0.18631552
H	-1.16664982	-2.02525115	1.00420967

Time = 50.50 fs
E(CASSCF/6-31G(d)) = -479.5653451

C	-0.90838492	-0.20391255	-0.15228219
C	0.60546766	0.14773580	-0.07821785
C	1.19905454	1.41754390	-0.18182057
C	1.57552907	-0.83536298	0.07263783
C	2.87018279	-0.15237001	0.04395490
C	2.62954751	1.21773946	-0.09113182
N	-1.92782133	0.59859882	0.04295315
C	-3.24525818	-0.07321989	0.12404645
H	3.35226110	2.01223092	-0.14579647
C	-1.39272087	-1.62584473	0.00750846

C	-2.91610191	-1.52981620	-0.24219894
H	-3.12122687	-1.74359631	-1.28681427
H	-3.49662151	-2.22562995	0.34986241
H	-3.63142087	0.00369534	1.13566298
H	-3.93980267	0.42104708	-0.55265175
H	-0.93612572	-2.30142608	-0.70257245
H	0.76196989	2.39830561	-0.29484146
C	-1.93961427	2.04805540	0.01047040
H	-1.44786011	2.45486680	0.88581148
H	-1.40089860	2.36362889	-0.87440046
H	-2.95979215	2.40703724	-0.03941664
H	1.52917814	-1.92563961	0.16292439
C	4.20269338	-0.88917250	0.09444135
H	4.79774634	-0.71927139	-0.80163594
H	4.80570738	-0.57273881	0.94471526
H	4.08530028	-1.96447515	0.18659499
H	-1.16464261	-2.02650850	1.00147870

Time = 51.00 fs
E(CASSCF/6-31G(d)) = -479.5637135

C	-0.90726659	-0.20390386	-0.15454780
C	0.60590889	0.14872913	-0.07822180
C	1.19587057	1.41570109	-0.18271223
C	1.57134846	-0.83796844	0.07379403
C	2.87142467	-0.14904506	0.04437996
C	2.63247056	1.21629299	-0.09091875
N	-1.92724867	0.60043680	0.04577728
C	-3.24569298	-0.07480280	0.12390260
H	3.34670263	2.01450769	-0.14645347
C	-1.39161085	-1.62611251	0.00662961
C	-2.91656439	-1.52909100	-0.24227351
H	-3.12236982	-1.74451169	-1.28547967
H	-3.49668481	-2.22487046	0.35103296
H	-3.63210682	0.00644031	1.13564494
H	-3.93480238	0.42712450	-0.55522844
H	-0.94173132	-2.30367370	-0.70315444
H	0.77791673	2.40096525	-0.29622064
C	-1.94065858	2.04762319	0.01032657
H	-1.44413178	2.45548753	0.88294232
H	-1.40041919	2.35985176	-0.87455822
H	-2.96126976	2.40560676	-0.03918921
H	1.54464804	-1.92392571	0.16540970
C	4.20172161	-0.88947666	0.09437925
H	4.79808388	-0.71515949	-0.79948982
H	4.80707781	-0.56886292	0.94092766
H	4.08842781	-1.96492559	0.18686883

H	-1.16258822	-2.02720775	0.99853679

Time = 51.50 fs
E(CASSCF/6-31G(d)) = -479.5619800

C	-0.90581975	-0.20419187	-0.15686109
C	0.60590240	0.14973034	-0.07826134
C	1.19293363	1.41397546	-0.18359547
C	1.56770208	-0.84059424	0.07501049
C	2.87259634	-0.14643267	0.04487331
C	2.63512036	1.21525111	-0.09076625
N	-1.92703138	0.60248022	0.04867027
C	-3.24597028	-0.07639362	0.12372804
H	3.34148550	2.01657361	-0.14709254
C	-1.39055157	-1.62639381	0.00570465
C	-2.91699494	-1.52834858	-0.24231776
H	-3.12365956	-1.74496372	-1.28407800
H	-3.49657233	-2.22420460	0.35223039
H	-3.63285023	0.00937048	1.13556959
H	-3.92958121	0.43262937	-0.55744740
H	-0.94669469	-2.30592803	-0.70389240
H	0.79325733	2.40269846	-0.29752801
C	-1.94156848	2.04716808	0.01014638
H	-1.44065742	2.45632427	0.88002184
H	-1.40023720	2.35679288	-0.87460012
H	-2.96247121	2.40397860	-0.03883225
H	1.55869156	-1.92059668	0.16773801
C	4.20071817	-0.88966974	0.09430841
H	4.79841282	-0.71093836	-0.79735860
H	4.80843116	-0.56484330	0.93711556
H	4.09092809	-1.96526007	0.18713676
H	-1.16047968	-2.02736796	0.99549050

Time = 52.00 fs
E(CASSCF/6-31G(d)) = -479.5602323

C	-0.90406483	-0.20475028	-0.15921599
C	0.60542429	0.15070619	-0.07833719
C	1.19031382	1.41236702	-0.18446616
C	1.56464352	-0.84316921	0.07628321
C	2.87368068	-0.14458616	0.04544042
C	2.63744311	1.21463163	-0.09067903
N	-1.92715322	0.60468440	0.05162781
C	-3.24608689	-0.07795676	0.12352425
H	3.33683133	2.01857014	-0.14771828
C	-1.38955842	-1.62668012	0.00472828
C	-2.91738905	-1.52759735	-0.24232142

H	-3.12510320	-1.74495893	-1.28268637
H	-3.49626541	-2.22361633	0.35343660
H	-3.63367241	0.01242064	1.13537315
H	-3.92417170	0.43742777	-0.55925138
H	-0.95092052	-2.30822626	-0.70487216
H	0.80762407	2.40373377	-0.29880270
C	-1.94233535	2.04669742	0.00993315
H	-1.43749899	2.45739751	0.87706154
H	-1.40037552	2.35449022	-0.87458633
H	-2.96338370	2.40219741	-0.03833851
H	1.57106037	-1.91615020	0.16993390
C	4.19969312	-0.88973967	0.09422790
H	4.79871698	-0.70666505	-0.79532216
H	4.80975237	-0.56072141	0.93336654
H	4.09277864	-1.96547388	0.18739847
H	-1.15830721	-2.02702071	0.99246091

Time = 52.50 fs
E(CASSCF/6-31G(d)) = -479.5585857

C	-0.90203200	-0.20554671	-0.16160425
C	0.60446067	0.15162289	-0.07844946
C	1.18807235	1.41087381	-0.18532059
C	1.56221312	-0.84561603	0.07760691
C	2.87466219	-0.14354247	0.04608543
C	2.63939360	1.21444130	-0.09066037
N	-1.92758896	0.60699948	0.05464329
C	-3.24603993	-0.07945830	0.12329576
H	3.33293960	2.02063093	-0.14833529
C	-1.38864385	-1.62696461	0.00369245
C	-2.91774170	-1.52684548	-0.24227485
H	-3.12670596	-1.74450987	-1.28138196
H	-3.49574884	-2.22309029	0.35463421
H	-3.63459166	0.01552306	1.13499550
H	-3.91863262	0.44141098	-0.56061572
H	-0.95433959	-2.31058477	-0.70615076
H	0.82066882	2.40432310	-0.30008629
C	-1.94295228	2.04621986	0.00969065
H	-1.43470818	2.45872470	0.87408013
H	-1.40084831	2.35296886	-0.87458238
H	-2.96401277	2.40031588	-0.03770272
H	1.58154407	-1.91117907	0.17203231
C	4.19865701	-0.88967664	0.09413702
H	4.79897558	-0.70239800	-0.79345457
H	4.81102218	-0.55654158	0.92976288
H	4.09397277	-1.96555797	0.18765343
H	-1.15606014	-2.02620641	0.98957456

Time = 53.00 fs
E(CASSCF/6-31G(d)) = -479.5571170

C	-0.89975952	-0.20654385	-0.16401517
C	0.60300926	0.15244757	-0.07859796
C	1.18625918	1.40949255	-0.18615557
C	1.56043716	-0.84785465	0.07897519
C	2.87552619	-0.14332017	0.04681093
C	2.64093681	1.21467553	-0.09071193
N	-1.92830547	0.60937165	0.05770778
C	-3.24582695	-0.08086685	0.12304995
H	3.32997039	2.02286533	-0.14894738
C	-1.38781718	-1.62724290	0.00258676
C	-2.91804743	-1.52610084	-0.24216970
H	-3.12847050	-1.74363406	-1.28023501
H	-3.49501230	-2.22261336	0.35580806
H	-3.63562366	0.01860926	1.13438323
H	-3.91304873	0.44449930	-0.56154961
H	-0.95691066	-2.31299564	-0.70775128
H	0.83207973	2.40471278	-0.30141924
C	-1.94341421	2.04574582	0.00942273
H	-1.43232519	2.46031986	0.87110335
H	-1.40166278	2.35224020	-0.87465503
H	-2.96438006	2.39839231	-0.03692130
H	1.58997425	-1.90632553	0.17407479
C	4.19762087	-0.88947317	0.09403522
H	4.79916397	-0.69819689	-0.79182247
H	4.81221795	-0.55235095	0.92638002
H	4.09451869	-1.96550129	0.18790100
H	-1.15372844	-2.02497078	0.98695321

Time = 53.50 fs
E(CASSCF/6-31G(d)) = -479.5559508

C	-0.89729233	-0.20770114	-0.16643633
C	0.60107860	0.15314958	-0.07878211
C	1.18491148	1.40822100	-0.18696879
C	1.55932858	-0.84980856	0.08038111
C	2.87625880	-0.14391904	0.04761791
C	2.64204965	1.21531953	-0.09083385
N	-1.92926329	0.61174517	0.06081053
C	-3.24544554	-0.08215427	0.12279656
H	3.32803263	2.02534446	-0.14955658
C	-1.38708422	-1.62751331	0.00139929
C	-2.91830038	-1.52537071	-0.24199964
H	-3.13039551	-1.74235210	-1.27930174

H	-3.49405119	-2.22217557	0.35694625
H	-3.63678170	0.02161213	1.13349402
H	-3.90752753	0.44664434	-0.56209609
H	-0.95862223	-2.31542487	-0.70966037
H	0.84159276	2.40511687	-0.30283746
C	-1.94371808	2.04528680	0.00913298
H	-1.43037765	2.46219289	0.86816324
H	-1.40282111	2.35229999	-0.87486769
H	-2.96452080	2.39648771	-0.03599218
H	1.59622515	-1.90222472	0.17610490
C	4.19659630	-0.88912433	0.09392219
H	4.79925325	-0.69412148	-0.79048214
H	4.81331340	-0.54819915	0.92328336
H	4.09443904	-1.96529063	0.18814048
H	-1.15130546	-2.02336022	0.98470302

Time = 54.00 fs
E(CASSCF/6-31G(d)) = -479.5551751

C	-0.89468073	-0.20897608	-0.16885355
C	0.59868775	0.15370131	-0.07900110
C	1.18405315	1.40705958	-0.18775901
C	1.55888791	-0.85141045	0.08181777
C	2.87684693	-0.14532054	0.04850573
C	2.64272126	1.21634959	-0.09102478
N	-1.93041791	0.61406366	0.06393906
C	-3.24489357	-0.08329619	0.12254719
H	3.32717478	2.02808788	-0.15016217
C	-1.38644723	-1.62777700	0.00011753
C	-2.91849436	-1.52466183	-0.24176082
H	-3.13247487	-1.74068697	-1.27861986
H	-3.49286754	-2.22177174	0.35804103
H	-3.63807810	0.02446760	1.13230087
H	-3.90219630	0.44782946	-0.56233015
H	-0.95949025	-2.31781447	-0.71183080
H	0.84900135	2.40569027	-0.30436917
C	-1.94386297	2.04485560	0.00882452
H	-1.42887979	2.46434983	0.86529743
H	-1.40432152	2.35312837	-0.87527708
H	-2.96448016	2.39466396	-0.03491480
H	1.60021046	-1.89943669	0.17816189
C	4.19559540	-0.88862765	0.09379785
H	4.79921041	-0.69023056	-0.78947750
H	4.81427859	-0.54413745	0.92052627
H	4.09376988	-1.96491165	0.18837111
H	-1.14878954	-2.02141833	0.98290516

Time = 54.50 fs
E(CASSCF/6-31G(d)) = -479.5548762

C	-0.89197870	-0.21032589	-0.17125153
C	0.59586580	0.15407926	-0.07925386
C	1.18369443	1.40601251	-0.18852631
C	1.55910432	-0.85260847	0.08327891
C	2.87727824	-0.14748868	0.04947220
C	2.64295297	1.21773434	-0.09128197
N	-1.93172145	0.61627209	0.06707982
C	-3.24416931	-0.08427231	0.12231452
H	3.32738109	2.03105866	-0.15076030
C	-1.38590503	-1.62803775	-0.00127049
C	-2.91862301	-1.52398022	-0.24145219
H	-3.13469739	-1.73866278	-1.27820456
H	-3.49147017	-2.22140172	0.35908942
H	-3.63952486	0.02711673	1.13079438
H	-3.89719525	0.44806824	-0.56235392
H	-0.95955524	-2.32008745	-0.71418643
H	0.85416208	2.40651140	-0.30603286
C	-1.94385012	2.04446598	0.00849972
H	-1.42783268	2.46679238	0.86254675
H	-1.40616027	2.35468936	-0.87592946
H	-2.96430880	2.39298041	-0.03368965
H	1.60187880	-1.89837970	0.18027474
C	4.19463058	-0.88798318	0.09366233
H	4.79899925	-0.68658052	-0.78883959
H	4.81508108	-0.54021749	0.91814913
H	4.09255983	-1.96435043	0.18859229
H	-1.14618594	-2.01918218	0.98160766

Time = 55.00 fs
E(CASSCF/6-31G(d)) = -479.5550614

C	-0.88924213	-0.21170841	-0.17361375
C	0.59265069	0.15426465	-0.07953924
C	1.18383155	1.40508859	-0.18927203
C	1.55995620	-0.85337141	0.08475938
C	2.87754183	-0.15037133	0.05051362
C	2.64275812	1.21943598	-0.09160146
N	-1.93312393	0.61831769	0.07021809
C	-3.24327169	-0.08506648	0.12211164
H	3.32857269	2.03416244	-0.15134384
C	-1.38545334	-1.62830139	-0.00277530
C	-2.91867993	-1.52333123	-0.24107555
H	-3.13704620	-1.73630560	-1.27804867
H	-3.48987525	-2.22107195	0.36009330

H	-3.64113459	0.02950622	1.12898599
H	-3.89267312	0.44740065	-0.56229060
H	-0.95887584	-2.32215575	-0.71663145
H	0.85699996	2.40756837	-0.30783459
C	-1.94368319	2.04413275	0.00816031
H	-1.42722414	2.46951847	0.85995533
H	-1.40833093	2.35693280	-0.87685964
H	-2.96405847	2.39149380	-0.03231897
H	1.60121056	-1.89927343	0.18245611
C	4.19371445	-0.88719333	0.09351597
H	4.79858074	-0.68322424	-0.78858503
H	4.81568560	-0.53649051	0.91617811
H	4.09086701	-1.96359341	0.18880337
H	-1.14350747	-2.01668174	0.98082223

Time = 55.50 fs
E(CASSCF/6-31G(d)) = -479.5557688

C	-0.88652896	-0.21308388	-0.17592358
C	0.58908969	0.15424428	-0.07985575
C	1.18444810	1.40430078	-0.18999878
C	1.56141275	-0.85369154	0.08625555
C	2.87762758	-0.15390228	0.05162491
C	2.64216005	1.22141137	-0.09197818
N	-1.93457470	0.62015191	0.07333851
C	-3.24220085	-0.08566659	0.12195124
H	3.33061795	2.03726009	-0.15190311
C	-1.38508504	-1.62857498	-0.00440470
C	-2.91865872	-1.52271952	-0.24063567
H	-3.13949890	-1.73364121	-1.27812103
H	-3.48810483	-2.22079241	0.36105846
H	-3.64291811	0.03159158	1.12690504
H	-3.88877316	0.44588832	-0.56227334
H	-0.95752538	-2.32392646	-0.71905737
H	0.85750226	2.40876767	-0.30976921
C	-1.94336812	2.04387115	0.00780732
H	-1.42703163	2.47251835	0.85756459
H	-1.41082743	2.35979170	-0.87808560
H	-2.96377573	2.39025112	-0.03080575
H	1.59821315	-1.90211180	0.18469996
C	4.19285957	-0.88626297	0.09335934
H	4.79791645	-0.68020985	-0.78871836
H	4.81605797	-0.53300559	0.91462699
H	4.08875897	-1.96263174	0.18900409
H	-1.14077621	-2.01393702	0.98052031

Time = 56.00 fs

E(CASSCF/6-31G(d)) = -479.5568986

C	-0.88389680	-0.21441616	-0.17816487
C	0.58523803	0.15401118	-0.08020163
C	1.18551585	1.40366449	-0.19071030
C	1.56343462	-0.85358466	0.08776558
C	2.87752672	-0.15800399	0.05279984
C	2.64119045	1.22361317	-0.09240614
N	-1.93602403	0.62173202	0.07642568
C	-3.24095880	-0.08606449	0.12184469
H	3.33334465	2.04018042	-0.15242682
C	-1.38479087	-1.62886612	-0.00616305
C	-2.91855321	-1.52214903	-0.24013980
H	-3.14202894	-1.73069557	-1.27836906
H	-3.48618538	-2.22057573	0.36199375
H	-3.64488568	0.03333802	1.12459747
H	-3.88562394	0.44360671	-0.56243525
H	-0.95558608	-2.32531175	-0.72135312
H	0.85571533	2.40994724	-0.31182194
C	-1.94291319	2.04369653	0.00744110
H	-1.42722349	2.47577583	0.85541041
H	-1.41364476	2.36318545	-0.87960866
H	-2.96349835	2.38928796	-0.02915313
H	1.59292210	-1.90666321	0.18698143
C	4.19207791	-0.88519913	0.09319320
H	4.79697033	-0.67757881	-0.78923359
H	4.81616699	-0.52980737	0.91349817
H	4.08631155	-1.96146236	0.18919483
H	-1.13802328	-2.01095866	0.98063518

Time = 56.50 fs
E(CASSCF/6-31G(d)) = -479.5582916

C	-0.88140239	-0.21567314	-0.18032195
C	0.58115824	0.15356444	-0.08057494
C	1.18699547	1.40319657	-0.19141124
C	1.56597326	-0.85308821	0.08928911
C	2.87723265	-0.16258923	0.05403116
C	2.63988777	1.22599035	-0.09287852
N	-1.93742374	0.62302137	0.07946422
C	-3.23954992	-0.08625549	0.12180133
H	3.33655173	2.04273425	-0.15290295
C	-1.38455999	-1.62918188	-0.00805110
C	-2.91835756	-1.52162300	-0.23959711
H	-3.14460575	-1.72749670	-1.27872510
H	-3.48414751	-2.22043764	0.36291044
H	-3.64704641	0.03472028	1.12212519

H	-3.88333205	0.44063864	-0.56289941
H	-0.95314166	-2.32623783	-0.72341700
H	0.85174284	2.41088898	-0.31396877
C	-1.94232900	2.04362420	0.00706176
H	-1.42775975	2.47926850	0.85352321
H	-1.41677612	2.36702235	-0.88141605
H	-2.96325255	2.38862971	-0.02736549
H	1.58540730	-1.91249387	0.18925866
C	4.19138060	-0.88401105	0.09301850
H	4.79570985	-0.67536496	-0.79011478
H	4.81598508	-0.52693516	0.91278348
H	4.08360516	-1.96008780	0.18937632
H	-1.13528673	-2.00775100	0.98106869

Time = 57.00 fs
E(CASSCF/6-31G(d)) = -479.5598040

C	-0.87910117	-0.21682754	-0.18238034
C	0.57691988	0.15290935	-0.08097335
C	1.18883828	1.40291305	-0.19210688
C	1.56897128	-0.85225641	0.09082695
C	2.87674099	-0.16756373	0.05531079
C	2.63829474	1.22848880	-0.09338783
N	-1.93872818	0.62399049	0.08243911
C	-3.23798166	-0.08623807	0.12182784
H	3.34002652	2.04473372	-0.15332007
C	-1.38438060	-1.62952787	-0.01006572
C	-2.91806643	-1.52114404	-0.23901810
H	-3.14719624	-1.72407483	-1.27911146
H	-3.48202390	-2.22039422	0.36382061
H	-3.64940659	0.03572361	1.11956110
H	-3.88197082	0.43706672	-0.56376705
H	-0.95027296	-2.32665158	-0.72516434
H	0.84573646	2.41134660	-0.31617975
C	-1.94162828	2.04366903	0.00666942
H	-1.42859464	2.48296659	0.85192431
H	-1.42021295	2.37120130	-0.88348120
H	-2.96305067	2.38828895	-0.02544801
H	1.57577751	-1.91902415	0.19147824
C	4.19077752	-0.88270992	0.09283638
H	4.79410876	-0.67359336	-0.79133877
H	4.81549104	-0.52442114	0.91246648
H	4.08072366	-1.95851799	0.18954989
H	-1.13261012	-2.00431424	0.98169861

Time = 57.50 fs
E(CASSCF/6-31G(d)) = -479.5612860

C	-0.87704648	-0.21785749	-0.18432721
C	0.57259836	0.15205720	-0.08139417
C	1.19098827	1.40282693	-0.19280279
C	1.57236301	-0.85115426	0.09238068
C	2.87604988	-0.17282914	0.05663007
C	2.63645585	1.23105238	-0.09392613
N	-1.93989499	0.62461768	0.08533603
C	-3.23626510	-0.08601338	0.12192777
H	3.34356086	2.04600938	-0.15366846
C	-1.38424063	-1.62990747	-0.01220023
C	-2.91767509	-1.52071413	-0.23841383
H	-3.14976651	-1.72046283	-1.27944699
H	-3.47984722	-2.22045977	0.36473551
H	-3.65196851	0.03634407	1.11698395
H	-3.88157194	0.43296685	-0.56510810
H	-0.94705365	-2.32652586	-0.72653417
H	0.83788655	2.41107254	-0.31842240
C	-1.94082564	2.04384486	0.00626451
H	-1.42967844	2.48683308	0.85062371
H	-1.42394417	2.37561461	-0.88576648
H	-2.96289100	2.38826475	-0.02340644
H	1.56418419	-1.92559663	0.19358206
C	4.19027684	-0.88130874	0.09264811
H	4.79214913	-0.67227915	-0.79287759
H	4.81467224	-0.52228873	0.91252453
H	4.07775288	-1.95677082	0.18971756
H	-1.13004004	-2.00064740	0.98238878

Time = 58.00 fs
E(CASSCF/6-31G(d)) = -479.5626152

C	-0.87528847	-0.21874675	-0.18615187
C	0.56827310	0.15102468	-0.08183428
C	1.19338443	1.40294625	-0.19350460
C	1.57607555	-0.84985109	0.09395214
C	2.87516005	-0.17828612	0.05798009
C	2.63441507	1.23362414	-0.09448518
N	-1.94088579	0.62488940	0.08814178
C	-3.23441531	-0.08558497	0.12210142
H	3.34696610	2.04642406	-0.15394098
C	-1.38412839	-1.63032123	-0.01444497
C	-2.91717947	-1.52033485	-0.23779511
H	-3.15228391	-1.71669659	-1.27965385
H	-3.47764799	-2.22064461	0.36566369
H	-3.65472912	0.03658857	1.11447211
H	-3.88212007	0.42840316	-0.56695451

H	-0.94354691	-2.32586267	-0.72749313
H	0.82841208	2.40984323	-0.32066503
C	-1.93993719	2.04416402	0.00584814
H	-1.43095911	2.49082392	0.84961834
H	-1.42795532	2.38015123	-0.88822650
H	-2.96275912	2.38854260	-0.02124668
H	1.55082200	-1.93154535	0.19551398
C	4.18988461	-0.87982198	0.09245507
H	4.78982291	-0.67142679	-0.79470086
H	4.81352626	-0.52055125	0.91293070
H	4.07477920	-1.95487175	0.18988206
H	-1.12762342	-1.99675153	0.98300029

Time = 58.50 fs
E(CASSCF/6-31G(d)) = -479.5637233

C	-0.87387517	-0.21948461	-0.18784594
C	0.56402791	0.14983350	-0.08229008
C	1.19596213	1.40327304	-0.19421761
C	1.58002886	-0.84841594	0.09554306
C	2.87407512	-0.18383536	0.05935173
C	2.63221409	1.23614619	-0.09505666
N	-1.94166622	0.62480003	0.09084394
C	-3.23245143	-0.08495868	0.12234601
H	3.35008345	2.04588505	-0.15413398
C	-1.38403298	-1.63076636	-0.01678776
C	-2.91657635	-1.52000728	-0.23717194
H	-3.15471747	-1.71281596	-1.27966384
H	-3.47545370	-2.22095375	0.36661049
H	-3.65767702	0.03647413	1.11209899
H	-3.88355014	0.42342748	-0.56929600
H	-0.93980303	-2.32469417	-0.72803928
H	0.81755273	2.40747830	-0.32287859
C	-1.93898021	2.04463697	0.00542248
H	-1.43238379	2.49488795	0.84889259
H	-1.43222604	2.38469875	-0.89081105
H	-2.96262951	2.38909573	-0.01897562
H	1.53592832	-1.93625197	0.19722525
C	4.18960461	-0.87826552	0.09225881
H	4.78713360	-0.67103021	-0.79677725
H	4.81206213	-0.51921185	0.91365560
H	4.07188712	-1.95285415	0.19004671
H	-1.12540422	-1.99263329	0.98340193

Time = 59.00 fs
E(CASSCF/6-31G(d)) = -479.5645666

C	-0.87285123	-0.22006566	-0.18940348
C	0.55994864	0.14850953	-0.08275756
C	1.19865551	1.40380222	-0.19494665
C	1.58413778	-0.84691277	0.09715477
C	2.87280148	-0.18938005	0.06073581
C	2.62989059	1.23856075	-0.09563222
N	-1.94220621	0.62435176	0.09343121
C	-3.23039652	-0.08414265	0.12265613
H	3.35279388	2.04435065	-0.15424763
C	-1.38394489	-1.63123659	-0.01921481
C	-2.91586337	-1.51973213	-0.23655288
H	-3.15703956	-1.70886467	-1.27942354
H	-3.47328690	-2.22138520	0.36757670
H	-3.66079122	0.03602718	1.10992709
H	-3.88574901	0.41807957	-0.57208062
H	-0.93585845	-2.32308158	-0.72820157
H	0.80555880	2.40385714	-0.32503922
C	-1.93797272	2.04527195	0.00499095
H	-1.43389952	2.49896810	0.84841850
H	-1.43672931	2.38914673	-0.89346902
H	-2.96246943	2.38988645	-0.01660074
H	1.51977640	-1.93919334	0.19867901
C	4.18943796	-0.87665623	0.09206095
H	4.78409668	-0.67107265	-0.79907597
H	4.81030089	-0.51826294	0.91466855
H	4.06915819	-1.95075794	0.19021539
H	-1.12342070	-1.98830724	0.98347981

Time = 59.50 fs
E(CASSCF/6-31G(d)) = -479.5651391

C	-0.87225670	-0.22048965	-0.19082124
C	0.55612147	0.14708204	-0.08323226
C	1.20139921	1.40452120	-0.19569592
C	1.58831352	-0.84539738	0.09878791
C	2.87134826	-0.19482752	0.06212340
C	2.62747716	1.24081108	-0.09620372
N	-1.94248045	0.62355446	0.09589358
C	-3.22827728	-0.08314745	0.12302444
H	3.35502295	2.04183362	-0.15428609
C	-1.38385609	-1.63172233	-0.02171159
C	-2.91503918	-1.51950993	-0.23594459
H	-3.15922759	-1.70489054	-1.27889899
H	-3.47116472	-2.22192974	0.36855814
H	-3.66404069	0.03528292	1.10800412
H	-3.88856209	0.41239076	-0.57521981
H	-0.93173597	-2.32111209	-0.72803777

H	0.79268343	2.39892954	-0.32713017
C	-1.93693283	2.04607454	0.00455839
H	-1.43545393	2.50300354	0.84815689
H	-1.44143076	2.39339049	-0.89615217
H	-2.96224379	2.39086837	-0.01413014
H	1.50266745	-1.93997625	0.19985325
C	4.18938317	-0.87501181	0.09186324
H	4.78073976	-0.67152723	-0.80156818
H	4.80827571	-0.51768655	0.91593879
H	4.06666919	-1.94862768	0.19039237
H	-1.12170301	-1.98379833	0.98314614

Time = 60.00 fs
E(CASSCF/6-31G(d)) = -479.5654564

C	-0.87212640	-0.22076076	-0.19209841
C	0.55263029	0.14558256	-0.08370938
C	1.20413107	1.40541017	-0.19646892
C	1.59246664	-0.84391485	0.10044246
C	2.86972668	-0.20009115	0.06350578
C	2.62499986	1.24284237	-0.09676334
N	-1.94246837	0.62242496	0.09822227
C	-3.22612315	-0.08198637	0.12344263
H	3.35674498	2.03839907	-0.15425709
C	-1.38376037	-1.63221105	-0.02426368
C	-2.91410355	-1.51934102	-0.23535163
H	-3.16126416	-1.70094513	-1.27807809
H	-3.46909800	-2.22257069	0.36954552
H	-3.66738318	0.03428387	1.10636023
H	-3.89180164	0.40638930	-0.57859626
H	-0.92744619	-2.31889336	-0.72763016
H	0.77917338	2.39271882	-0.32914288
C	-1.93587843	2.04704739	0.00413108
H	-1.43699466	2.50693152	0.84806050
H	-1.44628704	2.39733345	-0.89881920
H	-2.96191960	2.39198917	-0.01157265
H	1.48491807	-1.93835646	0.20074263
C	4.18943608	-0.87335041	0.09166749
H	4.77710181	-0.67235702	-0.80422695
H	4.80603085	-0.51745418	0.91743556
H	4.06449149	-1.94651051	0.19058206
H	-1.12027084	-1.97914248	0.98234452

Time = 60.50 fs
E(CASSCF/6-31G(d)) = -479.5655495

C	-0.87248863	-0.22088719	-0.19323694

C	0.54955439	0.14404393	-0.08418388
C	1.20679295	1.40644277	-0.19726832
C	1.59650863	-0.84249857	0.10211768
C	2.86795035	-0.20509090	0.06487464
C	2.62247867	1.24460235	-0.09730370
N	-1.94215450	0.62098674	0.10040997
C	-3.22396526	-0.08067560	0.12390228
H	3.35798039	2.03416135	-0.15417197
C	-1.38365315	-1.63268790	-0.02685762
C	-2.91305735	-1.51922556	-0.23477637
H	-3.16313794	-1.69708277	-1.27697162
H	-3.46709189	-2.22328486	0.37052535
H	-3.67076564	0.03307912	1.10500675
H	-3.89525829	0.40010649	-0.58207419
H	-0.92299047	-2.31654659	-0.72707901
H	0.76526320	2.38532378	-0.33107727
C	-1.93482649	2.04818987	0.00371659
H	-1.43846964	2.51068981	0.84807635
H	-1.45124704	2.40088971	-0.90143743
H	-2.96147059	2.39319282	-0.00893748
H	1.46685012	-1.93425246	0.20135895
C	4.18958995	-0.87169038	0.09147563
H	4.77323201	-0.67351664	-0.80702743
H	4.80362071	-0.51752858	0.91912820
H	4.06268939	-1.94445416	0.19078890
H	-1.11913248	-1.97438711	0.98105413

Time = 61.00 fs
E(CASSCF/6-31G(d)) = -479.5654281

C	-0.87336327	-0.22088037	-0.19424114
C	0.54696570	0.14249913	-0.08465078
C	1.20933124	1.40758716	-0.19809606
C	1.60035347	-0.84116986	0.10381201
C	2.86603567	-0.20975437	0.06622224
C	2.61992836	1.24604240	-0.09781793
N	-1.94152891	0.61926924	0.10245092
C	-3.22183511	-0.07923440	0.12439579
H	3.35879116	2.02927505	-0.15404489
C	-1.38353137	-1.63313655	-0.02948156
C	-2.91190259	-1.51916350	-0.23421910
H	-3.16484342	-1.69335976	-1.27561211
H	-3.46514600	-2.22404389	0.37148083
H	-3.67412544	0.03172218	1.10393633
H	-3.89871401	0.39358199	-0.58551084
H	-0.91836532	-2.31419781	-0.72649448
H	0.75117014	2.37691258	-0.33294176

C	-1.93379269	2.04949761	0.00332360
H	-1.43982657	2.51421994	0.84814945
H	-1.45625211	2.40398698	-0.90398550
H	-2.96088099	2.39442256	-0.00623433
H	1.44877861	-1.92774633	0.20173098
C	4.18983575	-0.87004992	0.09128963
H	4.76918799	-0.67495363	-0.80994570
H	4.80110758	-0.51786495	0.92098535
H	4.06131897	-1.94250389	0.19101708
H	-1.11828454	-1.96959042	0.97929009

Time = 61.50 fs
E(CASSCF/6-31G(d)) = -479.5651321

C	-0.87476133	-0.22075453	-0.19511786
C	0.54492698	0.14098054	-0.08510525
C	1.21169838	1.40880730	-0.19895333
C	1.60392051	-0.83993758	0.10552331
C	2.86400096	-0.21401759	0.06754141
C	2.61735867	1.24711849	-0.09829984
N	-1.94058734	0.61730725	0.10434109
C	-3.21976334	-0.07768510	0.12491707
H	3.35927316	2.02392465	-0.15389216
C	-1.38339310	-1.63354024	-0.03212593
C	-2.91064249	-1.51915455	-0.23367838
H	-3.16638052	-1.68983280	-1.27405109
H	-3.46325531	-2.22481586	0.37239319
H	-3.67739181	0.03027026	1.10312512
H	-3.90195436	0.38686926	-0.58876793
H	-0.91356729	-2.31196816	-0.72598709
H	0.73708970	2.36771396	-0.33475238
C	-1.93279094	2.05096256	0.00296150
H	-1.44101276	2.51746916	0.84822580
H	-1.46123785	2.40656743	-0.90645355
H	-2.96014817	2.39562271	-0.00347299
H	1.43099908	-1.91908246	0.20190363
C	4.19016237	-0.86844671	0.09111152
H	4.76503401	-0.67660986	-0.81295819
H	4.79855992	-0.51841260	0.92297430
H	4.06042755	-1.94070106	0.19127038
H	-1.11771205	-1.96481974	0.97710174

Time = 62.00 fs
E(CASSCF/6-31G(d)) = -479.5646483

C	-0.87668261	-0.22052588	-0.19587598
C	0.54348932	0.13951890	-0.08554300

C	1.21385170	1.41006453	-0.19984069
C	1.60713495	-0.83879911	0.10724876
C	2.86186747	-0.21782508	0.06882576
C	2.61477683	1.24779234	-0.09874393
N	-1.93933172	0.61514021	0.10607837
C	-3.21777836	-0.07605296	0.12546199
H	3.35954367	2.01830877	-0.15373119
C	-1.38323709	-1.63388282	-0.03478383
C	-2.90928150	-1.51919814	-0.23315142
H	-3.16775394	-1.68655809	-1.27235553
H	-3.46141167	-2.22556863	0.37324365
H	-3.68048963	0.02878218	1.10253729
H	-3.90478225	0.38003848	-0.59172304
H	-0.90859807	-2.30996552	-0.72565924
H	0.72319621	2.35800268	-0.33653132
C	-1.93183308	2.05257276	0.00263997
H	-1.44197413	2.52039450	0.84825667
H	-1.46613557	2.40859024	-0.90884422
H	-2.95928497	2.39674224	-0.00066334
H	1.41377850	-1.90865581	0.20193603
C	4.19055708	-0.86689768	0.09094336
H	4.76083789	-0.67842304	-0.81603985
H	4.79604889	-0.51911658	0.92505955
H	4.06005243	-1.93907985	0.19155192
H	-1.11738953	-1.96014989	0.97456879

Time = 62.50 fs
E(CASSCF/6-31G(d)) = -479.5640281

C	-0.87911553	-0.22021214	-0.19652625
C	0.54269129	0.13814275	-0.08596037
C	1.21575344	1.41131911	-0.20075822
C	1.60992945	-0.83774133	0.10898500
C	2.85965905	-0.22113026	0.07006973
C	2.61218904	1.24803251	-0.09914546
N	-1.93777037	0.61281156	0.10766255
C	-3.21590518	-0.07436577	0.12602873
H	3.35972903	2.01262706	-0.15357952
C	-1.38306218	-1.63414986	-0.03745109
C	-2.90782535	-1.51929333	-0.23263475
H	-3.16897162	-1.68358901	-1.27060175
H	-3.45960481	-2.22627104	0.37401491
H	-3.68334136	0.02731745	1.10212856
H	-3.90702650	0.37317877	-0.59427749
H	-0.90347055	-2.30827463	-0.72559564
H	0.70964130	2.34808443	-0.33830568
C	-1.93092855	2.05431231	0.00236851

H	-1.44265608	2.52296323	0.84820019
H	-1.47087515	2.41003067	-0.91117002
H	-2.95831913	2.39773480	0.00218484
H	1.39734443	-1.89699618	0.20189970
C	4.19100590	-0.86541866	0.09078713
H	4.75666949	-0.68032850	-0.81916356
H	4.79364640	-0.51991953	0.92720255
H	4.06022077	-1.93766668	0.19186407
H	-1.11728310	-1.95565960	0.97179365

Time = 63.00 fs
E(CASSCF/6-31G(d)) = -479.5632684

C	-0.88203649	-0.21983210	-0.19708098
C	0.54255826	0.13687808	-0.08635466
C	1.21736977	1.41253181	-0.20170563
C	1.61224468	-0.83674271	0.11072831
C	2.85740209	-0.22389536	0.07126874
C	2.60960249	1.24781547	-0.09950050
N	-1.93591791	0.61036783	0.10909552
C	-3.21416433	-0.07265348	0.12661774
H	3.35994815	2.00706177	-0.15345368
C	-1.38286693	-1.63432955	-0.04012633
C	-2.90628101	-1.51943879	-0.23212478
H	-3.17004378	-1.68097502	-1.26886967
H	-3.45782400	-2.22689640	0.37469320
H	-3.68587168	0.02593485	1.10185258
H	-3.90855200	0.36639791	-0.59636421
H	-0.89821221	-2.30695135	-0.72585704
H	0.69655901	2.33827649	-0.34010485
C	-1.93008429	2.05616182	0.00215588
H	-1.44300287	2.52515548	0.84802421
H	-1.47538765	2.41088057	-0.91345179
H	-2.95729282	2.39856137	0.00506233
H	1.38188101	-1.88473920	0.20187500
C	4.19149408	-0.86402412	0.09064472
H	4.75259744	-0.68226084	-0.82229825
H	4.79142174	-0.52076349	0.92935999
H	4.06094876	-1.93647819	0.19220827
H	-1.11735205	-1.95142865	0.96889380

Time = 63.50 fs
E(CASSCF/6-31G(d)) = -479.5624147

C	-0.88541013	-0.21940536	-0.19755387
C	0.54310267	0.13574811	-0.08672423
C	1.21867035	1.41366538	-0.20268240

C	1.61403015	-0.83577578	0.11247487
C	2.85512481	-0.22609150	0.07241919
C	2.60702674	1.24712648	-0.09980605
N	-1.93379527	0.60785781	0.11038128
C	-3.21257115	-0.07094758	0.12723162
H	3.36029811	2.00176378	-0.15336828
C	-1.38264913	-1.63441342	-0.04281047
C	-2.90465680	-1.51963283	-0.23161844
H	-3.17098138	-1.67875990	-1.26723586
H	-3.45605914	-2.22742414	0.37526960
H	-3.68801081	0.02469136	1.10166588
H	-3.90926489	0.35982112	-0.59795175
H	-0.89286818	-2.30601806	-0.72647544
H	0.68406990	2.32888932	-0.34195837
C	-1.92930469	2.05809886	0.00200970
H	-1.44295865	2.52696441	0.84770703
H	-1.47960837	2.41114683	-0.91571498
H	-2.95625975	2.39919042	0.00796066
H	1.36752708	-1.87258840	0.20194723
C	4.19200660	-0.86272685	0.09051790
H	4.74868707	-0.68415561	-0.82540820
H	4.78943927	-0.52159164	0.93148352
H	4.06224145	-1.93552107	0.19258510
H	-1.11755140	-1.94753397	0.96599138

Time = 64.00 fs
E(CASSCF/6-31G(d)) = -479.5615338

C	-0.88918985	-0.21895218	-0.19795948
C	0.54432427	0.13477329	-0.08706854
C	1.21962777	1.41468583	-0.20368791
C	1.61524429	-0.83481087	0.11422101
C	2.85285668	-0.22769840	0.07351854
C	2.60447486	1.24596018	-0.10006003
N	-1.93142953	0.60533203	0.11152549
C	-3.21113518	-0.06928063	0.12787488
H	3.36084091	1.99684201	-0.15333525
C	-1.38240554	-1.63439680	-0.04550579
C	-2.90296238	-1.51987321	-0.23111388
H	-3.17179385	-1.67698049	-1.26576719
H	-3.45430182	-2.22784098	0.37574109
H	-3.68969739	0.02364093	1.10153272
H	-3.90911541	0.35358918	-0.59904565
H	-0.88750291	-2.30546210	-0.72745313
H	0.67228734	2.32020943	-0.34389357
C	-1.92859167	2.06009823	0.00193605
H	-1.44246896	2.52839603	0.84723803

H	-1.48347911	2.41084950	-0.91798543
H	-2.95528112	2.39959815	0.01087174
H	1.35438070	-1.86126713	0.20220201
C	4.19252861	-0.86153793	0.09040824
H	4.74499909	-0.68595081	-0.82845258
H	4.78775640	-0.52234988	0.93351996
H	4.06409272	-1.93479206	0.19299407
H	-1.11783475	-1.94404618	0.96320181

Time = 64.50 fs
E(CASSCF/6-31G(d)) = -479.5606785

C	-0.89331946	-0.21849331	-0.19831458
C	0.54621086	0.13397061	-0.08738818
C	1.22021909	1.41556423	-0.20472162
C	1.61585620	-0.83381988	0.11596391
C	2.85062613	-0.22870628	0.07456560
C	2.60196237	1.24432150	-0.10026151
N	-1.92885292	0.60284107	0.11253706
C	-3.20986003	-0.06768489	0.12855343
H	3.36159664	1.99235079	-0.15336321
C	-1.38213191	-1.63427878	-0.04821583
C	-2.90120847	-1.52015788	-0.23061080
H	-3.17249079	-1.67566441	-1.26451480
H	-3.45254616	-2.22814367	0.37611216
H	-3.69088359	0.02283466	1.10142935
H	-3.90809944	0.34785109	-0.59968926
H	-0.88219464	-2.30523991	-0.72876370
H	0.66132156	2.31247977	-0.34593377
C	-1.92794480	2.06213401	0.00193894
H	-1.44148114	2.52946787	0.84661412
H	-1.48695406	2.41001948	-0.92028591
H	-2.95442200	2.39976808	0.01379075
H	1.34250764	-1.85145009	0.20271890
C	4.19304578	-0.86046578	0.09031707
H	4.74158530	-0.68758818	-0.83138544
H	4.78642112	-0.52298771	0.93541021
H	4.06648475	-1.93427872	0.19343429
H	-1.11815599	-1.94102585	0.96062648

Time = 65.00 fs
E(CASSCF/6-31G(d)) = -479.5599682

C	-0.89773223	-0.21804986	-0.19863572
C	0.54873786	0.13335437	-0.08768489
C	1.22042288	1.41627697	-0.20578300
C	1.61584251	-0.83278200	0.11770149

C	2.84846164	-0.22911308	0.07556011
C	2.59950982	1.24222489	-0.10041052
N	-1.92610373	0.60043620	0.11342561
C	-3.20874308	-0.06619237	0.12927410
H	3.36253762	1.98829613	-0.15345767
C	-1.38182286	-1.63406240	-0.05094283
C	-2.89940729	-1.52048397	-0.23011100
H	-3.17307638	-1.67483096	-1.26351059
H	-3.45078886	-2.22833667	0.37639341
H	-3.69153427	0.02231789	1.10134641
H	-3.90625494	0.34276427	-0.59995803
H	-0.87703823	-2.30527874	-0.73035831
H	0.65128701	2.30589334	-0.34809533
C	-1.92736160	2.06417818	0.00202047
H	-1.43994810	2.53020799	0.84584256
H	-1.48999738	2.40869614	-0.92263132
H	-2.95374355	2.39969083	0.01671222
H	1.33195817	-1.84371431	0.20356622
C	4.19354482	-0.85951711	0.09024550
H	4.73849154	-0.68901443	-0.83415671
H	4.78547220	-0.52345997	0.93709264
H	4.06938928	-1.93396100	0.19390378
H	-1.11847350	-1.93852081	0.95833794

Time = 65.50 fs
E(CASSCF/6-31G(d)) = -479.5594812

C	-0.90235354	-0.21764326	-0.19894087
C	0.55186966	0.13293553	-0.08796145
C	1.22022223	1.41680672	-0.20687171
C	1.61518915	-0.83168710	0.11943323
C	2.84638899	-0.22892638	0.07650303
C	2.59713969	1.23969410	-0.10050824
N	-1.92322483	0.59816784	0.11420299
C	-3.20777609	-0.06483349	0.13004408
H	3.36359268	1.98463436	-0.15362116
C	-1.38147228	-1.63375405	-0.05368755
C	-2.89757210	-1.52084855	-0.22961842
H	-3.17355191	-1.67448918	-1.26276399
H	-3.44902932	-2.22843331	0.37660233
H	-3.69163097	0.02213057	1.10129032
H	-3.90365728	0.33848500	-0.59995480
H	-0.87213585	-2.30548588	-0.73217164
H	0.64230239	2.30058319	-0.35038789
C	-1.92683777	2.06620285	0.00218058
H	-1.43782992	2.53065222	0.84493471
H	-1.49258846	2.40692507	-0.92502577

H	-2.95329919	2.39936292	0.01963290
H	1.32278170	-1.83847226	0.20479590
C	4.19401362	-0.85869604	0.09019441
H	4.73575419	-0.69018217	-0.83671377
H	4.78493781	-0.52372694	0.93850348
H	4.07276726	-1.93381303	0.19440018
H	-1.11875107	-1.93656407	0.95637680

Time = 66.00 fs
E(CASSCF/6-31G(d)) = -479.5592744

C	-0.90710219	-0.21729518	-0.19924915
C	0.55555985	0.13272183	-0.08822158
C	1.21960483	1.41714276	-0.20798753
C	1.61389083	-0.83053890	0.12116027
C	2.84443056	-0.22816310	0.07739650
C	2.59487575	1.23676139	-0.10055691
N	-1.92026316	0.59608505	0.11488302
C	-3.20694596	-0.06363618	0.13087008
H	3.36465508	1.98128105	-0.15385362
C	-1.38107370	-1.63336270	-0.05644818
C	-2.89571716	-1.52124870	-0.22913901
H	-3.17391546	-1.67463823	-1.26226226
H	-3.44726939	-2.22845481	0.37676227
H	-3.69117235	0.02230639	1.10128359
H	-3.90041201	0.33516257	-0.59980061
H	-0.86759109	-2.30575828	-0.73413068
H	0.63449119	2.29662017	-0.35281339
C	-1.92636759	2.06818069	0.00241708
H	-1.43509611	2.53084178	0.84390443
H	-1.49472214	2.40475592	-0.92746094
H	-2.95312848	2.39878618	0.02255149
H	1.31504141	-1.83593333	0.20643988
C	4.19444160	-0.85800424	0.09016440
H	4.73339990	-0.69105050	-0.83900277
H	4.78483484	-0.52375489	0.93957860
H	4.07657013	-1.93380383	0.19492080
H	-1.11895961	-1.93517319	0.95474725

Time = 66.50 fs
E(CASSCF/6-31G(d)) = -479.5593880

C	-0.91189127	-0.21702698	-0.19958054
C	0.55975136	0.13271773	-0.08846986
C	1.21856395	1.41728066	-0.20913022
C	1.61195117	-0.82935617	0.12288560
C	2.84260450	-0.22684929	0.07824379

C	2.59274135	1.23346629	-0.10055980
N	-1.91726904	0.59423492	0.11548099
C	-3.20623558	-0.06262509	0.13175775
H	3.36559429	1.97812497	-0.15415338
C	-1.38062075	-1.63289947	-0.05921943
C	-2.89385761	-1.52168152	-0.22868058
H	-3.17416207	-1.67526771	-1.26197226
H	-3.44551271	-2.22842846	0.37690105
H	-3.69017425	0.02287165	1.10136294
H	-3.89664482	0.33293203	-0.59962408
H	-0.86350297	-2.30599047	-0.73616349
H	0.62797807	2.29401661	-0.35536666
C	-1.92594414	2.07008546	0.00272601
H	-1.43172841	2.53082095	0.84276481
H	-1.49640875	2.40224099	-0.92991498
H	-2.95325333	2.39796728	0.02546812
H	1.30882096	-1.83608708	0.20850824
C	4.19481969	-0.85744090	0.09015583
H	4.73144632	-0.69158546	-0.84097200
H	4.78517014	-0.52351610	0.94025714
H	4.08074043	-1.93390088	0.19546288
H	-1.11907770	-1.93434990	0.95341630

Time = 67.00 fs
E(CASSCF/6-31G(d)) = -479.5597900

C	-0.91663010	-0.21685926	-0.19995569
C	0.56437712	0.13292439	-0.08871169
C	1.21709921	1.41722193	-0.21029951
C	1.60938321	-0.82817188	0.12461403
C	2.84092421	-0.22501999	0.07904920
C	2.59075778	1.22985418	-0.10052118
N	-1.91429503	0.59266167	0.11601347
C	-3.20562511	-0.06182066	0.13271082
H	3.36627056	1.97504155	-0.15451807
C	-1.38010770	-1.63237672	-0.06199225
C	-2.89200921	-1.52214417	-0.22825221
H	-3.17428457	-1.67635813	-1.26184365
H	-3.44376330	-2.22838537	0.37704909
H	-3.68866857	0.02384419	1.10157613
H	-3.89249019	0.33190616	-0.59954866
H	-0.85995928	-2.30608375	-0.73820708
H	0.62288300	2.29273313	-0.35803630
C	-1.92555966	2.07189256	0.00310192
H	-1.42772296	2.53063426	0.84152515
H	-1.49767367	2.39943405	-0.93235313
H	-2.95367429	2.39691688	0.02838457

H	1.30422360	-1.83871136	0.21098980
C	4.19514042	-0.85700269	0.09016875
H	4.72990253	-0.69176063	-0.84257422
H	4.78594099	-0.52298886	0.94048395
H	4.08521311	-1.93407187	0.19602378
H	-1.11909174	-1.93408102	0.95231637

Time = 67.50 fs
E(CASSCF/6-31G(d)) = -479.5604247

C	-0.92122615	-0.21681117	-0.20039568
C	0.56936087	0.13333979	-0.08895317
C	1.21521749	1.41697333	-0.21149497
C	1.60621043	-0.82703031	0.12635192
C	2.83939785	-0.22271907	0.07981817
C	2.58894265	1.22597509	-0.10044620
N	-1.91139468	0.59140591	0.11649806
C	-3.20509332	-0.06123833	0.13373042
H	3.36654990	1.97190764	-0.15494553
C	-1.37953004	-1.63180730	-0.06475386
C	-2.89018812	-1.52263399	-0.22786371
H	-3.17427506	-1.67788199	-1.26181426
H	-3.44202446	-2.22835833	0.37723766
H	-3.68670145	0.02523275	1.10197756
H	-3.88807943	0.33216806	-0.59967939
H	-0.85703081	-2.30595408	-0.74021445
H	0.61931328	2.29268840	-0.36080635
C	-1.92520584	2.07357933	0.00353836
H	-1.42309194	2.53032418	0.84018854
H	-1.49855579	2.39638974	-0.93473015
H	-2.95436907	2.39564950	0.03130410
H	1.30136162	-1.84340729	0.21385484
C	4.19539794	-0.85668390	0.09020298
H	4.72876954	-0.69155721	-0.84376952
H	4.78713613	-0.52215722	0.94021255
H	4.08991685	-1.93428648	0.19660118
H	-1.11899481	-1.93434047	0.95135192

Time = 68.00 fs
E(CASSCF/6-31G(d)) = -479.5612108

C	-0.92558721	-0.21689942	-0.20092161
C	0.57461789	0.13395881	-0.08920095
C	1.21293389	1.41654609	-0.21271588
C	1.60246828	-0.82598277	0.12810688
C	2.83802815	-0.21999879	0.08055708
C	2.58730830	1.22188224	-0.10034085

N	-1.90862100	0.59050353	0.11695291
C	-3.20461918	-0.06088783	0.13481450
H	3.36631809	1.96861490	-0.15543454
C	-1.37888490	-1.63120392	-0.06748809
C	-2.88841059	-1.52314864	-0.22752487
H	-3.17412666	-1.67980510	-1.26181619
H	-3.44029777	-2.22837933	0.37749679
H	-3.68433089	0.02703638	1.10262312
H	-3.88352933	0.33376457	-0.60009064
H	-0.85476703	-2.30553833	-0.74216013
H	0.61735595	2.29376982	-0.36365803
C	-1.92487399	2.07512548	0.00402839
H	-1.41786442	2.52992950	0.83875015
H	-1.49910523	2.39316329	-0.93699373
H	-2.95529348	2.39418380	0.03423133
H	1.30034034	-1.84965014	0.21705912
C	4.19558783	-0.85647646	0.09025806
H	4.72804072	-0.69096412	-0.84452768
H	4.78873662	-0.52101066	0.93940759
H	4.09477559	-1.93451776	0.19719328
H	-1.11878513	-1.93509213	0.95040821

Time = 68.50 fs
E(CASSCF/6-31G(d)) = -479.5620639

C	-0.92962371	-0.21713733	-0.20155418
C	0.58005616	0.13477340	-0.08946212
C	1.21027226	1.41595514	-0.21396118
C	1.59820500	-0.82508310	0.12988738
C	2.83681248	-0.21691929	0.08127328
C	2.58586063	1.21763088	-0.10021185
N	-1.90602486	0.58998476	0.11739620
C	-3.20418317	-0.06077265	0.13595759
H	3.36549223	1.96508119	-0.15598559
C	-1.37817141	-1.63057861	-0.07017607
C	-2.88669269	-1.52368613	-0.22724478
H	-3.17383516	-1.68208793	-1.26178222
H	-3.43858184	-2.22847728	0.37785340
H	-3.68162346	0.02924378	1.10356415
H	-3.87893296	0.33670098	-0.60081593
H	-0.85319305	-2.30479861	-0.74404341
H	0.61706975	2.29584507	-0.36657168
C	-1.92455521	2.07651319	0.00456506
H	-1.41208656	2.52948392	0.83719670
H	-1.49938065	2.38981046	-0.93908878
H	-2.95638354	2.39254288	0.03717211
H	1.30123999	-1.85684724	0.22054869

C	4.19570707	-0.85637017	0.09033326
H	4.72770273	-0.68997794	-0.84483051
H	4.79071706	-0.51954375	0.93804720
H	4.09971019	-1.93474337	0.19779887
H	-1.11846413	-1.93629287	0.94936142

Time = 69.00 fs
E(CASSCF/6-31G(d)) = -479.5629099

C	-0.93325096	-0.21753370	-0.20231314
C	0.58557723	0.13577248	-0.08974415
C	1.20726544	1.41521838	-0.21522937
C	1.59348205	-0.82438320	0.13170235
C	2.83574331	-0.21354801	0.08197502
C	2.58459848	1.21327748	-0.10006656
N	-1.90365333	0.58987311	0.11784568
C	-3.20376828	-0.06088974	0.13715077
H	3.36402942	1.96125892	-0.15660127
C	-1.37739096	-1.62994221	-0.07279704
C	-2.88504995	-1.52424481	-0.22703113
H	-3.17340036	-1.68468691	-1.26165163
H	-3.43687132	-2.22867580	0.37832964
H	-3.67865037	0.03183279	1.10484129
H	-3.87435338	0.34093756	-0.60184127
H	-0.85230845	-2.30372456	-0.74588952
H	0.61847855	2.29877307	-0.36952858
C	-1.92424050	2.07772704	0.00514180
H	-1.40582077	2.52901541	0.83550696
H	-1.49944635	2.38638755	-0.94096211
H	-2.95755965	2.39075484	0.04013345
H	1.30409953	-1.86439424	0.22426482
C	4.19575387	-0.85635283	0.09042762
H	4.72773624	-0.68860277	-0.84467364
H	4.79304685	-0.51775571	0.93612489
H	4.10464020	-1.93494658	0.19841752
H	-1.11803445	-1.93789565	0.94808896

Time = 69.50 fs
E(CASSCF/6-31G(d)) = -479.5636960

C	-0.93639190	-0.21809168	-0.20321656
C	0.59107809	0.13694223	-0.09005457
C	1.20395510	1.41435620	-0.21651857
C	1.58837346	-0.82392949	0.13356077
C	2.83480890	-0.20995876	0.08267139
C	2.58351324	1.20887884	-0.09991298
N	-1.90154791	0.59018430	0.11831799

C	-3.20336071	-0.06122942	0.13838210
H	3.36193202	1.95714088	-0.15728662
C	-1.37654721	-1.62930413	-0.07532951
C	-2.88349712	-1.52482339	-0.22688967
H	-3.17282780	-1.68755621	-1.26137620
H	-3.43515662	-2.22899198	0.37894188
H	-3.67548384	0.03477031	1.10647898
H	-3.86982201	0.34638954	-0.60310395
H	-0.85208748	-2.30233350	-0.74774911
H	0.62156721	2.30241279	-0.37251237
C	-1.92392080	2.07875401	0.00575285
H	-1.39914374	2.52854595	0.83365335
H	-1.49936899	2.38295133	-0.94256738
H	-2.95873176	2.38885327	0.04312341
H	1.30890486	-1.87172427	0.22814830
C	4.19572767	-0.85641062	0.09053994
H	4.72811645	-0.68685029	-0.84406691
H	4.79569091	-0.51565039	0.93364999
H	4.10948585	-1.93511580	0.19904951
H	-1.11749762	-1.93985289	0.94648014

Time = 70.00 fs
E(CASSCF/6-31G(d)) = -479.5643554

C	-0.93897859	-0.21880783	-0.20428012
C	0.59645357	0.13826595	-0.09040095
C	1.20039096	1.41339039	-0.21782648
C	1.58296424	-0.82375953	0.13547129
C	2.83399382	-0.20623017	0.08337206
C	2.58258906	1.20449086	-0.09975942
N	-1.89974289	0.59092533	0.11882804
C	-3.20295051	-0.06177583	0.13963739
H	3.35924849	1.95276163	-0.15804943
C	-1.37564609	-1.62867244	-0.07775231
C	-2.88204779	-1.52542089	-0.22682367
H	-3.17212944	-1.69064904	-1.26092377
H	-3.43342369	-2.22943513	0.37969950
H	-3.67219425	0.03801180	1.10848077
H	-3.86534138	0.35292887	-0.60449601
H	-0.85248159	-2.30066799	-0.74969514
H	0.62627900	2.30663154	-0.37551066
C	-1.92358709	2.07958376	0.00639340
H	-1.39214418	2.52809264	0.83160449
H	-1.49921487	2.37955946	-0.94386892
H	-2.95980572	2.38687813	0.04615074
H	1.31558231	-1.87834610	0.23214292
C	4.19562875	-0.85652815	0.09066881

H	4.72881363	-0.68473922	-0.84303532
H	4.79861063	-0.51323576	0.93064857
H	4.11416964	-1.93524535	0.19969587
H	-1.11685238	-1.94211951	0.94444394

Time = 70.50 fs
E(CASSCF/6-31G(d)) = -479.5648772

C	-0.94095474	-0.21967118	-0.20551663
C	0.60159690	0.13972378	-0.09079064
C	1.19663047	1.41234423	-0.21915037
C	1.57734899	-0.82389992	0.13744197
C	2.83328005	-0.20244537	0.08408731
C	2.58180334	1.20016889	-0.09961463
N	-1.89826407	0.59209361	0.11938839
C	-3.20253102	-0.06250732	0.14090121
H	3.35607222	1.94819648	-0.15889989
C	-1.37469551	-1.62805370	-0.08004569
C	-2.88071413	-1.52603650	-0.22683376
H	-3.17132416	-1.69391886	-1.26028147
H	-3.43165450	-2.23000670	0.38060513
H	-3.66884643	0.04150169	1.11082680
H	-3.86089099	0.36038951	-0.60587291
H	-0.85342307	-2.29879193	-0.75181805
H	0.63251650	2.31130907	-0.37851516
C	-1.92323042	2.08020813	0.00705975
H	-1.38491896	2.52766817	0.82932927
H	-1.49904692	2.37626961	-0.94484576
H	-2.96068942	2.38487603	0.04922496
H	1.32399729	-1.88387266	0.23619899
C	4.19545828	-0.85668889	0.09081261
H	4.72979342	-0.68229506	-0.84161787
H	4.80176429	-0.51052396	0.92716255
H	4.11861847	-1.93533394	0.20035821
H	-1.11609312	-1.94465495	0.94191643

Time = 71.00 fs
E(CASSCF/6-31G(d)) = -479.5652296

C	-0.94227737	-0.22066298	-0.20693540
C	0.60640322	0.14129281	-0.09123054
C	1.19273693	1.41124208	-0.22048731
C	1.57162827	-0.82436503	0.13947988
C	2.83264802	-0.19868928	0.08482779
C	2.58112793	1.19596638	-0.09948754
N	-1.89712764	0.59367655	0.12000890
C	-3.20209868	-0.06339727	0.14215810

H	3.35253464	1.94355804	-0.15985047
C	-1.37370489	-1.62745332	-0.08219248
C	-2.87950662	-1.52666946	-0.22691780
H	-3.17043790	-1.69732033	-1.25945655
H	-3.42982794	-2.23070040	0.38165485
H	-3.66549746	0.04517394	1.11347248
H	-3.85643619	0.36857484	-0.60706558
H	-0.85483088	-2.29678411	-0.75421867
H	0.64014493	2.31634172	-0.38152246
C	-1.92284194	2.08062128	0.00774919
H	-1.37756946	2.52728181	0.82679980
H	-1.49892315	2.37313869	-0.94549327
H	-2.96129929	2.38289994	0.05235633
H	1.33395861	-1.88804174	0.24027536
C	4.19521822	-0.85687553	0.09096959
H	4.73101728	-0.67955015	-0.83986669
H	4.80510781	-0.50753150	0.92324877
H	4.12276481	-1.93538421	0.20103875
H	-1.11520899	-1.94742524	0.93886524

Time = 71.50 fs
E(CASSCF/6-31G(d)) = -479.5653901

C	-0.94291761	-0.22175654	-0.20854168
C	0.61077151	0.14294687	-0.09172690
C	1.18877803	1.41010906	-0.22183427
C	1.56590580	-0.82515578	0.14159081
C	2.83207749	-0.19504726	0.08560434
C	2.58053045	1.19193459	-0.09938713
N	-1.89633955	0.59565134	0.12069620
C	-3.20165234	-0.06441522	0.14339375
H	3.34879641	1.93898756	-0.16091519
C	-1.37268456	-1.62687595	-0.08417911
C	-2.87843382	-1.52731891	-0.22707102
H	-3.16950305	-1.70081028	-1.25847584
H	-3.42792107	-2.23150325	0.38283903
H	-3.66219552	0.04895283	1.11635011
H	-3.85193863	0.37726685	-0.60789508
H	-0.85661706	-2.29473050	-0.75700014
H	0.64899684	2.32164275	-0.38453386
C	-1.92241304	2.08081971	0.00845991
H	-1.37019713	2.52694091	0.82399551
H	-1.49889490	2.37022213	-0.94582453
H	-2.96156573	2.38100907	0.05555579
H	1.34522455	-1.89072702	0.24434126
C	4.19491129	-0.85707036	0.09113790
H	4.73244233	-0.67654300	-0.83784491

H	4.80859472	-0.50427916	0.91897690
H	4.12654845	-1.93540108	0.20174013
H	-1.11418317	-1.95040420	0.93529184

Time = 72.00 fs
E(CASSCF/6-31G(d)) = -479.5653460

C	-0.94286114	-0.22291763	-0.21033627
C	0.61460683	0.14465646	-0.09228512
C	1.18482405	1.40897084	-0.22318827
C	1.56028520	-0.82625919	0.14377902
C	2.83154828	-0.19160306	0.08642775
C	2.57997613	1.18812218	-0.09932227
N	-1.89589525	0.59798514	0.12145349
C	-3.20119247	-0.06552795	0.14459599
H	3.34503516	1.93464401	-0.16210883
C	-1.37164524	-1.62632591	-0.08599662
C	-2.87750222	-1.52798371	-0.22728626
H	-3.16855753	-1.70434822	-1.25738354
H	-3.42591080	-2.23239699	0.38414356
H	-3.65897956	0.05275428	1.11937269
H	-3.84736646	0.38623625	-0.60818769
H	-0.85869398	-2.29271561	-0.76025882
H	0.65887874	2.32714083	-0.38755488
C	-1.92193542	2.08080230	0.00919073
H	-1.36289924	2.52665212	0.82090652
H	-1.49900588	2.36757261	-0.94587002
H	-2.96143744	2.37926812	0.05883483
H	1.35751214	-1.89194250	0.24837737
C	4.19454079	-0.85725557	0.09131558
H	4.73402140	-0.67331782	-0.83562411
H	4.81217625	-0.50079202	0.91442705
H	4.12991779	-1.93539025	0.20246518
H	-1.11299274	-1.95357392	0.93123152

Time = 72.50 fs
E(CASSCF/6-31G(d)) = -479.5650944

C	-0.94210821	-0.22410535	-0.21231534
C	0.61782253	0.14638884	-0.09290957
C	1.18094583	1.40785341	-0.22454651
C	1.55486681	-0.82764840	0.14604699
C	2.83104100	-0.18843685	0.08730850
C	2.57942976	1.18457486	-0.09930152
N	-1.89578003	0.60063575	0.12228046
C	-3.20072035	-0.06670085	0.14575559
H	3.34143111	1.93069089	-0.16344589

C	-1.37059725	-1.62580770	-0.08764136
C	-2.87671609	-1.52866227	-0.22755444
H	-3.16764324	-1.70789665	-1.25623745
H	-3.42377567	-2.23335989	0.38555141
H	-3.65587990	0.05648767	1.12243961
H	-3.84270346	0.39525290	-0.60778903
H	-0.86098148	-2.29081372	-0.76407503
H	0.66957877	2.33277686	-0.39059437
C	-1.92140128	2.08057042	0.00994081
H	-1.35576485	2.52642233	0.81753638
H	-1.49929189	2.36523875	-0.94567602
H	-2.96088414	2.37774603	0.06220546
H	1.37050833	-1.89183960	0.25237641
C	4.19411069	-0.85741364	0.09150069
H	4.73570304	-0.66992397	-0.83328148
H	4.81580133	-0.49709962	0.90968687
H	4.13283092	-1.93535661	0.20321677
H	-1.11160906	-1.95692458	0.92675109

Time = 73.00 fs
E(CASSCF/6-31G(d)) = -479.5646467

C	-0.94067295	-0.22527340	-0.21447038
C	0.62034226	0.14810832	-0.09360349
C	1.17721275	1.40678283	-0.22590655
C	1.54974467	-0.82928342	0.14839524
C	2.83053757	-0.18562309	0.08825656
C	2.57885782	1.18133501	-0.09933303
N	-1.89596961	0.60355249	0.12317329
C	-3.20023729	-0.06789913	0.14686671
H	3.33815157	1.92728150	-0.16493935
C	-1.36954994	-1.62532637	-0.08911552
C	-2.87607741	-1.52935238	-0.22786512
H	-3.16680430	-1.71142105	-1.25510402
H	-3.42149772	-2.23436864	0.38704419
H	-3.65291938	0.06005803	1.12544388
H	-3.83795611	0.40409580	-0.60657625
H	-0.86341307	-2.28908101	-0.76850461
H	0.68087482	2.33849954	-0.39366347
C	-1.92080350	2.08012806	0.01070934
H	-1.34887133	2.52625929	0.81390384
H	-1.49978110	2.36326351	-0.94530169
H	-2.95989775	2.37651424	0.06568003
H	1.38388264	-1.89069599	0.25634280
C	4.19362550	-0.85752772	0.09169127
H	4.73743157	-0.66641527	-0.83089671
H	4.81941659	-0.49323585	0.90484851

H	4.13525637	-1.93530277	0.20399758
H	-1.10999865	-1.96045357	0.92194428

Time = 73.50 fs
E(CASSCF/6-31G(d)) = -479.5640316

C	-0.93858246	-0.22637168	-0.21678831
C	0.62210173	0.14977686	-0.09436881
C	1.17369074	1.40578487	-0.22726645
C	1.54500385	-0.83111285	0.15082230
C	2.83002149	-0.18322854	0.08928110
C	2.57823051	1.17844130	-0.09942427
N	-1.89643129	0.60667763	0.12412495
C	-3.19974401	-0.06908909	0.14792708
H	3.33533584	1.92454438	-0.16659946
C	-1.36851105	-1.62488785	-0.09042726
C	-2.87558590	-1.53005108	-0.22820711
H	-3.16608497	-1.71488966	-1.25405239
H	-3.41906418	-2.23540024	0.38860379
H	-3.65011510	0.06336864	1.12828012
H	-3.83315844	0.41256207	-0.60446735
H	-0.86594071	-2.28754942	-0.77357229
H	0.69254230	2.34426021	-0.39677439
C	-1.92013579	2.07948204	0.01149521
H	-1.34228153	2.52617185	0.81004343
H	-1.50049475	2.36168260	-0.94481527
H	-2.95849171	2.37564456	0.06927116
H	1.39730039	-1.88889444	0.26029143
C	4.19309030	-0.85758195	0.09188543
H	4.73914741	-0.66284921	-0.82854896
H	4.82296653	-0.48923894	0.90000550
H	4.13717358	-1.93522789	0.20480998
H	-1.10812454	-1.96416386	0.91692527

Time = 74.00 fs
E(CASSCF/6-31G(d)) = -479.5632981

C	-0.93587541	-0.22734823	-0.21925178
C	0.62305006	0.15135501	-0.09520618
C	1.17044046	1.40488449	-0.22862484
C	1.54071827	-0.83307663	0.15332478
C	2.82947807	-0.18131035	0.09039023
C	2.57752354	1.17592824	-0.09958194
N	-1.89712518	0.60994788	0.12512544
C	-3.19924010	-0.07023930	0.14893788
H	3.33308242	1.92257038	-0.16843245
C	-1.36748641	-1.62449898	-0.09159048

C	-2.87523910	-1.53075462	-0.22856921
H	-3.16552748	-1.71827302	-1.25314812
H	-3.41646893	-2.23643372	0.39021381
H	-3.64748017	0.06632386	1.13085260
H	-3.82837439	0.42047465	-0.60142759
H	-0.86853726	-2.28622326	-0.77926757
H	0.70436104	2.35000829	-0.39993928
C	-1.91939299	2.07864213	0.01229674
H	-1.33604185	2.52616971	0.80600498
H	-1.50144828	2.36052291	-0.94428941
H	-2.95669860	2.37520660	0.07299145
H	1.41043598	-1.88689078	0.26424512
C	4.19251064	-0.85756179	0.09208138
H	4.74078742	-0.65928600	-0.82631394
H	4.82639378	-0.48515104	0.89524978
H	4.13857321	-1.93512687	0.20565584
H	-1.10594824	-1.96806181	0.91182035

Time = 74.50 fs
E(CASSCF/6-31G(d)) = -479.5625146

C	-0.93260063	-0.22815123	-0.22183963
C	0.62315080	0.15280301	-0.09611492
C	1.16751579	1.40410524	-0.22998103
C	1.53694903	-0.83510983	0.15589758
C	2.82889444	-0.17991450	0.09159080
C	2.57671948	1.17382557	-0.09981176
N	-1.89800583	0.61329617	0.12616228
C	-3.19872369	-0.07132160	0.14990341
H	3.33143988	1.92140343	-0.17043962
C	-1.36647956	-1.62416741	-0.09262417
C	-2.87503244	-1.53145841	-0.22894077
H	-3.16516991	-1.72154339	-1.25244699
H	-3.41371358	-2.23745152	0.39186070
H	-3.64502545	0.06883207	1.13308269
H	-3.82369784	0.42768827	-0.59747258
H	-0.87119655	-2.28507879	-0.78554382
H	0.71612116	2.35568726	-0.40316921
C	-1.91857118	2.07762130	0.01311145
H	-1.33018127	2.52626272	0.80185182
H	-1.50265260	2.35980106	-0.94379622
H	-2.95456629	2.37526496	0.07685327
H	1.42298518	-1.88517064	0.26823071
C	4.19189250	-0.85745427	0.09227752
H	4.74228575	-0.65578745	-0.82426148
H	4.82963972	-0.48101775	0.89066904
H	4.13945695	-1.93499000	0.20653648

H	-1.10343202	-1.97215432	0.90675838

Time = 75.00 fs
E(CASSCF/6-31G(d)) = -479.5617689

C	-0.92881538	-0.22873094	-0.22452742
C	0.62238270	0.15408243	-0.09709310
C	1.16496225	1.40346855	-0.23133501
C	1.53374308	-0.83714774	0.15853436
C	2.82825965	-0.17907440	0.09288817
C	2.57580877	1.17215769	-0.10011835
N	-1.89902388	0.61665364	0.12722095
C	-3.19819114	-0.07231190	0.15083060
H	3.33040295	1.92103654	-0.17261652
C	-1.36549182	-1.62390133	-0.09355135
C	-2.87495948	-1.53215701	-0.22931232
H	-3.16504449	-1.72467419	-1.25198976
H	-3.41080817	-2.23844059	0.39353473
H	-3.64276084	0.07080873	1.13491528
H	-3.81925045	0.43409388	-0.59266836
H	-0.87393114	-2.28406725	-0.79232076
H	0.72762728	2.36123283	-0.40647320
C	-1.91766796	2.07643557	0.01393595
H	-1.32471154	2.52645953	0.79765767
H	-1.50411563	2.35952202	-0.94340187
H	-2.95215244	2.37587599	0.08086846
H	1.43467553	-1.88419819	0.27227425
C	4.19124227	-0.85724838	0.09247241
H	4.74357502	-0.65241603	-0.82245330
H	4.83264544	-0.47688755	0.88634418
H	4.13983735	-1.93480292	0.20745259
H	-1.10054162	-1.97644585	0.90186071

Time = 75.50 fs
E(CASSCF/6-31G(d)) = -479.5611386

C	-0.92458387	-0.22904176	-0.22728785
C	0.62073991	0.15515739	-0.09813766
C	1.16281701	1.40299246	-0.23268735
C	1.53113341	-0.83913006	0.16122791
C	2.82756420	-0.17881016	0.09428601
C	2.57478912	1.17094232	-0.10050497
N	-1.90012746	0.61995103	0.12828542
C	-3.19763744	-0.07319095	0.15172838
H	3.32991429	1.92141202	-0.17495320
C	-1.36452236	-1.62370897	-0.09439786
C	-2.87501199	-1.53284435	-0.22967596

H	-3.16517581	-1.72763938	-1.25179725
H	-3.40777105	-2.23939226	0.39522965
H	-3.64069647	0.07217864	1.13632275
H	-3.81517756	0.43962001	-0.58712806
H	-0.87676762	-2.28311989	-0.79948963
H	0.73870085	2.36657137	-0.40985843
C	-1.91668265	2.07510476	0.01476590
H	-1.31962776	2.52676693	0.79350369
H	-1.50584285	2.35967896	-0.94316293
H	-2.94951965	2.37708531	0.08504762
H	1.44527286	-1.88435941	0.27639476
C	4.19056626	-0.85693495	0.09266489
H	4.74458770	-0.64923293	-0.82094242
H	4.83535304	-0.47281038	0.88234833
H	4.13973682	-1.93454846	0.20840430
H	-1.09724900	-1.98093484	0.89723127

Time = 76.00 fs
E(CASSCF/6-31G(d)) = -479.5607237

C	-0.91997574	-0.22904377	-0.23009156
C	0.61823208	0.15599636	-0.09924448
C	1.16110726	1.40269109	-0.23403925
C	1.52913849	-0.84100655	0.16397077
C	2.82680025	-0.17912800	0.09578621
C	2.57366598	1.17018992	-0.10097346
N	-1.90126399	0.62312077	0.12933865
C	-3.19705590	-0.07394460	0.15260692
H	3.32987171	1.92242799	-0.17743417
C	-1.36356842	-1.62359789	-0.09519076
C	-2.87518023	-1.53351365	-0.23002561
H	-3.16557995	-1.73041324	-1.25186859
H	-3.40462854	-2.24030270	0.39694316
H	-3.63884298	0.07287839	1.13730811
H	-3.81164193	0.44423306	-0.58100605
H	-0.87974119	-2.28215547	-0.80692157
H	0.74918162	2.37162335	-0.41332989
C	-1.91561634	2.07365179	0.01559617
H	-1.31491033	2.52718777	0.78947346
H	-1.50783867	2.36025235	-0.94312240
H	-2.94672859	2.37892455	0.08940003
H	1.45458561	-1.88591526	0.28059932
C	4.18987084	-0.85650707	0.09285406
H	4.74525735	-0.64629686	-0.81977139
H	4.83770668	-0.46883647	0.87874453
H	4.13918708	-1.93420631	0.20939118
H	-1.09353512	-1.98561056	0.89294705

Time = 76.50 fs
E(CASSCF/6-31G(d)) = -479.5605929

C	-0.91506495	-0.22870429	-0.23290802
C	0.61488376	0.15657357	-0.10040846
C	1.15985061	1.40257399	-0.23539243
C	1.52776347	-0.84274074	0.16675576
C	2.82596162	-0.18002051	0.09738886
C	2.57245124	1.16990276	-0.10152414
N	-1.90238182	0.62609868	0.13036316
C	-3.19643872	-0.07456380	0.15347686
H	3.33013959	1.92394969	-0.18003921
C	-1.36262561	-1.62357440	-0.09595689
C	-2.87545323	-1.53415776	-0.23035713
H	-3.16626435	-1.73297014	-1.25218051
H	-3.40141415	-2.24117205	0.39867626
H	-3.63721103	0.07285863	1.13790510
H	-3.80881511	0.44793462	-0.57448846
H	-0.88288932	-2.28108918	-0.81447653
H	0.75892714	2.37630665	-0.41689067
C	-1.91447197	2.07210260	0.01642101
H	-1.31052817	2.52771868	0.78564798
H	-1.51010698	2.36120978	-0.94330616
H	-2.94383211	2.38140824	0.09393289
H	1.46246449	-1.88896577	0.28487912
C	4.18916206	-0.85596008	0.09303937
H	4.74552064	-0.64366245	-0.81897196
H	4.83965413	-0.46501514	0.87558506
H	4.13822817	-1.93375478	0.21041238
H	-1.08939256	-1.99045087	0.88905237

Time = 77.00 fs
E(CASSCF/6-31G(d)) = -479.5607600

C	-0.90992830	-0.22799897	-0.23570607
C	0.61073452	0.15687018	-0.10162368
C	1.15905473	1.40264486	-0.23674901
C	1.52700052	-0.84431133	0.16957614
C	2.82504351	-0.18146698	0.09909220
C	2.57116194	1.17007409	-0.10215571
N	-1.90343138	0.62882530	0.13134144
C	-3.19577753	-0.07504452	0.15434845
H	3.33056259	1.92582274	-0.18274478
C	-1.36168838	-1.62364292	-0.09672149
C	-2.87581889	-1.53476919	-0.23066808
H	-3.16722781	-1.73528499	-1.25268879

H	-3.39816704	-2.24200346	0.40043226
H	-3.63581099	0.07208481	1.13817662
H	-3.80686806	0.45075601	-0.56778132
H	-0.88624543	-2.27984088	-0.82201389
H	0.76781352	2.38054090	-0.42054252
C	-1.91325432	2.07048597	0.01723444
H	-1.30644103	2.52834926	0.78210120
H	-1.51265106	2.36250743	-0.94372307
H	-2.94087101	2.38453248	0.09865123
H	1.46880111	-1.89343580	0.28920757
C	4.18844546	-0.85529169	0.09322057
H	4.74531945	-0.64137877	-0.81856579
H	4.84114908	-0.46139304	0.87291130
H	4.13690715	-1.93317290	0.21146679
H	-1.08482708	-1.99542054	0.88555398

Time = 77.50 fs
E(CASSCF/6-31G(d)) = -479.5612165

C	-0.90464465	-0.22691247	-0.23845464
C	0.60583828	0.15687514	-0.10288336
C	1.15871765	1.40290129	-0.23811138
C	1.52683004	-0.84571214	0.17242574
C	2.82404259	-0.18343437	0.10089270
C	2.56981882	1.17068783	-0.10286524
N	-1.90436648	0.63124713	0.13225639
C	-3.19506417	-0.07538719	0.15523071
H	3.33098056	1.92788766	-0.18552533
C	-1.36075045	-1.62380543	-0.09750723
C	-2.87626428	-1.53534040	-0.23095745
H	-3.16846139	-1.73733372	-1.25333192
H	-3.39493044	-2.24280171	0.40221550
H	-3.63465166	0.07053803	1.13821061
H	-3.80595976	0.45275084	-0.56109669
H	-0.88983312	-2.27834313	-0.82940198
H	0.77573447	2.38425249	-0.42428625
C	-1.91196986	2.06883304	0.01803067
H	-1.30260313	2.52906064	0.77889566
H	-1.51547307	2.36409126	-0.94436485
H	-2.93787070	2.38827343	0.10355727
H	1.47352465	-1.89908521	0.29354139
C	4.18772582	-0.85450195	0.09339780
H	4.74460276	-0.63948785	-0.81856484
H	4.84215264	-0.45801264	0.87075355
H	4.13527677	-1.93244196	0.21255321
H	-1.07985856	-2.00047080	0.88242126

Time = 78.00 fs
E(CASSCF/6-31G(d)) = -479.5619172

C	-0.89929415	-0.22543905	-0.24112357
C	0.60026284	0.15658574	-0.10417993
C	1.15882823	1.40333441	-0.23948213
C	1.52722164	-0.84694992	0.17529883
C	2.82295702	-0.18587856	0.10278512
C	2.56844470	1.17171839	-0.10364817
N	-1.90514526	0.63331775	0.13309170
C	-3.19429160	-0.07559593	0.15613063
H	3.33124297	1.92999399	-0.18835501
C	-1.35980526	-1.62406099	-0.09833341
C	-2.87677582	-1.53586400	-0.23122514
H	-3.16994976	-1.73909436	-1.25403622
H	-3.39174981	-2.24357169	0.40403007
H	-3.63373845	0.06821517	1.13811423
H	-3.80622497	0.45398608	-0.55463709
H	-0.89366166	-2.27654762	-0.83652707
H	0.78260058	2.38737945	-0.42812279
C	-1.91062652	2.06717669	0.01880454
H	-1.29896624	2.52982446	0.77607859
H	-1.51857322	2.36589893	-0.94520795
H	-2.93483961	2.39258671	0.10865037
H	1.47659869	-1.90554052	0.29782421
C	4.18700699	-0.85359325	0.09357153
H	4.74332862	-0.63802336	-0.81897210
H	4.84263509	-0.45491063	0.86913123
H	4.13339428	-1.93154747	0.21367055
H	-1.07452098	-2.00554064	0.87958919

Time = 78.50 fs
E(CASSCF/6-31G(d)) = -479.5627913

C	-0.89395740	-0.22358271	-0.24368436
C	0.59408881	0.15600764	-0.10550509
C	1.15936684	1.40392919	-0.24086393
C	1.52813526	-0.84804105	0.17818996
C	2.82178664	-0.18874591	0.10476270
C	2.56706302	1.17313109	-0.10449836
N	-1.90573114	0.63499871	0.13383224
C	-3.19345485	-0.07567766	0.15705254
H	3.33122153	1.93201221	-0.19120902
C	-1.35884644	-1.62440543	-0.09921561
C	-2.87733947	-1.53633289	-0.23147148
H	-3.17167244	-1.74054761	-1.25472130
H	-3.38867058	-2.24431636	0.40587806

H	-3.63307108	0.06512838	1.13800587
H	-3.80776177	0.45453149	-0.54857975
H	-0.89772315	-2.27443003	-0.84330012
H	0.78833886	2.38987567	-0.43205364
C	-1.90923334	2.06555077	0.01955194
H	-1.29548251	2.53060275	0.77367936
H	-1.52194878	2.36786201	-0.94621629
H	-2.93177020	2.39740807	0.11392697
H	1.47801874	-1.91234065	0.30199205
C	4.18629152	-0.85257021	0.09374262
H	4.74146576	-0.63700941	-0.81978281
H	4.84257737	-0.45211655	0.86805347
H	4.13131979	-1.93048071	0.21481805
H	-1.06886164	-2.01055888	0.87696412

Time = 79.00 fs
E(CASSCF/6-31G(d)) = -479.5637600

C	-0.88871496	-0.22135699	-0.24611082
C	0.58740865	0.15515457	-0.10684975
C	1.16030612	1.40466481	-0.24225949
C	1.52952214	-0.84900762	0.18109354
C	2.82053306	-0.19197498	0.10681727
C	2.56569654	1.17488297	-0.10540828
N	-1.90609336	0.63626013	0.13446426
C	-3.19255203	-0.07564119	0.15799767
H	3.33082018	1.93384329	-0.19406490
C	-1.35786822	-1.62483114	-0.10016572
C	-2.87794086	-1.53674048	-0.23169658
H	-3.17360558	-1.74167808	-1.25530681
H	-3.38573634	-2.24503538	0.40775834
H	-3.63264120	0.06130423	1.13800641
H	-3.81062084	0.45445031	-0.54306288
H	-0.90199067	-2.27199261	-0.84966218
H	0.79289250	2.39171339	-0.43608115
C	-1.90780006	2.06398914	0.02027028
H	-1.29210673	2.53134827	0.77170802
H	-1.52559282	2.36990875	-0.94734510
H	-2.92864140	2.40265482	0.11938075
H	1.47781008	-1.91898802	0.30597967
C	4.18558060	-0.85143960	0.09391225
H	4.73899501	-0.63645974	-0.82098534
H	4.84197220	-0.44965159	0.86751949
H	4.12911505	-1.92923991	0.21599536
H	-1.06293903	-2.01544770	0.87443283

Time = 79.50 fs

E(CASSCF/6-31G(d)) = -479.5647421

C	-0.88364693	-0.21878467	-0.24837957
C	0.58032572	0.15404785	-0.10820401
C	1.16161190	1.40551554	-0.24367159
C	1.53132580	-0.84987364	0.18400365
C	2.81919976	-0.19549820	0.10893948
C	2.56436614	1.17692370	-0.10636909
N	-1.90620746	0.63708101	0.13497569
C	-3.19158512	-0.07549636	0.15896410
H	3.32998187	1.93542485	-0.19690362
C	-1.35686590	-1.62532705	-0.10119231
C	-2.87856551	-1.53708088	-0.23189980
H	-3.17572377	-1.74247533	-1.25571878
H	-3.38298720	-2.24572384	0.40966542
H	-3.63243011	0.05678256	1.13822989
H	-3.81479722	0.45379154	-0.53817514
H	-0.90641794	-2.26926505	-0.85558740
H	0.79622147	2.39288357	-0.44020862
C	-1.90633671	2.06252481	0.02095872
H	-1.28879761	2.53200598	0.77015526
H	-1.52949299	2.37196687	-0.94854535
H	-2.92542278	2.40822849	0.12500295
H	1.47602602	-1.92499714	0.30972648
C	4.18487395	-0.85021019	0.09408196
H	4.73591016	-0.63637722	-0.82256211
H	4.84082471	-0.44752778	0.86751920
H	4.12684201	-1.92783096	0.21720273
H	-1.05681992	-2.02012692	0.87187281

Time = 80.00 fs
E(CASSCF/6-31G(d)) = -479.5656652

C	-0.87883230	-0.21589708	-0.25047062
C	0.57295262	0.15271554	-0.10955726
C	1.16324409	1.40645182	-0.24510302
C	1.53348312	-0.85066191	0.18691381
C	2.81779232	-0.19924385	0.11111905
C	2.56309017	1.17919713	-0.10737098
N	-1.90605560	0.63744960	0.13535623
C	-3.19056039	-0.07525347	0.15994701
H	3.32869254	1.93673442	-0.19971025
C	-1.35583615	-1.62587874	-0.10230114
C	-2.87919895	-1.53734894	-0.23207931
H	-3.17800146	-1.74293464	-1.25589522
H	-3.38045818	-2.24637141	0.41158882
H	-3.63240643	0.05161509	1.13877420

H	-3.82022573	0.45258510	-0.53394956
H	-0.91094065	-2.26630277	-0.86108355
H	0.79830225	2.39339599	-0.44444020
C	-1.90485304	2.06118875	0.02161839
H	-1.28551851	2.53251473	0.76899360
H	-1.53363073	2.37396637	-0.94976793
H	-2.92207967	2.41401793	0.13078298
H	1.47274540	-1.92993653	0.31318189
C	4.18416977	-0.84889259	0.09425354
H	4.73221847	-0.63675360	-0.82449046
H	4.83915283	-0.44574745	0.86803372
H	4.12456152	-1.92626783	0.21844104
H	-1.05057622	-2.02451839	0.86916293

Time = 80.50 fs
E(CASSCF/6-31G(d)) = -479.5664608

C	-0.87434835	-0.21273338	-0.25236767
C	0.56540960	0.15119144	-0.11089819
C	1.16515782	1.40744149	-0.24655663
C	1.53592556	-0.85139150	0.18981688
C	2.81631834	-0.20313764	0.11334496
C	2.56188376	1.18164267	-0.10840328
N	-1.90562662	0.63736326	0.13559753
C	-3.18948863	-0.07492293	0.16093934
H	3.32698140	1.93778911	-0.20247442
C	-1.35477727	-1.62646878	-0.10349601
C	-2.87982688	-1.53754038	-0.23223178
H	-3.18041427	-1.74305765	-1.25579069
H	-3.37817831	-2.24696201	0.41351280
H	-3.63252460	0.04586412	1.13971328
H	-3.82678126	0.45084107	-0.53036324
H	-0.91547896	-2.26318355	-0.86619017
H	0.79912896	2.39327633	-0.44878044
C	-1.90335813	2.06000895	0.02225244
H	-1.28223718	2.53281007	0.76817982
H	-1.53798055	2.37584211	-0.95096791
H	-2.91857911	2.41990333	0.13670907
H	1.46806989	-1.93346000	0.31630913
C	4.18346479	-0.84749902	0.09442911
H	4.72794075	-0.63756979	-0.82674323
H	4.83698705	-0.44430314	0.86903590
H	4.12233212	-1.92457226	0.21971184
H	-1.04428123	-2.02855054	0.86619374

Time = 81.00 fs
E(CASSCF/6-31G(d)) = -479.5670684

C	-0.87026995	-0.20933963	-0.25405839
C	0.55782260	0.14951397	-0.11221500
C	1.16730458	1.40845123	-0.24803541
C	1.53858061	-0.85207606	0.19270508
C	2.81478741	-0.20710437	0.11560562
C	2.56075854	1.18419693	-0.10945478
N	-1.90491606	0.63682828	0.13569321
C	-3.18838482	-0.07451536	0.16193274
H	3.32491803	1.93864213	-0.20519019
C	-1.35368939	-1.62707718	-0.10477958
C	-2.88043530	-1.53765180	-0.23235220
H	-3.18293987	-1.74285247	-1.25537914
H	-3.37616984	-2.24747393	0.41541661
H	-3.63272396	0.03960115	1.14109093
H	-3.83428370	0.44855247	-0.52734227
H	-0.91994137	-2.26000199	-0.87097390
H	0.79871400	2.39256258	-0.45323350
C	-1.90186002	2.05900947	0.02286595
H	-1.27892468	2.53282721	0.76765840
H	-1.54250987	2.37753603	-0.95210791
H	-2.91489555	2.42576037	0.14276913
H	1.46212030	-1.93532807	0.31908790
C	4.18275434	-0.84604310	0.09461097
H	4.72311087	-0.63879637	-0.82928899
H	4.83436964	-0.44317798	0.87049056
H	4.12020899	-1.92277288	0.22101725
H	-1.03800620	-2.03216235	0.86287629

Time = 81.50 fs
E(CASSCF/6-31G(d)) = -479.5674372

C	-0.86666848	-0.20576769	-0.25553462
C	0.55032082	0.14772495	-0.11349540
C	1.16963338	1.40944782	-0.24954247
C	1.54137326	-0.85272270	0.19557000
C	2.81321098	-0.21106950	0.11788916
C	2.55972276	1.18679562	-0.11051402
N	-1.90392599	0.63585960	0.13563895
C	-3.18726743	-0.07404182	0.16291867
H	3.32260632	1.93937638	-0.20785572
C	-1.35257445	-1.62768211	-0.10615433
C	-2.88101065	-1.53768072	-0.23243392
H	-3.18555880	-1.74233394	-1.25465600
H	-3.37444844	-2.24788124	0.41727563
H	-3.63292947	0.03290600	1.14291810
H	-3.84250792	0.44570178	-0.52477183

H	-0.92422948	-2.25686273	-0.87552228
H	0.79708893	2.39130023	-0.45780260
C	-1.90036528	2.05820957	0.02346569
H	-1.27555429	2.53250436	0.76736528
H	-1.54717935	2.37899923	-0.95316095
H	-2.91101599	2.43146487	0.14895148
H	1.45503220	-1.93542129	0.32151575
C	4.18203257	-0.84453962	0.09480169
H	4.71777479	-0.64039434	-0.83209270
H	4.83135367	-0.44234617	0.87235515
H	4.11824281	-1.92090406	0.22235992
H	-1.03181693	-2.03530683	0.85914963

Time = 82.00 fs
E(CASSCF/6-31G(d)) = -479.5675287

C	-0.86361109	-0.20207418	-0.25679248
C	0.54303439	0.14586844	-0.11472676
C	1.17209220	1.41039950	-0.25108118
C	1.54422793	-0.85333134	0.19840287
C	2.81160166	-0.21496024	0.12018351
C	2.55878129	1.18937501	-0.11156950
N	-1.90266467	0.63448013	0.13543244
C	-3.18615743	-0.07351438	0.16388950
H	3.32017517	1.94009521	-0.21047250
C	-1.35143616	-1.62826065	-0.10762353
C	-2.88153998	-1.53762557	-0.23246889
H	-3.18825465	-1.74152316	-1.25363802
H	-3.37302352	-2.24815527	0.41906273
H	-3.63305338	0.02586603	1.14517303
H	-3.85119674	0.44226945	-0.52250926
H	-0.92824322	-2.25387249	-0.87993520
H	0.79430559	2.38953635	-0.46248901
C	-1.89887889	2.05762324	0.02405983
H	-1.27209949	2.53178624	0.76723235
H	-1.55194352	2.38019308	-0.95411216
H	-2.90694409	2.43689732	0.15524578
H	1.44695115	-1.93374487	0.32360810
C	4.18129271	-0.84300432	0.09500406
H	4.71198927	-0.64231619	-0.83511590
H	4.82800166	-0.44177405	0.87458001
H	4.11647894	-1.91900422	0.22374279
H	-1.02577046	-2.03795347	0.85498574

Time = 82.50 fs
E(CASSCF/6-31G(d)) = -479.5673285

C	-0.86115895	-0.19831933	-0.25783225
C	0.53609174	0.14398944	-0.11589652
C	1.17462887	1.41127684	-0.25265514
C	1.54706985	-0.85389461	0.20119459
C	2.80997322	-0.21870712	0.12247668
C	2.55793645	1.19187406	-0.11261005
N	-1.90114640	0.63272030	0.13507349
C	-3.18507688	-0.07294661	0.16483950
H	3.31776704	1.94091062	-0.21304396
C	-1.35027974	-1.62878976	-0.10919202
C	-2.88201105	-1.53748561	-0.23244801
H	-3.19101364	-1.74044654	-1.25236084
H	-3.37189836	-2.24826603	0.42074977
H	-3.63299791	0.01857479	1.14780365
H	-3.86007578	0.43824239	-0.52039820
H	-0.93188757	-2.25113048	-0.88431410
H	0.79043540	2.38731703	-0.46729140
C	-1.89740399	2.05725858	0.02465752
H	-1.26853285	2.53062685	0.76719111
H	-1.55675234	2.38109037	-0.95495925
H	-2.90270277	2.44194613	0.16164377
H	1.43802714	-1.93042682	0.32539799
C	4.18052728	-0.84145357	0.09522105
H	4.70582006	-0.64450741	-0.83831617
H	4.82438340	-0.44142140	0.87710836
H	4.11495676	-1.91711357	0.22516896
H	-1.01991349	-2.04008926	0.85039132

Time = 83.00 fs
E(CASSCF/6-31G(d)) = -479.5668304

C	-0.85936594	-0.19456576	-0.25865837
C	0.52961661	0.14213277	-0.11699224
C	1.17719209	1.41205366	-0.25426827
C	1.54982672	-0.85439828	0.20393587
C	2.80834014	-0.22224499	0.12475705
C	2.55718880	1.19423620	-0.11362514
N	-1.89939123	0.63061727	0.13456397
C	-3.18404746	-0.07235399	0.16576550
H	3.31552341	1.94193082	-0.21557404
C	-1.34911170	-1.62924711	-0.11086705
C	-2.88241250	-1.53726085	-0.23236168
H	-3.19382451	-1.73913512	-1.25087621
H	-3.37107156	-2.24818507	0.42230999
H	-3.63265963	0.01113142	1.15073392
H	-3.86886944	0.43362268	-0.51828317
H	-0.93507761	-2.24872121	-0.88875226

H	0.78557153	2.38468081	-0.47220443
C	-1.89594192	2.05711759	0.02526843
H	-1.26482451	2.52899245	0.76717712
H	-1.56155269	2.38167590	-0.95571214
H	-2.89833476	2.44651140	0.16813976
H	1.42841084	-1.92570787	0.32693419
C	4.17972848	-0.83990421	0.09545578
H	4.69934055	-0.64690822	-0.84164767
H	4.82057436	-0.44124305	0.87987725
H	4.11370860	-1.91527178	0.22664140
H	-1.01428068	-2.04171958	0.84540893

Time = 83.50 fs
E(CASSCF/6-31G(d)) = -479.5660729

C	-0.85827637	-0.19087724	-0.25927904
C	0.52372508	0.14034166	-0.11800216
C	1.17973246	1.41270749	-0.25592478
C	1.55243022	-0.85482196	0.20661770
C	2.80671712	-0.22551408	0.12701341
C	2.55653803	1.19641101	-0.11460513
N	-1.89742461	0.62821409	0.13390793
C	-3.18308916	-0.07175427	0.16666735
H	3.31357281	1.94324550	-0.21806529
C	-1.34793946	-1.62961214	-0.11265866
C	-2.88273406	-1.53695208	-0.23220041
H	-3.19667691	-1.73762284	-1.24924698
H	-3.37053716	-2.24788713	0.42371927
H	-3.63193432	0.00363921	1.15387145
H	-3.87731589	0.42843413	-0.51602311
H	-0.93774425	-2.24670540	-0.89332418
H	0.77982494	2.38165837	-0.47721923
C	-1.89449222	2.05719624	0.02590223
H	-1.26094133	2.52686374	0.76713292
H	-1.56629017	2.38194619	-0.95639152
H	-2.89390246	2.45050749	0.17473071
H	1.41824729	-1.91992704	0.32827874
C	4.17888843	-0.83837317	0.09571152
H	4.69262910	-0.64945464	-0.84506071
H	4.81665291	-0.44119007	0.88281740
H	4.11275986	-1.91351576	0.22816264
H	-1.00889434	-2.04286626	0.84011271

Time = 84.00 fs
E(CASSCF/6-31G(d)) = -479.5651043

C	-0.85792273	-0.18731742	-0.25970590

C	0.51852249	0.13865666	-0.11891544
C	1.18220314	1.41321977	-0.25762908
C	1.55481707	-0.85514063	0.20923147
C	2.80511893	-0.22846103	0.12923526
C	2.55598413	1.19835584	-0.11554152
N	-1.89527718	0.62555888	0.13311162
C	-3.18221888	-0.07116753	0.16754804
H	3.31201747	1.94491365	-0.22051727
C	-1.34677093	-1.62986694	-0.11457981
C	-2.88296665	-1.53656076	-0.23195550
H	-3.19956050	-1.73594530	-1.24754162
H	-3.37028590	-2.24735260	0.42495814
H	-3.63072278	-0.00379596	1.15711692
H	-3.88518123	0.42272701	-0.51350237
H	-0.93983831	-2.24511457	-0.89807765
H	0.77332340	2.37827094	-0.48232336
C	-1.89305273	2.05748479	0.02656806
H	-1.25684651	2.52423763	0.76701050
H	-1.57091152	2.38190961	-0.95702643
H	-2.88948518	2.45386509	0.18141644
H	1.40767312	-1.91349701	0.32950364
C	4.17799947	-0.83687721	0.09599157
H	4.68576721	-0.65208016	-0.84850203
H	4.81269818	-0.44121128	0.88585399
H	4.11212839	-1.91187768	0.22973461
H	-1.00376504	-2.04356591	0.83460334

Time = 84.50 fs
E(CASSCF/6-31G(d)) = -479.5640124

C	-0.85832465	-0.18394891	-0.25995387
C	0.51410172	0.13711493	-0.11972246
C	1.18455967	1.41357558	-0.25938579
C	1.55692909	-0.85532691	0.21176909
C	2.80356046	-0.23103890	0.13141309
C	2.55552885	1.20003672	-0.11642715
N	-1.89298442	0.62270393	0.13218348
C	-3.18144913	-0.07061593	0.16841344
H	3.31092228	1.94695525	-0.22292542
C	-1.34561422	-1.62999671	-0.11664610
C	-2.88310260	-1.53608897	-0.23161976
H	-3.20246385	-1.73413823	-1.24582810
H	-3.37030594	-2.24656946	0.42601385
H	-3.62893531	-0.01106685	1.16037324
H	-3.89227008	0.41658044	-0.51063809
H	-0.94133269	-2.24394825	-0.90302842
H	0.76621022	2.37453119	-0.48750053

C	-1.89161984	2.05796792	0.02727405
H	-1.25250019	2.52112693	0.76677281
H	-1.57536759	2.38158505	-0.95765036
H	-2.88517411	2.45653140	0.18819977
H	1.39681695	-1.90687212	0.33068645
C	4.17705452	-0.83543276	0.09629926
H	4.67883769	-0.65471798	-0.85191557
H	4.80878837	-0.44125514	0.88890783
H	4.11182411	-1.91038329	0.23135854
H	-0.99889311	-2.04386689	0.82900114

Time = 85.00 fs
E(CASSCF/6-31G(d)) = -479.5628807

C	-0.85948680	-0.18083217	-0.26004073
C	0.51054066	0.13574933	-0.12041519
C	1.18676064	1.41376338	-0.26119963
C	1.55871377	-0.85535374	0.21422351
C	2.80205648	-0.23320789	0.13353858
C	2.55517614	1.20142900	-0.11725637
N	-1.89058629	0.61970472	0.13113421
C	-3.18078717	-0.07012342	0.16927207
H	3.31030890	1.94934475	-0.22528002
C	-1.34447735	-1.62999029	-0.11887505
C	-2.88313587	-1.53553949	-0.23118812
H	-3.20537296	-1.73223599	-1.24416811
H	-3.37058346	-2.24553480	0.42688096
H	-3.62649755	-0.01806730	1.16355481
H	-3.89843512	0.41010257	-0.50738653
H	-0.94222274	-2.24317382	-0.90815879
H	0.75864064	2.37044517	-0.49273143
C	-1.89018883	2.05862560	0.02802697
H	-1.24785996	2.51756091	0.76639467
H	-1.57961515	2.38100116	-0.95829816
H	-2.88106690	2.45847087	0.19508569
H	1.38580026	-1.90051051	0.33190511
C	4.17604720	-0.83405556	0.09663783
H	4.67192288	-0.65730224	-0.85524306
H	4.80499850	-0.44127080	0.89189650
H	4.11184898	-1.90905064	0.23303482
H	-0.99427101	-2.04382521	0.82343620

Time = 85.50 fs
E(CASSCF/6-31G(d)) = -479.5618275

C	-0.86139788	-0.17802464	-0.25998665
C	0.50790099	0.13458784	-0.12098729

C	1.18876779	1.41377446	-0.26307523
C	1.56012389	-0.85519739	0.21658887
C	2.80062169	-0.23493535	0.13560470
C	2.55493263	1.20251749	-0.11802499
N	-1.88812681	0.61661904	0.12997662
C	-3.18023444	-0.06971513	0.17013461
H	3.31015534	1.95201174	-0.22756616
C	-1.34336790	-1.62984044	-0.12128499
C	-2.88306214	-1.53491571	-0.23065817
H	-3.20827025	-1.73026989	-1.24261157
H	-3.37110302	-2.24425514	0.42756195
H	-3.62335406	-0.02469376	1.16659420
H	-3.90358168	0.40342911	-0.50374536
H	-0.94252515	-2.24272877	-0.91341881
H	0.75077674	2.36601603	-0.49799520
C	-1.88875419	2.05943374	0.02883187
H	-1.24288358	2.51358327	0.76586192
H	-1.58361892	2.38019411	-0.95900100
H	-2.87726040	2.45966338	0.20208019
H	1.37473990	-1.89483243	0.33323305
C	4.17497213	-0.83276048	0.09701037
H	4.66510377	-0.65976979	-0.85842547
H	4.80139930	-0.44120953	0.89473637
H	4.11219721	-1.90788978	0.23476283
H	-0.98988710	-2.04349938	0.81803771

Time = 86.00 fs
E(CASSCF/6-31G(d)) = -479.5609368

C	-0.86402944	-0.17557996	-0.25981412
C	0.50622659	0.13365294	-0.12143442
C	1.19054682	1.41360232	-0.26501699
C	1.56111780	-0.85484042	0.21886091
C	2.79927011	-0.23619623	0.13760596
C	2.55480710	1.20329616	-0.11873045
N	-1.88565364	0.61350603	0.12872568
C	-3.17978626	-0.06941674	0.17101334
H	3.31039934	1.95484437	-0.22976409
C	-1.34229297	-1.62954380	-0.12389351
C	-2.88287898	-1.53422179	-0.23003049
H	-3.21113373	-1.72826730	-1.24119252
H	-3.37184761	-2.24274696	0.42806714
H	-3.61947199	-0.03084795	1.16944863
H	-3.90767145	0.39671949	-0.49975360
H	-0.94227483	-2.24252647	-0.91873253
H	0.74278361	2.36124844	-0.50327068
C	-1.88731008	2.06036522	0.02969200

H	-1.23753040	2.50925078	0.76517030
H	-1.58735346	2.37920632	-0.95978335
H	-2.87384314	2.46010368	0.20918968
H	1.36375485	-1.89017845	0.33473365
C	4.17382499	-0.83156121	0.09741973
H	4.65845783	-0.66206139	-0.86140418
H	4.79805536	-0.44102550	0.89734400
H	4.11285530	-1.90690236	0.23654113
H	-0.98572893	-2.04294618	0.81292285

Time = 86.50 fs
E(CASSCF/6-31G(d)) = -479.5603092

C	-0.86733632	-0.17354746	-0.25954746
C	0.50554351	0.13296148	-0.12175413
C	1.19206795	1.41324194	-0.26702880
C	1.56165910	-0.85427397	0.22103711
C	2.79801468	-0.23697288	0.13953841
C	2.55480971	1.20376702	-0.11937161
N	-1.88321726	0.61042521	0.12739814
C	-3.17943203	-0.06925370	0.17192157
H	3.31094763	1.95769915	-0.23185069
C	-1.34125903	-1.62910077	-0.12671602
C	-2.88258590	-1.53346264	-0.22930890
H	-3.21393693	-1.72625079	-1.23992648
H	-3.37279853	-2.24103590	0.42841432
H	-3.61484254	-0.03643836	1.17210319
H	-3.91072092	0.39015206	-0.49548810
H	-0.94152094	-2.24246302	-0.92400544
H	0.73482271	2.35615409	-0.50853857
C	-1.88585073	2.06139099	0.03060879
H	-1.23176522	2.50462971	0.76432273
H	-1.59080467	2.37808403	-0.96065876
H	-2.87088780	2.45979871	0.21641962
H	1.35297110	-1.88677484	0.33645641
C	4.17260277	-0.83047010	0.09786850
H	4.65205796	-0.66412318	-0.86412243
H	4.79502447	-0.44067698	0.89963821
H	4.11380253	-1.90608214	0.23836743
H	-0.98178695	-2.04221544	0.80818628

Time = 87.00 fs
E(CASSCF/6-31G(d)) = -479.5599811

C	-0.87125652	-0.17197132	-0.25921242
C	0.50585948	0.13252438	-0.12194610
C	1.19330622	1.41268938	-0.26911389

C	1.56171658	-0.85349935	0.22311682
C	2.79686701	-0.23725510	0.14139976
C	2.55495109	1.20393911	-0.11994874
N	-1.88087040	0.60743559	0.12601241
C	-3.17915563	-0.06925022	0.17287300
H	3.31168667	1.96041208	-0.23380089
C	-1.34027190	-1.62851520	-0.12976402
C	-2.88218444	-1.53264403	-0.22850031
H	-3.21664921	-1.72423795	-1.23881010
H	-3.37393497	-2.23915608	0.42862744
H	-3.60948245	-0.04138278	1.17457239
H	-3.91279829	0.38391706	-0.49105833
H	-0.94032174	-2.24242618	-0.92913507
H	0.72704792	2.35075529	-0.51378223
C	-1.88437086	2.06248091	0.03158202
H	-1.22556087	2.49979354	0.76332722
H	-1.59397005	2.37687624	-0.96162835
H	-2.86844427	2.45876622	0.22377305
H	1.34252764	-1.88471223	0.33843347
C	4.17130378	-0.82949788	0.09835898
H	4.64597114	-0.66590761	-0.86652695
H	4.79235643	-0.44012691	0.90154216
H	4.11501115	-1.90541562	0.24023874
H	-0.97805814	-2.04134685	0.80389174

Time = 87.50 fs
E(CASSCF/6-31G(d)) = -479.5599764

C	-0.87571226	-0.17089012	-0.25883611
C	0.50716406	0.13234658	-0.12201186
C	1.19424239	1.41194148	-0.27127462
C	1.56126475	-0.85252801	0.22510119
C	2.79583679	-0.23704019	0.14318940
C	2.55524086	1.20382711	-0.12046336
N	-1.87866715	0.60459468	0.12458826
C	-3.17893635	-0.06942838	0.17388103
H	3.31249566	1.96281244	-0.23558969
C	-1.33933686	-1.62779396	-0.13304382
C	-2.88167815	-1.53177262	-0.22761451
H	-3.21923686	-1.72224135	-1.23782240
H	-3.37523349	-2.23714851	0.42873516
H	-3.60343369	-0.04561007	1.17689917
H	-3.91401590	0.37820872	-0.48659773
H	-0.93873955	-2.24230426	-0.93402153
H	0.71960087	2.34508802	-0.51898898
C	-1.88286609	2.06360483	0.03261009
H	-1.21890146	2.49481961	0.76219378

H	-1.59685875	2.37563312	-0.96267969
H	-2.86653407	2.45703316	0.23124942
H	1.33257807	-1.88394094	0.34067861
C	4.16992773	-0.82865344	0.09889311
H	4.64025751	-0.66737436	-0.86856977
H	4.79009279	-0.43934348	0.90298553
H	4.11644693	-1.90488346	0.24215156
H	-0.97454842	-2.04036766	0.80006639

Time = 88.00 fs
E(CASSCF/6-31G(d)) = -479.5603266

C	-0.88061220	-0.17033652	-0.25844692
C	0.50942938	0.13242715	-0.12195470
C	1.19486414	1.41099577	-0.27351225
C	1.56028512	-0.85138012	0.22699316
C	2.79493079	-0.23633296	0.14490835
C	2.55568594	1.20344994	-0.12091812
N	-1.87666189	0.60195777	0.12314643
C	-3.17875023	-0.06980702	0.17495814
H	3.31326134	1.96473890	-0.23719472
C	-1.33845861	-1.62694647	-0.13655542
C	-2.88107246	-1.53085602	-0.22666373
H	-3.22166426	-1.72026844	-1.23692711
H	-3.37666723	-2.23505821	0.42876867
H	-3.59676101	-0.04906122	1.17915075
H	-3.91451780	0.37321594	-0.48225166
H	-0.93683670	-2.24199348	-0.93857617
H	0.71260440	2.33920520	-0.52415222
C	-1.88133308	2.06473332	0.03369039
H	-1.21178651	2.48978448	0.76093048
H	-1.59949142	2.37440350	-0.96378564
H	-2.86514647	2.45463293	0.23884370
H	1.32328650	-1.88428511	0.34318892
C	4.16847573	-0.82794368	0.09947249
H	4.63497031	-0.66849134	-0.87021068
H	4.78826729	-0.43830053	0.90390718
H	4.11807032	-1.90446337	0.24410231
H	-0.97127418	-2.03929082	0.79669795

Time = 88.50 fs
E(CASSCF/6-31G(d)) = -479.5609809

C	-0.88585344	-0.17033642	-0.25807402
C	0.51261070	0.13275941	-0.12177949
C	1.19516630	1.40985059	-0.27582688
C	1.55876722	-0.85008235	0.22879713

C	2.79415260	-0.23514567	0.14655925
C	2.55628953	1.20282953	-0.12131664
N	-1.87490811	0.59957676	0.12170820
C	-3.17857160	-0.07040071	0.17611517
H	3.31388892	1.96605111	-0.23859803
C	-1.33764160	-1.62598415	-0.14029196
C	-2.88037460	-1.52990282	-0.22566202
H	-3.22389593	-1.71832248	-1.23607742
H	-3.37820520	-2.23293249	0.42875978
H	-3.58954979	-0.05169100	1.18141347
H	-3.91446673	0.36911175	-0.47816444
H	-0.93467107	-2.24140571	-0.94273084
H	0.70616055	2.33317631	-0.52927156
C	-1.87976969	2.06583841	0.03481976
H	-1.20423267	2.48476175	0.75954143
H	-1.60189889	2.37323477	-0.96490679
H	-2.86423759	2.45160552	0.24654582
H	1.31482208	-1.88546883	0.34594687
C	4.16695032	-0.82737326	0.10009831
H	4.63015474	-0.66923518	-0.87141841
H	4.78690550	-0.43697788	0.90425676
H	4.11983701	-1.90413132	0.24608756
H	-0.96826199	-2.03811625	0.79373656

Time = 89.00 fs
E(CASSCF/6-31G(d)) = -479.5618980

C	-0.89132442	-0.17090803	-0.25774687
C	0.51664768	0.13333117	-0.12149271
C	1.19515133	1.40850541	-0.27821732
C	1.55671010	-0.84866476	0.23051875
C	2.79350216	-0.23349798	0.14814628
C	2.55705013	1.20198958	-0.12166342
N	-1.87345701	0.59749909	0.12029478
C	-3.17837487	-0.07121869	0.17736070
H	3.31431153	1.96664031	-0.23978772
C	-1.33689013	-1.62491998	-0.14423969
C	-2.87959341	-1.52892255	-0.22462459
H	-3.22589868	-1.71640345	-1.23522112
H	-3.37981169	-2.23081863	0.42873884
H	-3.58190274	-0.05346906	1.18378490
H	-3.91402788	0.36604267	-0.47446498
H	-0.93229322	-2.24047386	-0.94644411
H	0.70034827	2.32708667	-0.53435297
C	-1.87817502	2.06689391	0.03599489
H	-1.19627567	2.47981972	0.75802507
H	-1.60412037	2.37217231	-0.96599393

H	-2.86373218	2.44799728	0.25434026
H	1.30734941	-1.88715171	0.34892421
C	4.16535536	-0.82694453	0.10077138
H	4.62584742	-0.66959166	-0.87217198
H	4.78602475	-0.43536150	0.90399660
H	4.12169880	-1.90386374	0.24810420
H	-0.96554718	-2.03683322	0.79109991

Time = 89.50 fs
E(CASSCF/6-31G(d)) = -479.5630403

C	-0.89690844	-0.17206127	-0.25749474
C	0.52146524	0.13412526	-0.12110210
C	1.19482934	1.40696155	-0.28068126
C	1.55412375	-0.84715810	0.23216456
C	2.79297533	-0.23141666	0.14967508
C	2.55796109	1.20095478	-0.12196368
N	-1.87235567	0.59576666	0.11892674
C	-3.17813661	-0.07226389	0.17870051
H	3.31449803	1.96643843	-0.24075930
C	-1.33620870	-1.62376788	-0.14837837
C	-2.87873906	-1.52792568	-0.22356705
H	-3.22764397	-1.71450908	-1.23430660
H	-3.38144570	-2.22876197	0.42873294
H	-3.57393470	-0.05438025	1.18636551
H	-3.91335294	0.36411875	-0.47125186
H	-0.92974356	-2.23915618	-0.94970575
H	0.69522179	2.32103405	-0.53940861
C	-1.87654932	2.06787578	0.03721268
H	-1.18796982	2.47501874	0.75637334
H	-1.60620185	2.37125863	-0.96699175
H	-2.86352699	2.44386150	0.26220703
H	1.30101665	-1.88896851	0.35208605
C	4.16369616	-0.82665740	0.10149209
H	4.62207612	-0.66955635	-0.87246171
H	4.78563476	-0.43344393	0.90310270
H	4.12360495	-1.90363914	0.25015004
H	-0.96317056	-2.03542342	0.78868100

Time = 90.00 fs
E(CASSCF/6-31G(d)) = -479.5643318

C	-0.90248737	-0.17379641	-0.25734589
C	0.52697612	0.13512018	-0.12061652
C	1.19421814	1.40522248	-0.28321551
C	1.55103036	-0.84559076	0.23374170
C	2.79256395	-0.22893502	0.15115262

C	2.55901004	1.19974966	-0.12222309
N	-1.87164541	0.59441440	0.11762327
C	-3.17783752	-0.07353221	0.18013744
H	3.31445531	1.96542164	-0.24151626
C	-1.33560210	-1.62254258	-0.15268204
C	-2.87782286	-1.52692362	-0.22250468
H	-3.22911029	-1.71263558	-1.23328779
H	-3.38306095	-2.22680362	0.42876388
H	-3.56576845	-0.05442524	1.18924738
H	-3.91256495	0.36340436	-0.46858092
H	-0.92705218	-2.23743852	-0.95253861
H	0.69081240	2.31512394	-0.54445624
C	-1.87489385	2.06876247	0.03847058
H	-1.17938719	2.47041092	0.75457180
H	-1.60819406	2.37053390	-0.96784325
H	-2.86349851	2.43925948	0.27012238
H	1.29594561	-1.89056685	0.35539476
C	4.16197910	-0.82650927	0.10226047
H	4.61885845	-0.66913422	-0.87228965
H	4.78573698	-0.43122397	0.90156555
H	4.12550288	-1.90343944	0.25222384
H	-0.96117537	-2.03386675	0.78635836

Time = 90.50 fs
E(CASSCF/6-31G(d)) = -479.5657429

C	-0.90794546	-0.17610292	-0.25732668
C	0.53308119	0.13629041	-0.12004601
C	1.19334273	1.40329517	-0.28581609
C	1.54746495	-0.84398710	0.23525794
C	2.79225609	-0.22609274	0.15258702
C	2.56017937	1.19839847	-0.12244787
N	-1.87135990	0.59346901	0.11640132
C	-3.17746356	-0.07501199	0.18167136
H	3.31422961	1.96361263	-0.24207022
C	-1.33507570	-1.62125918	-0.15712025
C	-2.87685699	-1.52592847	-0.22145195
H	-3.23028466	-1.71077885	-1.23212943
H	-3.38460594	-2.22497984	0.42884780
H	-3.55752936	-0.05362073	1.19250547
H	-3.91174753	0.36391276	-0.46645732
H	-0.92423818	-2.23533400	-0.95499723
H	0.68712955	2.30946472	-0.54951700
C	-1.87321060	2.06953454	0.03976668
H	-1.17061504	2.46603911	0.75260155
H	-1.61014979	2.37003518	-0.96849352
H	-2.86351029	2.43426170	0.27806027

H	1.29222212	-1.89163667	0.35881399
C	4.16021178	-0.82649502	0.10307606
H	4.61620238	-0.66834025	-0.87166920
H	4.78632512	-0.42870720	0.89939061
H	4.12733963	-1.90325139	0.25432564
H	-0.95960251	-2.03214441	0.78400673

Time = 91.00 fs
E(CASSCF/6-31G(d)) = -479.5671790

C	-0.91317242	-0.17895831	-0.25746076
C	0.53967218	0.13760708	-0.11940170
C	1.19223414	1.40119008	-0.28847838
C	1.54347528	-0.84236545	0.23672135
C	2.79203624	-0.22293528	0.15398745
C	2.56144670	1.19692480	-0.12264461
N	-1.87152354	0.59294776	0.11527493
C	-3.17700713	-0.07668406	0.18329970
H	3.31390329	1.96107862	-0.24244102
C	-1.33463563	-1.61993323	-0.16165938
C	-2.87585412	-1.52495286	-0.22042192
H	-3.23116421	-1.70893527	-1.23081043
H	-3.38602464	-2.22332096	0.42899417
H	-3.54934147	-0.05199985	1.19618730
H	-3.91093918	0.36560274	-0.46483165
H	-0.92131107	-2.23288126	-0.95716471
H	0.68416466	2.30416040	-0.55461450
C	-1.87150210	2.07017496	0.04109985
H	-1.16175232	2.46193803	0.75044167
H	-1.61212204	2.36979742	-0.96889429
H	-2.86342225	2.42894928	0.28599374
H	1.28988980	-1.89193396	0.36231170
C	4.15840273	-0.82660698	0.10393796
H	4.61410574	-0.66719899	-0.87062546
H	4.78738553	-0.42590582	0.89659870
H	4.12906278	-1.90306780	0.25645674
H	-0.95848666	-2.03024421	0.78150730

Time = 91.50 fs
E(CASSCF/6-31G(d)) = -479.5685972

C	-0.91806748	-0.18232733	-0.25776829
C	0.54663296	0.13903878	-0.11869563
C	1.19092898	1.39892253	-0.29119764
C	1.53912160	-0.84073794	0.23814033
C	2.79188610	-0.21951313	0.15536394
C	2.56278573	1.19535169	-0.12282026

N	-1.87214991	0.59285744	0.11425454
C	-3.17646719	-0.07852185	0.18501811
H	3.31358954	1.95792679	-0.24265554
C	-1.33428870	-1.61858067	-0.16626409
C	-2.87482719	-1.52400976	-0.21942597
H	-3.23175715	-1.70710227	-1.22932675
H	-3.38725763	-2.22185152	0.42920632
H	-3.54132258	-0.04961158	1.20030664
H	-3.91013358	0.36838052	-0.46360256
H	-0.91827281	-2.23014098	-0.95914701
H	0.68189440	2.29930452	-0.55977238
C	-1.86977121	2.07066871	0.04246961
H	-1.15290484	2.45813389	0.74807209
H	-1.61416199	2.36985242	-0.96900712
H	-2.86309994	2.42341440	0.29389718
H	1.28894615	-1.89129875	0.36586279
C	4.15656149	-0.82683516	0.10484480
H	4.61255584	-0.66574454	-0.86919327
H	4.78889674	-0.42283893	0.89322467
H	4.13062186	-1.90288712	0.25861982
H	-0.95785245	-2.02816392	0.77875733

Time = 92.00 fs
E(CASSCF/6-31G(d)) = -479.5698972

C	-0.92254159	-0.18616132	-0.25826516
C	0.55384151	0.14055208	-0.11794082
C	1.18946823	1.39651305	-0.29396930
C	1.53447565	-0.83911005	0.23952370
C	2.79178531	-0.21588081	0.15672712
C	2.56416729	1.19370164	-0.12298204
N	-1.87324068	0.59319365	0.11334645
C	-3.17584921	-0.08049218	0.18682150
H	3.31342353	1.95429480	-0.24274603
C	-1.33404232	-1.61721819	-0.17089894
C	-2.87378907	-1.52311224	-0.21847365
H	-3.23208300	-1.70527872	-1.22769205
H	-3.38824347	-2.22059048	0.42948166
H	-3.53358123	-0.04652080	1.20483923
H	-3.90928635	0.37210456	-0.46262478
H	-0.91512112	-2.22719050	-0.96106515
H	0.68028475	2.29497328	-0.56501253
C	-1.86802096	2.07100297	0.04387596
H	-1.14417952	2.45464579	0.74547700
H	-1.61631758	2.37022909	-0.96880622
H	-2.86242361	2.41776107	0.30174851
H	1.28934154	-1.88966419	0.36945081

C	4.15469823	-0.82716728	0.10579479
H	4.61152905	-0.66401974	-0.86741608
H	4.79082938	-0.41953210	0.88931646
H	4.13196935	-1.90271313	0.26081901
H	-0.95771155	-2.02591434	0.77567730

Time = 92.50 fs
E(CASSCF/6-31G(d)) = -479.5709983

C	-0.92652044	-0.19039830	-0.25896264
C	0.56117199	0.14211248	-0.11715084
C	1.18789608	1.39398796	-0.29678940
C	1.52961890	-0.83748123	0.24088045
C	2.79171237	-0.21209622	0.15808831
C	2.56556081	1.19199752	-0.12313753
N	-1.87478500	0.59394029	0.11255256
C	-3.17516396	-0.08255624	0.18870508
H	3.31355061	1.95034149	-0.24274885
C	-1.33390421	-1.61586326	-0.17552992
C	-2.87275231	-1.52227309	-0.21757265
H	-3.23217280	-1.70346542	-1.22593811
H	-3.38892109	-2.21955276	0.42981344
H	-3.52621473	-0.04280753	1.20972224
H	-3.90832617	0.37659469	-0.46172197
H	-0.91185306	-2.22411751	-0.96304636
H	0.67929741	2.29121925	-0.57035249
C	-1.86625446	2.07116678	0.04531913
H	-1.13567920	2.45148648	0.74264819
H	-1.61863208	2.37095300	-0.96828032
H	-2.86129495	2.41210441	0.30953069
H	1.29098258	-1.88705984	0.37306934
C	4.15282396	-0.82758921	0.10678566
H	4.61099137	-0.66207627	-0.86534417
H	4.79314671	-0.41601782	0.88493385
H	4.13306168	-1.90255393	0.26305954
H	-0.95806022	-2.02352151	0.77221750

Time = 93.00 fs
E(CASSCF/6-31G(d)) = -479.5718111

C	-0.92994555	-0.19496355	-0.25986712
C	0.56849669	0.14368497	-0.11633980
C	1.18625869	1.39137944	-0.29965481
C	1.52464054	-0.83584561	0.24221985
C	2.79164559	-0.20821942	0.15945924
C	2.56693588	1.19026304	-0.12329472
N	-1.87675948	0.59506977	0.11187027

C	-3.17442627	-0.08467089	0.19066527
H	3.31411316	1.94623342	-0.24270218
C	-1.33388204	-1.61453445	-0.18012584
C	-2.87172891	-1.52150453	-0.21672896
H	-3.23206803	-1.70166487	-1.22411244
H	-3.38923170	-2.21875021	0.43019201
H	-3.51930782	-0.03856607	1.21485760
H	-3.90716882	0.38164302	-0.46070182
H	-0.90846954	-2.22101266	-0.96521373
H	0.67889300	2.28806795	-0.57580415
C	-1.86447485	2.07115117	0.04679936
H	-1.12749749	2.44866317	0.73958794
H	-1.62114330	2.37204570	-0.96743291
H	-2.85964304	2.40656927	0.31723340
H	1.29373669	-1.88360856	0.37672252
C	4.15095022	-0.82808525	0.10781473
H	4.61089866	-0.65997374	-0.86303295
H	4.79580483	-0.41233516	0.88014695
H	4.13386005	-1.90242083	0.26534763
H	-0.95887840	-2.02102723	0.76836068

Time = 93.50 fs
E(CASSCF/6-31G(d)) = -479.5722416

C	-0.93277403	-0.19977070	-0.26097997
C	0.57568799	0.14523467	-0.11552233
C	1.18460321	1.38872520	-0.30256352
C	1.51963526	-0.83419315	0.24355159
C	2.79156389	-0.20431152	0.16085174
C	2.56826357	1.18852322	-0.12346192
N	-1.87912880	0.59654370	0.11129261
C	-3.17365379	-0.08679035	0.19270048
H	3.31523699	1.94212897	-0.24264308
C	-1.33398299	-1.61325206	-0.18465960
C	-2.87073014	-1.52081792	-0.21594711
H	-3.23181911	-1.69988119	-1.22227500
H	-3.38912104	-2.21819310	0.43060610
H	-3.51293300	-0.03390391	1.22011935
H	-3.90573259	0.38702616	-0.45937267
H	-0.90497998	-2.21796203	-0.96767636
H	0.67903348	2.28551506	-0.58137248
C	-1.86268540	2.07094945	0.04831653
H	-1.11971313	2.44617889	0.73631093
H	-1.62388306	2.37352448	-0.96628209
H	-2.85742827	2.40128892	0.32485438
H	1.29743822	-1.87951570	0.38042436
C	4.14908883	-0.82863839	0.10887890

H	4.61119651	-0.65777837	-0.86054076
H	4.79875257	-0.40852883	0.87503394
H	4.13433143	-1.90232626	0.26769023
H	-0.96012955	-2.01848909	0.76412260

Time = 94.00 fs
E(CASSCF/6-31G(d)) = -479.5722406

C	-0.93497856	-0.20472366	-0.26229778
C	0.58262042	0.14672753	-0.11471336
C	1.18297668	1.38606785	-0.30551477
C	1.51470054	-0.83251127	0.24488576
C	2.79144772	-0.20043349	0.16227756
C	2.56951799	1.18680504	-0.12364779
N	-1.88184683	0.59831399	0.11080867
C	-3.17286538	-0.08886773	0.19481141
H	3.31701776	1.93816531	-0.24260542
C	-1.33421327	-1.61203808	-0.18910908
C	-2.86976646	-1.52022345	-0.21523057
H	-3.23148339	-1.69811960	-1.22049362
H	-3.38854158	-2.21789151	0.43104452
H	-3.50715098	-0.02893972	1.22536342
H	-3.90395192	0.39251787	-0.45755862
H	-0.90140641	-2.21504030	-0.97052025
H	0.67968422	2.28352739	-0.58705527
C	-1.86088959	2.07055730	0.04986998
H	-1.11238673	2.44403268	0.73284544
H	-1.62687701	2.37540128	-0.96485866
H	-2.85464338	2.39640178	0.33239990
H	1.30189770	-1.87505253	0.38419751
C	4.14725175	-0.82923082	0.10997476
H	4.61182135	-0.65556203	-0.85792730
H	4.80193236	-0.40464877	0.86967946
H	4.13444903	-1.90228214	0.27009470
H	-0.96176175	-2.01597887	0.75955041

Time = 94.50 fs
E(CASSCF/6-31G(d)) = -479.5717833

C	-0.93654619	-0.20971900	-0.26381271
C	0.58917221	0.14813096	-0.11392801
C	1.18142530	1.38345394	-0.30850908
C	1.50993426	-0.83078689	0.24623297
C	2.79127960	-0.19664524	0.16374824
C	2.57067742	1.18513794	-0.12386140
N	-1.88485820	0.60032440	0.11040415
C	-3.17207971	-0.09085679	0.19700117

H	3.31951013	1.93444582	-0.24261759
C	-1.33457759	-1.61091635	-0.19345767
C	-2.86884737	-1.51972984	-0.21458221
H	-3.23112269	-1.69638583	-1.21883867
H	-3.38745451	-2.21785644	0.43149742
H	-3.50201174	-0.02380089	1.23043939
H	-3.90178890	0.39790078	-0.45511283
H	-0.89778675	-2.21230510	-0.97380133
H	0.68081375	2.28204584	-0.59284339
C	-1.85909124	2.06997305	0.05145828
H	-1.10555788	2.44221991	0.72923323
H	-1.63014462	2.37768174	-0.96320354
H	-2.85131236	2.39204802	0.33988460
H	1.30691143	-1.87053221	0.38807058
C	4.14545087	-0.82984435	0.11109857
H	4.61270125	-0.65340073	-0.85525175
H	4.80528087	-0.40074911	0.86417245
H	4.13419298	-1.90229783	0.27256836
H	-0.96370963	-2.01358005	0.75471850

Time = 95.00 fs
E(CASSCF/6-31G(d)) = -479.5708901

C	-0.93747741	-0.21464909	-0.26551326
C	0.59522808	0.14941478	-0.11318134
C	1.17999302	1.38093232	-0.31154803
C	1.50543148	-0.82900879	0.24760436
C	2.79104476	-0.19300397	0.16527482
C	2.57172549	1.18355357	-0.12411205
N	-1.88810018	0.60251254	0.11006211
C	-3.17131402	-0.09271346	0.19927488
H	3.32271935	1.93103352	-0.24270157
C	-1.33507870	-1.60991205	-0.19769449
C	-2.86798151	-1.51934410	-0.21400469
H	-3.23080079	-1.69468559	-1.21737711
H	-3.38583138	-2.21810077	0.43195761
H	-3.49755578	-0.01862037	1.23520287
H	-3.89924174	0.40297700	-0.45192760
H	-0.89417631	-2.20979401	-0.97754110
H	0.68239422	2.28099129	-0.59872215
C	-1.85729471	2.06919787	0.05307911
H	-1.09924436	2.44073190	0.72552838
H	-1.63369958	2.38036423	-0.96136407
H	-2.84748640	2.38836494	0.34733078
H	1.31227385	-1.86627983	0.39207522
C	4.14369781	-0.83046096	0.11224645
H	4.61375787	-0.65137349	-0.85257194

H	4.80873077	-0.39688737	0.85860500
H	4.13355012	-1.90237879	0.27511829
H	-0.96589722	-2.01138452	0.74972276

Time = 95.50 fs
E(CASSCF/6-31G(d)) = -479.5696188

C	-0.93778436	-0.21940522	-0.26738480
C	0.60068098	0.15055189	-0.11248818
C	1.17872084	1.37855239	-0.31463418
C	1.50128188	-0.82717010	0.24901169
C	2.79073145	-0.18956306	0.16686768
C	2.57265159	1.18208525	-0.12440918
N	-1.89150474	0.60481197	0.10976373
C	-3.17058318	-0.09439748	0.20163927
H	3.32659860	1.92794752	-0.24287220
C	-1.33571697	-1.60905119	-0.20181402
C	-2.86717668	-1.51907133	-0.21350091
H	-3.23058080	-1.69302389	-1.21616673
H	-3.38365527	-2.21863965	0.43242114
H	-3.49381518	-0.01353314	1.23952748
H	-3.89634996	0.40757677	-0.44794144
H	-0.89064776	-2.20752366	-0.98172597
H	0.68439937	2.28027170	-0.60467293
C	-1.85550514	2.06823607	0.05472926
H	-1.09344208	2.43955545	0.72179500
H	-1.63755024	2.38343895	-0.95939020
H	-2.84323812	2.38548221	0.35476693
H	1.31778799	-1.86259830	0.39624167
C	4.14200372	-0.83106335	0.11341443
H	4.61490801	-0.64956067	-0.84994329
H	4.81221224	-0.39312307	0.85307051
H	4.13251421	-1.90252507	0.27775099
H	-0.96824142	-2.00948835	0.74467276

Time = 96.00 fs
E(CASSCF/6-31G(d)) = -479.5680671

C	-0.93748885	-0.22388079	-0.26941015
C	0.60543367	0.15151894	-0.11186304
C	1.17764630	1.37636206	-0.31777067
C	1.49756774	-0.82527048	0.25046744
C	2.79033114	-0.18637120	0.16853628
C	2.57345076	1.18076724	-0.12476209
N	-1.89500063	0.60715432	0.10948911
C	-3.16989910	-0.09587364	0.20410196
H	3.33105194	1.92516454	-0.24313710

C	-1.33649018	-1.60835986	-0.20581546
C	-2.86643992	-1.51891470	-0.21307435
H	-3.23052236	-1.69140447	-1.21525086
H	-3.38092157	-2.21949018	0.43288738
H	-3.49081428	-0.00867243	1.24331500
H	-3.89319629	0.41156502	-0.44314296
H	-0.88728944	-2.20549096	-0.98630972
H	0.68680159	2.27979030	-0.61067526
C	-1.85372864	2.06709551	0.05640474
H	-1.08812665	2.43867230	0.71810397
H	-1.64169983	2.38688682	-0.95733049
H	-2.83865412	2.38351695	0.36222539
H	1.32327413	-1.85973414	0.40059365
C	4.14037894	-0.83163529	0.11459855
H	4.61606548	-0.64804188	-0.84741789
H	4.81565444	-0.38951610	0.84766203
H	4.13108598	-1.90273026	0.28047202
H	-0.97065625	-2.00798701	0.73968242

Time = 96.50 fs
E(CASSCF/6-31G(d)) = -479.5663654

C	-0.93662077	-0.22797451	-0.27157006
C	0.60940045	0.15229694	-0.11131986
C	1.17680281	1.37440578	-0.32096094
C	1.49436187	-0.82331786	0.25198482
C	2.78983845	-0.18347128	0.17028897
C	2.57412327	1.17963333	-0.12517975
N	-1.89851536	0.60947130	0.10921794
C	-3.16927028	-0.09711299	0.20667077
H	3.33594171	1.92262545	-0.24349761
C	-1.33739323	-1.60786311	-0.20970157
C	-2.86577763	-1.51887533	-0.21272927
H	-3.23067933	-1.68982936	-1.21465425
H	-3.37763793	-2.22067086	0.43335862
H	-3.48857030	-0.00416590	1.24650359
H	-3.88990536	0.41484571	-0.43757164
H	-0.88420163	-2.20367655	-0.99121903
H	0.68956915	2.27945470	-0.61670966
C	-1.85197251	2.06578777	0.05810100
H	-1.08325602	2.43805823	0.71452893
H	-1.64614692	2.39067882	-0.95522843
H	-2.83382638	2.38256853	0.36973977
H	1.32857674	-1.85784868	0.40514396
C	4.13883278	-0.83216210	0.11579501
H	4.61714306	-0.64689416	-0.84504393
H	4.81898741	-0.38612509	0.84247059

H	4.12927298	-1.90298099	0.28328582
H	-0.97305719	-2.00696985	0.73485974

Time = 97.00 fs
E(CASSCF/6-31G(d)) = -479.5646580

C	-0.93521667	-0.23159339	-0.27384382
C	0.61250885	0.15287189	-0.11087186
C	1.17621900	1.37272237	-0.32420828
C	1.49172594	-0.82132926	0.25357754
C	2.78925116	-0.18089940	0.17213284
C	2.57467385	1.17871514	-0.12567039
N	-1.90197706	0.61169671	0.10893023
C	-3.16870169	-0.09809370	0.20935298
H	3.34109986	1.92024637	-0.24395050
C	-1.33841818	-1.60758376	-0.21347749
C	-2.86519567	-1.51895238	-0.21247076
H	-3.23109813	-1.68829866	-1.21438033
H	-3.37382392	-2.22220022	0.43383935
H	-3.48709313	-0.00013176	1.24907311
H	-3.88663943	0.41736392	-0.43131530
H	-0.88149174	-2.20205021	-0.99636124
H	0.69266439	2.27918477	-0.62275971
C	-1.85024536	2.06432839	0.05981332
H	-1.07877448	2.43768191	0.71114170
H	-1.65088575	2.39477548	-0.95311901
H	-2.82884298	2.38271339	0.37734193
H	1.33356908	-1.85699795	0.40989111
C	4.13737335	-0.83263101	0.11700028
H	4.61805499	-0.64619014	-0.84286564
H	4.82214435	-0.38300577	0.83758408
H	4.12708909	-1.90325736	0.28619569
H	-0.97536560	-2.00651521	0.73029731

Time = 97.50 fs
E(CASSCF/6-31G(d)) = -479.5630918

C	-0.93331846	-0.23465533	-0.27620964
C	0.61470109	0.15323507	-0.11053131
C	1.17591821	1.37134331	-0.32751554
C	1.48970918	-0.81933032	0.25525958
C	2.78857001	-0.17868410	0.17407348
C	2.57511063	1.17804037	-0.12624116
N	-1.90531615	0.61376815	0.10860681
C	-3.16819490	-0.09880155	0.21215457
H	3.34634176	1.91793150	-0.24448980
C	-1.33955422	-1.60754113	-0.21714936

C	-2.86469953	-1.51914314	-0.21230464
H	-3.23181654	-1.68681058	-1.21441039
H	-3.36951013	-2.22409522	0.43433504
H	-3.48638511	0.00332466	1.25104747
H	-3.88359123	0.41910534	-0.42450478
H	-0.87926847	-2.20057692	-1.00163365
H	0.69604209	2.27891950	-0.62881421
C	-1.84855722	2.06273692	0.06153718
H	-1.07461716	2.43750400	0.70800756
H	-1.65590641	2.39912679	-0.95102635
H	-2.82377949	2.38400051	0.38505892
H	1.33815463	-1.85712628	0.41481765
C	4.13600725	-0.83303147	0.11821120
H	4.61871923	-0.64599620	-0.84092311
H	4.82506376	-0.38020934	0.83308583
H	4.12455411	-1.90353382	0.28920373
H	-0.97751274	-2.00668557	0.72606329

Time = 98.00 fs
E(CASSCF/6-31G(d)) = -479.5618014

C	-0.93097241	-0.23709132	-0.27864511
C	0.61593534	0.15338327	-0.11030929
C	1.17591810	1.37029126	-0.33088476
C	1.48834757	-0.81735367	0.25704458
C	2.78779849	-0.17684566	0.17611488
C	2.57544398	1.17763111	-0.12689785
N	-1.90846676	0.61562851	0.10822985
C	-3.16774851	-0.09923017	0.21507958
H	3.35148092	1.91558646	-0.24510889
C	-1.34078781	-1.60774993	-0.22072307
C	-2.86429432	-1.51944320	-0.21223717
H	-3.23286327	-1.68536171	-1.21470467
H	-3.36473685	-2.22636926	0.43485066
H	-3.48644030	0.00611383	1.25249393
H	-3.88097389	0.42009310	-0.41730552
H	-0.87763587	-2.19922305	-1.00693300
H	0.69964889	2.27862177	-0.63486861
C	-1.84691943	2.06103683	0.06326876
H	-1.07071504	2.43747631	0.70518082
H	-1.66119490	2.40367263	-0.94896230
H	-2.81869195	2.38644760	0.39291028
H	1.34226603	-1.85807419	0.41989064
C	4.13473945	-0.83335536	0.11942509
H	4.61905972	-0.64637079	-0.83925220
H	4.82769137	-0.37778089	0.82905332
H	4.12169314	-1.90378063	0.29231103

H	-0.97944305	-2.00752377	0.72219465

Time = 98.50 fs
E(CASSCF/6-31G(d)) = -479.5608993

C	-0.92822834	-0.23884700	-0.28112748
C	0.61618666	0.15331868	-0.11021546
C	1.17623033	1.36957905	-0.33431696
C	1.48766322	-0.81543620	0.25894528
C	2.78694260	-0.17539580	0.17825930
C	2.57568530	1.17750240	-0.12764452
N	-1.91136798	0.61722711	0.10778323
C	-3.16735884	-0.09938080	0.21812940
H	3.35634310	1.91313073	-0.24580237
C	-1.34210298	-1.60821916	-0.22420323
C	-2.86398487	-1.51984668	-0.21227460
H	-3.23425806	-1.68394752	-1.21520525
H	-3.35955252	-2.22903003	0.43538914
H	-3.48724343	0.00816429	1.25351920
H	-3.87900822	0.42038186	-0.40990645
H	-0.87668763	-2.19796198	-1.01216458
H	0.70342373	2.27828082	-0.64092587
C	-1.84534444	2.05925531	0.06500540
H	-1.06699969	2.43754137	0.70270092
H	-1.66673308	2.40834360	-0.94692638
H	-2.81361198	2.39003845	0.40090582
H	1.34586233	-1.85959909	0.42506434
C	4.13357312	-0.83359711	0.12063985
H	4.61900842	-0.64736279	-0.83788441
H	4.82998196	-0.37575807	0.82555683
H	4.11853591	-1.90396586	0.29551785
H	-0.98111645	-2.00905046	0.71869297

Time = 99.00 fs
E(CASSCF/6-31G(d)) = -479.5604690

C	-0.92513894	-0.23988380	-0.28363412
C	0.61544772	0.15304863	-0.11025778
C	1.17686030	1.36920930	-0.33781203
C	1.48766413	-0.81361577	0.26097273
C	2.78601061	-0.17433749	0.18050723
C	2.57584600	1.17766121	-0.12848338
N	-1.91396486	0.61852070	0.10725288
C	-3.16702079	-0.09926170	0.22130226
H	3.36077845	1.91050783	-0.24656763
C	-1.34348164	-1.60895146	-0.22759249
C	-2.86377571	-1.52034650	-0.21242265

H	-3.23601244	-1.68256304	-1.21584053
H	-3.35401189	-2.23207743	0.43594965
H	-3.48876884	0.00942402	1.25426271
H	-3.87790772	0.42004939	-0.40250637
H	-0.87650206	-2.19677869	-1.01725021
H	0.70729948	2.27791202	-0.64699644
C	-1.84384544	2.05742277	0.06674599
H	-1.06340740	2.43763264	0.70058956
H	-1.67249857	2.41306259	-0.94490715
H	-2.80854491	2.39472187	0.40904453
H	1.34892537	-1.86140485	0.43028445
C	4.13250954	-0.83375372	0.12185399
H	4.61850709	-0.64901031	-0.83684654
H	4.83190073	-0.37416992	0.82265802
H	4.11511598	-1.90405754	0.29882388
H	-0.98250935	-2.01126298	0.71552330

Time = 99.50 fs
E(CASSCF/6-31G(d)) = -479.5605619

C	-0.92175941	-0.24017945	-0.28614277
C	0.61372917	0.15258522	-0.11044230
C	1.17780717	1.36917450	-0.34136865
C	1.48834426	-0.81192774	0.26313558
C	2.78501286	-0.17366497	0.18285728
C	2.57593655	1.17810573	-0.12941459
N	-1.91620925	0.61947410	0.10662696
C	-3.16672911	-0.09888720	0.22459275
H	3.36467147	1.90769308	-0.24740602
C	-1.34490407	-1.60994253	-0.23089114
C	-2.86367094	-1.52093461	-0.21268591
H	-3.23813087	-1.68120353	-1.21653068
H	-3.34817452	-2.23550165	0.43652643
H	-3.49097885	0.00986129	1.25488693
H	-3.87786181	0.41918632	-0.39529860
H	-0.87713819	-2.19567317	-1.02213433
H	0.71120539	2.27755410	-0.65309740
C	-1.84243588	2.05557221	0.06849127
H	-1.05988282	2.43767514	0.69884892
H	-1.67846452	2.41774672	-0.94288386
H	-2.80347105	2.40041180	0.41731421
H	1.35145594	-1.86317545	0.43549356
C	4.13154807	-0.83382471	0.12306670
H	4.61750875	-0.65133951	-0.83616017
H	4.83342419	-0.37303607	0.82040861
H	4.11146999	-1.90402616	0.30222860
H	-0.98361445	-2.01413627	0.71261734

Time = 100.00 fs
E(CASSCF/6-31G(d)) = -479.5611993

C	-0.91814694	-0.23972810	-0.28863216
C	0.61105920	0.15194464	-0.11077285
C	1.17906396	1.36945797	-0.34498449
C	1.48968387	-0.81040189	0.26543949
C	2.78396163	-0.17336455	0.18530646
C	2.57596576	1.17882570	-0.13043641
N	-1.91806065	0.62006079	0.10589616
C	-3.16647946	-0.09827646	0.22799154
H	3.36794735	1.90469786	-0.24832346
C	-1.34634943	-1.61118099	-0.23409717
C	-2.86367424	-1.52160227	-0.21306725
H	-3.24061224	-1.67986527	-1.21719398
H	-3.34210301	-2.23928203	0.43710788
H	-3.49382215	0.00946428	1.25556574
H	-3.87901916	0.41788483	-0.38845539
H	-0.87863304	-2.19466180	-1.02678841
H	0.71507106	2.27726365	-0.65925053
C	-1.84112879	2.05373811	0.07024395
H	-1.05638057	2.43758704	0.69746152
H	-1.68459983	2.42231006	-0.94083019
H	-2.79834989	2.40698971	0.42569293
H	1.35347011	-1.86460808	0.44063685
C	4.13068625	-0.83381203	0.12427784
H	4.61597886	-0.65436403	-0.83584113
H	4.83454098	-0.37236625	0.81884888
H	4.10763688	-1.90384690	0.30573165
H	-0.98443926	-2.01762489	0.70987862

Time = 100.50 fs
E(CASSCF/6-31G(d)) = -479.5623335

C	-0.91436045	-0.23853985	-0.29108213
C	0.60748478	0.15114703	-0.11125094
C	1.18061710	1.37003443	-0.34865630
C	1.49164949	-0.80905998	0.26788658
C	2.78287078	-0.17341390	0.18784963
C	2.57594048	1.17980156	-0.13154474
N	-1.91948607	0.62026285	0.10505391
C	-3.16627010	-0.09745250	0.23148530
H	3.37057516	1.90157051	-0.24933082
C	-1.34779632	-1.61264864	-0.23720664
C	-2.86378865	-1.52234037	-0.21356727
H	-3.24345080	-1.67854647	-1.21775252

H	-3.33586216	-2.24338575	0.43767567
H	-3.49723304	0.00824035	1.25647109
H	-3.88147258	0.41622808	-0.38211356
H	-0.88099988	-2.19377800	-1.03121284
H	0.71883016	2.27710791	-0.66548089
C	-1.83993625	2.05195613	0.07200870
H	-1.05286668	2.43728264	0.69639153
H	-1.69086813	2.42666608	-0.93871727
H	-2.79312705	2.41430798	0.43414968
H	1.35499633	-1.86544079	0.44566641
C	4.12991966	-0.83371984	0.12548790
H	4.61389574	-0.65808410	-0.83589836
H	4.83525097	-0.37216025	0.81800657
H	4.10365667	-1.90350134	0.30933273
H	-0.98500344	-2.02166652	0.70719091

Time = 101.00 fs
E(CASSCF/6-31G(d)) = -479.5639229

C	-0.91046007	-0.23663997	-0.29347443
C	0.60306932	0.15021545	-0.11187556
C	1.18244722	1.37087220	-0.35238035
C	1.49419533	-0.80791409	0.27047535
C	2.78175589	-0.17378415	0.19048024
C	2.57586524	1.18100651	-0.13273383
N	-1.92046111	0.62007145	0.10409639
C	-3.16610239	-0.09644068	0.23505708
H	3.37256803	1.89839468	-0.25044346
C	-1.34922354	-1.61432091	-0.24021433
C	-2.86401646	-1.52313950	-0.21418388
H	-3.24663826	-1.67724745	-1.21813825
H	-3.32951739	-2.24776850	0.43820546
H	-3.50112898	0.00621449	1.25775939
H	-3.88524687	0.41428132	-0.37636262
H	-0.88422861	-2.19307007	-1.03543689
H	0.72242523	2.27715637	-0.67181297
C	-1.83886859	2.05026104	0.07379198
H	-1.04931699	2.43667467	0.69558682
H	-1.69722948	2.43073125	-0.93651824
H	-2.78774207	2.42219548	0.44264901
H	1.35607220	-1.86547573	0.45054635
C	4.12924231	-0.83355431	0.12669806
H	4.61125082	-0.66248711	-0.83633341
H	4.83556565	-0.37240805	0.81789522
H	4.09956983	-1.90297935	0.31303243
H	-0.98533567	-2.02618661	0.70442770

Time = 101.50 fs
E(CASSCF/6-31G(d)) = -479.5658561

C	-0.90650672	-0.23406776	-0.29579305
C	0.59789210	0.14917550	-0.11264315
C	1.18452922	1.37193479	-0.35615276
C	1.49726421	-0.80696592	0.27320055
C	2.78063408	-0.17444038	0.19319021
C	2.57574247	1.18240730	-0.13399625
N	-1.92097000	0.61948660	0.10302269
C	-3.16598161	-0.09526779	0.23868691
H	3.37397939	1.89528411	-0.25168042
C	-1.35061059	-1.61616764	-0.24311460
C	-2.86435906	-1.52399015	-0.21491206
H	-3.25016474	-1.67597117	-1.21829760
H	-3.32313442	-2.25237518	0.43866759
H	-3.50540986	0.00342752	1.25955853
H	-3.89029233	0.41208664	-0.37123798
H	-0.88828705	-2.19259820	-1.03951609
H	0.72581196	2.27747159	-0.67826798
C	-1.83793382	2.04868578	0.07560170
H	-1.04571574	2.43567824	0.69498269
H	-1.70364018	2.43442802	-0.93421180
H	-2.78213760	2.43046363	0.45115380
H	1.35674191	-1.86459385	0.45525561
C	4.12864679	-0.83332339	0.12791006
H	4.60804856	-0.66754760	-0.83713928
H	4.83550678	-0.37309044	0.81851327
H	4.09541640	-1.90227997	0.31683217
H	-0.98546998	-2.03110310	0.70146268

Time = 102.00 fs
E(CASSCF/6-31G(d)) = -479.5679960

C	-0.90256187	-0.23087526	-0.29802478
C	0.59204684	0.14805503	-0.11354766
C	1.18683287	1.37318292	-0.35996990
C	1.50078903	-0.80620699	0.27605356
C	2.77952384	-0.17534245	0.19597012
C	2.57557275	1.18396558	-0.13532322
N	-1.92100558	0.61851689	0.10183488
C	-3.16591705	-0.09396150	0.24235274
H	3.37489597	1.89237465	-0.25306331
C	-1.35193835	-1.61815415	-0.24590240
C	-2.86481682	-1.52488280	-0.21574378
H	-3.25401964	-1.67472341	-1.21819517
H	-3.31677898	-2.25714172	0.43902871

H	-3.50995769	-0.00006588	1.26195737
H	-3.89648487	0.40966221	-0.36671972
H	-0.89312345	-2.19242983	-1.04352753
H	0.72896308	2.27810077	-0.68486088
C	-1.83713713	2.04726018	0.07744674
H	-1.04205270	2.43421478	0.69450611
H	-1.71005354	2.43768778	-0.93178562
H	-2.77626824	2.43891384	0.45962906
H	1.35705394	-1.86276242	0.45978984
C	4.12812447	-0.83303654	0.12912618
H	4.60430577	-0.67322796	-0.83829959
H	4.83510508	-0.37417991	0.81984317
H	4.09123506	-1.90141168	0.32073429
H	-0.98544173	-2.03633141	0.69817975

Time = 102.50 fs
E(CASSCF/6-31G(d)) = -479.5701877

C	-0.89868716	-0.22712575	-0.30015973
C	0.58564008	0.14688364	-0.11458061
C	1.18932338	1.37457624	-0.36382871
C	1.50469438	-0.80561941	0.27902271
C	2.77844487	-0.17644622	0.19880948
C	2.57535542	1.18563984	-0.13670500
N	-1.92056942	0.61717918	0.10053802
C	-3.16592156	-0.09254992	0.24603161
H	3.37542762	1.88981501	-0.25461495
C	-1.35318951	-1.62024241	-0.24857435
C	-2.86538890	-1.52580790	-0.21666804
H	-3.25819236	-1.67351244	-1.21781542
H	-3.31051672	-2.26199746	0.43925390
H	-3.51463692	-0.00419893	1.26499807
H	-3.90363233	0.40700538	-0.36273650
H	-0.89867077	-2.19263381	-1.04756296
H	0.73187167	2.27906906	-0.69159762
C	-1.83648048	2.04600971	0.07933649
H	-1.03832043	2.43221562	0.69407990
H	-1.71642125	2.44045347	-0.92923843
H	-2.77010779	2.44734433	0.46804581
H	1.35705935	-1.86003656	0.46416210
C	4.12766591	-0.83270456	0.13034922
H	4.60005108	-0.67947959	-0.83978830
H	4.83439919	-0.37564186	0.82185108
H	4.08706236	-1.90039272	0.32474224
H	-0.98528439	-2.04178920	0.69448206

Time = 103.00 fs

E(CASSCF/6-31G(d)) = -479.5722586

C	-0.89494355	-0.22289212	-0.30219142
C	0.57878901	0.14569240	-0.11573131
C	1.19196216	1.37607514	-0.36772707
C	1.50889843	-0.80517743	0.28209391
C	2.77741785	-0.17770440	0.20169674
C	2.57508945	1.18738693	-0.13813115
N	-1.91967158	0.61549799	0.09914015
C	-3.16601060	-0.09106168	0.24970077
H	3.37569537	1.88775324	-0.25635701
C	-1.35434893	-1.62239248	-0.25112987
C	-2.86607333	-1.52675597	-0.21767116
H	-3.26267232	-1.67234916	-1.21716391
H	-3.30441359	-2.26686830	0.43930905
H	-3.51929686	-0.00889466	1.26867378
H	-3.91148777	0.40409988	-0.35917555
H	-0.90485082	-2.19327388	-1.05172069
H	0.73455088	2.28037506	-0.69847396
C	-1.83596243	2.04495465	0.08128020
H	-1.03451094	2.42962607	0.69362762
H	-1.72269373	2.44268165	-0.92658212
H	-2.76365569	2.45555779	0.47638359
H	1.35680870	-1.85655288	0.46840205
C	4.12726106	-0.83233929	0.13158241
H	4.59532334	-0.68624349	-0.84156846
H	4.83343290	-0.37743563	0.82448647
H	4.08293234	-1.89924892	0.32885993
H	-0.98502579	-2.04740019	0.69029949

Time = 103.50 fs
E(CASSCF/6-31G(d)) = -479.5740511

C	-0.89139060	-0.21825516	-0.30411715
C	0.57161889	0.14451337	-0.11698725
C	1.19470787	1.37764195	-0.37166385
C	1.51331513	-0.80484929	0.28525134
C	2.77646420	-0.17906775	0.20461957
C	2.57477440	1.18916413	-0.13959092
N	-1.91833044	0.61350495	0.09765220
C	-3.16620088	-0.08952616	0.25333880
H	3.37581739	1.88632445	-0.25830814
C	-1.35540396	-1.62456384	-0.25357209
C	-2.86686691	-1.52771747	-0.21873725
H	-3.26744837	-1.67124607	-1.21626544
H	-3.29853571	-2.27167962	0.43916322
H	-3.52377457	-0.01406753	1.27293015

H	-3.91976626	0.40092421	-0.35589493
H	-0.91158009	-2.19440146	-1.05609579
H	0.73703413	2.28198958	-0.70547488
C	-1.83557799	2.04410915	0.08328648
H	-1.03061294	2.42640849	0.69307755
H	-1.72882199	2.44434411	-0.92384179
H	-2.75694105	2.46336811	0.48463359
H	1.35635109	-1.85251786	0.47255402
C	4.12689957	-0.83195342	0.13282936
H	4.59017065	-0.69345176	-0.84359300
H	4.83225395	-0.37951593	0.82768283
H	4.07887579	-1.89801286	0.33309183
H	-0.98468605	-2.05309719	0.68559320

Time = 104.00 fs
E(CASSCF/6-31G(d)) = -479.5754289

C	-0.88807761	-0.21329084	-0.30594233
C	0.56424356	0.14337647	-0.11833748
C	1.19752405	1.37924561	-0.37564822
C	1.51786670	-0.80459880	0.28848564
C	2.77560352	-0.18048940	0.20757187
C	2.57441050	1.19093508	-0.14107711
N	-1.91656881	0.61123305	0.09608438
C	-3.16650924	-0.08797013	0.25693487
H	3.37589559	1.88563517	-0.26048647
C	-1.35634692	-1.62672181	-0.25591423
C	-2.86776726	-1.52868508	-0.21985122
H	-3.27251950	-1.67021427	-1.21515951
H	-3.29293619	-2.27637080	0.43879051
H	-3.52790985	-0.01963743	1.27768243
H	-3.92818413	0.39745365	-0.35273095
H	-0.91879085	-2.19605205	-1.06078103
H	0.73937765	2.28386125	-0.71259158
C	-1.83531792	2.04347913	0.08536746
H	-1.02660053	2.42253665	0.69236469
H	-1.73477280	2.44543197	-0.92104874
H	-2.75000841	2.47062393	0.49281910
H	1.35573119	-1.84818028	0.47668343
C	4.12657024	-0.83155938	0.13409696
H	4.58463648	-0.70104601	-0.84580979
H	4.83090919	-0.38183900	0.83136643
H	4.07491087	-1.89671851	0.33745232
H	-0.98427473	-2.05883748	0.68034717

Time = 104.50 fs
E(CASSCF/6-31G(d)) = -479.5763021

C	-0.88506702	-0.20810218	-0.30766768
C	0.55681413	0.14231728	-0.11976379
C	1.20036066	1.38085097	-0.37967208
C	1.52245389	-0.80438978	0.29177243
C	2.77485963	-0.18191581	0.21053361
C	2.57400200	1.19265880	-0.14257613
N	-1.91442437	0.60873073	0.09445658
C	-3.16694916	-0.08642879	0.26046408
H	3.37600226	1.88576198	-0.26289404
C	-1.35716816	-1.62882358	-0.25816360
C	-2.86876705	-1.52964688	-0.22099194
H	-3.27785988	-1.66926982	-1.21390318
H	-3.28769486	-2.28086525	0.43817436
H	-3.53152033	-0.02549360	1.28279361
H	-3.93641970	0.39369194	-0.34953392
H	-0.92638744	-2.19823012	-1.06582675
H	0.74164128	2.28590930	-0.71977370
C	-1.83517116	2.04306703	0.08752416
H	-1.02245997	2.41802133	0.69143730
H	-1.74048888	2.44594609	-0.91825711
H	-2.74296383	2.47716017	0.50093958
H	1.35499466	-1.84383844	0.48084384
C	4.12626375	-0.83117203	0.13538665
H	4.57879595	-0.70892809	-0.84814858
H	4.82945277	-0.38434885	0.83543416
H	4.07107030	-1.89540851	0.34193584
H	-0.98379259	-2.06456443	0.67460326

Time = 105.00 fs
E(CASSCF/6-31G(d)) = -479.5766150

C	-0.88240928	-0.20278412	-0.30930197
C	0.54946525	0.14136832	-0.12125024
C	1.20317479	1.38243001	-0.38373728
C	1.52698921	-0.80418671	0.29509530
C	2.77425368	-0.18329754	0.21349145
C	2.57355532	1.19430332	-0.14407855
N	-1.91193837	0.60604610	0.09278759
C	-3.16753077	-0.08493506	0.26391282
H	3.37617178	1.88673543	-0.26552951
C	-1.35786307	-1.63083438	-0.26033839
C	-2.86986011	-1.53059309	-0.22214047
H	-3.28345151	-1.66842546	-1.21255931
H	-3.28287788	-2.28510621	0.43730732
H	-3.53443836	-0.03153377	1.28811853
H	-3.94419065	0.38965535	-0.34615962

H	-0.93429813	-2.20091459	-1.07126512
H	0.74389919	2.28804259	-0.72698120
C	-1.83512285	2.04286691	0.08976029
H	-1.01816214	2.41288657	0.69025438
H	-1.74593221	2.44590601	-0.91552155
H	-2.73592429	2.48285413	0.50902703
H	1.35418450	-1.83978363	0.48510037
C	4.12597043	-0.83080573	0.13670310
H	4.57271721	-0.71701295	-0.85053730
H	4.82793583	-0.38699348	0.83977963
H	4.06737361	-1.89412252	0.34654606
H	-0.98322887	-2.07024722	0.66842452

Time = 105.50 fs
E(CASSCF/6-31G(d)) = -479.5763676

C	-0.88014960	-0.19743304	-0.31085742
C	0.54232933	0.14056107	-0.12278061
C	1.20592541	1.38395806	-0.38784628
C	1.53138817	-0.80395783	0.29843911
C	2.77380572	-0.18458687	0.21643214
C	2.57308013	1.19584242	-0.14557599
N	-1.90915860	0.60323083	0.09109836
C	-3.16825885	-0.08352305	0.26727039
H	3.37639571	1.88853881	-0.26838274
C	-1.35842973	-1.63272328	-0.26246190
C	-2.87103969	-1.53151390	-0.22327833
H	-3.28927137	-1.66769201	-1.21119334
H	-3.27855027	-2.28904784	0.43619286
H	-3.53650774	-0.03765367	1.29350691
H	-3.95125086	0.38538512	-0.34248759
H	-0.94246100	-2.20405385	-1.07709544
H	0.74622862	2.29016368	-0.73417110
C	-1.83515559	2.04286650	0.09207725
H	-1.01367289	2.40717744	0.68878787
H	-1.75107168	2.44534555	-0.91289947
H	-2.72902764	2.48760991	0.51712547
H	1.35334418	-1.83628158	0.48951435
C	4.12568127	-0.83047511	0.13805129
H	4.56647257	-0.72521335	-0.85289952
H	4.82640870	-0.38972071	0.84428794
H	4.06383638	-1.89289773	0.35128552
H	-0.98256313	-2.07586664	0.66190224

Time = 106.00 fs
E(CASSCF/6-31G(d)) = -479.5756050

C	-0.87832674	-0.19214524	-0.31234936
C	0.53553396	0.13992528	-0.12433881
C	1.20857442	1.38541326	-0.39200171
C	1.53557049	-0.80367729	0.30179059
C	2.77353412	-0.18573864	0.21934283
C	2.57258923	1.19725589	-0.14706122
N	-1.90613873	0.60033914	0.08941175
C	-3.16913176	-0.08222778	0.27052866
H	3.37662354	1.89110912	-0.27143487
C	-1.35886887	-1.63446373	-0.26456188
C	-2.87229875	-1.53239955	-0.22438816
H	-3.29528995	-1.66707745	-1.20986770
H	-3.27477483	-2.29265650	0.43484571
H	-3.53759001	-0.04374908	1.29881638
H	-3.95740315	0.38095004	-0.33842951
H	-0.95082408	-2.20756657	-1.08328323
H	0.74870364	2.29217761	-0.74130111
C	-1.83524986	2.04304794	0.09447402
H	-1.00895533	2.40095862	0.68702230
H	-1.75588451	2.44431090	-0.91044617
H	-2.72242485	2.49135848	0.52528819
H	1.35251992	-1.83354568	0.49413777
C	4.12538800	-0.83019500	0.13943644
H	4.56013746	-0.73344160	-0.85515714
H	4.82492037	-0.39247887	0.84883882
H	4.06047028	-1.89176675	0.35615519
H	-0.98176720	-2.08141297	0.65514905

Time = 106.50 fs
E(CASSCF/6-31G(d)) = -479.5744092

C	-0.87697175	-0.18701546	-0.31379572
C	0.52919948	0.13948828	-0.12590931
C	1.21108752	1.38677610	-0.39620601
C	1.53946134	-0.80332659	0.30513872
C	2.77345500	-0.18671093	0.22221127
C	2.57209801	1.19852945	-0.14852825
N	-1.90293748	0.59742675	0.08775210
C	-3.17014057	-0.08108471	0.27368178
H	3.37676854	1.89434236	-0.27465921
C	-1.35918378	-1.63603387	-0.26666934
C	-2.87363022	-1.53324044	-0.22545435
H	-3.30147003	-1.66658604	-1.20863665
H	-3.27161074	-2.29591113	0.43329151
H	-3.53756998	-0.04971814	1.30392396
H	-3.96250879	0.37644665	-0.33393463
H	-0.95934384	-2.21134507	-1.08976282

H	0.75138950	2.29399994	-0.74833290
C	-1.83538473	2.04338870	0.09694720
H	-1.00397313	2.39431234	0.68495373
H	-1.76035754	2.44285858	-0.90820974
H	-2.71627046	2.49405622	0.53357391
H	1.35176295	-1.83171500	0.49900841
C	4.12508318	-0.82998024	0.14086403
H	4.55378934	-0.74161130	-0.85723343
H	4.82351842	-0.39521733	0.85331012
H	4.05728330	-1.89075585	0.36115414
H	-0.98080793	-2.08688288	0.64829033

Time = 107.00 fs
E(CASSCF/6-31G(d)) = -479.5728890

C	-0.87610708	-0.18213553	-0.31521651
C	0.52343706	0.13927431	-0.12747753
C	1.21343475	1.38802870	-0.40046113
C	1.54299193	-0.80289528	0.30847493
C	2.77358170	-0.18746550	0.22502603
C	2.57162362	1.19965407	-0.14997235
N	-1.89961769	0.59454978	0.08614496
C	-3.17126877	-0.08012916	0.27672571
H	3.37671736	1.89810312	-0.27802280
C	-1.35938022	-1.63741649	-0.26881703
C	-2.87502737	-1.53402731	-0.22646345
H	-3.30776565	-1.66621753	-1.20754154
H	-3.26911182	-2.29880257	0.43156554
H	-3.53635973	-0.05546365	1.30873509
H	-3.96649290	0.37199760	-0.32899235
H	-0.96798248	-2.21526110	-1.09644296
H	0.75433856	2.29556389	-0.75523466
C	-1.83553855	2.04386263	0.09949115
H	-0.99869453	2.38733527	0.68258800
H	-1.76448831	2.44105277	-0.90622586
H	-2.71071174	2.49568161	0.54204219
H	1.35113179	-1.83084018	0.50414639
C	4.12476018	-0.82984554	0.14233972
H	4.54750691	-0.74963945	-0.85905627
H	4.82224917	-0.39788699	0.85758163
H	4.05427970	-1.88988414	0.36627959
H	-0.97965093	-2.09227553	0.64145406

Time = 107.50 fs
E(CASSCF/6-31G(d)) = -479.5711681

C	-0.87574517	-0.17759289	-0.31663335

C	0.51834622	0.13930376	-0.12903004
C	1.21559126	1.38915430	-0.40476806
C	1.54610096	-0.80238062	0.31179307
C	2.77392398	-0.18796886	0.22777685
C	2.57118387	1.20062557	-0.15139007
N	-1.89624523	0.59176361	0.08461661
C	-3.17249238	-0.07939561	0.27965786
H	3.37634413	1.90223491	-0.28148865
C	-1.35946624	-1.63859906	-0.27103766
C	-2.87648395	-1.53475144	-0.22740449
H	-3.31412243	-1.66596718	-1.20660888
H	-3.26732532	-2.30133281	0.42971120
H	-3.53390254	-0.06089543	1.31319082
H	-3.96934736	0.36774858	-0.32363274
H	-0.97670408	-2.21917297	-1.10321584
H	0.75758475	2.29682512	-0.76198485
C	-1.83568961	2.04444134	0.10209846
H	-0.99309654	2.38013553	0.67993854
H	-1.76828542	2.43896325	-0.90451442
H	-2.70587774	2.49623229	0.55074772
H	1.35069015	-1.83088007	0.50955264
C	4.12441340	-0.82980534	0.14386926
H	4.54136851	-0.75744743	-0.86056080
H	4.82115737	-0.40044047	0.86153813
H	4.05146078	-1.88916227	0.37152635
H	-0.97826421	-2.09758852	0.63476024

Time = 108.00 fs
E(CASSCF/6-31G(d)) = -479.5693791

C	-0.87588774	-0.17346933	-0.31806874
C	0.51401323	0.13959262	-0.13055501
C	1.21753743	1.39013677	-0.40912670
C	1.54873523	-0.80178648	0.31508922
C	2.77448755	-0.18819255	0.23045480
C	2.57079604	1.20144353	-0.15277908
N	-1.89288779	0.58912180	0.08319363
C	-3.17378043	-0.07891697	0.28247663
H	3.37552449	1.90657290	-0.28501766
C	-1.35945202	-1.63957341	-0.27336199
C	-2.87799443	-1.53540488	-0.22826910
H	-3.32047732	-1.66582518	-1.20584822
H	-3.26628963	-2.30351226	0.42777704
H	-3.53017416	-0.06593256	1.31727101
H	-3.97112865	0.36386323	-0.31792374
H	-0.98547110	-2.22293336	-1.10996617
H	0.76114111	2.29776577	-0.76857340

C	-1.83581698	2.04509535	0.10476037
H	-0.98716941	2.37282905	0.67702411
H	-1.77176879	2.43666273	-0.90307579
H	-2.70186842	2.49571989	0.55973473
H	1.35050529	-1.83170801	0.51520949
C	4.12403805	-0.82987347	0.14545839
H	4.53545151	-0.76496283	-0.86169227
H	4.82028653	-0.40283225	0.86507317
H	4.04882503	-1.88859303	0.37688718
H	-0.97662242	-2.10281287	0.62831019

Time = 108.50 fs
E(CASSCF/6-31G(d)) = -479.5676413

C	-0.87652582	-0.16983957	-0.31954566
C	0.51050983	0.14015208	-0.13204231
C	1.21925926	1.39096008	-0.41353557
C	1.55085074	-0.80112114	0.31836128
C	2.77527338	-0.18811362	0.23305250
C	2.57047553	1.20211029	-0.15413820
N	-1.88961324	0.58667481	0.08190234
C	-3.17509610	-0.07872358	0.28518109
H	3.37414849	1.91095728	-0.28857138
C	-1.35934959	-1.64033538	-0.27581712
C	-2.87955419	-1.53598072	-0.22905134
H	-3.32676084	-1.66577691	-1.20525251
H	-3.26603283	-2.30535753	0.42581449
H	-3.52518268	-0.07050519	1.32099459
H	-3.97195190	0.36051640	-0.31196459
H	-0.99424056	-2.22639911	-1.11658250
H	0.76500087	2.29839384	-0.77500141
C	-1.83590127	2.04579546	0.10746742
H	-0.98092017	2.36553558	0.67386614
H	-1.77496980	2.43422518	-0.90188892
H	-2.69874487	2.49416536	0.56903192
H	1.35064586	-1.83312744	0.52108256
C	4.12363031	-0.83006292	0.14711277
H	4.52983120	-0.77212144	-0.86240859
H	4.81967959	-0.40501934	0.86809226
H	4.04636824	-1.88817244	0.38235325
H	-0.97471021	-2.10793035	0.62217886

Time = 109.00 fs
E(CASSCF/6-31G(d)) = -479.5661030

C	-0.87763959	-0.16677046	-0.32108674
C	0.50789258	0.14098823	-0.13348373

C	1.22074723	1.39160885	-0.41799184
C	1.55241266	-0.80039570	0.32160836
C	2.77627783	-0.18771446	0.23556415
C	2.57023560	1.20262977	-0.15546705
N	-1.88648878	0.58446936	0.08076843
C	-3.17639738	-0.07884239	0.28777039
H	3.37213275	1.91524371	-0.29211410
C	-1.35917262	-1.64088432	-0.27842481
C	-2.88115953	-1.53647298	-0.22974749
H	-3.33289748	-1.66580321	-1.20480037
H	-3.26657045	-2.30688784	0.42387415
H	-3.51896738	-0.07455598	1.32441676
H	-3.97198001	0.35788649	-0.30587852
H	-1.00296193	-2.22943711	-1.12296496
H	0.76913592	2.29874346	-0.78128222
C	-1.83592517	2.04651298	0.11020997
H	-0.97437556	2.35837547	0.67048864
H	-1.77792988	2.43172345	-0.90091277
H	-2.69652398	2.49159519	0.57864756
H	1.35117544	-1.83489476	0.52712553
C	4.12318731	-0.83038565	0.14883782
H	4.52457956	-0.77886772	-0.86268113
H	4.81937763	-0.40696160	0.87051531
H	4.04408400	-1.88789013	0.38791391
H	-0.97252605	-2.11290867	0.61640660

Time = 109.50 fs
E(CASSCF/6-31G(d)) = -479.5648362

C	-0.87919871	-0.16431941	-0.32271410
C	0.50620154	0.14210175	-0.13487292
C	1.22199738	1.39206756	-0.42249124
C	1.55339717	-0.79962046	0.32483030
C	2.77749154	-0.18698368	0.23798576
C	2.57008568	1.20300698	-0.15676619
N	-1.88357854	0.58254687	0.07981618
C	-3.17764002	-0.07929592	0.29024355
H	3.36942979	1.91931322	-0.29561496
C	-1.35893608	-1.64122268	-0.28120052
C	-2.88280767	-1.53687728	-0.23035564
H	-3.33881023	-1.66588111	-1.20445906
H	-3.26790507	-2.30812279	0.42200360
H	-3.51159646	-0.07804140	1.32762400
H	-3.97141038	0.35614547	-0.29979955
H	-1.01157484	-2.23193339	-1.12903625
H	0.77350048	2.29886908	-0.78743936
C	-1.83587404	2.04722153	0.11297896

H	-0.96758294	2.35146656	0.66691475
H	-1.78070038	2.42922909	-0.90008671
H	-2.69517340	2.48803919	0.58856742
H	1.35214787	-1.83674373	0.53328375
C	4.12270717	-0.83085212	0.15063863
H	4.51976369	-0.78515688	-0.86249649
H	4.81942079	-0.40862228	0.87227847
H	4.04196378	-1.88773197	0.39355797
H	-0.97008249	-2.11770264	0.61099819

Time = 110.00 fs
E(CASSCF/6-31G(d)) = -479.5639820

C	-0.88116343	-0.16253368	-0.32444849
C	0.50545968	0.14348766	-0.13620553
C	1.22301019	1.39232179	-0.42702820
C	1.55379157	-0.79880343	0.32802707
C	2.77889994	-0.18591611	0.24031524
C	2.57003151	1.20324750	-0.15803697
N	-1.88094266	0.58094286	0.07906799
C	-3.17877868	-0.08010121	0.29259894
H	3.36603468	1.92307879	-0.29904933
C	-1.35865607	-1.64135527	-0.28415261
C	-2.88449649	-1.53719055	-0.23087530
H	-3.34442184	-1.66598437	-1.20418888
H	-3.27002441	-2.30907878	0.42024422
H	-3.50316301	-0.08093215	1.33072583
H	-3.97045588	0.35544861	-0.29386012
H	-1.02000939	-2.23379603	-1.13474535
H	0.77803227	2.29884150	-0.79350510
C	-1.83573610	2.04789656	0.11576621
H	-0.96061075	2.34492081	0.66316788
H	-1.78334033	2.42681052	-0.89933470
H	-2.69461319	2.48352907	0.59875338
H	1.35360031	-1.83841198	0.53950067
C	4.12218903	-0.83147103	0.15251978
H	4.51544460	-0.79095499	-0.86185579
H	4.81984647	-0.40996863	0.87333560
H	4.03999759	-1.88768116	0.39927364
H	-0.96740780	-2.12225183	0.60592148

Time = 110.50 fs
E(CASSCF/6-31G(d)) = -479.5635640

C	-0.88348581	-0.16144889	-0.32630868
C	0.50567322	0.14513550	-0.13747915
C	1.22379042	1.39235826	-0.43159605

C	1.55359479	-0.79794788	0.33119837
C	2.78048281	-0.18451257	0.24255223
C	2.57007461	1.20335669	-0.15928144
N	-1.87863506	0.57968563	0.07854367
C	-3.17977009	-0.08126879	0.29483408
H	3.36198698	1.92648919	-0.30240009
C	-1.35834960	-1.64128913	-0.28728235
C	-2.88622468	-1.53741142	-0.23130683
H	-3.34965970	-1.66608480	-1.20394809
H	-3.27290176	-2.30976775	0.41862936
H	-3.49378153	-0.08321397	1.33384373
H	-3.96932532	0.35592219	-0.28817512
H	-1.02818532	-2.23496145	-1.14007476
H	0.78265874	2.29873810	-0.79951684
C	-1.83550273	2.04851633	0.11856498
H	-0.95354547	2.33884328	0.65927159
H	-1.78591488	2.42453377	-0.89857012
H	-2.69471930	2.47809963	0.60914432
H	1.35554980	-1.83966191	0.54572057
C	4.12163322	-0.83224897	0.15448530
H	4.51167544	-0.79623996	-0.86077420
H	4.82068902	-0.41097294	0.87365857
H	4.03817414	-1.88772080	0.40504928
H	-0.96454297	-2.12648539	0.60111306

Time = 111.00 fs
E(CASSCF/6-31G(d)) = -479.5636507

C	-0.88611161	-0.16108833	-0.32831108
C	0.50683147	0.14702937	-0.13869320
C	1.22434669	1.39216579	-0.43618748
C	1.55281757	-0.79705103	0.33434329
C	2.78221480	-0.18278001	0.24469825
C	2.57021238	1.20333977	-0.16050232
N	-1.87670201	0.57879552	0.07825985
C	-3.18057530	-0.08280167	0.29694547
H	3.35737047	1.92953053	-0.30565790
C	-1.35803438	-1.64103274	-0.29058427
C	-2.88799128	-1.53754013	-0.23165104
H	-3.35445832	-1.66615290	-1.20369761
H	-3.27649544	-2.31019534	0.41718283
H	-3.48358171	-0.08488749	1.33709920
H	-3.96820203	0.35765242	-0.28282695
H	-1.03601460	-2.23539495	-1.14504086
H	0.78730103	2.29863508	-0.80551381
C	-1.83516840	2.04906164	0.12137003
H	-0.94648772	2.33332943	0.65524997

H	-1.78849382	2.42246178	-0.89770123
H	-2.69533174	2.47179068	0.61966039
H	1.35798920	-1.84030016	0.55189358
C	4.12104123	-0.83319013	0.15653843
H	4.50849979	-0.80100217	-0.85927914
H	4.82197806	-0.41161326	0.87323685
H	4.03648113	-1.88783529	0.41087434
H	-0.96153975	-2.13032493	0.59648438

Time = 111.50 fs
E(CASSCF/6-31G(d)) = -479.5642495

C	-0.88898221	-0.16146211	-0.33046911
C	0.50890745	0.14914812	-0.13984884
C	1.22469093	1.39173620	-0.44079487
C	1.55148225	-0.79610332	0.33746015
C	2.78406598	-0.18073114	0.24675658
C	2.57043846	1.20320172	-0.16170300
N	-1.87518067	0.57828406	0.07822929
C	-3.18116195	-0.08469449	0.29892874
H	3.35230919	1.93222451	-0.30882114
C	-1.35772874	-1.64059569	-0.29404691
C	-2.88979547	-1.53757854	-0.23190883
H	-3.35876239	-1.66615892	-1.20340532
H	-3.28074962	-2.31036053	0.41591827
H	-3.47270339	-0.08596799	1.34060031
H	-3.96722557	0.36067532	-0.27785299
H	-1.04340382	-2.23509109	-1.14969352
H	0.79187897	2.29859811	-0.81153330
C	-1.83473050	2.04951564	0.12417772
H	-0.93954707	2.32846398	0.65112892
H	-1.79114942	2.42065452	-0.89663702
H	-2.69626541	2.46465028	0.63020822
H	1.36088538	-1.84019080	0.55797865
C	4.12041583	-0.83429604	0.15868161
H	4.50595001	-0.80524461	-0.85740803
H	4.82373698	-0.41187436	0.87207656
H	4.03490564	-1.88801199	0.41673989
H	-0.95845673	-2.13368998	0.59193106

Time = 112.00 fs
E(CASSCF/6-31G(d)) = -479.5653351

C	-0.89203709	-0.16256637	-0.33279263
C	0.51185879	0.15146557	-0.14094891
C	1.22483772	1.39106524	-0.44541071
C	1.54962206	-0.79508819	0.34054632

C	2.78600259	-0.17838426	0.24873211
C	2.57074357	1.20294762	-0.16288757
N	-1.87409791	0.57815336	0.07846042
C	-3.18150572	-0.08693298	0.30077908
H	3.34695986	1.93462352	-0.31189538
C	-1.35745148	-1.63998836	-0.29765400
C	-2.89163642	-1.53752993	-0.23208098
H	-3.36252943	-1.66607389	-1.20304988
H	-3.28559551	-2.31025636	0.41483948
H	-3.46129198	-0.08648522	1.34442913
H	-3.96647855	0.36496950	-0.27323825
H	-1.05025696	-2.23407184	-1.15411265
H	0.79631663	2.29867303	-0.81760631
C	-1.83418908	2.04986348	0.12698580
H	-0.93283591	2.32432014	0.64693865
H	-1.79395409	2.41916912	-0.89529366
H	-2.69732217	2.45673859	0.64068668
H	1.36417927	-1.83926199	0.56394570
C	4.11976113	-0.83556536	0.16091627
H	4.50404594	-0.80898245	-0.85520534
H	4.82598103	-0.41174895	0.87019939
H	4.03343461	-1.88824208	0.42263858
H	-0.95535498	-2.13650345	0.58734279

Time = 112.50 fs
E(CASSCF/6-31G(d)) = -479.5668468

C	-0.89521571	-0.16438302	-0.33528749
C	0.51562900	0.15395092	-0.14199770
C	1.22480404	1.39015338	-0.45002799
C	1.54728025	-0.79398253	0.34359833
C	2.78798807	-0.17576258	0.25063119
C	2.57111612	1.20258295	-0.16406078
N	-1.87346939	0.57839577	0.07895677
C	-3.18159146	-0.08949381	0.30249203
H	3.34150270	1.93680376	-0.31489202
C	-1.35722175	-1.63922182	-0.30138584
C	-2.89351285	-1.53739896	-0.23216806
H	-3.36573156	-1.66587024	-1.20262289
H	-3.29095328	-2.30987106	0.41394141
H	-3.44949365	-0.08648273	1.34863041
H	-3.96598020	0.37045283	-0.26891284
H	-1.05647964	-2.23238317	-1.15840237
H	0.80054545	2.29887977	-0.82375515
C	-1.83354649	2.05009219	0.12979317
H	-0.92646315	2.32095842	0.64271402
H	-1.79697871	2.41805948	-0.89359879

H	-2.69830435	2.44813094	0.65099478
H	1.36778704	-1.83750965	0.56977697
C	4.11908266	-0.83699379	0.16324279
H	4.50279346	-0.81224291	-0.85271961
H	4.82871552	-0.41123841	0.86764055
H	4.03205522	-1.88852160	0.42856541
H	-0.95229382	-2.13869784	0.58261359

Time = 113.00 fs
E(CASSCF/6-31G(d)) = -479.5686852

C	-0.89845969	-0.16687958	-0.33795517
C	0.52014875	0.15656913	-0.14300087
C	1.22460888	1.38900647	-0.45464059
C	1.54450889	-0.79275760	0.34661198
C	2.78998415	-0.17289375	0.25246147
C	2.57154332	1.20211430	-0.16522810
N	-1.87329902	0.57899365	0.07971661
C	-3.18141332	-0.09234480	0.30406460
H	3.33612915	1.93885535	-0.31782644
C	-1.35705888	-1.63830783	-0.30522078
C	-2.89542291	-1.53719142	-0.23217049
H	-3.36835670	-1.66552255	-1.20213037
H	-3.29673432	-2.30919044	0.41321186
H	-3.43745200	-0.08601753	1.35320406
H	-3.96568858	0.37698392	-0.26475684
H	-1.06198326	-2.23008982	-1.16268294
H	0.80450747	2.29920731	-0.82999085
C	-1.83280704	2.05019033	0.13259953
H	-0.92052769	2.31842619	0.63849590
H	-1.80029086	2.41737618	-0.89149593
H	-2.69902721	2.43892072	0.66103895
H	1.37160319	-1.83499423	0.57546713
C	4.11838730	-0.83857398	0.16566044
H	4.50218343	-0.81506468	-0.85000023
H	4.83193413	-0.41035362	0.86444670
H	4.03075527	-1.88885137	0.43451783
H	-0.94932641	-2.14022057	0.57765086

Time = 113.50 fs
E(CASSCF/6-31G(d)) = -479.5707247

C	-0.90171439	-0.17000939	-0.34079256
C	0.52533753	0.15928142	-0.14396528
C	1.22427301	1.38763615	-0.45924359
C	1.54136738	-0.79138047	0.34958273
C	2.79195207	-0.16980906	0.25423163

C	2.57201235	1.20154990	-0.16639576
N	-1.87357878	0.57991957	0.08073279
C	-3.18097395	-0.09544568	0.30549635
H	3.33102857	1.94087077	-0.32071577
C	-1.35698217	-1.63725910	-0.30913687
C	-2.89736387	-1.53691397	-0.23208874
H	-3.37040890	-1.66500813	-1.20159266
H	-3.30284391	-2.30820062	0.41263359
H	-3.42530561	-0.08515961	1.35810195
H	-3.96551048	0.38436846	-0.26061092
H	-1.06668945	-2.22726882	-1.16708097
H	0.80815651	2.29961237	-0.83631225
C	-1.83197664	2.05014793	0.13540503
H	-0.91511283	2.31675708	0.63433155
H	-1.80395332	2.41716592	-0.88894727
H	-2.69933012	2.42922093	0.67073994
H	1.37550456	-1.83183284	0.58102246
C	4.11768325	-0.84029559	0.16816735
H	4.50219095	-0.81749702	-0.84709442
H	4.83561741	-0.40911538	0.86067383
H	4.02952360	-1.88923659	0.44049583
H	-0.94649622	-2.14103853	0.57238296

Time = 114.00 fs
E(CASSCF/6-31G(d)) = -479.5728236

C	-0.90493031	-0.17371226	-0.34379192
C	0.53110518	0.16204581	-0.14489881
C	1.22381899	1.38605989	-0.46383344
C	1.53792095	-0.78981594	0.35250603
C	2.79385371	-0.16654295	0.25595126
C	2.57251133	1.20090059	-0.16757089
N	-1.87428914	0.58113672	0.08199269
C	-3.18028321	-0.09874917	0.30679056
H	3.32637484	1.94293279	-0.32357632
C	-1.35701065	-1.63608957	-0.31311336
C	-2.89933200	-1.53657394	-0.23192359
H	-3.37190794	-1.66430732	-1.20104319
H	-3.30918397	-2.30689112	0.41218670
H	-3.41318709	-0.08399142	1.36322982
H	-3.96531696	0.39236958	-0.25629169
H	-1.07053414	-2.22400231	-1.17171840
H	0.81145791	2.30002165	-0.84270629
C	-1.83106246	2.04995641	0.13820992
H	-0.91028187	2.31597068	0.63027475
H	-1.80802254	2.41747072	-0.88593524
H	-2.69908570	2.41916454	0.68003848

H	1.37935551	-1.82818660	0.58645857
C	4.11697988	-0.84214545	0.17076045
H	4.50277522	-0.81959866	-0.84404469
H	4.83973189	-0.40755462	0.85638545
H	4.02835045	-1.88968578	0.44650163
H	-0.94383426	-2.14114129	0.56676493

Time = 114.50 fs
E(CASSCF/6-31G(d)) = -479.5748361

C	-0.90806419	-0.17791554	-0.34694114
C	0.53735398	0.16481785	-0.14581021
C	1.22327104	1.38430075	-0.46840795
C	1.53423879	-0.78802877	0.35537780
C	2.79565289	-0.16313210	0.25763064
C	2.57303041	1.20018042	-0.16876155
N	-1.87539970	0.58259972	0.08347851
C	-3.17935632	-0.10220249	0.30795496
H	3.32231351	1.94510341	-0.32642154
C	-1.35716276	-1.63481479	-0.31713213
C	-2.90132249	-1.53617898	-0.23167658
H	-3.37288813	-1.66340376	-1.20052586
H	-3.31565619	-2.30525778	0.41185106
H	-3.40122266	-0.08260699	1.36845432
H	-3.96496236	0.40072153	-0.25160875
H	-1.07347180	-2.22037009	-1.17670128
H	0.81438823	2.30033616	-0.84914949
C	-1.83007265	2.04960861	0.14101416
H	-0.90607513	2.31607224	0.62638474
H	-1.81254783	2.41832708	-0.88246220
H	-2.69820628	2.40890323	0.68889983
H	1.38301436	-1.82424388	0.59179773
C	4.11628763	-0.84410784	0.17343557
H	4.50388002	-0.82143687	-0.84088727
H	4.84423003	-0.40571224	0.85165104
H	4.02722775	-1.89020970	0.45253955
H	-0.94135728	-2.14054324	0.56078187

Time = 115.00 fs
E(CASSCF/6-31G(d)) = -479.5766293

C	-0.91107926	-0.18253556	-0.35022401
C	0.54398038	0.16755133	-0.14670898
C	1.22265500	1.38238676	-0.47296633
C	1.53039244	-0.78598609	0.35819501
C	2.79731624	-0.15961473	0.25928052
C	2.57356251	1.19940715	-0.16997678

N	-1.87687031	0.58425580	0.08516759
C	-3.17821207	-0.10574904	0.30900213
H	3.31895220	1.94741412	-0.32925974
C	-1.35745603	-1.63345235	-0.32117881
C	-2.90332939	-1.53573690	-0.23135039
H	-3.37339636	-1.66228445	-1.20009142
H	-3.32216470	-2.30330547	0.41160843
H	-3.38953225	-0.08111072	1.37361447
H	-3.96430377	0.40914479	-0.24638201
H	-1.07547848	-2.21644295	-1.18210945
H	0.81693214	2.30043969	-0.85561096
C	-1.82901602	2.04909904	0.14381724
H	-0.90250873	2.31705246	0.62272464
H	-1.81757033	2.41976460	-0.87854880
H	-2.69664771	2.39860479	0.69731660
H	1.38633925	-1.82020026	0.59706476
C	4.11561761	-0.84616479	0.17618747
H	4.50543463	-0.82308597	-0.83765109
H	4.84905072	-0.40363822	0.84654476
H	4.02614939	-1.89081944	0.45861556
H	-0.93906698	-2.13928413	0.55445010

Time = 115.50 fs
E(CASSCF/6-31G(d)) = -479.5780924

C	-0.91394520	-0.18747935	-0.35362074
C	0.55087674	0.17019915	-0.14760532
C	1.22199836	1.38034999	-0.47750890
C	1.52645426	-0.78365987	0.36095626
C	2.79881411	-0.15602993	0.26091189
C	2.57410373	1.19860236	-0.17122653
N	-1.87865251	0.58604619	0.08703300
C	-3.17687096	-0.10933022	0.30994946
H	3.31635446	1.94985969	-0.33209253
C	-1.35790680	-1.63202222	-0.32524350
C	-2.90534563	-1.53525538	-0.23094932
H	-3.37348956	-1.66093956	-1.19979284
H	-3.32861863	-2.30104989	0.41144406
H	-3.37822974	-0.07961560	1.37853598
H	-3.96321853	0.41736132	-0.24045694
H	-1.07655422	-2.21227722	-1.18798827
H	0.81907933	2.30020890	-0.86205619
C	-1.82790191	2.04842407	0.14661793
H	-0.89957503	2.31888727	0.61935926
H	-1.82312240	2.42180443	-0.87423088
H	-2.69441012	2.38844916	0.70530971
H	1.38919409	-1.81623708	0.60228257

C	4.11498128	-0.84829657	0.17901001
H	4.50735539	-0.82462575	-0.83435788
H	4.85412064	-0.40139031	0.84114473
H	4.02511125	-1.89152481	0.46473687
H	-0.93695059	-2.13742791	0.54781575

Time = 116.00 fs
E(CASSCF/6-31G(d)) = -479.5791446

C	-0.91663797	-0.19264690	-0.35710863
C	0.55793346	0.17271440	-0.14850986
C	1.22133003	1.37822545	-0.48203702
C	1.52249582	-0.78102922	0.36366227
C	2.80012121	-0.15241690	0.26253584
C	2.57465355	1.19779130	-0.17252155
N	-1.88069110	0.58790777	0.08904433
C	-3.17535359	-0.11288724	0.31081864
H	3.31453831	1.95239649	-0.33491467
C	-1.35852984	-1.63054691	-0.32932126
C	-2.90736326	-1.53474186	-0.23047967
H	-3.37323224	-1.65936249	-1.19968080
H	-3.33493445	-2.29851939	0.41134828
H	-3.36742343	-0.07824097	1.38304700
H	-3.96161911	0.42510904	-0.23371689
H	-1.07672386	-2.20791178	-1.19434373
H	0.82082114	2.29952386	-0.86845113
C	-1.82674001	2.04758236	0.14941426
H	-0.89724459	2.32153783	0.61635222
H	-1.82922696	2.42445790	-0.86955574
H	-2.69153542	2.37862340	0.71292725
H	1.39145386	-1.81250107	0.60746765
C	4.11439010	-0.85048214	0.18189630
H	4.50954761	-0.82613989	-0.83102251
H	4.85935628	-0.39903237	0.83553220
H	4.02411153	-1.89233193	0.47091112
H	-0.93498187	-2.13506057	0.54095182

Time = 116.50 fs
E(CASSCF/6-31G(d)) = -479.5797381

C	-0.91913914	-0.19793335	-0.36066273
C	0.56504062	0.17505140	-0.14943372
C	1.22068008	1.37604977	-0.48655268
C	1.51858673	-0.77808227	0.36631637
C	2.80121700	-0.14881438	0.26416334
C	2.57521445	1.19700220	-0.17387322
N	-1.88292592	0.58977487	0.09116845

C	-3.17367942	-0.11636293	0.31163486
H	3.31347902	1.95494520	-0.33771435
C	-1.35933803	-1.62905140	-0.33341188
C	-2.90937350	-1.53420331	-0.22995012
H	-3.37269338	-1.65754959	-1.19979853
H	-3.34103758	-2.29575515	0.41131686
H	-3.35721563	-0.07710970	1.38699368
H	-3.95946369	0.43215453	-0.22609174
H	-1.07603683	-2.20336720	-1.20114100
H	0.82214755	2.29827877	-0.87476630
C	-1.82554033	2.04657518	0.15220359
H	-0.89546961	2.32495047	0.61376282
H	-1.83589702	2.42772478	-0.86457711
H	-2.68810197	2.36931565	0.72024141
H	1.39300889	-1.80908708	0.61262616
C	4.11385499	-0.85269978	0.18483891
H	4.51190828	-0.82771418	-0.82765473
H	4.86466690	-0.39663213	0.82979151
H	4.02315052	-1.89324171	0.47714590
H	-0.93312387	-2.13228616	0.53395242

Time = 117.00 fs
E(CASSCF/6-31G(d)) = -479.5798607

C	-0.92143541	-0.20323155	-0.36425672
C	0.57208993	0.17716692	-0.15038843
C	1.22007926	1.37385986	-0.49105829
C	1.51479342	-0.77481748	0.36892482
C	2.80208599	-0.14525982	0.26580510
C	2.57579146	1.19626519	-0.17529323
N	-1.88529350	0.59158115	0.09337050
C	-3.17186582	-0.11970337	0.31242583
H	3.31311581	1.95739814	-0.34047474
C	-1.36034207	-1.62756279	-0.33751922
C	-2.91136709	-1.53364620	-0.22937191
H	-3.37194368	-1.65549995	-1.20017718
H	-3.34686348	-2.29281068	0.41135105
H	-3.34770181	-0.07634489	1.39025408
H	-3.95676302	0.43830347	-0.21756281
H	-1.07456554	-2.19864776	-1.20830716
H	0.82304507	2.29639144	-0.88098051
C	-1.82431323	2.04540671	0.15498274
H	-0.89418878	2.32905681	0.61164269
H	-1.84313545	2.43159177	-0.85934987
H	-2.68421633	2.36070815	0.72734337
H	1.39376846	-1.80602676	0.61775137
C	4.11338589	-0.85492765	0.18783009

H	4.51432908	-0.82943483	-0.82426137
H	4.86995809	-0.39425868	0.82401010
H	4.02223042	-1.89424838	0.48344830
H	-0.93133248	-2.12922177	0.52692542

Time = 117.50 fs
E(CASSCF/6-31G(d)) = -479.5795301

C	-0.92351763	-0.20843442	-0.36786353
C	0.57897608	0.17902126	-0.15138596
C	1.21955853	1.37169143	-0.49555631
C	1.51117844	-0.77124400	0.37149681
C	2.80271776	-0.14178906	0.26747135
C	2.57639127	1.19561118	-0.17679323
N	-1.88772885	0.59326142	0.09561478
C	-3.16992752	-0.12285951	0.31322063
H	3.31336207	1.95962916	-0.34317586
C	-1.36155014	-1.62610984	-0.34165017
C	-2.91333443	-1.53307637	-0.22875895
H	-3.37105305	-1.65321512	-1.20083207
H	-3.35235817	-2.28975052	0.41145667
H	-3.33896943	-0.07606638	1.39274927
H	-3.95358334	0.44340867	-0.20816487
H	-1.07240256	-2.19374529	-1.21573733
H	0.82349465	2.29381069	-0.88708375
C	-1.82306960	2.04408442	0.15774837
H	-0.89333241	2.33377415	0.61003252
H	-1.85093442	2.43603100	-0.85392516
H	-2.68000295	2.35297037	0.73433642
H	1.39366230	-1.80328463	0.62282243
C	4.11299121	-0.85714434	0.19086203
H	4.51669964	-0.83138633	-0.82084854
H	4.87513551	-0.39197973	0.81827833
H	4.02135513	-1.89533857	0.48982428
H	-0.92956088	-2.12599135	0.51998363

Time = 118.00 fs
E(CASSCF/6-31G(d)) = -479.5787974

C	-0.92538013	-0.21343738	-0.37145600
C	0.58559824	0.18057940	-0.15243873
C	1.21914847	1.36957779	-0.50004900
C	1.50779975	-0.76738128	0.37404440
C	2.80310678	-0.13843567	0.26917174
C	2.57702126	1.19507034	-0.17838444
N	-1.89016699	0.59475335	0.09786562
C	-3.16787628	-0.12578837	0.31404871

H	3.31411691	1.96150557	-0.34579704
C	-1.36296760	-1.62472227	-0.34581325
C	-2.91526592	-1.53249909	-0.22812771
H	-3.37008851	-1.65069910	-1.20176024
H	-3.35747803	-2.28664774	0.41164271
H	-3.33109630	-0.07638730	1.39445124
H	-3.95004590	0.44737544	-0.19798475
H	-1.06965689	-2.18864458	-1.22330389
H	0.82347058	2.29052174	-0.89307967
C	-1.82182107	2.04261940	0.16049745
H	-0.89282778	2.33900588	0.60895903
H	-1.85927520	2.44099906	-0.84834604
H	-2.67559216	2.34625178	0.74132775
H	1.39264116	-1.80076285	0.62780549
C	4.11267747	-0.85932939	0.19392708
H	4.51891066	-0.83364960	-0.81742386
H	4.88010863	-0.38985878	0.81268909
H	4.02053000	-1.89649097	0.49627852
H	-0.92776452	-2.12271882	0.51323484

Time = 118.50 fs
E(CASSCF/6-31G(d)) = -479.5777395

C	-0.92702017	-0.21814069	-0.37500748
C	0.59186160	0.18181213	-0.15355967
C	1.21887840	1.36754862	-0.50453818
C	1.50471013	-0.76325795	0.37658212
C	2.80325223	-0.13523046	0.27091514
C	2.57768868	1.19467058	-0.18007727
N	-1.89254439	0.59599913	0.10008825
C	-3.16572086	-0.12845412	0.31493880
H	3.31527679	1.96290097	-0.34831976
C	-1.36459672	-1.62342984	-0.35001710
C	-2.91715214	-1.53191905	-0.22749692
H	-3.36911296	-1.64795828	-1.20293948
H	-3.36218913	-2.28358097	0.41191946
H	-3.32414870	-0.07741052	1.39538696
H	-3.94632280	0.45016410	-0.18715714
H	-1.06644957	-2.18333003	-1.23086736
H	0.82294116	2.28654830	-0.89898631
C	-1.82058034	2.04102643	0.16322783
H	-0.89260446	2.34464222	0.60843253
H	-1.86812802	2.44643647	-0.84264401
H	-2.67110746	2.34067487	0.74841962
H	1.39067717	-1.79831270	0.63265663
C	4.11244882	-0.86146379	0.19701801
H	4.52085726	-0.83630025	-0.81399859

H	4.88479472	-0.38795265	0.80733729
H	4.01976127	-1.89767672	0.50281429
H	-0.92590626	-2.11952134	0.50677232

Time = 119.00 fs
E(CASSCF/6-31G(d)) = -479.5764534

C	-0.92843751	-0.22245159	-0.37849236
C	0.59767875	0.18269700	-0.15476213
C	1.21877549	1.36562889	-0.50902498
C	1.50195651	-0.75891000	0.37912633
C	2.80315767	-0.13220093	0.27270952
C	2.57839984	1.19443618	-0.18188085
N	-1.89480025	0.59694679	0.10224957
C	-3.16346735	-0.13082887	0.31591799
H	3.31674626	1.96370710	-0.35073018
C	-1.36643640	-1.62226129	-0.35426897
C	-2.91898414	-1.53134038	-0.22688712
H	-3.36818456	-1.64500152	-1.20432892
H	-3.36646585	-2.28063095	0.41229629
H	-3.31817962	-0.07922537	1.39563849
H	-3.94262951	0.45178938	-0.17585814
H	-1.06290885	-2.17779188	-1.23828767
H	0.82187022	2.28195157	-0.90483551
C	-1.81936145	2.03932408	0.16593876
H	-0.89259860	2.35056144	0.60844564
H	-1.87745169	2.45226765	-0.83683709
H	-2.66665365	2.33632931	0.75570017
H	1.38776335	-1.79575173	0.63732618
C	4.11230681	-0.86353037	0.20012817
H	4.52244224	-0.83940662	-0.81058914
H	4.88912206	-0.38630920	0.80231915
H	4.01905546	-1.89886060	0.50943328
H	-0.92396140	-2.11650282	0.50066627

Time = 119.50 fs
E(CASSCF/6-31G(d)) = -479.5750544

C	-0.92963383	-0.22628623	-0.38188635
C	0.60297066	0.18321899	-0.15605999
C	1.21886395	1.36383837	-0.51350995
C	1.49957964	-0.75437862	0.38169462
C	2.80283067	-0.12937083	0.27456181
C	2.57915940	1.19438658	-0.18380276
N	-1.89687767	0.59755156	0.10431884
C	-3.16111967	-0.13289287	0.31701089
H	3.31844730	1.96384356	-0.35302108

C	-1.36848200	-1.62124342	-0.35857337
C	-2.92075362	-1.53076681	-0.22632002
H	-3.36735676	-1.64184022	-1.20587146
H	-3.37028961	-2.27787608	0.41277913
H	-3.31322629	-0.08190512	1.39533922
H	-3.93921315	0.45231604	-0.16429492
H	-1.05916571	-2.17203241	-1.24543578
H	0.82021927	2.27682645	-0.91067182
C	-1.81818005	2.03753437	0.16863155
H	-0.89275588	2.35663123	0.60897179
H	-1.88719460	2.45840190	-0.83092941
H	-2.66230687	2.33326665	0.76323648
H	1.38391245	-1.79288452	0.64176421
C	4.11225018	-0.86551409	0.20325168
H	4.52357827	-0.84302871	-0.80721794
H	4.89303293	-0.38496533	0.79773025
H	4.01841881	-1.90000239	0.51613639
H	-0.92192155	-2.11374896	0.49495676

Time = 120.00 fs
E(CASSCF/6-31G(d)) = -479.5736625

C	-0.93061252	-0.22957129	-0.38516679
C	0.60766773	0.18337103	-0.15746756
C	1.21916415	1.36219123	-0.51799297
C	1.49761358	-0.74970784	0.38430491
C	2.80228239	-0.12675980	0.27647779
C	2.57996992	1.19453540	-0.18584873
N	-1.89872458	0.59777675	0.10626826
C	-3.15868049	-0.13463447	0.31823892
H	3.32032603	1.96326500	-0.35519344
C	-1.37072523	-1.62040000	-0.36293111
C	-2.92245304	-1.53020171	-0.22581775
H	-3.36667881	-1.63848848	-1.20749743
H	-3.37364687	-2.27538854	0.41336804
H	-3.30930872	-0.08550469	1.39466596
H	-3.93633739	0.45185076	-0.15269426
H	-1.05534934	-2.16607024	-1.25220308
H	0.81795113	2.27129437	-0.91654946
C	-1.81705347	2.03568249	0.17131000
H	-0.89303295	2.36271107	0.60996601
H	-1.89729491	2.46473503	-0.82491261
H	-2.65810803	2.33149740	0.77106868
H	1.37915575	-1.78952353	0.64592611
C	4.11227476	-0.86740218	0.20638357
H	4.52419022	-0.84721656	-0.80391336
H	4.89648535	-0.38394579	0.79366384

H	4.01785659	-1.90105908	0.52292399
H	-0.91979751	-2.11132359	0.48964974

Time = 120.50 fs
E(CASSCF/6-31G(d)) = -479.5723978

C	-0.93137846	-0.23224535	-0.38831289
C	0.61171063	0.18315414	-0.15899940
C	1.21969182	1.36069606	-0.52247342
C	1.49608530	-0.74494219	0.38697469
C	2.80152723	-0.12438305	0.27846198
C	2.58083162	1.19488992	-0.18802240
N	-1.90029451	0.59759449	0.10807350
C	-3.15615246	-0.13604958	0.31961989
H	3.32235694	1.96196552	-0.35725734
C	-1.37315422	-1.61975070	-0.36733876
C	-2.92407576	-1.52964821	-0.22540207
H	-3.36619674	-1.63496327	-1.20912952
H	-3.37652744	-2.27323071	0.41405509
H	-3.30642826	-0.09005894	1.39382733
H	-3.93426364	0.45053049	-0.14128869
H	-1.05158317	-2.15994352	-1.25850924
H	0.81503435	2.26549359	-0.92252823
C	-1.81600064	2.03379607	0.17398064
H	-0.89339724	2.36865488	0.61136625
H	-1.90768145	2.47115179	-0.81876877
H	-2.65406032	2.33098988	0.77920703
H	1.37354171	-1.78550856	0.64977781
C	4.11237355	-0.86918422	0.20951980
H	4.52421682	-0.85200908	-0.80070886
H	4.89945416	-0.38326246	0.79020846
H	4.01737240	-1.90198733	0.52979640
H	-0.91762021	-2.10926719	0.48471641

Time = 121.00 fs
E(CASSCF/6-31G(d)) = -479.5713715

C	-0.93193780	-0.23425993	-0.39130586
C	0.61505095	0.18257736	-0.16067018
C	1.22045731	1.35935621	-0.52695044
C	1.49501427	-0.74012457	0.38972023
C	2.80058239	-0.12225117	0.28051754
C	2.58174236	1.19545073	-0.19032523
N	-1.90154729	0.59698620	0.10971406
C	-3.15353967	-0.13714071	0.32116761
H	3.32454431	1.95997973	-0.35923216
C	-1.37575363	-1.61931021	-0.37178853

C	-2.92561606	-1.52910925	-0.22509352
H	-3.36595449	-1.63128448	-1.21068833
H	-3.37892287	-2.27145206	0.41482288
H	-3.30456639	-0.09558158	1.39304889
H	-3.93322954	0.44850772	-0.13030150
H	-1.04798150	-2.15371124	-1.26430764
H	0.81144836	2.25956744	-0.92866865
C	-1.81504173	2.03190440	0.17665280
H	-0.89382525	2.37431442	0.61309579
H	-1.91827504	2.47752902	-0.81247440
H	-2.65013104	2.33167124	0.78763221
H	1.36713432	-1.78072285	0.65330024
C	4.11253687	-0.87085215	0.21265732
H	4.52361163	-0.85743314	-0.79764126
H	4.90193110	-0.38291436	0.78744520
H	4.01696755	-1.90274606	0.53675462
H	-0.91543992	-2.10759761	0.48009594

Time = 121.50 fs
E(CASSCF/6-31G(d)) = -479.5706776

C	-0.93229779	-0.23558025	-0.39412909
C	0.61765172	0.18165726	-0.16249432
C	1.22146500	1.35817054	-0.53142327
C	1.49441203	-0.73529447	0.39255582
C	2.79946758	-0.12037002	0.28264627
C	2.58269774	1.19621191	-0.19275647
N	-1.90244948	0.59594286	0.11117362
C	-3.15084923	-0.13791577	0.32289194
H	3.32692077	1.95738064	-0.36114600
C	-1.37850503	-1.61908742	-0.37626864
C	-2.92706913	-1.52858768	-0.22491071
H	-3.36599493	-1.62747491	-1.21209799
H	-3.38082532	-2.27008694	0.41564365
H	-3.30368388	-0.10206460	1.39255560
H	-3.93342616	0.44593410	-0.11993150
H	-1.04464687	-2.14745294	-1.26958820
H	0.80718890	2.25365173	-0.93502653
C	-1.81419763	2.03003736	0.17933839
H	-0.89429947	2.37954312	0.61506629
H	-1.92899027	2.48373950	-0.80600540
H	-2.64625753	2.33343040	0.79629880
H	1.36001128	-1.77510484	0.65649277
C	4.11275265	-0.87240006	0.21579405
H	4.52234352	-0.86350296	-0.79474841
H	4.90392391	-0.38288828	0.78544479
H	4.01664042	-1.90329890	0.54380099

H	-0.91332343	-2.10631273	0.47570130

Time = 122.00 fs
E(CASSCF/6-31G(d)) = -479.5703851

C	-0.93246663	-0.23618558	-0.39676829
C	0.61948769	0.18041731	-0.16448562
C	1.22271291	1.35713416	-0.53589163
C	1.49428197	-0.73048654	0.39549323
C	2.79820466	-0.11874076	0.28484848
C	2.58369152	1.19716150	-0.19531323
N	-1.90297482	0.59446511	0.11244031
C	-3.14809288	-0.13838686	0.32479901
H	3.32954312	1.95427448	-0.36303441
C	-1.38138729	-1.61908494	-0.38076405
C	-2.92843101	-1.52808620	-0.22486969
H	-3.36636064	-1.62356005	-1.21329134
H	-3.38222682	-2.26915366	0.41647944
H	-3.30372035	-0.10947770	1.39255302
H	-3.93497680	0.44294490	-0.11033995
H	-1.04166844	-2.14126671	-1.27437761
H	0.80227352	2.24786291	-0.94164741
C	-1.81348916	2.02822427	0.18205148
H	-0.89480477	2.38420021	0.61718178
H	-1.93973728	2.48965594	-0.79934207
H	-2.64235628	2.33612298	0.80514082
H	1.35226206	-1.76865524	0.65937507
C	4.11300681	-0.87382412	0.21892876
H	4.52039668	-0.87021951	-0.79206595
H	4.90545449	-0.38316010	0.78426461
H	4.01638607	-1.90361637	0.55093953
H	-0.91135005	-2.10539479	0.47142744

Time = 122.50 fs
E(CASSCF/6-31G(d)) = -479.5705290

C	-0.93245327	-0.23606937	-0.39921172
C	0.62054560	0.17888699	-0.16665691
C	1.22419239	1.35623945	-0.54035613
C	1.49461900	-0.72572966	0.39854121
C	2.79681743	-0.11736000	0.28712307
C	2.58471615	1.19828229	-0.19799068
N	-1.90310442	0.59256309	0.11350683
C	-3.14528819	-0.13856905	0.32689175
H	3.33248536	1.95079257	-0.36493848
C	-1.38437709	-1.61929869	-0.38525750
C	-2.92969859	-1.52760736	-0.22498344

H	-3.36709450	-1.61956774	-1.21421445
H	-3.38311920	-2.26865438	0.41728300
H	-3.30459418	-0.11776862	1.39320892
H	-3.93791972	0.43964578	-0.10163985
H	-1.03912112	-2.13526565	-1.27873678
H	0.79674592	2.24228784	-0.94856199
C	-1.81293629	2.02649270	0.18480769
H	-0.89532406	2.38815515	0.61934241
H	-1.95042444	2.49515554	-0.79247432
H	-2.63833404	2.33957770	0.81408002
H	1.34398599	-1.76143871	0.66198778
C	4.11328372	-0.87512225	0.22206109
H	4.51776992	-0.87757060	-0.78962413
H	4.90655640	-0.38369655	0.78394561
H	4.01619572	-1.90367757	0.55817659
H	-0.90960621	-2.10481550	0.46716085

Time = 123.00 fs
E(CASSCF/6-31G(d)) = -479.5711075

C	-0.93226714	-0.23523907	-0.40145040
C	0.62082411	0.17710072	-0.16901951
C	1.22588823	1.35547680	-0.54481857
C	1.49540943	-0.72104650	0.40170525
C	2.79533131	-0.11621996	0.28946754
C	2.58576355	1.19955291	-0.20078228
N	-1.90282695	0.59025624	0.11437066
C	-3.14245923	-0.13847970	0.32917066
H	3.33582948	1.94708090	-0.36690247
C	-1.38744954	-1.61971804	-0.38973077
C	-2.93086944	-1.52715336	-0.22526164
H	-3.36823962	-1.61552764	-1.21482992
H	-3.38349459	-2.26857646	0.41799972
H	-3.30620297	-0.12686334	1.39463756
H	-3.94219824	0.43610511	-0.09389040
H	-1.03706569	-2.12957292	-1.28275596
H	0.79067924	2.23697714	-0.95578281
C	-1.81255719	2.02486722	0.18762349
H	-0.89583441	2.39129179	0.62144851
H	-1.96096069	2.50012435	-0.78540600
H	-2.63409942	2.34360386	0.82303446
H	1.33529029	-1.75358058	0.66439167
C	4.11356675	-0.87629399	0.22519137
H	4.51447568	-0.88553088	-0.78744433
H	4.90727167	-0.38445738	0.78450956
H	4.01605661	-1.90347118	0.56552103
H	-0.90817970	-2.10454170	0.46278957

Time = 123.50 fs
E(CASSCF/6-31G(d)) = -479.5720801

C	-0.93191785	-0.23371561	-0.40347841
C	0.62033360	0.17509675	-0.17158286
C	1.22777891	1.35483532	-0.54928216
C	1.49663099	-0.71645362	0.40498749
C	2.79377322	-0.11530891	0.29187805
C	2.58682588	1.20094919	-0.20368012
N	-1.90213871	0.58757287	0.11503411
C	-3.13963652	-0.13813805	0.33163464
H	3.33965457	1.94328820	-0.36897086
C	-1.39057872	-1.62032610	-0.39416603
C	-2.93194179	-1.52672596	-0.22571051
H	-3.36983902	-1.61147058	-1.21511890
H	-3.38334646	-2.26889455	0.41857035
H	-3.30842462	-0.13666647	1.39688862
H	-3.94765977	0.43235257	-0.08709645
H	-1.03554998	-2.12431584	-1.28654752
H	0.78417716	2.23194263	-0.96330266
C	-1.81236742	2.02336827	0.19051540
H	-0.89630378	2.39351224	0.62340403
H	-1.97125843	2.50446136	-0.77815830
H	-2.62957370	2.34799919	0.83192745
H	1.32628840	-1.74525815	0.66666526
C	4.11383877	-0.87734016	0.22832060
H	4.51053870	-0.89406239	-0.78553597
H	4.90764735	-0.38539785	0.78595666
H	4.01595193	-1.90299573	0.57298436
H	-0.90715380	-2.10454090	0.45821280

Time = 124.00 fs
E(CASSCF/6-31G(d)) = -479.5733678

C	-0.93141501	-0.23153270	-0.40529312
C	0.61909610	0.17291603	-0.17435401
C	1.22983728	1.35430321	-0.55375155
C	1.49825282	-0.71196206	0.40838681
C	2.79217129	-0.11461140	0.29434944
C	2.58789643	1.20244548	-0.20667534
N	-1.90104357	0.58454952	0.11550428
C	-3.13685619	-0.13756550	0.33428182
H	3.34402530	1.93955336	-0.37118560
C	-1.39373827	-1.62110047	-0.39854722
C	-2.93291447	-1.52632625	-0.22633292
H	-3.37193458	-1.60742786	-1.21508154

H	-3.38267083	-2.26957394	0.41893411
H	-3.31111964	-0.14706200	1.39994256
H	-3.95406480	0.42838447	-0.08121339
H	-1.03461075	-2.11961898	-1.29023677
H	0.77737230	2.22716004	-0.97109530
C	-1.81237917	2.02201133	0.19349924
H	-0.89668893	2.39474020	0.62512011
H	-1.98123554	2.50808209	-0.77077204
H	-2.62470051	2.35255810	0.84069525
H	1.31709884	-1.73668795	0.66890103
C	4.11408271	-0.87826273	0.23145031
H	4.50599467	-0.90311485	-0.78389357
H	4.90773174	-0.38647121	0.78826401
H	4.01586090	-1.90225914	0.58058009
H	-0.90660200	-2.10478583	0.45334885

Time = 124.50 fs
E(CASSCF/6-31G(d)) = -479.5748688

C	-0.93076786	-0.22873585	-0.40689555
C	0.61714471	0.17060111	-0.17733729
C	1.23203139	1.35386787	-0.55823270
C	1.50023576	-0.70757845	0.41189918
C	2.79055469	-0.11410875	0.29687538
C	2.58897044	1.20401603	-0.20975845
N	-1.89955287	0.58123035	0.11579306
C	-3.13415832	-0.13678599	0.33711012
H	3.34898058	1.93599375	-0.37358331
C	-1.39690185	-1.62201408	-0.40286140
C	-2.93378682	-1.52595443	-0.22712864
H	-3.37456566	-1.60343046	-1.21473601
H	-3.38146788	-2.27057398	0.41903227
H	-3.31413400	-0.15791453	1.40371307
H	-3.96110403	0.42417394	-0.07615590
H	-1.03427637	-2.11559712	-1.29395174
H	0.77042255	2.22257536	-0.97911820
C	-1.81260059	2.02080612	0.19658945
H	-0.89693441	2.39492340	0.62651736
H	-1.99081789	2.51092153	-0.76330832
H	-2.61945278	2.35707936	0.84929382
H	1.30784457	-1.72810841	0.67120058
C	4.11428202	-0.87906466	0.23458257
H	4.50088865	-0.91262663	-0.78249519
H	4.90757116	-0.38763104	0.79138471
H	4.01575913	-1.90127759	0.58832343
H	-0.90658329	-2.10525820	0.44814170

Time = 125.00 fs
E(CASSCF/6-31G(d)) = -479.5764651

C	-0.92998494	-0.22538133	-0.40829056
C	0.61452315	0.16819517	-0.18053403
C	1.23432562	1.35351575	-0.56273264
C	1.50253261	-0.70330653	0.41551806
C	2.78895338	-0.11377935	0.29944838
C	2.59004575	1.20563616	-0.21291967
N	-1.89768531	0.57766628	0.11591709
C	-3.13158474	-0.13582675	0.34011752
H	3.35452384	1.93269606	-0.37619317
C	-1.40004362	-1.62303636	-0.40709986
C	-2.93455881	-1.52560963	-0.22809471
H	-3.37776733	-1.59950817	-1.21411611
H	-3.37974344	-2.27185178	0.41881154
H	-3.31730327	-0.16907133	1.40805665
H	-3.96842191	0.41968440	-0.07180933
H	-1.03456976	-2.11234831	-1.29781239
H	0.76350534	2.21811428	-0.98731677
C	-1.81303531	2.01975621	0.19979841
H	-0.89697327	2.39403528	0.62752760
H	-1.99994139	2.51293612	-0.75584761
H	-2.61383706	2.36137326	0.85770384
H	1.29865323	-1.71975991	0.67366884
C	4.11442105	-0.87974977	0.23771992
H	4.49527382	-0.92252603	-0.78130205
H	4.90720739	-0.38883327	0.79524799
H	4.01561917	-1.90007377	0.59623051
H	-0.90713856	-2.10595146	0.44256527

Time = 125.50 fs
E(CASSCF/6-31G(d)) = -479.5780438

C	-0.92907383	-0.22153485	-0.40948709
C	0.61128483	0.16574102	-0.18394233
C	1.23668208	1.35323217	-0.56725909
C	1.50508875	-0.69914868	0.41923505
C	2.78739782	-0.11359925	0.30205998
C	2.59112313	1.20728318	-0.21614933
N	-1.89546673	0.57391407	0.11589751
C	-3.12917656	-0.13471873	0.34330193
H	3.36061740	1.92970993	-0.37903516
C	-1.40313861	-1.62413444	-0.41125914
C	-2.93523097	-1.52528973	-0.22922594
H	-3.38156825	-1.59568868	-1.21326755
H	-3.37751022	-2.27336589	0.41822732

H	-3.32045734	-0.18036520	1.41278763
H	-3.97564293	0.41488370	-0.06804204
H	-1.03551145	-2.10994775	-1.30192040
H	0.75680890	2.21369458	-0.99563036
C	-1.81368209	2.01885881	0.20313596
H	-0.89672945	2.39207520	0.62809463
H	-2.00855353	2.51410455	-0.74848695
H	-2.60789531	2.36526748	0.86593355
H	1.28965760	-1.71186400	0.67640795
C	4.11448538	-0.88032268	0.24086544
H	4.48920999	-0.93273244	-0.78025899
H	4.90667549	-0.39003757	0.79976034
H	4.01541111	-1.89867457	0.60431749
H	-0.90828817	-2.10687203	0.43662559

Time = 126.00 fs
E(CASSCF/6-31G(d)) = -479.5794996

C	-0.92804093	-0.21727019	-0.41049823
C	0.60749202	0.16328033	-0.18755699
C	1.23906206	1.35300098	-0.57182003
C	1.50784287	-0.69510759	0.42304049
C	2.78591862	-0.11354254	0.30470081
C	2.59220629	1.20893691	-0.21943807
N	-1.89292981	0.57003527	0.11575983
C	-3.12697154	-0.13349699	0.34666074
H	3.36718018	1.92704639	-0.38211958
C	-1.40616304	-1.62527436	-0.41534158
C	-2.93580460	-1.52499133	-0.23051550
H	-3.38598834	-1.59199683	-1.21224341
H	-3.37478880	-2.27507941	0.41724638
H	-3.32342620	-0.19161838	1.41769704
H	-3.98239815	0.40975674	-0.06471738
H	-1.03712198	-2.10844290	-1.30635057
H	0.75052365	2.20923805	-1.00399831
C	-1.81453465	2.01810494	0.20660896
H	-0.89612166	2.38906789	0.62817421
H	-2.01661450	2.51442759	-0.74133559
H	-2.60170372	2.36861208	0.87401959
H	1.28099664	-1.70460420	0.67951141
C	4.11446196	-0.88078886	0.24402279
H	4.48276256	-0.94315807	-0.77929662
H	4.90600288	-0.39120825	0.80480780
H	4.01510344	-1.89710854	0.61259940
H	-0.91003093	-2.10803933	0.43036044

Time = 126.50 fs

E(CASSCF/6-31G(d)) = -479.5807485

C	-0.92689127	-0.21266779	-0.41134123
C	0.60321481	0.16085292	-0.19136953
C	1.24142750	1.35280427	-0.57642329
C	1.51072784	-0.69118758	0.42692410
C	2.78454608	-0.11358173	0.30736071
C	2.59330150	1.21057979	-0.22277695
N	-1.89011366	0.56609498	0.11553353
C	-3.12500182	-0.13220058	0.35019026
H	3.37409026	1.92468046	-0.38544747
C	-1.40909459	-1.62642224	-0.41935552
C	-2.93628180	-1.52470970	-0.23195545
H	-3.39103648	-1.58845389	-1.21109903
H	-3.37160801	-2.27696238	0.41584867
H	-3.32604579	-0.20264746	1.42257264
H	-3.98834927	0.40431550	-0.06170424
H	-1.03942357	-2.10785052	-1.31114480
H	0.74483302	2.20468180	-1.01236594
C	-1.81558163	2.01747997	0.21022104
H	-0.89506879	2.38506182	0.62773344
H	-2.02409776	2.51392664	-0.73450907
H	-2.59536895	2.37128301	0.88202547
H	1.27281612	-1.69811057	0.68305898
C	4.11433919	-0.88115461	0.24719628
H	4.47600155	-0.95370981	-0.77833456
H	4.90520945	-0.39231455	0.81025926
H	4.01466401	-1.89540340	0.62108915
H	-0.91234441	-2.10948457	0.42383676

Time = 127.00 fs
E(CASSCF/6-31G(d)) = -479.5817315

C	-0.92562851	-0.20781315	-0.41203730
C	0.59852996	0.15849617	-0.19536831
C	1.24374238	1.35262224	-0.58107603
C	1.51367195	-0.68739551	0.43087547
C	2.78330976	-0.11368815	0.31002886
C	2.59441682	1.21219656	-0.22615759
N	-1.88706329	0.56216060	0.11525169
C	-3.12329187	-0.13087215	0.35388499
H	3.38119186	1.92255770	-0.38901195
C	-1.41191264	-1.62754527	-0.42331503
C	-2.93666568	-1.52443887	-0.23353731
H	-3.39670829	-1.58507698	-1.20988688
H	-3.36800481	-2.27899346	0.41402846
H	-3.32816391	-0.21326882	1.42721824

H	-3.99320899	0.39860569	-0.05888573
H	-1.04244041	-2.10815565	-1.31630921
H	0.73990460	2.19998750	-1.02069022
C	-1.81680681	2.01696429	0.21397245
H	-0.89349632	2.38012673	0.62674948
H	-2.03099000	2.51264131	-0.72812191
H	-2.58902189	2.37318410	0.89003786
H	1.26526812	-1.69245042	0.68711324
C	4.11410685	-0.88142716	0.25039092
H	4.46900092	-0.96429129	-0.77728569
H	4.90430846	-0.39333029	0.81597076
H	4.01406096	-1.89358353	0.62979632
H	-0.91518666	-2.11124798	0.41714569

Time = 127.50 fs
E(CASSCF/6-31G(d)) = -479.5824070

C	-0.92425511	-0.20279521	-0.41261125
C	0.59351974	0.15624460	-0.19953875
C	1.24597425	1.35243312	-0.58578444
C	1.51660031	-0.68374091	0.43488444
C	2.78223774	-0.11383225	0.31269390
C	2.59556097	1.21377377	-0.22957214
N	-1.88382885	0.55830036	0.11495040
C	-3.12185701	-0.12955715	0.35773695
H	3.38830519	1.92060390	-0.39280016
C	-1.41459851	-1.62861270	-0.42723923
C	-2.93696052	-1.52417181	-0.23525252
H	-3.40298448	-1.58187864	-1.20865181
H	-3.36402371	-2.28116045	0.41179429
H	-3.32964560	-0.22330464	1.43147036
H	-3.99675669	0.39270994	-0.05616560
H	-1.04619778	-2.10931310	-1.32181513
H	0.73588357	2.19514752	-1.02894291
C	-1.81818944	2.01653451	0.21786010
H	-0.89134350	2.37435046	0.62520789
H	-2.03729076	2.51062617	-0.72227940
H	-2.58280924	2.37424732	0.89816068
H	1.25851006	-1.68762621	0.69171748
C	4.11375606	-0.88161471	0.25361257
H	4.46183778	-0.97480548	-0.77606121
H	4.90330866	-0.39423306	0.82179043
H	4.01326370	-1.89166841	0.63872654
H	-0.91849941	-2.11337571	0.41039568

Time = 128.00 fs
E(CASSCF/6-31G(d)) = -479.5827777

C	-0.92277260	-0.19770469	-0.41309100
C	0.58827039	0.15412933	-0.20386356
C	1.24809519	1.35221338	-0.59055343
C	1.51943681	-0.68023570	0.43894135
C	2.78135597	-0.11398425	0.31534411
C	2.59674224	1.21529912	-0.23301330
N	-1.88046479	0.55458188	0.11466813
C	-3.12070220	-0.12830286	0.36173524
H	3.39524040	1.91873572	-0.39679567
C	-1.41713553	-1.62959642	-0.43115104
C	-2.93717186	-1.52390067	-0.23709278
H	-3.40982949	-1.57886652	-1.20742750
H	-3.35971545	-2.28345999	0.40916817
H	-3.33037777	-0.23258862	1.43521185
H	-3.99884947	0.38674840	-0.05347303
H	-1.05071962	-2.11125033	-1.32760291
H	0.73288502	2.19018915	-1.03711497
C	-1.81970483	2.01616444	0.22187771
H	-0.88857044	2.36783523	0.62310115
H	-2.04301136	2.50794639	-0.71706906
H	-2.57688297	2.37443153	0.90650695
H	1.25269960	-1.68358052	0.69689599
C	4.11327905	-0.88172642	0.25686787
H	4.45459172	-0.98515676	-0.77457569
H	4.90221626	-0.39500298	0.82756369
H	4.01224393	-1.88967077	0.64788055
H	-0.92221230	-2.11591428	0.40370360

Time = 128.50 fs
E(CASSCF/6-31G(d)) = -479.5828697

C	-0.92118208	-0.19263223	-0.41350687
C	0.58287021	0.15217778	-0.20832326
C	1.25008282	1.35193838	-0.59538641
C	1.52210653	-0.67689333	0.44303723
C	2.78068734	-0.11411498	0.31796784
C	2.59796739	1.21676112	-0.23647437
N	-1.87702880	0.55107060	0.11444492
C	-3.11982147	-0.12715709	0.36586572
H	3.40181197	1.91687139	-0.40098106
C	-1.41950914	-1.63047155	-0.43507575
C	-2.93730659	-1.52361703	-0.23905030
H	-3.41719096	-1.57604310	-1.20623411
H	-3.35513528	-2.28589604	0.40618365
H	-3.33027262	-0.24097145	1.43838112
H	-3.99942819	0.38087650	-0.05076523

H	-1.05602540	-2.11387209	-1.33358878
H	0.73098931	2.18517470	-1.04521829
C	-1.82132520	2.01582633	0.22601620
H	-0.88516451	2.36069371	0.62042717
H	-2.04817359	2.50467305	-0.71255246
H	-2.57138843	2.37371942	0.91518876
H	1.24798766	-1.68020752	0.70265578
C	4.11266909	-0.88177235	0.26016424
H	4.44734406	-0.99525320	-0.77275195
H	4.90103752	-0.39562122	0.83313837
H	4.01097696	-1.88759602	0.65725385
H	-0.92624823	-2.11890480	0.39718469

Time = 129.00 fs
E(CASSCF/6-31G(d)) = -479.5826775

C	-0.91948473	-0.18766705	-0.41389066
C	0.57740989	0.15041367	-0.21289625
C	1.25192053	1.35158209	-0.60028530
C	1.52453654	-0.67372724	0.44716323
C	2.78025123	-0.11419486	0.32055305
C	2.59924038	1.21814714	-0.23994865
N	-1.87358031	0.54782838	0.11432143
C	-3.11919828	-0.12616721	0.37011077
H	3.40784806	1.91494263	-0.40534043
C	-1.42170695	-1.63121685	-0.43903923
C	-2.93737315	-1.52331230	-0.24111761
H	-3.42500029	-1.57340631	-1.20507730
H	-3.35034118	-2.28847766	0.40288323
H	-3.32927100	-0.24832567	1.44097734
H	-3.99851967	0.37527992	-0.04802784
H	-1.06212629	-2.11706706	-1.33967416
H	0.73024374	2.18019913	-1.05328327
C	-1.82302055	2.01549250	0.23026411
H	-0.88114509	2.35304705	0.61718917
H	-2.05280846	2.50088091	-0.70875858
H	-2.56645367	2.37211451	0.92430744
H	1.24451650	-1.67736809	0.70898882
C	4.11192011	-0.88176342	0.26350991
H	4.44017681	-1.00500972	-0.77052650
H	4.89978167	-0.39606922	0.83837107
H	4.00944112	-1.88544254	0.66683678
H	-0.93052903	-2.12237869	0.39094242

Time = 129.50 fs
E(CASSCF/6-31G(d)) = -479.5822824

C	-0.91768276	-0.18289486	-0.41427478
C	0.57197990	0.14885669	-0.21755946
C	1.25359799	1.35111831	-0.60525056
C	1.52665906	-0.67074940	0.45131079
C	2.78006238	-0.11419532	0.32308793
C	2.60056159	1.21944362	-0.24342971
N	-1.87017893	0.54491178	0.11433791
C	-3.11880582	-0.12537866	0.37444944
H	3.41320386	1.91290199	-0.40986154
C	-1.42371871	-1.63181492	-0.44306620
C	-2.93738137	-1.52297808	-0.24328756
H	-3.43317420	-1.57094916	-1.20394913
H	-3.34539135	-2.29121630	0.39931528
H	-3.32734306	-0.25454940	1.44306167
H	-3.99623282	0.37016827	-0.04527369
H	-1.06902139	-2.12071390	-1.34575531
H	0.73066137	2.17538388	-1.06135775
C	-1.82475987	2.01513623	0.23460830
H	-0.87656598	2.34502118	0.61339490
H	-2.05695504	2.49664387	-0.70567846
H	-2.56217696	2.36963602	0.93394247
H	1.24240926	-1.67490732	0.71587632
C	4.11102672	-0.88171115	0.26691373
H	4.43317158	-1.01435022	-0.76785266
H	4.89846290	-0.39632718	0.84313105
H	4.00761920	-1.88320229	0.67661505
H	-0.93498148	-2.12635118	0.38505832

Time = 130.00 fs
E(CASSCF/6-31G(d)) = -479.5817539

C	-0.91578025	-0.17839623	-0.41469109
C	0.56666901	0.14752239	-0.22228880
C	1.25511104	1.35052148	-0.61028135
C	1.52841360	-0.66796891	0.45547138
C	2.78013011	-0.11408934	0.32556118
C	2.60192732	1.22063577	-0.24691138
N	-1.86688300	0.54237069	0.11453318
C	-3.11860819	-0.12483368	0.37885763
H	3.41777109	1.91072861	-0.41453735
C	-1.42553625	-1.63225230	-0.44717857
C	-2.93734239	-1.52260647	-0.24555298
H	-3.44161672	-1.56866011	-1.20283054
H	-3.34034207	-2.29412221	0.39553041
H	-3.32448859	-0.25956891	1.44475398
H	-3.99274980	0.36576630	-0.04253922
H	-1.07669426	-2.12468745	-1.35173298

H	0.73222285	2.17086900	-1.06950444
C	-1.82651228	2.01473278	0.23903462
H	-0.87151543	2.33674455	0.60905781
H	-2.06065900	2.49203248	-0.70326308
H	-2.55861750	2.36631426	0.94414121
H	1.24176133	-1.67267189	0.72329225
C	4.10998414	-0.88162741	0.27038498
H	4.42640859	-1.02320918	-0.76470337
H	4.89710205	-0.39637339	0.84730441
H	4.00549912	-1.88086232	0.68657010
H	-0.93954203	-2.13081617	0.37958344

Time = 130.50 fs
E(CASSCF/6-31G(d)) = -479.5811583

C	-0.91378402	-0.17424498	-0.41516981
C	0.56156323	0.14642232	-0.22705952
C	1.25646111	1.34976751	-0.61537589
C	1.52974870	-0.66539059	0.45963616
C	2.78045762	-0.11385163	0.32796211
C	2.60332953	1.22170716	-0.25038761
N	-1.86374764	0.54024693	0.11494346
C	-3.11856218	-0.12457001	0.38330846
H	3.42148297	1.90843190	-0.41936723
C	-1.42715350	-1.63251948	-0.45139400
C	-2.93726850	-1.52219042	-0.24790622
H	-3.45022243	-1.56652344	-1.20169493
H	-3.33524559	-2.29720121	0.39157799
H	-3.32073592	-0.26333994	1.44622557
H	-3.98831185	0.36230288	-0.03987824
H	-1.08511029	-2.12886491	-1.35752149
H	0.73488225	2.16680244	-1.07779517
C	-1.82824815	2.01426002	0.24352856
H	-0.86611289	2.32834684	0.60419839
H	-2.06397187	2.48711301	-0.70142397
H	-2.55578889	2.36218680	0.95491162
H	1.24263486	-1.67052511	0.73120696
C	4.10878826	-0.88152415	0.27393313
H	4.41996504	-1.03153369	-0.76107263
H	4.89572762	-0.39618388	0.85079741
H	4.00307399	-1.87840674	0.69668013
H	-0.94416046	-2.13574316	0.37453257

Time = 131.00 fs
E(CASSCF/6-31G(d)) = -479.5805777

C	-0.91170450	-0.17050671	-0.41573848

C	0.55674438	0.14556403	-0.23184661
C	1.25765436	1.34883484	-0.62053191
C	1.53062348	-0.66301401	0.46379579
C	2.78104147	-0.11345919	0.33028091
C	2.60475610	1.22263983	-0.25385241
N	-1.86082310	0.53857308	0.11560128
C	-3.11861956	-0.12461966	0.38777271
H	3.42431726	1.90605216	-0.42435755
C	-1.42856641	-1.63261061	-0.45572499
C	-2.93717284	-1.52172399	-0.25033878
H	-3.45888004	-1.56451951	-1.20051257
H	-3.33014842	-2.30045152	0.38750296
H	-3.31613956	-0.26584754	1.44768761
H	-3.98319933	0.35999816	-0.03735326
H	-1.09421498	-2.13313065	-1.36305598
H	0.73857331	2.16332782	-1.08630519
C	-1.82994010	2.01369884	0.24807595
H	-0.86050304	2.31995779	0.59884606
H	-2.06695046	2.48194728	-0.70003719
H	-2.55365646	2.35729572	0.96621790
H	1.24505315	-1.66835909	0.73959036
C	4.10743570	-0.88141296	0.27756764
H	4.41391373	-1.03928466	-0.75697552
H	4.89437590	-0.39573270	0.85353855
H	4.00034245	-1.87581894	0.70692122
H	-0.94880150	-2.14107535	0.36988160

Time = 131.50 fs
E(CASSCF/6-31G(d)) = -479.5800639

C	-0.90955663	-0.16723738	-0.41642090
C	0.55228932	0.14495139	-0.23662533
C	1.25870089	1.34770532	-0.62574715
C	1.53100893	-0.66083255	0.46794018
C	2.78187107	-0.11289131	0.33250865
C	2.60619111	1.22341414	-0.25729988
N	-1.85815287	0.53737145	0.11653444
C	-3.11873022	-0.12500766	0.39221945
H	3.42629371	1.90365850	-0.42952122
C	-1.42977314	-1.63252330	-0.46017834
C	-2.93706909	-1.52120258	-0.25284079
H	-3.46747694	-1.56262552	-1.19925516
H	-3.32509006	-2.30386170	0.38334333
H	-3.31077658	-0.26710450	1.44937531
H	-3.97770731	0.35904865	-0.03502401
H	-1.10393356	-2.13738109	-1.36829844
H	0.74321908	2.16057092	-1.09510414

C	-1.83156388	2.01303338	0.25266347
H	-0.85484711	2.31170688	0.59304102
H	-2.06965684	2.47659422	-0.69894968
H	-2.55213876	2.35168614	0.97798073
H	1.24899997	-1.66610237	0.74841404
C	4.10592391	-0.88130472	0.28129757
H	4.40832074	-1.04643795	-0.75244682
H	4.89309000	-0.39499243	0.85547984
H	3.99730809	-1.87308385	0.71726906
H	-0.95344466	-2.14673178	0.36556885

Time = 132.00 fs
E(CASSCF/6-31G(d)) = -479.5797098

C	-0.90736022	-0.16448239	-0.41723641
C	0.54826830	0.14458475	-0.24137137
C	1.25961321	1.34636531	-0.63101998
C	1.53088856	-0.65883349	0.47205847
C	2.78292869	-0.11213031	0.33463769
C	2.60761590	1.22400969	-0.26072427
N	-1.85577249	0.53665338	0.11776517
C	-3.11884496	-0.12575092	0.39661685
H	3.42747171	1.90134427	-0.43487727
C	-1.43077408	-1.63225805	-0.46475508
C	-2.93697102	-1.52062303	-0.25540073
H	-3.47590282	-1.56081560	-1.19790074
H	-3.32010238	-2.30740868	0.37912829
H	-3.30474199	-0.26714849	1.45152817
H	-3.97211775	0.35961189	-0.03293510
H	-1.11417151	-2.14152653	-1.37324034
H	0.74873794	2.15862720	-1.10425075
C	-1.83309875	2.01225061	0.25727895
H	-0.84931319	2.30372223	0.58683614
H	-2.07215804	2.47111098	-0.69798854
H	-2.55111352	2.34540659	0.99008086
H	1.25441620	-1.66372481	0.75765295
C	4.10425152	-0.88120923	0.28513133
H	4.40324418	-1.05298432	-0.74753818
H	4.89191842	-0.39393563	0.85659659
H	3.99398016	-1.87019015	0.72769971
H	-0.95808166	-2.15260879	0.36149953

Time = 132.50 fs
E(CASSCF/6-31G(d)) = -479.5795548

C	-0.90514016	-0.16227569	-0.41819914
C	0.54474424	0.14446117	-0.24606131

C	1.26040520	1.34480639	-0.63634991
C	1.53025868	-0.65699797	0.47613899
C	2.78418941	-0.11116157	0.33666165
C	2.60901004	1.22440566	-0.26412006
N	-1.85370817	0.53641875	0.11930937
C	-3.11891892	-0.12685726	0.40093325
H	3.42794161	1.89921989	-0.44044915
C	-1.43157206	-1.63181773	-0.46945096
C	-2.93689204	-1.51998364	-0.25800518
H	-3.48405405	-1.55906155	-1.19643752
H	-3.31520965	-2.31105781	0.37487763
H	-3.29814346	-0.26603823	1.45436776
H	-3.96667253	0.36179132	-0.03110304
H	-1.12481641	-2.14549317	-1.37790377
H	0.75505235	2.15755019	-1.11378485
C	-1.83452783	2.01134019	0.26191158
H	-0.84406515	2.29613025	0.58029792
H	-2.07452620	2.46555543	-0.69697113
H	-2.55042691	2.33851051	1.00236757
H	1.26120312	-1.66123669	0.76728591
C	4.10241861	-0.88113478	0.28907633
H	4.39873179	-1.05892947	-0.74231414
H	4.89091227	-0.39253634	0.85688649
H	3.99037286	-1.86713195	0.73819123
H	-0.96271259	-2.15858598	0.35755364

Time = 133.00 fs
E(CASSCF/6-31G(d)) = -479.5796438

C	-0.90292633	-0.16063936	-0.41931767
C	0.54177170	0.14457475	-0.25067287
C	1.26109111	1.34302596	-0.64173808
C	1.52912819	-0.65530161	0.48016942
C	2.78562161	-0.10997378	0.33857553
C	2.61035261	1.22458176	-0.26748218
N	-1.85197612	0.53665588	0.12117615
C	-3.11891423	-0.12832473	0.40513834
H	3.42781495	1.89740387	-0.44626267
C	-1.43217246	-1.63120704	-0.47425726
C	-2.93684473	-1.51928410	-0.26063882
H	-3.49183726	-1.55733327	-1.19486713
H	-3.31042915	-2.31476358	0.37060188
H	-3.29109572	-0.26384935	1.45807419
H	-3.96155005	0.36562342	-0.02950547
H	-1.13574075	-2.14922300	-1.38233974
H	0.76209394	2.15734313	-1.12372233
C	-1.83583807	2.01029397	0.26655192

H	-0.83925220	2.28905453	0.57350688
H	-2.07683861	2.45998813	-0.69571597
H	-2.54990538	2.33105851	1.01466962
H	1.26922553	-1.65868457	0.77729503
C	4.10042703	-0.88108787	0.29313869
H	4.39481917	-1.06429324	-0.73684718
H	4.89012208	-0.39077216	0.85636759
H	3.98650523	-1.86391015	0.74872472
H	-0.96734094	-2.16453251	0.35359572

Time = 133.50 fs
E(CASSCF/6-31G(d)) = -479.5799962

C	-0.90075295	-0.15958347	-0.42059486
C	0.53939591	0.14491670	-0.25518504
C	1.26168478	1.34102755	-0.64718748
C	1.52751770	-0.65371568	0.48413706
C	2.78718766	-0.10855908	0.34037584
C	2.61162359	1.22451928	-0.27080620
N	-1.85058221	0.53734171	0.12336763
C	-3.11880199	-0.13014124	0.40920468
H	3.42721244	1.89601245	-0.45234333
C	-1.43258336	-1.63043195	-0.47916186
C	-2.93684039	-1.51852535	-0.26328455
H	-3.49917212	-1.55559921	-1.19320662
H	-3.30577247	-2.31847152	0.36630355
H	-3.28371526	-0.26067032	1.46276432
H	-3.95684965	0.37106902	-0.02807443
H	-1.14680548	-2.15267233	-1.38662434
H	0.76980637	2.15795485	-1.13405184
C	-1.83702001	2.00910564	0.27119174
H	-0.83499949	2.28261442	0.56655695
H	-2.07917735	2.45447396	-0.69405330
H	-2.54936879	2.32312079	1.02680796
H	1.27831698	-1.65614319	0.78766382
C	4.09828074	-0.88107293	0.29732292
H	4.39152812	-1.06910838	-0.73121214
H	4.88959412	-0.38862655	0.85507579
H	3.98240096	-1.86053326	0.75928501
H	-0.97196826	-2.17031438	0.34948552

Time = 134.00 fs
E(CASSCF/6-31G(d)) = -479.5805979

C	-0.89865757	-0.15910623	-0.42202795
C	0.53765218	0.14547553	-0.25957839
C	1.26219939	1.33882057	-0.65270304

C	1.52545843	-0.65220828	0.48802918
C	2.78884483	-0.10691307	0.34206056
C	2.61280511	1.22420209	-0.27408865
N	-1.84952208	0.53844232	0.12587893
C	-3.11856332	-0.13228473	0.41310921
H	3.42625068	1.89514812	-0.45871320
C	-1.43281556	-1.62949925	-0.48415059
C	-2.93688863	-1.51770936	-0.26592381
H	-3.50599384	-1.55382694	-1.19148907
H	-3.30124698	-2.32212133	0.36197920
H	-3.27611584	-0.25659928	1.46847454
H	-3.95258676	0.37801057	-0.02669524
H	-1.15786450	-2.15580962	-1.39085277
H	0.77814589	2.15928013	-1.14473439
C	-1.83806742	2.00777036	0.27582386
H	-0.83140053	2.27692344	0.55955371
H	-2.08162911	2.44908330	-0.69183421
H	-2.54864327	2.31477928	1.03860951
H	1.28828715	-1.65370305	0.79837495
C	4.09598613	-0.88109209	0.30163172
H	4.38886483	-1.07341900	-0.72548035
H	4.88936621	-0.38609095	0.85306167
H	3.97808782	-1.85701721	0.76986146
H	-0.97658929	-2.17580233	0.34508905

Time = 134.50 fs
E(CASSCF/6-31G(d)) = -479.5814132

C	-0.89667972	-0.15919453	-0.42360878
C	0.53656516	0.14623695	-0.26383512
C	1.26264750	1.33641997	-0.65829141
C	1.52299053	-0.65074617	0.49183343
C	2.79054655	-0.10503464	0.34362908
C	2.61388259	1.22361774	-0.27732728
N	-1.84878171	0.53991374	0.12869843
C	-3.11818908	-0.13472370	0.41683465
H	3.42502972	1.89488888	-0.46538794
C	-1.43288257	-1.62841621	-0.48920860
C	-2.93699699	-1.51683890	-0.26853707
H	-3.51225435	-1.55198359	-1.18976309
H	-3.29685779	-2.32565046	0.35762183
H	-3.26840535	-0.25174222	1.47515103
H	-3.94869904	0.38625590	-0.02521194
H	-1.16876917	-2.15861172	-1.39513173
H	0.78707829	2.16116527	-1.15570585
C	-1.83897685	2.00628469	0.28044195
H	-0.82851230	2.27208733	0.55261196

H	-2.08428428	2.44389246	-0.68893825
H	-2.54757307	2.30612907	1.04991980
H	1.29892904	-1.65145643	0.80940669
C	4.09355222	-0.88114511	0.30606575
H	4.38681887	-1.07727877	-0.71971421
H	4.88946386	-0.38316699	0.85038724
H	3.97359738	-1.85338492	0.78044806
H	-0.98118763	-2.18087892	0.34028824

Time = 135.00 fs
E(CASSCF/6-31G(d)) = -479.5823785

C	-0.89485942	-0.15982462	-0.42532450
C	0.53614839	0.14718390	-0.26793918
C	1.26304144	1.33384536	-0.66396063
C	1.52016130	-0.64929644	0.49553849
C	2.79224374	-0.10292597	0.34508226
C	2.61484570	1.22275839	-0.28052143
N	-1.84833838	0.54170307	0.13180837
C	-3.11767865	-0.13741830	0.42037060
H	3.42362234	1.89527940	-0.47237350
C	-1.43280042	-1.62719043	-0.49432168
C	-2.93717077	-1.51591718	-0.27110445
H	-3.51792266	-1.55003634	-1.18809079
H	-3.29260956	-2.32899803	0.35322366
H	-3.26068397	-0.24621278	1.48264932
H	-3.94506239	0.39554828	-0.02343834
H	-1.17937297	-2.16105933	-1.39957088
H	0.79657370	2.16341817	-1.16688141
C	-1.83974704	2.00464644	0.28504024
H	-0.82635268	2.26820162	0.54585236
H	-2.08723601	2.43898311	-0.68527897
H	-2.54603031	2.29727865	1.06061414
H	1.31002623	-1.64948160	0.82072824
C	4.09099079	-0.88122933	0.31062353
H	4.38536244	-1.08074903	-0.71396257
H	4.88989695	-0.37986812	0.84712291
H	3.96896457	-1.84966506	0.79104340
H	-0.98573277	-2.18544501	0.33498895

Time = 135.50 fs
E(CASSCF/6-31G(d)) = -479.5834167

C	-0.89323533	-0.16096312	-0.42715799
C	0.53640400	0.14829644	-0.27187636
C	1.26339393	1.33111999	-0.66971950
C	1.51702329	-0.64782835	0.49913446

C	2.79388629	-0.10059216	0.34642229
C	2.61568876	1.22162151	-0.28367222
N	-1.84816180	0.54374974	0.13518547
C	-3.11703806	-0.14032173	0.42371408
H	3.42206709	1.89632394	-0.47966420
C	-1.43258743	-1.62582969	-0.49947750
C	-2.93741285	-1.51494768	-0.27360637
H	-3.52298415	-1.54795293	-1.18654463
H	-3.28850836	-2.33210885	0.34877871
H	-3.25304374	-0.24013298	1.49074399
H	-3.94151348	0.40558184	-0.02117328
H	-1.18953599	-2.16313274	-1.40427379
H	0.80660060	2.16582111	-1.17816328
C	-1.84037841	2.00285488	0.28961337
H	-0.82490155	2.26534943	0.53939756
H	-2.09057859	2.43444119	-0.68080665
H	-2.54392251	2.28834926	1.07060652
H	1.32135988	-1.64782834	0.83229639
C	4.08831641	-0.88133979	0.31530143
H	4.38445042	-1.08389676	-0.70825721
H	4.89065710	-0.37622082	0.84334470
H	3.96422720	-1.84589028	0.80165015
H	-0.99017814	-2.18942483	0.32912741

Time = 136.00 fs
E(CASSCF/6-31G(d)) = -479.5844449

C	-0.89184320	-0.16256805	-0.42908876
C	0.53732258	0.14955170	-0.27563428
C	1.26371877	1.32826954	-0.67557697
C	1.51363259	-0.64631473	0.50261345
C	2.79542445	-0.09804124	0.34765259
C	2.61641088	1.22021026	-0.28678275
N	-1.84821552	0.54598700	0.13880178
C	-3.11627759	-0.14338196	0.42686944
H	3.42036542	1.89798401	-0.48724092
C	-1.43226392	-1.62434207	-0.50466663
C	-2.93772372	-1.51393382	-0.27602434
H	-3.52743899	-1.54570206	-1.18520323
H	-3.28456319	-2.33493694	0.34428520
H	-3.24556892	-0.23363487	1.49914689
H	-3.93787489	0.41602000	-0.01821748
H	-1.19912872	-2.16480708	-1.40932955
H	0.81711904	2.16814590	-1.18944823
C	-1.84087243	2.00091094	0.29415618
H	-0.82410411	2.26359892	0.53336790
H	-2.09440570	2.43035475	-0.67550885

H	-2.54119713	2.27947287	1.07985574
H	1.33271388	-1.64650578	0.84405139
C	4.08554621	-0.88146935	0.32009374
H	4.38402083	-1.08679286	-0.70261114
H	4.89171656	-0.37226481	0.83913226
H	3.95942550	-1.84209506	0.81227430
H	-0.99446075	-2.19276976	0.32267377

Time = 136.50 fs
E(CASSCF/6-31G(d)) = -479.5853881

C	-0.89071418	-0.16459005	-0.43109363
C	0.53888339	0.15092406	-0.27920249
C	1.26403139	1.32532084	-0.68154141
C	1.51004713	-0.64473310	0.50596987
C	2.79681021	-0.09528379	0.34877781
C	2.61701550	1.21853348	-0.28985819
N	-1.84845835	0.54834349	0.14262555
C	-3.11540955	-0.14654351	0.42984764
H	3.41848397	1.90017889	-0.49507105
C	-1.43185189	-1.62273601	-0.50988326
C	-2.93810157	-1.51287880	-0.27834167
H	-3.53129973	-1.54325393	-1.18414636
H	-3.28078716	-2.33744854	0.33974732
H	-3.23833688	-0.22686177	1.50753186
H	-3.93397933	0.42651530	-0.01438995
H	-1.20803521	-2.16604914	-1.41480575
H	0.82807492	2.17016906	-1.20063685
C	-1.84123106	1.99881762	0.29866366
H	-0.82387747	2.26300087	0.52787691
H	-2.09880805	2.42681120	-0.66940800
H	-2.53784344	2.27078924	1.08836840
H	1.34387918	-1.64547555	0.85591540
C	4.08269953	-0.88160900	0.32499287
H	4.38399625	-1.08951038	-0.69701834
H	4.89302814	-0.36805255	0.83456735
H	3.95460157	-1.83831314	0.82292413
H	-0.99850195	-2.19546040	0.31563378

Time = 137.00 fs
E(CASSCF/6-31G(d)) = -479.5861877

C	-0.88987368	-0.16697353	-0.43314758
C	0.54105463	0.15238513	-0.28257244
C	1.26434942	1.32230064	-0.68762001
C	1.50632553	-0.64306613	0.50920065
C	2.79799845	-0.09233301	0.34980370

C	2.61750967	1.21660538	-0.29290573
N	-1.84884576	0.55074483	0.14662216
C	-3.11444626	-0.14974941	0.43266509
H	3.41636121	1.90279120	-0.50310921
C	-1.43137455	-1.62102047	-0.51512553
C	-2.93854257	-1.51178540	-0.28054418
H	-3.53458818	-1.54058067	-1.18344949
H	-3.27719829	-2.33962419	0.33517633
H	-3.23141882	-0.21996900	1.51556214
H	-3.92969028	0.43672834	-0.00954216
H	-1.21615520	-2.16681470	-1.42074363
H	0.83939535	2.17168624	-1.21164112
C	-1.84145633	1.99658038	0.30313082
H	-0.82411887	2.26358634	0.52302687
H	-2.10387081	2.42389379	-0.66255687
H	-2.53389137	2.26244201	1.09619873
H	1.35465575	-1.64465034	0.86779174
C	4.07979741	-0.88174817	0.32998960
H	4.38428571	-1.09212333	-0.69145565
H	4.89452719	-0.36364758	0.82973330
H	3.94979873	-1.83457496	0.83360904
H	-1.00220957	-2.19750692	0.30804759

Time = 137.50 fs
E(CASSCF/6-31G(d)) = -479.5868083

C	-0.88934030	-0.16965777	-0.43522428
C	0.54379405	0.15390410	-0.28573747
C	1.26469305	1.31923457	-0.69381812
C	1.50252612	-0.64130137	0.51230516
C	2.79894780	-0.08920474	0.35073714
C	2.61790294	1.21444493	-0.29593452
N	-1.84933123	0.55311515	0.15075495
C	-3.11339841	-0.15294298	0.43534213
H	3.41391801	1.90567370	-0.51129889
C	-1.43085601	-1.61920508	-0.52039548
C	-2.93904112	-1.51065591	-0.28262075
H	-3.53733190	-1.53765687	-1.18317835
H	-3.27381969	-2.34145972	0.33059089
H	-3.22488029	-0.21312336	1.52291770
H	-3.92491761	0.44634495	-0.00356902
H	-1.22340531	-2.16704822	-1.42715598
H	0.85098511	2.17252537	-1.22239287
C	-1.84154987	1.99420770	0.30755273
H	-0.82471572	2.26536433	0.51890474
H	-2.10967056	2.42167762	-0.65503194
H	-2.52940785	2.25457394	1.10344505

H	1.36485365	-1.64390002	0.87956742
C	4.07686191	-0.88187508	0.33507348
H	4.38478705	-1.09470487	-0.68588547
H	4.89613393	-0.35912184	0.82471454
H	3.94506102	-1.83090485	0.84433810
H	-1.00548115	-2.19894737	0.29998629

Time = 138.00 fs
E(CASSCF/6-31G(d)) = -479.5872332

C	-0.88912535	-0.17257861	-0.43729721
C	0.54705034	0.15544851	-0.28869292
C	1.26508448	1.31614587	-0.70013899
C	1.49870548	-0.63943110	0.51528519
C	2.79962190	-0.08591680	0.35158589
C	2.61820625	1.21207476	-0.29895521
N	-1.84986745	0.55537910	0.15498638
C	-3.11227400	-0.15606988	0.43790149
H	3.41106923	1.90866301	-0.51957676
C	-1.43032067	-1.61730011	-0.52569861
C	-2.93959034	-1.50949208	-0.28456380
H	-3.53956091	-1.53446002	-1.18338356
H	-3.27067934	-2.34296632	0.32601656
H	-3.21878119	-0.20650125	1.52932049
H	-3.91962883	0.45509136	0.00358379
H	-1.22971969	-2.16668384	-1.43402758
H	0.86272799	2.17255498	-1.23284744
C	-1.84151280	1.99171140	0.31192458
H	-0.82555665	2.26832025	0.51557833
H	-2.11627203	2.42022546	-0.64692436
H	-2.52448926	2.24732149	1.11024249
H	1.37429460	-1.64306097	0.89111677
C	4.07391529	-0.88197729	0.34023328
H	4.38539097	-1.09732789	-0.68026139
H	4.89775969	-0.35455301	0.81959765
H	3.94043218	-1.82731967	0.85511933
H	-1.00820831	-2.19984478	0.29154610

Time = 138.50 fs
E(CASSCF/6-31G(d)) = -479.5874788

C	-0.88923253	-0.17566910	-0.43933982
C	0.55076364	0.15698436	-0.29143598
C	1.26554830	1.31305491	-0.70658326
C	1.49491841	-0.63745079	0.51814431
C	2.79998952	-0.08248966	0.35235883
C	2.61843061	1.20952043	-0.30197994

N	-1.85040744	0.55746298	0.15927856
C	-3.11107753	-0.15907954	0.44036667
H	3.40773763	1.91158794	-0.52787471
C	-1.42979286	-1.61531673	-0.53104296
C	-2.94018238	-1.50829514	-0.28636957
H	-3.54130428	-1.53097103	-1.18409664
H	-3.26780935	-2.34416911	0.32148439
H	-3.21317523	-0.20028588	1.53455420
H	-3.91385429	0.46274526	0.01191570
H	-1.23504946	-2.16564817	-1.44131886
H	0.87448506	2.17169286	-1.24299006
C	-1.84134566	1.98910729	0.31624189
H	-0.82654114	2.27241413	0.51309359
H	-2.12372473	2.41958310	-0.63833126
H	-2.51925302	2.24080906	1.11675323
H	1.38281087	-1.64195279	0.90230806
C	4.07097921	-0.88204204	0.34545745
H	4.38598346	-1.10006272	-0.67453217
H	4.89931026	-0.35002005	0.81447101
H	3.93595530	-1.82382619	0.86595816
H	-1.01028191	-2.20028138	0.28284005

Time = 139.00 fs
E(CASSCF/6-31G(d)) = -479.5875703

C	-0.88965818	-0.17886108	-0.44132639
C	0.55486745	0.15847716	-0.29396572
C	1.26611056	1.30997834	-0.71314896
C	1.49121691	-0.63535819	0.52088752
C	2.80002536	-0.07894593	0.35306571
C	2.61858604	1.20680921	-0.30502178
N	-1.85090551	0.55929657	0.16359422
C	-3.10980971	-0.16192684	0.44276054
H	3.40386525	1.91428345	-0.53612464
C	-1.42929623	-1.61326699	-0.53643796
C	-2.94080889	-1.50706593	-0.28803815
H	-3.54258780	-1.52717469	-1.18532679
H	-3.26524488	-2.34510491	0.31702908
H	-3.20810900	-0.19466339	1.53847960
H	-3.90768895	0.46914490	0.02137065
H	-1.23936214	-2.16386465	-1.44897124
H	0.88609927	2.16990835	-1.25283607
C	-1.84104875	1.98641535	0.32050085
H	-0.82758866	2.27758009	0.51147256
H	-2.13205946	2.41977520	-0.62934556
H	-2.51382584	2.23514280	1.12315333
H	1.39024701	-1.64039379	0.91301026

C	4.06807381	-0.88205673	0.35073457
H	4.38645022	-1.10297751	-0.66864799
H	4.90069291	-0.34559959	0.80942570
H	3.93167157	-1.82042065	0.87685704
H	-1.01159803	-2.20035245	0.27398857

Time = 139.50 fs
E(CASSCF/6-31G(d)) = -479.5875507

C	-0.89039170	-0.18208627	-0.44323251
C	0.55929010	0.15989265	-0.29628308
C	1.26679810	1.30692878	-0.71983153
C	1.48764970	-0.63315198	0.52352060
C	2.79971020	-0.07531024	0.35371721
C	2.61868072	1.20396905	-0.30809441
N	-1.85131818	0.56081457	0.16789746
C	-3.10846741	-0.16457341	0.44510411
H	3.39942343	1.91660116	-0.54426201
C	-1.42885318	-1.61116379	-0.54189304
C	-2.94146135	-1.50580499	-0.28957334
H	-3.54343243	-1.52306007	-1.18705939
H	-3.26302273	-2.34581888	0.31268645
H	-3.20362081	-0.18981843	1.54104414
H	-3.90128979	0.47419455	0.03183917
H	-1.24264066	-2.16125873	-1.45691417
H	0.89739980	2.16722252	-1.26243077
C	-1.84062269	1.98365991	0.32469869
H	-0.82864505	2.28372646	0.51071253
H	-2.14128523	2.42080148	-0.62004696
H	-2.50833151	2.23040449	1.12961807
H	1.39646119	-1.63821761	0.92310066
C	4.06521696	-0.88200933	0.35605382
H	4.38668023	-1.10613752	-0.66256551
H	4.90182187	-0.34136186	0.80455560
H	3.92761933	-1.81708844	0.88781491
H	-1.01206430	-2.20015860	0.26510871

Time = 140.00 fs
E(CASSCF/6-31G(d)) = -479.5874718

C	-0.89141607	-0.18527722	-0.44503530
C	0.56395600	0.16119750	-0.29839078
C	1.26763775	1.30391473	-0.72662394
C	1.48426189	-0.63083050	0.52604953
C	2.79903093	-0.07160942	0.35432491
C	2.61872033	1.20102787	-0.31121188
N	-1.85160488	0.56195773	0.17215430

C	-3.10704382	-0.16698849	0.44741554
H	3.39442103	1.91841703	-0.55222880
C	-1.42848431	-1.60902106	-0.54741619
C	-2.94213138	-1.50451282	-0.29098237
H	-3.54385353	-1.51862097	-1.18925655
H	-3.26117951	-2.34636048	0.30849036
H	-3.19973964	-0.18592995	1.54228632
H	-3.89487002	0.47786661	0.04316266
H	-1.24488199	-2.15776284	-1.46507281
H	0.90820590	2.16370535	-1.27184845
C	-1.84006916	1.98086970	0.32883428
H	-0.82968501	2.29073656	0.51078610
H	-2.15138673	2.42263399	-0.61049552
H	-2.50287868	2.22664651	1.13630692
H	1.40132557	-1.63528988	0.93247264
C	4.06242354	-0.88188861	0.36140536
H	4.38656884	-1.10960401	-0.65625184
H	4.90262307	-0.33736682	0.79995654
H	3.92383346	-1.81380441	0.89882727
H	-1.01160601	-2.19979677	0.25630264

Time = 140.50 fs
E(CASSCF/6-31G(d)) = -479.5873798

C	-0.89270868	-0.18836853	-0.44671388
C	0.56878743	0.16236031	-0.30029331
C	1.26865524	1.30094069	-0.73351711
C	1.48109443	-0.62839086	0.52847998
C	2.79798059	-0.06787216	0.35490122
C	2.61870780	1.19801271	-0.31438820
N	-1.85172865	0.56267416	0.17633335
C	-3.10552905	-0.16914965	0.44970936
H	3.38890864	1.91963885	-0.55997650
C	-1.42820782	-1.60685366	-0.55301257
C	-2.94281092	-1.50319011	-0.29227533
H	-3.54386159	-1.51385621	-1.19185906
H	-3.25974985	-2.34677907	0.30446978
H	-3.19648402	-0.18316662	1.54233471
H	-3.88868936	0.48020005	0.05514073
H	-1.24609602	-2.15332161	-1.47337549
H	0.91833417	2.15946811	-1.28118706
C	-1.83939173	1.97807757	0.33290867
H	-0.83071181	2.29847100	0.51164245
H	-2.16232263	2.42521566	-0.60072681
H	-2.49754982	2.22388725	1.14334934
H	1.40472879	-1.63151978	0.94104203
C	4.05970494	-0.88168445	0.36678058

H	4.38602104	-1.11343383	-0.64968813
H	4.90303883	-0.33366120	0.79572553
H	3.92034425	-1.81053442	0.90988666
H	-1.01017185	-2.19935243	0.24764774

Time = 141.00 fs
E(CASSCF/6-31G(d)) = -479.5873173

C	-0.89424224	-0.19129793	-0.44824971
C	0.57370620	0.16335250	-0.30199691
C	1.26987389	1.29800754	-0.74050048
C	1.47818372	-0.62582831	0.53081691
C	2.79655827	-0.06412871	0.35545930
C	2.61864325	1.19494917	-0.31763707
N	-1.85165674	0.56292041	0.18040637
C	-3.10391089	-0.17104291	0.45199589
H	3.38297973	1.92021058	-0.56746822
C	-1.42803899	-1.60467724	-0.55868347
C	-2.94349236	-1.50183795	-0.29346457
H	-3.54346383	-1.50876976	-1.19479001
H	-3.25876460	-2.34711938	0.30064604
H	-3.19386102	-0.18168261	1.54140116
H	-3.88304026	0.48129450	0.06754107
H	-1.24630458	-2.14789634	-1.48176030
H	0.92760611	2.15465248	-1.29056105
C	-1.83859677	1.97531969	0.33692555
H	-0.83175351	2.30677113	0.51320954
H	-2.17402509	2.42846032	-0.59075037
H	-2.49239298	2.22210815	1.15083347
H	1.40657815	-1.62686830	0.94875220
C	4.05706864	-0.88138803	0.37217238
H	4.38495414	-1.11767868	-0.64287167
H	4.90303122	-0.33027619	0.79195926
H	3.91717622	-1.80723739	0.92098357
H	-1.00773855	-2.19889298	0.23918870

Time = 141.50 fs
E(CASSCF/6-31G(d)) = -479.5873200

C	-0.89598559	-0.19400723	-0.44962679
C	0.57863492	0.16414917	-0.30350954
C	1.27131354	1.29511325	-0.74756271
C	1.47556127	-0.62313586	0.53306429
C	2.79476885	-0.06041068	0.35601299
C	2.61852429	1.19186106	-0.32097162
N	-1.85136112	0.56266239	0.18434881
C	-3.10217601	-0.17266239	0.45428108

H	3.37676942	1.92011365	-0.57467971
C	-1.42798977	-1.60250812	-0.56442553
C	-2.94416855	-1.50045814	-0.29456399
H	-3.54266638	-1.50337088	-1.19795984
H	-3.25824907	-2.34741747	0.29703025
H	-3.19186562	-0.18161399	1.53976879
H	-3.87823038	0.48129950	0.08011144
H	-1.24554032	-2.14146795	-1.49018048
H	0.93585523	2.14941679	-1.30009283
C	-1.83769401	1.97263471	0.34089168
H	-0.83285595	2.31546346	0.51539700
H	-2.18640063	2.43225468	-0.58055182
H	-2.48741747	2.22125294	1.15879939
H	1.40680099	-1.62135327	0.95557772
C	4.05451810	-0.88099181	0.37757527
H	4.38329970	-1.12238411	-0.63581637
H	4.90258399	-0.32722603	0.78875181
H	3.91434748	-1.80386794	0.93210766
H	-1.00431375	-2.19846266	0.23093236

Time = 142.00 fs
E(CASSCF/6-31G(d)) = -479.5874184

C	-0.89790455	-0.19644333	-0.45083196
C	0.58349846	0.16472986	-0.30484087
C	1.27298923	1.29225374	-0.75469255
C	1.47325344	-0.62030433	0.53522491
C	2.79262275	-0.05675058	0.35657667
C	2.61834654	1.18877020	-0.32440425
N	-1.85081894	0.56187605	0.18814018
C	-3.10031145	-0.17400945	0.45656660
H	3.37044949	1.91936580	-0.58159965
C	-1.42806853	-1.60036284	-0.57023051
C	-2.94483277	-1.49905330	-0.29558833
H	-3.54147715	-1.49767394	-1.20127203
H	-3.25822172	-2.34769746	0.29362177
H	-3.19048026	-0.18307534	1.53777337
H	-3.87456061	0.48039890	0.09259416
H	-1.24384595	-2.13403911	-1.49860785
H	0.94293523	2.14391992	-1.30990199
C	-1.83669696	1.97006238	0.34481697
H	-0.83407408	2.32436518	0.51809969
H	-2.19933241	2.43646185	-0.57009749
H	-2.48259396	2.22122871	1.16723749
H	1.40534651	-1.61504902	0.96152582
C	4.05205276	-0.88048964	0.38298534
H	4.38100493	-1.12758850	-0.62855194

H	4.90170317	-0.32450771	0.78619221
H	3.91186876	-1.80037928	0.94324918
H	-0.99993719	-2.19808080	0.22284680

Time = 142.50 fs
E(CASSCF/6-31G(d)) = -479.5876425

C	-0.89996275	-0.19855913	-0.45185515
C	0.58822500	0.16507930	-0.30600227
C	1.27491013	1.28942387	-0.76187976
C	1.47128122	-0.61732275	0.53730051
C	2.79013555	-0.05318145	0.35716511
C	2.61810447	1.18569637	-0.32794643
N	-1.85001287	0.56054802	0.19176451
C	-3.09830645	-0.17509132	0.45885038
H	3.36422089	1.91801697	-0.58822871
C	-1.42827983	-1.59825771	-0.57608548
C	-2.94547856	-1.49762707	-0.29655251
H	-3.53990870	-1.49169817	-1.20462916
H	-3.25869306	-2.34796931	0.29040737
H	-3.18967447	-0.18615695	1.53577881
H	-3.87230019	0.47879124	0.10474181
H	-1.24127363	-2.12563466	-1.50703443
H	0.94872724	2.13830454	-1.32009409
C	-1.83562286	1.96764199	0.34871434
H	-0.83546231	2.33329029	0.52120154
H	-2.21268400	2.44092660	-0.55934158
H	-2.47785899	2.22190930	1.17609263
H	1.40218699	-1.60808208	0.96663594
C	4.04966832	-0.87987664	0.38840023
H	4.37803345	-1.13332196	-0.62112148
H	4.90041607	-0.32210160	0.78436137
H	3.90974267	-1.79672614	0.95440042
H	-0.99467958	-2.19774269	0.21486418

Time = 143.00 fs
E(CASSCF/6-31G(d)) = -479.5880241

C	-0.90212225	-0.20031430	-0.45268956
C	0.59274704	0.16518798	-0.30700686
C	1.27707851	1.28661843	-0.76911597
C	1.46966007	-0.61417911	0.53929194
C	2.78732769	-0.04973629	0.35779327
C	2.61779234	1.18265754	-0.33160865
N	-1.84893139	0.55867590	0.19521065
C	-3.09615442	-0.17591943	0.46112768
H	3.35830380	1.91614251	-0.59457774

C	-1.42862452	-1.59620817	-0.58197352
C	-2.94609963	-1.49618409	-0.29747113
H	-3.53798091	-1.48546714	-1.20793889
H	-3.25966514	-2.34822816	0.28736148
H	-3.18940478	-0.19092240	1.53414734
H	-3.87166048	0.47666772	0.11633289
H	-1.23788443	-2.11630069	-1.51547194
H	0.95314621	2.13268155	-1.33075001
C	-1.83449225	1.96541061	0.35259930
H	-0.83706489	2.34205610	0.52457957
H	-2.22630431	2.44548175	-0.54823479
H	-2.47312310	2.22314072	1.18527293
H	1.39731855	-1.60062232	0.97097697
C	4.04735712	-0.87914913	0.39381893
H	4.37436530	-1.13960509	-0.61357793
H	4.89876898	-0.31997328	0.78332900
H	3.90796318	-1.79286753	0.96555712
H	-0.98863961	-2.19742340	0.20688715

Time = 143.50 fs
E(CASSCF/6-31G(d)) = -479.5885934

C	-0.90434422	-0.20167603	-0.45333188
C	0.59700233	0.16505265	-0.30786940
C	1.27948890	1.28383283	-0.77639538
C	1.46839973	-0.61086148	0.54119961
C	2.78422403	-0.04644742	0.35847595
C	2.61740537	1.17967002	-0.33540024
N	-1.84756885	0.55626837	0.19847244
C	-3.09385487	-0.17650783	0.46339261
H	3.35292554	1.91383406	-0.60066488
C	-1.42909982	-1.59422815	-0.58787481
C	-2.94668971	-1.49472993	-0.29835800
H	-3.53572314	-1.47900834	-1.21111925
H	-3.26113133	-2.34845505	0.28444740
H	-3.18961487	-0.19740634	1.53320664
H	-3.87277071	0.47419098	0.12718575
H	-1.23374789	-2.10610215	-1.52394915
H	0.95614572	2.12711748	-1.34191692
C	-1.83332832	1.96340138	0.35648932
H	-0.83890705	2.35048963	0.52810804
H	-2.24003321	2.44995548	-0.53673356
H	-2.46828151	2.22474829	1.19466208
H	1.39076160	-1.59286966	0.97464211
C	4.04510874	-0.87830453	0.39924160
H	4.36999658	-1.14644744	-0.60597937
H	4.89682347	-0.31807614	0.78315054

H	3.90651538	-1.78876933	0.97671936
H	-0.98193922	-2.19708334	0.19879797

Time = 144.00 fs
E(CASSCF/6-31G(d)) = -479.5893859

C	-0.90658929	-0.20261963	-0.45378257
C	0.60093400	0.16467659	-0.30860639
C	1.28212775	1.28106375	-0.78371536
C	1.46750443	-0.60735946	0.54302412
C	2.78085350	-0.04334613	0.35922780
C	2.61694100	1.17674911	-0.33932958
N	-1.84592567	0.55334526	0.20154908
C	-3.09141492	-0.17687164	0.46563989
H	3.34830725	1.91118819	-0.60651198
C	-1.42969961	-1.59232945	-0.59376791
C	-2.94724236	-1.49327091	-0.29922594
H	-3.53317573	-1.47235205	-1.21410326
H	-3.26307707	-2.34861881	0.28161949
H	-3.19023514	-0.20561257	1.53321639
H	-3.87565820	0.47147740	0.13716950
H	-1.22894239	-2.09511940	-1.53250788
H	0.95772047	2.12162738	-1.35360286
C	-1.83215583	1.96164169	0.36040306
H	-0.84098882	2.35843357	0.53166122
H	-2.25370886	2.45417882	-0.52480850
H	-2.46322509	2.22654413	1.20413420
H	1.38256089	-1.58503690	0.97774186
C	4.04291074	-0.87734113	0.40466935
H	4.36493781	-1.15384687	-0.59838357
H	4.89465161	-0.31635480	0.78386339
H	3.90537551	-1.78440553	0.98789314
H	-0.97471745	-2.19667648	0.19046941

Time = 144.50 fs
E(CASSCF/6-31G(d)) = -479.5904061

C	-0.90881776	-0.20312889	-0.45404602
C	0.60449114	0.16406982	-0.30923599
C	1.28497298	1.27830898	-0.79107650
C	1.46697264	-0.60366586	0.54476697
C	2.77724872	-0.04046190	0.36006280
C	2.61640022	1.17390948	-0.34340391
N	-1.84400821	0.54993718	0.20444510
C	-3.08885028	-0.17702637	0.46786685
H	3.34465037	1.90829489	-0.61214055
C	-1.43041471	-1.59052125	-0.59963131

C	-2.94775091	-1.49181385	-0.30008664
H	-3.53039061	-1.46553093	-1.21684200
H	-3.26548041	-2.34867967	0.27882604
H	-3.19118340	-0.21551166	1.53434037
H	-3.88023867	0.46858732	0.14620911
H	-1.22355487	-2.08344397	-1.54119695
H	0.95790685	2.11617332	-1.36577463
C	-1.83100012	1.96015185	0.36435954
H	-0.84327997	2.36575139	0.53511730
H	-2.26717339	2.45799312	-0.51245218
H	-2.45785180	2.22833602	1.21356757
H	1.37278583	-1.57732982	0.98039489
C	4.04074944	-0.87625800	0.41010391
H	4.35921296	-1.16178765	-0.59084237
H	4.89233038	-0.31474917	0.78548470
H	3.90451133	-1.77975978	0.99908994
H	-0.96712396	-2.19615761	0.18177583

Time = 145.00 fs
E(CASSCF/6-31G(d)) = -479.5916459

C	-0.91098999	-0.20319650	-0.45413075
C	0.60762906	0.16324913	-0.30977793
C	1.28799406	1.27556713	-0.79848252
C	1.46679706	-0.59977845	0.54643129
C	2.77344565	-0.03782175	0.36099395
C	2.61578864	1.17116544	-0.34762937
N	-1.84182858	0.54608509	0.20717045
C	-3.08618520	-0.17698782	0.47007499
H	3.34212361	1.90522623	-0.61756781
C	-1.43123322	-1.58880954	-0.60544494
C	-2.94820850	-1.49036565	-0.30095071
H	-3.52743108	-1.45857886	-1.21930665
H	-3.26831336	-2.34859335	0.27601261
H	-3.19236520	-0.22703887	1.53662665
H	-3.88631628	0.46552372	0.15428582
H	-1.21768119	-2.07117378	-1.55006570
H	0.95678069	2.11066932	-1.37836038
C	-1.82988586	1.95894392	0.36837734
H	-0.84571810	2.37233133	0.53836061
H	-2.28027998	2.46125692	-0.49968478
H	-2.45207607	2.22993520	1.22285822
H	1.36153086	-1.56992586	0.98271837
C	4.03861070	-0.87505487	0.41554751
H	4.35285748	-1.17023993	-0.58339611
H	4.88993579	-0.31319889	0.78800863
H	3.90388283	-1.77482530	1.01032699

H	-0.95931175	-2.19549059	0.17260400

Time = 145.50 fs
E(CASSCF/6-31G(d)) = -479.5930573

C	-0.91306642	-0.20282413	-0.45404966
C	0.61030927	0.16223803	-0.31025346
C	1.29115235	1.27283677	-0.80593965
C	1.46696454	-0.59570158	0.54802264
C	2.76948327	-0.03544933	0.36203282
C	2.61511760	1.16853101	-0.35201072
N	-1.83940457	0.54183962	0.20974053
C	-3.08345150	-0.17677271	0.47227104
H	3.34085212	1.90202671	-0.62280357
C	-1.43214096	-1.58719701	-0.61119144
C	-2.94860798	-1.48893289	-0.30182780
H	-3.52437019	-1.45153023	-1.22148851
H	-3.27154294	-2.34831542	0.27312527
H	-3.19367535	-0.24009259	1.54000070
H	-3.89359566	0.46223982	0.16143189
H	-1.21142669	-2.05840807	-1.55915609
H	0.95445295	2.10499203	-1.39125653
C	-1.82883600	1.95802087	0.37247388
H	-0.84820981	2.37808909	0.54128371
H	-2.29289857	2.46385191	-0.48655791
H	-2.44583618	2.23116418	1.23193065
H	1.34891648	-1.56295430	0.98481827
C	4.03648075	-0.87373202	0.42100271
H	4.34591641	-1.17915901	-0.57606965
H	4.88753738	-0.31164709	0.79140513
H	3.90344290	-1.76960480	1.02162673
H	-0.95143116	-2.19465418	0.16286144

Time = 146.00 fs
E(CASSCF/6-31G(d)) = -479.5945608

C	-0.91500773	-0.20202241	-0.45382011
C	0.61249949	0.16106652	-0.31068513
C	1.29440153	1.27011537	-0.81345591
C	1.46745599	-0.59144743	0.54954943
C	2.76540318	-0.03336422	0.36318911
C	2.61440474	1.16601982	-0.35655113
N	-1.83675920	0.53726013	0.21217597
C	-3.08068677	-0.17639982	0.47446714
H	3.34091010	1.89870714	-0.62784807
C	-1.43312183	-1.58568278	-0.61685745
C	-2.94894207	-1.48752140	-0.30272683

H	-3.52128881	-1.44441905	-1.22339797
H	-3.27513264	-2.34780622	0.27011388
H	-3.19500017	-0.25453341	1.54427313
H	-3.90170375	0.45865424	0.16772136
H	-1.20490630	-2.04524322	-1.56849637
H	0.95106356	2.09899484	-1.40433726
C	-1.82787060	1.95737635	0.37666487
H	-0.85063419	2.38296886	0.54378888
H	-2.30492081	2.46568781	-0.47315622
H	-2.43909933	2.23186335	1.24074573
H	1.33509008	-1.55648070	0.98678132
C	4.03434691	-0.87229034	0.42647230
H	4.33844288	-1.18848520	-0.56886882
H	4.88519322	-0.31004434	0.79561928
H	3.90313886	-1.76410921	1.03301544
H	-0.94362378	-2.19364778	0.15248371

Time = 146.50 fs
E(CASSCF/6-31G(d)) = -479.5960597

C	-0.91677445	-0.20081071	-0.45346426
C	0.61417324	0.15977077	-0.31109662
C	1.29768806	1.26739814	-0.82104013
C	1.46824643	-0.58703671	0.55102321
C	2.76124955	-0.03158126	0.36447010
C	2.61367468	1.16364458	-0.36125168
N	-1.83392059	0.53241395	0.21450267
C	-3.07793187	-0.17589135	0.47668000
H	3.34231746	1.89524228	-0.63269137
C	-1.43415816	-1.58426254	-0.62243435
C	-2.94920359	-1.48613586	-0.30365627
H	-3.51827261	-1.43727829	-1.22506210
H	-3.27904399	-2.34703500	0.26693521
H	-3.19622073	-0.27018506	1.54916030
H	-3.91021800	0.45466983	0.17325867
H	-1.19824479	-2.03176947	-1.57809580
H	0.94677379	2.09252463	-1.41746669
C	-1.82700593	1.95699473	0.38096379
H	-0.85284937	2.38694280	0.54578859
H	-2.31626425	2.46670573	-0.45959629
H	-2.43186373	2.23189634	1.24930572
H	1.32022669	-1.55049792	0.98867039
C	4.03219811	-0.87073118	0.43195947
H	4.33049582	-1.19814418	-0.56177858
H	4.88294596	-0.30835171	0.80057145
H	3.90291382	-1.75835550	1.04452175
H	-0.93601733	-2.19249547	0.14143945

Time = 147.00 fs
E(CASSCF/6-31G(d)) = -479.5974393

C	-0.91832710	-0.19921660	-0.45300899
C	0.61530978	0.15839278	-0.31151247
C	1.30095172	1.26467682	-0.82870083
C	1.46930481	-0.58249850	0.55245842
C	2.75706885	-0.03010946	0.36588000
C	2.61295901	1.16141596	-0.36611059
N	-1.83092148	0.52737495	0.21675138
C	-3.07522818	-0.17527395	0.47892948
H	3.34504065	1.89157337	-0.63731468
C	-1.43523111	-1.58292866	-0.62791878
C	-2.94938567	-1.48477945	-0.30462446
H	-3.51540879	-1.43013934	-1.22652078
H	-3.28323748	-2.34598383	0.26355499
H	-3.19721724	-0.28683728	1.55431460
H	-3.91869753	0.45019329	0.17816640
H	-1.19157628	-2.01806903	-1.58794119
H	0.94175845	2.08543790	-1.43051100
C	-1.82625363	1.95685167	0.38538160
H	-0.85470137	2.39000890	0.54720620
H	-2.32687447	2.46688027	-0.44602384
H	-2.42415897	2.23115436	1.25765537
H	1.30452945	-1.54492619	0.99052294
C	4.03002532	-0.86905654	0.43746783
H	4.32213922	-1.20804813	-0.55476319
H	4.88082054	-0.30654272	0.80615927
H	3.90270848	-1.75236472	1.05617431
H	-0.92872220	-2.19124816	0.12973284

Time = 147.50 fs
E(CASSCF/6-31G(d)) = -479.5986059

C	-0.91962602	-0.19727527	-0.45248629
C	0.61589363	0.15697990	-0.31195763
C	1.30412609	1.26193862	-0.83644523
C	1.47059423	-0.57786936	0.55387154
C	2.75290981	-0.02895122	0.36741932
C	2.61229625	1.15934121	-0.37112238
N	-1.82779897	0.52222248	0.21895761
C	-3.07261475	-0.17457945	0.48123657
H	3.34899814	1.88761411	-0.64169308
C	-1.43632096	-1.58167040	-0.63331259
C	-2.94948201	-1.48345361	-0.30563990
H	-3.51278211	-1.42303153	-1.22782216

H	-3.28767354	-2.34465004	0.25994950
H	-3.19787375	-0.30425125	1.55935965
H	-3.92671354	0.44515258	0.18257400
H	-1.18504337	-2.00421541	-1.59799590
H	0.93619703	2.07761487	-1.44335052
C	-1.82562006	1.95691497	0.38992648
H	-0.85603511	2.39218724	0.54797559
H	-2.33672617	2.46621975	-0.43260796
H	-2.41604336	2.22955881	1.26588020
H	1.28822786	-1.53962122	0.99235403
C	4.02782178	-0.86726912	0.44300179
H	4.31344058	-1.21809780	-0.54776843
H	4.87882392	-0.30460401	0.81226024
H	3.90246291	-1.74615940	1.06799985
H	-0.92182900	-2.18998430	0.11740373

Time = 148.00 fs
E(CASSCF/6-31G(d)) = -479.5994874

C	-0.92063091	-0.19502900	-0.45193373
C	0.61591365	0.15558443	-0.31245685
C	1.30713880	1.25916533	-0.84427835
C	1.47207224	-0.57319168	0.55527987
C	2.74882358	-0.02810146	0.36908425
C	2.61173163	1.15742246	-0.37627673
N	-1.82459432	0.51704024	0.22116167
C	-3.07012554	-0.17384489	0.48362115
H	3.35406928	1.88326005	-0.64579945
C	-1.43740741	-1.58047429	-0.63862237
C	-2.94948722	-1.48215791	-0.30671158
H	-3.51047071	-1.41598173	-1.22901756
H	-3.29231341	-2.34304768	0.25610605
H	-3.19808346	-0.32216760	1.56392609
H	-3.93387688	0.43951044	0.18660822
H	-1.17879571	-1.99027386	-1.60820208
H	0.93026557	2.06897105	-1.45588985
C	-1.82510596	1.95714567	0.39460362
H	-0.85670668	2.39351553	0.54804031
H	-2.34582262	2.46476520	-0.41953307
H	-2.40759888	2.22706271	1.27410166
H	1.27157424	-1.53439000	0.99416268
C	4.02558309	-0.86537236	0.44856694
H	4.30446978	-1.22818572	-0.54072528
H	4.87694661	-0.30253473	0.81873513
H	3.90211857	-1.73976080	1.08002151
H	-0.91540788	-2.18880955	0.10452512

Time = 148.50 fs
E(CASSCF/6-31G(d)) = -479.6000434

C	-0.92130066	-0.19252597	-0.45139473
C	0.61536283	0.15426275	-0.31303412
C	1.30991114	1.25633286	-0.85220187
C	1.47369061	-0.56851188	0.55669963
C	2.74486446	-0.02754582	0.37086539
C	2.61131728	1.15565438	-0.38155679
N	-1.82135278	0.51191500	0.22340823
C	-3.06778723	-0.17311200	0.48610010
H	3.36010557	1.87839945	-0.64960982
C	-1.43846976	-1.57932462	-0.64385841
C	-2.94939704	-1.48088998	-0.30784917
H	-3.50854252	-1.40901461	-1.23015629
H	-3.29711908	-2.34120784	0.25202284
H	-3.19775409	-0.34031549	1.56768309
H	-3.93986107	0.43327341	0.19038473
H	-1.17298770	-1.97630375	-1.61848441
H	0.92412974	2.05946472	-1.46806698
C	-1.82470646	1.95749949	0.39941513
H	-0.85659561	2.39404376	0.54735417
H	-2.35419193	2.46258797	-0.40698963
H	-2.39892534	2.22365121	1.28246799
H	1.25483805	-1.52901209	0.99594122
C	4.02330711	-0.86337061	0.45417038
H	4.29529816	-1.23819879	-0.53355498
H	4.87516532	-0.30034523	0.82543325
H	3.90162004	-1.73318701	1.09225618
H	-0.90950955	-2.18785430	0.09119939

Time = 149.00 fs
E(CASSCF/6-31G(d)) = -479.6002790

C	-0.92159262	-0.18981943	-0.45091949
C	0.61423728	0.15307421	-0.31371149
C	1.31235765	1.25341098	-0.86021337
C	1.47539479	-0.56387831	0.55814351
C	2.74109116	-0.02725883	0.37274668
C	2.61111252	1.15402192	-0.38693741
N	-1.81812384	0.50693574	0.22574667
C	-3.06561710	-0.17242604	0.48868552
H	3.36694347	1.87292420	-0.65310871
C	-1.43948697	-1.57820406	-0.64903331
C	-2.94920865	-1.47964559	-0.30906313
H	-3.50705184	-1.40215258	-1.23128108
H	-3.30205338	-2.33917767	0.24770775

H	-3.19681293	-0.35842339	1.57036453
H	-3.94442051	0.42649629	0.19400217
H	-1.16777614	-1.96236119	-1.62875661
H	0.91793849	2.04910072	-1.47985902
C	-1.82441160	1.95792803	0.40435991
H	-0.85561654	2.39382924	0.54588104
H	-2.36188222	2.45978642	-0.39516246
H	-2.39013181	2.21934054	1.29114291
H	1.23829777	-1.52326236	0.99768562
C	4.02099387	-0.86126918	0.45982123
H	4.28599712	-1.24802247	-0.52617467
H	4.87344721	-0.29805442	0.83219728
H	3.90091672	-1.72645087	1.10471324
H	-0.90416804	-2.18727134	0.07755190

Time = 149.50 fs
E(CASSCF/6-31G(d)) = -479.6002363

C	-0.92146186	-0.18696659	-0.45056597
C	0.61253557	0.15207855	-0.31450761
C	1.31438619	1.25036380	-0.86830540
C	1.47712199	-0.55933932	0.55961769
C	2.73756914	-0.02720107	0.37470380
C	2.61118420	1.15249801	-0.39238316
N	-1.81496166	0.50219298	0.22823129
C	-3.06362156	-0.17183430	0.49138352
H	3.37441699	1.86673940	-0.65629419
C	-1.44043746	-1.57709447	-0.65416005
C	-2.94892081	-1.47841889	-0.31036474
H	-3.50603669	-1.39541605	-1.23242437
H	-3.30707966	-2.33701811	0.24317652
H	-3.19521134	-0.37623002	1.57178879
H	-3.94740394	0.41928268	0.19753793
H	-1.16331734	-1.94850210	-1.63892903
H	0.91181927	2.03792995	-1.49128545
C	-1.82420725	1.95838019	0.40943341
H	-0.85372892	2.39293241	0.54359576
H	-2.36895488	2.45648176	-0.38421773
H	-2.38132745	2.21417614	1.30029155
H	1.22223021	-1.51693190	0.99940563
C	4.01864523	-0.85907442	0.46553085
H	4.27663721	-1.25754522	-0.51850383
H	4.87175442	-0.29568638	0.83886896
H	3.89996448	-1.71955899	1.11739392
H	-0.89940482	-2.18723180	0.06372316

Time = 150.00 fs

E(CASSCF/6-31G(d)) = -479.5999944

C	-0.92086074	-0.18402748	-0.45040030
C	0.61025889	0.15133136	-0.31543517
C	1.31589989	1.24715124	-0.87646489
C	1.47879714	-0.55494155	0.56111821
C	2.73437375	-0.02731572	0.37670275
C	2.61160615	1.15104218	-0.39784701
N	-1.81192556	0.49777814	0.23092147
C	-3.06179494	-0.17138423	0.49419332
H	3.38237000	1.85977084	-0.65918253
C	-1.44129888	-1.57597773	-0.65925006
C	-2.94853401	-1.47720266	-0.31176588
H	-3.50551714	-1.38882356	-1.23360596
H	-3.31216119	-2.33480062	0.23845022
H	-3.19292849	-0.39349505	1.57187128
H	-3.94876258	0.41178226	0.20104564
H	-1.15976450	-1.93478536	-1.64891655
H	0.90587426	2.02604713	-1.50240831
C	-1.82407653	1.95880327	0.41462749
H	-0.85094396	2.39141355	0.54048568
H	-2.37547655	2.45281310	-0.37428996
H	-2.37261121	2.20822985	1.31006458
H	1.20689929	-1.50984697	1.00113365
C	4.01626452	-0.85679393	0.47131288
H	4.26728687	-1.26666289	-0.51046981
H	4.87004804	-0.29326614	0.84529410
H	3.89872691	-1.71251092	1.13029104
H	-0.89523355	-2.18792039	0.04985955

Time = 150.50 fs
E(CASSCF/6-31G(d)) = -479.5996657

C	-0.91974076	-0.18106289	-0.45049430
C	0.60741316	0.15087669	-0.31649794
C	1.31680450	1.24373240	-0.88467274
C	1.48032794	-0.55072794	0.56262835
C	2.73159184	-0.02752668	0.37869965
C	2.61245484	1.14960172	-0.40327166
N	-1.80907898	0.49378141	0.23388009
C	-3.06011939	-0.17112177	0.49710704
H	3.39066650	1.85197111	-0.66181147
C	-1.44204826	-1.57483648	-0.66431173
C	-2.94805049	-1.47598874	-0.31327876
H	-3.50549470	-1.38239199	-1.23483210
H	-3.31725990	-2.33260269	0.23355239
H	-3.18997325	-0.41000818	1.57063007

H	-3.94855153	0.40418551	0.20455434
H	-1.15726372	-1.92127482	-1.65864539
H	0.90017790	2.01358640	-1.51332909
C	-1.82400131	1.95914477	0.41993067
H	-0.84732856	2.38933001	0.53655248
H	-2.38151124	2.44892984	-0.36546957
H	-2.36406182	2.20159613	1.32058134
H	1.19254386	-1.50188424	1.00292998
C	4.01385578	-0.85443686	0.47718275
H	4.25801244	-1.27528344	-0.50201373
H	4.86829141	-0.29081448	0.85132741
H	3.89717601	-1.70529951	1.14338961
H	-0.89166307	-2.18952519	0.03610268

Time = 151.00 fs
E(CASSCF/6-31G(d)) = -479.5993986

C	-0.91805868	-0.17813098	-0.45091753
C	0.60401354	0.15073937	-0.31768861
C	1.31702259	1.24007031	-0.89290439
C	1.48160376	-0.54673462	0.56411908
C	2.72931742	-0.02774285	0.38064390
C	2.61379842	1.14811755	-0.40859619
N	-1.80648451	0.49028668	0.23716828
C	-3.05856657	-0.17109045	0.50011017
H	3.39919772	1.84332648	-0.66424054
C	-1.44266314	-1.57365464	-0.66935020
C	-2.94747433	-1.47476854	-0.31491559
H	-3.50595294	-1.37613657	-1.23609545
H	-3.32233440	-2.33050180	0.22850612
H	-3.18638156	-0.42559451	1.56818357
H	-3.94692176	0.39671709	0.20807035
H	-1.15594784	-1.90803991	-1.66805859
H	0.89477592	2.00071697	-1.52418144
C	-1.82396322	1.95935544	0.42532943
H	-0.84300598	2.38673248	0.53181331
H	-2.38711353	2.44497765	-0.35779205
H	-2.35572905	2.19438636	1.33191359
H	1.17936737	-1.49298568	1.00488284
C	4.01142276	-0.85201431	0.48315657
H	4.24888049	-1.28333171	-0.49309619
H	4.86645385	-0.28834229	0.85683716
H	3.89529223	-1.69791255	1.15666817
H	-0.88869785	-2.19221866	0.02257729

Time = 151.50 fs
E(CASSCF/6-31G(d)) = -479.5993741

C	-0.91578686	-0.17528269	-0.45172499
C	0.60008902	0.15092382	-0.31899014
C	1.31650845	1.23613590	-0.90113159
C	1.48250489	-0.54298675	0.56555451
C	2.72763890	-0.02787051	0.38248478
C	2.61568239	1.14653306	-0.41376659
N	-1.80419463	0.48736369	0.24083674
C	-3.05710125	-0.17133136	0.50318261
H	3.40788597	1.83386436	-0.66654754
C	-1.44312348	-1.57241776	-0.67436849
C	-2.94681134	-1.47353368	-0.31668833
H	-3.50685898	-1.37007030	-1.23737592
H	-3.32733730	-2.32856823	0.22333151
H	-3.18220875	-0.44011548	1.56474046
H	-3.94410173	0.38962782	0.21158157
H	-1.15592788	-1.89515404	-1.67711975
H	0.88968592	1.98763856	-1.53512293
C	-1.82394405	1.95939327	0.43081037
H	-0.83815160	2.38365993	0.52629998
H	-2.39232358	2.44107877	-0.35123038
H	-2.34762832	2.18672141	1.34407195
H	1.16753123	-1.48317406	1.00710422
C	4.00896792	-0.84953926	0.48924993
H	4.23996132	-1.29075341	-0.48370228
H	4.86451628	-0.28584660	0.86170984
H	3.89306353	-1.69033501	1.17010056
H	-0.88633798	-2.19613112	0.00937883

Time = 152.00 fs
E(CASSCF/6-31G(d)) = -479.5997655

C	-0.91292395	-0.17255721	-0.45294429
C	0.59568422	0.15142220	-0.32038097
C	1.31525620	1.23190905	-0.90932472
C	1.48292234	-0.53949438	0.56690056
C	2.72662053	-0.02783108	0.38417967
C	2.61811772	1.14480243	-0.41874633
N	-1.80224074	0.48505954	0.24491582
C	-3.05568610	-0.17188333	0.50629998
H	3.41668497	1.82365961	-0.66882269
C	-1.44341396	-1.57111354	-0.67936916
C	-2.94606898	-1.47227654	-0.31860847
H	-3.50816624	-1.36420322	-1.23864254
H	-3.33221252	-2.32685804	0.21804365
H	-3.17751894	-0.45346655	1.56058417
H	-3.94037170	0.38318442	0.21506398

H	-1.15728386	-1.88269187	-1.68581602
H	0.88490173	1.97457158	-1.54632442
C	-1.82392566	1.95922757	0.43636295
H	-0.83298258	2.38013521	0.52005691
H	-2.39716372	2.43731186	-0.34569218
H	-2.33973963	2.17872409	1.35699732
H	1.15715232	-1.47256664	1.00972336
C	4.00649212	-0.84702590	0.49547731
H	4.23133172	-1.29751761	-0.47384458
H	4.86247884	-0.28331009	0.86585444
H	3.89048426	-1.68255200	1.18365784
H	-0.88458088	-2.20132416	-0.00343704

Time = 152.50 fs
E(CASSCF/6-31G(d)) = -479.6006797

C	-0.90950476	-0.16997833	-0.45456150
C	0.59085878	0.15222946	-0.32184384
C	1.31330184	1.22737724	-0.91745590
C	1.48278415	-0.53624877	0.56813568
C	2.72628066	-0.02758108	0.38570323
C	2.62107186	1.14289790	-0.42352630
N	-1.80062168	0.48339027	0.24940453
C	-3.05428741	-0.17278349	0.50943502
H	3.42557697	1.81283927	-0.67116096
C	-1.44352654	-1.56973220	-0.68435670
C	-2.94525615	-1.47099089	-0.32068688
H	-3.50981753	-1.35854115	-1.23985570
H	-3.33689290	-2.32540661	0.21265143
H	-3.17237144	-0.46557143	1.55605215
H	-3.93603066	0.37765731	0.21849049
H	-1.16005636	-1.87072497	-1.69415915
H	0.88040079	1.96174480	-1.55795760
C	-1.82388937	1.95884281	0.44198246
H	-0.82774282	2.37616100	0.51313759
H	-2.40163821	2.43369207	-0.34102225
H	-2.33201131	2.17051156	1.37055929
H	1.14830493	-1.46138525	1.01287750
C	4.00399472	-0.84448841	0.50185168
H	4.22307789	-1.30361801	-0.46356411
H	4.86036882	-0.28070311	0.86920645
H	3.88755391	-1.67455233	1.19731095
H	-0.88342288	-2.20776459	-0.01586241

Time = 153.00 fs
E(CASSCF/6-31G(d)) = -479.6020794

C	-0.90562090	-0.16754725	-0.45648044
C	0.58569164	0.15336154	-0.32338261
C	1.31074692	1.22253634	-0.92550492
C	1.48209531	-0.53321496	0.56927319
C	2.72654959	-0.02715019	0.38706912
C	2.62443825	1.14083021	-0.42815179
N	-1.79927920	0.48232169	0.25424326
C	-3.05288583	-0.17407122	0.51256024
H	3.43456383	1.80159607	-0.67364769
C	-1.44346791	-1.56826529	-0.68934583
C	-2.94438310	-1.46967288	-0.32293424
H	-3.51174913	-1.35308381	-1.24096742
H	-3.34129609	-2.32421923	0.20715842
H	-3.16679050	-0.47636452	1.55150302
H	-3.93134330	0.37331410	0.22184499
H	-1.16423045	-1.85931298	-1.70218369
H	0.87615013	1.94939514	-1.57017744
C	-1.82381143	1.95824896	0.44767733
H	-0.82269238	2.37170408	0.50559563
H	-2.40574036	2.43012341	-0.33700802
H	-2.32436678	2.16218039	1.38455808
H	1.14103131	-1.44997936	1.01668775
C	4.00147214	-0.84194055	0.50838218
H	4.21530796	-1.30907365	-0.45293203
H	4.85824900	-0.27798399	0.87173252
H	3.88427529	-1.66633417	1.21103330
H	-0.88284163	-2.21526694	-0.02794623

Time = 153.50 fs
E(CASSCF/6-31G(d)) = -479.6035665

C	-0.90146355	-0.16522821	-0.45841075
C	0.58031352	0.15479155	-0.32503679
C	1.30791676	1.21742396	-0.93347352
C	1.48093380	-0.53031572	0.57039722
C	2.72721551	-0.02668588	0.38837948
C	2.62791877	1.13871704	-0.43280241
N	-1.79805801	0.48173114	0.25925716
C	-3.05149250	-0.17579736	0.51565434
H	3.44365119	1.79021299	-0.67634110
C	-1.44327786	-1.56669659	-0.69438586
C	-2.94346184	-1.46832321	-0.32536277
H	-3.51389471	-1.34782311	-1.24191781
H	-3.34531621	-2.32325671	0.20156534
H	-3.16071588	-0.48576322	1.54726914
H	-3.92645868	0.37043262	0.22514384
H	-1.16970902	-1.84850001	-1.70994357

H	0.87209304	1.93782880	-1.58306665
C	-1.82365125	1.95750407	0.45348327
H	-0.81813601	2.36666003	0.49747886
H	-2.40948925	2.42630184	-0.33338743
H	-2.31669730	2.15378218	1.39872915
H	1.13533257	-1.43885061	1.02120849
C	3.99890630	-0.83940426	0.51505868
H	4.20820036	-1.31393753	-0.44206109
H	4.85619217	-0.27506734	0.87342980
H	3.88065184	-1.65791401	1.22480530
H	-0.88269401	-2.22336509	-0.03980170

Time = 154.00 fs
E(CASSCF/6-31G(d)) = -479.6044392

C	-0.89674121	-0.16310441	-0.46089099
C	0.57466417	0.15608228	-0.32653088
C	1.30466586	1.21205092	-0.94139778
C	1.47857300	-0.52759401	0.57116312
C	2.72890708	-0.02555771	0.38933583
C	2.63186911	1.13626682	-0.43720219
N	-1.79729117	0.48188357	0.26488977
C	-3.04993123	-0.17791640	0.51870268
H	3.45305535	1.77836744	-0.67958136
C	-1.44284649	-1.56501158	-0.69943281
C	-2.94248536	-1.46690830	-0.32799589
H	-3.51621320	-1.34270685	-1.24276680
H	-3.34898794	-2.32262186	0.19576206
H	-3.15458498	-0.49419658	1.54393230
H	-3.92196678	0.36898124	0.22833978
H	-1.17677105	-1.83834387	-1.71765868
H	0.86822418	1.92675612	-1.59683365
C	-1.82347504	1.95642867	0.45936872
H	-0.81414702	2.36131017	0.48894771
H	-2.41289330	2.42292229	-0.32992971
H	-2.30876295	2.14555735	1.41313133
H	1.13084490	-1.42761094	1.02695962
C	3.99627459	-0.83687465	0.52199541
H	4.20153869	-1.31832307	-0.43082676
H	4.85402017	-0.27183050	0.87424429
H	3.87667541	-1.64911556	1.23879577
H	-0.88317745	-2.23284033	-0.05151628

Time = 154.50 fs
E(CASSCF/6-31G(d)) = -479.6050528

C	-0.89208238	-0.16113522	-0.46305011

C	0.56916314	0.15736754	-0.32804871
C	1.30171815	1.20666845	-0.94901655
C	1.47566502	-0.52498552	0.57187389
C	2.73084820	-0.02438674	0.39025136
C	2.63548124	1.13391794	-0.44164679
N	-1.79657157	0.48240882	0.27041436
C	-3.04837985	-0.18042865	0.52157943
H	3.46235334	1.76706445	-0.68316393
C	-1.44232028	-1.56325471	-0.70441908
C	-2.94150850	-1.46549885	-0.33074253
H	-3.51854806	-1.33790887	-1.24338885
H	-3.35200050	-2.32218312	0.18999381
H	-3.14829327	-0.50111045	1.54176252
H	-3.91778103	0.36928062	0.23143440
H	-1.18480915	-1.82921980	-1.72508921
H	0.86463603	1.91703142	-1.61085560
C	-1.82319928	1.95525764	0.46520806
H	-0.81120153	2.35556033	0.48032631
H	-2.41587521	2.41922848	-0.32643150
H	-2.30078034	2.13774157	1.42680192
H	1.12777936	-1.41761461	1.03354876
C	3.99365058	-0.83447491	0.52886886
H	4.19592914	-1.32215900	-0.41987123
H	4.85202079	-0.26830786	0.87423606
H	3.87250721	-1.64037293	1.25242927
H	-0.88392329	-2.24211576	-0.06287394

Time = 155.00 fs
E(CASSCF/6-31G(d)) = -479.6056052

C	-0.88743525	-0.15926332	-0.46489960
C	0.56371854	0.15866647	-0.32962009
C	1.29904481	1.20114853	-0.95654148
C	1.47215414	-0.52238082	0.57253053
C	2.73303001	-0.02312355	0.39114332
C	2.63880974	1.13158634	-0.44626222
N	-1.79584749	0.48328025	0.27594878
C	-3.04678014	-0.18338477	0.52433685
H	3.47179480	1.75611851	-0.68728601
C	-1.44168878	-1.56138317	-0.70947621
C	-2.94051273	-1.46405518	-0.33367424
H	-3.52090775	-1.33327633	-1.24380863
H	-3.35436420	-2.32190154	0.18408502
H	-3.14176881	-0.50679140	1.54100048
H	-3.91388022	0.37130683	0.23456867
H	-1.19395806	-1.82091835	-1.73254856
H	0.86129768	1.90831358	-1.62544015

C	-1.82281969	1.95395838	0.47115652
H	-0.80928191	2.34932828	0.47150613
H	-2.41851185	2.41510990	-0.32254829
H	-2.29244760	2.13025190	1.43989782
H	1.12590238	-1.40883899	1.04128786
C	3.99096745	-0.83214750	0.53587341
H	4.19124888	-1.32564217	-0.40897233
H	4.85020189	-0.26436897	0.87345694
H	3.86806512	-1.63145740	1.26608352
H	-0.88497556	-2.25124563	-0.07430026

Time = 155.50 fs
E(CASSCF/6-31G(d)) = -479.6062008

C	-0.88287858	-0.15748685	-0.46639094
C	0.55838972	0.15997635	-0.33123650
C	1.29668262	1.19551429	-0.96397789
C	1.46807976	-0.51974825	0.57312089
C	2.73537843	-0.02175909	0.39200443
C	2.64181889	1.12925459	-0.45105062
N	-1.79508643	0.48445529	0.28145507
C	-3.04512495	-0.18676146	0.52695039
H	3.48132492	1.74563075	-0.69203395
C	-1.44095764	-1.55940320	-0.71459738
C	-2.93950527	-1.46257740	-0.33678511
H	-3.52324703	-1.32878789	-1.24404891
H	-3.35600420	-2.32175358	0.17802212
H	-3.13510600	-0.51131679	1.54187582
H	-3.91027483	0.37496708	0.23782463
H	-1.20412668	-1.81347667	-1.74013688
H	0.85832077	1.90046761	-1.64044373
C	-1.82234106	1.95252808	0.47720658
H	-0.80838334	2.34271241	0.46261674
H	-2.42080930	2.41056894	-0.31806033
H	-2.28370647	2.12322619	1.45222894
H	1.12509014	-1.40147458	1.05026828
C	3.98823473	-0.82989982	0.54301146
H	4.18752508	-1.32885908	-0.39819826
H	4.84861977	-0.25999385	0.87196184
H	3.86338627	-1.62238807	1.27976081
H	-0.88635981	-2.26002775	-0.08589744

Time = 156.00 fs
E(CASSCF/6-31G(d)) = -479.6068604

C	-0.87849014	-0.15580367	-0.46748090
C	0.55323425	0.16129893	-0.33288998

C	1.29465704	1.18979281	-0.97133791
C	1.46349270	-0.51706334	0.57363971
C	2.73781100	-0.02028852	0.39282804
C	2.64448442	1.12690764	-0.45601349
N	-1.79425744	0.48588669	0.28689578
C	-3.04341443	-0.19052628	0.52940288
H	3.49083775	1.73566341	-0.69747576
C	-1.44013544	-1.55732146	-0.71977736
C	-2.93849188	-1.46106622	-0.34006620
H	-3.52553210	-1.32441745	-1.24415054
H	-3.35685602	-2.32172040	0.17179564
H	-3.12839317	-0.51475855	1.54450692
H	-3.90689324	0.38008731	0.24130794
H	-1.21521061	-1.80691084	-1.74793967
H	0.85584498	1.89329812	-1.65566688
C	-1.82177022	1.95096229	0.48334931
H	-0.80842312	2.33583726	0.45378602
H	-2.42279726	2.40562940	-0.31278747
H	-2.27452527	2.11680035	1.46365915
H	1.12523313	-1.39560253	1.06051621
C	3.98546558	-0.82773687	0.55028297
H	4.18476512	-1.33189517	-0.38759536
H	4.84731199	-0.25518063	0.86981639
H	3.85851423	-1.61318885	1.29346892
H	-0.88808966	-2.26827526	-0.09775253

Time = 156.50 fs
E(CASSCF/6-31G(d)) = -479.6075730

C	-0.87434506	-0.15421167	-0.46813269
C	0.54830765	0.16263732	-0.33457247
C	1.29298594	1.18401393	-0.97863858
C	1.45845023	-0.51431065	0.57408907
C	2.74024080	-0.01870946	0.39360770
C	2.64679235	1.12453324	-0.46115197
N	-1.79333270	0.48752369	0.29223538
C	-3.04165460	-0.19463914	0.53168651
H	3.50017736	1.72623922	-0.70366026
C	-1.43923378	-1.55514427	-0.72501379
C	-2.93747661	-1.45952272	-0.34350585
H	-3.52774042	-1.32013496	-1.24417018
H	-3.35686807	-2.32179070	0.16540155
H	-3.12171100	-0.51717745	1.54888562
H	-3.90359686	0.38642229	0.24514012
H	-1.22709542	-1.80121396	-1.75601762
H	0.85402385	1.88657306	-1.67087407
C	-1.82111492	1.94925488	0.48957431

H	-0.80924974	2.32884939	0.44513631
H	-2.42453215	2.40033901	-0.30659672
H	-2.26490033	2.11110566	1.47411529
H	1.12624256	-1.39117891	1.07198222
C	3.98267652	-0.82566143	0.55768496
H	4.18295601	-1.33483065	-0.37718403
H	4.84629192	-0.24994682	0.86709477
H	3.85349815	-1.60388759	1.30722049
H	-0.89016076	-2.27582664	-0.10992977

Time = 157.00 fs
E(CASSCF/6-31G(d)) = -479.6082991

C	-0.87051446	-0.15270898	-0.46831674
C	0.54366269	0.16399473	-0.33627540
C	1.29168183	1.17820829	-0.98589972
C	1.45301303	-0.51148447	0.57447852
C	2.74257947	-0.01702130	0.39433715
C	2.64873771	1.12212208	-0.46646722
N	-1.79228830	0.48931291	0.29744116
C	-3.03985545	-0.19905395	0.53380392
H	3.50914613	1.71734658	-0.71061788
C	-1.43826729	-1.55287694	-0.73030821
C	-2.93646190	-1.45794830	-0.34709016
H	-3.52986033	-1.31590725	-1.24417751
H	-3.35600420	-2.32196360	0.15884229
H	-3.11513164	-0.51862121	1.55487487
H	-3.90020271	0.39367202	0.24944809
H	-1.23965865	-1.79635496	-1.76439994
H	0.85300969	1.88004820	-1.68581599
C	-1.82038267	1.94739872	0.49586985
H	-0.81065612	2.32191118	0.43678038
H	-2.42609829	2.39476984	-0.29940641
H	-2.25485521	2.10626567	1.48359181
H	1.12805108	-1.38803462	1.08453794
C	3.97988722	-0.82367420	0.56521141
H	4.18206484	-1.33773760	-0.36695538
H	4.84554591	-0.24433049	0.86387759
H	3.84839258	-1.59451427	1.32103170
H	-0.89254595	-2.28255518	-0.12246485

Time = 157.50 fs
E(CASSCF/6-31G(d)) = -479.6089792

C	-0.86706400	-0.15129383	-0.46801092
C	0.53934863	0.16537405	-0.33798988
C	1.29075343	1.17240535	-0.99314192

C	1.44724357	-0.50858798	0.57482498
C	2.74474007	-0.01522467	0.39501004
C	2.65032321	1.11966682	-0.47196007
N	-1.79110465	0.49119916	0.30248367
C	-3.03802884	-0.20372036	0.53576823
H	3.51751618	1.70894682	-0.71836178
C	-1.43725359	-1.55052323	-0.73566655
C	-2.93544859	-1.45634451	-0.35080353
H	-3.53188940	-1.31169906	-1.24424989
H	-3.35424494	-2.32224962	0.15212696
H	-3.10871818	-0.51912733	1.56221924
H	-3.89650884	0.40150075	0.25435321
H	-1.25277209	-1.79227797	-1.77307955
H	0.85293918	1.87349216	-1.70025390
C	-1.81957944	1.94538641	0.50222312
H	-0.81239809	2.31519327	0.42881780
H	-2.42760694	2.38901642	-0.29118666
H	-2.24443816	2.10239258	1.49215125
H	1.13060861	-1.38589086	1.09798133
C	3.97711974	-0.82177383	0.57285333
H	4.18203895	-1.34067790	-0.35687108
H	4.84503295	-0.23838872	0.86025072
H	3.84325605	-1.58509865	1.33492117
H	-0.89519385	-2.28837488	-0.13536236

Time = 158.00 fs
E(CASSCF/6-31G(d)) = -479.6095638

C	-0.86405283	-0.14996463	-0.46720078
C	0.53541016	0.16677757	-0.33970693
C	1.29020715	1.16663164	-1.00038448
C	1.44120553	-0.50563143	0.57515145
C	2.74663883	-0.01332162	0.39562013
C	2.65155718	1.11716181	-0.47763121
N	-1.78976681	0.49312616	0.30733732
C	-3.03618630	-0.20858521	0.53760205
H	3.52504680	1.70098450	-0.72689149
C	-1.43621314	-1.54808505	-0.74109926
C	-2.93443617	-1.45471312	-0.35462937
H	-3.53383183	-1.30747471	-1.24446710
H	-3.35158813	-2.32267038	0.14527082
H	-3.10252502	-0.51872952	1.57056779
H	-3.89231955	0.40955759	0.25996016
H	-1.26630199	-1.78890375	-1.78201185
H	0.85392192	1.86670412	-1.71397848
C	-1.81870843	1.94321132	0.50862042
H	-0.81421665	2.30886534	0.42133377

H	-2.42919215	2.38319212	-0.28195724
H	-2.23371837	2.09958357	1.49991875
H	1.13387258	-1.38438604	1.11204987
C	3.97439785	-0.81995710	0.58059904
H	4.18280796	-1.34370223	-0.34686639
H	4.84468793	-0.23219551	0.85630556
H	3.83815046	-1.57566942	1.34890791
H	-0.89802878	-2.29324298	-0.14859476

Time = 158.50 fs
E(CASSCF/6-31G(d)) = -479.6100193

C	-0.86153223	-0.14871973	-0.46587940
C	0.53188641	0.16820697	-0.34141767
C	1.29004790	1.16090931	-1.00764387
C	1.43496347	-0.50262944	0.57548548
C	2.74819712	-0.01131559	0.39616136
C	2.65245188	1.11460215	-0.48348094
N	-1.78826446	0.49503739	0.31198052
C	-3.03433727	-0.21359383	0.53933559
H	3.53150178	1.69339775	-0.73619598
C	-1.43516898	-1.54556223	-0.74662080
C	-2.93342304	-1.45305609	-0.35855071
H	-3.53569570	-1.30319960	-1.24490507
H	-3.34804884	-2.32325744	0.13829418
H	-3.09659829	-0.51746645	1.57950534
H	-3.88746774	0.41749651	0.26634803
H	-1.28010973	-1.78613155	-1.79111810
H	0.85603151	1.85952955	-1.72682696
C	-1.81776895	1.94086875	0.51504752
H	-0.81586226	2.30308670	0.41439802
H	-2.43100520	2.37742406	-0.27178161
H	-2.22278002	2.09791707	1.50707274
H	1.13779727	-1.38311105	1.12643986
C	3.97174600	-0.81821900	0.58843463
H	4.18428563	-1.34685070	-0.33685463
H	4.84442652	-0.22583769	0.85213874
H	3.83313996	-1.56625191	1.36300973
H	-0.90095286	-2.29716064	-0.16210421

Time = 159.00 fs
E(CASSCF/6-31G(d)) = -479.6103544

C	-0.85954451	-0.14755732	-0.46404730
C	0.52880981	0.16966338	-0.34311372
C	1.29027966	1.15525515	-1.01493247
C	1.42858291	-0.49959830	0.57585735

C	2.74934301	-0.00921171	0.39662799
C	2.65302165	1.11198300	-0.48950890
N	-1.78659186	0.49687689	0.31639609
C	-3.03248765	-0.21869133	0.54100428
H	3.53666698	1.68612787	-0.74625754
C	-1.43414636	-1.54295248	-0.75224882
C	-2.93240693	-1.45137571	-0.36255079
H	-3.53748994	-1.29884196	-1.24563004
H	-3.34365854	-2.32404980	0.13122086
H	-3.09097646	-0.51539070	1.58858738
H	-3.88183255	0.42499416	0.27356342
H	-1.29405151	-1.78384278	-1.80029223
H	0.85929988	1.85187017	-1.73869512
C	-1.81675567	1.93835710	0.52148999
H	-0.81711894	2.29799738	0.40806615
H	-2.43320627	2.37184682	-0.26075862
H	-2.21171573	2.09744911	1.51383043
H	1.14232315	-1.38164592	1.14082855
C	3.96918803	-0.81655291	0.59634453
H	4.18637263	-1.35015377	-0.32673403
H	4.84415192	-0.21940984	0.84785273
H	3.82828968	-1.55686686	1.37724094
H	-0.90384982	-2.30017023	-0.17580585

Time = 159.50 fs
E(CASSCF/6-31G(d)) = -479.6106118

C	-0.85812181	-0.14647525	-0.46171207
C	0.52620522	0.17114744	-0.34478743
C	1.29090564	1.14967982	-1.02225779
C	1.42213006	-0.49655310	0.57629814
C	2.75001253	-0.00701690	0.39701477
C	2.65328147	1.10929887	-0.49571393
N	-1.78474768	0.49859019	0.32057127
C	-3.03063892	-0.22382366	0.54264613
H	3.54036531	1.67912727	-0.75705463
C	-1.43317218	-1.54025156	-0.75800277
C	-2.93138531	-1.44967466	-0.36661364
H	-3.53922134	-1.29437430	-1.24669284
H	-3.33846355	-2.32509069	0.12407597
H	-3.08569039	-0.51257650	1.59737529
H	-3.87535190	0.43176548	0.28161648
H	-1.30797882	-1.78190492	-1.80941120
H	0.86371438	1.84369025	-1.74954569
C	-1.81565836	1.93567904	0.52793367
H	-0.81782549	2.29370987	0.40238051
H	-2.43595501	2.36659472	-0.24901050

H	-2.20061804	2.09820962	1.52042963
H	1.14736913	-1.37959473	1.15489742
C	3.96674599	-0.81495077	0.60431219
H	4.18895951	-1.35363504	-0.31639487
H	4.84376269	-0.21300840	0.84355519
H	3.82366461	-1.54752815	1.39161101
H	-0.90659087	-2.30235022	-0.18959418

Time = 160.00 fs
E(CASSCF/6-31G(d)) = -479.6108696

C	-0.85728513	-0.14547096	-0.45888816
C	0.52408904	0.17265949	-0.34643216
C	1.29192817	1.14418764	-1.02962207
C	1.41567173	-0.49350547	0.57683790
C	2.75015075	-0.00474018	0.39731711
C	2.65324580	1.10654311	-0.50209383
N	-1.78273475	0.50012517	0.32449789
C	-3.02878771	-0.22893874	0.54429918
H	3.54246894	1.67236539	-0.76856471
C	-1.43227426	-1.53745367	-0.76390229
C	-2.93035583	-1.44795618	-0.37072443
H	-3.54089202	-1.28977491	-1.24812449
H	-3.33252292	-2.32642345	0.11688355
H	-3.08076333	-0.50912508	1.60546778
H	-3.86803000	0.43757599	0.29047912
H	-1.32173904	-1.78017634	-1.81834707
H	0.86921769	1.83501829	-1.75941197
C	-1.81446230	1.93284249	0.53436515
H	-0.81789091	2.29030328	0.39737244
H	-2.43939896	2.36179332	-0.23666957
H	-2.18957097	2.10019887	1.52710700
H	1.15282791	-1.37661433	1.16835329
C	3.96443893	-0.81340316	0.61232084
H	4.19193012	-1.35731387	-0.30572765
H	4.84316080	-0.20672567	0.83935852
H	3.81932828	-1.53824170	1.40612318
H	-0.90904205	-2.30380672	-0.20335063

Time = 160.50 fs
E(CASSCF/6-31G(d)) = -479.6112232

C	-0.85704351	-0.14454151	-0.45559696
C	0.52246845	0.17419960	-0.34804234
C	1.29334842	1.13877656	-1.03702215
C	1.40927499	-0.49046180	0.57750409
C	2.74971245	-0.00239303	0.39753139

C	2.65292769	1.10370783	-0.50864531
N	-1.78055993	0.50143316	0.32817276
C	-3.02692562	-0.23398760	0.54599922
H	3.54290804	1.66583271	-0.78076671
C	-1.43148042	-1.53455201	-0.76996547
C	-2.92931671	-1.44622421	-0.37486973
H	-3.54249760	-1.28502896	-1.24993355
H	-3.32590619	-2.32808693	0.10966411
H	-3.07621087	-0.50516754	1.61252679
H	-3.85994051	0.44225103	0.30008520
H	-1.33517632	-1.77851129	-1.82697939
H	0.87571026	1.82594391	-1.76839655
C	-1.81314940	1.92986133	0.54077235
H	-0.81730159	2.28782045	0.39306471
H	-2.44366152	2.35755131	-0.22386454
H	-2.17864233	2.10338449	1.53407510
H	1.15856440	-1.37243624	1.18094595
C	3.96228180	-0.81189952	0.62035418
H	4.19516525	-1.36120773	-0.29463108
H	4.84225962	-0.20064405	0.83537915
H	3.81534139	-1.52900520	1.42077313
H	-0.91107287	-2.30466256	-0.21695239

Time = 161.00 fs
E(CASSCF/6-31G(d)) = -479.6117553

C	-0.85739334	-0.14368371	-0.45186677
C	0.52134087	0.17576787	-0.34961358
C	1.29516571	1.13343859	-1.04444992
C	1.40300681	-0.48742202	0.57832035
C	2.74866272	0.00001032	0.39765528
C	2.65233844	1.10078380	-0.51536388
N	-1.77823390	0.50246989	0.33159785
C	-3.02503950	-0.23892540	0.54777785
H	3.54167529	1.65954222	-0.79364201
C	-1.43081749	-1.53153951	-0.77620719
C	-2.92826705	-1.44448355	-0.37903751
H	-3.54402670	-1.28012925	-1.25210510
H	-3.31869097	-2.33011120	0.10243258
H	-3.07204068	-0.50086494	1.61829747
H	-3.85122567	0.44568213	0.31033293
H	-1.34813341	-1.77676539	-1.83520767
H	0.88305531	1.81661210	-1.77666643
C	-1.81169997	1.92675579	0.54714508
H	-0.81611971	2.28626793	0.38947311
H	-2.44883018	2.35395263	-0.21070686
H	-2.16787723	2.10769866	1.54150079

H	1.16441755	-1.36688109	1.19248220
C	3.96028450	-0.81042829	0.62839704
H	4.19854604	-1.36533438	-0.28301863
H	4.84099036	-0.19483115	0.83173592
H	3.81176042	-1.51980777	1.43554826
H	-0.91256560	-2.30504549	-0.23028178

Time = 161.50 fs
E(CASSCF/6-31G(d)) = -479.6125200

C	-0.85831813	-0.14289434	-0.44773294
C	0.52069390	0.17736484	-0.35114286
C	1.29737640	1.12816049	-1.05189315
C	1.39693359	-0.48437870	0.57930560
C	2.74697711	0.00245275	0.39768775
C	2.65148770	1.09776036	-0.52224372
N	-1.77577106	0.50319654	0.33478049
C	-3.02311198	-0.24371232	0.54966094
H	3.53882586	1.65352840	-0.80717495
C	-1.43031019	-1.52840977	-0.78263728
C	-2.92720694	-1.44273996	-0.38321715
H	-3.54546062	-1.27507636	-1.25460119
H	-3.31096017	-2.33251299	0.09519607
H	-3.06825216	-0.49640656	1.62262189
H	-3.84209130	0.44782953	0.32108859
H	-1.36045448	-1.77479934	-1.84296127
H	0.89108627	1.80721145	-1.78444249
C	-1.81009485	1.92355240	0.55347557
H	-0.81447410	2.28561829	0.38660629
H	-2.45494655	2.35104933	-0.19727883
H	-2.15729311	2.11303646	1.54948763
H	1.17020399	-1.35986793	1.20283667
C	3.95845138	-0.80897700	0.63643598
H	4.20195712	-1.36971451	-0.27082451
H	4.83930756	-0.18933525	0.82854746
H	3.80863604	-1.51063019	1.45042879
H	-0.91342531	-2.30507360	-0.24323704

Time = 162.00 fs
E(CASSCF/6-31G(d)) = -479.6135319

C	-0.85978844	-0.14217065	-0.44323840
C	0.52050531	0.17899225	-0.35262848
C	1.29997239	1.12292479	-1.05933681
C	1.39112094	-0.48131674	0.58047361
C	2.74464170	0.00491397	0.39762934
C	2.65038406	1.09462567	-0.52927767

N	-1.77318946	0.50358065	0.33773387
C	-3.02112217	-0.24831427	0.55166735
H	3.53447387	1.64784496	-0.82135199
C	-1.42998002	-1.52515788	-0.78925934
C	-2.92613756	-1.44100025	-0.38739915
H	-3.54677499	-1.26987835	-1.25736375
H	-3.30280001	-2.33529262	0.08795286
H	-3.06483651	-0.49200617	1.62544712
H	-3.83279843	0.44872151	0.33219183
H	-1.37198800	-1.77248352	-1.85020775
H	0.89961711	1.79795878	-1.79198376
C	-1.80831762	1.92028348	0.55975882
H	-0.81254350	2.28581543	0.38446546
H	-2.46199920	2.34885751	-0.18362633
H	-2.14687884	2.11925550	1.55806535
H	1.17572334	-1.35141702	1.21195823
C	3.95678090	-0.80753242	0.64445971
H	4.20528957	-1.37437246	-0.25800730
H	4.83719248	-0.18418294	0.82592974
H	3.80601175	-1.50144606	1.46538824
H	-0.91358853	-2.30484395	-0.25574037

Time = 162.50 fs
E(CASSCF/6-31G(d)) = -479.6147608

C	-0.86176212	-0.14151097	-0.43843487
C	0.52074317	0.18065413	-0.35407019
C	1.30293889	1.11771107	-1.06676476
C	1.38563379	-0.47821350	0.58183289
C	2.74165282	0.00737002	0.39748241
C	2.64903623	1.09136718	-0.53645718
N	-1.77051088	0.50359727	0.34047777
C	-3.01904658	-0.25270320	0.55380816
H	3.52878498	1.64256024	-0.83615995
C	-1.42984419	-1.52178114	-0.79606992
C	-2.92506105	-1.43927227	-0.39157490
H	-3.54794193	-1.26455009	-1.26031908
H	-3.29429839	-2.33843197	0.08069202
H	-3.06177746	-0.48789740	1.62682771
H	-3.82365139	0.44844937	0.34346218
H	-1.38259034	-1.76970158	-1.85695796
H	0.90845488	1.78907860	-1.79956666
C	-1.80635658	1.91698605	0.56599264
H	-0.81053582	2.28678147	0.38304309
H	-2.46992067	2.34735645	-0.16975651
H	-2.13659686	2.12617790	1.56718803
H	1.18076447	-1.34164754	1.21987318

C	3.95526566	-0.80608059	0.65245944
H	4.20844318	-1.37933634	-0.24455214
H	4.83465475	-0.17937783	0.82399313
H	3.80392229	-1.49222329	1.48039462
H	-0.91303066	-2.30442313	-0.26774454

Time = 163.00 fs
E(CASSCF/6-31G(d)) = -479.6161356

C	-0.86418496	-0.14091573	-0.43338488
C	0.52136639	0.18235904	-0.35546954
C	1.30625084	1.11249700	-1.07416181
C	1.38053715	-0.47503930	0.58338726
C	2.73801676	0.00979283	0.39725112
C	2.64745526	1.08797165	-0.54377195
N	-1.76776098	0.50323004	0.34303986
C	-3.01686016	-0.25685703	0.55608620
H	3.52196485	1.63775121	-0.85158240
C	-1.42991465	-1.51827963	-0.80305807
C	-2.92398030	-1.43756476	-0.39573625
H	-3.54893303	-1.25911205	-1.26338328
H	-3.28554365	-2.34189414	0.07339382
H	-3.05905251	-0.48432810	1.62692260
H	-3.81498174	0.44715765	0.35470688
H	-1.39212981	-1.76635333	-1.86326806
H	0.91741394	1.78077908	-1.80746024
C	-1.80420636	1.91370021	0.57217735
H	-0.80866609	2.28842472	0.38232066
H	-2.47858913	2.34649159	-0.15564047
H	-2.12638827	2.13359443	1.57674154
H	1.18511258	-1.33077014	1.22668412
C	3.95389283	-0.80460683	0.66042917
H	4.21132807	-1.38463738	-0.23047071
H	4.83173214	-0.17490116	0.82283923
H	3.80239230	-1.48292585	1.49541244
H	-0.91177162	-2.30384288	-0.27923680

Time = 163.50 fs
E(CASSCF/6-31G(d)) = -479.6175581

C	-0.86699135	-0.14038904	-0.42816628
C	0.52232499	0.18412466	-0.35683115
C	1.30986634	1.10725868	-1.08151544
C	1.37589839	-0.47175876	0.58513732
C	2.73374947	0.01214962	0.39694101
C	2.64565863	1.08442470	-0.55120859
N	-1.76497007	0.50247299	0.34545825

C	-3.01453742	-0.26075892	0.55849608
H	3.51424469	1.63349615	-0.86759425
C	-1.43019698	-1.51465649	-0.81020540
C	-2.92289858	-1.43588711	-0.39987525
H	-3.54972246	-1.25358891	-1.26646853
H	-3.27662412	-2.34562501	0.06603116
H	-3.05663564	-0.48155462	1.62598605
H	-3.80712913	0.44502936	0.36572949
H	-1.40049134	-1.76235751	-1.86923833
H	0.92633067	1.77322548	-1.81590010
C	-1.80186910	1.91046656	0.57831479
H	-0.80713519	2.29064839	0.38226674
H	-2.48783436	2.34618301	-0.14122127
H	-2.11618054	2.14127154	1.58655925
H	1.18855694	-1.31907296	1.23256409
C	3.95264509	-0.80309561	0.66836591
H	4.21386482	-1.39030782	-0.21579929
H	4.82848867	-0.17071442	0.82255801
H	3.80143493	-1.47351561	1.51040465
H	-0.90988038	-2.30310324	-0.29023943

Time = 164.00 fs
E(CASSCF/6-31G(d)) = -479.6189101

C	-0.87010449	-0.13994220	-0.42288355
C	0.52356001	0.18598767	-0.35816619
C	1.31371143	1.10196841	-1.08881614
C	1.37179253	-0.46833387	0.58708263
C	2.72887807	0.01440294	0.39655692
C	2.64368001	1.08070772	-0.55874548
N	-1.76217583	0.50133470	0.34778841
C	-3.01205265	-0.26439561	0.56102480
H	3.50586488	1.62986537	-0.88415410
C	-1.43068835	-1.51091823	-0.81748583
C	-2.92181894	-1.43424885	-0.40398371
H	-3.55028994	-1.24800811	-1.26949001
H	-3.26762843	-2.34955736	0.05857125
H	-3.05450370	-0.47983758	1.62435267
H	-3.80042149	0.44226623	0.37633851
H	-1.40758314	-1.75765531	-1.87500947
H	0.93507683	1.76651130	-1.82506471
C	-1.79935581	1.90732207	0.58440621
H	-0.80611030	2.29336151	0.38283506
H	-2.49744749	2.34634448	-0.12642658
H	-2.10589696	2.14896257	1.59644467
H	1.19089920	-1.30689887	1.23774536
C	3.95150231	-0.80152995	0.67627044

H	4.21598183	-1.39637737	-0.20059450
H	4.82501177	-0.16676471	0.82322551
H	3.80105094	-1.46395388	1.52533467
H	-0.90748206	-2.30218884	-0.30080776

Time = 164.50 fs
E(CASSCF/6-31G(d)) = -479.6200263

C	-0.87343279	-0.13960326	-0.41770093
C	0.52500272	0.18802475	-0.35950292
C	1.31763661	1.09658572	-1.09605180
C	1.36831028	-0.46473370	0.58922118
C	2.72345397	0.01652027	0.39609217
C	2.64159873	1.07678446	-0.56633124
N	-1.75943579	0.49985294	0.35012475
C	-3.00937680	-0.26775306	0.56365255
H	3.49705999	1.62690271	-0.90119695
C	-1.43137102	-1.50707537	-0.82486199
C	-2.92074357	-1.43265870	-0.40805226
H	-3.55062299	-1.24239939	-1.27237478
H	-3.25864827	-2.35362036	0.05097665
H	-3.05265437	-0.47944624	1.62241936
H	-3.79516437	0.43906414	0.38635343
H	-1.41334935	-1.75221843	-1.88075913
H	0.94357035	1.76062235	-1.83505869
C	-1.79669042	1.90429016	0.59044581
H	-0.80570623	2.29649831	0.38396401
H	-2.50719297	2.34693473	-0.11118745
H	-2.09546836	2.15642789	1.60620214
H	1.19196082	-1.29460548	1.24250432
C	3.95044504	-0.79988997	0.68414952
H	4.21760371	-1.40286711	-0.18492496
H	4.82140751	-0.16299526	0.82490206
H	3.80122928	-1.45420161	1.54016790
H	-0.90477750	-2.30112100	-0.31102605

Time = 165.00 fs
E(CASSCF/6-31G(d)) = -479.6205549

C	-0.87685153	-0.13943658	-0.41290356
C	0.52658050	0.19034505	-0.36090259
C	1.32133423	1.09104953	-1.10318596
C	1.36551966	-0.46096763	0.59151372
C	2.71762346	0.01854429	0.39548514
C	2.63961268	1.07256103	-0.57381439
N	-1.75687446	0.49814036	0.35265066
C	-3.00646533	-0.27080506	0.56635121

H	3.48805940	1.62455248	-0.91866012
C	-1.43219553	-1.50314175	-0.83226775
C	-2.91967290	-1.43112320	-0.41206786
H	-3.55071882	-1.23679892	-1.27507605
H	-3.24978774	-2.35775980	0.04320276
H	-3.05114861	-0.48068516	1.62063163
H	-3.79166427	0.43558084	0.39560495
H	-1.41778721	-1.74606378	-1.88670311
H	0.95177857	1.75539783	-1.84590099
C	-1.79392136	1.90135658	0.59640450
H	-0.80596914	2.30005892	0.38558323
H	-2.51682668	2.34808692	-0.09546548
H	-2.08484402	2.16347554	1.61568107
H	1.19157529	-1.28248977	1.24715232
C	3.94945904	-0.79815266	0.69202022
H	4.21862131	-1.40978237	-0.16885488
H	4.81777771	-0.15935795	0.82762209
H	3.80195423	-1.44421777	1.55487541
H	-0.90206037	-2.30008928	-0.32099648

Time = 165.50 fs
E(CASSCF/6-31G(d)) = -479.6201052

C	-0.88033429	-0.13934384	-0.40807528
C	0.52836071	0.19231621	-0.36217467
C	1.32544108	1.08546709	-1.11043983
C	1.36283372	-0.45691897	0.59376345
C	2.71148197	0.02076391	0.39470928
C	2.63744835	1.06804066	-0.58145118
N	-1.75450088	0.49613970	0.35530385
C	-3.00326945	-0.27361985	0.56914692
H	3.47890021	1.62251800	-0.93715618
C	-1.43321614	-1.49899951	-0.83983823
C	-2.91858916	-1.42962467	-0.41611726
H	-3.55060734	-1.23111458	-1.27759088
H	-3.24092586	-2.36195319	0.03504062
H	-3.04981090	-0.48373258	1.61931236
H	-3.78996262	0.43184915	0.40421477
H	-1.42079316	-1.73893185	-1.89311865
H	0.95982490	1.75091809	-1.85769963
C	-1.79098543	1.89852142	0.60245619
H	-0.80702499	2.30397548	0.38768613
H	-2.52640832	2.34949896	-0.07877532
H	-2.07362834	2.16997034	1.62487688
H	1.18947528	-1.27065617	1.25212086
C	3.94845053	-0.79630702	0.70003048
H	4.21912617	-1.41735369	-0.15212015

H	4.81395746	-0.15556465	0.83136448
H	3.80321813	-1.43376149	1.56980651
H	-0.89892873	-2.29871811	-0.33096988

Time = 166.00 fs
E(CASSCF/6-31G(d)) = -479.6187537

C	-0.88352412	-0.13951092	-0.40412803
C	0.53011906	0.19438337	-0.36351078
C	1.32881703	1.07992640	-1.11731179
C	1.36073126	-0.45290078	0.59588020
C	2.70557346	0.02307604	0.39366763
C	2.63576259	1.06322941	-0.58844748
N	-1.75265367	0.49421217	0.35832820
C	-2.99989922	-0.27599594	0.57185674
H	3.47025500	1.62079171	-0.95542273
C	-1.43425506	-1.49491774	-0.84705090
C	-2.91754581	-1.42824417	-0.41995710
H	-3.55030804	-1.22569675	-1.27979107
H	-3.23267589	-2.36595659	0.02689686
H	-3.04890531	-0.48874544	1.61894240
H	-3.79035790	0.42811106	0.41157994
H	-1.42237393	-1.73134122	-1.89989802
H	0.96734311	1.74685655	-1.86969934
C	-1.78814063	1.89585170	0.60818811
H	-0.80867408	2.30817024	0.39003424
H	-2.53513582	2.35158180	-0.06210559
H	-2.06249447	2.17547233	1.63327852
H	1.18573013	-1.25986290	1.25739483
C	3.94749366	-0.79440252	0.70778928
H	4.21888695	-1.42510191	-0.13568802
H	4.81035021	-0.15187066	0.83595793
H	3.80491492	-1.42336899	1.58411140
H	-0.89622630	-2.29766700	-0.34056887

Time = 166.50 fs
E(CASSCF/6-31G(d)) = -479.6176030

C	-0.88670209	-0.13979195	-0.40030135
C	0.53197577	0.19617090	-0.36466426
C	1.33273085	1.07423549	-1.12457814
C	1.35885528	-0.44835427	0.59808088
C	2.69918437	0.02535947	0.39258008
C	2.63377740	1.05812462	-0.59594939
N	-1.75099261	0.49196997	0.36170126
C	-2.99617075	-0.27817773	0.57470484
H	3.46144896	1.61938923	-0.97481553

C	-1.43549662	-1.49054095	-0.85459182
C	-2.91645925	-1.42688099	-0.42391239
H	-3.54983819	-1.22009612	-1.28178312
H	-3.22438425	-2.36991971	0.01811184
H	-3.04808963	-0.49605302	1.61959313
H	-3.79253410	0.42409242	0.41833205
H	-1.42251231	-1.72257320	-1.90762899
H	0.97497219	1.74303829	-1.88264665
C	-1.78510963	1.89327848	0.61417227
H	-0.81109451	2.31271875	0.39271127
H	-2.54358660	2.35368103	-0.04395655
H	-2.05038636	2.18006813	1.64133712
H	1.17997266	-1.24963810	1.26351796
C	3.94643216	-0.79230503	0.71588238
H	4.21811316	-1.43368977	-0.11838545
H	4.80668623	-0.14776097	0.84169915
H	3.80704666	-1.41217884	1.59895336
H	-0.89344969	-2.29595000	-0.35064912

Time = 167.00 fs
E(CASSCF/6-31G(d)) = -479.6162746

C	-0.88944252	-0.14030255	-0.39689231
C	0.53364391	0.19762778	-0.36551767
C	1.33697552	1.06896077	-1.13172300
C	1.35751434	-0.44376909	0.59995380
C	2.69261655	0.02727712	0.39161802
C	2.63154119	1.05306172	-0.60379495
N	-1.74985841	0.48980314	0.36526469
C	-2.99228292	-0.28002330	0.57747391
H	3.45307063	1.61799904	-0.99441540
C	-1.43676212	-1.48618659	-0.86181877
C	-2.91538676	-1.42560963	-0.42776773
H	-3.54929730	-1.21462068	-1.28348641
H	-3.21658112	-2.37359998	0.00907245
H	-3.04722647	-0.50534404	1.62140696
H	-3.79646258	0.41976217	0.42411193
H	-1.42107337	-1.71265374	-1.91598749
H	0.98250971	1.73922161	-1.89571746
C	-1.78210268	1.89089122	0.62009723
H	-0.81382107	2.31748722	0.39544662
H	-2.55089026	2.35595884	-0.02524589
H	-2.03803915	2.18328220	1.64870861
H	1.17256744	-1.24061357	1.27019206
C	3.94531850	-0.79002808	0.72401415
H	4.21652527	-1.44252823	-0.10121281
H	4.80339223	-0.14350212	0.84831414

H	3.80941945	-1.40073607	1.61358905
H	-0.89106796	-2.29415871	-0.36095256

Time = 167.50 fs
E(CASSCF/6-31G(d)) = -479.6149434

C	-0.89169381	-0.14101113	-0.39381090
C	0.53507935	0.19880677	-0.36614140
C	1.34143951	1.06409839	-1.13876670
C	1.35668242	-0.43917669	0.60149442
C	2.68593518	0.02886290	0.39076607
C	2.62911828	1.04800404	-0.61194776
N	-1.74922880	0.48769314	0.36894513
C	-2.98829693	-0.28155022	0.58015950
H	3.44516153	1.61643147	-1.01410865
C	-1.43805401	-1.48186968	-0.86871587
C	-2.91432503	-1.42443481	-0.43152375
H	-3.54874896	-1.20927069	-1.28490132
H	-3.20933553	-2.37691329	-0.00020928
H	-3.04617600	-0.51651990	1.62430136
H	-3.80180561	0.41497467	0.42899299
H	-1.41803206	-1.70147184	-1.92494183
H	0.99001695	1.73519219	-1.90869141
C	-1.77912540	1.88873451	0.62600252
H	-0.81671387	2.32235935	0.39809981
H	-2.55690463	2.35814861	-0.00599524
H	-2.02546898	2.18491084	1.65541114
H	1.16358824	-1.23275565	1.27740202
C	3.94412606	-0.78755584	0.73219740
H	4.21415706	-1.45158848	-0.08419053
H	4.80053101	-0.13905581	0.85572936
H	3.81196812	-1.38905969	1.62808843
H	-0.88912527	-2.29210111	-0.37158451

Time = 168.00 fs
E(CASSCF/6-31G(d)) = -479.6136043

C	-0.89341543	-0.14189385	-0.39101951
C	0.53624782	0.19973958	-0.36657832
C	1.34602684	1.05964747	-1.14573049
C	1.35631796	-0.43461213	0.60271497
C	2.67922092	0.03013557	0.39001964
C	2.62657151	1.04293979	-0.62037980
N	-1.74908740	0.48563662	0.37270435
C	-2.98427419	-0.28277541	0.58276741
H	3.43773016	1.61450018	-1.03372154
C	-1.43936628	-1.47760289	-0.87527634

C	-2.91326959	-1.42335784	-0.43517851
H	-3.54825793	-1.20404879	-1.28604514
H	-3.20272284	-2.37980308	-0.00969824
H	-3.04484056	-0.52943934	1.62806190
H	-3.80817760	0.40957038	0.43306755
H	-1.41341799	-1.68897467	-1.93440288
H	0.99754482	1.73074801	-1.92135071
C	-1.77618841	1.88683638	0.63191139
H	-0.81962378	2.32724105	0.40053709
H	-2.56156264	2.36007611	0.01370299
H	-2.01271055	2.18481608	1.66153228
H	1.15316330	-1.22588463	1.28501595
C	3.94283320	-0.78487813	0.74043268
H	4.21105112	-1.46080053	-0.06733647
H	4.79812203	-0.13440822	0.86383640
H	3.81460807	-1.37717595	1.64250113
H	-0.88766469	-2.28966511	-0.38260846

Time = 168.50 fs
E(CASSCF/6-31G(d)) = -479.6122013

C	-0.89457993	-0.14292556	-0.38848464
C	0.53712319	0.20045755	-0.36686881
C	1.35064170	1.05560120	-1.15263397
C	1.35636876	-0.43011843	0.60363904
C	2.67255791	0.03111471	0.38937418
C	2.62396708	1.03786047	-0.62906352
N	-1.74941122	0.48363131	0.37650743
C	-2.98027796	-0.28372184	0.58531215
H	3.43076562	1.61201862	-1.05306830
C	-1.44069160	-1.47339479	-0.88150371
C	-2.91221499	-1.42237654	-0.43873177
H	-3.54788895	-1.19895395	-1.28694660
H	-3.19681558	-2.38223966	-0.01934352
H	-3.04313422	-0.54390832	1.63236243
H	-3.81516719	0.40338931	0.43645044
H	-1.40729101	-1.67512540	-1.94422468
H	1.00512901	1.72572431	-1.93351557
C	-1.77329807	1.88521483	0.63784367
H	-0.82240183	2.33204302	0.40263455
H	-2.56487151	2.36161998	0.03371379
H	-1.99980896	2.18291176	1.66722605
H	1.14147162	-1.21971015	1.29281157
C	3.94142233	-0.78198696	0.74871892
H	4.20725677	-1.47006800	-0.05064421
H	4.79614782	-0.12957021	0.87250620
H	3.81724762	-1.36511155	1.65687585

H	-0.88670304	-2.28679267	-0.39405694

Time = 169.00 fs
E(CASSCF/6-31G(d)) = -479.6106584

C	-0.89517261	-0.14407912	-0.38617192
C	0.53768672	0.20099312	-0.36705286
C	1.35518794	1.05194613	-1.15949301
C	1.35677367	-0.42574437	0.60429819
C	2.66603233	0.03182212	0.38882403
C	2.62137349	1.03275823	-0.63796970
N	-1.75016979	0.48167510	0.38031899
C	-2.97637162	-0.28441991	0.58781409
H	3.42425243	1.60881051	-1.07196344
C	-1.44202208	-1.46924969	-0.88741033
C	-2.91115542	-1.42148543	-0.44218515
H	-3.54770370	-1.19398205	-1.28764175
H	-3.19168187	-2.38422012	-0.02907965
H	-3.04098069	-0.55968614	1.63680161
H	-3.82236508	0.39629231	0.43927199
H	-1.39973828	-1.65990209	-1.95421339
H	1.01278373	1.72000681	-1.94505907
C	-1.77045508	1.88387863	0.64381624
H	-0.82490917	2.33667720	0.40428195
H	-2.56691285	2.36270986	0.05386552
H	-1.98681652	2.17916175	1.67270486
H	1.12873596	-1.21387167	1.30050515
C	3.93987980	-0.77887647	0.75705416
H	4.20282922	-1.47927214	-0.03408513
H	4.79455799	-0.12457626	0.88159331
H	3.81979108	-1.35289133	1.67125680
H	-0.88622669	-2.28348056	-0.40593104

Time = 169.50 fs
E(CASSCF/6-31G(d)) = -479.6089028

C	-0.89519090	-0.14532594	-0.38404541
C	0.53792666	0.20137936	-0.36716985
C	1.35957081	1.04866248	-1.16631844
C	1.35746444	-0.42154089	0.60472813
C	2.65973101	0.03228154	0.38836223
C	2.61885934	1.02762483	-0.64706704
N	-1.75132557	0.47976640	0.38410289
C	-2.97261623	-0.28490780	0.59029630
H	3.41818453	1.60471997	-1.09023199
C	-1.44334938	-1.46516718	-0.89301588
C	-2.91008528	-1.42067590	-0.44554217

H	-3.54775711	-1.18912696	-1.28817005
H	-3.18738356	-2.38576684	-0.03882896
H	-3.03831434	-0.57649664	1.64094547
H	-3.82939002	0.38817900	0.44167086
H	-1.39087330	-1.64330097	-1.96414099
H	1.02049694	1.71353868	-1.95591752
C	-1.76765417	1.88282753	0.64984307
H	-0.82702629	2.34105498	0.40538736
H	-2.56783934	2.36332666	0.07395728
H	-1.97378902	2.17357779	1.67822674
H	1.11521161	-1.20798163	1.30778684
C	3.93819565	-0.77554305	0.76543620
H	4.19782874	-1.48827646	-0.01761351
H	4.79327499	-0.11948076	0.89094077
H	3.82214109	-1.34053622	1.68567909
H	-0.88619091	-2.27978023	-0.41820127

Time = 170.00 fs
E(CASSCF/6-31G(d)) = -479.6069024

C	-0.89464360	-0.14663676	-0.38206816
C	0.53783792	0.20164923	-0.36725771
C	1.36369898	1.04572469	-1.17311545
C	1.35836763	-0.41755754	0.60496527
C	2.65374011	0.03251820	0.38798055
C	2.61649116	1.02245095	-0.65632216
N	-1.75283506	0.47790500	0.38782281
C	-2.96906819	-0.28523116	0.59278141
H	3.41257579	1.59962092	-1.10771895
C	-1.44466446	-1.46114218	-0.89834543
C	-2.90899979	-1.41993654	-0.44880817
H	-3.54809407	-1.18438154	-1.28857042
H	-3.18397396	-2.38692397	-0.04850505
H	-3.03508236	-0.59404200	1.64436957
H	-3.83591105	0.37900081	0.44378756
H	-1.38083450	-1.62533932	-1.97376207
H	1.02822879	1.70632335	-1.96609544
C	-1.76488426	1.88205271	0.65593537
H	-0.82866086	2.34508658	0.40588077
H	-2.56786758	2.36350103	0.09376925
H	-1.96078073	2.16621570	1.68407795
H	1.10117358	-1.20166624	1.31435442
C	3.93636296	-0.77198526	0.77386320
H	4.19232019	-1.49693384	-0.00117268
H	4.79220202	-0.11435233	0.90038493
H	3.82420069	-1.32806079	1.70016510
H	-0.88652227	-2.27579562	-0.43081119

Time = 170.50 fs
E(CASSCF/6-31G(d)) = -479.6046702

C	-0.89355002	-0.14798249	-0.38020278
C	0.53742171	0.20183482	-0.36735214
C	1.36748666	1.04310236	-1.17988317
C	1.35940697	-0.41383931	0.60504347
C	2.64814357	0.03255826	0.38766956
C	2.61433122	1.01722568	-0.66569972
N	-1.75464994	0.47609261	0.39144280
C	-2.96577723	-0.28544224	0.59528890
H	3.40746804	1.59342367	-1.12429695
C	-1.44595734	-1.45716525	-0.90342726
C	-2.90789561	-1.41925371	-0.45199018
H	-3.54874659	-1.17973870	-1.28887770
H	-3.18149546	-2.38775199	-0.05801698
H	-3.03124644	-0.61201708	1.64669574
H	-3.84166494	0.36876899	0.44575905
H	-1.36978299	-1.60605801	-1.98283114
H	1.03591123	1.69842326	-1.97566633
C	-1.76212933	1.88153771	0.66210205
H	-0.82975315	2.34868094	0.40571717
H	-2.56726534	2.36330818	0.11307550
H	-1.94783971	2.15716936	1.69055051
H	1.08690262	-1.19459966	1.31994323
C	3.93437724	-0.76820387	0.78233431
H	4.18637268	-1.50509434	0.01529795
H	4.79123188	-0.10926686	0.90976055
H	3.82587532	-1.31547187	1.71472181
H	-0.88712313	-2.27167676	-0.44368289

Time = 171.00 fs
E(CASSCF/6-31G(d)) = -479.6022737

C	-0.89193900	-0.14933507	-0.37841215
C	0.53668541	0.20196688	-0.36748596
C	1.37085527	1.04076139	-1.18661467
C	1.36050552	-0.41042442	0.60499151
C	2.64302146	0.03242830	0.38741861
C	2.61243575	1.01193626	-0.67516283
N	-1.75671843	0.47433343	0.39492791
C	-2.96278481	-0.28559867	0.59783301
H	3.40293446	1.58607902	-1.13987276
C	-1.44721675	-1.45322327	-0.90829070
C	-2.90677129	-1.41861217	-0.45509669
H	-3.54973154	-1.17519237	-1.28911997

H	-3.17997694	-2.38832072	-0.06727398
H	-3.02678440	-0.63012347	1.64762067
H	-3.84646883	0.35755790	0.44771418
H	-1.35789971	-1.58552314	-1.99111934
H	1.04345122	1.68995398	-1.98476793
C	-1.75936988	1.88125972	0.66835041
H	-0.83027827	2.35174706	0.40487853
H	-2.56633387	2.36285958	0.13165864
H	-1.93500339	2.14656370	1.69791608
H	1.07267218	-1.18652890	1.32434923
C	3.93223569	-0.76420167	0.79085013
H	4.18005938	-1.51261393	0.03185583
H	4.79025680	-0.10430043	0.91890613
H	3.82707467	-1.30276802	1.72933962
H	-0.88787860	-2.26760940	-0.45672438

Time = 171.50 fs
E(CASSCF/6-31G(d)) = -479.5998113

C	-0.88984803	-0.15066821	-0.37665989
C	0.53564234	0.20207440	-0.36768867
C	1.37373526	1.03866532	-1.19329726
C	1.36158829	-0.40734250	0.60483115
C	2.63844818	0.03215489	0.38721593
C	2.61085318	1.00656791	-0.68467348
N	-1.75898656	0.47263437	0.39824465
C	-2.96012289	-0.28576194	0.60042121
H	3.39908069	1.57757925	-1.15439076
C	-1.44842972	-1.44930055	-0.91296388
C	-2.90562776	-1.41799589	-0.45813732
H	-3.55104941	-1.17073818	-1.28931684
H	-3.17943141	-2.38870129	-0.07619016
H	-3.02169076	-0.64808214	1.64693600
H	-3.85022832	0.34550451	0.44977008
H	-1.34538154	-1.56382621	-1.99842926
H	1.05073440	1.68107493	-1.99359314
C	-1.75658501	1.88119122	0.67468697
H	-0.83024569	2.35419577	0.40337458
H	-2.56538481	2.36229057	0.14932645
H	-1.92229515	2.13454667	1.70639782
H	1.05873574	-1.17729089	1.32744582
C	3.92993653	-0.75998326	0.79941296
H	4.17345717	-1.51936271	0.04854898
H	4.78917727	-0.09952215	0.92766863
H	3.82771429	-1.28993986	1.74399278
H	-0.88866537	-2.26380080	-0.46983847

Time = 172.00 fs
E(CASSCF/6-31G(d)) = -479.5974023

C	-0.88732220	-0.15195791	-0.37491083
C	0.53431153	0.20218431	-0.36798605
C	1.37606738	1.03677683	-1.19991325
C	1.36258480	-0.40461345	0.60457577
C	2.63449046	0.03176401	0.38704872
C	2.60962286	1.00110395	-0.69419319
N	-1.76139951	0.47100505	0.40136143
C	-2.95781310	-0.28599564	0.60305346
H	3.39604199	1.56795623	-1.16783416
C	-1.44958118	-1.44538032	-0.91747182
C	-2.90446863	-1.41738890	-0.46112242
H	-3.55268413	-1.16637387	-1.28947884
H	-3.17985369	-2.38895793	-0.08468912
H	-3.01597667	-0.66564455	1.64454051
H	-3.85294092	0.33280433	0.45202968
H	-1.33243703	-1.54108306	-2.00460699
H	1.05763125	1.67197612	-2.00237548
C	-1.75375482	1.88130182	0.68111837
H	-0.82969583	2.35594255	0.40124227
H	-2.56471341	2.36174542	0.16592849
H	-1.90972227	2.12128169	1.71614365
H	1.04531569	-1.16682180	1.32919440
C	3.92747842	-0.75555476	0.80802686
H	4.16664623	-1.52523244	0.06541085
H	4.78790969	-0.09498802	0.93590786
H	3.82771682	-1.27697102	1.75864139
H	-0.88936243	-2.26046257	-0.48293258

Time = 172.50 fs
E(CASSCF/6-31G(d)) = -479.5951642

C	-0.88441323	-0.15318297	-0.37313154
C	0.53271754	0.20232133	-0.36839997
C	1.37780382	1.03505948	-1.20644119
C	1.36343163	-0.40224679	0.60422959
C	2.63120547	0.03128051	0.38690335
C	2.60877409	0.99552617	-0.70368389
N	-1.76390290	0.46945771	0.40424899
C	-2.95586634	-0.28636356	0.60572180
H	3.39397689	1.55727684	-1.18022360
C	-1.45065358	-1.44144637	-0.92183469
C	-2.90330029	-1.41677605	-0.46406267
H	-3.55460394	-1.16209930	-1.28960817
H	-3.18121873	-2.38914046	-0.09270791

H	-3.00966918	-0.68260203	1.64044539
H	-3.85469463	0.31970406	0.45458006
H	-1.31928165	-1.51743166	-2.00955105
H	1.06400548	1.66286111	-2.01136922
C	-1.75086299	1.88155997	0.68765190
H	-0.82869430	2.35691120	0.39854389
H	-2.56457071	2.36136131	0.18137142
H	-1.89727570	2.10694174	1.72720481
H	1.03259389	-1.15516013	1.32964959
C	3.92486004	-0.75092345	0.81669752
H	4.15970926	-1.53014295	0.08245670
H	4.78639254	-0.09073608	0.94350024
H	3.82701321	-1.26383987	1.77323468
H	-0.88986256	-2.25779181	-0.49592861

Time = 173.00 fs
E(CASSCF/6-31G(d)) = -479.5931939

C	-0.88117849	-0.15432535	-0.37129073
C	0.53089015	0.20250779	-0.36894826
C	1.37890887	1.03347960	-1.21285750
C	1.36407472	-0.40024142	0.60378734
C	2.62863900	0.03072764	0.38676559
C	2.60832568	0.98981546	-0.71310888
N	-1.76644413	0.46800687	0.40688081
C	-2.95428291	-0.28692769	0.60841045
H	3.39305744	1.54563556	-1.19161304
C	-1.45162652	-1.43748481	-0.92606657
C	-2.90213198	-1.41614375	-0.46696859
H	-3.55676302	-1.15791633	-1.28970031
H	-3.18348046	-2.38927823	-0.10020006
H	-3.00280989	-0.69879278	1.63477367
H	-3.85566108	0.30649128	0.45749190
H	-1.30613318	-1.49302932	-2.01321773
H	1.06972351	1.65392727	-2.02082528
C	-1.74789918	1.88193439	0.69429581
H	-0.82732455	2.35703761	0.39536422
H	-2.56513788	2.36125311	0.19563018
H	-1.88493213	2.09170565	1.73952323
H	1.02070398	-1.14244464	1.32896029
C	3.92207984	-0.74609740	0.82543187
H	4.15273066	-1.53404665	0.09968131
H	4.78459040	-0.08678315	0.95034193
H	3.82554356	-1.25052180	1.78771518
H	-0.89008339	-2.25595221	-0.50877145

Time = 173.50 fs

E(CASSCF/6-31G(d)) = -479.5915472

C	-0.87767989	-0.15537046	-0.36935988
C	0.52886385	0.20276352	-0.36964458
C	1.37935933	1.03200805	-1.21913854
C	1.36447154	-0.39858593	0.60323444
C	2.62682380	0.03012635	0.38662099
C	2.60828596	0.98395282	-0.72243407
N	-1.76897377	0.46666920	0.40923367
C	-2.95305308	-0.28774606	0.61109618
H	3.39345627	1.53314510	-1.20208323
C	-1.45247660	-1.43348566	-0.93017454
C	-2.90097555	-1.41548042	-0.46985012
H	-3.55910461	-1.15382793	-1.28974674
H	-3.18657156	-2.38937664	-0.10713747
H	-2.99545318	-0.71410792	1.62775521
H	-3.85608277	0.29348016	0.46082054
H	-1.29320682	-1.46804809	-2.01562374
H	1.07466532	1.64534525	-2.03096377
C	-1.74486081	1.88239492	0.70105894
H	-0.82567996	2.35627440	0.39180614
H	-2.56650702	2.36150211	0.20875486
H	-1.87265666	2.07575693	1.75293139
H	1.00972641	-1.12890640	1.32736448
C	3.91913603	-0.74108509	0.83423767
H	4.14579501	-1.53693195	0.11705906
H	4.78249617	-0.08312325	0.95635064
H	3.82325780	-1.23699073	1.80202388
H	-0.88997724	-2.25505892	-0.52143620

Time = 174.00 fs
E(CASSCF/6-31G(d)) = -479.5902430

C	-0.87398288	-0.15630733	-0.36731364
C	0.52667748	0.20310574	-0.37049843
C	1.37914459	1.03062197	-1.22526268
C	1.36459257	-0.39725943	0.60254776
C	2.62577842	0.02949492	0.38645516
C	2.60865333	0.97792041	-0.73162914
N	-1.77144669	0.46546300	0.41128804
C	-2.95215842	-0.28887059	0.61374896
H	3.39533147	1.51992512	-1.21173292
C	-1.45317750	-1.42944403	-0.93415854
C	-2.89984512	-1.41477679	-0.47271617
H	-3.56156408	-1.14983749	-1.28973809
H	-3.19040422	-2.38941632	-0.11351098
H	-2.98766380	-0.72849373	1.61971579

H	-3.85625324	0.28099466	0.46460810
H	-1.28071106	-1.44267086	-2.01684467
H	1.07873407	1.63723979	-2.04194778
C	-1.74175394	1.88291287	0.70795014
H	-0.82385588	2.35459439	0.38798625
H	-2.56867131	2.36214936	0.22086938
H	-1.86040773	2.05928428	1.76716371
H	0.99968635	-1.11485575	1.32518006
C	3.91602673	-0.73589507	0.84312288
H	4.13898554	-1.53882382	0.13454507
H	4.78013078	-0.07972788	0.96146721
H	3.82011628	-1.22322145	1.81610497
H	-0.88953702	-2.25516684	-0.53393133

Time = 174.50 fs
E(CASSCF/6-31G(d)) = -479.5892635

C	-0.87015538	-0.15712862	-0.36513018
C	0.52437357	0.20354896	-0.37151518
C	1.37826640	1.02930592	-1.23121216
C	1.36442220	-0.39623296	0.60169688
C	2.62550626	0.02884857	0.38625404
C	2.60941735	0.97170274	-0.74066868
N	-1.77382329	0.46440780	0.41302851
C	-2.95157373	-0.29034501	0.61633300
H	3.39881000	1.50609048	-1.22066879
C	-1.45370045	-1.42536086	-0.93801172
C	-2.89875660	-1.41402587	-0.47557429
H	-3.56407248	-1.14594788	-1.28966745
H	-3.19487196	-2.38935536	-0.11932960
H	-2.97951418	-0.74195111	1.61106090
H	-3.85649059	0.26934881	0.46888701
H	-1.26884412	-1.41708699	-2.01701040
H	1.08186370	1.62967496	-2.05386103
C	-1.73859311	1.88346084	0.71497722
H	-0.82194260	2.35199364	0.38403004
H	-2.57152660	2.36319547	0.23216338
H	-1.84814215	2.04248303	1.78187906
H	0.99055609	-1.10066272	1.32278845
C	3.91275032	-0.73053571	0.85209508
H	4.13238227	-1.53978244	0.15207825
H	4.77754084	-0.07654793	0.96565618
H	3.81609018	-1.20919139	1.82991040
H	-0.88879812	-2.25626580	-0.54629835

Time = 175.00 fs
E(CASSCF/6-31G(d)) = -479.5885744

C	-0.86626664	-0.15783070	-0.36279164
C	0.52199774	0.20410484	-0.37269613
C	1.37673856	1.02805252	-1.23697448
C	1.36395857	-0.39547181	0.60064626
C	2.62599534	0.02819931	0.38600421
C	2.61056016	0.96528791	-0.74953325
N	-1.77607039	0.46352401	0.41444419
C	-2.95126949	-0.29220308	0.61880817
H	3.40397151	1.49173975	-1.22899513
C	-1.45401501	-1.42124294	-0.94172144
C	-2.89772707	-1.41322274	-0.47843038
H	-3.56656037	-1.14216043	-1.28953385
H	-3.19985252	-2.38913423	-0.12461849
H	-2.97108173	-0.75453110	1.60225346
H	-3.85710572	0.25882529	0.47368468
H	-1.25779100	-1.39148806	-2.01629760
H	1.08402362	1.62264602	-2.06669394
C	-1.73540025	1.88401200	0.72214592
H	-0.82001960	2.34849367	0.38006706
H	-2.57488417	2.36460631	0.24287819
H	-1.83581961	2.02555640	1.79669095
H	0.98226302	-1.08672961	1.32061067
C	3.90930599	-0.72501502	0.86116082
H	4.12605973	-1.53989957	0.16958583
H	4.77479430	-0.07351750	0.96890570
H	3.81116185	-1.19488182	1.84340361
H	-0.88783535	-2.25828254	-0.55860787

Time = 175.50 fs
E(CASSCF/6-31G(d)) = -479.5881414

C	-0.86238619	-0.15841350	-0.36028469
C	0.51959783	0.20478198	-0.37403844
C	1.37458699	1.02686236	-1.24254317
C	1.36321296	-0.39493811	0.59935828
C	2.62721848	0.02755552	0.38569321
C	2.61205788	0.95866860	-0.75821044
N	-1.77816252	0.46283233	0.41552915
C	-2.95121463	-0.29446719	0.62113214
H	3.41083420	1.47694692	-1.23680364
C	-1.45409014	-1.41710247	-0.94527083
C	-2.89677403	-1.41236415	-0.48128856
H	-3.56896059	-1.13847397	-1.28934464
H	-3.20521149	-2.38868291	-0.12941671
H	-2.96244622	-0.76632527	1.59378452
H	-3.85836822	0.24965477	0.47902805

H	-1.24772230	-1.36606467	-2.01491917
H	1.08521751	1.61608119	-2.08033893
C	-1.73220268	1.88453954	0.72945894
H	-0.81815119	2.34414200	0.37622649
H	-2.57849168	2.36632344	0.25328779
H	-1.82340683	2.00871495	1.81119990
H	0.97470283	-1.07345956	1.31907413
C	3.90569427	-0.71934049	0.87032499
H	4.12008545	-1.53929291	0.18698877
H	4.77197373	-0.07055844	0.97122659
H	3.80532494	-1.18027922	1.85656260
H	-0.88675620	-2.26108945	-0.57095251

Time = 176.00 fs
E(CASSCF/6-31G(d)) = -479.5879580

C	-0.85858254	-0.15888049	-0.35760077
C	0.51722297	0.20558578	-0.37553524
C	1.37184920	1.02574303	-1.24791753
C	1.36220766	-0.39459439	0.59779673
C	2.62913403	0.02692219	0.38530970
C	2.61388232	0.95184293	-0.76669532
N	-1.78008250	0.46235360	0.41628276
C	-2.95137918	-0.29714775	0.62326283
H	3.41934514	1.46175505	-1.24416695
C	-1.45389546	-1.41295596	-0.94864064
C	-2.89591465	-1.41144793	-0.48415101
H	-3.57121177	-1.13488404	-1.28911807
H	-3.21080697	-2.38792937	-0.13377359
H	-2.95368786	-0.77745221	1.58613857
H	-3.86047400	0.24199723	0.48494822
H	-1.23879301	-1.34100313	-2.01311207
H	1.08548054	1.60984959	-2.09459904
C	-1.72903094	1.88501567	0.73691502
H	-0.81638439	2.33901250	0.37263300
H	-2.58206002	2.36827690	0.26367971
H	-1.81087943	1.99217512	1.82502671
H	0.96775955	-1.06121661	1.31857374
C	3.90191798	-0.71351918	0.87959026
H	4.11451702	-1.53809865	0.20420882
H	4.76916875	-0.06758694	0.97265109
H	3.79858443	-1.16537512	1.86938147
H	-0.88569016	-2.26451845	-0.58343807

Time = 176.50 fs
E(CASSCF/6-31G(d)) = -479.5880436

C	-0.85492192	-0.15923870	-0.35473667
C	0.51492258	0.20651809	-0.37717561
C	1.36857412	1.02470756	-1.25310185
C	1.36097349	-0.39440689	0.59593095
C	2.63168701	0.02630086	0.38484364
C	2.61600218	0.94481494	-0.77499070
N	-1.78182224	0.46210841	0.41671015
C	-2.95173630	-0.30024337	0.62516146
H	3.42937641	1.44617507	-1.25113451
C	-1.45340259	-1.40882275	-0.95181146
C	-2.89516500	-1.41047241	-0.48701797
H	-3.57326055	-1.13138228	-1.28888458
H	-3.21649443	-2.38680831	-0.13774531
H	-2.94488633	-0.78803955	1.57975473
H	-3.86352015	0.23592905	0.49148282
H	-1.23114300	-1.31648400	-2.01112351
H	1.08487195	1.60377714	-2.10920552
C	-1.72591628	1.88541111	0.74450829
H	-0.81474812	2.33320401	0.36940349
H	-2.58529098	2.37039845	0.27433564
H	-1.79822324	1.97615577	1.83784091
H	0.96132831	-1.05028784	1.31942929
C	3.89798300	-0.70755776	0.88895659
H	4.10940015	-1.53646244	0.22117550
H	4.76646701	-0.06452017	0.97323112
H	3.79095648	-1.15016620	1.88187086
H	-0.88477678	-2.26837770	-0.59617354

Time = 177.00 fs
E(CASSCF/6-31G(d)) = -479.5884352

C	-0.85146693	-0.15949858	-0.35169507
C	0.51274539	0.20757701	-0.37894457
C	1.36482175	1.02377250	-1.25810414
C	1.35954712	-0.39434841	0.59373971
C	2.63481061	0.02568973	0.38428660
C	2.61838399	0.93759473	-0.78310702
N	-1.78338336	0.46211693	0.41682267
C	-2.95226339	-0.30374190	0.62679590
H	3.44072809	1.43018910	-1.25773262
C	-1.45258639	-1.40472343	-0.95476603
C	-2.89453926	-1.40943587	-0.48988785
H	-3.57506317	-1.12795609	-1.28868718
H	-3.22213177	-2.38526961	-0.14139166
H	-2.93612111	-0.79820407	1.57498795
H	-3.86749123	0.23143744	0.49867610
H	-1.22489828	-1.29268149	-2.00919695

H	1.08346618	1.59766623	-2.12384272
C	-1.72288848	1.88569480	0.75222795
H	-0.81325418	2.32683799	0.36664442
H	-2.58790324	2.37263355	0.28551479
H	-1.78543408	1.96087355	1.84938393
H	0.95533775	-1.04085089	1.32184599
C	3.89389906	-0.70146279	0.89842086
H	4.10476675	-1.53452973	0.23783305
H	4.76394507	-0.06128321	0.97303647
H	3.78246808	-1.13465382	1.89405717
H	-0.88415342	-2.27246929	-0.60926163

Time = 177.50 fs
E(CASSCF/6-31G(d)) = -479.5891671

C	-0.84827520	-0.15967412	-0.34848496
C	0.51073819	0.20875652	-0.38082309
C	1.36066265	1.02295563	-1.26293469
C	1.35796843	-0.39439995	0.59121447
C	2.63842793	0.02508372	0.38363188
C	2.62099270	0.93019824	-0.79106185
N	-1.78477755	0.46239865	0.41663833
C	-2.95294206	-0.30762219	0.62814390
H	3.45313815	1.41375766	-1.26396798
C	-1.45142623	-1.40067794	-0.95749158
C	-2.89404912	-1.40833603	-0.49275738
H	-3.57658641	-1.12458857	-1.28858070
H	-3.22758418	-2.38328542	-0.14477291
H	-2.92747288	-0.80803309	1.57207550
H	-3.87225975	0.22842322	0.50657592
H	-1.22017235	-1.26976414	-2.00755896
H	1.08134386	1.59131602	-2.13817635
C	-1.71997391	1.88583392	0.76005808
H	-0.81189954	2.32005524	0.36445002
H	-2.58965468	2.37495096	0.29744080
H	-1.77251672	1.94653770	1.85948591
H	0.94976738	-1.03295349	1.32588580
C	3.88968042	-0.69524093	0.90797659
H	4.10063357	-1.53243609	0.25414662
H	4.76165984	-0.05781538	0.97215293
H	3.77315665	-1.11884320	1.90598040
H	-0.88394393	-2.27660542	-0.62279002

Time = 178.00 fs
E(CASSCF/6-31G(d)) = -479.5902396

C	-0.84539806	-0.15978282	-0.34512212

C	0.50894478	0.21004635	-0.38278824
C	1.35617717	1.02227370	-1.26760486
C	1.35627874	-0.39455068	0.58836137
C	2.64245380	0.02447460	0.38287495
C	2.62379179	0.92264680	-0.79887905
N	-1.78602666	0.46297206	0.41618216
C	-2.95375695	-0.31185614	0.62919558
H	3.46629765	1.39683004	-1.26983421
C	-1.44990705	-1.39670380	-0.95998197
C	-2.89370312	-1.40716970	-0.49562192
H	-3.57780762	-1.12125879	-1.28863003
H	-3.23272849	-2.38085543	-0.14794733
H	-2.91902584	-0.81757061	1.57111417
H	-3.87760011	0.22671132	0.51522857
H	-1.21706750	-1.24789614	-2.00640826
H	1.07858357	1.58454164	-2.15188120
C	-1.71719399	1.88579422	0.76797776
H	-0.81066958	2.31301169	0.36290098
H	-2.59035973	2.37734934	0.31029235
H	-1.75948280	1.93334450	1.86807602
H	0.94465543	-1.02651001	1.33145539
C	3.88534637	-0.68889935	0.91761387
H	4.09700154	-1.53029875	0.27010710
H	4.75964163	-0.05407513	0.97068060
H	3.76306947	-1.10274243	1.91769110
H	-0.88424913	-2.28062212	-0.63682429

Time = 178.50 fs
E(CASSCF/6-31G(d)) = -479.5916080

C	-0.84287923	-0.15984555	-0.34162917
C	0.50740480	0.21143192	-0.38481355
C	1.35145415	1.02173987	-1.27212615
C	1.35451972	-0.39479617	0.58520190
C	2.64679670	0.02385125	0.38201347
C	2.62674333	0.91496628	-0.80658760
N	-1.78716230	0.46385414	0.41548634
C	-2.95469392	-0.31641069	0.62995498
H	3.47987004	1.37935622	-1.27531966
C	-1.44802049	-1.39281439	-0.96223954
C	-2.89350629	-1.40593269	-0.49847589
H	-3.57871422	-1.11794232	-1.28890720
H	-3.23745688	-2.37800984	-0.15096921
H	-2.91086944	-0.82681114	1.57205228
H	-3.88321326	0.22606741	0.52467220
H	-1.21567500	-1.22723839	-2.00590786
H	1.07525530	1.57719085	-2.16466629

C	-1.71456406	1.88554063	0.77596143
H	-0.80954221	2.30587329	0.36206365
H	-2.58990180	2.37986051	0.32419782
H	-1.74634817	1.92147197	1.87518687
H	0.94009571	-1.02131598	1.33831396
C	3.88092130	-0.68244612	0.92731932
H	4.09385605	-1.52821005	0.28573454
H	4.75788951	-0.05004316	0.96873190
H	3.75226289	-1.08636079	1.92924643
H	-0.88513956	-2.28438921	-0.65140282

Time = 179.00 fs
E(CASSCF/6-31G(d)) = -479.5931846

C	-0.84075383	-0.15988626	-0.33803491
C	0.50615247	0.21289481	-0.38686981
C	1.34658903	1.02136144	-1.27650953
C	1.35273310	-0.39513522	0.58177193
C	2.65136065	0.02320006	0.38104723
C	2.62980810	0.90718595	-0.81421981
N	-1.78822455	0.46505928	0.41458952
C	-2.95573817	-0.32124956	0.63044017
H	3.49351181	1.36129864	-1.28041623
C	-1.44576602	-1.38901742	-0.96427651
C	-2.89345986	-1.40461975	-0.50131324
H	-3.57930233	-1.11461220	-1.28948736
H	-3.24167962	-2.37480963	-0.15388747
H	-2.90309875	-0.83570178	1.57469793
H	-3.88875710	0.22621793	0.53492995
H	-1.21607346	-1.20794887	-2.00618014
H	1.07141590	1.56915561	-2.17629547
C	-1.71209252	1.88503835	0.78397968
H	-0.80849176	2.29880971	0.36198983
H	-2.58824067	2.38254876	0.33923265
H	-1.73313041	1.91107460	1.88095223
H	0.93622414	-1.01707648	1.34609989
C	3.87643443	-0.67589059	0.93707646
H	4.09116852	-1.52623368	0.30107844
H	4.75637023	-0.04572293	0.96643038
H	3.74080198	-1.06970798	1.94070589
H	-0.88664991	-2.28781510	-0.66653292

Time = 179.50 fs
E(CASSCF/6-31G(d)) = -479.5948556

C	-0.83904755	-0.15993100	-0.33437273
C	0.50521558	0.21441377	-0.38892643

C	1.34168104	1.02113772	-1.28076485
C	1.35096067	-0.39556640	0.57811951
C	2.65604725	0.02250610	0.37997745
C	2.63294595	0.89933680	-0.82180902
N	-1.78925975	0.46659788	0.41353522
C	-2.95687288	-0.32633446	0.63068195
H	3.50689311	1.34264275	-1.28512722
C	-1.44315211	-1.38531394	-0.96611538
C	-2.89356123	-1.40322493	-0.50412802
H	-3.57957494	-1.11124027	-1.29044392
H	-3.24532685	-2.37134412	-0.15674497
H	-2.89581208	-0.84415201	1.57874152
H	-3.89387786	0.22687079	0.54600441
H	-1.21832527	-1.19018016	-2.00730463
H	1.06710621	1.56038124	-2.18660378
C	-1.70978030	1.88425414	0.79200053
H	-0.80749277	2.29198723	0.36271651
H	-2.58541512	2.38550665	0.35542000
H	-1.71984590	1.90227797	1.88559865
H	0.93319903	-1.01344417	1.35437051
C	3.87191912	-0.66924381	0.94686606
H	4.08889902	-1.52440423	0.31621698
H	4.75502025	-0.04113897	0.96390887
H	3.72876004	-1.05279239	1.95212695
H	-0.88877582	-2.29084775	-0.68218974

Time = 180.00 fs
E(CASSCF/6-31G(d)) = -479.5965075

C	-0.83777641	-0.16000643	-0.33067806
C	0.50461448	0.21596608	-0.39095311
C	1.33682986	1.02105878	-1.28489994
C	1.34924436	-0.39608491	0.57430139
C	2.66075763	0.02175435	0.37880567
C	2.63611637	0.89144958	-0.82938697
N	-1.79031746	0.46847461	0.41236916
C	-2.95807872	-0.33162596	0.63072143
H	3.51971627	1.32340482	-1.28947477
C	-1.44019718	-1.38169814	-0.96778831
C	-2.89380419	-1.40174196	-0.50691493
H	-3.57954001	-1.10779862	-1.29184360
H	-3.24834951	-2.36772628	-0.15957839
H	-2.88910555	-0.85204798	1.58378880
H	-3.89823944	0.22773498	0.55787341
H	-1.22247081	-1.17407437	-2.00931801
H	1.06235001	1.55086961	-2.19550752
C	-1.70762060	1.88315800	0.79999098

H	-0.80652269	2.28556128	0.36426602
H	-2.58154028	2.38884784	0.37273341
H	-1.70650793	1.89517367	1.88943038
H	0.93117897	-1.01005803	1.36264768
C	3.86741176	-0.66251870	0.95666694
H	4.08699989	-1.52272950	0.33125333
H	4.75375116	-0.03633284	0.96130829
H	3.71621880	-1.03562048	1.96356077
H	-0.89147177	-2.29347203	-0.69831669

Time = 180.50 fs
E(CASSCF/6-31G(d)) = -479.5980505

C	-0.83694653	-0.16013798	-0.32698552
C	0.50436143	0.21752918	-0.39292157
C	1.33213214	1.02110522	-1.28891956
C	1.34762611	-0.39668095	0.57037863
C	2.66539451	0.02093154	0.37753214
C	2.63927931	0.88355261	-0.83698099
N	-1.79144726	0.47068706	0.41113596
C	-2.95933398	-0.33708404	0.63060676
H	3.53173065	1.30363600	-1.29350521
C	-1.43693004	-1.37815798	-0.96933533
C	-2.89417931	-1.40016484	-0.50966963
H	-3.57920777	-1.10426171	-1.29374121
H	-3.25071878	-2.36408565	-0.16241897
H	-2.88306637	-0.85926881	1.58939719
H	-3.90154740	0.22853681	0.57048792
H	-1.22852317	-1.15975673	-2.01221394
H	1.05715399	1.54067918	-2.20301001
C	-1.70559917	1.88172488	0.80791900
H	-0.80556393	2.27966975	0.36664624
H	-2.57679945	2.39269787	0.39110178
H	-1.69312545	1.88981484	1.89280774
H	0.93030251	-1.00657881	1.37046225
C	3.86295033	-0.65573008	0.96645700
H	4.08541914	-1.52119602	0.34631029
H	4.75245881	-0.03135738	0.95877576
H	3.70326808	-1.01819622	1.97504861
H	-0.89465252	-2.29570508	-0.71482871

Time = 181.00 fs
E(CASSCF/6-31G(d)) = -479.5994337

C	-0.83655435	-0.16034782	-0.32332626
C	0.50446012	0.21908201	-0.39480696
C	1.32767837	1.02124934	-1.29282419

C	1.34614789	-0.39733949	0.56641214
C	2.66986397	0.02002739	0.37615441
C	2.64239579	0.87567005	-0.84461188
N	-1.79269592	0.47322506	0.40987607
C	-2.96061522	-0.34266836	0.63038965
H	3.54274478	1.28342265	-1.29729320
C	-1.43338962	-1.37467674	-0.97080174
C	-2.89467455	-1.39848841	-0.51238898
H	-3.57858848	-1.10060818	-1.29617541
H	-3.25242488	-2.36056007	-0.16529397
H	-2.87776480	-0.86570246	1.59511315
H	-3.90356868	0.22903330	0.58377206
H	-1.23646325	-1.14732768	-2.01594498
H	1.05150909	1.52992066	-2.20920170
C	-1.70369523	1.87993613	0.81575543
H	-0.80460504	2.27442796	0.36985089
H	-2.57143050	2.39718450	0.41041694
H	-1.67970261	1.88621265	1.89612004
H	0.93067173	-1.00271689	1.37739302
C	3.85857298	-0.64889439	0.97621440
H	4.08410368	-1.51977543	0.36152381
H	4.75103268	-0.02627012	0.95646241
H	3.69000581	-1.00052040	1.98661850
H	-0.89819688	-2.29759084	-0.73161781

Time = 181.50 fs
E(CASSCF/6-31G(d)) = -479.6006400

C	-0.83658701	-0.16065358	-0.31972679
C	0.50490591	0.22060567	-0.39658839
C	1.32355083	1.02145701	-1.29660949
C	1.34485148	-0.39804067	0.56245873
C	2.67407727	0.01903525	0.37466665
C	2.64542826	0.86782099	-0.85229289
N	-1.79410577	0.47607098	0.40862397
C	-2.96189772	-0.34833848	0.63012218
H	3.55263293	1.26288383	-1.30094294
C	-1.42962390	-1.37123487	-0.97223559
C	-2.89527592	-1.39670895	-0.51507103
H	-3.57769017	-1.09682262	-1.29916548
H	-3.25347474	-2.35728682	-0.16822876
H	-2.87324840	-0.87126030	1.60050495
H	-3.90414613	0.22902237	0.59762454
H	-1.24623753	-1.13685806	-2.02042657
H	1.04539333	1.51874877	-2.21425331
C	-1.70188298	1.87778052	0.82347588
H	-0.80364121	2.26992617	0.37385993

H	-2.56570690	2.40242809	0.43054325
H	-1.66623930	1.88433550	1.89975325
H	0.93234089	-0.99825141	1.38309638
C	3.85431636	-0.64202925	0.98591879
H	4.08300198	-1.51843173	0.37703520
H	4.74936637	-0.02112642	0.95452048
H	3.67653714	-0.98259055	1.99828219
H	-0.90195481	-2.29919382	-0.74855902

Time = 182.00 fs
E(CASSCF/6-31G(d)) = -479.6016810

C	-0.83702307	-0.16106756	-0.31620886
C	0.50568611	0.22208367	-0.39824909
C	1.31982299	1.02168972	-1.30026657
C	1.34377901	-0.39876022	0.55856825
C	2.67795187	0.01795178	0.37305989
C	2.64834038	0.86001960	-0.86003006
N	-1.79571385	0.47920057	0.40740774
C	-2.96315574	-0.35405394	0.62985409
H	3.56133722	1.24216628	-1.30458637
C	-1.42568838	-1.36781202	-0.97368564
C	-2.89596813	-1.39482470	-0.51771493
H	-3.57651712	-1.09289690	-1.30270936
H	-3.25388903	-2.35439366	-0.17124938
H	-2.86953806	-0.87588902	1.60518968
H	-3.90320812	0.22835038	0.61192165
H	-1.25775757	-1.12838604	-2.02554387
H	1.03877559	1.50735040	-2.21840102
C	-1.70013381	1.87525509	0.83106248
H	-0.80267441	2.26622882	0.37863979
H	-2.55991433	2.40853091	0.45132922
H	-1.65273249	1.88411025	1.90405552
H	0.93530915	-0.99304129	1.38732671
C	3.85021417	-0.63515291	0.99555232
H	4.08206640	-1.51712876	0.39298341
H	4.74736760	-0.01597267	0.95309992
H	3.66297325	-0.96440098	2.01003343
H	-0.90575694	-2.30059107	-0.76551680

Time = 182.50 fs
E(CASSCF/6-31G(d)) = -479.6025816

C	-0.83783350	-0.16159663	-0.31279021
C	0.50678117	0.22350189	-0.39977598
C	1.31655810	1.02190644	-1.30378365
C	1.34297236	-0.39946968	0.55478213

C	2.68141311	0.01677634	0.37132254
C	2.65109755	0.85227540	-0.86782265
N	-1.79755149	0.48258409	0.40624939
C	-2.96436268	-0.35977465	0.62963069
H	3.56886483	1.22143915	-1.30837913
C	-1.42164430	-1.36438869	-0.97519997
C	-2.89673523	-1.39283629	-0.52032073
H	-3.57506929	-1.08883068	-1.30678281
H	-3.25369878	-2.35199017	-0.17438497
H	-2.86662576	-0.87957911	1.60885408
H	-3.90077395	0.22691748	0.62652151
H	-1.27090090	-1.12191618	-2.03116056
H	1.03162193	1.49593125	-2.22192591
C	-1.69841877	1.87236569	0.83850551
H	-0.80171288	2.26337492	0.38414357
H	-2.55432436	2.41556487	0.47262046
H	-1.63917856	1.88542479	1.90930289
H	0.93951858	-0.98703072	1.38994977
C	3.84629568	-0.62828367	1.00510036
H	4.08125567	-1.51583669	0.40949803
H	4.74496612	-0.01084090	0.95234437
H	3.64942909	-0.94594300	2.02184707
H	-0.90942558	-2.30186184	-0.78235047

Time = 183.00 fs
E(CASSCF/6-31G(d)) = -479.6033636

C	-0.83898295	-0.16224236	-0.30948572
C	0.50816570	0.22484871	-0.40115956
C	1.31380869	1.02206502	-1.30714845
C	1.34247337	-0.40013608	0.55113327
C	2.68439503	0.01551003	0.36944143
C	2.65366714	0.84459413	-0.87566441
N	-1.79964392	0.48618703	0.40516584
C	-2.96549128	-0.36546124	0.62949137
H	3.57528052	1.20088724	-1.31249352
C	-1.41755691	-1.36094773	-0.97682520
C	-2.89756110	-1.39074712	-0.52288927
H	-3.57334260	-1.08463131	-1.31133984
H	-3.25294193	-2.35016000	-0.17766996
H	-2.86447382	-0.88237009	1.61126907
H	-3.89695444	0.22468057	0.64126908
H	-1.28551284	-1.11741951	-2.03712903
H	1.02390299	1.48469995	-2.22512625
C	-1.69671093	1.86912774	0.84580464
H	-0.80077045	2.26137879	0.39031126
H	-2.54916814	2.42355782	0.49427203

H	-1.62557646	1.88813144	1.91567094
H	0.94485473	-0.98024904	1.39094934
C	3.84258450	-0.62143934	1.01455184
H	4.08053712	-1.51453773	0.42669347
H	4.74212006	-0.00574422	0.95238707
H	3.63602103	-0.92720558	2.03368034
H	-0.91278595	-2.30307507	-0.79891866

Time = 183.50 fs
E(CASSCF/6-31G(d)) = -479.6040262

C	-0.84043155	-0.16300134	-0.30630877
C	0.50980921	0.22611557	-0.40239438
C	1.31161622	1.02212259	-1.31035120
C	1.34232408	-0.40072107	0.54764715
C	2.68684084	0.01415457	0.36740307
C	2.65601887	0.83697914	-0.88354473
N	-1.80200960	0.48997003	0.40417048
C	-2.96651384	-0.37107525	0.62946854
H	3.58069476	1.18070380	-1.31710918
C	-1.41349415	-1.35747535	-0.97860646
C	-2.89842977	-1.38856370	-0.52542199
H	-3.57133013	-1.08031336	-1.31631444
H	-3.25166011	-2.34895470	-0.18114512
H	-2.86301600	-0.88435249	1.61229912
H	-3.89194751	0.22165417	0.65600114
H	-1.30140877	-1.11483524	-2.04330144
H	1.01560295	1.47385054	-2.22828610
C	-1.69498749	1.86556706	0.85296946
H	-0.79986569	2.26023042	0.39707077
H	-2.54461374	2.43247885	0.51615806
H	-1.61193157	1.89205117	1.92321608
H	0.95115026	-0.97280576	1.39042968
C	3.83909784	-0.61463708	1.02389902
H	4.07988917	-1.51322994	0.44466416
H	4.73881996	-0.00067395	0.95334695
H	3.62286481	-0.90817727	2.04547596
H	-0.91567638	-2.30427547	-0.81508175

Time = 184.00 fs
E(CASSCF/6-31G(d)) = -479.6045418

C	-0.84213736	-0.16386535	-0.30327350
C	0.51167796	0.22729811	-0.40347951
C	1.31000844	1.02203447	-1.31338898
C	1.34256621	-0.40117862	0.54434555
C	2.68870384	0.01271139	0.36519434

C	2.65812672	0.82943145	-0.89144917
N	-1.80465825	0.49388785	0.40327480
C	-2.96740317	-0.37657998	0.62958713
H	3.58524802	1.16108730	-1.32240054
C	-1.40952573	-1.35396106	-0.98058825
C	-2.89932570	-1.38629564	-0.52792091
H	-3.56902440	-1.07589688	-1.32162286
H	-3.24989611	-2.34838956	-0.18485829
H	-2.86215824	-0.88566642	1.61190677
H	-3.88602893	0.21790811	0.67055254
H	-1.31837634	-1.11407153	-2.04954055
H	1.00672896	1.46354929	-2.23164236
C	-1.69323113	1.86172054	0.86001982
H	-0.79901996	2.25989529	0.40433731
H	-2.54075182	2.44222594	0.53817922
H	-1.59825760	1.89697686	1.93186972
H	0.95819173	-0.96488323	1.38861102
C	3.83584565	-0.60789322	1.03313688
H	4.07930520	-1.51193072	0.46348398
H	4.73508918	0.00440162	0.95532404
H	3.61007185	-0.88884575	2.05716659
H	-0.91795661	-2.30546941	-0.83070328

Time = 184.50 fs
E(CASSCF/6-31G(d)) = -479.6048450

C	-0.84406108	-0.16481991	-0.30039806
C	0.51373636	0.22840028	-0.40442185
C	1.30899489	1.02174793	-1.31627103
C	1.34324092	-0.40144876	0.54125500
C	2.68994721	0.01118140	0.36280192
C	2.65997178	0.82194930	-0.89935766
N	-1.80758436	0.49788338	0.40249109
C	-2.96813464	-0.38194150	0.62986489
H	3.58909234	1.14224502	-1.32851932
C	-1.40572458	-1.35039570	-0.98281770
C	-2.90023374	-1.38395571	-0.53038917
H	-3.56642063	-1.07140549	-1.32716568
H	-3.24769192	-2.34844078	-0.18886321
H	-2.86178034	-0.88649376	1.61015100
H	-3.87953531	0.21356305	0.68476094
H	-1.33617420	-1.11500626	-2.05573098
H	0.99732059	1.45392367	-2.23535696
C	-1.69142987	1.85763765	0.86698589
H	-0.79825478	2.26030937	0.41201309
H	-2.53759502	2.45261078	0.56026427
H	-1.58457779	1.90267658	1.94144307

H	0.96572916	-0.95672246	1.38582318
C	3.83283036	-0.60122458	1.04226136
H	4.07880093	-1.51067955	0.48320821
H	4.73098166	0.00953781	0.95839558
H	3.59774708	-0.86919901	2.06868178
H	-0.91951068	-2.30660706	-0.84564550

Time = 185.00 fs
E(CASSCF/6-31G(d)) = -479.6048518

C	-0.84617883	-0.16582810	-0.29770760
C	0.51594700	0.22945113	-0.40524939
C	1.30854620	1.02117973	-1.31902709
C	1.34438463	-0.40143927	0.53843007
C	2.69054410	0.00957186	0.36019890
C	2.66155320	0.81451763	-0.90722613
N	-1.81073644	0.50185879	0.40183105
C	-2.96869585	-0.38713342	0.63031388
H	3.59237273	1.12442777	-1.33556256
C	-1.40217715	-1.34676642	-0.98535148
C	-2.90113943	-1.38156034	-0.53283327
H	-3.56352226	-1.06686369	-1.33282488
H	-3.24508693	-2.34903861	-0.19321577
H	-2.86172748	-0.88702244	1.60717107
H	-3.87283032	0.20878444	0.69846960
H	-1.35450527	-1.11746338	-2.06177731
H	0.98745632	1.44505802	-2.23950456
C	-1.68957250	1.85338868	0.87391206
H	-0.79758466	2.26135039	0.41998354
H	-2.53510148	2.46331383	0.58236499
H	-1.57092519	1.90888309	1.95162501
H	0.97348992	-0.94860289	1.38251282
C	3.83004635	-0.59465381	1.05126502
H	4.07843993	-1.50954801	0.50389066
H	4.72657521	0.01481631	0.96261179
H	3.58599042	-0.84922403	2.07996281
H	-0.92023595	-2.30753620	-0.85975466

Time = 185.50 fs
E(CASSCF/6-31G(d)) = -479.6044946

C	-0.84841632	-0.16650758	-0.29481698
C	0.51817101	0.23051471	-0.40603617
C	1.30851870	1.02051906	-1.32150267
C	1.34586494	-0.40145909	0.53551592
C	2.69057679	0.00809929	0.35702738
C	2.66296592	0.80686442	-0.91485617

N	-1.81394085	0.50566811	0.40087329
C	-2.96920130	-0.39223485	0.63087137
H	3.59526451	1.10724560	-1.34398288
C	-1.39891315	-1.34326298	-0.98796843
C	-2.90206149	-1.37912249	-0.53526716
H	-3.56031122	-1.06225624	-1.33856457
H	-3.24210057	-2.35003151	-0.19799299
H	-2.86148208	-0.88709172	1.60308150
H	-3.86619239	0.20360367	0.71168866
H	-1.37287988	-1.12050540	-2.06675511
H	0.97706835	1.43668325	-2.24426771
C	-1.68757573	1.84911432	0.88094170
H	-0.79711652	2.26273267	0.42838212
H	-2.53289017	2.47387172	0.60453957
H	-1.55744843	1.91486203	1.96207870
H	0.98132588	-0.94087960	1.37932708
C	3.82744515	-0.58808061	1.06028987
H	4.07817509	-1.50863382	0.52586027
H	4.72201857	0.02037645	0.96816925
H	3.57485224	-0.82875532	2.09123393
H	-0.92003809	-2.30797657	-0.87333777

Time = 186.00 fs
E(CASSCF/6-31G(d)) = -479.6037885

C	-0.85066050	-0.16701975	-0.29187552
C	0.52038292	0.23148737	-0.40669745
C	1.30903422	1.01979311	-1.32372773
C	1.34776061	-0.40145458	0.53254458
C	2.69002169	0.00666837	0.35348278
C	2.66411766	0.79919232	-0.92232488
N	-1.81736498	0.50940473	0.39976823
C	-2.96954299	-0.39711408	0.63153101
H	3.59778222	1.09089588	-1.35377510
C	-1.39597077	-1.33989693	-0.99071236
C	-2.90296828	-1.37667937	-0.53765535
H	-3.55682662	-1.05765764	-1.34424976
H	-3.23881357	-2.35137690	-0.20322070
H	-2.86103994	-0.88728968	1.59836532
H	-3.86021577	0.19828401	0.72414698
H	-1.39110054	-1.12427653	-2.07088273
H	0.96646534	1.42890930	-2.24935604
C	-1.68550648	1.84485060	0.88799863
H	-0.79688219	2.26456990	0.43698305
H	-2.53077632	2.48415545	0.62655685
H	-1.54429158	1.92048864	1.97251410
H	0.98887243	-0.93390241	1.37653294

C	3.82504656	-0.58156799	1.06924087
H	4.07793911	-1.50794830	0.54874724
H	4.71747095	0.02622120	0.97499489
H	3.56447393	-0.80801797	2.10220795
H	-0.91897877	-2.30806004	-0.88612521

Time = 186.50 fs
E(CASSCF/6-31G(d)) = -479.6026884

C	-0.85298564	-0.16707108	-0.28904379
C	0.52248432	0.23260540	-0.40740033
C	1.30973540	1.01865956	-1.32578880
C	1.34997809	-0.40110332	0.52989862
C	2.68892210	0.00540817	0.34941785
C	2.66514867	0.79150323	-0.92916283
N	-1.82046103	0.51249480	0.39853767
C	-2.96982870	-0.40170490	0.63228076
H	3.59991101	1.07646524	-1.36438805
C	-1.39363160	-1.33668385	-0.99364365
C	-2.90383188	-1.37431780	-0.53998296
H	-3.55321702	-1.05317854	-1.34954509
H	-3.23533463	-2.35279637	-0.20880092
H	-2.86008031	-0.88747455	1.59335354
H	-3.85521141	0.19317571	0.73543808
H	-1.40806358	-1.12811869	-2.07395222
H	0.95608347	1.42185225	-2.25456672
C	-1.68335282	1.84089923	0.89504979
H	-0.79679660	2.26626707	0.44539896
H	-2.52888497	2.49292019	0.64787078
H	-1.53182412	1.92513822	1.98196270
H	0.99576243	-0.92796749	1.37501496
C	3.82286881	-0.57534338	1.07787117
H	4.07814384	-1.50774179	0.57226111
H	4.71311127	0.03238921	0.98287843
H	3.55518411	-0.78743063	2.11273556
H	-0.91690304	-2.30702773	-0.89751643

Time = 187.00 fs
E(CASSCF/6-31G(d)) = -479.6015497

C	-0.85538717	-0.16662311	-0.28626767
C	0.52447596	0.23389627	-0.40815109
C	1.31059364	1.01711437	-1.32775288
C	1.35254156	-0.40041514	0.52748823
C	2.68730923	0.00429123	0.34472710
C	2.66610526	0.78362417	-0.93550983
N	-1.82328870	0.51497543	0.39714037

C	-2.97006133	-0.40608173	0.63310318
H	3.60165191	1.06360783	-1.37602457
C	-1.39187407	-1.33358609	-0.99680545
C	-2.90466142	-1.37200735	-0.54229897
H	-3.54945028	-1.04873064	-1.35449404
H	-3.23164602	-2.35421404	-0.21489177
H	-2.85840447	-0.88795670	1.58838311
H	-3.85139954	0.18830819	0.74573452
H	-1.42390391	-1.13187067	-2.07604276
H	0.94591670	1.41522524	-2.25974982
C	-1.68107364	1.83723843	0.90228236
H	-0.79686158	2.26778379	0.45371636
H	-2.52696731	2.50007774	0.66890250
H	-1.51985392	1.92861758	1.99038548
H	1.00200726	-0.92307806	1.37500617
C	3.82083691	-0.56925670	1.08638214
H	4.07880182	-1.50805654	0.59686749
H	4.70896187	0.03908380	0.99195121
H	3.54678862	-0.76654821	2.12302054
H	-0.91380705	-2.30477276	-0.90771782

Time = 187.50 fs
E(CASSCF/6-31G(d)) = -479.6005181

C	-0.85781244	-0.16565301	-0.28357101
C	0.52631702	0.23536019	-0.40893565
C	1.31156984	1.01517146	-1.32964831
C	1.35542096	-0.39940324	0.52530353
C	2.68524449	0.00330765	0.33940898
C	2.66701079	0.77556338	-0.94136586
N	-1.82584671	0.51682601	0.39557795
C	-2.97023704	-0.41021263	0.63396464
H	3.60289695	1.05228639	-1.38860261
C	-1.39072579	-1.33062677	-1.00017851
C	-2.90544548	-1.36976945	-0.54460005
H	-3.54558809	-1.04431787	-1.35903714
H	-3.22781236	-2.35554080	-0.22152004
H	-2.85585634	-0.88904718	1.58390524
H	-3.84904172	0.18378817	0.75496297
H	-1.43840719	-1.13531684	-2.07725344
H	0.93620783	1.40890258	-2.26460900
C	-1.67867726	1.83392117	0.90971819
H	-0.79706513	2.26906748	0.46182788
H	-2.52486052	2.50538691	0.68958784
H	-1.50845104	1.93066167	1.99762441
H	1.00753303	-0.91927614	1.37662500
C	3.81892531	-0.56329550	1.09477831

H	4.07992198	-1.50892239	0.62250296
H	4.70512036	0.04635446	1.00215587
H	3.53928244	-0.74538377	2.13303093
H	-0.90973424	-2.30124061	-0.91667017

Time = 188.00 fs
E(CASSCF/6-31G(d)) = -479.5997347

C	-0.86021246	-0.16415190	-0.28098511
C	0.52797131	0.23699448	-0.40973673
C	1.31262682	1.01284323	-1.33150613
C	1.35857387	-0.39808540	0.52334946
C	2.68280146	0.00244053	0.33347542
C	2.66788966	0.76733633	-0.94674336
N	-1.82813895	0.51803950	0.39386154
C	-2.97035610	-0.41406652	0.63483403
H	3.60349600	1.04243415	-1.40199070
C	-1.39020578	-1.32781949	-1.00373955
C	-2.90617211	-1.36762403	-0.54688297
H	-3.54170627	-1.03993574	-1.36313430
H	-3.22390617	-2.35670879	-0.22869795
H	-2.85230934	-0.89102460	1.58032692
H	-3.84829498	0.17966794	0.76306555
H	-1.45139266	-1.13825932	-2.07776129
H	0.92719974	1.40276759	-2.26885325
C	-1.67617238	1.83098477	0.91736962
H	-0.79738015	2.27008734	0.46962558
H	-2.52245653	2.50867144	0.70985395
H	-1.49766349	1.93104308	2.00362723
H	1.01234553	-0.91649756	1.37981823
C	3.81710866	-0.55744448	1.10306168
H	4.08150791	-1.51034088	0.64909565
H	4.70165377	0.05422348	1.01341254
H	3.53263342	-0.72395234	2.14274230
H	-0.90473527	-2.29641889	-0.92432125

Time = 188.50 fs
E(CASSCF/6-31G(d)) = -479.5992927

C	-0.86254262	-0.16212178	-0.27854593
C	0.52940699	0.23879366	-0.41053469
C	1.31373072	1.01014062	-1.33335626
C	1.36194540	-0.39648509	0.52164350
C	2.68006437	0.00166801	0.32694989
C	2.66876525	0.75896430	-0.95166514
N	-1.83017220	0.51862080	0.39200895
C	-2.97042399	-0.41761533	0.63568660

H	3.60327660	1.03395993	-1.41601862
C	-1.39032469	-1.32516912	-1.00746002
C	-2.90682907	-1.36558916	-0.54914475
H	-3.53789228	-1.03557184	-1.36676448
H	-3.22000743	-2.35767475	-0.23642162
H	-2.84767352	-0.89410427	1.57796075
H	-3.84919923	0.17593660	0.76999848
H	-1.46271184	-1.14051659	-2.07780503
H	0.91912013	1.39672380	-2.27222846
C	-1.67356694	1.82845043	0.92523901
H	-0.79776744	2.27083192	0.47700514
H	-2.51971103	2.50982818	0.72962012
H	-1.48751503	1.92957302	2.00844809
H	1.01652485	-0.91458254	1.38437197
C	3.81536332	-0.55168620	1.11123171
H	4.08355591	-1.51227942	0.67656956
H	4.69859311	0.06268155	1.02562144
H	3.52678331	-0.70227142	2.15213960
H	-0.89886552	-2.29033772	-0.93062890

Time = 189.00 fs
E(CASSCF/6-31G(d)) = -479.5992194

C	-0.86476337	-0.15957390	-0.27629284
C	0.53059671	0.24074956	-0.41130818
C	1.31485258	1.00707258	-1.33522494
C	1.36547000	-0.39463019	0.52021272
C	2.67712593	0.00096410	0.31986656
C	2.66965760	0.75047367	-0.95616325
N	-1.83195509	0.51858585	0.39004342
C	-2.97045106	-0.42083614	0.63650749
H	3.60206567	1.02675460	-1.43048917
C	-1.39108500	-1.32267220	-1.01130665
C	-2.90740409	-1.36368062	-0.55138299
H	-3.53424168	-1.03120682	-1.36992397
H	-3.21620248	-2.35842181	-0.24467064
H	-2.84190329	-0.89841328	1.57698252
H	-3.85167703	0.17251992	0.77573180
H	-1.47224838	-1.14192595	-2.07766741
H	0.91216833	1.39070346	-2.27454235
C	-1.67086732	1.82632292	0.93331851
H	-0.79817993	2.27130683	0.48387016
H	-2.51664843	2.50883263	0.74880080
H	-1.47800329	1.92610312	2.01224341
H	1.02021122	-0.91329737	1.38994325
C	3.81366856	-0.54600189	1.11928531
H	4.08605335	-1.51466722	0.70484747

H	4.69593116	0.07168567	1.03866519
H	3.52164913	-0.68036094	2.16121722
H	-0.89218429	-2.28306774	-0.93556330

Time = 189.50 fs
E(CASSCF/6-31G(d)) = -479.5994685

C	-0.86684051	-0.15652826	-0.27426797
C	0.53151791	0.24285204	-0.41203486
C	1.31596923	1.00364532	-1.33713292
C	1.36907466	-0.39255054	0.51909039
C	2.67408464	0.00029901	0.31226991
C	2.67058147	0.74189532	-0.96027804
N	-1.83349769	0.51796082	0.38799327
C	-2.97045099	-0.42371317	0.63729384
H	3.59971039	1.02069817	-1.44519019
C	-1.39248120	-1.32031738	-1.01524307
C	-2.90788530	-1.36191157	-0.55359616
H	-3.53085373	-1.02681616	-1.37262340
H	-3.21258230	-2.35895944	-0.25340869
H	-2.83500384	-0.90397568	1.57740520
H	-3.85554552	0.16928873	0.78024983
H	-1.47991796	-1.14234863	-2.07765507
H	0.90650483	1.38467291	-2.27568209
C	-1.66807789	1.82459087	0.94159030
H	-0.79856751	2.27153222	0.49013659
H	-2.51336277	2.50574112	0.76730981
H	-1.46909895	1.92052745	2.01526281
H	1.02358494	-0.91236249	1.39610537
C	3.81200737	-0.54037229	1.12721726
H	4.08897738	-1.51739391	0.73385199
H	4.69362392	0.08116011	1.05241094
H	3.51712536	-0.65824304	2.16997753
H	-0.88475575	-2.27471509	-0.93910868

Time = 190.00 fs
E(CASSCF/6-31G(d)) = -479.5999348

C	-0.86874506	-0.15301442	-0.27251668
C	0.53215315	0.24509005	-0.41269239
C	1.31706340	0.99986113	-1.33909467
C	1.37268317	-0.39027430	0.51831297
C	2.67104168	-0.00036132	0.30421498
C	2.67154506	0.73326428	-0.96405765
N	-1.83481194	0.51678188	0.38589254
C	-2.97043841	-0.42623910	0.63805520
H	3.59609660	1.01566712	-1.45990547

C	-1.39450013	-1.31808548	-1.01923283
C	-2.90826160	-1.36029218	-0.55578391
H	-3.52782657	-1.02237228	-1.37488365
H	-3.20924015	-2.35932162	-0.26258500
H	-2.82703491	-0.91071042	1.57907528
H	-3.86053815	0.16607422	0.78355267
H	-1.48566901	-1.14167726	-2.07807650
H	0.90224380	1.37863559	-2.27562485
C	-1.66520090	1.82322724	0.95002712
H	-0.79888211	2.27154021	0.49573705
H	-2.51001480	2.50068951	0.78506582
H	-1.46074617	1.91278453	2.01783456
H	1.02684496	-0.91148193	1.40239838
C	3.81036699	-0.53477860	1.13502073
H	4.09229459	-1.52031139	0.76350527
H	4.69159572	0.09100020	1.06671246
H	3.51308677	-0.63594170	2.17842781
H	-0.87665160	-2.26541433	-0.94126444

Time = 190.50 fs
E(CASSCF/6-31G(d)) = -479.6004804

C	-0.87045257	-0.14907556	-0.27108987
C	0.53249109	0.24745428	-0.41326031
C	1.31812242	0.99571566	-1.34111897
C	1.37622346	-0.38782230	0.51791968
C	2.66809652	-0.00105763	0.29576959
C	2.67254949	0.72462005	-0.96755874
N	-1.83591425	0.51509619	0.38378406
C	-2.97042544	-0.42841522	0.63881245
H	3.59116319	1.01154269	-1.47442390
C	-1.39712112	-1.31594841	-1.02324486
C	-2.90852296	-1.35882935	-0.55794738
H	-3.52525163	-1.01784702	-1.37673114
H	-3.20626873	-2.35956350	-0.27213699
H	-2.81811244	-0.91844497	1.58169292
H	-3.86633287	0.16268774	0.78565812
H	-1.48948742	-1.13984988	-2.07922241
H	0.89944924	1.37263324	-2.27444066
C	-1.66223693	1.82218970	0.95859355
H	-0.79908182	2.27137348	0.50062368
H	-2.50682546	2.49388911	0.80199965
H	-1.45286364	1.90286181	2.02034535
H	1.03018784	-0.91036849	1.40837508
C	3.80873904	-0.52920339	1.14268793
H	4.09596158	-1.52323850	0.79372748
H	4.68974698	0.10107885	1.08141184

H	3.50939135	-0.61348173	2.18657543
H	-0.86795579	-2.25532023	-0.94204504

Time = 191.00 fs
E(CASSCF/6-31G(d)) = -479.6009666

C	-0.87193719	-0.14479840	-0.27006015
C	0.53253065	0.24994655	-0.41372988
C	1.31913395	0.99118503	-1.34321541
C	1.37965733	-0.38518901	0.51796822
C	2.66533239	-0.00185485	0.28703633
C	2.67358728	0.71601455	-0.97086098
N	-1.83684573	0.51297652	0.38174020
C	-2.97041084	-0.43024707	0.63959849
H	3.58491254	1.00822292	-1.48853920
C	-1.40031471	-1.31385755	-1.02727348
C	-2.90865929	-1.35752509	-0.56008942
H	-3.52320547	-1.01321426	-1.37819367
H	-3.20375620	-2.35976113	-0.28199619
H	-2.80842230	-0.92696478	1.58485538
H	-3.87258793	0.15894476	0.78660429
H	-1.49143786	-1.13691001	-2.08138338
H	0.89813803	1.36675148	-2.27227967
C	-1.65918929	1.82141381	0.96724670
H	-0.79912712	2.27109743	0.50476214
H	-2.50406975	2.48563439	0.81807194
H	-1.44533622	1.89082786	2.02322423
H	1.03378773	-0.90875414	1.41362557
C	3.80712052	-0.52363288	1.15021007
H	4.09992820	-1.52596385	0.82443202
H	4.68796200	0.11125566	1.09633885
H	3.50587931	-0.59088527	2.19441328
H	-0.85877692	-2.24461208	-0.94147445

Time = 191.50 fs
E(CASSCF/6-31G(d)) = -479.6012831

C	-0.87300293	-0.14070709	-0.26946499
C	0.53229118	0.25244526	-0.41419829
C	1.32030557	0.98641361	-1.34535875
C	1.38335928	-0.38250340	0.51844456
C	2.66261915	-0.00303189	0.27856064
C	2.67449029	0.70770384	-0.97444146
N	-1.83821605	0.51100207	0.37993830
C	-2.97021112	-0.43166727	0.64047655
H	3.57734123	1.00500626	-1.50234494
C	-1.40390572	-1.31170516	-1.03135592

C	-2.90864572	-1.35635502	-0.56218981
H	-3.52168138	-1.00844158	-1.37939815
H	-3.20177971	-2.36014682	-0.29215825
H	-2.79842443	-0.93664349	1.58827564
H	-3.87913471	0.15468139	0.78649520
H	-1.49208917	-1.13348619	-2.08523773
H	0.89829501	1.36119048	-2.26911479
C	-1.65612611	1.82064170	0.97588679
H	-0.79903265	2.27127546	0.50813751
H	-2.50186459	2.47684305	0.83342702
H	-1.43784378	1.87715760	2.02736841
H	1.03776394	-0.90651378	1.41731329
C	3.80553004	-0.51800442	1.15762201
H	4.10395247	-1.52811509	0.85539500
H	4.68611872	0.12136568	1.11135286
H	3.50229999	-0.56812689	2.20175580
H	-0.84930814	-2.23405700	-0.93972769

Time = 192.00 fs
E(CASSCF/6-31G(d)) = -479.6012782

C	-0.87385275	-0.13621214	-0.26926692
C	0.53173173	0.25502269	-0.41448938
C	1.32147605	0.98126741	-1.34753923
C	1.38674164	-0.37972436	0.51930315
C	2.66029815	-0.00432686	0.26977909
C	2.67540049	0.69939782	-0.97792233
N	-1.83939090	0.50864363	0.37816876
C	-2.97005044	-0.43281900	0.64143531
H	3.56858497	1.00231128	-1.51566816
C	-1.40802994	-1.30957650	-1.03537374
C	-2.90850713	-1.35533960	-0.56429687
H	-3.52079788	-1.00347733	-1.38026358
H	-3.20039899	-2.36061039	-0.30255658
H	-2.78789847	-0.94644409	1.59128620
H	-3.88550134	0.14967095	0.78535653
H	-1.49074445	-1.12872711	-2.09012383
H	0.89978365	1.35590974	-2.26556616
C	-1.65292152	1.82003796	0.98461741
H	-0.79885741	2.27136597	0.51090645
H	-2.50058533	2.46707593	0.84797851
H	-1.43049614	1.86122816	2.03258445
H	1.04229942	-0.90349150	1.41986986
C	3.80392523	-0.51228473	1.16495765
H	4.10816381	-1.52967731	0.88695958
H	4.68411497	0.13138065	1.12641107
H	3.49856955	-0.54508176	2.20891555

H	-0.83950158	-2.22299724	-0.93677810

Time = 192.50 fs
E(CASSCF/6-31G(d)) = -479.6010983

C	-0.87424761	-0.13201850	-0.26945334
C	0.53089592	0.25745741	-0.41476453
C	1.32280825	0.97612293	-1.34960865
C	1.39015227	-0.37713918	0.52043694
C	2.65827333	-0.00597132	0.26160187
C	2.67610566	0.69164601	-0.98167824
N	-1.84102359	0.50663365	0.37663528
C	-2.96972209	-0.43359861	0.64249700
H	3.55907753	0.99941900	-1.52829701
C	-1.41232785	-1.30745551	-1.03920863
C	-2.90823154	-1.35448419	-0.56631283
H	-3.52052630	-0.99847479	-1.38088143
H	-3.19971016	-2.36136232	-0.31282781
H	-2.77771335	-0.95644578	1.59355450
H	-3.89138068	0.14398132	0.78338671
H	-1.48804429	-1.12348401	-2.09630248
H	0.90243501	1.35134307	-2.26183213
C	-1.64975719	1.81934814	0.99303391
H	-0.79870657	2.27197111	0.51304211
H	-2.50028762	2.45764130	0.86143194
H	-1.42324184	1.84412996	2.03944516
H	1.04725357	-0.89984863	1.42055903
C	3.80238645	-0.50660813	1.17200632
H	4.11214825	-1.53023347	0.91776625
H	4.68192929	0.14080433	1.14085674
H	3.49457357	-0.52253336	2.21544076
H	-0.82992720	-2.21262607	-0.93300329

Time = 193.00 fs
E(CASSCF/6-31G(d)) = -479.6007041

C	-0.87421622	-0.12804604	-0.27003713
C	0.52977997	0.25980068	-0.41503438
C	1.32431437	0.97086283	-1.35158197
C	1.39361561	-0.37470220	0.52184091
C	2.65657596	-0.00801545	0.25389338
C	2.67661002	0.68427857	-0.98581902
N	-1.84314618	0.50496783	0.37530973
C	-2.96922038	-0.43406827	0.64370084
H	3.54888194	0.99621044	-1.54051885
C	-1.41684326	-1.30527843	-1.04293726
C	-2.90782184	-1.35376406	-0.56829259

H	-3.52085342	-0.99332124	-1.38125154
H	-3.19972409	-2.36243627	-0.32313306
H	-2.76783889	-0.96653765	1.59475130
H	-3.89668105	0.13741850	0.78066740
H	-1.48396698	-1.11770682	-2.10365404
H	0.90613430	1.34753330	-2.25824126
C	-1.64655135	1.81850924	1.00131193
H	-0.79865925	2.27308857	0.51471995
H	-2.50111304	2.44855764	0.87416569
H	-1.41587424	1.82558653	2.04810773
H	1.05273557	-0.89554385	1.41951618
C	3.80087691	-0.50083250	1.17893544
H	4.11594192	-1.52965268	0.94842881
H	4.67945744	0.14976293	1.15486606
H	3.49008326	-0.49998886	2.22149666
H	-0.82055275	-2.20273062	-0.92845208

Time = 193.50 fs
E(CASSCF/6-31G(d)) = -479.6001871

C	-0.87378730	-0.12430787	-0.27102419
C	0.52840354	0.26204335	-0.41529783
C	1.32597926	0.96549023	-1.35342783
C	1.39707652	-0.37242146	0.52347414
C	2.65528359	-0.01046878	0.24670456
C	2.67689631	0.67730549	-0.99036016
N	-1.84574605	0.50368411	0.37419519
C	-2.96854358	-0.43427199	0.64505449
H	3.53832771	0.99266422	-1.55236831
C	-1.42151759	-1.30303495	-1.04655421
C	-2.90728985	-1.35317511	-0.57024438
H	-3.52175548	-0.98803018	-1.38134064
H	-3.20045435	-2.36381842	-0.33339840
H	-2.75847074	-0.97640237	1.59456748
H	-3.90123277	0.12996045	0.77733234
H	-1.47855324	-1.11149059	-2.11182596
H	0.91070701	1.34463400	-2.25519949
C	-1.64330019	1.81748099	1.00944110
H	-0.79881387	2.27468601	0.51608507
H	-2.50312986	2.44000288	0.88627001
H	-1.40839373	1.80573293	2.05840288
H	1.05869096	-0.89069311	1.41698199
C	3.79939458	-0.49494873	1.18574809
H	4.11949265	-1.52782316	0.97882944
H	4.67667679	0.15818736	1.16827894
H	3.48497198	-0.47748652	2.22708777
H	-0.81160069	-2.19329883	-0.92326563

Time = 194.00 fs
E(CASSCF/6-31G(d)) = -479.5995837

C	-0.87300390	-0.12080790	-0.27241228
C	0.52679233	0.26417330	-0.41554748
C	1.32778981	0.96001204	-1.35512121
C	1.40047949	-0.37029807	0.52528288
C	2.65446814	-0.01333369	0.24007445
C	2.67694548	0.67073010	-0.99530644
N	-1.84879754	0.50281134	0.37328629
C	-2.96768886	-0.43425688	0.64655607
H	3.52777975	0.98878725	-1.56392186
C	-1.42628839	-1.30071926	-1.05005983
C	-2.90665139	-1.35271251	-0.57217706
H	-3.52318772	-0.98262470	-1.38110319
H	-3.20189277	-2.36546080	-0.34355540
H	-2.74977995	-0.98575939	1.59279372
H	-3.90493732	0.12163510	0.77352839
H	-1.47186109	-1.10494950	-2.12039086
H	0.91598508	1.34277820	-2.25306674
C	-1.64000597	1.81623163	1.01742348
H	-0.79927477	2.27671292	0.51730167
H	-2.50629822	2.43207965	0.89786885
H	-1.40083846	1.78473384	2.06998902
H	1.06501464	-0.88548640	1.41333022
C	3.79793506	-0.48894809	1.19244968
H	4.12275695	-1.52468416	1.00884467
H	4.67360014	0.16604202	1.18094456
H	3.47912920	-0.45506583	2.23221399
H	-0.80331298	-2.18428392	-0.91759624

Time = 194.50 fs
E(CASSCF/6-31G(d)) = -479.5988929

C	-0.87192259	-0.11754097	-0.27419104
C	0.52497708	0.26617584	-0.41576941
C	1.32973406	0.95444060	-1.35664692
C	1.40376971	-0.36832730	0.52720240
C	2.65419410	-0.01660566	0.23402953
C	2.67673865	0.66454936	-1.00065238
N	-1.85226180	0.50236905	0.37256929
C	-2.96665301	-0.43407198	0.64819365
H	3.51761569	0.98460919	-1.57528757
C	-1.43109065	-1.29832946	-1.05345754
C	-2.90592591	-1.35237078	-0.57409969
H	-3.52508823	-0.97713574	-1.38048659

H	-3.20400988	-2.36728385	-0.35354115
H	-2.74190530	-0.99437541	1.58933072
H	-3.90777011	0.11251929	0.76941178
H	-1.46396870	-1.09820815	-2.12888293
H	0.92182476	1.34206092	-2.25211694
C	-1.63667851	1.81473768	1.02527161
H	-0.80014145	2.27910506	0.51854403
H	-2.51046681	2.42481305	0.90911406
H	-1.39327865	1.76278458	2.08238833
H	1.07155659	-0.88017604	1.40904794
C	3.79649123	-0.48282189	1.19904781
H	4.12570152	-1.52023019	1.03834865
H	4.67027552	0.17332587	1.19272506
H	3.47246416	-0.43276707	2.23686894
H	-0.79593793	-2.17561945	-0.91160249

Time = 195.00 fs
E(CASSCF/6-31G(d)) = -479.5981022

C	-0.87061175	-0.11449317	-0.27634265
C	0.52299220	0.26803451	-0.41594357
C	1.33180004	0.94879471	-1.35800241
C	1.40689399	-0.36650010	0.52916019
C	2.65451714	-0.02027348	0.22858308
C	2.67625965	0.65875420	-1.00638285
N	-1.85608736	0.50236754	0.37202256
C	-2.96543306	-0.43376697	0.64994538
H	3.50819890	0.98017444	-1.58658941
C	-1.43585843	-1.29586685	-1.05675128
C	-2.90513586	-1.35214372	-0.57602107
H	-3.52738280	-0.97159975	-1.37943709
H	-3.20675660	-2.36918198	-0.36329775
H	-2.73494788	-1.00207210	1.58419276
H	-3.90977850	0.10273469	0.76514390
H	-1.45497592	-1.09139312	-2.13683491
H	0.92812099	1.34252361	-2.25250620
C	-1.63333573	1.81298299	1.03300555
H	-0.80149864	2.28178945	0.51998720
H	-2.51538338	2.41815794	0.92017759
H	-1.38580670	1.74010930	2.09503680
H	1.07813255	-0.87505829	1.40470348
C	3.79505314	-0.47656119	1.20555175
H	4.12830404	-1.51451199	1.06721677
H	4.66678322	0.18007149	1.20349865
H	3.46490884	-0.41063067	2.24103890
H	-0.78971558	-2.16723545	-0.90544389

Time = 195.50 fs
E(CASSCF/6-31G(d)) = -479.5972055

C	-0.86914923	-0.11164335	-0.27884298
C	0.52087458	0.26973238	-0.41604463
C	1.33397445	0.94309995	-1.35919913
C	1.40980176	-0.36480488	0.53108048
C	2.65548264	-0.02432013	0.22373542
C	2.67549786	0.65333009	-1.01247448
N	-1.86021160	0.50280849	0.37161779
C	-2.96402716	-0.43339071	0.65178013
H	3.49985079	0.97553288	-1.59794901
C	-1.44052671	-1.29333492	-1.05994360
C	-2.90430589	-1.35202431	-0.57794932
H	-3.52999059	-0.96605578	-1.37790666
H	-3.21006692	-2.37103270	-0.37277121
H	-2.72896684	-1.00873189	1.57750735
H	-3.91107545	0.09244117	0.76088712
H	-1.44500446	-1.08462547	-2.14381289
H	0.93481546	1.34414386	-2.25425361
C	-1.63000330	1.81095746	1.04064952
H	-0.80340735	2.28468944	0.52179768
H	-2.52071752	2.41201447	0.93124253
H	-1.37852503	1.71695528	2.10733993
H	1.08453988	-0.87044640	1.40090013
C	3.79360821	-0.47015666	1.21197194
H	4.13055350	-1.50763414	1.09533025
H	4.66323018	0.18634181	1.21316221
H	3.45642024	-0.38869632	2.24470304
H	-0.78486316	-2.15907319	-0.89927501

Time = 196.00 fs
E(CASSCF/6-31G(d)) = -479.5962286

C	-0.86761950	-0.10896495	-0.28166304
C	0.51866270	0.27125340	-0.41604302
C	1.33624151	0.93738814	-1.36026257
C	1.41244524	-0.36322977	0.53288981
C	2.65712474	-0.02872351	0.21947442
C	2.67445123	0.64825791	-1.01889716
N	-1.86456286	0.50368589	0.37132149
C	-2.96243537	-0.43298956	0.65365842
H	3.49282408	0.97072994	-1.60946597
C	-1.44503324	-1.29073831	-1.06303426
C	-2.90346190	-1.35200431	-0.57989155
H	-3.53282946	-0.96054252	-1.37585883
H	-3.21386132	-2.37270632	-0.38191042

H	-2.72397723	-1.01430215	1.56951082
H	-3.91182782	0.08182727	0.75680012
H	-1.43419644	-1.07801536	-2.14944551
H	0.94189647	1.34683188	-2.25723962
C	-1.62671336	1.80865542	1.04822879
H	-0.80589843	2.28772937	0.52412533
H	-2.52609176	2.40624789	0.94249377
H	-1.37153416	1.69358526	2.11872494
H	1.09057827	-0.86663760	1.39821745
C	3.79214184	-0.46359860	1.21831932
H	4.13244936	-1.49974932	1.12257988
H	4.65974227	0.19222503	1.22163338
H	3.44698193	-0.36700233	2.24783450
H	-0.78156205	-2.15109748	-0.89323975

Time = 196.50 fs
E(CASSCF/6-31G(d)) = -479.5952295

C	-0.86611054	-0.10642838	-0.28477045
C	0.51639601	0.27258366	-0.41590642
C	1.33858245	0.93169608	-1.36123057
C	1.41477982	-0.36176499	0.53452313
C	2.65946541	-0.03345775	0.21577682
C	2.67312867	0.64351492	-1.02561583
N	-1.86906341	0.50498730	0.37109683
C	-2.96066057	-0.43260574	0.65553365
H	3.48728116	0.96579745	-1.62119981
C	-1.44932045	-1.28808184	-1.06601956
C	-2.90263008	-1.35207397	-0.58185352
H	-3.53582094	-0.95509544	-1.37327374
H	-3.21805064	-2.37407655	-0.39066610
H	-2.71994950	-1.01879756	1.56053988
H	-3.91223992	0.07109855	0.75303329
H	-1.42271202	-1.07165844	-2.15344745
H	0.94939115	1.35043499	-2.26122279
C	-1.62350274	1.80607404	1.05576697
H	-0.80896866	2.29083917	0.52709681
H	-2.53111596	2.40071000	0.95410966
H	-1.36492216	1.67026755	2.12868477
H	1.09607332	-0.86387602	1.39714547
C	3.79063821	-0.45687704	1.22460462
H	4.13399986	-1.49104920	1.14886985
H	4.65645467	0.19782755	1.22885184
H	3.43660476	-0.34558475	2.25040231
H	-0.77994726	-2.14330596	-0.88746617

Time = 197.00 fs

E(CASSCF/6-31G(d)) = -479.5942949

C	-0.86471060	-0.10400322	-0.28813084
C	0.51411456	0.27371244	-0.41560100
C	1.34097535	0.92606377	-1.36215021
C	1.41676396	-0.36040521	0.53592933
C	2.66251378	-0.03849431	0.21260969
C	2.67155173	0.63907567	-1.03259240
N	-1.87363269	0.50669528	0.37090529
C	-2.95870946	-0.43227555	0.65735369
H	3.48328115	0.96074750	-1.63315758
C	-1.45333701	-1.28536979	-1.06889188
C	-2.90183594	-1.35222169	-0.58383948
H	-3.53889499	-0.94974430	-1.37015230
H	-3.22254028	-2.37503115	-0.39899006
H	-2.71681088	-1.02229951	1.55101934
H	-3.91253309	0.06046453	0.74972471
H	-1.41072514	-1.06563331	-2.15563671
H	0.95735151	1.35474909	-2.26586969
C	-1.62041080	1.80321207	1.06328405
H	-0.81257940	2.29395790	0.53081097
H	-2.53542025	2.39525861	0.96625518
H	-1.35875608	1.64726538	2.13681173
H	1.10089892	-0.86231739	1.39802152
C	3.78908146	-0.44998197	1.23083775
H	4.13521945	-1.48175241	1.17412088
H	4.65350094	0.20326549	1.23478029
H	3.42532649	-0.32447642	2.25237420
H	-0.78009937	-2.13573524	-0.88206208

Time = 197.50 fs
E(CASSCF/6-31G(d)) = -479.5934964

C	-0.86350520	-0.10166083	-0.29170903
C	0.51185910	0.27463280	-0.41509300
C	1.34339538	0.92053243	-1.36307411
C	1.41835970	-0.35915121	0.53707539
C	2.66626560	-0.04380322	0.20993269
C	2.66975521	0.63491287	-1.03978718
N	-1.87819101	0.50878903	0.37070842
C	-2.95659329	-0.43202800	0.65906287
H	3.48077690	0.95556962	-1.64528857
C	-1.45703902	-1.28260516	-1.07163976
C	-2.90110337	-1.35243369	-0.58585197
H	-3.54199340	-0.94451099	-1.36651876
H	-3.22723423	-2.37548112	-0.40683501
H	-2.71444928	-1.02495159	1.54144349

H	-3.91292270	0.05012542	0.74699651
H	-1.39841934	-1.06000041	-2.15594673
H	0.96583743	1.35953542	-2.27079485
C	-1.61747727	1.80006863	1.07079481
H	-0.81665801	2.29703665	0.53533610
H	-2.53868344	2.38977408	0.97907694
H	-1.35307562	1.62482650	2.14282091
H	1.10499307	-0.86200370	1.40098387
C	3.78745703	-0.44290374	1.23702710
H	4.13612562	-1.47209000	1.19827261
H	4.65100140	0.20865522	1.23940437
H	3.41321067	-0.30370596	2.25371977
H	-0.78203974	-2.12846369	-0.87711170

Time = 198.00 fs
E(CASSCF/6-31G(d)) = -479.5928948

C	-0.86257394	-0.09937530	-0.29547018
C	0.50966981	0.27534183	-0.41434982
C	1.34581577	0.91514242	-1.36405632
C	1.41953300	-0.35801084	0.53794819
C	2.67070335	-0.04935375	0.20769903
C	2.66778665	0.63099776	-1.04715945
N	-1.88266246	0.51124537	0.37046890
C	-2.95432855	-0.43188415	0.66060450
H	3.47962370	0.95023272	-1.65748730
C	-1.46039113	-1.27978966	-1.07424760
C	-2.90045397	-1.35269382	-0.58789176
H	-3.54507186	-0.93940853	-1.36242142
H	-3.23203843	-2.37536766	-0.41415457
H	-2.71272044	-1.02694858	1.53234966
H	-3.91359381	0.04025867	0.74495188
H	-1.38598114	-1.05480048	-2.15442990
H	0.97489924	1.36454036	-2.27560107
C	-1.61474022	1.79664280	1.07830771
H	-0.82110130	2.30004029	0.54070960
H	-2.54065561	2.38417116	0.99269962
H	-1.34788861	1.60317300	2.14656570
H	1.10836391	-0.86285330	1.40595097
C	3.78575303	-0.43563355	1.24317925
H	4.13673489	-1.46228953	1.22128436
H	4.64905172	0.21410383	1.24273268
H	3.40034487	-0.28329723	2.25441457
H	-0.78572906	-2.12161027	-0.87267348

Time = 198.50 fs
E(CASSCF/6-31G(d)) = -479.5924887

C	-0.86198803	-0.09712417	-0.29938051
C	0.50758555	0.27584049	-0.41334115
C	1.34820875	0.90993137	-1.36514847
C	1.42025524	-0.35699850	0.53855358
C	2.67579631	-0.05511502	0.20585723
C	2.66570458	0.62730028	-1.05466779
N	-1.88697759	0.51403965	0.37015145
C	-2.95193708	-0.43185718	0.66192398
H	3.47959716	0.94468996	-1.66960465
C	-1.46336710	-1.27692375	-1.07669588
C	-2.89990644	-1.35298345	-0.58995782
H	-3.54810166	-0.93444013	-1.35793198
H	-3.23686424	-2.37466713	-0.42090397
H	-2.71145848	-1.02851778	1.52428337
H	-3.91467988	0.03100900	0.74367294
H	-1.37359425	-1.05005466	-2.15125881
H	0.98456246	1.36951396	-2.27991749
C	-1.61223421	1.79293330	1.08582414
H	-0.82578234	2.30294733	0.54693963
H	-2.54117353	2.37840626	1.00722414
H	-1.34316836	1.58249218	2.14804484
H	1.11108316	-0.86466930	1.41263335
C	3.78396153	-0.42816404	1.24929845
H	4.13705882	-1.45255890	1.24313542
H	4.64771296	0.21970033	1.24479576
H	3.38683792	-0.26326829	2.25444384
H	-0.79106827	-2.11533178	-0.86877897

Time = 199.00 fs
E(CASSCF/6-31G(d)) = -479.5922375

C	-0.86180825	-0.09488847	-0.30340818
C	0.50564249	0.27613336	-0.41204003
C	1.35054673	0.90493221	-1.36639644
C	1.42050505	-0.35613320	0.53891311
C	2.68150121	-0.06105665	0.20435226
C	2.66357593	0.62378923	-1.06226994
N	-1.89107542	0.51714633	0.36972348
C	-2.94944578	-0.43195359	0.66297248
H	3.48041861	0.93888603	-1.68146319
C	-1.46594998	-1.27400699	-1.07896150
C	-2.89947627	-1.35328152	-0.59204741
H	-3.55107013	-0.92959935	-1.35314340
H	-3.24163097	-2.37339354	-0.42704093
H	-2.71048930	-1.02989337	1.51775590
H	-3.91624664	0.02248082	0.74321943

H	-1.36143286	-1.04576527	-2.14671914
H	0.99481662	1.37422843	-2.28342914
C	-1.60998872	1.78893877	1.09333789
H	-0.83055824	2.30574915	0.55400856
H	-2.54017027	2.37248129	1.02272743
H	-1.33885145	1.56292805	2.14740490
H	1.11327017	-0.86716487	1.42057369
C	3.78207978	-0.42048991	1.25538659
H	4.13710035	-1.44307133	1.26382412
H	4.64700407	0.22550871	1.24564518
H	3.37281641	-0.24363033	2.25380635
H	-0.79790344	-2.10981650	-0.86543316

Time = 199.50 fs
E(CASSCF/6-31G(d)) = -479.5921184

C	-0.86208391	-0.09265111	-0.30752281
C	0.50387376	0.27622860	-0.41042248
C	1.35280190	0.90017216	-1.36783742
C	1.42026659	-0.35543649	0.53905886
C	2.68776459	-0.06714691	0.20312341
C	2.66147462	0.62043025	-1.06991989
N	-1.89490212	0.52053721	0.36915475
C	-2.94688641	-0.43217566	0.66371044
H	3.48178138	0.93277167	-1.69287715
C	-1.46813235	-1.27103903	-1.08101784
C	-2.89917558	-1.35356480	-0.59415630
H	-3.55397835	-0.92487191	-1.34816563
H	-3.24626728	-2.37159647	-0.43252642
H	-2.70964401	-1.03128109	1.51319615
H	-3.91828217	0.01473707	0.74362860
H	-1.34965599	-1.04191480	-2.14119311
H	1.00560874	1.37848951	-2.28590539
C	-1.60802675	1.78465914	1.10083504
H	-0.83527887	2.30844396	0.56187818
H	-2.53767795	2.36644177	1.03926084
H	-1.33484022	1.54457258	2.14493279
H	1.11506858	-0.86999948	1.42920887
C	3.78011055	-0.41260842	1.26144271
H	4.13685156	-1.43395432	1.28336661
H	4.64689818	0.23156345	1.24535280
H	3.35842076	-0.22438815	2.25251898
H	-0.80603507	-2.10526883	-0.86261732

Time = 200.00 fs
E(CASSCF/6-31G(d)) = -479.5920409

C	-0.86285214	-0.09039734	-0.31169645
C	0.50230881	0.27613778	-0.40846873
C	1.35494710	0.89567068	-1.36949809
C	1.41953267	-0.35492864	0.53902827
C	2.69452335	-0.07335452	0.20210683
C	2.65947758	0.61718696	-1.07756865
N	-1.89841288	0.52418251	0.36841814
C	-2.94429376	-0.43252383	0.66411132
H	3.48338062	0.92630879	-1.70366957
C	-1.46991562	-1.26802011	-1.08283659
C	-2.89901322	-1.35380818	-0.59627902
H	-3.55684028	-0.92023701	-1.34312089
H	-3.25071259	-2.36935857	-0.43732624
H	-2.70877401	-1.03282816	1.51090674
H	-3.92069819	0.00779964	0.74491536
H	-1.33840314	-1.03847172	-2.13513896
H	1.01684040	1.38215064	-2.28721149
C	-1.60636389	1.78009609	1.10829407
H	-0.83979692	2.31103464	0.57049530
H	-2.53382659	2.36037495	1.05685297
H	-1.33100233	1.52746104	2.14104702
H	1.11662482	-0.87281744	1.43793265
C	3.77806276	-0.40452004	1.26746319
H	4.13629222	-1.42528137	1.30179480
H	4.64732318	0.23786833	1.24400949
H	3.34380069	-0.20553837	2.25061866
H	-0.81522940	-2.10189752	-0.86029134

Time = 200.50 fs
E(CASSCF/6-31G(d)) = -479.5919592

C	-0.86413750	-0.08811408	-0.31590338
C	0.50097326	0.27587602	-0.40616369
C	1.35695529	0.89143845	-1.37139315
C	1.41830442	-0.35462585	0.53885918
C	2.70170686	-0.07964821	0.20123527
C	2.65766252	0.61402019	-1.08516289
N	-1.90157117	0.52805014	0.36748965
C	-2.94170402	-0.43299938	0.66416412
H	3.48493804	0.91947940	-1.71368843
C	-1.47130915	-1.26495191	-1.08438951
C	-2.89899504	-1.35398525	-0.59840924
H	-3.55967934	-0.91567012	-1.33813748
H	-3.25491685	-2.36678920	-0.44141230
H	-2.70776176	-1.03459617	1.51102898
H	-3.92333905	0.00165510	0.74707345
H	-1.32779190	-1.03539556	-2.12905973

H	1.02836846	1.38512028	-2.28731893
C	-1.60500769	1.77525419	1.11568674
H	-0.84397713	2.31352338	0.57979787
H	-2.52883616	2.35440334	1.07551140
H	-1.32717503	1.51156887	2.13627707
H	1.11806867	-0.87528283	1.44615653
C	3.77595115	-0.39622881	1.27344171
H	4.13539053	-1.41706955	1.31915493
H	4.64816638	0.24439842	1.24172371
H	3.32911049	-0.18706894	2.24816393
H	-0.82523349	-2.09989585	-0.85839861

Time = 201.00 fs
E(CASSCF/6-31G(d)) = -479.5918528

C	-0.86595169	-0.08578874	-0.32012060
C	0.49988863	0.27546249	-0.40349741
C	1.35879864	0.88747604	-1.37352441
C	1.41659036	-0.35453790	0.53858643
C	2.70923934	-0.08599641	0.20043721
C	2.65610583	0.61088746	-1.09264341
N	-1.90434755	0.53210460	0.36634887
C	-2.93915326	-0.43360614	0.66387450
H	3.48622240	0.91229318	-1.72281906
C	-1.47232913	-1.26183857	-1.08565080
C	-2.89912435	-1.35406895	-0.60054011
H	-3.56252406	-0.91114653	-1.33334331
H	-3.25883997	-2.36401564	-0.44476389
H	-2.70652708	-1.03654580	1.51352490
H	-3.92599679	-0.00373672	0.75007689
H	-1.31791806	-1.03264260	-2.12346426
H	1.04000820	1.38736561	-2.28630877
C	-1.60395744	1.77014191	1.12297959
H	-0.84770436	2.31590557	0.58972104
H	-2.52300273	2.34867485	1.09522405
H	-1.32317294	1.49681372	2.13123257
H	1.11949886	-0.87710775	1.45335863
C	3.77379537	-0.38774241	1.27936924
H	4.13410528	-1.40928264	1.33550458
H	4.64928297	0.25110499	1.23862034
H	3.31450493	-0.16895981	2.24523607
H	-0.83579223	-2.09941955	-0.85687293

Time = 201.50 fs
E(CASSCF/6-31G(d)) = -479.5916915

C	-0.86829338	-0.08340909	-0.32432787

C	0.49907173	0.27492103	-0.40046680
C	1.36044775	0.88377310	-1.37588064
C	1.41440717	-0.35466643	0.53823984
C	2.71704140	-0.09236776	0.19963784
C	2.65488049	0.60774323	-1.09994501
N	-1.90671938	0.53630696	0.36497938
C	-2.93667560	-0.43435111	0.66326420
H	3.48706529	0.90479065	-1.73099273
C	-1.47299686	-1.25868729	-1.08660031
C	-2.89940246	-1.35403236	-0.60266465
H	-3.56540382	-0.90664480	-1.32885924
H	-3.26245014	-2.36117337	-0.44736925
H	-2.70502730	-1.03853893	1.51818096
H	-3.92843219	-0.00843361	0.75388180
H	-1.30885871	-1.03017543	-2.11882445
H	1.05153894	1.38891360	-2.28436710
C	-1.60320447	1.76477209	1.13013671
H	-0.85089026	2.31816617	0.60020173
H	-2.51667798	2.34335057	1.11596238
H	-1.31879819	1.48306306	2.12656073
H	1.12097464	-0.87807079	1.45911851
C	3.77161884	-0.37907221	1.28523433
H	4.13238990	-1.40183838	1.35091166
H	4.65050741	0.25792285	1.23483834
H	3.30013507	-0.15118245	2.24193705
H	-0.84666525	-2.10056564	-0.85564502

Time = 202.00 fs
E(CASSCF/6-31G(d)) = -479.5914687

C	-0.87114844	-0.08096295	-0.32850785
C	0.49853398	0.27428086	-0.39707746
C	1.36187079	0.88030790	-1.37843800
C	1.41178069	-0.35500414	0.53784351
C	2.72503113	-0.09873168	0.19876014
C	2.65405419	0.60453935	-1.10699660
N	-1.90867011	0.54061470	0.36336932
C	-2.93430201	-0.43524385	0.66236878
H	3.48737107	0.89704426	-1.73819110
C	-1.47333707	-1.25550876	-1.08722685
C	-2.89982938	-1.35384953	-0.60477605
H	-3.56834446	-0.90215024	-1.32479302
H	-3.26572168	-2.35839529	-0.44922710
H	-2.70325175	-1.04035583	1.52463217
H	-3.93039719	-0.01250163	0.75842858
H	-1.30067748	-1.02797212	-2.11553169
H	1.06271206	1.38984794	-2.28177391

C	-1.60273285	1.75916208	1.13712354
H	-0.85347747	2.32027717	0.61118178
H	-2.51024228	2.33858881	1.13768386
H	-1.31385508	1.47014808	2.12289190
H	1.12251195	-0.87802767	1.46314015
C	3.76944723	-0.37023308	1.29102335
H	4.13019742	-1.39461954	1.36545313
H	4.65166652	0.26477899	1.23052798
H	3.28614503	-0.13370014	2.23838628
H	-0.85764191	-2.10335358	-0.85464927

Time = 202.50 fs
E(CASSCF/6-31G(d)) = -479.5911985

C	-0.87449041	-0.07843775	-0.33264632
C	0.49828057	0.27357734	-0.39334600
C	1.36303291	0.87704733	-1.38116148
C	1.40874748	-0.35553447	0.53741614
C	2.73312481	-0.10505917	0.19772649
C	2.65368745	0.60122597	-1.11372195
N	-1.91018864	0.54498164	0.36151198
C	-2.93205939	-0.43629486	0.66123415
H	3.48712156	0.88915737	-1.74444672
C	-1.47337625	-1.25231736	-1.08753116
C	-2.90040433	-1.35349634	-0.60686799
H	-3.57136408	-0.89765809	-1.32123419
H	-3.26863260	-2.35580086	-0.45034781
H	-2.70121248	-1.04172344	1.53240092
H	-3.93165657	-0.01600647	0.76364427
H	-1.29343078	-1.02603377	-2.11385885
H	1.07326070	1.39030086	-2.27888399
C	-1.60252053	1.75333361	1.14391161
H	-0.85544118	2.32219636	0.62260948
H	-2.50407222	2.33452629	1.16033348
H	-1.30816893	1.45788358	2.12077717
H	1.12408326	-0.87691547	1.46526511
C	3.76730676	-0.36124310	1.29672101
H	4.12748639	-1.38748708	1.37921362
H	4.65259288	0.27160161	1.22584764
H	3.27266985	-0.11646919	2.23471554
H	-0.86855206	-2.10771151	-0.85382932

Time = 203.00 fs
E(CASSCF/6-31G(d)) = -479.5909111

C	-0.87828118	-0.07582058	-0.33673194
C	0.49830929	0.27285271	-0.38930236

C	1.36389593	0.87394781	-1.38400714
C	1.40535732	-0.35623193	0.53697197
C	2.74123665	-0.11132383	0.19646095
C	2.65383195	0.59775261	-1.12004108
N	-1.91126876	0.54935820	0.35940628
C	-2.92997000	-0.43751361	0.65991245
H	3.48637548	0.88126141	-1.74984064
C	-1.47314160	-1.24913085	-1.08752837
C	-2.90112636	-1.35295142	-0.60893477
H	-3.57447015	-0.89317571	-1.31825015
H	-3.27116194	-2.35348619	-0.45075479
H	-2.69893311	-1.04234903	1.54094392
H	-3.93200759	-0.01900645	0.76944518
H	-1.28717320	-1.02438979	-2.11393436
H	1.08291240	1.39043933	-2.27609929
C	-1.60254081	1.74731226	1.15048357
H	-0.85678815	2.32386862	0.63443890
H	-2.49850562	2.33125749	1.18384455
H	-1.30160589	1.44609034	2.12062599
H	1.12561968	-0.87475159	1.46547916
C	3.76522243	-0.35212308	1.30231082
H	4.12422679	-1.38029393	1.39228575
H	4.65313784	0.27832865	1.22095943
H	3.25983435	-0.09943943	2.23106205
H	-0.87927083	-2.11346987	-0.85314086

Time = 203.50 fs
E(CASSCF/6-31G(d)) = -479.5906349

C	-0.88247211	-0.07309799	-0.34075643
C	0.49860948	0.27215605	-0.38499179
C	1.36441927	0.87095696	-1.38692555
C	1.40167606	-0.35706316	0.53652217
C	2.74927813	-0.11750299	0.19489243
C	2.65452838	0.59407008	-1.12587268
N	-1.91190889	0.55369192	0.35705729
C	-2.92805109	-0.43890649	0.65845802
H	3.48526220	0.87351034	-1.75449599
C	-1.47266033	-1.24596990	-1.08724892
C	-2.90199464	-1.35219678	-0.61097142
H	-3.57765737	-0.88872392	-1.31588421
H	-3.27328763	-2.35151615	-0.45048519
H	-2.69643892	-1.04195310	1.54969865
H	-3.93129588	-0.02154691	0.77573960
H	-1.28196116	-1.02309844	-2.11573296
H	1.09140273	1.39044583	-2.27383271
C	-1.60276400	1.74112688	1.15683754

H	-0.85755340	2.32522875	0.64662825
H	-2.49380791	2.32881530	1.20813701
H	-1.29409209	1.43461761	2.12265631
H	1.12701536	-0.87162894	1.46391255
C	3.76321642	-0.34289605	1.30777579
H	4.12040525	-1.37289801	1.40476859
H	4.65318243	0.28491497	1.21602474
H	3.24775317	-0.08255643	2.22756080
H	-0.88971710	-2.12036570	-0.85255268

Time = 204.00 fs
E(CASSCF/6-31G(d)) = -479.5903838

C	-0.88700517	-0.07025632	-0.34471423
C	0.49916110	0.27154278	-0.38047609
C	1.36456149	0.86801627	-1.38986594
C	1.39778829	-0.35798838	0.53607616
C	2.75715688	-0.12357885	0.19295820
C	2.65580475	0.59013253	-1.13113736
N	-1.91211240	0.55792866	0.35447661
C	-2.92631442	-0.44047503	0.65692380
H	3.48396959	0.86607322	-1.75856814
C	-1.47195965	-1.24285705	-1.08673829
C	-2.90300863	-1.35121847	-0.61297364
H	-3.58090732	-0.88433680	-1.31415509
H	-3.27498490	-2.34991928	-0.44959049
H	-2.69374853	-1.04029770	1.55812499
H	-3.92942731	-0.02365650	0.78243076
H	-1.27785335	-1.02224370	-2.11908536
H	1.09849080	1.39049432	-2.27246609
C	-1.60315916	1.73480882	1.16298971
H	-0.85779547	2.32620642	0.65913719
H	-2.49014541	2.32715569	1.23311492
H	-1.28562652	1.42335950	2.12686771
H	1.12813356	-0.86770728	1.46083415
C	3.76130670	-0.33358673	1.31309900
H	4.11602915	-1.36517337	1.41676741
H	4.65264605	0.29133771	1.21119878
H	3.23653156	-0.06576285	2.22433617
H	-0.89984600	-2.12805630	-0.85204444

Time = 204.50 fs
E(CASSCF/6-31G(d)) = -479.5901346

C	-0.89181485	-0.06728199	-0.34860254
C	0.49993485	0.27107199	-0.37583263
C	1.36428431	0.86506515	-1.39278085

C	1.39379748	-0.35896378	0.53564225
C	2.76477666	-0.12953917	0.19060798
C	2.65767377	0.58590049	-1.13576237
N	-1.91188859	0.56201452	0.35168238
C	-2.92476595	-0.44221475	0.65535848
H	3.48272601	0.85912356	-1.76223167
C	-1.47106671	-1.23981598	-1.08605466
C	-2.90416804	-1.35000686	-0.61493756
H	-3.58418914	-0.88006063	-1.31305884
H	-3.27622622	-2.34868664	-0.44813594
H	-2.69086878	-1.03720771	1.56573781
H	-3.92637597	-0.02534452	0.78941970
H	-1.27490897	-1.02192738	-2.12370309
H	1.10397354	1.39072466	-2.27230547
C	-1.60369566	1.72839111	1.16897449
H	-0.85759046	2.32673168	0.67192441
H	-2.48757008	2.32615110	1.25866143
H	-1.27628652	1.41226334	2.13304488
H	1.12881488	-0.86320184	1.45663727
C	3.75950605	-0.32422075	1.31826435
H	4.11112903	-1.35701929	1.42839049
H	4.65149230	0.29759943	1.20662619
H	3.22626606	-0.04900222	2.22149434
H	-0.90963750	-2.13614324	-0.85160407

Time = 205.00 fs
E(CASSCF/6-31G(d)) = -479.5898417

C	-0.89682958	-0.06416208	-0.35242080
C	0.50089274	0.27080310	-0.37115147
C	1.36355689	0.86204546	-1.39563042
C	1.38982368	-0.35994444	0.53522774
C	2.77203835	-0.13537739	0.18780717
C	2.66013153	0.58134339	-1.13968642
N	-1.91125395	0.56589829	0.34869872
C	-2.92340544	-0.44411432	0.65380416
H	3.48177723	0.85282728	-1.76566412
C	-1.47000901	-1.23687039	-1.08526544
C	-2.90547251	-1.34855685	-0.61685944
H	-3.58746208	-0.87595104	-1.31257199
H	-3.27698211	-2.34777408	-0.44619993
H	-2.68779108	-1.03258593	1.57213290
H	-3.92218768	-0.02660024	0.79660839
H	-1.27318294	-1.02225964	-2.12921467
H	1.10769887	1.39121874	-2.27354189
C	-1.60434446	1.72190767	1.17484220
H	-0.85702618	2.32674181	0.68494540

H	-2.48601873	2.32559310	1.28463559
H	-1.26622174	1.40132651	2.14079053
H	1.12888764	-0.85836774	1.45181559
C	3.75782137	-0.31482380	1.32325696
H	4.10575864	-1.34836638	1.43974769
H	4.64973176	0.30372889	1.20243604
H	3.21704473	-0.03222082	2.21911650
H	-0.91908288	-2.14420127	-0.85122332

Time = 205.50 fs
E(CASSCF/6-31G(d)) = -479.5894508

C	-0.90197351	-0.06088493	-0.35617028
C	0.50198998	0.27079186	-0.36653025
C	1.36236025	0.85890595	-1.39838543
C	1.38599721	-0.36088750	0.53483855
C	2.77884295	-0.14109192	0.18453866
C	2.66315709	0.57644166	-1.14286395
N	-1.91023323	0.56953405	0.34555477
C	-2.92222640	-0.44615540	0.65229462
H	3.48136018	0.84732943	-1.76902798
C	-1.46881480	-1.23404334	-1.08444257
C	-2.90692123	-1.34686774	-0.61873534
H	-3.59067869	-0.87206962	-1.31265538
H	-3.27722328	-2.34710723	-0.44387211
H	-2.68449006	-1.02642078	1.57700382
H	-3.91697861	-0.02739361	0.80390214
H	-1.27272032	-1.02334753	-2.13520545
H	1.10957402	1.39198256	-2.27622382
C	-1.60507875	1.71539233	1.18065473
H	-0.85619650	2.32618720	0.69815119
H	-2.48532705	2.32520558	1.31086946
H	-1.25564059	1.39058493	2.14957917
H	1.12818062	-0.85348164	1.44692645
C	3.75625338	-0.30542051	1.32806352
H	4.09999309	-1.33918110	1.45094718
H	4.64742185	0.30977949	1.19873800
H	3.20894581	-0.01537066	2.21725471
H	-0.92817157	-2.15180945	-0.85089368

Time = 206.00 fs
E(CASSCF/6-31G(d)) = -479.5889342

C	-0.90716751	-0.05744087	-0.35985391
C	0.50317708	0.27108776	-0.36206928
C	1.36068936	0.85560474	-1.40102876
C	1.38245186	-0.36175495	0.53448000

C	2.78509506	-0.14668546	0.18080319
C	2.66671433	0.57118779	-1.14526790
N	-1.90885988	0.57288299	0.34228388
C	-2.92121672	-0.44831252	0.65085402
H	3.48167530	0.84274278	-1.77245032
C	-1.46751377	-1.23135621	-1.08365832
C	-2.90851235	-1.34494308	-0.62056092
H	-3.59378945	-0.86847966	-1.31325879
H	-3.27692321	-2.34658930	-0.44125145
H	-2.68092216	-1.01878946	1.58015440
H	-3.91092823	-0.02767810	0.81121211
H	-1.27355021	-1.02528567	-2.14125743
H	1.10956955	1.39293940	-2.28024947
C	-1.60587412	1.70887795	1.18648003
H	-0.85519681	2.32503664	0.71148779
H	-2.48525535	2.32466470	1.33716991
H	-1.24478907	1.38009563	2.15882156
H	1.12653787	-0.84881730	1.44254314
C	3.75479659	-0.29603353	1.33267196
H	4.09392527	-1.32946691	1.46209458
H	4.64466310	0.31582532	1.19561885
H	3.20203613	0.00159043	2.21593082
H	-0.93688051	-2.15857859	-0.85060167

Time = 206.50 fs
E(CASSCF/6-31G(d)) = -479.5882969

C	-0.91233051	-0.05382246	-0.36347606
C	0.50440219	0.27173126	-0.35786640
C	1.35855487	0.85211085	-1.40355501
C	1.37931650	-0.36251603	0.53415824
C	2.79070677	-0.15216379	0.17661885
C	2.67075405	0.56558673	-1.14689164
N	-1.90717524	0.57591438	0.33892243
C	-2.92035976	-0.45055353	0.64949617
H	3.48286274	0.83913837	-1.77600457
C	-1.46613761	-1.22882779	-1.08298113
C	-2.91024246	-1.34279032	-0.62233127
H	-3.59674729	-0.86524194	-1.31432604
H	-3.27606192	-2.34611080	-0.43844298
H	-2.67702592	-1.00985555	1.58150475
H	-3.90426830	-0.02739435	0.81845702
H	-1.27568060	-1.02814752	-2.14698314
H	1.10771674	1.39393414	-2.28537743
C	-1.60670823	1.70239549	1.19238659
H	-0.85411932	2.32328031	0.72489640
H	-2.48552154	2.32362493	1.36332224

H	-1.23392972	1.36991920	2.16792529
H	1.12383498	-0.84461903	1.43919728
C	3.75343980	-0.28668272	1.33707122
H	4.08766204	-1.31926318	1.47329035
H	4.64159207	0.32195569	1.19314080
H	3.19636836	0.01869696	2.21513680
H	-0.94516596	-2.16417489	-0.85032574

Time = 207.00 fs
E(CASSCF/6-31G(d)) = -479.5875926

C	-0.91738068	-0.05002478	-0.36704278
C	0.50561335	0.27275251	-0.35401301
C	1.35598289	0.84840344	-1.40596894
C	1.37670852	-0.36314861	0.53388306
C	2.79560054	-0.15753528	0.17202026
C	2.67521678	0.55965592	-1.14774973
N	-1.90522712	0.57860582	0.33550930
C	-2.91963604	-0.45284011	0.64822411
H	3.48498480	0.83653979	-1.77969694
C	-1.46472050	-1.22647314	-1.08247265
C	-2.91210601	-1.34042024	-0.62404090
H	-3.59951188	-0.86241061	-1.31579932
H	-3.27462923	-2.34555937	-0.43555428
H	-2.67272256	-0.99986296	1.58109309
H	-3.89726641	-0.02647611	0.82556412
H	-1.27909462	-1.03197983	-2.15205346
H	1.10410039	1.39474877	-2.29125529
C	-1.60756019	1.69597290	1.19843857
H	-0.85304909	2.32093136	0.73831345
H	-2.48583554	2.32174533	1.38909739
H	-1.22332109	1.36010421	2.17634570
H	1.11999557	-0.84107314	1.43731893
C	3.75216718	-0.27738474	1.34125097
H	4.08132031	-1.30864194	1.48462840
H	4.63837196	0.32826804	1.19134093
H	3.19197836	0.03597555	2.21483808
H	-0.95295887	-2.16833650	-0.85003304

Time = 207.50 fs
E(CASSCF/6-31G(d)) = -479.5869010

C	-0.92223673	-0.04604566	-0.37056204
C	0.50676021	0.27417027	-0.35059123
C	1.35301474	0.84446994	-1.40828313
C	1.37472943	-0.36363966	0.53367086
C	2.79971044	-0.16281076	0.16705824

C	2.68003489	0.55342522	-1.14787815
N	-1.90306809	0.58094276	0.33208561
C	-2.91902520	-0.45512840	0.64703044
H	3.48801844	0.83492092	-1.78345963
C	-1.46329928	-1.22430226	-1.08218586
C	-2.91409488	-1.33784640	-0.62568390
H	-3.60205350	-0.86002961	-1.31762319
H	-3.27262777	-2.34482969	-0.43269192
H	-2.66791788	-0.98912668	1.57907252
H	-3.89020600	-0.02485768	0.83246932
H	-1.28374827	-1.03679982	-2.15621885
H	1.09884809	1.39512567	-2.29745949
C	-1.60840997	1.68963410	1.20469193
H	-0.85206065	2.31802639	0.75167090
H	-2.48593071	2.31871385	1.41426135
H	-1.21320005	1.35067579	2.18362337
H	1.11500536	-0.83828338	1.43718411
C	3.75095955	-0.26815294	1.34520123
H	4.07502301	-1.29770154	1.49619456
H	4.63518026	0.33485953	1.19023141
H	3.18888264	0.05344494	2.21497756
H	-0.96016319	-2.17088460	-0.84967728

Time = 208.00 fs
E(CASSCF/6-31G(d)) = -479.5863020

C	-0.92681922	-0.04188564	-0.37404406
C	0.50779536	0.27599047	-0.34767142
C	1.34970730	0.84030354	-1.41051580
C	1.37346235	-0.36398455	0.53354675
C	2.80298218	-0.16800385	0.16180037
C	2.68513324	0.54693706	-1.14733463
N	-1.90075379	0.58291795	0.32869466
C	-2.91850793	-0.45736964	0.64589818
H	3.49185882	0.83420705	-1.78715316
C	-1.46191337	-1.22231886	-1.08216402
C	-2.91619792	-1.33508447	-0.62725406
H	-3.60435580	-0.85812977	-1.31974778
H	-3.27007516	-2.34383216	-0.42995791
H	-2.66250542	-0.97802014	1.57570180
H	-3.88336391	-0.02248284	0.83911655
H	-1.28957019	-1.04259499	-2.15932462
H	1.09211828	1.39479441	-2.30353898
C	-1.60923789	1.68339793	1.21119157
H	-0.85121547	2.31462502	0.76489648
H	-2.48558962	2.31426770	1.43858586
H	-1.20376778	1.34162853	2.18940885

H	1.10892175	-0.83625580	1.43888047
C	3.74979613	-0.25899758	1.34891215
H	4.06889504	-1.28655801	1.50806493
H	4.63219481	0.34181774	1.18980062
H	3.18707649	0.07111628	2.21548044
H	-0.96665646	-2.17172902	-0.84919724

Time = 208.50 fs
E(CASSCF/6-31G(d)) = -479.5858490

C	-0.93105212	-0.03754803	-0.37750173
C	0.50867529	0.27820367	-0.34530922
C	1.34613497	0.83590088	-1.41268932
C	1.37297073	-0.36418486	0.53354454
C	2.80537214	-0.17313165	0.15633250
C	2.69042818	0.54024724	-1.14619942
N	-1.89834189	0.58453098	0.32538222
C	-2.91806760	-0.45951102	0.64480260
H	3.49633315	0.83427870	-1.79057761
C	-1.46060401	-1.22051917	-1.08244031
C	-2.91840083	-1.33215150	-0.62874516
H	-3.60641744	-0.85672677	-1.32213104
H	-3.26700526	-2.34250022	-0.42744660
H	-2.65637217	-0.96695906	1.57132830
H	-3.87698770	-0.01931452	0.84545656
H	-1.29646281	-1.04932548	-2.16132076
H	1.08408873	1.39349855	-2.30905634
C	-1.61002431	1.67727705	1.21796933
H	-0.85056023	2.31080877	0.77791416
H	-2.48466298	2.30820879	1.46185932
H	-1.19517851	1.33292299	2.19347713
H	1.10187668	-0.83489713	1.44229877
C	3.74865646	-0.24992612	1.35237389
H	4.06305868	-1.27533428	1.52030371
H	4.62957920	0.34921103	1.19001493
H	3.18653255	0.08899358	2.21625913
H	-0.97229247	-2.17086938	-0.84851638

Time = 209.00 fs
E(CASSCF/6-31G(d)) = -479.5855559

C	-0.93486428	-0.03303893	-0.38095134
C	0.50936138	0.28077992	-0.34354070
C	1.34239417	0.83126045	-1.41483014
C	1.37329692	-0.36424481	0.53370385
C	2.80684649	-0.17821539	0.15076103
C	2.69582491	0.53342639	-1.14457706

N	-1.89589236	0.58578885	0.32219713
C	-2.91769106	-0.46149673	0.64371398
H	3.50122166	0.83497641	-1.79349173
C	-1.45941310	-1.21889101	-1.08303787
C	-2.92068598	-1.32906526	-0.63015117
H	-3.60825230	-0.85582012	-1.32474011
H	-3.26346837	-2.34079558	-0.42524211
H	-2.64940748	-0.95638202	1.56636152
H	-3.87127567	-0.01534399	0.85144547
H	-1.30430546	-1.05692871	-2.16226742
H	1.07494675	1.39101978	-2.31362136
C	-1.61074963	1.67127680	1.22504256
H	-0.85012668	2.30667977	0.79064520
H	-2.48308194	2.30041474	1.48389724
H	-1.18753058	1.32448552	2.19573555
H	1.09407202	-0.83402872	1.44715160
C	3.74752236	-0.24094362	1.35557666
H	4.05762836	-1.26414874	1.53296011
H	4.62746943	0.35707942	1.19082073
H	3.18719941	0.10707396	2.21721797
H	-0.97690604	-2.16839252	-0.84754377

Time = 209.50 fs
E(CASSCF/6-31G(d)) = -479.5854002

C	-0.93819064	-0.02836743	-0.38441421
C	0.50982088	0.28366006	-0.34237316
C	1.33861127	0.82638261	-1.41697053
C	1.37445756	-0.36416674	0.53406362
C	2.80738190	-0.18327986	0.14521499
C	2.70121405	0.52656159	-1.14259890
N	-1.89347014	0.58670812	0.31919206
C	-2.91736803	-0.46326911	0.64260147
H	3.50628196	0.83610592	-1.79563680
C	-1.45838082	-1.21741348	-1.08396970
C	-2.92303266	-1.32584353	-0.63146645
H	-3.60988844	-0.85539299	-1.32755212
H	-3.25953039	-2.33871208	-0.42341664
H	-2.64151554	-0.94672911	1.56123714
H	-3.86636186	-0.01059846	0.85704380
H	-1.31295983	-1.06532728	-2.16233571
H	1.06488227	1.38719954	-2.31691497
C	-1.61139464	1.66539367	1.23241292
H	-0.84993267	2.30235997	0.80300986
H	-2.48086366	2.29084626	1.50455233
H	-1.18085750	1.31620985	2.19622902
H	1.08576749	-0.83341315	1.45301331

C	3.74637988	-0.23205298	1.35851076
H	4.05270382	-1.25310258	1.54606487
H	4.62596212	0.36542637	1.19214707
H	3.18899969	0.12534780	2.21825668
H	-0.98032003	-2.16446768	-0.84617709

Time = 210.00 fs
E(CASSCF/6-31G(d)) = -479.5853386

C	-0.94097256	-0.02354714	-0.38791968
C	0.51003027	0.28674417	-0.34176999
C	1.33495025	0.82127203	-1.41915078
C	1.37643098	-0.36394691	0.53465135
C	2.80697214	-0.18834945	0.13984287
C	2.70647183	0.51975525	-1.14042282
N	-1.89115182	0.58732067	0.31642401
C	-2.91708849	-0.46476996	0.64143789
H	3.51127317	0.83744117	-1.79676275
C	-1.45754222	-1.21605811	-1.08523738
C	-2.92541732	-1.32250351	-0.63268566
H	-3.61136550	-0.85541304	-1.33055411
H	-3.25527147	-2.33627755	-0.42202969
H	-2.63263252	-0.93842134	1.55637298
H	-3.86230878	-0.00514569	0.86221628
H	-1.32227972	-1.07443914	-2.16180165
H	1.05408320	1.38195717	-2.31870350
C	-1.61194113	1.65961304	1.24006500
H	-0.84998714	2.29799164	0.81493205
H	-2.47811083	2.27955191	1.52372389
H	-1.17511955	1.30796015	2.19514464
H	1.07726286	-0.83279180	1.45936815
C	3.74522107	-0.22325448	1.36116640
H	4.04835956	-1.24226945	1.55962576
H	4.62510662	0.37421235	1.19390797
H	3.19182461	0.14379924	2.21927266
H	-0.98235681	-2.15934099	-0.84430903

Time = 210.50 fs
E(CASSCF/6-31G(d)) = -479.5853301

C	-0.94315723	-0.01859782	-0.39150847
C	0.50998053	0.28988671	-0.34163596
C	1.33160770	0.81594227	-1.42141741
C	1.37913612	-0.36357495	0.53547077
C	2.80564274	-0.19343720	0.13479497
C	2.71147166	0.51311370	-1.13821996
N	-1.88903213	0.58767961	0.31395224

C	-2.91683918	-0.46594242	0.64020444
H	3.51597635	0.83873325	-1.79665295
C	-1.45692351	-1.21479304	-1.08682767
C	-2.92781420	-1.31906162	-0.63380352
H	-3.61273119	-0.85583353	-1.33374214
H	-3.25078485	-2.33355275	-0.42112775
H	-2.62274642	-0.93184047	1.55212303
H	-3.85910785	0.00090505	0.86693142
H	-1.33212134	-1.08418280	-2.16102458
H	1.04273133	1.37530090	-2.31884826
C	-1.61237207	1.65390813	1.24796516
H	-0.85029892	2.29373417	0.82634768
H	-2.47500563	2.26667059	1.54136321
H	-1.17020133	1.29957466	2.19281052
H	1.06887574	-0.83192439	1.46565500
C	3.74404516	-0.21454462	1.36353307
H	4.04463294	-1.23168998	1.57362259
H	4.62490128	0.38335319	1.19600821
H	3.19552606	0.16240587	2.22016652
H	-0.98285595	-2.15332662	-0.84184026

Time = 211.00 fs
E(CASSCF/6-31G(d)) = -479.5853378

C	-0.94469732	-0.01354690	-0.39523129
C	0.50968261	0.29292229	-0.34182996
C	1.32877515	0.81041498	-1.42381102
C	1.38242749	-0.36303942	0.53649986
C	2.80346221	-0.19853424	0.13019223
C	2.71610951	0.50672409	-1.13614186
N	-1.88721935	0.58785704	0.31183177
C	-2.91660283	-0.46673454	0.63889393
H	3.52021690	0.83973754	-1.79513623
C	-1.45654139	-1.21358950	-1.08871112
C	-2.93019661	-1.31553384	-0.63481485
H	-3.61403730	-0.85659708	-1.33711884
H	-3.24617496	-2.33062619	-0.42074299
H	-2.61190984	-0.92729922	1.54872902
H	-3.85668365	0.00740673	0.87116348
H	-1.34235213	-1.09447655	-2.16040469
H	1.03099249	1.36732217	-2.31732167
C	-1.61267099	1.64824324	1.25606297
H	-0.85088854	2.28975423	0.83721513
H	-2.47179916	2.25242271	1.55747635
H	-1.16592211	1.29086503	2.18967459
H	1.06092114	-0.83061489	1.47131707
C	3.74285853	-0.20591687	1.36559918

H	4.04151935	-1.22136643	1.58801311
H	4.62529283	0.39272501	1.19834736
H	3.19991501	0.18114463	2.22084675
H	-0.98169624	-2.14677480	-0.83869174

Time = 211.50 fs
E(CASSCF/6-31G(d)) = -479.5853537

C	-0.94555451	-0.00842496	-0.39914065
C	0.50916394	0.29570471	-0.34220520
C	1.32659867	0.80471194	-1.42635275
C	1.38612433	-0.36233325	0.53770416
C	2.80053119	-0.20361108	0.12611092
C	2.72031780	0.50063966	-1.13429520
N	-1.88581499	0.58792745	0.31010699
C	-2.91636141	-0.46710385	0.63751205
H	3.52388246	0.84025112	-1.79209678
C	-1.45640649	-1.21242544	-1.09084668
C	-2.93253805	-1.31193634	-0.63571555
H	-3.61533559	-0.85763960	-1.34068836
H	-3.24155353	-2.32760246	-0.42089119
H	-2.60024583	-0.92500417	1.54628129
H	-3.85490259	0.01418123	0.87489286
H	-1.35284558	-1.10522916	-2.16033331
H	1.01900669	1.35817725	-2.31422385
C	-1.61282047	1.64258162	1.26429649
H	-0.85179174	2.28620425	0.84751800
H	-2.46879996	2.23708999	1.57211818
H	-1.16206292	1.28162176	2.18625551
H	1.05369609	-0.82871462	1.47585835
C	3.74167298	-0.19736480	1.36735343
H	4.03898462	-1.21126192	1.60273998
H	4.62618338	0.40217773	1.20082345
H	3.20477833	0.19999399	2.22123699
H	-0.97881054	-2.14001667	-0.83481230

Time = 212.00 fs
E(CASSCF/6-31G(d)) = -479.5853894

C	-0.94570406	-0.00326080	-0.40328330
C	0.50846138	0.29812527	-0.34263937
C	1.32517070	0.79884739	-1.42904128
C	1.39004658	-0.36145371	0.53904963
C	2.79696139	-0.20862891	0.12259336
C	2.72405670	0.49488475	-1.13274373
N	-1.88489863	0.58795447	0.30881003
C	-2.91609799	-0.46702019	0.63607749

H	3.52693600	0.84013197	-1.78748297
C	-1.45652563	-1.21128531	-1.09318993
C	-2.93481311	-1.30828544	-0.63650373
H	-3.61667246	-0.85889485	-1.34445060
H	-3.23703362	-2.32458976	-0.42157168
H	-2.58794880	-0.92503467	1.54470049
H	-3.85358774	0.02102877	0.87810809
H	-1.36347691	-1.11634200	-2.16115474
H	1.00688314	1.34807291	-2.30978301
C	-1.61280188	1.63689028	1.27259855
H	-0.85305631	2.28320906	0.85726185
H	-2.46635588	2.22099012	1.58538634
H	-1.15839106	1.27162805	2.18309035
H	1.04746803	-0.82612055	1.47889256
C	3.74050364	-0.18888501	1.36878698
H	4.03697955	-1.20130134	1.61773573
H	4.62744341	0.41154831	1.20333135
H	3.20990014	0.21893432	2.22127498
H	-0.97419357	-2.13331412	-0.83018065

Time = 212.50 fs
E(CASSCF/6-31G(d)) = -479.5854663

C	-0.94513739	0.00192109	-0.40769776
C	0.50761666	0.30010870	-0.34304010
C	1.32454373	0.79282657	-1.43185712
C	1.39403168	-0.36040009	0.54050621
C	2.79286303	-0.21354792	0.11966363
C	2.72729994	0.48946556	-1.13152190
N	-1.88452561	0.58798793	0.30796188
C	-2.91579567	-0.46646611	0.63462091
H	3.52941944	0.83930034	-1.78131617
C	-1.45690083	-1.21015942	-1.09569878
C	-2.93699803	-1.30459742	-0.63718020
H	-3.61808396	-0.86029762	-1.34839696
H	-3.23272439	-2.32169031	-0.42276915
H	-2.57528162	-0.92734767	1.54375091
H	-3.85254069	0.02773949	0.88080816
H	-1.37412355	-1.12771689	-2.16313674
H	0.99470278	1.33725808	-2.30433860
C	-1.61259637	1.63114059	1.28090172
H	-0.85473827	2.28086155	0.86647109
H	-2.46482617	2.20445339	1.59741327
H	-1.15468056	1.26068141	2.18069081
H	1.04246229	-0.82277942	1.48016903
C	3.73936705	-0.18047772	1.36989493
H	4.03544591	-1.19137749	1.63292072

H	4.62892644	0.42067168	1.20576138
H	3.21507540	0.23794441	2.22090793
H	-0.96790783	-2.12683464	-0.82480974

Time = 213.00 fs
E(CASSCF/6-31G(d)) = -479.5856008

C	-0.94386266	0.00710123	-0.41241418
C	0.50667427	0.30160401	-0.34333974
C	1.32474473	0.78664762	-1.43476843
C	1.39793698	-0.35917204	0.54204609
C	2.78834125	-0.21833085	0.11733615
C	2.73002531	0.48437785	-1.13064573
N	-1.88472987	0.58806571	0.30757317
C	-2.91543554	-0.46543594	0.63318211
H	3.53145381	0.83773196	-1.77369296
C	-1.45752743	-1.20904379	-1.09833678
C	-2.93907150	-1.30088829	-0.63774826
H	-3.61959185	-0.86178601	-1.35250784
H	-3.22872671	-2.31899257	-0.42445652
H	-2.56256395	-0.93179862	1.54307725
H	-3.85156328	0.03410622	0.88300564
H	-1.38466873	-1.13926378	-2.16644414
H	0.98252154	1.32601269	-2.29831026
C	-1.61218647	1.62530725	1.28914340
H	-0.85689873	2.27922540	0.87518655
H	-2.46454610	2.18779779	1.60835636
H	-1.15073167	1.24861609	2.17950091
H	1.03885249	-0.81869301	1.47958460
C	3.73827969	-0.17214628	1.37067709
H	4.03431823	-1.18136043	1.64820481
H	4.63048415	0.42939052	1.20799797
H	3.22011563	0.25700052	2.22008685
H	-0.96008753	-2.12064350	-0.81875115

Time = 213.50 fs
E(CASSCF/6-31G(d)) = -479.5857964

C	-0.94190486	0.01226472	-0.41745414
C	0.50568126	0.30257732	-0.34348875
C	1.32578382	0.78030514	-1.43773594
C	1.40163696	-0.35776925	0.54364113
C	2.78349705	-0.22294258	0.11561786
C	2.73221205	0.47960957	-1.13011758
N	-1.88552770	0.58821686	0.30764435
C	-2.91499580	-0.46393457	0.63180590
H	3.53322996	0.83545251	-1.76478302

C	-1.45839296	-1.20794025	-1.10107446
C	-2.94101505	-1.29717373	-0.63821365
H	-3.62120030	-0.86330369	-1.35674993
H	-3.22512975	-2.31656337	-0.42659742
H	-2.55015394	-0.93816796	1.54225522
H	-3.85047759	0.03993652	0.88472841
H	-1.39500408	-1.15090452	-2.17112003
H	0.97038013	1.31462986	-2.29216070
C	-1.61155786	1.61936761	1.29727033
H	-0.85960106	2.27833654	0.88346543
H	-2.46578685	2.17130340	1.61838395
H	-1.14639352	1.23532559	2.17985725
H	1.03674831	-0.81392341	1.47718368
C	3.73725630	-0.16389580	1.37113779
H	4.03352371	-1.17111043	1.66348684
H	4.63197897	0.43756385	1.20992043
H	3.22485251	0.27607370	2.21876254
H	-0.95093821	-2.11471112	-0.81209776

Time = 214.00 fs
E(CASSCF/6-31G(d)) = -479.5860247

C	-0.93930520	0.01740111	-0.42283059
C	0.50468637	0.30300815	-0.34345174
C	1.32765808	0.77379345	-1.44071753
C	1.40502107	-0.35619183	0.54526128
C	2.77842849	-0.22734939	0.11450789
C	2.73384066	0.47514191	-1.12992853
N	-1.88691990	0.58846364	0.30816549
C	-2.91445184	-0.46197626	0.63053762
H	3.53499663	0.83253028	-1.75481944
C	-1.45947713	-1.20685586	-1.10388976
C	-2.94281346	-1.29346913	-0.63858415
H	-3.62289471	-0.86480097	-1.36107545
H	-3.22200843	-2.31444213	-0.42914859
H	-2.53842525	-0.94618840	1.54084425
H	-3.84914224	0.04506308	0.88602100
H	-1.40502982	-1.16257209	-2.17707811
H	0.95831532	1.30339191	-2.28635001
C	-1.61070117	1.61330143	1.30524286
H	-0.86290744	2.27820575	0.89137957
H	-2.46871786	2.15518900	1.62766047
H	-1.14158358	1.22078113	2.18195597
H	1.03618772	-0.80859422	1.47315457
C	3.73630853	-0.15573203	1.37128565
H	4.03298398	-1.16049124	1.67865784
H	4.63329465	0.44507466	1.21140387

H	3.22914018	0.29512921	2.21688339
H	-0.94072984	-2.10892981	-0.80498217

Time = 214.50 fs
E(CASSCF/6-31G(d)) = -479.5862211

C	-0.93612124	0.02250369	-0.42854703
C	0.50373972	0.30288760	-0.34320463
C	1.33035170	0.76710986	-1.44367235
C	1.40799096	-0.35444078	0.54687343
C	2.77323264	-0.23151729	0.11399525
C	2.73489574	0.47094902	-1.13005848
N	-1.88889276	0.58882268	0.30911588
C	-2.91377729	-0.45958354	0.62941891
H	3.53704152	0.82906708	-1.74408248
C	-1.46075258	-1.20580169	-1.10676748
C	-2.94445498	-1.28978964	-0.63886932
H	-3.62464290	-0.86623450	-1.36542336
H	-3.21942216	-2.31263729	-0.43206145
H	-2.52774250	-0.95556779	1.53843386
H	-3.84746416	0.04935246	0.88694505
H	-1.41465423	-1.17420728	-2.18411032
H	0.94637526	1.29254361	-2.28128918
C	-1.60961356	1.60709123	1.31303798
H	-0.86687546	2.27882016	0.89901286
H	-2.47337711	2.13959478	1.63633246
H	-1.13630042	1.20504220	2.18583305
H	1.03713122	-0.80288991	1.46782199
C	3.73544389	-0.14766044	1.37113287
H	4.03261717	-1.14938307	1.69360565
H	4.63434455	0.45183604	1.21232087
H	3.23285601	0.31412688	2.21439488
H	-0.92978419	-2.10313879	-0.79757099

Time = 215.00 fs
E(CASSCF/6-31G(d)) = -479.5862920

C	-0.93242915	0.02756778	-0.43459742
C	0.50289786	0.30221697	-0.34273088
C	1.33383202	0.76025796	-1.44656366
C	1.41045473	-0.35251685	0.54843842
C	2.76800871	-0.23540992	0.11405550
C	2.73537108	0.46699640	-1.13047482
N	-1.89141789	0.58930609	0.31046455
C	-2.91294639	-0.45678659	0.62848379
H	3.53966653	0.82518739	-1.73287665
C	-1.46218641	-1.20479173	-1.10969757

C	-2.94593125	-1.28615023	-0.63908035
H	-3.62639795	-0.86756661	-1.36972096
H	-3.21741482	-2.31112449	-0.43528272
H	-2.51843671	-0.96600377	1.53467753
H	-3.84540507	0.05271104	0.88757897
H	-1.42379282	-1.18575329	-2.19190651
H	0.93463829	1.28226615	-2.27729990
C	-1.60830017	1.60072347	1.32065022
H	-0.87155446	2.28014337	0.90645899
H	-2.47965595	2.12457417	1.64451903
H	-1.13062600	1.18825583	2.19136204
H	1.03946170	-0.79705560	1.46163461
C	3.73466516	-0.13968528	1.37069396
H	4.03234073	-1.13769422	1.70822088
H	4.63507737	0.45779492	1.21254499
H	3.23590039	0.33302211	2.21124074
H	-0.91845814	-2.09715202	-0.79005577

Time = 215.50 fs
E(CASSCF/6-31G(d)) = -479.5861178

C	-0.92832509	0.03258863	-0.44096844
C	0.50223083	0.30100746	-0.34201853
C	1.33804270	0.75325009	-1.44936003
C	1.41232058	-0.35041922	0.54990898
C	2.76286136	-0.23898699	0.11464711
C	2.73527630	0.46323916	-1.13113024
N	-1.89445281	0.58992267	0.31217198
C	-2.91193624	-0.45362250	0.62775581
H	3.54315635	0.82102591	-1.72150261
C	-1.46374236	-1.20384192	-1.11267266
C	-2.94723716	-1.28256580	-0.63922978
H	-3.62810258	-0.86876387	-1.37388726
H	-3.21601593	-2.30984787	-0.43875441
H	-2.51078500	-0.97719622	1.52931676
H	-3.84298298	0.05508810	0.88801709
H	-1.43236627	-1.19714880	-2.20008353
H	0.92323084	1.27265637	-2.27458710
C	-1.60677508	1.59418945	1.32809006
H	-0.87698174	2.28211605	0.91381914
H	-2.48730195	2.11009704	1.65230887
H	-1.12471483	1.17064540	2.19827330
H	1.04299182	-0.79139309	1.45514429
C	3.73397016	-0.13180889	1.36998427
H	4.03207423	-1.12537023	1.72240348
H	4.63548044	0.46293408	1.21195459
H	3.23819542	0.35176836	2.20736549

H	-0.90712448	-2.09078490	-0.78264135

Time = 216.00 fs
E(CASSCF/6-31G(d)) = -479.5855774

C	-0.92392199	0.03755999	-0.44764228
C	0.50182354	0.29928319	-0.34106180
C	1.34289566	0.74610826	-1.45203448
C	1.41349413	-0.34814714	0.55123231
C	2.75790343	-0.24220299	0.11570674
C	2.73464451	0.45961976	-1.13195950
N	-1.89794308	0.59067937	0.31419136
C	-2.91072794	-0.45013414	0.62724679
H	3.54774298	0.81671441	-1.71022714
C	-1.46538337	-1.20296904	-1.11568514
C	-2.94837053	-1.27905109	-0.63933114
H	-3.62969365	-0.86979515	-1.37783754
H	-3.21524227	-2.30872502	-0.44241414
H	-2.50499884	-0.98886198	1.52219740
H	-3.84027005	0.05647558	0.88836740
H	-1.44029712	-1.20832130	-2.20821991
H	0.91233717	1.26371457	-2.27322583
C	-1.60506143	1.58748523	1.33538006
H	-0.88317872	2.28465949	0.92119932
H	-2.49593951	2.09606465	1.65976394
H	-1.11877287	1.15249269	2.20619276
H	1.04747731	-0.78624769	1.44896895
C	3.73335150	-0.12403132	1.36901878
H	4.03174205	-1.11239955	1.73606839
H	4.63557838	0.46727222	1.21043621
H	3.23968396	0.37032026	2.20271801
H	-0.89615264	-2.08387814	-0.77553406

Time = 216.50 fs
E(CASSCF/6-31G(d)) = -479.5845715

C	-0.91934350	0.04247311	-0.45459710
C	0.50176989	0.29708885	-0.33986938
C	1.34826309	0.73886267	-1.45456120
C	1.41388388	-0.34570421	0.55235760
C	2.75325505	-0.24500788	0.11714637
C	2.73353945	0.45606682	-1.13287660
N	-1.90182336	0.59158133	0.31646892
C	-2.90930756	-0.44636923	0.62695677
H	3.55357079	0.81236928	-1.69925586
C	-1.46707431	-1.20218904	-1.11872498
C	-2.94933179	-1.27562046	-0.63939847

H	-3.63110662	-0.87062975	-1.38148869
H	-3.21509968	-2.30765386	-0.44619537
H	-2.50121796	-1.00074615	1.51327636
H	-3.83738686	0.05690527	0.88874762
H	-1.44750796	-1.21918352	-2.21589287
H	0.90219778	1.25534239	-2.27316526
C	-1.60319039	1.58061126	1.34255070
H	-0.89014751	2.28767934	0.92870637
H	-2.50510456	2.08233134	1.66692707
H	-1.11303392	1.13411621	2.21468571
H	1.05263568	-0.78198329	1.44373910
C	3.73279647	-0.11635024	1.36781138
H	4.03127577	-1.09881581	1.74914949
H	4.63542876	0.47086275	1.20788740
H	3.24033021	0.38863706	2.19725648
H	-0.88589050	-2.07631630	-0.76892978

Time = 217.00 fs
E(CASSCF/6-31G(d)) = -479.5830521

C	-0.91426499	0.04766160	-0.46205012
C	0.50185570	0.29494657	-0.33930399
C	1.35392818	0.73086194	-1.45689728
C	1.41443646	-0.34297749	0.55366588
C	2.74818670	-0.24785929	0.11926312
C	2.73185396	0.45238439	-1.13385505
N	-1.90596164	0.59242238	0.31906836
C	-2.90766242	-0.44214917	0.62684390
H	3.56117262	0.80812982	-1.68786484
C	-1.46901324	-1.20142920	-1.12215348
C	-2.95018132	-1.27206711	-0.63946842
H	-3.63236045	-0.87129539	-1.38495543
H	-3.21556009	-2.30637629	-0.45025044
H	-2.49922784	-1.01305405	1.50181494
H	-3.83424019	0.05642832	0.88928764
H	-1.45431652	-1.23034054	-2.22304150
H	0.89240015	1.24647172	-2.27449973
C	-1.60105294	1.57320050	1.35016518
H	-0.89823502	2.29107673	0.93683307
H	-2.51495353	2.06767296	1.67422446
H	-1.10762818	1.11464419	2.22354159
H	1.05874072	-0.77828688	1.44025137
C	3.73221406	-0.10831741	1.36633540
H	4.03091425	-1.08373672	1.76256857
H	4.63511067	0.47410879	1.20396819
H	3.24055322	0.40794452	2.19067498
H	-0.87600405	-2.06682232	-0.76249547

Time = 217.50 fs
E(CASSCF/6-31G(d)) = -479.5810527

C	-0.90909596	0.05282095	-0.46951034
C	0.50207265	0.29292348	-0.33939574
C	1.35958799	0.72262322	-1.45896818
C	1.41514888	-0.34011020	0.55506678
C	2.74299923	-0.25058936	0.12202033
C	2.72964445	0.44886074	-1.13491812
N	-1.91003494	0.59314428	0.32180610
C	-2.90588299	-0.43778191	0.62690709
H	3.56999044	0.80421357	-1.67688018
C	-1.47105068	-1.20071965	-1.12578734
C	-2.95086243	-1.26862031	-0.63955299
H	-3.63330649	-0.87170582	-1.38794377
H	-3.21657688	-2.30485381	-0.45426061
H	-2.49922444	-1.02484986	1.48865647
H	-3.83123799	0.05515101	0.89008472
H	-1.46022899	-1.24107463	-2.22891340
H	0.88395328	1.23752095	-2.27687206
C	-1.59882535	1.56570494	1.35778572
H	-0.90688487	2.29455805	0.94514218
H	-2.52433548	2.05282695	1.68123312
H	-1.10311021	1.09566080	2.23179268
H	1.06520077	-0.77559399	1.43935537
C	3.73162368	-0.10043840	1.36469984
H	4.03066176	-1.06819384	1.77542975
H	4.63475851	0.47687522	1.19876608
H	3.24037202	0.42697850	2.18329557
H	-0.86737780	-2.05593458	-0.75675838

Time = 218.00 fs
E(CASSCF/6-31G(d)) = -479.5786975

C	-0.90393974	0.05796627	-0.47695678
C	0.50243062	0.29094227	-0.34009020
C	1.36527821	0.71418556	-1.46085843
C	1.41599618	-0.33709054	0.55655236
C	2.73771335	-0.25319166	0.12545691
C	2.72691512	0.44553334	-1.13612906
N	-1.91398524	0.59375585	0.32465326
C	-2.90396507	-0.43331920	0.62712273
H	3.57975483	0.80051205	-1.66633225
C	-1.47315431	-1.20003876	-1.12963600
C	-2.95137773	-1.26528286	-0.63966725
H	-3.63389775	-0.87181762	-1.39040886

H	-3.21811108	-2.30298849	-0.45815672
H	-2.50114654	-1.03604352	1.47410409
H	-3.82860969	0.05320227	0.89125956
H	-1.46521352	-1.25136420	-2.23333201
H	0.87723118	1.22833667	-2.27992984
C	-1.59655756	1.55811803	1.36544058
H	-0.91601301	2.29804302	0.95369986
H	-2.53275069	2.03765323	1.68800655
H	-1.09962163	1.07749338	2.23910966
H	1.07183470	-0.77399749	1.44139176
C	3.73100912	-0.09271012	1.36292280
H	4.03048955	-1.05233144	1.78770500
H	4.63448787	0.47925022	1.19217895
H	3.23982738	0.44569306	2.17507356
H	-0.86018828	-2.04376174	-0.75179529

Time = 218.50 fs
E(CASSCF/6-31G(d)) = -479.5760635

C	-0.89889935	0.06310590	-0.48437113
C	0.50295171	0.28894274	-0.34133204
C	1.37099907	0.70559199	-1.46264332
C	1.41692226	-0.33392197	0.55813162
C	2.73237367	-0.25564595	0.12957250
C	2.72371020	0.44241732	-1.13751995
N	-1.91775475	0.59427079	0.32757783
C	-2.90191208	-0.42881189	0.62745845
H	3.59007960	0.79688956	-1.65618564
C	-1.47529647	-1.19936210	-1.13369630
C	-2.95173067	-1.26205531	-0.63982511
H	-3.63410658	-0.87158181	-1.39232892
H	-3.22012360	-2.30070366	-0.46186722
H	-2.50486121	-1.04660715	1.45855793
H	-3.82657508	0.05072177	0.89291867
H	-1.46924803	-1.26117719	-2.23622822
H	0.87258146	1.21875874	-2.28333519
C	-1.59429878	1.55043576	1.37314913
H	-0.92552517	2.30145350	0.96257058
H	-2.53977533	2.02205450	1.69461578
H	-1.09724497	1.06041158	2.24525251
H	1.07853035	-0.77347469	1.44643094
C	3.73035506	-0.08512648	1.36101780
H	4.03036924	-1.03631668	1.79938634
H	4.63439650	0.48131999	1.18412604
H	3.23896079	0.46405980	2.16599333
H	-0.85454460	-2.03051016	-0.74764514

Time = 219.00 fs
E(CASSCF/6-31G(d)) = -479.5731801

C	-0.89407298	0.06824300	-0.49173524
C	0.50366112	0.28688406	-0.34307551
C	1.37672130	0.69688419	-1.46438722
C	1.41785842	-0.33062235	0.55983662
C	2.72703608	-0.25792604	0.13433991
C	2.72010345	0.43951197	-1.13909787
N	-1.92128382	0.59470384	0.33054652
C	-2.89973737	-0.42430848	0.62787521
H	3.60049745	0.79319851	-1.64636472
C	-1.47745615	-1.19866118	-1.13795602
C	-2.95192501	-1.25893556	-0.64003953
H	-3.63392628	-0.87094434	-1.39370627
H	-3.22257580	-2.29794814	-0.46531939
H	-2.51017010	-1.05656999	1.44249611
H	-3.82532042	0.04784687	0.89514636
H	-1.47232083	-1.27047748	-2.23764600
H	0.87030216	1.20864175	-2.28679886
C	-1.59209520	1.54265753	1.38092156
H	-0.93531704	2.30471278	0.97181329
H	-2.54508631	2.00598135	1.70115244
H	-1.09600027	1.04461826	2.25007670
H	1.08525006	-0.77387509	1.45427318
C	3.72964819	-0.07767984	1.35899441
H	4.03027757	-1.02032845	1.81048479
H	4.63455359	0.48316105	1.17454959
H	3.23781979	0.48207045	2.15607071
H	-0.85048445	-2.01646615	-0.74430414

Time = 219.50 fs
E(CASSCF/6-31G(d)) = -479.5700300

C	-0.88955127	0.07337700	-0.49903080
C	0.50458174	0.28473925	-0.34528367
C	1.38239691	0.68810020	-1.46614501
C	1.41873093	-0.32722088	0.56172088
C	2.72176078	-0.26000357	0.13971477
C	2.71618827	0.43680587	-1.14085132
N	-1.92451191	0.59506970	0.33352650
C	-2.89746495	-0.41985380	0.62833009
H	3.61050296	0.78929280	-1.63678358
C	-1.47961910	-1.19790296	-1.14239597
C	-2.95196467	-1.25591856	-0.64032260
H	-3.63337090	-0.86984718	-1.39456714
H	-3.22542975	-2.29469895	-0.46844122

H	-2.51681998	-1.06601249	1.42645194
H	-3.82497634	0.04470108	0.89799748
H	-1.47443150	-1.27923166	-2.23774296
H	0.87062304	1.19787540	-2.29010430
C	-1.58998846	1.53478606	1.38875844
H	-0.94527632	2.30774634	0.98147897
H	-2.54847705	1.98943422	1.70773010
H	-1.09584161	1.03024273	2.25353809
H	1.09201851	-0.77493154	1.46448352
C	3.72887844	-0.07036174	1.35685877
H	4.03019609	-1.00454526	1.82102595
H	4.63499120	0.48483206	1.16341703
H	3.23645890	0.49973724	2.14535185
H	-0.84797290	-2.00198188	-0.74172326

Time = 220.00 fs
E(CASSCF/6-31G(d)) = -479.5665610

C	-0.88541551	0.07850491	-0.50624000
C	0.50573147	0.28248983	-0.34792518
C	1.38796843	0.67927318	-1.46796294
C	1.41946504	-0.32375410	0.56385300
C	2.71660874	-0.26184992	0.14564158
C	2.71206944	0.43427983	-1.14275458
N	-1.92737995	0.59538308	0.33648683
C	-2.89512891	-0.41548841	0.62877932
H	3.61959706	0.78504111	-1.62737096
C	-1.48177718	-1.19705042	-1.14699088
C	-2.95185358	-1.25299624	-0.64068543
H	-3.63247414	-0.86822986	-1.39495988
H	-3.22864858	-2.29096210	-0.47116401
H	-2.52451778	-1.07505517	1.41098596
H	-3.82560125	0.04138562	0.90149174
H	-1.47559128	-1.28741312	-2.23678455
H	0.87369119	1.18639911	-2.29312294
C	-1.58801451	1.52682731	1.39665058
H	-0.95528488	2.31048386	0.99160940
H	-2.54986592	1.97246325	1.71448346
H	-1.09665625	1.01733636	2.25569520
H	1.09889731	-0.77629115	1.47645565
C	3.72804021	-0.06316348	1.35461396
H	4.03010946	-0.98913317	1.83104394
H	4.63569791	0.48636804	1.15072371
H	3.23493902	0.51709144	2.13391049
H	-0.84690678	-1.98746089	-0.73981058

Time = 220.50 fs

E(CASSCF/6-31G(d)) = -479.5627366

C	-0.88173648	0.08362203	-0.51334597
C	0.50712193	0.28012232	-0.35097179
C	1.39337478	0.67043077	-1.46987888
C	1.41998795	-0.32026161	0.56630866
C	2.71163960	-0.26343701	0.15205735
C	2.70785701	0.43190820	-1.14476990
N	-1.92983215	0.59565855	0.33939913
C	-2.89277210	-0.41124882	0.62918144
H	3.62732748	0.78033833	-1.61808896
C	-1.48392749	-1.19606385	-1.15171032
C	-2.95159581	-1.25015770	-0.64113825
H	-3.63128675	-0.86603181	-1.39495141
H	-3.23219670	-2.28677101	-0.47342478
H	-2.53294887	-1.08383981	1.39665194
H	-3.82717171	0.03797492	0.90561051
H	-1.47582284	-1.29500473	-2.23513012
H	0.87956132	1.17421022	-2.29582404
C	-1.58620272	1.51879052	1.40457927
H	-0.96522174	2.31286070	1.00223695
H	-2.54929900	1.95516532	1.72156655
H	-1.09826592	1.00587017	2.25670755
H	1.10595599	-0.77755568	1.48949129
C	3.72713307	-0.05607664	1.35226046
H	4.03000293	-0.97423416	1.84057552
H	4.63661673	0.48777714	1.13649479
H	3.23332662	0.53418170	2.12184420
H	-0.84712407	-1.97333937	-0.73843684

Time = 221.00 fs
E(CASSCF/6-31G(d)) = -479.5585660

C	-0.87857372	0.08872254	-0.52033300
C	0.50875780	0.27762625	-0.35439678
C	1.39855518	0.66159477	-1.47192214
C	1.42023154	-0.31678225	0.56916272
C	2.70690995	-0.26473797	0.15889424
C	2.70366145	0.42965973	-1.14684920
N	-1.93181743	0.59591035	0.34223810
C	-2.89044377	-0.40716876	0.62950051
H	3.63332220	0.77511440	-1.60894506
C	-1.48607103	-1.19490275	-1.15651965
C	-2.95119587	-1.24738954	-0.64169043
H	-3.62987288	-0.86319515	-1.39462211
H	-3.23603974	-2.28218265	-0.47516901
H	-2.54179759	-1.09250362	1.38395918

H	-3.82958186	0.03451684	0.91029655
H	-1.47515933	-1.30199962	-2.23321042
H	0.88819096	1.16136750	-2.29827914
C	-1.58457543	1.51068819	1.41251676
H	-0.97496566	2.31481920	1.01338456
H	-2.54694702	1.93767881	1.72914942
H	-1.10043099	0.99573536	2.25682968
H	1.11324586	-0.77832230	1.50287863
C	3.72616202	-0.04909365	1.34979674
H	4.02986006	-0.95995647	1.84965409
H	4.63764754	0.48904086	1.12078669
H	3.23169340	0.55107101	2.10926973
H	-0.84841899	-1.96006297	-0.73744527

Time = 221.50 fs
E(CASSCF/6-31G(d)) = -479.5541239

C	-0.87597494	0.09379981	-0.52718670
C	0.51063630	0.27499282	-0.35817396
C	1.40345215	0.65278089	-1.47411339
C	1.42013506	-0.31335086	0.57248222
C	2.70247154	-0.26572775	0.16608174
C	2.69958944	0.42749835	-1.14893566
N	-1.93329058	0.59615195	0.34498175
C	-2.88819642	-0.40328103	0.62970886
H	3.63731547	0.76933988	-1.59999779
C	-1.48821093	-1.19352817	-1.16138153
C	-2.95065907	-1.24467634	-0.64235053
H	-3.62830542	-0.85966785	-1.39405982
H	-3.24014403	-2.27727202	-0.47635345
H	-2.55076745	-1.10114920	1.37333481
H	-3.83265222	0.03103710	0.91545686
H	-1.47364309	-1.30840038	-2.23149561
H	0.89943986	1.14799041	-2.30066137
C	-1.58314764	1.50253592	1.42042751
H	-0.98439749	2.31631018	1.02506562
H	-2.54309648	1.92017630	1.73741372
H	-1.10285806	0.98674793	2.25639920
H	1.12078061	-0.77821772	1.51595909
C	3.72513722	-0.04220824	1.34722007
H	4.02966124	-0.94636741	1.85830512
H	4.63865427	0.49011725	1.10368677
H	3.23011633	0.56783332	2.09631751
H	-0.85056260	-1.94805878	-0.73666542

Time = 222.00 fs
E(CASSCF/6-31G(d)) = -479.5495451

C	-0.87397601	0.09884676	-0.53389395
C	0.51274721	0.27221422	-0.36227723
C	1.40801359	0.64399896	-1.47646468
C	1.41964720	-0.30999600	0.57632146
C	2.69836961	-0.26638342	0.17354841
C	2.69574030	0.42538401	-1.15096527
N	-1.93421308	0.59639577	0.34761152
C	-2.88608244	-0.39961976	0.62978920
H	3.63916512	0.76302854	-1.59135864
C	-1.49035047	-1.19190538	-1.16625773
C	-2.94999201	-1.24200130	-0.64312644
H	-3.62666062	-0.85540737	-1.39335243
H	-3.24447598	-2.27212504	-0.47694871
H	-2.55959857	-1.10981567	1.36509210
H	-3.83614654	0.02754642	0.92096834
H	-1.47132415	-1.31421610	-2.23045553
H	0.91307561	1.13425389	-2.30323930
C	-1.58192706	1.49435206	1.42827000
H	-0.99340250	2.31729422	1.03728451
H	-2.53813289	1.90285473	1.74654663
H	-1.10521233	0.97865925	2.25581754
H	1.12852538	-0.77692465	1.52817602
C	3.72407323	-0.03541575	1.34452732
H	4.02938230	-0.93348988	1.86654166
H	4.63947531	0.49094595	1.08531218
H	3.22867879	0.58454814	2.08312500
H	-0.85332717	-1.93770613	-0.73593047

Time = 222.50 fs
E(CASSCF/6-31G(d)) = -479.5450226

C	-0.87260089	0.10385587	-0.54044267
C	0.51507241	0.26928325	-0.36668024
C	1.41219445	0.63525336	-1.47898007
C	1.41872792	-0.30673845	0.58071833
C	2.69464124	-0.26668466	0.18122341
C	2.69220287	0.42327353	-1.15286853
N	-1.93455384	0.59665253	0.35011243
C	-2.88415060	-0.39622193	0.62973557
H	3.63886404	0.75623769	-1.58318989
C	-1.49249088	-1.19000655	-1.17111137
C	-2.94920306	-1.23934680	-0.64402539
H	-3.62501238	-0.85038374	-1.39258059
H	-3.24900077	-2.26683031	-0.47694196
H	-2.56807896	-1.11845597	1.35940853
H	-3.83979254	0.02404934	0.92668477

H	-1.46825941	-1.31945788	-2.23051366
H	0.92878474	1.12037921	-2.30636331
C	-1.58091449	1.48615676	1.43599945
H	-1.00187244	2.31774299	1.05003625
H	-2.53251626	1.88592258	1.75673244
H	-1.10713559	0.97117307	2.25552129
H	1.13639225	-0.77419846	1.53910772
C	3.72298792	-0.02871321	1.34171586
H	4.02899449	-0.92130239	1.87436260
H	4.63993684	0.49145510	1.06580702
H	3.22747162	0.60129495	2.06982988
H	-0.85651240	-1.92930660	-0.73509635

Time = 223.00 fs
E(CASSCF/6-31G(d)) = -479.5407652

C	-0.87186166	0.10881948	-0.54682220
C	0.51758699	0.26619333	-0.37135645
C	1.41595816	0.62654348	-1.48165646
C	1.41734992	-0.30359044	0.58569147
C	2.69131326	-0.26661420	0.18903851
C	2.68905193	0.42112218	-1.15457308
N	-1.93429004	0.59693100	0.35247311
C	-2.88244338	-0.39312814	0.62955317
H	3.63654464	0.74906502	-1.57569668
C	-1.49462931	-1.18781349	-1.17590942
C	-2.94830289	-1.23669521	-0.64505408
H	-3.62342751	-0.84458281	-1.39181169
H	-3.25368139	-2.26147211	-0.47633890
H	-2.57604824	-1.12692852	1.35632030
H	-3.84330748	0.02055241	0.93244638
H	-1.46451168	-1.32413285	-2.23200120
H	0.94618991	1.10661770	-2.31044130
C	-1.58010442	1.47797129	1.44357207
H	-1.00970670	2.31764039	1.06330690
H	-2.52674583	1.86958529	1.76814264
H	-1.10827031	0.96397075	2.25594412
H	1.14423956	-0.76987593	1.54848704
C	3.72190128	-0.02209916	1.33878432
H	4.02846692	-0.90974425	1.88175286
H	4.63986771	0.49156984	1.04533733
H	3.22659341	0.61814811	2.05656438
H	-0.85996879	-1.92305909	-0.73406031

Time = 223.50 fs
E(CASSCF/6-31G(d)) = -479.5369897

C	-0.87175909	0.11372956	-0.55302268
C	0.52025867	0.26293832	-0.37627879
C	1.41927645	0.61786562	-1.48448632
C	1.41549913	-0.30055533	0.59123933
C	2.68840166	-0.26615769	0.19692853
C	2.68634683	0.41888415	-1.15600467
N	-1.93340730	0.59723742	0.35468576
C	-2.88099452	-0.39038183	0.62925668
H	3.63247504	0.74164514	-1.56912282
C	-1.49675738	-1.18531926	-1.18062476
C	-2.94730485	-1.23402945	-0.64621859
H	-3.62196091	-0.83800844	-1.39109386
H	-3.25847722	-2.25612264	-0.47516559
H	-2.58339529	-1.13500243	1.35572961
H	-3.84641973	0.01707154	0.93808777
H	-1.46015044	-1.32823808	-2.23511736
H	0.96487704	1.09323008	-2.31590448
C	-1.57948608	1.46981628	1.45094927
H	-1.01681476	2.31698354	1.07707375
H	-2.52131810	1.85402685	1.78092289
H	-1.10828801	0.95674158	2.25747179
H	1.15187713	-0.76388118	1.55621113
C	3.72083401	-0.01557319	1.33573329
H	4.02776675	-0.89872187	1.88868528
H	4.63911350	0.49121875	1.02408636
H	3.22615166	0.63517028	2.04344779
H	-0.86361453	-1.91904272	-0.73277520

Time = 224.00 fs
E(CASSCF/6-31G(d)) = -479.5338891

C	-0.87228341	0.11857795	-0.55903519
C	0.52304903	0.25951269	-0.38141996
C	1.42212970	0.60921462	-1.48746052
C	1.41317543	-0.29762760	0.59733938
C	2.68591030	-0.26530413	0.20483278
C	2.68412937	0.41651427	-1.15708993
N	-1.93190008	0.59757509	0.35674612
C	-2.87982777	-0.38802756	0.62886790
H	3.62704657	0.73414178	-1.56373835
C	-1.49886046	-1.18252913	-1.18523792
C	-2.94622522	-1.23133356	-0.64752438
H	-3.62065250	-0.83068417	-1.39045246
H	-3.26334315	-2.25083670	-0.47346914
H	-2.59004979	-1.14237726	1.35742579
H	-3.84888979	0.01363708	0.94344679
H	-1.45525280	-1.33175473	-2.23990402

H	0.98442547	1.08045833	-2.32316075
C	-1.57904486	1.46171051	1.45810296
H	-1.02311745	2.31578228	1.09130578
H	-2.51667766	1.83939190	1.79517815
H	-1.10692069	0.94921646	2.26039484
H	1.15907074	-0.75622527	1.56234553
C	3.71980627	-0.00913529	1.33256574
H	4.02686310	-0.88811948	1.89512401
H	4.63754949	0.49034125	1.00224835
H	3.22626198	0.65240768	2.03058285
H	-0.86744257	-1.91721144	-0.73125781

Time = 224.50 fs
E(CASSCF/6-31G(d)) = -479.5315914

C	-0.87341481	0.12335622	-0.56485161
C	0.52591365	0.25591152	-0.38675200
C	1.42450594	0.60058642	-1.49057257
C	1.41039223	-0.29479310	0.60394812
C	2.68383078	-0.26404572	0.21269548
C	2.68242379	0.41396902	-1.15775817
N	-1.92977183	0.59794423	0.35865331
C	-2.87895596	-0.38610822	0.62841273
H	3.62075018	0.72673796	-1.55982733
C	-1.50091802	-1.17946015	-1.18973767
C	-2.94508328	-1.22859324	-0.64897613
H	-3.61952568	-0.82265354	-1.38988878
H	-3.26822893	-2.24564793	-0.47131797
H	-2.59597224	-1.14871322	1.36111371
H	-3.85052680	0.01029683	0.94837159
H	-1.44990459	-1.33464538	-2.24623948
H	1.00444197	1.06849497	-2.33254001
C	-1.57876380	1.45366972	1.46501998
H	-1.02854901	2.31405909	1.10596460
H	-2.51316975	1.82576823	1.81095781
H	-1.10398795	0.94119797	2.26486824
H	1.16555049	-0.74700398	1.56712114
C	3.71883662	-0.00278513	1.32928734
H	4.02572886	-0.87780943	1.90102969
H	4.63509170	0.48889214	0.98002161
H	3.22704758	0.66988634	2.01805119
H	-0.87151571	-1.91740035	-0.72958811

Time = 225.00 fs
E(CASSCF/6-31G(d)) = -479.5301496

C	-0.87512423	0.12805570	-0.57046459

C	0.52880296	0.25213067	-0.39224642
C	1.42640038	0.59198016	-1.49382261
C	1.40717580	-0.29202981	0.61100197
C	2.68214256	-0.26237790	0.22046637
C	2.68123774	0.41120816	-1.15794384
N	-1.92703493	0.59834177	0.36040965
C	-2.87838073	-0.38466216	0.62791825
H	3.61413908	0.71962199	-1.55766980
C	-1.50290483	-1.17613959	-1.19412083
C	-2.94390109	-1.22579615	-0.65057765
H	-3.61858755	-0.81397914	-1.38938102
H	-3.27307935	-2.24056783	-0.46880099
H	-2.60114353	-1.15366451	1.36644505
H	-3.85120102	0.00711726	0.95272690
H	-1.44420121	-1.33685371	-2.25385333
H	1.02459000	1.05745383	-2.34423898
C	-1.57862528	1.44570595	1.47170536
H	-1.03305885	2.31184718	1.12100581
H	-2.51100056	1.81317432	1.82824216
H	-1.09941698	0.93258155	2.27088574
H	1.17101951	-0.73639076	1.57092428
C	3.71794128	0.00347865	1.32590655
H	4.02434393	-0.86766358	1.90636503
H	4.63170497	0.48684541	0.95760191
H	3.22863680	0.68761021	2.00591192
H	-0.87595191	-1.91934352	-0.72790097

Time = 225.50 fs
E(CASSCF/6-31G(d)) = -479.5295420

C	-0.87737461	0.13266754	-0.57586749
C	0.53166328	0.24816694	-0.39787426
C	1.42781517	0.58340007	-1.49722065
C	1.40356397	-0.28930890	0.61841943
C	2.68081358	-0.26029932	0.22810129
C	2.68056487	0.40819642	-1.15758932
N	-1.92371034	0.59876125	0.36202041
C	-2.87809265	-0.38372051	0.62741015
H	3.60777870	0.71297023	-1.55752034
C	-1.50479300	-1.17260257	-1.19839079
C	-2.94270315	-1.22293212	-0.65233164
H	-3.61783058	-0.80474085	-1.38888716
H	-3.27783490	-2.23558722	-0.46602562
H	-2.60555658	-1.15690973	1.37304623
H	-3.85085119	0.00418305	0.95639846
H	-1.43824794	-1.33830705	-2.26235879
H	1.04460861	1.04734847	-2.35827804

C	-1.57861215	1.43782767	1.47818336
H	-1.03661315	2.30918703	1.13638050
H	-2.51021475	1.80155471	1.84693305
H	-1.09325085	0.92336275	2.27827553
H	1.17516571	-0.72462471	1.57427702
C	3.71713365	0.00965912	1.32243437
H	4.02269778	-0.85756366	1.91109883
H	4.62740691	0.48419633	0.93517712
H	3.23115939	0.70555953	1.99420175
H	-0.88090281	-1.92269955	-0.72637176

Time = 226.00 fs
E(CASSCF/6-31G(d)) = -479.5296432

C	-0.88012156	0.13718266	-0.58105430
C	0.53443758	0.24401812	-0.40360617
C	1.42875983	0.57485602	-1.50078808
C	1.39960399	-0.28659645	0.62610442
C	2.67980136	-0.25781180	0.23556213
C	2.68038867	0.40490530	-1.15664759
N	-1.91982719	0.59919306	0.36349347
C	-2.87807166	-0.38330509	0.62691070
H	3.60218544	0.70692761	-1.55958244
C	-1.50655425	-1.16888910	-1.20255568
C	-2.94151564	-1.21999324	-0.65423957
H	-3.61723610	-0.79503225	-1.38835014
H	-3.28243336	-2.23068004	-0.46311546
H	-2.60921011	-1.15817911	1.38054464
H	-3.84948860	0.00159552	0.95929636
H	-1.43215789	-1.33892206	-2.27129617
H	1.06431704	1.03808438	-2.37447860
C	-1.57870850	1.43004046	1.48449599
H	-1.03919681	2.30612332	1.15203753
H	-2.51069370	1.79078296	1.86685205
H	-1.08564124	0.91362875	2.28671957
H	1.17767596	-0.71199120	1.57780022
C	3.71642425	0.01576143	1.31888407
H	4.02079125	-0.84741052	1.91521242
H	4.62227089	0.48096176	0.91292078
H	3.23474207	0.72369396	1.98293689
H	-0.88652895	-1.92708091	-0.72519809

Time = 226.50 fs
E(CASSCF/6-31G(d)) = -479.5302978

C	-0.88331444	0.14159185	-0.58601964
C	0.53706655	0.23968327	-0.40941270

C	1.42925230	0.56636297	-1.50455681
C	1.39534995	-0.28385583	0.63395169
C	2.67905458	-0.25492015	0.24281698
C	2.68068720	0.40131465	-1.15508500
N	-1.91542204	0.59962474	0.36483900
C	-2.87828790	-0.38342700	0.62643737
H	3.59776399	0.70158785	-1.56398192
C	-1.50816233	-1.16504091	-1.20662609
C	-2.94036567	-1.21697377	-0.65630147
H	-3.61677864	-0.78495666	-1.38770391
H	-3.28681212	-2.22580908	-0.46020687
H	-2.61210505	-1.15727335	1.38858788
H	-3.84719611	-0.00053029	0.96135652
H	-1.42604956	-1.33861174	-2.28018076
H	1.08360473	1.02946695	-2.39247001
C	-1.57889952	1.42234817	1.49069935
H	-1.04081505	2.30270051	1.16792570
H	-2.51217618	1.78067382	1.88774636
H	-1.07683007	0.90353783	2.29579107
H	1.17825373	-0.69879476	1.58215953
C	3.71582098	0.02179313	1.31527073
H	4.01863777	-0.83713073	1.91870300
H	4.61642385	0.47717854	0.89098805
H	3.23950347	0.74195522	1.97211575
H	-0.89297611	-1.93208392	-0.72458070

Time = 227.00 fs
E(CASSCF/6-31G(d)) = -479.5313491

C	-0.88689736	0.14588574	-0.59075874
C	0.53948979	0.23516283	-0.41526465
C	1.42932045	0.55793923	-1.50856610
C	1.39085966	-0.28105079	0.64185383
C	2.67851477	-0.25163199	0.24984013
C	2.68143735	0.39741409	-1.15288382
N	-1.91053809	0.60004136	0.36606911
C	-2.87870286	-0.38408587	0.62600198
H	3.59475374	0.69697719	-1.57074258
C	-1.50959519	-1.16109868	-1.21061296
C	-2.93928033	-1.21386996	-0.65851575
H	-3.61643078	-0.77462231	-1.38688012
H	-3.29091068	-2.22093280	-0.45744516
H	-2.61424262	-1.15407589	1.39685868
H	-3.84412266	-0.00206950	0.96254063
H	-1.42004315	-1.33729265	-2.28854747
H	1.10241052	1.02122377	-2.41172398
C	-1.57917081	1.41475408	1.49685843

H	-1.04149432	2.29895882	1.18399547
H	-2.51429588	1.77100141	1.90930223
H	-1.06712292	0.89329139	2.30500220
H	1.17663929	-0.68532488	1.58799222
C	3.71532959	0.02776413	1.31161068
H	4.01626377	-0.82668142	1.92158640
H	4.61004156	0.47290085	0.86951275
H	3.24554872	0.76027165	1.96172320
H	-0.90035543	-1.93731560	-0.72470620

Time = 227.50 fs
E(CASSCF/6-31G(d)) = -479.5326785

C	-0.89081026	0.15005483	-0.59526738
C	0.54164707	0.23045890	-0.42113371
C	1.42900322	0.54960395	-1.51285773
C	1.38619138	-0.27814876	0.64970950
C	2.67811829	-0.24795756	0.25661188
C	2.68261782	0.39320359	-1.15004401
N	-1.90522411	0.60042606	0.36719749
C	-2.87927109	-0.38526961	0.62561044
H	3.59319675	0.69304502	-1.57976855
C	-1.51083669	-1.15709954	-1.21452577
C	-2.93828578	-1.21067988	-0.66087916
H	-3.61616800	-0.76413740	-1.38581469
H	-3.29467373	-2.21601257	-0.45498033
H	-2.61562372	-1.14855953	1.40508599
H	-3.84047309	-0.00289256	0.96283437
H	-1.41425645	-1.33489162	-2.29598864
H	1.12069767	1.01303635	-2.43160922
C	-1.57950709	1.40726190	1.50304145
H	-1.04128269	2.29493099	1.20020043
H	-2.51662829	1.76152027	1.93116434
H	-1.05685947	0.88310461	2.31385309
H	1.17263097	-0.67181914	1.59582157
C	3.71495449	0.03368663	1.30792086
H	4.01370829	-0.81605235	1.92389844
H	4.60334072	0.46819597	0.84860604
H	3.25296435	0.77856357	1.95173491
H	-0.90872869	-1.94241626	-0.72573257

Time = 228.00 fs
E(CASSCF/6-31G(d)) = -479.5342144

C	-0.89498988	0.15408954	-0.59954184
C	0.54347961	0.22557543	-0.42699312
C	1.42835112	0.54137452	-1.51747046

C	1.38140057	-0.27512376	0.65743163
C	2.67779825	-0.24390941	0.26311847
C	2.68421018	0.38869334	-1.14658388
N	-1.89953337	0.60076066	0.36823904
C	-2.87994224	-0.38695459	0.62526269
H	3.59293572	0.68966414	-1.59083783
C	-1.51187787	-1.15307519	-1.21837119
C	-2.93740624	-1.20740309	-0.66338667
H	-3.61597310	-0.75360520	-1.38445371
H	-3.29805398	-2.21101967	-0.45296278
H	-2.61624891	-1.14078852	1.41305326
H	-3.83649301	-0.00287162	0.96224385
H	-1.40880070	-1.33135115	-2.30218296
H	1.13843125	1.00457426	-2.45145467
C	-1.57989094	1.39987643	1.50931442
H	-1.04024924	2.29064041	1.21649800
H	-2.51873843	1.75198598	1.95295788
H	-1.04638542	0.87318039	2.32187493
H	1.16610476	-0.65843042	1.60597290
C	3.71469962	0.03957478	1.30421819
H	4.01102170	-0.80526569	1.92569488
H	4.59656831	0.46313903	0.82835679
H	3.26181383	0.79674907	1.94212173
H	-0.91809889	-1.94707744	-0.72777832

Time = 228.50 fs
E(CASSCF/6-31G(d)) = -479.5359061

C	-0.89937071	0.15798011	-0.60357887
C	0.54493146	0.22051844	-0.43281842
C	1.42742584	0.53326402	-1.52243484
C	1.37653717	-0.27195832	0.66495382
C	2.67748644	-0.23950203	0.26935174
C	2.68619754	0.38390262	-1.14253956
N	-1.89352259	0.60102635	0.36920961
C	-2.88066353	-0.38910593	0.62495316
H	3.59364274	0.68664067	-1.60360882
C	-1.51271778	-1.14905043	-1.22215225
C	-2.93666320	-1.20404046	-0.66603157
H	-3.61583988	-0.74311944	-1.38275808
H	-3.30101491	-2.20594102	-0.45153932
H	-2.61611902	-1.13091687	1.42060430
H	-3.83244893	-0.00188867	0.96079024
H	-1.40377667	-1.32663356	-2.30691542
H	1.15556160	0.99552639	-2.47061000
C	-1.58030165	1.39260371	1.51573659
H	-1.03848264	2.28610106	1.23284963

H	-2.52022327	1.74217268	1.97431104
H	-1.03602828	0.86368903	2.32866347
H	1.15702892	-0.64520687	1.61850997
C	3.71456953	0.04544422	1.30051894
H	4.00826328	-0.79437325	1.92705006
H	4.58998884	0.45780656	0.80883362
H	3.27213473	0.81474988	1.93285346
H	-0.92840596	-1.95105504	-0.73091502

Time = 229.00 fs
E(CASSCF/6-31G(d)) = -479.5377117

C	-0.90388611	0.16171672	-0.60737558
C	0.54595109	0.21529604	-0.43858807
C	1.42629868	0.52527964	-1.52776878
C	1.37164347	-0.26864387	0.67223345
C	2.67711508	-0.23475151	0.27530892
C	2.68856178	0.37885798	-1.13796318
N	-1.88725057	0.60120423	0.37012564
C	-2.88138269	-0.39167801	0.62467125
H	3.59487240	0.68373235	-1.61764013
C	-1.51336375	-1.14504223	-1.22586775
C	-2.93607475	-1.20059396	-0.66880556
H	-3.61577560	-0.73276072	-1.38070739
H	-3.30353282	-2.20078363	-0.45084901
H	-2.61523547	-1.11918373	1.42764729
H	-3.82860474	0.00015543	0.95850384
H	-1.39927153	-1.32072344	-2.31008953
H	1.17201517	0.98562482	-2.48849732
C	-1.58071456	1.38545092	1.52235703
H	-1.03608795	2.28131893	1.24921973
H	-2.52074504	1.73188715	1.99487480
H	-1.02607911	0.85475463	2.33390141
H	1.14547138	-0.63208915	1.63320927
C	3.71457043	0.05131158	1.29683821
H	4.00549891	-0.78345161	1.92805317
H	4.58386969	0.45227096	0.79008944
H	3.28393579	0.83249581	1.92390260
H	-0.93952613	-1.95417818	-0.73516327

Time = 229.50 fs
E(CASSCF/6-31G(d)) = -479.5395479

C	-0.90846918	0.16528938	-0.61092937
C	0.54649263	0.20991852	-0.44428400
C	1.42504828	0.51742142	-1.53347438
C	1.36675308	-0.26517945	0.67925079

C	2.67661848	-0.22967507	0.28099233
C	2.69128002	0.37359104	-1.13292006
N	-1.88077715	0.60127592	0.37100392
C	-2.88205074	-0.39461510	0.62440212
H	3.59612578	0.68067069	-1.63241929
C	-1.51383143	-1.14105916	-1.22951217
C	-2.93565501	-1.19706658	-0.67169887
H	-3.61580223	-0.72259349	-1.37830167
H	-3.30559828	-2.19557689	-0.45101990
H	-2.61360131	-1.10590545	1.43415611
H	-3.82519628	0.00333081	0.95541692
H	-1.39535626	-1.31362892	-2.31173240
H	1.18769217	0.97466193	-2.50464965
C	-1.58110084	1.37842598	1.52921225
H	-1.03318306	2.27629528	1.26557469
H	-2.52005508	1.72097803	2.01434024
H	-1.01677854	0.84644662	2.33737207
H	1.13159706	-0.61892783	1.64957997
C	3.71471117	0.05719371	1.29318938
H	4.00279734	-0.77259508	1.92880385
H	4.57846616	0.44659409	0.77216645
H	3.29719675	0.84992897	1.91524669
H	-0.95127512	-1.95635444	-0.74049135

Time = 230.00 fs
E(CASSCF/6-31G(d)) = -479.5413058

C	-0.91305366	0.16868798	-0.61423793
C	0.54651715	0.20439838	-0.44989197
C	1.42375779	0.50968176	-1.53953605
C	1.36189077	-0.26156926	0.68600472
C	2.67593443	-0.22429053	0.28640894
C	2.69432145	0.36813613	-1.12748536
N	-1.87416220	0.60122409	0.37186140
C	-2.88262470	-0.39785221	0.62412767
H	3.59691362	0.67718370	-1.64739620
C	-1.51414432	-1.13710130	-1.23307576
C	-2.93541391	-1.19346219	-0.67470055
H	-3.61595634	-0.71266420	-1.37556193
H	-3.30721666	-2.19037271	-0.45216633
H	-2.61122271	-1.09146390	1.44016841
H	-3.82240699	0.00766853	0.95155754
H	-1.39208391	-1.30538145	-2.31199346
H	1.20246877	0.96250035	-2.51873747
C	-1.58142766	1.37153695	1.53632462
H	-1.02989431	2.27103100	1.28188159
H	-2.51800899	1.70934150	2.03245224

H	-1.00830705	0.83877562	2.33896584
H	1.11565620	-0.60551454	1.66692426
C	3.71500405	0.06310703	1.28958390
H	4.00022637	-0.76190707	1.92940629
H	4.57400698	0.44082198	0.75510219
H	3.31186917	0.86700655	1.90686979
H	-0.96341424	-1.95757137	-0.74681675

Time = 230.50 fs
E(CASSCF/6-31G(d)) = -479.5428729

C	-0.91757477	0.17190222	-0.61729912
C	0.54599384	0.19875015	-0.45540161
C	1.42251176	0.50204546	-1.54591948
C	1.35707295	-0.25781973	0.69250600
C	2.67500550	-0.21861588	0.29157002
C	2.69764519	0.36252824	-1.12174055
N	-1.86746451	0.60103283	0.37271499
C	-2.88306976	-0.40131631	0.62382762
H	3.59680908	0.67301578	-1.66201700
C	-1.51433301	-1.13316045	-1.23654502
C	-2.93535706	-1.18978564	-0.67779875
H	-3.61628759	-0.70300076	-1.37252934
H	-3.30840782	-2.18524351	-0.45438664
H	-2.60811095	-1.07629126	1.44577923
H	-3.82034774	0.01315192	0.94694533
H	-1.38948881	-1.29603442	-2.31113668
H	1.21620226	0.94907694	-2.53058540
C	-1.58165876	1.36479157	1.54370158
H	-1.02635134	2.26553115	1.29810664
H	-2.51457477	1.69692410	2.04901982
H	-1.00077861	0.83169248	2.33868125
H	1.09796634	-0.59161805	1.68442189
C	3.71546541	0.06906661	1.28603106
H	3.99784892	-0.75149073	1.92996227
H	4.57068193	0.43498176	0.73893559
H	3.32787790	0.88370193	1.89876346
H	-0.97565895	-1.95789385	-0.75401047

Time = 231.00 fs
E(CASSCF/6-31G(d)) = -479.5441212

C	-0.92196995	0.17492168	-0.62011103
C	0.54490061	0.19299039	-0.46080661
C	1.42139333	0.49448965	-1.55257190
C	1.35230895	-0.25393660	0.69877059
C	2.67378011	-0.21266876	0.29649077

C	2.70119920	0.35680127	-1.11577010
N	-1.86074129	0.60068825	0.37358148
C	-2.88336035	-0.40492830	0.62348104
H	3.59548627	0.66794379	-1.67575348
C	-1.51443407	-1.12922109	-1.23990349
C	-2.93548591	-1.18604276	-0.68098102
H	-3.61685573	-0.69361269	-1.36926202
H	-3.30920485	-2.18027823	-0.45776241
H	-2.60428682	-1.06084895	1.45112788
H	-3.81904473	0.01971112	0.94158896
H	-1.38758683	-1.28566005	-2.30952517
H	1.22873813	0.93440475	-2.54017956
C	-1.58175524	1.35819653	1.55133534
H	-1.02268270	2.25981058	1.31421523
H	-2.50983550	1.68372210	2.06392404
H	-0.99423596	0.82508879	2.33662295
H	1.07888918	-0.57702033	1.70121957
C	3.71611569	0.07508548	1.28253816
H	3.99571809	-0.74143861	1.93056449
H	4.56863213	0.42907800	0.72371048
H	3.34512609	0.90000504	1.89092503
H	-0.98769163	-1.95745728	-0.76190469

Time = 231.50 fs
E(CASSCF/6-31G(d)) = -479.5449952

C	-0.92617969	0.17773586	-0.62267191
C	0.54322498	0.18713726	-0.46610385
C	1.42048135	0.48698496	-1.55942260
C	1.34760197	-0.24992307	0.70481278
C	2.67221339	-0.20646608	0.30118988
C	2.70492053	0.35098668	-1.10965842
N	-1.85404704	0.60017839	0.37447733
C	-2.88348055	-0.40860513	0.62306764
H	3.59274368	0.66178747	-1.68812971
C	-1.51448854	-1.12526160	-1.24313290
C	-2.93579816	-1.18224053	-0.68423475
H	-3.61772665	-0.68449345	-1.36583245
H	-3.30965191	-2.17557616	-0.46235694
H	-2.59978436	-1.04560555	1.45638024
H	-3.81843625	0.02722111	0.93548886
H	-1.38637716	-1.27434556	-2.30759789
H	1.23991709	0.91856756	-2.54767005
C	-1.58167651	1.35175719	1.55920303
H	-1.01901037	2.25389680	1.33017039
H	-2.50398736	1.66978057	2.07712240
H	-0.98864990	0.81880032	2.33299560

H	1.05880970	-0.56153960	1.71651062
C	3.71697932	0.08117373	1.27911068
H	3.99387480	-0.73182423	1.93128935
H	4.56794465	0.42309522	0.70947960
H	3.36349704	0.91591989	1.88335776
H	-0.99917496	-1.95645472	-0.77030331

Time = 232.00 fs
E(CASSCF/6-31G(d)) = -479.5454270

C	-0.93014817	0.18033423	-0.62498024
C	0.54096429	0.18121034	-0.47129334
C	1.41984803	0.47949604	-1.56638477
C	1.34295046	-0.24577839	0.71064096
C	2.67026796	-0.20002355	0.30568907
C	2.70873625	0.34511249	-1.10348718
N	-1.84743321	0.59949367	0.37541867
C	-2.88342299	-0.41226287	0.62256970
H	3.58851620	0.65441703	-1.69874189
C	-1.51453997	-1.12125632	-1.24621491
C	-2.93628834	-1.17838708	-0.68754753
H	-3.61896708	-0.67562308	-1.36232210
H	-3.30980143	-2.17123971	-0.46821578
H	-2.59465793	-1.03100987	1.46170348
H	-3.81838047	0.03550550	0.92864115
H	-1.38584518	-1.26218762	-2.30583655
H	1.24958278	0.90171487	-2.55336338
C	-1.58138134	1.34547713	1.56726744
H	-1.01544600	2.24783367	1.34593361
H	-2.49733218	1.65518853	2.08865029
H	-0.98392010	0.81261177	2.32809504
H	1.03811428	-0.54504855	1.72959514
C	3.71808395	0.08733794	1.27575280
H	3.99234405	-0.72269448	1.93219183
H	4.56865111	0.41699860	0.69630379
H	3.38286027	0.93146214	1.87606770
H	-1.00977016	-1.95511871	-0.77899634

Time = 232.50 fs
E(CASSCF/6-31G(d)) = -479.5454154

C	-0.93382393	0.18270638	-0.62703475
C	0.53812585	0.17523023	-0.47637721
C	1.41955702	0.47198305	-1.57335819
C	1.33834916	-0.24149732	0.71625419
C	2.66791448	-0.19335545	0.31001276
C	2.71256493	0.33920268	-1.09733311

N	-1.84094764	0.59862692	0.37642122
C	-2.88318704	-0.41581993	0.62197374
H	3.58287795	0.64575664	-1.70727423
C	-1.51463230	-1.11717812	-1.24913323
C	-2.93694854	-1.17449188	-0.69090760
H	-3.62063876	-0.66697214	-1.35881632
H	-3.30971073	-2.16736588	-0.47536689
H	-2.58898862	-1.01746573	1.46723828
H	-3.81867164	0.04434479	0.92104485
H	-1.38596689	-1.24928642	-2.30472423
H	1.25758949	0.88405063	-2.55770652
C	-1.58082901	1.33935789	1.57547846
H	-1.01208720	2.24168246	1.36146536
H	-2.49026408	1.64007263	2.09861810
H	-0.97988076	0.80626498	2.32229448
H	1.01717345	-0.52748089	1.73992130
C	3.71945935	0.09358075	1.27246796
H	3.99113349	-0.71406491	1.93330184
H	4.57073058	0.41073838	0.68424972
H	3.40307595	0.94665489	1.86906135
H	-1.01915609	-1.95369689	-0.78777680

Time = 233.00 fs
E(CASSCF/6-31G(d)) = -479.5449921

C	-0.93716041	0.18484210	-0.62883450
C	0.53472678	0.16921825	-0.48135898
C	1.41966187	0.46440337	-1.58023304
C	1.33379020	-0.23707027	0.72164057
C	2.66513196	-0.18647430	0.31418756
C	2.71631842	0.33327669	-1.09126599
N	-1.83463417	0.59757333	0.37750016
C	-2.88277645	-0.41920022	0.62127218
H	3.57603811	0.63578526	-1.71351087
C	-1.51480744	-1.11300122	-1.25187607
C	-2.93776927	-1.17056569	-0.69430424
H	-3.62279220	-0.65850651	-1.35539896
H	-3.30943835	-2.16403747	-0.48382112
H	-2.58289027	-1.00531051	1.47307138
H	-3.81906252	0.05348874	0.91270969
H	-1.38671427	-1.23573985	-2.30469802
H	1.26380997	0.86581699	-2.56125757
C	-1.57998055	1.33339881	1.58377544
H	-1.00901486	2.23552146	1.37672608
H	-2.48324836	1.62458810	2.10720298
H	-0.97631190	0.79947137	2.31602551
H	0.99632708	-0.50883173	1.74711021

C	3.72113609	0.09990071	1.26925941
H	3.99023303	-0.70591804	1.93462297
H	4.57411563	0.40425521	0.67338538
H	3.42399925	0.96152369	1.86234285
H	-1.02704954	-1.95242357	-0.79645925

Time = 233.50 fs
E(CASSCF/6-31G(d)) = -479.5442348

C	-0.94011645	0.18673158	-0.63037901
C	0.53079368	0.16319601	-0.48624254
C	1.42020495	0.45671387	-1.58689531
C	1.32926403	-0.23248372	0.72677642
C	2.66190800	-0.17939080	0.31824193
C	2.71990365	0.32734931	-1.08534710
N	-1.82853233	0.59633046	0.37867015
C	-2.88219676	-0.42233601	0.62046438
H	3.56833266	0.62453554	-1.71734369
C	-1.51510302	-1.10870416	-1.25443867
C	-2.93874024	-1.16662063	-0.69772814
H	-3.62546079	-0.65019219	-1.35214697
H	-3.30904052	-2.16131478	-0.49357301
H	-2.57651344	-0.99479991	1.47921346
H	-3.81928966	0.06267149	0.90366350
H	-1.38806074	-1.22163865	-2.30610105
H	1.26814345	0.84727286	-2.56464067
C	-1.57880010	1.32759673	1.59209063
H	-1.00629144	2.22944387	1.39167770
H	-2.47679046	1.60890640	2.11463383
H	-0.97295734	0.79192956	2.30975218
H	0.97587220	-0.48915389	1.75096849
C	3.72314391	0.10629243	1.26613077
H	3.98961574	-0.69820501	1.93613398
H	4.57870118	0.39748566	0.66377359
H	3.44548459	0.97609163	1.85591112
H	-1.03322452	-1.95148985	-0.80489778

Time = 234.00 fs
E(CASSCF/6-31G(d)) = -479.5432592

C	-0.94265685	0.18836555	-0.63166832
C	0.52636206	0.15718513	-0.49103134
C	1.42121661	0.44887353	-1.59323339
C	1.32476050	-0.22772083	0.73162623
C	2.65823869	-0.17211372	0.32220572
C	2.72322458	0.32143066	-1.07962806
N	-1.82267712	0.59489812	0.37994518

C	-2.88145307	-0.42516998	0.61955702
H	3.56021093	0.61209026	-1.71877576
C	-1.51555037	-1.10427238	-1.25682545
C	-2.93985123	-1.16267004	-0.70117170
H	-3.62865566	-0.64199999	-1.34912592
H	-3.30856797	-2.15922862	-0.50460118
H	-2.57004554	-0.98610185	1.48558701
H	-3.81909826	0.07162764	0.89395799
H	-1.38998593	-1.20706379	-2.30914138
H	1.27052415	0.82866709	-2.56848439
C	-1.57725640	1.32194570	1.60035378
H	-1.00396019	2.22355415	1.40628512
H	-2.47139502	1.59320061	2.12116923
H	-0.96955026	0.78334876	2.30393890
H	0.95605255	-0.46855139	1.75149298
C	3.72551013	0.11274701	1.26308646
H	3.98923995	-0.69085010	1.93779275
H	4.58435452	0.39036792	0.65546501
H	3.46738895	0.99037471	1.84975751
H	-1.03752845	-1.95101655	-0.81300089

Time = 234.50 fs
E(CASSCF/6-31G(d)) = -479.5422067

C	-0.94475275	0.18973544	-0.63270309
C	0.52147583	0.15120692	-0.49572756
C	1.42271471	0.44084620	-1.59914585
C	1.32026981	-0.22276216	0.73614315
C	2.65412846	-0.16464994	0.32610981
C	2.72618444	0.31552649	-1.07415019
N	-1.81709878	0.59327822	0.38133852
C	-2.88054833	-0.42765657	0.61856352
H	3.55221707	0.59857640	-1.71791860
C	-1.51617318	-1.09969989	-1.25905134
C	-2.94109265	-1.15872831	-0.70462928
H	-3.63236195	-0.63390929	-1.34638639
H	-3.30806363	-2.15777531	-0.51686891
H	-2.56370653	-0.97929890	1.49202678
H	-3.81826443	0.08010793	0.88367233
H	-1.39247846	-1.19208616	-2.31386542
H	1.27092988	0.81020972	-2.57334773
C	-1.57532441	1.31643645	1.60849771
H	-1.00204549	2.21796313	1.42051800
H	-2.46751619	1.57762893	2.12706918
H	-0.96584453	0.77347682	2.29901393
H	0.93704965	-0.44717195	1.74886911
C	3.72825805	0.11925269	1.26013187

H	3.98905209	-0.68375777	1.93954247
H	4.59092611	0.38284633	0.64849087
H	3.48957483	1.00437833	1.84386392
H	-1.03989296	-1.95103453	-0.82074073

Time = 235.00 fs
E(CASSCF/6-31G(d)) = -479.5412166

C	-0.94638208	0.19083357	-0.63348461
C	0.51618658	0.14528207	-0.50033145
C	1.42470413	0.43260315	-1.60454910
C	1.31578337	-0.21758650	0.74026980
C	2.64958980	-0.15700456	0.32998571
C	2.72868823	0.30963879	-1.06894461
N	-1.81182261	0.59147456	0.38286257
C	-2.87948245	-0.42976240	0.61750246
H	3.54496401	0.58415578	-1.71498159
C	-1.51698695	-1.09498985	-1.26114205
C	-2.94245600	-1.15481060	-0.70809728
H	-3.63653690	-0.62591086	-1.34396207
H	-3.30756094	-2.15691423	-0.53032415
H	-2.55773975	-0.97439871	1.49829272
H	-3.81661321	0.08789312	0.87291462
H	-1.39553652	-1.17676888	-2.32015112
H	1.26939010	0.79204454	-2.57964372
C	-1.57298684	1.31105580	1.61646403
H	-1.00055399	2.21278185	1.43435217
H	-2.46550507	1.56231940	2.13256398
H	-0.96164871	0.76213039	2.29533269
H	0.91897592	-0.42519723	1.74346421
C	3.73140559	0.12579561	1.25727340
H	3.98899048	-0.67682107	1.94131902
H	4.59825964	0.37487537	0.64285686
H	3.51191202	1.01809501	1.83820204
H	-1.04033789	-1.95147600	-0.82815505

Time = 235.50 fs
E(CASSCF/6-31G(d)) = -479.5403818

C	-0.94752990	0.19165325	-0.63401487
C	0.51055291	0.13943057	-0.50484090
C	1.42717673	0.42412496	-1.60938390
C	1.31129466	-0.21217191	0.74393990
C	2.64464272	-0.14918099	0.33386502
C	2.73064609	0.30376663	-1.06403323
N	-1.80686886	0.58949265	0.38452871
C	-2.87825198	-0.43146604	0.61639543

H	3.53909725	0.56901194	-1.71025089
C	-1.51799925	-1.09015385	-1.26313305
C	-2.94393404	-1.15093248	-0.71157412
H	-3.64111016	-0.61800805	-1.34186956
H	-3.30708355	-2.15656857	-0.54489918
H	-2.55239952	-0.97134941	1.50409290
H	-3.81403206	0.09480558	0.86182112
H	-1.39916585	-1.16117133	-2.32772326
H	1.26599340	0.77423002	-2.58757558
C	-1.57023553	1.30578600	1.62420818
H	-0.99947668	2.20811469	1.44777083
H	-2.46556188	1.54735837	2.13782561
H	-0.95685643	0.74922254	2.29314727
H	0.90187056	-0.40283230	1.73581278
C	3.73496421	0.13236064	1.25451833
H	3.98898903	-0.66993064	1.94305878
H	4.60620097	0.36642255	0.63853839
H	3.53427856	1.03150391	1.83273368
H	-1.03896788	-1.95217907	-0.83534120

Time = 236.00 fs
E(CASSCF/6-31G(d)) = -479.5397227

C	-0.94818870	0.19218882	-0.63429653
C	0.50463984	0.13367156	-0.50925136
C	1.43011144	0.41540210	-1.61361925
C	1.30679962	-0.20649666	0.74708087
C	2.63931430	-0.14118123	0.33777887
C	2.73197732	0.29790741	-1.05943083
N	-1.80225266	0.58733955	0.38634721
C	-2.87685040	-0.43275738	0.61526450
H	3.53524801	0.55333378	-1.70405597
C	-1.51921072	-1.08521018	-1.26506749
C	-2.94552068	-1.14710948	-0.71506011
H	-3.64598639	-0.61021636	-1.34010975
H	-3.30664598	-2.15662912	-0.56051049
H	-2.54793703	-0.97005678	1.50911177
H	-3.81047973	0.10071805	0.85055363
H	-1.40337600	-1.14535347	-2.33618720
H	1.26089177	0.75673367	-2.59710047
C	-1.56707225	1.30060430	1.63170283
H	-0.99879197	2.20405218	1.46076572
H	-2.46770261	1.53278486	2.14294729
H	-0.95146692	0.73478245	2.29258742
H	0.88570030	-0.38029415	1.72659041
C	3.73893809	0.13893213	1.25187446
H	3.98898074	-0.66298342	1.94470643

H	4.61460555	0.35746950	0.63547735
H	3.55656176	1.04457224	1.82741188
H	-1.03596238	-1.95290464	-0.84244390

Time = 236.50 fs
E(CASSCF/6-31G(d)) = -479.5391925

C	-0.94835860	0.19243583	-0.63433284
C	0.49851774	0.12802325	-0.51355562
C	1.43347472	0.40643449	-1.61725287
C	1.30229631	-0.20054069	0.74961736
C	2.63363806	-0.13300608	0.34175726
C	2.73261517	0.29205837	-1.05514793
N	-1.79798390	0.58502355	0.38832712
C	-2.87526877	-0.43363668	0.61412978
H	3.53397862	0.53729675	-1.69672809
C	-1.52061650	-1.08018138	-1.26699351
C	-2.94721073	-1.14335652	-0.71855724
H	-3.65104978	-0.60256184	-1.33867113
H	-3.30625506	-2.15696064	-0.57705778
H	-2.54458554	-0.97040103	1.51303881
H	-3.80599026	0.10555924	0.83929580
H	-1.40817548	-1.12937954	-2.34507350
H	1.25430016	0.73943935	-2.60793174
C	-1.56350891	1.29548288	1.63893881
H	-0.99846881	2.20066349	1.47333693
H	-2.47174820	1.51859339	2.14793596
H	-0.94559073	0.71896265	2.29365689
H	0.87036753	-0.35779635	1.71657387
C	3.74332377	0.14549449	1.24934976
H	3.98890154	-0.65589038	1.94622147
H	4.62334419	0.34801360	0.63358171
H	3.57865736	1.05725815	1.82218401
H	-1.03156050	-1.95336114	-0.84963818

Time = 237.00 fs
E(CASSCF/6-31G(d)) = -479.5386864

C	-0.94804754	0.19239104	-0.63412750
C	0.49226200	0.12250293	-0.51774428
C	1.43722144	0.39722946	-1.62030808
C	1.29778389	-0.19428646	0.75147667
C	2.62765332	-0.12465542	0.34582842
C	2.73251197	0.28621800	-1.05119442
N	-1.79406716	0.58255404	0.39047606
C	-2.87349678	-0.43411363	0.61300734
H	3.53572117	0.52104490	-1.68855037

C	-1.52220804	-1.07509157	-1.26896135
C	-2.94899937	-1.13968735	-0.72206883
H	-3.65617013	-0.59507777	-1.33753321
H	-3.30591243	-2.15741058	-0.59442442
H	-2.54254807	-0.97225117	1.51559517
H	-3.80067315	0.10931655	0.82824774
H	-1.41356741	-1.11331976	-2.35388770
H	1.24649121	0.72216977	-2.61957816
C	-1.55956685	1.29038953	1.64592357
H	-0.99847106	2.19798978	1.48549362
H	-2.47733914	1.50474247	2.15271953
H	-0.93943849	0.70203129	2.29624584
H	0.85572861	-0.33553657	1.70658180
C	3.74810994	0.15203272	1.24695190
H	3.98869240	-0.64858243	1.94758402
H	4.63230642	0.33806621	0.63272617
H	3.60047059	1.06951520	1.81699381
H	-1.02604322	-1.95323557	-0.85711168

Time = 237.50 fs
E(CASSCF/6-31G(d)) = -479.5381013

C	-0.94727139	0.19205249	-0.63368450
C	0.48595211	0.11712700	-0.52180601
C	1.44129620	0.38779933	-1.62282842
C	1.29326054	-0.18772018	0.75259511
C	2.62140459	-0.11612831	0.35001784
C	2.73164400	0.28038709	-1.04758253
N	-1.79050177	0.57994128	0.39280023
C	-2.87152367	-0.43420618	0.61190771
H	3.54072087	0.50467652	-1.67971350
C	-1.52397473	-1.06996387	-1.27102053
C	-2.95088159	-1.13611398	-0.72559914
H	-3.66121056	-0.58780033	-1.33667154
H	-3.30561720	-2.15781943	-0.61247753
H	-2.54198706	-0.97547668	1.51655464
H	-3.79470819	0.11203528	0.81762002
H	-1.41954615	-1.09725040	-2.36215671
H	1.23778480	0.70471451	-2.63141056
C	-1.55527545	1.28528879	1.65267739
H	-0.99876141	2.19603805	1.49725337
H	-2.48397307	1.49116863	2.15716619
H	-0.93329586	0.68435126	2.30015548
H	0.84162255	-0.31368335	1.69740175
C	3.75327771	0.15853274	1.24468774
H	3.98830141	-0.64101472	1.94879838
H	4.64140234	0.32765159	0.63275506

H	3.62191608	1.08129747	1.81178495
H	-1.01971488	-1.95222391	-0.86504754

Time = 238.00 fs
E(CASSCF/6-31G(d)) = -479.5373731

C	-0.94605376	0.19141952	-0.63300805
C	0.47967086	0.11191093	-0.52572798
C	1.44563491	0.37815886	-1.62487093
C	1.28872068	-0.18083253	0.75292510
C	2.61494095	-0.10742331	0.35434752
C	2.73001492	0.27456929	-1.04432890
N	-1.78728189	0.57719621	0.39530427
C	-2.86933963	-0.43393934	0.61083495
H	3.54899522	0.48823783	-1.67028589
C	-1.52590542	-1.06481827	-1.27321750
C	-2.95285161	-1.13264607	-0.72915293
H	-3.66603496	-0.58076431	-1.33606224
H	-3.30536885	-2.15803129	-0.63106881
H	-2.54301697	-0.97995661	1.51575910
H	-3.78833586	0.11381398	0.80762686
H	-1.42609508	-1.08125214	-2.36946754
H	1.22853265	0.68685884	-2.64274031
C	-1.55067017	1.28014379	1.65922861
H	-0.99930474	2.19477723	1.50864112
H	-2.49105719	1.47780162	2.16111131
H	-0.92748972	0.66634959	2.30512981
H	0.82790872	-0.29236605	1.68970733
C	3.75880113	0.16498152	1.24256290
H	3.98768507	-0.63316857	1.94989486
H	4.65056194	0.31680483	0.63348749
H	3.64291812	1.09256496	1.80650431
H	-1.01288537	-1.95005848	-0.87361001

Time = 238.50 fs
E(CASSCF/6-31G(d)) = -479.5364871

C	-0.94442589	0.19049282	-0.63210235
C	0.47350346	0.10686929	-0.52949624
C	1.45016660	0.36832302	-1.62649963
C	1.28415200	-0.17361901	0.75244171
C	2.60831525	-0.09853869	0.35883499
C	2.72765718	0.26877082	-1.04145454
N	-1.78439657	0.57433023	0.39799119
C	-2.86693726	-0.43334377	0.60978633
H	3.56031786	0.47172604	-1.66020703
C	-1.52798965	-1.05967004	-1.27559377

C	-2.95490230	-1.12929043	-0.73273508
H	-3.67051550	-0.57399877	-1.33568628
H	-3.30516997	-2.15790411	-0.65003612
H	-2.54569981	-0.98558609	1.51312809
H	-3.78184233	0.11479583	0.79847869
H	-1.43318581	-1.06540713	-2.37549697
H	1.21910101	0.66840833	-2.65289454
C	-1.54579044	1.27491835	1.66560866
H	-1.00007045	2.19413640	1.51968761
H	-2.49796565	1.46457814	2.16438560
H	-0.92235228	0.64848262	2.31088816
H	0.81450633	-0.27167061	1.68398082
C	3.76464795	0.17136717	1.24058139
H	3.98680892	-0.62505139	1.95092955
H	4.65973248	0.30556937	0.63472404
H	3.66341117	1.10328872	1.80110501
H	-1.00585423	-1.94653047	-0.88293359

Time = 239.00 fs
E(CASSCF/6-31G(d)) = -479.5354621

C	-0.94242607	0.18927439	-0.63097157
C	0.46753642	0.10201560	-0.53309613
C	1.45481519	0.35830532	-1.62777999
C	1.27953321	-0.16607955	0.75114723
C	2.60158344	-0.08947254	0.36349255
C	2.72463082	0.26299947	-1.03898274
N	-1.78182997	0.57135501	0.40086227
C	-2.86431329	-0.43245420	0.60875257
H	3.57423236	0.45510154	-1.64930655
C	-1.53021866	-1.05452861	-1.27818459
C	-2.95702472	-1.12605057	-0.73635020
H	-3.67453933	-0.56752307	-1.33553307
H	-3.30502851	-2.15731890	-0.66920579
H	-2.55004331	-0.99228091	1.50866304
H	-3.77553908	0.11515610	0.79037438
H	-1.44077861	-1.04979525	-2.38003207
H	1.20985298	0.64920616	-2.66127827
C	-1.54067756	1.26957905	1.67184771
H	-1.00103360	2.19400579	1.53042743
H	-2.50409371	1.45145218	2.16684021
H	-0.91818860	0.63120197	2.31715418
H	0.80142675	-0.25164195	1.68045762
C	3.77078076	0.17767876	1.23874532
H	3.98564747	-0.61669430	1.95198211
H	4.66887414	0.29399421	0.63625469
H	3.68334070	1.11345530	1.79554877

H	-0.99889718	-1.94150657	-0.89311596

Time = 239.50 fs
E(CASSCF/6-31G(d)) = -479.5343176

C	-0.94009908	0.18776753	-0.62961967
C	0.46185628	0.09736252	-0.53651275
C	1.45950137	0.34811675	-1.62877476
C	1.27483346	-0.15821774	0.74907263
C	2.59480354	-0.08022305	0.36832643
C	2.72101981	0.25726315	-1.03693519
N	-1.77956166	0.56828223	0.40391703
C	-2.86147044	-0.43130767	0.60771852
H	3.59009388	0.43830539	-1.63734425
C	-1.53258607	-1.04939705	-1.28101792
C	-2.95920780	-1.12292636	-0.74000228
H	-3.67801405	-0.56134376	-1.33560305
H	-3.30495957	-2.15618715	-0.68839546
H	-2.55600167	-0.99998011	1.50244646
H	-3.76973784	0.11508606	0.78349346
H	-1.44882354	-1.03448994	-2.38298245
H	1.20113211	0.62914355	-2.66741994
C	-1.53537303	1.26409704	1.67797136
H	-1.00217433	2.19424102	1.54089651
H	-2.50890277	1.43840142	2.16836568
H	-0.91525049	0.61492450	2.32367871
H	0.78878872	-0.23229239	1.67910634
C	3.77715809	0.18390616	1.23705468
H	3.98418378	-0.60814789	1.95315145
H	4.67795397	0.28213145	0.63786779
H	3.70266382	1.12306991	1.78980771
H	-0.99225516	-1.93494012	-0.90421410

Time = 240.00 fs
E(CASSCF/6-31G(d)) = -479.5330587

C	-0.93749544	0.18597684	-0.62805045
C	0.45654835	0.09292159	-0.53973108
C	1.46414422	0.33776525	-1.62954087
C	1.27001344	-0.15003960	0.74627531
C	2.58803473	-0.07078861	0.37333633
C	2.71692675	0.25156836	-1.03532750
N	-1.77756682	0.56512335	0.40715315
C	-2.85841942	-0.42994175	0.60666428
H	3.60712974	0.42127798	-1.62406013
C	-1.53508829	-1.04427195	-1.28411386
C	-2.96143825	-1.11991384	-0.74369447

H	-3.68087182	-0.55545229	-1.33590919
H	-3.30498659	-2.15445601	-0.70741744
H	-2.56347853	-1.00864607	1.49463613
H	-3.76472216	0.11477462	0.77798862
H	-1.45726247	-1.01955408	-2.38438539
H	1.19324797	0.60816331	-2.67100144
C	-1.52991713	1.25844944	1.68399846
H	-1.00347604	2.19466951	1.55112962
H	-2.51195461	1.42542998	2.16890445
H	-0.91371773	0.60000790	2.33025616
H	0.77681202	-0.21361284	1.67964964
C	3.78373585	0.19003973	1.23550741
H	3.98240847	-0.59947610	1.95454990
H	4.68693933	0.27003371	0.63935940
H	3.72135025	1.13215840	1.78386492
H	-0.98612563	-1.92687657	-0.91624328

Time = 240.50 fs
E(CASSCF/6-31G(d)) = -479.5316655

C	-0.93467006	0.18390817	-0.62626765
C	0.45169500	0.08870335	-0.54273647
C	1.46866276	0.32725552	-1.63012741
C	1.26502862	-0.14155258	0.74283413
C	2.58133622	-0.06116802	0.37851501
C	2.71246673	0.24591912	-1.03416469
N	-1.77581670	0.56188947	0.41056650
C	-2.85518064	-0.42839292	0.60556671
H	3.62450767	0.40397459	-1.60922402
C	-1.53772474	-1.03914366	-1.28748442
C	-2.96370059	-1.11700514	-0.74742887
H	-3.68307144	-0.54982447	-1.33647733
H	-3.30514136	-2.15211096	-0.72608235
H	-2.57233235	-1.01826303	1.48545453
H	-3.76071954	0.11438955	0.77397940
H	-1.46603126	-1.00503639	-2.38440463
H	1.18646383	0.58625942	-2.67187601
C	-1.52434800	1.25262027	1.68993989
H	-1.00492195	2.19510022	1.56115791
H	-2.51293521	1.41256886	2.16845786
H	-0.91368596	0.58673216	2.33673574
H	0.76579044	-0.19558476	1.68161829
C	3.79046853	0.19606992	1.23409943
H	3.98031905	-0.59074880	1.95629551
H	4.69579074	0.25775268	0.64054238
H	3.73938294	1.14076734	1.77771465
H	-0.98065652	-1.91745452	-0.92917817

Time = 241.00 fs
E(CASSCF/6-31G(d)) = -479.5301097

C	-0.93168108	0.18156845	-0.62427503
C	0.44737396	0.08471726	-0.54551477
C	1.47297783	0.31658908	-1.63057410
C	1.25983372	-0.13276445	0.73884239
C	2.57476577	-0.05136063	0.38384847
C	2.70776112	0.24031614	-1.03343813
N	-1.77427900	0.55859112	0.41415119
C	-2.85178551	-0.42669535	0.60440123
H	3.64140141	0.38637610	-1.59267522
C	-1.54049782	-1.03399687	-1.29113359
C	-2.96597747	-1.11418858	-0.75120646
H	-3.68459911	-0.54442063	-1.33734539
H	-3.30546371	-2.14917576	-0.74420296
H	-2.58238379	-1.02883183	1.47517314
H	-3.75787666	0.11406117	0.77154715
H	-1.47506242	-0.99096764	-2.38332278
H	1.18098631	0.56347387	-2.67007751
C	-1.51870088	1.24660089	1.69579796
H	-1.00649128	2.19533432	1.57100651
H	-2.51166961	1.39987424	2.16708835
H	-0.91516014	0.57528600	2.34302798
H	0.75605197	-0.17818929	1.68442514
C	3.79731051	0.20198692	1.23282500
H	3.97791880	-0.58203349	1.95850357
H	4.70445567	0.24533779	0.64125451
H	3.75675860	1.14896269	1.77136111
H	-0.97594274	-1.90690193	-0.94295636

Time = 241.50 fs
E(CASSCF/6-31G(d)) = -479.5283657

C	-0.92858827	0.17896564	-0.62207650
C	0.44365655	0.08097183	-0.54805300
C	1.47701342	0.30576445	-1.63091016
C	1.25438738	-0.12368278	0.73440098
C	2.56837853	-0.04136665	0.38931589
C	2.70293231	0.23475672	-1.03312345
N	-1.77291847	0.55523811	0.41789969
C	-2.84827683	-0.42488042	0.60314389
H	3.65704621	0.36849410	-1.57434983
C	-1.54341262	-1.02881158	-1.29505787
C	-2.96825011	-1.11144883	-0.75502711
H	-3.68546725	-0.53918737	-1.33856137

H	-3.30600031	-2.14570976	-0.76159783
H	-2.59342495	-1.04036128	1.46409187
H	-3.75624213	0.11387087	0.77073198
H	-1.48428760	-0.97735830	-2.38152650
H	1.17695705	0.53989160	-2.66582350
C	-1.51300774	1.24039005	1.70156634
H	-1.00815538	2.19517669	1.58069250
H	-2.50812974	1.38742482	2.16491808
H	-0.91805253	0.56575807	2.34910840
H	0.74791460	-0.16141267	1.68744261
C	3.80421711	0.20778021	1.23167721
H	3.97521629	-0.57338705	1.96127765
H	4.71286402	0.23283621	0.64136502
H	3.77348762	1.15682673	1.76481697
H	-0.97202573	-1.89552746	-0.95748371

Time = 242.00 fs
E(CASSCF/6-31G(d)) = -479.5264095

C	-0.92545129	0.17610848	-0.61967631
C	0.44060568	0.07747467	-0.55033964
C	1.48069855	0.29477748	-1.63115353
C	1.24865670	-0.11431459	0.72961197
C	2.56222557	-0.03118721	0.39489036
C	2.69809885	0.22923495	-1.03318016
N	-1.77169737	0.55183945	0.42180296
C	-2.84470832	-0.42297701	0.60177363
H	3.67078304	0.35037127	-1.55429399
C	-1.54647633	-1.02356438	-1.29924729
C	-2.97049898	-1.10876737	-0.75888973
H	-3.68571168	-0.53406060	-1.34018026
H	-3.30680251	-2.14180236	-0.77809497
H	-2.60522963	-1.05285626	1.45251493
H	-3.75576026	0.11384629	0.77153174
H	-1.49364045	-0.96419668	-2.37948325
H	1.17444608	0.51563381	-2.65951264
C	-1.50729686	1.23399366	1.70723029
H	-1.00987390	2.19444763	1.59022358
H	-2.50243648	1.37531864	2.16212560
H	-0.92218436	0.55813197	2.35501860
H	0.74164696	-0.14524808	1.69007077
C	3.81114538	0.21343840	1.23064859
H	3.97222469	-0.56484811	1.96470119
H	4.72092559	0.22029263	0.64077906
H	3.78959427	1.16445349	1.75810048
H	-0.96889678	-1.88370623	-0.97264192

Time = 242.50 fs
E(CASSCF/6-31G(d)) = -479.5242211

C	-0.92232781	0.17300640	-0.61707917
C	0.43827374	0.07423248	-0.55236521
C	1.48396923	0.28362198	-1.63131035
C	1.24262117	-0.10466635	0.72457359
C	2.55635239	-0.02082476	0.40053963
C	2.69337138	0.22374241	-1.03355271
N	-1.77057623	0.54840330	0.42585072
C	-2.84114295	-0.42101266	0.60027441
H	3.68209026	0.33207774	-1.53266722
C	-1.54969731	-1.01823007	-1.30368711
C	-2.97270456	-1.10612290	-0.76279242
H	-3.68538800	-0.52896916	-1.34226022
H	-3.30792412	-2.13756558	-0.79353487
H	-2.61756338	-1.06630232	1.44072524
H	-3.75627583	0.11396399	0.77390325
H	-1.50305905	-0.95144922	-2.37770929
H	1.17344828	0.49084932	-2.65171511
C	-1.50159263	1.22742418	1.71276712
H	-1.01159222	2.19299287	1.59959672
H	-2.49485499	1.36366919	2.15893926
H	-0.92729160	0.55228608	2.36086501
H	0.73743762	-0.12969382	1.69179073
C	3.81805458	0.21894910	1.22973183
H	3.96896209	-0.55643136	1.96883020
H	4.72853093	0.20775053	0.63943961
H	3.80511538	1.17194288	1.75123301
H	-0.96650477	-1.87186008	-0.98829765

Time = 243.00 fs
E(CASSCF/6-31G(d)) = -479.5217841

C	-0.91927168	0.16966925	-0.61429042
C	0.43670078	0.07125115	-0.55412268
C	1.48676987	0.27229028	-1.63137549
C	1.23627522	-0.09474398	0.71937661
C	2.55079793	-0.01028309	0.40622714
C	2.68884920	0.21826885	-1.03417220
N	-1.76951439	0.54493680	0.43003161
C	-2.83765062	-0.41901526	0.59863711
H	3.69060676	0.31370617	-1.50973712
C	-1.55308378	-1.01278369	-1.30836009
C	-2.97484829	-1.10349201	-0.76673282
H	-3.68456642	-0.52383989	-1.34485783
H	-3.30941826	-2.13312431	-0.80777323

H	-2.63019276	-1.08065017	1.42896032
H	-3.75755055	0.11415856	0.77776547
H	-1.51248806	-0.93906191	-2.37672840
H	1.17388442	0.46570435	-2.64315158
C	-1.49591555	1.22069977	1.71814726
H	-1.01323942	2.19069262	1.60879789
H	-2.48578339	1.35260121	2.15562878
H	-0.93303548	0.54799877	2.36681378
H	0.73537512	-0.11475130	1.69220196
C	3.82490642	0.22429913	1.22892048
H	3.96545144	-0.54812390	1.97368823
H	4.73555430	0.19525229	0.63732700
H	3.82009974	1.17939387	1.74423509
H	-0.96476851	-1.86043164	-1.00431382

Time = 243.50 fs
E(CASSCF/6-31G(d)) = -479.5190946

C	-0.91633112	0.16610704	-0.61131622
C	0.43591261	0.06853607	-0.55560841
C	1.48905437	0.26077393	-1.63133436
C	1.22963025	-0.08455299	0.71410238
C	2.54559363	0.00043267	0.41191297
C	2.68461743	0.21280291	-1.03495867
N	-1.76847078	0.54144615	0.43433348
C	-2.83430509	-0.41701491	0.59686078
H	3.69614723	0.29536582	-1.48586994
C	-1.55664224	-1.00720271	-1.31324917
C	-2.97691360	-1.10084986	-0.77070834
H	-3.68332597	-0.51860301	-1.34802295
H	-3.31133413	-2.12860548	-0.82068322
H	-2.64289279	-1.09580248	1.41739359
H	-3.75928739	0.11433620	0.78300455
H	-1.52188052	-0.92696363	-2.37702451
H	1.17560910	0.44037040	-2.63465693
C	-1.49028233	1.21384297	1.72333590
H	-1.01472775	2.18746852	1.61780221
H	-2.47573163	1.34224585	2.15249312
H	-0.93901907	0.54496133	2.37308117
H	0.73543726	-0.10042234	1.69104550
C	3.83166485	0.22947499	1.22820962
H	3.96172047	-0.53988424	1.97926406
H	4.74185916	0.18283887	0.63445617
H	3.83460721	1.18689695	1.73712235
H	-0.96359360	-1.84985447	-1.02056234

Time = 244.00 fs

E(CASSCF/6-31G(d)) = -479.5162194

C	-0.91354744	0.16233015	-0.60816357
C	0.43591995	0.06609172	-0.55682175
C	1.49078651	0.24906531	-1.63116611
C	1.22271488	-0.07409863	0.70882052
C	2.54076296	0.01131568	0.41755519
C	2.68074404	0.20733331	-1.03582424
N	-1.76740412	0.53793608	0.43874359
C	-2.83118120	-0.41504561	0.59495296
H	3.69871294	0.27717436	-1.46151756
C	-1.56037578	-1.00146939	-1.31834061
C	-2.97888679	-1.09817089	-0.77471677
H	-3.68174913	-0.51319780	-1.35179332
H	-3.31371318	-2.12412660	-0.83215685
H	-2.65544916	-1.11160256	1.40612413
H	-3.76115794	0.11438969	0.78947944
H	-1.53119760	-0.91507275	-2.37899129
H	1.17842569	0.41500766	-2.62712212
C	-1.48470654	1.20687913	1.72829542
H	-1.01595297	2.18328675	1.62657364
H	-2.46528841	1.33273467	2.14984481
H	-0.94481044	0.54279886	2.37991581
H	0.73748772	-0.08670536	1.68821956
C	3.83829582	0.23446345	1.22759619
H	3.95780435	-0.53164591	1.98551217
H	4.74730652	0.17055213	0.63087208
H	3.84870520	1.19452774	1.72990398
H	-0.96289308	-1.84051779	-1.03693426

Time = 244.50 fs
E(CASSCF/6-31G(d)) = -479.5132105

C	-0.91095293	0.15834872	-0.60484059
C	0.43671625	0.06392204	-0.55776645
C	1.49194004	0.23715854	-1.63084905
C	1.21557564	-0.06338615	0.70358932
C	2.53632123	0.02235761	0.42311078
C	2.67727773	0.20184937	-1.03667622
N	-1.76627449	0.53441049	0.44324892
C	-2.82835156	-0.41314601	0.59292927
H	3.69849545	0.25925160	-1.43720099
C	-1.56428231	-0.99557290	-1.32362647
C	-2.98075798	-1.09542958	-0.77875634
H	-3.67991613	-0.50757788	-1.35619010
H	-3.31658616	-2.11978539	-0.84210647
H	-2.66766152	-1.12783539	1.39517696

H	-3.76283186	0.11421362	0.79702889
H	-1.54040841	-0.90330294	-2.38288962
H	1.18211163	0.38975072	-2.62141488
C	-1.47919949	1.19983395	1.73298883
H	-1.01679619	2.17816074	1.63506659
H	-2.45507525	1.32419298	2.14799023
H	-0.94997264	0.54110176	2.38757591
H	0.74127984	-0.07359431	1.68378149
C	3.84476682	0.23925253	1.22707945
H	3.95374457	-0.52332158	1.99235606
H	4.75176267	0.15843275	0.62664358
H	3.86246758	1.20233992	1.72257872
H	-0.96260756	-1.83273609	-1.05335111

Time = 245.00 fs
E(CASSCF/6-31G(d)) = -479.5102249

C	-0.90857006	0.15417270	-0.60135651
C	0.43827725	0.06203024	-0.55845076
C	1.49249754	0.22505076	-1.63036755
C	1.20827594	-0.05242088	0.69845557
C	2.53227595	0.03354866	0.42853672
C	2.67424633	0.19634183	-1.03742032
N	-1.76504395	0.53087203	0.44783639
C	-2.82588399	-0.41135941	0.59081189
H	3.69587403	0.24171178	-1.41349235
C	-1.56835325	-0.98951097	-1.32910652
C	-2.98252183	-1.09260107	-0.78282608
H	-3.67790069	-0.50171563	-1.36121377
H	-3.31997010	-2.11565052	-0.85046552
H	-2.67934249	-1.14423353	1.38451278
H	-3.76400234	0.11371691	0.80547840
H	-1.54948826	-0.89157007	-2.38881639
H	1.18644961	0.36469356	-2.61828375
C	-1.47377179	1.19273095	1.73738410
H	-1.01712684	2.17214895	1.64322683
H	-2.44568901	1.31673189	2.14720639
H	-0.95409906	0.53946468	2.39629656
H	0.74646574	-0.06107710	1.67794629
C	3.85104644	0.24383251	1.22666090
H	3.94958974	-0.51481086	1.99969429
H	4.75510809	0.14651999	0.62185676
H	3.87597176	1.21036082	1.71513374
H	-0.96272331	-1.82672153	-1.06977117

Time = 245.50 fs
E(CASSCF/6-31G(d)) = -479.5074381

C	-0.90641098	0.14981173	-0.59772165
C	0.44056082	0.06041874	-0.55888767
C	1.49244926	0.21274299	-1.62971919
C	1.20089444	-0.04120821	0.69345535
C	2.52862750	0.04487768	0.43379101
C	2.67165620	0.19080347	-1.03796411
N	-1.76367727	0.52732204	0.45249298
C	-2.82383926	-0.40973274	0.58862746
H	3.69140208	0.22465647	-1.39099337
C	-1.57257286	-0.98329085	-1.33478894
C	-2.98417808	-1.08966185	-0.78692597
H	-3.67576711	-0.49560514	-1.36684149
H	-3.32386618	-2.11175485	-0.85718925
H	-2.69031646	-1.16049163	1.37404513
H	-3.76440763	0.11283259	0.81464718
H	-1.55841633	-0.87979750	-2.39669318
H	1.19125954	0.33987976	-2.61825962
C	-1.46843505	1.18558894	1.74145912
H	-1.01680673	2.16534988	1.65099315
H	-2.43763837	1.31044004	2.14771683
H	-0.95685032	0.53752945	2.40625270
H	0.75261142	-0.04913512	1.67107492
C	3.85710391	0.24819681	1.22634409
H	3.94539534	-0.50600897	2.00740838
H	4.75724540	0.13485026	0.61660808
H	3.88929620	1.21858885	1.70754425
H	-0.96328361	-1.82256638	-1.08619155

Time = 246.00 fs
E(CASSCF/6-31G(d)) = -479.5050074

C	-0.90447738	0.14527509	-0.59394749
C	0.44350727	0.05908886	-0.55909494
C	1.49179220	0.20024046	-1.62892071
C	1.19352248	-0.02975361	0.68861588
C	2.52537018	0.05633233	0.43883356
C	2.66949313	0.18522967	-1.03822045
N	-1.76214285	0.52376036	0.45720587
C	-2.82226918	-0.40831484	0.58640468
H	3.68577764	0.20816790	-1.37031033
C	-1.57691837	-0.97692930	-1.34068964
C	-2.98573172	-1.08659013	-0.79105694
H	-3.67356894	-0.48926355	-1.37302625
H	-3.32825842	-2.10809222	-0.86225519
H	-2.70041880	-1.17628645	1.36366149
H	-3.76384763	0.11152429	0.82435487

H	-1.56717347	-0.86791989	-2.40627670
H	1.19642377	0.31530028	-2.62157595
C	-1.46320370	1.17841999	1.74520632
H	-1.01569538	2.15789372	1.65829943
H	-2.43128372	1.30537568	2.14966977
H	-0.95798668	0.53502498	2.41752347
H	0.75921875	-0.03774367	1.66365107
C	3.86290886	0.25234281	1.22613408
H	3.94122294	-0.49681584	2.01537165
H	4.75810577	0.12345561	0.61099784
H	3.90251738	1.22699318	1.69977428
H	-0.96439114	-1.82023821	-1.10264510

Time = 246.50 fs
E(CASSCF/6-31G(d)) = -479.5030357

C	-0.90276090	0.14057171	-0.59004657
C	0.44704030	0.05804068	-0.55909508
C	1.49053023	0.18755211	-1.62801073
C	1.18626078	-0.01806256	0.68395847
C	2.52249354	0.06789933	0.44362692
C	2.66772521	0.17961871	-1.03811098
N	-1.76041347	0.52018511	0.46196253
C	-2.82121501	-0.40715429	0.58417186
H	3.67979365	0.19230378	-1.35202575
C	-1.58136094	-0.97045169	-1.34683026
C	-2.98719297	-1.08336628	-0.79522077
H	-3.67134962	-0.48273026	-1.37969767
H	-3.33311366	-2.10461755	-0.86566377
H	-2.70949607	-1.19129896	1.35324443
H	-3.76219529	0.10978993	0.83442775
H	-1.57574026	-0.85588414	-2.41718849
H	1.20189737	0.29090038	-2.62812894
C	-1.45809625	1.17122841	1.74863604
H	-1.01365635	2.14993065	1.66507714
H	-2.42679212	1.30156041	2.15312254
H	-0.95739058	0.53179599	2.43006474
H	0.76575389	-0.02687300	1.65624295
C	3.86843114	0.25627241	1.22603688
H	3.93713873	-0.48714474	2.02345830
H	4.75765366	0.11236189	0.60512448
H	3.91570746	1.23551612	1.69177879
H	-0.96620048	-1.81958824	-1.11919317

Time = 247.00 fs
E(CASSCF/6-31G(d)) = -479.5015518

C	-0.90124405	0.13571007	-0.58603240
C	0.45106853	0.05727273	-0.55891504
C	1.48867518	0.17468956	-1.62704835
C	1.17921531	-0.00614036	0.67950270
C	2.51998374	0.07956463	0.44813693
C	2.66630782	0.17397181	-1.03756940
N	-1.75846699	0.51659255	0.46675066
C	-2.82070609	-0.40629712	0.58195482
H	3.67426579	0.17709444	-1.33666788
C	-1.58586734	-0.96389020	-1.35323548
C	-2.98857682	-1.07997294	-0.79941979
H	-3.66914459	-0.47606431	-1.38676468
H	-3.33838246	-2.10125067	-0.86743878
H	-2.71740744	-1.20523607	1.34269021
H	-3.75940359	0.10766294	0.84470448
H	-1.58409598	-0.84364839	-2.42895789
H	1.20770090	0.26659384	-2.63748980
C	-1.45313561	1.16401136	1.75177788
H	-1.01056447	2.14161631	1.67125831
H	-2.42411757	1.29897559	2.15803572
H	-0.95507419	0.52781417	2.44369734
H	0.77168210	-0.01648978	1.64944884
C	3.87364085	0.25999229	1.22605858
H	3.93321158	-0.47692969	2.03155207
H	4.75588929	0.10158706	0.59907999
H	3.92893184	1.24407771	1.68350672
H	-0.96890227	-1.82037092	-1.13591543

Time = 247.50 fs
E(CASSCF/6-31G(d)) = -479.5005327

C	-0.89990147	0.13069824	-0.58191929
C	0.45548753	0.05678174	-0.55858588
C	1.48624888	0.16166581	-1.62610780
C	1.17249285	0.00600799	0.67527165
C	2.51782495	0.09131367	0.45233323
C	2.66519053	0.16829297	-1.03654458
N	-1.75628688	0.51297675	0.47155813
C	-2.82075900	-0.40578469	0.57977511
H	3.66994159	0.16254322	-1.32468042
C	-1.59040200	-0.95728162	-1.35992986
C	-2.98990247	-1.07639514	-0.80365651
H	-3.66698464	-0.46934003	-1.39411967
H	-3.34400131	-2.09788319	-0.86762773
H	-2.72402690	-1.21784908	1.33192314
H	-3.75550804	0.10521136	0.85504111
H	-1.59221846	-0.83117923	-2.44107053

H	1.21389834	0.24228080	-2.64896571
C	-1.44834833	1.15676095	1.75467939
H	-1.00631358	2.13309534	1.67677864
H	-2.42301120	1.29756235	2.16427825
H	-0.95117129	0.52316953	2.45811417
H	0.77650588	-0.00655842	1.64382848
C	3.87850855	0.26351393	1.22620463
H	3.92951079	-0.46613138	2.03955397
H	4.75284913	0.09113948	0.59294658
H	3.94224760	1.25258223	1.67490475
H	-0.97270190	-1.82227160	-1.15289881

Time = 248.00 fs
E(CASSCF/6-31G(d)) = -479.4999482

C	-0.89870153	0.12554386	-0.57772208
C	0.46018240	0.05656244	-0.55814214
C	1.48328522	0.14849392	-1.62527060
C	1.16619642	0.01837796	0.67129739
C	2.51600060	0.10313148	0.45618964
C	2.66432501	0.16258829	-1.03500294
N	-1.75386261	0.50932950	0.47637286
C	-2.82137695	-0.40565181	0.57764888
H	3.66740285	0.14863076	-1.31639523
C	-1.59492921	-0.95066481	-1.36693484
C	-2.99119245	-1.07262022	-0.80793313
H	-3.66490013	-0.46264147	-1.40164379
H	-3.34989553	-2.09438792	-0.86630211
H	-2.72924609	-1.22894873	1.32090541
H	-3.75062508	0.10253511	0.86531522
H	-1.60008464	-0.81844825	-2.45301534
H	1.22056809	0.21786476	-2.66169282
C	-1.44376258	1.14946737	1.75740254
H	-1.00082400	2.12448523	1.68158076
H	-2.42305873	1.29722540	2.17164280
H	-0.94591516	0.51804477	2.47290597
H	0.77980305	0.00295806	1.63982835
C	3.88300558	0.26685325	1.22647897
H	3.92610339	-0.45474072	2.04738815
H	4.74860401	0.08101685	0.58679483
H	3.95570198	1.26092565	1.66592125
H	-0.97779639	-1.82493724	-1.17022719

Time = 248.50 fs
E(CASSCF/6-31G(d)) = -479.4997908

C	-0.89760799	0.12025401	-0.57345596

C	0.46503076	0.05660742	-0.55762110
C	1.47983165	0.13518614	-1.62461706
C	1.16042118	0.03096551	0.66762557
C	2.51449442	0.11500293	0.45968458
C	2.66367227	0.15686516	-1.03293012
N	-1.75118988	0.50564024	0.48118265
C	-2.82254975	-0.40592522	0.57558607
H	3.66698005	0.13532252	-1.31201179
C	-1.59941517	-0.94407821	-1.37426613
C	-2.99247166	-1.06863771	-0.81225112
H	-3.66292534	-0.45605604	-1.40921295
H	-3.35598301	-2.09062956	-0.86355722
H	-2.73297731	-1.23841568	1.30964264
H	-3.74494617	0.09976133	0.87542884
H	-1.60767169	-0.80542821	-2.46432504
H	1.22777519	0.19326556	-2.67473892
C	-1.43940553	1.14212260	1.76001847
H	-0.99404908	2.11586209	1.68561714
H	-2.42373607	1.29783987	2.17986770
H	-0.93960838	0.51267937	2.48759952
H	0.78125834	0.01209813	1.63771418
C	3.88710454	0.27003011	1.22688329
H	3.92305155	-0.44278051	2.05500617
H	4.74325533	0.07120547	0.58068337
H	3.96933158	1.26900363	1.65651017
H	-0.98435298	-1.82800666	-1.18797309

Time = 249.00 fs
E(CASSCF/6-31G(d)) = -479.5000738

C	-0.89658188	0.11483530	-0.56913619
C	0.46990612	0.05690705	-0.55706168
C	1.47594955	0.12175333	-1.62421867
C	1.15525140	0.04376710	0.66431782
C	2.51329118	0.12691273	0.46280127
C	2.66320757	0.15113120	-1.03033152
N	-1.74827060	0.50189597	0.48597500
C	-2.82425442	-0.40662235	0.57359024
H	3.66870019	0.12257814	-1.31158600
C	-1.60382966	-0.93755773	-1.38193166
C	-2.99376633	-1.06443913	-0.81661074
H	-3.66110258	-0.44966812	-1.41670372
H	-3.36217821	-2.08647590	-0.85951153
H	-2.73515607	-1.24620653	1.29818587
H	-3.73872679	0.09703770	0.88530983
H	-1.61495847	-0.79208955	-2.47460790
H	1.23555035	0.16842672	-2.68719691

C	-1.43530054	1.13472363	1.76260168
H	-0.98598004	2.10725047	1.68885244
H	-2.42447121	1.29926017	2.18866137
H	-0.93259022	0.50733145	2.50170043
H	0.78068341	0.02090283	1.63752645
C	3.89077988	0.27306754	1.22741642
H	3.92041008	-0.43030482	2.06238909
H	4.73692957	0.06168029	0.57465989
H	3.98316209	1.27671923	1.64663450
H	-0.99249163	-1.83113769	-1.20619191

Time = 249.50 fs
E(CASSCF/6-31G(d)) = -479.5008020

C	-0.89558329	0.10929364	-0.56477788
C	0.47468155	0.05744955	-0.55650326
C	1.47171346	0.10820498	-1.62413206
C	1.15075843	0.05677957	0.66145084
C	2.51237721	0.13884553	0.46552807
C	2.66292168	0.14539334	-1.02723192
N	-1.74511277	0.49808152	0.49073697
C	-2.82645630	-0.40775041	0.57165871
H	3.67228187	0.11036034	-1.31502955
C	-1.60814729	-0.93113495	-1.38993014
C	-2.99510308	-1.06001785	-0.82101068
H	-3.65948550	-0.44355293	-1.42399909
H	-3.36839619	-2.08180857	-0.85430555
H	-2.73574334	-1.25235647	1.28663071
H	-3.73227071	0.09452339	0.89491106
H	-1.62192682	-0.77839735	-2.48357042
H	1.24387841	0.14331774	-2.69825810
C	-1.43146467	1.12727490	1.76522458
H	-0.97664816	2.09861964	1.69126526
H	-2.42470240	1.30132955	2.19772598
H	-0.92520615	0.50224349	2.51473461
H	0.77802279	0.02941740	1.63907016
C	3.89400857	0.27599085	1.22807382
H	3.91822414	-0.41739631	2.06954714
H	4.72977138	0.05240574	0.56876350
H	3.99720824	1.28398953	1.63626909
H	-1.00227239	-1.83402909	-1.22491865

Time = 250.00 fs
E(CASSCF/6-31G(d)) = -479.5019480

C	-0.89457293	0.10363421	-0.56039578
C	0.47923350	0.05822114	-0.55598410

C	1.46720911	0.09454927	-1.62439512
C	1.14699975	0.07000008	0.65911220
C	2.51174072	0.15078598	0.46785856
C	2.66281847	0.13965700	-1.02367383
N	-1.74173012	0.49417973	0.49545504
C	-2.82911098	-0.40930566	0.56978325
H	3.67717962	0.09864183	-1.32211961
C	-1.61234839	-0.92483579	-1.39825011
C	-2.99650787	-1.05536887	-0.82544784
H	-3.65814134	-0.43777125	-1.43099339
H	-3.37455652	-2.07653240	-0.84809986
H	-2.73472717	-1.25697817	1.27511389
H	-3.72590861	0.09237747	0.90420738
H	-1.62856298	-0.76430815	-2.49103168
H	1.25269482	0.11793267	-2.70726401
C	-1.42790692	1.11978942	1.76795276
H	-0.96612411	2.08988607	1.69284897
H	-2.42392588	1.30388963	2.20677756
H	-0.91778291	0.49761545	2.52628264
H	0.77334512	0.03769328	1.64194205
C	3.89677078	0.27882662	1.22884731
H	3.91652716	-0.40416118	2.07651748
H	4.72193551	0.04333671	0.56302804
H	4.01147472	1.29075109	1.62540222
H	-1.01368833	-1.83643777	-1.24416748

Time = 250.50 fs
E(CASSCF/6-31G(d)) = -479.5034481

C	-0.89351370	0.09786139	-0.55600410
C	0.48344559	0.05920629	-0.55553965
C	1.46253075	0.08079354	-1.62502445
C	1.14401891	0.08342583	0.65739328
C	2.51137193	0.16271880	0.46979171
C	2.66290965	0.13392570	-1.01971530
N	-1.73814162	0.49017187	0.50011504
C	-2.83216675	-0.41127307	0.56795116
H	3.68266402	0.08740821	-1.33251685
C	-1.61641938	-0.91867967	-1.40686950
C	-2.99800525	-1.05048870	-0.82991716
H	-3.65715198	-0.43236522	-1.43759616
H	-3.38058668	-2.07058311	-0.84107279
H	-2.73212366	-1.26025636	1.26380735
H	-3.71997271	0.09074548	0.91318964
H	-1.63485867	-0.74976792	-2.49693052
H	1.26188839	0.09228594	-2.71373848
C	-1.42462725	1.11228872	1.77084149

H	-0.95451436	2.08092284	1.69361191
H	-2.42173088	1.30678872	2.21556177
H	-0.91060924	0.49358639	2.53600564
H	0.76682415	0.04578911	1.64558828
C	3.89905056	0.28160168	1.22972502
H	3.91533933	-0.39072269	2.08335906
H	4.71357877	0.03442081	0.55748614
H	4.02595676	1.29696347	1.61403667
H	-1.02666311	-1.83819051	-1.26393251

Time = 251.00 fs
E(CASSCF/6-31G(d)) = -479.5052208

C	-0.89237193	0.09197868	-0.55161646
C	0.48721221	0.06038808	-0.55520076
C	1.45777811	0.06694475	-1.62601415
C	1.14184605	0.09705398	0.65638083
C	2.51126296	0.17462875	0.47133185
C	2.66320843	0.12820102	-1.01542705
N	-1.73437096	0.48603819	0.50470221
C	-2.83556741	-0.41362625	0.56614684
H	3.68791793	0.07665771	-1.34578919
C	-1.62035287	-0.91267894	-1.41575565
C	-2.99961763	-1.04537529	-0.83441166
H	-3.65661356	-0.42735552	-1.44373486
H	-3.38642477	-2.06393256	-0.83341744
H	-2.72797762	-1.26243831	1.25290961
H	-3.71476904	0.08974454	0.92185654
H	-1.64081190	-0.73471023	-2.50132622
H	1.27130755	0.06640692	-2.71740602
C	-1.42161638	1.10480180	1.77393318
H	-0.94195506	2.07157396	1.69357691
H	-2.41782249	1.30988924	2.22386546
H	-0.90392213	0.49022349	2.54366290
H	0.75871348	0.05377076	1.64937833
C	3.90083647	0.28434206	1.23069159
H	3.91466649	-0.37721316	2.09014572
H	4.70485221	0.02560086	0.55217351
H	4.04064119	1.30261080	1.60218961
H	-1.04105279	-1.83919110	-1.28419031

Time = 251.50 fs
E(CASSCF/6-31G(d)) = -479.5071828

C	-0.89111842	0.08598872	-0.54724582
C	0.49044179	0.06174863	-0.55499208
C	1.45305313	0.05301003	-1.62733529

C	1.14049856	0.11088140	0.65614871
C	2.51140779	0.18650078	0.47248856
C	2.66372360	0.12248271	-1.01088937
N	-1.73044589	0.48175849	0.50920126
C	-2.83925521	-0.41632800	0.56435370
H	3.69212891	0.06639851	-1.36143824
C	-1.62414747	-0.90683882	-1.42486587
C	-3.00136476	-1.04002801	-0.83892259
H	-3.65663460	-0.42274011	-1.44935605
H	-3.39202141	-2.05659148	-0.82533832
H	-2.72236289	-1.26381918	1.24263404
H	-3.71055021	0.08944877	0.93020498
H	-1.64642767	-0.71905533	-2.50439276
H	1.28076878	0.04033366	-2.71820075
C	-1.41885622	1.09736338	1.77725573
H	-0.92860413	2.06167182	1.69278048
H	-2.41203497	1.31307393	2.23152491
H	-0.89789770	0.48751670	2.54912251
H	0.74932000	0.06171006	1.65268057
C	3.90212208	0.28707198	1.23172866
H	3.91449953	-0.36376494	2.09695720
H	4.69589410	0.01681770	0.54713294
H	4.05550752	1.30770129	1.58989163
H	-1.05665151	-1.83942310	-1.30490342

Time = 252.00 fs
E(CASSCF/6-31G(d)) = -479.5092509

C	-0.88972932	0.07989323	-0.54290448
C	0.49305953	0.06326960	-0.55493055
C	1.44845666	0.03899725	-1.62893621
C	1.13998171	0.12490446	0.65675073
C	2.51180202	0.19832008	0.47327636
C	2.66445512	0.11676897	-1.00618893
N	-1.72639754	0.47731282	0.51359667
C	-2.84317339	-0.41933151	0.56255632
H	3.69456495	0.05664493	-1.37892627
C	-1.62780723	-0.90115756	-1.43414844
C	-3.00326342	-1.03444767	-0.84343968
H	-3.65733277	-0.41849436	-1.45442536
H	-3.39734071	-2.04860968	-0.81704775
H	-2.71538253	-1.26472231	1.23319396
H	-3.70749259	0.08987717	0.93822043
H	-1.65171779	-0.70270995	-2.50640565
H	1.29006557	0.01410623	-2.71626832
C	-1.41632064	1.09001174	1.78082152
H	-0.91463209	2.05105537	1.69127128

H	-2.40433749	1.31625125	2.23843127
H	-0.89264525	0.48537771	2.55236637
H	0.73897872	0.06968277	1.65492799
C	3.90290628	0.28981306	1.23281563
H	3.91481431	-0.35050116	2.10386929
H	4.68682489	0.00801301	0.54241768
H	4.07052908	1.31226487	1.57718511
H	-1.07320018	-1.83894746	-1.32602453

Time = 252.50 fs
E(CASSCF/6-31G(d)) = -479.5113315

C	-0.88818678	0.07369306	-0.53860418
C	0.49500959	0.06493261	-0.55502436
C	1.44408549	0.02491569	-1.63074335
C	1.14028935	0.13911881	0.65821545
C	2.51244266	0.21007220	0.47371427
C	2.66539148	0.11105683	-1.00141575
N	-1.72225975	0.47268216	0.51787292
C	-2.84726822	-0.42258238	0.56074273
H	3.69462784	0.04741344	-1.39770138
C	-1.63134096	-0.89562703	-1.44354438
C	-3.00532730	-1.02863656	-0.84795153
H	-3.65883049	-0.41457284	-1.45892612
H	-3.40236041	-2.04007368	-0.80876219
H	-2.70716949	-1.26547495	1.22478509
H	-3.70568179	0.09098594	0.94586931
H	-1.65670058	-0.68556890	-2.50772099
H	1.29897833	-0.01224009	-2.71196057
C	-1.41397661	1.08278651	1.78462693
H	-0.90021285	2.03958708	1.68910866
H	-2.39483436	1.31935992	2.24453404
H	-0.88820444	0.48364209	2.55349095
H	0.72803109	0.07776592	1.65566856
C	3.90319351	0.29258361	1.23393057
H	3.91557181	-0.33752698	2.11094360
H	4.67774375	-0.00086806	0.53809339
H	4.08567376	1.31634893	1.56412198
H	-1.09039843	-1.83789555	-1.34750146

Time = 253.00 fs
E(CASSCF/6-31G(d)) = -479.5133073

C	-0.88647949	0.06738828	-0.53435613
C	0.49625643	0.06671981	-0.55527243
C	1.44002961	0.01077651	-1.63266320
C	1.14140486	0.15351912	0.66054306

C	2.51332791	0.22174313	0.47382526
C	2.66650873	0.10534248	-0.99666032
N	-1.71806841	0.46784907	0.52201494
C	-2.85148992	-0.42602139	0.55890640
H	3.69188619	0.03871921	-1.41722058
C	-1.63476123	-0.89023354	-1.45298988
C	-3.00756709	-1.02259862	-0.85244611
H	-3.66124917	-0.41091256	-1.46285656
H	-3.40707101	-2.03110191	-0.80069850
H	-2.69788762	-1.26638086	1.21756624
H	-3.70510832	0.09266715	0.95309466
H	-1.66139979	-0.66751900	-2.50874533
H	1.30728455	-0.03868131	-2.70582021
C	-1.41178539	1.07572645	1.78865244
H	-0.88551477	2.02716705	1.68636091
H	-2.38376024	1.32237259	2.24984243
H	-0.88454531	0.48207439	2.55270356
H	0.71680673	0.08603497	1.65459942
C	3.90299370	0.29539832	1.23505130
H	3.91671859	-0.32492218	2.11821821
H	4.66872727	-0.00987470	0.53423858
H	4.10090466	1.32001266	1.55076095
H	-1.10791938	-1.83645641	-1.36928221

Time = 253.50 fs
E(CASSCF/6-31G(d)) = -479.5150364

C	-0.88460301	0.06097826	-0.53017113
C	0.49678552	0.06861426	-0.55566443
C	1.43636983	-0.00340666	-1.63458564
C	1.14330207	0.16809882	0.66370430
C	2.51445683	0.23331944	0.47363552
C	2.66777064	0.09962158	-0.99201097
N	-1.71386084	0.46279832	0.52600848
C	-2.85579279	-0.42958789	0.55704784
H	3.68609192	0.03057327	-1.43697019
C	-1.63808322	-0.88495898	-1.46241959
C	-3.00999080	-1.01633952	-0.85691122
H	-3.66470261	-0.40743753	-1.46622597
H	-3.41147404	-2.02183779	-0.79307049
H	-2.68773303	-1.26769333	1.21164084
H	-3.70567417	0.09475424	0.95981631
H	-1.66584291	-0.64844585	-2.50989689
H	1.31476943	-0.06521036	-2.69855046
C	-1.40970391	1.06886743	1.79286333
H	-0.87069273	2.01374347	1.68310346
H	-2.37146988	1.32529817	2.25442434

H	-0.88157201	0.48037663	2.55031383
H	0.70560821	0.09456096	1.65158643
C	3.90232211	0.29826816	1.23615650
H	3.91818742	-0.31273585	2.12570048
H	4.65983016	-0.01904894	0.53094334
H	4.11618037	1.32332035	1.53716492
H	-1.12542668	-1.83485791	-1.39132002

Time = 254.00 fs
E(CASSCF/6-31G(d)) = -479.5163696

C	-0.88256003	0.05446174	-0.52605973
C	0.49660310	0.07060032	-0.55618143
C	1.43317582	-0.01761752	-1.63638910
C	1.14594669	0.18284996	0.66764131
C	2.51582905	0.24478831	0.47317372
C	2.66912964	0.09388956	-0.98755151
N	-1.70967518	0.45751741	0.52984064
C	-2.86013436	-0.43322329	0.55517549
H	3.67718537	0.02298038	-1.45648409
C	-1.64132343	-0.87978217	-1.47177037
C	-3.01260419	-1.00986675	-0.86133513
H	-3.66928987	-0.40406427	-1.46905015
H	-3.41557968	-2.01244119	-0.78608576
H	-2.67693524	-1.26959244	1.20704198
H	-3.70720884	0.09703405	0.96593541
H	-1.67005892	-0.62824393	-2.51156040
H	1.32123685	-0.09184052	-2.69096414
C	-1.40768618	1.06224051	1.79721125
H	-0.85588143	1.99931881	1.67941700
H	-2.35842076	1.32818206	2.25840186
H	-0.87913144	0.47820138	2.54671877
H	0.69469864	0.10340787	1.64667127
C	3.90119909	0.30120064	1.23722661
H	3.91989808	-0.30098388	2.13336295
H	4.65108776	-0.02842471	0.52830636
H	4.13145488	1.32633437	1.52339825
H	-1.14259346	-1.83334235	-1.41357775

Time = 254.50 fs
E(CASSCF/6-31G(d)) = -479.5171815

C	-0.88036035	0.04783703	-0.52203228
C	0.49573540	0.07266387	-0.55679693
C	1.43050468	-0.03183712	-1.63794795
C	1.14929786	0.19776314	0.67226991
C	2.51744441	0.25613765	0.47247016

C	2.67052812	0.08814192	-0.98335901
N	-1.70554989	0.45199697	0.53350028
C	-2.86447418	-0.43687420	0.55330570
H	3.66529264	0.01593748	-1.47535997
C	-1.64449836	-0.87468038	-1.48098534
C	-3.01541141	-1.00318979	-0.86570705
H	-3.67508792	-0.40070779	-1.47134633
H	-3.41940376	-2.00307865	-0.77994277
H	-2.66575732	-1.27217072	1.20372402
H	-3.70949164	0.09926360	0.97134246
H	-1.67407575	-0.60682937	-2.51404048
H	1.32652006	-0.11860501	-2.68390886
C	-1.40568489	1.05587027	1.80163690
H	-0.84119031	1.98395184	1.67538585
H	-2.34514672	1.33110334	2.26194229
H	-0.87702830	0.47517059	2.54237990
H	0.68429230	0.11263037	1.64006786
C	3.89964959	0.30420034	1.23824468
H	3.92175813	-0.28964928	2.14114304
H	4.64251958	-0.03802774	0.52643060
H	4.14667733	1.32910857	1.50952447
H	-1.15912134	-1.83213778	-1.43603112

Time = 255.00 fs
E(CASSCF/6-31G(d)) = -479.5174040

C	-0.87802094	0.04110205	-0.51809896
C	0.49422708	0.07479264	-0.55747831
C	1.42839975	-0.04604456	-1.63914184
C	1.15330968	0.21282757	0.67748338
C	2.51930267	0.26735613	0.47155592
C	2.67190003	0.08237440	-0.97950187
N	-1.70152321	0.44623099	0.53697838
C	-2.86877236	-0.44049483	0.55146167
H	3.65071756	0.00943242	-1.49327376
C	-1.64762323	-0.86963077	-1.49001748
C	-3.01841555	-0.99632013	-0.87001765
H	-3.68214478	-0.39728770	-1.47312837
H	-3.42296452	-1.99391326	-0.77482826
H	-2.65449351	-1.27543005	1.20156247
H	-3.71227660	0.10118919	0.97592753
H	-1.67791740	-0.58415350	-2.51752028
H	1.33049140	-0.14555129	-2.67817057
C	-1.40365321	1.04977319	1.80607399
H	-0.82669986	1.96775581	1.67109608
H	-2.33222060	1.33416791	2.26524328
H	-0.87504735	0.47090100	2.53779147

H	0.67454885	0.12227165	1.63214801
C	3.89770276	0.30726966	1.23919671
H	3.92366385	-0.27868508	2.14894692
H	4.63413341	-0.04787654	0.52541799
H	4.16179207	1.33168260	1.49560417
H	-1.17475842	-1.83142787	-1.45867055

Time = 255.50 fs
E(CASSCF/6-31G(d)) = -479.5170371

C	-0.87556554	0.03425452	-0.51426992
C	0.49213924	0.07697610	-0.55818866
C	1.42689028	-0.06021784	-1.63986565
C	1.15793308	0.22803105	0.68315745
C	2.52140295	0.27843311	0.47046202
C	2.67317252	0.07658328	-0.97603816
N	-1.69763267	0.44021700	0.54026843
C	-2.87298864	-0.44404829	0.54967180
H	3.63392905	0.00344405	-1.50999131
C	-1.65071102	-0.86461168	-1.49883246
C	-3.02161928	-0.98927146	-0.87425948
H	-3.69047384	-0.39373332	-1.47440255
H	-3.42627980	-1.98509464	-0.77091522
H	-2.64346421	-1.27929020	1.20036360
H	-3.71531671	0.10256598	0.97959068
H	-1.68160218	-0.56021797	-2.52203399
H	1.33306964	-0.17272997	-2.67436781
C	-1.40154670	1.04395643	1.81045440
H	-0.81246076	1.95089277	1.66663466
H	-2.32020732	1.33749597	2.26851245
H	-0.87298216	0.46503632	2.53344108
H	0.66557247	0.13236343	1.62341657
C	3.89539145	0.31040959	1.24007189
H	3.92550155	-0.26802035	2.15665644
H	4.62593005	-0.05798226	0.52536392
H	4.17673735	1.33407813	1.48169438
H	-1.18931255	-1.83132446	-1.48149941

Time = 256.00 fs
E(CASSCF/6-31G(d)) = -479.5161309

C	-0.87302427	0.02729218	-0.51055538
C	0.48954732	0.07920544	-0.55888822
C	1.42599135	-0.07433546	-1.64003872
C	1.16311672	0.24336008	0.68915603
C	2.52374360	0.28935862	0.46921854
C	2.67426787	0.07076551	-0.97301428

N	-1.69391466	0.43395612	0.54336663
C	-2.87708170	-0.44750693	0.54796719
H	3.61554164	-0.00205789	-1.52537661
C	-1.65377178	-0.85960367	-1.50740986
C	-3.02502528	-0.98205974	-0.87842718
H	-3.70004999	-0.38998792	-1.47516488
H	-3.42936438	-1.97675052	-0.76836084
H	-2.63300729	-1.28360770	1.19988095
H	-3.71838555	0.10317545	0.98225204
H	-1.68514124	-0.53508412	-2.52745841
H	1.33422446	-0.20017875	-2.67285541
C	-1.39932545	1.03841688	1.81471422
H	-0.79849406	1.93356538	1.66208855
H	-2.30960924	1.34120563	2.27194233
H	-0.87066823	0.45728300	2.52976629
H	0.65741837	0.14292591	1.61447415
C	3.89275161	0.31362051	1.24086238
H	3.92714888	-0.25756706	2.16413941
H	4.61790713	-0.06835044	0.52635159
H	4.19144564	1.33629727	1.46784765
H	-1.20266026	-1.83184713	-1.50453146

Time = 256.50 fs
E(CASSCF/6-31G(d)) = -479.5147810

C	-0.87043301	0.02021250	-0.50696518
C	0.48653783	0.08147369	-0.55953692
C	1.42570378	-0.08837810	-1.63961091
C	1.16880793	0.25880001	0.69533875
C	2.52632153	0.30012342	0.46785378
C	2.67510664	0.06491881	-0.97046411
N	-1.69040394	0.42745288	0.54627196
C	-2.88100913	-0.45085186	0.54637897
H	3.59628982	-0.00711191	-1.53939474
C	-1.65681238	-0.85459006	-1.51574256
C	-3.02863649	-0.97470310	-0.88251763
H	-3.71080687	-0.38601092	-1.47539944
H	-3.43222753	-1.96897862	-0.76730457
H	-2.62346569	-1.28819992	1.19983645
H	-3.72129343	0.10284018	0.98385972
H	-1.68853816	-0.50887662	-2.53352378
H	1.33397772	-0.22790557	-2.67366596
C	-1.39695619	1.03314074	1.81879950
H	-0.78479409	1.91600529	1.65754356
H	-2.30080993	1.34539276	2.27568250
H	-0.86801519	0.44744563	2.52711160
H	0.65010534	0.15397045	1.60596800

C	3.88982183	0.31690285	1.24156295
H	3.92847662	-0.24722750	2.17126109
H	4.61006265	-0.07898229	0.52844771
H	4.20584437	1.33832290	1.45411203
H	-1.21474939	-1.83291195	-1.52778513

Time = 257.00 fs
E(CASSCF/6-31G(d)) = -479.5130288

C	-0.86783251	0.01301319	-0.50350906
C	0.48320636	0.08377531	-0.56009520
C	1.42601524	-0.10232993	-1.63856419
C	1.17495204	0.27433493	0.70156756
C	2.52913235	0.31071889	0.46639344
C	2.67561049	0.05904173	-0.96840906
N	-1.68713327	0.42071514	0.54898627
C	-2.88472791	-0.45407186	0.54493565
H	3.57698938	-0.01176282	-1.55210498
C	-1.65983670	-0.84955718	-1.52383487
C	-3.03245620	-0.96722195	-0.88652990
H	-3.72263833	-0.38177850	-1.47507955
H	-3.43487247	-1.96184278	-0.76786640
H	-2.61517454	-1.29287213	1.19994367
H	-3.72390072	0.10143492	0.98439640
H	-1.69178977	-0.48178060	-2.53984635
H	1.33240080	-0.25587506	-2.67649906
C	-1.39441412	1.02810395	1.82267126
H	-0.77133343	1.89846039	1.65308499
H	-2.29402870	1.35011171	2.27981658
H	-0.86503119	0.43545567	2.52569741
H	0.64363973	0.16550330	1.59853455
C	3.88664267	0.32025757	1.24217036
H	3.92935043	-0.23690218	2.17789649
H	4.60239739	-0.08987605	0.53169830
H	4.21985700	1.34012207	1.44053136
H	-1.22559457	-1.83433550	-1.55127813

Time = 257.50 fs
E(CASSCF/6-31G(d)) = -479.5109045

C	-0.86526726	0.00569218	-0.50019644
C	0.47965456	0.08610602	-0.56052557
C	1.42690101	-0.11617924	-1.63690935
C	1.18149153	0.28994754	0.70771402
C	2.53217014	0.32113701	0.46485988
C	2.67570672	0.05313358	-0.96685903
N	-1.68413298	0.41375382	0.55151418

C	-2.88819525	-0.45716219	0.54366078
H	3.55848874	-0.01605885	-1.56364334
C	-1.66284618	-0.84449398	-1.53169946
C	-3.03648774	-0.95963890	-0.89046503
H	-3.73540138	-0.37728193	-1.47417022
H	-3.43729607	-1.95537037	-0.77014502
H	-2.60844712	-1.29744205	1.19992918
H	-3.72612547	0.09889405	0.98388302
H	-1.69488763	-0.45403146	-2.54597114
H	1.32960926	-0.28400290	-2.68076820
C	-1.39168403	1.02327346	1.82630816
H	-0.75807029	1.88118022	1.64879800
H	-2.28929259	1.35535976	2.28434625
H	-0.86183369	0.42138722	2.52560404
H	0.63804355	0.17753139	1.59273876
C	3.88325604	0.32368635	1.24268271
H	3.92963320	-0.22649667	2.18394169
H	4.59491655	-0.10102805	0.53612638
H	4.23340506	1.34165086	1.42714592
H	-1.23526763	-1.83584930	-1.57502119

Time = 258.00 fs
E(CASSCF/6-31G(d)) = -479.5084141

C	-0.86278416	-0.00175227	-0.49703621
C	0.47598738	0.08846250	-0.56079356
C	1.42832508	-0.12991845	-1.63467916
C	1.18836475	0.30561894	0.71366537
C	2.53542730	0.33137029	0.46327143
C	2.67533333	0.04719432	-0.96581446
N	-1.68143060	0.40658265	0.55386298
C	-2.89136965	-0.46012331	0.54257117
H	3.54160941	-0.02004797	-1.57419241
C	-1.66584064	-0.83939139	-1.53935376
C	-3.04073408	-0.95197865	-0.89432575
H	-3.74892187	-0.37252439	-1.47263225
H	-3.43948986	-1.94955258	-0.77421600
H	-2.60356196	-1.30176031	1.19955074
H	-3.72794702	0.09521535	0.98238045
H	-1.69782000	-0.42589951	-2.55141918
H	1.32575525	-0.31215916	-2.68568777
C	-1.38876072	1.01860933	1.82970646
H	-0.74495770	1.86440081	1.64476837
H	-2.28643392	1.36106973	2.28918743
H	-0.85864323	0.40545603	2.52677578
H	0.63338423	0.19006862	1.58901954
C	3.87970463	0.32719158	1.24309876

H	3.92918739	-0.21592710	2.18932362
H	4.58763036	-0.11243349	0.54173097
H	4.24641087	1.34286070	1.41399309
H	-1.24388441	-1.83712438	-1.59901313

Time = 258.50 fs
E(CASSCF/6-31G(d)) = -479.5055813

C	-0.86043108	-0.00932142	-0.49403653
C	0.47231054	0.09084211	-0.56086836
C	1.43024121	-0.14354329	-1.63192000
C	1.19550475	0.32132846	0.71932878
C	2.53889472	0.34141178	0.46164184
C	2.67444479	0.04122448	-0.96526935
N	-1.67905053	0.39921790	0.55604237
C	-2.89421206	-0.46295972	0.54167564
H	3.52707995	-0.02377393	-1.58394019
C	-1.66881919	-0.83424142	-1.54681648
C	-3.04519717	-0.94426775	-0.89811608
H	-3.76300212	-0.36751643	-1.47042743
H	-3.44144184	-1.94434724	-0.78013010
H	-2.60075199	-1.30572596	1.19861118
H	-3.72940571	0.09046026	0.97998860
H	-1.70057378	-0.39767141	-2.55573223
H	1.32101870	-0.34017943	-2.69037708
C	-1.38564822	1.01406729	1.83287738
H	-0.73195395	1.84833009	1.64108360
H	-2.28511393	1.36711236	2.29417941
H	-0.85576089	0.38800163	2.52904292
H	0.62979841	0.20314128	1.58765120
C	3.87603125	0.33077610	1.24341733
H	3.92787754	-0.20512416	2.19400737
H	4.58055376	-0.12408676	0.54848740
H	4.25880080	1.34370522	1.40110787
H	-1.25159008	-1.83780130	-1.62323790

Time = 259.00 fs
E(CASSCF/6-31G(d)) = -479.5024816

C	-0.85825490	-0.01701590	-0.49120465
C	0.46872749	0.09324254	-0.56072302
C	1.43259425	-0.15705178	-1.62868384
C	1.20283913	0.33705376	0.72463331
C	2.54256153	0.35125492	0.45997984
C	2.67301726	0.03522506	-0.96521461
N	-1.67701367	0.39167799	0.55806419
C	-2.89668722	-0.46567885	0.54097439

H	3.51547368	-0.02727303	-1.59303422
C	-1.67178107	-0.82903617	-1.55410466
C	-3.04987722	-0.93653434	-0.90184093
H	-3.77742993	-0.36227103	-1.46752403
H	-3.44313888	-1.93968336	-0.78791156
H	-2.60019598	-1.30929640	1.19696786
H	-3.73059800	0.08475021	0.97684302
H	-1.70313643	-0.36963156	-2.55850949
H	1.31559709	-0.36788013	-2.69396109
C	-1.38235824	1.00960162	1.83584270
H	-0.71903291	1.83313536	1.63783352
H	-2.28486675	1.37330786	2.29910430
H	-0.85353492	0.36945620	2.53215516
H	0.62750396	0.21679084	1.58872681
C	3.87227834	0.33444284	1.24363684
H	3.92557278	-0.19403548	2.19800096
H	4.57370521	-0.13598143	0.55634939
H	4.27050921	1.34414715	1.38852344
H	-1.25854453	-1.83752168	-1.64766363

Time = 259.50 fs
E(CASSCF/6-31G(d)) = -479.4992566

C	-0.85629983	-0.02483549	-0.48854648
C	0.46533696	0.09566151	-0.56033448
C	1.43532121	-0.17044285	-1.62502186
C	1.21029093	0.35277099	0.72952944
C	2.54641511	0.36089347	0.45828885
C	2.67105280	0.02919763	-0.96564120
N	-1.67533719	0.38398313	0.55994202
C	-2.89876494	-0.46828986	0.54045906
H	3.50715890	-0.03057208	-1.60153991
C	-1.67472651	-0.82376691	-1.56123123
C	-3.05477192	-0.92880778	-0.90550567
H	-3.79198815	-0.35679819	-1.46390236
H	-3.44456981	-1.93546744	-0.79755679
H	-2.60201276	-1.31249311	1.19453844
H	-3.73166749	0.07825969	0.97310893
H	-1.70549762	-0.34204497	-2.55943562
H	1.30969566	-0.39507540	-2.69565039
C	-1.37890802	1.00516768	1.83862938
H	-0.70619355	1.81893410	1.63511031
H	-2.28515467	1.37944324	2.30371228
H	-0.85232274	0.35030689	2.53581908
H	0.62679439	0.23107401	1.59216771
C	3.86848750	0.33819423	1.24375497
H	3.92214956	-0.18262658	2.20135652

H	4.56710520	-0.14810894	0.56525186
H	4.28148155	1.34416456	1.37627182
H	-1.26490877	-1.83595719	-1.67224295

Time = 260.00 fs
E(CASSCF/6-31G(d)) = -479.4960863

C	-0.85460540	-0.03277890	-0.48606663
C	0.46223057	0.09809654	-0.55968304
C	1.43835207	-0.18371556	-1.62098014
C	1.21778065	0.36845541	0.73398628
C	2.55044125	0.37032153	0.45656672
C	2.66858202	0.02314468	-0.96654166
N	-1.67403426	0.37615494	0.56169072
C	-2.90042170	-0.47080250	0.54011325
H	3.50227061	-0.03368957	-1.60941359
C	-1.67765711	-0.81842361	-1.56820320
C	-3.05987575	-0.92111819	-0.90911584
H	-3.80646456	-0.35110043	-1.45955930
H	-3.44572800	-1.93159095	-0.80903372
H	-2.60625821	-1.31540389	1.19130307
H	-3.73279191	0.07120514	0.96897239
H	-1.70764963	-0.31513945	-2.55829852
H	1.30351838	-0.42159214	-2.69479711
C	-1.37531791	1.00072409	1.84126466
H	-0.69346570	1.80578815	1.63300779
H	-2.28542397	1.38529382	2.30774860
H	-0.85245571	0.33105648	2.53973405
H	0.62801805	0.24605807	1.59775220
C	3.86469887	0.34203172	1.24376852
H	3.91749328	-0.17087908	2.20416920
H	4.56077378	-0.16045919	0.57511525
H	4.29167931	1.34375554	1.36438287
H	-1.27083560	-1.83283460	-1.69691781

Time = 260.50 fs
E(CASSCF/6-31G(d)) = -479.4931768

C	-0.85320463	-0.04084340	-0.48376802
C	0.45949049	0.10054463	-0.55875208
C	1.44161099	-0.19686822	-1.61659709
C	1.22522936	0.38408199	0.73798772
C	2.55462392	0.37953341	0.45480570
C	2.66566359	0.01707014	-0.96791033
N	-1.67311397	0.36821600	0.56332588
C	-2.90164219	-0.47322591	0.53991363
H	3.50071176	-0.03663863	-1.61649692

C	-1.68057643	-0.81299451	-1.57502050
C	-3.06517934	-0.91349600	-0.91267681
H	-3.82066012	-0.34516909	-1.45451166
H	-3.44661412	-1.92793693	-0.82228164
H	-2.61292517	-1.31818029	1.18730282
H	-3.73416425	0.06383033	0.96462867
H	-1.70958843	-0.28909374	-2.55499897
H	1.29725889	-0.44728241	-2.69092870
C	-1.37160924	0.99623410	1.84377197
H	-0.68091301	1.79370359	1.63161933
H	-2.28515479	1.39064256	2.31097651
H	-0.85421028	0.31218916	2.54362030
H	0.63154456	0.26181556	1.60515995
C	3.86095091	0.34595506	1.24367353
H	3.91150032	-0.15878829	2.20657152
H	4.55472803	-0.17301828	0.58585022
H	4.30108213	1.34294144	1.35288479
H	-1.27646100	-1.82795369	-1.72162250

Time = 261.00 fs
E(CASSCF/6-31G(d)) = -479.4907274

C	-0.85212295	-0.04902441	-0.48165162
C	0.45718792	0.10300201	-0.55752753
C	1.44501734	-0.20989768	-1.61190200
C	1.23256244	0.39962602	0.74152790
C	2.55894483	0.38852343	0.45299248
C	2.66238156	0.01098010	-0.96974152
N	-1.67258088	0.36018921	0.56486321
C	-2.90242097	-0.47556743	0.53983154
H	3.50218193	-0.03943210	-1.62253564
C	-1.68349009	-0.80746604	-1.58167536
C	-3.07066904	-0.90597140	-0.91619365
H	-3.83439646	-0.33898154	-1.44879829
H	-3.44723861	-1.92438698	-0.83721153
H	-2.62194544	-1.32103070	1.18263517
H	-3.73597013	0.05638952	0.96026927
H	-1.71131383	-0.26402699	-2.54955363
H	1.29109193	-0.47203362	-2.68376727
C	-1.36780263	0.99166636	1.84616815
H	-0.66863178	1.78263434	1.63103420
H	-2.28390016	1.39529639	2.31319488
H	-0.85778797	0.29414297	2.54723835
H	0.63772626	0.27841731	1.61401992
C	3.85728009	0.34996182	1.24346560
H	3.90408023	-0.14635950	2.20872538
H	4.54898014	-0.18576683	0.59736278

H	4.30969039	1.34176872	1.34180417
H	-1.28189834	-1.82120010	-1.74628843

Time = 261.50 fs
E(CASSCF/6-31G(d)) = -479.4888696

C	-0.85137676	-0.05731544	-0.47971621
C	0.45538153	0.10546403	-0.55599753
C	1.44848718	-0.22279898	-1.60691419
C	1.23971339	0.41506392	0.74460708
C	2.56338280	0.39728608	0.45110856
C	2.65884049	0.00488333	-0.97202677
N	-1.67243491	0.35209727	0.56631789
C	-2.90276422	-0.47783185	0.53983486
H	3.50622463	-0.04208782	-1.62721490
C	-1.68640585	-0.80182301	-1.58815208
C	-3.07632678	-0.89857379	-0.91967096
H	-3.84752161	-0.33250021	-1.44248043
H	-3.44762320	-1.92082770	-0.85370795
H	-2.63319552	-1.32420967	1.17744527
H	-3.73836420	0.04912862	0.95606779
H	-1.71282949	-0.23999028	-2.54209227
H	1.28516560	-0.49577760	-2.67323700
C	-1.36391683	0.98699519	1.84846212
H	-0.65674384	1.77249048	1.63133371
H	-2.28131353	1.39910036	2.31425124
H	-0.86330331	0.27728808	2.55040300
H	0.64686307	0.29592600	1.62395342
C	3.85372061	0.35404682	1.24314049
H	3.89515667	-0.13360210	2.21081078
H	4.54353472	-0.19867944	0.60955966
H	4.31752720	1.34030786	1.33116476
H	-1.28723525	-1.81255189	-1.77085030

Time = 262.00 fs
E(CASSCF/6-31G(d)) = -479.4876857

C	-0.85097334	-0.06570739	-0.47795821
C	0.45411648	0.10792473	-0.55415183
C	1.45193515	-0.23556457	-1.60164270
C	1.24662718	0.43037341	0.74722801
C	2.56791278	0.40581563	0.44913075
C	2.65515948	-0.00120807	-0.97475169
N	-1.67267080	0.34396209	0.56770393
C	-2.90269083	-0.48002089	0.53989022
H	3.51228312	-0.04463369	-1.63020350
C	-1.68933348	-0.79604900	-1.59442719

C	-3.08213005	-0.89133121	-0.92311285
H	-3.85991374	-0.32567241	-1.43564144
H	-3.44780249	-1.91715636	-0.87163062
H	-2.64650344	-1.32800079	1.17191399
H	-3.74144710	0.04226818	0.95216768
H	-1.71414261	-0.21696275	-2.53285080
H	1.27959423	-0.51849692	-2.65946501
C	-1.35996829	0.98220031	1.85065421
H	-0.64538736	1.76314926	1.63258613
H	-2.27716691	1.40194676	2.31404931
H	-0.87077869	0.26191252	2.55298971
H	0.65917420	0.31439257	1.63460950
C	3.85030441	0.35820183	1.24269506
H	3.88466938	-0.12052417	2.21301114
H	4.53838676	-0.21172172	0.62235273
H	4.32463750	1.33865089	1.32098795
H	-1.29253092	-1.80208138	-1.79524996

Time = 262.50 fs
E(CASSCF/6-31G(d)) = -479.4871757

C	-0.85091069	-0.07418855	-0.47637159
C	0.45342327	0.11037662	-0.55198139
C	1.45527700	-0.24818386	-1.59608594
C	1.25326339	0.44553399	0.74939335
C	2.57250470	0.41410619	0.44703197
C	2.65146568	-0.00727852	-0.97789356
N	-1.67327784	0.33580418	0.56903378
C	-2.90223307	-0.48213373	0.53996525
H	3.51975804	-0.04711146	-1.63119755
C	-1.69228449	-0.79012690	-1.60047010
C	-3.08805210	-0.88426976	-0.92652300
H	-3.87148248	-0.31843186	-1.42838423
H	-3.44782428	-1.91328481	-0.89081703
H	-2.66165852	-1.33269299	1.16624052
H	-3.74524589	0.03598879	0.94867223
H	-1.71526374	-0.19485063	-2.52215816
H	1.27445063	-0.54023113	-2.64277823
C	-1.35597095	0.97726642	1.85273630
H	-0.63470500	1.75446640	1.63484185
H	-2.27136053	1.40378173	2.31255219
H	-0.88014536	0.24821198	2.55493802
H	0.67477759	0.33385401	1.64569098
C	3.84706109	0.36241534	1.24212824
H	3.87257581	-0.10712615	2.21549769
H	4.53352038	-0.22484944	0.63566289
H	4.33108716	1.33690668	1.31129232

H	-1.29781614	-1.78995076	-1.81944090

Time = 263.00 fs
E(CASSCF/6-31G(d)) = -479.4872353

C	-0.85117715	-0.08274425	-0.47494860
C	0.45331767	0.11281041	-0.54947812
C	1.45843215	-0.26064295	-1.59023131
C	1.25959828	0.46052701	0.75110365
C	2.57712300	0.42215174	0.44478164
C	2.64788806	-0.01330889	-0.98141922
N	-1.67423997	0.32764238	0.57031827
C	-2.90143673	-0.48416854	0.54003099
H	3.52805631	-0.04957907	-1.62995696
C	-1.69527173	-0.78403946	-1.60624447
C	-3.09406261	-0.87741317	-0.92990488
H	-3.88216871	-0.31070189	-1.42082783
H	-3.44774952	-1.90914194	-0.91108624
H	-2.67842414	-1.33855228	1.16062196
H	-3.74970346	0.03042222	0.94563982
H	-1.71620626	-0.17349135	-2.51041744
H	1.26976063	-0.56108003	-2.62369402
C	-1.35193648	0.97218276	1.85469269
H	-0.62483024	1.74628865	1.63812926
H	-2.26392659	1.40460959	2.30978295
H	-0.89124832	0.23628379	2.55625441
H	0.69368127	0.35433134	1.65697024
C	3.84401745	0.36667267	1.24144210
H	3.85885185	-0.09339530	2.21841434
H	4.52890888	-0.23800859	0.64942282
H	4.33696042	1.33519530	1.30209299
H	-1.30309612	-1.77640233	-1.84339263

Time = 263.50 fs
E(CASSCF/6-31G(d)) = -479.4877205

C	-0.85175146	-0.09135696	-0.47367955
C	0.45379947	0.11521490	-0.54663469
C	1.46132583	-0.27292447	-1.58405627
C	1.26562463	0.47533584	0.75235667
C	2.58172743	0.42994631	0.44234674
C	2.64455264	-0.01927693	-0.98528474
N	-1.67553599	0.31949372	0.57156657
C	-2.90035969	-0.48612468	0.54006397
H	3.53663284	-0.05211015	-1.62633021
C	-1.69830888	-0.77776980	-1.61171022
C	-3.10012830	-0.87078214	-0.93326180

H	-3.89194136	-0.30239917	-1.41310277
H	-3.44765161	-1.90467526	-0.93224251
H	-2.69654947	-1.34578908	1.15523004
H	-3.75467664	0.02564854	0.94308374
H	-1.71698618	-0.15266419	-2.49808059
H	1.26549851	-0.58120493	-2.60290100
C	-1.34787465	0.96694222	1.85650043
H	-0.61587479	1.73846527	1.64245069
H	-2.25502699	1.40449430	2.30582225
H	-0.90385430	0.22612366	2.55701228
H	0.71578152	0.37583048	1.66829571
C	3.84119751	0.37095630	1.24064292
H	3.84349459	-0.07930125	2.22186228
H	4.52451534	-0.25113515	0.66357812
H	4.34235575	1.33364017	1.29340186
H	-1.30835409	-1.76174293	-1.86709164

Time = 264.00 fs
E(CASSCF/6-31G(d)) = -479.4884179

C	-0.85260267	-0.10000687	-0.47255320
C	0.45485265	0.11757712	-0.54344427
C	1.46388984	-0.28500834	-1.57753062
C	1.27134990	0.48994618	0.75314743
C	2.58627494	0.43748416	0.43969210
C	2.64157914	-0.02515765	-0.98943507
N	-1.67714011	0.31137351	0.57278627
C	-2.89906984	-0.48800567	0.54004785
H	3.54502235	-0.05479303	-1.62027058
C	-1.70140974	-0.77130173	-1.61682633
C	-3.10621387	-0.86439377	-0.93659707
H	-3.90079254	-0.29343896	-1.40534507
H	-3.44761478	-1.89985111	-0.95408031
H	-2.71578159	-1.35452673	1.15018883
H	-3.75994587	0.02169954	0.94097705
H	-1.71762125	-0.13210710	-2.48561528
H	1.26158714	-0.60082540	-2.58122501
C	-1.34379401	0.96154061	1.85813035
H	-0.60791605	1.73085775	1.64778053
H	-2.24494517	1.40355916	2.30080336
H	-0.91766398	0.21762636	2.55735106
H	0.74087147	0.39834389	1.67959006
C	3.83862205	0.37524657	1.23974202
H	3.82652326	-0.06479264	2.22588891
H	4.52029421	-0.26415759	0.67808898
H	4.34738148	1.33235872	1.28522645
H	-1.31355871	-1.74632148	-1.89054228

Time = 264.50 fs
E(CASSCF/6-31G(d)) = -479.4891148

C	-0.85369077	-0.10867144	-0.47155670
C	0.45644598	0.11988206	-0.53990074
C	1.46606362	-0.29687236	-1.57062007
C	1.27679378	0.50434559	0.75346896
C	2.59072167	0.44475988	0.43678124
C	2.63907758	-0.03092403	-0.99380508
N	-1.67902232	0.30329570	0.57398347
C	-2.89764266	-0.48982192	0.53997432
H	3.55285946	-0.05772784	-1.61184091
C	-1.70458748	-0.76462012	-1.62155415
C	-3.11228307	-0.85826118	-0.93991422
H	-3.90873113	-0.28374103	-1.39769141
H	-3.44773272	-1.89465407	-0.97638808
H	-2.73587190	-1.36477697	1.14555876
H	-3.76523352	0.01856682	0.93926178
H	-1.71813064	-0.11154292	-2.47346935
H	1.25790406	-0.62020615	-2.55957412
C	-1.33970263	0.95597576	1.85954799
H	-0.60098774	1.72334918	1.65406372
H	-2.23407289	1.40198230	2.29490566
H	-0.93232460	0.21059082	2.55747296
H	0.76865627	0.42185241	1.69083853
C	3.83630831	0.37952263	1.23875617
H	3.80798292	-0.04979831	2.23047929
H	4.51619378	-0.27699707	0.69292778
H	4.35214860	1.33145418	1.27757100
H	-1.31867123	-1.73050335	-1.91376185

Time = 265.00 fs
E(CASSCF/6-31G(d)) = -479.4896507

C	-0.85496763	-0.11732563	-0.47067565
C	0.45853449	0.12211285	-0.53599878
C	1.46779413	-0.30849416	-1.56329172
C	1.28198498	0.51852347	0.75331391
C	2.59502504	0.45176859	0.43357670
C	2.63714559	-0.03654772	-0.99832087
N	-1.68114885	0.29527295	0.57516273
C	-2.89615863	-0.49159276	0.53984302
H	3.55989425	-0.06102417	-1.60121375
C	-1.70785363	-0.75771108	-1.62586114
C	-3.11829979	-0.85239297	-0.94321710
H	-3.91577498	-0.27323607	-1.39027251

H	-3.44810599	-1.88908464	-0.99895321
H	-2.75657947	-1.37642588	1.14132830
H	-3.77022789	0.01621308	0.93786005
H	-1.71853379	-0.09070909	-2.46203065
H	1.25429481	-0.63963565	-2.53886218
C	-1.33560913	0.95024683	1.86071538
H	-0.59507295	1.71584957	1.66121605
H	-2.22288694	1.39998808	2.28834267
H	-0.94744843	0.20473185	2.55763397
H	0.79877454	0.44632829	1.70206987
C	3.83426947	0.38376373	1.23770763
H	3.78794575	-0.03422945	2.23555557
H	4.51215990	-0.28957186	0.70807834
H	4.35676533	1.33100730	1.27043614
H	-1.32365666	-1.71463906	-1.93677570

Time = 265.50 fs
E(CASSCF/6-31G(d)) = -479.4899383

C	-0.85637900	-0.12594246	-0.46989404
C	0.46106153	0.12425118	-0.53173424
C	1.46903716	-0.31985311	-1.55552075
C	1.28695834	0.53247084	0.75267653
C	2.59914575	0.45850594	0.43004161
C	2.63586456	-0.04200032	-1.00290276
N	-1.68348250	0.28731707	0.57632728
C	-2.89470089	-0.49334728	0.53966046
H	3.56600346	-0.06479700	-1.58866330
C	-1.71121727	-0.75056182	-1.62972472
C	-3.12422917	-0.84679304	-0.94651008
H	-3.92194343	-0.26187241	-1.38320839
H	-3.44883994	-1.88315741	-1.02156556
H	-2.77766954	-1.38923642	1.13741699
H	-3.77460958	0.01458341	0.93668605
H	-1.71885156	-0.06939303	-2.45159350
H	1.25059147	-0.65939508	-2.51991118
C	-1.33152385	0.94435375	1.86159339
H	-0.59010235	1.70829954	1.66912634
H	-2.21191753	1.39783223	2.28134823
H	-0.96263239	0.19970004	2.55812936
H	0.83082167	0.47173819	1.71333072
C	3.83251421	0.38795057	1.23662359
H	3.76651487	-0.01798612	2.24097918
H	4.50813866	-0.30179868	0.72353161
H	4.36132993	1.33106911	1.26381944
H	-1.32849224	-1.69903372	-1.95960590

Time = 266.00 fs
E(CASSCF/6-31G(d)) = -479.4899930

C	-0.85786616	-0.13449288	-0.46919401
C	0.46396102	0.12627727	-0.52710456
C	1.46975516	-0.33093356	-1.54729794
C	1.29175131	0.54618027	0.75155557
C	2.60304955	0.46496829	0.42613969
C	2.63529829	-0.04725400	-1.00746663
N	-1.68598302	0.27943889	0.57747887
C	-2.89335276	-0.49512360	0.53943810
H	3.57119330	-0.06916517	-1.57455880
C	-1.71468396	-0.74316092	-1.63313493
C	-3.13003867	-0.84146016	-0.94979795
H	-3.92724931	-0.24962146	-1.37660268
H	-3.45004162	-1.87689736	-1.04402227
H	-2.79890969	-1.40286529	1.13368787
H	-3.77807653	0.01361663	0.93565619
H	-1.71910432	-0.04745895	-2.44233026
H	1.24664164	-0.67971915	-2.50335104
C	-1.32746063	0.93829631	1.86214482
H	-0.58595353	1.70067155	1.67765866
H	-2.20170514	1.39578187	2.27415651
H	-0.97748190	0.19510951	2.55927074
H	0.86437641	0.49804514	1.72465351
C	3.83104619	0.39206685	1.23553524
H	3.74382339	-0.00096281	2.24656125
H	4.50408042	-0.31359853	0.73928545
H	4.36592620	1.33165500	1.25771494
H	-1.33317849	-1.68391870	-1.98226335

Time = 266.50 fs
E(CASSCF/6-31G(d)) = -479.4898896

C	-0.85936837	-0.14294643	-0.46855593
C	0.46716012	0.12817056	-0.52210912
C	1.46991852	-0.34172715	-1.53863538
C	1.29640158	0.55964578	0.74995674
C	2.60670875	0.47115273	0.42183670
C	2.63548940	-0.05228291	-1.01192752
N	-1.68860750	0.27164839	0.57861795
C	-2.89219592	-0.49696686	0.53919000
H	3.57559928	-0.07424809	-1.55934809
C	-1.71825510	-0.73549843	-1.63609643
C	-3.13569892	-0.83638807	-0.95308600
H	-3.93169360	-0.23648291	-1.37053955
H	-3.45181704	-1.87033693	-1.06613087

H	-2.82006632	-1.41689389	1.12996816
H	-3.78036667	0.01325423	0.93469860
H	-1.71931385	-0.02487092	-2.43428052
H	1.24233163	-0.70075832	-2.48953168
C	-1.32343843	0.93207371	1.86233835
H	-0.58245787	1.69296756	1.68665696
H	-2.19275049	1.39408917	2.26698144
H	-0.99163464	0.19057537	2.56135603
H	0.89902159	0.52521010	1.73602437
C	3.82986365	0.39610060	1.23447636
H	3.72003563	0.01694181	2.25207622
H	4.49994296	-0.32489971	0.75534024
H	4.37061950	1.33274316	1.25211365
H	-1.33774501	-1.66943099	-2.00473546

Time = 267.00 fs
E(CASSCF/6-31G(d)) = -479.4897360

C	-0.86082488	-0.15127157	-0.46795837
C	0.47058172	0.12990991	-0.51674964
C	1.46950561	-0.35223458	-1.52956936
C	1.30094499	0.57286285	0.74789504
C	2.61010337	0.47705712	0.41710115
C	2.63645790	-0.05706416	-1.01620236
N	-1.69131089	0.26395469	0.57974372
C	-2.89130833	-0.49892630	0.53893021
H	3.57947686	-0.08016387	-1.54353996
C	-1.72192755	-0.72756622	-1.63862862
C	-3.14118436	-0.83156546	-0.95637987
H	-3.93526148	-0.22248775	-1.36508140
H	-3.45426818	-1.86351247	-1.08771241
H	-2.84090272	-1.43086549	1.12607239
H	-3.78127551	0.01344733	0.93375968
H	-1.71950379	-0.00170013	-2.42735494
H	1.23760575	-0.72255029	-2.47847547
C	-1.31948306	0.92568392	1.86215272
H	-0.57941030	1.68521412	1.69594991
H	-2.18546197	1.39296286	2.25999535
H	-1.00478282	0.18575501	2.56463462
H	0.93436015	0.55319105	1.74735525
C	3.82895917	0.40004518	1.23348173
H	3.69534473	0.03581698	2.25727837
H	4.49569425	-0.33564190	0.77169710
H	4.37545488	1.33427496	1.24700339
H	-1.34225385	-1.65560313	-2.02697805

Time = 267.50 fs

E(CASSCF/6-31G(d)) = -479.4896051

C	-0.86217710	-0.15943605	-0.46737803
C	0.47414702	0.13147418	-0.51103057
C	1.46850407	-0.36246578	-1.52015895
C	1.30541407	0.58582856	0.74539596
C	2.61322216	0.48268003	0.41190528
C	2.63820027	-0.06157884	-1.02021300
N	-1.69404650	0.25636582	0.58085424
C	-2.89076236	-0.50105203	0.53867064
H	3.58318224	-0.08702737	-1.52767965
C	-1.72569362	-0.71935856	-1.64076433
C	-3.14647357	-0.82697625	-0.95968536
H	-3.93792205	-0.20769940	-1.36026894
H	-3.45749040	-1.85646174	-1.10860387
H	-2.86118023	-1.44432389	1.12182522
H	-3.78066982	0.01416170	0.93280901
H	-1.71970072	0.02188191	-2.42135616
H	1.23247287	-0.74500937	-2.46988160
C	-1.31562841	0.91912340	1.86158040
H	-0.57658400	1.67745448	1.70535648
H	-2.18010863	1.39254102	2.25331212
H	-1.01669002	0.18038860	2.56927238
H	0.97002282	0.58194115	1.75846608
C	3.82831960	0.40390000	1.23258546
H	3.66996974	0.05573156	2.26191914
H	4.49131450	-0.34577887	0.78835505
H	4.38045673	1.33615919	1.24236894
H	-1.34679662	-1.64236654	-2.04891001

Time = 268.00 fs
E(CASSCF/6-31G(d)) = -479.4895262

C	-0.86337009	-0.16740745	-0.46679032
C	0.47777723	0.13284243	-0.50495933
C	1.46691265	-0.37243847	-1.51048074
C	1.30983813	0.59854197	0.74249557
C	2.61606324	0.48802081	0.40622556
C	2.64069072	-0.06581280	-1.02388904
N	-1.69676706	0.24888867	0.58194642
C	-2.89062292	-0.50339213	0.53841907
H	3.58713949	-0.09494907	-1.51232200
C	-1.72954151	-0.71087280	-1.64254671
C	-3.15154936	-0.82260005	-0.96300818
H	-3.93962880	-0.19221268	-1.35612065
H	-3.46156856	-1.84921913	-1.12866014
H	-2.88066263	-1.45684685	1.11707838

H	-3.77849459	0.01537980	0.93184084
H	-1.71993446	0.04562380	-2.41600525
H	1.22699884	-0.76793719	-2.46318225
C	-1.31191651	0.91238713	1.86063031
H	-0.57374724	1.66973711	1.71469268
H	-2.17679018	1.39287236	2.24697765
H	-1.02720192	0.17433021	2.57532372
H	1.00566813	0.61140373	1.76908330
C	3.82792590	0.40767082	1.23181913
H	3.64414903	0.07672933	2.26576436
H	4.48679637	-0.35528187	0.80531195
H	4.38563087	1.33827751	1.23819127
H	-1.35149049	-1.62956376	-2.07041377

Time = 268.50 fs
E(CASSCF/6-31G(d)) = -479.4894702

C	-0.86435412	-0.17515346	-0.46616920
C	0.48139570	0.13399441	-0.49854654
C	1.46474349	-0.38217521	-1.50062067
C	1.31424287	0.61100409	0.73923881
C	2.61863435	0.49307954	0.40004359
C	2.64388346	-0.06975745	-1.02717111
N	-1.69942531	0.24152866	0.58301625
C	-2.89094560	-0.50598967	0.53817810
H	3.59179418	-0.10403279	-1.49799638
C	-1.73345606	-0.70210986	-1.64402586
C	-3.15639869	-0.81841270	-0.96635359
H	-3.94032598	-0.17614974	-1.35263675
H	-3.46657576	-1.84181547	-1.14775570
H	-2.89912219	-1.46807530	1.11172603
H	-3.77477893	0.01710321	0.93087597
H	-1.72023820	0.06922362	-2.41098108
H	1.22128968	-0.79105039	-2.45763163
C	-1.30839645	0.90546915	1.85932832
H	-0.57068401	1.66210287	1.72377724
H	-2.17542634	1.39390747	2.24097038
H	-1.03624595	0.16756388	2.58271571
H	1.04097711	0.64151087	1.77885261
C	3.82775367	0.41136944	1.23121045
H	3.61813487	0.09882419	2.26860902
H	4.48214510	-0.36414026	0.82256244
H	4.39096783	1.34049370	1.23444738
H	-1.35646608	-1.61697454	-2.09134142

Time = 269.00 fs
E(CASSCF/6-31G(d)) = -479.4894234

C	-0.86508582	-0.18264262	-0.46548788
C	0.48492968	0.13491092	-0.49180598
C	1.46202392	-0.39170000	-1.49066565
C	1.31865110	0.62321810	0.73567676
C	2.62095288	0.49785702	0.39334658
C	2.64771740	-0.07341023	-1.03001349
N	-1.70197524	0.23428958	0.58405893
C	-2.89177525	-0.50888077	0.53794442
H	3.59755466	-0.11437346	-1.48517263
C	-1.73741977	-0.69307452	-1.64525463
C	-3.16101216	-0.81438711	-0.96972608
H	-3.93995416	-0.15965505	-1.34980153
H	-3.47256996	-1.83427473	-1.16578522
H	-2.91634689	-1.47773198	1.10571131
H	-3.76963442	0.01935154	0.92996011
H	-1.72064858	0.09236038	-2.40595271
H	1.21546779	-0.81401738	-2.45240809
C	-1.30512176	0.89836378	1.85771595
H	-0.56721392	1.65457098	1.73243756
H	-2.17577217	1.39550428	2.23521172
H	-1.04382398	0.16020033	2.59124916
H	1.07564420	0.67217940	1.78736707
C	3.82777350	0.41501316	1.23078192
H	3.59218589	0.12199806	2.27029032
H	4.47737728	-0.37236208	0.84009860
H	4.39644786	1.34266353	1.23111018
H	-1.36185711	-1.60434267	-2.11152361

Time = 269.50 fs
E(CASSCF/6-31G(d)) = -479.4893938

C	-0.86552902	-0.18984468	-0.46471909
C	0.48831172	0.13557415	-0.48475457
C	1.45879730	-0.40103495	-1.48069614
C	1.32308315	0.63518916	0.73186304
C	2.62304549	0.50235483	0.38612791
C	2.65212188	-0.07677493	-1.03238627
N	-1.70437306	0.22717327	0.58506901
C	-2.89314486	-0.51209275	0.53770867
H	3.60472898	-0.12605322	-1.47422813
C	-1.74141391	-0.68377554	-1.64628495
C	-3.16538357	-0.81049403	-0.97312903
H	-3.93845862	-0.14288795	-1.34758753
H	-3.47959239	-1.82661329	-1.18266416
H	-2.93214762	-1.48563503	1.09903093
H	-3.76325057	0.02216070	0.92916157

H	-1.72120462	0.11472559	-2.40061023
H	1.20965094	-0.83649639	-2.44670607
C	-1.30214692	0.89106710	1.85584695
H	-0.56320998	1.64712672	1.74051411
H	-2.17745239	1.39744527	2.22958357
H	-1.04999811	0.15245558	2.60061603
H	1.10936998	0.70331126	1.79420297
C	3.82795176	0.41862382	1.23054982
H	3.56655945	0.14620083	2.27069710
H	4.47251860	-0.37997289	0.85791056
H	4.40204616	1.34464497	1.22814815
H	-1.36778794	-1.59140628	-2.13078284

Time = 270.00 fs
E(CASSCF/6-31G(d)) = -479.4894398

C	-0.86565530	-0.19673103	-0.46383558
C	0.49148083	0.13596791	-0.47741219
C	1.45512244	-0.41019766	-1.47078078
C	1.32755711	0.64692400	0.72785005
C	2.62494744	0.50657534	0.37838739
C	2.65702293	-0.07986200	-1.03427661
N	-1.70657824	0.22017943	0.58604054
C	-2.89507489	-0.51564271	0.53745562
H	3.61346786	-0.13913589	-1.46542255
C	-1.74541940	-0.67422555	-1.64716435
C	-3.16950935	-0.80670302	-0.97656435
H	-3.93579755	-0.12601453	-1.34595947
H	-3.48766594	-1.81883937	-1.19832886
H	-2.94636479	-1.49170585	1.09173615
H	-3.75588492	0.02557893	0.92856699
H	-1.72194657	0.13605045	-2.39468811
H	1.20393548	-0.85816830	-2.43980756
C	-1.29952337	0.88357854	1.85378288
H	-0.55861127	1.63971282	1.74786328
H	-2.18000822	1.39946296	2.22394927
H	-1.05487379	0.14461676	2.61042968
H	1.14185671	0.73479563	1.79895808
C	3.82825151	0.42222662	1.23052355
H	3.54150407	0.17135213	2.26977624
H	4.46760059	-0.38701328	0.87598763
H	4.40773842	1.34630789	1.22552508
H	-1.37436268	-1.57792495	-2.14894756

Time = 270.50 fs
E(CASSCF/6-31G(d)) = -479.4896552

C	-0.86544425	-0.20327507	-0.46281066
C	0.49438346	0.13607792	-0.46980137
C	1.45107210	-0.41919942	-1.46097324
C	1.33208902	0.65843043	0.72368542
C	2.62670169	0.51052183	0.37013159
C	2.66234857	-0.08268863	-1.03568886
N	-1.70855458	0.21330534	0.58696722
C	-2.89757332	-0.51953628	0.53716476
H	3.62372668	-0.15366000	-1.45888415
C	-1.74941771	-0.66444088	-1.64793336
C	-3.17338796	-0.80298334	-0.98003222
H	-3.93194971	-0.10919912	-1.34487787
H	-3.49679407	-1.81095296	-1.21273637
H	-2.95887397	-1.49597300	1.08393175
H	-3.74784779	0.02966003	0.92827525
H	-1.72291442	0.15612723	-2.38798152
H	1.19838525	-0.87876185	-2.43113034
C	-1.29729590	0.87590214	1.85158815
H	-0.55342868	1.63222668	1.75435875
H	-2.18294736	1.40126829	2.21817389
H	-1.05858301	0.13700246	2.62026178
H	1.17280788	0.76651316	1.80128696
C	3.82863362	0.42584879	1.23070531
H	3.51725196	0.19734459	2.26753579
H	4.46265670	-0.39353511	0.89431973
H	4.41350577	1.34754250	1.22319993
H	-1.38165648	-1.56370218	-2.16586608

Time = 271.00 fs
E(CASSCF/6-31G(d)) = -479.4901369

C	-0.86488362	-0.20945256	-0.46161863
C	0.49697428	0.13589194	-0.46194700
C	1.44673054	-0.42804411	-1.45131068
C	1.33669275	0.66971657	0.71940906
C	2.62835762	0.51419869	0.36137409
C	2.66803224	-0.08527889	-1.03664365
N	-1.71027107	0.20654574	0.58784257
C	-2.90063626	-0.52376648	0.53681109
H	3.63525566	-0.16963135	-1.45461001
C	-1.75339143	-0.65444125	-1.64862342
C	-3.17701945	-0.79930483	-0.98353080
H	-3.92692090	-0.09259600	-1.34430213
H	-3.50696012	-1.80294612	-1.22586388
H	-2.96959021	-1.49857254	1.07577348
H	-3.73948181	0.03445389	0.92838950
H	-1.72414657	0.17482478	-2.38035632

H	1.19302567	-0.89807132	-2.42025632
C	-1.29549990	0.86804732	1.84932556
H	-0.54774291	1.62452425	1.75989144
H	-2.18578975	1.40257771	2.21214113
H	-1.06126993	0.12992457	2.62967953
H	1.20193107	0.79834212	1.80092986
C	3.82905801	0.42951817	1.23109005
H	3.49401252	0.22404827	2.26404521
H	4.45771794	-0.39959672	0.91289886
H	4.41933917	1.34826601	1.22112685
H	-1.38970931	-1.54860214	-2.18141894

Time = 271.50 fs
E(CASSCF/6-31G(d)) = -479.4909586

C	-0.86396901	-0.21524176	-0.46023542
C	0.49921656	0.13540007	-0.45387602
C	1.44219112	-0.43672777	-1.44181343
C	1.34138004	0.68079004	0.71505079
C	2.62996953	0.51761174	0.35213591
C	2.67401354	-0.08766379	-1.03717605
N	-1.71170266	0.19989276	0.58866005
C	-2.90424948	-0.52831276	0.53636614
H	3.64762173	-0.18701526	-1.45247884
C	-1.75732475	-0.64424964	-1.64925559
C	-3.18040501	-0.79563875	-0.98705617
H	-3.92074863	-0.07634252	-1.34419286
H	-3.51812679	-1.79480372	-1.23770773
H	-2.97847110	-1.49974564	1.06746421
H	-3.73113765	0.03999373	0.92900772
H	-1.72567828	0.19209784	-2.37175334
H	1.18784006	-0.91596838	-2.40694619
C	-1.29415960	0.86002889	1.84705286
H	-0.54169542	1.61642988	1.76436879
H	-2.18810464	1.40313568	2.20576541
H	-1.06307977	0.12365679	2.63827829
H	1.22894295	0.83016381	1.79773456
C	3.82948489	0.43326187	1.23166570
H	3.47196724	0.25131548	2.25943307
H	4.45280876	-0.40525747	0.93171963
H	4.42524291	1.34842724	1.21925531
H	-1.39852261	-1.53256030	-2.19552863

Time = 272.00 fs
E(CASSCF/6-31G(d)) = -479.4921301

C	-0.86270431	-0.22062379	-0.45863885

C	0.50108324	0.13459471	-0.44561692
C	1.43755197	-0.44523849	-1.43248552
C	1.34615981	0.69165726	0.71062898
C	2.63159526	0.52076812	0.34244513
C	2.68023814	-0.08988062	-1.03733243
N	-1.71283077	0.19333581	0.58941314
C	-2.90838986	-0.53314019	0.53579932
H	3.66025422	-0.20573550	-1.45227499
C	-1.76120357	-0.63389187	-1.64983969
C	-3.18354670	-0.79195843	-0.99060208
H	-3.91350484	-0.06055224	-1.34451354
H	-3.53023667	-1.78650461	-1.24828293
H	-2.98551788	-1.49983070	1.05924745
H	-3.72314665	0.04628166	0.93021190
H	-1.72753990	0.20799361	-2.36218963
H	1.18277291	-0.93240972	-2.39114348
C	-1.29328757	0.85186619	1.84481972
H	-0.53547187	1.60775279	1.76771293
H	-2.18953856	1.40273276	2.19899944
H	-1.06415062	0.11840979	2.64570879
H	1.25357785	0.86187191	1.79166869
C	3.82987606	0.43710498	1.23241381
H	3.45126473	0.27898742	2.25388011
H	4.44794253	-0.41057151	0.95078089
H	4.43123588	1.34800848	1.21753042
H	-1.40805691	-1.51558981	-2.20816804

Time = 272.50 fs
E(CASSCF/6-31G(d)) = -479.4935962

C	-0.86110061	-0.22558277	-0.45680937
C	0.50255624	0.13347093	-0.43719932
C	1.43291486	-0.45355662	-1.42331527
C	1.35103831	0.70232228	0.70614945
C	2.63329431	0.52367720	0.33233780
C	2.68665406	-0.09197286	-1.03716848
N	-1.71364389	0.18686203	0.59009555
C	-2.91302746	-0.53819954	0.53507948
H	3.67250627	-0.22567290	-1.45371378
C	-1.76501561	-0.62339662	-1.65037414
C	-3.18644733	-0.78823983	-0.99415994
H	-3.90529653	-0.04531004	-1.34523185
H	-3.54321342	-1.77802378	-1.25762276
H	-2.99077551	-1.49925155	1.05139863
H	-3.71579684	0.05327401	0.93205834
H	-1.72975598	0.22265231	-2.35175419
H	1.17772936	-0.94744258	-2.37297113

C	-1.29288493	0.84358192	1.84266602
H	-0.52928279	1.59830477	1.76985995
H	-2.18983402	1.40121922	2.19183878
H	-1.06460793	0.11431555	2.65169832
H	1.27559606	0.89337982	1.78282464
C	3.83019614	0.44106956	1.23331038
H	3.43201761	0.30690180	2.24761043
H	4.44311701	-0.41558306	0.97008623
H	4.43735292	1.34702427	1.21589269
H	-1.41823281	-1.49778219	-2.21936610

Time = 273.00 fs
E(CASSCF/6-31G(d)) = -479.4952864

C	-0.85917718	-0.23010587	-0.45473010
C	0.50362827	0.13202626	-0.42865354
C	1.42837989	-0.46165516	-1.41427671
C	1.35601858	0.71278667	0.70160555
C	2.63512619	0.52634981	0.32185666
C	2.69321075	-0.09398849	-1.03674498
N	-1.71413736	0.18045610	0.59070131
C	-2.91812769	-0.54342831	0.53417670
H	3.68372034	-0.24667270	-1.45647430
C	-1.76875024	-0.61279491	-1.65084640
C	-3.18911037	-0.78446200	-0.99771889
H	-3.89626373	-0.03066964	-1.34631870
H	-3.55696213	-1.76933210	-1.26577688
H	-2.99432883	-1.49849087	1.04421025
H	-3.70930948	0.06086824	0.93456852
H	-1.73234365	0.23630184	-2.34060074
H	1.17258093	-0.96120558	-2.35272606
C	-1.29294237	0.83520050	1.84062063
H	-0.52334407	1.58791837	1.77075840
H	-2.18884018	1.39851166	2.18432077
H	-1.06456204	0.11141915	2.65606114
H	1.29479110	0.92462709	1.77142085
C	3.83041345	0.44517388	1.23432711
H	3.41430178	0.33489806	2.24088014
H	4.43831335	-0.42032102	0.98964369
H	4.44364301	1.34551940	1.21427958
H	-1.42893398	-1.47930208	-2.22920859

Time = 273.50 fs
E(CASSCF/6-31G(d)) = -479.4970295

C	-0.85695983	-0.23418371	-0.45238767
C	0.50430194	0.13026116	-0.42001019

C	1.42404482	-0.46950030	-1.40533138
C	1.36110065	0.72304844	0.69697839
C	2.63714856	0.52879924	0.31105226
C	2.69985550	-0.09597938	-1.03612628
N	-1.71431372	0.17410112	0.59122514
C	-2.92365313	-0.54875325	0.53306421
H	3.69328352	-0.26855003	-1.46022164
C	-1.77239827	-0.60211974	-1.65123417
C	-3.19154017	-0.78060730	-1.00126597
H	-3.88657688	-0.01665380	-1.34774872
H	-3.57137051	-1.76039967	-1.27281020
H	-2.99629803	-1.49805994	1.03797407
H	-3.70382964	0.06889479	0.93772421
H	-1.73531208	0.24924632	-2.32893814
H	1.16716872	-0.97392600	-2.33086262
C	-1.29344125	0.82674668	1.83870128
H	-0.51785779	1.57646406	1.77036957
H	-2.18651829	1.39459887	2.17652530
H	-1.06410500	0.10967566	2.65870765
H	1.31099658	0.95558327	1.75779259
C	3.83050069	0.44943195	1.23543267
H	3.39815882	0.36282221	2.23396383
H	4.43349506	-0.42479548	1.00946221
H	4.45016647	1.34356377	1.21262615
H	-1.44001125	-1.46037931	-2.23783576

Time = 274.00 fs
E(CASSCF/6-31G(d)) = -479.4987159

C	-0.85448115	-0.23781006	-0.44977228
C	0.50458997	0.12817906	-0.41129975
C	1.42000285	-0.47705273	-1.39643069
C	1.36628171	0.73310154	0.69223791
C	2.63941550	0.53104145	0.29998270
C	2.70653150	-0.09800018	-1.03537823
N	-1.71418237	0.16777877	0.59166261
C	-2.92956457	-0.55409465	0.53172057
H	3.70067362	-0.29110058	-1.46462936
C	-1.77595156	-0.59140576	-1.65150726
C	-3.19374206	-0.77666135	-1.00478635
H	-3.87643053	-0.00325463	-1.34949828
H	-3.58631148	-1.75119675	-1.27880184
H	-2.99683111	-1.49845126	1.03295617
H	-3.69942185	0.07711758	0.94146668
H	-1.73866219	0.26184915	-2.31701205
H	1.16130940	-0.98591247	-2.30796620
C	-1.29435502	0.81824403	1.83691410

H	-0.51299650	1.56386176	1.76866672
H	-2.18294223	1.38954331	2.16857061
H	-1.06331045	0.10895150	2.65964705
H	1.32409209	0.98625338	1.74237692
C	3.83043565	0.45385359	1.23659432
H	3.38359796	0.39053425	2.22713904
H	4.42860779	-0.42899755	1.02955182
H	4.45699235	1.34124449	1.21086582
H	-1.45129012	-1.44129357	-2.24543762

Time = 274.50 fs
E(CASSCF/6-31G(d)) = -479.5002173

C	-0.85178001	-0.24098199	-0.44687797
C	0.50451522	0.12578658	-0.40255217
C	1.41634023	-0.48426950	-1.38751994
C	1.37155611	0.74293577	0.68734505
C	2.64197607	0.53309525	0.28871330
C	2.71317713	-0.10010694	-1.03456628
N	-1.71375920	0.16146993	0.59201036
C	-2.93582171	-0.55937191	0.53013167
H	3.70548943	-0.31411207	-1.46939669
C	-1.77940236	-0.58068839	-1.65163032
C	-3.19572271	-0.77261292	-1.00826360
H	-3.86603676	0.00956234	-1.35154395
H	-3.60164510	-1.74169606	-1.28384359
H	-2.99609552	-1.50008611	1.02936905
H	-3.69607810	0.08524197	0.94570014
H	-1.74238649	0.27450693	-2.30508522
H	1.15480701	-0.99753611	-2.28471099
C	-1.29565066	0.80971365	1.83525398
H	-0.50889060	1.55009010	1.76563528
H	-2.17829481	1.38348027	2.16060960
H	-1.06223264	0.10902964	2.65898623
H	1.33400767	1.01667781	1.72568730
C	3.83020132	0.45844480	1.23777949
H	3.37060169	0.41791163	2.22066982
H	4.42358171	-0.43289952	1.04992024
H	4.46419250	1.33865738	1.20893182
H	-1.46257900	-1.42235522	-2.25224302

Time = 275.00 fs
E(CASSCF/6-31G(d)) = -479.5014719

C	-0.84890115	-0.24369984	-0.44370280
C	0.50411061	0.12309362	-0.39379653
C	1.41313416	-0.49110702	-1.37854364

C	1.37691554	0.75253704	0.68225488
C	2.64487308	0.53498242	0.27731624
C	2.71972568	-0.10235582	-1.03375399
N	-1.71306609	0.15515517	0.59226644
C	-2.94238319	-0.56451007	0.52829249
H	3.70746979	-0.33737547	-1.47426181
C	-1.78274268	-0.57000275	-1.65156635
C	-3.19749058	-0.76845345	-1.01168015
H	-3.85561698	0.02185237	-1.35386030
H	-3.61722085	-1.73187470	-1.28803825
H	-2.99427018	-1.50326104	1.02734351
H	-3.69373287	0.09293082	0.95029917
H	-1.74646935	0.28761609	-2.29341286
H	1.14746853	-1.00920039	-2.26180042
C	-1.29729011	0.80117336	1.83370538
H	-0.50562051	1.53519043	1.76127304
H	-2.17285869	1.37661315	2.15282415
H	-1.06090670	0.10961886	2.65692386
H	1.34072603	1.04692816	1.70827840
C	3.82978592	0.46320846	1.23895744
H	3.35913256	0.44485174	2.21479103
H	4.41833520	-0.43645734	1.07056979
H	4.47183533	1.33589745	1.20675879
H	-1.47367952	-1.40388090	-2.25850429

Time = 275.50 fs
E(CASSCF/6-31G(d)) = -479.5024752

C	-0.84589440	-0.24596708	-0.44024896
C	0.50341870	0.12011336	-0.38506069
C	1.41045023	-0.49752513	-1.36945214
C	1.38234903	0.76188820	0.67692089
C	2.64814240	0.53672766	0.26586954
C	2.72610571	-0.10480162	-1.03300163
N	-1.71213041	0.14881544	0.59243054
C	-2.94920612	-0.56944579	0.52620833
H	3.70650141	-0.36069721	-1.47901150
C	-1.78596347	-0.55938250	-1.65128073
C	-3.19905623	-0.76417650	-1.01501771
H	-3.84539287	0.03368456	-1.35641764
H	-3.63288096	-1.72171670	-1.29149851
H	-2.99153964	-1.50810495	1.02690580
H	-3.69228318	0.09982671	0.95511919
H	-1.75088825	0.30153444	-2.28221568
H	1.13912656	-1.02129867	-2.23988984
C	-1.29923206	0.79263690	1.83224349
H	-0.50321170	1.51926739	1.75559026

H	-2.16700068	1.36920488	2.14541787
H	-1.05934949	0.11036724	2.65374024
H	1.34428481	1.07709833	1.69069731
C	3.82918274	0.46814529	1.24010063
H	3.34913977	0.47127449	2.20969307
H	4.41277834	-0.43961512	1.09149538
H	4.47997871	1.33304870	1.20428414
H	-1.48439800	-1.38616569	-2.26447849

Time = 276.00 fs
E(CASSCF/6-31G(d)) = -479.5032869

C	-0.84281391	-0.24779033	-0.43652284
C	0.50249097	0.11686227	-0.37637116
C	1.40834002	-0.50349171	-1.36020898
C	1.38784289	0.77097058	0.67130007
C	2.65181252	0.53835866	0.25445632
C	2.73224185	-0.10749667	-1.03236496
N	-1.71098453	0.14243263	0.59250439
C	-2.95624537	-0.57413195	0.52389492
H	3.70262139	-0.38390852	-1.48348895
C	-1.78905389	-0.54885855	-1.65074536
C	-3.20043277	-0.75977704	-1.01825783
H	-3.83557763	0.04513283	-1.35917969
H	-3.64846288	-1.71121508	-1.29434503
H	-2.98809100	-1.51455648	1.02796524
H	-3.69160905	0.10557574	0.96000730
H	-1.75561402	0.31654118	-2.27165531
H	1.12966380	-1.03416286	-2.21950505
C	-1.30143375	0.78411386	1.83083642
H	-0.50163585	1.50248451	1.74861038
H	-2.16114776	1.36156269	2.13860647
H	-1.05756203	0.11088124	2.64977938
H	1.34477529	1.10728728	1.67343152
C	3.82838984	0.47325495	1.24118593
H	3.34056694	0.49712108	2.20551130
H	4.40681861	-0.44230986	1.11268103
H	4.48866309	1.33017581	1.20144990
H	-1.49455669	-1.36945443	-2.27040504

Time = 276.50 fs
E(CASSCF/6-31G(d)) = -479.5039922

C	-0.83971719	-0.24917920	-0.43253512
C	0.50138685	0.11335992	-0.36775290
C	1.40683884	-0.50898731	-1.35079727
C	1.39338084	0.77976594	0.66535811

C	2.65590445	0.53990592	0.24316331
C	2.73805564	-0.11048952	-1.03189402
N	-1.70966527	0.13599019	0.59249208
C	-2.96345281	-0.57853976	0.52137748
H	3.69601267	-0.40687548	-1.48759865
C	-1.79200070	-0.53845807	-1.64994232
C	-3.20163611	-0.75525062	-1.02138246
H	-3.82636716	0.05626712	-1.36210131
H	-3.66380252	-1.70037398	-1.29670482
H	-2.98411327	-1.52236838	1.03031598
H	-3.69159206	0.10985163	0.96481349
H	-1.76061178	0.33280198	-2.26181402
H	1.11903896	-1.04801317	-2.20096840
C	-1.30385320	0.77560977	1.82944798
H	-0.50081556	1.48505597	1.74037054
H	-2.15575378	1.35401637	2.13260525
H	-1.05553409	0.11075022	2.64542412
H	1.34234159	1.13757828	1.65685319
C	3.82740984	0.47853705	1.24219540
H	3.33335786	0.52235326	2.20231878
H	4.40036561	-0.44447754	1.13409833
H	4.49790547	1.32731729	1.19820458
H	-1.50400408	-1.35391515	-2.27648361

Time = 277.00 fs
E(CASSCF/6-31G(d)) = -479.5046506

C	-0.83666387	-0.25014605	-0.42830062
C	0.50017252	0.10962872	-0.35922936
C	1.40596382	-0.51400869	-1.34122367
C	1.39894378	0.78825877	0.65907405
C	2.66043192	0.54140229	0.23207917
C	2.74346702	-0.11382366	-1.03163194
N	-1.70821343	0.12947349	0.59240038
C	-2.97077671	-0.58265840	0.51868866
H	3.68699500	-0.42950682	-1.49130943
C	-1.79478801	-0.52820386	-1.64886649
C	-3.20268509	-0.75059272	-1.02437448
H	-3.81793286	0.06714511	-1.36512664
H	-3.67873746	-1.68921028	-1.29870932
H	-2.97979806	-1.53113789	1.03365294
H	-3.69212991	0.11237674	0.96940038
H	-1.76584102	0.35034592	-2.25268471
H	1.10730704	-1.06291957	-2.18435348
C	-1.30645151	0.76712667	1.82804133
H	-0.50063389	1.46723427	1.73092256
H	-2.15125805	1.34688960	2.12761405

H	-1.05325099	0.10957623	2.64106359
H	1.33717973	1.16801491	1.64117406
C	3.82624952	0.48399206	1.24311660
H	3.32746047	0.54695119	2.20012446
H	4.39333614	-0.44605877	1.15570514
H	4.50769505	1.32448153	1.19450444
H	-1.51262262	-1.33961924	-2.28285483

Time = 277.50 fs
E(CASSCF/6-31G(d)) = -479.5052284

C	-0.83371341	-0.25070589	-0.42383866
C	0.49891938	0.10569333	-0.35082255
C	1.40571304	-0.51857039	-1.33151872
C	1.40450925	0.79643867	0.65244360
C	2.66540202	0.54288206	0.22129214
C	2.74839626	-0.11753613	-1.03161383
N	-1.70667342	0.12287040	0.59223912
C	-2.97816172	-0.58649262	0.51586559
H	3.67600793	-0.45176034	-1.49465383
C	-1.79739742	-0.51811432	-1.64752670
C	-3.20360139	-0.74579816	-1.02721825
H	-3.81041616	0.07780587	-1.36818861
H	-3.69311087	-1.67775536	-1.30049316
H	-2.97534105	-1.54035857	1.03759902
H	-3.69314895	0.11293896	0.97365196
H	-1.77125593	0.36905891	-2.24417265
H	1.09463021	-1.07878644	-2.16947869
C	-1.30919514	0.75866426	1.82658354
H	-0.50094558	1.44929667	1.72033470
H	-2.14803908	1.34046877	2.12380275
H	-1.05070172	0.10700672	2.63705795
H	1.32953895	1.19857998	1.62641607
C	3.82491944	0.48962191	1.24394255
H	3.32282737	0.57091250	2.19887490
H	4.38565713	-0.44700416	1.17744598
H	4.51799094	1.32164512	1.19031399
H	-1.52033281	-1.32653223	-2.28958870

Time = 278.00 fs
E(CASSCF/6-31G(d)) = -479.5056640

C	-0.83092471	-0.25087614	-0.41917263
C	0.49770230	0.10158042	-0.34255301
C	1.40606676	-0.52270356	-1.32173369
C	1.41005212	0.80430155	0.64547952
C	2.67081504	0.54438080	0.21088844

C	2.75276545	-0.12165710	-1.03186623
N	-1.70509274	0.11617153	0.59202134
C	-2.98554884	-0.59005911	0.51294671
H	3.66358845	-0.47364399	-1.49772291
C	-1.79980840	-0.50820376	-1.64594483
C	-3.20440922	-0.74086065	-1.02990001
H	-3.80392450	0.08826503	-1.37120868
H	-3.70677361	-1.66605531	-1.30219163
H	-2.97093935	-1.54947798	1.04173781
H	-3.69460946	0.11140397	0.97747985
H	-1.77680424	0.38869387	-2.23610733
H	1.08127076	-1.09536522	-2.15594714
C	-1.31205768	0.75022101	1.82504943
H	-0.50159561	1.43152601	1.70869329
H	-2.14636886	1.33496775	2.12129416
H	-1.04788980	0.10276698	2.63369824
H	1.31971856	1.22918169	1.61241302
C	3.82343384	0.49543026	1.24467114
H	3.31941611	0.59424923	2.19846299
H	4.37727000	-0.44727894	1.19925201
H	4.52872308	1.31875632	1.18560813
H	-1.52709327	-1.31451548	-2.29667854

Time = 278.50 fs
E(CASSCF/6-31G(d)) = -479.5058158

C	-0.82835405	-0.25067635	-0.41433000
C	0.49659776	0.09731804	-0.33443998
C	1.40698921	-0.52645293	-1.31193439
C	1.41554463	0.81185025	0.63821007
C	2.67666487	0.54593425	0.20095001
C	2.75650138	-0.12620932	-1.03240696
N	-1.70352152	0.10937048	0.59176343
C	-2.99287621	-0.59338262	0.50996862
H	3.65033843	-0.49521290	-1.50065556
C	-1.80199920	-0.49848316	-1.64415363
C	-3.20513480	-0.73577269	-1.03240824
H	-3.79853061	0.09851344	-1.37409831
H	-3.71958730	-1.65417141	-1.30393890
H	-2.96678882	-1.55795514	1.04564619
H	-3.69651022	0.10772256	0.98082803
H	-1.78242650	0.40889770	-2.22826527
H	1.06757349	-1.11229010	-2.14321509
C	-1.31502062	0.74179568	1.82342531
H	-0.50243664	1.41419309	1.69610559
H	-2.14637749	1.33050102	2.12015270
H	-1.04484226	0.09668710	2.63117440

H	1.30806646	1.25965122	1.59883593
C	3.82181035	0.50142230	1.24530438
H	3.31718596	0.61698613	2.19873844
H	4.36813272	-0.44686602	1.22104219
H	4.53979484	1.31574034	1.18037219
H	-1.53289660	-1.30334140	-2.30404622

Time = 279.00 fs
E(CASSCF/6-31G(d)) = -479.5055676

C	-0.82605332	-0.25012776	-0.40934218
C	0.49568181	0.09293499	-0.32650150
C	1.40843134	-0.52987252	-1.30219394
C	1.42095674	0.81909405	0.63067538
C	2.68293941	0.54757743	0.19155226
C	2.75953911	-0.13120820	-1.03324538
N	-1.70201197	0.10246401	0.59148516
C	-3.00008015	-0.59649240	0.50696346
H	3.63688235	-0.51655903	-1.50362140
C	-1.80394795	-0.48896108	-1.64219333
C	-3.20580562	-0.73052573	-1.03473385
H	-3.79427353	0.10851904	-1.37676161
H	-3.73142682	-1.64217925	-1.30586616
H	-2.96307901	-1.56530853	1.04892294
H	-3.69888922	0.10193341	0.98367590
H	-1.78805594	0.42924858	-2.22039610
H	1.05393767	-1.12912801	-2.13067470
C	-1.31807300	0.73338851	1.82171118
H	-0.50334748	1.39753905	1.68270249
H	-2.14803480	1.32706729	2.12037760
H	-1.04161637	0.08871913	2.62955397
H	1.29497416	1.28975273	1.58524209
C	3.82006961	0.50760439	1.24584738
H	3.31609359	0.63916040	2.19952066
H	4.35822048	-0.44576798	1.24272561
H	4.55108903	1.31250656	1.17460180
H	-1.53776276	-1.29271718	-2.31155541

Time = 279.50 fs
E(CASSCF/6-31G(d)) = -479.5048718

C	-0.82406862	-0.24925303	-0.40424415
C	0.49502827	0.08846024	-0.31875452
C	1.41033389	-0.53302092	-1.29258587
C	1.42625715	0.82604707	0.62292262
C	2.68962071	0.54934358	0.18276206
C	2.76182581	-0.13666254	-1.03438320

N	-1.70061790	0.09545199	0.59120949
C	-3.00709646	-0.59941933	0.50395699
H	3.62381934	-0.53779429	-1.50679826
C	-1.80563378	-0.47964439	-1.64010769
C	-3.20644956	-0.72511061	-1.03687036
H	-3.79116146	0.11823111	-1.37909855
H	-3.74218188	-1.63016687	-1.30809906
H	-2.95998731	-1.57115067	1.05121087
H	-3.70182103	0.09416120	0.98603874
H	-1.79361866	0.44929667	-2.21224909
H	1.04078488	-1.14543080	-2.11773179
C	-1.32120967	0.72500231	1.81992112
H	-0.50424961	1.38175987	1.66864142
H	-2.15115409	1.32454792	2.12190283
H	-1.03830211	0.07894078	2.62877628
H	1.28086640	1.31920435	1.57113537
C	3.81823503	0.51398337	1.24630729
H	3.31608846	0.66082067	2.20061344
H	4.34752627	-0.44400726	1.26420361
H	4.56247517	1.30895767	1.16830295
H	-1.54173097	-1.28231381	-2.31902931

Time = 280.00 fs
E(CASSCF/6-31G(d)) = -479.5037700

C	-0.82243869	-0.24807569	-0.39907415
C	0.49470684	0.08392243	-0.31121493
C	1.41263036	-0.53595730	-1.28317848
C	1.43141469	0.83272626	0.61500087
C	2.69668476	0.55126341	0.17463615
C	2.76332501	-0.14257582	-1.03581614
N	-1.69939395	0.08833731	0.59096246
C	-3.01386205	-0.60219343	0.50096741
H	3.61167110	-0.55902738	-1.51034440
C	-1.80703782	-0.47053896	-1.63794014
C	-3.20709407	-0.71951831	-1.03881386
H	-3.78917626	0.12758734	-1.38100954
H	-3.75175862	-1.61823218	-1.31075582
H	-2.95767228	-1.57521246	1.05221347
H	-3.70541144	0.08460969	0.98796635
H	-1.79903434	0.46860474	-2.20359628
H	1.02852842	-1.16078187	-2.10386720
C	-1.32442863	0.71664290	1.81808170
H	-0.50511974	1.36699532	1.65410775
H	-2.15541499	1.32272021	2.12460369
H	-1.03501966	0.06754568	2.62866266
H	1.26618772	1.34770519	1.55602705

C	3.81633235	0.52056563	1.24669219
H	3.31710812	0.68202830	2.20181877
H	4.33605968	-0.44162660	1.28537371
H	4.57381929	1.30499864	1.16149087
H	-1.54485081	-1.27179677	-2.32627303

Time = 280.50 fs
E(CASSCF/6-31G(d)) = -479.5024025

C	-0.82119360	-0.24661996	-0.39387320
C	0.49478081	0.07934947	-0.30389769
C	1.41524961	-0.53873825	-1.27403142
C	1.43639963	0.83914906	0.60695706
C	2.70410196	0.55336415	0.16721951
C	2.76402047	-0.14894789	-1.03753512
N	-1.69839493	0.08112586	0.59077261
C	-3.02031638	-0.60484187	0.49800485
H	3.60083466	-0.58033642	-1.51437167
C	-1.80814419	-0.46164986	-1.63573029
C	-3.20776517	-0.71374055	-1.04056305
H	-3.78827869	0.13652098	-1.38239981
H	-3.76007965	-1.60648011	-1.31394446
H	-2.95626879	-1.57735669	1.05170507
H	-3.70978943	0.07355059	0.98953810
H	-1.80421804	0.48678126	-2.19425142
H	1.01754793	-1.17483042	-2.08867840
C	-1.32772821	0.70831919	1.81622890
H	-0.50599380	1.35332473	1.63931543
H	-2.16040474	1.32128407	2.12830979
H	-1.03191104	0.05482056	2.62894336
H	1.25138561	1.37496364	1.53948835
C	3.81438929	0.52735629	1.24701024
H	3.31907536	0.70285544	2.20294954
H	4.32384606	-0.43868492	1.30613062
H	4.58499135	1.30054532	1.15418962
H	-1.54717452	-1.26085450	-2.33309282

Time = 281.00 fs
E(CASSCF/6-31G(d)) = -479.5009714

C	-0.82035350	-0.24491027	-0.38868460
C	0.49530562	0.07476815	-0.29681679
C	1.41811792	-0.54141557	-1.26519325
C	1.44118532	0.84533133	0.59883251
C	2.71183689	0.55566883	0.16054430
C	2.76391890	-0.15577635	-1.03952778
N	-1.69767511	0.07382621	0.59067061

C	-3.02640354	-0.60738689	0.49507157
H	3.59154852	-0.60174565	-1.51892374
C	-1.80894068	-0.45298171	-1.63351124
C	-3.20848664	-0.70777088	-1.04211911
H	-3.78841378	0.14496952	-1.38318409
H	-3.76708420	-1.59501799	-1.31776080
H	-2.95588293	-1.57758561	1.04953781
H	-3.71509443	0.06130601	0.99085484
H	-1.80908232	0.50350827	-2.18408216
H	1.00816914	-1.18731639	-2.07190267
C	-1.33110451	0.70004271	1.81440431
H	-0.50696390	1.34076867	1.62450448
H	-2.16566397	1.31989433	2.13282056
H	-1.02912912	0.04111613	2.62929134
H	1.23689354	1.40072344	1.52118867
C	3.81243508	0.53435827	1.24726890
H	3.32189562	0.72338698	2.20383925
H	4.31092341	-0.43525519	1.32637084
H	4.59587493	1.29553106	1.14643031
H	-1.54875065	-1.24922154	-2.33931637

Time = 281.50 fs
E(CASSCF/6-31G(d)) = -479.4996915

C	-0.81992830	-0.24297084	-0.38355328
C	0.49632744	0.07020376	-0.28998520
C	1.42116097	-0.54403492	-1.25670037
C	1.44574995	0.85128564	0.59066039
C	2.71984807	0.55819563	0.15462923
C	2.76305082	-0.16305752	-1.04177902
N	-1.69728716	0.06644915	0.59068857
C	-3.03207426	-0.60984389	0.49216270
H	3.58387832	-0.62320860	-1.52396509
C	-1.80941914	-0.44453865	-1.63130757
C	-3.20927934	-0.70160545	-1.04348572
H	-3.78951638	0.15288243	-1.38329075
H	-3.77272742	-1.58395118	-1.32228583
H	-2.95658831	-1.57604261	1.04564531
H	-3.72146018	0.04822760	0.99202748
H	-1.81353967	0.51856079	-2.17301757
H	1.00064890	-1.19808571	-2.05342724
C	-1.33454964	0.69182696	1.81265118
H	-0.50816737	1.32929652	1.60993617
H	-2.17073356	1.31819398	2.13792130
H	-1.02682509	0.02681489	2.62935918
H	1.22311319	1.42478581	1.50092267
C	3.81049989	0.54157162	1.24747438

H	3.32545686	0.74371982	2.20435097
H	4.29734023	-0.43142015	1.34599492
H	4.60637534	1.28991201	1.13824982
H	-1.54961889	-1.23669603	-2.34480754

Time = 282.00 fs
E(CASSCF/6-31G(d)) = -479.4987436

C	-0.81991736	-0.24082539	-0.37852503
C	0.49788175	0.06568007	-0.28341485
C	1.42430551	-0.54663488	-1.24857686
C	1.45007790	0.85701990	0.58246451
C	2.72808805	0.56095737	0.14947917
C	2.76146952	-0.17078661	-1.04427082
N	-1.69728121	0.05900739	0.59085922
C	-3.03728805	-0.61221938	0.48926748
H	3.57772552	-0.64460368	-1.52938294
C	-1.80957581	-0.43632436	-1.62913405
C	-3.21016063	-0.69524381	-1.04466898
H	-3.79151584	0.16022741	-1.38266523
H	-3.77697890	-1.57337798	-1.32758334
H	-2.95842368	-1.57301022	1.04004381
H	-3.72899546	0.03467165	0.99316246
H	-1.81750513	0.53181975	-2.16105133
H	0.99516330	-1.20710007	-2.03329127
C	-1.33805106	0.68368635	1.81101107
H	-0.50976935	1.31883857	1.59588530
H	-2.17519442	1.31584420	2.14339854
H	-1.02513647	0.01230067	2.62881362
H	1.21039859	1.44702621	1.47862352
C	3.80861442	0.54899293	1.24763135
H	3.32963088	0.76396230	2.20438204
H	4.28315245	-0.42726726	1.36491018
H	4.61642574	1.28367028	1.12968960
H	-1.54980665	-1.22315144	-2.34947781

Time = 282.50 fs
E(CASSCF/6-31G(d)) = -479.4982251

C	-0.82030943	-0.23849689	-0.37364574
C	0.49999201	0.06121913	-0.27711646
C	1.42748082	-0.54924650	-1.24083474
C	1.45416070	0.86253646	0.57425893
C	2.73650369	0.56396129	0.14508511
C	2.75924841	-0.17895688	-1.04698133
N	-1.69770387	0.05151507	0.59121515
C	-3.04201516	-0.61450933	0.48637082

H	3.57285528	-0.66574278	-1.53500126
C	-1.80941135	-0.42834213	-1.62699505
C	-3.21114394	-0.68868953	-1.04567742
H	-3.79434009	0.16699519	-1.38127293
H	-3.77982118	-1.56338487	-1.33369798
H	-2.96139237	-1.56890377	1.03283047
H	-3.73776403	0.02097358	0.99434625
H	-1.82089878	0.54327894	-2.14824124
H	0.99179916	-1.21444243	-2.01168198
C	-1.34159189	0.67563510	1.80952120
H	-0.51194137	1.30930127	1.58263045
H	-2.17869827	1.31254897	2.14905358
H	-1.02417644	-0.00206778	2.62736466
H	1.19904303	1.46740634	1.45436630
C	3.80680941	0.55661497	1.24774294
H	3.33427633	0.78423293	2.20386591
H	4.26842043	-0.42288345	1.38303313
H	4.62599157	1.27681481	1.12079418
H	-1.54932706	-1.20854287	-2.35329428

Time = 283.00 fs
E(CASSCF/6-31G(d)) = -479.4981340

C	-0.82108304	-0.23600723	-0.36896045
C	0.50266866	0.05684126	-0.27109943
C	1.43062005	-0.55189312	-1.23347454
C	1.45799776	0.86783173	0.56604807
C	2.74503651	0.56720878	0.14142461
C	2.75647736	-0.18755801	-1.04988360
N	-1.69859731	0.04398741	0.59178783
C	-3.04623824	-0.61669822	0.48345520
H	3.56893833	-0.68639074	-1.54060385
C	-1.80893076	-0.42059486	-1.62488452
C	-3.21223858	-0.68195066	-1.04652193
H	-3.79791878	0.17320225	-1.37910105
H	-3.78124808	-1.55404178	-1.34065348
H	-2.96546344	-1.56425741	1.02417740
H	-3.74776598	0.00742482	0.99563040
H	-1.82364814	0.55304626	-2.13470546
H	0.99054772	-1.22031966	-1.98892719
C	-1.34515182	0.66768613	1.80821282
H	-0.51483948	1.30058263	1.57044282
H	-2.18098896	1.30807315	2.15471337
H	-1.02402585	-0.01597742	2.62478728
H	1.18926876	1.48598142	1.42836378
C	3.80511512	0.56442657	1.24781091
H	3.33924301	0.80465887	2.20277101

H	4.25320565	-0.41835012	1.40029180
H	4.63507311	1.26938014	1.11160977
H	-1.54817827	-1.19290821	-2.35628392

Time = 283.50 fs
E(CASSCF/6-31G(d)) = -479.4983850

C	-0.82220750	-0.23337724	-0.36451225
C	0.50590852	0.05256504	-0.26537194
C	1.43366134	-0.55458999	-1.22648634
C	1.46159646	0.87289581	0.55782766
C	2.75362360	0.57069548	0.13846243
C	2.75325820	-0.19657460	-1.05294424
N	-1.69999817	0.03644048	0.59260656
C	-3.04995328	-0.61875952	0.48050279
H	3.56559688	-0.70629023	-1.54596099
C	-1.80814311	-0.41308491	-1.62278651
C	-3.21344955	-0.67503977	-1.04721597
H	-3.80218507	0.17889087	-1.37615943
H	-3.78126267	-1.54539810	-1.34845086
H	-2.97057586	-1.55970118	1.01432078
H	-3.75892323	-0.00574357	0.99702090
H	-1.82569047	0.56133829	-2.12061672
H	0.99130102	-1.22506124	-1.96548480
C	-1.34870835	0.65985010	1.80711007
H	-0.51858510	1.29258546	1.55957502
H	-2.18191633	1.30225468	2.16023853
H	-1.02472776	-0.02917605	2.62093633
H	1.18121900	1.50290318	1.40095748
C	3.80356099	0.57241270	1.24783611
H	3.34437811	0.82537064	2.20109763
H	4.23757011	-0.41373688	1.41662574
H	4.64370407	1.26142482	1.10218433
H	-1.54634410	-1.17636504	-2.35853231

Time = 284.00 fs
E(CASSCF/6-31G(d)) = -479.4988103

C	-0.82364401	-0.23062623	-0.36034116
C	0.50969463	0.04840727	-0.25994068
C	1.43654820	-0.55734502	-1.21985113
C	1.46497210	0.87771319	0.54958600
C	2.76219829	0.57441149	0.13615165
C	2.74970099	-0.20598410	-1.05612257
N	-1.70193664	0.02889064	0.59369760
C	-3.05316973	-0.62065796	0.47749762
H	3.56244959	-0.72519008	-1.55085543

C	-1.80706109	-0.40581404	-1.62067652
C	-3.21477771	-0.66797400	-1.04777540
H	-3.80707692	0.18412802	-1.37248033
H	-3.77987643	-1.53747928	-1.35706789
H	-2.97664429	-1.55592233	1.00354054
H	-3.77107297	-0.01837088	0.99847260
H	-1.82697385	0.56846946	-2.10619158
H	0.99385326	-1.22911227	-1.94192368
C	-1.35223812	0.65213457	1.80622974
H	-0.52324888	1.28522918	1.55025004
H	-2.18144212	1.29501196	2.16553018
H	-1.02628381	-0.04148289	2.61575527
H	1.17495514	1.51841769	1.37259897
C	3.80217504	0.58055489	1.24781900
H	3.34953237	0.84650113	2.19887104
H	4.22157265	-0.40909938	1.43198948
H	4.65195102	1.25302591	1.09256541
H	-1.54379570	-1.15910162	-2.36018121

Time = 284.50 fs
E(CASSCF/6-31G(d)) = -479.4992222

C	-0.82534715	-0.22777195	-0.35648301
C	0.51399627	0.04438287	-0.25481083
C	1.43922982	-0.56015984	-1.21354308
C	1.46814722	0.88226370	0.54130601
C	2.77069135	0.57834184	0.13443506
C	2.74592007	-0.21575583	-1.05937046
N	-1.70443558	0.02135429	0.59508309
C	-3.05590947	-0.62235391	0.47442792
H	3.55914817	-0.74286924	-1.55510377
C	-1.80570051	-0.39878297	-1.61852336
C	-3.21621993	-0.66077459	-1.04821856
H	-3.81253730	0.18900162	-1.36811768
H	-3.77710864	-1.53028526	-1.36645899
H	-2.98356793	-1.55361335	0.99213292
H	-3.78397143	-0.03036565	0.99989191
H	-1.82745814	0.57483259	-2.09167777
H	0.99791057	-1.23301571	-1.91889574
C	-1.35571817	0.64454353	1.80558120
H	-0.52884032	1.27845801	1.54265216
H	-2.17964165	1.28634743	2.17053494
H	-1.02865225	-0.05279512	2.60928048
H	1.17045884	1.53285543	1.34382477
C	3.80098351	0.58883186	1.24776028
H	3.35456738	0.86818060	2.19613295
H	4.20526791	-0.40447568	1.44635334

H	4.65990825	1.24427320	1.08279996
H	-1.54049496	-1.14136405	-2.36142007

Time = 285.00 fs
E(CASSCF/6-31G(d)) = -479.4994727

C	-0.82726718	-0.22483051	-0.35296817
C	0.51876986	0.04050508	-0.24998621
C	1.44166055	-0.56303184	-1.20753272
C	1.47115042	0.88652412	0.53296806
C	2.77903218	0.58246698	0.13324699
C	2.74203065	-0.22585045	-1.06263286
N	-1.70750943	0.01384773	0.59678010
C	-3.05820503	-0.62380957	0.47128841
H	3.55540596	-0.75915580	-1.55857156
C	-1.80407959	-0.39199079	-1.61629183
C	-3.21776938	-0.65346622	-1.04856628
H	-3.81851273	0.19361479	-1.36314520
H	-3.77298612	-1.52378962	-1.37655534
H	-2.99124016	-1.55340608	0.98037610
H	-3.79730604	-0.04169664	1.00114602
H	-1.82711542	0.58087029	-2.07733851
H	1.00311066	-1.23738073	-1.89709488
C	-1.35912684	0.63707710	1.80516656
H	-0.53530334	1.27224506	1.53691988
H	-2.17670149	1.27634752	2.17524761
H	-1.03174800	-0.06309123	2.60164149
H	1.16764076	1.54661206	1.31522244
C	3.80001055	0.59722043	1.24766162
H	3.35936266	0.89053024	2.19293157
H	4.18870655	-0.39988359	1.45970337
H	4.66768966	1.23526345	1.07293465
H	-1.53639948	-1.12343843	-2.36247143

Time = 285.50 fs
E(CASSCF/6-31G(d)) = -479.4994923

C	-0.82935210	-0.22181635	-0.34982046
C	0.52395994	0.03678542	-0.24546927
C	1.44379853	-0.56595777	-1.20179144
C	1.47401464	0.89047088	0.52455344
C	2.78715018	0.58676356	0.13251517
C	2.73814569	-0.23622015	-1.06584885
N	-1.71116338	0.00638691	0.59879976
C	-3.06009705	-0.62499627	0.46808226
H	3.55101781	-0.77394103	-1.56118346
C	-1.80221813	-0.38543392	-1.61394600

C	-3.21941585	-0.64607609	-1.04884183
H	-3.82495160	0.19807897	-1.35765374
H	-3.76754402	-1.51794062	-1.38726643
H	-2.99955772	-1.55579877	0.96849293
H	-3.81071547	-0.05237833	1.00207750
H	-1.82592923	0.58703833	-2.06343351
H	1.00905516	-1.24283330	-1.87720103
C	-1.36244459	0.62973177	1.80498118
H	-0.54251769	1.26659337	1.53314131
H	-2.17291145	1.26517822	2.17971217
H	-1.03544419	-0.07243093	2.59305690
H	1.16635575	1.56011828	1.28738711
C	3.79927788	0.60569658	1.24752621
H	3.36382223	0.91365704	2.18931126
H	4.17193512	-0.39532063	1.47204254
H	4.67542038	1.22609308	1.06301617
H	-1.53146912	-1.10562819	-2.36357252

Time = 286.00 fs
E(CASSCF/6-31G(d)) = -479.4993199

C	-0.83154995	-0.21874218	-0.34705617
C	0.52950056	0.03323374	-0.24126116
C	1.44560364	-0.56893852	-1.19629679
C	1.47677501	0.89408364	0.51604842
C	2.79497609	0.59120512	0.13216269
C	2.73437299	-0.24680940	-1.06895305
N	-1.71539256	-0.00101260	0.60114672
C	-3.06163170	-0.62590144	0.46482239
H	3.54587259	-0.78718784	-1.56292804
C	-1.80013667	-0.37910500	-1.61145311
C	-3.22114619	-0.63863283	-1.04907070
H	-3.83180139	0.20250605	-1.35174795
H	-3.76082729	-1.51266368	-1.39848228
H	-3.00842735	-1.56108932	0.95661895
H	-3.82381405	-0.06245390	1.00252152
H	-1.82389340	0.59376267	-2.05019925
H	1.01535278	-1.24995016	-1.85981247
C	-1.36565458	0.62250085	1.80501449
H	-0.55030582	1.26153344	1.53135246
H	-2.16865105	1.25307589	2.18401993
H	-1.03957561	-0.08095121	2.58382619
H	1.16642485	1.57379853	1.26087108
C	3.79880471	0.61423667	1.24735916
H	3.36788015	0.93764802	2.18530277
H	4.15499672	-0.39076475	1.48339117
H	4.68322651	1.21685142	1.05309198

H	-1.52567380	-1.08822852	-2.36495200

Time = 286.50 fs
E(CASSCF/6-31G(d)) = -479.4990776

C	-0.83381083	-0.21561918	-0.34468328
C	0.53531677	0.02985824	-0.23736188
C	1.44703550	-0.57198456	-1.19103810
C	1.47946645	0.89734940	0.50744844
C	2.80244347	0.59576288	0.13210995
C	2.73081223	-0.25755615	-1.07187725
N	-1.72018150	-0.00833557	0.60381868
C	-3.06285838	-0.62653378	0.46153146
H	3.53995966	-0.79893451	-1.56385870
C	-1.79785546	-0.37299166	-1.60878750
C	-3.22294474	-0.63116529	-1.04928039
H	-3.83900566	0.20700025	-1.34554257
H	-3.75289254	-1.50786527	-1.41007672
H	-3.01776828	-1.56932955	0.94478389
H	-3.83621718	-0.07197862	1.00232298
H	-1.82101060	0.60139459	-2.03783023
H	1.02166751	-1.25918377	-1.84537564
C	-1.36874335	0.61537538	1.80525121
H	-0.55844496	1.25711776	1.53153866
H	-2.16436873	1.24033198	2.18830405
H	-1.04394468	-0.08885752	2.57431641
H	1.16766151	1.58802173	1.23613187
C	3.79860765	0.62281872	1.24716774
H	3.37150444	0.96256486	2.18091546
H	4.13793219	-0.38617668	1.49378719
H	4.69122453	1.20761460	1.04321146
H	-1.51900225	-1.07150005	-2.36680442

Time = 287.00 fs
E(CASSCF/6-31G(d)) = -479.4989092

C	-0.83608893	-0.21245698	-0.34270108
C	0.54132655	0.02666564	-0.23377036
C	1.44805187	-0.57512092	-1.18602092
C	1.48212102	0.90026654	0.49876167
C	2.80949018	0.60040636	0.13227657
C	2.72755234	-0.26839332	-1.07455205
N	-1.72550372	-0.01556706	0.60680633
C	-3.06382790	-0.62692534	0.45824043
H	3.53337025	-0.80929393	-1.56409081
C	-1.79539351	-0.36707542	-1.60593418
C	-3.22479377	-0.62370131	-1.04949992

H	-3.84650061	0.21165016	-1.33915847
H	-3.74381010	-1.50343783	-1.42191146
H	-3.02750989	-1.58031291	0.93291243
H	-3.84756546	-0.08100544	1.00135199
H	-1.81729088	0.61017052	-2.02646420
H	1.02775955	-1.27079318	-1.83412770
C	-1.37170139	0.60834539	1.80567332
H	-0.56668342	1.25341238	1.53363836
H	-2.16055222	1.22727087	2.19272902
H	-1.04833048	-0.09640872	2.56494237
H	1.16989777	1.60305069	1.21348671
C	3.79870068	0.63142348	1.24696110
H	3.37469882	0.98843920	2.17613175
H	4.12078127	-0.38150257	1.50328606
H	4.69951066	1.19844092	1.03342706
H	-1.51147035	-1.05564516	-2.36926503

Time = 287.50 fs
E(CASSCF/6-31G(d)) = -479.4989039

C	-0.83834375	-0.20926394	-0.34110002
C	0.54744238	0.02366113	-0.23048463
C	1.44860910	-0.57839007	-1.18126928
C	1.48476617	0.90284817	0.49001108
C	2.81605964	0.60510407	0.13258312
C	2.72466873	-0.27925062	-1.07690851
N	-1.73132180	-0.02269226	0.61009363
C	-3.06459116	-0.62713017	0.45498549
H	3.52629254	-0.81844867	-1.56379615
C	-1.79276777	-0.36133125	-1.60289146
C	-3.22667383	-0.61626663	-1.04975933
H	-3.85421243	0.21652247	-1.33271882
H	-3.73366628	-1.49926593	-1.43384108
H	-3.03758674	-1.59359918	0.92084426
H	-3.85754537	-0.08957425	0.99951603
H	-1.81275078	0.62018425	-2.01617364
H	1.03351024	-1.28479797	-1.82606550
C	-1.37452391	0.60140134	1.80626271
H	-0.57476030	1.25048635	1.53754998
H	-2.15769083	1.21422106	2.19747566
H	-1.05250075	-0.10389538	2.55614073
H	1.17300500	1.61900088	1.19308315
C	3.79909522	0.64003540	1.24674988
H	3.37750255	1.01526971	2.17090560
H	4.10358393	-0.37667811	1.51196060
H	4.70815254	1.18936769	1.02379531
H	-1.50312810	-1.04079057	-2.37238998

Time = 288.00 fs
E(CASSCF/6-31G(d)) = -479.4990540

C	-0.84054110	-0.20604726	-0.33986191
C	0.55357308	0.02084828	-0.22750201
C	1.44866463	-0.58185154	-1.17682484
C	1.48742353	0.90512374	0.48123454
C	2.82210214	0.60982389	0.13295264
C	2.72222106	-0.29005655	-1.07887968
N	-1.73758760	-0.02969630	0.61365828
C	-3.06519828	-0.62722009	0.45180419
H	3.51900094	-0.82664134	-1.56319325
C	-1.78999246	-0.35572772	-1.59967217
C	-3.22856414	-0.60888392	-1.05008898
H	-3.86205514	0.22165726	-1.32634558
H	-3.72256502	-1.49523257	-1.44571833
H	-3.04793209	-1.60856979	0.90836783
H	-3.86590597	-0.09770442	0.99676864
H	-1.80741308	0.63137625	-2.00696491
H	1.03892096	-1.30096984	-1.82095134
C	-1.37721162	0.59453561	1.80700451
H	-0.58242679	1.24839945	1.54314032
H	-2.15623148	1.20148158	2.20272135
H	-1.05622700	-0.11161140	2.54833796
H	1.17690435	1.63581839	1.17489280
C	3.79980011	0.64864325	1.24654535
H	3.37998801	1.04302049	2.16516469
H	4.08638186	-0.37163261	1.51990019
H	4.71718293	1.18041045	1.01437743
H	-1.49406341	-1.02697903	-2.37614428

Time = 288.50 fs
E(CASSCF/6-31G(d)) = -479.4992609

C	-0.84265357	-0.20281334	-0.33896051
C	0.55962576	0.01822945	-0.22481932
C	1.44818126	-0.58557847	-1.17274302
C	1.49010874	0.90713824	0.47248297
C	2.82757584	0.61453353	0.13331198
C	2.72025170	-0.30074070	-1.08040237
N	-1.74424294	-0.03656411	0.61747253
C	-3.06569799	-0.62727857	0.44873118
H	3.51183726	-0.83415906	-1.56253343
C	-1.78707882	-0.35022805	-1.59630320
C	-3.23044282	-0.60157206	-1.05051877
H	-3.86992987	0.22706559	-1.32015671

H	-3.71062904	-1.49122568	-1.45739978
H	-3.05847345	-1.62450051	0.89525888
H	-3.87247162	-0.10539051	0.99311500
H	-1.80130768	0.64354334	-1.99878621
H	1.04408825	-1.31886415	-1.81835362
C	-1.37977130	0.58774383	1.80789096
H	-0.58946807	1.24718916	1.55025465
H	-2.15653275	1.18928798	2.20861794
H	-1.05930186	-0.11982023	2.54191486
H	1.18156583	1.65328179	1.15872961
C	3.80082168	0.65724038	1.24635837
H	3.38225574	1.07162161	2.15881538
H	4.06921899	-0.36629322	1.52720966
H	4.72659639	1.17156460	1.00523901
H	-1.48440206	-1.01417203	-2.38040125

Time = 289.00 fs
E(CASSCF/6-31G(d)) = -479.4993876

C	-0.84466052	-0.19956794	-0.33836234
C	0.56550745	0.01580570	-0.22243310
C	1.44713248	-0.58965137	-1.16908731
C	1.49283213	0.90894940	0.46381666
C	2.83244763	0.61920091	0.13359288
C	2.71878512	-0.31123630	-1.08141888
N	-1.75122052	-0.04328025	0.62150422
C	-3.06613677	-0.62739429	0.44579470
H	3.50518277	-0.84131239	-1.56208185
C	-1.78403514	-0.34479179	-1.59282353
C	-3.23228708	-0.59434573	-1.05107731
H	-3.87772581	0.23273035	-1.31426412
H	-3.69800018	-1.48714470	-1.46875147
H	-3.06912958	-1.64063574	0.88131557
H	-3.87715062	-0.11260034	0.98861446
H	-1.79447208	0.65636617	-1.99154053
H	1.04916446	-1.33788200	-1.81770986
C	-1.38221599	0.58102553	1.80892485
H	-0.59572286	1.24685807	1.55872578
H	-2.15882490	1.17778331	2.21527046
H	-1.06155615	-0.12872106	2.53717124
H	1.18699601	1.67102927	1.14428792
C	3.80216368	0.66582464	1.24619823
H	3.38442834	1.10097179	2.15175177
H	4.05214252	-0.36058969	1.53400808
H	4.73635057	1.16280884	0.99644910
H	-1.47430262	-1.00226225	-2.38495298

Time = 289.50 fs
E(CASSCF/6-31G(d)) = -479.4993414

C	-0.84654778	-0.19631638	-0.33802771
C	0.57112714	0.01357671	-0.22033990
C	1.44550688	-0.59415104	-1.16592275
C	1.49559989	0.91062349	0.45530047
C	2.83669381	0.62379424	0.13373290
C	2.71782919	-0.32148277	-1.08187867
N	-1.75844511	-0.04982884	0.62571791
C	-3.06655825	-0.62765550	0.44301380
H	3.49942247	-0.84840919	-1.56209517
C	-1.78086721	-0.33937664	-1.58928107
C	-3.23407329	-0.58721522	-1.05179120
H	-3.88532200	0.23860885	-1.30877218
H	-3.68483852	-1.48290536	-1.47965385
H	-3.07981097	-1.65625283	0.86638435
H	-3.87994071	-0.11927474	0.98338035
H	-1.78695285	0.66944701	-1.98510123
H	1.05431398	-1.35734716	-1.81839980
C	-1.38456458	0.57438420	1.81012230
H	-0.60109801	1.24736444	1.56838259
H	-2.16318357	1.16700060	2.22272170
H	-1.06287346	-0.13841966	2.53429740
H	1.19321878	1.68860307	1.13119156
C	3.80382694	0.67439794	1.24607143
H	3.38664385	1.13094270	2.14386509
H	4.03520303	-0.35445681	1.54042655
H	4.74636980	1.15410957	0.98807857
H	-1.46394888	-0.99109265	-2.38953046

Time = 290.00 fs
E(CASSCF/6-31G(d)) = -479.4990978

C	-0.84830713	-0.19306375	-0.33791186
C	0.57639773	0.01154097	-0.21853659
C	1.44331178	-0.59915188	-1.16331001
C	1.49841535	0.91223018	0.44699914
C	2.84030050	0.62828230	0.13367606
C	2.71737711	-0.33142846	-1.08174043
N	-1.76583500	-0.05619365	0.63007614
C	-3.06700259	-0.62814529	0.44039682
H	3.49490228	-0.85572419	-1.56279459
C	-1.77757909	-0.33394051	-1.58572891
C	-3.23577701	-0.58018651	-1.05268429
H	-3.89259123	0.24463797	-1.30377708
H	-3.67132063	-1.47844419	-1.49000627

H	-3.09042214	-1.67071088	0.85037565
H	-3.88093003	-0.12532933	0.97757729
H	-1.77880619	0.68235352	-1.97932921
H	1.05967669	-1.37657983	-1.81980993
C	-1.38684058	0.56782658	1.81151397
H	-0.60557763	1.24861592	1.57905641
H	-2.16952163	1.15686042	2.23094473
H	-1.06319997	-0.14890935	2.53335632
H	1.20025545	1.70550203	1.11904420
C	3.80580919	0.68296549	1.24598065
H	3.38904788	1.16138544	2.13505463
H	4.01845399	-0.34783752	1.54660648
H	4.75655259	1.14542661	0.98019736
H	-1.45353871	-0.98048000	-2.39382976

Time = 290.50 fs
E(CASSCF/6-31G(d)) = -479.4987053

C	-0.84993530	-0.18981499	-0.33796623
C	0.58123766	0.00969582	-0.21702071
C	1.44057468	-0.60471638	-1.16130082
C	1.50127983	0.91383745	0.43897333
C	2.84326379	0.63263457	0.13337334
C	2.71741070	-0.34103312	-1.08097391
N	-1.77330359	-0.06235805	0.63454078
C	-3.06750583	-0.62893816	0.43794107
H	3.49188557	-0.86346750	-1.56433786
C	-1.77417402	-0.32844348	-1.58222156
C	-3.23737313	-0.57326173	-1.05377702
H	-3.89940545	0.25074126	-1.29936707
H	-3.65763724	-1.47372196	-1.49973077
H	-3.10086570	-1.68348419	0.83327061
H	-3.88029452	-0.13065804	0.97141547
H	-1.77009773	0.69466217	-1.97408938
H	1.06534465	-1.39495700	-1.82138225
C	-1.38907049	0.56136134	1.81314424
H	-0.60922319	1.25046994	1.59058488
H	-2.17760164	1.14718503	2.23984620
H	-1.06254692	-0.16006728	2.53428195
H	1.20810927	1.72123241	1.10747136
C	3.80810478	0.69153493	1.24592383
H	3.39178593	1.19213691	2.12523730
H	4.00195128	-0.34068474	1.55269622
H	4.76678052	1.13671927	0.97287116
H	-1.44327125	-0.97023986	-2.39754173

Time = 291.00 fs

E(CASSCF/6-31G(d)) = -479.4982870

C	-0.85143327	-0.18657500	-0.33813967
C	0.58557281	0.00803756	-0.21579084
C	1.43734280	-0.61089131	-1.15993442
C	1.50419318	0.91550733	0.43127622
C	2.84558981	0.63682134	0.13278288
C	2.71790403	-0.35026971	-1.07956143
N	-1.78076108	-0.06830527	0.63907413
C	-3.06809970	-0.63009748	0.43563323
H	3.49051698	-0.87176032	-1.56679686
C	-1.77065535	-0.32284915	-1.57881136
C	-3.23883584	-0.56643962	-1.05508611
H	-3.90564083	0.25683630	-1.29562221
H	-3.64398915	-1.46872469	-1.50877428
H	-3.11104604	-1.69418096	0.81512051
H	-3.87828859	-0.13513916	0.96514164
H	-1.76090189	0.70599305	-1.96926167
H	1.07134740	-1.41195681	-1.82265041
C	-1.39128177	0.55499764	1.81506864
H	-0.61216736	1.25273836	1.60281333
H	-2.18706606	1.13772427	2.24927863
H	-1.06098621	-0.17166634	2.53689152
H	1.21675168	1.73535209	1.09615520
C	3.81070439	0.70011527	1.24589370
H	3.39499664	1.22302868	2.11435653
H	3.98575178	-0.33296196	1.55884903
H	4.77692914	1.12795133	0.96615794
H	-1.43333571	-0.96020638	-2.40037816

Time = 291.50 fs
E(CASSCF/6-31G(d)) = -479.4979718

C	-0.85280520	-0.18334879	-0.33837960
C	0.58933798	0.00656138	-0.21484688
C	1.43368115	-0.61770521	-1.15923525
C	1.50715396	0.91729254	0.42395111
C	2.84729452	0.64081375	0.13187014
C	2.71882660	-0.35912526	-1.07749860
N	-1.78811634	-0.07401870	0.64364003
C	-3.06881165	-0.63167351	0.43345063
H	3.49080077	-0.88061981	-1.57014281
C	-1.76702751	-0.31712583	-1.57554552
C	-3.24013875	-0.55971620	-1.05662433
H	-3.91118368	0.26284231	-1.29261468
H	-3.63058254	-1.46346329	-1.51710988
H	-3.12087364	-1.70255367	0.79604229

H	-3.87523030	-0.13864423	0.95902653
H	-1.75130046	0.71603800	-1.96474998
H	1.07764829	-1.42718697	-1.82326042
C	-1.39350102	0.54874367	1.81734973
H	-0.61460028	1.25519880	1.61559460
H	-2.19747855	1.12818878	2.25905973
H	-1.05863928	-0.18340078	2.54091121
H	1.22611547	1.74750485	1.08485682
C	3.81359477	0.70871578	1.24587747
H	3.39880607	1.25389546	2.10238867
H	3.96991163	-0.32464248	1.56522018
H	4.78687849	1.11909472	0.96010438
H	-1.42390087	-0.95024921	-2.40209540

Time = 292.00 fs
E(CASSCF/6-31G(d)) = -479.4978687

C	-0.85405783	-0.18014134	-0.33863297
C	0.59247824	0.00526156	-0.21419062
C	1.42966943	-0.62516698	-1.15921161
C	1.51015901	0.91923366	0.41703059
C	2.84840324	0.64458389	0.13060767
C	2.72014560	-0.36760036	-1.07479430
N	-1.79527853	-0.07948203	0.64820462
C	-3.06966542	-0.63370157	0.43136314
H	3.49259917	-0.88995666	-1.57424264
C	-1.76329684	-0.31124752	-1.57246400
C	-3.24125508	-0.55308573	-1.05840023
H	-3.91593582	0.26868817	-1.29040920
H	-3.61762474	-1.45797181	-1.52473648
H	-3.13026822	-1.70850247	0.77621234
H	-3.87147950	-0.14104973	0.95334800
H	-1.74138031	0.72458064	-1.96048890
H	1.08414762	-1.44040218	-1.82298123
C	-1.39575278	0.54260543	1.82005265
H	-0.61674924	1.25761008	1.62878631
H	-2.20836705	1.11828408	2.26899240
H	-1.05566272	-0.19491929	2.54600897
H	1.23609366	1.75744270	1.07342898
C	3.81675874	0.71734502	1.24585704
H	3.40332177	1.28458052	2.08934697
H	3.95448621	-0.31570866	1.57196192
H	4.79652239	1.11013489	0.95474286
H	-1.41510593	-0.94028654	-2.40251267

Time = 292.50 fs
E(CASSCF/6-31G(d)) = -479.4980544

C	-0.85519952	-0.17695766	-0.33884741
C	0.59494997	0.00413152	-0.21382571
C	1.42539908	-0.63326515	-1.15985451
C	1.51320321	0.92135766	0.41053509
C	2.84895019	0.64810485	0.12897501
C	2.72182628	-0.37570791	-1.07146946
N	-1.80215899	-0.08467996	0.65273714
C	-3.07068199	-0.63620011	0.42933552
H	3.49565111	-0.89958849	-1.57886915
C	-1.75947208	-0.30519431	-1.56959777
C	-3.24215792	-0.54654142	-1.06041862
H	-3.91981941	0.27431793	-1.28906278
H	-3.60531745	-1.45230317	-1.53167759
H	-3.13916285	-1.71207734	0.75585846
H	-3.86740961	-0.14225240	0.94837203
H	-1.73123125	0.73150820	-1.95644620
H	1.09068618	-1.45151346	-1.82171126
C	-1.39805848	0.53658592	1.82324023
H	-0.61886046	1.25972914	1.64225038
H	-2.21926599	1.10773916	2.27888630
H	-1.05223178	-0.20586065	2.55182814
H	1.24653886	1.76504274	1.06182391
C	3.82017520	0.72600986	1.24580939
H	3.40863029	1.31494629	2.07528257
H	3.93952602	-0.30614883	1.57922260
H	4.80577770	1.10106948	0.95008793
H	-1.40705485	-0.93029138	-2.40152426

Time = 293.00 fs
E(CASSCF/6-31G(d)) = -479.4985463

C	-0.85623988	-0.17380277	-0.33897194
C	0.59672157	0.00316377	-0.21375802
C	1.42096918	-0.64196763	-1.16113740
C	1.51627893	0.92367669	0.40447319
C	2.84897767	0.65135084	0.12695832
C	2.72383209	-0.38347041	-1.06755511
N	-1.80867294	-0.08959839	0.65721016
C	-3.07188075	-0.63916896	0.42732997
H	3.49960725	-0.90926329	-1.58372204
C	-1.75556495	-0.29895288	-1.56696801
C	-3.24282060	-0.54007642	-1.06268058
H	-3.92277998	0.27969610	-1.28862458
H	-3.59385190	-1.44652357	-1.53797806
H	-3.14750637	-1.71347610	0.73525209
H	-3.86337550	-0.14218643	0.94433047

H	-1.72094350	0.73681698	-1.95262443
H	1.09705412	-1.46059280	-1.81947948
C	-1.40043643	0.53068460	1.82696924
H	-0.62117541	1.26132992	1.65585039
H	-2.22975005	1.09632973	2.28857395
H	-1.04852526	-0.21588654	2.55801947
H	1.25726743	1.77031425	1.05009352
C	3.82381924	0.73471469	1.24570738
H	3.41479394	1.34487688	2.06028361
H	3.92507777	-0.29595471	1.58714082
H	4.81458991	1.09191126	0.94613525
H	-1.39981197	-0.92029551	-2.39910670

Time = 293.50 fs
E(CASSCF/6-31G(d)) = -479.4993099

C	-0.85718921	-0.17068159	-0.33895790
C	0.59777369	0.00235006	-0.21399551
C	1.41648323	-0.65122205	-1.16301612
C	1.51937569	0.92618776	0.39884161
C	2.84853520	0.65429729	0.12455002
C	2.72612337	-0.39091724	-1.06308993
N	-1.81474124	-0.09422508	0.66159982
C	-3.07328055	-0.64258754	0.42530881
H	3.50407318	-0.91868922	-1.58845545
C	-1.75159026	-0.29251662	-1.56458557
C	-3.24321706	-0.53368466	-1.06518407
H	-3.92478852	0.28481053	-1.28913531
H	-3.58340262	-1.44070555	-1.54370033
H	-3.15526643	-1.71304104	0.71469908
H	-3.85968125	-0.14084126	0.94140044
H	-1.71060504	0.74061245	-1.94906017
H	1.10299882	-1.46787263	-1.81644338
C	-1.40290172	0.52489708	1.83128707
H	-0.62391304	1.26221984	1.66945151
H	-2.23946085	1.08389317	2.29792441
H	-1.04471207	-0.22470852	2.56426671
H	1.26806397	1.77340112	1.03838707
C	3.82766254	0.74346085	1.24552065
H	3.42185034	1.37428373	2.04447122
H	3.91118226	-0.28511775	1.59584293
H	4.82293738	1.08268604	0.94285998
H	-1.39340033	-0.91038870	-2.39532043

Time = 294.00 fs
E(CASSCF/6-31G(d)) = -479.5002585

C	-0.85805833	-0.16759896	-0.33875964
C	0.59809926	0.00168147	-0.21454804
C	1.41204579	-0.66095675	-1.16542968
C	1.52247985	0.92887307	0.39362590
C	2.84767842	0.65692103	0.12174842
C	2.72865644	-0.39808173	-1.05811759
N	-1.82029201	-0.09855007	0.66588590
C	-3.07490052	-0.64641392	0.42323698
H	3.50865300	-0.92756508	-1.59270760
C	-1.74756602	-0.28588566	-1.56245117
C	-3.24332241	-0.52736172	-1.06792437
H	-3.92584198	0.28967373	-1.29062640
H	-3.57412223	-1.43492007	-1.54891955
H	-3.16243142	-1.71124998	0.69452974
H	-3.85655270	-0.13827665	0.93968703
H	-1.70029921	0.74310400	-1.94582239
H	1.10823544	-1.47373869	-1.81288219
C	-1.40546650	0.51921506	1.83622950
H	-0.62725441	1.26225359	1.68292097
H	-2.24812522	1.07033697	2.30685178
H	-1.04094104	-0.23210761	2.57030548
H	1.27868799	1.77457701	1.02694396
C	3.83167373	0.75224628	1.24521682
H	3.42981263	1.40310741	2.02799346
H	3.89787399	-0.27362587	1.60543915
H	4.83083258	1.07343099	0.94021681
H	-1.38780216	-0.90071478	-2.39030643

Time = 294.50 fs
E(CASSCF/6-31G(d)) = -479.5012746

C	-0.85885843	-0.16455963	-0.33833518
C	0.59770319	0.00114872	-0.21542689
C	1.40775944	-0.67108286	-1.16830190
C	1.52557419	0.93170107	0.38880157
C	2.84646805	0.65920049	0.11855729
C	2.73138246	-0.40499859	-1.05268441
N	-1.82526204	-0.10256612	0.67005162
C	-3.07676003	-0.65058501	0.42108414
H	3.51298757	-0.93560773	-1.59612839
C	-1.74351310	-0.27906668	-1.56055615
C	-3.24311347	-0.52110543	-1.07089475
H	-3.92596218	0.29432227	-1.29311900
H	-3.56613653	-1.42922854	-1.55371843
H	-3.16901134	-1.70869851	0.67508656
H	-3.85412030	-0.13463225	0.93921309
H	-1.69010234	0.74459386	-1.94300874

H	1.11245940	-1.47871158	-1.80918162
C	-1.40814017	0.51362638	1.84181923
H	-0.63133211	1.26134332	1.69612944
H	-2.25556726	1.05564189	2.31532086
H	-1.03733311	-0.23794744	2.57593592
H	1.28888233	1.77423176	1.01608138
C	3.83581905	0.76106540	1.24476264
H	3.43867126	1.43131851	2.01101745
H	3.88518069	-0.26146065	1.61602091
H	4.83832088	1.06419130	0.93814089
H	-1.38296179	-0.89146402	-2.38427726

Time = 295.00 fs
E(CASSCF/6-31G(d)) = -479.5022408

C	-0.85960101	-0.16156813	-0.33764696
C	0.59660163	0.00074239	-0.21664422
C	1.40372168	-0.68149794	-1.17154445
C	1.52863771	0.93462870	0.38433594
C	2.84496851	0.66111600	0.11498544
C	2.73424736	-0.41170192	-1.04683731
N	-1.82959817	-0.10626907	0.67408354
C	-3.07887790	-0.65501875	0.41882627
H	3.51678350	-0.94257337	-1.59840249
C	-1.73945482	-0.27207254	-1.55888374
C	-3.24256939	-0.51491647	-1.07408699
H	-3.92519308	0.29881429	-1.29662272
H	-3.55954065	-1.42367530	-1.55818208
H	-3.17503720	-1.70606927	0.65670967
H	-3.85241521	-0.13012878	0.93991764
H	-1.68008176	0.74545987	-1.94074006
H	1.11536353	-1.48341292	-1.80580612
C	-1.41092947	0.50811531	1.84806498
H	-0.63622359	1.25946511	1.70895331
H	-2.26171516	1.03986145	2.32334971
H	-1.03397579	-0.24218154	2.58103049
H	1.29838420	1.77284706	1.00617442
C	3.84006299	0.76990940	1.24412530
H	3.44839674	1.45891532	1.99371988
H	3.87312360	-0.24859506	1.62765776
H	4.84547609	1.05501677	0.93654997
H	-1.37879103	-0.88286164	-2.37750275

Time = 295.50 fs
E(CASSCF/6-31G(d)) = -479.5030895

C	-0.86029780	-0.15862881	-0.33666252

C	0.59482110	0.00045327	-0.21821223
C	1.40002199	-0.69209148	-1.17506159
C	1.53164529	0.93760479	0.38019076
C	2.84324647	0.66265002	0.11104641
C	2.73719191	-0.41822356	-1.04062215
N	-1.83325843	-0.10965805	0.67797142
C	-3.08127085	-0.65961861	0.41644660
H	3.51983270	-0.94827309	-1.59926735
C	-1.73541605	-0.26492161	-1.55741119
C	-3.24167234	-0.50879861	-1.07749198
H	-3.92359654	0.30322543	-1.30113421
H	-3.55439634	-1.41828130	-1.56239341
H	-3.18055750	-1.70408467	0.63972020
H	-3.85137929	-0.12505978	0.94166337
H	-1.67029445	0.74613119	-1.93915245
H	1.11665967	-1.48851068	-1.80325562
C	-1.41383847	0.50266307	1.85496125
H	-0.64194914	1.25666085	1.72127729
H	-2.26660371	1.02311851	2.33100996
H	-1.03091947	-0.24485625	2.58553733
H	1.30693824	1.77095835	0.99762757
C	3.84436911	0.77876668	1.24327365
H	3.45894301	1.48591943	1.97627742
H	3.86171872	-0.23499338	1.64039499
H	4.85239353	1.04595842	0.93534743
H	-1.37517714	-0.87515196	-2.37028985

Time = 296.00 fs
E(CASSCF/6-31G(d)) = -479.5038633

C	-0.86096119	-0.15574580	-0.33535483
C	0.59239759	0.00027267	-0.22014254
C	1.39673841	-0.70275231	-1.17875613
C	1.53456744	0.94057477	0.37632584
C	2.84136958	0.66378762	0.10675807
C	2.74015273	-0.42459213	-1.03408229
N	-1.83621254	-0.11273556	0.68170791
C	-3.08395143	-0.66428074	0.41393601
H	3.52202387	-0.95258306	-1.59852642
C	-1.73142228	-0.25763647	-1.55611243
C	-3.24040812	-0.50275856	-1.08110003
H	-3.92124706	0.30764243	-1.30663592
H	-3.55072959	-1.41304062	-1.56642945
H	-3.18563232	-1.70343913	0.62440062
H	-3.85088574	-0.11977435	0.94425243
H	-1.66078663	0.74705456	-1.93838703
H	1.11610547	-1.49464567	-1.80200804

C	-1.41686856	0.49724844	1.86248833
H	-0.64847451	1.25303764	1.73299763
H	-2.27037300	1.00559780	2.33842472
H	-1.02817458	-0.24610996	2.58948041
H	1.31431258	1.76910442	0.99083724
C	3.84870101	0.78762361	1.24217912
H	3.47025320	1.51237150	1.95885382
H	3.85097470	-0.22060888	1.65425290
H	4.85918098	1.03706170	0.93442653
H	-1.37199375	-0.86857969	-2.36295684

Time = 296.50 fs
E(CASSCF/6-31G(d)) = -479.5046954

C	-0.86160392	-0.15292295	-0.33370294
C	0.58937546	0.00019285	-0.22244525
C	1.39393504	-0.71337709	-1.18253657
C	1.53736996	0.94348593	0.37270279
C	2.83940479	0.66451672	0.10214248
C	2.74306283	-0.43083224	-1.02725780
N	-1.83844253	-0.11550738	0.68528858
C	-3.08692636	-0.66890241	0.41129292
H	3.52334648	-0.95544913	-1.59605669
C	-1.72749818	-0.25024275	-1.55496141
C	-3.23876686	-0.49680581	-1.08490135
H	-3.91822513	0.31215665	-1.31309444
H	-3.54853086	-1.40791814	-1.57035882
H	-3.19032455	-1.70472354	0.61097808
H	-3.85076636	-0.11465419	0.94744588
H	-1.65159463	0.74865732	-1.93857718
H	1.11353281	-1.50234345	-1.80245072
C	-1.42001829	0.49184882	1.87061301
H	-0.65571659	1.24876083	1.74402539
H	-2.27326135	0.98753762	2.34576357
H	-1.02571152	-0.24616772	2.59295625
H	1.32031581	1.76776640	0.98614743
C	3.85302322	0.79646548	1.24081676
H	3.48226301	1.53832174	1.94159155
H	3.84089647	-0.20538707	1.66922457
H	4.86594703	1.02836549	0.93367507
H	-1.36911325	-0.86336665	-2.35580492

Time = 297.00 fs
E(CASSCF/6-31G(d)) = -479.5058040

C	-0.86223928	-0.15016381	-0.33169237
C	0.58580639	0.00020729	-0.22512825

C	1.39165932	-0.72387854	-1.18632381
C	1.54001384	0.94629309	0.36928926
C	2.83741690	0.66482853	0.09722567
C	2.74585290	-0.43696409	-1.02018479
N	-1.83994260	-0.11798235	0.68871186
C	-3.09019497	-0.67339133	0.40852277
H	3.52389014	-0.95688879	-1.59181340
C	-1.72366617	-0.24276751	-1.55393553
C	-3.23674362	-0.49095189	-1.08888621
H	-3.91461040	0.31685630	-1.32045930
H	-3.54775559	-1.40285108	-1.57424062
H	-3.19468984	-1.70834600	0.59960922
H	-3.85083967	-0.11008735	0.95098547
H	-1.64274659	0.75130548	-1.93983418
H	1.10887459	-1.51193088	-1.80481580
C	-1.42328345	0.48644141	1.87928966
H	-0.66355357	1.24404436	1.75428974
H	-2.27559227	0.96921646	2.35323419
H	-1.02346150	-0.24533242	2.59612694
H	1.32481294	1.76730640	0.98380474
C	3.85730226	0.80527758	1.23916579
H	3.49490571	1.56382247	1.92460224
H	3.83148502	-0.18926734	1.68527602
H	4.87278928	1.01989787	0.93298078
H	-1.36641999	-0.85968817	-2.34908836

Time = 297.50 fs
E(CASSCF/6-31G(d)) = -479.5073803

C	-0.86288099	-0.14747153	-0.32931533
C	0.58174832	0.00031093	-0.22819659
C	1.38994045	-0.73419160	-1.19005632
C	1.54245556	0.94896313	0.36606223
C	2.83546695	0.66471794	0.09203751
C	2.74845245	-0.44300334	-1.01289501
N	-1.84071897	-0.12017195	0.69197906
C	-3.09374820	-0.67767351	0.40563713
H	3.52383914	-0.95698945	-1.58583057
C	-1.71994504	-0.23523770	-1.55301890
C	-3.23433888	-0.48520950	-1.09304504
H	-3.91047494	0.32181889	-1.32866172
H	-3.54832625	-1.39775276	-1.57812465
H	-3.19876751	-1.71446990	0.59037128
H	-3.85093595	-0.10644267	0.95461373
H	-1.63426527	0.75526478	-1.94223294
H	1.10218376	-1.52347666	-1.80913424
C	-1.42665743	0.48100465	1.88846199

H	-0.67183799	1.23913667	1.76374138
H	-2.27775319	0.95093644	2.36106960
H	-1.02132029	-0.24397056	2.59920902
H	1.32773806	1.76791680	0.98392002
C	3.86150766	0.81404613	1.23721013
H	3.50811572	1.58891933	1.90795997
H	3.82273880	-0.17218643	1.70234618
H	4.87978243	1.01167482	0.93223641
H	-1.36382229	-0.85765089	-2.34298849

Time = 298.00 fs
E(CASSCF/6-31G(d)) = -479.5094979

C	-0.86354317	-0.14484892	-0.32657106
C	0.57726473	0.00050030	-0.23165223
C	1.38878881	-0.74427577	-1.19369210
C	1.54464778	0.95147760	0.36301017
C	2.83361080	0.66418389	0.08661151
C	2.75079121	-0.44896128	-1.00541571
N	-1.84078913	-0.12208987	0.69509447
C	-3.09756815	-0.68169842	0.40265221
H	3.52346230	-0.95590355	-1.57821774
C	-1.71634880	-0.22767878	-1.55220478
C	-3.23155886	-0.47959147	-1.09736850
H	-3.90587749	0.32710443	-1.33761420
H	-3.55013520	-1.39251927	-1.58205287
H	-3.20257486	-1.72298364	0.58325986
H	-3.85091767	-0.10404702	0.95809182
H	-1.62617086	0.76066939	-1.94579982
H	1.09363897	-1.53677410	-1.81522292
C	-1.43013171	0.47551972	1.89806577
H	-0.68041253	1.23430391	1.77235573
H	-2.28016512	0.93300229	2.36951119
H	-1.01915457	-0.24249236	2.60245718
H	1.32909973	1.76959321	0.98644656
C	3.86561285	0.82275923	1.23493836
H	3.52183190	1.61364535	1.89169644
H	3.81465324	-0.15408180	1.72034865
H	4.88696874	1.00369821	0.93134443
H	-1.36126107	-0.85727591	-2.33759480

Time = 298.50 fs
E(CASSCF/6-31G(d)) = -479.5120535

C	-0.86424011	-0.14229848	-0.32346590
C	0.57242384	0.00077364	-0.23549381
C	1.38819710	-0.75411329	-1.19720749

C	1.54654099	0.95383264	0.36013313
C	2.83189747	0.66322964	0.08098475
C	2.75280078	-0.45484538	-0.99776997
N	-1.84018105	-0.12375153	0.69806552
C	-3.10162814	-0.68544042	0.39958732
H	3.52309763	-0.95383980	-1.56915147
C	-1.71288605	-0.22011385	-1.55149676
C	-3.22841567	-0.47410961	-1.10184748
H	-3.90085927	0.33275027	-1.34721034
H	-3.55304872	-1.38703715	-1.58606149
H	-3.20610621	-1.73351153	0.57819432
H	-3.85069328	-0.10316726	0.96121337
H	-1.61848409	0.76750535	-1.95050552
H	1.08353463	-1.55137191	-1.82270879
C	-1.43369685	0.46997204	1.90803246
H	-0.68912753	1.22981001	1.78013635
H	-2.28324348	0.91569718	2.37878701
H	-1.01681289	-0.24132490	2.60614192
H	1.32897911	1.77213741	0.99117932
C	3.86959592	0.83140750	1.23234369
H	3.53599919	1.63801625	1.87579899
H	3.80722090	-0.13489529	1.73917385
H	4.89435077	0.99595594	0.93022055
H	-1.35871418	-0.85848998	-2.33289762

Time = 299.00 fs
E(CASSCF/6-31G(d)) = -479.5148092

C	-0.86498600	-0.13982224	-0.32001330
C	0.56729759	0.00113084	-0.23971640
C	1.38814265	-0.76370402	-1.20059335
C	1.54808553	0.95603632	0.35744096
C	2.83036788	0.66186309	0.07519786
C	2.75441656	-0.46066014	-0.98997767
N	-1.83893227	-0.12517351	0.70090289
C	-3.10589255	-0.68889641	0.39646283
H	3.52312998	-0.95104948	-1.55886086
C	-1.70955987	-0.21256346	-1.55090839
C	-3.22492709	-0.46877376	-1.10647288
H	-3.89544166	0.33876845	-1.35732556
H	-3.55691171	-1.38119239	-1.59018373
H	-3.20933600	-1.74546332	0.57502905
H	-3.85022568	-0.10399706	0.96381414
H	-1.61122775	0.77561218	-1.95626267
H	1.07225789	-1.56664280	-1.83108196
C	-1.43734358	0.46435225	1.91829314
H	-0.69785749	1.22589573	1.78711746

H	-2.28735378	0.89925875	2.38908753
H	-1.01414083	-0.24088074	2.61052338
H	1.32752074	1.77519010	0.99777548
C	3.87344003	0.83998438	1.22942366
H	3.55056815	1.66202650	1.86021322
H	3.80043294	-0.11457783	1.75869025
H	4.90188774	0.98842502	0.92879599
H	-1.35619476	-0.86112616	-2.32879372

Time = 299.50 fs
E(CASSCF/6-31G(d)) = -479.5174791

C	-0.86579454	-0.13742179	-0.31623375
C	0.56196095	0.00157336	-0.24431155
C	1.38859050	-0.77305841	-1.20384978
C	1.54923398	0.95810430	0.35495004
C	2.82905364	0.66009698	0.06929490
C	2.75558019	-0.46640849	-0.98205695
N	-1.83708881	-0.12637314	0.70362073
C	-3.11031724	-0.69208115	0.39329836
H	3.52396180	-0.94780819	-1.54760702
C	-1.70636841	-0.20504601	-1.55046136
C	-3.22111624	-0.46359098	-1.11123543
H	-3.88962661	0.34514542	-1.36781869
H	-3.56155291	-1.37487955	-1.59445252
H	-3.21222528	-1.75810834	0.57356762
H	-3.84953630	-0.10664818	0.96577931
H	-1.60442719	0.78470136	-1.96293095
H	1.06025670	-1.58187071	-1.83976337
C	-1.44106381	0.45865683	1.92878297
H	-0.70651610	1.22275895	1.79336613
H	-2.29276729	0.88385661	2.40054204
H	-1.01099935	-0.24152284	2.61582150
H	1.32491680	1.77828453	1.00579239
C	3.87713359	0.84848617	1.22617961
H	3.56549392	1.68565021	1.84484690
H	3.79427858	-0.09309294	1.77874752
H	4.90949690	0.98107372	0.92701836
H	-1.35374479	-0.86493518	-2.32510299

Time = 300.00 fs
E(CASSCF/6-31G(d)) = -479.5198328

C	-0.86667907	-0.13509680	-0.31215219
C	0.55648791	0.00210448	-0.24927037
C	1.38949708	-0.78219622	-1.20698266
C	1.54994424	0.96005581	0.35267807

C	2.82797532	0.65794778	0.06331966
C	2.75624313	-0.47209704	-0.97402130
N	-1.83470273	-0.12736869	0.70623826
C	-3.11485281	-0.69502330	0.39010907
H	3.52597757	-0.94439150	-1.53564906
C	-1.70330397	-0.19757398	-1.55018231
C	-3.21700843	-0.45856189	-1.11612840
H	-3.88339562	0.35184854	-1.37854103
H	-3.56679343	-1.36800601	-1.59890576
H	-3.21473062	-1.77066576	0.57357740
H	-3.84870525	-0.11114829	0.96704857
H	-1.59810505	0.79439441	-1.97033175
H	1.04799863	-1.59634514	-1.84817597
C	-1.44485346	0.45288435	1.93945174
H	-0.71507209	1.22053708	1.79898698
H	-2.29962843	0.86956732	2.41320694
H	-1.00728227	-0.24353142	2.62219070
H	1.32138686	1.78091031	1.01473840
C	3.88067225	0.85691683	1.22261403
H	3.58074363	1.70885886	1.82956674
H	3.78874115	-0.07040636	1.79919246
H	4.91706366	0.97385978	0.92484959
H	-1.35142349	-0.86960747	-2.32159129

Time = 300.50 fs
E(CASSCF/6-31G(d)) = -479.5217595

C	-0.86765047	-0.13284934	-0.30780474
C	0.55096025	0.00272825	-0.25457501
C	1.39081205	-0.79112416	-1.20999365
C	1.55018027	0.96190534	0.35064446
C	2.82714398	0.65543931	0.05732618
C	2.75636822	-0.47772763	-0.96589622
N	-1.83183479	-0.12817698	0.70877495
C	-3.11943737	-0.69775427	0.38690998
H	3.52950026	-0.94105694	-1.52323573
C	-1.70035992	-0.19016857	-1.55009999
C	-3.21263752	-0.45368960	-1.12113861
H	-3.87672542	0.35881894	-1.38932053
H	-3.57244324	-1.36051096	-1.60358043
H	-3.21680542	-1.78235735	0.57480504
H	-3.84787016	-0.11743390	0.96761485
H	-1.59229097	0.80424608	-1.97824939
H	1.03596153	-1.60940311	-1.85578301
C	-1.44870568	0.44704502	1.95024775
H	-0.72353849	1.21930069	1.80411121
H	-2.30792285	0.85639475	2.42703339

H	-1.00294066	-0.24707439	2.62968657
H	1.31716443	1.78256628	1.02410319
C	3.88405187	0.86527222	1.21873605
H	3.59626623	1.73158408	1.81423470
H	3.78380871	-0.04652899	1.81983542
H	4.92443709	0.96674746	0.92226784
H	-1.34929978	-0.87478974	-2.31800573

Time = 301.00 fs
E(CASSCF/6-31G(d)) = -479.5232575

C	-0.86871827	-0.13067945	-0.30323120
C	0.54545682	0.00345025	-0.26020756
C	1.39248223	-0.79984867	-1.21288288
C	1.54991545	0.96366233	0.34886285
C	2.82655871	0.65259909	0.05136765
C	2.75593461	-0.48330951	-0.95770697
N	-1.82854945	-0.12881505	0.71125525
C	-3.12400595	-0.70030897	0.38370921
H	3.53475314	-0.93801506	-1.51055450
C	-1.69752644	-0.18284709	-1.55024096
C	-3.20803760	-0.44897055	-1.12625488
H	-3.86958304	0.36599019	-1.39998535
H	-3.57831573	-1.35235860	-1.60852207
H	-3.21840932	-1.79249189	0.57698740
H	-3.84721865	-0.12536068	0.96752561
H	-1.58700649	0.81380327	-1.98646001
H	1.02458593	-1.62050480	-1.86214143
C	-1.45261694	0.44114880	1.96113839
H	-0.73198636	1.21904540	1.80890505
H	-2.31748802	0.84424709	2.44188975
H	-0.99798906	-0.25219356	2.63826356
H	1.31247494	1.78281371	1.03341295
C	3.88727433	0.87355469	1.21455461
H	3.61201782	1.75376779	1.79868811
H	3.77946265	-0.02147829	1.84049181
H	4.93145568	0.95969639	0.91926107
H	-1.34743600	-0.88011952	-2.31409668

Time = 301.50 fs
E(CASSCF/6-31G(d)) = -479.5244012

C	-0.86988937	-0.12858681	-0.29847681
C	0.54005569	0.00427712	-0.26614723
C	1.39445337	-0.80836802	-1.21564524
C	1.54913339	0.96532740	0.34734001
C	2.82620761	0.64945920	0.04549969

C	2.75493972	-0.48885705	-0.94948505
N	-1.82491504	-0.12929922	0.71370675
C	-3.12848926	-0.70272022	0.38050951
H	3.54182365	-0.93541428	-1.49771522
C	-1.69479361	-0.17562836	-1.55062668
C	-3.20324445	-0.44439786	-1.13146488
H	-3.86193650	0.37329150	-1.41036217
H	-3.58422987	-1.34354560	-1.61378277
H	-3.21950910	-1.80050033	0.57986221
H	-3.84698011	-0.13470879	0.96687705
H	-1.58226638	0.82263830	-1.99474281
H	1.01426337	-1.62925353	-1.86691967
C	-1.45658365	0.43520732	1.97210328
H	-0.74053069	1.21969669	1.81356239
H	-2.32802970	0.83296030	2.45756594
H	-0.99251245	-0.25880317	2.64777298
H	1.30752438	1.78130905	1.04225607
C	3.89034432	0.88176726	1.21007981
H	3.62794954	1.77535418	1.78275841
H	3.77568159	0.00470638	1.86097216
H	4.93795521	0.95266975	0.91582473
H	-1.34587948	-0.88524774	-2.30964536

Time = 302.00 fs
E(CASSCF/6-31G(d)) = -479.5252890

C	-0.87116782	-0.12657039	-0.29359153
C	0.53483318	0.00521646	-0.27237085
C	1.39667190	-0.81667114	-1.21826935
C	1.54782847	0.96689069	0.34607424
C	2.82606825	0.64605600	0.03977974
C	2.75340055	-0.49439033	-0.94126880
N	-1.82100260	-0.12964491	0.71615981
C	-3.13281591	-0.70501576	0.37730833
H	3.55064476	-0.93333397	-1.48473908
C	-1.69215247	-0.16853311	-1.55127088
C	-3.19829428	-0.43996187	-1.13675489
H	-3.85376132	0.38065561	-1.42028094
H	-3.59001455	-1.33410143	-1.61942088
H	-3.22007913	-1.80596352	0.58317607
H	-3.84741052	-0.14519655	0.96580418
H	-1.57807550	0.83037986	-2.00289397
H	1.00532137	-1.63541017	-1.86990959
C	-1.46060087	0.42923233	1.98313231
H	-0.74931760	1.22112038	1.81829835
H	-2.33915726	0.82231749	2.47379314
H	-0.98666139	-0.26670281	2.65797618

H	1.30248872	1.77782403	1.05030571
C	3.89326844	0.88991300	1.20532233
H	3.64400698	1.79629866	1.76627765
H	3.77244000	0.03196473	1.88108800
H	4.94378218	0.94563432	0.91195898
H	-1.34465426	-0.88986115	-2.30448236

Time = 302.50 fs
E(CASSCF/6-31G(d)) = -479.5259981

C	-0.87255359	-0.12462852	-0.28862934
C	0.52986223	0.00627645	-0.27885306
C	1.39908619	-0.82473715	-1.22073732
C	1.54600572	0.96833164	0.34505444
C	2.82610875	0.64242949	0.03426636
C	2.75135335	-0.49993451	-0.93310267
N	-1.81688487	-0.12986653	0.71864710
C	-3.13691478	-0.70721573	0.37409874
H	3.56099303	-0.93178846	-1.47156129
C	-1.68959575	-0.16158434	-1.55217784
C	-3.19322281	-0.43565084	-1.14210978
H	-3.84504676	0.38802615	-1.42957955
H	-3.59551263	-1.32408729	-1.62550029
H	-3.22010093	-1.80862858	0.58669132
H	-3.84877430	-0.15649830	0.96446617
H	-1.57442663	0.83673796	-2.01073814
H	0.99801322	-1.63889850	-1.87103109
C	-1.46466139	0.42323467	1.99422242
H	-0.75850688	1.22313867	1.82334161
H	-2.35042868	0.81207255	2.49026945
H	-0.98063821	-0.27560408	2.66856996
H	1.29750621	1.77225646	1.05733410
C	3.89605419	0.89799449	1.20029287
H	3.66012947	1.81657514	1.74908620
H	3.76970824	0.06021440	1.90065669
H	4.94880644	0.93856125	0.90766507
H	-1.34375570	-0.89370040	-2.29850160

Time = 303.00 fs
E(CASSCF/6-31G(d)) = -479.5265622

C	-0.87404274	-0.12275890	-0.28364720
C	0.52521176	0.00746580	-0.28556650
C	1.40164789	-0.83253544	-1.22302455
C	1.54368028	0.96961895	0.34425967
C	2.82628940	0.63862252	0.02901843
C	2.74885169	-0.50551815	-0.92503530

N	-1.81263520	-0.12997747	0.72120296
C	-3.14071770	-0.70933034	0.37087065
H	3.57250724	-0.93074076	-1.45804872
C	-1.68711848	-0.15480712	-1.55334127
C	-3.18806439	-0.43145169	-1.14751289
H	-3.83580046	0.39536280	-1.43810804
H	-3.60058367	-1.31359236	-1.63208831
H	-3.21956259	-1.80841654	0.59019172
H	-3.85132058	-0.16826713	0.96302686
H	-1.57129938	0.84151997	-2.01813742
H	0.99250943	-1.63980581	-1.87033374
C	-1.46875535	0.41722354	2.00537391
H	-0.76825470	1.22554794	1.82892599
H	-2.36139495	0.80197158	2.50668459
H	-0.97467878	-0.28516258	2.67921661
H	1.29267321	1.76463367	1.06322138
C	3.89870886	0.90601328	1.19500165
H	3.67625038	1.83617881	1.73103730
H	3.76745274	0.08935376	1.91950356
H	4.95292905	0.93142660	0.90294334
H	-1.34314761	-0.89657374	-2.29166853

Time = 303.50 fs
E(CASSCF/6-31G(d)) = -479.5269805

C	-0.87562680	-0.12095850	-0.27870418
C	0.52094517	0.00879340	-0.29248216
C	1.40431331	-0.84002580	-1.22509981
C	1.54087674	0.97071139	0.34365917
C	2.82656385	0.63467983	0.02409428
C	2.74596264	-0.51117117	-0.91711687
N	-1.80832646	-0.12999009	0.72386254
C	-3.14416244	-0.71135755	0.36761267
H	3.58472240	-0.93012382	-1.44402565
C	-1.68471821	-0.14822849	-1.55474394
C	-3.18285131	-0.42735088	-1.15294623
H	-3.82605183	0.40264593	-1.44573213
H	-3.60510578	-1.30272848	-1.63925311
H	-3.21845694	-1.80542548	0.59348799
H	-3.85525642	-0.18016057	0.96163138
H	-1.56865894	0.84464091	-2.02499757
H	0.98889090	-1.63837981	-1.86799535
C	-1.47287024	0.41120627	2.01658712
H	-0.77869667	1.22813821	1.83528058
H	-2.37164124	0.79177205	2.52274342
H	-0.96903058	-0.29501283	2.68957493
H	1.28804067	1.75510894	1.06796009

C	3.90123887	0.91396980	1.18945808
H	3.69229846	1.85513083	1.71200158
H	3.76563562	0.11926249	1.93746746
H	4.95609099	0.92421025	0.89779099
H	-1.34276120	-0.89836586	-2.28402227

Time = 304.00 fs
E(CASSCF/6-31G(d)) = -479.5272114

C	-0.87729272	-0.11922351	-0.27386032
C	0.51711912	0.01026813	-0.29956967
C	1.40704429	-0.84715907	-1.22692561
C	1.53762823	0.97155884	0.34321313
C	2.82688091	0.63064730	0.01955064
C	2.74276279	-0.51692231	-0.90939600
N	-1.80403018	-0.12991570	0.72666089
C	-3.14719535	-0.71328110	0.36431402
H	3.59711370	-0.92986332	-1.42930437
C	-1.68239528	-0.14187713	-1.55635785
C	-3.17761330	-0.42333525	-1.15839057
H	-3.81585330	0.40987830	-1.45233572
H	-3.60897600	-1.29162244	-1.64705984
H	-3.21678083	-1.79992817	0.59642243
H	-3.86071895	-0.19186662	0.96038148
H	-1.56645609	0.84612704	-2.03127216
H	0.98714386	-1.63502237	-1.86431787
C	-1.47699141	0.40518806	2.02785960
H	-0.78993466	1.23071064	1.84262074
H	-2.38081858	0.78125675	2.53818393
H	-0.96393249	-0.30479894	2.69932726
H	1.28361380	1.74395265	1.07165425
C	3.90364908	0.92186320	1.18367048
H	3.70819943	1.87347820	1.69186821
H	3.76421452	0.14980532	1.95440377
H	4.95827640	0.91689489	0.89220116
H	-1.34249621	-0.89904269	-2.27567381

Time = 304.50 fs
E(CASSCF/6-31G(d)) = -479.5271909

C	-0.87902295	-0.11754930	-0.26917531
C	0.51378284	0.01189847	-0.30679757
C	1.40980865	-0.85387814	-1.22845876
C	1.53397474	0.97210421	0.34287331
C	2.82718638	0.62657053	0.01544173
C	2.73933390	-0.52279677	-0.90191667
N	-1.79981530	-0.12976468	0.72963175

C	-3.14977393	-0.71506929	0.36096617
H	3.60914198	-0.92989792	-1.41371420
C	-1.68015251	-0.13578283	-1.55814498
C	-3.17237730	-0.41939273	-1.16382587
H	-3.80528034	0.41708528	-1.45782316
H	-3.61211041	-1.28040845	-1.65556729
H	-3.21453461	-1.79236653	0.59887370
H	-3.86775395	-0.20312512	0.95931553
H	-1.56462791	0.84611428	-2.03696443
H	0.98715911	-1.63027634	-1.85971915
C	-1.48110270	0.39917220	2.03918431
H	-0.80202579	1.23309384	1.85114021
H	-2.38866819	0.77024382	2.55279228
H	-0.95959604	-0.31420152	2.70820247
H	1.27935350	1.73153740	1.07451269
C	3.90594227	0.92969154	1.17764590
H	3.72387882	1.89129151	1.67054630
H	3.76314323	0.18083519	1.97018693
H	4.95951328	0.90946470	0.88616252
H	-1.34222356	-0.89865211	-2.26680102

Time = 305.00 fs
E(CASSCF/6-31G(d)) = -479.5268443

C	-0.88079556	-0.11593044	-0.26470747
C	0.51097696	0.01369220	-0.31413352
C	1.41258024	-0.86011991	-1.22965187
C	1.52996173	0.97228584	0.34258500
C	2.82742477	0.62249384	0.01181830
C	2.73575875	-0.52881424	-0.89471574
N	-1.79574733	-0.12954643	0.73280660
C	-3.15186817	-0.71667495	0.35756420
H	3.62029639	-0.93019573	-1.39712517
C	-1.67799501	-0.12997600	-1.56005851
C	-3.16716738	-0.41551301	-1.16923167
H	-3.79442927	0.42431293	-1.46211995
H	-3.61444296	-1.26921909	-1.66482316
H	-3.21172125	-1.78333597	0.60075982
H	-3.87630048	-0.21374272	0.95839474
H	-1.56309900	0.84484108	-2.04212583
H	0.98873815	-1.62480264	-1.85471496
C	-1.48518724	0.39316007	2.05054812
H	-0.81497685	1.23515527	1.86100386
H	-2.39503693	0.75859243	2.56641218
H	-0.95619108	-0.32295764	2.71599098
H	1.27518122	1.71831674	1.07683461
C	3.90811897	0.93745202	1.17138997

H	3.73926464	1.90866096	1.64796437
H	3.76237210	0.21219698	1.98471324
H	4.95987150	0.90190397	0.87965950
H	-1.34178983	-0.89731943	-2.25763900

Time = 305.50 fs
E(CASSCF/6-31G(d)) = -479.5261394

C	-0.88258563	-0.11436108	-0.26051215
C	0.50873366	0.01565628	-0.32154473
C	1.41533777	-0.86581835	-1.23045576
C	1.52563807	0.97204047	0.34228951
C	2.82754096	0.61845917	0.00872670
C	2.73211777	-0.53498747	-0.88782113
N	-1.79188681	-0.12926932	0.73621303
C	-3.15346125	-0.71803729	0.35410713
H	3.63012944	-0.93076496	-1.37946418
C	-1.67592964	-0.12448718	-1.56204467
C	-3.16200486	-0.41168790	-1.17458761
H	-3.78341367	0.43162389	-1.46517283
H	-3.61592380	-1.25817655	-1.67485948
H	-3.20834478	-1.77355744	0.60203915
H	-3.88618830	-0.22359824	0.95750062
H	-1.56178356	0.84263396	-2.04685140
H	0.99160955	-1.61934179	-1.84988882
C	-1.48922800	0.38715143	2.06193149
H	-0.82874239	1.23680938	1.87234240
H	-2.39988649	0.74620527	2.57895096
H	-0.95383502	-0.33087515	2.72255394
H	1.27098813	1.70479418	1.07898555
C	3.91017738	0.94514139	1.16490691
H	3.75429056	1.92568997	1.62406839
H	3.76184859	0.24373235	1.99790287
H	4.95945721	0.89419563	0.87267373
H	-1.34102404	-0.89523746	-2.24846642

Time = 306.00 fs
E(CASSCF/6-31G(d)) = -479.5250789

C	-0.88436505	-0.11283446	-0.25664083
C	0.50707553	0.01779614	-0.32899825
C	1.41806289	-0.87090952	-1.23082315
C	1.52105438	0.97130738	0.34192773
C	2.82748136	0.61450515	0.00620823
C	2.72848636	-0.54132135	-0.88125076
N	-1.78828893	-0.12894069	0.73987410
C	-3.15454855	-0.71908594	0.35059805

H	3.63828349	-0.93165945	-1.36072408
C	-1.67396405	-0.11934620	-1.56404529
C	-3.15690864	-0.40791154	-1.17987390
H	-3.77235932	0.43909153	-1.46694957
H	-3.61651725	-1.24738517	-1.68568891
H	-3.20441130	-1.76383502	0.60270654
H	-3.89714845	-0.23263830	0.95644446
H	-1.56058847	0.83988674	-2.05127177
H	0.99545774	-1.61465496	-1.84584665
C	-1.49320833	0.38114458	2.07330813
H	-0.84322533	1.23802270	1.88524876
H	-2.40329669	0.73302935	2.59038303
H	-0.95258513	-0.33784183	2.72782981
H	1.26664732	1.69148162	1.08136019
C	3.91211372	0.95275658	1.15819950
H	3.76889845	1.94248613	1.59882070
H	3.76151852	0.27528378	2.00970000
H	4.95840438	0.88632108	0.86518569
H	-1.33974590	-0.89265075	-2.23958846

Time = 306.50 fs
E(CASSCF/6-31G(d)) = -479.5237947

C	-0.88610391	-0.11134339	-0.25313983
C	0.50601560	0.02011563	-0.33646124
C	1.42073709	-0.87533795	-1.23071396
C	1.51626072	0.97003297	0.34144452
C	2.82719538	0.61066595	0.00429843
C	2.72493283	-0.54781216	-0.87501190
N	-1.78500228	-0.12856673	0.74380703
C	-3.15513618	-0.71974725	0.34704349
H	3.64450915	-0.93298004	-1.34096685
C	-1.67210573	-0.11458137	-1.56600093
C	-3.15189557	-0.40418028	-1.18507183
H	-3.76139777	0.44679229	-1.46743775
H	-3.61619977	-1.23692499	-1.69730084
H	-3.19992780	-1.75499241	0.60278423
H	-3.90883337	-0.24086595	0.95498578
H	-1.55941742	0.83703023	-2.05553982
H	0.99996549	-1.61144797	-1.84315365
C	-1.49711248	0.37513678	2.08464515
H	-0.85828213	1.23881416	1.89977660
H	-2.40546382	0.71905363	2.60074955
H	-0.95243434	-0.34383066	2.73183519
H	1.26203116	1.67885309	1.08433663
C	3.91392259	0.96029522	1.15126916
H	3.78304077	1.95915106	1.57219761

H	3.76132706	0.30669924	2.02007274
H	4.95686413	0.87826028	0.85717719
H	-1.33777707	-0.88983352	-2.23131559

Time = 307.00 fs
E(CASSCF/6-31G(d)) = -479.5225662

C	-0.88777267	-0.10988035	-0.25004810
C	0.50555802	0.02261674	-0.34390153
C	1.42333710	-0.87906354	-1.23010094
C	1.51130426	0.96817508	0.34079359
C	2.82663654	0.60697080	0.00302650
C	2.72151732	-0.55444757	-0.86910119
N	-1.78206713	-0.12815231	0.74802124
C	-3.15523910	-0.71995229	0.34345279
H	3.64867784	-0.93487309	-1.32032179
C	-1.67036057	-0.11021869	-1.56785450
C	-3.14698093	-0.40049230	-1.19016432
H	-3.75065947	0.45479733	-1.46664313
H	-3.61495864	-1.22684729	-1.70965858
H	-3.19489967	-1.74779240	0.60230758
H	-3.92084548	-0.24832482	0.95285697
H	-1.55817567	0.83449559	-2.05981357
H	1.00486371	-1.61028511	-1.84226428
C	-1.50092571	0.36912507	2.09590425
H	-0.87373191	1.23925083	1.91594005
H	-2.40669234	0.70430477	2.61015494
H	-0.95330971	-0.34890145	2.73466102
H	1.25702925	1.66729455	1.08822370
C	3.91559768	0.96775622	1.14411599
H	3.79668262	1.97577055	1.54418696
H	3.76122007	0.33783708	2.02901330
H	4.95499224	0.86999178	0.84863442
H	-1.33495351	-0.88706260	-2.22394021

Time = 307.50 fs
E(CASSCF/6-31G(d)) = -479.5216846

C	-0.88934033	-0.10843702	-0.24739653
C	0.50569356	0.02529889	-0.35128747
C	1.42583297	-0.88206894	-1.22897610
C	1.50622895	0.96570790	0.33994212
C	2.82576344	0.60344271	0.00241458
C	2.71829082	-0.56120625	-0.86350439
N	-1.77951562	-0.12770117	0.75251808
C	-3.15487774	-0.71964583	0.33983811
H	3.65078600	-0.93752511	-1.29898175

C	-1.66873142	-0.10628010	-1.56955523
C	-3.14217884	-0.39684697	-1.19513603
H	-3.74026614	0.46316377	-1.46458821
H	-3.61279095	-1.21717290	-1.72269899
H	-3.18933367	-1.74285047	0.60130425
H	-3.93276824	-0.25508188	0.94978933
H	-1.55677629	0.83267294	-2.06423732
H	1.00997471	-1.61151036	-1.84345045
C	-1.50463503	0.36310678	2.10704188
H	-0.88936651	1.23944228	1.93371656
H	-2.40738032	0.68884077	2.61876032
H	-0.95507306	-0.35319828	2.73647000
H	1.25156192	1.65706096	1.09320895
C	3.91713245	0.97514024	1.13673898
H	3.80980291	1.99240408	1.51478615
H	3.76114544	0.36857066	2.03653574
H	4.95293484	0.86149398	0.83955072
H	-1.33113826	-0.88458813	-2.21771303

Time = 308.00 fs
E(CASSCF/6-31G(d)) = -479.5214049

C	-0.89077300	-0.10700348	-0.24520794
C	0.50639700	0.02815812	-0.35858818
C	1.42818684	-0.88436428	-1.22735453
C	1.50107521	0.96262499	0.33887441
C	2.82454020	0.60009784	0.00247740
C	2.71529462	-0.56805822	-0.85819715
N	-1.77737235	-0.12721607	0.75729116
C	-3.15407477	-0.71879437	0.33621458
H	3.65095461	-0.94115683	-1.27719501
C	-1.66721669	-0.10278204	-1.57106247
C	-3.13750272	-0.39324404	-1.19997355
H	-3.73032359	0.47192609	-1.46131096
H	-3.60970327	-1.20789300	-1.73633347
H	-3.18324210	-1.74055723	0.59977292
H	-3.94419574	-0.26121209	0.94553712
H	-1.55514537	0.83187013	-2.06892086
H	1.01524086	-1.61519476	-1.84675452
C	-1.50822997	0.35708036	2.11800984
H	-0.90496191	1.23953112	1.95305164
H	-2.40799612	0.67274234	2.62677272
H	-0.95752504	-0.35694281	2.73749107
H	1.24559074	1.64824836	1.09932023
C	3.91852095	0.98245016	1.12913613
H	3.82239448	2.00907540	1.48400159
H	3.76105437	0.39879180	2.04267325

H	4.95081514	0.85274718	0.82992908
H	-1.32623374	-0.88260452	-2.21282066

Time = 308.50 fs
E(CASSCF/6-31G(d)) = -479.5218477

C	-0.89203552	-0.10556879	-0.24349735
C	0.50762982	0.03118722	-0.36577418
C	1.43035287	-0.88598737	-1.22527471
C	1.49587874	0.95893955	0.33759335
C	2.82293691	0.59694544	0.00322242
C	2.71255969	-0.57496600	-0.85314649
N	-1.77565439	-0.12669843	0.76232687
C	-3.15285252	-0.71739181	0.33260007
H	3.64942232	-0.94601555	-1.25525271
C	-1.66580928	-0.09973430	-1.57234895
C	-3.13296551	-0.38968269	-1.20466549
H	-3.72091622	0.48108940	-1.45686342
H	-3.60571286	-1.19897263	-1.75045007
H	-3.17664558	-1.74102568	0.59766854
H	-3.95475710	-0.26678577	0.93989816
H	-1.55322634	0.83227809	-2.07391986
H	1.02073056	-1.62112836	-1.85197931
C	-1.51170367	0.35104658	2.12875694
H	-0.92029230	1.23967800	1.97386431
H	-2.40904550	0.65610015	2.63442755
H	-0.96041409	-0.36042218	2.73800684
H	1.23912482	1.64079060	1.10641313
C	3.91975869	0.98969134	1.12130433
H	3.83446397	2.02576646	1.45184963
H	3.76090318	0.42841367	2.04747546
H	4.94872262	0.84373588	0.81978494
H	-1.32019198	-0.88122643	-2.20936822

Time = 309.00 fs
E(CASSCF/6-31G(d)) = -479.5229483

C	-0.89309498	-0.10412215	-0.24227219
C	0.50934574	0.03437613	-0.37281799
C	1.43227890	-0.88699916	-1.22279501
C	1.49066961	0.95468225	0.33611956
C	2.82092978	0.59398852	0.00465047
C	2.71010641	-0.58188649	-0.84831319
N	-1.77437083	-0.12614799	0.76760470
C	-3.15123144	-0.71546107	0.32901414
H	3.64653054	-0.95236569	-1.23347097
C	-1.66449581	-0.09713971	-1.57340284

C	-3.12857980	-0.38616074	-1.20920237
H	-3.71210275	0.49062396	-1.45131053
H	-3.60084926	-1.19035528	-1.76491607
H	-3.16957435	-1.74407772	0.59489878
H	-3.96413623	-0.27185936	0.93272862
H	-1.55098300	0.83394884	-2.07922255
H	1.02661660	-1.62885920	-1.85871962
C	-1.51505382	0.34500970	2.13923222
H	-0.93514543	1.24004288	1.99605270
H	-2.41102911	0.63900051	2.64196599
H	-0.96345160	-0.36397003	2.73833400
H	1.23222071	1.63447817	1.11418490
C	3.92084325	0.99687151	1.11323913
H	3.84603027	2.04241366	1.41835784
H	3.76065428	0.45737335	2.05100477
H	4.94670439	0.83445154	0.80914872
H	-1.31302190	-0.88047363	-2.20736938

Time = 309.50 fs
E(CASSCF/6-31G(d)) = -479.5245015

C	-0.89392237	-0.10265354	-0.24153208
C	0.51149275	0.03771215	-0.37969452
C	1.43391080	-0.88747697	-1.21998812
C	1.48547288	0.94989779	0.33448854
C	2.81850147	0.59122426	0.00675606
C	2.70794533	-0.58877300	-0.84365414
N	-1.77352262	-0.12556251	0.77309734
C	-3.14922868	-0.71305180	0.32547648
H	3.64269824	-0.96047623	-1.21217235
C	-1.66325652	-0.09499396	-1.57422798
C	-3.12435759	-0.38267400	-1.21357641
H	-3.70391429	0.50046362	-1.44472956
H	-3.59515635	-1.18196975	-1.77958232
H	-3.16207035	-1.74927355	0.59133158
H	-3.97208570	-0.27646968	0.92395219
H	-1.54840095	0.83679095	-2.08474578
H	1.03313368	-1.63776425	-1.86642072
C	-1.51828383	0.33897818	2.14938858
H	-0.94933877	1.24076407	2.01949990
H	-2.41439316	0.62151077	2.64960862
H	-0.96633345	-0.36793968	2.73879805
H	1.22497220	1.62899605	1.12221043
C	3.92177470	1.00400048	1.10493469
H	3.85712250	2.05890941	1.38356792
H	3.76027760	0.48563256	2.05333395
H	4.94476216	0.82489510	0.79806642

H	-1.30479153	-0.88026887	-2.20674745

Time = 310.00 fs
E(CASSCF/6-31G(d)) = -479.5262514

C	-0.89449330	-0.10115396	-0.24126894
C	0.51401427	0.04117999	-0.38638138
C	1.43519756	-0.88750675	-1.21693453
C	1.48030977	0.94464101	0.33274654
C	2.81564132	0.58864427	0.00952768
C	2.70607905	-0.59557722	-0.83912470
N	-1.77310281	-0.12493797	0.77877114
C	-3.14685703	-0.71023496	0.32200496
H	3.63838468	-0.97060400	-1.19166517
C	-1.66206565	-0.09328599	-1.57484249
C	-3.12031000	-0.37921586	-1.21778098
H	-3.69635454	0.51050738	-1.43721047
H	-3.58869456	-1.17373772	-1.79428908
H	-3.15418941	-1.75597725	0.58681199
H	-3.97843756	-0.28063002	0.91356549
H	-1.54548693	0.84058341	-2.09034120
H	1.04052767	-1.64713507	-1.87445084
C	-1.52140349	0.33296457	2.15918687
H	-0.96273319	1.24193871	2.04407935
H	-2.41947920	0.60366702	2.65752928
H	-0.96876692	-0.37267078	2.73970481
H	1.21749467	1.62396987	1.12999184
C	3.92255565	1.01108952	1.09638377
H	3.86777765	2.07510652	1.34753881
H	3.75975172	0.51317795	2.05454247
H	4.94285238	0.81507870	0.78659887
H	-1.29562453	-0.88044903	-2.20734627

Time = 310.50 fs
E(CASSCF/6-31G(d)) = -479.5279942

C	-0.89478823	-0.09961544	-0.24146743
C	0.51684955	0.04476182	-0.39285903
C	1.43609628	-0.88717581	-1.21371609
C	1.47519933	0.93897274	0.33094605
C	2.81234552	0.58623476	0.01294813
C	2.70450481	-0.60225152	-0.83468113
N	-1.77309704	-0.12426856	0.78458669
C	-3.14412419	-0.70709553	0.31861347
H	3.63404198	-0.98297342	-1.17222176
C	-1.66089251	-0.09199867	-1.57527591
C	-3.11644675	-0.37577723	-1.22181002

H	-3.68940202	0.52062458	-1.42885617
H	-3.58154268	-1.16558139	-1.80887227
H	-3.14600169	-1.76344050	0.58118387
H	-3.98311005	-0.28432847	0.90163927
H	-1.54226604	0.84500464	-2.09581001
H	1.04901040	-1.65625873	-1.88217124
C	-1.52442904	0.32698442	2.16859981
H	-0.97524340	1.24360623	2.06965877
H	-2.42648144	0.58546634	2.66583307
H	-0.97050061	-0.37845172	2.74131001
H	1.20990847	1.61901253	1.13701186
C	3.92319093	1.01815069	1.08757798
H	3.87803824	2.09082689	1.31034913
H	3.75906466	0.54002113	2.05471335
H	4.94089106	0.80502608	0.77481974
H	-1.28569165	-0.88078674	-2.20894818

Time = 311.00 fs
E(CASSCF/6-31G(d)) = -479.5296301

C	-0.89479239	-0.09803113	-0.24210547
C	0.51993420	0.04843732	-0.39911100
C	1.43657599	-0.88656698	-1.21041072
C	1.47015969	0.93295636	0.32914167
C	2.80861718	0.58397695	0.01699490
C	2.70321774	-0.60875139	-0.83028304
N	-1.77348425	-0.12354694	0.79049980
C	-3.14103259	-0.70372511	0.31531016
H	3.63006158	-0.99775401	-1.15405805
C	-1.65970301	-0.09110991	-1.57556561
C	-3.11277554	-0.37234665	-1.22565748
H	-3.68301433	0.53066292	-1.41978315
H	-3.57379908	-1.15743072	-1.82317000
H	-3.13759039	-1.77088674	0.57431096
H	-3.98611084	-0.28752782	0.88831665
H	-1.53877759	0.84967118	-2.10092350
H	1.05872710	-1.66448263	-1.88899355
C	-1.52738237	0.32105434	2.17761498
H	-0.98684331	1.24573716	2.09610228
H	-2.43541959	0.56686631	2.67454302
H	-0.97135360	-0.38548367	2.74379171
H	1.20232418	1.61376469	1.14278252
C	3.92368701	1.02519594	1.07850798
H	3.88794994	2.10587282	1.27209948
H	3.75821446	0.56619720	2.05393008
H	4.93876256	0.79477232	0.76281224
H	-1.27519858	-0.88101848	-2.21129659

Time = 311.50 fs
E(CASSCF/6-31G(d)) = -479.5311548

C	-0.89449546	-0.09639518	-0.24315518
C	0.52320081	0.05218379	-0.40512393
C	1.43662003	-0.88575444	-1.20708846
C	1.46520890	0.92665489	0.32738679
C	2.80446624	0.58184744	0.02164056
C	2.70221406	-0.61503790	-0.82589550
N	-1.77423756	-0.12276451	0.79646248
C	-3.13757957	-0.70021531	0.31209627
H	3.62672246	-1.01503749	-1.13731668
C	-1.65846138	-0.09059369	-1.57575278
C	-3.10930145	-0.36891081	-1.22931674
H	-3.67713376	0.54045891	-1.41012189
H	-3.56558210	-1.14922989	-1.83702877
H	-3.12904867	-1.77758315	0.56609359
H	-3.98753670	-0.29016709	0.87380786
H	-1.53506993	0.85417902	-2.10544561
H	1.06973807	-1.67125975	-1.89442063
C	-1.53028935	0.31518944	2.18623642
H	-0.99756560	1.24822958	2.12327035
H	-2.44613333	0.54779203	2.68359830
H	-0.97123819	-0.39385239	2.74723059
H	1.19483188	1.60792577	1.14688409
C	3.92405127	1.03223629	1.06916394
H	3.89755914	2.12004007	1.23291378
H	3.75720939	0.59176246	2.05227365
H	4.93633096	0.78436247	0.75066504
H	-1.26437151	-0.88087476	-2.21411950

Time = 312.00 fs
E(CASSCF/6-31G(d)) = -479.5326159

C	-0.89389141	-0.09470301	-0.24458345
C	0.52657993	0.05597632	-0.41088813
C	1.43622710	-0.88480102	-1.20380889
C	1.46036574	0.92012902	0.32573101
C	2.79990956	0.57981870	0.02685346
C	2.70149404	-0.62107999	-0.82149014
N	-1.77532502	-0.12191131	0.80242399
C	-3.13375836	-0.69665276	0.30896570
H	3.62415033	-1.03481771	-1.12205994
C	-1.65713212	-0.09042151	-1.57587860
C	-3.10602621	-0.36545507	-1.23278038
H	-3.67169332	0.54984902	-1.40001620

H	-3.55702940	-1.14094184	-1.85030818
H	-3.12047572	-1.78289352	0.55647945
H	-3.98756709	-0.29216514	0.85838210
H	-1.53119584	0.85814268	-2.10915385
H	1.08201118	-1.67617696	-1.89807397
C	-1.53317750	0.30940075	2.19448454
H	-1.00749724	1.25091284	2.15101894
H	-2.45829989	0.52814830	2.69286383
H	-0.97017173	-0.40351347	2.75160041
H	1.18749151	1.60127639	1.14899534
C	3.92429099	1.03928094	1.05953595
H	3.90691174	2.13313268	1.19293745
H	3.75606825	0.61679338	2.04982169
H	4.93345282	0.77384788	0.73846786
H	-1.25344343	-0.88010762	-2.21715191

Time = 312.50 fs
E(CASSCF/6-31G(d)) = -479.5340368

C	-0.89297776	-0.09295104	-0.24635335
C	0.53000059	0.05978794	-0.41639725
C	1.43541159	-0.88375666	-1.20061949
C	1.45564967	0.91343545	0.32421852
C	2.79497056	0.57785973	0.03259816
C	2.70106277	-0.62685603	-0.81704604
N	-1.77671061	-0.12097650	0.80833217
C	-3.12955919	-0.69311512	0.30590553
H	3.62229858	-1.05697729	-1.10826779
C	-1.65568162	-0.09056313	-1.57598118
C	-3.10294781	-0.36196434	-1.23603984
H	-3.66662322	0.55868186	-1.38962246
H	-3.54829629	-1.13255158	-1.86288568
H	-3.11197262	-1.78631494	0.54546866
H	-3.98645346	-0.29342668	0.84235409
H	-1.52720713	0.86122719	-2.11185777
H	1.09542350	-1.67897042	-1.89970782
C	-1.53607370	0.30369324	2.20239376
H	-1.01676872	1.25356079	2.17919759
H	-2.47147040	0.50783638	2.70214987
H	-0.96827392	-0.41429583	2.75677457
H	1.18032979	1.59369142	1.14891050
C	3.92441263	1.04633661	1.04961474
H	3.91605156	2.14497591	1.15233560
H	3.75481981	0.64138320	2.04664585
H	4.92999006	0.76328313	0.72630690
H	-1.24263977	-0.87851441	-2.22015420

Time = 313.00 fs
E(CASSCF/6-31G(d)) = -479.5353963

C	-0.89175570	-0.09113687	-0.24842455
C	0.53339188	0.06358987	-0.42164845
C	1.43420287	-0.88265754	-1.19755462
C	1.45108078	0.90662595	0.32288678
C	2.78967908	0.57593657	0.03883602
C	2.70092970	-0.63235428	-0.81254951
N	-1.77835481	-0.11994849	0.81413431
C	-3.12497141	-0.68966841	0.30289723
H	3.62095455	-1.08128561	-1.09584608
C	-1.65407966	-0.09098769	-1.57609303
C	-3.10006011	-0.35842375	-1.23908551
H	-3.66185669	0.56682841	-1.37910800
H	-3.53955274	-1.12406792	-1.87465870
H	-3.10363744	-1.78749718	0.53311471
H	-3.98449996	-0.29385049	0.82606824
H	-1.52315118	0.86317259	-2.11341224
H	1.10976701	-1.67953322	-1.89921868
C	-1.53900237	0.29806471	2.21000910
H	-1.02554114	1.25590894	2.20764692
H	-2.48511700	0.48677026	2.71123564
H	-0.96575151	-0.42592129	2.76254270
H	1.17333705	1.58514760	1.14655186
C	3.92442092	1.05340704	1.03939219
H	3.92501895	2.15542855	1.11129011
H	3.75350207	0.66563758	2.04281115
H	4.92582189	0.75272293	0.71426089
H	-1.23216738	-0.87595482	-2.22292703

Time = 313.50 fs
E(CASSCF/6-31G(d)) = -479.5366032

C	-0.89022946	-0.08925920	-0.25075450
C	0.53668377	0.06735179	-0.42664231
C	1.43264427	-0.88152561	-1.19463492
C	1.44667959	0.89974646	0.32176582
C	2.78407093	0.57401310	0.04552593
C	2.70110614	-0.63757269	-0.80799321
N	-1.78021544	-0.11881510	0.81977828
C	-3.11998596	-0.68636471	0.29991842
H	3.61977032	-1.10740652	-1.08464204
C	-1.65230074	-0.09166445	-1.57623939
C	-3.09735268	-0.35481953	-1.24190688
H	-3.65733503	0.57419161	-1.36864829
H	-3.53097960	-1.11552269	-1.88554598

H	-3.09556184	-1.78625475	0.51952210
H	-3.98203716	-0.29334107	0.80987792
H	-1.51906741	0.86381066	-2.11372687
H	1.12475763	-1.67791892	-1.89664910
C	-1.54198431	0.29250570	2.21738170
H	-1.03399214	1.25767693	2.23619834
H	-2.49868376	0.46488950	2.71989433
H	-0.96287215	-0.43803680	2.76863703
H	1.16646636	1.57572694	1.14197441
C	3.92431830	1.06049258	1.02886200
H	3.93385053	2.16439291	1.06999357
H	3.75216150	0.68967221	2.03837552
H	4.92085461	0.74221741	0.70239764
H	-1.22220481	-0.87236230	-2.22532176

Time = 314.00 fs
E(CASSCF/6-31G(d)) = -479.5375168

C	-0.88840597	-0.08731781	-0.25329923
C	0.53980812	0.07104216	-0.43138269
C	1.43079159	-0.88036853	-1.19186723
C	1.44246668	0.89283663	0.32087792
C	2.77818749	0.57205172	0.05262494
C	2.70160172	-0.64251764	-0.80337496
N	-1.78224831	-0.11756378	0.82521345
C	-3.11459825	-0.68324034	0.29694503
H	3.61831086	-1.13491712	-1.07446328
C	-1.65032498	-0.09256342	-1.57643708
C	-3.09481091	-0.35113976	-1.24449298
H	-3.65301149	0.58071354	-1.35842365
H	-3.52276369	-1.10696844	-1.89548706
H	-3.08782785	-1.78257213	0.50484218
H	-3.97938930	-0.29182241	0.79412278
H	-1.51498476	0.86307466	-2.11277125
H	1.14004629	-1.67434139	-1.89218826
C	-1.54503619	0.28700008	2.22456423
H	-1.04230196	1.25859167	2.26467494
H	-2.51163360	0.44216786	2.72791528
H	-0.95993362	-0.45025274	2.77476184
H	1.15963405	1.56561599	1.13536514
C	3.92410453	1.06759006	1.01802028
H	3.94257909	2.17182197	1.02864288
H	3.75085183	0.71360956	2.03338971
H	4.91502903	0.73180829	0.69077160
H	-1.21289490	-0.86775002	-2.22724714

Time = 314.50 fs

E(CASSCF/6-31G(d)) = -479.5379707

C	-0.88629457	-0.08531346	-0.25601417
C	0.54269969	0.07462856	-0.43587634
C	1.42871111	-0.87918000	-1.18924478
C	1.43846214	0.88592956	0.32023762
C	2.77207510	0.57001417	0.06008886
C	2.70242058	-0.64720199	-0.79869637
N	-1.78440780	-0.11618193	0.83039145
C	-3.10881131	-0.68031419	0.29395359
H	3.61610929	-1.16333377	-1.06509634
C	-1.64813890	-0.09365599	-1.57669399
C	-3.09241627	-0.34737504	-1.24683285
H	-3.64885382	0.58638000	-1.34861566
H	-3.51509216	-1.09847466	-1.90444016
H	-3.08050534	-1.77660507	0.48926781
H	-3.97683848	-0.28925257	0.77910654
H	-1.51091999	0.86100316	-2.11057686
H	1.15523254	-1.66916970	-1.88616769
C	-1.54817080	0.28152622	2.23160681
H	-1.05064062	1.25840934	2.29289347
H	-2.52348695	0.41861757	2.73512125
H	-0.95723345	-0.46218066	2.78062157
H	1.15272223	1.55510095	1.12703633
C	3.92377642	1.07469288	1.00686613
H	3.95123301	2.17772307	0.98743306
H	3.74963257	0.73757465	2.02789697
H	4.90832515	0.72152630	0.67942230
H	-1.20433981	-0.86221181	-2.22867264

Time = 315.00 fs
E(CASSCF/6-31G(d)) = -479.5378077

C	-0.88390681	-0.08324798	-0.25885485
C	0.54529731	0.07807830	-0.44013271
C	1.42647707	-0.87794088	-1.18674839
C	1.43468490	0.87905192	0.31985235
C	2.76578446	0.56786229	0.06787307
C	2.70355792	-0.65164299	-0.79396146
N	-1.78664742	-0.11465687	0.83526679
C	-3.10263856	-0.67758659	0.29092327
H	3.61272043	-1.19214201	-1.05632549
C	-1.64573577	-0.09491538	-1.57700914
C	-3.09014681	-0.34351905	-1.24891632
H	-3.64484542	0.59122255	-1.33940242
H	-3.50814645	-1.09012189	-1.91237852
H	-3.07365037	-1.76867763	0.47302691

H	-3.97459039	-0.28563738	0.76507759
H	-1.50687656	0.85773860	-2.10723579
H	1.16988070	-1.66291406	-1.87904837
C	-1.55139730	0.27605828	2.23855352
H	-1.05915895	1.25693373	2.32066894
H	-2.53385164	0.39428792	2.74138073
H	-0.95504207	-0.47346616	2.78594380
H	1.14558223	1.54455661	1.11741039
C	3.92332796	1.08179115	0.99540197
H	3.95983736	2.18215926	0.94654837
H	3.74856806	0.76169383	2.02193504
H	4.90076404	0.71138735	0.66837429
H	-1.19659819	-0.85591839	-2.22962808

Time = 315.50 fs
E(CASSCF/6-31G(d)) = -479.5368930

C	-0.88125629	-0.08112419	-0.26177753
C	0.54754484	0.08135886	-0.44416327
C	1.42416864	-0.87662152	-1.18434859
C	1.43115178	0.87222484	0.31972386
C	2.75936992	0.56555872	0.07593303
C	2.70499772	-0.65585983	-0.78917567
N	-1.78892027	-0.11297615	0.83979733
C	-3.09610573	-0.67503887	0.28783748
H	3.60776407	-1.22082598	-1.04794490
C	-1.64311570	-0.09631709	-1.57737317
C	-3.08797787	-0.33956899	-1.25073456
H	-3.64098515	0.59531753	-1.33095467
H	-3.50209601	-1.08199589	-1.91928585
H	-3.06730413	-1.75927338	0.45637470
H	-3.97274856	-0.28104020	0.75221639
H	-1.50284466	0.85352016	-2.10289654
H	1.18354114	-1.65619843	-1.87139570
C	-1.55472176	0.27056748	2.24543985
H	-1.06798040	1.25403155	2.34781888
H	-2.54244342	0.36926243	2.74661558
H	-0.95358221	-0.48381446	2.79049701
H	1.13804346	1.53442756	1.10699476
C	3.92275055	1.08887211	0.98363388
H	3.96841325	2.18524582	0.90615670
H	3.74772542	0.78609029	2.01553541
H	4.89240653	0.70139224	0.65763763
H	-1.18968502	-0.84910979	-2.23020065

Time = 316.00 fs
E(CASSCF/6-31G(d)) = -479.5351698

C	-0.87835872	-0.07894589	-0.26473976
C	0.54939232	0.08443862	-0.44798089
C	1.42186648	-0.87518576	-1.18200944
C	1.42787610	0.86546552	0.31985066
C	2.75288872	0.56306774	0.08422490
C	2.70671150	-0.65987181	-0.78434493
N	-1.79117954	-0.11112766	0.84394464
C	-3.08925157	-0.67263443	0.28468495
H	3.60095739	-1.24889626	-1.03976976
C	-1.64028533	-0.09783911	-1.57776946
C	-3.08588295	-0.33552572	-1.25228094
H	-3.63728550	0.59878222	-1.32343117
H	-3.49709197	-1.07418056	-1.92515094
H	-3.06149300	-1.74901621	0.43958420
H	-3.97130208	-0.27558558	0.74063134
H	-1.49880188	0.84867090	-2.09775655
H	1.19577387	-1.64971377	-1.86383676
C	-1.55814753	0.26502333	2.25229098
H	-1.07719711	1.24964205	2.37416900
H	-2.54909867	0.34365244	2.75080462
H	-0.95301409	-0.49300817	2.79410144
H	1.12992477	1.52519789	1.09634297
C	3.92203359	1.09592074	0.97157168
H	3.97697866	2.18714490	0.86640261
H	3.74717366	0.81088230	2.00872586
H	4.88334990	0.69152600	0.64721025
H	-1.18357417	-0.84208273	-2.23052758

Time = 316.50 fs
E(CASSCF/6-31G(d)) = -479.5326716

C	-0.87523193	-0.07671789	-0.26770077
C	0.55079686	0.08728756	-0.45159907
C	1.41964899	-0.87359660	-1.17969389
C	1.42486591	0.85878981	0.32023140
C	2.74640019	0.56035594	0.09270605
C	2.70865824	-0.66369649	-0.77947516
N	-1.79337898	-0.10909973	0.84767418
C	-3.08212684	-0.67032153	0.28146009
H	3.59213448	-1.27591280	-1.03164222
C	-1.63725711	-0.09946198	-1.57817591
C	-3.08383473	-0.33139378	-1.25355155
H	-3.63376953	0.60176809	-1.31697487
H	-3.49326158	-1.06675154	-1.92996246
H	-3.05622966	-1.73863662	0.42293488
H	-3.97012953	-0.26945581	0.73036326

H	-1.49471510	0.84357783	-2.09205137
H	1.20617611	-1.64415097	-1.85699945
C	-1.56167546	0.25939469	2.25912062
H	-1.08686761	1.24378304	2.39955886
H	-2.55378121	0.31758995	2.75398443
H	-0.95342596	-0.50091856	2.79663563
H	1.12105245	1.51735078	1.08600435
C	3.92116520	1.10292045	0.95922890
H	3.98554826	2.18805591	0.82740247
H	3.74698198	0.83618054	2.00153086
H	4.87372134	0.68175953	0.63708083
H	-1.17820400	-0.83517382	-2.23078532

Time = 317.00 fs
E(CASSCF/6-31G(d)) = -479.5295454

C	-0.87189591	-0.07444598	-0.27062209
C	0.55172350	0.08987796	-0.45503100
C	1.41758871	-0.87182337	-1.17737034
C	1.42212219	0.85221557	0.32086891
C	2.73996485	0.55739272	0.10133552
C	2.71078523	-0.66734822	-0.77457192
N	-1.79547338	-0.10688138	0.85095559
C	-3.07479212	-0.66803824	0.27816302
H	3.58125539	-1.30150353	-1.02343378
C	-1.63404820	-0.10116847	-1.57856719
C	-3.08180616	-0.32718107	-1.25454565
H	-3.63046658	0.60445156	-1.31170946
H	-3.49070326	-1.05977073	-1.93370443
H	-3.05151503	-1.72891948	0.40669962
H	-3.96901756	-0.26288065	0.72139675
H	-1.49054377	0.83866430	-2.08604005
H	1.21441025	-1.64011770	-1.85143740
C	-1.56530412	0.25365093	2.26593040
H	-1.09701625	1.23655283	2.42384696
H	-2.55658396	0.29122024	2.75624788
H	-0.95482886	-0.50751139	2.79803956
H	1.11128348	1.51132049	1.07646622
C	3.92013321	1.10985377	0.94662251
H	3.99413256	2.18820230	0.78924180
H	3.74721769	0.86208472	1.99397025
H	4.86366885	0.67205338	0.62723219
H	-1.17348657	-0.82873868	-2.23117489

Time = 317.50 fs
E(CASSCF/6-31G(d)) = -479.5260503

C	-0.86837298	-0.07213696	-0.27346777
C	0.55214603	0.09218517	-0.45828868
C	1.41574906	-0.86984907	-1.17501938
C	1.41963666	0.84576611	0.32177425
C	2.73364341	0.55415091	0.11007441
C	2.71302939	-0.67083708	-0.76964013
N	-1.79741907	-0.10446232	0.85376273
C	-3.06731445	-0.66571960	0.27479916
H	3.56840790	-1.32537923	-1.01504734
C	-1.63067892	-0.10294307	-1.57891778
C	-3.07977157	-0.32289833	-1.25526600
H	-3.62740678	0.60702226	-1.30773629
H	-3.48948294	-1.05328170	-1.93635288
H	-3.04734092	-1.72063173	0.39113096
H	-3.96769117	-0.25612132	0.71367609
H	-1.48624412	0.83435554	-2.07998742
H	1.22022656	-1.63805191	-1.84755231
C	-1.56902995	0.24776299	2.27270978
H	-1.10763557	1.22812686	2.44691557
H	-2.55772475	0.26469318	2.75773992
H	-0.95715477	-0.51284806	2.79831409
H	1.10052994	1.50744094	1.06809543
C	3.91892628	1.11670329	0.93377256
H	4.00273753	2.18781711	0.75197338
H	3.74794498	0.88868203	1.98605995
H	4.85335107	0.66236238	0.61764588
H	-1.16931980	-0.82312664	-2.23190285

Time = 318.00 fs
E(CASSCF/6-31G(d)) = -479.5224834

C	-0.86468796	-0.06979860	-0.27620478
C	0.55204782	0.09418835	-0.46138198
C	1.41418210	-0.86767625	-1.17263923
C	1.41739005	0.83947305	0.32296956
C	2.72749570	0.55060717	0.11888624
C	2.71531898	-0.67416811	-0.76468396
N	-1.79917438	-0.10183305	0.85607384
C	-3.05976364	-0.66330620	0.27137864
H	3.55380278	-1.34734443	-1.00641719
C	-1.62717091	-0.10477109	-1.57920530
C	-3.07770777	-0.31855823	-1.25571899
H	-3.62461469	0.60966974	-1.30513190
H	-3.48963166	-1.04730707	-1.93787419
H	-3.04369229	-1.71443618	0.37644859
H	-3.96584890	-0.24945173	0.70712225
H	-1.48177450	0.83103895	-2.07414406

H	1.22347790	-1.63815225	-1.84553205
C	-1.57284752	0.24170461	2.27943647
H	-1.11869024	1.21874988	2.46867451
H	-2.55753577	0.23815491	2.75865052
H	-0.96025790	-0.51708275	2.79751823
H	1.08878155	1.50590217	1.06109300
C	3.91753511	1.12345241	0.92070160
H	4.01136351	2.18712675	0.71561766
H	3.74922345	0.91604527	1.97781155
H	4.84292609	0.65264178	0.60830677
H	-1.16560203	-0.81865371	-2.23315971

Time = 318.50 fs
E(CASSCF/6-31G(d)) = -479.5190929

C	-0.86086837	-0.06743969	-0.27880315
C	0.55142252	0.09587108	-0.46431782
C	1.41292721	-0.86532967	-1.17024824
C	1.41535118	0.83337763	0.32448897
C	2.72157952	0.54674242	0.12773713
C	2.71757564	-0.67734080	-0.75970672
N	-1.80070007	-0.09898487	0.85787153
C	-3.05220870	-0.66075286	0.26791565
H	3.53776392	-1.36730408	-0.99750781
C	-1.62354517	-0.10663775	-1.57941396
C	-3.07559504	-0.31417443	-1.25591445
H	-3.62210285	0.61256940	-1.30394621
H	-3.49114469	-1.04184771	-1.93822546
H	-3.04054854	-1.71080481	0.36282998
H	-3.96319760	-0.24313960	0.70164853
H	-1.47710114	0.82902340	-2.06872660
H	1.22412388	-1.64034504	-1.84532158
C	-1.57675007	0.23545347	2.28607743
H	-1.13012281	1.20872339	2.48906338
H	-2.55644531	0.21174015	2.75920442
H	-0.96391929	-0.52045518	2.79576388
H	1.07612085	1.50672454	1.05547268
C	3.91595371	1.13008617	0.90743407
H	4.02000396	2.18633467	0.68016514
H	3.75110676	0.94423132	1.96923146
H	4.83254025	0.64285449	0.59920748
H	-1.16224725	-0.81557591	-2.23509754

Time = 319.00 fs
E(CASSCF/6-31G(d)) = -479.5160018

C	-0.85694456	-0.06507001	-0.28123622

C	0.55027474	0.09722184	-0.46709948
C	1.41201090	-0.86285498	-1.16788382
C	1.41347730	0.82753004	0.32637699
C	2.71594924	0.54254209	0.13659603
C	2.71971655	-0.68034898	-0.75471089
N	-1.80195976	-0.09590996	0.85914291
C	-3.04471493	-0.65803559	0.26442748
H	3.52071346	-1.38526559	-0.98831145
C	-1.61982032	-0.10852713	-1.57953750
C	-3.07341797	-0.30976043	-1.25586545
H	-3.61986564	0.61586902	-1.30420127
H	-3.49398222	-1.03688390	-1.93735698
H	-3.03788300	-1.70995036	0.35040592
H	-3.95948241	-0.23742951	0.69717319
H	-1.47220334	0.82850330	-2.06390080
H	1.22222448	-1.64429802	-1.84663560
C	-1.58073024	0.22899225	2.29259058
H	-1.14186106	1.19838808	2.50805200
H	-2.55494999	0.18556577	2.75964686
H	-0.96785580	-0.52327798	2.79320769
H	1.06272646	1.50975750	1.05106934
C	3.91418051	1.13659190	0.89399562
H	4.02864365	2.18560632	0.64558045
H	3.75364128	0.97327968	1.96031947
H	4.82231839	0.63297856	0.59035209
H	-1.15919876	-0.81406662	-2.23780991

Time = 319.50 fs
E(CASSCF/6-31G(d)) = -479.5131887

C	-0.85294990	-0.06270033	-0.28348069
C	0.54862061	0.09823433	-0.46972601
C	1.41144740	-0.86031364	-1.16559804
C	1.41171569	0.82198669	0.32868470
C	2.71065433	0.53799637	0.14543483
C	2.72165700	-0.68318118	-0.74969827
N	-1.80292034	-0.09260133	0.85987961
C	-3.03734161	-0.65515527	0.26093354
H	3.50315132	-1.40133450	-0.97884443
C	-1.61601131	-0.11042162	-1.57958123
C	-3.07116601	-0.30532848	-1.25558779
H	-3.61787434	0.61967721	-1.30589058
H	-3.49807145	-1.03237810	-1.93521625
H	-3.03566069	-1.71179408	0.33926125
H	-3.95451002	-0.23252940	0.69362884
H	-1.46707748	0.82953375	-2.05977064
H	1.21792586	-1.64947619	-1.84900833

C	-1.58478116	0.22230933	2.29892734
H	-1.15382681	1.18810172	2.52563876
H	-2.55357715	0.15972652	2.76022527
H	-0.97173426	-0.52591765	2.79003936
H	1.04886294	1.51470272	1.04757477
C	3.91221929	1.14295966	0.88041248
H	4.03725697	2.18505717	0.61180722
H	3.75686502	1.00321166	1.95106752
H	4.81235653	0.62301389	0.58175876
H	-1.15643877	-0.81420124	-2.24131704

Time = 320.00 fs
E(CASSCF/6-31G(d)) = -479.5105323

C	-0.84892085	-0.06034232	-0.28551674
C	0.54648823	0.09890759	-0.47219183
C	1.41123975	-0.85777586	-1.16345156
C	1.41000645	0.81680598	0.33146454
C	2.70573796	0.53310041	0.15422834
C	2.72331314	-0.68582154	-0.74467013
N	-1.80355231	-0.08905304	0.86007770
C	-3.03014062	-0.65213718	0.25745427
H	3.48562867	-1.41570227	-0.96914163
C	-1.61212883	-0.11230168	-1.57956260
C	-3.06883378	-0.30088874	-1.25509950
H	-3.61607408	0.62405523	-1.30897888
H	-3.50330986	-1.02827899	-1.93175345
H	-3.03383525	-1.71597899	0.32943975
H	-3.94816451	-0.22860097	0.69096850
H	-1.46173851	0.83202181	-2.05637520
H	1.21144170	-1.65522328	-1.85186544
C	-1.58889760	0.21539890	2.30503591
H	-1.16594532	1.17821348	2.54184673
H	-2.55283877	0.13429404	2.76116806
H	-0.97519213	-0.52876824	2.78646643
H	1.03485938	1.52115590	1.04459114
C	3.91007985	1.14918254	0.86671094
H	4.04580638	2.18474507	0.57877370
H	3.76080648	1.03402937	1.94145815
H	4.80271792	0.61298764	0.57346077
H	-1.15399210	-0.81595175	-2.24555928

Time = 320.50 fs
E(CASSCF/6-31G(d)) = -479.5078982

C	-0.84489687	-0.05800847	-0.28732803
C	0.54391771	0.09924583	-0.47448681

C	1.41138089	-0.85531279	-1.16150685
C	1.40828645	0.81204360	0.33476484
C	2.70123517	0.52785444	0.16295451
C	2.72460542	-0.68825159	-0.73962769
N	-1.80383023	-0.08526020	0.85973778
C	-3.02315543	-0.64902671	0.25401009
H	3.46871555	-1.42862506	-0.95924920
C	-1.60817956	-0.11414631	-1.57951030
C	-3.06642101	-0.29644862	-1.25442023
H	-3.61438342	0.62901311	-1.31340240
H	-3.50956967	-1.02452636	-1.92692760
H	-3.03234621	-1.72192458	0.32095108
H	-3.94041656	-0.22575395	0.68916907
H	-1.45621928	0.83573566	-2.05369407
H	1.20303424	-1.66084728	-1.85459920
C	-1.59307698	0.20826037	2.31086517
H	-1.17815473	1.16903628	2.55671793
H	-2.55318094	0.10931941	2.76266189
H	-0.97786481	-0.53221770	2.78269524
H	1.02108107	1.52865749	1.04169003
C	3.90777820	1.15525668	0.85291695
H	4.05424102	2.18466742	0.54639805
H	3.76548358	1.06571512	1.93146280
H	4.79343274	0.60295720	0.56550583
H	-1.15192317	-0.81919232	-2.25039995

Time = 321.00 fs
E(CASSCF/6-31G(d)) = -479.5052213

C	-0.84092026	-0.05571192	-0.28890164
C	0.54096091	0.09925813	-0.47659629
C	1.41185502	-0.85299013	-1.15982258
C	1.40649323	0.80774805	0.33862516
C	2.69717149	0.52226392	0.17159441
C	2.72546208	-0.69045282	-0.73457258
N	-1.80373302	-0.08121914	0.85886485
C	-3.01642055	-0.64588269	0.25062056
H	3.45296319	-1.44039239	-0.94921551
C	-1.60416717	-0.11593397	-1.57946194
C	-3.06393218	-0.29201252	-1.25357072
H	-3.61269645	0.63451023	-1.31906966
H	-3.51670276	-1.02105606	-1.92071233
H	-3.03111770	-1.72890736	0.31377781
H	-3.93132663	-0.22404372	0.68823250
H	-1.45056723	0.84033008	-2.05165975
H	1.19299593	-1.66569536	-1.85663374
C	-1.59731984	0.20089693	2.31636876

H	-1.19041427	1.16081995	2.57030617
H	-2.55493576	0.08483914	2.76483089
H	-0.97941821	-0.53660927	2.77890992
H	1.00789994	1.53674254	1.03846511
C	3.90533639	1.16118110	0.83905595
H	4.06249636	2.18476402	0.51459260
H	3.77090259	1.09823113	1.92104071
H	4.78450165	0.59301054	0.55795386
H	-1.15032706	-0.82371462	-2.25563727

Time = 321.50 fs
E(CASSCF/6-31G(d)) = -479.5025420

C	-0.83703595	-0.05346645	-0.29022817
C	0.53768109	0.09895812	-0.47850145
C	1.41263852	-0.85086312	-1.15844938
C	1.40456867	0.80395701	0.34307287
C	2.69356168	0.51633949	0.18013214
C	2.72582291	-0.69240966	-0.72950737
N	-1.80324420	-0.07692761	0.85746812
C	-3.00996135	-0.64276996	0.24730384
H	3.43886412	-1.45128823	-0.93908209
C	-1.60009373	-0.11764370	-1.57946081
C	-3.06137587	-0.28758190	-1.25257225
H	-3.61088817	0.64046199	-1.32586318
H	-3.52454684	-1.01780564	-1.91310253
H	-3.03005902	-1.73614899	0.30788181
H	-3.92104342	-0.22347155	0.68818567
H	-1.44483940	0.84538269	-2.05017401
H	1.18163422	-1.66920873	-1.85747306
C	-1.60162951	0.19331361	2.32150875
H	-1.20271021	1.15372869	2.58266975
H	-2.55828436	0.06088311	2.76772165
H	-0.97958290	-0.54220241	2.77525203
H	0.99566771	1.54498226	1.03457321
C	3.90278187	1.16695733	0.82515281
H	4.07049477	2.18492515	0.48326672
H	3.77705738	1.13151956	1.91013893
H	4.77590299	0.58326315	0.55087330
H	-1.14931568	-0.82925039	-2.26102297

Time = 322.00 fs
E(CASSCF/6-31G(d)) = -479.4999888

C	-0.83329097	-0.05128631	-0.29130153
C	0.53415179	0.09836342	-0.48017982
C	1.41370107	-0.84897354	-1.15742706

C	1.40246241	0.80069485	0.34812113
C	2.69040876	0.51009703	0.18855482
C	2.72564249	-0.69411297	-0.72443611
N	-1.80235209	-0.07238489	0.85556085
C	-3.00379412	-0.63975259	0.24407633
H	3.42681479	-1.46154874	-0.92887449
C	-1.59596144	-0.11925643	-1.57955220
C	-3.05876389	-0.28315569	-1.25144613
H	-3.60882150	0.64674902	-1.33364170
H	-3.53293108	-1.01471861	-1.90411878
H	-3.02906695	-1.74289431	0.30320868
H	-3.90979679	-0.22398783	0.68907802
H	-1.43909664	0.85043534	-2.04912586
H	1.16925817	-1.67095854	-1.85673186
C	-1.60601074	0.18551518	2.32625822
H	-1.21505823	1.14782534	2.59386478
H	-2.56323781	0.03748284	2.77129639
H	-0.97818555	-0.54913907	2.77180417
H	0.98469406	1.55301626	1.02976121
C	3.90014653	1.17258898	0.81123193
H	4.07814702	2.18500311	0.45232850
H	3.78392845	1.16550275	1.89869271
H	4.76760185	0.57385274	0.54433657
H	-1.14900280	-0.83549774	-2.26628491

Time = 322.50 fs
E(CASSCF/6-31G(d)) = -479.4977234

C	-0.82973375	-0.04918599	-0.29211888
C	0.53045541	0.09749499	-0.48160594
C	1.41500673	-0.84734800	-1.15678315
C	1.40013461	0.79797108	0.35376816
C	2.68770350	0.50355755	0.19685261
C	2.72489283	-0.69556307	-0.71936470
N	-1.80104998	-0.06759196	0.85316012
C	-2.99792654	-0.63688843	0.24095274
H	3.41708594	-1.47132382	-0.91859584
C	-1.59177431	-0.12075610	-1.57977981
C	-3.05611029	-0.27873094	-1.25021331
H	-3.60635600	0.65322959	-1.34224339
H	-3.54168192	-1.01174822	-1.89381092
H	-3.02802906	-1.74847145	0.29969112
H	-3.89788544	-0.22549768	0.69097729
H	-1.43339796	0.85503437	-2.04840809
H	1.15616706	-1.67066591	-1.85415265
C	-1.61046773	0.17750445	2.33060232
H	-1.22750207	1.14306543	2.60394044

H	-2.56964029	0.01467771	2.77543607
H	-0.97517139	-0.55742258	2.76858129
H	0.97523028	1.56057474	1.02388066
C	3.89746553	1.17808120	0.79731740
H	4.08535486	2.18482628	0.42168502
H	3.79148224	1.20008386	1.88662729
H	4.75956027	0.56493167	0.53841505
H	-1.14948921	-0.84214772	-2.27115037

Time = 323.00 fs
E(CASSCF/6-31G(d)) = -479.4958751

C	-0.82641322	-0.04718002	-0.29268047
C	0.52668122	0.09637649	-0.48275210
C	1.41651500	-0.84599738	-1.15653215
C	1.39755799	0.79577976	0.35999743
C	2.68542444	0.49674711	0.20501861
C	2.72356465	-0.69677186	-0.71430096
N	-1.79933621	-0.06255164	0.85028653
C	-2.99235872	-0.63422528	0.23794634
H	3.40980599	-1.48065013	-0.90822460
C	-1.58753953	-0.12213054	-1.58018256
C	-3.05343033	-0.27430360	-1.24889396
H	-3.60335708	0.65975321	-1.35148979
H	-3.55062847	-1.00886013	-1.88226014
H	-3.02682748	-1.75233223	0.29725153
H	-3.88565884	-0.22786930	0.69396287
H	-1.42779555	0.85876570	-2.04793105
H	1.14264013	-1.66821119	-1.84961346
C	-1.61500184	0.16928143	2.33453754
H	-1.24010823	1.13930163	2.61293676
H	-2.57719317	-0.00748261	2.77995265
H	-0.97061292	-0.56691378	2.76553170
H	0.96745809	1.56749252	1.01689310
C	3.89477594	1.18344020	0.78343304
H	4.09201429	2.18421385	0.39124160
H	3.79967071	1.23514827	1.87386085
H	4.75174740	0.55665806	0.53317438
H	-1.15085023	-0.84890857	-2.27536737

Time = 323.50 fs
E(CASSCF/6-31G(d)) = -479.4944951

C	-0.82337748	-0.04528273	-0.29298956
C	0.52292289	0.09503366	-0.48358909
C	1.41818221	-0.84491680	-1.15667546
C	1.39471916	0.79409950	0.36677859

C	2.68353843	0.48969661	0.21304885
C	2.72166730	-0.69776342	-0.70925415
N	-1.79721423	-0.05726865	0.84696389
C	-2.98708465	-0.63179814	0.23506948
H	3.40496012	-1.48944129	-0.89771753
C	-1.58326846	-0.12337200	-1.58079207
C	-3.05073948	-0.26986938	-1.24750712
H	-3.59970490	0.66617416	-1.36119063
H	-3.55960733	-1.00603396	-1.86957953
H	-3.02534264	-1.75407601	0.29580388
H	-3.87349334	-0.23094496	0.69811616
H	-1.42233061	0.86128285	-2.04763288
H	1.12892663	-1.66363660	-1.84312957
C	-1.61960960	0.16084352	2.33806946
H	-1.25295796	1.13629839	2.62088542
H	-2.58549472	-0.02894003	2.78460799
H	-0.96470287	-0.57734566	2.76254780
H	0.96148268	1.57371677	1.00886836
C	3.89211530	1.18867273	0.76960248
H	4.09801935	2.18298960	0.36090004
H	3.80843121	1.27056589	1.86030856
H	4.74414841	0.54918635	0.52867027
H	-1.15312632	-0.85552600	-2.27872235

Time = 324.00 fs
E(CASSCF/6-31G(d)) = -479.4935471

C	-0.82067258	-0.04350797	-0.29305243
C	0.51927596	0.09349374	-0.48408689
C	1.41996283	-0.84408622	-1.15720153
C	1.39161923	0.79289389	0.37406878
C	2.68200181	0.48244143	0.22094213
C	2.71922715	-0.69857290	-0.70423445
N	-1.79469256	-0.05174977	0.84321898
C	-2.98209437	-0.62962753	0.23233442
H	3.40240437	-1.49749779	-0.88701627
C	-1.57897724	-0.12447745	-1.58163080
C	-3.04805267	-0.26542459	-1.24607046
H	-3.59530242	0.67236363	-1.37114878
H	-3.56846662	-1.00326353	-1.85591328
H	-3.02345648	-1.75346104	0.29525588
H	-3.86176347	-0.23455402	0.70350821
H	-1.41703041	0.86232709	-2.04748618
H	1.11523646	-1.65714433	-1.83485117
C	-1.62428150	0.15218676	2.34120949
H	-1.26613683	1.13375428	2.62781242
H	-2.59408740	-0.04963271	2.78913577

H	-0.95773347	-0.58835383	2.75948407
H	0.95732950	1.57931011	0.99997786
C	3.88952029	1.19378567	0.75584897
H	4.10326613	2.18099443	0.33055833
H	3.81768647	1.30619278	1.84588770
H	4.73677169	0.54265879	0.52494484
H	-1.15631709	-0.86179895	-2.28105384

Time = 324.50 fs
E(CASSCF/6-31G(d)) = -479.4929223

C	-0.81834088	-0.04186893	-0.29287823
C	0.51583500	0.09178486	-0.48421534
C	1.42181092	-0.84347121	-1.15808644
C	1.38827365	0.79211256	0.38181446
C	2.68076196	0.47502104	0.22869990
C	2.71628497	-0.69924403	-0.69925190
N	-1.79178467	-0.04600379	0.83908109
C	-2.97737629	-0.62771811	0.22975431
H	3.40189211	-1.50453368	-0.87605782
C	-1.57468695	-0.12544862	-1.58271081
C	-3.04538353	-0.26096699	-1.24460003
H	-3.59008194	0.67821996	-1.38116569
H	-3.57706910	-1.00055588	-1.84143404
H	-3.02105531	-1.75040868	0.29551092
H	-3.85081006	-0.23852745	0.71018516
H	-1.41190637	0.86174084	-2.04750212
H	1.10173137	-1.64908982	-1.82505691
C	-1.62900165	0.14330743	2.34397135
H	-1.27972470	1.13132889	2.63374264
H	-2.60250503	-0.06950288	2.79326295
H	-0.95006552	-0.59951584	2.75617791
H	0.95494491	1.58444812	0.99048337
C	3.88702551	1.19878564	0.74219519
H	4.10765655	2.17809541	0.30010870
H	3.82734542	1.34187548	1.83052324
H	4.72965356	0.53719732	0.52202459
H	-1.16037864	-0.86759085	-2.28226138

Time = 325.00 fs
E(CASSCF/6-31G(d)) = -479.4924785

C	-0.81641945	-0.04037790	-0.29247907
C	0.51269054	0.08993552	-0.48394483
C	1.42368167	-0.84302450	-1.15929491
C	1.38471123	0.79169280	0.38995366
C	2.67975954	0.46747849	0.23632601

C	2.71289257	-0.69982572	-0.69431560
N	-1.78850891	-0.04004157	0.83458178
C	-2.97291963	-0.62605782	0.22734412
H	3.40310654	-1.51021400	-0.86478481
C	-1.57042354	-0.12629193	-1.58403325
C	-3.04274393	-0.25649646	-1.24311019
H	-3.58400988	0.68367603	-1.39104632
H	-3.58529392	-0.99792895	-1.82633914
H	-3.01803251	-1.74500275	0.29647028
H	-3.84090949	-0.24271179	0.71815252
H	-1.40695298	0.85947468	-2.04773133
H	1.08851758	-1.63996884	-1.81414154
C	-1.63374821	0.13420390	2.34636764
H	-1.29378563	1.12867184	2.63870475
H	-2.61031482	-0.08850417	2.79672908
H	-0.94209382	-0.61039333	2.75247061
H	0.95420146	1.58941088	0.98072027
C	3.88466242	1.20367872	0.72866278
H	4.11110199	2.17419269	0.26943900
H	3.83730364	1.37745369	1.81415364
H	4.72286021	0.53289787	0.51991863
H	-1.16522384	-0.87283630	-2.28231028

Time = 325.50 fs
E(CASSCF/6-31G(d)) = -479.4920616

C	-0.81493789	-0.03904566	-0.29187021
C	0.50992569	0.08797428	-0.48324672
C	1.42553245	-0.84268843	-1.16078233
C	1.38097243	0.79156185	0.39841861
C	2.67893040	0.45985976	0.24382664
C	2.70910946	-0.70036830	-0.68943243
N	-1.78488863	-0.03387604	0.82975467
C	-2.96871629	-0.62461737	0.22512163
H	3.40569616	-1.51419554	-0.85315729
C	-1.56621723	-0.12701828	-1.58558819
C	-3.04014375	-0.25201561	-1.24161337
H	-3.57708958	0.68870480	-1.40060457
H	-3.59303810	-0.99540841	-1.81084631
H	-3.01429093	-1.73748728	0.29803555
H	-3.83224867	-0.24698097	0.72736223
H	-1.40214707	0.85558957	-2.04826484
H	1.07564353	-1.63039351	-1.80259500
C	-1.63849433	0.12487814	2.34840709
H	-1.30836167	1.12544935	2.64273707
H	-2.61714978	-0.10661035	2.79930042
H	-0.93421290	-0.62056988	2.74822344

H	0.95490919	1.59456403	0.97107499
C	3.88245841	1.20847014	0.71527189
H	4.11352525	2.16922078	0.23843033
H	3.84744664	1.41276779	1.79673587
H	4.71648759	0.52982529	0.51861947
H	-1.17072507	-0.87754397	-2.28123239

Time = 326.00 fs
E(CASSCF/6-31G(d)) = -479.4915805

C	-0.81391718	-0.03788166	-0.29106988
C	0.50761413	0.08592900	-0.48209398
C	1.42732331	-0.84239826	-1.16249729
C	1.37710667	0.79164042	0.40713901
C	2.67820849	0.45221294	0.25120988
C	2.70499949	-0.70092002	-0.68460671
N	-1.78095175	-0.02752206	0.82463495
C	-2.96476234	-0.62335045	0.22310778
H	3.40930512	-1.51616506	-0.84115862
C	-1.56210198	-0.12764272	-1.58735518
C	-3.03759069	-0.24753025	-1.24012027
H	-3.56936230	0.69332074	-1.40966697
H	-3.60021592	-0.99302328	-1.79518700
H	-3.00974469	-1.72825698	0.30011112
H	-3.82490879	-0.25124409	0.73770253
H	-1.39744923	0.85025200	-2.04923010
H	1.06310370	-1.62105483	-1.79097046
C	-1.64320930	0.11533677	2.35009272
H	-1.32346669	1.12136868	2.64589082
H	-2.62273366	-0.12382122	2.80078183
H	-0.92678727	-0.62968251	2.74333071
H	0.95683332	1.60033010	0.96195607
C	3.88043622	1.21316408	0.70204062
H	4.11486353	2.16314955	0.20696104
H	3.85765053	1.44766239	1.77825033
H	4.71065779	0.52801202	0.51810325
H	-1.17671987	-0.88179472	-2.27912246

Time = 326.50 fs
E(CASSCF/6-31G(d)) = -479.4909801

C	-0.81336752	-0.03689342	-0.29009970
C	0.50581657	0.08382667	-0.48046157
C	1.42901691	-0.84208724	-1.16438559
C	1.37316909	0.79184725	0.41604591
C	2.67752797	0.44458759	0.25848533
C	2.70062798	-0.70152403	-0.67983956

N	-1.77673097	-0.02099636	0.81925944
C	-2.96105805	-0.62219496	0.22132665
H	3.41360059	-1.51587075	-0.82880125
C	-1.55811429	-0.12818394	-1.58930430
C	-3.03509047	-0.24304969	-1.23863983
H	-3.56090634	0.69757966	-1.41807640
H	-3.60675804	-0.99080134	-1.77960128
H	-3.00432136	-1.71784504	0.30260694
H	-3.81886340	-0.25544754	0.74899554
H	-1.39280442	0.84372471	-2.05078638
H	1.05085172	-1.61266937	-1.77983886
C	-1.64785969	0.10559165	2.35142052
H	-1.33908370	1.11619739	2.64823344
H	-2.62689619	-0.14016734	2.80102374
H	-0.92012801	-0.63744428	2.73772709
H	0.95971896	1.60714448	0.95375717
C	3.87861366	1.21776353	0.68898441
H	4.11506872	2.15598020	0.17490986
H	3.86778503	1.48199193	1.75870400
H	4.70551390	0.52745849	0.51833132
H	-1.18301796	-0.88573541	-2.27613065

Time = 327.00 fs
E(CASSCF/6-31G(d)) = -479.4902978

C	-0.81328706	-0.03608654	-0.28898472
C	0.50457885	0.08169285	-0.47832696
C	1.43057744	-0.84169259	-1.16639495
C	1.36921674	0.79210469	0.42507560
C	2.67682572	0.43703380	0.26566376
C	2.69605934	-0.70221619	-0.67512871
N	-1.77226357	-0.01431725	0.81366632
C	-2.95760730	-0.62107557	0.21980471
H	3.41829300	-1.51314484	-0.81612862
C	-1.55429214	-0.12866359	-1.59139789
C	-3.03264695	-0.23858677	-1.23717934
H	-3.55183395	0.70157487	-1.42569472
H	-3.61260950	-0.98876413	-1.76433102
H	-2.99796320	-1.70689827	0.30544014
H	-3.81398849	-0.25957255	0.76100198
H	-1.38814516	0.83634867	-2.05311707
H	1.03882625	-1.60590982	-1.76973307
C	-1.65241047	0.09566013	2.35237901
H	-1.35516446	1.10977802	2.64985087
H	-2.62958214	-0.15571382	2.79992686
H	-0.91447415	-0.64365971	2.73139170
H	0.96332134	1.61540073	0.94681878

C	3.87700351	1.22227016	0.67611546
H	4.11410755	2.14773872	0.14216036
H	3.87771612	1.51562656	1.73813177
H	4.70121194	0.52813573	0.51925248
H	-1.18941127	-0.88956784	-2.27245030

Time = 327.50 fs
E(CASSCF/6-31G(d)) = -479.4896616

C	-0.81366149	-0.03546503	-0.28775327
C	0.50393106	0.07955130	-0.47567057
C	1.43196824	-0.84116172	-1.16847998
C	1.36530463	0.79234467	0.43417344
C	2.67604335	0.42960129	0.27275666
C	2.69135493	-0.70302348	-0.67046851
N	-1.76759089	-0.00750436	0.80789489
C	-2.95441623	-0.61990774	0.21856955
H	3.42315170	-1.50791847	-0.80321507
C	-1.55067341	-0.12910533	-1.59359291
C	-3.03026262	-0.23415772	-1.23574468
H	-3.54228664	0.70543090	-1.43240407
H	-3.61772769	-0.98692232	-1.74961361
H	-2.99062664	-1.69613801	0.30853682
H	-3.81008313	-0.26362809	0.77343351
H	-1.38339567	0.82851756	-2.05641834
H	1.02698605	-1.60133019	-1.76108960
C	-1.65682610	0.08556496	2.35294932
H	-1.37163097	1.10203687	2.65084747
H	-2.63085342	-0.17056191	2.79744343
H	-0.90998016	-0.64823298	2.72434786
H	0.96743587	1.62539006	0.94139219
C	3.87561377	1.22668432	0.66344209
H	4.11196103	2.13846774	0.10860762
H	3.88730903	1.54845711	1.71659682
H	4.69791155	0.52999034	0.52080543
H	-1.19568542	-0.89353187	-2.26830130

Time = 328.00 fs
E(CASSCF/6-31G(d)) = -479.4892176

C	-0.81446334	-0.03503059	-0.28643699
C	0.50388662	0.07742350	-0.47247614
C	1.43314969	-0.84045838	-1.17060630
C	1.36148265	0.79251404	0.44329651
C	2.67512877	0.42233865	0.27977597
C	2.68657165	-0.70396302	-0.66585009
N	-1.76275819	-0.00057836	0.80198565

C	-2.95149136	-0.61860362	0.21764841
H	3.42801501	-1.50022844	-0.79016387
C	-1.54729422	-0.12953369	-1.59584411
C	-3.02793901	-0.22978162	-1.23434056
H	-3.53242883	0.70929485	-1.43810799
H	-3.62208061	-0.98527216	-1.73567645
H	-2.98228216	-1.68630490	0.31183093
H	-3.80689782	-0.26764125	0.78597118
H	-1.37847713	0.82064438	-2.06088482
H	1.01534657	-1.59929631	-1.75419844
C	-1.66107159	0.07533401	2.35310551
H	-1.38837926	1.09298791	2.65134570
H	-2.63088506	-0.18484956	2.79357617
H	-0.90670687	-0.65117126	2.71666185
H	0.97192257	1.63724483	0.93761146
C	3.87444806	1.23100494	0.65096827
H	4.10862294	2.12821606	0.07416468
H	3.89643116	1.58040032	1.69418823
H	4.69576480	0.53295122	0.52292191
H	-1.20163244	-0.89788453	-2.26391080

Time = 328.50 fs
E(CASSCF/6-31G(d)) = -479.4891030

C	-0.81565201	-0.03478278	-0.28507080
C	0.50444195	0.07532836	-0.46873116
C	1.43407779	-0.83956649	-1.17275304
C	1.35779347	0.79257845	0.45241513
C	2.67403732	0.41529249	0.28673364
C	2.68176053	-0.70504175	-0.66126174
N	-1.75781428	0.00643942	0.79598031
C	-2.94883764	-0.61707938	0.21706667
H	3.43279675	-1.49021785	-0.77710425
C	-1.54418710	-0.12997260	-1.59810753
C	-3.02567729	-0.22547958	-1.23297070
H	-3.52243941	0.71332481	-1.44273056
H	-3.62564460	-0.98379258	-1.72273186
H	-2.97291295	-1.67808741	0.31526138
H	-3.80416325	-0.27164737	0.79828551
H	-1.37331452	0.81312086	-2.06669108
H	1.00400905	-1.59994006	-1.74917241
C	-1.66511361	0.06499995	2.35281535
H	-1.40528417	1.08273164	2.65148427
H	-2.62995506	-0.19874931	2.78837440
H	-0.90461681	-0.65258387	2.70843994
H	0.97671855	1.65090141	0.93548169
C	3.87350640	1.23522963	0.63869317

H	4.10409802	2.11702838	0.03877170
H	3.90495488	1.61140209	1.67101744
H	4.69490502	0.53693776	0.52552904
H	-1.20706410	-0.90287394	-2.25949047

Time = 329.00 fs
E(CASSCF/6-31G(d)) = -479.4893928

C	-0.81717441	-0.03471912	-0.28369250
C	0.50557706	0.07328184	-0.46442726
C	1.43470410	-0.83849090	-1.17491284
C	1.35427192	0.79252360	0.46151262
C	2.67273275	0.40850683	0.29364121
C	2.67696551	-0.70625672	-0.65668943
N	-1.75281105	0.01352738	0.78992177
C	-2.94645678	-0.61526309	0.21684650
H	3.43748798	-1.47813008	-0.76418706
C	-1.54137928	-0.13044388	-1.60034430
C	-3.02347885	-0.22127359	-1.23163820
H	-3.51250268	0.71767656	-1.44621484
H	-3.62840321	-0.98244401	-1.71097289
H	-2.96251421	-1.67204238	0.31876731
H	-3.80161764	-0.27568069	0.81005714
H	-1.36784455	0.80627494	-2.07397194
H	0.99317271	-1.60314833	-1.74594517
C	-1.66892186	0.05459976	2.35204155
H	-1.42220553	1.07144826	2.65141511
H	-2.62842646	-0.21246375	2.78192702
H	-0.90357348	-0.65267723	2.69982399
H	0.98183599	1.66609064	0.93488599
C	3.87278598	1.23935470	0.62661086
H	4.09840016	2.10493544	0.00240552
H	3.91275984	1.64143912	1.64721299
H	4.69543531	0.54186847	0.52855143
H	-1.21182453	-0.90871204	-2.25521436

Time = 329.50 fs
E(CASSCF/6-31G(d)) = -479.4900646

C	-0.81896604	-0.03483512	-0.28234249
C	0.50725662	0.07129677	-0.45956061
C	1.43497765	-0.83725512	-1.17708933
C	1.35094576	0.79235380	0.47058342
C	2.67118757	0.40202247	0.30050951
C	2.67222262	-0.70759592	-0.65211752
N	-1.74780291	0.02066407	0.78385425
C	-2.94434579	-0.61310212	0.21700575

H	3.44215426	-1.46429693	-0.75157983
C	-1.53889107	-0.13096593	-1.60252414
C	-3.02134583	-0.21718512	-1.23034573
H	-3.50279839	0.72248927	-1.44852050
H	-3.63034655	-0.98116877	-1.70057021
H	-2.95109381	-1.66852089	0.32228218
H	-3.79902952	-0.27976684	0.82099537
H	-1.36202319	0.80033136	-2.08280308
H	0.98312498	-1.60858810	-1.74429710
C	-1.67247063	0.04417409	2.35074331
H	-1.43899560	1.05938522	2.65129937
H	-2.62672080	-0.22621810	2.77435207
H	-0.90334573	-0.65174505	2.69098568
H	0.98734578	1.68235912	0.93560917
C	3.87228200	1.24337529	0.61471017
H	4.09155094	2.09194615	-0.03491150
H	3.91973512	1.67051938	1.62291362
H	4.69741854	0.54766916	0.53191281
H	-1.21580047	-0.91554897	-2.25119976

Time = 330.00 fs
E(CASSCF/6-31G(d)) = -479.4910146

C	-0.82095256	-0.03512439	-0.28106324
C	0.50943154	0.06938269	-0.45413212
C	1.43484846	-0.83589662	-1.17929298
C	1.34783728	0.79208815	0.47963030
C	2.66938323	0.39587657	0.30734836
C	2.66755957	-0.70903970	-0.64752958
N	-1.74284618	0.02782860	0.77782326
C	-2.94249578	-0.61056858	0.21755705
H	3.44692691	-1.44912028	-0.73946112
C	-1.53673475	-0.13155272	-1.60462795
C	-3.01928164	-0.21323368	-1.22909582
H	-3.49349222	0.72787136	-1.44962102
H	-3.63147155	-0.97989310	-1.69166972
H	-2.93867547	-1.66761601	0.32572898
H	-3.79621518	-0.28391727	0.83085371
H	-1.35583227	0.79538298	-2.09318545
H	0.97421248	-1.61576032	-1.74390102
C	-1.67574038	0.03376641	2.34887852
H	-1.45550718	1.04683886	2.65130229
H	-2.62528236	-0.24024943	2.76578259
H	-0.90361900	-0.65015273	2.68211846
H	0.99335228	1.69911635	0.93737221
C	3.87198845	1.24728542	0.60297489
H	4.08357871	2.07804291	-0.07310392

H	3.92578087	1.69868050	1.59826049
H	4.70086951	0.55427970	0.53553696
H	-1.21892799	-0.92345304	-2.24749423

Time = 330.50 fs
E(CASSCF/6-31G(d)) = -479.4920959

C	-0.82305176	-0.03557876	-0.27989863
C	0.51204091	0.06754582	-0.44814765
C	1.43427188	-0.83446085	-1.18153629
C	1.34496529	0.79175550	0.48866116
C	2.66730992	0.39010222	0.31416633
C	2.66299594	-0.71056281	-0.64290936
N	-1.73799843	0.03500100	0.77187550
C	-2.94089098	-0.60766140	0.21850692
H	3.45198727	-1.43304735	-0.72801432
C	-1.53491404	-0.13221351	-1.60664915
C	-3.01729116	-0.20943535	-1.22789100
H	-3.48472725	0.73388824	-1.44950084
H	-3.63178286	-0.97853054	-1.68439121
H	-2.92530379	-1.66914622	0.32901846
H	-3.79305100	-0.28812551	0.83944181
H	-1.34928361	0.79137841	-2.10503662
H	0.96680104	-1.62406790	-1.74437534
C	-1.67871924	0.02342167	2.34640644
H	-1.47160220	1.03413046	2.65158684
H	-2.62453483	-0.25479272	2.75635004
H	-0.90401544	-0.64831457	2.67342705
H	0.99996564	1.71569652	0.93986966
C	3.87189865	1.25107814	0.59138422
H	4.07451885	2.06318102	-0.11203831
H	3.93080938	1.72598630	1.57338861
H	4.70575027	0.56165846	0.53934818
H	-1.22119480	-0.93239995	-2.24407124

Time = 331.00 fs
E(CASSCF/6-31G(d)) = -479.4931689

C	-0.82517590	-0.03618849	-0.27889313
C	0.51501428	0.06578906	-0.44161809
C	1.43321274	-0.83299536	-1.18382929
C	1.34234678	0.79138906	0.49768611
C	2.66496639	0.38472816	0.32097050
C	2.65854410	-0.71213697	-0.63824170
N	-1.73331766	0.04216257	0.76605861
C	-2.93950807	-0.60440490	0.21985475
H	3.45754320	-1.41654033	-0.71742135

C	-1.53342421	-0.13295323	-1.60859347
C	-3.01538094	-0.20580162	-1.22673385
H	-3.47661748	0.74055329	-1.44815309
H	-3.63129425	-0.97698642	-1.67882760
H	-2.91104995	-1.67267965	0.33205174
H	-3.78948129	-0.29236516	0.84663419
H	-1.34241999	0.78812893	-2.11819101
H	0.96123451	-1.63288339	-1.74533375
C	-1.68140390	0.01318462	2.34329079
H	-1.48715987	1.02157792	2.65230627
H	-2.62483406	-0.27006472	2.74616662
H	-0.90412436	-0.64666366	2.66511381
H	1.00727746	1.73142266	0.94280360
C	3.87200546	1.25474564	0.57991354
H	4.06441500	2.04729256	-0.15152202
H	3.93474584	1.75252157	1.54841824
H	4.71196915	0.56978386	0.54327118
H	-1.22263803	-0.94227295	-2.24083519

Time = 331.50 fs
E(CASSCF/6-31G(d)) = -479.4941445

C	-0.82723431	-0.03694251	-0.27809073
C	0.51827400	0.06411212	-0.43455939
C	1.43164883	-0.83154486	-1.18617613
C	1.33999823	0.79102159	0.50671517
C	2.66235948	0.37977853	0.32776624
C	2.65421095	-0.71373370	-0.63351342
N	-1.72886141	0.04929610	0.76042068
C	-2.93831587	-0.60084411	0.22159174
H	3.46379848	-1.40004241	-0.70785638
C	-1.53225282	-0.13377344	-1.61047718
C	-3.01355899	-0.20233854	-1.22562689
H	-3.46924384	0.74782357	-1.44557820
H	-3.63003034	-0.97516300	-1.67504489
H	-2.89601603	-1.67759246	0.33472696
H	-3.78552246	-0.29658920	0.85237556
H	-1.33531269	0.78533318	-2.13240953
H	0.95779984	-1.64160655	-1.74642461
C	-1.68379955	0.00309775	2.33950294
H	-1.50208376	1.00946537	2.65359601
H	-2.62642313	-0.28624704	2.73530977
H	-0.90354257	-0.64561523	2.65736246
H	1.01534320	1.74566376	0.94591002
C	3.87230133	1.25827943	0.56853567
H	4.05332107	2.03029429	-0.19130626
H	3.93752860	1.77838512	1.52344665

H	4.71938368	0.57865285	0.54723086
H	-1.22333788	-0.95287433	-2.23763491

Time = 332.00 fs
E(CASSCF/6-31G(d)) = -479.4950034

C	-0.82913606	-0.03782854	-0.27753381
C	0.52173768	0.06251167	-0.42699245
C	1.42957308	-0.83014739	-1.18857258
C	1.33793629	0.79068160	0.51575651
C	2.65950362	0.37527272	0.33455717
C	2.65000027	-0.71532720	-0.62871419
N	-1.72468580	0.05638619	0.75500965
C	-2.93727554	-0.59703839	0.22369989
H	3.47091778	-1.38394427	-0.69947982
C	-1.53138098	-0.13467365	-1.61232417
C	-3.01183449	-0.19904641	-1.22457243
H	-3.46265313	0.75560084	-1.44178376
H	-3.62802807	-0.97296451	-1.67308243
H	-2.88033639	-1.68314671	0.33694809
H	-3.78126335	-0.30073091	0.85668292
H	-1.32805609	0.78261436	-2.14739685
H	0.95670205	-1.64970722	-1.74735847
C	-1.68591843	-0.00680102	2.33502495
H	-1.51630703	0.99801403	2.65556564
H	-2.62939763	-0.30347135	2.72381147
H	-0.90192135	-0.64552706	2.65032033
H	1.02417148	1.75787956	0.94897725
C	3.87277792	1.26167059	0.55722223
H	4.04130363	2.01209823	-0.23109448
H	3.93910917	1.80368229	1.49854057
H	4.72780748	0.58827676	0.55115242
H	-1.22340869	-0.96394574	-2.23428299

Time = 332.50 fs
E(CASSCF/6-31G(d)) = -479.4957875

C	-0.83079280	-0.03883337	-0.27726175
C	0.52532049	0.06098151	-0.41894299
C	1.42699469	-0.82883182	-1.19100476
C	1.33617793	0.79039054	0.52481546
C	2.65642034	0.37122513	0.34134501
C	2.64591536	-0.71689675	-0.62383708
N	-1.72084440	0.06341931	0.74987235
C	-2.93634150	-0.59305461	0.22615135
H	3.47899208	-1.36855538	-0.69243329
C	-1.53078486	-0.13565268	-1.61416244

C	-3.01021720	-0.19591995	-1.22357226
H	-3.45685958	0.76373812	-1.43678556
H	-3.62533803	-0.97030207	-1.67295359
H	-2.86417532	-1.68857089	0.33863419
H	-3.77686185	-0.30470582	0.85964399
H	-1.32076025	0.77956312	-2.16282360
H	0.95804895	-1.65675524	-1.74792461
C	-1.68777726	-0.01648040	2.32985176
H	-1.52979490	0.98735799	2.65829149
H	-2.63368770	-0.32180828	2.71165521
H	-0.89901367	-0.64666259	2.64408139
H	1.03371984	1.76765320	0.95185666
C	3.87342559	1.26491005	0.54594530
H	4.02844424	1.99262412	-0.27055432
H	3.93945207	1.82851652	1.47373079
H	4.73701998	0.59867494	0.55496149
H	-1.22298835	-0.97519411	-2.23057786

Time = 333.00 fs
E(CASSCF/6-31G(d)) = -479.4965590

C	-0.83212145	-0.03994311	-0.27730963
C	0.52893723	0.05951285	-0.41044132
C	1.42393933	-0.82761599	-1.19344849
C	1.33474045	0.79016081	0.53389390
C	2.65313770	0.36764530	0.34812941
C	2.64196113	-0.71842851	-0.61887903
N	-1.71738716	0.07038400	0.74505352
C	-2.93546276	-0.58896099	0.22890827
H	3.48801111	-1.35408459	-0.68683620
C	-1.53043726	-0.13670968	-1.61602037
C	-3.00871692	-0.19294894	-1.22262736
H	-3.45184886	0.77205068	-1.43060966
H	-3.62202492	-0.96709789	-1.67464664
H	-2.84772170	-1.69313013	0.33972676
H	-3.77253715	-0.30841515	0.86141106
H	-1.31354255	0.77577935	-2.17834977
H	0.96184446	-1.66243969	-1.74799904
C	-1.68939374	-0.02591721	2.32399240
H	-1.54254382	0.97752896	2.66181074
H	-2.63906115	-0.34126222	2.69878191
H	-0.89471238	-0.64916419	2.63867382
H	1.04389525	1.77471293	0.95446687
C	3.87423275	1.26798905	0.53467903
H	4.01484157	1.97181219	-0.30933227
H	3.93853441	1.85298037	1.44900938
H	4.74677820	0.60986715	0.55858449

H	-1.22222749	-0.98631903	-2.22632548

Time = 333.50 fs
E(CASSCF/6-31G(d)) = -479.4973675

C	-0.83304698	-0.04114305	-0.27770662
C	0.53250471	0.05809442	-0.40152186
C	1.42044803	-0.82650605	-1.19586966
C	1.33364059	0.78999476	0.54298990
C	2.64968984	0.36453743	0.35490809
C	2.63814592	-0.71991577	-0.61384088
N	-1.71435905	0.07727090	0.74059442
C	-2.93458514	-0.58482230	0.23192358
H	3.49784772	-1.34063541	-0.68278102
C	-1.53030925	-0.13784506	-1.61792338
C	-3.00734271	-0.19011916	-1.22173759
H	-3.44758327	0.78033061	-1.42329472
H	-3.61816775	-0.96328799	-1.67812538
H	-2.83118100	-1.69617969	0.34019458
H	-3.76855509	-0.31175144	0.86218996
H	-1.30651920	0.77090762	-2.19364773
H	0.96798793	-1.66657986	-1.74754895
C	-1.69078313	-0.03509702	2.31746964
H	-1.55457902	0.96845239	2.66611909
H	-2.64514632	-0.36177357	2.68510160
H	-0.88907054	-0.65304049	2.63405238
H	1.05455685	1.77894521	0.95679719
C	3.87518529	1.27089960	0.52340110
H	4.00061150	1.94963264	-0.34707303
H	3.93634618	1.87715044	1.42432767
H	4.75682998	0.62186651	0.56195103
H	-1.22128009	-0.99703963	-2.22135795

Time = 334.00 fs
E(CASSCF/6-31G(d)) = -479.4981945

C	-0.83350467	-0.04241791	-0.27847464
C	0.53594319	0.05671280	-0.39222300
C	1.41657624	-0.82549569	-1.19822421
C	1.33289452	0.78988375	0.55209760
C	2.64611634	0.36190076	0.36167670
C	2.63448159	-0.72135860	-0.60872761
N	-1.71179897	0.08407276	0.73653166
C	-2.93365395	-0.58069628	0.23514240
H	3.50825950	-1.32821283	-0.68033245
C	-1.53037143	-0.13906082	-1.61989130
C	-3.00610232	-0.18741363	-1.22090151

H	-3.44400835	0.78836287	-1.41489458
H	-3.61385861	-0.95882421	-1.68333015
H	-2.81476702	-1.69720049	0.34003590
H	-3.76520846	-0.31460515	0.86222280
H	-1.29979788	0.76466509	-2.20842160
H	0.97627877	-1.66913085	-1.74663114
C	-1.69195563	-0.04401361	2.31031863
H	-1.56594844	0.95995529	2.67117018
H	-2.65147072	-0.38322591	2.67051145
H	-0.88229917	-0.65817348	2.63010104
H	1.06552104	1.78040176	0.95890476
C	3.87626588	1.27363486	0.51209404
H	3.98588734	1.92609498	-0.38343601
H	3.93288986	1.90108019	1.39959962
H	4.76692696	0.63467205	0.56499472
H	-1.22029498	-1.00711668	-2.21554838

Time = 334.50 fs
E(CASSCF/6-31G(d)) = -479.4989665

C	-0.83344234	-0.04375193	-0.27962709
C	0.53917819	0.05535278	-0.38258651
C	1.41239187	-0.82456628	-1.20045919
C	1.33251704	0.78980790	0.56120727
C	2.64246164	0.35972955	0.36842891
C	2.63098266	-0.72276227	-0.60354806
N	-1.70973850	0.09078446	0.73289583
C	-2.93261725	-0.57663123	0.23850427
H	3.51890556	-1.31674245	-0.67952560
C	-1.53059512	-0.14036067	-1.62193626
C	-3.00500166	-0.18481396	-1.22011636
H	-3.44105899	0.79594131	-1.40548033
H	-3.60920107	-0.95367486	-1.69017869
H	-2.79869328	-1.69582047	0.33927814
H	-3.76279043	-0.31687327	0.86176578
H	-1.29347122	0.75686029	-2.22242315
H	0.98642520	-1.67018342	-1.74538865
C	-1.69291513	-0.05266772	2.30258439
H	-1.57671615	0.95178525	2.67687957
H	-2.65750731	-0.40545825	2.65491412
H	-0.87474160	-0.66434134	2.62664249
H	1.07656760	1.77930045	0.96091218
C	3.87745353	1.27618964	0.50074602
H	3.97081807	1.90125286	-0.41811310
H	3.92818084	1.92479689	1.37470679
H	4.77683673	0.64826303	0.56765555
H	-1.21940913	-1.01637024	-2.20882053

Time = 335.00 fs
E(CASSCF/6-31G(d)) = -479.4995667

C	-0.83282198	-0.04512853	-0.28116794
C	0.54214172	0.05399743	-0.37265708
C	1.40797301	-0.82368754	-1.20251385
C	1.33252080	0.78973643	0.57030556
C	2.63877451	0.35801316	0.37515643
C	2.62766473	-0.72413511	-0.59831466
N	-1.70820102	0.09740293	0.72971040
C	-2.93142942	-0.57266423	0.24194603
H	3.52937665	-1.30609572	-0.68036545
C	-1.53095328	-0.14175000	-1.62406143
C	-3.00404435	-0.18230155	-1.21937811
H	-3.43866546	0.80288375	-1.39514109
H	-3.60430739	-0.94782365	-1.69856678
H	-2.78316474	-1.69182605	0.33797613
H	-3.76156253	-0.31846899	0.86106248
H	-1.28761129	0.74740396	-2.23546411
H	0.99805726	-1.66995743	-1.74404319
C	-1.69365926	-0.06106519	2.29431870
H	-1.58695592	0.94363641	2.68313131
H	-2.66272164	-0.42828037	2.63823401
H	-0.86683111	-0.67125178	2.62345378
H	1.08744689	1.77601980	0.96300202
C	3.87872329	1.27856063	0.48935141
H	3.95556547	1.87520510	-0.45084523
H	3.92224744	1.94830150	1.34950484
H	4.78635364	0.66259521	0.56988275
H	-1.21874310	-1.02469248	-2.20115414

Time = 335.50 fs
E(CASSCF/6-31G(d)) = -479.4998623

C	-0.83162125	-0.04653074	-0.28309091
C	0.54477358	0.05262879	-0.36248171
C	1.40340527	-0.82281903	-1.20432139
C	1.33291574	0.78962865	0.57937593
C	2.63510738	0.35673647	0.38184923
C	2.62454207	-0.72548656	-0.59304299
N	-1.70720072	0.10392697	0.72699058
C	-2.93005481	-0.56881998	0.24540471
H	3.53923194	-1.29611730	-0.68282858
C	-1.53142123	-0.14323566	-1.62626032
C	-3.00323159	-0.17985887	-1.21868171
H	-3.43675840	0.80904401	-1.38398448

H	-3.59929547	-0.94126853	-1.70836883
H	-2.76837062	-1.68516608	0.33620960
H	-3.76172227	-0.31933041	0.86031719
H	-1.28226579	0.73631400	-2.24742460
H	1.01074597	-1.66878508	-1.74288176
C	-1.69418065	-0.06921509	2.28557710
H	-1.59674430	0.93517963	2.68978614
H	-2.66661376	-0.45148728	2.62043064
H	-0.85904105	-0.67857979	2.62028495
H	1.09788897	1.77108317	0.96540801
C	3.88004614	1.28074667	0.47791087
H	3.94030110	1.84809606	-0.48143428
H	3.91513156	1.97156832	1.32383427
H	4.79530749	0.67759818	0.57163612
H	-1.21839726	-1.03205433	-2.19258631

Time = 336.00 fs
E(CASSCF/6-31G(d)) = -479.4997436

C	-0.82983418	-0.04794091	-0.28537942
C	0.54702233	0.05122807	-0.35210911
C	1.39877839	-0.82191285	-1.20581140
C	1.33370790	0.78943631	0.58839972
C	2.63151580	0.35588012	0.38849554
C	2.62162557	-0.72682513	-0.58775117
N	-1.70674231	0.11035720	0.72474274
C	-2.92847093	-0.56510984	0.24882043
H	3.54803707	-1.28664954	-0.68686496
C	-1.53197717	-0.14482575	-1.62851664
C	-3.00256200	-0.17747039	-1.21802144
H	-3.43527286	0.81432172	-1.37213532
H	-3.59428501	-0.93401890	-1.71943847
H	-2.75447740	-1.67595373	0.33407987
H	-3.76337330	-0.31942830	0.85967121
H	-1.27745503	0.72371429	-2.25825863
H	1.02402772	-1.66708140	-1.74223601
C	-1.69446856	-0.07712812	2.27641597
H	-1.60615592	0.92609214	2.69669162
H	-2.66875230	-0.47487454	2.60150672
H	-0.85183515	-0.68600243	2.61687777
H	1.10761488	1.76512906	0.96840148
C	3.88138910	1.28274879	0.46643113
H	3.92520187	1.82011090	-0.50975420
H	3.90688866	1.99454903	1.29752982
H	4.80356998	0.69317532	0.57288867
H	-1.21845030	-1.03850811	-2.18320918

Time = 336.50 fs
E(CASSCF/6-31G(d)) = -479.4991387

C	-0.82747126	-0.04934088	-0.28800693
C	0.54884600	0.04977625	-0.34158903
C	1.39418268	-0.82091770	-1.20691309
C	1.33489832	0.78910723	0.59735759
C	2.62805785	0.35542102	0.39508197
C	2.61892086	-0.72815694	-0.58245915
N	-1.70682089	0.11669581	0.72296409
C	-2.92667077	-0.56153130	0.25213858
H	3.55539772	-1.27755191	-0.69240138
C	-1.53260252	-0.14652941	-1.63080475
C	-3.00203181	-0.17512344	-1.21739135
H	-3.43415128	0.81866850	-1.35973406
H	-3.58939381	-0.92609324	-1.73160950
H	-2.74162277	-1.66446585	0.33170621
H	-3.76650540	-0.31877070	0.85918737
H	-1.27317020	0.70982907	-2.26799622
H	1.03743430	-1.66529946	-1.74245250
C	-1.69451060	-0.08481539	2.26689017
H	-1.61525969	0.91608442	2.70369249
H	-2.66879976	-0.49825112	2.58151289
H	-0.84562510	-0.69322773	2.61298256
H	1.11635064	1.75886421	0.97227196
C	3.88271554	1.28457011	0.45492456
H	3.91044579	1.79147023	-0.53575753
H	3.89758770	2.01717650	1.27043123
H	4.81105845	0.70920495	0.57362789
H	-1.21895849	-1.04418477	-2.17316495

Time = 337.00 fs
E(CASSCF/6-31G(d)) = -479.4980417

C	-0.82455960	-0.05071168	-0.29093750
C	0.55021299	0.04825424	-0.33097146
C	1.38970527	-0.81978418	-1.20755911
C	1.33648172	0.78859063	0.60623152
C	2.62479332	0.35533264	0.40159392
C	2.61642689	-0.72948460	-0.57718812
N	-1.70742212	0.12294652	0.72164253
C	-2.92466381	-0.55806805	0.25531110
H	3.56098825	-1.26871566	-0.69934521
C	-1.53328202	-0.14835661	-1.63309062
C	-3.00163495	-0.17280858	-1.21678571
H	-3.43334497	0.82209093	-1.34693311
H	-3.58473384	-0.91751568	-1.74469676

H	-2.72990969	-1.65113785	0.32922224
H	-3.77098684	-0.31740351	0.85884564
H	-1.26937315	0.69497055	-2.27674119
H	1.05052521	-1.66387109	-1.74385574
C	-1.69429424	-0.09228768	2.25705132
H	-1.62411754	0.90492144	2.71064096
H	-2.66652952	-0.52145185	2.56054721
H	-0.84073759	-0.70001483	2.60837102
H	1.12384308	1.75299810	0.97730233
C	3.88398583	1.28621561	0.44340840
H	3.89620717	1.76242041	-0.55947986
H	3.88731047	2.03937281	1.24239193
H	4.81773718	0.72554452	0.57385810
H	-1.21995765	-1.04928642	-2.16263685

Time = 337.50 fs
E(CASSCF/6-31G(d)) = -479.4964788

C	-0.82114207	-0.05203387	-0.29412719
C	0.55110260	0.04664354	-0.32030578
C	1.38542654	-0.81847065	-1.20768992
C	1.33844524	0.78784351	0.61500718
C	2.62178303	0.35558567	0.40801553
C	2.61413522	-0.73080677	-0.57195964
N	-1.70852292	0.12911438	0.72075705
C	-2.92247556	-0.55469084	0.25829679
H	3.56457175	-1.26007216	-0.70758998
C	-1.53400348	-0.15031788	-1.63533341
C	-3.00136342	-0.17051992	-1.21619947
H	-3.43281468	0.82464983	-1.33389359
H	-3.58040811	-0.90831392	-1.75849754
H	-2.71940258	-1.63655303	0.32677150
H	-3.77657301	-0.31540643	0.85855099
H	-1.26599854	0.67952201	-2.28466456
H	1.06291886	-1.66314078	-1.74670597
C	-1.69380787	-0.09955490	2.24694654
H	-1.63278335	0.89243838	2.71740543
H	-2.66183547	-0.54434646	2.53875304
H	-0.83739223	-0.70618735	2.60284618
H	1.12987740	1.74816381	0.98373883
C	3.88515810	1.28769186	0.43190387
H	3.88265230	1.73322488	-0.58103855
H	3.87615082	2.06105454	1.21328819
H	4.82361554	0.74203528	0.57360055
H	-1.22146555	-1.05407285	-2.15183844

Time = 338.00 fs

E(CASSCF/6-31G(d)) = -479.4945057

C	-0.81727632	-0.05328761	-0.29752535
C	0.55150543	0.04492662	-0.30964018
C	1.38141715	-0.81694884	-1.20725750
C	1.34076699	0.78683733	0.62367645
C	2.61908816	0.35614844	0.41433009
C	2.61202981	-0.73211786	-0.56679513
N	-1.71009232	0.13520564	0.72027827
C	-2.92014550	-0.55135917	0.26106068
H	3.56601277	-1.25159725	-0.71701980
C	-1.53475729	-0.15242395	-1.63748763
C	-3.00120762	-0.16825487	-1.21562866
H	-3.43253004	0.82645647	-1.32078141
H	-3.57650838	-0.89851799	-1.77279421
H	-2.71012556	-1.62142244	0.32450253
H	-3.78292899	-0.31288568	0.85815151
H	-1.26295791	0.66391363	-2.29199168
H	1.07431662	-1.66330333	-1.75115974
C	-1.69304165	-0.10662573	2.23661781
H	-1.64130341	0.87855041	2.72387843
H	-2.65473585	-0.56684770	2.51631475
H	-0.83568723	-0.71164074	2.59624955
H	1.13429425	1.74483592	0.99176086
C	3.88618933	1.28900673	0.42043511
H	3.86993591	1.70415294	-0.60062768
H	3.86421304	2.08213933	1.18302526
H	4.82874284	0.75850889	0.57289348
H	-1.22348645	-1.05884317	-2.14100045

Time = 338.50 fs
E(CASSCF/6-31G(d)) = -479.4921683

C	-0.81303348	-0.05445285	-0.30107647
C	0.55142376	0.04308737	-0.29902065
C	1.37773640	-0.81520767	-1.20622834
C	1.34341556	0.78556331	0.63223920
C	2.61676923	0.35698772	0.42052014
C	2.61008724	-0.73340833	-0.56171511
N	-1.71209265	0.14122746	0.72016922
C	-2.91772395	-0.54802466	0.26357295
H	3.56528419	-1.24331137	-0.72751570
C	-1.53553555	-0.15468515	-1.63950608
C	-3.00115681	-0.16601377	-1.21507066
H	-3.43246773	0.82766523	-1.30776299
H	-3.57311374	-0.88815969	-1.78735700
H	-2.70206451	-1.60654869	0.32256346

H	-3.78966144	-0.30996381	0.85746279
H	-1.26014728	0.64859206	-2.29898328
H	1.08451501	-1.66436132	-1.75724120
C	-1.69198808	-0.11350738	2.22610168
H	-1.64971744	0.86325640	2.72998263
H	-2.64537089	-0.58891674	2.49345113
H	-0.83559306	-0.71634451	2.58846508
H	1.13700310	1.74326223	1.00145587
C	3.88703657	1.29016899	0.40902793
H	3.85819780	1.67546843	-0.61850792
H	3.85161010	2.10255150	1.15154260
H	4.83320058	0.77479503	0.57179100
H	-1.22601673	-1.06391187	-2.13035562

Time = 339.00 fs
E(CASSCF/6-31G(d)) = -479.4894772

C	-0.80849643	-0.05550973	-0.30472203
C	0.55087175	0.04111154	-0.28849032
C	1.37443165	-0.81325456	-1.20458461
C	1.34634987	0.78403510	0.64070433
C	2.61488511	0.35806936	0.42656769
C	2.60827735	-0.73466504	-0.55673870
N	-1.71448095	0.14718784	0.72038646
C	-2.91526808	-0.54463577	0.26580768
H	3.56246765	-1.23527745	-0.73896072
C	-1.53633081	-0.15711088	-1.64134329
C	-3.00119950	-0.16379902	-1.21452441
H	-3.43260861	0.82846340	-1.29500129
H	-3.57029005	-0.87727316	-1.80194798
H	-2.69517394	-1.59277211	0.32109568
H	-3.79635477	-0.30676874	0.85629401
H	-1.25745762	0.63398433	-2.30590975
H	1.09340388	-1.66611262	-1.76483182
C	-1.69064255	-0.12020532	2.21542939
H	-1.65805999	0.84663724	2.73567474
H	-2.63399445	-0.61056603	2.47040775
H	-0.83695184	-0.72034087	2.57942153
H	1.13798910	1.74342713	1.01280548
C	3.88765826	1.29118801	0.39770843
H	3.84756124	1.64741815	-0.63499089
H	3.83846187	2.12222648	1.11881652
H	4.83709144	0.79073000	0.57036080
H	-1.22905168	-1.06958099	-2.12012334

Time = 339.50 fs
E(CASSCF/6-31G(d)) = -479.4864136

C	-0.80375794	-0.05643881	-0.30840237
C	0.54987539	0.03898692	-0.27808876
C	1.37153898	-0.81111363	-1.20232340
C	1.34952008	0.78228798	0.64908936
C	2.61349160	0.35935897	0.43245434
C	2.60656417	-0.73587187	-0.55188312
N	-1.71721041	0.15309535	0.72088099
C	-2.91283740	-0.54114414	0.26774173
H	3.55774711	-1.22759531	-0.75124467
C	-1.53713470	-0.15970881	-1.64295935
C	-3.00132394	-0.16161410	-1.21399044
H	-3.43293378	0.82905835	-1.28265189
H	-3.56809048	-0.86589626	-1.81632580
H	-2.68938948	-1.58089748	0.32022720
H	-3.80260615	-0.30342330	0.85447167
H	-1.25478709	0.62045971	-2.31302061
H	1.10095334	-1.66817138	-1.77368004
C	-1.68900399	-0.12672325	2.20462737
H	-1.66636182	0.82884968	2.74094772
H	-2.62095925	-0.63185879	2.44744773
H	-0.83948144	-0.72374045	2.56909262
H	1.13731281	1.74505569	1.02568605
C	3.88801578	1.29207343	0.38650153
H	3.83813135	1.62022138	-0.65042071
H	3.82489240	2.14111531	1.08486202
H	4.84052786	0.80616468	0.56868140
H	-1.23259217	-1.07611096	-2.11049461

Time = 340.00 fs
E(CASSCF/6-31G(d)) = -479.4829552

C	-0.79891865	-0.05722130	-0.31205850
C	0.54847210	0.03670370	-0.26785124
C	1.36908433	-0.80882173	-1.19945436
C	1.35286913	0.78037448	0.65741867
C	2.61264025	0.36082258	0.43816167
C	2.60490760	-0.73701048	-0.54716345
N	-1.72023197	0.15895888	0.72159943
C	-2.91048929	-0.53751174	0.26935388
H	3.55139645	-1.22039390	-0.76426731
C	-1.53793661	-0.16248420	-1.64432344
C	-3.00151850	-0.15946225	-1.21347075
H	-3.43342016	0.82966311	-1.27085975
H	-3.56655691	-0.85407291	-1.83025146
H	-2.68464487	-1.57160951	0.32006608
H	-3.80805672	-0.30003586	0.85185897

H	-1.25205408	0.60829382	-2.32051361
H	1.10719353	-1.67001799	-1.78342933
C	-1.68707561	-0.13306314	2.19371806
H	-1.67465133	0.81011487	2.74583059
H	-2.60669388	-0.65290422	2.42484163
H	-0.84278553	-0.72671454	2.55749542
H	1.13510261	1.74765774	1.03988418
C	3.88807490	1.29283497	0.37542928
H	3.82999561	1.59405959	-0.66514973
H	3.81102753	2.15918654	1.04973122
H	4.84361757	0.82097299	0.56683846
H	-1.23665041	-1.08369354	-2.10161945

Time = 340.50 fs
E(CASSCF/6-31G(d)) = -479.4791201

C	-0.79408499	-0.05783948	-0.31563352
C	0.54671031	0.03425464	-0.25780838
C	1.36708504	-0.80642289	-1.19599611
C	1.35633550	0.77835762	0.66572087
C	2.61237653	0.36242753	0.44367133
C	2.60326530	-0.73806139	-0.54259254
N	-1.72349550	0.16478730	0.72248500
C	-2.90827523	-0.53371791	0.27062435
H	3.54376045	-1.21382093	-0.77794038
C	-1.53872254	-0.16543941	-1.64541684
C	-3.00177212	-0.15734517	-1.21296866
H	-3.43403586	0.83048103	-1.25975669
H	-3.56572158	-0.84185542	-1.84349391
H	-2.68088960	-1.56538773	0.32069502
H	-3.81241530	-0.29669311	0.84836929
H	-1.24920904	0.59764112	-2.32850687
H	1.11219241	-1.67106346	-1.79365811
C	-1.68486569	-0.13922579	2.18272135
H	-1.68295498	0.79070245	2.75038420
H	-2.59167175	-0.67384649	2.40285575
H	-0.84637160	-0.72948235	2.54468512
H	1.13153985	1.75060111	1.05512342
C	3.88780706	1.29348217	0.36450933
H	3.82322516	1.56906975	-0.67951275
H	3.79699286	2.17642683	1.01351145
H	4.84645058	0.83505849	0.56492010
H	-1.24125352	-1.09242963	-2.09359828

Time = 341.00 fs
E(CASSCF/6-31G(d)) = -479.4749752

C	-0.78936674	-0.05827692	-0.31907416
C	0.54464823	0.03163523	-0.24798563
C	1.36555098	-0.80396286	-1.19197274
C	1.35985574	0.77630341	0.67402567
C	2.61273852	0.36414309	0.44896560
C	2.60159553	-0.73900520	-0.53818106
N	-1.72695147	0.17058928	0.72347871
C	-2.90623756	-0.52976448	0.27153456
H	3.53522801	-1.20802976	-0.79218598
C	-1.53947475	-0.16857377	-1.64623462
C	-3.00207455	-0.15526179	-1.21248847
H	-3.43473565	0.83169128	-1.24945954
H	-3.56560937	-0.82930700	-1.85583657
H	-2.67810408	-1.56244218	0.32216644
H	-3.81547632	-0.29345495	0.84397415
H	-1.24624225	0.58852082	-2.33702127
H	1.11603685	-1.67071510	-1.80392112
C	-1.68238828	-0.14521187	2.17165638
H	-1.69129740	0.77091098	2.75469529
H	-2.57637317	-0.69484871	2.38174078
H	-0.84967637	-0.73229408	2.53074769
H	1.12684253	1.75319392	1.07109487
C	3.88719050	1.29402437	0.35375346
H	3.81787664	1.54533955	-0.69380158
H	3.78291119	2.19283989	0.97632081
H	4.84908712	0.84835937	0.56301280
H	-1.24644413	-1.10231714	-2.08647828

Time = 341.50 fs
E(CASSCF/6-31G(d)) = -479.4706515

C	-0.78487445	-0.05851861	-0.32233147
C	0.54235244	0.02884343	-0.23840339
C	1.36448536	-0.80148428	-1.18741012
C	1.36336791	0.77427376	0.68236115
C	2.61375479	0.36594111	0.45402732
C	2.59986012	-0.73982379	-0.53393791
N	-1.73055183	0.17637281	0.72452017
C	-2.90440791	-0.52567794	0.27206703
H	3.52620004	-1.20316427	-0.80693254
C	-1.54017200	-0.17188395	-1.64678579
C	-3.00241667	-0.15320725	-1.21203518
H	-3.43545776	0.83343670	-1.24006905
H	-3.56623994	-0.81650377	-1.86708319
H	-2.67630582	-1.56268397	0.32449981
H	-3.81713076	-0.29035158	0.83870669
H	-1.24318534	0.58081975	-2.34597527

H	1.11881773	-1.66843229	-1.81378871
C	-1.67966345	-0.15102358	2.16054387
H	-1.69969998	0.75104481	2.75886602
H	-2.56124245	-0.71607067	2.36172023
H	-0.85210216	-0.73540699	2.51578754
H	1.12124676	1.75476209	1.08748733
C	3.88621100	1.29447064	0.34316654
H	3.81399439	1.52290653	-0.70824280
H	3.76890119	2.20844428	0.93830346
H	4.85154930	0.86085083	0.56119687
H	-1.25227693	-1.11324969	-2.08025490

Time = 342.00 fs
E(CASSCF/6-31G(d)) = -479.4663131

C	-0.78071683	-0.05855115	-0.32536191
C	0.53989593	0.02587980	-0.22907673
C	1.36388504	-0.79902314	-1.18233352
C	1.36681407	0.77232094	0.69075119
C	2.61544346	0.36779643	0.45884054
C	2.59802816	-0.74050190	-0.52987068
N	-1.73425131	0.18214499	0.72554873
C	-2.90280658	-0.52150755	0.27220552
H	3.51705360	-1.19934286	-0.82210706
C	-1.54079066	-0.17536451	-1.64709184
C	-3.00279055	-0.15117221	-1.21161405
H	-3.43612197	0.83581508	-1.23166940
H	-3.56762977	-0.80353602	-1.87706353
H	-2.67554654	-1.56574101	0.32768012
H	-3.81737150	-0.28738278	0.83266082
H	-1.24010747	0.57431027	-2.35519431
H	1.12062073	-1.66376853	-1.82287739
C	-1.67671674	-0.15666691	2.14940870
H	-1.70817877	0.73139027	2.76300176
H	-2.54664660	-0.73764450	2.34298094
H	-0.85306125	-0.73905705	2.49991309
H	1.11499134	1.75471100	1.10401273
C	3.88486235	1.29482995	0.33274593
H	3.81161180	1.50176041	-0.72298169
H	3.75507516	2.22326894	0.89962321
H	4.85381675	0.87254490	0.55954351
H	-1.25881273	-1.12502703	-2.07487806

Time = 342.50 fs
E(CASSCF/6-31G(d)) = -479.4621451

C	-0.77699810	-0.05836289	-0.32812749

C	0.53735592	0.02274726	-0.22001580
C	1.36374054	-0.79660646	-1.17676554
C	1.37014268	0.77048373	0.69921367
C	2.61781097	0.36968712	0.46339066
C	2.59607947	-0.74102836	-0.52598628
N	-1.73800831	0.18791167	0.72650426
C	-2.90144353	-0.51732030	0.27193521
H	3.50810764	-1.19664314	-0.83762536
C	-1.54130644	-0.17900884	-1.64718413
C	-3.00318940	-0.14914271	-1.21123031
H	-3.43662988	0.83887461	-1.22432812
H	-3.56979355	-0.79050836	-1.88563765
H	-2.67589943	-1.57101195	0.33165867
H	-3.81629256	-0.28451907	0.82598707
H	-1.23710608	0.56868042	-2.36443140
H	1.12152021	-1.65639834	-1.83087346
C	-1.67357769	-0.16215410	2.13828242
H	-1.71674090	0.71219244	2.76719596
H	-2.53284090	-0.75965014	2.32566549
H	-0.85202688	-0.74342679	2.48321976
H	1.10830326	1.75256942	1.12042597
C	3.88314632	1.29511137	0.32248152
H	3.81075233	1.48184921	-0.73807446
H	3.74153814	2.23734799	0.86045777
H	4.85582753	0.88348720	0.55811233
H	-1.26611010	-1.13737451	-2.07026179

Time = 343.00 fs
E(CASSCF/6-31G(d)) = -479.4583162

C	-0.77381471	-0.05794410	-0.33059594
C	0.53481080	0.01945086	-0.21122626
C	1.36403598	-0.79425100	-1.17072488
C	1.37331072	0.76878510	0.70775936
C	2.62085139	0.37159458	0.46766485
C	2.59400748	-0.74139719	-0.52229174
N	-1.74178562	0.19367735	0.72732817
C	-2.90032006	-0.51319368	0.27124294
H	3.49959436	-1.19509014	-0.85338102
C	-1.54169638	-0.18281001	-1.64710038
C	-3.00360745	-0.14710040	-1.21088897
H	-3.43686729	0.84261444	-1.21809629
H	-3.57274526	-0.77753883	-1.89269930
H	-2.67744188	-1.57774610	0.33635517
H	-3.81408357	-0.28170466	0.81888539
H	-1.23429451	0.56357015	-2.37339607
H	1.12157627	-1.64613183	-1.83754893

C	-1.67027727	-0.16750585	2.12720546
H	-1.72538060	0.69363584	2.77151614
H	-2.51994786	-0.78209697	2.30986839
H	-0.84858361	-0.74861463	2.46577456
H	1.10138671	1.74801629	1.13653854
C	3.88107235	1.29532444	0.31235640
H	3.81142881	1.46308656	-0.75348953
H	3.72838681	2.25071457	0.82099215
H	4.85748351	0.89375193	0.55695047
H	-1.27421634	-1.14996970	-2.06629557

Time = 343.50 fs
E(CASSCF/6-31G(d)) = -479.4549683

C	-0.77125243	-0.05728711	-0.33274059
C	0.53233696	0.01599765	-0.20270985
C	1.36474876	-0.79196275	-1.16422581
C	1.37628492	0.76723125	0.71639118
C	2.62454627	0.37350338	0.47165232
C	2.59182134	-0.74160813	-0.51879461
N	-1.74555115	0.19944514	0.72796433
C	-2.89943113	-0.50920799	0.27011772
H	3.49164234	-1.19464942	-0.86923656
C	-1.54194090	-0.18676140	-1.64688103
C	-3.00403968	-0.14502330	-1.21059470
H	-3.43670850	0.84698966	-1.21300883
H	-3.57649845	-0.76475685	-1.89817784
H	-2.68023795	-1.58512925	0.34166138
H	-3.81101759	-0.27886185	0.81159467
H	-1.23178854	0.55860797	-2.38178533
H	1.12083291	-1.63292037	-1.84277216
C	-1.66684470	-0.17275272	2.11622817
H	-1.73407489	0.67583119	2.77599127
H	-2.50795334	-0.80491433	2.29563556
H	-0.84246948	-0.75461085	2.44760528
H	1.09441407	1.74089459	1.15222782
C	3.87865696	1.29547960	0.30234802
H	3.81364225	1.44536071	-0.76911759
H	3.71570881	2.26339477	0.78141303
H	4.85865986	0.90343507	0.55609305
H	-1.28315914	-1.16247120	-2.06285601

Time = 344.00 fs
E(CASSCF/6-31G(d)) = -479.4521881

C	-0.76938351	-0.05638634	-0.33453996
C	0.53000563	0.01239660	-0.19446492

C	1.36584960	-0.78973715	-1.15727804
C	1.37904271	0.76581147	0.72510385
C	2.62886489	0.37540088	0.47534456
C	2.58954658	-0.74166662	-0.51550305
N	-1.74927830	0.20521659	0.72836002
C	-2.89876792	-0.50543946	0.26855147
H	3.48427431	-1.19522727	-0.88501871
C	-1.54202556	-0.19085706	-1.64656592
C	-3.00448160	-0.14288683	-1.21035184
H	-3.43602211	0.85191987	-1.20908483
H	-3.58106566	-0.75229990	-1.90203840
H	-2.68432447	-1.59236017	0.34744585
H	-3.80743213	-0.27589686	0.80437877
H	-1.22969386	0.55344337	-2.38931292
H	1.11931648	-1.61685575	-1.84651420
C	-1.66330432	-0.17793514	2.10541063
H	-1.74277983	0.65881038	2.78060322
H	-2.49671970	-0.82795330	2.28296678
H	-0.83360213	-0.76128572	2.42869735
H	1.08751863	1.73121596	1.16744278
C	3.87592293	1.29558868	0.29242979
H	3.81737974	1.42854359	-0.78478840
H	3.70358224	2.27540224	0.74190415
H	4.85921738	0.91264679	0.55556461
H	-1.29293986	-1.17454674	-2.05981755

Time = 344.50 fs
E(CASSCF/6-31G(d)) = -479.4499946

C	-0.76826434	-0.05523819	-0.33597731
C	0.52787996	0.00865849	-0.18648685
C	1.36730266	-0.78755978	-1.14988693
C	1.38157280	0.76449870	0.73388376
C	2.63376455	0.37727691	0.47873558
C	2.58722423	-0.74158318	-0.51242545
N	-1.75294600	0.21099163	0.72846687
C	-2.89832059	-0.50195483	0.26653997
H	3.47741884	-1.19667757	-0.90051951
C	-1.54194238	-0.19509191	-1.64619142
C	-3.00492896	-0.14066522	-1.21016457
H	-3.43467802	0.85730044	-1.20632812
H	-3.58645735	-0.74030947	-1.90428066
H	-2.68970214	-1.59871020	0.35355934
H	-3.80370339	-0.27270700	0.79750937
H	-1.22809551	0.54777317	-2.39573403
H	1.11703501	-1.59816313	-1.84885151
C	-1.65967302	-0.18310198	2.09482098

H	-1.75142792	0.64253025	2.78528396
H	-2.48601289	-0.85099938	2.27182071
H	-0.82208336	-0.76839330	2.40899984
H	1.08078909	1.71915752	1.18220709
C	3.87289842	1.29566532	0.28257287
H	3.82261215	1.41249911	-0.80029166
H	3.69207519	2.28673436	0.70264301
H	4.85901616	0.92150390	0.55538152
H	-1.30352881	-1.18589846	-2.05706173

Time = 345.00 fs
E(CASSCF/6-31G(d)) = -479.4483462

C	-0.76793377	-0.05384102	-0.33703992
C	0.52601228	0.00479600	-0.17876859
C	1.36906634	-0.78540778	-1.14205365
C	1.38387558	0.76325067	0.74270900
C	2.63919108	0.37912347	0.48182216
C	2.58490839	-0.74137219	-0.50957017
N	-1.75653846	0.21676818	0.72824166
C	-2.89808127	-0.49880757	0.26408404
H	3.47093488	-1.19881504	-0.91550366
C	-1.54169078	-0.19946155	-1.64578824
C	-3.00537760	-0.13833284	-1.21003721
H	-3.43255408	0.86301381	-1.20472766
H	-3.59267922	-0.72892561	-1.90493517
H	-2.69633178	-1.60356252	0.35984064
H	-3.80021307	-0.26918906	0.79124522
H	-1.22705033	0.54135990	-2.40086415
H	1.11397788	-1.57718886	-1.84996461
C	-1.65595897	-0.18830773	2.08453230
H	-1.75992767	0.62688477	2.78991913
H	-2.47553815	-0.87379353	2.26212056
H	-0.80818146	-0.77559068	2.38843934
H	1.07426615	1.70505344	1.19661977
C	3.86961599	1.29572525	0.27274779
H	3.82929252	1.39708985	-0.81539933
H	3.68124515	2.29737107	0.66379879
H	4.85792929	0.93012297	0.55555496
H	-1.31486343	-1.19628263	-2.05448369

Time = 345.50 fs
E(CASSCF/6-31G(d)) = -479.4471593

C	-0.76841205	-0.05219491	-0.33771827
C	0.52444235	0.00082341	-0.17130127
C	1.37109373	-0.78325170	-1.13377578

C	1.38596287	0.76201192	0.75154983
C	2.64507959	0.38093424	0.48460392
C	2.58266306	-0.74105038	-0.50694455
N	-1.76004447	0.22254178	0.72764694
C	-2.89804711	-0.49603530	0.26119088
H	3.46464168	-1.20143053	-0.92972083
C	-1.54127792	-0.20396200	-1.64537983
C	-3.00582332	-0.13586581	-1.20997462
H	-3.42954310	0.86894177	-1.20425841
H	-3.59972976	-0.71828134	-1.90405903
H	-2.70413431	-1.60643636	0.36612254
H	-3.79731219	-0.26524814	0.78581046
H	-1.22658154	0.53404446	-2.40459285
H	1.11011760	-1.55437914	-1.85013046
C	-1.65216146	-0.19360883	2.07461874
H	-1.76816516	0.61172314	2.79435739
H	-2.46497679	-0.89605705	2.25376090
H	-0.79229658	-0.78246833	2.36693862
H	1.06794170	1.68937556	1.21085229
C	3.86611178	1.29578637	0.26292619
H	3.83735485	1.38218224	-0.82988714
H	3.67113817	2.30727577	0.62553125
H	4.85585496	0.93861459	0.55609404
H	-1.32684793	-1.20552572	-2.05199773

Time = 346.00 fs
E(CASSCF/6-31G(d)) = -479.4463439

C	-0.76970037	-0.05030159	-0.33800603
C	0.52319607	-0.00324347	-0.16407421
C	1.37333283	-0.78105784	-1.12504926
C	1.38785687	0.76071668	0.76036991
C	2.65135596	0.38270408	0.48708318
C	2.58055888	-0.74063521	-0.50455347
N	-1.76345717	0.22830564	0.72665212
C	-2.89822280	-0.49365840	0.25787530
H	3.45835242	-1.20431005	-0.94292185
C	-1.54071861	-0.20858961	-1.64498138
C	-3.00626209	-0.13324324	-1.20998256
H	-3.42555799	0.87497572	-1.20488194
H	-3.60759632	-0.70849609	-1.90172990
H	-2.71299388	-1.60700006	0.37223742
H	-3.79528424	-0.26080657	0.78137320
H	-1.22667588	0.52575203	-2.40689107
H	1.10541268	-1.53025339	-1.84970569
C	-1.64827177	-0.19905982	2.06515088
H	-1.77600794	0.59687014	2.79842451

H	-2.45401962	-0.91751736	2.24661373
H	-0.77491670	-0.78858639	2.34443594
H	1.06176201	1.67270601	1.22513911
C	3.86242448	1.29586863	0.25308197
H	3.84671428	1.36764959	-0.84355254
H	3.66178752	2.31639953	0.58799023
H	4.85272685	0.94707957	0.55700922
H	-1.33935562	-1.21353301	-2.04953912

Time = 346.50 fs
E(CASSCF/6-31G(d)) = -479.4458291

C	-0.77178088	-0.04816454	-0.33789918
C	0.52228467	-0.00738721	-0.15707545
C	1.37572674	-0.77879189	-1.11587065
C	1.38958807	0.75929322	0.76912797
C	2.65793863	0.38442841	0.48926495
C	2.57867004	-0.74014339	-0.50239840
N	-1.76677342	0.23405031	0.72523401
C	-2.89862256	-0.49167900	0.25416072
H	3.45190168	-1.20724933	-0.95487442
C	-1.54003497	-0.21334069	-1.64459936
C	-3.00669015	-0.13044855	-1.21006819
H	-3.42053677	0.88102571	-1.20654791
H	-3.61625194	-0.69967015	-1.89804040
H	-2.72276210	-1.60507776	0.37802291
H	-3.79431597	-0.25581154	0.77802855
H	-1.22728269	0.51649496	-2.40781582
H	1.09981679	-1.50536328	-1.84909733
C	-1.64427473	-0.20470978	2.05619149
H	-1.78331034	0.58214864	2.80194041
H	-2.44239482	-0.93793141	2.24053548
H	-0.75657039	-0.79351127	2.32090243
H	1.05563755	1.65569298	1.23976141
C	3.85859474	1.29599366	0.24319233
H	3.85726848	1.35337505	-0.85623041
H	3.65321263	2.32468673	0.55131576
H	4.84852065	0.95560552	0.55831483
H	-1.35223244	-1.22029528	-2.04706691

Time = 347.00 fs
E(CASSCF/6-31G(d)) = -479.4455845

C	-0.77461774	-0.04578896	-0.33739608
C	0.52170461	-0.01158874	-0.15029180
C	1.37821369	-0.77642309	-1.10624080
C	1.39119266	0.75766928	0.77778054

C	2.66474076	0.38610261	0.49115677
C	2.57707120	-0.73958963	-0.50047637
N	-1.76999339	0.23976357	0.72337750
C	-2.89927089	-0.49008064	0.25007944
H	3.44517088	-1.21006872	-0.96537858
C	-1.53925552	-0.21821138	-1.64423137
C	-3.00710440	-0.12747042	-1.21024036
H	-3.41444525	0.88702609	-1.20919527
H	-3.62565088	-0.69187858	-1.89309160
H	-2.73326275	-1.60065172	0.38332731
H	-3.79447795	-0.25024102	0.77578773
H	-1.22831534	0.50636963	-2.40750840
H	1.09328962	-1.48024692	-1.84872215
C	-1.64015041	-0.21059937	2.04779175
H	-1.78991981	0.56739944	2.80473619
H	-2.42988861	-0.95710468	2.23537229
H	-0.73778493	-0.79684723	2.29635401
H	1.04945909	1.63899506	1.25502090
C	3.85466430	1.29618418	0.23323841
H	3.86890007	1.33925370	-0.86780342
H	3.64541795	2.33208312	0.51563891
H	4.84325792	0.96426515	0.56003085
H	-1.36530224	-1.22588751	-2.04456312

Time = 347.50 fs
E(CASSCF/6-31G(d)) = -479.4456146

C	-0.77815850	-0.04318220	-0.33649731
C	0.52143853	-0.01582730	-0.14370870
C	1.38072645	-0.77392825	-1.09616911
C	1.39270904	0.75577844	0.78628533
C	2.67167234	0.38772153	0.49276834
C	2.57583519	-0.73898561	-0.49877895
N	-1.77312028	0.24543071	0.72107612
C	-2.90020207	-0.48882816	0.24567208
H	3.43810435	-1.21262333	-0.97428020
C	-1.53841420	-0.22319743	-1.64386674
C	-3.00750279	-0.12430359	-1.21050995
H	-3.40727891	0.89294025	-1.21275402
H	-3.63572628	-0.68516665	-1.88698825
H	-2.74429778	-1.59386455	0.38801560
H	-3.79572228	-0.24410541	0.77457733
H	-1.22965444	0.49554993	-2.40618994
H	1.08581499	-1.45537869	-1.84895551
C	-1.63587575	-0.21675874	2.03998841
H	-1.79568434	0.55249691	2.80667051
H	-2.41635739	-0.97490682	2.23096593

H	-0.71905094	-0.79825979	2.27085750
H	1.04312152	1.62321191	1.27120484
C	3.85067548	1.29646325	0.22320584
H	3.88148002	1.32519158	-0.87820858
H	3.63839134	2.33854411	0.48107990
H	4.83700627	0.97311703	0.56218483
H	-1.37837272	-1.23046518	-2.04203100

Time = 348.00 fs
E(CASSCF/6-31G(d)) = -479.4459300

C	-0.78233611	-0.04035347	-0.33520621
C	0.52145645	-0.02008022	-0.13730996
C	1.38319244	-0.77129594	-1.08567789
C	1.39417393	0.75356657	0.79460479
C	2.67864251	0.38927915	0.49411139
C	2.57503049	-0.73833950	-0.49729220
N	-1.77616011	0.25103428	0.71833247
C	-2.90145812	-0.48786799	0.24098631
H	3.43071999	-1.21480989	-0.98148019
C	-1.53754897	-0.22829411	-1.64348741
C	-3.00788469	-0.12094927	-1.21088988
H	-3.39906353	0.89876187	-1.21714724
H	-3.64638797	-0.67954672	-1.87983535
H	-2.75565259	-1.58501891	0.39197513
H	-3.79789515	-0.23744696	0.77424722
H	-1.23115242	0.48427646	-2.40415050
H	1.07741915	-1.43112378	-1.85007613
C	-1.63142609	-0.22320612	2.03280180
H	-1.80045869	0.53736176	2.80764310
H	-2.40173525	-0.99128164	2.22715915
H	-0.70079580	-0.79749322	2.24453251
H	1.03655264	1.60881434	1.28854499
C	3.84667069	1.29685347	0.21308499
H	3.89487127	1.31110756	-0.88743999
H	3.63210386	2.34404262	0.44774854
H	4.82987581	0.98220546	0.56481117
H	-1.39124216	-1.23425533	-2.03949315

Time = 348.50 fs
E(CASSCF/6-31G(d)) = -479.4465171

C	-0.78707093	-0.03731443	-0.33352906
C	0.52171695	-0.02432279	-0.13107747
C	1.38553377	-0.76852886	-1.07480622
C	1.39561919	0.75099716	0.80271028
C	2.68556147	0.39076836	0.49519906

C	2.57471960	-0.73765531	-0.49599619
N	-1.77912177	0.25655456	0.71515837
C	-2.90308573	-0.48712933	0.23607477
H	3.42311433	-1.21657199	-0.98694275
C	-1.53670023	-0.23349589	-1.64306971
C	-3.00825113	-0.11741515	-1.21139504
H	-3.38985325	0.90451323	-1.22229285
H	-3.65752161	-0.67499592	-1.87173548
H	-2.76710320	-1.57457134	0.39512050
H	-3.80076179	-0.23033493	0.77458636
H	-1.23264278	0.47283713	-2.40173183
H	1.06818810	-1.40771195	-1.85222038
C	-1.62677648	-0.22994697	2.02623483
H	-1.80411164	0.52196775	2.80760525
H	-2.38603535	-1.00625486	2.22379980
H	-0.68336368	-0.79437956	2.21754814
H	1.02974015	1.59608663	1.30717808
C	3.84269202	1.29737599	0.20287119
H	3.90893399	1.29693086	-0.89554691
H	3.62650873	2.34857702	0.41574036
H	4.82201281	0.99156439	0.56795142
H	-1.40370701	-1.23754037	-2.03698502

Time = 349.00 fs
E(CASSCF/6-31G(d)) = -479.4473033

C	-0.79227357	-0.03407894	-0.33147582
C	0.52216939	-0.02852843	-0.12499103
C	1.38766855	-0.76564445	-1.06361142
C	1.39706887	0.74805461	0.81058458
C	2.69234273	0.39218103	0.49604567
C	2.57495669	-0.73693291	-0.49486575
N	-1.78201692	0.26196951	0.71157488
C	-2.90513250	-0.48652588	0.23099267
H	3.41546235	-1.21790215	-0.99069712
C	-1.53590898	-0.23879617	-1.64258660
C	-3.00860503	-0.11371514	-1.21204196
H	-3.37972870	0.91024188	-1.22810579
H	-3.66899001	-0.67145648	-1.86278922
H	-2.77842296	-1.56311949	0.39739759
H	-3.80403919	-0.22285945	0.77534384
H	-1.23394984	0.46154475	-2.39930326
H	1.05827776	-1.38522885	-1.85535513
C	-1.62190288	-0.23697375	2.02027294
H	-1.80653093	0.50634598	2.80656614
H	-2.36934779	-1.01993557	2.22074517
H	-0.66700298	-0.78884372	2.19011758

H	1.02275298	1.58509202	1.32711620
C	3.83878093	1.29804969	0.19256465
H	3.92352927	1.28260273	-0.90262836
H	3.62154235	2.35217618	0.38513629
H	4.81358970	1.00121875	0.57165084
H	-1.41556884	-1.24063873	-2.03454973

Time = 349.50 fs
E(CASSCF/6-31G(d)) = -479.4481588

C	-0.79784755	-0.03066320	-0.32906067
C	0.52275609	-0.03266866	-0.11902796
C	1.38951354	-0.76267349	-1.05216809
C	1.39853797	0.74474426	0.81822314
C	2.69890449	0.39350816	0.49666638
C	2.57578544	-0.73616803	-0.49387145
N	-1.78485983	0.26725528	0.70761197
C	-2.90764313	-0.48595907	0.22579536
H	3.40801138	-1.21884067	-0.99283769
C	-1.53521485	-0.24418692	-1.64201074
C	-3.00895122	-0.10986866	-1.21284834
H	-3.36879359	0.91601515	-1.23450031
H	-3.68063688	-0.66883817	-1.85309736
H	-2.78939120	-1.55137601	0.39878475
H	-3.80743140	-0.21512377	0.77625203
H	-1.23490140	0.45070750	-2.39723158
H	1.04791380	-1.36363381	-1.85927661
C	-1.61678329	-0.24426615	2.01488511
H	-1.80762797	0.49058381	2.80459612
H	-2.35183241	-1.03251519	2.21786582
H	-0.65185703	-0.78090344	2.16249006
H	1.01574813	1.57567362	1.34823487
C	3.83497816	1.29889033	0.18217052
H	3.93852356	1.26807516	-0.90882466
H	3.61712499	2.35490329	0.35599901
H	4.80479349	1.01118868	0.57595520
H	-1.42664065	-1.24388065	-2.03222980

Time = 350.00 fs
E(CASSCF/6-31G(d)) = -479.4489273

C	-0.80369226	-0.02708526	-0.32630254
C	0.52341497	-0.03671311	-0.11316311
C	1.39098834	-0.75965701	-1.04056431
C	1.40003302	0.74108944	0.82563318
C	2.70517051	0.39474034	0.49707679
C	2.57723651	-0.73535256	-0.49298122

N	-1.78766706	0.27238603	0.70330781
C	-2.91065606	-0.48532283	0.22053621
H	3.40107062	-1.21947107	-0.99352046
C	-1.53465419	-0.24965818	-1.64131776
C	-3.00929632	-0.10589957	-1.21383247
H	-3.35716986	0.92191163	-1.24139199
H	-3.69229153	-0.66702185	-1.84276370
H	-2.79980231	-1.54012324	0.39928985
H	-3.81066042	-0.20723654	0.77704794
H	-1.23534274	0.44059609	-2.39584593
H	1.03737935	-1.34279669	-1.86363676
C	-1.61139901	-0.25179174	2.01002563
H	-1.80734143	0.47482048	2.80182478
H	-2.33370777	-1.04425988	2.21504777
H	-0.63796014	-0.77066530	2.13494112
H	1.00896237	1.56748886	1.37028168
C	3.83132367	1.29990977	0.17169875
H	3.95379181	1.25331105	-0.91430509
H	3.61316266	2.35685625	0.32837038
H	4.79581264	1.02149314	0.58090539
H	-1.43675252	-1.24758034	-2.03005838

Time = 350.50 fs
E(CASSCF/6-31G(d)) = -479.4494811

C	-0.80970488	-0.02336523	-0.32322529
C	0.52408087	-0.04063003	-0.10736897
C	1.39201935	-0.75664212	-1.02889721
C	1.40155311	0.73712668	0.83283194
C	2.71107204	0.39586802	0.49729223
C	2.57932670	-0.73447522	-0.49216132
N	-1.79045733	0.27733478	0.69870819
C	-2.91420048	-0.48451003	0.21526536
H	3.39499374	-1.21991307	-0.99295307
C	-1.53425814	-0.25519780	-1.64048985
C	-3.00964843	-0.10183479	-1.21501250
H	-3.34499306	0.92801205	-1.24869953
H	-3.70377609	-0.66586533	-1.83189967
H	-2.80947552	-1.53014937	0.39894237
H	-3.81349201	-0.19930497	0.77749221
H	-1.23515056	0.43141262	-2.39540388
H	1.02699438	-1.32254134	-1.86799027
C	-1.60573601	-0.25950719	2.00563649
H	-1.80563921	0.45923835	2.79843500
H	-2.31523447	-1.05549714	2.21219419
H	-0.62523809	-0.75831807	2.10776196
H	1.00269027	1.56006750	1.39290359

C	3.82785665	1.30111549	0.16116376
H	3.96921988	1.23828499	-0.91925325
H	3.60954964	2.35816555	0.30226895
H	4.78682278	1.03215291	0.58653154
H	-1.44575601	-1.25200884	-2.02805072

Time = 351.00 fs
E(CASSCF/6-31G(d)) = -479.4497839

C	-0.81578347	-0.01952479	-0.31985809
C	0.52468846	-0.04438645	-0.10161585
C	1.39254543	-0.75367832	-1.01726596
C	1.40309261	0.73289972	0.83984304
C	2.71654822	0.39688229	0.49732782
C	2.58205711	-0.73352243	-0.49137926
N	-1.79325103	0.28207350	0.69386538
C	-2.91829426	-0.48342037	0.21002898
H	3.39015621	-1.22031053	-0.99138159
C	-1.53405120	-0.26079121	-1.63951858
C	-3.01001680	-0.09770274	-1.21640581
H	-3.33240688	0.93438906	-1.25634644
H	-3.71491358	-0.66521014	-1.82062862
H	-2.81826448	-1.52216620	0.39778019
H	-3.81575402	-0.19142867	0.77738376
H	-1.23424434	0.42326438	-2.39606250
H	1.01708821	-1.30269099	-1.87185007
C	-1.59978620	-0.26736017	2.00165074
H	-1.80251936	0.44405094	2.79465250
H	-2.29669440	-1.06659567	2.20922494
H	-0.61351321	-0.74412211	2.08124774
H	0.99725461	1.55288054	1.41568534
C	3.82461540	1.30251028	0.15058406
H	3.98470559	1.22298378	-0.92385109
H	3.60617140	2.35898921	0.27768763
H	4.77797472	1.04319355	0.59284658
H	-1.45352683	-1.25736875	-2.02619628

Time = 351.50 fs
E(CASSCF/6-31G(d)) = -479.4499156

C	-0.82182880	-0.01558687	-0.31623535
C	0.52517394	-0.04794878	-0.09587235
C	1.39252226	-0.75081415	-1.00576634
C	1.40464357	0.72845398	0.84669310
C	2.72154692	0.39777533	0.49719799
C	2.58541309	-0.73247971	-0.49060571
N	-1.79606975	0.28657365	0.68883708

C	-2.92294252	-0.48196882	0.20486937
H	3.38692622	-1.22081522	-0.98907196
C	-1.53405033	-0.26642177	-1.63840677
C	-3.01041126	-0.09353161	-1.21802837
H	-3.31955786	0.94109655	-1.26426214
H	-3.72553584	-0.66488888	-1.80908968
H	-2.82606546	-1.51672108	0.39583289
H	-3.81734818	-0.18369496	0.77657050
H	-1.23259368	0.41614873	-2.39786008
H	1.00797286	-1.28310335	-1.87474257
C	-1.59354875	-0.27529182	1.99799621
H	-1.79800934	0.42948733	2.79073090
H	-2.27836572	-1.07793816	2.20607483
H	-0.60251690	-0.72839564	2.05568620
H	0.99297533	1.54540472	1.43819148
C	3.82163728	1.30409216	0.13998176
H	4.00015851	1.20740858	-0.92826240
H	3.60290855	2.35950470	0.25459231
H	4.76938387	1.05464668	0.59983981
H	-1.45996637	-1.26377496	-2.02445329

Time = 352.00 fs
E(CASSCF/6-31G(d)) = -479.4500416

C	-0.82774627	-0.01157536	-0.31239642
C	0.52547689	-0.05128332	-0.09010606
C	1.39192581	-0.74809481	-0.99448551
C	1.40619786	0.72383209	0.85340852
C	2.72602548	0.39854093	0.49691624
C	2.58936514	-0.73133317	-0.48981687
N	-1.79893571	0.29080676	0.68368519
C	-2.92813709	-0.48009300	0.19982516
H	3.38563029	-1.22156765	-0.98628697
C	-1.53426449	-0.27207144	-1.63716884
C	-3.01084173	-0.08934778	-1.21989422
H	-3.30659106	0.94816061	-1.27238281
H	-3.73549188	-0.66473315	-1.79744086
H	-2.83282326	-1.51411941	0.39310578
H	-3.81825646	-0.17617521	0.77495637
H	-1.23021971	0.40995222	-2.40071345
H	0.99991973	-1.26369452	-1.87625427
C	-1.58703092	-0.28323988	1.99459977
H	-1.79216384	0.41577331	2.78693308
H	-2.26049483	-1.08988806	2.20269005
H	-0.59191076	-0.71149722	2.03134676
H	0.99014245	1.53717469	1.46000473
C	3.81895834	1.30585467	0.12938196

H	4.01549802	1.19157700	-0.93261771
H	3.59964106	2.35989819	0.23292157
H	4.76112392	1.06655015	0.60747151
H	-1.46500259	-1.27124494	-2.02274691

Time = 352.50 fs
E(CASSCF/6-31G(d)) = -479.4503454

C	-0.83344747	-0.00751491	-0.30838551
C	0.52554169	-0.05435714	-0.08428404
C	1.39075399	-0.74556056	-0.98349902
C	1.40774883	0.71907075	0.86001296
C	2.72995128	0.39917513	0.49649495
C	2.59387130	-0.73007164	-0.48899619
N	-1.80187158	0.29474517	0.67847492
C	-2.93385617	-0.47775813	0.19493147
H	3.38651881	-1.22267589	-0.98326250
C	-1.53469498	-0.27772179	-1.63582962
C	-3.01131751	-0.08517440	-1.22201508
H	-3.29364556	0.95557222	-1.28065174
H	-3.74465486	-0.66458081	-1.78586051
H	-2.83853381	-1.51437708	0.38956881
H	-3.81854350	-0.16892170	0.77250572
H	-1.22719149	0.40446339	-2.40442960
H	0.99314184	-1.24445100	-1.87606762
C	-1.58024826	-0.29114214	1.99139120
H	-1.78506129	0.40311102	2.78350995
H	-2.24326874	-1.10275473	2.19902380
H	-0.58131676	-0.69380568	2.00847109
H	0.98899463	1.52781963	1.48075834
C	3.81661286	1.30778762	0.11881219
H	4.03065193	1.17552548	-0.93700289
H	3.59625305	2.36035239	0.21258620
H	4.75322435	1.07894681	0.61566999
H	-1.46858975	-1.27969830	-2.02097063

Time = 353.00 fs
E(CASSCF/6-31G(d)) = -479.4509664

C	-0.83885120	-0.00343071	-0.30425115
C	0.52531855	-0.05713876	-0.07837367
C	1.38902724	-0.74324596	-0.97286836
C	1.40929245	0.71419889	0.86652546
C	2.73330216	0.39967657	0.49594522
C	2.59887993	-0.72868836	-0.48813583
N	-1.80490003	0.29836286	0.67327383
C	-2.94006487	-0.47495798	0.19021954

H	3.38973485	-1.22419615	-0.98018497
C	-1.53533604	-0.28335515	-1.63442182
C	-3.01184672	-0.08103033	-1.22439992
H	-3.28085206	0.96328393	-1.28901943
H	-3.75292830	-0.66428300	-1.77454765
H	-2.84324339	-1.51721587	0.38515503
H	-3.81835516	-0.16196572	0.76924477
H	-1.22361699	0.39939743	-2.40873130
H	0.98778149	-1.22543350	-1.87398602
C	-1.57322378	-0.29893990	1.98830697
H	-1.77679951	0.39165850	2.78067862
H	-2.22679208	-1.11676009	2.19503192
H	-0.57035642	-0.67569857	1.98726659
H	0.98970515	1.51708581	1.50015970
C	3.81463255	1.30987792	0.10830171
H	4.04555368	1.15931124	-0.94145184
H	3.59263750	2.36103293	0.19347065
H	4.74567286	1.09188192	0.62433084
H	-1.47070802	-1.28896739	-2.01899172

Time = 353.50 fs
E(CASSCF/6-31G(d)) = -479.4519515

C	-0.84388435	0.00065174	-0.30004555
C	0.52476444	-0.05959917	-0.07234358
C	1.38678751	-0.74117929	-0.96263971
C	1.41082812	0.70923671	0.87295912
C	2.73606641	0.40004684	0.49527660
C	2.60433272	-0.72718238	-0.48723728
N	-1.80804337	0.30163596	0.66815086
C	-2.94671619	-0.47171209	0.18571571
H	3.39529260	-1.22611834	-0.97717510
C	-1.53617598	-0.28895557	-1.63298262
C	-3.01243572	-0.07692966	-1.22705470
H	-3.26833138	0.97121006	-1.29744325
H	-3.76025043	-0.66370964	-1.76371997
H	-2.84704443	-1.52210257	0.37976914
H	-3.81791272	-0.15531668	0.76525992
H	-1.21963090	0.39442811	-2.41329067
H	0.98390337	-1.20677461	-1.86994951
C	-1.56598600	-0.30658091	1.98529317
H	-1.76749100	0.38151133	2.77860204
H	-2.21107212	-1.13201206	2.19066891
H	-0.55869640	-0.65753129	1.96790308
H	0.99237400	1.50484781	1.51800718
C	3.81304551	1.31211075	0.09788120
H	4.06013990	1.14301333	-0.94594483

H	3.58870008	2.36207466	0.17543530
H	4.73842152	1.10539981	0.63331937
H	-1.47136287	-1.29881704	-2.01666010

Time = 354.00 fs
E(CASSCF/6-31G(d)) = -479.4532522

C	-0.84848313	0.00470725	-0.29582335
C	0.52384445	-0.06171232	-0.06616460
C	1.38409645	-0.73938239	-0.95284312
C	1.41235899	0.70419528	0.87932011
C	2.73824261	0.40029062	0.49449705
C	2.61016690	-0.72555919	-0.48631129
N	-1.81132262	0.30454283	0.66317504
C	-2.95375291	-0.46805987	0.18144015
H	3.40306928	-1.22835960	-0.97428067
C	-1.53719833	-0.29450945	-1.63155002
C	-3.01308864	-0.07288162	-1.22998215
H	-3.25619415	0.97923110	-1.30588726
H	-3.76659618	-0.66275393	-1.75361096
H	-2.85006803	-1.52832099	0.37330385
H	-3.81750318	-0.14896247	0.76069288
H	-1.21538085	0.38922025	-2.41776402
H	0.98149164	-1.18867306	-1.86404187
C	-1.55856588	-0.31402129	1.98230749
H	-1.75725880	0.37268983	2.77737373
H	-2.19601662	-1.14848994	2.18588614
H	-0.54609334	-0.63962033	1.95051309
H	0.99702420	1.49111178	1.53420074
C	3.81187523	1.31447069	0.08758247
H	4.07434823	1.12673325	-0.95041290
H	3.58436341	2.36357013	0.15832055
H	4.73139693	1.11954006	0.64247446
H	-1.47058433	-1.30896894	-2.01381850

Time = 354.50 fs
E(CASSCF/6-31G(d)) = -479.4547288

C	-0.85259391	0.00871132	-0.29164057
C	0.52253229	-0.06345596	-0.05981099
C	1.38103248	-0.73787082	-0.94349233
C	1.41389215	0.69907660	0.88560719
C	2.73983909	0.40041565	0.49361290
C	2.61631709	-0.72383052	-0.48537683
N	-1.81475683	0.30706449	0.65841439
C	-2.96110974	-0.46405343	0.17740564
H	3.41281346	-1.23076785	-0.97147954

C	-1.53838292	-0.30000580	-1.63015929
C	-3.01380703	-0.06889088	-1.23318169
H	-3.24454120	0.98720271	-1.31432231
H	-3.77197784	-0.66133551	-1.74446542
H	-2.85247515	-1.53505945	0.36565928
H	-3.81746465	-0.14287190	0.75573167
H	-1.21101461	0.38345891	-2.42182400
H	0.98045079	-1.17138455	-1.85649202
C	-1.55099342	-0.32122618	1.97931990
H	-1.74623309	0.36513301	2.77701008
H	-2.18144398	-1.16604597	2.18063303
H	-0.53243016	-0.62223301	1.93519473
H	1.00360102	1.47601456	1.54874915
C	3.81113940	1.31694283	0.07743829
H	4.08811714	1.11059304	-0.95474757
H	3.57957002	2.36556127	0.14195065
H	4.72451217	1.13433400	0.65161582
H	-1.46842580	-1.31912758	-2.01031296

Time = 355.00 fs
E(CASSCF/6-31G(d)) = -479.4562024

C	-0.85617406	0.01264055	-0.28755314
C	0.52081107	-0.06481241	-0.05326152
C	1.37768684	-0.73665393	-0.93458557
C	1.41543847	0.69387417	0.89181167
C	2.74087350	0.40043251	0.49262869
C	2.62271648	-0.72201340	-0.48445894
N	-1.81836230	0.30918466	0.65393490
C	-2.96871628	-0.45975030	0.17361702
H	3.42416769	-1.23313497	-0.96869257
C	-1.53970706	-0.30543609	-1.62884016
C	-3.01458970	-0.06495838	-1.23664948
H	-3.23346377	0.99496634	-1.32272604
H	-3.77644262	-0.65940112	-1.73653300
H	-2.85444683	-1.54149447	0.35676073
H	-3.81816436	-0.13699934	0.75059650
H	-1.20666902	0.37687328	-2.42518659
H	0.98061109	-1.15521048	-1.84766634
C	-1.54329439	-0.32816922	1.97631207
H	-1.73454954	0.35870057	2.77745050
H	-2.16710337	-1.18442057	2.17485997
H	-0.51773793	-0.60558404	1.92201568
H	1.01197484	1.45981713	1.56177269
C	3.81084888	1.31951385	0.06748234
H	4.10138731	1.09473160	-0.95881402
H	3.57428399	2.36803532	0.12613807

H	4.71767875	1.14980199	0.66055315
H	-1.46496274	-1.32900579	-2.00600229

Time = 355.50 fs
E(CASSCF/6-31G(d)) = -479.4574877

C	-0.85919243	0.01647293	-0.28361560
C	0.51867357	-0.06576904	-0.04650032
C	1.37415969	-0.73573540	-0.92610711
C	1.41701206	0.68857392	0.89791758
C	2.74137216	0.40035448	0.49154720
C	2.62929705	-0.72012875	-0.48358639
N	-1.82215188	0.31089000	0.64979954
C	-2.97650034	-0.45520723	0.17007157
H	3.43670103	-1.23521652	-0.96580184
C	-1.54114638	-0.31079384	-1.62761466
C	-3.01543283	-0.06108229	-1.24037846
H	-3.22304434	1.00236183	-1.33108305
H	-3.78006831	-0.65692390	-1.73006116
H	-2.85617473	-1.54686046	0.34657214
H	-3.81997140	-0.13129197	0.74552077
H	-1.20246126	0.36925437	-2.42763184
H	0.98173923	-1.14048259	-1.83805001
C	-1.53548815	-0.33483104	1.97327572
H	-1.72234849	0.35318320	2.77856663
H	-2.15270269	-1.20326953	2.16852347
H	-0.50219676	-0.58983995	1.91101728
H	1.02194769	1.44289258	1.57350018
C	3.81100681	1.32217266	0.05774907
H	4.11410349	1.07929971	-0.96246645
H	3.56849148	2.37092528	0.11068895
H	4.71081781	1.16595093	0.66909541
H	-1.46029112	-1.33834744	-2.00076683

Time = 356.00 fs
E(CASSCF/6-31G(d)) = -479.4584658

C	-0.86163049	0.02018849	-0.27987934
C	0.51612298	-0.06631889	-0.03951788
C	1.37055543	-0.73511408	-0.91802973
C	1.41862943	0.68315586	0.90390240
C	2.74136926	0.40019674	0.49036976
C	2.63598948	-0.71819933	-0.48278924
N	-1.82613389	0.31216992	0.64606697
C	-2.98439188	-0.45047546	0.16676017
H	3.44994621	-1.23675492	-0.96267262
C	-1.54267578	-0.31607412	-1.62649565

C	-3.01633024	-0.05725917	-1.24435874
H	-3.21335697	1.00924050	-1.33938530
H	-3.78295651	-0.65390058	-1.72528704
H	-2.85785215	-1.55049965	0.33510423
H	-3.82322357	-0.12569936	0.74072792
H	-1.19848155	0.36046622	-2.42901704
H	0.98355422	-1.12754493	-1.82821555
C	-1.52758675	-0.34119722	1.97021066
H	-1.70977532	0.34831946	2.78017806
H	-2.13793861	-1.22219741	2.16159095
H	-0.48611634	-0.57512754	1.90221623
H	1.03326362	1.42570765	1.58425939
C	3.81160809	1.32491084	0.04827359
H	4.12621790	1.06445382	-0.96556426
H	3.56219990	2.37411621	0.09540840
H	4.70387008	1.18277353	0.67706074
H	-1.45452501	-1.34694494	-1.99451426

Time = 356.50 fs
E(CASSCF/6-31G(d)) = -479.4590887

C	-0.86348240	0.02376949	-0.27639130
C	0.51317260	-0.06646095	-0.03231176
C	1.36697780	-0.73478541	-0.91031874
C	1.42030832	0.67759632	0.90973915
C	2.74090590	0.39997625	0.48909588
C	2.64272328	-0.71624796	-0.48209621
N	-1.83031157	0.31301675	0.64279078
C	-2.99232690	-0.44559727	0.16366931
H	3.46343694	-1.23750331	-0.95917432
C	-1.54427014	-0.32127306	-1.62548610
C	-3.01727384	-0.05348515	-1.24857791
H	-3.20446711	1.01547617	-1.34763226
H	-3.78522350	-0.65034680	-1.72242824
H	-2.85966624	-1.55189449	0.32241709
H	-3.82819245	-0.12018451	0.73640606
H	-1.19478873	0.35045192	-2.42928588
H	0.98575072	-1.11673001	-1.81877560
C	-1.51959555	-0.34725646	1.96712236
H	-1.69698134	0.34381647	2.78207261
H	-2.12252459	-1.24079309	2.15404538
H	-0.46989999	-0.56154406	1.89560431
H	1.04562532	1.40879383	1.59445620
C	3.81263897	1.32772271	0.03909148
H	4.13769349	1.05034848	-0.96798616
H	3.55543593	2.37745498	0.08010571
H	4.69680327	1.20024773	0.68428538

H	-1.44779461	-1.35465301	-1.98718389

Time = 357.00 fs
E(CASSCF/6-31G(d)) = -479.4594252

C	-0.86475574	0.02720080	-0.27319247
C	0.50984596	-0.06620030	-0.02488690
C	1.36352535	-0.73474310	-0.90293709
C	1.42206574	0.67187122	0.91539907
C	2.74002912	0.39971103	0.48772362
C	2.64942637	-0.71429582	-0.48153296
N	-1.83468218	0.31342557	0.64001820
C	-3.00025109	-0.44060373	0.16078319
H	3.47673992	-1.23724510	-0.95519853
C	-1.54590500	-0.32638726	-1.62457884
C	-3.01825439	-0.04975705	-1.25302166
H	-3.19643082	1.02097576	-1.35583145
H	-3.78699038	-0.64629120	-1.72167386
H	-2.86179136	-1.55068654	0.30861923
H	-3.83505112	-0.11473430	0.73268479
H	-1.19140697	0.33923500	-2.42847193
H	0.98802654	-1.10833400	-1.81031953
C	-1.51151488	-0.35299895	1.96401985
H	-1.68412481	0.33937223	2.78402859
H	-2.10621403	-1.25866300	2.14588821
H	-0.45399869	-0.54916562	1.89114573
H	1.05871671	1.39270755	1.60454083
C	3.81407726	1.33060504	0.03023845
H	4.14850796	1.03712817	-0.96964206
H	3.54824191	2.38076344	0.06459838
H	4.68961729	1.21833860	0.69063248
H	-1.44024261	-1.36139680	-1.97874769

Time = 357.50 fs
E(CASSCF/6-31G(d)) = -479.4596122

C	-0.86547098	0.03047010	-0.27031715
C	0.50617623	-0.06554821	-0.01725591
C	1.36028705	-0.73498140	-0.89585157
C	1.42391586	0.66596043	0.92085570
C	2.73879109	0.39941963	0.48624971
C	2.65602537	-0.71236107	-0.48112064
N	-1.83923688	0.31339469	0.63778983
C	-3.00812259	-0.43551356	0.15808580
H	3.48947993	-1.23581052	-0.95067182
C	-1.54755712	-0.33141351	-1.62375695
C	-3.01926226	-0.04607345	-1.25767440

H	-3.18929458	1.02568665	-1.36399837
H	-3.78837211	-0.64176921	-1.72317647
H	-2.86438401	-1.54668634	0.29386396
H	-3.84385152	-0.10936710	0.72961803
H	-1.18832411	0.32691716	-2.42669775
H	0.99011402	-1.10258867	-1.80334247
C	-1.50334247	-0.35841547	1.96091379
H	-1.67137059	0.33469721	2.78583610
H	-2.08881804	-1.27545913	2.13714181
H	-0.43886313	-0.53805485	1.88877497
H	1.07222993	1.37797961	1.61496136
C	3.81589256	1.33355668	0.02174984
H	4.15865604	1.02492088	-0.97048288
H	3.54067249	2.38385284	0.04871801
H	4.68234663	1.23699871	0.69599784
H	-1.43202187	-1.36717528	-1.96921147

Time = 358.00 fs
E(CASSCF/6-31G(d)) = -479.4598430

C	-0.86566192	0.03356824	-0.26779177
C	0.50220576	-0.06452227	-0.00943902
C	1.35733790	-0.73549684	-0.88903860
C	1.42586758	0.65985259	0.92608983
C	2.73724794	0.39912034	0.48466952
C	2.66244641	-0.71045768	-0.48087471
N	-1.84396023	0.31292517	0.63613852
C	-3.01591353	-0.43033264	0.15556233
H	3.50135793	-1.23308826	-0.94556584
C	-1.54920497	-0.33634850	-1.62299445
C	-3.02028825	-0.04243528	-1.26251992
H	-3.18309316	1.02960116	-1.37215622
H	-3.78946650	-0.63681554	-1.72704361
H	-2.86757802	-1.53987717	0.27834376
H	-3.85451309	-0.10413529	0.72717591
H	-1.18549209	0.31367172	-2.42416915
H	0.99180961	-1.09963790	-1.79817605
C	-1.49507598	-0.36349687	1.95781476
H	-1.65889040	0.32953322	2.78731558
H	-2.07021606	-1.29090123	2.12784907
H	-0.42489903	-0.52826270	1.88839293
H	1.08589487	1.36505789	1.62610666
C	3.81804715	1.33657802	0.01366006
H	4.16815276	1.01383116	-0.97050586
H	3.53278962	2.38653905	0.03231310
H	4.67506071	1.25617186	0.70031577
H	-1.42329173	-1.37205982	-1.95861173

Time = 358.50 fs
E(CASSCF/6-31G(d)) = -479.4602500

C	-0.86537399	0.03648896	-0.26563514
C	0.49798475	-0.06314594	-0.00146349
C	1.35473712	-0.73629001	-0.88248913
C	1.42792278	0.65354950	0.93109440
C	2.73545893	0.39883095	0.48297745
C	2.66861525	-0.70859493	-0.48080460
N	-1.84883083	0.31202167	0.63508972
C	-3.02361009	-0.42505436	0.15319978
H	3.51216334	-1.22903198	-0.93990136
C	-1.55082893	-0.34118849	-1.62225780
C	-3.02132441	-0.03884645	-1.26754208
H	-3.17784923	1.03275798	-1.38033529
H	-3.79034508	-0.63145873	-1.73333214
H	-2.87148162	-1.53041644	0.26228358
H	-3.86682896	-0.09912247	0.72524838
H	-1.18282966	0.29973287	-2.42116534
H	0.99299317	-1.09952132	-1.79493599
C	-1.48671551	-0.36823408	1.95473207
H	-1.64686098	0.32366790	2.78833252
H	-2.05036172	-1.30479263	2.11807342
H	-0.41243117	-0.51982990	1.88986630
H	1.09950414	1.35425372	1.63824838
C	3.82049670	1.33967037	0.00600182
H	4.17703385	1.00393549	-0.96975687
H	3.52465881	2.38865534	0.01525354
H	4.66786139	1.27579426	0.70356114
H	-1.41421551	-1.37618850	-1.94701360

Time = 359.00 fs
E(CASSCF/6-31G(d)) = -479.4608973

C	-0.86466445	0.03922894	-0.26385765
C	0.49357106	-0.06144788	0.00663695
C	1.35252728	-0.73736586	-0.87621028
C	1.43007491	0.64706901	0.93587792
C	2.73348535	0.39856841	0.48116737
C	2.67445718	-0.70677708	-0.48091399
N	-1.85382077	0.31069175	0.63466054
C	-3.03121142	-0.41966038	0.15098667
H	3.52178095	-1.22366240	-0.93375009
C	-1.55241134	-0.34592886	-1.62150775
C	-3.02236461	-0.03531391	-1.27272530
H	-3.17357110	1.03524014	-1.38857242

H	-3.79104606	-0.62571636	-1.74204298
H	-2.87617468	-1.51863135	0.24593288
H	-3.88048533	-0.09443458	0.72365917
H	-1.18022819	0.28537891	-2.41802194
H	0.99363457	-1.10217079	-1.79350035
C	-1.47826537	-0.37261824	1.95167302
H	-1.63546343	0.31694481	2.78880781
H	-2.02928349	-1.31703069	2.10789571
H	-0.40167601	-0.51278318	1.89302650
H	1.11292936	1.34570264	1.65149456
C	3.82319164	1.34283524	-0.00119440
H	4.18535650	0.99527706	-0.96832703
H	3.51634432	2.39006318	-0.00256914
H	4.66087874	1.29579988	0.70575070
H	-1.40495694	-1.37975586	-1.93450392

Time = 359.50 fs
E(CASSCF/6-31G(d)) = -479.4616826

C	-0.86360051	0.04178764	-0.26246182
C	0.48902873	-0.05946160	0.01482293
C	1.35073471	-0.73873340	-0.87022448
C	1.43230934	0.64044618	0.94046646
C	2.73138951	0.39834859	0.47923281
C	2.67989810	-0.70500336	-0.48120088
N	-1.85889661	0.30894621	0.63486016
C	-3.03872724	-0.41412233	0.14891235
H	3.53019099	-1.21706622	-0.92723268
C	-1.55393615	-0.35056386	-1.62070195
C	-3.02340496	-0.03184733	-1.27805512
H	-3.17025173	1.03716954	-1.39690957
H	-3.79157058	-0.61959284	-1.75311932
H	-2.88170789	-1.50501065	0.22955651
H	-3.89509262	-0.09018729	0.72218712
H	-1.17756055	0.27091142	-2.41510788
H	0.99378771	-1.10741810	-1.79352442
C	-1.46973581	-0.37664111	1.94864232
H	-1.62488074	0.30926960	2.78872412
H	-2.00708186	-1.32761176	2.09741219
H	-0.39272300	-0.50713282	1.89767219
H	1.12612332	1.33934860	1.66576599
C	3.82607858	1.34607381	-0.00790120
H	4.19319772	0.98786462	-0.96634745
H	3.50790559	2.39066024	-0.02123929
H	4.65426400	1.31612314	0.70694081
H	-1.39567850	-1.38299795	-1.92118606

Time = 360.00 fs
E(CASSCF/6-31G(d)) = -479.4623855

C	-0.86225724	0.04416671	-0.26144310
C	0.48442646	-0.05722494	0.02305028
C	1.34937131	-0.74040435	-0.86456594
C	1.43460499	0.63373191	0.94490279
C	2.72923369	0.39818616	0.47716750
C	2.68486528	-0.70326841	-0.48165823
N	-1.86402049	0.30679943	0.63569038
C	-3.04617472	-0.40840415	0.14696621
H	3.53746494	-1.20939075	-0.92051374
C	-1.55538818	-0.35508600	-1.61979814
C	-3.02444387	-0.02845841	-1.28351845
H	-3.16786754	1.03869831	-1.40539204
H	-3.79188293	-0.61307996	-1.76644714
H	-2.88810372	-1.49018723	0.21342442
H	-3.91022168	-0.08649167	0.72059115
H	-1.17469407	0.25662937	-2.41279642
H	0.99357147	-1.11501454	-1.79448770
C	-1.46114416	-0.38029557	1.94564170
H	-1.61529525	0.30061160	2.78812794
H	-1.98392267	-1.33663121	2.08673126
H	-0.38552545	-0.50286895	1.90357309
H	1.13910739	1.33495678	1.68080202
C	3.82910170	1.34938637	-0.01409504
H	4.20065186	0.98167249	-0.96397998
H	3.49939446	2.39038543	-0.04081658
H	4.64818045	1.33670200	0.70722319
H	-1.38654020	-1.38617337	-1.90717284

Time = 360.50 fs
E(CASSCF/6-31G(d)) = -479.4627529

C	-0.86071551	0.04636989	-0.26079035
C	0.47983599	-0.05477915	0.03127099
C	1.34843687	-0.74239142	-0.85927526
C	1.43693680	0.62698930	0.94924298
C	2.72707893	0.39809446	0.47496554
C	2.68928892	-0.70156279	-0.48227469
N	-1.86915097	0.30426903	0.63714582
C	-3.05357519	-0.40246545	0.14513683
H	3.54375394	-1.20083524	-0.91379410
C	-1.55675207	-0.35948576	-1.61875748
C	-3.02548186	-0.02515974	-1.28910384
H	-3.16637759	1.03999731	-1.41406620
H	-3.79191412	-0.60615943	-1.78185823

H	-2.89535939	-1.47490602	0.19780097
H	-3.92544005	-0.08344024	0.71863455
H	-1.17150578	0.24280085	-2.41143029
H	0.99314561	-1.12465702	-1.79576250
C	-1.45251558	-0.38357616	1.94267007
H	-1.60688565	0.29100155	2.78712718
H	-1.96002834	-1.34427708	2.07596948
H	-0.37990030	-0.49995850	1.91047633
H	1.15194932	1.33215195	1.69619628
C	3.83220472	1.35277188	-0.01975686
H	4.20782616	0.97664363	-0.96140472
H	3.49085299	2.38921994	-0.06133503
H	4.64279309	1.35748091	0.70671728
H	-1.37769941	-1.38954097	-1.89258011

Time = 361.00 fs
E(CASSCF/6-31G(d)) = -479.4625626

C	-0.85905885	0.04840223	-0.26048713
C	0.47532993	-0.05216829	0.03943416
C	1.34792173	-0.74470636	-0.85439378
C	1.43927906	0.62028873	0.95355133
C	2.72498396	0.39808566	0.47262188
C	2.69310385	-0.69987407	-0.48303552
N	-1.87424484	0.30137640	0.63921502
C	-3.06095072	-0.39626610	0.14341155
H	3.54927201	-1.19163756	-0.90729953
C	-1.55801105	-0.36375127	-1.61754826
C	-3.02652104	-0.02196338	-1.29480149
H	-3.16572309	1.04124230	-1.42297677
H	-3.79156907	-0.59880793	-1.79913583
H	-2.90345341	-1.45997662	0.18293368
H	-3.94034459	-0.08109528	0.71610599
H	-1.16789943	0.22963533	-2.41128719
H	0.99268462	-1.13601518	-1.79668709
C	-1.44388323	-0.38647982	1.93972363
H	-1.59982332	0.28052502	2.78588475
H	-1.93566525	-1.35081869	2.06524766
H	-0.37553799	-0.49834268	1.91811436
H	1.16473601	1.33047113	1.71145013
C	3.83533247	1.35622762	-0.02487277
H	4.21483516	0.97269445	-0.95880528
H	3.48231328	2.38718596	-0.08280310
H	4.63825630	1.37841221	0.70556078
H	-1.36931183	-1.39333655	-1.87752055

Time = 361.50 fs

E(CASSCF/6-31G(d)) = -479.4617152

C	-0.85736874	0.05026827	-0.26051365
C	0.47097802	-0.04943888	0.04748676
C	1.34780918	-0.74735839	-0.84995895
C	1.44160921	0.61370333	0.95789502
C	2.72300433	0.39817060	0.47013289
C	2.69625231	-0.69818815	-0.48392352
N	-1.87926014	0.29814811	0.64188248
C	-3.06832014	-0.38977202	0.14177656
H	3.55427049	-1.18205664	-0.90126585
C	-1.55914558	-0.36786818	-1.61614904
C	-3.02756443	-0.01887937	-1.30060314
H	-3.16582695	1.04260008	-1.43216404
H	-3.79073673	-0.59100381	-1.81802335
H	-2.91235608	-1.44620863	0.16904271
H	-3.95458834	-0.07947969	0.71283639
H	-1.16382201	0.21726135	-2.41255002
H	0.99235555	-1.14875646	-1.79663144
C	-1.43528812	-0.38900692	1.93679584
H	-1.59426807	0.26931212	2.78460815
H	-1.91112694	-1.35658917	2.05468741
H	-0.37202343	-0.49793551	1.92621583
H	1.17754854	1.32941927	1.72603361
C	3.83843245	1.35974917	-0.02943517
H	4.22179535	0.96972147	-0.95635244
H	3.47379786	2.38434233	-0.10520440
H	4.63469946	1.39945668	0.70389995
H	-1.36153321	-1.39774967	-1.86209892

Time = 362.00 fs
E(CASSCF/6-31G(d)) = -479.4602591

C	-0.85570800	0.05196367	-0.26085438
C	0.46683232	-0.04664257	0.05537242
C	1.34807681	-0.75035433	-0.84600295
C	1.44391812	0.60730770	0.96234131
C	2.72119218	0.39835808	0.46749727
C	2.69869098	-0.69649061	-0.48491668
N	-1.88416945	0.29462638	0.64513935
C	-3.07569161	-0.38296290	0.14021877
H	3.55900746	-1.17235117	-0.89592069
C	-1.56013136	-0.37181947	-1.61455233
C	-3.02861504	-0.01591415	-1.30650157
H	-3.16659309	1.04421447	-1.44166003
H	-3.78930192	-0.58273507	-1.83823653
H	-2.92204943	-1.43434422	0.15630724

H	-3.96790199	-0.07857083	0.70871057
H	-1.15928212	0.20571422	-2.41529480
H	0.99230556	-1.16255386	-1.79505324
C	-1.42678008	-0.39116365	1.93387307
H	-1.59036238	0.25752409	2.78353787
H	-1.88670815	-1.36196953	2.04441719
H	-0.36886810	-0.49862865	1.93452403
H	1.19044047	1.32851249	1.73944369
C	3.84145416	1.36333143	-0.03344641
H	4.22882259	0.96761033	-0.95418971
H	3.46532623	2.38078181	-0.12849757
H	4.63220613	1.42057531	0.70188542
H	-1.35452689	-1.40290092	-1.84641177

Time = 362.50 fs
E(CASSCF/6-31G(d)) = -479.4582243

C	-0.85419104	0.05352323	-0.26146442
C	0.46299477	-0.04381179	0.06305442
C	1.34870406	-0.75369489	-0.84253035
C	1.44617353	0.60114352	0.96694747
C	2.71958818	0.39866356	0.46471799
C	2.70036579	-0.69476971	-0.48601653
N	-1.88890360	0.29080387	0.64893477
C	-3.08309760	-0.37582112	0.13871244
H	3.56372634	-1.16275262	-0.89146554
C	-1.56094738	-0.37559628	-1.61275439
C	-3.02967701	-0.01306504	-1.31250059
H	-3.16791585	1.04619568	-1.45150467
H	-3.78714841	-0.57398535	-1.85949016
H	-2.93250617	-1.42488714	0.14488159
H	-3.98011543	-0.07829794	0.70366552
H	-1.15430586	0.19491042	-2.41943652
H	0.99262050	-1.17721140	-1.79150791
C	-1.41839063	-0.39295960	1.93096200
H	-1.58822587	0.24531296	2.78289989
H	-1.86269509	-1.36730969	2.03448889
H	-0.36555612	-0.50025529	1.94277461
H	1.20345825	1.32736333	1.75125186
C	3.84436778	1.36696877	-0.03691024
H	4.23601520	0.96624008	-0.95241173
H	3.45687634	2.37660019	-0.15265515
H	4.63083923	1.44180345	0.69961843
H	-1.34841889	-1.40886026	-1.83050529

Time = 363.00 fs
E(CASSCF/6-31G(d)) = -479.4562746

C	-0.85289929	0.05495667	-0.26230840
C	0.45953851	-0.04099613	0.07046822
C	1.34966250	-0.75736782	-0.83955027
C	1.44836119	0.59527301	0.97174980
C	2.71823402	0.39909635	0.46180170
C	2.70124932	-0.69301953	-0.48721122
N	-1.89340839	0.28671221	0.65322512
C	-3.09052772	-0.36837701	0.13723934
H	3.56858844	-1.15347982	-0.88807584
C	-1.56156400	-0.37917663	-1.61077181
C	-3.03074929	-0.01033267	-1.31858559
H	-3.16966890	1.04860455	-1.46169341
H	-3.78418500	-0.56478861	-1.88143213
H	-2.94370858	-1.41817132	0.13490643
H	-3.99112126	-0.07854276	0.69771685
H	-1.14900473	0.18471941	-2.42478497
H	0.99335745	-1.19249548	-1.78570936
C	-1.41018427	-0.39440778	1.92806837
H	-1.58795699	0.23286451	2.78292042
H	-1.83940185	-1.37293641	2.02499629
H	-0.36160506	-0.50263272	1.95071756
H	1.21656234	1.32565151	1.76110228
C	3.84713651	1.37064548	-0.03982902
H	4.24343692	0.96549742	-0.95106154
H	3.44846372	2.37191415	-0.17755485
H	4.63060249	1.46310050	0.69719294
H	-1.34337098	-1.41559135	-1.81447138

Time = 363.50 fs
E(CASSCF/6-31G(d)) = -479.4543836

C	-0.85190296	0.05627628	-0.26334923
C	0.45652361	-0.03823863	0.07756289
C	1.35092247	-0.76135967	-0.83705304
C	1.45047723	0.58973744	0.97677791
C	2.71716150	0.39966560	0.45875321
C	2.70133537	-0.69123629	-0.48849473
N	-1.89764043	0.28237822	0.65796764
C	-3.09797333	-0.36067562	0.13577695
H	3.57366896	-1.14468176	-0.88586629
C	-1.56195165	-0.38254654	-1.60862804
C	-3.03182989	-0.00770700	-1.32475111
H	-3.17171505	1.05145529	-1.47222204
H	-3.78034320	-0.55519009	-1.90374419
H	-2.95567706	-1.41424101	0.12647170
H	-4.00093049	-0.07913992	0.69093610

H	-1.14352447	0.17492846	-2.43105970
H	0.99452644	-1.20825284	-1.77751935
C	-1.40221070	-0.39553210	1.92519836
H	-1.58961546	0.22032171	2.78378517
H	-1.81706136	-1.37911985	2.01599633
H	-0.35661031	-0.50555034	1.95814522
H	1.22969142	1.32316204	1.76878185
C	3.84973592	1.37435091	-0.04221699
H	4.25114000	0.96527546	-0.95011609
H	3.44009350	2.36682947	-0.20308995
H	4.63143824	1.48446293	0.69466704
H	-1.33953178	-1.42298992	-1.79836716

Time = 364.00 fs
E(CASSCF/6-31G(d)) = -479.4526841

C	-0.85126178	0.05749426	-0.26454813
C	0.45400257	-0.03557996	0.08428950
C	1.35245276	-0.76565037	-0.83501583
C	1.45252483	0.58456619	0.98204591
C	2.71639452	0.40037978	0.45557979
C	2.70063735	-0.68942185	-0.48986601
N	-1.90156070	0.27782963	0.66311550
C	-3.10541639	-0.35278622	0.13430094
H	3.57893190	-1.13644402	-0.88489165
C	-1.56208134	-0.38569538	-1.60635288
C	-3.03291362	-0.00517222	-1.33099109
H	-3.17390843	1.05471458	-1.48307095
H	-3.77558777	-0.54526245	-1.92612076
H	-2.96845107	-1.41287015	0.11963948
H	-4.00965005	-0.07988297	0.68345387
H	-1.13803604	0.16528706	-2.43791511
H	0.99609011	-1.22439404	-1.76695237
C	-1.39451491	-0.39636516	1.92236519
H	-1.59321890	0.20778777	2.78561841
H	-1.79584991	-1.38603653	2.00752425
H	-0.35030098	-0.50877134	1.96488389
H	1.24274458	1.31979598	1.77421080
C	3.85214922	1.37807467	-0.04409429
H	4.25915151	0.96548070	-0.94948938
H	3.43177833	2.36143694	-0.22913159
H	4.63323126	1.50588755	0.69206487
H	-1.33704312	-1.43088668	-1.78224427

Time = 364.50 fs
E(CASSCF/6-31G(d)) = -479.4511818

C	-0.85102437	0.05862234	-0.26586500
C	0.45202074	-0.03305711	0.09060176
C	1.35422056	-0.77021292	-0.83340287
C	1.45451354	0.57977615	0.98755096
C	2.71594833	0.40124587	0.45229093
C	2.69918914	-0.68758382	-0.49132856
N	-1.90513576	0.27309499	0.66861839
C	-3.11283052	-0.34479762	0.13278570
H	3.58422836	-1.12878750	-0.88514868
C	-1.56192670	-0.38861718	-1.60397915
C	-3.03399160	-0.00270738	-1.33729815
H	-3.17609843	1.05830663	-1.49420455
H	-3.76992107	-0.53510602	-1.94827775
H	-2.98207962	-1.41360182	0.11444691
H	-4.01747273	-0.08053151	0.67545181
H	-1.13271720	0.15553505	-2.44497553
H	0.99796605	-1.24089344	-1.75417267
C	-1.38713509	-0.39694844	1.91959037
H	-1.59873678	0.19531498	2.78846476
H	-1.77587999	-1.39375041	1.99959377
H	-0.34257684	-0.51203678	1.97079499
H	1.25558611	1.31557348	1.77744760
C	3.85436612	1.38180754	-0.04548685
H	4.26747423	0.96603444	-0.94903661
H	3.42354326	2.35580337	-0.25552842
H	4.63581188	1.52736761	0.68938012
H	-1.33603568	-1.43906594	-1.76615371

Time = 365.00 fs
E(CASSCF/6-31G(d)) = -479.4497671

C	-0.85122952	0.05966996	-0.26725959
C	0.45062017	-0.03070131	0.09645665
C	1.35619081	-0.77501380	-0.83216604
C	1.45645964	0.57537278	0.99327084
C	2.71582750	0.40226575	0.44889961
C	2.69704585	-0.68573435	-0.49288872
N	-1.90833996	0.26820574	0.67442450
C	-3.12018229	-0.33681145	0.13120563
H	3.58930917	-1.12166981	-0.88658580
C	-1.56146553	-0.39131204	-1.60153926
C	-3.03505085	-0.00028752	-1.34366308
H	-3.17813384	1.06212326	-1.50557258
H	-3.76338503	-0.52484841	-1.96996124
H	-2.99661017	-1.41581833	0.11090627
H	-4.02466166	-0.08082103	0.66715051
H	-1.12773569	0.14542729	-2.45186941

H	1.00003452	-1.25777834	-1.73948889
C	-1.38010202	-0.39733184	1.91690312
H	-1.60608580	0.18290035	2.79228125
H	-1.75719861	-1.40221127	1.99220130
H	-0.33352107	-0.51507274	1.97577656
H	1.26805029	1.31062742	1.77869463
C	3.85638073	1.38554241	-0.04642776
H	4.27609225	0.96687072	-0.94856454
H	3.41543050	2.34996978	-0.28210686
H	4.63896188	1.54888842	0.68658932
H	-1.33662777	-1.44728549	-1.75015246

Time = 365.50 fs
E(CASSCF/6-31G(d)) = -479.4482825

C	-0.85190250	0.06063451	-0.26869787
C	0.44983814	-0.02854131	0.10180920
C	1.35832144	-0.78001596	-0.83124768
C	1.45840113	0.57135875	0.99916310
C	2.71602064	0.40342877	0.44542986
C	2.69428664	-0.68388415	-0.49454903
N	-1.91116651	0.26320827	0.68049095
C	-3.12742925	-0.32893606	0.12953823
H	3.59385353	-1.11499063	-0.88911706
C	-1.56067959	-0.39378697	-1.59906382
C	-3.03607406	0.00211502	-1.35007405
H	-3.17986654	1.06603657	-1.51711159
H	-3.75606200	-0.51464349	-1.99095609
H	-3.01208559	-1.41882989	0.10899834
H	-4.03152884	-0.08047366	0.65879323
H	-1.12324315	0.13475629	-2.45827076
H	1.00215026	-1.27510721	-1.72334412
C	-1.37344232	-0.39757340	1.91433334
H	-1.61512388	0.17048908	2.79694356
H	-1.73978425	-1.41127354	1.98534096
H	-0.32338577	-0.51760581	1.97977227
H	1.27994275	1.30516346	1.77831521
C	3.85818669	1.38927612	-0.04696340
H	4.28498253	0.96793855	-0.94784849
H	3.40750584	2.34395961	-0.30867221
H	4.64241090	1.57041560	0.68367661
H	-1.33893509	-1.45529000	-1.73431551

Time = 366.00 fs
E(CASSCF/6-31G(d)) = -479.4467696

C	-0.85298912	0.06150109	-0.27014700

C	0.44960163	-0.02663551	0.10659358
C	1.36056830	-0.78517984	-0.83059907
C	1.46039159	0.56772633	1.00520029
C	2.71654900	0.40475285	0.44189518
C	2.69099265	-0.68203350	-0.49630803
N	-1.91362335	0.25815795	0.68677583
C	-3.13451970	-0.32128416	0.12776737
H	3.59749464	-1.10872363	-0.89257628
C	-1.55956222	-0.39603398	-1.59658702
C	-3.03703984	0.00452647	-1.35651292
H	-3.18115586	1.06990961	-1.52873793
H	-3.74807865	-0.50467012	-2.01106820
H	-3.02852531	-1.42194682	0.10867139
H	-4.03839188	-0.07921521	0.65062994
H	-1.11936915	0.12336432	-2.46392317
H	1.00409092	-1.29308919	-1.70620679
C	-1.36718032	-0.39773791	1.91191863
H	-1.62565622	0.15799646	2.80224002
H	-1.72357324	-1.42070897	1.97900319
H	-0.31257249	-0.51936460	1.98275566
H	1.29105250	1.29954454	1.77672974
C	3.85978921	1.39300567	-0.04712833
H	4.29408745	0.96919202	-0.94667867
H	3.39983849	2.33776386	-0.33500790
H	4.64588194	1.59190708	0.68055538
H	-1.34301396	-1.46281147	-1.71869422

Time = 366.50 fs
E(CASSCF/6-31G(d)) = -479.4446811

C	-0.85450595	0.06230436	-0.27154643
C	0.44993054	-0.02499584	0.11078584
C	1.36289955	-0.79046280	-0.83015379
C	1.46243243	0.56443336	1.01132520
C	2.71740911	0.40625107	0.43830533
C	2.68723566	-0.68019904	-0.49819103
N	-1.91567631	0.25304873	0.69319664
C	-3.14143957	-0.31393844	0.12587045
H	3.59987681	-1.10278748	-0.89682863
C	-1.55811972	-0.39806743	-1.59412281
C	-3.03792429	0.00697706	-1.36296942
H	-3.18188258	1.07361563	-1.54038053
H	-3.73957327	-0.49510007	-2.03016590
H	-3.04589425	-1.42447134	0.10988481
H	-4.04554581	-0.07678370	0.64287756
H	-1.11614686	0.11112971	-2.46858414
H	1.00563024	-1.31198735	-1.68858468

C	-1.36131493	-0.39788593	1.90972516
H	-1.63746728	0.14528889	2.80788464
H	-1.70848062	-1.43020180	1.97310620
H	-0.30157148	-0.52006560	1.98469615
H	1.30118836	1.29423212	1.77444107
C	3.86120264	1.39673218	-0.04695239
H	4.30335194	0.97057782	-0.94482239
H	3.39248576	2.33132402	-0.36091738
H	4.64912644	1.61337031	0.67714391
H	-1.34887037	-1.46967754	-1.70332316

Time = 367.00 fs
E(CASSCF/6-31G(d)) = -479.4421840

C	-0.85643706	0.06305899	-0.27284652
C	0.45081679	-0.02364068	0.11435445
C	1.36527375	-0.79582023	-0.82985016
C	1.46454712	0.56144240	1.01747060
C	2.71858877	0.40792983	0.43467949
C	2.68310283	-0.67839239	-0.50021219
N	-1.91731564	0.24789958	0.69968325
C	-3.14816679	-0.30697766	0.12383618
H	3.60067605	-1.09711218	-0.90173848
C	-1.55636587	-0.39989990	-1.59168293
C	-3.03869974	0.00949304	-1.36942525
H	-3.18194166	1.07704424	-1.55195514
H	-3.73071726	-0.48612310	-2.04814479
H	-3.06413349	-1.42581730	0.11256244
H	-4.05322286	-0.07294185	0.63571756
H	-1.11357680	0.09800350	-2.47211286
H	1.00655557	-1.33203794	-1.67100011
C	-1.35585310	-0.39808028	1.90781220
H	-1.65029366	0.13223633	2.81356199
H	-1.69439761	-1.43942679	1.96759161
H	-0.29091521	-0.51945802	1.98560630
H	1.31015320	1.28971977	1.77201239
C	3.86243526	1.40045737	-0.04647488
H	4.31272354	0.97204363	-0.94207009
H	3.38552691	2.32458704	-0.38617624
H	4.65188470	1.63477543	0.67339832
H	-1.35648850	-1.47574050	-1.68825866

Time = 367.50 fs
E(CASSCF/6-31G(d)) = -479.4394441

C	-0.85875244	0.06377599	-0.27399947
C	0.45224061	-0.02258599	0.11727097

C	1.36764343	-0.80121067	-0.82963402
C	1.46676189	0.55871466	1.02356628
C	2.72007071	0.40979383	0.43103883
C	2.67868415	-0.67662126	-0.50238216
N	-1.91854019	0.24273193	0.70616696
C	-3.15468743	-0.30046447	0.12166179
H	3.59963208	-1.09165053	-0.90718546
C	-1.55432371	-0.40154549	-1.58927210
C	-3.03933628	0.01209744	-1.37585989
H	-3.18124850	1.08011248	-1.56337699
H	-3.72169696	-0.47792852	-2.06493594
H	-3.08315861	-1.42551972	0.11660493
H	-4.06156098	-0.06748742	0.62926964
H	-1.11160836	0.08399763	-2.47446067
H	1.00668464	-1.35345387	-1.65390697
C	-1.35080262	-0.39838185	1.90623683
H	-1.66384877	0.11871933	2.81895153
H	-1.68120228	-1.44807337	1.96240324
H	-0.28112129	-0.51732458	1.98552910
H	1.31776881	1.28649812	1.77000201
C	3.86349407	1.40418430	-0.04573691
H	4.32215861	0.97353136	-0.93824427
H	3.37904254	2.31750080	-0.41056139
H	4.65391679	1.65608889	0.66930427
H	-1.36581994	-1.48090367	-1.67355681

Time = 368.00 fs
E(CASSCF/6-31G(d)) = -479.4367105

C	-0.86141080	0.06446383	-0.27495902
C	0.45417281	-0.02184485	0.11951108
C	1.36995409	-0.80659929	-0.82946307
C	1.46910269	0.55621457	1.02954453
C	2.72183353	0.41184617	0.42740580
C	2.67406906	-0.67488879	-0.50470775
N	-1.91935554	0.23756796	0.71258014
C	-3.16099803	-0.29444345	0.11935346
H	3.59657129	-1.08638657	-0.91307376
C	-1.55202527	-0.40301936	-1.58688780
C	-3.03980284	0.01480896	-1.38225110
H	-3.17974159	1.08277171	-1.57456441
H	-3.71270391	-0.47069686	-2.08050000
H	-3.10286306	-1.42324857	0.12189369
H	-4.07058044	-0.06025992	0.62357977
H	-1.11013921	0.06918212	-2.47567194
H	1.00589032	-1.37638462	-1.63763618
C	-1.34617354	-0.39884743	1.90505153

H	-1.67783696	0.10464446	2.82375439
H	-1.66876987	-1.45587237	1.95748886
H	-0.27264795	-0.51348769	1.98453609
H	1.32388899	1.28499795	1.76889998
C	3.86438443	1.40791640	-0.04477997
H	4.33162621	0.97497445	-0.93320907
H	3.37310730	2.31002420	-0.43385922
H	4.65501386	1.67727729	0.66488276
H	-1.37678399	-1.48512673	-1.65927097

Time = 368.50 fs
E(CASSCF/6-31G(d)) = -479.4342675

C	-0.86436200	0.06512895	-0.27568079
C	0.45657663	-0.02142742	0.12105525
C	1.37214322	-0.81196124	-0.82931026
C	1.47159285	0.55391474	1.03534583
C	2.72385253	0.41408825	0.42380348
C	2.66934392	-0.67319314	-0.50719165
N	-1.91977278	0.23242984	0.71885633
C	-3.16710625	-0.28894027	0.11692599
H	3.59142225	-1.08134026	-0.91933883
C	-1.54951072	-0.40433732	-1.58452052
C	-3.04006825	0.01764099	-1.38857552
H	-3.17738407	1.08501139	-1.58544184
H	-3.70392412	-0.46459116	-2.09481941
H	-3.12312307	-1.41881513	0.12829492
H	-4.08017547	-0.05114448	0.61861673
H	-1.10901829	0.05367885	-2.47588020
H	1.00412147	-1.40087861	-1.62235548
C	-1.34197912	-0.39952808	1.90430189
H	-1.69196762	0.08995573	2.82771704
H	-1.65697987	-1.46261693	1.95280142
H	-0.26585907	-0.50781336	1.98272530
H	1.32841131	1.28552994	1.76906541
C	3.86510982	1.41165641	-0.04364460
H	4.34111135	0.97629670	-0.92687646
H	3.36778323	2.30213623	-0.45587459
H	4.65500630	1.69831097	0.66019371
H	-1.38926998	-1.48842593	-1.64544920

Time = 369.00 fs
E(CASSCF/6-31G(d)) = -479.4323483

C	-0.86754919	0.06577600	-0.27612283
C	0.45940937	-0.02134094	0.12188940
C	1.37414039	-0.81728382	-0.82916489

C	1.47425147	0.55180026	1.04092345
C	2.72610042	0.41651973	0.42025512
C	2.66458977	-0.67152743	-0.50983231
N	-1.91980763	0.22733919	0.72493084
C	-3.17302984	-0.28396139	0.11440175
H	3.58422425	-1.07656887	-0.92595136
C	-1.54682712	-0.40551544	-1.58215449
C	-3.04010243	0.02060097	-1.39480941
H	-3.17416432	1.08685977	-1.59594235
H	-3.69552746	-0.45974843	-2.10788934
H	-3.14380357	-1.41217427	0.13566387
H	-4.09012209	-0.04007259	0.61427783
H	-1.10805189	0.03765253	-2.47529903
H	1.00141500	-1.42685615	-1.60805390
C	-1.33823632	-0.40046784	1.90402454
H	-1.70596874	0.07464199	2.83065020
H	-1.64572047	-1.46817705	1.94829991
H	-0.26099951	-0.50021271	1.98021895
H	1.33128574	1.28823379	1.77067676
C	3.86567182	1.41540565	-0.04236932
H	4.35061709	0.97741265	-0.91920879
H	3.36311404	2.29384328	-0.47643947
H	4.65376881	1.71916701	0.65533699
H	-1.40314099	-1.49087012	-1.63213191

Time = 369.50 fs
E(CASSCF/6-31G(d)) = -479.4310669

C	-0.87091060	0.06640772	-0.27624620
C	0.46262381	-0.02158983	0.12200524
C	1.37586856	-0.82256716	-0.82903129
C	1.47709234	0.54987119	1.04624589
C	2.72854799	0.41913842	0.41678365
C	2.65988057	-0.66987997	-0.51262393
N	-1.91947986	0.22231679	0.73074152
C	-3.17879430	-0.27949441	0.11180881
H	3.57512878	-1.07216557	-0.93291870
C	-1.54402683	-0.40656961	-1.57976901
C	-3.03987754	0.02368994	-1.40092939
H	-3.17009593	1.08838196	-1.60600955
H	-3.68765802	-0.45627198	-2.11970882
H	-3.16476493	-1.40342416	0.14384946
H	-4.10010092	-0.02702013	0.61040253
H	-1.10701336	0.02129858	-2.47420667
H	0.99789584	-1.45410042	-1.59455427
C	-1.33496586	-0.40170300	1.90424568
H	-1.71959891	0.05874009	2.83244262

H	-1.63489046	-1.47250858	1.94394982
H	-0.25817994	-0.49064106	1.97716094
H	1.33251907	1.29304901	1.77370735
C	3.86607030	1.41916325	-0.04098977
H	4.36016524	0.97822930	-0.91021790
H	3.35912067	2.28518387	-0.49542128
H	4.65122222	1.73983172	0.65045083
H	-1.41823845	-1.49257334	-1.61935026

Time = 370.00 fs
E(CASSCF/6-31G(d)) = -479.4303969

C	-0.87438132	0.06702496	-0.27601572
C	0.46616956	-0.02217580	0.12140077
C	1.37724676	-0.82782300	-0.82892537
C	1.48012347	0.54814244	1.05129669
C	2.73116468	0.42194021	0.41341111
C	2.65528157	-0.66823478	-0.51555690
N	-1.91881268	0.21738223	0.73622957
C	-3.18442964	-0.27550856	0.10917871
H	3.56439385	-1.06825552	-0.94028357
C	-1.54116545	-0.40751518	-1.57734035
C	-3.03936897	0.02690236	-1.40691306
H	-3.16521661	1.08967536	-1.61559890
H	-3.68042639	-0.45422481	-2.13027262
H	-3.18586982	-1.39280347	0.15269932
H	-4.10972933	-0.01200318	0.60679116
H	-1.10565625	0.00482840	-2.47292296
H	0.99376409	-1.48226825	-1.58155118
C	-1.33219185	-0.40326157	1.90498007
H	-1.73265784	0.04233478	2.83306925
H	-1.62439966	-1.47565594	1.93972267
H	-0.25737145	-0.47909507	1.97371396
H	1.33217398	1.29971675	1.77793268
C	3.86630396	1.42292567	-0.03953791
H	4.36979552	0.97864950	-0.89996052
H	3.35579857	2.27623043	-0.51272955
H	4.64733194	1.76030197	0.64570722
H	-1.43438682	-1.49368398	-1.60712490

Time = 370.50 fs
E(CASSCF/6-31G(d)) = -479.4302051

C	-0.87789491	0.06762661	-0.27540044
C	0.46999402	-0.02309791	0.12008043
C	1.37819402	-0.83307207	-0.82886956
C	1.48334736	0.54664116	1.05607151

C	2.73391886	0.42491893	0.41015834
C	2.65084817	-0.66657238	-0.51861836
N	-1.91783221	0.21255335	0.74134022
C	-3.18996674	-0.27195581	0.10654397
H	3.55237238	-1.06498927	-0.94812062
C	-1.53829960	-0.40836641	-1.57484421
C	-3.03855618	0.03022638	-1.41273963
H	-3.15958636	1.09086215	-1.62467806
H	-3.67390426	-0.45362503	-2.13956509
H	-3.20699021	-1.38068403	0.16206514
H	-4.11859757	0.00492697	0.60322531
H	-1.10373154	-0.01154856	-2.47177834
H	0.98927150	-1.51092003	-1.56866547
C	-1.32994087	-0.40516311	1.90623065
H	-1.74499517	0.02555601	2.83259484
H	-1.61416827	-1.47775123	1.93559514
H	-0.25840894	-0.46560817	1.97005597
H	1.33036336	1.30781181	1.78296714
C	3.86637088	1.42668650	-0.03804143
H	4.37956341	0.97857618	-0.88853134
H	3.35311628	2.26708918	-0.52832152
H	4.64210438	1.78058565	0.64130570
H	-1.45139849	-1.49437070	-1.59546477

Time = 371.00 fs
E(CASSCF/6-31G(d)) = -479.4303242

C	-0.88138479	0.06820951	-0.27437408
C	0.47404356	-0.02435272	0.11805516
C	1.37863379	-0.83834025	-0.82888750
C	1.48676153	0.54540235	1.06057364
C	2.73677799	0.42806615	0.40704450
C	2.64662498	-0.66487085	-0.52179323
N	-1.91656691	0.20784576	0.74602335
C	-3.19543399	-0.26877243	0.10393575
H	3.53949034	-1.06253257	-0.95652893
C	-1.53548448	-0.40913605	-1.57225863
C	-3.03742321	0.03364439	-1.41839027
H	-3.15328505	1.09207984	-1.63322669
H	-3.66812205	-0.45444356	-2.14755686
H	-3.22801415	-1.36755719	0.17180797
H	-4.12630327	0.02368972	0.59948726
H	-1.10100688	-0.02764261	-2.47107707
H	0.98469386	-1.53955956	-1.55550054
C	-1.32824050	-0.40741879	1.90798873
H	-1.75651723	0.00857361	2.83117258
H	-1.60412516	-1.47900803	1.93154763

H	-0.26100527	-0.45024465	1.96637636
H	1.32724072	1.31679638	1.78831999
C	3.86626959	1.43043629	-0.03652333
H	4.38953631	0.97791806	-0.87605294
H	3.35101591	2.25789687	-0.54220551
H	4.63558009	1.80070065	0.63746423
H	-1.46907778	-1.49480718	-1.58436692

Time = 371.50 fs
E(CASSCF/6-31G(d)) = -479.4306257

C	-0.88478556	0.06876809	-0.27291572
C	0.47826421	-0.02593419	0.11534290
C	1.37849874	-0.84365465	-0.82899975
C	1.49035945	0.54446365	1.06480888
C	2.73970890	0.43137140	0.40408664
C	2.64264585	-0.66310746	-0.52506552
N	-1.91504724	0.20327253	0.75023446
C	-3.20085420	-0.26588158	0.10138187
H	3.52621753	-1.06105339	-0.96562045
C	-1.53277145	-0.40983504	-1.56956705
C	-3.03595899	0.03713390	-1.42384818
H	-3.14640916	1.09347046	-1.64123529
H	-3.66307047	-0.45660430	-2.15420520
H	-3.24885185	-1.35401050	0.18180312
H	-4.13248065	0.04418057	0.59537635
H	-1.09728674	-0.04329962	-2.47105954
H	0.98030308	-1.56767777	-1.54169151
C	-1.32711730	-0.41003176	1.91023441
H	-1.76719038	-0.00841032	2.82903720
H	-1.59420518	-1.47970982	1.92756343
H	-0.26477838	-0.43309400	1.96287183
H	1.32298890	1.32608290	1.79345789
C	3.86600006	1.43416286	-0.03500168
H	4.39978718	0.97659711	-0.86266364
H	3.34941631	2.24881483	-0.55444090
H	4.62782396	1.82067095	0.63440717
H	-1.48722388	-1.49515587	-1.57381728

Time = 372.00 fs
E(CASSCF/6-31G(d)) = -479.4310983

C	-0.88803438	0.06929413	-0.27100916
C	0.48260224	-0.02783411	0.11196781
C	1.37773565	-0.84903973	-0.82921957
C	1.49413227	0.54386094	1.06878079
C	2.74267699	0.43482145	0.40129949

C	2.63893326	-0.66126007	-0.52842005
N	-1.91330456	0.19884309	0.75393426
C	-3.20624270	-0.26319698	0.09890520
H	3.51303044	-1.06070506	-0.97550565
C	-1.53020608	-0.41047205	-1.56676111
C	-3.03415728	0.04066884	-1.42909854
H	-3.13906827	1.09516811	-1.64870342
H	-3.65870395	-0.45998694	-2.15945707
H	-3.26943924	-1.34068922	0.19194417
H	-4.13682235	0.06627701	0.59072170
H	-1.09243029	-0.05841385	-2.47186583
H	0.97634274	-1.59479362	-1.52694370
C	-1.32659372	-0.41299761	1.91293821
H	-1.77704285	-0.02517085	2.82649289
H	-1.58434660	-1.48019417	1.92362607
H	-0.26928966	-0.41426436	1.95974108
H	1.31780783	1.33509473	1.79786081
C	3.86556508	1.43785163	-0.03348945
H	4.41038847	0.97455567	-0.84850460
H	3.34821668	2.24002119	-0.56513669
H	4.61891472	1.84052331	0.63235341
H	-1.50563208	-1.49555300	-1.56378967

Time = 372.50 fs
E(CASSCF/6-31G(d)) = -479.4318743

C	-0.89107259	0.06977698	-0.26864283
C	0.48700514	-0.03004226	0.10796024
C	1.37631241	-0.85451431	-0.82954938
C	1.49807171	0.54362456	1.07248647
C	2.74564430	0.43840013	0.39869562
C	2.63549611	-0.65930875	-0.53184528
N	-1.91137008	0.19456209	0.75708845
C	-3.21160654	-0.26062782	0.09652256
H	3.50037023	-1.06160803	-0.98627495
C	-1.52782653	-0.41105327	-1.56384266
C	-3.03201660	0.04422114	-1.43412840
H	-3.13138172	1.09728609	-1.65563767
H	-3.65494704	-0.46443253	-2.16325508
H	-3.28973933	-1.32824280	0.20214493
H	-4.13909439	0.08984417	0.58539117
H	-1.08636429	-0.07293509	-2.47351028
H	0.97300902	-1.62048720	-1.51105854
C	-1.32668441	-0.41630488	1.91606343
H	-1.78616284	-0.04147248	2.82389532
H	-1.57448744	-1.48083176	1.91971704
H	-0.27409349	-0.39387806	1.95717966

H	1.31190261	1.34331781	1.80106686
C	3.86497141	1.44148605	-0.03199441
H	4.42140493	0.97176368	-0.83370635
H	3.34730265	2.23169989	-0.57444697
H	4.60893336	1.86028222	0.63150325
H	-1.52409425	-1.49609685	-1.55424583

Time = 373.00 fs
E(CASSCF/6-31G(d)) = -479.4339044

C	-0.89374163	0.07019477	-0.26586879
C	0.49135050	-0.03250752	0.10336412
C	1.37401821	-0.86010270	-0.83008076
C	1.50205168	0.54373932	1.07595109
C	2.74872940	0.44211123	0.39625498
C	2.63258025	-0.65724128	-0.53527549
N	-1.90935937	0.19052961	0.75976752
C	-3.21689859	-0.25810472	0.09426567
H	3.48857245	-1.06389922	-0.99783501
C	-1.52564836	-0.41163565	-1.56081930
C	-3.02953853	0.04776087	-1.43892399
H	-3.12346848	1.09992109	-1.66204988
H	-3.65170942	-0.46974997	-2.16558554
H	-3.30987294	-1.31733892	0.21224707
H	-4.13919937	0.11475438	0.57926742
H	-1.07915282	-0.08682612	-2.47596031
H	0.97049269	-1.64441452	-1.49384166
C	-1.32741683	-0.41996248	1.91951481
H	-1.79463396	-0.05708025	2.82171390
H	-1.56451959	-1.48214946	1.91596146
H	-0.27895882	-0.37204738	1.95550697
H	1.30550874	1.35014841	1.80280464
C	3.86421133	1.44506302	-0.03054562
H	4.43288637	0.96823778	-0.81839260
H	3.34658404	2.22408316	-0.58259382
H	4.59787558	1.87989092	0.63204723
H	-1.54255681	-1.49681536	-1.54532623

Time = 373.50 fs
E(CASSCF/6-31G(d)) = -479.4386362

C	-0.89598723	0.07049604	-0.26264445
C	0.49561923	-0.03522564	0.09817991
C	1.37077825	-0.86577896	-0.83078166
C	1.50604258	0.54422360	1.07915379
C	2.75190084	0.44591239	0.39397532
C	2.63022960	-0.65504218	-0.53868146

N	-1.90729248	0.18674428	0.76192351
C	-3.22210805	-0.25554892	0.09214019
H	3.47790057	-1.06748095	-1.01027138
C	-1.52369615	-0.41220668	-1.55771950
C	-3.02673211	0.05125994	-1.44347395
H	-3.11545509	1.10311166	-1.66795719
H	-3.64887356	-0.47572263	-2.16641004
H	-3.32985622	-1.30843794	0.22218540
H	-4.13706300	0.14086251	0.57229972
H	-1.07086975	-0.10014576	-2.47894322
H	0.96885789	-1.66636817	-1.47530615
C	-1.32877401	-0.42394820	1.92325431
H	-1.80263389	-0.07177489	2.82031847
H	-1.55438751	-1.48449981	1.91233235
H	-0.28366363	-0.34891771	1.95488487
H	1.29882121	1.35532624	1.80278079
C	3.86330249	1.44856372	-0.02914220
H	4.44485242	0.96402072	-0.80263735
H	3.34594101	2.21732979	-0.58978990
H	4.58579964	1.89936054	0.63412251
H	-1.56076236	-1.49769821	-1.53689377

Time = 374.00 fs
E(CASSCF/6-31G(d)) = -479.4416744

C	-0.89778099	0.07067168	-0.25897921
C	0.49976568	-0.03817943	0.09246757
C	1.36669578	-0.87153592	-0.83160744
C	1.51006291	0.54507780	1.08205438
C	2.75508892	0.44978727	0.39187368
C	2.62836501	-0.65271582	-0.54209101
N	-1.90519171	0.18318381	0.76353732
C	-3.22722247	-0.25289122	0.09014427
H	3.46851962	-1.07230863	-1.02351209
C	-1.52198396	-0.41276651	-1.55457319
C	-3.02360618	0.05469439	-1.44776860
H	-3.10746780	1.10684961	-1.67337230
H	-3.64632597	-0.48212295	-2.16570247
H	-3.34968372	-1.30179346	0.23191945
H	-4.13269454	0.16801947	0.56449542
H	-1.06164406	-0.11297487	-2.48210630
H	0.96807431	-1.68625760	-1.45560697
C	-1.33073255	-0.42823166	1.92724160
H	-1.81037413	-0.08538502	2.82003800
H	-1.54400287	-1.48815387	1.90878945
H	-0.28800417	-0.32466453	1.95546237
H	1.29200178	1.35870108	1.80083926

C	3.86226940	1.45197248	-0.02777637
H	4.45730615	0.95919930	-0.78648530
H	3.34526957	2.21156459	-0.59626689
H	4.57274278	1.91867512	0.63780022
H	-1.57848674	-1.49872871	-1.52888572

Time = 374.50 fs
E(CASSCF/6-31G(d)) = -479.4449616

C	-0.89908340	0.07070936	-0.25489685
C	0.50373941	-0.04134975	0.08628348
C	1.36184364	-0.87735306	-0.83251961
C	1.51410792	0.54628701	1.08461528
C	2.75825568	0.45371755	0.38995431
C	2.62695679	-0.65027268	-0.54552558
N	-1.90309105	0.17983826	0.76461249
C	-3.23221621	-0.25008407	0.08827536
H	3.46045068	-1.07826134	-1.03740239
C	-1.52051662	-0.41332317	-1.55141152
C	-3.02017113	0.05804330	-1.45179780
H	-3.09962469	1.11107656	-1.67830489
H	-3.64395812	-0.48872374	-2.16346990
H	-3.36936514	-1.29743570	0.24138331
H	-4.12618383	0.19607384	0.55589786
H	-1.05166757	-0.12540507	-2.48504106
H	0.96806007	-1.70412985	-1.43497431
C	-1.33325884	-0.43278357	1.93143179
H	-1.81805714	-0.09778699	2.82114326
H	-1.53326441	-1.49331585	1.90532214
H	-0.29197577	-0.29949684	1.95738976
H	1.28518658	1.36024539	1.79696298
C	3.86113767	1.45527902	-0.02644192
H	4.47021478	0.95390792	-0.76994901
H	3.34449630	2.20687976	-0.60225543
H	4.55870351	1.93777597	0.64309613
H	-1.59553191	-1.49984135	-1.52126385

Time = 375.00 fs
E(CASSCF/6-31G(d)) = -479.4484952

C	-0.89985397	0.07059338	-0.25043098
C	0.50748883	-0.04471135	0.07969059
C	1.35630752	-0.88319901	-0.83347797
C	1.51816653	0.54782208	1.08679103
C	2.76137205	0.45768487	0.38821687
C	2.62598182	-0.64773249	-0.54901029
N	-1.90102832	0.17669970	0.76516648

C	-3.23705672	-0.24710122	0.08652960
H	3.45359921	-1.08514481	-1.05170900
C	-1.51929221	-0.41388882	-1.54826692
C	-3.01643932	0.06129029	-1.45555101
H	-3.09203763	1.11569128	-1.68276410
H	-3.64167472	-0.49530327	-2.15975733
H	-3.38890593	-1.29516028	0.25049490
H	-4.11769758	0.22488280	0.54658916
H	-1.04116222	-0.13752487	-2.48732231
H	0.96866167	-1.72016074	-1.41371946
C	-1.33630868	-0.43757294	1.93578253
H	-1.82586648	-0.10892124	2.82380742
H	-1.52205889	-1.50008540	1.90193378
H	-0.29574724	-0.27365716	1.96080598
H	1.27848380	1.36005557	1.79125477
C	3.85993548	1.45847854	-0.02513158
H	4.48351776	0.94832009	-0.75301075
H	3.34358237	2.20331686	-0.60797588
H	4.54364959	1.95657434	0.64997179
H	-1.61171126	-1.50094955	-1.51400031

Time = 375.50 fs
E(CASSCF/6-31G(d)) = -479.4520174

C	-0.90005481	0.07030821	-0.24562525
C	0.51096273	-0.04823605	0.07275634
C	1.35018939	-0.88903293	-0.83443970
C	1.52222659	0.54964136	1.08853035
C	2.76441389	0.46167075	0.38665603
C	2.62541587	-0.64512185	-0.55257499
N	-1.89904272	0.17375975	0.76522901
C	-3.24170784	-0.24393538	0.08490186
H	3.44777687	-1.09270838	-1.06613684
C	-1.51830350	-0.41447852	-1.54516869
C	-3.01242423	0.06442368	-1.45901731
H	-3.08480946	1.12055489	-1.68675820
H	-3.63939859	-0.50165561	-2.15464899
H	-3.40829849	-1.29455069	0.25915565
H	-4.10746358	0.25431127	0.53668358
H	-1.03036113	-0.14941121	-2.48854421
H	0.96966772	-1.73464994	-1.39221614
C	-1.33983043	-0.44256738	1.94025783
H	-1.83395154	-0.11880108	2.82808195
H	-1.51026399	-1.50843798	1.89864226
H	-0.29962838	-0.24742645	1.96583111
H	1.27196514	1.35835394	1.78393210
C	3.85869204	1.46157184	-0.02383703

H	4.49713010	0.94264248	-0.73562840
H	3.34252889	2.20085808	-0.61362495
H	4.52752647	1.97495559	0.65833739
H	-1.62685138	-1.50195391	-1.50707547

Time = 376.00 fs
E(CASSCF/6-31G(d)) = -479.4552763

C	-0.89965990	0.06983918	-0.24053114
C	0.51411070	-0.05189395	0.06555588
C	1.34361126	-0.89480784	-0.83536232
C	1.52628195	0.55169524	1.08977573
C	2.76735403	0.46565659	0.38526603
C	2.62522624	-0.64247357	-0.55625534
N	-1.89716995	0.17100426	0.76483722
C	-3.24613067	-0.24059320	0.08338580
H	3.44274196	-1.10066266	-1.08034840
C	-1.51754009	-0.41510803	-1.54213958
C	-3.00813958	0.06743616	-1.46218389
H	-3.07802906	1.12550279	-1.69029203
H	-3.63707689	-0.50759835	-2.14826816
H	-3.42750313	-1.29502391	0.26725500
H	-4.09575197	0.28422397	0.52632319
H	-1.01949102	-0.16111592	-2.48835426
H	0.97082286	-1.74800796	-1.37088050
C	-1.34377011	-0.44773125	1.94482837
H	-1.84241351	-0.12750709	2.83387897
H	-1.49776801	-1.51821046	1.89547578
H	-0.30402174	-0.22113254	1.97255496
H	1.26566441	1.35548943	1.77531203
C	3.85743789	1.46456647	-0.02254811
H	4.51094390	0.93710614	-0.71775101
H	3.34138230	2.19942579	-0.61935551
H	4.51026929	1.99277527	0.66804875
H	-1.64078772	-1.50276104	-1.50047121

Time = 376.50 fs
E(CASSCF/6-31G(d)) = -479.4579285

C	-0.89866720	0.06917681	-0.23520308
C	0.51689787	-0.05565775	0.05817206
C	1.33673682	-0.90047235	-0.83619436
C	1.53034336	0.55392903	1.09046088
C	2.77014635	0.46962244	0.38404019
C	2.62533936	-0.63982349	-0.56009556
N	-1.89543396	0.16840590	0.76402592
C	-3.25029213	-0.23708820	0.08197145

H	3.43823287	-1.10870842	-1.09399638
C	-1.51698952	-0.41578842	-1.53919585
C	-3.00359975	0.07032450	-1.46503754
H	-3.07177406	1.13035395	-1.69337163
H	-3.63468080	-0.51297836	-2.14077127
H	-3.44644953	-1.29589549	0.27468930
H	-4.08285573	0.31448678	0.51566940
H	-1.00875087	-0.17267045	-2.48646736
H	0.97184581	-1.76071289	-1.35016193
C	-1.34807264	-0.45302573	1.94948005
H	-1.85131081	-0.13518927	2.84097519
H	-1.48447430	-1.52910234	1.89246265
H	-0.30933569	-0.19513476	1.98102589
H	1.25957326	1.35192944	1.76578554
C	3.85620367	1.46747420	-0.02125072
H	4.52484347	0.93195387	-0.69932435
H	3.34022618	2.19887666	-0.62527682
H	4.49182197	2.00988461	0.67892436
H	-1.65336024	-1.50329286	-1.49415813

Time = 377.00 fs
E(CASSCF/6-31G(d)) = -479.4599752

C	-0.89709806	0.06830976	-0.22969154
C	0.51930891	-0.05950270	0.05068959
C	1.32974472	-0.90597257	-0.83688064
C	1.53442937	0.55628634	1.09051473
C	2.77273390	0.47354539	0.38296981
C	2.62565808	-0.63720818	-0.56413975
N	-1.89384630	0.16592750	0.76282421
C	-3.25416394	-0.23343537	0.08064630
H	3.43399924	-1.11654610	-1.10675380
C	-1.51663803	-0.41652426	-1.53634676
C	-2.99881999	0.07308843	-1.46756406
H	-3.06610783	1.13492609	-1.69600582
H	-3.63220616	-0.51767563	-2.13234679
H	-3.46504358	-1.29645016	0.28136637
H	-4.06907227	0.34496091	0.50489117
H	-0.99830307	-0.18408060	-2.48268387
H	0.97245805	-1.77325581	-1.33051164
C	-1.35268511	-0.45840966	1.95421140
H	-1.86066068	-0.14205228	2.84903186
H	-1.47031499	-1.54070231	1.88963323
H	-0.31594688	-0.16981085	1.99125020
H	1.25364979	1.34822934	1.75578538
C	3.85501792	1.47031086	-0.01992788
H	4.53871278	0.92742819	-0.68030275

H	3.33917916	2.19901392	-0.63145135
H	4.47214556	2.02613502	0.69075633
H	-1.66441555	-1.50349485	-1.48808624

Time = 377.50 fs
E(CASSCF/6-31G(d)) = -479.4613395

C	-0.89497667	0.06722212	-0.22404852
C	0.52133414	-0.06340208	0.04319060
C	1.32279154	-0.91125854	-0.83737843
C	1.53854851	0.55870835	1.08987160
C	2.77507207	0.47740144	0.38204008
C	2.62609912	-0.63466231	-0.56842164
N	-1.89241776	0.16353526	0.76126813
C	-3.25771799	-0.22964815	0.07939851
H	3.42982437	-1.12388917	-1.11832806
C	-1.51646970	-0.41731971	-1.53359260
C	-2.99381628	0.07572939	-1.46974821
H	-3.06107862	1.13905184	-1.69820821
H	-3.62967312	-0.52160480	-2.12321607
H	-3.48319332	-1.29601049	0.28720200
H	-4.05469092	0.37549758	0.49414933
H	-0.98827221	-0.19532158	-2.47691468
H	0.97241792	-1.78608376	-1.31231661
C	-1.35756156	-0.46384307	1.95902457
H	-1.87044497	-0.14834043	2.85763759
H	-1.45525118	-1.55254011	1.88703633
H	-0.32418569	-0.14553505	2.00320580
H	1.24783370	1.34496586	1.74575767
C	3.85390310	1.47309675	-0.01856304
H	4.55244284	0.92375934	-0.66065714
H	3.33838987	2.19960842	-0.63790304
H	4.45121838	2.04137996	0.70332498
H	-1.67382798	-1.50334173	-1.48220584

Time = 378.00 fs
E(CASSCF/6-31G(d)) = -479.4621458

C	-0.89232436	0.06589547	-0.21833044
C	0.52296205	-0.06732581	0.03575502
C	1.31600572	-0.91629216	-0.83766438
C	1.54269675	0.56113751	1.08847662
C	2.77713284	0.48116682	0.38122947
C	2.62659959	-0.63221646	-0.57296266
N	-1.89116190	0.16120235	0.75940452
C	-3.26092614	-0.22573461	0.07821794
H	3.42554696	-1.13048301	-1.12847467

C	-1.51646646	-0.41818100	-1.53092373
C	-2.98860541	0.07824988	-1.47157432
H	-3.05671835	1.14259004	-1.69999745
H	-3.62712279	-0.52471398	-2.11362514
H	-3.50081911	-1.29398392	0.29212515
H	-4.03997421	0.40592886	0.48358516
H	-0.97874101	-0.20633956	-2.46918902
H	0.97154379	-1.79954004	-1.29585865
C	-1.36266483	-0.46928854	1.96392089
H	-1.88062533	-0.15432128	2.86634655
H	-1.43927569	-1.56412698	1.88473903
H	-0.33430889	-0.12265682	2.01684387
H	1.24206051	1.34267570	1.73612641
C	3.85287428	1.47585541	-0.01714030
H	4.56594045	0.92115431	-0.64038131
H	3.33802593	2.20041574	-0.64462590
H	4.42904248	2.05548360	0.71641078
H	-1.68150807	-1.50284145	-1.47647567

Time = 378.50 fs
E(CASSCF/6-31G(d)) = -479.4624075

C	-0.88916539	0.06431163	-0.21259658
C	0.52418272	-0.07124211	0.02846200
C	1.30949660	-0.92105295	-0.83773268
C	1.54686148	0.56352403	1.08628863
C	2.77889757	0.48481810	0.38051119
C	2.62710431	-0.62989588	-0.57777444
N	-1.89009159	0.15890494	0.75728689
C	-3.26376272	-0.22169331	0.07709552
H	3.42107272	-1.13612187	-1.13700938
C	-1.51660881	-0.41911428	-1.52832113
C	-2.98320518	0.08065235	-1.47302683
H	-3.05304265	1.14543555	-1.70139871
H	-3.62461272	-0.52698314	-2.10383491
H	-3.51785545	-1.28989512	0.29608749
H	-4.02514530	0.43606061	0.47331448
H	-0.96974281	-0.21705503	-2.45965408
H	0.96973597	-1.81380274	-1.28129822
C	-1.36796667	-0.47471068	1.96890054
H	-1.89116116	-0.16026950	2.87471441
H	-1.42241675	-1.57498553	1.88281796
H	-0.34646187	-0.10148294	2.03208834
H	1.23628350	1.34179943	1.72723993
C	3.85193961	1.47861177	-0.01564324
H	4.57913563	0.91978726	-0.61949459
H	3.33826065	2.20118959	-0.65159138

H	4.40565124	2.06833173	0.72980341
H	-1.68740137	-1.50204092	-1.47086022

Time = 379.00 fs
E(CASSCF/6-31G(d)) = -479.4623071

C	-0.88552976	0.06245368	-0.20690695
C	0.52499045	-0.07511926	0.02138882
C	1.30335573	-0.92554096	-0.83759243
C	1.55102222	0.56583186	1.08328313
C	2.78035371	0.48833201	0.37985368
C	2.62756178	-0.62771926	-0.58285946
N	-1.88921697	0.15662065	0.75497235
C	-3.26620571	-0.21751102	0.07602283
H	3.41638248	-1.14066024	-1.14382126
C	-1.51687628	-0.42012455	-1.52575737
C	-2.97763469	0.08293823	-1.47409109
H	-3.05005068	1.14752580	-1.70244436
H	-3.62221017	-0.52841947	-2.09410995
H	-3.53425637	-1.28340554	0.29906981
H	-4.01037930	0.46567068	0.46342368
H	-0.96125917	-0.22736841	-2.44856546
H	0.96698589	-1.82884894	-1.26867553
C	-1.37344709	-0.48007634	1.97396159
H	-1.90202672	-0.16645416	2.88232892
H	-1.40473582	-1.58467728	1.88135194
H	-0.36066366	-0.08225931	2.04883975
H	1.23049526	1.34263150	1.71932625
C	3.85110067	1.48139006	-0.01405431
H	4.59198880	0.91979286	-0.59803949
H	3.33926155	2.20169386	-0.65875897
H	4.38111060	2.07984275	0.74331174
H	-1.69148785	-1.50102445	-1.46532686

Time = 379.50 fs
E(CASSCF/6-31G(d)) = -479.4620111

C	-0.88145535	0.06030619	-0.20132081
C	0.52538453	-0.07892411	0.01461258
C	1.29766174	-0.92977674	-0.83726834
C	1.55514791	0.56804049	1.07945422
C	2.78149598	0.49168587	0.37922140
C	2.62792069	-0.62569905	-0.58821138
N	-1.88854515	0.15432903	0.75252027
C	-3.26823612	-0.21316203	0.07499208
H	3.41153500	-1.14401779	-1.14886956
C	-1.51724747	-0.42121591	-1.52319834

C	-2.97191521	0.08510753	-1.47475390
H	-3.04772384	1.14884405	-1.70317270
H	-3.61998275	-0.52905014	-2.08470647
H	-3.54999236	-1.27432758	0.30108297
H	-3.99580416	0.49450305	0.45397147
H	-0.95322486	-0.23716324	-2.43627437
H	0.96336543	-1.84445596	-1.25791918
C	-1.37909381	-0.48535473	1.97909814
H	-1.91321978	-0.17312142	2.88883389
H	-1.38633308	-1.59282832	1.88041640
H	-0.37680297	-0.06516416	2.06697318
H	1.22474694	1.34528250	1.71246908
C	3.85035285	1.48421294	-0.01235487
H	4.60449158	0.92126145	-0.57608799
H	3.34117846	2.20172258	-0.66608155
H	4.35552639	2.08996940	0.75676981
H	-1.69377986	-1.49990859	-1.45984648

Time = 380.00 fs
E(CASSCF/6-31G(d)) = -479.4615716

C	-0.87698795	0.05785595	-0.19589480
C	0.52537126	-0.08262531	0.00820645
C	1.29247776	-0.93379798	-0.83679440
C	1.55919851	0.57014778	1.07481553
C	2.78232375	0.49485732	0.37857438
C	2.62813105	-0.62384041	-0.59381547
N	-1.88807893	0.15201062	0.74998988
C	-3.26983811	-0.20860813	0.07399545
H	3.40666372	-1.14618481	-1.15219275
C	-1.51769993	-0.42239088	-1.52060506
C	-2.96607033	0.08715789	-1.47500456
H	-3.04602721	1.14941955	-1.70362848
H	-3.61799072	-0.52891562	-2.07586121
H	-3.56506129	-1.26262806	0.30217141
H	-3.98150630	0.52228237	0.44499024
H	-0.94553177	-0.24631465	-2.42320638
H	0.95901929	-1.86022695	-1.24887631
C	-1.38490104	-0.49051780	1.98430010
H	-1.92477555	-0.18049143	2.89395057
H	-1.36733407	-1.59914833	1.88007760
H	-0.39464425	-0.05029199	2.08635129
H	1.21915614	1.34967068	1.70659583
C	3.84968605	1.48709955	-0.01052501
H	4.61667111	0.92423772	-0.55372847
H	3.34413614	2.20110342	-0.67351802
H	4.32903423	2.09870830	0.77004376

H	-1.69432204	-1.49883772	-1.45439184

Time = 380.50 fs
E(CASSCF/6-31G(d)) = -479.4609681

C	-0.87218230	0.05509192	-0.19068188
C	0.52496476	-0.08619267	0.00223995
C	1.28785523	-0.93765455	-0.83621165
C	1.56312437	0.57216767	1.06939848
C	2.78284206	0.49782458	0.37786899
C	2.62814334	-0.62214187	-0.59964941
N	-1.88781677	0.14964763	0.74743939
C	-3.27099749	-0.20379993	0.07302473
H	3.40197079	-1.14721984	-1.15390074
C	-1.51820974	-0.42365095	-1.51793632
C	-2.96012655	0.08908452	-1.47483540
H	-3.04490878	1.14932464	-1.70386119
H	-3.61627756	-0.52806135	-2.06778087
H	-3.57948825	-1.24843027	0.30241214
H	-3.96754189	0.54872289	0.43649294
H	-0.93804269	-0.25469498	-2.40983381
H	0.95413866	-1.87564690	-1.24134293
C	-1.39086843	-0.49554036	1.98955221
H	-1.93676943	-0.18874927	2.89748747
H	-1.34788811	-1.60344411	1.88038877
H	-0.41384230	-0.03765256	2.10682716
H	1.21390377	1.35554014	1.70149720
C	3.84908560	1.49006484	-0.00854397
H	4.62858691	0.92872146	-0.53106516
H	3.34822708	2.19970990	-0.68103790
H	4.30179958	2.10609961	0.78303445
H	-1.69318743	-1.49797229	-1.44893884

Time = 381.00 fs
E(CASSCF/6-31G(d)) = -479.4600713

C	-0.86710128	0.05200567	-0.18573071
C	0.52418809	-0.08959823	-0.00322247
C	1.28383486	-0.94140210	-0.83556390
C	1.56686799	0.57412678	1.06324924
C	2.78306113	0.50056676	0.37705815
C	2.62790932	-0.62059589	-0.60568427
N	-1.88775295	0.14722432	0.74492534
C	-3.27170068	-0.19867898	0.07207087
H	3.39771487	-1.14724452	-1.15416785
C	-1.51875054	-0.42499705	-1.51515174
C	-2.95411364	0.09088004	-1.47424252

H	-3.04430066	1.14866877	-1.70392404
H	-3.61486308	-0.52653111	-2.06063414
H	-3.59333090	-1.23200823	0.30191392
H	-3.95395083	0.57354783	0.42848003
H	-0.93060818	-0.26218152	-2.39664279
H	0.94893981	-1.89014591	-1.23509647
C	-1.39699986	-0.50040001	1.99483391
H	-1.94932042	-0.19804302	2.89934727
H	-1.32815897	-1.60562872	1.88138715
H	-0.43396285	-0.02716942	2.12825412
H	1.20922025	1.36250161	1.69685973
C	3.84853328	1.49311871	-0.00639063
H	4.64032684	0.93467093	-0.50820839
H	3.35350800	2.19746452	-0.68862711
H	4.27400875	2.11222757	0.79567873
H	-1.69047528	-1.49747905	-1.44346859

Time = 381.50 fs
E(CASSCF/6-31G(d)) = -479.4588649

C	-0.86181426	0.04859118	-0.18108524
C	0.52307141	-0.09281684	-0.00812324
C	1.28044695	-0.94509635	-0.83489495
C	1.57036671	0.57605976	1.05642504
C	2.78299674	0.50306388	0.37609217
C	2.62738548	-0.61918962	-0.61188602
N	-1.88787831	0.14472762	0.74250258
C	-3.27193304	-0.19318076	0.07112400
H	3.39419329	-1.14643240	-1.15321867
C	-1.51929255	-0.42643017	-1.51221476
C	-2.94806460	0.09253495	-1.47322621
H	-3.04411884	1.14759061	-1.70387207
H	-3.61373837	-0.52436239	-2.05454618
H	-3.60668075	-1.21377496	0.30081343
H	-3.94076972	0.59650838	0.42094676
H	-0.92308961	-0.26866415	-2.38409492
H	0.94364227	-1.90316389	-1.22991876
C	-1.40330201	-0.50507689	2.00011863
H	-1.96258906	-0.20848216	2.89952651
H	-1.30831774	-1.60572586	1.88309185
H	-0.45451342	-0.01868611	2.15049129
H	1.20536126	1.37008689	1.69231273
C	3.84800853	1.49626573	-0.00404412
H	4.65199938	0.94200819	-0.48526768
H	3.35999829	2.19434185	-0.69629086
H	4.24586214	2.11721652	0.80794731
H	-1.68630636	-1.49751607	-1.43796936

Time = 382.00 fs
E(CASSCF/6-31G(d)) = -479.4575136

C	-0.85639873	0.04484570	-0.17678227
C	0.52165413	-0.09582732	-0.01241154
C	1.27771389	-0.94878715	-0.83424546
C	1.57355529	0.57800479	1.04898823
C	2.78266929	0.50529709	0.37491929
C	2.62653272	-0.61790609	-0.61821757
N	-1.88817891	0.14214622	0.74022177
C	-3.27167910	-0.18723833	0.07017306
H	3.39172104	-1.14499593	-1.15130879
C	-1.51980109	-0.42795185	-1.50909630
C	-2.94201582	0.09403846	-1.47179130
H	-3.04426443	1.14624578	-1.70375958
H	-3.61286292	-0.52158305	-2.04959478
H	-3.61966024	-1.19425722	0.29927132
H	-3.92804246	0.61740331	0.41388976
H	-0.91538427	-0.27405287	-2.37258660
H	0.93845040	-1.91420346	-1.22561917
C	-1.40978186	-0.50955261	2.00537549
H	-1.97677455	-0.22013898	2.89810928
H	-1.28853606	-1.60386550	1.88550026
H	-0.47497944	-0.01197869	2.17340931
H	1.20258067	1.37780283	1.68747383
C	3.84749014	1.49950463	-0.00148407
H	4.66372510	0.95062728	-0.46234244
H	3.36768036	2.19036522	-0.70405380
H	4.21756571	2.12122784	0.81984103
H	-1.68081813	-1.49821910	-1.43243751

Time = 382.50 fs
E(CASSCF/6-31G(d)) = -479.4560330

C	-0.85093992	0.04076978	-0.17285092
C	0.51998508	-0.09861280	-0.01604371
C	1.27565078	-0.95251476	-0.83365031
C	1.57636850	0.57999937	1.04100178
C	2.78210262	0.50724860	0.37348696
C	2.62531868	-0.61672669	-0.62464115
N	-1.88863549	0.13946980	0.73812814
C	-3.27092257	-0.18078669	0.06920622
H	3.39060030	-1.14316254	-1.14870562
C	-1.52023612	-0.42956482	-1.50577725
C	-2.93600621	0.09537960	-1.46994728
H	-3.04462372	1.14479478	-1.70363783

H	-3.61216525	-0.51821079	-2.04580936
H	-3.63241502	-1.17406034	0.29746465
H	-3.91582720	0.63609663	0.40731262
H	-0.90744743	-0.27828354	-2.36241381
H	0.93354042	-1.92286849	-1.22205217
C	-1.41644454	-0.51380907	2.01057064
H	-1.99210620	-0.23305216	2.89525348
H	-1.26897919	-1.60027504	1.88858474
H	-0.49486740	-0.00677415	2.19689110
H	1.20110602	1.38518611	1.68198987
C	3.84695813	1.50282850	0.00130899
H	4.67562444	0.96040400	-0.43951384
H	3.37650257	2.18560405	-0.71195898
H	4.18932314	2.12445318	0.83138417
H	-1.67415844	-1.49968836	-1.42687798

Time = 383.00 fs
E(CASSCF/6-31G(d)) = -479.4545031

C	-0.84552919	0.03636731	-0.16931324
C	0.51812297	-0.10116162	-0.01898390
C	1.27426597	-0.95630657	-0.83313773
C	1.57874356	0.58207694	1.03252665
C	2.78132372	0.50890218	0.37174248
C	2.62372022	-0.61563296	-0.63112058
N	-1.88922475	0.13669006	0.73626225
C	-3.26964719	-0.17376841	0.06821181
H	3.39108807	-1.14115279	-1.14565969
C	-1.52055116	-0.43127362	-1.50225051
C	-2.93007676	0.09654861	-1.46770813
H	-3.04506955	1.14338917	-1.70355234
H	-3.61154672	-0.51425538	-2.04317415
H	-3.64510201	-1.15381941	0.29557439
H	-3.90420223	0.65253051	0.40122901
H	-0.89931001	-0.28132142	-2.35375087
H	0.92905201	-1.92889196	-1.21912260
C	-1.42329134	-0.51782743	2.01566849
H	-2.00883106	-0.24723085	2.89117472
H	-1.24980006	-1.59526817	1.89229529
H	-0.51374551	-0.00277442	2.22083301
H	1.20111940	1.39183765	1.67556448
C	3.84639490	1.50622510	0.00435293
H	4.68780359	0.97120549	-0.41683870
H	3.38638327	2.18016634	-0.72006249
H	4.16132772	2.12710501	0.84261592
H	-1.66648228	-1.50197849	-1.42130697

Time = 383.50 fs
E(CASSCF/6-31G(d)) = -479.4531038

C	-0.84026193	0.03164535	-0.16618450
C	0.51613501	-0.10346754	-0.02120455
C	1.27356087	-0.96017544	-0.83272831
C	1.58062262	0.58426462	1.02361943
C	2.78036163	0.51024324	0.36963418
C	2.62172676	-0.61460893	-0.63762362
N	-1.88991986	0.13380069	0.73465987
C	-3.26783835	-0.16613967	0.06717958
H	3.39336149	-1.13915412	-1.14238141
C	-1.52069373	-0.43308506	-1.49852132
C	-2.92426941	0.09753842	-1.46509215
H	-3.04546219	1.14215937	-1.70354050
H	-3.61088823	-0.50972282	-2.04163280
H	-3.65786943	-1.13413828	0.29377071
H	-3.89326736	0.66673463	0.39566466
H	-0.89108579	-0.28316298	-2.34664170
H	0.92508282	-1.93215153	-1.21678959
C	-1.43031793	-0.52158703	2.02063385
H	-2.02719714	-0.26266165	2.88612284
H	-1.23113447	-1.58922518	1.89656057
H	-0.53128521	0.00031797	2.24514128
H	1.20274245	1.39745076	1.66797932
C	3.84578657	1.50967773	0.00766355
H	4.70034113	0.98289978	-0.39434416
H	3.39721775	2.17418953	-0.72842742
H	4.13375491	2.12940678	0.85358014
H	-1.65794800	-1.50509127	-1.41575295

Time = 384.00 fs
E(CASSCF/6-31G(d)) = -479.4520270

C	-0.83523938	0.02661765	-0.16347553
C	0.51409705	-0.10552818	-0.02268531
C	1.27353036	-0.96411607	-0.83243478
C	1.58195364	0.58657984	1.01433729
C	2.77924693	0.51125823	0.36711435
C	2.61934470	-0.61364388	-0.64411884
N	-1.89069052	0.13079948	0.73335288
C	-3.26548716	-0.15788203	0.06610308
H	3.39748508	-1.13729901	-1.13902561
C	-1.52060662	-0.43500704	-1.49460919
C	-2.91862999	0.09834530	-1.46212376
H	-3.04565070	1.14120523	-1.70362962
H	-3.61005948	-0.50462431	-2.04109558

H	-3.67082174	-1.11553829	0.29219851
H	-3.88315024	0.67882034	0.39066172
H	-0.88297230	-0.28383383	-2.34101261
H	0.92168863	-1.93267472	-1.21506884
C	-1.43750823	-0.52506211	2.02543097
H	-2.04741834	-0.27930515	2.88035743
H	-1.21311193	-1.58257126	1.90128700
H	-0.54728155	0.00277357	2.26970820
H	1.20602622	1.40182495	1.65911208
C	3.84512442	1.51316386	0.01125149
H	4.71326691	0.99535460	-0.37204095
H	3.40887847	2.16783320	-0.73710879
H	4.10677607	2.13158074	0.86430648
H	-1.64872108	-1.50897139	-1.41026145

Time = 384.50 fs
E(CASSCF/6-31G(d)) = -479.4514348

C	-0.83055461	0.02129320	-0.16118643
C	0.51208726	-0.10734982	-0.02341710
C	1.27416191	-0.96811368	-0.83226102
C	1.58269565	0.58903474	1.00471609
C	2.77800821	0.51193737	0.36413520
C	2.61659433	-0.61273119	-0.65059142
N	-1.89150531	0.12768188	0.73236588
C	-3.26258850	-0.14898931	0.06497876
H	3.40339820	-1.13564691	-1.13567229
C	-1.52023026	-0.43705254	-1.49053737
C	-2.91319383	0.09897192	-1.45882677
H	-3.04547584	1.14058675	-1.70383568
H	-3.60892732	-0.49897103	-2.04144474
H	-3.68402105	-1.09835734	0.29095878
H	-3.87397887	0.68900702	0.38626577
H	-0.87521606	-0.28338833	-2.33667984
H	0.91887303	-1.93064289	-1.21402724
C	-1.44485045	-0.52823030	2.03003743
H	-2.06969212	-0.29713945	2.87410436
H	-1.19581576	-1.57573075	1.90637346
H	-0.56170953	0.00482176	2.29446002
H	1.21095230	1.40488580	1.64892528
C	3.84440394	1.51666430	0.01512961
H	4.72658106	1.00847004	-0.34987439
H	3.42124853	2.16125237	-0.74616097
H	4.08049377	2.13384203	0.87482447
H	-1.63895185	-1.51351783	-1.40488173

Time = 385.00 fs

E(CASSCF/6-31G(d)) = -479.4514310

C	-0.82630183	0.01568727	-0.15931327
C	0.51018877	-0.10894174	-0.02339704
C	1.27543735	-0.97213634	-0.83220330
C	1.58281652	0.59163038	0.99478922
C	2.77667264	0.51227217	0.36065532
C	2.61351466	-0.61187101	-0.65702860
N	-1.89233049	0.12444723	0.73171915
C	-3.25914857	-0.13948777	0.06380955
H	3.41090250	-1.13418391	-1.13233631
C	-1.51950563	-0.43923506	-1.48633877
C	-2.90799704	0.09942592	-1.45523013
H	-3.04477199	1.14032356	-1.70416269
H	-3.60736677	-0.49279008	-2.04254429
H	-3.69743490	-1.08275384	0.29010257
H	-3.86589771	0.69758294	0.38253484
H	-0.86810714	-0.28190243	-2.33339260
H	0.91659587	-1.92637860	-1.21378538
C	-1.45232248	-0.53106571	2.03443607
H	-2.09413067	-0.31613138	2.86753698
H	-1.17932809	-1.56910652	1.91170598
H	-0.57468886	0.00663331	2.31929595
H	1.21742643	1.40667886	1.63748706
C	3.84362630	1.52015705	0.01930487
H	4.74022196	1.02215387	-0.32777753
H	3.43420566	2.15459778	-0.75560680
H	4.05500109	2.13638517	0.88513070
H	-1.62879368	-1.51858334	-1.39967796

Time = 385.50 fs
E(CASSCF/6-31G(d)) = -479.4520158

C	-0.82257026	0.00981750	-0.15784592
C	0.50848585	-0.11031721	-0.02262929
C	1.27733302	-0.97613977	-0.83225079
C	1.58229551	0.59435847	0.98458003
C	2.77526363	0.51225667	0.35663910
C	2.61016055	-0.61106890	-0.66342371
N	-1.89313098	0.12109619	0.73142725
C	-3.25518533	-0.12942940	0.06260485
H	3.41967628	-1.13282945	-1.12897665
C	-1.51837743	-0.44156926	-1.48205025
C	-2.90307071	0.09972080	-1.45136538
H	-3.04337077	1.14039562	-1.70460297
H	-3.60526962	-0.48612494	-2.04424685
H	-3.71093300	-1.06868300	0.28962719

H	-3.85904965	0.70489823	0.37953104
H	-0.86194458	-0.27946902	-2.33086172
H	0.91477282	-1.92033038	-1.21451065
C	-1.45989960	-0.53354407	2.03862065
H	-2.12075081	-0.33625254	2.86074568
H	-1.16372294	-1.56303929	1.91716657
H	-0.58648192	0.00832044	2.34410295
H	1.22528280	1.40737168	1.62496333
C	3.84279731	1.52362137	0.02378061
H	4.75407689	1.03633017	-0.30565976
H	3.44763833	2.14799891	-0.76543207
H	4.03036085	2.13937867	0.89519154
H	-1.61839498	-1.52399012	-1.39472268

Time = 386.00 fs
E(CASSCF/6-31G(d)) = -479.4531405

C	-0.81944238	0.00370398	-0.15676807
C	0.50706148	-0.11149242	-0.02112366
C	1.27982072	-0.98007066	-0.83238669
C	1.58112454	0.59720192	0.97410081
C	2.77379844	0.51188769	0.35205814
C	2.60659963	-0.61033448	-0.66977489
N	-1.89387040	0.11762996	0.73149848
C	-3.25072946	-0.11888762	0.06138062
H	3.42929988	-1.13145338	-1.12551562
C	-1.51679745	-0.44406993	-1.47770989
C	-2.89843924	0.09987544	-1.44726593
H	-3.04110497	1.14074845	-1.70513777
H	-3.60255200	-0.47903788	-2.04640027
H	-3.72428769	-1.05591840	0.28948160
H	-3.85356212	0.71134052	0.37731427
H	-0.85700857	-0.27619205	-2.32879093
H	0.91328174	-1.91304342	-1.21641068
C	-1.46755743	-0.53564553	2.04259686
H	-2.14945502	-0.35747887	2.85372190
H	-1.14907546	-1.55777765	1.92263784
H	-0.59746419	0.00994319	2.36875506
H	1.23429140	1.40724651	1.61161264
C	3.84192673	1.52703922	0.02855636
H	4.76798770	1.05093642	-0.28341489
H	3.46145104	2.14155409	-0.77557519
H	4.00660791	2.14295983	0.90494047
H	-1.60789764	-1.52954519	-1.39009374

Time = 386.50 fs
E(CASSCF/6-31G(d)) = -479.4548668

C	-0.81699427	-0.00262929	-0.15605647
C	0.50599850	-0.11248468	-0.01889216
C	1.28287034	-0.98387138	-0.83258954
C	1.57931111	0.60013574	0.96334954
C	2.77228198	0.51116536	0.34689382
C	2.60290576	-0.60967981	-0.67608366
N	-1.89450974	0.11404856	0.73193273
C	-3.24582529	-0.10795031	0.06015862
H	3.43929311	-1.12990071	-1.12186342
C	-1.51472740	-0.44675110	-1.47335534
C	-2.89411914	0.09991337	-1.44296662
H	-3.03781486	1.14130263	-1.70574054
H	-3.59916165	-0.47161237	-2.04885487
H	-3.73718849	-1.04409964	0.28958087
H	-3.84953289	0.71730597	0.37593658
H	-0.85353503	-0.27218169	-2.32690267
H	0.91197316	-1.90511760	-1.21972648
C	-1.47527447	-0.53735752	2.04638376
H	-2.18002053	-0.37978447	2.84635552
H	-1.13547332	-1.55345699	1.92800621
H	-0.60808505	0.01151967	2.39311464
H	1.24416983	1.40668842	1.59777894
C	3.84102733	1.53039580	0.03362799
H	4.78176139	1.06591792	-0.26093171
H	3.47556433	2.13532379	-0.78592043
H	3.98375445	2.14723303	0.91428178
H	-1.59743486	-1.53505777	-1.38587274

Time = 387.00 fs
E(CASSCF/6-31G(d)) = -479.4576156

C	-0.81525426	-0.00916858	-0.15568803
C	0.50533649	-0.11331813	-0.01597525
C	1.28640256	-0.98747853	-0.83284299
C	1.57685654	0.60312561	0.95233910
C	2.77075335	0.51009178	0.34112967
C	2.59920701	-0.60910833	-0.68234773
N	-1.89502830	0.11037077	0.73274040
C	-3.24052288	-0.09671468	0.05897039
H	3.44915310	-1.12801530	-1.11791784
C	-1.51213931	-0.44963138	-1.46901805
C	-2.89011784	0.09986113	-1.43850328
H	-3.03335319	1.14196747	-1.70637917
H	-3.59508699	-0.46395260	-2.05147508
H	-3.74929204	-1.03279444	0.28979792
H	-3.84702171	0.72316518	0.37541950

H	-0.85170391	-0.26754301	-2.32498452
H	0.91069917	-1.89717863	-1.22462487
C	-1.48303770	-0.53867909	2.04999879
H	-2.21209887	-0.40314180	2.83846622
H	-1.12300731	-1.55011617	1.93318494
H	-0.61883921	0.01305209	2.41705678
H	1.25459683	1.40612558	1.58387636
C	3.84010798	1.53368388	0.03898079
H	4.79517240	1.08121394	-0.23809736
H	3.48992904	2.12935167	-0.79630954
H	3.96178117	2.15225602	0.92309242
H	-1.58715857	-1.54035054	-1.38216360

Time = 387.50 fs
E(CASSCF/6-31G(d)) = -479.4607670

C	-0.81423360	-0.01590700	-0.15562989
C	0.50510959	-0.11401526	-0.01242268
C	1.29032005	-0.99083363	-0.83312885
C	1.57375304	0.60613285	0.94108268
C	2.76926297	0.50866870	0.33475111
C	2.59564078	-0.60861926	-0.68855920
N	-1.89540669	0.10661899	0.73392523
C	-3.23488175	-0.08527687	0.05785364
H	3.45839642	-1.12565286	-1.11361755
C	-1.50901861	-0.45272362	-1.46472694
C	-2.88643385	0.09974717	-1.43391178
H	-3.02758825	1.14264722	-1.70702039
H	-3.59035115	-0.45618002	-2.05413830
H	-3.76023946	-1.02155843	0.28999404
H	-3.84602697	0.72923094	0.37575218
H	-0.85162377	-0.26237809	-2.32287622
H	0.90932265	-1.88978591	-1.23126340
C	-1.49084033	-0.53962084	2.05346473
H	-2.24523683	-0.42750540	2.82980726
H	-1.11177999	-1.54768855	1.93810322
H	-0.63022167	0.01452997	2.44046514
H	1.26523222	1.40603532	1.57032811
C	3.83917609	1.53690026	0.04459469
H	4.80798755	1.09675870	-0.21480893
H	3.50451534	2.12365850	-0.80653827
H	3.94065087	2.15805531	0.93123420
H	-1.57720184	-1.54527474	-1.37904916

Time = 388.00 fs
E(CASSCF/6-31G(d)) = -479.4639334

C	-0.81394906	-0.02282639	-0.15584118
C	0.50535657	-0.11460065	-0.00827538
C	1.29455495	-0.99389285	-0.83342515
C	1.57003170	0.60912472	0.92957337
C	2.76781281	0.50690597	0.32776177
C	2.59230402	-0.60821666	-0.69471566
N	-1.89561462	0.10280244	0.73547458
C	-3.22897373	-0.07372293	0.05684731
H	3.46659778	-1.12271175	-1.10895443
C	-1.50536728	-0.45603184	-1.46050870
C	-2.88305561	0.09960108	-1.42922765
H	-3.02040773	1.14324898	-1.70763015
H	-3.58501239	-0.44842983	-2.05673179
H	-3.76966809	-1.00997912	0.29005398
H	-3.84646056	0.73573850	0.37689824
H	-0.85331433	-0.25678494	-2.32047186
H	0.90773918	-1.88336371	-1.23979273
C	-1.49868220	-0.54020502	2.05681536
H	-2.27890407	-0.45279898	2.82008698
H	-1.10191682	-1.54599368	1.94269117
H	-0.64267797	0.01594553	2.46322645
H	1.27574093	1.40690286	1.55754476
C	3.83824249	1.54004341	0.05045111
H	4.81999051	1.11248385	-0.19097788
H	3.51929530	2.11821815	-0.81635828
H	3.92031815	2.16463858	0.93856372
H	-1.56766716	-1.54972614	-1.37659678

Time = 388.50 fs
E(CASSCF/6-31G(d)) = -479.4672609

C	-0.81439828	-0.02990541	-0.15627746
C	0.50609815	-0.11510039	-0.00357766
C	1.29903819	-0.99662999	-0.83371545
C	1.56573964	0.61207570	0.91780450
C	2.76639011	0.50481954	0.32017618
C	2.58928355	-0.60790164	-0.70081471
N	-1.89562417	0.09892983	0.73737127
C	-3.22287705	-0.06212615	0.05599354
H	3.47341741	-1.11914366	-1.10396974
C	-1.50120262	-0.45955585	-1.45638536
C	-2.87996178	0.09945211	-1.42448574
H	-3.01172370	1.14369373	-1.70817800
H	-3.57916434	-0.44084744	-2.05915936
H	-3.77725329	-0.99771660	0.28988287
H	-3.84815017	0.74283116	0.37878469
H	-0.85670849	-0.25085148	-2.31773691

H	0.90590596	-1.87816271	-1.25028334
C	-1.50657106	-0.54046565	2.06008457
H	-2.31252389	-0.47891293	2.80901517
H	-1.09355023	-1.54478050	1.94689243
H	-0.65658748	0.01730734	2.48524831
H	1.28581994	1.40914296	1.54588101
C	3.83731697	1.54311463	0.05652952
H	4.83098786	1.12830960	-0.16653389
H	3.53424478	2.11298177	-0.82549889
H	3.90072689	2.17199103	0.94494261
H	-1.55864310	-1.55365122	-1.37487026

Time = 389.00 fs
E(CASSCF/6-31G(d)) = -479.4709068

C	-0.81555619	-0.03712200	-0.15688995
C	0.50733411	-0.11554151	0.00162032
C	1.30369120	-0.99903851	-0.83399178
C	1.56092936	0.61497140	0.90577758
C	2.76497807	0.50243013	0.31201716
C	2.58666112	-0.60767000	-0.70685176
N	-1.89541153	0.09501143	0.73959465
C	-3.21667335	-0.05054472	0.05533698
H	3.47862041	-1.11495912	-1.09875712
C	-1.49655672	-0.46329205	-1.45237409
C	-2.87712198	0.09932798	-1.41971977
H	-3.00147524	1.14392257	-1.70863975
H	-3.57293135	-0.43358211	-2.06134184
H	-3.78273163	-0.98452791	0.28941104
H	-3.85084336	0.75055906	0.38130105
H	-0.86165629	-0.24465132	-2.31470913
H	0.90385900	-1.87423166	-1.26270105
C	-1.51452168	-0.54044666	2.06330214
H	-2.34550984	-0.50570165	2.79633665
H	-1.08680631	-1.54376172	1.95066382
H	-0.67224880	0.01864687	2.50646819
H	1.29522088	1.41303935	1.53558653
C	3.83640678	1.54611719	0.06280711
H	4.84082015	1.14414127	-0.14142730
H	3.54934014	2.10789391	-0.83368725
H	3.88181119	2.18007951	0.95024824
H	-1.55020500	-1.55704973	-1.37392859

Time = 389.50 fs
E(CASSCF/6-31G(d)) = -479.4749379

C	-0.81737621	-0.04445403	-0.15762489

C	0.50904404	-0.11595209	0.00726219
C	1.30842575	-1.00113121	-0.83425547
C	1.55565546	0.61781270	0.89350639
C	2.76355832	0.49976312	0.30331488
C	2.58451179	-0.60751213	-0.71281961
N	-1.89495702	0.09105911	0.74212055
C	-3.21044487	-0.03902133	0.05492352
H	3.48209078	-1.11022991	-1.09346304
C	-1.49147559	-0.46723361	-1.44848782
C	-2.87449795	0.09925391	-1.41496213
H	-2.98962993	1.14390053	-1.70899966
H	-3.56646183	-0.42678032	-2.06321559
H	-3.78591576	-0.97027922	0.28859887
H	-3.85421920	0.75889094	0.38430520
H	-0.86793311	-0.23824179	-2.31149312
H	0.90171699	-1.87141880	-1.27690021
C	-1.52255449	-0.54019933	2.06649119
H	-2.37730155	-0.53298526	2.78185603
H	-1.08179137	-1.54264339	1.95397386
H	-0.68987117	0.02002199	2.52685964
H	1.30376334	1.41869866	1.52676762
C	3.83551611	1.54905505	0.06925968
H	4.84937070	1.15986910	-0.11562964
H	3.56455449	2.10290312	-0.84066746
H	3.86349668	2.18885772	0.95438244
H	-1.54241341	-1.55997460	-1.37382409

Time = 390.00 fs
E(CASSCF/6-31G(d)) = -479.4793233

C	-0.81979227	-0.05187931	-0.15842362
C	0.51118856	-0.11636176	0.01328557
C	1.31314704	-1.00293719	-0.83451634
C	1.54997413	0.62061778	0.88101869
C	2.76211167	0.49684872	0.29410650
C	2.58290076	-0.60741359	-0.71870876
N	-1.89424498	0.08708610	0.74492130
C	-3.20427154	-0.02758411	0.05479854
H	3.48383916	-1.10508845	-1.08828352
C	-1.48601754	-0.47137081	-1.44473612
C	-2.87204503	0.09925191	-1.41024365
H	-2.97618457	1.14361755	-1.70925212
H	-3.55991997	-0.42057840	-2.06472952
H	-3.78670453	-0.95495018	0.28743950
H	-3.85790795	0.76773537	0.38763346
H	-0.87525197	-0.23166355	-2.30825001
H	0.89967097	-1.86940355	-1.29263126

C	-1.53069426	-0.53977977	2.06966675
H	-2.40739572	-0.56055456	2.76545491
H	-1.07857934	-1.54115125	1.95680155
H	-0.70957018	0.02151819	2.54643663
H	1.31133714	1.42602762	1.51936826
C	3.83464579	1.55193172	0.07586260
H	4.85657197	1.17536890	-0.08913274
H	3.57985493	2.09797032	-0.84621855
H	3.84570159	2.19827117	0.95727878
H	-1.53531285	-1.56252746	-1.37460130

Time = 390.50 fs
E(CASSCF/6-31G(d)) = -479.4839193

C	-0.82272174	-0.05937572	-0.15922290
C	0.51371144	-0.11680203	0.01962242
C	1.31775778	-1.00449695	-0.83479099
C	1.54394338	0.62342173	0.86835602
C	2.76061888	0.49372209	0.28443569
C	2.58188079	-0.60735605	-0.72450801
N	-1.89326365	0.08310681	0.74796590
C	-3.19822779	-0.01624852	0.05500511
H	3.48400441	-1.09972422	-1.08345861
C	-1.48025099	-0.47569169	-1.44112655
C	-2.86971400	0.09934031	-1.40559351
H	-2.96116488	1.14308715	-1.70940256
H	-3.55347636	-0.41509521	-2.06584098
H	-3.78508927	-0.93863293	0.28596006
H	-3.86151556	0.77696644	0.39111312
H	-0.88328040	-0.22494199	-2.30518189
H	0.89796217	-1.86775011	-1.30956122
C	-1.53896861	-0.53924624	2.07283484
H	-2.43537193	-0.58817936	2.74710105
H	-1.07720038	-1.53905130	1.95913566
H	-0.73136952	0.02324760	2.56525658
H	1.31789344	1.43474157	1.51317554
C	3.83379324	1.55474928	0.08259175
H	4.86240846	1.19050447	-0.06194631
H	3.59520192	2.09307502	-0.85017007
H	3.82833728	2.20826140	0.95890712
H	-1.52893128	-1.56485121	-1.37629682

Time = 391.00 fs
E(CASSCF/6-31G(d)) = -479.4885200

C	-0.82606847	-0.06692102	-0.15995582
C	0.51654176	-0.11730690	0.02619948

C	1.32216332	-1.00585666	-0.83510023
C	1.53762423	0.62627365	0.85557138
C	2.75906112	0.49042336	0.27435216
C	2.58148969	-0.60731859	-0.73020522
N	-1.89200521	0.07913673	0.75122040
C	-3.19237951	-0.00501951	0.05558209
H	3.48284793	-1.09437686	-1.07926407
C	-1.47425178	-0.48018262	-1.43766628
C	-2.86745293	0.09953350	-1.40103917
H	-2.94462465	1.14234267	-1.70946733
H	-3.54729797	-0.41042492	-2.06651088
H	-3.78115681	-0.92152745	0.28422148
H	-3.86464912	0.78644914	0.39457526
H	-0.89166142	-0.21809088	-2.30250977
H	0.89685338	-1.86597010	-1.32730205
C	-1.54740655	-0.53865675	2.07599237
H	-2.46091223	-0.61561789	2.72685186
H	-1.07763221	-1.53616539	1.96097461
H	-0.75520689	0.02534576	2.58342089
H	1.32342770	1.44440203	1.50784862
C	3.83295268	1.55750761	0.08942430
H	4.86691636	1.20513089	-0.03409519
H	3.61054960	2.08821911	-0.85241393
H	3.81130957	2.21876910	0.95927587
H	-1.52327999	-1.56711845	-1.37893903

Time = 391.50 fs
E(CASSCF/6-31G(d)) = -479.4929069

C	-0.82972652	-0.07449324	-0.16055286
C	0.51959696	-0.11791304	0.03293899
C	1.32627632	-1.00706244	-0.83546629
C	1.53108171	0.62923208	0.84272559
C	2.75742061	0.48699756	0.26391100
C	2.58174886	-0.60727929	-0.73578819
N	-1.89046568	0.07519230	0.75464841
C	-3.18678134	0.00610631	0.05656244
H	3.48073968	-1.08932565	-1.07600067
C	-1.46810009	-0.48482927	-1.43436399
C	-2.86520910	0.09984187	-1.39660633
H	-2.92664418	1.14143215	-1.70947306
H	-3.54153880	-0.40663080	-2.06669880
H	-3.77508956	-0.90393395	0.28231733
H	-3.86694124	0.79606146	0.39786703
H	-0.90003726	-0.21111833	-2.30044700
H	0.89659914	-1.86358254	-1.34544316
C	-1.55603670	-0.53806664	2.07912748

H	-2.48381612	-0.64262810	2.70485158
H	-1.07979349	-1.53238069	1.96232654
H	-0.78094658	0.02796567	2.60107272
H	1.32796065	1.45447305	1.50296337
C	3.83211540	1.56020392	0.09633940
H	4.87017955	1.21909902	-0.00561504
H	3.62584830	2.08342816	-0.85291285
H	3.79451900	2.22973643	0.95843123
H	-1.51835418	-1.56951718	-1.38254889

Time = 392.00 fs
E(CASSCF/6-31G(d)) = -479.4969010

C	-0.83358373	-0.08207084	-0.16094339
C	0.52278574	-0.11865971	0.03975985
C	1.33002142	-1.00815586	-0.83591041
C	1.52438544	0.63235967	0.82988321
C	2.75568141	0.48349439	0.25317188
C	2.58266281	-0.60721689	-0.74124562
N	-1.88864487	0.07129092	0.75821164
C	-3.18147465	0.01713860	0.05797181
H	3.47813454	-1.08487381	-1.07398094
C	-1.46187719	-0.48961702	-1.43123186
C	-2.86293078	0.10027204	-1.39231891
H	-2.90732890	1.14041134	-1.70945492
H	-3.53633162	-0.40374084	-2.06635880
H	-3.76716175	-0.88624203	0.28037111
H	-3.86807064	0.80571061	0.40086160
H	-0.90807255	-0.20403562	-2.29917124
H	0.89741819	-1.86016397	-1.36358273
C	-1.56488545	-0.53752643	2.08222068
H	-2.50400948	-0.66897796	2.68132547
H	-1.08354135	-1.52765503	1.96320934
H	-0.80839555	0.03126808	2.61838975
H	1.33152159	1.46438477	1.49806274
C	3.83127010	1.56283243	0.10331875
H	4.87232094	1.23226032	0.02345070
H	3.64104712	2.07875062	-0.85170363
H	3.77786284	2.24110706	0.95645417
H	-1.51413378	-1.57223481	-1.38713893

Time = 392.50 fs
E(CASSCF/6-31G(d)) = -479.5004020

C	-0.83752528	-0.08963311	-0.16105723
C	0.52601127	-0.11958887	0.04657861
C	1.33333849	-1.00917041	-0.83645049

C	1.51760985	0.63571836	0.81710830
C	2.75382987	0.47996797	0.24219822
C	2.58422015	-0.60711320	-0.74656847
N	-1.88654633	0.06745088	0.76187061
C	-3.17648626	0.02809121	0.05982759
H	3.47553461	-1.08132741	-1.07351211
C	-1.45566242	-0.49453191	-1.42828733
C	-2.86056884	0.10082728	-1.38819897
H	-2.88680724	1.13933699	-1.70945464
H	-3.53178173	-0.40174457	-2.06543665
H	-3.75773046	-0.86891277	0.27853160
H	-3.86777788	0.81534419	0.40346583
H	-0.91547590	-0.19686590	-2.29879663
H	0.89947446	-1.85538382	-1.38135376
C	-1.57397476	-0.53707948	2.08524635
H	-2.52155064	-0.69445730	2.65656950
H	-1.08866962	-1.52201769	1.96365145
H	-0.83732682	0.03540910	2.63557381
H	1.33413565	1.47359215	1.49270397
C	3.83040373	1.56538448	0.11034699
H	4.87349201	1.24447285	0.05305588
H	3.65609875	2.07425486	-0.84889779
H	3.76123569	2.25282600	0.95345472
H	-1.51058483	-1.57544112	-1.39271459

Time = 393.00 fs
E(CASSCF/6-31G(d)) = -479.5033929

C	-0.84143706	-0.09716062	-0.16082669
C	0.52917422	-0.12074445	0.05331101
C	1.33618543	-1.01012945	-0.83709966
C	1.51083317	0.63936507	0.80446112
C	2.75185540	0.47647622	0.23105621
C	2.58639592	-0.60695536	-0.75175110
N	-1.88417724	0.06369126	0.76558538
C	-3.17182819	0.03897970	0.06213842
H	3.47344048	-1.07896881	-1.07487724
C	-1.44953061	-0.49956096	-1.42555415
C	-2.85807790	0.10150805	-1.38426643
H	-2.86522850	1.13825938	-1.70951786
H	-3.52796308	-0.40059292	-2.06386917
H	-3.74721943	-0.85245322	0.27696550
H	-3.86587645	0.82495587	0.40562498
H	-0.92201552	-0.18965345	-2.29935135
H	0.90286202	-1.84902726	-1.39844560
C	-1.58331989	-0.53675990	2.08817491
H	-2.53663029	-0.71888779	2.63093835

H	-1.09491127	-1.51556702	1.96369223
H	-0.86750451	0.04052398	2.65283482
H	1.33581739	1.48162180	1.48649721
C	3.82950219	1.56784870	0.11741196
H	4.87385854	1.25560718	0.08315459
H	3.67096327	2.07002518	-0.84467685
H	3.74453217	2.26483763	0.94956519
H	-1.50765999	-1.57927106	-1.39927284

Time = 393.50 fs
E(CASSCF/6-31G(d)) = -479.5059276

C	-0.84520872	-0.10463617	-0.16018840
C	0.53217639	-0.12217144	0.05987355
C	1.33853915	-1.01104497	-0.83786500
C	1.50413579	0.64334834	0.79199521
C	2.74975094	0.47308034	0.21981372
C	2.58915546	-0.60673830	-0.75679279
N	-1.88154828	0.06003193	0.76931638
C	-3.16749876	0.04981837	0.06490405
H	3.47229520	-1.07802782	-1.07831289
C	-1.44355021	-0.50469268	-1.42306254
C	-2.85541710	0.10231260	-1.38053895
H	-2.84276188	1.13721532	-1.70969033
H	-3.52491672	-0.40019943	-2.06158502
H	-3.73609497	-0.83738028	0.27584771
H	-3.86225890	0.83458683	0.40732508
H	-0.92752667	-0.18247043	-2.30076746
H	0.90759780	-1.84100508	-1.41462164
C	-1.59292739	-0.53659062	2.09097548
H	-2.54956584	-0.74213074	2.60483125
H	-1.10194462	-1.50846424	1.96338300
H	-0.89870920	0.04670842	2.67036928
H	1.33656874	1.48810630	1.47913456
C	3.82855122	1.57021157	0.12450480
H	4.87358491	1.26555209	0.11370218
H	3.68561246	2.06615640	-0.83928304
H	3.72765064	2.27708237	0.94493174
H	-1.50530125	-1.58381075	-1.40680119

Time = 394.00 fs
E(CASSCF/6-31G(d)) = -479.5080981

C	-0.84873584	-0.11204514	-0.15908562
C	0.53492328	-0.12391506	0.06618499
C	1.34039619	-1.01191735	-0.83874682
C	1.49759866	0.64770585	0.77975525

C	2.74751321	0.46984419	0.20853944
C	2.59245865	-0.60646716	-0.76169907
N	-1.87867356	0.05649353	0.77302525
C	-3.16348454	0.06061654	0.06811595
H	3.47242771	-1.07865172	-1.08398889
C	-1.43778219	-0.50991749	-1.42084813
C	-2.85255057	0.10323767	-1.37703150
H	-2.81959434	1.13622344	-1.71001529
H	-3.52265212	-0.40044436	-2.05850835
H	-3.72483412	-0.82417386	0.27534859
H	-3.85689963	0.84432502	0.40859321
H	-0.93191143	-0.17541929	-2.30288098
H	0.91361841	-1.83135766	-1.42973040
C	-1.60279348	-0.53658203	2.09361894
H	-2.56078853	-0.76409440	2.57867392
H	-1.10940177	-1.50092257	1.96278826
H	-0.93076246	0.05400005	2.68833831
H	1.33637921	1.49280638	1.47040919
C	3.82753736	1.57245816	0.13161979
H	4.87281759	1.27422014	0.14465691
H	3.70003414	2.06274628	-0.83300571
H	3.71049621	2.28949297	0.93970534
H	-1.50344114	-1.58908723	-1.41527671

Time = 394.50 fs
E(CASSCF/6-31G(d)) = -479.5100160

C	-0.85192312	-0.11937653	-0.15747019
C	0.53732786	-0.12601887	0.07216899
C	1.34177173	-1.01273579	-0.83973864
C	1.49130047	0.65246216	0.76777483
C	2.74514313	0.46683321	0.19730135
C	2.59626442	-0.60615846	-0.76648205
N	-1.87557004	0.05309699	0.77667556
C	-3.15976309	0.07137449	0.07175826
H	3.47400754	-1.08088267	-1.09199146
C	-1.43227950	-0.51522775	-1.41894965
C	-2.84944767	0.10427910	-1.37375594
H	-2.79592892	1.13528183	-1.71053062
H	-3.52115076	-0.40118068	-2.05456390
H	-3.71388676	-0.81322184	0.27562054
H	-3.84985234	0.85430069	0.40949621
H	-0.93513051	-0.16863110	-2.30544584
H	0.92078126	-1.82025091	-1.44371364
C	-1.61290372	-0.53673132	2.09608079
H	-2.57082014	-0.78473607	2.55289647
H	-1.11688275	-1.49319049	1.96198692

H	-0.96354473	0.06236206	2.70684450
H	1.33522942	1.49562537	1.46022801
C	3.82644864	1.57457315	0.13875397
H	4.87166957	1.28155224	0.17597957
H	3.71423556	2.05988714	-0.82616249
H	3.69298386	2.30198924	0.93403260
H	-1.50200489	-1.59506422	-1.42466407

Time = 395.00 fs
E(CASSCF/6-31G(d)) = -479.5117441

C	-0.85468516	-0.12662285	-0.15530459
C	0.53931216	-0.12852393	0.07775495
C	1.34269858	-1.01347935	-0.84082708
C	1.48531628	0.65762730	0.75607532
C	2.74264539	0.46411392	0.18616629
C	2.60053333	-0.60584102	-0.77116118
N	-1.87225763	0.04986359	0.78023373
C	-3.15630621	0.08207920	0.07580903
H	3.47701959	-1.08464364	-1.10231345
C	-1.42708721	-0.52061792	-1.41740610
C	-2.84608289	0.10543240	-1.37072058
H	-2.77198242	1.13436782	-1.71126710
H	-3.52037105	-0.40224117	-2.04968356
H	-3.70363976	-0.80476617	0.27678417
H	-3.84124565	0.86468165	0.41013683
H	-0.93719017	-0.16225860	-2.30815691
H	0.92887030	-1.80796866	-1.45660774
C	-1.62323376	-0.53702355	2.09834406
H	-2.58023999	-0.80406168	2.52791141
H	-1.12397225	-1.48553167	1.96107463
H	-0.99700232	0.07167418	2.72591586
H	1.33309234	1.49661487	1.44861762
C	3.82527524	1.57654194	0.14590634
H	4.87020835	1.28752067	0.20763193
H	3.72824613	2.05765656	-0.81907757
H	3.67504280	2.31447516	0.92804814
H	-1.50091224	-1.60164412	-1.43491453

Time = 395.50 fs
E(CASSCF/6-31G(d)) = -479.5132916

C	-0.85694779	-0.13378103	-0.15256323
C	0.54081012	-0.13146723	0.08287934
C	1.34322429	-1.01411875	-0.84199197
C	1.47971476	0.66319549	0.74466545
C	2.74002890	0.46175293	0.17519849

C	2.60522906	-0.60555449	-0.77576266
N	-1.86875903	0.04681484	0.78366963
C	-3.15308273	0.09270177	0.08024159
H	3.48126706	-1.08973882	-1.11485275
C	-1.42224309	-0.52608459	-1.41625341
C	-2.84243564	0.10669305	-1.36792960
H	-2.74798157	1.13344043	-1.71224734
H	-3.52025524	-0.40344638	-2.04381313
H	-3.69438654	-0.79885941	0.27891841
H	-3.83127432	0.87566431	0.41064749
H	-0.93812811	-0.15646565	-2.31067997
H	0.93760562	-1.79489570	-1.46854609
C	-1.63375162	-0.53743387	2.10040136
H	-2.58964133	-0.82212105	2.50408850
H	-1.13025999	-1.47819764	1.96016504
H	-1.03114547	0.08173175	2.74549844
H	1.32993638	1.49597473	1.43572695
C	3.82400987	1.57835177	0.15307663
H	4.86844781	1.29213088	0.23957538
H	3.74211898	2.05610566	-0.81205696
H	3.65661971	2.32683717	0.92186840
H	-1.50007885	-1.60867533	-1.44596436

Time = 396.00 fs
E(CASSCF/6-31G(d)) = -479.5146308

C	-0.85864931	-0.14085239	-0.14923407
C	0.54176969	-0.13487993	0.08748769
C	1.34340863	-1.01461860	-0.84320804
C	1.47455651	0.66914413	0.73353997
C	2.73730597	0.45981588	0.16445899
C	2.61031757	-0.60534751	-0.78031797
N	-1.86509920	0.04397194	0.78695730
C	-3.15006110	0.10319651	0.08502556
H	3.48640036	-1.09587048	-1.12942007
C	-1.41777796	-0.53162582	-1.41552086
C	-2.83849002	0.10805664	-1.36538278
H	-2.72416091	1.13244574	-1.71348519
H	-3.52073523	-0.40461301	-2.03691862
H	-3.68631238	-0.79534211	0.28205593
H	-3.82018344	0.88746085	0.41118228
H	-0.93799745	-0.15141605	-2.31268204
H	0.94665887	-1.78149389	-1.47974422
C	-1.64442150	-0.53793151	2.10225606
H	-2.59957858	-0.83899350	2.48173313
H	-1.13536679	-1.47140082	1.95939289
H	-1.06603237	0.09225469	2.76545486

H	1.32572763	1.49404936	1.42182393
C	3.82264764	1.57999295	0.16026416
H	4.86634650	1.29542059	0.27176665
H	3.75592956	2.05525238	-0.80536322
H	3.63768257	2.33894175	0.91558769
H	-1.49942031	-1.61596630	-1.45773461

Time = 396.50 fs
E(CASSCF/6-31G(d)) = -479.5156887

C	-0.85974118	-0.14784298	-0.14531941
C	0.54215460	-0.13878570	0.09153567
C	1.34331995	-1.01494085	-0.84444702
C	1.46989227	0.67543392	0.72267981
C	2.73449190	0.45836612	0.15400439
C	2.61576524	-0.60527394	-0.78486159
N	-1.86130491	0.04135553	0.79007574
C	-3.14721089	0.11350290	0.09012792
H	3.49196553	-1.10266691	-1.14575484
C	-1.41371626	-0.53724060	-1.41522826
C	-2.83423458	0.10951891	-1.36307529
H	-2.70075898	1.13132381	-1.71498606
H	-3.52173875	-0.40556186	-2.02899152
H	-3.67949550	-0.79384798	0.28618473
H	-3.80824807	0.90028195	0.41190618
H	-0.93685394	-0.14726166	-2.31385946
H	0.95567417	-1.76826873	-1.49047878
C	-1.65520817	-0.53848473	2.10392212
H	-2.61051322	-0.85477075	2.46106803
H	-1.13897035	-1.46528732	1.95891579
H	-1.10174750	0.10290542	2.78557391
H	1.32043216	1.49131314	1.40728552
C	3.82118575	1.58145991	0.16746660
H	4.86381161	1.29745729	0.30415500
H	3.76977263	2.05507522	-0.79919309
H	3.61822317	2.35063611	0.90927682
H	-1.49885606	-1.62330205	-1.47013174

Time = 397.00 fs
E(CASSCF/6-31G(d)) = -479.5164021

C	-0.86018823	-0.15476332	-0.14083611
C	0.54194547	-0.14319978	0.09498955
C	1.34303116	-1.01504874	-0.84568035
C	1.46576168	0.68201012	0.71205393
C	2.73160468	0.45746366	0.14388608
C	2.62153602	-0.60538906	-0.78942875

N	-1.85740457	0.03898529	0.79300920
C	-3.14450339	0.12355035	0.09551373
H	3.49746120	-1.10971469	-1.16354636
C	-1.41007653	-0.54292821	-1.41538367
C	-2.82966212	0.11107552	-1.36099743
H	-2.67801288	1.13001645	-1.71674834
H	-3.52319514	-0.40612493	-2.02005269
H	-3.67392336	-0.79383758	0.29125534
H	-3.79574987	0.91431509	0.41298164
H	-0.93474629	-0.14413113	-2.31396104
H	0.96429354	-1.75572653	-1.50105702
C	-1.66608099	-0.53906601	2.10542308
H	-2.62276656	-0.86954076	2.44222221
H	-1.14082764	-1.45991391	1.95891406
H	-1.13837878	0.11331362	2.80559016
H	1.31401854	1.48834356	1.39257634
C	3.81962303	1.58275169	0.17467864
H	4.86070814	1.29833435	0.33668104
H	3.78375779	2.05550835	-0.79366117
H	3.59825839	2.36175267	0.90298415
H	-1.49831159	-1.63046261	-1.48304897

Time = 397.50 fs
E(CASSCF/6-31G(d)) = -479.5167606

C	-0.85996901	-0.16162819	-0.13581539
C	0.54114055	-0.14812796	0.09782663
C	1.34261567	-1.01491142	-0.84688306
C	1.46219222	0.68880567	0.70162214
C	2.72866441	0.45716368	0.13414926
C	2.62758895	-0.60574534	-0.79405261
N	-1.85342768	0.03687953	0.79574762
C	-3.14191195	0.13326489	0.10114688
H	3.50239360	-1.11659327	-1.18245706
C	-1.40687182	-0.54868747	-1.41598206
C	-2.82476958	0.11272185	-1.35913485
H	-2.65615317	1.12847531	-1.71876503
H	-3.52503974	-0.40615148	-2.01015431
H	-3.66951866	-0.79465148	0.29719145
H	-3.78294972	0.92969910	0.41455433
H	-0.93170909	-0.14212104	-2.31280483
H	0.97218583	-1.74432562	-1.51177387
C	-1.67701711	-0.53965643	2.10678999
H	-2.63648740	-0.88337183	2.42522924
H	-1.14079347	-1.45523504	1.95958880
H	-1.17599520	0.12310329	2.82520595
H	1.30646207	1.48577822	1.37821379

C	3.81795923	1.58387216	0.18189124
H	4.85687235	1.29816557	0.36927619
H	3.79800306	2.05644233	-0.78879143
H	3.57783025	2.37211474	0.89673875
H	-1.49772210	-1.63724113	-1.49636892

Time = 398.00 fs
E(CASSCF/6-31G(d)) = -479.5168301

C	-0.85907592	-0.16845612	-0.13030205
C	0.53975588	-0.15356615	0.10003517
C	1.34214344	-1.01450839	-0.84803798
C	1.45919853	0.69574607	0.69133974
C	2.72569289	0.45751531	0.12483210
C	2.63387623	-0.60638883	-0.79876185
N	-1.84940432	0.03505482	0.79828678
C	-3.13941213	0.14257662	0.10699069
H	3.50632311	-1.12290548	-1.20214498
C	-1.40411010	-0.55451609	-1.41700473
C	-2.81955803	0.11445272	-1.35746875
H	-2.63539830	1.12666911	-1.72102553
H	-3.52721689	-0.40551272	-1.99937974
H	-3.66616804	-0.79557254	0.30390157
H	-3.77006157	0.94649811	0.41673905
H	-0.92775855	-0.14128916	-2.31028842
H	0.97907478	-1.73442777	-1.52286410
C	-1.68800295	-0.54024862	2.10805882
H	-2.65164157	-0.89630303	2.41003468
H	-1.13883077	-1.45110189	1.96115679
H	-1.21462945	0.13191905	2.84411502
H	1.29775139	1.48425450	1.36471749
C	3.81619444	1.58482974	0.18909132
H	4.85212727	1.29707898	0.40186357
H	3.81262760	2.05772874	-0.78451821
H	3.55700487	2.38154425	0.89055502
H	-1.49703542	-1.64346014	-1.50996678

Time = 398.50 fs
E(CASSCF/6-31G(d)) = -479.5167267

C	-0.85751544	-0.17526894	-0.12435305
C	0.53782532	-0.15950031	0.10161400
C	1.34167794	-1.01383313	-0.84913945
C	1.45678182	0.70275604	0.68116323
C	2.72271325	0.45856012	0.11596503
C	2.64034201	-0.60735599	-0.80357852
N	-1.84536457	0.03352545	0.80062848

C	-3.13698179	0.15142669	0.11300867
H	3.50889725	-1.12830178	-1.22228355
C	-1.40179453	-0.56041013	-1.41841970
C	-2.81403263	0.11626202	-1.35597648
H	-2.61594892	1.12459039	-1.72351785
H	-3.52968211	-0.40410512	-1.98784208
H	-3.66374824	-0.79588633	0.31128948
H	-3.75723144	0.96467983	0.41960848
H	-0.92289067	-0.14165009	-2.30639375
H	0.98476266	-1.72625761	-1.53445737
C	-1.69903407	-0.54084754	2.10926758
H	-2.66802507	-0.90834264	2.39651123
H	-1.13501169	-1.44727427	1.96384332
H	-1.25426691	0.13944924	2.86202460
H	1.28789856	1.48433558	1.35254662
C	3.81432840	1.58563665	0.19626195
H	4.84629902	1.29521010	0.43436053
H	3.82774453	2.05918868	-0.78069639
H	3.53586971	2.38987092	0.88443822
H	-1.49621440	-1.64898549	-1.52371404

Time = 399.00 fs
E(CASSCF/6-31G(d)) = -479.5165790

C	-0.85530797	-0.18209105	-0.11803596
C	0.53539967	-0.16590666	0.10257185
C	1.34127420	-1.01289523	-0.85019612
C	1.45492931	0.70976648	0.67105703
C	2.71974976	0.46033097	0.10757006
C	2.64692191	-0.60867145	-0.80851620
N	-1.84133794	0.03230331	0.80278044
C	-3.13460100	0.15977272	0.11916490
H	3.50987372	-1.13249805	-1.24257898
C	-1.39992386	-0.56636370	-1.42018268
C	-2.80820275	0.11814255	-1.35463221
H	-2.59798265	1.12225917	-1.72623000
H	-3.53240176	-0.40185202	-1.97568017
H	-3.66214595	-0.79493237	0.31926229
H	-3.74452582	0.98410166	0.42318579
H	-0.91708167	-0.14317387	-2.30118559
H	0.98914267	-1.71988052	-1.54654689
C	-1.71011347	-0.54146948	2.11045368
H	-2.68529748	-0.91947460	2.38447814
H	-1.12950928	-1.44344370	1.96787175
H	-1.29484032	0.14544396	2.87867489
H	1.27695182	1.48643380	1.34203328
C	3.81236012	1.58630785	0.20338307

H	4.83923160	1.29269560	0.46668186
H	3.84345413	2.06062390	-0.77711848
H	3.51453022	2.39694161	0.87839010
H	-1.49523919	-1.65373620	-1.53748287

Time = 399.50 fs
E(CASSCF/6-31G(d)) = -479.5164817

C	-0.85248808	-0.18894863	-0.11142691
C	0.53254601	-0.17275260	0.10292662
C	1.34097774	-1.01172027	-0.85123163
C	1.45361395	0.71672096	0.66099904
C	2.71682757	0.46285076	0.09966013
C	2.65354340	-0.61034626	-0.81357876
N	-1.83735265	0.03139740	0.80475605
C	-3.13225156	0.16759212	0.12542435
H	3.50913246	-1.13528830	-1.26278469
C	-1.39849271	-0.57236877	-1.42223883
C	-2.80208205	0.12008581	-1.35340780
H	-2.58164916	1.11972503	-1.72915115
H	-3.53535207	-0.39870354	-1.96305301
H	-3.66127131	-0.79214223	0.32773628
H	-3.73193022	1.00450971	0.42744369
H	-0.91029065	-0.14578693	-2.29480390
H	0.99219931	-1.71520466	-1.55897909
C	-1.72124917	-0.54213939	2.11165160
H	-2.70302771	-0.92967156	2.37371969
H	-1.12258106	-1.43926170	1.97345282
H	-1.33623187	0.14972838	2.89385430
H	1.26500848	1.49074912	1.33332721
C	3.81028749	1.58685982	0.21043246
H	4.83079946	1.28966723	0.49874587
H	3.85984083	2.06182971	-0.77353654
H	3.49310428	2.40263075	0.87241376
H	-1.49410808	-1.65769044	-1.55114989

Total electronic energies of current state (E, in a.u.) and Cartesian coordinates (in Å) along the 2-Z → 2-E photoisomerization trajectory in Figure 2

Time = 0.00 fs
E(CASSCF/6-31G(d)) = -479.5485893

C	-0.88325504	-0.25053701	-0.05698551
C	0.47722062	-0.32215638	-0.03193811
C	1.20939530	-1.59122561	0.08290988
C	1.52200720	0.71663657	-0.08578619
C	2.74519788	0.12692914	-0.01661727
C	2.54086933	-1.32583754	0.08986913
N	-1.70416269	0.85924982	-0.17291111
C	-3.08246329	0.53551499	0.15241580
H	3.33413807	-2.04931685	0.16512030
C	-1.79685795	-1.46491694	0.06912303
C	-3.19170532	-0.92573413	-0.25691601
H	-3.38333406	-0.99032718	-1.32133248
H	-3.98016489	-1.45274134	0.26171537
H	-3.28493247	0.66642013	1.21830826
H	-3.75859215	1.17978651	-0.39375661
H	-1.49994932	-2.27591193	-0.57930980
H	0.76318269	-2.56573491	0.15286080
C	-1.25734917	2.21509728	0.02078325
H	-0.76521390	2.35488740	0.98254259
H	-0.57825542	2.51525140	-0.76515434
H	-2.11613119	2.87123774	-0.01945775
H	1.36258774	1.77164474	-0.16465441
C	4.08755540	0.80303559	-0.03392854
H	4.64804351	0.59588703	0.87530017
H	4.69355674	0.46148535	-0.87042319
H	3.98078431	1.87869881	-0.11959099
H	-1.76212371	-1.83471765	1.08954931

Time = 0.50 fs
E(CASSCF/6-31G(d)) = -479.5490643

C	-0.88482115	-0.25035484	-0.05694015
C	0.47884693	-0.32237372	-0.03194036
C	1.20844864	-1.59116811	0.08293616
C	1.52082542	0.71670636	-0.08584830
C	2.74588382	0.12624437	-0.01655060
C	2.54195967	-1.32500287	0.08984573
N	-1.70373829	0.85901877	-0.17273934
C	-3.08253227	0.53539593	0.15223068

H	3.33423317	-2.04895396	0.16513048
C	-1.79696834	-1.46452952	0.06905680
C	-3.19182466	-0.92580158	-0.25693645
H	-3.38333701	-0.99045030	-1.32106415
H	-3.98006476	-1.45262792	0.26167547
H	-3.28411151	0.66676338	1.21807771
H	-3.75820018	1.17994898	-0.39358900
H	-1.49958204	-2.27571834	-0.57934875
H	0.76390878	-2.56578784	0.15308691
C	-1.25740108	2.21528757	0.02080408
H	-0.76577925	2.35420504	0.98218338
H	-0.57859564	2.51514587	-0.76475924
H	-2.11647637	2.87068900	-0.01913017
H	1.36210931	1.77112265	-0.16468825
C	4.08757286	0.80274763	-0.03395002
H	4.64830425	0.59618468	0.87505943
H	4.69382102	0.46159735	-0.87020516
H	3.98058962	1.87832958	-0.11975718
H	-1.76143035	-1.83462737	1.08934927

Time = 1.00 fs
E(CASSCF/6-31G(d)) = -479.5503849

C	-0.88741827	-0.25010205	-0.05695572
C	0.48151096	-0.32262308	-0.03193032
C	1.20684336	-1.59106414	0.08295127
C	1.51897226	0.71689473	-0.08594327
C	2.74700966	0.12507668	-0.01643396
C	2.54364622	-1.32385883	0.08981461
N	-1.70308988	0.85864399	-0.17242101
C	-3.08267848	0.53524144	0.15198828
H	3.33420074	-2.04846142	0.16509330
C	-1.79703338	-1.46405770	0.06897345
C	-3.19192380	-0.92587685	-0.25695464
H	-3.38334861	-0.99060900	-1.32089106
H	-3.98011929	-1.45248427	0.26172323
H	-3.28276535	0.66726907	1.21769401
H	-3.75773343	1.18032331	-0.39344328
H	-1.49893295	-2.27563295	-0.57950689
H	0.76449162	-2.56565817	0.15330224
C	-1.25744058	2.21558341	0.02080416
H	-0.76647742	2.35324327	0.98157211
H	-0.57890710	2.51481158	-0.76427337
H	-2.11692365	2.87005371	-0.01882949
H	1.36155076	1.77067267	-0.16474250
C	4.08755383	0.80243904	-0.03397187
H	4.64866832	0.59656088	0.87480466

H	4.69418537	0.46178827	-0.86997946
H	3.98046640	1.87812693	-0.11993611
H	-1.76044081	-1.83477422	1.08941117

Time = 1.50 fs
E(CASSCF/6-31G(d)) = -479.5524462

C	-0.89098589	-0.24978104	-0.05703105
C	0.48515514	-0.32290437	-0.03190856
C	1.20461025	-1.59090161	0.08295455
C	1.51647380	0.71718540	-0.08606905
C	2.74855956	0.12344995	-0.01626960
C	2.54590273	-1.32242358	0.08977670
N	-1.70223151	0.85813076	-0.17196040
C	-3.08289664	0.53505439	0.15169023
H	3.33410606	-2.04787459	0.16501372
C	-1.79705564	-1.46350884	0.06887357
C	-3.19200435	-0.92595938	-0.25696920
H	-3.38337158	-0.99080562	-1.32082908
H	-3.98033527	-1.45231881	0.26186203
H	-3.28092822	0.66792620	1.21717605
H	-3.75721078	1.18091137	-0.39333431
H	-1.49801367	-2.27562063	-0.57976559
H	0.76487929	-2.56540077	0.15350879
C	-1.25747017	2.21597562	0.02078351
H	-0.76726981	2.35203769	0.98077472
H	-0.57915995	2.51427940	-0.76373922
H	-2.11748260	2.86934642	-0.01856127
H	1.36088440	1.77035622	-0.16482140
C	4.08750162	0.80211259	-0.03399425
H	4.64914083	0.59701162	0.87454838
H	4.69465617	0.46205215	-0.86975774
H	3.98040633	1.87807897	-0.12012697
H	-1.75916690	-1.83514411	1.08971316

Time = 2.00 fs
E(CASSCF/6-31G(d)) = -479.5550689

C	-0.89544150	-0.24939656	-0.05716438
C	0.48970071	-0.32321733	-0.03187598
C	1.20179282	-1.59066473	0.08294529
C	1.51336781	0.71755740	-0.08622291
C	2.75051063	0.12139831	-0.01606072
C	2.54869208	-1.32072212	0.08973325
N	-1.70118285	0.85748739	-0.17136341
C	-3.08318035	0.53483890	0.15133786
H	3.33403978	-2.04724412	0.16489866

C	-1.79703919	-1.46289307	0.06875897
C	-3.19206990	-0.92604960	-0.25697880
H	-3.38340695	-0.99104266	-1.32088406
H	-3.98070960	-1.45213526	0.26208798
H	-3.27864754	0.66872099	1.21655263
H	-3.75665075	1.18170882	-0.39327469
H	-1.49684180	-2.27563119	-0.58009698
H	0.76500668	-2.56508356	0.15370920
C	-1.25749257	2.21645287	0.02074178
H	-0.76810921	2.35063494	0.97987394
H	-0.57932112	2.51358823	-0.76320567
H	-2.11816135	2.86858239	-0.01833181
H	1.36007466	1.77023438	-0.16492915
C	4.08742133	0.80177187	-0.03401732
H	4.64972582	0.59753366	0.87430032
H	4.69523875	0.46238418	-0.86954923
H	3.98039699	1.87815908	-0.12032784
H	-1.75762398	-1.83571236	1.09020903

Time = 2.50 fs
E(CASSCF/6-31G(d)) = -479.5580545

C	-0.90068427	-0.24895591	-0.05735342
C	0.49504990	-0.32356133	-0.03183376
C	1.19844689	-1.59033598	0.08292290
C	1.50970397	0.71798669	-0.08640161
C	2.75283296	0.11896615	-0.01581148
C	2.55196695	-1.31878651	0.08968584
N	-1.69996894	0.85672576	-0.17063694
C	-3.08352206	0.53460037	0.15093205
H	3.33411037	-2.04663190	0.16475642
C	-1.79698939	-1.46222284	0.06863267
C	-3.19212578	-0.92614878	-0.25698232
H	-3.38345386	-0.99132248	-1.32105188
H	-3.98123022	-1.45193277	0.26239037
H	-3.27598070	0.66963780	1.21585961
H	-3.75606946	1.18270500	-0.39327414
H	-1.49543947	-2.27560459	-0.58046783
H	0.76480118	-2.56477989	0.15390632
C	-1.25751056	2.21700199	0.02067836
H	-0.76894420	2.34908925	0.97896150
H	-0.57935851	2.51278150	-0.76272336
H	-2.11896505	2.86777534	-0.01814797
H	1.35908133	1.77036044	-0.16506942
C	4.08731992	0.80142116	-0.03404124
H	4.65042585	0.59812494	0.87406668
H	4.69593690	0.46278118	-0.86936007

H	3.98042166	1.87832837	-0.12053600
H	-1.75583074	-1.83644635	1.09083330

Time = 3.00 fs
E(CASSCF/6-31G(d)) = -479.5611595

C	-0.90659879	-0.24846937	-0.05759554
C	0.50108907	-0.32393533	-0.03178340
C	1.19464054	-1.58989778	0.08288704
C	1.50554447	0.71844748	-0.08660152
C	2.75548907	0.11620788	-0.01552695
C	2.55567024	-1.31665623	0.08963639
N	-1.69861965	0.85586174	-0.16978853
C	-3.08391356	0.53434532	0.15047315
H	3.33443381	-2.04610396	0.16459582
C	-1.79691294	-1.46151232	0.06849864
C	-3.19217883	-0.92625900	-0.25697879
H	-3.38350994	-0.99164716	-1.32131857
H	-3.98187747	-1.45170603	0.26275260
H	-3.27299191	0.67065975	1.21513655
H	-3.75547954	1.18388304	-0.39333858
H	-1.49383233	-2.27547611	-0.58084323
H	0.76418862	-2.56455821	0.15410277
C	-1.25752656	2.21760864	0.02059246
H	-0.76972379	2.34745954	0.97812972
H	-0.57924500	2.51190436	-0.76233946
H	-2.11989469	2.86693645	-0.01801686
H	1.35786189	1.77077212	-0.16524488
C	4.08720629	0.80106521	-0.03406615
H	4.65124156	0.59878459	0.87384980
H	4.69675239	0.46324173	-0.86919293
H	3.98045964	1.87853895	-0.12074815
H	-1.75380917	-1.83730855	1.09150769

Time = 3.50 fs
E(CASSCF/6-31G(d)) = -479.5641357

C	-0.91305822	-0.24795102	-0.05788777
C	0.50769062	-0.32433775	-0.03172666
C	1.19045456	-1.58933468	0.08283790
C	1.50096479	0.71891368	-0.08681875
C	2.75843383	0.11318790	-0.01521311
C	2.55973552	-1.31437864	0.08958722
N	-1.69716925	0.85491540	-0.16882631
C	-3.08434653	0.53408112	0.14996126
H	3.33512327	-2.04572075	0.16442515
C	-1.79681788	-1.46077655	0.06836166

C	-3.19223713	-0.92638319	-0.25696765
H	-3.38357138	-0.99201842	-1.32166096
H	-3.98262490	-1.45144570	0.26315297
H	-3.26975023	0.67176838	1.21442373
H	-3.75488927	1.18521996	-0.39346901
H	-1.49204816	-2.27518057	-0.58119006
H	0.76309689	-2.56447059	0.15430008
C	-1.25754233	2.21825819	0.02048327
H	-0.77040137	2.34580722	0.97746392
H	-0.57896122	2.51100114	-0.76209337
H	-2.12094605	2.86607391	-0.01794536
H	1.35637129	1.77148433	-0.16545675
C	4.08709095	0.80070893	-0.03409216
H	4.65217163	0.59951224	0.87364825
H	4.69768476	0.46376551	-0.86904702
H	3.98048756	1.87873826	-0.12096064
H	-1.75158433	-1.83825826	1.09214685

Time = 4.00 fs
E(CASSCF/6-31G(d)) = -479.5667363

C	-0.91992788	-0.24741849	-0.05822683
C	0.51471663	-0.32476658	-0.03166557
C	1.18597990	-1.58863531	0.08277624
C	1.49605216	0.71936094	-0.08704930
C	2.76161552	0.10997885	-0.01487673
C	2.56408939	-1.31200822	0.08954106
N	-1.69565579	0.85391109	-0.16775877
C	-3.08481275	0.53381594	0.14939644
H	3.33627923	-2.04553046	0.16425131
C	-1.79671349	-1.46003094	0.06822695
C	-3.19230960	-0.92652482	-0.25694856
H	-3.38363356	-0.99243787	-1.32204933
H	-3.98344289	-1.45114043	0.26356708
H	-3.26632745	0.67294423	1.21375868
H	-3.75430358	1.18668797	-0.39366269
H	-1.49011519	-2.27465900	-0.58148141
H	0.76146349	-2.56454348	0.15449818
C	-1.25755852	2.21893619	0.02035035
H	-0.77093866	2.34419382	0.97703423
H	-0.57849833	2.51011337	-0.76201316
H	-2.12210972	2.86519300	-0.01794002
H	1.35456592	1.77248367	-0.16570451
C	4.08698593	0.80035712	-0.03411933
H	4.65321211	0.60030790	0.87345688
H	4.69873075	0.46435320	-0.86891804
H	3.98047917	1.87887339	-0.12116993

H	-1.74918403	-1.83925603	1.09266737

Time = 4.50 fs
E(CASSCF/6-31G(d)) = -479.5687853

C	-0.92707037	-0.24689263	-0.05860938
C	0.52202331	-0.32521929	-0.03160235
C	1.18131361	-1.58779417	0.08270350
C	1.49090243	0.71976844	-0.08728933
C	2.76497813	0.10665959	-0.01452510
C	2.56865541	-1.30960586	0.08950107
N	-1.69412019	0.85287709	-0.16659460
C	-3.08530397	0.53355852	0.14877892
H	3.33797898	-2.04556469	0.16407916
C	-1.79660993	-1.45929049	0.06809983
C	-3.19240553	-0.92668758	-0.25692145
H	-3.38369167	-0.99290668	-1.32244981
H	-3.98430115	-1.45077813	0.26396997
H	-3.26279654	0.67416711	1.21317194
H	-3.75372290	1.18825520	-0.39391294
H	-1.48806081	-2.27386268	-0.58169949
H	0.75924132	-2.56477140	0.15469507
C	-1.25757442	2.21962850	0.02019380
H	-0.77131030	2.34267903	0.97688780
H	-0.57786189	2.50927684	-0.76211166
H	-2.12336988	2.86429411	-0.01800687
H	1.35240672	1.77372746	-0.16598558
C	4.08690450	0.80001432	-0.03414765
H	4.65435765	0.60117279	0.87326804
H	4.69988542	0.46500705	-0.86879943
H	3.98040563	1.87889613	-0.12137290
H	-1.74663820	-1.84026592	1.09299426

Time = 5.00 fs
E(CASSCF/6-31G(d)) = -479.5701130

C	-0.93434669	-0.24639744	-0.05903183
C	0.52946257	-0.32569298	-0.03153943
C	1.17655770	-1.58681288	0.08262184
C	1.48561900	0.72012005	-0.08753521
C	2.76846230	0.10331327	-0.01416595
C	2.57335540	-1.30723758	0.08947073
N	-1.69260559	0.85184532	-0.16534280
C	-3.08581240	0.53331801	0.14810969
H	3.34027055	-2.04582937	0.16391088
C	-1.79651837	-1.45856933	0.06798541
C	-3.19253423	-0.92687526	-0.25688649

H	-3.38374107	-0.99342616	-1.32282762
H	-3.98517179	-1.45034826	0.26433801
H	-3.25923076	0.67541570	1.21268564
H	-3.75314618	1.18988748	-0.39421091
H	-1.48591033	-2.27275738	-0.58183826
H	0.75640069	-2.56511134	0.15488640
C	-1.25758780	2.22032185	0.02001462
H	-0.77150456	2.34131959	0.97704608
H	-0.57707021	2.50852188	-0.76238708
H	-2.12470578	2.86337572	-0.01815106
H	1.34985869	1.77514290	-0.16629536
C	4.08686078	0.79968452	-0.03417708
H	4.65560053	0.60210749	0.87307217
H	4.70114126	0.46572916	-0.86868266
H	3.98023687	1.87876658	-0.12156713
H	-1.74397846	-1.84125812	1.09306748

Time = 5.50 fs
E(CASSCF/6-31G(d)) = -479.5706369

C	-0.94161947	-0.24595933	-0.05949053
C	0.53688559	-0.32618451	-0.03147934
C	1.17181532	-1.58570076	0.08253412
C	1.48030941	0.72040527	-0.08778366
C	2.77200766	0.10002472	-0.01380714
C	2.57811277	-1.30497300	0.08945385
N	-1.69115642	0.85085071	-0.16401272
C	-3.08633069	0.53310368	0.14739069
H	3.34316780	-2.04630280	0.16374571
C	-1.79645069	-1.45788034	0.06788830
C	-3.19270462	-0.92709142	-0.25684399
H	-3.38377800	-0.99399786	-1.32315023
H	-3.98603212	-1.44984365	0.26465108
H	-3.25570310	0.67666768	1.21231146
H	-3.75257194	1.19154988	-0.39454653
H	-1.48368628	-2.27132612	-0.58190506
H	0.75293218	-2.56548314	0.15506554
C	-1.25759491	2.22100392	0.01981483
H	-0.77152456	2.34016856	0.97750259
H	-0.57615447	2.50787272	-0.76282298
H	-2.12609193	2.86243472	-0.01837679
H	1.34689306	1.77663134	-0.16662738
C	4.08686943	0.79937111	-0.03420751
H	4.65693135	0.60311210	0.87285911
H	4.70248892	0.46652133	-0.86855837
H	3.97994243	1.87845648	-0.12175108
H	-1.74123777	-1.84221088	1.09284686

Time = 6.00 fs
E(CASSCF/6-31G(d)) = -479.5703385

C	-0.94875479	-0.24560658	-0.05998172
C	0.54414514	-0.32669053	-0.03142469
C	1.16718776	-1.58447489	0.08244382
C	1.47508220	0.72061960	-0.08803188
C	2.77555499	0.09687826	-0.01345646
C	2.58285496	-1.30288358	0.08945447
N	-1.68981767	0.84993056	-0.16261417
C	-3.08685178	0.53292483	0.14662521
H	3.34665026	-2.04693616	0.16357984
C	-1.79641918	-1.45723484	0.06781245
C	-3.19292484	-0.92733909	-0.25679441
H	-3.38379970	-0.99462333	-1.32338924
H	-3.98686588	-1.44926131	0.26489400
H	-3.25228564	0.67789934	1.21204830
H	-3.75199883	1.19320753	-0.39490939
H	-1.48140900	-2.26956996	-0.58192023
H	0.74884598	-2.56577489	0.15522382
C	-1.25759075	2.22166335	0.01959774
H	-0.77138817	2.33927428	0.97822289
H	-0.57515740	2.50734705	-0.76338985
H	-2.12749908	2.86146673	-0.01868727
H	1.34348756	1.77807620	-0.16697386
C	4.08694511	0.79907667	-0.03423881
H	4.65833975	0.60418601	0.87261917
H	4.70391789	0.46738468	-0.86841751
H	3.97949267	1.87795186	-0.12192436
H	-1.73845078	-1.84311128	1.09231408

Time = 6.50 fs
E(CASSCF/6-31G(d)) = -479.5692634

C	-0.95562446	-0.24536810	-0.06050172
C	0.55109843	-0.32720761	-0.03137809
C	1.16277206	-1.58315920	0.08235492
C	1.47004420	0.72076412	-0.08827749
C	2.77904758	0.09395523	-0.01312146
C	2.58751497	-1.30104042	0.08947670
N	-1.68863363	0.84912335	-0.16115735
C	-3.08736900	0.53279046	0.14581801
H	3.35066568	-2.04765704	0.16340686
C	-1.79643623	-1.45664246	0.06776094
C	-3.19320190	-0.92762056	-0.25673823
H	-3.38380505	-0.99530438	-1.32352329

H	-3.98766529	-1.44860371	0.26505787
H	-3.24904910	0.67908601	1.21188290
H	-3.75142743	1.19482752	-0.39529014
H	-1.47909653	-2.26750821	-0.58191667
H	0.74417061	-2.56585137	0.15535123
C	-1.25756937	2.22228973	0.01936781
H	-0.77112603	2.33868009	0.97914829
H	-0.57413032	2.50695673	-0.76404830
H	-2.12889546	2.86046779	-0.01908463
H	1.33962732	1.77935228	-0.16732630
C	4.08710215	0.79880312	-0.03427077
H	4.65981481	0.60532760	0.87234443
H	4.70541703	0.46831914	-0.86825245
H	3.97885996	1.87725411	-0.12208775
H	-1.73565317	-1.84395615	1.09147460

Time = 7.00 fs
E(CASSCF/6-31G(d)) = -479.5675218

C	-0.96210793	-0.24527222	-0.06104696
C	0.55760950	-0.32773218	-0.03134209
C	1.15865917	-1.58178318	0.08227177
C	1.46529797	0.72084442	-0.08851857
C	2.78243245	0.09133174	-0.01280922
C	2.59203231	-1.29951185	0.08952460
N	-1.68764668	0.84846731	-0.15965265
C	-3.08787610	0.53270908	0.14497533
H	3.35513586	-2.04837606	0.16321850
C	-1.79651406	-1.45611114	0.06773583
C	-3.19354153	-0.92793719	-0.25667591
H	-3.38379466	-0.99604307	-1.32353946
H	-3.98843155	-1.44787918	0.26514069
H	-3.24606229	0.68020234	1.21179073
H	-3.75086140	1.19638002	-0.39568158
H	-1.47676542	-2.26517699	-0.58193774
H	0.73894996	-2.56556559	0.15543723
C	-1.25752440	2.22287371	0.01913051
H	-0.77077851	2.33842386	0.98020143
H	-0.57312938	2.50670852	-0.76475318
H	-2.13024830	2.85943576	-0.01956982
H	1.33530536	1.78033658	-0.16767623
C	4.08735407	0.79855173	-0.03430321
H	4.66134571	0.60653399	0.87202998
H	4.70697525	0.46932300	-0.86805821
H	3.97801984	1.87638025	-0.12224320
H	-1.73288135	-1.84475197	1.09035690

Time = 7.50 fs
E(CASSCF/6-31G(d)) = -479.5652782

C	-0.96809445	-0.24534482	-0.06161423
C	0.56355199	-0.32826053	-0.03131919
C	1.15493229	-1.58037998	0.08219889
C	1.46093924	0.72086934	-0.08875357
C	2.78566106	0.08907646	-0.01252616
C	2.59635352	-1.29836082	0.08960192
N	-1.68689575	0.84799862	-0.15811045
C	-3.08836742	0.53268847	0.14410475
H	3.35996429	-2.04899548	0.16300547
C	-1.79666432	-1.45564735	0.06773801
C	-3.19394797	-0.92828933	-0.25660781
H	-3.38377141	-0.99684194	-1.32343501
H	-3.98917518	-1.44710203	0.26514771
H	-3.24339173	0.68122327	1.21173864
H	-3.75030850	1.19783983	-0.39607965
H	-1.47443106	-2.26262679	-0.58203477
H	0.73323955	-2.56477228	0.15547179
C	-1.25744958	2.22340706	0.01889210
H	-0.77039204	2.33853768	0.98129313
H	-0.57221170	2.50660477	-0.76545774
H	-2.13152498	2.85837131	-0.02014278
H	1.33052289	1.78091949	-0.16801584
C	4.08771337	0.79832340	-0.03433592
H	4.66292221	0.60780111	0.87167460
H	4.70858206	0.47039289	-0.86783301
H	3.97695248	1.87536149	-0.12239364
H	-1.73017153	-1.84551410	1.08901079

Time = 8.00 fs
E(CASSCF/6-31G(d)) = -479.5627397

C	-0.97348466	-0.24560775	-0.06220077
C	0.56881119	-0.32878870	-0.03131175
C	1.15166575	-1.57898444	0.08214078
C	1.45705482	0.72084929	-0.08898123
C	2.78868994	0.08724868	-0.01227791
C	2.60043221	-1.29764250	0.08971200
N	-1.68641501	0.84774963	-0.15654081
C	-3.08883796	0.53273545	0.14321497
H	3.36504536	-2.04941911	0.16275844
C	-1.79689780	-1.45525630	0.06776727
C	-3.19442394	-0.92867621	-0.25653413
H	-3.38374020	-0.99770374	-1.32321704
H	-3.98991474	-1.44629164	0.26509079

H	-3.24110079	0.68212463	1.21168706
H	-3.74978058	1.19918679	-0.39648392
H	-1.47210920	-2.25991843	-0.58226302
H	0.72710210	-2.56334189	0.15544637
C	-1.25733934	2.22388273	0.01865926
H	-0.77001510	2.33904732	0.98233005
H	-0.57143154	2.50664412	-0.76611801
H	-2.13269462	2.85727878	-0.02080242
H	1.32528934	1.78101547	-0.16833885
C	4.08819107	0.79811884	-0.03436874
H	4.66453525	0.60912368	0.87128156
H	4.71022825	0.47152360	-0.86757917
H	3.97564397	1.87424126	-0.12254287
H	-1.72755939	-1.84626515	1.08750309

Time = 8.50 fs
E(CASSCF/6-31G(d)) = -479.5601352

C	-0.97819273	-0.24607698	-0.06280450
C	0.57328687	-0.32931249	-0.03132196
C	1.14892406	-1.57763082	0.08210169
C	1.45372069	0.72079450	-0.08920051
C	2.79148064	0.08589608	-0.01206913
C	2.60422879	-1.29740122	0.08985746
N	-1.68623224	0.84774672	-0.15495304
C	-3.08928372	0.53285567	0.14231543
H	3.37027304	-2.04956213	0.16246899
C	-1.79722402	-1.45494225	0.06782220
C	-3.19497069	-0.92909599	-0.25645495
H	-3.38370809	-0.99863143	-1.32290202
H	-3.99067520	-1.44547122	0.26498742
H	-3.23924866	0.68288442	1.21159342
H	-3.74929365	1.20040632	-0.39689779
H	-1.46981697	-2.25711884	-0.58267740
H	0.72060386	-2.56117320	0.15535488
C	-1.25718932	2.22429519	0.01843870
H	-0.76969423	2.33997152	0.98322228
H	-0.57083635	2.50682210	-0.76669672
H	-2.13372951	2.85616701	-0.02154666
H	1.31962286	1.78057118	-0.16864106
C	4.08879650	0.79793889	-0.03440158
H	4.66617728	0.61049548	0.87085891
H	4.71190624	0.47270825	-0.86730346
H	3.97408756	1.87307197	-0.12269518
H	-1.72507914	-1.84703318	1.08591277

Time = 9.00 fs

E(CASSCF/6-31G(d)) = -479.5576837

C	-0.98214738	-0.24676094	-0.06342400
C	0.57689454	-0.32982718	-0.03135185
C	1.14676133	-1.57635095	0.08208551
C	1.45100031	0.72071355	-0.08941046
C	2.79400015	0.08505339	-0.01190335
C	2.60771020	-1.29766874	0.09004012
N	-1.68636767	0.84800841	-0.15335554
C	-3.08970189	0.53305344	0.14141592
H	3.37554884	-2.04935926	0.16213043
C	-1.79765103	-1.45470883	0.06790028
C	-3.19558815	-0.92954582	-0.25637013
H	-3.38368394	-0.99962844	-1.32251440
H	-3.99148569	-1.44466664	0.26485936
H	-3.23789013	0.68348340	1.21141642
H	-3.74886816	1.20148928	-0.39732856
H	-1.46757383	-2.25429633	-0.58332823
H	0.71381173	-2.55820134	0.15519419
C	-1.25699688	2.22464054	0.01823674
H	-0.76946950	2.34132198	0.98389146
H	-0.57046283	2.50713223	-0.76716744
H	-2.13460606	2.85505075	-0.02237252
H	1.31355040	1.77956897	-0.16892055
C	4.08953706	0.79778484	-0.03443440
H	4.66784231	0.61190956	0.87041921
H	4.71361019	0.47393847	-0.86701687
H	3.97228481	1.87191070	-0.12285510
H	-1.72276259	-1.84784889	1.08432468

Time = 9.50 fs
E(CASSCF/6-31G(d)) = -479.5556079

C	-0.98529325	-0.24765950	-0.06405864
C	0.57956756	-0.33032766	-0.03140320
C	1.14522059	-1.57517236	0.08209556
C	1.44894339	0.72061196	-0.08961020
C	2.79622074	0.08474136	-0.01178298
C	2.61084956	-1.29846254	0.09026085
N	-1.68683314	0.84854425	-0.15175545
C	-3.09009100	0.53333165	0.14052618
H	3.38078856	-2.04877208	0.16173842
C	-1.79818497	-1.45455947	0.06799805
C	-3.19627504	-0.93002189	-0.25627945
H	-3.38367805	-1.00069783	-1.32208420
H	-3.99237640	-1.44390392	0.26473066
H	-3.23707428	0.68390631	1.21111832

H	-3.74852680	1.20243135	-0.39778669
H	-1.46540347	-2.25151555	-0.58425654
H	0.70678970	-2.55440790	0.15496491
C	-1.25676146	2.22491658	0.01805901
H	-0.76937223	2.34310278	0.98427578
H	-0.57033582	2.50756571	-0.76751599
H	-2.13530574	2.85394948	-0.02327621
H	1.30710776	1.77803197	-0.16917814
C	4.09041802	0.79765868	-0.03446728
H	4.66952606	0.61335847	0.86997919
H	4.71533609	0.47520474	-0.86673437
H	3.97024666	1.87081533	-0.12302697
H	-1.72063919	-1.84874298	1.08282265

Time = 10.00 fs
E(CASSCF/6-31G(d)) = -479.5540717

C	-0.98759145	-0.24876317	-0.06470853
C	0.58125830	-0.33080835	-0.03147753
C	1.14433339	-1.57411695	0.08213446
C	1.44758489	0.72049134	-0.08979886
C	2.79811997	0.08496617	-0.01170915
C	2.61362583	-1.29978492	0.09051947
N	-1.68763144	0.84935368	-0.15015857
C	-3.09045115	0.53369168	0.13965550
H	3.38592679	-2.04779315	0.16129131
C	-1.79882983	-1.45449764	0.06811131
C	-3.19702917	-0.93051965	-0.25618258
H	-3.38370177	-1.00184228	-1.32164446
H	-3.99337563	-1.44320735	0.26462564
H	-3.23684390	0.68414258	1.21066842
H	-3.74829366	1.20323233	-0.39828522
H	-1.46333461	-2.24883339	-0.58549035
H	0.69959760	-2.54982347	0.15467158
C	-1.25648476	2.22512296	0.01791010
H	-0.76942233	2.34531027	0.98433476
H	-0.57046649	2.50811175	-0.76774210
H	-2.13581564	2.85288768	-0.02425322
H	1.30033986	1.77602167	-0.16941719
C	4.09144248	0.79756344	-0.03450043
H	4.67122566	0.61483470	0.86955885
H	4.71708159	0.47649684	-0.86647422
H	3.96799411	1.86983987	-0.12321457
H	-1.71873544	-1.84974338	1.08148279

Time = 10.50 fs
E(CASSCF/6-31G(d)) = -479.5531895

C	-0.98902025	-0.25005277	-0.06537435
C	0.58193909	-0.33126329	-0.03157613
C	1.14411948	-1.57320013	0.08220406
C	1.44694437	0.72034914	-0.08997557
C	2.79968075	0.08571899	-0.01168177
C	2.61602352	-1.30162242	0.09081478
N	-1.68875587	0.85042541	-0.14856911
C	-3.09078430	0.53413340	0.13881229
H	3.39091905	-2.04644769	0.16079050
C	-1.79958729	-1.45452707	0.06823523
C	-3.19784769	-0.93103393	-0.25607920
H	-3.38376704	-1.00306389	-1.32122839
H	-3.99450687	-1.44259764	0.26456696
H	-3.23723456	0.68418707	1.21004556
H	-3.74819282	1.20389509	-0.39883890
H	-1.46140148	-2.24629607	-0.58704189
H	0.69229104	-2.54452601	0.15432242
C	-1.25617088	2.22526145	0.01779326
H	-0.76962680	2.34793295	0.98405216
H	-0.57085184	2.50875751	-0.76785965
H	-2.13612860	2.85189445	-0.02529846
H	1.29330120	1.77363154	-0.16964316
C	4.09261128	0.79750335	-0.03453418
H	4.67293926	0.61633103	0.86918026
H	4.71884537	0.47780434	-0.86625686
H	3.96555856	1.86903038	-0.12342086
H	-1.71707418	-1.85087241	1.08036717

Time = 11.00 fs
E(CASSCF/6-31G(d)) = -479.5530083

C	-0.98957443	-0.25150025	-0.06605743
C	0.58160227	-0.33168630	-0.03169990
C	1.14458646	-1.57243055	0.08230541
C	1.44702578	0.72017890	-0.09013949
C	2.80089147	0.08697689	-0.01169953
C	2.61803263	-1.30394665	0.09114456
N	-1.69019061	0.85173796	-0.14698980
C	-3.09109420	0.53465515	0.13800379
H	3.39574199	-2.04479222	0.16023975
C	-1.80045645	-1.45465181	0.06836479
C	-3.19872731	-0.93155911	-0.25596905
H	-3.38388587	-1.00436388	-1.32086651
H	-3.99578632	-1.44209020	0.26457377
H	-3.23827439	0.68404039	1.20924003
H	-3.74824671	1.20442442	-0.39946316

H	-1.45964395	-2.24393686	-0.58890596
H	0.68492194	-2.53863802	0.15392927
C	-1.25582628	2.22533573	0.01771029
H	-0.76997914	2.35095177	0.98343703
H	-0.57147548	2.50948818	-0.76789577
H	-2.13624308	2.85100262	-0.02640629
H	1.28605642	1.77098012	-0.16986303
C	4.09392302	0.79748396	-0.03456902
H	4.67466533	0.61784075	0.86886601
H	4.72062666	0.47911716	-0.86610348
H	3.96298185	1.86842143	-0.12364759
H	-1.71567525	-1.85214543	1.07951914

Time = 11.50 fs
E(CASSCF/6-31G(d)) = -479.5535131

C	-0.98926538	-0.25306943	-0.06675921
C	0.58026036	-0.33207125	-0.03184947
C	1.14572986	-1.57181028	0.08243876
C	1.44781761	0.71997101	-0.09028989
C	2.80174602	0.08870357	-0.01175996
C	2.61964842	-1.30671532	0.09150568
N	-1.69191111	0.85326023	-0.14542191
C	-3.09138663	0.53525387	0.13723582
H	3.40039054	-2.04291067	0.15964544
C	-1.80143384	-1.45487616	0.06849484
C	-3.19966456	-0.93208933	-0.25585201
H	-3.38406988	-1.00574220	-1.32058376
H	-3.99722070	-1.44169372	0.26466032
H	-3.23998303	0.68370914	1.20825400
H	-3.74847433	1.20482585	-0.40017310
H	-1.45810752	-2.24177606	-0.59105973
H	0.67754131	-2.53231722	0.15350662
C	-1.25545958	2.22535157	0.01766138
H	-0.77046004	2.35434033	0.98252260
H	-0.57230946	2.51028628	-0.76788861
H	-2.13616277	2.85024765	-0.02757064
H	1.27868107	1.76819832	-0.17008448
C	4.09537405	0.79751212	-0.03460557
H	4.67640217	0.61935794	0.86863775
H	4.72242439	0.48042592	-0.86603470
H	3.96031592	1.86803414	-0.12389524
H	-1.71455485	-1.85356851	1.07895931

Time = 12.00 fs
E(CASSCF/6-31G(d)) = -479.5546226

C	-0.98811976	-0.25471809	-0.06748129
C	0.57794556	-0.33241229	-0.03202508
C	1.14753284	-1.57133541	0.08260356
C	1.44929326	0.71971385	-0.09042624
C	2.80224411	0.09085097	-0.01185957
C	2.62087149	-1.30987405	0.09189425
N	-1.69388503	0.85495307	-0.14386556
C	-3.09166939	0.53592513	0.13651276
H	3.40487369	-2.04090800	0.15901543
C	-1.80251329	-1.45520443	0.06862046
C	-3.20065594	-0.93261865	-0.25572824
H	-3.38432997	-1.00719730	-1.32039734
H	-3.99880592	-1.44140956	0.26483478
H	-3.24237170	0.68320574	1.20710163
H	-3.74888997	1.20510458	-0.40098257
H	-1.45684209	-2.23982136	-0.59346350
H	0.67020269	-2.52574589	0.15307104
C	-1.25508120	2.22531676	0.01764517
H	-0.77103857	2.35806552	0.98136357
H	-0.57331654	2.51113175	-0.76788440
H	-2.13589603	2.84966657	-0.02878500
H	1.27126262	1.76541702	-0.17031480
C	4.09695855	0.79759582	-0.03464456
H	4.67814705	0.62087749	0.86851451
H	4.72423651	0.48172231	-0.86606901
H	3.95762207	1.86787490	-0.12416284
H	-1.71372640	-1.85513809	1.07868416

Time = 12.50 fs
E(CASSCF/6-31G(d)) = -479.5562150

C	-0.98617843	-0.25639961	-0.06822475
C	0.57470840	-0.33270424	-0.03222662
C	1.14996627	-1.57099738	0.08279860
C	1.45141182	0.71939540	-0.09054833
C	2.80239152	0.09336120	-0.01199395
C	2.62170809	-1.31335894	0.09230578
N	-1.69607353	0.85677116	-0.14231993
C	-3.09195204	0.53666329	0.13583744
H	3.40920777	-2.03890171	0.15835886
C	-1.80368612	-1.45564060	0.06873700
C	-3.20169804	-0.93314117	-0.25559818
H	-3.38467588	-1.00872595	-1.32031513
H	-4.00052706	-1.44123157	0.26509903
H	-3.24544300	0.68254799	1.20580803
H	-3.74950190	1.20526465	-0.40190346
H	-1.45590085	-2.23807093	-0.59606387

H	0.66296633	-2.51911763	0.15263984
C	-1.25470300	2.22524069	0.01765882
H	-0.77167441	2.36208847	0.98003207
H	-0.57445340	2.51200138	-0.76793395
H	-2.13545478	2.84929635	-0.03004260
H	1.26390132	1.76275368	-0.17056013
C	4.09866864	0.79774395	-0.03468683
H	4.67989561	0.62239515	0.86851126
H	4.72605908	0.48299930	-0.86622132
H	3.95496950	1.86793600	-0.12444816
H	-1.71320025	-1.85684096	1.07866653

Time = 13.00 fs
E(CASSCF/6-31G(d)) = -479.5581329

C	-0.98349528	-0.25806529	-0.06899031
C	0.57061670	-0.33294280	-0.03245360
C	1.15298894	-1.57078428	0.08302208
C	1.45411905	0.71900486	-0.09065635
C	2.80220030	0.09616847	-0.01215793
C	2.62217024	-1.31709903	0.09273539
N	-1.69843247	0.85866501	-0.14078339
C	-3.09224584	0.53746154	0.13521123
H	3.41340926	-2.03701259	0.15768491
C	-1.80494129	-1.45618788	0.06884032
C	-3.20278755	-0.93365106	-0.25546268
H	-3.38511601	-1.01032312	-1.32033492
H	-4.00235891	-1.44114656	0.26544887
H	-3.24919058	0.68175848	1.20440730
H	-3.75031157	1.20530843	-0.40294513
H	-1.45533801	-2.23651600	-0.59879704
H	0.65590425	-2.51262348	0.15223021
C	-1.25433776	2.22513432	0.01769818
H	-0.77232088	2.36636491	0.97861165
H	-0.57567393	2.51286855	-0.76808856
H	-2.13485361	2.84917256	-0.03133635
H	1.25671101	1.76030071	-0.17082438
C	4.10049450	0.79796587	-0.03473327
H	4.68164138	0.62390748	0.86863761
H	4.72788584	0.48425136	-0.86650171
H	3.95243368	1.86819733	-0.12474780
H	-1.71298416	-1.85865547	1.07885821

Time = 13.50 fs
E(CASSCF/6-31G(d)) = -479.5602026

C	-0.98013568	-0.25966657	-0.06977802

C	0.56575379	-0.33312479	-0.03270511
C	1.15654782	-1.57068245	0.08327176
C	1.45734871	0.71853403	-0.09075104
C	2.80168899	0.09920136	-0.01234575
C	2.62227606	-1.32101910	0.09317803
N	-1.70091398	0.86058325	-0.13925382
C	-3.09256342	0.53831212	0.13463427
H	3.41748677	-2.03535514	0.15700212
C	-1.80626567	-1.45684819	0.06892680
C	-3.20392122	-0.93414271	-0.25532295
H	-3.38565733	-1.01198185	-1.32044459
H	-4.00426759	-1.44113542	0.26587482
H	-3.25359926	0.68086382	1.20293974
H	-3.75131316	1.20523642	-0.40411426
H	-1.45520670	-2.23514488	-0.60159316
H	0.64910465	-2.50643888	0.15185816
C	-1.25399857	2.22500983	0.01775810
H	-0.77292839	2.37084605	0.97719042
H	-0.57693281	2.51370300	-0.76839584
H	-2.13410868	2.84932780	-0.03265898
H	1.24981933	1.75811696	-0.17110866
C	4.10242453	0.79827099	-0.03478479
H	4.68337541	0.62541163	0.86889691
H	4.72970768	0.48547439	-0.86691446
H	3.95009419	1.86862923	-0.12505753
H	-1.71308359	-1.86055314	1.07919428

Time = 14.00 fs
E(CASSCF/6-31G(d)) = -479.5622495

C	-0.97617497	-0.26115719	-0.07058713
C	0.56021675	-0.33324832	-0.03297984
C	1.16057866	-1.57067798	0.08354507
C	1.46102399	0.71797849	-0.09083371
C	2.80088274	0.10238499	-0.01255122
C	2.62205003	-1.32504240	0.09362867
N	-1.70346799	0.86247486	-0.13772884
C	-3.09291847	0.53920635	0.13410570
H	3.42143377	-2.03402880	0.15631765
C	-1.80764434	-1.45762168	0.06899332
C	-3.20509579	-0.93461065	-0.25518061
H	-3.38630541	-1.01369326	-1.32062317
H	-4.00621284	-1.44117465	0.26636340
H	-3.25864512	0.67989366	1.20144863
H	-3.75249354	1.20504745	-0.40541482
H	-1.45555657	-2.23394685	-0.60438106
H	0.64267541	-2.50071295	0.15153768

C	-1.25369829	2.22488031	0.01783276
H	-0.77344828	2.37547937	0.97585370
H	-0.57818870	2.51447084	-0.76889577
H	-2.13323669	2.84979030	-0.03400295
H	1.24336676	1.75622347	-0.17141091
C	4.10444549	0.79866826	-0.03484231
H	4.68508623	0.62690509	0.86928573
H	4.73151260	0.48666555	-0.86745748
H	3.94803237	1.86919621	-0.12537258
H	-1.71350177	-1.86250095	1.07959881

Time = 14.50 fs
E(CASSCF/6-31G(d)) = -479.5641104

C	-0.97169701	-0.26249492	-0.07141603
C	0.55411435	-0.33331284	-0.03327610
C	1.16500688	-1.57075795	0.08383927
C	1.46505927	0.71733826	-0.09090632
C	2.79981325	0.10564298	-0.01276785
C	2.62152307	-1.32909299	0.09408245
N	-1.70604378	0.86429109	-0.13620602
C	-3.09332534	0.54013482	0.13362396
H	3.42522291	-2.03311091	0.15563676
C	-1.80906111	-1.45850631	0.06903722
C	-3.20630779	-0.93504962	-0.25503763
H	-3.38706458	-1.01544658	-1.32084260
H	-4.00815090	-1.44123823	0.26689879
H	-3.26429573	0.67887971	1.19997662
H	-3.75383276	1.20473922	-0.40684838
H	-1.45643138	-2.23291582	-0.60709285
H	0.63674538	-2.49556145	0.15128027
C	-1.25344894	2.22475941	0.01791609
H	-0.77383653	2.38020922	0.97467654
H	-0.57940691	2.51513473	-0.76961738
H	-2.13225391	2.85058249	-0.03536060
H	1.23750459	1.75460424	-0.17172600
C	4.10654272	0.79916566	-0.03490667
H	4.68676000	0.62838529	0.86979386
H	4.73328576	0.48782304	-0.86812213
H	3.94632874	1.86986099	-0.12568810
H	-1.71423971	-1.86446390	1.07999127

Time = 15.00 fs
E(CASSCF/6-31G(d)) = -479.5656445

C	-0.96679272	-0.26364309	-0.07226231
C	0.54756503	-0.33331904	-0.03359179

C	1.16974875	-1.57091138	0.08415150
C	1.46936195	0.71661805	-0.09097138
C	2.79851863	0.10889920	-0.01298902
C	2.62073254	-1.33309767	0.09453482
N	-1.70859130	0.86598721	-0.13468299
C	-3.09379863	0.54108747	0.13318714
H	3.42880279	-2.03265228	0.15496246
C	-1.81049891	-1.45949749	0.06905612
C	-3.20755344	-0.93545449	-0.25489622
H	-3.38793811	-1.01722938	-1.32107021
H	-4.01003762	-1.44129969	0.26746466
H	-3.27051033	0.67785474	1.19856209
H	-3.75530485	1.20430910	-0.40841468
H	-1.45786668	-2.23205336	-0.60966808
H	0.63146317	-2.49106397	0.15109460
C	-1.25326121	2.22466099	0.01800216
H	-0.77405716	2.38497750	0.97371712
H	-0.58056129	2.51565442	-0.77057630
H	-2.13117543	2.85171993	-0.03672407
H	1.23239116	1.75321170	-0.17204619
C	4.10870039	0.79976976	-0.03497862
H	4.68838098	0.62984935	0.87040475
H	4.73500999	0.48894568	-0.86889357
H	3.94506034	1.87058870	-0.12599949
H	-1.71529613	-1.86640760	1.08029328

Time = 15.50 fs
E(CASSCF/6-31G(d)) = -479.5667424

C	-0.96155875	-0.26457144	-0.07312279
C	0.54069484	-0.33326878	-0.03392452
C	1.17471297	-1.57112978	0.08447890
C	1.47383447	0.71582694	-0.09103198
C	2.79704290	0.11207927	-0.01320809
C	2.61972184	-1.33698739	0.09498163
N	-1.71106244	0.86752371	-0.13315750
C	-3.09435277	0.54205371	0.13279327
H	3.43209771	-2.03267558	0.15429545
C	-1.81194038	-1.46058790	0.06904777
C	-3.20882844	-0.93582029	-0.25475870
H	-3.38892871	-1.01902782	-1.32127189
H	-4.01183180	-1.44133427	0.26804611
H	-3.27724029	0.67685182	1.19723630
H	-3.75687910	1.20375536	-0.41011239
H	-1.45988758	-2.23137090	-0.61205740
H	0.62699288	-2.48726561	0.15098672
C	-1.25314404	2.22459882	0.01808557

H	-0.77408497	2.38972430	0.97301148
H	-0.58163526	2.51598756	-0.77177340
H	-2.13001439	2.85321026	-0.03808549
H	1.22818674	1.75197628	-0.17236189
C	4.11090171	0.80048535	-0.03505881
H	4.68993214	0.63129373	0.87109622
H	4.73666636	0.49003260	-0.86975121
H	3.94429818	1.87135036	-0.12630281
H	-1.71666698	-1.86830075	1.08043518

Time = 16.00 fs
E(CASSCF/6-31G(d)) = -479.5673278

C	-0.95609584	-0.26525687	-0.07399365
C	0.53363498	-0.33316491	-0.03427154
C	1.17980270	-1.57140724	0.08481863
C	1.47837661	0.71497770	-0.09109159
C	2.79543544	0.11511165	-0.01341850
C	2.61853964	-1.34069809	0.09541914
N	-1.71341212	0.86886724	-0.13162755
C	-3.09500162	0.54302254	0.13244060
H	3.43501134	-2.03317687	0.15363434
C	-1.81336838	-1.46176743	0.06900990
C	-3.21012792	-0.93614213	-0.25462739
H	-3.39003864	-1.02082678	-1.32141450
H	-4.01349770	-1.44132061	0.26863113
H	-3.28442922	0.67590328	1.19602007
H	-3.75852137	1.20307776	-0.41193966
H	-1.46250744	-2.23089074	-0.61422474
H	0.62350760	-2.48418211	0.15096033
C	-1.25310433	2.22458623	0.01816174
H	-0.77390722	2.39438838	0.97257024
H	-0.58262156	2.51609103	-0.77319471
H	-2.12878219	2.85505305	-0.03943687
H	1.22504744	1.75081789	-0.17266266
C	4.11312911	0.80131511	-0.03514772
H	4.69139621	0.63271384	0.87184207
H	4.73823534	0.49108263	-0.87067025
H	3.94410461	1.87212632	-0.12659506
H	-1.71834539	-1.87011738	1.08036141

Time = 16.50 fs
E(CASSCF/6-31G(d)) = -479.5673684

C	-0.95050758	-0.26568358	-0.07487054
C	0.52651930	-0.33301098	-0.03462991
C	1.18491784	-1.57174024	0.08516796

C	1.48288774	0.71408578	-0.09115400
C	2.79375006	0.11792875	-0.01361385
C	2.61723889	-1.34417128	0.09584408
N	-1.71559919	0.86999114	-0.13009131
C	-3.09575800	0.54398263	0.13212782
H	3.43743242	-2.03412961	0.15297609
C	-1.81476650	-1.46302325	0.06893999
C	-3.21144622	-0.93641523	-0.25450444
H	-3.39126985	-1.02261042	-1.32146891
H	-4.01500740	-1.44124248	0.26921212
H	-3.29201384	0.67503993	1.19492266
H	-3.76019584	1.20227869	-0.41389512
H	-1.46572647	-2.23064577	-0.61614896
H	0.62117998	-2.48180746	0.15101734
C	-1.25314684	2.22463570	0.01822713
H	-0.77352381	2.39890837	0.97237838
H	-0.58352092	2.51592255	-0.77481303
H	-2.12748829	2.85723983	-0.04077024
H	1.22311791	1.74965869	-0.17293840
C	4.11536455	0.80225945	-0.03524570
H	4.69275681	0.63410392	0.87261358
H	4.73969793	0.49209391	-0.87162296
H	3.94453117	1.87290822	-0.12687447
H	-1.72032087	-1.87183797	1.08003471

Time = 17.00 fs
E(CASSCF/6-31G(d)) = -479.5668952

C	-0.94489932	-0.26584286	-0.07574877
C	0.51948277	-0.33281110	-0.03499645
C	1.18995787	-1.57212706	0.08552424
C	1.48726969	0.71316795	-0.09122313
C	2.79204334	0.12046764	-0.01378802
C	2.61587509	-1.34735389	0.09625355
N	-1.71758704	0.87087554	-0.12854685
C	-3.09663344	0.54492243	0.13185402
H	3.43924440	-2.03549079	0.15231667
C	-1.81611950	-1.46433998	0.06883511
C	-3.21277697	-0.93663494	-0.25439171
H	-3.39262446	-1.02436205	-1.32141207
H	-4.01634217	-1.44108897	0.26978651
H	-3.29992393	0.67429125	1.19393982
H	-3.76186483	1.20136408	-0.41597800
H	-1.46953205	-2.23067755	-0.61782334
H	0.62016974	-2.48012663	0.15115864
C	-1.25327414	2.22475861	0.01827918
H	-0.77294791	2.40322296	0.97239518

H	-0.58434183	2.51544109	-0.77658862
H	-2.12614034	2.85975220	-0.04207788
H	1.22252311	1.74843900	-0.17318075
C	4.11758981	0.80331643	-0.03535289
H	4.69399952	0.63545682	0.87338116
H	4.74103690	0.49306349	-0.87258015
H	3.94561732	1.87370061	-0.12714059
H	-1.72257914	-1.87345019	1.07943854

Time = 17.50 fs
E(CASSCF/6-31G(d)) = -479.5659540

C	-0.93937655	-0.26573270	-0.07662344
C	0.51265875	-0.33256974	-0.03536793
C	1.19482446	-1.57256714	0.08588496
C	1.49142894	0.71224111	-0.09130284
C	2.79037333	0.12267080	-0.01393524
C	2.61450456	-1.35019823	0.09664500
N	-1.71934410	0.87150721	-0.12699218
C	-3.09763780	0.54583031	0.13161872
H	3.44033494	-2.03720803	0.15165164
C	-1.81741378	-1.46570021	0.06869177
C	-3.21411308	-0.93679677	-0.25429058
H	-3.39410484	-1.02606468	-1.32122915
H	-4.01749352	-1.44085571	0.27035735
H	-3.30808339	0.67368478	1.19305502
H	-3.76349107	1.20034403	-0.41818893
H	-1.47389821	-2.23103419	-0.61925492
H	0.62061425	-2.47912318	0.15138471
C	-1.25348675	2.22496485	0.01831636
H	-0.77220356	2.40727247	0.97255884
H	-0.58509739	2.51460936	-0.77847287
H	-2.12474493	2.86256346	-0.04335233
H	1.22336225	1.74712575	-0.17338389
C	4.11978676	0.80448186	-0.03546929
H	4.69511306	0.63676402	0.87411615
H	4.74223799	0.49398704	-0.87351292
H	3.94738880	1.87452053	-0.12739438
H	-1.72510157	-1.87494938	1.07857874

Time = 18.00 fs
E(CASSCF/6-31G(d)) = -479.5646432

C	-0.93404315	-0.26535719	-0.07748960
C	0.50617636	-0.33229154	-0.03574106
C	1.19942433	-1.57306034	0.08624776
C	1.49527915	0.71132107	-0.09139680

C	2.78879799	0.12448685	-0.01405018
C	2.61318252	-1.35266178	0.09701622
N	-1.72084424	0.87187916	-0.12542511
C	-3.09877903	0.54669464	0.13142176
H	3.44060650	-2.03922701	0.15097660
C	-1.81863758	-1.46708488	0.06850586
C	-3.21544689	-0.93689647	-0.25420191
H	-3.39571361	-1.02770115	-1.32091421
H	-4.01846260	-1.44054439	0.27093316
H	-3.31641117	0.67324597	1.19224017
H	-3.76503818	1.19923270	-0.42052993
H	-1.47878688	-2.23176646	-0.62046194
H	0.62261816	-2.47878818	0.15169600
C	-1.25378336	2.22526263	0.01833795
H	-0.77132346	2.41099995	0.97279173
H	-0.58580311	2.51339528	-0.78041168
H	-2.12330827	2.86563876	-0.04458663
H	1.22570281	1.74572058	-0.17354533
C	4.12193748	0.80574934	-0.03559473
H	4.69609035	0.63801562	0.87479235
H	4.74329119	0.49485861	-0.87439417
H	3.94985696	1.87539659	-0.12763809
H	-1.72786530	-1.87633771	1.07748254

Time = 18.50 fs
E(CASSCF/6-31G(d)) = -479.5630874

C	-0.92899982	-0.26472583	-0.07834237
C	0.50015841	-0.33198114	-0.03611260
C	1.20367182	-1.57360605	0.08661036
C	1.49874331	0.71042135	-0.09150830
C	2.78737327	0.12587128	-0.01412808
C	2.61196102	-1.35470717	0.09736524
N	-1.72206700	0.87199020	-0.12384316
C	-3.10006287	0.54750402	0.13126306
H	3.43998527	-2.04149767	0.15028809
C	-1.81978128	-1.46847405	0.06827250
C	-3.21677034	-0.93693014	-0.25412583
H	-3.39745378	-1.02925464	-1.32047139
H	-4.01926013	-1.44016289	0.27152735
H	-3.32482288	0.67299833	1.19145947
H	-3.76647223	1.19804901	-0.42300498
H	-1.48414869	-2.23292395	-0.62147127
H	0.62624547	-2.47912620	0.15209349
C	-1.25416113	2.22565803	0.01834383
H	-0.77034540	2.41435326	0.97300784
H	-0.58647402	2.51177396	-0.78234942

H	-2.12183719	2.86893667	-0.04577447
H	1.22957664	1.74426359	-0.17366632
C	4.12402471	0.80711067	-0.03572889
H	4.69692914	0.63920046	0.87538762
H	4.74419143	0.49567050	-0.87520000
H	3.95301814	1.87636601	-0.12787506
H	-1.73084273	-1.87762363	1.07619712

Time = 19.00 fs
E(CASSCF/6-31G(d)) = -479.5614399

C	-0.92434238	-0.26385279	-0.07917711
C	0.49471906	-0.33164316	-0.03647948
C	1.20749155	-1.57420241	0.08697063
C	1.50175599	0.70955208	-0.09164013
C	2.78615114	0.12678719	-0.01416484
C	2.61088682	-1.35630195	0.09769033
N	-1.72299762	0.87184428	-0.12224336
C	-3.10149277	0.54824739	0.13114232
H	3.43842917	-2.04397989	0.14958398
C	-1.82083752	-1.46984745	0.06798617
C	-3.21807522	-0.93689442	-0.25406179
H	-3.39932849	-1.03070877	-1.31991415
H	-4.01990457	-1.43972409	0.27215710
H	-3.33323227	0.67296381	1.19067216
H	-3.76776182	1.19681621	-0.42562004
H	-1.48992521	-2.23455003	-0.62231437
H	0.63151302	-2.48016041	0.15257889
C	-1.25461599	2.22615482	0.01833410
H	-0.76930908	2.41728641	0.97311990
H	-0.58712259	2.50972878	-0.78423263
H	-2.12034014	2.87241004	-0.04691042
H	1.23497747	1.74283445	-0.17375209
C	4.12603206	0.80855616	-0.03587133
H	4.69763243	0.64030612	0.87588482
H	4.74493919	0.49641318	-0.87591071
H	3.95685418	1.87747156	-0.12810942
H	-1.73400181	-1.87882002	1.07478538

Time = 19.50 fs
E(CASSCF/6-31G(d)) = -479.5598416

C	-0.92015884	-0.26275647	-0.07998936
C	0.48996079	-0.33128214	-0.03683880
C	1.21081951	-1.57484596	0.08732653
C	1.50426393	0.70871999	-0.09179448
C	2.78517895	0.12720603	-0.01415709

C	2.61000044	-1.35741907	0.09799004
N	-1.72362749	0.87145019	-0.12062262
C	-3.10306948	0.54891428	0.13105887
H	3.43593261	-2.04664735	0.14886338
C	-1.82180112	-1.47118534	0.06764082
C	-3.21935350	-0.93678653	-0.25400849
H	-3.40134058	-1.03204776	-1.31926381
H	-4.02041951	-1.43924470	0.27284189
H	-3.34155364	0.67316174	1.18983629
H	-3.76887903	1.19556046	-0.42838208
H	-1.49605109	-2.23667714	-0.62302307
H	0.63838949	-2.48193093	0.15315439
C	-1.25514297	2.22675404	0.01830898
H	-0.76825219	2.41976196	0.97304727
H	-0.58775627	2.50725347	-0.78601375
H	-2.11882812	2.87600794	-0.04799014
H	1.24186145	1.74154529	-0.17381129
C	4.12794413	0.81007490	-0.03602153
H	4.69820894	0.64131967	0.87627239
H	4.74554092	0.49707576	-0.87651162
H	3.96133262	1.87875714	-0.12834577
H	-1.73730715	-1.87994183	1.07332067

Time = 20.00 fs
E(CASSCF/6-31G(d)) = -479.5584421

C	-0.91652762	-0.26145874	-0.08077493
C	0.48597247	-0.33090266	-0.03718794
C	1.21360432	-1.57553126	0.08767617
C	1.50622714	0.70792821	-0.09197291
C	2.78449807	0.12710800	-0.01410230
C	2.60933498	-1.35803681	0.09826321
N	-1.72395395	0.87082086	-0.11897769
C	-3.10479107	0.54949508	0.13101130
H	3.43252868	-2.04948912	0.14812685
C	-1.82266901	-1.47246910	0.06723002
C	-3.22059767	-0.93660447	-0.25396394
H	-3.40349270	-1.03325680	-1.31854824
H	-4.02083182	-1.43874413	0.27360185
H	-3.34970389	0.67360946	1.18891291
H	-3.76979994	1.19431024	-0.43129888
H	-1.50245583	-2.23932347	-0.62362610
H	0.64679615	-2.48449339	0.15382249
C	-1.25573653	2.22745396	0.01826846
H	-0.76720747	2.42175253	0.97272243
H	-0.58837627	2.50435317	-0.78765402
H	-2.11731539	2.87967720	-0.04901061

H	1.25014971	1.74053250	-0.17385527
C	4.12974677	0.81165525	-0.03617885
H	4.69867273	0.64222805	0.87654459
H	4.74600890	0.49764644	-0.87699332
H	3.96640733	1.88026296	-0.12858874
H	-1.74072040	-1.88100457	1.07188159

Time = 20.50 fs
E(CASSCF/6-31G(d)) = -479.5573745

C	-0.91351520	-0.25998451	-0.08152997
C	0.48282704	-0.33050929	-0.03752459
C	1.21580793	-1.57625075	0.08801774
C	1.50761926	0.70717655	-0.09217632
C	2.78414301	0.12648270	-0.01399885
C	2.60891542	-1.35813937	0.09850898
N	-1.72398040	0.86997297	-0.11730514
C	-3.10665281	0.54998122	0.13099724
H	3.42828861	-2.05250947	0.14737628
C	-1.82344016	-1.47368196	0.06674737
C	-3.22180106	-0.93634719	-0.25392548
H	-3.40578667	-1.03432193	-1.31779912
H	-4.02116852	-1.43824259	0.27445579
H	-3.35760489	0.67432204	1.18786940
H	-3.77050432	1.19309493	-0.43437821
H	-1.50906651	-2.24248864	-0.62414520
H	0.65661020	-2.48791380	0.15458557
C	-1.25639073	2.22824980	0.01821209
H	-0.76620006	2.42324235	0.97209653
H	-0.58897691	2.50104482	-0.78912560
H	-2.11582006	2.88336382	-0.04997025
H	1.25973203	1.73994423	-0.17389713
C	4.13142728	0.81328514	-0.03634265
H	4.69904268	0.64301857	0.87670113
H	4.74636072	0.49811295	-0.87735156
H	3.97202001	1.88202112	-0.12884254
H	-1.74420135	-1.88202127	1.07054460

Time = 21.00 fs
E(CASSCF/6-31G(d)) = -479.5567419

C	-0.91117409	-0.25836133	-0.08225097
C	0.48057963	-0.33010680	-0.03784683
C	1.21740554	-1.57699501	0.08834961
C	1.50842745	0.70646237	-0.09240502
C	2.78414096	0.12532940	-0.01384605
C	2.60875855	-1.35771743	0.09872691

N	-1.72371629	0.86892652	-0.11560157
C	-3.10864713	0.55036547	0.13101317
H	3.42331795	-2.05572547	0.14661456
C	-1.82411542	-1.47480950	0.06618678
C	-3.22295818	-0.93601471	-0.25388998
H	-3.40822296	-1.03523021	-1.31704913
H	-4.02145402	-1.43775950	0.27541936
H	-3.36518569	0.67531218	1.18668250
H	-3.77097542	1.19194326	-0.43762703
H	-1.51580966	-2.24615111	-0.62459241
H	0.66767158	-2.49226036	0.15544528
C	-1.25709948	2.22913369	0.01813884
H	-0.76524569	2.42422831	0.97114323
H	-0.58954570	2.49735726	-0.79041279
H	-2.11436426	2.88701430	-0.05086905
H	1.27047223	1.73992465	-0.17395039
C	4.13297466	0.81495250	-0.03651224
H	4.69934175	0.64367942	0.87674653
H	4.74661858	0.49846321	-0.87758675
H	3.97810160	1.88405135	-0.12911064
H	-1.74770898	-1.88299999	1.06937712

Time = 21.50 fs
E(CASSCF/6-31G(d)) = -479.5566088

C	-0.90954147	-0.25661883	-0.08293476
C	0.47926623	-0.32970018	-0.03815317
C	1.21838563	-1.57775323	0.08867032
C	1.50865230	0.70578158	-0.09265880
C	2.78451119	0.12365754	-0.01364423
C	2.60887269	-1.35676890	0.09891698
N	-1.72317676	0.86770426	-0.11386354
C	-3.11076381	0.55064208	0.13105424
H	3.41774982	-2.05916247	0.14584523
C	-1.82469734	-1.47584004	0.06554297
C	-3.22406498	-0.93560821	-0.25385390
H	-3.41080037	-1.03596976	-1.31632917
H	-4.02170782	-1.43731205	0.27650343
H	-3.37238411	0.67659016	1.18534064
H	-3.77119972	1.19088187	-0.44105048
H	-1.52261258	-2.25026725	-0.62496869
H	0.67979243	-2.49759230	0.15640171
C	-1.25785658	2.23009481	0.01804690
H	-0.76434957	2.42472040	0.96986098
H	-0.59006398	2.49333079	-0.79151229
H	-2.11297410	2.89057714	-0.05170863
H	1.28221469	1.74059579	-0.17402747

C	4.13437980	0.81664546	-0.03668691
H	4.69959579	0.64420043	0.87668889
H	4.74680809	0.49868617	-0.87770298
H	3.98457361	1.88635742	-0.12939537
H	-1.75120212	-1.88394177	1.06843164

Time = 22.00 fs
E(CASSCF/6-31G(d)) = -479.5569828

C	-0.90863627	-0.25478894	-0.08357858
C	0.47890171	-0.32929479	-0.03844253
C	1.21874822	-1.57851405	0.08897861
C	1.50830641	0.70513019	-0.09293705
C	2.78526591	0.12148653	-0.01339470
C	2.60925941	-1.35529969	0.09907975
N	-1.72238295	0.86633181	-0.11208795
C	-3.11298998	0.55080716	0.13111428
H	3.41173525	-2.06284699	0.14507156
C	-1.82519009	-1.47676492	0.06481183
C	-3.22511908	-0.93513024	-0.25381349
H	-3.41351610	-1.03652994	-1.31566584
H	-4.02194326	-1.43691449	0.27771325
H	-3.37914987	0.67816358	1.18384572
H	-3.77116759	1.18993423	-0.44465144
H	-1.52940510	-2.25477300	-0.62526408
H	0.69276927	-2.50394689	0.15745253
C	-1.25865579	2.23111949	0.01793380
H	-0.76350588	2.42474269	0.96827353
H	-0.59050795	2.48901707	-0.79243293
H	-2.11167903	2.89400462	-0.05249223
H	1.29479115	1.74203915	-0.17413801
C	4.13563565	0.81835261	-0.03686597
H	4.69983158	0.64457323	0.87653833
H	4.74695635	0.49877232	-0.87770673
H	3.99134954	1.88892442	-0.12969766
H	-1.75464140	-1.88483971	1.06774132

Time = 22.50 fs
E(CASSCF/6-31G(d)) = -479.5578646

C	-0.90846087	-0.25290500	-0.08418009
C	0.47948040	-0.32889623	-0.03871435
C	1.21850589	-1.57926668	0.08927361
C	1.50741529	0.70450515	-0.09323882
C	2.78640878	0.11884658	-0.01309985
C	2.60991223	-1.35332492	0.09921638
N	-1.72136104	0.86483672	-0.11027166

C	-3.11531050	0.55085863	0.13118571
H	3.40543418	-2.06680082	0.14429648
C	-1.82559927	-1.47757832	0.06399088
C	-3.22611983	-0.93458463	-0.25376505
H	-3.41636429	-1.03690093	-1.31507843
H	-4.02216510	-1.43657615	0.27904669
H	-3.38544247	0.68003742	1.18221201
H	-3.77087067	1.18911863	-0.44842850
H	-1.53611922	-2.25958350	-0.62545806
H	0.70639087	-2.51132727	0.15859203
C	-1.25949083	2.23219151	0.01779623
H	-0.76269970	2.42433067	0.96642727
H	-0.59085157	2.48447603	-0.79319303
H	-2.11051108	2.89725165	-0.05322453
H	1.30802426	1.74428268	-0.17428802
C	4.13673752	0.82006304	-0.03704872
H	4.70007666	0.64479251	0.87630603
H	4.74709173	0.49871497	-0.87760609
H	3.99833664	1.89171892	-0.13001702
H	-1.75798866	-1.88567681	1.06731555

Time = 23.00 fs
E(CASSCF/6-31G(d)) = -479.5591657

C	-0.90900066	-0.25100198	-0.08473745
C	0.48097623	-0.32851041	-0.03896850
C	1.21768239	-1.58000186	0.08955490
C	1.50601632	0.70390540	-0.09356295
C	2.78793531	0.11577863	-0.01276315
C	2.61081766	-1.35086986	0.09932868
N	-1.72014180	0.86324829	-0.10841170
C	-3.11770828	0.55079638	0.13125968
H	3.39900429	-2.07103362	0.14352206
C	-1.82593185	-1.47827708	0.06307948
C	-3.22706834	-0.93397658	-0.25370504
H	-3.41933625	-1.03707437	-1.31457770
H	-4.02236984	-1.43630172	0.28049446
H	-3.39123306	0.68221374	1.18046676
H	-3.77030307	1.18844776	-0.45237562
H	-1.54268862	-2.26459751	-0.62552255
H	0.72044982	-2.51968860	0.15981011
C	-1.26035519	2.23329276	0.01763024
H	-0.76190804	2.42353101	0.96438842
H	-0.59106825	2.47977500	-0.79381859
H	-2.10950348	2.90027738	-0.05391139
H	1.32173216	1.74728881	-0.17447887
C	4.13768319	0.82176629	-0.03723445

H	4.70035783	0.64485661	0.87600270
H	4.74724224	0.49851117	-0.87740960
H	4.00543682	1.89468967	-0.13035154
H	-1.76120728	-1.88642691	1.06713977

Time = 23.50 fs
E(CASSCF/6-31G(d)) = -479.5607665

C	-0.91022455	-0.24911612	-0.08524952
C	0.48334352	-0.32814371	-0.03920526
C	1.21631229	-1.58071266	0.08982258
C	1.50415868	0.70333239	-0.09390810
C	2.78983284	0.11233453	-0.01238919
C	2.61195579	-1.34797086	0.09941920
N	-1.71876018	0.86159728	-0.10650519
C	-3.12016472	0.55062238	0.13132622
H	3.39259005	-2.07553437	0.14274882
C	-1.82619605	-1.47886039	0.06207902
C	-3.22796742	-0.93331263	-0.25363020
H	-3.42242085	-1.03704322	-1.31416491
H	-4.02254661	-1.43609126	0.28204081
H	-3.39650420	0.68469193	1.17864763
H	-3.76946083	1.18792835	-0.45648171
H	-1.54904919	-2.26970307	-0.62542561
H	0.73475090	-2.52893013	0.16109192
C	-1.26124202	2.23440381	0.01743138
H	-0.76110278	2.42239955	0.96223727
H	-0.59113367	2.47498632	-0.79434005
H	-2.10868931	2.90304514	-0.05455963
H	1.33573099	1.75095131	-0.17470695
C	4.13847299	0.82345228	-0.03742241
H	4.70069935	0.64476763	0.87563705
H	4.74743376	0.49816204	-0.87712470
H	4.01254808	1.89776953	-0.13069803
H	-1.76426320	-1.88705611	1.06717741

Time = 24.00 fs
E(CASSCF/6-31G(d)) = -479.5625226

C	-0.91208718	-0.24728445	-0.08571598
C	0.48651893	-0.32780297	-0.03942524
C	1.21444083	-1.58139507	0.09007737
C	1.50190314	0.70278996	-0.09427273
C	2.79208048	0.10857694	-0.01198367
C	2.61330033	-1.34467596	0.09949123
N	-1.71725431	0.85991539	-0.10454911
C	-3.12266014	0.55034067	0.13137448

H	3.38631486	-2.08026531	0.14197562
C	-1.82640131	-1.47932918	0.06099297
C	-3.22882137	-0.93260057	-0.25353779
H	-3.42560439	-1.03680164	-1.31383136
H	-4.02267826	-1.43594016	0.28366423
H	-3.40124777	0.68746881	1.17679911
H	-3.76834091	1.18756095	-0.46073002
H	-1.55513862	-2.27478232	-0.62513505
H	0.74911448	-2.53889241	0.16241763
C	-1.26214394	2.23550450	0.01719496
H	-0.76025462	2.42099803	0.96006092
H	-0.59102901	2.47018412	-0.79478836
H	-2.10810007	2.90552141	-0.05517670
H	1.34983481	1.75510097	-0.17496415
C	4.13910987	0.82511100	-0.03761180
H	4.70112230	0.64453190	0.87521484
H	4.74768918	0.49767337	-0.87675699
H	4.01956599	1.90087968	-0.13105230
H	-1.76712506	-1.88752429	1.06737299

Time = 24.50 fs
E(CASSCF/6-31G(d)) = -479.5642560

C	-0.91453156	-0.24554443	-0.08613749
C	0.49042449	-0.32749554	-0.03962929
C	1.21212324	-1.58204823	0.09032069
C	1.49932134	0.70228387	-0.09465517
C	2.79464937	0.10457874	-0.01155333
C	2.61481912	-1.34104496	0.09954875
N	-1.71566464	0.85823470	-0.10254027
C	-3.12517431	0.54995729	0.13139305
H	3.38027602	-2.08515858	0.14119965
C	-1.82655820	-1.47968551	0.05982677
C	-3.22963572	-0.93184928	-0.25342559
H	-3.42887082	-1.03634545	-1.31355942
H	-4.02274332	-1.43584011	0.28533883
H	-3.40546435	0.69053827	1.17496959
H	-3.76694171	1.18734013	-0.46509802
H	-1.56089554	-2.27971756	-0.62462305
H	0.76337784	-2.54935786	0.16376260
C	-1.26305288	2.23657474	0.01691627
H	-0.75933635	2.41939211	0.95794647
H	-0.59074287	2.46544239	-0.79519226
H	-2.10776381	2.90767569	-0.05577024
H	1.36385520	1.75951658	-0.17523883
C	4.13959944	0.82673233	-0.03780176
H	4.70164368	0.64416022	0.87473831

H	4.74802747	0.49705603	-0.87630972
H	4.02638492	1.90393439	-0.13140959
H	-1.76976397	-1.88778810	1.06765747

Time = 25.00 fs
E(CASSCF/6-31G(d)) = -479.5657769

C	-0.91749084	-0.24393367	-0.08651563
C	0.49496949	-0.32722921	-0.03981852
C	1.20942370	-1.58267421	0.09055457
C	1.49649424	0.70182118	-0.09505355
C	2.79750396	0.10042205	-0.01110591
C	2.61647501	-1.33714908	0.09959643
N	-1.71403328	0.85658729	-0.10047555
C	-3.12768672	0.54948016	0.13137065
H	3.37454150	-2.09011598	0.14041645
C	-1.82667822	-1.47993197	0.05858758
C	-3.23041690	-0.93106846	-0.25329204
H	-3.43220201	-1.03567304	-1.31332445
H	-4.02271851	-1.43578135	0.28703671
H	-3.40916285	0.69389057	1.17320762
H	-3.76526489	1.18725507	-0.46955892
H	-1.56625896	-2.28439722	-0.62387012
H	0.77739519	-2.56005644	0.16509796
C	-1.26395992	2.23759499	0.01659115
H	-0.75832569	2.41764961	0.95597479
H	-0.59027184	2.46083369	-0.79557708
H	-2.10770354	2.90948187	-0.05634786
H	1.37760165	1.76393992	-0.17551695
C	4.13994974	0.82830580	-0.03799131
H	4.70227582	0.64366793	0.87420598
H	4.74846296	0.49632598	-0.87578361
H	4.03289923	1.90684601	-0.13176492
H	-1.77215349	-1.88780357	1.06795467

Time = 25.50 fs
E(CASSCF/6-31G(d)) = -479.5669409

C	-0.92089072	-0.24248948	-0.08685307
C	0.50005317	-0.32701226	-0.03999410
C	1.20641456	-1.58327744	0.09078161
C	1.49351107	0.70140917	-0.09546580
C	2.80060287	0.09619697	-0.01064993
C	2.61822676	-1.33306991	0.09963950
N	-1.71240338	0.85500523	-0.09835194
C	-3.13017685	0.54891920	0.13129655
H	3.36915383	-2.09501153	0.13962056

C	-1.82677370	-1.48007108	0.05728413
C	-3.23117193	-0.93026850	-0.25313621
H	-3.43557812	-1.03478488	-1.31309681
H	-4.02258143	-1.43575278	0.28872909
H	-3.41235870	0.69751209	1.17155746
H	-3.76331457	1.18729038	-0.47408121
H	-1.57116883	-2.28872079	-0.62286858
H	0.79102997	-2.57068340	0.16639206
C	-1.26485521	2.23854654	0.01621625
H	-0.75720992	2.41583799	0.95421254
H	-0.58962341	2.45642652	-0.79596108
H	-2.10793586	2.91091573	-0.05691663
H	1.39087968	1.76810130	-0.17578387
C	4.14017123	0.82982036	-0.03817937
H	4.70302622	0.64307463	0.87361308
H	4.74900524	0.49550405	-0.87517714
H	4.03900503	1.90953124	-0.13211357
H	-1.77427027	-1.88752951	1.06818755

Time = 26.00 fs
E(CASSCF/6-31G(d)) = -479.5676169

C	-0.92465244	-0.24124803	-0.08715355
C	0.50556863	-0.32685342	-0.04015731
C	1.20317478	-1.58386378	0.09100488
C	1.49046693	0.70105414	-0.09588960
C	2.80390017	0.09199907	-0.01019454
C	2.62002971	-1.32889687	0.09968366
N	-1.71081805	0.85351969	-0.09616649
C	-3.13262473	0.54828604	0.13116122
H	3.36413309	-2.09969840	0.13880572
C	-1.82685755	-1.48010494	0.05592614
C	-3.23190801	-0.92946016	-0.25295779
H	-3.43897894	-1.03368416	-1.31284485
H	-4.02231380	-1.43574466	0.29038941
H	-3.41507279	0.70138585	1.17005380
H	-3.76109917	1.18742774	-0.47863176
H	-1.57556646	-2.29260476	-0.62162530
H	0.80415260	-2.58091730	0.16761201
C	-1.26572790	2.23941228	0.01578955
H	-0.75598857	2.41402248	0.95270593
H	-0.58881582	2.45228423	-0.79635482
H	-2.10846986	2.91195610	-0.05748307
H	1.40349423	1.77174115	-0.17602610
C	4.14027650	0.83126428	-0.03836477
H	4.70389690	0.64240358	0.87295203
H	4.74965842	0.49461521	-0.87448686

H	4.04460157	1.91191598	-0.13245145
H	-1.77609431	-1.88693116	1.06828664

Time = 26.50 fs
E(CASSCF/6-31G(d)) = -479.5677290

C	-0.92869346	-0.24024417	-0.08742182
C	0.51140360	-0.32676169	-0.04030944
C	1.19978794	-1.58443965	0.09122776
C	1.48746015	0.70076103	-0.09632246
C	2.80734762	0.08792728	-0.00974925
C	2.62183774	-1.32472528	0.09973489
N	-1.70932017	0.85216107	-0.09391680
C	-3.13501066	0.54759403	0.13095680
H	3.35948511	-2.10401769	0.13796549
C	-1.82694305	-1.48003471	0.05452416
C	-3.23263221	-0.92865425	-0.25275702
H	-3.44238349	-1.03237726	-1.31253740
H	-4.02190274	-1.43574905	0.29199413
H	-3.41733168	0.70548905	1.16872013
H	-3.75863235	1.18764607	-0.48317505
H	-1.57939529	-2.29598441	-0.62016298
H	0.81663365	-2.59043918	0.16872542
C	-1.26656644	2.24017642	0.01531060
H	-0.75467433	2.41226584	0.95147911
H	-0.58787725	2.44846449	-0.79676178
H	-2.10930599	2.91258438	-0.05805270
H	1.41525022	1.77463094	-0.17623251
C	4.14027986	0.83262504	-0.03854622
H	4.70488559	0.64168156	0.87221399
H	4.75042232	0.49368824	-0.87370892
H	4.04959297	1.91394042	-0.13277555
H	-1.77760968	-1.88598195	1.06819349

Time = 27.00 fs
E(CASSCF/6-31G(d)) = -479.5672648

C	-0.93293000	-0.23951054	-0.08766359
C	0.51744399	-0.32674628	-0.04045171
C	1.19634047	-1.58501078	0.09145377
C	1.48458969	0.70053232	-0.09676165
C	2.81089581	0.08408084	-0.00932366
C	2.62360396	-1.32065280	0.09979924
N	-1.70795138	0.85095823	-0.09160102
C	-3.13731568	0.54685803	0.13067760
H	3.35520890	-2.10780999	0.13709387
C	-1.82704355	-1.47986059	0.05308913

C	-3.23335113	-0.92786139	-0.25253465
H	-3.44577123	-1.03087411	-1.31214689
H	-4.02134332	-1.43576152	0.29352489
H	-3.41916649	0.70979369	1.16756536
H	-3.75593413	1.18792332	-0.48767589
H	-1.58260191	-2.29881669	-0.61852053
H	0.82834090	-2.59895433	0.16970219
C	-1.26735880	2.24082498	0.01478077
H	-0.75329376	2.41062719	0.95053186
H	-0.58684466	2.44501842	-0.79717903
H	-2.11043575	2.91278519	-0.05863001
H	1.42595713	1.77659230	-0.17639571
C	4.14019700	0.83388945	-0.03872234
H	4.70598571	0.64093803	0.87139009
H	4.75129250	0.49275463	-0.87284006
H	4.05388979	1.91556230	-0.13308413
H	-1.77880486	-1.88466626	1.06786674

Time = 27.50 fs
E(CASSCF/6-31G(d)) = -479.5662535

C	-0.93727769	-0.23907675	-0.08788537
C	0.52357513	-0.32681636	-0.04058528
C	1.19291957	-1.58558115	0.09168636
C	1.48195228	0.70036754	-0.09720428
C	2.81449587	0.08055639	-0.00892714
C	2.62528224	-1.31677626	0.09988266
N	-1.70675149	0.84993795	-0.08921818
C	-3.13952147	0.54609425	0.13032039
H	3.35130384	-2.11092574	0.13618597
C	-1.82717232	-1.47958174	0.05163191
C	-3.23407070	-0.92709174	-0.25229171
H	-3.44912257	-1.02918908	-1.31165247
H	-4.02064023	-1.43578260	0.29496979
H	-3.42061382	0.71426536	1.16658448
H	-3.75303335	1.18823783	-0.49210088
H	-1.58513695	-2.30108126	-0.61675298
H	0.83913799	-2.60620916	0.17051615
C	-1.26809286	2.24134566	0.01420331
H	-0.75188514	2.40916266	0.94984267
H	-0.58576046	2.44199134	-0.79760035
H	-2.11184170	2.91254845	-0.05921835
H	1.43543369	1.77750913	-0.17651270
C	4.14004454	0.83504385	-0.03889170
H	4.70718667	0.64020418	0.87047283
H	4.75226055	0.49184746	-0.87187872
H	4.05741050	1.91675848	-0.13337687

H	-1.77967300	-1.88298116	1.06728532

Time = 28.00 fs
E(CASSCF/6-31G(d)) = -479.5648264

C	-0.94165500	-0.23896804	-0.08809446
C	0.52968530	-0.32698093	-0.04071123
C	1.18961179	-1.58615191	0.09192883
C	1.47964094	0.70026229	-0.09764728
C	2.81809946	0.07744528	-0.00856865
C	2.62682739	-1.31318798	0.09999078
N	-1.70575724	0.84912396	-0.08676794
C	-3.14161078	0.54532000	0.12988458
H	3.34777959	-2.11323991	0.13523839
C	-1.82734202	-1.47919651	0.05016300
C	-3.23479597	-0.92635487	-0.25202960
H	-3.45241961	-1.02733976	-1.31104099
H	-4.01980738	-1.43581686	0.29632361
H	-3.42171261	0.71886498	1.16575480
H	-3.74996489	1.18856910	-0.49641942
H	-1.58695755	-2.30277822	-0.61492742
H	0.84888146	-2.61201208	0.17114658
C	-1.26875681	2.24172820	0.01358325
H	-0.75049882	2.40792264	0.94936781
H	-0.58467301	2.43941988	-0.79801529
H	-2.11349781	2.91186668	-0.05982010
H	1.44351120	1.77734291	-0.17658591
C	4.13983975	0.83607445	-0.03905284
H	4.70847555	0.63951230	0.86945818
H	4.75331581	0.49100044	-0.87082695
H	4.06008372	1.91752711	-0.13365497
H	-1.78021245	-1.88093649	1.06644978

Time = 28.50 fs
E(CASSCF/6-31G(d)) = -479.5631147

C	-0.94598398	-0.23920437	-0.08829885
C	0.53566730	-0.32724852	-0.04083055
C	1.18650089	-1.58672070	0.09218416
C	1.47774224	0.70020808	-0.09808742
C	2.82165997	0.07483054	-0.00825644
C	2.62819654	-1.30997234	0.10012873
N	-1.70500159	0.84853621	-0.08425073
C	-3.14356750	0.54455350	0.12937226
H	3.34466115	-2.11466138	0.13424975
C	-1.82756455	-1.47870275	0.04869225
C	-3.23553098	-0.92565956	-0.25174992

H	-3.45564649	-1.02534726	-1.31030853
H	-4.01886775	-1.43587292	0.29758781
H	-3.42250469	0.72354853	1.16503844
H	-3.74677027	1.18889868	-0.50060460
H	-1.58802881	-2.30392555	-0.61311990
H	0.85742182	-2.61624486	0.17157938
C	-1.26933965	2.24196424	0.01292719
H	-0.74919350	2.40695221	0.94904726
H	-0.58363343	2.43733221	-0.79841215
H	-2.11537045	2.91073629	-0.06043661
H	1.45003831	1.77613661	-0.17662328
C	4.13960007	0.83696776	-0.03920435
H	4.70983792	0.63889492	0.86834708
H	4.75444629	0.49024675	-0.86969162
H	4.06184976	1.91788676	-0.13392110
H	-1.78042691	-1.87855478	1.06538172

Time = 29.00 fs
E(CASSCF/6-31G(d)) = -479.5612766

C	-0.95019191	-0.23979894	-0.08850701
C	0.54142052	-0.32762684	-0.04094421
C	1.18366611	-1.58728125	0.09245493
C	1.47633425	0.70019222	-0.09852136
C	2.82513293	0.07278386	-0.00799778
C	2.62934998	-1.30720238	0.10030093
N	-1.70451250	0.84818963	-0.08166765
C	-3.14537688	0.54381349	0.12878792
H	3.34199185	-2.11514024	0.13322062
C	-1.82785067	-1.47809830	0.04722846
C	-3.23627880	-0.92501371	-0.25145437
H	-3.45879001	-1.02323617	-1.30946105
H	-4.01785237	-1.43596339	0.29877043
H	-3.42303453	0.72826831	1.16438504
H	-3.74349772	1.18921080	-0.50463473
H	-1.58832521	-2.30455587	-0.61141114
H	0.86460718	-2.61886854	0.17180739
C	-1.26983183	2.24204749	0.01224289
H	-0.74803169	2.40629103	0.94881113
H	-0.58269227	2.43574808	-0.79878086
H	-2.11741964	2.90915901	-0.06106847
H	1.45488536	1.77401226	-0.17663790
C	4.13934276	0.83771117	-0.03934491
H	4.71125855	0.63838399	0.86714647
H	4.75563939	0.48961802	-0.86848534
H	4.06266193	1.91787376	-0.13417913
H	-1.78032527	-1.87587072	1.06412282

Time = 29.50 fs
E(CASSCF/6-31G(d)) = -479.5594618

C	-0.95421175	-0.24075737	-0.08872755
C	0.54685252	-0.32812219	-0.04105305
C	1.18118022	-1.58782295	0.09274311
C	1.47548379	0.70019806	-0.09894580
C	2.82847691	0.07136294	-0.00779866
C	2.63025255	-1.30493716	0.10051088
N	-1.70431231	0.84809322	-0.07902081
C	-3.14702556	0.54311896	0.12813811
H	3.33983256	-2.11467064	0.13215392
C	-1.82820997	-1.47738167	0.04577929
C	-3.23704153	-0.92442417	-0.25114464
H	-3.46183946	-1.02103526	-1.30851475
H	-4.01679920	-1.43610420	0.29988500
H	-3.42335085	0.73297267	1.16373817
H	-3.74020291	1.18949308	-0.50849352
H	-1.58783172	-2.30471184	-0.60988275
H	0.87028760	-2.61992372	0.17183073
C	-1.27022572	2.24197368	0.01153892
H	-0.74707405	2.40597447	0.94858870
H	-0.58189562	2.43467954	-0.79911679
H	-2.11960097	2.90714448	-0.06171557
H	1.45794906	1.77116174	-0.17664708
C	4.13908439	0.83829344	-0.03947334
H	4.71272189	0.63800999	0.86586998
H	4.75688240	0.48914339	-0.86722688
H	4.06248762	1.91753820	-0.13443375
H	-1.77992096	-1.87292965	1.06273058

Time = 30.00 fs
E(CASSCF/6-31G(d)) = -479.5578548

C	-0.95798392	-0.24207652	-0.08896912
C	0.55188053	-0.32873921	-0.04115796
C	1.17910865	-1.58833124	0.09305015
C	1.47524610	0.70020540	-0.09935745
C	2.83165299	0.07061006	-0.00766373
C	2.63087397	-1.30322001	0.10076115
N	-1.70441686	0.84824925	-0.07631300
C	-3.14850183	0.54248882	0.12743102
H	3.33826228	-2.11329339	0.13105445
C	-1.82865051	-1.47655261	0.04435113
C	-3.23782043	-0.92389681	-0.25082241
H	-3.46478679	-1.01877600	-1.30749421

H	-4.01575006	-1.43631245	0.30094897
H	-3.42350468	0.73760851	1.16303737
H	-3.73694565	1.18973615	-0.51217004
H	-1.58654598	-2.30444017	-0.60861071
H	0.87431793	-2.61952985	0.17165651
C	-1.27051603	2.24174066	0.01082421
H	-0.74637659	2.40603198	0.94831244
H	-0.58128466	2.43412950	-0.79942039
H	-2.12186730	2.90470884	-0.06237742
H	1.45915338	1.76783709	-0.17667164
C	4.13884072	0.83870541	-0.03958866
H	4.71421317	0.63780137	0.86453845
H	4.75816362	0.48884880	-0.86594170
H	4.06130982	1.91694011	-0.13469017
H	-1.77923203	-1.86978522	1.06127380

Time = 30.50 fs
E(CASSCF/6-31G(d)) = -479.5566008

C	-0.96145831	-0.24374355	-0.08924028
C	0.55643418	-0.32948048	-0.04125978
C	1.17750819	-1.58878815	0.09337699
C	1.47566334	0.70019150	-0.09975322
C	2.83462467	0.07054994	-0.00759610
C	2.63118962	-1.30207659	0.10105318
N	-1.70483439	0.84865215	-0.07354761
C	-3.14979606	0.54194137	0.12667584
H	3.33737366	-2.11109303	0.12992866
C	-1.82917873	-1.47561272	0.04294911
C	-3.23861598	-0.92343640	-0.25048936
H	-3.46762613	-1.01649224	-1.30643032
H	-4.01474768	-1.43660429	0.30198165
H	-3.42354854	0.74212265	1.16222308
H	-3.73378740	1.18993335	-0.51565769
H	-1.58447959	-2.30378544	-0.60766031
H	0.87656265	-2.61787653	0.17129829
C	-1.27070013	2.24134856	0.01010748
H	-0.74598714	2.40648628	0.94792405
H	-0.58089434	2.43409088	-0.79969825
H	-2.12417052	2.90187475	-0.06305325
H	1.45845143	1.76433136	-0.17673445
C	4.13862638	0.83894056	-0.03969017
H	4.71571902	0.63778406	0.86317999
H	4.75947303	0.48875645	-0.86466179
H	4.05912837	1.91614413	-0.13495355
H	-1.77828022	-1.86649644	1.05982581

Time = 31.00 fs
E(CASSCF/6-31G(d)) = -479.5557843

C	-0.96459308	-0.24573593	-0.08954909
C	0.56045454	-0.33034603	-0.04135946
C	1.17642591	-1.58917346	0.09372401
C	1.47676391	0.70013274	-0.10013028
C	2.83735833	0.07118973	-0.00759727
C	2.63118150	-1.30151546	0.10138742
N	-1.70556564	0.84928851	-0.07072873
C	-3.15090062	0.54149428	0.12588236
H	3.33726609	-2.10819084	0.12878466
C	-1.82979901	-1.47456618	0.04157698
C	-3.23942837	-0.92304689	-0.25014713
H	-3.47035435	-1.01421978	-1.30535880
H	-4.01383373	-1.43699462	0.30300303
H	-3.42353817	0.74646383	1.16124313
H	-3.73079217	1.19008065	-0.51895579
H	-1.58165837	-2.30278750	-0.60708216
H	0.87690441	-2.61520359	0.17077418
C	-1.27077828	2.24079974	0.00939696
H	-0.74594000	2.40735477	0.94738059
H	-0.58074997	2.43454814	-0.79996494
H	-2.12646458	2.89867489	-0.06374240
H	1.45582683	1.76094844	-0.17685828
C	4.13845467	0.83899564	-0.03977748
H	4.71722729	0.63798119	0.86182886
H	4.76080201	0.48888461	-0.86342461
H	4.05595920	1.91521277	-0.13522855
H	-1.77709060	-1.86312586	1.05846040

Time = 31.50 fs
E(CASSCF/6-31G(d)) = -479.5555077

C	-0.96735721	-0.24802118	-0.08990314
C	0.56389736	-0.33133322	-0.04145790
C	1.17589803	-1.58946627	0.09409125
C	1.47856158	0.70000669	-0.10048631
C	2.83982295	0.07251813	-0.00766711
C	2.63083890	-1.30152793	0.10176319
N	-1.70660321	0.85013670	-0.06786095
C	-3.15181033	0.54116392	0.12506022
H	3.33803741	-2.10473505	0.12763100
C	-1.83051376	-1.47342005	0.04023747
C	-3.24025741	-0.92273119	-0.24979736
H	-3.47296976	-1.01199551	-1.30431682
H	-4.01304523	-1.43749393	0.30403125

H	-3.42353016	0.75058415	1.16005578
H	-3.72802136	1.19017584	-0.52206661
H	-1.57812346	-2.30147521	-0.60690845
H	0.87524815	-2.61178400	0.17010587
C	-1.27075364	2.24009888	0.00869995
H	-0.74625556	2.40864772	0.94665659
H	-0.58086908	2.43547544	-0.80024128
H	-2.12870618	2.89514891	-0.06444438
H	1.45129328	1.75797731	-0.17706371
C	4.13833751	0.83887117	-0.03985057
H	4.71872754	0.63841264	0.86052448
H	4.76214382	0.48924744	-0.86227210
H	4.05183616	1.91420185	-0.13551882
H	-1.77569051	-1.85973562	1.05724338

Time = 32.00 fs
E(CASSCF/6-31G(d)) = -479.5557900

C	-0.96972983	-0.25055772	-0.09030921
C	0.56673312	-0.33243649	-0.04155612
C	1.17594941	-1.58964717	0.09447849
C	1.48105574	0.69979451	-0.10081968
C	2.84199020	0.07450590	-0.00780383
C	2.63015907	-1.30208921	0.10217880
N	-1.70793180	0.85116738	-0.06494929
C	-3.15252280	0.54096515	0.12421843
H	3.33977283	-2.10088663	0.12647675
C	-1.83132330	-1.47218454	0.03893240
C	-3.24110299	-0.92249137	-0.24944172
H	-3.47547245	-1.00985694	-1.30334015
H	-4.01241235	-1.43810720	0.30508095
H	-3.42358239	0.75444093	1.15863372
H	-3.72553265	1.19021806	-0.52499512
H	-1.57393162	-2.29986574	-0.60715104
H	0.87152989	-2.60789606	0.16931640
C	-1.27063224	2.23925315	0.00802255
H	-0.74693830	2.41036894	0.94574620
H	-0.58126072	2.43683750	-0.80055381
H	-2.13085695	2.89134440	-0.06515914
H	1.44489587	1.75565752	-0.17736683
C	4.13828518	0.83857166	-0.03990980
H	4.72021018	0.63909476	0.85930942
H	4.76349259	0.48985492	-0.86124868
H	4.04681039	1.91315489	-0.13582654
H	-1.77410934	-1.85638560	1.05622744

Time = 32.50 fs

E(CASSCF/6-31G(d)) = -479.5566342

C	-0.97169980	-0.25329590	-0.09077327
C	0.56894632	-0.33364725	-0.04165511
C	1.17659261	-1.58970055	0.09488541
C	1.48423101	0.69948336	-0.10112961
C	2.84383508	0.07710780	-0.00800414
C	2.62914796	-1.30316162	0.10263174
N	-1.70952891	0.85234450	-0.06199927
C	-3.15303817	0.54091101	0.12336508
H	3.34253455	-2.09680680	0.12533037
C	-1.83222569	-1.47087298	0.03766316
C	-3.24196501	-0.92232864	-0.24908208
H	-3.47786261	-1.00784100	-1.30245989
H	-4.01195581	-1.43883241	0.30616161
H	-3.42375258	0.75799753	1.15696607
H	-3.72337704	1.19020644	-0.52774762
H	-1.56915459	-2.29796211	-0.60780060
H	0.86572326	-2.60379536	0.16842800
C	-1.27042280	2.23827172	0.00736951
H	-0.74797669	2.41251509	0.94466330
H	-0.58192644	2.43858943	-0.80093331
H	-2.13288460	2.88731590	-0.06588711
H	1.43671087	1.75415072	-0.17777713
C	4.13830642	0.83810584	-0.03995592
H	4.72166692	0.64004054	0.85822785
H	4.76484374	0.49071329	-0.86039977
H	4.04095027	1.91210059	-0.13615224
H	-1.77237800	-1.85312999	1.05544642

Time = 33.00 fs
E(CASSCF/6-31G(d)) = -479.5579739

C	-0.97326556	-0.25617934	-0.09130023
C	0.57053580	-0.33495417	-0.04175591
C	1.17782727	-1.58961680	0.09531178
C	1.48805761	0.69906848	-0.10141636
C	2.84533610	0.08026388	-0.00826334
C	2.62782079	-1.30469670	0.10311875
N	-1.71136518	0.85362616	-0.05901683
C	-3.15335954	0.54101237	0.12250692
H	3.34634884	-2.09264014	0.12419970
C	-1.83321685	-1.46950166	0.03643084
C	-3.24284363	-0.92224335	-0.24872044
H	-3.48014113	-1.00598383	-1.30170065
H	-4.01168680	-1.43966032	0.30727725
H	-3.42409797	0.76122474	1.15506039

H	-3.72159780	1.19014022	-0.53033091
H	-1.56387803	-2.29575654	-0.60882878
H	0.85784978	-2.59968510	0.16746025
C	-1.27013640	2.23716602	0.00674415
H	-0.74934362	2.41507613	0.94344022
H	-0.58286102	2.44067761	-0.80141291
H	-2.13476372	2.88312484	-0.06662927
H	1.42684890	1.75351541	-0.17829561
C	4.13840832	0.83748655	-0.03999007
H	4.72308930	0.64125913	0.85732259
H	4.76619230	0.49182500	-0.85976891
H	4.03434070	1.91105001	-0.13649447
H	-1.77052833	-1.85001681	1.05491340

Time = 33.50 fs
E(CASSCF/6-31G(d)) = -479.5597169

C	-0.97443433	-0.25914675	-0.09189402
C	0.57151408	-0.33634357	-0.04185949
C	1.17963991	-1.58939397	0.09575751
C	1.49249208	0.69855433	-0.10168126
C	2.84647575	0.08390206	-0.00857551
C	2.62620225	-1.30663846	0.10363602
N	-1.71340536	0.85496634	-0.05600816
C	-3.15349296	0.54127779	0.12164910
H	3.35119684	-2.08850100	0.12309163
C	-1.83429060	-1.46808929	0.03523656
C	-3.24373936	-0.92223508	-0.24835904
H	-3.48230913	-1.00431967	-1.30107829
H	-4.01160623	-1.44057399	0.30842593
H	-3.42467358	0.76410165	1.15294173
H	-3.72022772	1.19001829	-0.53275123
H	-1.55820009	-2.29323327	-0.61019084
H	0.84798544	-2.59569424	0.16642864
C	-1.26978607	2.23594947	0.00614837
H	-0.75099884	2.41803487	0.94212392
H	-0.58405454	2.44303976	-0.80202524
H	-2.13647647	2.87883736	-0.06738722
H	1.41545634	1.75369670	-0.17891412
C	4.13859630	0.83673031	-0.04001374
H	4.72446793	0.64275558	0.85663257
H	4.76753206	0.49318892	-0.85939533
H	4.02708222	1.90999664	-0.13684995
H	-1.76859295	-1.84708656	1.05461919

Time = 34.00 fs
E(CASSCF/6-31G(d)) = -479.5617178

C	-0.97522164	-0.26213382	-0.09255741
C	0.57190673	-0.33779938	-0.04196673
C	1.18200372	-1.58903923	0.09622279
C	1.49747730	0.69795464	-0.10192677
C	2.84724110	0.08794032	-0.00893369
C	2.62432623	-1.30892601	0.10417928
N	-1.71560929	0.85631620	-0.05297972
C	-3.15344741	0.54171320	0.12079536
H	3.35700760	-2.08446678	0.12201207
C	-1.83543872	-1.46665625	0.03408175
C	-3.24465293	-0.92230253	-0.24800048
H	-3.48436699	-1.00288059	-1.30059866
H	-4.01170489	-1.44154946	0.30959984
H	-3.42553113	0.76661548	1.15065152
H	-3.71928842	1.18983878	-0.53501387
H	-1.55222855	-2.29037174	-0.61182935
H	0.83626620	-2.59186159	0.16534369
C	-1.26938634	2.23463723	0.00558282
H	-0.75289097	2.42136721	0.94077247
H	-0.58549303	2.44560617	-0.80280111
H	-2.13801251	2.87452360	-0.06816316
H	1.40271830	1.75452712	-0.17961551
C	4.13887410	0.83585699	-0.04002873
H	4.72579245	0.64453143	0.85619084
H	4.76885541	0.49480120	-0.85931205
H	4.01928922	1.90891890	-0.13721371
H	-1.76660431	-1.84437120	1.05453197

Time = 34.50 fs
E(CASSCF/6-31G(d)) = -479.5637983

C	-0.97565022	-0.26507521	-0.09329209
C	0.57175127	-0.33930357	-0.04207842
C	1.18487876	-1.58856890	0.09670808
C	1.50294336	0.69729152	-0.10215639
C	2.84762460	0.09228934	-0.00933003
C	2.62223554	-1.31149620	0.10474399
N	-1.71793302	0.85762595	-0.04993818
C	-3.15323475	0.54232178	0.11994805
H	3.36365512	-2.08057273	0.12096595
C	-1.83665116	-1.46522382	0.03296828
C	-3.24558529	-0.92244354	-0.24764777
H	-3.48631457	-1.00169581	-1.30025728
H	-4.01196500	-1.44255684	0.31078633
H	-3.42671766	0.76876159	1.14824444
H	-3.71878920	1.18959926	-0.53712270

H	-1.54607824	-2.28715239	-0.61367944
H	0.82289116	-2.58813366	0.16421113
C	-1.26895279	2.23324596	0.00504712
H	-0.75496104	2.42504230	0.93944928
H	-0.58716022	2.44830081	-0.80376701
H	-2.13936848	2.87025543	-0.06895975
H	1.38885990	1.75574319	-0.18037476
C	4.13924383	0.83488898	-0.04003706
H	4.72705067	0.64658438	0.85602223
H	4.77015224	0.49665536	-0.85954359
H	4.01108793	1.90778328	-0.13757949
H	-1.76459456	-1.84189483	1.05460057

Time = 35.00 fs
E(CASSCF/6-31G(d)) = -479.5657684

C	-0.97574908	-0.26790665	-0.09409874
C	0.57109586	-0.34083686	-0.04219510
C	1.18821271	-1.58800723	0.09721400
C	1.50880882	0.69659325	-0.10237448
C	2.84762448	0.09685543	-0.00975609
C	2.61998074	-1.31428581	0.10532544
N	-1.72032994	0.85884656	-0.04689025
C	-3.15286967	0.54310384	0.11910836
H	3.37096146	-2.07681324	0.11995771
C	-1.83791626	-1.46381319	0.03189854
C	-3.24653739	-0.92265505	-0.24730427
H	-3.48815137	-1.00079128	-1.30004003
H	-4.01236235	-1.44356175	0.31196936
H	-3.42827361	0.77054301	1.14578440
H	-3.71872652	1.18929746	-0.53908009
H	-1.53986803	-2.28356206	-0.61567406
H	0.80812113	-2.58437479	0.16303310
C	-1.26850148	2.23179361	0.00454019
H	-0.75714628	2.42902238	0.93821692
H	-0.58903888	2.45104227	-0.80494302
H	-2.14054719	2.86610454	-0.06978006
H	1.37414488	1.75701418	-0.18116127
C	4.13970587	0.83385037	-0.04004092
H	4.72822820	0.64890806	0.85614155
H	4.77140951	0.49874236	-0.86010422
H	4.00261427	1.90654951	-0.13794020
H	-1.76259511	-1.83967460	1.05475849

Time = 35.50 fs
E(CASSCF/6-31G(d)) = -479.5674432

C	-0.97555257	-0.27056671	-0.09497713
C	0.56999793	-0.34237906	-0.04231709
C	1.19194207	-1.58738445	0.09774123
C	1.51498210	0.69589172	-0.10258605
C	2.84724544	0.10154288	-0.01020300
C	2.61761869	-1.31723301	0.10591886
N	-1.72275193	0.85993133	-0.04384261
C	-3.15236950	0.54405675	0.11827670
H	3.37870464	-2.07314887	0.11899159
C	-1.83922101	-1.46244454	0.03087539
C	-3.24751006	-0.92293301	-0.24697369
H	-3.48987668	-1.00018927	-1.29992451
H	-4.01286869	-1.44452730	0.31313122
H	-3.43023132	0.77196952	1.14333953
H	-3.71908405	1.18893184	-0.54088725
H	-1.53371738	-2.27959905	-0.61774935
H	0.79227264	-2.58038838	0.16180983
C	-1.26804850	2.23029907	0.00406061
H	-0.75938385	2.43326292	0.93713109
H	-0.59111161	2.45374534	-0.80634152
H	-2.14155646	2.86214020	-0.07062732
H	1.35887152	1.75797748	-0.18194149
C	4.14025890	0.83276607	-0.04004255
H	4.72930844	0.65149198	0.85655251
H	4.77261113	0.50105075	-0.86099684
H	3.99401088	1.90517625	-0.13828851
H	-1.76063615	-1.83772212	1.05492946

Time = 36.00 fs
E(CASSCF/6-31G(d)) = -479.5686702

C	-0.97509942	-0.27299852	-0.09592618
C	0.56852267	-0.34390947	-0.04244437
C	1.19599376	-1.58673426	0.09829028
C	1.52136300	0.69521955	-0.10279650
C	2.84649920	0.10625622	-0.01066166
C	2.61521070	-1.32027824	0.10651950
N	-1.72515044	0.86083736	-0.04080178
C	-3.15175399	0.54517489	0.11745313
H	3.38663121	-2.06951823	0.11807220
C	-1.84055135	-1.46113612	0.02990203
C	-3.24850370	-0.92327239	-0.24666002
H	-3.49148989	-0.99990823	-1.29988212
H	-4.01345447	-1.44541618	0.31425448
H	-3.43261347	0.77305652	1.14097693
H	-3.71983333	1.18850253	-0.54254483
H	-1.52774294	-2.27527709	-0.61984925

H	0.77570745	-2.57594549	0.16054169
C	-1.26760951	2.22878193	0.00360698
H	-0.76161434	2.43771300	0.93623508
H	-0.59336127	2.45632271	-0.80796638
H	-2.14240777	2.85842735	-0.07150481
H	1.34336576	1.75827650	-0.18268199
C	4.14089990	0.83166086	-0.04004421
H	4.73027306	0.65432165	0.85724729
H	4.77373836	0.50356693	-0.86221256
H	3.98542384	1.90362716	-0.13861748
H	-1.75874596	-1.83604475	1.05503317

Time = 36.50 fs
E(CASSCF/6-31G(d)) = -479.5693493

C	-0.97443166	-0.27515091	-0.09694420
C	0.56674126	-0.34540757	-0.04257659
C	1.20028748	-1.58609076	0.09886148
C	1.52784518	0.69460745	-0.10301139
C	2.84540462	0.11090222	-0.01112292
C	2.61282033	-1.32336463	0.10712269
N	-1.72747758	0.86152678	-0.03777393
C	-3.15104498	0.54644976	0.11663777
H	3.39447242	-2.06584937	0.11720431
C	-1.84189252	-1.45990368	0.02898182
C	-3.24951819	-0.92366712	-0.24636743
H	-3.49299124	-0.99996247	-1.29988096
H	-4.01409164	-1.44619282	0.31532391
H	-3.43543138	0.77382393	1.13875707
H	-3.72093447	1.18801259	-0.54405367
H	-1.52205516	-2.27062916	-0.62193000
H	0.75881748	-2.57081720	0.15923139
C	-1.26719943	2.22726196	0.00317829
H	-0.76378493	2.44231523	0.93555482
H	-0.59577110	2.45868652	-0.80981263
H	-2.14311434	2.85502378	-0.07241557
H	1.32796940	1.75759621	-0.18335218
C	4.14162405	0.83055860	-0.04004806
H	4.73110216	0.65737793	0.85820645
H	4.77476989	0.50627448	-0.86373041
H	3.97699968	1.90187605	-0.13892104
H	-1.75695061	-1.83464794	1.05499272

Time = 37.00 fs
E(CASSCF/6-31G(d)) = -479.5694367

C	-0.97359326	-0.27697970	-0.09802925

C	0.56472853	-0.34685356	-0.04271298
C	1.20473849	-1.58548568	0.09945477
C	1.53431895	0.69408186	-0.10323617
C	2.84398761	0.11539184	-0.01157771
C	2.61051095	-1.32643826	0.10772392
N	-1.72968716	0.86196787	-0.03476479
C	-3.15026610	0.54787005	0.11583127
H	3.40196244	-2.06207327	0.11639299
C	-1.84322945	-1.45875973	0.02811810
C	-3.25055268	-0.92411007	-0.24610013
H	-3.49438201	-1.00036190	-1.29988809
H	-4.01475567	-1.44682515	0.31632805
H	-3.43868364	0.77429469	1.13672849
H	-3.72233734	1.18746842	-0.54541551
H	-1.51675575	-2.26570848	-0.62396243
H	0.74200733	-2.56480540	0.15788577
C	-1.26683221	2.22575892	0.00277415
H	-0.76585144	2.44700625	0.93509609
H	-0.59832421	2.46075023	-0.81186701
H	-2.14369008	2.85197865	-0.07336214
H	1.31302692	1.75569320	-0.18392695
C	4.14242465	0.82948131	-0.04005610
H	4.73177522	0.66063656	0.85940015
H	4.77568295	0.50915363	-0.86551850
H	3.96888198	1.89991195	-0.13919459
H	-1.75527376	-1.83353667	1.05473967

Time = 37.50 fs
E(CASSCF/6-31G(d)) = -479.5689517

C	-0.97262958	-0.27844841	-0.09917876
C	0.56256173	-0.34822810	-0.04285247
C	1.20925978	-1.58494626	0.10006967
C	1.54067272	0.69366315	-0.10347600
C	2.84228153	0.11964089	-0.01201718
C	2.60834362	-1.32944769	0.10831882
N	-1.73173549	0.86213559	-0.03177959
C	-3.14944230	0.54942175	0.11503517
H	3.40885518	-2.05813885	0.11564464
C	-1.84454701	-1.45771324	0.02731381
C	-3.25160535	-0.92459314	-0.24586220
H	-3.49566503	-1.00111208	-1.29987253
H	-4.01542780	-1.44728628	0.31726065
H	-3.44235551	0.77449373	1.13492397
H	-3.72398333	1.18688098	-0.54663408
H	-1.51193527	-2.26058853	-0.62593380
H	0.72567693	-2.55776872	0.15651676

C	-1.26652054	2.22429202	0.00239495
H	-0.76777943	2.45171814	0.93484336
H	-0.60100306	2.46243070	-0.81410879
H	-2.14414824	2.84933097	-0.07434641
H	1.29887405	1.75241872	-0.18438868
C	4.14329317	0.82844883	-0.04007013
H	4.73227291	0.66406922	0.86078977
H	4.77645500	0.51218188	-0.86753562
H	3.96120728	1.89774274	-0.13943538
H	-1.75373547	-1.83271621	1.05421881

Time = 38.00 fs
E(CASSCF/6-31G(d)) = -479.5679600

C	-0.97158595	-0.27952870	-0.10039006
C	0.56031835	-0.34951286	-0.04299362
C	1.21376497	-1.58449330	0.10070519
C	1.54679597	0.69336433	-0.10373562
C	2.84032644	0.12357129	-0.01243279
C	2.60637502	-1.33234380	0.10890323
N	-1.73358215	0.86201215	-0.02882294
C	-3.14859948	0.55108835	0.11425218
H	3.41494030	-2.05402164	0.11496617
C	-1.84583041	-1.45676952	0.02657129
C	-3.25267340	-0.92510734	-0.24565745
H	-3.49684518	-1.00221408	-1.29980800
H	-4.01609641	-1.44755577	0.31812166
H	-3.44641838	0.77444704	1.13335790
H	-3.72580706	1.18626657	-0.54771592
H	-1.50767136	-2.25536133	-0.62784796
H	0.71020338	-2.54964292	0.15514225
C	-1.26627583	2.22287963	0.00204194
H	-0.76954440	2.45637919	0.93476048
H	-0.60378856	2.46364995	-0.81651108
H	-2.14450030	2.84710822	-0.07536944
H	1.28582310	1.74773431	-0.18472865
C	4.14421925	0.82747831	-0.04009175
H	4.73257804	0.66764291	0.86233006
H	4.77706487	0.51533316	-0.86973314
H	3.95410234	1.89539649	-0.13964275
H	-1.75235219	-1.83219358	1.05339248

Time = 38.50 fs
E(CASSCF/6-31G(d)) = -479.5665593

C	-0.97050660	-0.28020069	-0.10166025
C	0.55807429	-0.35069070	-0.04313472

C	1.21817094	-1.58413997	0.10135995
C	1.55258190	0.69319031	-0.10401909
C	2.83816838	0.12711198	-0.01281648
C	2.60465541	-1.33507983	0.10947322
N	-1.73519084	0.86158722	-0.02589877
C	-3.14776389	0.55285106	0.11348624
H	3.42005686	-2.04973177	0.11436506
C	-1.84706546	-1.45593033	0.02589197
C	-3.25375302	-0.92564289	-0.24548921
H	-3.49792967	-1.00366464	-1.29967493
H	-4.01675756	-1.44762041	0.31891758
H	-3.45083027	0.77418094	1.13202528
H	-3.72773837	1.18564710	-0.54867113
H	-1.50402770	-2.25013418	-0.62972432
H	0.69592426	-2.54045324	0.15378604
C	-1.26610815	2.22153878	0.00171709
H	-0.77113075	2.46091590	0.93479359
H	-0.60665926	2.46433717	-0.81904256
H	-2.14475535	2.84532573	-0.07643130
H	1.27414974	1.74171968	-0.18494788
C	4.14519078	0.82658398	-0.04012225
H	4.73267723	0.67132015	0.86397157
H	4.77749454	0.51857762	-0.87205742
H	3.94768124	1.89292224	-0.13981822
H	-1.75113608	-1.83197771	1.05224285

Time = 39.00 fs
E(CASSCF/6-31G(d)) = -479.5648677

C	-0.96943375	-0.28045293	-0.10298634
C	0.55590224	-0.35174572	-0.04327388
C	1.22240029	-1.58389111	0.10203213
C	1.55792991	0.69313743	-0.10432980
C	2.83585819	0.13019947	-0.01316072
C	2.60322718	-1.33761113	0.11002514
N	-1.73652994	0.86085796	-0.02301028
C	-3.14696160	0.55468912	0.11274249
H	3.42410302	-2.04531793	0.11384909
C	-1.84823887	-1.45519420	0.02527610
C	-3.25483949	-0.92618941	-0.24536018
H	-3.49892816	-1.00545613	-1.29946198
H	-4.01741485	-1.44747437	0.31966120
H	-3.45553670	0.77372182	1.13090250
H	-3.72970426	1.18505020	-0.54951391
H	-1.50105375	-2.24502501	-0.63159539
H	0.68312340	-2.53032065	0.15247744
C	-1.26602622	2.22028482	0.00142289

H	-0.77253015	2.46525495	0.93487549
H	-0.60959067	2.46443034	-0.82166919
H	-2.14491997	2.84398641	-0.07753105
H	1.26408182	1.73457416	-0.18505751
C	4.14619394	0.82577714	-0.04016271
H	4.73256221	0.67505925	0.86566330
H	4.77773060	0.52188144	-0.87445230
H	3.94204290	1.89038840	-0.13996547
H	-1.75009467	-1.83207957	1.05077294

Time = 39.50 fs
E(CASSCF/6-31G(d)) = -479.5630029

C	-0.96840628	-0.28028233	-0.10436530
C	0.55386996	-0.35266339	-0.04340907
C	1.22638328	-1.58374307	0.10271962
C	1.56274785	0.69319357	-0.10467028
C	2.83345037	0.13277834	-0.01345865
C	2.60212377	-1.33989524	0.11055566
N	-1.73757311	0.85982890	-0.02015986
C	-3.14621786	0.55658006	0.11202711
H	3.42704171	-2.04086829	0.11342589
C	-1.84933839	-1.45455691	0.02472252
C	-3.25592729	-0.92673610	-0.24527226
H	-3.49985278	-1.00757670	-1.29916684
H	-4.01807865	-1.44711891	0.32037095
H	-3.46047240	0.77309602	1.12994994
H	-3.73163072	1.18450899	-0.55026276
H	-1.49878530	-2.24015645	-0.63350312
H	0.67201991	-2.51946153	0.15125045
C	-1.26603743	2.21913097	0.00116203
H	-0.77373916	2.46932544	0.93493119
H	-0.61255478	2.46387782	-0.82435596
H	-2.14499844	2.84308116	-0.07866667
H	1.25579099	1.72661123	-0.18507848
C	4.14721343	0.82506621	-0.04021393
H	4.73223113	0.67881468	0.86735541
H	4.77776567	0.52520681	-0.87686176
H	3.93726911	1.88787936	-0.14009005
H	-1.74923103	-1.83251187	1.04900623

Time = 40.00 fs
E(CASSCF/6-31G(d)) = -479.5610789

C	-0.96745902	-0.27969392	-0.10579410
C	0.55203909	-0.35343056	-0.04353823
C	1.23005957	-1.58368397	0.10342004

C	1.56695405	0.69333860	-0.10504221
C	2.83100143	0.13480167	-0.01370414
C	2.60136900	-1.34189205	0.11106180
N	-1.73829975	0.85851164	-0.01734911
C	-3.14555650	0.55850008	0.11134690
H	3.42890198	-2.03650836	0.11310261
C	-1.85035299	-1.45401218	0.02422851
C	-3.25701033	-0.92727198	-0.24522648
H	-3.50071790	-1.01001038	-1.29879608
H	-4.01876445	-1.44656158	0.32106955
H	-3.46556360	0.77233008	1.12911582
H	-3.73344457	1.18406126	-0.55094047
H	-1.49724567	-2.23564912	-0.63549438
H	0.66276017	-2.50818171	0.15014262
C	-1.26614791	2.21808799	0.00093707
H	-0.77475671	2.47306135	0.93488406
H	-0.61552003	2.46263970	-0.82706840
H	-2.14499346	2.84258969	-0.07983517
H	1.24938732	1.71824585	-0.18504070
C	4.14823258	0.82445706	-0.04027652
H	4.73168942	0.68253766	0.86900162
H	4.77759960	0.52851201	-0.87923238
H	3.93342327	1.88549104	-0.14019903
H	-1.74854359	-1.83328814	1.04698462

Time = 40.50 fs
E(CASSCF/6-31G(d)) = -479.5591993

C	-0.96662191	-0.27870043	-0.10726973
C	0.55046409	-0.35403547	-0.04365943
C	1.23337940	-1.58369444	0.10413093
C	1.57047899	0.69354547	-0.10544637
C	2.82856833	0.13623144	-0.01389188
C	2.60097690	-1.34356423	0.11154097
N	-1.73869535	0.85692431	-0.01457879
C	-3.14499935	0.56042442	0.11070893
H	3.42977547	-2.03239543	0.11288531
C	-1.85127287	-1.45355230	0.02378964
C	-3.25808225	-0.92778612	-0.24522289
H	-3.50153956	-1.01273718	-1.29836410
H	-4.01949062	-1.44581484	0.32178234
H	-3.47073064	0.77145081	1.12834090
H	-3.73507487	1.18374819	-0.55157347
H	-1.49644744	-2.23161461	-0.63761570
H	0.65541430	-2.49686528	0.14919367
C	-1.26636251	2.21716393	0.00074999
H	-0.77558156	2.47640389	0.93466201

H	-0.61845168	2.46068874	-0.82977383
H	-2.14490715	2.84248173	-0.08103262
H	1.24491866	1.70997381	-0.18498148
C	4.14923359	0.82395322	-0.04035089
H	4.73095053	0.68617688	0.87056122
H	4.77724025	0.53175191	-0.88151537
H	3.93054969	1.88332513	-0.14030054
H	-1.74802653	-1.83442125	1.04476474

Time = 41.00 fs
E(CASSCF/6-31G(d)) = -479.5574614

C	-0.96591960	-0.27732196	-0.10878908
C	0.54919180	-0.35446778	-0.04377089
C	1.23630428	-1.58374860	0.10484972
C	1.57326644	0.69378170	-0.10588271
C	2.82620656	0.13703883	-0.01401752
C	2.60095200	-1.34487782	0.11199103
N	-1.73875171	0.85509120	-0.01184891
C	-3.14456568	0.56232769	0.11011997
H	3.42980788	-2.02871049	0.11277930
C	-1.85208952	-1.45316900	0.02339971
C	-3.25913669	-0.92826791	-0.24526049
H	-3.50233526	-1.01573348	-1.29789198
H	-4.02027642	-1.44489497	0.32253534
H	-3.47589134	0.77048620	1.12756462
H	-3.73645463	1.18361351	-0.55219153
H	-1.49639425	-2.22814909	-0.63990837
H	0.64997757	-2.48595711	0.14844408
C	-1.26668475	2.21636381	0.00060188
H	-0.77621008	2.47930410	0.93420322
H	-0.62131259	2.45801138	-0.83244229
H	-2.14474227	2.84271893	-0.08225441
H	1.24237490	1.70234281	-0.18494330
C	4.15019791	0.82355649	-0.04043729
H	4.73003592	0.68967931	0.87200082
H	4.77670364	0.53487837	-0.88366835
H	3.92867344	1.88148251	-0.14040312
H	-1.74766956	-1.83592237	1.04241448

Time = 41.50 fs
E(CASSCF/6-31G(d)) = -479.5559681

C	-0.96537088	-0.27558534	-0.11034918
C	0.54826035	-0.35471870	-0.04387113
C	1.23880725	-1.58381570	0.10557390
C	1.57527446	0.69401204	-0.10635046

C	2.82396824	0.13720501	-0.01407779
C	2.60129012	-1.34580355	0.11241036
N	-1.73846726	0.85304202	-0.00915851
C	-3.14427154	0.56418446	0.10958596
H	3.42918635	-2.02564631	0.11278848
C	-1.85279578	-1.45285419	0.02305084
C	-3.26016763	-0.92870736	-0.24533733
H	-3.50312305	-1.01897210	-1.29740515
H	-4.02113910	-1.44382008	0.32335317
H	-3.48096400	0.76946550	1.12673024
H	-3.73752131	1.18370141	-0.55282645
H	-1.49708284	-2.22532688	-0.64240348
H	0.64637705	-2.47593960	0.14793335
C	-1.26711679	2.21568948	0.00049259
H	-0.77663462	2.48172444	0.93346064
H	-0.62406465	2.45460767	-0.83504704
H	-2.14450348	2.84325648	-0.08349538
H	1.24169750	1.69591262	-0.18497057
C	4.15110662	0.82326726	-0.04053589
H	4.72897469	0.69299113	0.87329510
H	4.77601382	0.53784116	-0.88565625
H	3.92780087	1.88005631	-0.14051513
H	-1.74745862	-1.83779871	1.04000703

Time = 42.00 fs
E(CASSCF/6-31G(d)) = -479.5548131

C	-0.96498837	-0.27352372	-0.11194720
C	0.54769916	-0.35478102	-0.04395898
C	1.24087221	-1.58386227	0.10630115
C	1.57647518	0.69420152	-0.10684830
C	2.82190062	0.13672116	-0.01407054
C	2.60197968	-1.34631769	0.11279788
N	-1.73784695	0.85081129	-0.00650582
C	-3.14412955	0.56596957	0.10911152
H	3.42812098	-2.02339281	0.11291510
C	-1.85338577	-1.45260064	0.02273366
C	-3.26116971	-0.92909528	-0.24545046
H	-3.50392096	-1.02242268	-1.29693084
H	-4.02209160	-1.44260880	0.32425702
H	-3.48587089	0.76841952	1.12579032
H	-3.73821817	1.18405491	-0.55351097
H	-1.49850473	-2.22319607	-0.64511788
H	0.64448494	-2.46730437	0.14769829
C	-1.26765934	2.21513971	0.00042047
H	-0.77684222	2.48364047	0.93240559
H	-0.62666989	2.45049177	-0.83756473

H	-2.14419854	2.84404555	-0.08475005
H	1.24279459	1.69120873	-0.18510593
C	4.15194075	0.82308498	-0.04064680
H	4.72780265	0.69605869	0.87442702
H	4.77520196	0.54058877	-0.88745179
H	3.92792068	1.87912510	-0.14064413
H	-1.74737650	-1.84005204	1.03761545

Time = 42.50 fs
E(CASSCF/6-31G(d)) = -479.5541089

C	-0.96477849	-0.27117612	-0.11358048
C	0.54752863	-0.35464915	-0.04403372
C	1.24249259	-1.58385465	0.10702946
C	1.57685430	0.69431917	-0.10737460
C	2.82004475	0.13558887	-0.01399485
C	2.60300345	-1.34640340	0.11315308
N	-1.73690232	0.84843776	-0.00388822
C	-3.14414846	0.56765863	0.10869956
H	3.42682477	-2.02212239	0.11315976
C	-1.85385487	-1.45240244	0.02243761
C	-3.26213846	-0.92942350	-0.24559614
H	-3.50474611	-1.02605163	-1.29649520
H	-4.02314028	-1.44127852	0.32526283
H	-3.49054069	0.76738085	1.12471053
H	-3.73849426	1.18471374	-0.55427701
H	-1.50064758	-2.22177546	-0.64805130
H	0.64413759	-2.46051859	0.14777125
C	-1.26831156	2.21471015	0.00038228
H	-0.77681457	2.48504139	0.93102943
H	-0.62909239	2.44569138	-0.83997508
H	-2.14383910	2.84503484	-0.08601270
H	1.24555937	1.68867145	-0.18538606
C	4.15268166	0.82300858	-0.04077009
H	4.72656108	0.69882979	0.87538752
H	4.77430524	0.54306986	-0.88903544
H	3.92900515	1.87874705	-0.14079623
H	-1.74740368	-1.84267664	1.03530684

Time = 43.00 fs
E(CASSCF/6-31G(d)) = -479.5539496

C	-0.96474119	-0.26858696	-0.11524665
C	0.54775982	-0.35431929	-0.04409505
C	1.24366968	-1.58376199	0.10775726
C	1.57641044	0.69434177	-0.10792771
C	2.81843438	0.13382048	-0.01385106

C	2.60434045	-1.34605229	0.11347607
N	-1.73565141	0.84596377	-0.00130223
C	-3.14433297	0.56922845	0.10835088
H	3.42549099	-2.02197231	0.11352148
C	-1.85419973	-1.45225539	0.02215138
C	-3.26307057	-0.92968508	-0.24576991
H	-3.50561400	-1.02982257	-1.29612054
H	-4.02428339	-1.43984444	0.32637994
H	-3.49491146	0.76638357	1.12347302
H	-3.73830510	1.18571240	-0.55515422
H	-1.50349580	-2.22105382	-0.65118529
H	0.64515926	-2.45598796	0.14817812
C	-1.26907098	2.21439350	0.00037313
H	-0.77652818	2.48593064	0.92934387
H	-0.63129978	2.44024746	-0.84226062
H	-2.14344136	2.84617286	-0.08727756
H	1.24988840	1.68860586	-0.18583764
C	4.15331155	0.82303671	-0.04090582
H	4.72529491	0.70125477	0.87617444
H	4.77336503	0.54523446	-0.89039459
H	3.93101163	1.87895490	-0.14097572
H	-1.74751904	-1.84565769	1.03313685

Time = 43.50 fs
E(CASSCF/6-31G(d)) = -479.5544024

C	-0.96487010	-0.26580537	-0.11694370
C	0.54839438	-0.35378957	-0.04414314
C	1.24441043	-1.58355926	0.10848356
C	1.57515433	0.69425699	-0.10850615
C	2.81709511	0.13143915	-0.01364089
C	2.60596797	-1.34526577	0.11376761
N	-1.73411844	0.84343448	0.00125645
C	-3.14468364	0.57065751	0.10806402
H	3.42427237	-2.02302922	0.11399765
C	-1.85441837	-1.45215716	0.02186334
C	-3.26396394	-0.92987435	-0.24596680
H	-3.50653796	-1.03369650	-1.29582320
H	-4.02551029	-1.43831892	0.32761034
H	-3.49893252	0.76546305	1.12207841
H	-3.73761284	1.18707832	-0.55616835
H	-1.50703024	-2.22099139	-0.65448389
H	0.64738780	-2.45401953	0.14893672
C	-1.26993336	2.21417977	0.00038659
H	-0.77595563	2.48632536	0.92737974
H	-0.63326472	2.43421331	-0.84440621
H	-2.14302609	2.84740961	-0.08853890

H	1.25569612	1.69114128	-0.18647405
C	4.15381392	0.82316794	-0.04105407
H	4.72405060	0.70328761	0.87679089
H	4.77242473	0.54703537	-0.89152232
H	3.93388417	1.87975283	-0.14118465
H	-1.74770073	-1.84897011	1.03114547

Time = 44.00 fs
E(CASSCF/6-31G(d)) = -479.5554843

C	-0.96515250	-0.26288466	-0.11867019
C	0.54942421	-0.35306028	-0.04417863
C	1.24472569	-1.58323001	0.10920810
C	1.57310874	0.69406538	-0.10910874
C	2.81604387	0.12847913	-0.01336740
C	2.60786302	-1.34405625	0.11402912
N	-1.73233352	0.84089732	0.00379298
C	-3.14519688	0.57192639	0.10783509
H	3.42326482	-2.02531690	0.11458412
C	-1.85451028	-1.45210729	0.02156210
C	-3.26481773	-0.92998703	-0.24618149
H	-3.50752868	-1.03763211	-1.29561163
H	-4.02680146	-1.43671119	0.32894845
H	-3.50256602	0.76465539	1.12054617
H	-3.73638638	1.18883017	-0.55733975
H	-1.51122752	-2.22152319	-0.65789595
H	0.65069778	-2.45478951	0.15005529
C	-1.27089262	2.21405663	0.00041491
H	-0.77506742	2.48625554	0.92518408
H	-0.63496622	2.42765357	-0.84639847
H	-2.14261823	2.84869806	-0.08979101
H	1.26292192	1.69620848	-0.18729357
C	4.15417406	0.82340081	-0.04121492
H	4.72287384	0.70488705	0.87724333
H	4.77152744	0.54842961	-0.89241581
H	3.93755512	1.88111541	-0.14142272
H	-1.74792701	-1.85257790	1.02935389

Time = 44.50 fs
E(CASSCF/6-31G(d)) = -479.5571364

C	-0.96556953	-0.25988160	-0.12042533
C	0.55083155	-0.35213410	-0.04420258
C	1.24462857	-1.58276828	0.10993140
C	1.57030841	0.69378065	-0.10973465
C	2.81528883	0.12498559	-0.01303505
C	2.61000345	-1.34244786	0.11426271

N	-1.73033221	0.83840126	0.00631332
C	-3.14586512	0.57301816	0.10765790
H	3.42249852	-2.02878947	0.11527549
C	-1.85447650	-1.45210701	0.02123703
C	-3.26563234	-0.93002023	-0.24640852
H	-3.50859398	-1.04158621	-1.29548559
H	-4.02812950	-1.43502782	0.33038168
H	-3.50578793	0.76399677	1.11891354
H	-3.73460172	1.19097656	-0.55868212
H	-1.51605858	-2.22256426	-0.66135908
H	0.65501640	-2.45832113	0.15153152
C	-1.27194076	2.21400971	0.00044942
H	-0.77383451	2.48576275	0.92281579
H	-0.63639056	2.42064324	-0.84822551
H	-2.14224596	2.84999532	-0.09102827
H	1.27152764	1.70353970	-0.18827931
C	4.15437960	0.82373374	-0.04138842
H	4.72180712	0.70601763	0.87753950
H	4.77071347	0.54937983	-0.89307450
H	3.94194643	1.88298850	-0.14168730
H	-1.74817676	-1.85643431	1.02776360

Time = 45.00 fs
E(CASSCF/6-31G(d)) = -479.5592206

C	-0.96609655	-0.25685566	-0.12220909
C	0.55258871	-0.35101640	-0.04421645
C	1.24413390	-1.58217968	0.11065492
C	1.56680064	0.69342838	-0.11038329
C	2.81482972	0.12101432	-0.01264966
C	2.61236825	-1.34047655	0.11447114
N	-1.72815487	0.83599595	0.00882416
C	-3.14667705	0.57391872	0.10752421
H	3.42193713	-2.03333051	0.11606463
C	-1.85431974	-1.45215877	0.02087868
C	-3.26640915	-0.92997237	-0.24664242
H	-3.50973842	-1.04551396	-1.29543532
H	-4.02946018	-1.43327313	0.33189137
H	-3.50858796	0.76352212	1.11723268
H	-3.73224202	1.19351460	-0.56020131
H	-1.52148731	-2.22401554	-0.66480387
H	0.66032961	-2.46447628	0.15335198
C	-1.27306778	2.21402318	0.00048091
H	-0.77223121	2.48489784	0.92034046
H	-0.63753177	2.41326620	-0.84987664
H	-2.14193924	2.85126329	-0.09224494
H	1.28148701	1.71269146	-0.18940122

C	4.15442076	0.82416467	-0.04157455
H	4.72088767	0.70665067	0.87768633
H	4.77001817	0.54985562	-0.89349854
H	3.94697108	1.88529269	-0.14197376
H	-1.74843038	-1.86048208	1.02635579

Time = 45.50 fs
E(CASSCF/6-31G(d)) = -479.5615365

C	-0.96670345	-0.25386815	-0.12402226
C	0.55465843	-0.34971547	-0.04422202
C	1.24325824	-1.58148086	0.11138095
C	1.56264567	0.69304309	-0.11105408
C	2.81465846	0.11663136	-0.01221835
C	2.61493721	-1.33818961	0.11465787
N	-1.72584603	0.83373102	0.01133285
C	-3.14761803	0.57461723	0.10742409
H	3.42148590	-2.03875955	0.11694305
C	-1.85404450	-1.45226586	0.02047913
C	-3.26715037	-0.92984309	-0.24687793
H	-3.51096359	-1.04936939	-1.29544226
H	-4.03075491	-1.43145025	0.33345450
H	-3.51096906	0.76326412	1.11556645
H	-3.72929797	1.19642941	-0.56189463
H	-1.52746915	-2.22577104	-0.66815954
H	0.66667613	-2.47296377	0.15549244
C	-1.27426174	2.21408023	0.00050023
H	-0.77023861	2.48371894	0.91782405
H	-0.63839198	2.40561399	-0.85134223
H	-2.14172828	2.85246894	-0.09343515
H	1.29276920	1.72308809	-0.19061944
C	4.15429081	0.82469080	-0.04177320
H	4.72014533	0.70676499	0.87768812
H	4.76946974	0.54983452	-0.89368713
H	3.95253425	1.88792820	-0.14227581
H	-1.74867016	-1.86465516	1.02509424

Time = 46.00 fs
E(CASSCF/6-31G(d)) = -479.5638531

C	-0.96735521	-0.25098105	-0.12586648
C	0.55699398	-0.34824285	-0.04422134
C	1.24202110	-1.58069798	0.11211262
C	1.55791727	0.69266466	-0.11174632
C	2.81475983	0.11191227	-0.01174942
C	2.61768979	-1.33564462	0.11482698
N	-1.72345341	0.83165508	0.01384732

C	-3.14867051	0.57510624	0.10734643
H	3.42100726	-2.04484389	0.11790099
C	-1.85365713	-1.45243184	0.02003217
C	-3.26785870	-0.92963318	-0.24711004
H	-3.51226839	-1.05310609	-1.29548058
H	-4.03197325	-1.42956243	0.33504555
H	-3.51294604	0.76325200	1.11398341
H	-3.72576837	1.19969406	-0.56375087
H	-1.53394977	-2.22772488	-0.67135940
H	0.67413147	-2.48336176	0.15791888
C	-1.27550875	2.21416359	0.00049878
H	-0.76784755	2.48228884	0.91532720
H	-0.63898079	2.39778413	-0.85261427
H	-2.14164159	2.85358455	-0.09459271
H	1.30532090	1.73407518	-0.19188864
C	4.15398635	0.82530811	-0.04198408
H	4.71960087	0.70634731	0.87754508
H	4.76908740	0.54930270	-0.89363728
H	3.95853478	1.89078109	-0.14258603
H	-1.74888042	-1.86888095	1.02392905

Time = 46.50 fs
E(CASSCF/6-31G(d)) = -479.5659458

C	-0.96801334	-0.24825569	-0.12774424
C	0.55954010	-0.34661317	-0.04421663
C	1.24044604	-1.57986420	0.11285373
C	1.55270197	0.69233438	-0.11245883
C	2.81511282	0.10694103	-0.01125229
C	2.62060394	-1.33290771	0.11498319
N	-1.72102678	0.82981449	0.01637586
C	-3.14981453	0.57538197	0.10727963
H	3.42034084	-2.05131812	0.11892755
C	-1.85316585	-1.45265997	0.01953336
C	-3.26853700	-0.92934433	-0.24733407
H	-3.51364940	-1.05667791	-1.29551958
H	-4.03307583	-1.42761452	0.33663867
H	-3.51454428	0.76351017	1.11255204
H	-3.72166102	1.20326985	-0.56575071
H	-1.54086372	-2.22977765	-0.67434600
H	0.68278633	-2.49515187	0.16058908
C	-1.27679315	2.21425604	0.00046911
H	-0.76506151	2.48067318	0.91289990
H	-0.63931464	2.38987895	-0.85368718
H	-2.14170368	2.85458764	-0.09571112
H	1.31905317	1.74497689	-0.19316246
C	4.15350747	0.82601102	-0.04220673

H	4.71926513	0.70539329	0.87725289
H	4.76888059	0.54825609	-0.89334357
H	3.96486581	1.89373072	-0.14289656
H	-1.74904720	-1.87308289	1.02280167

Time = 47.00 fs
E(CASSCF/6-31G(d)) = -479.5676374

C	-0.96863711	-0.24575130	-0.12965889
C	0.56223392	-0.34484421	-0.04421025
C	1.23856219	-1.57901663	0.11360860
C	1.54709789	0.69209142	-0.11318982
C	2.81569196	0.10180856	-0.01073729
C	2.62365473	-1.33005151	0.11513180
N	-1.71861677	0.82825216	0.01892698
C	-3.15102823	0.57544440	0.10721225
H	3.41932497	-2.05790385	0.12001112
C	-1.85258064	-1.45295261	0.01898004
C	-3.26918789	-0.92897902	-0.24754570
H	-3.51510126	-1.06003959	-1.29552595
H	-4.03402685	-1.42561404	0.33820964
H	-3.51579726	0.76405691	1.11133408
H	-3.71699316	1.20710697	-0.56786739
H	-1.54813459	-2.23184191	-0.67707514
H	0.69272168	-2.50776099	0.16345484
C	-1.27809770	2.21434087	0.00040535
H	-0.76189900	2.47893807	0.91057707
H	-0.63941609	2.38200378	-0.85455832
H	-2.14193321	2.85546032	-0.09678334
H	1.33383217	1.75514974	-0.19439759
C	4.15285777	0.82679200	-0.04244041
H	4.71913873	0.70390791	0.87680303
H	4.76884859	0.54670069	-0.89279863
H	3.97141605	1.89665756	-0.14319972
H	-1.74915829	-1.87718342	1.02165031

Time = 47.50 fs
E(CASSCF/6-31G(d)) = -479.5688045

C	-0.96918456	-0.24352338	-0.13161426
C	0.56500620	-0.34295719	-0.04420449
C	1.23640559	-1.57819281	0.11438184
C	1.54121342	0.69197008	-0.11393684
C	2.81646831	0.09661153	-0.01021546
C	2.62681296	-1.32715300	0.11527856
N	-1.71627354	0.82700627	0.02150914
C	-3.15228848	0.57529731	0.10713377

H	3.41781737	-2.06432791	0.12113935
C	-1.85191321	-1.45331088	0.01837096
C	-3.26981361	-0.92854034	-0.24774091
H	-3.51661773	-1.06314787	-1.29546711
H	-4.03479659	-1.42357239	0.33973790
H	-3.51674424	0.76490313	1.11037853
H	-3.71179254	1.21114608	-0.57006832
H	-1.55567512	-2.23384734	-0.67951970
H	0.70398581	-2.52060285	0.16646441
C	-1.27940389	2.21440249	0.00030353
H	-0.75839447	2.47714837	0.90837587
H	-0.63931218	2.37426534	-0.85522934
H	-2.14234100	2.85618893	-0.09780184
H	1.34947414	1.76402577	-0.19555719
C	4.15204423	0.82764115	-0.04268412
H	4.71921156	0.70190489	0.87618259
H	4.76898001	0.54465203	-0.89199281
H	3.97807198	1.89944978	-0.14348857
H	-1.74920274	-1.88110751	1.02041658

Time = 48.00 fs
E(CASSCF/6-31G(d)) = -479.5694019

C	-0.96961431	-0.24162195	-0.13361453
C	0.56778303	-0.34097646	-0.04420159
C	1.23401959	-1.57742784	0.11517815
C	1.53516424	0.69199750	-0.11469668
C	2.81741098	0.09145026	-0.00969836
C	2.63004439	-1.32429097	0.11542963
N	-1.71404538	0.82610885	0.02413047
C	-3.15357142	0.57494832	0.10703525
H	3.41571235	-2.07034272	0.12229977
C	-1.85117675	-1.45373427	0.01770611
C	-3.27041565	-0.92803186	-0.24791601
H	-3.51819195	-1.06596231	-1.29531382
H	-4.03536323	-1.42150547	0.34120810
H	-3.51742784	0.76605131	1.10971602
H	-3.70609796	1.21531990	-0.57231670
H	-1.56338849	-2.23574334	-0.68167132
H	0.71657714	-2.53311775	0.16956504
C	-1.28069231	2.21442686	0.00016183
H	-0.75459866	2.47536638	0.90629479
H	-0.63903345	2.36676991	-0.85570690
H	-2.14292876	2.85676347	-0.09875861
H	1.36574878	1.77114757	-0.19661345
C	4.15107700	0.82854610	-0.04293654
H	4.71946379	0.69940698	0.87537615

H	4.76925391	0.54213518	-0.89091612
H	3.98471913	1.90201041	-0.14375752
H	-1.74917053	-1.88478538	1.01905042

Time = 48.50 fs
E(CASSCF/6-31G(d)) = -479.5694492

C	-0.96988734	-0.24008985	-0.13566387
C	0.57048746	-0.33892936	-0.04420364
C	1.23145516	-1.57675147	0.11600207
C	1.52907103	0.69219207	-0.11546541
C	2.81848802	0.08642666	-0.00919777
C	2.63330933	-1.32154345	0.11559138
N	-1.71197715	0.82558435	0.02679844
C	-3.15485297	0.57440868	0.10690990
H	3.41295382	-2.07574047	0.12348005
C	-1.85038571	-1.45422040	0.01698631
C	-3.27099458	-0.92745750	-0.24806748
H	-3.51981725	-1.06844570	-1.29504269
H	-4.03571398	-1.41943381	0.34261089
H	-3.51789141	0.76749491	1.10935479
H	-3.69995954	1.21955496	-0.57457327
H	-1.57117011	-2.23750074	-0.68354090
H	0.73043234	-2.54480658	0.17270538
C	-1.28194315	2.21440188	-0.00001954
H	-0.75057736	2.47365061	0.90431492
H	-0.63861281	2.35962111	-0.85600315
H	-2.14368810	2.85717665	-0.09964503
H	1.38238494	1.77619282	-0.19754930
C	4.14996890	0.82949181	-0.04319604
H	4.71986708	0.69644540	0.87436781
H	4.76964104	0.53918376	-0.88955998
H	3.99124413	1.90426219	-0.14400272
H	-1.74905235	-1.88815555	1.01751526

Time = 49.00 fs
E(CASSCF/6-31G(d)) = -479.5690000

C	-0.96996861	-0.23896110	-0.13776609
C	0.57304191	-0.33684563	-0.04421254
C	1.22876979	-1.57618622	0.11685779
C	1.52305566	0.69256231	-0.11623839
C	2.81966765	0.08164177	-0.00872545
C	2.63656292	-1.31898509	0.11577027
N	-1.71010895	0.82544833	0.02951956
C	-3.15610938	0.57369313	0.10675353
H	3.40954358	-2.08036778	0.12466860

C	-1.84955547	-1.45476498	0.01621284
C	-3.27154992	-0.92682146	-0.24819201
H	-3.52148745	-1.07056525	-1.29463824
H	-4.03584566	-1.41738262	0.34394366
H	-3.51817764	0.76921783	1.10927841
H	-3.69343913	1.22377405	-0.57679880
H	-1.57890994	-2.23911124	-0.68515760
H	0.74542164	-2.55525744	0.17583770
C	-1.28313660	2.21431775	-0.00023840
H	-0.74640903	2.47205558	0.90240286
H	-0.63808440	2.35291822	-0.85613640
H	-2.14460050	2.85742390	-0.10045219
H	1.39908144	1.77898982	-0.19835951
C	4.14873508	0.83046075	-0.04346072
H	4.72038630	0.69305952	0.87314354
H	4.77010570	0.53583934	-0.88791945
H	3.99753653	1.90615197	-0.14422243
H	-1.74883912	-1.89116674	1.01579108

Time = 49.50 fs
E(CASSCF/6-31G(d)) = -479.5681399

C	-0.96982868	-0.23825957	-0.13992419
C	0.57537028	-0.33475698	-0.04422999
C	1.22602636	-1.57574567	0.11774892
C	1.51723809	0.69310617	-0.11701037
C	2.82091892	0.07719327	-0.00829282
C	2.63975572	-1.31668462	0.11597270
N	-1.70847487	0.82570641	0.03229898
C	-3.15731753	0.57281958	0.10656479
H	3.40554497	-2.08413425	0.12585486
C	-1.84870197	-1.45536191	0.01538700
C	-3.27208008	-0.92612820	-0.24828639
H	-3.52319729	-1.07229305	-1.29409430
H	-4.03576442	-1.41538108	0.34521037
H	-3.51832718	0.77119457	1.10944572
H	-3.68661013	1.22789851	-0.57895622
H	-1.58649537	-2.24058556	-0.68656649
H	0.76134892	-2.56416530	0.17891982
C	-1.28425344	2.21416719	-0.00049101
H	-0.74218124	2.47063152	0.90051526
H	-0.63748263	2.34675399	-0.85613113
H	-2.14563778	2.85750299	-0.10117096
H	1.41551831	1.77952549	-0.19905118
C	4.14739240	0.83143314	-0.04372841
H	4.72098148	0.68929614	0.87169368
H	4.77060789	0.53215017	-0.88599548

H	4.00349093	1.90765270	-0.14441714
H	-1.74852168	-1.89377956	1.01387672

Time = 50.00 fs
E(CASSCF/6-31G(d)) = -479.5669600

C	-0.96944488	-0.23799783	-0.14213976
C	0.57740004	-0.33269652	-0.04425748
C	1.22329170	-1.57543346	0.11867831
C	1.51173357	0.69381054	-0.11777556
C	2.82221208	0.07317302	-0.00791070
C	2.64283477	-1.31470246	0.11620481
N	-1.70710194	0.82635338	0.03514019
C	-3.15845541	0.57180875	0.10634517
H	3.40108202	-2.08701550	0.12702958
C	-1.84784139	-1.45600356	0.01450970
C	-3.27258245	-0.92538238	-0.24834745
H	-3.52494269	-1.07360662	-1.29341464
H	-4.03548473	-1.41346137	0.34642091
H	-3.51837796	0.77339080	1.10979308
H	-3.67955699	1.23185057	-0.58101275
H	-1.59381429	-2.24195024	-0.68782482
H	0.77795580	-2.57134542	0.18191673
C	-1.28527551	2.21394573	-0.00077247
H	-0.73798639	2.46942435	0.89860418
H	-0.63684149	2.34121264	-0.85601760
H	-2.14676330	2.85741404	-0.10179228
H	1.43136820	1.77794707	-0.19964381
C	4.14595890	0.83238730	-0.04399672
H	4.72160969	0.68520821	0.87001529
H	4.77110515	0.52816957	-0.88379683
H	4.00900951	1.90876358	-0.14458954
H	-1.74809061	-1.89596730	1.01179052

Time = 50.50 fs
E(CASSCF/6-31G(d)) = -479.5655471

C	-0.96880263	-0.23817660	-0.14441287
C	0.57906494	-0.33069751	-0.04429636
C	1.22063425	-1.57524298	0.11964794
C	1.50664874	0.69465127	-0.11852771
C	2.82351929	0.06966401	-0.00758902
C	2.64574527	-1.31308826	0.11647226
N	-1.70600929	0.82737286	0.03804477
C	-3.15950229	0.57068368	0.10609881
H	3.39633367	-2.08905590	0.12818530
C	-1.84698965	-1.45668117	0.01358113

C	-3.27305343	-0.92458897	-0.24837210
H	-3.52672079	-1.07448928	-1.29261269
H	-4.03502784	-1.41165730	0.34758995
H	-3.51836529	0.77576448	1.11023871
H	-3.67237402	1.23555571	-0.58294180
H	-1.60075832	-2.24324295	-0.68899720
H	0.79493068	-2.57673942	0.18480166
C	-1.28618616	2.21365173	-0.00107721
H	-0.73391697	2.46847593	0.89662297
H	-0.63619407	2.33636797	-0.85583118
H	-2.14793371	2.85715964	-0.10230742
H	1.44631100	1.77455561	-0.20016861
C	4.14445323	0.83330042	-0.04426314
H	4.72222755	0.68085452	0.86811450
H	4.77155537	0.52395518	-0.88134227
H	4.01400346	1.90950835	-0.14474433
H	-1.74753624	-1.89771614	1.00956929

Time = 51.00 fs
E(CASSCF/6-31G(d)) = -479.5639775

C	-0.96789595	-0.23878463	-0.14674153
C	0.58030678	-0.32879270	-0.04434784
C	1.21812220	-1.57515749	0.12065883
C	1.50207942	0.69559373	-0.11926024
C	2.82481457	0.06673812	-0.00733649
C	2.64843217	-1.31187928	0.11678011
N	-1.70520775	0.82873737	0.04101214
C	-3.16043900	0.56946928	0.10583214
H	3.39152473	-2.09036258	0.12931638
C	-1.84616210	-1.45738538	0.01260045
C	-3.27348866	-0.92375325	-0.24835729
H	-3.52852981	-1.07493029	-1.29171061
H	-4.03441967	-1.41000269	0.34873536
H	-3.51832228	0.77826738	1.11068871
H	-3.66516406	1.23894470	-0.58472452
H	-1.60722599	-2.24450718	-0.69015011
H	0.81191926	-2.58041464	0.18755681
C	-1.28697071	2.21328647	-0.00139957
H	-0.73006069	2.46782410	0.89453191
H	-0.63557209	2.33228153	-0.85561127
H	-2.14910128	2.85674522	-0.10270829
H	1.46004470	1.76979110	-0.20066735
C	4.14289414	0.83414915	-0.04452512
H	4.72279300	0.67629819	0.86600807
H	4.77191857	0.51956725	-0.87866174
H	4.01839525	1.90993182	-0.14488791

H	-1.74684865	-1.89902445	1.00726591

Time = 51.50 fs
E(CASSCF/6-31G(d)) = -479.5623213

C	-0.96672763	-0.23979911	-0.14912149
C	0.58107708	-0.32701336	-0.04441303
C	1.21582141	-1.57515094	0.12171099
C	1.49810836	0.69659430	-0.11996643
C	2.82607377	0.06445395	-0.00716044
C	2.65084221	-1.31109921	0.11713259
N	-1.70469977	0.83040887	0.04403949
C	-3.16124809	0.56819171	0.10555336
H	3.38691214	-2.09109531	0.13041897
C	-1.84537309	-1.45810682	0.01156555
C	-3.27388325	-0.92288091	-0.24830004
H	-3.53036887	-1.07492491	-1.29073746
H	-4.03368833	-1.40852962	0.34987630
H	-3.51828065	0.78084697	1.11104428
H	-3.65803657	1.24195537	-0.58635098
H	-1.61312566	-2.24578646	-0.69134614
H	0.82853790	-2.58255609	0.19017350
C	-1.28761671	2.21285407	-0.00173420
H	-0.72649596	2.46750276	0.89230302
H	-0.63500578	2.32900113	-0.85539993
H	-2.15021657	2.85617949	-0.10298775
H	1.47229585	1.76420674	-0.20119044
C	4.14129999	0.83491050	-0.04478012
H	4.72326710	0.67160546	0.86372440
H	4.77215871	0.51506714	-0.87579699
H	4.02212035	1.91009471	-0.14502780
H	-1.74601795	-1.89990145	1.00494544

Time = 52.00 fs
E(CASSCF/6-31G(d)) = -479.5606574

C	-0.96530926	-0.24118660	-0.15154609
C	0.58133839	-0.32538830	-0.04449300
C	1.21379315	-1.57518987	0.12280344
C	1.49480351	0.69760300	-0.12063971
C	2.82727470	0.06285527	-0.00706659
C	2.65292615	-1.31075799	0.11753307
N	-1.70447974	0.83233988	0.04712173
C	-3.16191376	0.56687785	0.10527179
H	3.38276772	-2.09145164	0.13149093
C	-1.84463561	-1.45883669	0.01047301
C	-3.27423200	-0.92197811	-0.24819755

H	-3.53223738	-1.07447405	-1.28972696
H	-4.03286157	-1.40726659	0.35103121
H	-3.51827159	0.78344861	1.11120947
H	-3.65110512	1.24453405	-0.58782082
H	-1.61837809	-2.24711875	-0.69263855
H	0.84438910	-2.58345010	0.19265164
C	-1.28811422	2.21236119	-0.00207652
H	-0.72328808	2.46754168	0.88992381
H	-0.63452389	2.32655926	-0.85524017
H	-2.15123141	2.85547487	-0.10313998
H	1.48282786	1.75843081	-0.20179400
C	4.13968851	0.83556284	-0.04502573
H	4.72361544	0.66684471	0.86130345
H	4.77224520	0.51051643	-0.87280137
H	4.02512857	1.91006692	-0.14517208
H	-1.74503461	-1.90036520	1.00268034

Time = 52.50 fs
E(CASSCF/6-31G(d)) = -479.5590583

C	-0.96366030	-0.24290458	-0.15400618
C	0.58106573	-0.32394313	-0.04458896
C	1.21209272	-1.57523507	0.12393442
C	1.49221736	0.69856664	-0.12127376
C	2.82839645	0.06196912	-0.00705890
C	2.65464057	-1.31085141	0.11798378
N	-1.70453433	0.83447472	0.05025138
C	-3.16242254	0.56555476	0.10499730
H	3.37935693	-2.09164493	0.13253138
C	-1.84396112	-1.45956738	0.00931815
C	-3.27452974	-0.92105176	-0.24804727
H	-3.53413496	-1.07358425	-1.28871426
H	-4.03196420	-1.40623678	0.35221548
H	-3.51832683	0.78601778	1.11109799
H	-3.64448528	1.24663632	-0.58914260
H	-1.62291801	-2.24853186	-0.69406600
H	0.85907796	-2.58345826	0.19499825
C	-1.28845580	2.21181753	-0.00242302
H	-0.72048629	2.46796635	0.88739954
H	-0.63415348	2.32497312	-0.85517428
H	-2.15210135	2.85464785	-0.10316057
H	1.49144637	1.75311513	-0.20253584
C	4.13807605	0.83608636	-0.04525977
H	4.72380891	0.66208459	0.85879683
H	4.77215363	0.50597505	-0.86973945
H	4.02738467	1.90992156	-0.14532881
H	-1.74389001	-1.90044060	1.00054425

Time = 53.00 fs
E(CASSCF/6-31G(d)) = -479.5576463

C	-0.96180695	-0.24490294	-0.15649060
C	0.58024656	-0.32269942	-0.04470193
C	1.21076590	-1.57524568	0.12510151
C	1.49038488	0.69943446	-0.12186328
C	2.82942048	0.06180602	-0.00713953
C	2.65595126	-1.31136372	0.11848606
N	-1.70484362	0.83675185	0.05341935
C	-3.16276267	0.56424893	0.10473950
H	3.37691629	-2.09188259	0.13354021
C	-1.84335914	-1.46029302	0.00809525
C	-3.27477155	-0.92010912	-0.24784703
H	-3.53606098	-1.07226726	-1.28773369
H	-4.03101655	-1.40545707	0.35344108
H	-3.51848001	0.78850281	1.11064057
H	-3.63829064	1.24822854	-0.59033497
H	-1.62669673	-2.25003966	-0.69565019
H	0.87223216	-2.58298222	0.19722560
C	-1.28863682	2.21123434	-0.00277152
H	-0.71812106	2.46879807	0.88475404
H	-0.63392026	2.32424317	-0.85524293
H	-2.15278807	2.85371934	-0.10304675
H	1.49800149	1.74887203	-0.20347100
C	4.13647789	0.83646472	-0.04548038
H	4.72382523	0.65739277	0.85626640
H	4.77186710	0.50150016	-0.86668532
H	4.02887096	1.90972622	-0.14550521
H	-1.74257691	-1.90015605	0.99860639

Time = 53.50 fs
E(CASSCF/6-31G(d)) = -479.5565446

C	-0.95978237	-0.24712595	-0.15898552
C	0.57888069	-0.32167500	-0.04483301
C	1.20984977	-1.57518203	0.12630169
C	1.48932726	0.70016255	-0.12240399
C	2.83032855	0.06236049	-0.00730887
C	2.65683331	-1.31226928	0.11904021
N	-1.70538179	0.83910507	0.05661386
C	-3.16292462	0.56298616	0.10450750
H	3.37563353	-2.09233916	0.13451757
C	-1.84283707	-1.46100948	0.00679779
C	-3.27495289	-0.91915853	-0.24759520
H	-3.53801303	-1.07053898	-1.28681569

H	-4.03003246	-1.40493613	0.35471295
H	-3.51876504	0.79085578	1.10979005
H	-3.63263132	1.24928589	-0.59142279
H	-1.62968025	-2.25163960	-0.69739221
H	0.88351428	-2.58242229	0.19934805
C	-1.28865490	2.21062550	-0.00312102
H	-0.71620558	2.47005172	0.88202846
H	-0.63384899	2.32435329	-0.85548048
H	-2.15326242	2.85271405	-0.10279785
H	1.50238335	1.74621188	-0.20464562
C	4.13490803	0.83668481	-0.04568602
H	4.72364775	0.65283600	0.85378109
H	4.77137494	0.49714693	-0.86371959
H	4.02958367	1.90953773	-0.14570718
H	-1.74109065	-1.89954195	0.99692571

Time = 54.00 fs
E(CASSCF/6-31G(d)) = -479.5558614

C	-0.95762442	-0.24951445	-0.16147560
C	0.57698038	-0.32088269	-0.04498334
C	1.20936919	-1.57501074	0.12753191
C	1.48904928	0.70071990	-0.12289343
C	2.83110455	0.06361045	-0.00756550
C	2.65727461	-1.31353445	0.11964557
N	-1.70611829	0.84146633	0.05982211
C	-3.16290107	0.56179063	0.10430900
H	3.37562410	-2.09313411	0.13546366
C	-1.84240018	-1.46171469	0.00541898
C	-3.27506983	-0.91820877	-0.24729075
H	-3.53998749	-1.06841995	-1.28598394
H	-4.02901857	-1.40467501	0.35603014
H	-3.51921886	0.79303537	1.10852537
H	-3.62761020	1.24979371	-0.59243815
H	-1.63185170	-2.25331386	-0.69927433
H	0.89264515	-2.58212895	0.20137891
C	-1.28851028	2.21000605	-0.00347187
H	-0.71473330	2.47173766	0.87928015
H	-0.63396209	2.32527212	-0.85591782
H	-2.15350517	2.85166188	-0.10241504
H	1.50452468	1.74547558	-0.20609239
C	4.13337902	0.83673806	-0.04587563
H	4.72326551	0.64847763	0.85141452
H	4.77067263	0.49296627	-0.86092662
H	4.02953606	1.90939376	-0.14593854
H	-1.73942921	-1.89862740	0.99554479

Time = 54.50 fs
E(CASSCF/6-31G(d)) = -479.5557037

C	-0.95537512	-0.25200773	-0.16394439
C	0.57457033	-0.32033059	-0.04515384
C	1.20933664	-1.57470796	0.12878936
C	1.48954130	0.70109149	-0.12333105
C	2.83173358	0.06551889	-0.00790631
C	2.65727576	-1.31511993	0.12030058
N	-1.70701888	0.84376763	0.06303004
C	-3.16268708	0.56068464	0.10415018
H	3.37691925	-2.09431698	0.13637809
C	-1.84205143	-1.46240862	0.00395239
C	-3.27511919	-0.91726925	-0.24693348
H	-3.54197861	-1.06593416	-1.28525338
H	-4.02797413	-1.40466620	0.35738491
H	-3.51987919	0.79500830	1.10685296
H	-3.62331861	1.24974620	-0.59341711
H	-1.63320987	-2.25502960	-0.70126070
H	0.89941314	-2.58236127	0.20332714
C	-1.28820542	2.20939223	-0.00382544
H	-0.71368124	2.47385908	0.87657768
H	-0.63428079	2.32695238	-0.85657858
H	-2.15350830	2.85059548	-0.10190137
H	1.50439475	1.74679371	-0.20782648
C	4.13190203	0.83662054	-0.04604860
H	4.72267359	0.64437724	0.84924268
H	4.76976192	0.48900496	-0.85839204
H	4.02875653	1.90931203	-0.14620096
H	-1.73759411	-1.89743835	0.99448588

Time = 55.00 fs
E(CASSCF/6-31G(d)) = -479.5560923

C	-0.95307918	-0.25454567	-0.16637498
C	0.57168690	-0.32002217	-0.04534524
C	1.20975178	-1.57426201	0.13007177
C	1.49078012	0.70127978	-0.12371849
C	2.83220203	0.06803567	-0.00832670
C	2.65685024	-1.31698302	0.12100282
N	-1.70804683	0.84594337	0.06622306
C	-3.16228020	0.55968824	0.10403535
H	3.37946013	-2.09585997	0.13725972
C	-1.84179142	-1.46309315	0.00239252
C	-3.27509858	-0.91634982	-0.24652415
H	-3.54397839	-1.06310859	-1.28462874
H	-4.02689154	-1.40489434	0.35876335

H	-3.52078465	0.79675037	1.10480663
H	-3.61983355	1.24914543	-0.59439792
H	-1.63376847	-2.25674204	-0.70330084
H	0.90368398	-2.58325276	0.20519478
C	-1.28774464	2.20880085	-0.00418394
H	-0.71301219	2.47641195	0.87399575
H	-0.63482474	2.32933260	-0.85747867
H	-2.15327563	2.84954979	-0.10126167
H	1.50199826	1.75006873	-0.20984365
C	4.13048687	0.83633298	-0.04620479
H	4.72187216	0.64058943	0.84734030
H	4.76864957	0.48530505	-0.85619915
H	4.02728783	1.90928863	-0.14649386
H	-1.73559097	-1.89599598	0.99374796

Time = 55.50 fs
E(CASSCF/6-31G(d)) = -479.5569710

C	-0.95078345	-0.25707024	-0.16875017
C	0.56837727	-0.31995634	-0.04555796
C	1.21060119	-1.57367523	0.13137745
C	1.49272970	0.70130426	-0.12405947
C	2.83249801	0.07109941	-0.00882072
C	2.65602397	-1.31907917	0.12174925
N	-1.70916389	0.84793193	0.06938604
C	-3.16168055	0.55881899	0.10396693
H	3.38310166	-2.09766040	0.13810673
C	-1.84161863	-1.46377136	0.00073515
C	-3.27500628	-0.91546069	-0.24606452
H	-3.54597663	-1.05997306	-1.28410411
H	-4.02575766	-1.40533717	0.36014634
H	-3.52197282	0.79824671	1.10244495
H	-3.61721529	1.24799968	-0.59541777
H	-1.63355437	-2.25839979	-0.70533417
H	0.90540455	-2.58479458	0.20697564
C	-1.28713408	2.20824898	-0.00454987
H	-0.71267837	2.47938394	0.87160992
H	-0.63561126	2.33233909	-0.85862631
H	-2.15282158	2.84856015	-0.10050223
H	1.49737656	1.75498882	-0.21212018
C	4.12914238	0.83588065	-0.04634451
H	4.72086482	0.63716367	0.84577598
H	4.76734549	0.48190454	-0.85442399
H	4.02518562	1.90929751	-0.14681428
H	-1.73342998	-1.89431636	0.99330643

Time = 56.00 fs

E(CASSCF/6-31G(d)) = -479.5582153

C	-0.94853639	-0.25952660	-0.17105306
C	0.56469873	-0.32012779	-0.04579197
C	1.21185881	-1.57296377	0.13270529
C	1.49534150	0.70119852	-0.12435965
C	2.83261148	0.07463943	-0.00938130
C	2.65483383	-1.32136279	0.12253626
N	-1.71033106	0.84967713	0.07250366
C	-3.16089078	0.55809180	0.10394548
H	3.38762403	-2.09955299	0.13891669
C	-1.84152944	-1.46444696	-0.00102218
C	-3.27484120	-0.91461212	-0.24555738
H	-3.54796063	-1.05656020	-1.28366406
H	-4.02455591	-1.40596703	0.36151098
H	-3.52347786	0.79949153	1.09984767
H	-3.61550443	1.24632231	-0.59651011
H	-1.63260532	-2.25994989	-0.70729615
H	0.90460207	-2.58683601	0.20865511
C	-1.28638149	2.20775342	-0.00492554
H	-0.71262546	2.48275285	0.86949095
H	-0.63665444	2.33588682	-0.86002200
H	-2.15216954	2.84766060	-0.09963071
H	1.49061082	1.76106617	-0.21461479
C	4.12787673	0.83527298	-0.04646848
H	4.71965778	0.63414384	0.84460870
H	4.76586171	0.47883759	-0.85313198
H	4.02251624	1.90929382	-0.14715727
H	-1.73112575	-1.89241096	0.99311474

Time = 56.50 fs
E(CASSCF/6-31G(d)) = -479.5596447

C	-0.94638695	-0.26186461	-0.17326759
C	0.56071809	-0.32052743	-0.04604673
C	1.21348716	-1.57215527	0.13405477
C	1.49855525	0.70100526	-0.12462622
C	2.83253445	0.07857775	-0.01000038
C	2.65332512	-1.32378724	0.12335971
N	-1.71150940	0.85112964	0.07556092
C	-3.15991647	0.55751866	0.10396993
H	3.39275132	-2.10132979	0.13968716
C	-1.84151833	-1.46512380	-0.00288000
C	-3.27460275	-0.91381426	-0.24500654
H	-3.54991599	-1.05290541	-1.28328480
H	-4.02326859	-1.40675275	0.36283282
H	-3.52532961	0.80048782	1.09711033

H	-3.61472071	1.24413097	-0.59770310
H	-1.63096831	-2.26134315	-0.70912381
H	0.90137860	-2.58910522	0.21021205
C	-1.28549585	2.20733057	-0.00531263
H	-0.71279598	2.48648715	0.86769885
H	-0.63796399	2.33988245	-0.86165946
H	-2.15135095	2.84688317	-0.09865583
H	1.48182554	1.76769666	-0.21727339
C	4.12669734	0.83452283	-0.04657774
H	4.71825844	0.63156737	0.84388457
H	4.76421070	0.47613428	-0.85237458
H	4.01935491	1.90921908	-0.14751632
H	-1.72869701	-1.89028776	0.99310749

Time = 57.00 fs
E(CASSCF/6-31G(d)) = -479.5610902

C	-0.94438392	-0.26403970	-0.17537928
C	0.55650992	-0.32114294	-0.04632109
C	1.21543911	-1.57128552	0.13542579
C	1.50230028	0.70077091	-0.12486738
C	2.83226109	0.08283196	-0.01066926
C	2.65154879	-1.32630532	0.12421505
N	-1.71266082	0.85224811	0.07854350
C	-3.15876607	0.55710855	0.10403804
H	3.39817573	-2.10276441	0.14041612
C	-1.84157822	-1.46580521	-0.00483682
C	-3.27429067	-0.91307680	-0.24441673
H	-3.55182728	-1.04904627	-1.28293608
H	-4.02187900	-1.40766139	0.36408786
H	-3.52755079	0.80124640	1.09433638
H	-3.61486097	1.24144619	-0.59901762
H	-1.62869754	-2.26253876	-0.71076077
H	0.89590042	-2.59124564	0.21162174
C	-1.28448716	2.20699587	-0.00571176
H	-0.71313347	2.49054511	0.86627721
H	-0.63954501	2.34422627	-0.86352632
H	-2.15040310	2.84625526	-0.09758680
H	1.47118918	1.77422786	-0.22003462
C	4.12561104	0.83364567	-0.04667365
H	4.71667481	0.62946486	0.84363422
H	4.76240459	0.47382056	-0.85218662
H	4.01578400	1.90900781	-0.14788403
H	-1.72616622	-1.88795237	0.99320533

Time = 57.50 fs
E(CASSCF/6-31G(d)) = -479.5624171

C	-0.94257617	-0.26601312	-0.17737536
C	0.55215640	-0.32195936	-0.04661309
C	1.21765951	-1.57039462	0.13681836
C	1.50649649	0.70054087	-0.12509195
C	2.83178778	0.08731675	-0.01137870
C	2.64955848	-1.32886866	0.12509738
N	-1.71374843	0.85299959	0.08143772
C	-3.15745109	0.55686748	0.10414679
H	3.40358252	-2.10363685	0.14110269
C	-1.84170059	-1.46649338	-0.00688912
C	-3.27390489	-0.91240881	-0.24379356
H	-3.55367828	-1.04502255	-1.28258373
H	-4.02037364	-1.40865958	0.36525436
H	-3.53015321	0.80178447	1.09162926
H	-3.61589739	1.23829091	-0.60046476
H	-1.62585231	-2.26350798	-0.71216173
H	0.88838554	-2.59285879	0.21285921
C	-1.28336630	2.20676370	-0.00612203
H	-0.71358646	2.49487379	0.86524880
H	-0.64139744	2.34881364	-0.86560415
H	-2.14936627	2.84579685	-0.09643296
H	1.45891316	1.78002146	-0.22283487
C	4.12462422	0.83265879	-0.04675772
H	4.71491525	0.62785995	0.84387106
H	4.76045486	0.47191858	-0.85258462
H	4.01189059	1.90859499	-0.14825278
H	-1.72355886	-1.88541014	0.99332067

Time = 58.00 fs
E(CASSCF/6-31G(d)) = -479.5635181

C	-0.94101139	-0.26775255	-0.17924528
C	0.54774529	-0.32295963	-0.04691999
C	1.22008723	-1.56952295	0.13823236
C	1.51105587	0.70035543	-0.12530888
C	2.83111349	0.09194625	-0.01211916
C	2.64740784	-1.33142791	0.12600155
N	-1.71473720	0.85336010	0.08423085
C	-3.15598610	0.55679841	0.10429305
H	3.40867296	-2.10375479	0.14174735
C	-1.84187583	-1.46718884	-0.00903159
C	-3.27344539	-0.91181848	-0.24314331
H	-3.55545302	-1.04087613	-1.28219280
H	-4.01874434	-1.40971565	0.36631486
H	-3.53313601	0.80212387	1.08908461
H	-3.61777834	1.23469028	-0.60204555

H	-1.62249509	-2.26423745	-0.71329636
H	0.87909057	-2.59354773	0.21390268
C	-1.28214490	2.20664700	-0.00654083
H	-0.71411025	2.49941012	0.86461355
H	-0.64351494	2.35353802	-0.86787057
H	-2.14828134	2.84551969	-0.09520347
H	1.44524752	1.78450568	-0.22561336
C	4.12374280	0.83158044	-0.04683162
H	4.71298807	0.62676888	0.84459027
H	4.75837179	0.47044637	-0.85356561
H	4.00776419	1.90792282	-0.14861539
H	-1.72090252	-1.88266831	0.99336439

Time = 58.50 fs
E(CASSCF/6-31G(d)) = -479.5643601

C	-0.93973571	-0.26923208	-0.18098091
C	0.54336815	-0.32412517	-0.04723828
C	1.22265745	-1.56870774	0.13966740
C	1.51588454	0.70024683	-0.12552688
C	2.83023995	0.09663576	-0.01288100
C	2.64514777	-1.33393273	0.12692228
N	-1.71559433	0.85331471	0.08691130
C	-3.15438861	0.55690130	0.10447407
H	3.41318503	-2.10297164	0.14235212
C	-1.84209358	-1.46789011	-0.01125745
C	-3.27291204	-0.91131289	-0.24247276
H	-3.55713625	-1.03665070	-1.28173018
H	-4.01698951	-1.41080074	0.36725757
H	-3.53648275	0.80228918	1.08678244
H	-3.62042841	1.23067161	-0.60375022
H	-1.61868989	-2.26473053	-0.71415136
H	0.86829680	-2.59295644	0.21473663
C	-1.28083527	2.20665701	-0.00696369
H	-0.71466841	2.50408142	0.86434786
H	-0.64588464	2.35829277	-0.87030001
H	-2.14718745	2.84542545	-0.09390688
H	1.43047343	1.78721551	-0.22831622
C	4.12297200	0.83042891	-0.04689704
H	4.71090176	0.62620027	0.84576865
H	4.75616467	0.46941766	-0.85510709
H	4.00349465	1.90694740	-0.14896579
H	-1.71822587	-1.87973782	0.99325206

Time = 59.00 fs
E(CASSCF/6-31G(d)) = -479.5649451

C	-0.93879302	-0.27043212	-0.18257668
C	0.53911868	-0.32543641	-0.04756363
C	1.22530403	-1.56798002	0.14112265
C	1.52088494	0.70023716	-0.12575414
C	2.82917136	0.10130312	-0.01365460
C	2.64282411	-1.33633185	0.12785414
N	-1.71628953	0.85285732	0.08946861
C	-3.15267910	0.55717308	0.10468803
H	3.41690973	-2.10119872	0.14292144
C	-1.84234305	-1.46859347	-0.01355893
C	-3.27230462	-0.91089791	-0.24178894
H	-3.55871463	-1.03239174	-1.28116800
H	-4.01511553	-1.41189039	0.36807754
H	-3.54016001	0.80230665	1.08478186
H	-3.62375048	1.22626590	-0.60555955
H	-1.61450041	-2.26500778	-0.71473204
H	0.85629696	-2.59080119	0.21535415
C	-1.27945029	2.20680315	-0.00738434
H	-0.71523261	2.50880720	0.86440613
H	-0.64848655	2.36297350	-0.87286545
H	-2.14612009	2.84550549	-0.09255096
H	1.41489331	1.78781985	-0.23089995
C	4.12231638	0.82922200	-0.04695568
H	4.70866546	0.62615469	0.84736623
H	4.75384244	0.46884120	-0.85716831
H	3.99916997	1.90564380	-0.14929946
H	-1.71555703	-1.87663545	0.99291060

Time = 59.50 fs
E(CASSCF/6-31G(d)) = -479.5653035

C	-0.93822413	-0.27133939	-0.18402966
C	0.53509043	-0.32687293	-0.04789104
C	1.22796163	-1.56736289	0.14259674
C	1.52595789	0.70033736	-0.12599802
C	2.82791482	0.10587006	-0.01443056
C	2.64047590	-1.33857348	0.12879173
N	-1.71679539	0.85199045	0.09189358
C	-3.15088064	0.55760766	0.10493445
H	3.41970288	-2.09841392	0.14346207
C	-1.84261324	-1.46929299	-0.01592762
C	-3.27162280	-0.91057806	-0.24109891
H	-3.56017731	-1.02814607	-1.28048580
H	-4.01313686	-1.41296517	0.36877720
H	-3.54411654	0.80220276	1.08311675
H	-3.62762796	1.22150848	-0.60744581
H	-1.60998888	-2.26510555	-0.71506151

H	0.84338302	-2.58689307	0.21575831
C	-1.27800339	2.20709264	-0.00779492
H	-0.71578160	2.51350152	0.86472405
H	-0.65129384	2.36748035	-0.87553945
H	-2.14510957	2.84574044	-0.09114242
H	1.39882044	1.78613805	-0.23333361
C	4.12177947	0.82797650	-0.04700922
H	4.70629006	0.62662485	0.84932825
H	4.75141479	0.46872053	-0.85969235
H	3.99487404	1.90400964	-0.14961387
H	-1.71292261	-1.87338493	0.99228315

Time = 60.00 fs
E(CASSCF/6-31G(d)) = -479.5654692

C	-0.93806509	-0.27194652	-0.18533950
C	0.53137364	-0.32841442	-0.04821492
C	1.23056825	-1.56687005	0.14408788
C	1.53100592	0.70054679	-0.12626498
C	2.82647969	0.11026356	-0.01519977
C	2.63813369	-1.34060587	0.12972961
N	-1.71708782	0.85072484	0.09417832
C	-3.14901856	0.55819607	0.10521443
H	3.42149354	-2.09466182	0.14398296
C	-1.84289329	-1.46998071	-0.01835501
C	-3.27086624	-0.91035656	-0.24040957
H	-3.56151638	-1.02396147	-1.27967244
H	-4.01107564	-1.41401109	0.36936606
H	-3.54828330	0.80200337	1.08179485
H	-3.63192816	1.21644075	-0.60937517
H	-1.60521538	-2.26507368	-0.71517944
H	0.82983400	-2.58115284	0.21596320
C	-1.27650843	2.20753049	-0.00818644
H	-0.71629885	2.51807546	0.86522390
H	-0.65427262	2.37171933	-0.87829539
H	-2.14418035	2.84610117	-0.08968695
H	1.38256421	1.78214769	-0.23560102
C	4.12136355	0.82670765	-0.04705924
H	4.70378858	0.62759427	0.85158785
H	4.74889282	0.46905230	-0.86260841
H	3.99068594	1.90206697	-0.14990857
H	-1.71034646	-1.87001767	0.99133277

Time = 60.50 fs
E(CASSCF/6-31G(d)) = -479.5654704

C	-0.93834620	-0.27225205	-0.18650843

C	0.52805334	-0.33004015	-0.04852939
C	1.23306636	-1.56650577	0.14559380
C	1.53593417	0.70085348	-0.12656034
C	2.82487846	0.11441627	-0.01595353
C	2.63581951	-1.34237796	0.13066252
N	-1.71714647	0.84907898	0.09631640
C	-3.14711980	0.55892637	0.10553074
H	3.42228516	-2.09005367	0.14449507
C	-1.84317266	-1.47064691	-0.02083283
C	-3.27003472	-0.91023519	-0.23972741
H	-3.56272726	-1.01988608	-1.27872683
H	-4.00896057	-1.41501935	0.36986017
H	-3.55257519	0.80173300	1.08079781
H	-3.63650618	1.21111079	-0.61130959
H	-1.60023762	-2.26497105	-0.71513845
H	0.81590750	-2.57361644	0.21599365
C	-1.27497975	2.20811899	-0.00854920
H	-0.71677028	2.52244090	0.86582000
H	-0.65738309	2.37560506	-0.88110814
H	-2.14335061	2.84654960	-0.08818919
H	1.36642108	1.77598486	-0.23770150
C	4.12106930	0.82542913	-0.04710729
H	4.70117768	0.62903840	0.85406955
H	4.74629068	0.46982654	-0.86583548
H	3.98667750	1.89986120	-0.15018526
H	-1.70784934	-1.86657283	0.99004434

Time = 61.00 fs
E(CASSCF/6-31G(d)) = -479.5653086

C	-0.93909006	-0.27226011	-0.18754096
C	0.52520629	-0.33172924	-0.04882867
C	1.23540397	-1.56626516	0.14711187
C	1.54065218	0.70123495	-0.12688825
C	2.82312711	0.11826724	-0.01668361
C	2.63354727	-1.34384033	0.13158547
N	-1.71695527	0.84707873	0.09830297
C	-3.14521205	0.55978390	0.10588762
H	3.42215380	-2.08476023	0.14501093
C	-1.84344127	-1.47128071	-0.02335345
C	-3.26912829	-0.91021440	-0.23905836
H	-3.56380884	-1.01596781	-1.27765842
H	-4.00682536	-1.41598628	0.37028091
H	-3.55689415	0.80141483	1.08008532
H	-3.64121027	1.20557455	-0.61321017
H	-1.59511098	-2.26486130	-0.71500059
H	0.80183180	-2.56443383	0.21588448

C	-1.27343198	2.20885734	-0.00887337
H	-0.71718083	2.52651428	0.86642578
H	-0.66058025	2.37906267	-0.88395519
H	-2.14263269	2.84704107	-0.08665295
H	1.35066257	1.76793684	-0.23965018
C	4.12089553	0.82415305	-0.04715482
H	4.69847847	0.63092415	0.85669300
H	4.74362659	0.47102564	-0.86928588
H	3.98291245	1.89745885	-0.15044755
H	-1.70544838	-1.86309683	0.98842526

Time = 61.50 fs
E(CASSCF/6-31G(d)) = -479.5649956

C	-0.94031107	-0.27198021	-0.18844394
C	0.52289938	-0.33346080	-0.04910713
C	1.23753626	-1.56613479	0.14863922
C	1.54507629	0.70165918	-0.12725158
C	2.82124439	0.12176251	-0.01738238
C	2.63132277	-1.34494615	0.13249379
N	-1.71650268	0.84475657	0.10013486
C	-3.14332308	0.56075134	0.10629054
H	3.42124094	-2.07900207	0.14554439
C	-1.84368959	-1.47187063	-0.02591008
C	-3.26814757	-0.91029347	-0.23840766
H	-3.56476333	-1.01225352	-1.27648643
H	-4.00470640	-1.41691216	0.37065324
H	-3.56113248	0.80107129	1.07960041
H	-3.64588581	1.19989670	-0.61503973
H	-1.58988888	-2.26480713	-0.71483242
H	0.78779928	-2.55386243	0.21567953
C	-1.27187987	2.20974163	-0.00914965
H	-0.71751219	2.53021952	0.86695959
H	-0.66381571	2.38202874	-0.88681603
H	-2.14203400	2.84752611	-0.08508123
H	1.33552540	1.75842897	-0.24147759
C	4.12083893	0.82289002	-0.04720327
H	4.69571749	0.63321021	0.85937705
H	4.74092425	0.47262391	-0.87286933
H	3.97944555	1.89494388	-0.15070065
H	-1.70315661	-1.85964221	0.98650408

Time = 62.00 fs
E(CASSCF/6-31G(d)) = -479.5644917

C	-0.94201303	-0.27142704	-0.18922605
C	0.52118748	-0.33521379	-0.04935986

C	1.23942452	-1.56609411	0.15017279
C	1.54912921	0.70208682	-0.12765208
C	2.81925321	0.12485512	-0.01804275
C	2.62914642	-1.34565255	0.13338333
N	-1.71578238	0.84215115	0.10181079
C	-3.14147970	0.56180905	0.10674569
H	3.41973964	-2.07303340	0.14610961
C	-1.84390875	-1.47240531	-0.02849702
C	-3.26709406	-0.91047054	-0.23777968
H	-3.56559614	-1.00878858	-1.27523880
H	-4.00264061	-1.41780088	0.37100380
H	-3.56517853	0.80072423	1.07927687
H	-3.65038212	1.19415067	-0.61676622
H	-1.58462367	-2.26486540	-0.71470066
H	0.77396478	-2.54225256	0.21542956
C	-1.27033809	2.21076443	-0.00936986
H	-0.71773947	2.53349211	0.86735119
H	-0.66703872	2.38445357	-0.88967300
H	-2.14155826	2.84795415	-0.08347668
H	1.32120557	1.74800008	-0.24322801
C	4.12089380	0.82164949	-0.04725410
H	4.69292699	0.63584738	0.86204324
H	4.73821324	0.47458720	-0.87649651
H	3.97632187	1.89241117	-0.15095081
H	-1.70098334	-1.85626619	0.98432784

Time = 62.50 fs
E(CASSCF/6-31G(d)) = -479.5638272

C	-0.94418936	-0.27062013	-0.18989763
C	0.52011272	-0.33696718	-0.04958277
C	1.24103671	-1.56611699	0.15170944
C	1.55274159	0.70247349	-0.12809043
C	2.81717979	0.12750555	-0.01865826
C	2.62701457	-1.34592203	0.13425058
N	-1.71479340	0.83930662	0.10333130
C	-3.13970686	0.56293524	0.10725950
H	3.41787786	-2.06712558	0.14672044
C	-1.84409040	-1.47287425	-0.03110955
C	-3.26597037	-0.91074284	-0.23717805
H	-3.56631481	-1.00561566	-1.27394959
H	-4.00066245	-1.41865783	0.37135875
H	-3.56892047	0.80039575	1.07904584
H	-3.65455521	1.18841844	-0.61836423
H	-1.57936767	-2.26508071	-0.71466684
H	0.76044390	-2.53002892	0.21519071
C	-1.26882099	2.21191465	-0.00952742

H	-0.71783064	2.53628063	0.86754567
H	-0.67019881	2.38630097	-0.89250957
H	-2.14120684	2.84827481	-0.08184164
H	1.30785394	1.73727161	-0.24495736
C	4.12105202	0.82044006	-0.04730875
H	4.69014562	0.63877940	0.86461963
H	4.73552992	0.47687303	-0.88008292
H	3.97357696	1.88996065	-0.15120477
H	-1.69893449	-1.85302807	0.98195703

Time = 63.00 fs
E(CASSCF/6-31G(d)) = -479.5629981

C	-0.94682244	-0.26958358	-0.19047046
C	0.51970408	-0.33869992	-0.04977288
C	1.24234663	-1.56617376	0.15324613
C	1.55585173	0.70277327	-0.12856650
C	2.81505366	0.12968167	-0.01922316
C	2.62492209	-1.34572414	0.13509274
N	-1.71354028	0.83627171	0.10469895
C	-3.13802684	0.56410637	0.10783808
H	3.41589383	-2.06154352	0.14738978
C	-1.84422684	-1.47326842	-0.03374400
C	-3.26478056	-0.91110684	-0.23660550
H	-3.56692893	-1.00277419	-1.27265688
H	-3.99880173	-1.41948941	0.37174179
H	-3.57225242	0.80010902	1.07884423
H	-3.65827359	1.18278975	-0.61981763
H	-1.57417353	-2.26548207	-0.71478375
H	0.74731872	-2.51766422	0.21502160
C	-1.26734230	2.21317775	-0.00961780
H	-0.71774484	2.53854915	0.86750720
H	-0.67324727	2.38754916	-0.89531021
H	-2.14098026	2.84844166	-0.08017827
H	1.29558003	1.72690221	-0.24672845
C	4.12130304	0.81926987	-0.04736879
H	4.68741781	0.64194386	0.86704312
H	4.73291703	0.47943110	-0.88355144
H	3.97123661	1.88768919	-0.15146906
H	-1.69701267	-1.84998738	0.97946114

Time = 63.50 fs
E(CASSCF/6-31G(d)) = -479.5620684

C	-0.94988391	-0.26834578	-0.19095761
C	0.51997767	-0.34039097	-0.04992841
C	1.24333339	-1.56623360	0.15478010

C	1.55840575	0.70294244	-0.12907976
C	2.81290678	0.13135896	-0.01973239
C	2.62286450	-1.34503718	0.13590790
N	-1.71203314	0.83309905	0.10591829
C	-3.13645853	0.56529751	0.10848667
H	3.41401112	-2.05652354	0.14812891
C	-1.84431093	-1.47358077	-0.03639744
C	-3.26353031	-0.91155846	-0.23606402
H	-3.56744920	-1.00029945	-1.27139953
H	-3.99708115	-1.42030146	0.37217222
H	-3.57507880	0.79988889	1.07862101
H	-3.66142111	1.17736062	-0.62112027
H	-1.56909491	-2.26607926	-0.71509209
H	0.73464528	-2.50564892	0.21498091
C	-1.26591476	2.21453596	-0.00963879
H	-0.71743296	2.54027777	0.86722036
H	-0.67613939	2.38819042	-0.89805900
H	-2.14087940	2.84841403	-0.07848853
H	1.28445945	1.71753579	-0.24860563
C	4.12163398	0.81814696	-0.04743586
H	4.68479329	0.64527341	0.86926154
H	4.73042336	0.48220405	-0.88683472
H	3.96931728	1.88568377	-0.15174935
H	-1.69521773	-1.84720123	0.97691220

Time = 64.00 fs
E(CASSCF/6-31G(d)) = -479.5611379

C	-0.95333504	-0.26693930	-0.19137310
C	0.52093693	-0.34201917	-0.05004891
C	1.24398041	-1.56626719	0.15630897
C	1.56035687	0.70294369	-0.12962963
C	2.81077295	0.13252045	-0.02018171
C	2.62084023	-1.34384961	0.13669501
N	-1.71028771	0.82984469	0.10699553
C	-3.13501691	0.56648262	0.10920922
H	3.41241345	-2.05225300	0.14894689
C	-1.84433606	-1.47380673	-0.03906715
C	-3.26222716	-0.91209309	-0.23555499
H	-3.56788602	-0.99822227	-1.27021416
H	-3.99551465	-1.42109794	0.37266346
H	-3.57731828	0.79976180	1.07834269
H	-3.66389904	1.17223220	-0.62227579
H	-1.56418728	-2.26686070	-0.71561912
H	0.72246579	-2.49445836	0.21512486
C	-1.26455000	2.21596827	-0.00959057
H	-0.71683907	2.54146272	0.86669064

H	-0.67883571	2.38823034	-0.90073828
H	-2.14090608	2.84815894	-0.07677453
H	1.27454744	1.70974573	-0.25064822
C	4.12202983	0.81707973	-0.04751175
H	4.68232611	0.64869708	0.87123509
H	4.72810277	0.48512863	-0.88987684
H	3.96782721	1.88401477	-0.15204985
H	-1.69354778	-1.84472195	0.97437780

Time = 64.50 fs
E(CASSCF/6-31G(d)) = -479.5603045

C	-0.95712802	-0.26540017	-0.19173273
C	0.52257303	-0.34356396	-0.05013520
C	1.24427539	-1.56624938	0.15783114
C	1.56166666	0.70275057	-0.13021597
C	2.80868535	0.13315770	-0.02056785
C	2.61885159	-1.34216213	0.13745407
N	-1.70832482	0.82656644	0.10793959
C	-3.13371253	0.56763558	0.11000798
H	3.41122191	-2.04884822	0.14985044
C	-1.84429623	-1.47394423	-0.04175081
C	-3.26088050	-0.91270622	-0.23507919
H	-3.56825061	-0.99656692	-1.26913241
H	-3.99410647	-1.42188135	0.37322243
H	-3.57890787	0.79975715	1.07799709
H	-3.66562913	1.16750620	-0.62329809
H	-1.55950543	-2.26779565	-0.71637761
H	0.71082269	-2.48451457	0.21550398
C	-1.26325787	2.21745187	-0.00947575
H	-0.71590265	2.54211477	0.86594031
H	-0.68130538	2.38768726	-0.90332872
H	-2.14106444	2.84765237	-0.07503715
H	1.26589288	1.70397441	-0.25290346
C	4.12247403	0.81607666	-0.04759836
H	4.68007255	0.65214234	0.87293597
H	4.72601237	0.48813760	-0.89263353
H	3.96676560	1.88273141	-0.15237297
H	-1.69199882	-1.84259497	0.97191758

Time = 65.00 fs
E(CASSCF/6-31G(d)) = -479.5596885

C	-0.96120580	-0.26376807	-0.19205321
C	0.52486522	-0.34500487	-0.05018943
C	1.24420832	-1.56616163	0.15934574
C	1.56230297	0.70235031	-0.13083948

C	2.80667682	0.13327017	-0.02088849
C	2.61690661	-1.33998779	0.13818612
N	-1.70617067	0.82332419	0.10876101
C	-3.13255130	0.56873028	0.11088319
H	3.41048158	-2.04634752	0.15084339
C	-1.84418598	-1.47399388	-0.04444530
C	-3.25950158	-0.91339303	-0.23463707
H	-3.56855248	-0.99535197	-1.26817839
H	-3.99285079	-1.42265144	0.37384907
H	-3.57980476	0.79990567	1.07759532
H	-3.66655318	1.16328395	-0.62420870
H	-1.55510488	-2.26883439	-0.71736795
H	0.69977437	-2.47615903	0.21616141
C	-1.26204653	2.21896158	-0.00929902
H	-0.71456187	2.54225827	0.86500711
H	-0.68352531	2.38659117	-0.90580829
H	-2.14135993	2.84688040	-0.07327703
H	1.25855509	1.70049469	-0.25540064
C	4.12294878	0.81514690	-0.04769772
H	4.67808964	0.65553630	0.87434840
H	4.72421074	0.49116115	-0.89507247
H	3.96612471	1.88185763	-0.15271887
H	-1.69056675	-1.84085702	0.96957572

Time = 65.50 fs
E(CASSCF/6-31G(d)) = -479.5593741

C	-0.96550372	-0.26208573	-0.19235248
C	0.52778136	-0.34632195	-0.05021504
C	1.24377170	-1.56599387	0.16085282
C	1.56224117	0.70174547	-0.13150190
C	2.80477777	0.13286569	-0.02114234
C	2.61501845	-1.33735213	0.13889319
N	-1.70385620	0.82017881	0.10947220
C	-3.13153450	0.56974135	0.11183299
H	3.41015698	-2.04470773	0.15192686
C	-1.84400053	-1.47395883	-0.04714640
C	-3.25810338	-0.91414863	-0.23422897
H	-3.56879972	-0.99458931	-1.26736690
H	-3.99173233	-1.42340569	0.37453678
H	-3.57998775	0.80023929	1.07717159
H	-3.66663316	1.15966250	-0.62503471
H	-1.55103982	-2.26991253	-0.71858033
H	0.68940703	-2.46962813	0.21713109
C	-1.26092214	2.22047096	-0.00906673
H	-0.71275766	2.54192851	0.86393843
H	-0.68548156	2.38498243	-0.90815247

H	-2.14179901	2.84583930	-0.07149385
H	1.25261307	1.69938403	-0.25814706
C	4.12343567	0.81429992	-0.04781195
H	4.67643271	0.65880772	0.87546698
H	4.72275545	0.49412905	-0.89717218
H	3.96589008	1.88139138	-0.15308548
H	-1.68924753	-1.83953506	0.96737792

Time = 66.00 fs
E(CASSCF/6-31G(d)) = -479.5593876

C	-0.96995048	-0.26039844	-0.19264966
C	0.53127842	-0.34749604	-0.05021658
C	1.24296023	-1.56574534	0.16235347
C	1.56146495	0.70095367	-0.13220599
C	2.80301502	0.13196057	-0.02132931
C	2.61320466	-1.33429260	0.13957834
N	-1.70141667	0.81719136	0.11008735
C	-3.13065895	0.57064484	0.11285332
H	3.41013820	-2.04381080	0.15309919
C	-1.84373586	-1.47384445	-0.04984840
C	-3.25670031	-0.91496806	-0.23385519
H	-3.56899847	-0.99428390	-1.26670228
H	-3.99072730	-1.42413975	0.37527332
H	-3.57945747	0.80078981	1.07678071
H	-3.66585027	1.15673093	-0.62580559
H	-1.54736162	-2.27095543	-0.71999795
H	0.67984200	-2.46504058	0.21843667
C	-1.25988869	2.22195285	-0.00878638
H	-0.71043824	2.54116894	0.86278541
H	-0.68716932	2.38291073	-0.91033507
H	-2.14238784	2.84453523	-0.06968611
H	1.24816782	1.70052409	-0.26112694
C	4.12391634	0.81354528	-0.04794321
H	4.67515295	0.66188861	0.87629467
H	4.72170048	0.49697275	-0.89892047
H	3.96604128	1.88130535	-0.15346856
H	-1.68803803	-1.83864551	0.96532875

Time = 66.50 fs
E(CASSCF/6-31G(d)) = -479.5597026

C	-0.97446921	-0.25875327	-0.19296497
C	0.53530252	-0.34850919	-0.05019972
C	1.24177125	-1.56542458	0.16384979
C	1.55996801	0.70000549	-0.13295532
C	2.80141068	0.13057975	-0.02145049

C	2.61148545	-1.33085707	0.14024558
N	-1.69889097	0.81442218	0.11062217
C	-3.12991738	0.57141893	0.11393803
H	3.41025676	-2.04347870	0.15435623
C	-1.84338889	-1.47365801	-0.05254372
C	-3.25530793	-0.91584630	-0.23351615
H	-3.56915329	-0.99443368	-1.26617875
H	-3.98980593	-1.42484874	0.37604244
H	-3.57823559	0.80158785	1.07649479
H	-3.66420382	1.15456695	-0.62655010
H	-1.54411670	-2.27188469	-0.72160145
H	0.67123580	-2.46239702	0.22009110
C	-1.25894784	2.22338002	-0.00846595
H	-0.70756326	2.54002850	0.86159700
H	-0.68859230	2.38043379	-0.91232957
H	-2.14313129	2.84298422	-0.06785087
H	1.24533566	1.70362380	-0.26430325
C	4.12437309	0.81289225	-0.04809361
H	4.67429475	0.66471589	0.87684046
H	4.72109329	0.49962734	-0.90031250
H	3.96655213	1.88155019	-0.15386210
H	-1.68693601	-1.83819458	0.96341241

Time = 67.00 fs
E(CASSCF/6-31G(d)) = -479.5602445

C	-0.97897902	-0.25719810	-0.19331943
C	0.53978969	-0.34934520	-0.05017105
C	1.24020551	-1.56504817	0.16534479
C	1.55775595	0.69894099	-0.13375380
C	2.79998117	0.12875684	-0.02150826
C	2.60988157	-1.32710208	0.14089990
N	-1.69632076	0.81192988	0.11109365
C	-3.12929897	0.57204459	0.11507913
H	3.41030754	-2.04349337	0.15569188
C	-1.84295767	-1.47340822	-0.05522284
C	-3.25394252	-0.91677818	-0.23321248
H	-3.56926748	-0.99502975	-1.26578145
H	-3.98893492	-1.42552848	0.37682584
H	-3.57636324	0.80266114	1.07639728
H	-3.66170969	1.15323345	-0.62729268
H	-1.54134463	-2.27262409	-0.72337318
H	0.66377363	-2.46159071	0.22209734
C	-1.25809898	2.22472579	-0.00811320
H	-0.70410799	2.53855907	0.86041402
H	-0.68976148	2.37761648	-0.91411125
H	-2.14403086	2.84121137	-0.06598369

H	1.24423497	1.70825950	-0.26762236
C	4.12478963	0.81234932	-0.04826518
H	4.67389307	0.66723274	0.87711650
H	4.72097199	0.50203353	-0.90134836
H	3.96739125	1.88205864	-0.15425869
H	-1.68594024	-1.83817933	0.96159457

Time = 67.50 fs
E(CASSCF/6-31G(d)) = -479.5609443

C	-0.98339677	-0.25578051	-0.19373467
C	0.54466680	-0.34999020	-0.05013793
C	1.23826802	-1.56463916	0.16684235
C	1.55484779	0.69780592	-0.13460525
C	2.79873673	0.12653367	-0.02150629
C	2.60841209	-1.32309062	0.14154711
N	-1.69374947	0.80977014	0.11151974
C	-3.12878995	0.57250610	0.11626710
H	3.41007443	-2.04362037	0.15709793
C	-1.84244148	-1.47310462	-0.05787433
C	-3.25262046	-0.91775827	-0.23294504
H	-3.56934281	-0.99605626	-1.26548715
H	-3.98807908	-1.42617625	0.37760498
H	-3.57389683	0.80403210	1.07657478
H	-3.65839735	1.15277470	-0.62804890
H	-1.53907636	-2.27310400	-0.72529954
H	0.65765387	-2.46242897	0.22444908
C	-1.25733924	2.22596459	-0.00773513
H	-0.70006753	2.53681180	0.85926338
H	-0.69069449	2.37452893	-0.91565792
H	-2.14508224	2.83924834	-0.06407827
H	1.24496723	1.71392679	-0.27102017
C	4.12515171	0.81192363	-0.04845974
H	4.67397215	0.66939020	0.87713603
H	4.72136385	0.50413958	-0.90203137
H	3.96852280	1.88275276	-0.15465028
H	-1.68505033	-1.83858765	0.95982410

Time = 68.00 fs
E(CASSCF/6-31G(d)) = -479.5617064

C	-0.98763867	-0.25454608	-0.19423234
C	0.54985197	-0.35043340	-0.05010835
C	1.23596988	-1.56422450	0.16834707
C	1.55127814	0.69664753	-0.13551284
C	2.79768097	0.12396019	-0.02144954
C	2.60709189	-1.31888994	0.14219378

N	-1.69122106	0.80799420	0.11191886
C	-3.12837448	0.57279177	0.11749142
H	3.40935417	-2.04363022	0.15856499
C	-1.84184106	-1.47275724	-0.06048501
C	-3.25135789	-0.91878086	-0.23271489
H	-3.56938075	-0.99749099	-1.26526718
H	-3.98720471	-1.42679230	0.37836314
H	-3.57090522	0.80571602	1.07710947
H	-3.65430834	1.15321408	-0.62882318
H	-1.53733302	-2.27326701	-0.72737507
H	0.65307277	-2.46465777	0.22713215
C	-1.25666365	2.22707261	-0.00733732
H	-0.69545844	2.53483601	0.85815484
H	-0.69141348	2.37124559	-0.91695278
H	-2.14627438	2.83713313	-0.06212662
H	1.24760090	1.72009095	-0.27442869
C	4.12544742	0.81162029	-0.04867884
H	4.67454276	0.67114783	0.87691057
H	4.72228220	0.50590247	-0.90236554
H	3.96990656	1.88354940	-0.15502874
H	-1.68426646	-1.83940113	0.95803895

Time = 68.50 fs
E(CASSCF/6-31G(d)) = -479.5624743

C	-0.99162234	-0.25353676	-0.19483366
C	0.55525580	-0.35066775	-0.05009065
C	1.23332930	-1.56383267	0.16986402
C	1.54709788	0.69551126	-0.13647873
C	2.79681090	0.12109372	-0.02134420
C	2.60592988	-1.31456947	0.14284715
N	-1.68877870	0.80664734	0.11230933
C	-3.12803551	0.57289431	0.11874117
H	3.40797807	-2.04331902	0.16008299
C	-1.84115870	-1.47237619	-0.06304045
C	-3.25017012	-0.91983987	-0.23252325
H	-3.56938312	-0.99930578	-1.26509001
H	-3.98628177	-1.42738079	0.37908724
H	-3.56746519	0.80771889	1.07807029
H	-3.64949422	1.15455201	-0.62960606
H	-1.53612474	-2.27307150	-0.72960427
H	0.65020540	-2.46798958	0.23012599
C	-1.25606537	2.22802822	-0.00692366
H	-0.69031997	2.53267754	0.85707925
H	-0.69194373	2.36784378	-0.91798595
H	-2.14758768	2.83490836	-0.06011921
H	1.25215633	1.72623425	-0.27778237

C	4.12566772	0.81144198	-0.04892365
H	4.67560134	0.67247466	0.87644851
H	4.72372531	0.50728910	-0.90235431
H	3.97149861	1.88436716	-0.15538655
H	-1.68358858	-1.84059680	0.95617159

Time = 69.00 fs
E(CASSCF/6-31G(d)) = -479.5632052

C	-0.99526850	-0.25278913	-0.19555872
C	0.56078238	-0.35069059	-0.05009337
C	1.23037243	-1.56349138	0.17139861
C	1.54237415	0.69443821	-0.13750382
C	2.79611745	0.11799815	-0.02119762
C	2.60492767	-1.31019892	0.14351499
N	-1.68646340	0.80576735	0.11270895
C	-3.12775551	0.57281124	0.12000562
H	3.40582866	-2.04252445	0.16164195
C	-1.84039829	-1.47197135	-0.06552541
C	-3.24907116	-0.92092879	-0.23237137
H	-3.56935261	-1.00146709	-1.26492376
H	-3.98528573	-1.42795042	0.37976922
H	-3.56365678	0.81003532	1.07950460
H	-3.64401497	1.15676457	-0.63037273
H	-1.53545040	-2.27249394	-0.73200246
H	0.64918848	-2.47212996	0.23340506
C	-1.25553599	2.22881233	-0.00649614
H	-0.68471321	2.53037804	0.85600903
H	-0.69231230	2.36440219	-0.91875593
H	-2.14899333	2.83262000	-0.05804507
H	1.25859576	1.73189461	-0.28102359
C	4.12580660	0.81138844	-0.04919491
H	4.67712940	0.67334949	0.87575435
H	4.72567564	0.50827671	-0.90199994
H	3.97325180	1.88513276	-0.15571742
H	-1.68301569	-1.84214901	0.95415442

Time = 69.50 fs
E(CASSCF/6-31G(d)) = -479.5638902

C	-0.99850369	-0.25233214	-0.19642599
C	0.56633039	-0.35050414	-0.05012494
C	1.22713382	-1.56322535	0.17295632
C	1.53718950	0.69346330	-0.13858737
C	2.79558615	0.11474330	-0.02101829
C	2.60407887	-1.30584731	0.14420564
N	-1.68431268	0.80538275	0.11313415

C	-3.12751685	0.57254505	0.12127489
H	3.40285254	-2.04113979	0.16323238
C	-1.83956533	-1.47155194	-0.06792450
C	-3.24807333	-0.92204059	-0.23226039
H	-3.56929392	-1.00393687	-1.26473989
H	-3.98419945	-1.42851500	0.38040760
H	-3.55955959	0.81264701	1.08143192
H	-3.63793776	1.15980416	-0.63108327
H	-1.53529738	-2.27153008	-0.73459607
H	0.65010746	-2.47680070	0.23694129
C	-1.25506603	2.22940845	-0.00605490
H	-0.67871914	2.52797463	0.85490119
H	-0.69254684	2.36099919	-0.91927041
H	-2.15045203	2.83031557	-0.05589216
H	1.26681928	1.73669500	-0.28410665
C	4.12586133	0.81145633	-0.04949290
H	4.67909375	0.67376142	0.87482819
H	4.72809986	0.50885361	-0.90130287
H	3.97511703	1.88578603	-0.15601676
H	-1.68254479	-1.84403179	0.95192663

Time = 70.00 fs
E(CASSCF/6-31G(d)) = -479.5644596

C	-1.00126072	-0.25218609	-0.19745112
C	0.57179621	-0.35011623	-0.05019349
C	1.22365601	-1.56305405	0.17454252
C	1.53163942	0.69261379	-0.13972725
C	2.79519788	0.11140241	-0.02081536
C	2.60336858	-1.30158012	0.14492752
N	-1.68235896	0.80551148	0.11359958
C	-3.12730325	0.57210314	0.12254048
H	3.39906447	-2.03911890	0.16484546
C	-1.83866696	-1.47112673	-0.07022300
C	-3.24718695	-0.92316782	-0.23219108
H	-3.56921420	-1.00667347	-1.26451470
H	-3.98301371	-1.42909307	0.38100689
H	-3.55525046	0.81552161	1.08383914
H	-3.63133761	1.16360001	-0.63168387
H	-1.53564262	-2.27019423	-0.73742141
H	0.65298634	-2.48175783	0.24070419
C	-1.25464517	2.22980366	-0.00559848
H	-0.67243424	2.52550169	0.85370144
H	-0.69267434	2.35771225	-0.91954673
H	-2.15191480	2.82804419	-0.05364812
H	1.27666493	1.74036240	-0.28700109
C	4.12583199	0.81163888	-0.04981735

H	4.68144738	0.67370919	0.87366743
H	4.73094967	0.50901802	-0.90026352
H	3.97704387	1.88628487	-0.15628223
H	-1.68217010	-1.84622129	0.94943811

Time = 70.50 fs
E(CASSCF/6-31G(d)) = -479.5649688

C	-1.00348209	-0.25236019	-0.19864698
C	0.57707295	-0.34954002	-0.05030670
C	1.21998976	-1.56299146	0.17616232
C	1.52583132	0.69190963	-0.14091964
C	2.79493036	0.10805223	-0.02059890
C	2.60277400	-1.29745992	0.14568939
N	-1.68062898	0.80615952	0.11411735
C	-3.12709885	0.57149776	0.12379571
H	3.39455145	-2.03648246	0.16647470
C	-1.83771164	-1.47070369	-0.07240728
C	-3.24642001	-0.92430252	-0.23216390
H	-3.56912331	-1.00963208	-1.26423195
H	-3.98172739	-1.42970734	0.38157883
H	-3.55079926	0.81861234	1.08667937
H	-3.62429518	1.16806082	-0.63210929
H	-1.53645389	-2.26851714	-0.74052166
H	0.65778250	-2.48680834	0.24466422
C	-1.25426289	2.22998762	-0.00512434
H	-0.66596621	2.52298956	0.85235096
H	-0.69272139	2.35461453	-0.91961086
H	-2.15332444	2.82585429	-0.05130015
H	1.28791316	1.74274257	-0.28969352
C	4.12572176	0.81192613	-0.05016754
H	4.68413045	0.67320135	0.87226690
H	4.73416294	0.50877853	-0.89888202
H	3.97898216	1.88660674	-0.15651369
H	-1.68188245	-1.84869599	0.94665438

Time = 71.00 fs
E(CASSCF/6-31G(d)) = -479.5653252

C	-1.00512044	-0.25285229	-0.20002256
C	0.58205486	-0.34879407	-0.05047152
C	1.21619209	-1.56304451	0.17782030
C	1.51987992	0.69136260	-0.14215942
C	2.79475921	0.10476981	-0.02037925
C	2.60226522	-1.29354372	0.14649994
N	-1.67914240	0.80732004	0.11469668
C	-3.12688943	0.57074553	0.12503594

H	3.38946688	-2.03331627	0.16811568
C	-1.83670900	-1.47029019	-0.07446570
C	-3.24577809	-0.92543639	-0.23217866
H	-3.56903418	-1.01276581	-1.26388362
H	-3.98034686	-1.43038359	0.38214070
H	-3.54626926	0.82185854	1.08987321
H	-3.61689855	1.17307772	-0.63228675
H	-1.53769167	-2.26654253	-0.74394384
H	0.66438718	-2.49181754	0.24879251
C	-1.25390881	2.22995348	-0.00462930
H	-0.65942757	2.52046720	0.85079240
H	-0.69271362	2.35177465	-0.91949675
H	-2.15461712	2.82379396	-0.04883580
H	1.30029651	1.74380310	-0.29218849
C	4.12553613	0.81230511	-0.05054224
H	4.68707290	0.67225574	0.87062229
H	4.73766623	0.50815267	-0.89716158
H	3.98088254	1.88675039	-0.15671348
H	-1.68166861	-1.85143894	0.94355873

Time = 71.50 fs
E(CASSCF/6-31G(d)) = -479.5655021

C	-1.00613963	-0.25364812	-0.20158253
C	0.58663860	-0.34790200	-0.05069409
C	1.21232508	-1.56321289	0.17952033
C	1.51390438	0.69097673	-0.14344035
C	2.79465914	0.10163099	-0.02016688
C	2.60180659	-1.28988254	0.14736767
N	-1.67791137	0.80897298	0.11534347
C	-3.12666225	0.56986688	0.12625858
H	3.38402248	-2.02976533	0.16976648
C	-1.83566959	-1.46989315	-0.07638920
C	-3.24526436	-0.92656089	-0.23223445
H	-3.56896277	-1.01602659	-1.26347054
H	-3.97888479	-1.43114931	0.38271438
H	-3.54171689	0.82518704	1.09331385
H	-3.60924335	1.17852843	-0.63214067
H	-1.53931141	-2.26432281	-0.74773473
H	0.67262895	-2.49671360	0.25306230
C	-1.25357300	2.22969778	-0.00411015
H	-0.65292960	2.51796268	0.84897680
H	-0.69267603	2.34925478	-0.91924466
H	-2.15572498	2.82191040	-0.04624334
H	1.31350984	1.74363161	-0.29450841
C	4.12528259	0.81276020	-0.05093977
H	4.69019674	0.67089863	0.86873215

H	4.74137732	0.50716570	-0.89511027
H	3.98269818	1.88673479	-0.15688620
H	-1.68151086	-1.85443828	0.94015392

Time = 72.00 fs
E(CASSCF/6-31G(d)) = -479.5654599

C	-1.00651553	-0.25472147	-0.20332701
C	0.59072573	-0.34689162	-0.05097972
C	1.20845398	-1.56348919	0.18126554
C	1.50802424	0.69074880	-0.14475516
C	2.79460511	0.09870832	-0.01997208
C	2.60135859	-1.28652075	0.14830073
N	-1.67694027	0.81108514	0.11606010
C	-3.12640598	0.56888527	0.12746302
H	3.37847410	-2.02602418	0.17142790
C	-1.83460448	-1.46951919	-0.07817189
C	-3.24487958	-0.92766734	-0.23232951
H	-3.56892783	-1.01936594	-1.26300203
H	-3.97735846	-1.43203194	0.38332467
H	-3.53719283	0.82851445	1.09687461
H	-3.60143303	1.18428212	-0.63159778
H	-1.54126584	-2.26191398	-0.75193624
H	0.68228171	-2.50148670	0.25744996
C	-1.25324635	2.22922044	-0.00356426
H	-0.64657652	2.51550394	0.84686944
H	-0.69263344	2.34710929	-0.91889883
H	-2.15657907	2.82024951	-0.04351214
H	1.32722405	1.74242651	-0.29669252
C	4.12497001	0.81327377	-0.05135806
H	4.69341892	0.66916408	0.86660066
H	4.74520826	0.50584959	-0.89274342
H	3.98438588	1.88659685	-0.15703848
H	-1.68138688	-1.85768742	0.93646211

Time = 72.50 fs
E(CASSCF/6-31G(d)) = -479.5651740

C	-1.00623580	-0.25603493	-0.20525110
C	0.59422420	-0.34579429	-0.05133282
C	1.20464535	-1.56385946	0.18305816
C	1.50235641	0.69066938	-0.14609607
C	2.79457298	0.09606922	-0.01980462
C	2.60087998	-1.28349590	0.14930673
N	-1.67622575	0.81361078	0.11684527
C	-3.12611064	0.56782635	0.12865027
H	3.37310386	-2.02231953	0.17310315

C	-1.83352501	-1.46917489	-0.07981147
C	-3.24462234	-0.92874713	-0.23246120
H	-3.56895028	-1.02273571	-1.26249480
H	-3.97578764	-1.43305685	0.38399720
H	-3.53274353	0.83175012	1.10041842
H	-3.59357910	1.19020440	-0.63059249
H	-1.54350753	-2.25937033	-0.75658096
H	0.69307510	-2.50618232	0.26193481
C	-1.25292064	2.22852497	-0.00299005
H	-0.64045993	2.51311908	0.84445449
H	-0.69261098	2.34538314	-0.91850528
H	-2.15711278	2.81885588	-0.04063296
H	1.34109811	1.74047931	-0.29879449
C	4.12460811	0.81382679	-0.05179475
H	4.69665394	0.66709244	0.86423972
H	4.74906826	0.50424091	-0.89008540
H	3.98590795	1.88638715	-0.15717847
H	-1.68126971	-1.86118468	0.93252327

Time = 73.00 fs
E(CASSCF/6-31G(d)) = -479.5646452

C	-1.00529987	-0.25754132	-0.20734498
C	0.59705084	-0.34464362	-0.05175693
C	1.20096478	-1.56430450	0.18489951
C	1.49701144	0.69072414	-0.14745501
C	2.79454019	0.09377414	-0.01967356
C	2.60033042	-1.28083884	0.15039263
N	-1.67575722	0.81649286	0.11769416
C	-3.12576744	0.56671694	0.12982255
H	3.36819668	-2.01889028	0.17479753
C	-1.83244232	-1.46886697	-0.08130953
C	-3.24448923	-0.92979185	-0.23262594
H	-3.56905255	-1.02608882	-1.26197125
H	-3.97419243	-1.43424552	0.38475631
H	-3.52841321	0.83479967	1.10380830
H	-3.58580078	1.19616256	-0.62907180
H	-1.54599092	-2.25673950	-0.76168783
H	0.70470867	-2.51088980	0.26649920
C	-1.25258870	2.22761839	-0.00238736
H	-0.63465463	2.51083641	0.84173806
H	-0.69263473	2.34411103	-0.91810919
H	-2.15726513	2.81777245	-0.03759824
H	1.35479389	1.73814984	-0.30087900
C	4.12420687	0.81439965	-0.05224723
H	4.69981684	0.66472938	0.86167106
H	4.75286684	0.50237965	-0.88717146

H	3.98723341	1.88616473	-0.15731527
H	-1.68112814	-1.86493240	0.92839222

Time = 73.50 fs
E(CASSCF/6-31G(d)) = -479.5639167

C	-1.00371857	-0.25918551	-0.20959423
C	0.59913240	-0.34347442	-0.05225467
C	1.19747506	-1.56480135	0.18679005
C	1.49209136	0.69089569	-0.14882409
C	2.79448594	0.09187561	-0.01958696
C	2.59967309	-1.27857477	0.15156465
N	-1.67551751	0.81966451	0.11859868
C	-3.12536857	0.56558404	0.13098281
H	3.36401687	-2.01596442	0.17651719
C	-1.83136697	-1.46860224	-0.08267171
C	-3.24447509	-0.93079348	-0.23281926
H	-3.56925744	-1.02937944	-1.26145712
H	-3.97259093	-1.43561339	0.38562269
H	-3.52424517	0.83756876	1.10691750
H	-3.57822386	1.20202997	-0.62699945
H	-1.54867412	-2.25405831	-0.76725826
H	0.71686770	-2.51572314	0.27112772
C	-1.25224436	2.22651132	-0.00175778
H	-0.62921597	2.50868368	0.83874947
H	-0.69273223	2.34331589	-0.91775208
H	-2.15698371	2.81703970	-0.03440254
H	1.36798740	1.73583177	-0.30301642
C	4.12377591	0.81497292	-0.05271276
H	4.70282589	0.66212440	0.85892749
H	4.75651677	0.50030729	-0.88404861
H	3.98833934	1.88599141	-0.15745837
H	-1.68092751	-1.86893533	0.92413519

Time = 74.00 fs
E(CASSCF/6-31G(d)) = -479.5630627

C	-1.00151329	-0.26090667	-0.21197998
C	0.60040832	-0.34232094	-0.05282795
C	1.19423332	-1.56532567	0.18872948
C	1.48768581	0.69116594	-0.15019608
C	2.79439183	0.09041593	-0.01955161
C	2.59887760	-1.27672311	0.15282810
N	-1.67548372	0.82305120	0.11954778
C	-3.12490714	0.56445389	0.13213425
H	3.36078128	-2.01373516	0.17826891
C	-1.83030863	-1.46838780	-0.08390749

C	-3.24457332	-0.93174454	-0.23303588
H	-3.56958736	-1.03256382	-1.26097886
H	-3.97099743	-1.43716841	0.38661179
H	-3.52028354	0.83996686	1.10963927
H	-3.57097936	1.20769119	-0.62435913
H	-1.55152015	-2.25135008	-0.77327425
H	0.72924035	-2.52080105	0.27580580
C	-1.25188241	2.22521792	-0.00110467
H	-0.62417765	2.50668735	0.83554045
H	-0.69293277	2.34300886	-0.91747005
H	-2.15622740	2.81669535	-0.03104213
H	1.38038181	1.73391392	-0.30527750
C	4.12332413	0.81552835	-0.05318856
H	4.70560516	0.65933000	0.85605376
H	4.75993683	0.49806587	-0.88077628
H	3.98921149	1.88592509	-0.15761691
H	-1.68063054	-1.87319930	0.91982430

Time = 74.50 fs
E(CASSCF/6-31G(d)) = -479.5621901

C	-0.99871525	-0.26264070	-0.21447955
C	0.60083211	-0.34121546	-0.05347795
C	1.19128931	-1.56585398	0.19071687
C	1.48387022	0.69151838	-0.15156490
C	2.79424191	0.08942634	-0.01957287
C	2.59792220	-1.27529844	0.15418734
N	-1.67562824	0.82657283	0.12052791
C	-3.12437711	0.56335096	0.13327988
H	3.35863689	-2.01233919	0.18005916
C	-1.82927573	-1.46823083	-0.08502988
C	-3.24477619	-0.93263825	-0.23326985
H	-3.57006321	-1.03560063	-1.26056091
H	-3.96942095	-1.43890993	0.38773223
H	-3.51657406	0.84191078	1.11189482
H	-3.56420129	1.21304541	-0.62115648
H	-1.55449728	-2.24862354	-0.77969744
H	0.74153350	-2.52622331	0.28051825
C	-1.25149843	2.22375581	-0.00043311
H	-0.61955177	2.50487136	0.83218285
H	-0.69326725	2.34318877	-0.91729206
H	-2.15496834	2.81677342	-0.02751520
H	1.39171565	1.73273853	-0.30772700
C	4.12285928	0.81604956	-0.05367190
H	4.70808639	0.65640041	0.85310619
H	4.76305400	0.49569685	-0.87742564
H	3.98984487	1.88601379	-0.15779911

H	-1.68019862	-1.87772905	0.91553221

Time = 75.00 fs
E(CASSCF/6-31G(d)) = -479.5614261

C	-0.99536447	-0.26432270	-0.21706713
C	0.60037222	-0.34018687	-0.05420518
C	1.18868370	-1.56636627	0.19275087
C	1.48070434	0.69194057	-0.15292606
C	2.79402290	0.08892656	-0.01965462
C	2.59679559	-1.27431120	0.15564577
N	-1.67592001	0.83014629	0.12152356
C	-3.12377308	0.56229725	0.13442206
H	3.35764504	-2.01184012	0.18189345
C	-1.82827519	-1.46813845	-0.08605484
C	-3.24507508	-0.93346857	-0.23351471
H	-3.57070335	-1.03845120	-1.26022326
H	-3.96786452	-1.44082805	0.38898505
H	-3.51316397	0.84332786	1.11363930
H	-3.55802379	1.21800954	-0.61741989
H	-1.55757917	-2.24587340	-0.78647013
H	0.75348584	-2.53204954	0.28524744
C	-1.25108875	2.22214567	0.00025046
H	-0.61533014	2.50325528	0.82876416
H	-0.69376801	2.34384196	-0.91723876
H	-2.15319294	2.81730287	-0.02382154
H	1.40176743	1.73256354	-0.31041738
C	4.12238781	0.81652283	-0.05416018
H	4.71021051	0.65339035	0.85015129
H	4.76580492	0.49323998	-0.87407804
H	3.99024428	1.88628987	-0.15801160
H	-1.67959324	-1.88252596	0.91132612

Time = 75.50 fs
E(CASSCF/6-31G(d)) = -479.5608475

C	-0.99150869	-0.26588940	-0.21971403
C	0.59901353	-0.33925992	-0.05500957
C	1.18644740	-1.56684650	0.19482973
C	1.47823184	0.69242486	-0.15427691
C	2.79372324	0.08892411	-0.01979899
C	2.59549722	-1.27376747	0.15720555
N	-1.67632529	0.83368726	0.12251772
C	-3.12309093	0.56131156	0.13556225
H	3.35777482	-2.01222199	0.18377552
C	-1.82731236	-1.46811742	-0.08700079
C	-3.24546058	-0.93423041	-0.23376365

H	-3.57152264	-1.04107983	-1.25997867
H	-3.96632485	-1.44290317	0.39036274
H	-3.51010218	0.84415810	1.11486457
H	-3.55257862	1.22251914	-0.61319936
H	-1.56074332	-2.24308268	-0.79351722
H	0.76487771	-2.53827798	0.28997108
C	-1.25065002	2.22041192	0.00093851
H	-0.61148631	2.50185362	0.82538289
H	-0.69446670	2.34494347	-0.91732290
H	-2.15090299	2.81830719	-0.01996287
H	1.41035762	1.73353138	-0.31338439
C	4.12191437	0.81693729	-0.05465097
H	4.71192893	0.65035385	0.84726449
H	4.76813733	0.49073236	-0.87082362
H	3.99042360	1.88676769	-0.15825928
H	-1.67877714	-1.88758581	0.90726277

Time = 76.00 fs
E(CASSCF/6-31G(d)) = -479.5605656

C	-0.98720280	-0.26728125	-0.22239000
C	0.59675700	-0.33845396	-0.05589036
C	1.18460077	-1.56728529	0.19695162
C	1.47648007	0.69297006	-0.15561695
C	2.79333386	0.08941487	-0.02000651
C	2.59403759	-1.27366899	0.15886773
N	-1.67680910	0.83711311	0.12349260
C	-3.12232727	0.56040914	0.13670072
H	3.35890635	-2.01339086	0.18570768
C	-1.82639069	-1.46817370	-0.08788760
C	-3.24592280	-0.93491954	-0.23400980
H	-3.57253174	-1.04345374	-1.25983172
H	-3.96479317	-1.44510692	0.39185035
H	-3.50743710	0.84435639	1.11560061
H	-3.54799052	1.22652962	-0.60856460
H	-1.56397070	-2.24022638	-0.80075036
H	0.77553463	-2.54483941	0.29466190
C	-1.25017929	2.21858147	0.00162310
H	-0.60798035	2.50067283	0.82214104
H	-0.69539440	2.34645672	-0.91754835
H	-2.14811442	2.81980153	-0.01594230
H	1.41734755	1.73565635	-0.31664349
C	4.12144209	0.81728546	-0.05514203
H	4.71320350	0.64734320	0.84452620
H	4.77000990	0.48820810	-0.86775716
H	3.99040591	1.88743995	-0.15854485
H	-1.67771640	-1.89289677	0.90338371

Time = 76.50 fs
E(CASSCF/6-31G(d)) = -479.5605885

C	-0.98250752	-0.26844433	-0.22506377
C	0.59362062	-0.33778251	-0.05684611
C	1.18315356	-1.56767932	0.19911453
C	1.47545970	0.69358028	-0.15694780
C	2.79284778	0.09038325	-0.02027611
C	2.59243680	-1.27401226	0.16063219
N	-1.67733620	0.84034483	0.12442998
C	-3.12147985	0.55960131	0.13783644
H	3.36084384	-2.01518768	0.18769091
C	-1.82551180	-1.46831211	-0.08873573
C	-3.24645134	-0.93553257	-0.23424640
H	-3.57373669	-1.04554368	-1.25977738
H	-3.96325639	-1.44740318	0.39342645
H	-3.50521547	0.84389289	1.11591366
H	-3.54437397	1.23001554	-0.60360136
H	-1.56724405	-2.23727569	-0.80807357
H	0.78532794	-2.55159514	0.29928721
C	-1.24967374	2.21668360	0.00229649
H	-0.60476215	2.49971036	0.81913757
H	-0.69657953	2.34833551	-0.91791254
H	-2.14485656	2.82179233	-0.01176387
H	1.42263861	1.73882455	-0.32018940
C	4.12097259	0.81756325	-0.05563139
H	4.71400683	0.64440812	0.84201894
H	4.77139248	0.48569800	-0.86497501
H	3.99022238	1.88827850	-0.15886891
H	-1.67638145	-1.89843806	0.89971092

Time = 77.00 fs
E(CASSCF/6-31G(d)) = -479.5609035

C	-0.97748874	-0.26933174	-0.22770379
C	0.58963918	-0.33725312	-0.05787457
C	1.18210545	-1.56803070	0.20131619
C	1.47516553	0.69426352	-0.15827295
C	2.79226004	0.09180331	-0.02060515
C	2.59072273	-1.27478776	0.16249758
N	-1.67787198	0.84330889	0.12531183
C	-3.12054772	0.55889551	0.13896710
H	3.36333609	-2.01740735	0.18972525
C	-1.82467548	-1.46853587	-0.08956559
C	-3.24703538	-0.93606691	-0.23446698
H	-3.57513861	-1.04732389	-1.25980122

H	-3.96169893	-1.44974966	0.39506458
H	-3.50347992	0.84275320	1.11590147
H	-3.54182929	1.23296839	-0.59840672
H	-1.57054629	-2.23420316	-0.81538825
H	0.79416989	-2.55834647	0.30380974
C	-1.24913059	2.21474951	0.00295180
H	-0.60177696	2.49895323	0.81646143
H	-0.69804618	2.35052538	-0.91840731
H	-2.14117030	2.82427533	-0.00743262
H	1.42616975	1.74281103	-0.32399768
C	4.12050617	0.81776971	-0.05611725
H	4.71432163	0.64159490	0.83982326
H	4.77226532	0.48322947	-0.86257098
H	3.98991076	1.88923664	-0.15923020
H	-1.67474854	-1.90417871	0.89624556

Time = 77.50 fs
E(CASSCF/6-31G(d)) = -479.5614700

C	-0.97221682	-0.26990478	-0.23027922
C	0.58486397	-0.33686744	-0.05897260
C	1.18144706	-1.56834573	0.20355409
C	1.47557726	0.69502953	-0.15959745
C	2.79156761	0.09363975	-0.02098962
C	2.58892859	-1.27597884	0.16446133
N	-1.67838337	0.84593896	0.12612073
C	-3.11953149	0.55829542	0.14008926
H	3.36610098	-2.01982206	0.19181062
C	-1.82387978	-1.46884630	-0.09039666
C	-3.24766363	-0.93652069	-0.23466555
H	-3.57673369	-1.04877241	-1.25988001
H	-3.96010487	-1.45209980	0.39673539
H	-3.50226610	0.84093751	1.11568644
H	-3.54043821	1.23539383	-0.59308343
H	-1.57385913	-2.23098716	-0.82259919
H	0.80200642	-2.56485211	0.30818955
C	-1.24854698	2.21281177	0.00358368
H	-0.59897012	2.49837695	0.81418515
H	-0.69981245	2.35296520	-0.91902075
H	-2.13710594	2.82723426	-0.00295424
H	1.42791580	1.74730857	-0.32802842
C	4.12004205	0.81790663	-0.05659807
H	4.71413987	0.63894580	0.83801343
H	4.77261795	0.48082681	-0.86063194
H	3.98951380	1.89025332	-0.15962595
H	-1.67280086	-1.91007751	0.89296766

Time = 78.00 fs
E(CASSCF/6-31G(d)) = -479.5622263

C	-0.96676612	-0.27013358	-0.23276041
C	0.57936252	-0.33662176	-0.06013596
C	1.18116071	-1.56863289	0.20582525
C	1.47666026	0.69588732	-0.16092748
C	2.79076948	0.09584903	-0.02142424
C	2.58709053	-1.27756086	0.16651962
N	-1.67883939	0.84817716	0.12684017
C	-3.11843362	0.55780129	0.14119857
H	3.36885105	-2.02220491	0.19394741
C	-1.82312114	-1.46924246	-0.09124713
C	-3.24832439	-0.93689266	-0.23483670
H	-3.57851374	-1.04987145	-1.25998356
H	-3.95846022	-1.45440515	0.39840892
H	-3.50159959	0.83845895	1.11540664
H	-3.54026039	1.23730814	-0.58773391
H	-1.57716161	-2.22761623	-0.82962027
H	0.80880864	-2.57085046	0.31238635
C	-1.24791996	2.21090357	0.00418879
H	-0.59629146	2.49794512	0.81235973
H	-0.70188898	2.35558934	-0.91973919
H	-2.13272057	2.83063970	0.00166504
H	1.42788591	1.75196265	-0.33223106
C	4.11957871	0.81797804	-0.05707245
H	4.71346164	0.63649861	0.83665319
H	4.77244772	0.47851154	-0.85923344
H	3.98907743	1.89125857	-0.16005239
H	-1.67052882	-1.91608357	0.88983820

Time = 78.50 fs
E(CASSCF/6-31G(d)) = -479.5631069

C	-0.96121419	-0.26999756	-0.23511994
C	0.57321712	-0.33650764	-0.06135929
C	1.18122193	-1.56890102	0.20812613
C	1.47836700	0.69684309	-0.16226984
C	2.78986668	0.09838107	-0.02190260
C	2.58524514	-1.27950093	0.16866747
N	-1.67921189	0.84997528	0.12745502
C	-3.11725868	0.55741045	0.14229018
H	3.37131695	-2.02435096	0.19613719
C	-1.82239467	-1.46972095	-0.09213336
C	-3.24900542	-0.93718203	-0.23497569
H	-3.58046631	-1.05060757	-1.26007648
H	-3.95675493	-1.45661742	0.40005671

H	-3.50149246	0.83534130	1.11520515
H	-3.54132976	1.23873360	-0.58245374
H	-1.58042966	-2.22409228	-0.83637894
H	0.81456458	-2.57608395	0.31636237
C	-1.24724655	2.20905805	0.00476625
H	-0.59369838	2.49760990	0.81101142
H	-0.70427766	2.35832976	-0.92054918
H	-2.12807592	2.83444876	0.00641872
H	1.42612279	1.75640760	-0.33654970
C	4.11911416	0.81798946	-0.05753913
H	4.71229380	0.63428610	0.83579197
H	4.77175829	0.47630277	-0.85843582
H	3.98864860	1.89217975	-0.16050535
H	-1.66793086	-1.92213772	0.88680208

Time = 79.00 fs
E(CASSCF/6-31G(d)) = -479.5640506

C	-0.95564124	-0.26948544	-0.23733305
C	0.56652393	-0.33651282	-0.06263594
C	1.18160040	-1.56915733	0.21045250
C	1.48063827	0.69789844	-0.16363164
C	2.78886246	0.10118063	-0.02241740
C	2.58342738	-1.28175793	0.17089880
N	-1.67947596	0.85129551	0.12795171
C	-3.11601369	0.55711782	0.14335924
H	3.37326737	-2.02609469	0.19838317
C	-1.82169439	-1.47027571	-0.09306980
C	-3.24969394	-0.93738842	-0.23507843
H	-3.58257558	-1.05097207	-1.26012092
H	-3.95498483	-1.45869069	0.40165403
H	-3.50194000	0.83161641	1.11521871
H	-3.54365171	1.23969444	-0.57732579
H	-1.58363535	-2.22043261	-0.84282025
H	0.81927182	-2.58032189	0.32008505
C	-1.24652383	2.20730744	0.00531781
H	-0.59115792	2.49731309	0.81014033
H	-0.70697088	2.36111812	-0.92143918
H	-2.12323592	2.83860490	0.01130012
H	1.42270105	1.76029951	-0.34092819
C	4.11864632	0.81794728	-0.05799697
H	4.71064882	0.63233579	0.83546155
H	4.77055809	0.47421762	-0.85828074
H	3.98827341	1.89294814	-0.16098082
H	-1.66501312	-1.92817465	0.88379320

Time = 79.50 fs

E(CASSCF/6-31G(d)) = -479.5650038

C	-0.95012952	-0.26859504	-0.23937829
C	0.55939130	-0.33662227	-0.06395787
C	1.18226132	-1.56940586	0.21279935
C	1.48340493	0.69904929	-0.16501982
C	2.78776228	0.10418901	-0.02296061
C	2.58166872	-1.28428303	0.17320658
N	-1.67961030	0.85211098	0.12831844
C	-3.11470831	0.55691651	0.14440139
H	3.37452464	-2.02732278	0.20069032
C	-1.82101351	-1.47089812	-0.09406900
C	-3.25037668	-0.93751172	-0.23514151
H	-3.58482318	-1.05096114	-1.26007941
H	-3.95315292	-1.46058322	0.40318162
H	-3.50291772	0.82732159	1.11556655
H	-3.54720091	1.24021302	-0.57241520
H	-1.58674693	-2.21667034	-0.84890929
H	0.82293133	-2.58338007	0.32352951
C	-1.24574911	2.20568224	0.00584787
H	-0.58864747	2.49698788	0.80972090
H	-0.70995135	2.36388786	-0.92240062
H	-2.11826484	2.84303843	0.01630252
H	1.41772476	1.76334380	-0.34531471
C	4.11817320	0.81785807	-0.05844494
H	4.70854377	0.63066967	0.83567381
H	4.76885918	0.47227160	-0.85878865
H	3.98799522	1.89350515	-0.16147555
H	-1.66178908	-1.93412561	0.88074018

Time = 80.00 fs
E(CASSCF/6-31G(d)) = -479.5659051

C	-0.94476267	-0.26733283	-0.24123781
C	0.55193839	-0.33681917	-0.06531556
C	1.18316643	-1.56964638	0.21516081
C	1.48658973	0.70028542	-0.16644104
C	2.78657384	0.10734520	-0.02352356
C	2.57999568	-1.28702027	0.17558293
N	-1.67959749	0.85240602	0.12854537
C	-3.11335512	0.55679836	0.14541331
H	3.37497539	-2.02798167	0.20306571
C	-1.82034483	-1.47157703	-0.09514174
C	-3.25103996	-0.93755209	-0.23516213
H	-3.58718909	-1.05057614	-1.25991754
H	-3.95127013	-1.46225887	0.40462682
H	-3.50437888	0.82249740	1.11634021

H	-3.55192007	1.24030695	-0.56776544
H	-1.58972940	-2.21285411	-0.85463227
H	0.82554392	-2.58513506	0.32668013
C	-1.24492011	2.20421041	0.00636323
H	-0.58615388	2.49656141	0.80970462
H	-0.71319247	2.36657600	-0.92342859
H	-2.11322589	2.84766749	0.02141922
H	1.41132503	1.76531453	-0.34966552
C	4.11769307	0.81772797	-0.05888204
H	4.70599948	0.62930400	0.83641930
H	4.76667608	0.47047876	-0.85995722
H	3.98785285	1.89380712	-0.16198748
H	-1.65827903	-1.93992164	0.87757242

Time = 80.50 fs
E(CASSCF/6-31G(d)) = -479.5667046

C	-0.93962478	-0.26571335	-0.24289778
C	0.54429239	-0.33708584	-0.06669828
C	1.18427512	-1.56987374	0.21753035
C	1.49010904	0.70159058	-0.16790128
C	2.78530724	0.11058754	-0.02409728
C	2.57842892	-1.28990810	0.17801930
N	-1.67942410	0.85217605	0.12862460
C	-3.11196940	0.55675423	0.14639324
H	3.37457637	-2.02808093	0.20551830
C	-1.81968097	-1.47229897	-0.09629737
C	-3.25166970	-0.93750981	-0.23513796
H	-3.58965231	-1.04982372	-1.25960666
H	-3.94935549	-1.46368847	0.40598466
H	-3.50625382	0.81718571	1.11759690
H	-3.55772064	1.23998742	-0.56339700
H	-1.59254451	-2.20904540	-0.85999625
H	0.82710640	-2.58553401	0.32953220
C	-1.24403513	2.20291654	0.00687276
H	-0.58367112	2.49595806	0.81002451
H	-0.71665901	2.36912502	-0.92452218
H	-2.10818009	2.85240066	0.02664361
H	1.40365516	1.76606873	-0.35394757
C	4.11720469	0.81756234	-0.05930739
H	4.70304056	0.62824910	0.83766753
H	4.76402584	0.46885165	-0.86176126
H	3.98787945	1.89382995	-0.16251611
H	-1.65450823	-1.94549616	0.87422625

Time = 81.00 fs
E(CASSCF/6-31G(d)) = -479.5673373

C	-0.93479949	-0.26375848	-0.24434843
C	0.53658624	-0.33740453	-0.06809420
C	1.18554552	-1.57007776	0.21990099
C	1.49387489	0.70294302	-0.16940569
C	2.78397496	0.11385474	-0.02467261
C	2.57698251	-1.29288054	0.18050659
N	-1.67908078	0.85142723	0.12855023
C	-3.11056903	0.55677424	0.14734136
H	3.37335554	-2.02769187	0.20805861
C	-1.81901477	-1.47304843	-0.09754427
C	-3.25225160	-0.93738537	-0.23506713
H	-3.59219146	-1.04871573	-1.25912567
H	-3.94743531	-1.46485021	0.40725782
H	-3.50845010	0.81142860	1.11935503
H	-3.56448433	1.23925880	-0.55930715
H	-1.59515194	-2.20531520	-0.86502688
H	0.82761071	-2.58459768	0.33209223
C	-1.24309323	2.20182124	0.00738677
H	-0.58119718	2.49510297	0.81060074
H	-0.72030836	2.37148423	-0.92568391
H	-2.10318585	2.85713946	0.03196913
H	1.39488623	1.76555196	-0.35814022
C	4.11670720	0.81736536	-0.05972018
H	4.69969541	0.62750928	0.83936800
H	4.76092808	0.46740122	-0.86415349
H	3.98810142	1.89357120	-0.16306275
H	-1.65050573	-1.95078772	0.87065002

Time = 81.50 fs
E(CASSCF/6-31G(d)) = -479.5677406

C	-0.93036850	-0.26149677	-0.24558422
C	0.52895611	-0.33775805	-0.06949043
C	1.18693550	-1.57024360	0.22226515
C	1.49779738	0.70431617	-0.17095864
C	2.78259147	0.11708725	-0.02524031
C	2.57566387	-1.29586883	0.18303540
N	-1.67856242	0.85017630	0.12831846
C	-3.10917419	0.55684792	0.14825986
H	3.37140900	-2.02694219	0.21069896
C	-1.81833949	-1.47380843	-0.09889014
C	-3.25277148	-0.93717946	-0.23494804
H	-3.59478583	-1.04726911	-1.25846263
H	-3.94554214	-1.46572967	0.40845591
H	-3.51085401	0.80526887	1.12159362
H	-3.57206558	1.23812022	-0.55547168

H	-1.59751104	-2.20173999	-0.86976539
H	0.82704445	-2.58241864	0.33437761
C	-1.24209435	2.20094035	0.00791639
H	-0.57873131	2.49392523	0.81134619
H	-0.72409293	2.37361066	-0.92691838
H	-2.09829921	2.86178073	0.03738936
H	1.38520207	1.76379913	-0.36223659
C	4.11620001	0.81713999	-0.06011973
H	4.69599650	0.62708249	0.84145293
H	4.75740537	0.46613602	-0.86706696
H	3.98853736	1.89305039	-0.16363047
H	-1.64630377	-1.95574317	0.86680871

Time = 82.00 fs
E(CASSCF/6-31G(d)) = -479.5678621

C	-0.92641054	-0.25896262	-0.24660385
C	0.52153863	-0.33813022	-0.07087328
C	1.18840381	-1.57035248	0.22461514
C	1.50178693	0.70567968	-0.17256362
C	2.78117269	0.12022784	-0.02579116
C	2.57447374	-1.29880288	0.18559603
N	-1.67786791	0.84844971	0.12792753
C	-3.10780684	0.55696407	0.14915306
H	3.36889225	-2.02600633	0.21345324
C	-1.81764910	-1.47456095	-0.10034255
C	-3.25321552	-0.93689314	-0.23477934
H	-3.59741577	-1.04550568	-1.25761564
H	-3.94371313	-1.46631960	0.40959456
H	-3.51333429	0.79875080	1.12425644
H	-3.58029623	1.23656848	-0.55184898
H	-1.59958184	-2.19839696	-0.87426438
H	0.82539260	-2.57915308	0.33641543
C	-1.24103932	2.20028468	0.00847288
H	-0.57626992	2.49236154	0.81217311
H	-0.72796182	2.37546974	-0.92823103
H	-2.09357417	2.86622070	0.04289805
H	1.37479403	1.76092816	-0.36624332
C	4.11568270	0.81688803	-0.06050552
H	4.69198112	0.62696020	0.84384085
H	4.75348434	0.46506157	-0.87041835
H	3.98919760	1.89230792	-0.16422396
H	-1.64193565	-1.96031963	0.86268711

Time = 82.50 fs
E(CASSCF/6-31G(d)) = -479.5676730

C	-0.92299950	-0.25619565	-0.24741012
C	0.51446802	-0.33850612	-0.07222873
C	1.18991053	-1.57038288	0.22694332
C	1.50575586	0.70700057	-0.17422323
C	2.77973591	0.12322291	-0.02631620
C	2.57340730	-1.30161360	0.18817879
N	-1.67700038	0.84628303	0.12737781
C	-3.10648980	0.55711054	0.15002715
H	3.36600629	-2.02509106	0.21633613
C	-1.81693840	-1.47528762	-0.10190926
C	-3.25357058	-0.93652783	-0.23455980
H	-3.60006268	-1.04345154	-1.25659211
H	-3.94198704	-1.46661867	0.41069342
H	-3.51574684	0.79192085	1.12725790
H	-3.58899074	1.23460092	-0.54838440
H	-1.60132705	-2.19535744	-0.87858167
H	0.82263972	-2.57500937	0.33824124
C	-1.23992994	2.19985961	0.00906692
H	-0.57380398	2.49035901	0.81299841
H	-0.73186353	2.37703601	-0.92962613
H	-2.08906300	2.87035788	0.04848925
H	1.36385550	1.75713047	-0.37017975
C	4.11515470	0.81661029	-0.06087723
H	4.68769210	0.62712749	0.84644039
H	4.74919670	0.46417968	-0.87411162
H	3.99008454	1.89140176	-0.16484925
H	-1.63743517	-1.96448529	0.85829050

Time = 83.00 fs
E(CASSCF/6-31G(d)) = -479.5671590

C	-0.92020277	-0.25323998	-0.24800979
C	0.50787308	-0.33887207	-0.07354270
C	1.19141752	-1.57031206	0.22924235
C	1.50962010	0.70824469	-0.17593935
C	2.77829946	0.12602302	-0.02680686
C	2.57245552	-1.30423497	0.19077424
N	-1.67596704	0.84372007	0.12667173
C	-3.10524589	0.55727408	0.15088989
H	3.36297910	-2.02441856	0.21936254
C	-1.81620319	-1.47597014	-0.10359862
C	-3.25382458	-0.93608554	-0.23428827
H	-3.60270963	-1.04113682	-1.25540852
H	-3.94040246	-1.46663084	0.41177473
H	-3.51794089	0.78482959	1.13049287
H	-3.59795310	1.23222003	-0.54501634
H	-1.60271331	-2.19268306	-0.88277527

H	0.81877603	-2.57022877	0.33989647
C	-1.23876890	2.19966484	0.00970800
H	-0.57131624	2.48787803	0.81374927
H	-0.73574784	2.37829371	-0.93110517
H	-2.08481657	2.87409696	0.05415731
H	1.35257947	1.75265328	-0.37407536
C	4.11461509	0.81630699	-0.06123478
H	4.68317851	0.62756335	0.84915517
H	4.74458045	0.46348796	-0.87804273
H	3.99119205	1.89040238	-0.16551321
H	-1.63283536	-1.96822054	0.85364586

Time = 83.50 fs
E(CASSCF/6-31G(d)) = -479.5663638

C	-0.91807881	-0.25014362	-0.24841318
C	0.50187395	-0.33921587	-0.07480158
C	1.19288878	-1.57011768	0.23150559
C	1.51330090	0.70937825	-0.17771324
C	2.77688197	0.12858382	-0.02725505
C	2.57160689	-1.30660652	0.19337327
N	-1.67477919	0.84081187	0.12581393
C	-3.10409697	0.55744009	0.15175019
H	3.36004304	-2.02420334	0.22254665
C	-1.81544029	-1.47659112	-0.10541969
C	-3.25396687	-0.93556890	-0.23396371
H	-3.60534057	-1.03859478	-1.25408859
H	-3.93899437	-1.46636372	0.41286036
H	-3.51976577	0.77753279	1.13384714
H	-3.60698371	1.22943719	-0.54168272
H	-1.60371287	-2.19041963	-0.88689751
H	0.81380104	-2.56506679	0.34142583
C	-1.23755960	2.19969461	0.01040378
H	-0.56877969	2.48489406	0.81436666
H	-0.73956810	2.37923641	-0.93266507
H	-2.08088480	2.87735197	0.05989685
H	1.34115264	1.74778087	-0.37796747
C	4.11406237	0.81597810	-0.06157839
H	4.67849636	0.62824032	0.85188849
H	4.73968110	0.46297849	-0.88210428
H	3.99250700	1.88938772	-0.16622297
H	-1.62816777	-1.97151705	0.84879882

Time = 84.00 fs
E(CASSCF/6-31G(d)) = -479.5653524

C	-0.91667480	-0.24695791	-0.24863379

C	0.49657917	-0.33952672	-0.07599268
C	1.19429020	-1.56977958	0.23372735
C	1.51672567	0.71036950	-0.17954580
C	2.77550221	0.13086661	-0.02765331
C	2.57084981	-1.30867575	0.19596745
N	-1.67345231	0.83761580	0.12481125
C	-3.10306287	0.55759257	0.15261762
H	3.35740971	-2.02462969	0.22590066
C	-1.81464759	-1.47713464	-0.10738227
C	-3.25398862	-0.93498138	-0.23358520
H	-3.60794036	-1.03586125	-1.25266129
H	-3.93779181	-1.46582745	0.41396982
H	-3.52107819	0.77009234	1.13720874
H	-3.61588749	1.22627634	-0.53832683
H	-1.60430484	-2.18859359	-0.89099042
H	0.80773063	-2.55976990	0.34287443
C	-1.23630600	2.19993785	0.01115969
H	-0.56615679	2.48139903	0.81480801
H	-0.74328305	2.37986761	-0.93429695
H	-2.07731648	2.88004939	0.06570298
H	1.32975431	1.74281032	-0.38189739
C	4.11349425	0.81562384	-0.06190863
H	4.67370876	0.62912492	0.85454793
H	4.73455237	0.46263722	-0.88619029
H	3.99400986	1.88843644	-0.16698535
H	-1.62346210	-1.97437697	0.84381070

Time = 84.50 fs
E(CASSCF/6-31G(d)) = -479.5642386

C	-0.91602581	-0.24373691	-0.24868832
C	0.49208437	-0.33979480	-0.07710455
C	1.19558881	-1.56928155	0.23590303
C	1.51982812	0.71119033	-0.18143788
C	2.77417887	0.13283831	-0.02799487
C	2.57017505	-1.31039977	0.19854925
N	-1.67200582	0.83419451	0.12367271
C	-3.10216039	0.55771407	0.15350187
H	3.35524615	-2.02583249	0.22943389
C	-1.81382399	-1.47758660	-0.10949674
C	-3.25388310	-0.93432735	-0.23315200
H	-3.61049457	-1.03297378	-1.25115846
H	-3.93681576	-1.46503352	0.41511874
H	-3.52174784	0.76257704	1.14047795
H	-3.62447984	1.22277697	-0.53490187
H	-1.60447594	-2.18721015	-0.89508195
H	0.80060486	-2.55455266	0.34428483

C	-1.23501251	2.20037841	0.01197875
H	-0.56340057	2.47740104	0.81504841
H	-0.74685869	2.38020009	-0.93598455
H	-2.07415840	2.88212946	0.07157169
H	1.31855857	1.73802652	-0.38590543
C	4.11290751	0.81524520	-0.06222644
H	4.66888598	0.63017874	0.85704955
H	4.72925683	0.46244433	-0.89020066
H	3.99567479	1.88762123	-0.16780617
H	-1.61874622	-1.97681142	0.83875477

Time = 85.00 fs
E(CASSCF/6-31G(d)) = -479.5631271

C	-0.91615230	-0.24053703	-0.24859588
C	0.48846963	-0.34001119	-0.07812748
C	1.19675223	-1.56861272	0.23802922
C	1.52254838	0.71181788	-0.18339053
C	2.77293046	0.13447191	-0.02827373
C	2.56957789	-1.31174723	0.20111234
N	-1.67046317	0.83061496	0.12240957
C	-3.10140239	0.55778587	0.15441224
H	3.35365425	-2.02787821	0.23315138
C	-1.81296934	-1.47793518	-0.11177363
C	-3.25364595	-0.93361213	-0.23266367
H	-3.61298889	-1.02997104	-1.24961233
H	-3.93607728	-1.46399406	0.41631699
H	-3.52166395	0.75506209	1.14357636
H	-3.63259434	1.21899559	-0.53137557
H	-1.60422092	-2.18625238	-0.89918421
H	0.79249421	-2.54957902	0.34569452
C	-1.23368384	2.20099544	0.01286137
H	-0.56045577	2.47292459	0.81508053
H	-0.75026883	2.38025646	-0.93770481
H	-2.07145442	2.88354831	0.07749988
H	1.30773486	1.73367790	-0.39002660
C	4.11229786	0.81484440	-0.06253313
H	4.66410477	0.63135906	0.85932117
H	4.72386565	0.46237395	-0.89404490
H	3.99747082	1.88700206	-0.16868961
H	-1.61404640	-1.97883799	0.83371025

Time = 85.50 fs
E(CASSCF/6-31G(d)) = -479.5621390

C	-0.91705849	-0.23741641	-0.24837771
C	0.48579766	-0.34016790	-0.07905372

C	1.19774851	-1.56776833	0.24010376
C	1.52483364	0.71223571	-0.18540528
C	2.77177470	0.13574708	-0.02848482
C	2.56905925	-1.31269959	0.20365180
N	-1.66885171	0.82694736	0.12103527
C	-3.10079718	0.55778839	0.15535733
H	3.35266001	-2.03075462	0.23705276
C	-1.81208436	-1.47817115	-0.11422313
C	-3.25327543	-0.93284205	-0.23212021
H	-3.61540874	-1.02689186	-1.24805296
H	-3.93557645	-1.46272110	0.41756757
H	-3.52073974	0.74762842	1.14645331
H	-3.64008767	1.21500631	-0.52773288
H	-1.60354281	-2.18568189	-0.90329291
H	0.78350302	-2.54494896	0.34713416
C	-1.23232479	2.20176414	0.01380559
H	-0.55726231	2.46800900	0.81491244
H	-0.75349571	2.38006824	-0.93942770
H	-2.06924378	2.88427800	0.08348532
H	1.29744837	1.72995648	-0.39428696
C	4.11166014	0.81442506	-0.06283041
H	4.65944736	0.63261965	0.86130482
H	4.71845795	0.46239441	-0.89764507
H	3.99936317	1.88662150	-0.16963770
H	-1.60938813	-1.98047792	0.82875618

Time = 86.00 fs
E(CASSCF/6-31G(d)) = -479.5613661

C	-0.91873230	-0.23443441	-0.24805695
C	0.48411371	-0.34025750	-0.07987768
C	1.19854591	-1.56674996	0.24212578
C	1.52663776	0.71243433	-0.18748426
C	2.77072820	0.13664948	-0.02862394
C	2.56862604	-1.31325056	0.20616419
N	-1.66720225	0.82326396	0.11956523
C	-3.10034808	0.55770144	0.15634470
H	3.35221026	-2.03436938	0.24113200
C	-1.81117061	-1.47828798	-0.11685434
C	-3.25277242	-0.93202447	-0.23152225
H	-3.61773896	-1.02377444	-1.24650600
H	-3.93530192	-1.46122645	0.41886638
H	-3.51891562	0.74036151	1.14909055
H	-3.64684370	1.21090024	-0.52397700
H	-1.60245217	-2.18544158	-0.90738923
H	0.77377201	-2.54069245	0.34862653
C	-1.23094012	2.20265655	0.01480735

H	-0.55375867	2.46270626	0.81456468
H	-0.75652997	2.37967508	-0.94111749
H	-2.06755912	2.88430660	0.08952714
H	1.28786284	1.72698380	-0.39870028
C	4.11098834	0.81399250	-0.06312034
H	4.65499973	0.63391232	0.86295794
H	4.71311948	0.46246937	-0.90093781
H	4.00131385	1.88650062	-0.17064996
H	-1.60479648	-1.98175345	0.82396535

Time = 86.50 fs
E(CASSCF/6-31G(d)) = -479.5608766

C	-0.92114479	-0.23165118	-0.24765842
C	0.48344465	-0.34027299	-0.08059605
C	1.19911346	-1.56556520	0.24409560
C	1.52792186	0.71241121	-0.18963014
C	2.76980579	0.13717094	-0.02868792
C	2.56829029	-1.31340544	0.20864758
N	-1.66554876	0.81963819	0.11801671
C	-3.10005329	0.55750483	0.15738062
H	3.35218094	-2.03855758	0.24537730
C	-1.81023037	-1.47828180	-0.11967446
C	-3.25214049	-0.93116766	-0.23087119
H	-3.61996340	-1.02065568	-1.24499087
H	-3.93523139	-1.45952185	0.42020237
H	-3.51616121	0.73334976	1.15150375
H	-3.65277580	1.20678355	-0.52012936
H	-1.60096650	-2.18545949	-0.91144258
H	0.76347597	-2.53677280	0.35018669
C	-1.22953445	2.20364226	0.01586108
H	-0.54988694	2.45707885	0.81406618
H	-0.75936984	2.37912375	-0.94273443
H	-2.06642427	2.88363702	0.09562539
H	1.27914043	1.72480568	-0.40326672
C	4.11027597	0.81355365	-0.06340529
H	4.65084974	0.63518835	0.86425371
H	4.70794086	0.46255903	-0.90387533
H	4.00328258	1.88663729	-0.17172318
H	-1.60029717	-1.98268511	0.81939881

Time = 87.00 fs
E(CASSCF/6-31G(d)) = -479.5606903

C	-0.92425064	-0.22912663	-0.24720825
C	0.48379875	-0.34020819	-0.08120784
C	1.19942228	-1.56422612	0.24601459

C	1.52865575	0.71216991	-0.19184607
C	2.76901936	0.13731001	-0.02867470
C	2.56806725	-1.31318020	0.21110169
N	-1.66392778	0.81614317	0.11640860
C	-3.09990607	0.55717915	0.15847004
H	3.35239510	-2.04309793	0.24977181
C	-1.80926656	-1.47815136	-0.12268810
C	-3.25138575	-0.93028085	-0.23016938
H	-3.62206539	-1.01757025	-1.24351961
H	-3.93533274	-1.45761963	0.42155886
H	-3.51247547	0.72668192	1.15374199
H	-3.65782853	1.20277376	-0.51622662
H	-1.59910906	-2.18565368	-0.91541516
H	0.75281730	-2.53309594	0.35182305
C	-1.22811201	2.20468975	0.01696023
H	-0.54559757	2.45119602	0.81344814
H	-0.76202124	2.37846764	-0.94423708
H	-2.06585147	2.88228554	0.10178218
H	1.27143880	1.72339413	-0.40797340
C	4.10951634	0.81311675	-0.06368788
H	4.64708418	0.63639984	0.86517996
H	4.70301477	0.46262136	-0.90642540
H	4.00522776	1.88700683	-0.17285156
H	-1.59591704	-1.98328988	0.81510054

Time = 87.50 fs
E(CASSCF/6-31G(d)) = -479.5607914

C	-0.92798811	-0.22691997	-0.24673363
C	0.48516572	-0.34005729	-0.08171438
C	1.19944603	-1.56274825	0.24788481
C	1.52881747	0.71171930	-0.19413532
C	2.76837824	0.13707112	-0.02858332
C	2.56797371	-1.31259898	0.21352774
N	-1.66237801	0.81285095	0.11476111
C	-3.09989488	0.55670639	0.15961650
H	3.35264300	-2.04773178	0.25429396
C	-1.80828277	-1.47789785	-0.12589644
C	-3.25051665	-0.92937392	-0.22942019
H	-3.62402762	-1.01455038	-1.24209693
H	-3.93556595	-1.45553419	0.42291463
H	-3.50788715	0.72044513	1.15588403
H	-3.66197712	1.19899600	-0.51231916
H	-1.59690770	-2.18593824	-0.91926734
H	0.74201911	-2.52952769	0.35353804
C	-1.22667685	2.20576670	0.01809805
H	-0.54085396	2.44513270	0.81274129

H	-0.76449458	2.37776552	-0.94558589
H	-2.06583926	2.88028084	0.10799998
H	1.26490911	1.72265747	-0.41279514
C	4.10870301	0.81269106	-0.06397089
H	4.64378577	0.63750136	0.86573722
H	4.69843300	0.46261422	-0.90857002
H	4.00710665	1.88756311	-0.17402677
H	-1.59168561	-1.98358017	0.81109518

Time = 88.00 fs
E(CASSCF/6-31G(d)) = -479.5611835

C	-0.93228119	-0.22508853	-0.24626261
C	0.48751738	-0.33981517	-0.08211914
C	1.19916296	-1.56114879	0.24970902
C	1.52839512	0.71107194	-0.19650092
C	2.76788800	0.13646482	-0.02841406
C	2.56802514	-1.31169215	0.21592832
N	-1.66093936	0.80983126	0.11309535
C	-3.10000411	0.55607090	0.16082228
H	3.35270839	-2.05218824	0.25891921
C	-1.80728315	-1.47752465	-0.12929675
C	-3.24954374	-0.92845729	-0.22862817
H	-3.62583281	-1.01162512	-1.24072030
H	-3.93588522	-1.45328270	0.42424610
H	-3.50245121	0.71472205	1.15803215
H	-3.66522489	1.19557690	-0.50846486
H	-1.59439349	-2.18622845	-0.92296223
H	0.73131165	-2.52591371	0.35533065
C	-1.22523257	2.20684112	0.01926825
H	-0.53563762	2.43896517	0.81196914
H	-0.76680512	2.37708027	-0.94674509
H	-2.06637015	2.87766121	0.11428263
H	1.25968847	1.72245671	-0.41769838
C	4.10783032	0.81228628	-0.06425714
H	4.64103059	0.63845159	0.86593644
H	4.69428358	0.46249713	-0.91030407
H	4.00887623	1.88824428	-0.17523856
H	-1.58763465	-1.98356306	0.80738584

Time = 88.50 fs
E(CASSCF/6-31G(d)) = -479.5618438

C	-0.93704143	-0.22368652	-0.24582389
C	0.49080823	-0.33947761	-0.08242755
C	1.19855739	-1.55944489	0.25149042
C	1.52738803	0.71024252	-0.19894522

C	2.76755002	0.13550752	-0.02816846
C	2.56823343	-1.31049408	0.21830723
N	-1.65965201	0.80715013	0.11143294
C	-3.10021488	0.55526014	0.16208862
H	3.35239204	-2.05620659	0.26362160
C	-1.80627252	-1.47703706	-0.13288200
C	-3.24847926	-0.92754172	-0.22779905
H	-3.62746416	-1.00881983	-1.23938089
H	-3.93624147	-1.45088634	0.42552937
H	-3.49624578	0.70958846	1.16030330
H	-3.66759939	1.19263862	-0.50472387
H	-1.59159981	-2.18644584	-0.92647032
H	0.72092023	-2.52209993	0.35719862
C	-1.22378241	2.20788205	0.02046556
H	-0.52995172	2.43276921	0.81114501
H	-0.76897103	2.37647718	-0.94768541
H	-2.06740934	2.87447268	0.12063446
H	1.25589321	1.72262459	-0.42264520
C	4.10689387	0.81191185	-0.06454942
H	4.63888478	0.63921491	0.86579555
H	4.69064724	0.46223351	-0.91163253
H	4.01049377	1.88897761	-0.17647534
H	-1.58379856	-1.98324068	0.80395473

Time = 89.00 fs
E(CASSCF/6-31G(d)) = -479.5627378

C	-0.94217035	-0.22276332	-0.24544622
C	0.49497659	-0.33904165	-0.08264678
C	1.19762105	-1.55765224	0.25323245
C	1.52580774	0.70924653	-0.20146946
C	2.76736124	0.13422118	-0.02784938
C	2.56860490	-1.30904124	0.22066926
N	-1.65855533	0.80486844	0.10979537
C	-3.10050600	0.55426557	0.16341601
H	3.35153135	-2.05955602	0.26837525
C	-1.80525637	-1.47644199	-0.13664080
C	-3.24733669	-0.92663809	-0.22693968
H	-3.62890626	-1.00615598	-1.23806540
H	-3.93658496	-1.44837155	0.42674241
H	-3.48936776	0.70511042	1.16281864
H	-3.66914786	1.19029200	-0.50115219
H	-1.58856133	-2.18652273	-0.92977343
H	0.71105294	-2.51795121	0.35914023
C	-1.22232936	2.20886013	0.02168624
H	-0.52382299	2.42661810	0.81026956
H	-0.77101207	2.37602296	-0.94838652

H	-2.06890376	2.87076754	0.12706008
H	1.25361200	1.72298460	-0.42759733
C	4.10589102	0.81157632	-0.06485034
H	4.63740141	0.63976264	0.86533597
H	4.68759390	0.46179263	-0.91256753
H	4.01191735	1.88968562	-0.17772481
H	-1.58021398	-1.98261178	0.80076520

Time = 89.50 fs
E(CASSCF/6-31G(d)) = -479.5638493

C	-0.94756191	-0.22236164	-0.24515788
C	0.49994506	-0.33850591	-0.08278566
C	1.19635418	-1.55578394	0.25493875
C	1.52367868	0.70809933	-0.20407342
C	2.76731421	0.13263312	-0.02746107
C	2.56913899	-1.30737067	0.22302006
N	-1.65768658	0.80304033	0.10820337
C	-3.10085497	0.55308353	0.16480461
H	3.35001667	-2.06205318	0.27315601
C	-1.80424094	-1.47574777	-0.14055743
C	-3.24613042	-0.92575715	-0.22605804
H	-3.63014598	-1.00365092	-1.23675829
H	-3.93686801	-1.44577119	0.42786711
H	-3.48192785	0.70134190	1.16569220
H	-3.66993193	1.18862971	-0.49779548
H	-1.58531276	-2.18640603	-0.93286795
H	0.70188886	-2.51336753	0.36115579
C	-1.22087618	2.20974807	0.02292829
H	-0.51730204	2.42058122	0.80933064
H	-0.77294857	2.37578397	-0.94883816
H	-2.07078228	2.86660340	0.13356373
H	1.25289887	1.72336899	-0.43251976
C	4.10482140	0.81128663	-0.06516228
H	4.63661840	0.64007424	0.86457975
H	4.68518010	0.46115139	-0.91312575
H	4.01310642	1.89029337	-0.17897483
H	-1.57691842	-1.98167365	0.79776577

Time = 90.00 fs
E(CASSCF/6-31G(d)) = -479.5651159

C	-0.95310646	-0.22251532	-0.24498586
C	0.50562363	-0.33787107	-0.08285411
C	1.19476628	-1.55384970	0.25661296
C	1.52103877	0.70681510	-0.20675548
C	2.76739702	0.13077504	-0.02700875

C	2.56982729	-1.30551784	0.22536576
N	-1.65707924	0.80171115	0.10667615
C	-3.10123966	0.55171602	0.16625475
H	3.34780071	-2.06357294	0.27794299
C	-1.80323329	-1.47496404	-0.14461241
C	-3.24487531	-0.92490944	-0.22516293
H	-3.63117408	-1.00131763	-1.23544385
H	-3.93704712	-1.44312481	0.42889051
H	-3.47404490	0.69832250	1.16901823
H	-3.67002203	1.18771994	-0.49468233
H	-1.58188894	-2.18606002	-0.93576669
H	0.69356965	-2.50829565	0.36324849
C	-1.21942556	2.21052145	0.02419154
H	-0.51046089	2.41472303	0.80830402
H	-0.77480097	2.37582531	-0.94904178
H	-2.07295762	2.86204258	0.14014952
H	1.25376992	1.72363308	-0.43738448
C	4.10368691	0.81104746	-0.06548728
H	4.63655519	0.64013806	0.86354597
H	4.68344510	0.46029561	-0.91332513
H	4.01402178	1.89073336	-0.18021403
H	-1.57394969	-1.98042604	0.79489497

Time = 90.50 fs
E(CASSCF/6-31G(d)) = -479.5665096

C	-0.95869302	-0.22324715	-0.24495509
C	0.51190960	-0.33714036	-0.08286326
C	1.19287632	-1.55185554	0.25825879
C	1.51793900	0.70540703	-0.20951228
C	2.76759412	0.12868296	-0.02649901
C	2.57065336	-1.30351651	0.22771299
N	-1.65676194	0.80091602	0.10523067
C	-3.10163875	0.55017117	0.16776732
H	3.34490417	-2.06405650	0.28271939
C	-1.80224147	-1.47410147	-0.14878300
C	-3.24358629	-0.92410500	-0.22426396
H	-3.63198538	-0.99916484	-1.23410850
H	-3.93708456	-1.44047914	0.42980640
H	-3.46584113	0.69607527	1.17286046
H	-3.66949208	1.18760107	-0.49182011
H	-1.57832417	-2.18546665	-0.93849818
H	0.68619237	-2.50273621	0.36542579
C	-1.21798034	2.21115849	0.02547736
H	-0.50339031	2.40910234	0.80715683
H	-0.77658913	2.37620889	-0.94900925
H	-2.07532804	2.85715155	0.14682060

H	1.25619971	1.72366667	-0.44217262
C	4.10249219	0.81086076	-0.06582699
H	4.63721243	0.63995190	0.86224936
H	4.68241011	0.45922139	-0.91318307
H	4.01462689	1.89095197	-0.18143256
H	-1.57134395	-1.97887238	0.79208632

Time = 91.00 fs
E(CASSCF/6-31G(d)) = -479.5679539

C	-0.96421289	-0.22456662	-0.24508757
C	0.51868991	-0.33631986	-0.08282500
C	1.19071265	-1.54980390	0.25988010
C	1.51444305	0.70388738	-0.21233894
C	2.76788669	0.12639624	-0.02593944
C	2.57159288	-1.30139761	0.23006855
N	-1.65675689	0.80067782	0.10388086
C	-3.10203286	0.54846353	0.16934430
H	3.34141589	-2.06351406	0.28747369
C	-1.80127452	-1.47317177	-0.15304404
C	-3.24227800	-0.92335329	-0.22337118
H	-3.63258011	-0.99719715	-1.23274308
H	-3.93694976	-1.43788791	0.43061579
H	-3.45743778	0.69460531	1.17724334
H	-3.66841541	1.18827814	-0.48919178
H	-1.57465207	-2.18462588	-0.94110609
H	0.67980633	-2.49674585	0.36769940
C	-1.21654366	2.21164051	0.02678841
H	-0.49619398	2.40377223	0.80585192
H	-0.77833229	2.37699217	-0.94876257
H	-2.07778092	2.85200169	0.15357931
H	1.26012164	1.72339981	-0.44687579
C	4.10124461	0.81072540	-0.06618263
H	4.63857091	0.63952325	0.86069880
H	4.68207650	0.45793552	-0.91271472
H	4.01488819	1.89091289	-0.18262261
H	-1.56913386	-1.97702330	0.78927424

Time = 91.50 fs
E(CASSCF/6-31G(d)) = -479.5693744

C	-0.96956265	-0.22646815	-0.24540173
C	0.52584319	-0.33541895	-0.08275177
C	1.18831238	-1.54769383	0.26148087
C	1.51062604	0.70226785	-0.21522911
C	2.76825350	0.12395675	-0.02533859
C	2.57261449	-1.29918881	0.23243924

N	-1.65707867	0.80100591	0.10263702
C	-3.10240517	0.54661414	0.17098902
H	3.33748851	-2.06202377	0.29220046
C	-1.80034261	-1.47218776	-0.15736874
C	-3.24096448	-0.92266301	-0.22249494
H	-3.63296419	-0.99541500	-1.23134402
H	-3.93661982	-1.43541076	0.43132688
H	-3.44895185	0.69389903	1.18214625
H	-3.66686126	1.18972124	-0.48675496
H	-1.57090609	-2.18355390	-0.94364628
H	0.67441175	-2.49043687	0.37008566
C	-1.21511908	2.21195199	0.02812804
H	-0.48898326	2.39877952	0.80435262
H	-0.78004944	2.37822613	-0.94833101
H	-2.08019717	2.84666917	0.16042666
H	1.26542946	1.72280694	-0.45149726
C	4.09995413	0.81063710	-0.06655502
H	4.64059199	0.63886895	0.85889766
H	4.68242577	0.45645558	-0.91193254
H	4.01477595	1.89060055	-0.18377880
H	-1.56734709	-1.97489847	0.78639892

Time = 92.00 fs
E(CASSCF/6-31G(d)) = -479.5706537

C	-0.97464578	-0.22892979	-0.24591167
C	0.53324116	-0.33445043	-0.08265637
C	1.18572054	-1.54552192	0.26306538
C	1.50657311	0.70056048	-0.21817555
C	2.76867183	0.12140821	-0.02470570
C	2.57368080	-1.29691466	0.23483177
N	-1.65773355	0.80189508	0.10150531
C	-3.10274188	0.54465027	0.17270633
H	3.33332922	-2.05972387	0.29690030
C	-1.79945699	-1.47116352	-0.16172969
C	-3.23965901	-0.92204211	-0.22164559
H	-3.63314987	-0.99381494	-1.22991460
H	-3.93607933	-1.43311219	0.43195421
H	-3.44049411	0.69392359	1.18750300
H	-3.66489457	1.19186600	-0.48444344
H	-1.56711987	-2.18228088	-0.94618351
H	0.66996266	-2.48396936	0.37260436
C	-1.21371074	2.21208072	0.02949971
H	-0.48186908	2.39416475	0.80262828
H	-0.78175982	2.37995461	-0.94774952
H	-2.08245614	2.84123651	0.16736325
H	1.27198118	1.72190363	-0.45605103

C	4.09863299	0.81058838	-0.06694456
H	4.64321853	0.63801445	0.85684475
H	4.68341994	0.45480893	-0.91084669
H	4.01426493	1.89002041	-0.18489841
H	-1.56600486	-1.97252900	0.78341069

Time = 92.50 fs
E(CASSCF/6-31G(d)) = -479.5717018

C	-0.97937595	-0.23191243	-0.24662684
C	0.54075121	-0.33343044	-0.08255155
C	1.18298834	-1.54328346	0.26463821
C	1.50237740	0.69877849	-0.22117032
C	2.76911836	0.11879541	-0.02405066
C	2.57475041	-1.29459702	0.23725280
N	-1.65871880	0.80332494	0.10048747
C	-3.10303201	0.54260473	0.17450257
H	3.32918630	-2.05680387	0.30157990
C	-1.79862985	-1.47011434	-0.16609982
C	-3.23837388	-0.92149763	-0.22083324
H	-3.63315597	-0.99238989	-1.22846579
H	-3.93532032	-1.43105991	0.43251806
H	-3.43216855	0.69462786	1.19320504
H	-3.66257552	1.19461727	-0.48217130
H	-1.56332770	-2.18084740	-0.94878689
H	0.66637390	-2.47754101	0.37527829
C	-1.21232338	2.21201770	0.03090636
H	-0.47495648	2.38996178	0.80065898
H	-0.78348352	2.38221231	-0.94705503
H	-2.08444029	2.83579222	0.17438883
H	1.27960616	1.72074229	-0.46056073
C	4.09729551	0.81056900	-0.06735131
H	4.64637704	0.63699337	0.85453625
H	4.68500318	0.45303233	-0.90946607
H	4.01333482	1.88919839	-0.18598139
H	-1.56512030	-1.96995810	0.78027415

Time = 93.00 fs
E(CASSCF/6-31G(d)) = -479.5724196

C	-0.98369063	-0.23536897	-0.24755411
C	0.54825930	-0.33237536	-0.08244965
C	1.18016381	-1.54096756	0.26620879
C	1.49812583	0.69693223	-0.22421375
C	2.76957145	0.11615590	-0.02338191
C	2.57578280	-1.29224928	0.23971562
N	-1.66002585	0.80526481	0.09957809

C	-3.10326808	0.54050920	0.17639044
H	3.32531925	-2.05347900	0.30626435
C	-1.79787199	-1.46905394	-0.17046542
C	-3.23711685	-0.92103431	-0.22006534
H	-3.63300744	-0.99112566	-1.22701163
H	-3.93433843	-1.42931743	0.43304412
H	-3.42404949	0.69594647	1.19912564
H	-3.65995456	1.19786249	-0.47983210
H	-1.55955478	-2.17929633	-0.95153206
H	0.66352110	-2.47135466	0.37813947
C	-1.21095856	2.21175676	0.03235384
H	-0.46831957	2.38618614	0.79843375
H	-0.78524717	2.38503066	-0.94628113
H	-2.08604536	2.83041596	0.18152259
H	1.28813462	1.71940034	-0.46507013
C	4.09595370	0.81056639	-0.06777621
H	4.64998989	0.63584327	0.85196193
H	4.68710945	0.45116512	-0.90779639
H	4.01196762	1.88817600	-0.18703318
H	-1.56469578	-1.96723401	0.77696102

Time = 93.50 fs
E(CASSCF/6-31G(d)) = -479.5727200

C	-0.98751324	-0.23921844	-0.24869088
C	0.55560899	-0.33131027	-0.08236331
C	1.17731331	-1.53857768	0.26777855
C	1.49393248	0.69504524	-0.22728969
C	2.77000800	0.11354121	-0.02271173
C	2.57673159	-1.28989688	0.24221973
N	-1.66163113	0.80766042	0.09877281
C	-3.10344363	0.53841043	0.17837321
H	3.32201137	-2.04999482	0.31095623
C	-1.79719928	-1.46800357	-0.17479048
C	-3.23590177	-0.92065881	-0.21935307
H	-3.63273539	-0.99001251	-1.22558049
H	-3.93313861	-1.42795766	0.43355666
H	-3.41625177	0.69779308	1.20508260
H	-3.65709645	1.20145336	-0.47732539
H	-1.55584992	-2.17768099	-0.95447431
H	0.66127878	-2.46565224	0.38120392
C	-1.20962603	2.21129619	0.03383849
H	-0.46205262	2.38286815	0.79597064
H	-0.78706983	2.38841580	-0.94546163
H	-2.08716975	2.82522419	0.18874463
H	1.29733613	1.71797904	-0.46960281
C	4.09462793	0.81056625	-0.06821768

H	4.65394967	0.63461289	0.84912851
H	4.68964960	0.44926211	-0.90585505
H	4.01015826	1.88701418	-0.18805539
H	-1.56472800	-1.96443188	0.77347781

Time = 94.00 fs
E(CASSCF/6-31G(d)) = -479.5725682

C	-0.99079166	-0.24337496	-0.25003227
C	0.56266680	-0.33025949	-0.08230446
C	1.17449591	-1.53611450	0.26935418
C	1.48990026	0.69313978	-0.23039116
C	2.77040803	0.11099405	-0.02205050
C	2.57755610	-1.28755921	0.24477134
N	-1.66350638	0.81044958	0.09806070
C	-3.10355490	0.53635142	0.18045956
H	3.31951108	-2.04657758	0.31567285
C	-1.79662568	-1.46698311	-0.17905384
C	-3.23473803	-0.92037548	-0.21870458
H	-3.63237567	-0.98903424	-1.22420123
H	-3.93172614	-1.42704435	0.43408091
H	-3.40886264	0.70007189	1.21090055
H	-3.65406231	1.20523940	-0.47454556
H	-1.55225449	-2.17604827	-0.95766566
H	0.65950794	-2.46064065	0.38449260
C	-1.20833214	2.21063730	0.03535879
H	-0.45620997	2.38001874	0.79330042
H	-0.78897794	2.39236871	-0.94462144
H	-2.08773350	2.82032342	0.19605537
H	1.30699601	1.71658066	-0.47419660
C	4.09333501	0.81055335	-0.06867522
H	4.65815152	0.63335161	0.84604267
H	4.69253066	0.44737620	-0.90366114
H	4.00790536	1.88578032	-0.18905422
H	-1.56520234	-1.96162993	0.76983844

Time = 94.50 fs
E(CASSCF/6-31G(d)) = -479.5719700

C	-0.99348515	-0.24774145	-0.25156917
C	0.56930326	-0.32924866	-0.08228449
C	1.17176907	-1.53358394	0.27094340
C	1.48613046	0.69124225	-0.23351241
C	2.77075385	0.10855504	-0.02140838
C	2.57822076	-1.28525757	0.24737663
N	-1.66561658	0.81355949	0.09742754
C	-3.10359972	0.53437563	0.18265786

H	3.31801919	-2.04341797	0.32043053
C	-1.79616506	-1.46601385	-0.18323754
C	-3.23363416	-0.92018774	-0.21812702
H	-3.63196785	-0.98817235	-1.22290524
H	-3.93010713	-1.42663479	0.43463908
H	-3.40196563	0.70267809	1.21640899
H	-3.65091958	1.20906817	-0.47139627
H	-1.54881348	-2.17443884	-0.96114353
H	0.65807891	-2.45648808	0.38802103
C	-1.20708350	2.20978508	0.03691122
H	-0.45082269	2.37763990	0.79047188
H	-0.79100043	2.39687738	-0.94377678
H	-2.08767609	2.81582365	0.20345606
H	1.31689918	1.71529938	-0.47888788
C	4.09209126	0.81051240	-0.06914826
H	4.66248738	0.63211059	0.84272077
H	4.69565433	0.44556090	-0.90124205
H	4.00521520	1.88454458	-0.19003677
H	-1.56609511	-1.95891462	0.76607133

Time = 95.00 fs
E(CASSCF/6-31G(d)) = -479.5709778

C	-0.99556440	-0.25221294	-0.25328903
C	0.57539599	-0.32830336	-0.08231428
C	1.16918628	-1.53099868	0.27255504
C	1.48271944	0.68938299	-0.23664908
C	2.77103070	0.10626164	-0.02079504
C	2.57869752	-1.28301603	0.25004183
N	-1.66792113	0.81690932	0.09685652
C	-3.10357693	0.53252540	0.18497547
H	3.31766754	-2.04065153	0.32524283
C	-1.79583067	-1.46511815	-0.18732707
C	-3.23259801	-0.92009808	-0.21762647
H	-3.63155414	-0.98740638	-1.22172392
H	-3.92828688	-1.42677753	0.43524842
H	-3.39564081	0.70550153	1.22145604
H	-3.64774432	1.21279380	-0.46779730
H	-1.54557564	-2.17288457	-0.96492768
H	0.65688544	-2.45330511	0.39179746
C	-1.20588681	2.20874808	0.03849036
H	-0.44589868	2.37572386	0.78754979
H	-0.79316843	2.40191673	-0.94293385
H	-2.08695785	2.81183539	0.21094915
H	1.32683836	1.71421008	-0.48370866
C	4.09091194	0.81042903	-0.06963634
H	4.66685015	0.63094152	0.83919159

H	4.69892115	0.44386846	-0.89863596
H	4.00210146	1.88337350	-0.19101034
H	-1.56737424	-1.95637781	0.76221670

Time = 95.50 fs
E(CASSCF/6-31G(d)) = -479.5696761

C	-0.99701075	-0.25668022	-0.25517641
C	0.58083196	-0.32744792	-0.08240415
C	1.16679533	-1.52837890	0.27419910
C	1.47975636	0.68759560	-0.23979844
C	2.77122687	0.10414760	-0.02021950
C	2.57896730	-1.28086203	0.25277327
N	-1.67037526	0.82041286	0.09632899
C	-3.10348611	0.53084037	0.18741820
H	3.31850452	-2.03834320	0.33011830
C	-1.79563465	-1.46431891	-0.19131153
C	-3.23163685	-0.92010804	-0.21720791
H	-3.63117800	-0.98671437	-1.22068612
H	-3.92626850	-1.42751028	0.43591939
H	-3.38996460	0.70843054	1.22592022
H	-3.64462273	1.21628468	-0.46369048
H	-1.54259321	-2.17140691	-0.96901895
H	0.65585458	-2.45113104	0.39582103
C	-1.20474867	2.20753847	0.04008922
H	-0.44142390	2.37425246	0.78461126
H	-0.79551447	2.40744872	-0.94208883
H	-2.08556012	2.80846648	0.21853821
H	1.33661876	1.71335988	-0.48868414
C	4.08981062	0.81029065	-0.07013917
H	4.67113703	0.62989496	0.83549792
H	4.70223404	0.44234760	-0.89589313
H	3.99858550	1.88232358	-0.19198171
H	-1.56900044	-1.95411353	0.75832268

Time = 96.00 fs
E(CASSCF/6-31G(d)) = -479.5681748

C	-0.99781527	-0.26103338	-0.25721364
C	0.58550947	-0.32670415	-0.08256388
C	1.16463722	-1.52575229	0.27588665
C	1.47732118	0.68591565	-0.24295932
C	2.77133374	0.10224251	-0.01968997
C	2.57902063	-1.27882634	0.25557730
N	-1.67293140	0.82398122	0.09582522
C	-3.10332737	0.52935609	0.18998984
H	3.32048957	-2.03648073	0.33505887

C	-1.79558748	-1.46363899	-0.19518334
C	-3.23075751	-0.92021818	-0.21687519
H	-3.63088257	-0.98607323	-1.21981567
H	-3.92405203	-1.42885822	0.43665420
H	-3.38500894	0.71135559	1.22971980
H	-3.64165144	1.21942990	-0.45904412
H	-1.53992136	-2.17001613	-0.97339957
H	0.65495179	-2.44992789	0.40008066
C	-1.20367538	2.20617214	0.04169971
H	-0.43736534	2.37319662	0.78174096
H	-0.79807115	2.41342253	-0.94122873
H	-2.08348432	2.80581832	0.22622752
H	1.34606095	1.71276353	-0.49383131
C	4.08879874	0.81008717	-0.07065663
H	4.67525263	0.62901857	0.83169704
H	4.70550149	0.44104144	-0.89307630
H	3.99469681	1.88143586	-0.19295643
H	-1.57092865	-1.95221415	0.75444051

Time = 96.50 fs
E(CASSCF/6-31G(d)) = -479.5665978

C	-0.99797821	-0.26516542	-0.25938135
C	0.58934029	-0.32609000	-0.08280277
C	1.16274538	-1.52315333	0.27762956
C	1.47548262	0.68437899	-0.24613192
C	2.77134573	0.10057120	-0.01921369
C	2.57885755	-1.27694200	0.25846025
N	-1.67554051	0.82752545	0.09532512
C	-3.10310123	0.52810279	0.19269170
H	3.32349717	-2.03497835	0.34005964
C	-1.79569741	-1.46310033	-0.19893792
C	-3.22996641	-0.92042808	-0.21663105
H	-3.63070920	-0.98545920	-1.21912916
H	-3.92163409	-1.43083294	0.43744610
H	-3.38083994	0.71417274	1.23281912
H	-3.63893667	1.22214408	-0.45385560
H	-1.53761700	-2.16871199	-0.97803494
H	0.65418086	-2.44958383	0.40455537
C	-1.20267272	2.20466855	0.04331328
H	-0.43367520	2.37251586	0.77902549
H	-0.80086960	2.41977553	-0.94033341
H	-2.08074965	2.80398156	0.23402170
H	1.35500343	1.71240364	-0.49915877
C	4.08788524	0.80981157	-0.07118886
H	4.67911137	0.62835587	0.82786013
H	4.70864026	0.43998599	-0.89026025

H	3.99047287	1.88073168	-0.19393828
H	-1.57310959	-1.95076636	0.75061926

Time = 97.00 fs
E(CASSCF/6-31G(d)) = -479.5650702

C	-0.99750856	-0.26897551	-0.26165909
C	0.59225182	-0.32561832	-0.08312961
C	1.16114531	-1.52062157	0.27944020
C	1.47429627	0.68301956	-0.24931754
C	2.77126029	0.09915327	-0.01879680
C	2.57848657	-1.27524259	0.26142817
N	-1.67815325	0.83095899	0.09480891
C	-3.10280852	0.52710435	0.19552242
H	3.32732991	-2.03369057	0.34510973
C	-1.79596993	-1.46272306	-0.20257322
C	-3.22926965	-0.92073628	-0.21647723
H	-3.63069631	-0.98484825	-1.21863429
H	-3.91900823	-1.43343198	0.43827937
H	-3.37751605	0.71678665	1.23523174
H	-3.63659215	1.22437048	-0.44815184
H	-1.53573715	-2.16748574	-0.98287681
H	0.65357942	-2.44992451	0.40921559
C	-1.20174580	2.20305063	0.04492174
H	-0.43029607	2.37215817	0.77654717
H	-0.80393780	2.42643459	-0.93937805
H	-2.07738996	2.80303145	0.24192506
H	1.36330472	1.71223454	-0.50466730
C	4.08707639	0.80946032	-0.07173620
H	4.68263945	0.62794518	0.82407065
H	4.71157754	0.43920913	-0.88753041
H	3.98595871	1.88021048	-0.19492896
H	-1.57549147	-1.94984745	0.74690085

Time = 97.50 fs
E(CASSCF/6-31G(d)) = -479.5637145

C	-0.99642358	-0.27237189	-0.26402575
C	0.59418879	-0.32529586	-0.08355274
C	1.15985483	-1.51819936	0.28133087
C	1.47380320	0.68186705	-0.25251822
C	2.77107768	0.09800266	-0.01844418
C	2.57792313	-1.27375996	0.26448659
N	-1.68072108	0.83419989	0.09425762
C	-3.10245055	0.52637761	0.19847777
H	3.33173846	-2.03243243	0.35019392
C	-1.79640727	-1.46252455	-0.20608907

C	-3.22867294	-0.92114022	-0.21641452
H	-3.63087829	-0.98421654	-1.21832875
H	-3.91616603	-1.43663902	0.43913019
H	-3.37508582	0.71911313	1.23702000
H	-3.63473582	1.22608209	-0.44198789
H	-1.53433720	-2.16632281	-0.98786752
H	0.65321023	-2.45073124	0.41402559
C	-1.20089895	2.20134448	0.04651798
H	-0.42716665	2.37205995	0.77437800
H	-0.80729879	2.43331772	-0.93833597
H	-2.07344984	2.80302330	0.24994076
H	1.37084443	1.71218973	-0.51035128
C	4.08637570	0.80903343	-0.07229922
H	4.68577607	0.62781890	0.82042155
H	4.71425239	0.43873007	-0.88498041
H	3.98120630	1.87984986	-0.19592800
H	-1.57802171	-1.94952188	0.74331558

Time = 98.00 fs
E(CASSCF/6-31G(d)) = -479.5626451

C	-0.99474854	-0.27527417	-0.26646005
C	0.59511465	-0.32512266	-0.08407998
C	1.15888447	-1.51592914	0.28331335
C	1.47402885	0.68094477	-0.25573623
C	2.77080081	0.09712737	-0.01815935
C	2.57718754	-1.27252190	0.26764018
N	-1.68319723	0.83717264	0.09365361
C	-3.10202918	0.52593204	0.20155067
H	3.33644515	-2.03100318	0.35529500
C	-1.79700807	-1.46251848	-0.20948660
C	-3.22818153	-0.92163624	-0.21644284
H	-3.63128476	-0.98354088	-1.21820000
H	-3.91309865	-1.44042460	0.43996799
H	-3.37358556	0.72108081	1.23829136
H	-3.63348555	1.22728067	-0.43544369
H	-1.53346884	-2.16520605	-0.99294482
H	0.65315006	-2.45176349	0.41894656
C	-1.20013558	2.19957903	0.04809680
H	-0.42422725	2.37214652	0.77257410
H	-0.81096900	2.44033605	-0.93718185
H	-2.06898065	2.80398835	0.25806995
H	1.37752403	1.71219137	-0.51620076
C	4.08578401	0.80853436	-0.07287862
H	4.68847382	0.62800290	0.81701147
H	4.71661639	0.43855922	-0.88270871
H	3.97627359	1.87960778	-0.19693294

H	-1.58064845	-1.94983846	0.73987849

Time = 98.50 fs
E(CASSCF/6-31G(d)) = -479.5619619

C	-0.99251636	-0.27761513	-0.26894087
C	0.59501246	-0.32509186	-0.08471853
C	1.15823816	-1.51385059	0.28539820
C	1.47498241	0.68026782	-0.25897378
C	2.77043505	0.09652940	-0.01794443
C	2.57630293	-1.27154990	0.27089247
N	-1.68553749	0.83980977	0.09298092
C	-3.10154703	0.52576961	0.20473138
H	3.34116822	-2.02920992	0.36039626
C	-1.79776711	-1.46271414	-0.21276767
C	-3.22780008	-0.92221952	-0.21656135
H	-3.63194002	-0.98279917	-1.21822582
H	-3.90979874	-1.44474759	0.44075736
H	-3.37303687	0.72263211	1.23919141
H	-3.63295400	1.22799374	-0.42861908
H	-1.53317810	-2.16411885	-0.99804700
H	0.65347782	-2.45278254	0.42394006
C	-1.19945818	2.19778553	0.04965551
H	-0.42142443	2.37233296	0.77117146
H	-0.81495677	2.44739613	-0.93589500
H	-2.06403659	2.80593042	0.26631101
H	1.38326743	1.71216040	-0.52220373
C	4.08529975	0.80796959	-0.07347514
H	4.69069835	0.62851630	0.81394001
H	4.71863351	0.43869864	-0.88081445
H	3.97122341	1.87942676	-0.19793979
H	-1.58332196	-1.95082820	0.73658675

Time = 99.00 fs
E(CASSCF/6-31G(d)) = -479.5617445

C	-0.98976741	-0.27934198	-0.27144766
C	0.59388535	-0.32518998	-0.08547481
C	1.15791400	-1.51199804	0.28759431
C	1.47665680	0.67984184	-0.26223258
C	2.76998804	0.09620472	-0.01780007
C	2.57529325	-1.27085729	0.27424554
N	-1.68770090	0.84205299	0.09222563
C	-3.10100791	0.52588523	0.20800776
H	3.34564415	-2.02688860	0.36548403
C	-1.79867523	-1.46311573	-0.21593440
C	-3.22753245	-0.92288409	-0.21676848

H	-3.63286289	-0.98197086	-1.21837554
H	-3.90626289	-1.44955680	0.44146052
H	-3.37344413	0.72372342	1.23989367
H	-3.63324269	1.22826985	-0.42162727
H	-1.53350354	-2.16304832	-1.00311754
H	0.65426347	-2.45357382	0.42897155
C	-1.19886826	2.19599700	0.05119442
H	-0.41871495	2.37252558	0.77018300
H	-0.81926116	2.45440245	-0.93446160
H	-2.05867168	2.80882348	0.27465858
H	1.38802125	1.71202598	-0.52834823
C	4.08491925	0.80734807	-0.07408949
H	4.69242731	0.62937128	0.81130260
H	4.72027924	0.43914272	-0.87939268
H	3.96612190	1.87923952	-0.19894352
H	-1.58599571	-1.95250346	0.73341935

Time = 99.50 fs
E(CASSCF/6-31G(d)) = -479.5620474

C	-0.98654925	-0.28041702	-0.27396084
C	0.59175611	-0.32539765	-0.08635421
C	1.15790490	-1.51039833	0.28990834
C	1.47902919	0.67966263	-0.26551376
C	2.76946958	0.09614381	-0.01772560
C	2.57418164	-1.27044826	0.27769998
N	-1.68965051	0.84385433	0.09137605
C	-3.10041688	0.52626730	0.21136579
H	3.34964693	-2.02392114	0.37054987
C	-1.79971940	-1.46372203	-0.21898883
C	-3.22738153	-0.92362282	-0.21706205
H	-3.63406678	-0.98103739	-1.21861223
H	-3.90249364	-1.45479326	0.44203999
H	-3.37479170	0.72432425	1.24058699
H	-3.63443562	1.22817216	-0.41458712
H	-1.53447475	-2.16198743	-1.00810924
H	0.65555859	-2.45396568	0.43401362
C	-1.19836646	2.19424724	0.05271703
H	-0.41606756	2.37262370	0.76959767
H	-0.82387144	2.46126004	-0.93287723
H	-2.05293738	2.81261073	0.28310355
H	1.39175513	1.71173316	-0.53462389
C	4.08463723	0.80668049	-0.07472221
H	4.69364881	0.63057338	0.80918478
H	4.72153917	0.43987949	-0.87852912
H	3.96103678	1.87897545	-0.19993881
H	-1.58862728	-1.95485763	0.73033755

Time = 100.00 fs
E(CASSCF/6-31G(d)) = -479.5628675

C	-0.98291615	-0.28081776	-0.27646207
C	0.58866737	-0.32569075	-0.08736080
C	1.15819912	-1.50906893	0.29234442
C	1.48206140	0.67971611	-0.26881768
C	2.76889133	0.09633149	-0.01771877
C	2.57298889	-1.27031668	0.28125448
N	-1.69135357	0.84517652	0.09042302
C	-3.09978101	0.52689859	0.21479009
H	3.35300149	-2.02024649	0.37559197
C	-1.80088302	-1.46452637	-0.22193279
C	-3.22734907	-0.92442750	-0.21743918
H	-3.63555984	-0.97998260	-1.21889481
H	-3.89850166	-1.46039205	0.44246068
H	-3.37704222	0.72441605	1.24146081
H	-3.63659298	1.22777117	-0.40761499
H	-1.53611096	-2.16093718	-1.01298781
H	0.65738986	-2.45384317	0.43904806
C	-1.19795256	2.19257036	0.05423004
H	-0.41346366	2.37252247	0.76938118
H	-0.82876652	2.46787684	-0.93114792
H	-2.04688176	2.81720502	0.29163226
H	1.39446153	1.71124773	-0.54102378
C	4.08444732	0.80597849	-0.07537360
H	4.69435986	0.63212146	0.80765731
H	4.72240749	0.44089164	-0.87829575
H	3.95603571	1.87856797	-0.20092084
H	-1.59117858	-1.95786640	0.72728792

Time = 100.50 fs
E(CASSCF/6-31G(d)) = -479.5641629

C	-0.97892850	-0.28053679	-0.27893462
C	0.58468101	-0.32604174	-0.08849697
C	1.15878063	-1.50801693	0.29490384
C	1.48570090	0.67997922	-0.27214407
C	2.76826678	0.09674729	-0.01777584
C	2.57173250	-1.27044583	0.28490568
N	-1.69278194	0.84599340	0.08936006
C	-3.09910982	0.52775725	0.21826462
H	3.35559321	-2.01586669	0.38061634
C	-1.80214633	-1.46551671	-0.22476795
C	-3.22743552	-0.92528888	-0.21789635
H	-3.63734538	-0.97879324	-1.21918079

H	-3.89430719	-1.46628457	0.44269215
H	-3.38013479	0.72399020	1.24268991
H	-3.63974548	1.22713708	-0.40081651
H	-1.53842033	-2.15990718	-1.01773370
H	0.65975458	-2.45315702	0.44406776
C	-1.19762552	2.19099990	0.05574309
H	-0.41089562	2.37211607	0.76947869
H	-0.83391520	2.47416624	-0.92929094
H	-2.04054975	2.82249140	0.30022684
H	1.39615536	1.71055806	-0.54754512
C	4.08434247	0.80525392	-0.07604359
H	4.69456438	0.63400806	0.80677132
H	4.72288499	0.44215793	-0.87874617
H	3.95118435	1.87796086	-0.20188597
H	-1.59361610	-1.96148949	0.72420575

Time = 101.00 fs
E(CASSCF/6-31G(d)) = -479.5658690

C	-0.97465247	-0.27958101	-0.28136393
C	0.57987551	-0.32642131	-0.08976318
C	1.15962985	-1.50723891	0.29758506
C	1.48988324	0.68042186	-0.27549221
C	2.76761110	0.09736688	-0.01789193
C	2.57042606	-1.27080973	0.28864861
N	-1.69391274	0.84629035	0.08818346
C	-3.09841512	0.52881794	0.22177345
H	3.35737449	-2.01084982	0.38563740
C	-1.80348702	-1.46667598	-0.22749591
C	-3.22763987	-0.92619674	-0.21842935
H	-3.63942253	-0.97745917	-1.21942948
H	-3.88994110	-1.47240106	0.44271099
H	-3.38398292	0.72304576	1.24441988
H	-3.64388912	1.22633261	-0.39427901
H	-1.54139949	-2.15891506	-1.02234315
H	0.66261917	-2.45192673	0.44907778
C	-1.19738370	2.18956725	0.05726823
H	-0.40836395	2.37130072	0.76981917
H	-0.83927738	2.48004955	-0.92733489
H	-2.03398432	2.82833075	0.30886649
H	1.39687347	1.70967439	-0.55418827
C	4.08431563	0.80451817	-0.07673173
H	4.69427120	0.63621995	0.80655395
H	4.72297677	0.44365480	-0.87991116
H	3.94654439	1.87711352	-0.20283232
H	-1.59591064	-1.96567303	0.72101985

Time = 101.50 fs
E(CASSCF/6-31G(d)) = -479.5678528

C	-0.97015916	-0.27797083	-0.28373789
C	0.57434482	-0.32679984	-0.09115763
C	1.16072383	-1.50672126	0.30038375
C	1.49453365	0.68100870	-0.27886123
C	2.76694099	0.09816243	-0.01806107
C	2.56907891	-1.27137371	0.29247660
N	-1.69472853	0.84606415	0.08689237
C	-3.09771141	0.53005290	0.22530139
H	3.35836493	-2.00532667	0.39067796
C	-1.80488085	-1.46798268	-0.23011858
C	-3.22795967	-0.92714002	-0.21903326
H	-3.64178672	-0.97597363	-1.21960458
H	-3.88544539	-1.47867221	0.44250199
H	-3.38847433	0.72158766	1.24675457
H	-3.64898184	1.22540735	-0.38806562
H	-1.54503366	-2.15798549	-1.02682772
H	0.66592047	-2.45023779	0.45409523
C	-1.19722487	2.18830088	0.05881920
H	-0.40587363	2.36997861	0.77032052
H	-0.84480502	2.48545764	-0.92531832
H	-2.02722883	2.83456505	0.31752804
H	1.39667351	1.70862477	-0.56095639
C	4.08436008	0.80378162	-0.07743715
H	4.69349271	0.63873831	0.80700596
H	4.72269069	0.44535734	-0.88179618
H	3.94217196	1.87600515	-0.20376028
H	-1.59803691	-1.97035271	0.71765824

Time = 102.00 fs
E(CASSCF/6-31G(d)) = -479.5699621

C	-0.96552391	-0.27573888	-0.28604728
C	0.56819646	-0.32714866	-0.09267620
C	1.16203656	-1.50644122	0.30329311
C	1.49956882	0.68170123	-0.28225043
C	2.76627458	0.09910342	-0.01827632
C	2.56769636	-1.27209578	0.29638155
N	-1.69521743	0.84532273	0.08548880
C	-3.09701582	0.53143274	0.22883461
H	3.35864701	-1.99948416	0.39576843
C	-1.80630255	-1.46941166	-0.23263855
C	-3.22839089	-0.92810698	-0.21970242
H	-3.64443036	-0.97433325	-1.21967621
H	-3.88087270	-1.48503095	0.44205918

H	-3.39347139	0.71962514	1.24974754
H	-3.65494221	1.22439394	-0.38221118
H	-1.54929718	-2.15714774	-1.03121260
H	0.66956970	-2.44823450	0.45914821
C	-1.19714627	2.18722529	0.06041064
H	-0.40342989	2.36806114	0.77089488
H	-0.85044445	2.49033287	-0.92328733
H	-2.02032976	2.84102350	0.32618802
H	1.39563349	1.70745000	-0.56785370
C	4.08446979	0.80305327	-0.07815859
H	4.69224435	0.64153932	0.80810085
H	4.72203642	0.44724037	-0.88438017
H	3.93811576	1.87463779	-0.20467282
H	-1.59997297	-1.97545710	0.71405353

Time = 102.50 fs
E(CASSCF/6-31G(d)) = -479.5720263

C	-0.96082501	-0.27292883	-0.28828591
C	0.56154908	-0.32744144	-0.09431222
C	1.16353923	-1.50636845	0.30630426
C	1.50489945	0.68246013	-0.28565964
C	2.76563106	0.10015728	-0.01852989
C	2.56628010	-1.27292828	0.30035396
N	-1.69537333	0.84408470	0.08397767
C	-3.09634794	0.53292715	0.23236092
H	3.35835657	-1.99355175	0.40094563
C	-1.80772649	-1.47093501	-0.23505957
C	-3.22892819	-0.92908540	-0.22043031
H	-3.64734302	-0.97253823	-1.21962290
H	-3.87628555	-1.49141328	0.44138576
H	-3.39881323	0.71717113	1.25339813
H	-3.66165049	1.22330717	-0.37672142
H	-1.55415438	-2.15643315	-1.03553399
H	0.67345814	-2.44610689	0.46427402
C	-1.19714457	2.18636025	0.06205714
H	-0.40103425	2.36547263	0.77145452
H	-0.85613772	2.49462933	-0.92129292
H	-2.01333969	2.84752956	0.33482348
H	1.39384842	1.70619660	-0.57488446
C	4.08463950	0.80234048	-0.07889440
H	4.69054393	0.64459385	0.80978542
H	4.72102457	0.44927845	-0.88761533
H	3.93441677	1.87303650	-0.20557540
H	-1.60169950	-1.98091096	0.71014841

Time = 103.00 fs

E(CASSCF/6-31G(d)) = -479.5738790

C	-0.95614257	-0.26959396	-0.29045092
C	0.55453001	-0.32765509	-0.09605640
C	1.16520035	-1.50646729	0.30940674
C	1.51043250	0.68324734	-0.28908955
C	2.76503031	0.10129032	-0.01881337
C	2.56482926	-1.27382012	0.30438328
N	-1.69519594	0.84237877	0.08236680
C	-3.09572918	0.53450528	0.23586999
H	3.35766878	-1.98778564	0.40625073
C	-1.80912753	-1.47252295	-0.23738713
C	-3.22956501	-0.93006279	-0.22120957
H	-3.65051207	-0.97059238	-1.21943307
H	-3.87175467	-1.49775920	0.44049375
H	-3.40431968	0.71424217	1.25765341
H	-3.66895265	1.22214572	-0.37157478
H	-1.55956063	-2.15587148	-1.03983568
H	0.67746551	-2.44407275	0.46951621
C	-1.19721583	2.18572011	0.06377233
H	-0.39868060	2.36215320	0.77191687
H	-0.86182480	2.49831369	-0.91938773
H	-2.00631960	2.85390820	0.34341377
H	1.39142824	1.70490918	-0.58205115
C	4.08486485	0.80164900	-0.07964267
H	4.68841203	0.64786766	0.81198189
H	4.71966665	0.45144606	-0.89142865
H	3.93110755	1.87124811	-0.20647586
H	-1.60319880	-1.98663840	0.70590021

Time = 103.50 fs
E(CASSCF/6-31G(d)) = -479.5753834

C	-0.95155758	-0.26579580	-0.29254293
C	0.54727285	-0.32777004	-0.09789729
C	1.16698580	-1.50669938	0.31258921
C	1.51607303	0.68402764	-0.29254194
C	2.76449268	0.10246847	-0.01911788
C	2.56334184	-1.27471898	0.30845839
N	-1.69469084	0.84024298	0.08066673
C	-3.09518196	0.53613582	0.23935327
H	3.35677958	-1.98245110	0.41172702
C	-1.81048169	-1.47414479	-0.23962870
C	-3.23029373	-0.93102658	-0.22203195
H	-3.65392246	-0.96850262	-1.21910457
H	-3.86735644	-1.50401283	0.43940289
H	-3.40979588	0.71085882	1.26241433

H	-3.67666607	1.22089525	-0.36672549
H	-1.56546403	-2.15548719	-1.04416399
H	0.68147065	-2.44235673	0.47492072
C	-1.19735544	2.18531312	0.06556804
H	-0.39635275	2.35806118	0.77220887
H	-0.86744663	2.50136549	-0.91762174
H	-1.99934106	2.85999265	0.35194252
H	1.38849758	1.70362407	-0.58935223
C	4.08514217	0.80098316	-0.08040130
H	4.68587346	0.65132209	0.81459239
H	4.71797627	0.45371754	-0.89572572
H	3.92821110	1.86933873	-0.20738411
H	-1.60445462	-1.99256527	0.70128367

Time = 104.00 fs
E(CASSCF/6-31G(d)) = -479.5764337

C	-0.94715042	-0.26160270	-0.29456607
C	0.53991475	-0.32777068	-0.09982136
C	1.16885883	-1.50702632	0.31584012
C	1.52172653	0.68476993	-0.29601979
C	2.76403848	0.10365801	-0.01943430
C	2.56181646	-1.27557388	0.31256788
N	-1.69386949	0.83772385	0.07889066
C	-3.09472864	0.53778702	0.24280368
H	3.35588305	-1.97779989	0.41741668
C	-1.81176684	-1.47576980	-0.24179425
C	-3.23110603	-0.93196440	-0.22288836
H	-3.65755686	-0.96627883	-1.21864470
H	-3.86317029	-1.51012230	0.43813904
H	-3.41503889	0.70704675	1.26754679
H	-3.68458800	1.21953424	-0.36210986
H	-1.57180675	-2.15529593	-1.04856357
H	0.68536219	-2.44116710	0.48053160
C	-1.19755801	2.18514121	0.06745354
H	-0.39402289	2.35317487	0.77227021
H	-0.87294719	2.50377676	-0.91603850
H	-1.99248691	2.86563060	0.36039887
H	1.38519230	1.70236387	-0.59678106
C	4.08546831	0.80034622	-0.08116816
H	4.68295845	0.65491413	0.81750384
H	4.71597014	0.45606644	-0.90039481
H	3.92574142	1.86738887	-0.20831154
H	-1.60545138	-1.99862148	0.69629340

Time = 104.50 fs
E(CASSCF/6-31G(d)) = -479.5769735

C	-0.94299928	-0.25708853	-0.29652789
C	0.53259379	-0.32764543	-0.10181338
C	1.17078015	-1.50741227	0.31914834
C	1.52730080	0.68544800	-0.29952733
C	2.76368742	0.10482634	-0.01975349
C	2.56025420	-1.27633763	0.31670060
N	-1.69274917	0.83487538	0.07705428
C	-3.09439029	0.53942656	0.24621522
H	3.35514747	-1.97404807	0.42335739
C	-1.81296317	-1.47736807	-0.24389638
C	-3.23199315	-0.93286429	-0.22376890
H	-3.66139556	-0.96393355	-1.21806918
H	-3.85927575	-1.51603935	0.43673228
H	-3.41984502	0.70283771	1.27289529
H	-3.69250508	1.21804046	-0.35765333
H	-1.57852662	-2.15530150	-1.05307306
H	0.68904908	-2.44067320	0.48638659
C	-1.19781724	2.18519993	0.06943497
H	-0.39165142	2.34749354	0.77205536
H	-0.87827568	2.50555150	-0.91467150
H	-1.98585097	2.87068939	0.36877853
H	1.38165670	1.70113447	-0.60432513
C	4.08584026	0.79974078	-0.08194118
H	4.67970423	0.65859674	0.82059403
H	4.71366975	0.45846483	-0.90531225
H	3.92370406	1.86548764	-0.20927029
H	-1.60617382	-2.00474251	0.69094447

Time = 105.00 fs
E(CASSCF/6-31G(d)) = -479.5769943

C	-0.93917863	-0.25233141	-0.29843921
C	0.52544625	-0.32738663	-0.10385693
C	1.17270805	-1.50782609	0.32250378
C	1.53270785	0.68604071	-0.30306991
C	2.76345796	0.10594265	-0.02006653
C	2.55866046	-1.27696904	0.32084602
N	-1.69135283	0.83175795	0.07517561
C	-3.09418547	0.54102140	0.24958249
H	3.35469296	-1.97135601	0.42957896
C	-1.81405365	-1.47891121	-0.24595041
C	-3.23294629	-0.93371494	-0.22466293
H	-3.66541612	-0.96148146	-1.21740054
H	-3.85574939	-1.52171865	0.43521468
H	-3.42401745	0.69827044	1.27829762
H	-3.70020378	1.21639760	-0.35327806

H	-1.58555839	-2.15549349	-1.05772129
H	0.69246940	-2.44098608	0.49251295
C	-1.19812582	2.18547859	0.07151495
H	-0.38918815	2.34103769	0.77153429
H	-0.88338855	2.50670508	-0.91354097
H	-1.97953650	2.87505988	0.37708435
H	1.37804022	1.69992456	-0.61196606
C	4.08625489	0.79916936	-0.08271852
H	4.67615657	0.66231936	0.82373800
H	4.71110303	0.46088254	-0.91034835
H	3.92209691	1.86372595	-0.21027245
H	-1.60660685	-2.01087034	0.68527174

Time = 105.50 fs
E(CASSCF/6-31G(d)) = -479.5765292

C	-0.93575747	-0.24741246	-0.30031381
C	0.51860411	-0.32699039	-0.10593480
C	1.17459873	-1.50824295	0.32589782
C	1.53786549	0.68653174	-0.30665369
C	2.76336681	0.10697855	-0.02036492
C	2.55704628	-1.27743467	0.32499467
N	-1.68970892	0.82843718	0.07327475
C	-3.09412895	0.54253773	0.25290021
H	3.35457404	-1.96981335	0.43610038
C	-1.81502431	-1.48037293	-0.24797425
C	-3.23395689	-0.93450594	-0.22555924
H	-3.66959307	-0.95893905	-1.21666604
H	-3.85266179	-1.52711705	0.43361818
H	-3.42737368	0.69339098	1.28359935
H	-3.70748062	1.21460119	-0.34891013
H	-1.59283490	-2.15584611	-1.06252454
H	0.69559601	-2.44214521	0.49892425
C	-1.19847536	2.18596067	0.07369248
H	-0.38657507	2.33384867	0.77069196
H	-0.88825097	2.50726354	-0.91265168
H	-1.97365344	2.87865867	0.38532651
H	1.37449388	1.69870766	-0.61968030
C	4.08670854	0.79863488	-0.08349862
H	4.67237103	0.66602864	0.82681442
H	4.70830597	0.46328655	-0.91537347
H	3.92091140	1.86218951	-0.21132915
H	-1.60673571	-2.01695364	0.67932755

Time = 106.00 fs
E(CASSCF/6-31G(d)) = -479.5756420

C	-0.93279773	-0.24241473	-0.30216809
C	0.51219261	-0.32645623	-0.10802957
C	1.17640685	-1.50864493	0.32932358
C	1.54269874	0.68690888	-0.31028535
C	2.76342824	0.10790861	-0.02064084
C	2.55542907	-1.27770982	0.32913850
N	-1.68785109	0.82498271	0.07137365
C	-3.09423076	0.54394104	0.25616281
H	3.35477032	-1.96943130	0.44292785
C	-1.81586440	-1.48172947	-0.24998799
C	-3.23501701	-0.93522793	-0.22644618
H	-3.67389754	-0.95632408	-1.21589538
H	-3.85007497	-1.53219286	0.43197249
H	-3.42975224	0.68825260	1.28866659
H	-3.71415163	1.21266348	-0.34448598
H	-1.60028795	-2.15631819	-1.06748511
H	0.69843815	-2.44411304	0.50561844
C	-1.19885630	2.18662439	0.07596305
H	-0.38375012	2.32598757	0.76952666
H	-0.89283803	2.50726300	-0.91199172
H	-1.96831443	2.88142881	0.39352200
H	1.37116631	1.69744644	-0.62744064
C	4.08719678	0.79814121	-0.08428035
H	4.66841374	0.66966923	0.82971148
H	4.70532387	0.46564055	-0.92026383
H	3.92013374	1.86095201	-0.21244981
H	-1.60654633	-2.02294730	0.67317828

Time = 106.50 fs
E(CASSCF/6-31G(d)) = -479.5744183

C	-0.93035289	-0.23742211	-0.30402069
C	0.50632838	-0.32578683	-0.11012412
C	1.17808621	-1.50902083	0.33277588
C	1.54714081	0.68716303	-0.31397160
C	2.76365370	0.10871070	-0.02088731
C	2.55383254	-1.27777871	0.33327107
N	-1.68581772	0.82146691	0.06949578
C	-3.09449523	0.54519635	0.25936401
H	3.35518663	-1.97014419	0.45005396
C	-1.81656648	-1.48295983	-0.25201335
C	-3.23611951	-0.93587276	-0.22731192
H	-3.67829699	-0.95365507	-1.21511813
H	-3.84804002	-1.53690532	0.43030361
H	-3.43101818	0.68291502	1.29339632
H	-3.72006036	1.21061657	-0.33995707
H	-1.60784876	-2.15685483	-1.07259122

H	0.70103934	-2.44677847	0.51257758
C	-1.19925798	2.18744341	0.07831898
H	-0.38065221	2.31753359	0.76804745
H	-0.89713545	2.50674919	-0.91153255
H	-1.96363001	2.88333860	0.40169369
H	1.36820042	1.69609844	-0.63521776
C	4.08771417	0.79769353	-0.08506309
H	4.66436168	0.67318509	0.83233199
H	4.70221218	0.46790498	-0.92490693
H	3.91974596	1.86006929	-0.21364135
H	-1.60602592	-2.02881132	0.66689989

Time = 107.00 fs
E(CASSCF/6-31G(d)) = -479.5729522

C	-0.92846596	-0.23251820	-0.30589192
C	0.50111668	-0.32498775	-0.11220209
C	1.17959134	-1.50936516	0.33625125
C	1.55113449	0.68728716	-0.31771869
C	2.76405114	0.10936661	-0.02109844
C	2.55228535	-1.27763423	0.33738770
N	-1.68365147	0.81796348	0.06766582
C	-3.09492065	0.54626852	0.26249670
H	3.35566393	-1.97181677	0.45745814
C	-1.81712640	-1.48404602	-0.25407293
C	-3.23725833	-0.93643362	-0.22814469
H	-3.68275486	-0.95095094	-1.21436169
H	-3.84659561	-1.54121434	0.42863230
H	-3.43106795	0.67744316	1.29772429
H	-3.72508475	1.20851427	-0.33529402
H	-1.61544836	-2.15738942	-1.07781857
H	0.70346885	-2.44996813	0.51976937
C	-1.19966865	2.18838785	0.08075005
H	-0.37722648	2.30858215	0.76627087
H	-0.90113950	2.50577701	-0.91123059
H	-1.95970272	2.88437952	0.40986853
H	1.36572743	1.69462271	-0.64298346
C	4.08825423	0.79729856	-0.08584668
H	4.66030179	0.67652016	0.83459709
H	4.69903643	0.47003686	-0.92920546
H	3.91972771	1.85957493	-0.21490767
H	-1.60516381	-2.03450919	0.66057225

Time = 107.50 fs
E(CASSCF/6-31G(d)) = -479.5713494

C	-0.92716822	-0.22778534	-0.30780307

C	0.49664965	-0.32406712	-0.11424844
C	1.18087876	-1.50967662	0.33974760
C	1.55463269	0.68727530	-0.32153196
C	2.76462486	0.10986224	-0.02126959
C	2.55081939	-1.27727669	0.34148535
N	-1.68139853	0.81454605	0.06590925
C	-3.09549895	0.54712282	0.26555277
H	3.35599756	-1.97425990	0.46510802
C	-1.81754322	-1.48497308	-0.25618929
C	-3.23842854	-0.93690502	-0.22893303
H	-3.68723033	-0.94823058	-1.21364912
H	-3.84576668	-1.54508055	0.42697333
H	-3.42983222	0.67190529	1.30162923
H	-3.72914152	1.20643171	-0.33048814
H	-1.62301747	-2.15784709	-1.08313275
H	0.70581163	-2.45346443	0.52714974
C	-1.20007562	2.18942525	0.08324420
H	-0.37342991	2.29924275	0.76421767
H	-0.90485668	2.50441021	-0.91102969
H	-1.95662098	2.88456225	0.41807536
H	1.36386311	1.69298652	-0.65071276
C	4.08880935	0.79696452	-0.08663145
H	4.65632971	0.67961957	0.83644877
H	4.69587162	0.47199032	-0.93308063
H	3.92005730	1.85947788	-0.21624933
H	-1.60395225	-2.04000563	0.65427312

Time = 108.00 fs
E(CASSCF/6-31G(d)) = -479.5697240

C	-0.92647901	-0.22330350	-0.30977636
C	0.49300562	-0.32303530	-0.11624958
C	1.18190816	-1.50995586	0.34326364
C	1.55759878	0.68712126	-0.32541531
C	2.76537501	0.11018762	-0.02139748
C	2.54946759	-1.27671221	0.34556261
N	-1.67910771	0.81128681	0.06425197
C	-3.09621581	0.54772551	0.26852311
H	3.35596009	-1.97725202	0.47296323
C	-1.81781892	-1.48572891	-0.25838405
C	-3.23962643	-0.93728282	-0.22966606
H	-3.69167916	-0.94551224	-1.21299799
H	-3.84556477	-1.54846599	0.42533606
H	-3.42727679	0.66637163	1.30513382
H	-3.73218774	1.20446360	-0.32555080
H	-1.63048682	-2.15814981	-1.08849332
H	0.70815869	-2.45702606	0.53466702

C	-1.20046562	2.19052150	0.08578849
H	-0.36923673	2.28963618	0.76190945
H	-0.90830315	2.50272091	-0.91086478
H	-1.95445309	2.88391183	0.42634248
H	1.36270551	1.69117018	-0.65838580
C	4.08937114	0.79670112	-0.08741820
H	4.65254781	0.68243159	0.83785109
H	4.69280088	0.47371803	-0.93647434
H	3.92071189	1.85976207	-0.21766350
H	-1.60238760	-2.04526542	0.64807375

Time = 108.50 fs
E(CASSCF/6-31G(d)) = -479.5681889

C	-0.92640482	-0.21914927	-0.31183423
C	0.49024749	-0.32190471	-0.11819370
C	1.18264406	-1.51020345	0.34679850
C	1.56000704	0.68681799	-0.32937093
C	2.76629748	0.11033699	-0.02148030
C	2.54826142	-1.27595107	0.34961943
N	-1.67682952	0.80825503	0.06271984
C	-3.09705112	0.54804470	0.27139774
H	3.35532623	-1.98055936	0.48097858
C	-1.81795822	-1.48630415	-0.26067689
C	-3.24084946	-0.93756417	-0.23033376
H	-3.69605418	-0.94281325	-1.21241964
H	-3.84598834	-1.55133512	0.42372480
H	-3.42340282	0.66091207	1.30830261
H	-3.73422094	1.20272055	-0.32051168
H	-1.63778706	-2.15822072	-1.09385814
H	0.71059463	-2.46041025	0.54226621
C	-1.20082510	2.19164199	0.08836996
H	-0.36464233	2.27989222	0.75936616
H	-0.91150351	2.50078938	-0.91066650
H	-1.95324136	2.88246291	0.43469484
H	1.36233137	1.68917109	-0.66598988
C	4.08993076	0.79651922	-0.08820813
H	4.64906218	0.68490871	0.83878945
H	4.68991308	0.47517232	-0.93934957
H	3.92166832	1.86038808	-0.21914417
H	-1.60047116	-2.05025154	0.64203367

Time = 109.00 fs
E(CASSCF/6-31G(d)) = -479.5668476

C	-0.92693909	-0.21539449	-0.31399879
C	0.48842179	-0.32068949	-0.12007109

C	1.18305746	-1.51041791	0.35035133
C	1.56184287	0.68635682	-0.33339895
C	2.76738377	0.11030879	-0.02151774
C	2.54722810	-1.27500601	0.35365688
N	-1.67461482	0.80551541	0.06133810
C	-3.09797998	0.54805120	0.27416612
H	3.35389550	-1.98395575	0.48910705
C	-1.81796826	-1.48669202	-0.26308480
C	-3.24209613	-0.93774744	-0.23092720
H	-3.70030600	-0.94014935	-1.21191836
H	-3.84702352	-1.55365587	0.42213975
H	-3.41824469	0.65559382	1.31123621
H	-3.73527644	1.20132357	-0.31541435
H	-1.64484922	-2.15798891	-1.09918754
H	0.71318765	-2.46339290	0.54989276
C	-1.20114072	2.19275276	0.09097651
H	-0.35966580	2.27014692	0.75660389
H	-0.91448980	2.49870335	-0.91036503
H	-1.95299683	2.88025399	0.44315158
H	1.36279424	1.68700546	-0.67352012
C	4.09047922	0.79643010	-0.08900265
H	4.64597944	0.68700915	0.83926904
H	4.68730042	0.47630665	-0.94168992
H	3.92290329	1.86129775	-0.22068271
H	-1.59820995	-2.05492401	0.63619673

Time = 109.50 fs
E(CASSCF/6-31G(d)) = -479.5658367

C	-0.92806266	-0.21210534	-0.31629115
C	0.48755779	-0.31940538	-0.12187425
C	1.18312647	-1.51059460	0.35392077
C	1.56310244	0.68572813	-0.33749726
C	2.76862149	0.11010549	-0.02151114
C	2.54638929	-1.27389106	0.35767684
N	-1.67251411	0.80312711	0.06013089
C	-3.09897305	0.54771933	0.27681742
H	3.35151215	-1.98723949	0.49730422
C	-1.81785836	-1.48688799	-0.26562123
C	-3.24336573	-0.93783185	-0.23143876
H	-3.70438325	-0.93753474	-1.21149222
H	-3.84864491	-1.55540076	0.42057822
H	-3.41186727	0.65047935	1.31406254
H	-3.73542169	1.20039726	-0.31031204
H	-1.65160505	-2.15739200	-1.10444685
H	0.71597931	-2.46578656	0.55749784
C	-1.20139994	2.19382070	0.09359751

H	-0.35435117	2.26054042	0.75363600
H	-0.91729943	2.49655656	-0.90989553
H	-1.95369704	2.87732359	0.45172276
H	1.36412286	1.68470885	-0.68098049
C	4.09100800	0.79644502	-0.08980333
H	4.64340241	0.68869769	0.83931115
H	4.68505431	0.47707764	-0.94349695
H	3.92439364	1.86241837	-0.22226838
H	-1.59561785	-2.05923753	0.63058765

Time = 110.00 fs
E(CASSCF/6-31G(d)) = -479.5652229

C	-0.92974478	-0.20934067	-0.31873112
C	0.48766723	-0.31806949	-0.12359791
C	1.18283769	-1.51072410	0.35750485
C	1.56379296	0.68492075	-0.34166166
C	2.76999423	0.10973332	-0.02146335
C	2.54575892	-1.27261991	0.36168183
N	-1.67057557	0.80114163	0.05912054
C	-3.09999854	0.54702810	0.27934117
H	3.34807922	-1.99024729	0.50553101
C	-1.81763964	-1.48688952	-0.26829574
C	-3.24465812	-0.93781756	-0.23186228
H	-3.70823482	-0.93498141	-1.21113409
H	-3.85081801	-1.55654912	0.41903649
H	-3.40436110	0.64562411	1.31692548
H	-3.73475007	1.20006180	-0.30525970
H	-1.65798780	-2.15638086	-1.10961163
H	0.71898030	-2.46745310	0.56504047
C	-1.20159153	2.19481498	0.09622439
H	-0.34876472	2.25121364	0.75047230
H	-0.91997436	2.49444826	-0.90920211
H	-1.95528323	2.87370720	0.46040885
H	1.36632154	1.68233234	-0.68838254
C	4.09150960	0.79657426	-0.09061171
H	4.64142575	0.68994722	0.83894899
H	4.68326160	0.47744690	-0.94478739
H	3.92611583	1.86366964	-0.22388925
H	-1.59271491	-2.06314338	0.62521212

Time = 110.50 fs
E(CASSCF/6-31G(d)) = -479.5650660

C	-0.93194395	-0.20715063	-0.32133653
C	0.48874445	-0.31670036	-0.12523898
C	1.18218661	-1.51079161	0.36110057

C	1.56393196	0.68392282	-0.34588586
C	2.77148206	0.10920199	-0.02137870
C	2.54534224	-1.27120534	0.36567471
N	-1.66884402	0.79960171	0.05832704
C	-3.10102344	0.54596225	0.28172785
H	3.34356758	-1.99286374	0.51375612
C	-1.81732477	-1.48669599	-0.27111377
C	-3.24597356	-0.93770539	-0.23219313
H	-3.71181151	-0.93249900	-1.21083325
H	-3.85350112	-1.55708843	0.41751136
H	-3.39583819	0.64107464	1.31997076
H	-3.73337245	1.20042397	-0.30030696
H	-1.66393287	-2.15492237	-1.11466995
H	0.72216655	-2.46831186	0.57249019
C	-1.20170615	2.19570743	0.09885098
H	-0.34299176	2.24230641	0.74712184
H	-0.92255910	2.49248167	-0.90824141
H	-1.95766132	2.86943581	0.46919879
H	1.36937096	1.67993759	-0.69574434
C	4.09197819	0.79682647	-0.09142915
H	4.64013209	0.69073960	0.83822296
H	4.68200071	0.47738312	-0.94558948
H	3.92804603	1.86497043	-0.22553312
H	-1.58952752	-2.06659049	0.62005764

Time = 111.00 fs
E(CASSCF/6-31G(d)) = -479.5654021

C	-0.93460977	-0.20557519	-0.32412270
C	0.49076656	-0.31531783	-0.12679652
C	1.18117779	-1.51077677	0.36470390
C	1.56354677	0.68272263	-0.35016188
C	2.77306218	0.10852432	-0.02126289
C	2.54513537	-1.26965847	0.36965853
N	-1.66735945	0.79853988	0.05776732
C	-3.10201506	0.54451320	0.28396963
H	3.33801978	-1.99502675	0.52195834
C	-1.81692773	-1.48630842	-0.27407658
C	-3.24731234	-0.93749674	-0.23242832
H	-3.71506753	-0.93009453	-1.21057721
H	-3.85664725	-1.55701570	0.41600165
H	-3.38642603	0.63686667	1.32333072
H	-3.73140777	1.20156786	-0.29549015
H	-1.66937917	-2.15300051	-1.11962369
H	0.72547862	-2.46834341	0.57982882
C	-1.20173686	2.19647303	0.10147348
H	-0.33713086	2.23395429	0.74359546

H	-0.92509963	2.49076224	-0.90698504
H	-1.96070489	2.86453630	0.47807064
H	1.37322994	1.67759003	-0.70308835
C	4.09241016	0.79720790	-0.09225671
H	4.63958762	0.69106644	0.83717540
H	4.68133737	0.47686401	-0.94593840
H	3.93015978	1.86624515	-0.22718841
H	-1.58608768	-2.06952726	0.61509570

Time = 111.50 fs
E(CASSCF/6-31G(d)) = -479.5662205

C	-0.93768467	-0.20464273	-0.32710189
C	0.49369413	-0.31394312	-0.12827157
C	1.17982473	-1.51065398	0.36830972
C	1.56267387	0.68130975	-0.35448035
C	2.77470952	0.10771582	-0.02112279
C	2.54512545	-1.26798868	0.37363634
N	-1.66615592	0.79797742	0.05745473
C	-3.10294238	0.54267996	0.28606106
H	3.33154864	-1.99672882	0.53012725
C	-1.81646363	-1.48572933	-0.27718160
C	-3.24867457	-0.93719342	-0.23256645
H	-3.71796221	-0.92777229	-1.21035350
H	-3.86020586	-1.55633847	0.41450941
H	-3.37626280	0.63302373	1.32710904
H	-3.72897399	1.20354606	-0.29082570
H	-1.67427006	-2.15061713	-1.12448864
H	0.72882358	-2.46758836	0.58705044
C	-1.20167950	2.19709004	0.10409027
H	-0.33128749	2.22628620	0.73990933
H	-0.92764177	2.48939538	-0.90542099
H	-1.96426045	2.85903362	0.48699212
H	1.37783758	1.67535077	-0.71043833
C	4.09280463	0.79772175	-0.09309506
H	4.63983871	0.69092964	0.83584587
H	4.68132100	0.47587802	-0.94587219
H	3.93243204	1.86742944	-0.22884492
H	-1.58243229	-2.07190431	0.61028555

Time = 112.00 fs
E(CASSCF/6-31G(d)) = -479.5674899

C	-0.94110630	-0.20436868	-0.33028275
C	0.49747197	-0.31259874	-0.12966702
C	1.17814929	-1.51039331	0.37191201
C	1.56135819	0.67967624	-0.35883113

C	2.77639761	0.10679424	-0.02096638
C	2.54529125	-1.26620339	0.37761112
N	-1.66526037	0.79792322	0.05739831
C	-3.10377732	0.54046987	0.28799987
H	3.32433165	-1.99801492	0.53826439
C	-1.81594846	-1.48496258	-0.28042278
C	-3.25005984	-0.93679764	-0.23260779
H	-3.72046136	-0.92553355	-1.21015148
H	-3.86412452	-1.55507536	0.41304083
H	-3.36549170	0.62955589	1.33136762
H	-3.72617980	1.20637256	-0.28630446
H	-1.67855512	-2.14779071	-1.12929321
H	0.73207923	-2.46614199	0.59416142
C	-1.20153286	2.19754009	0.10670149
H	-0.32556669	2.21942067	0.73608736
H	-0.93023083	2.48848387	-0.90355316
H	-1.96815468	2.85295395	0.49592365
H	1.38311616	1.67326866	-0.71781659
C	4.09316376	0.79836767	-0.09394438
H	4.64090879	0.69034167	0.83426687
H	4.68198135	0.47442588	-0.94542756
H	3.93483706	1.86847487	-0.23049456
H	-1.57860171	-2.07367722	0.60557844

Time = 112.50 fs
E(CASSCF/6-31G(d)) = -479.5691244

C	-0.94480943	-0.20475440	-0.33366993
C	0.50203037	-0.31130854	-0.13098738
C	1.17618080	-1.50996176	0.37550416
C	1.55965218	0.67781791	-0.36320382
C	2.77809933	0.10577912	-0.02080243
C	2.54560438	-1.26430848	0.38158577
N	-1.66469177	0.79837315	0.05760233
C	-3.10449565	0.53789901	0.28978752
H	3.31659983	-1.99897556	0.54638232
C	-1.81539903	-1.48401338	-0.28379128
C	-3.25146706	-0.93631200	-0.23255420
H	-3.72253872	-0.92337658	-1.20996391
H	-3.86835030	-1.55325632	0.41160662
H	-3.35425760	0.62645890	1.33611686
H	-3.72311880	1.21001764	-0.28188892
H	-1.68219134	-2.14455485	-1.13407623
H	0.73510109	-2.46414335	0.60117831
C	-1.20129879	2.19780829	0.10930850
H	-0.32006587	2.21346329	0.73216397
H	-0.93291065	2.48812552	-0.90140016

H	-1.97220281	2.84632900	0.50482111
H	1.38897394	1.67137216	-0.72524081
C	4.09349294	0.79914140	-0.09480435
H	4.64279659	0.68932559	0.83246111
H	4.68332626	0.47252150	-0.94463690
H	3.93734854	1.86935216	-0.23213184
H	-1.57463824	-2.07480989	0.60092249

Time = 113.00 fs
E(CASSCF/6-31G(d)) = -479.5710164

C	-0.94872782	-0.20578644	-0.33726359
C	0.50728649	-0.31009768	-0.13223870
C	1.17395494	-1.50932500	0.37907939
C	1.55761489	0.67573541	-0.36758837
C	2.77978781	0.10469120	-0.02064028
C	2.54603086	-1.26230882	0.38556311
N	-1.66446056	0.79930970	0.05806592
C	-3.10507753	0.53499229	0.29142960
H	3.30862234	-1.99973564	0.55450306
C	-1.81483285	-1.48288843	-0.28727623
C	-3.25289432	-0.93573948	-0.23240902
H	-3.72417706	-0.92129673	-1.20978813
H	-3.87283078	-1.55092226	0.41022189
H	-3.34270461	0.62371378	1.34131116
H	-3.71986642	1.21440610	-0.27751342
H	-1.68514456	-2.14095569	-1.13888377
H	0.73773129	-2.46176046	0.60812499
C	-1.20098200	2.19788318	0.11191326
H	-0.31486831	2.20850380	0.72818556
H	-0.93572330	2.48841037	-0.89899267
H	-1.97621793	2.83920044	0.51363967
H	1.39530764	1.66966323	-0.73272166
C	4.09380083	0.80003465	-0.09567418
H	4.64547551	0.68791476	0.83044010
H	4.68534057	0.47019232	-0.94352637
H	3.93994006	1.87005294	-0.23375418
H	-1.57058458	-2.07527772	0.59626751

Time = 113.50 fs
E(CASSCF/6-31G(d)) = -479.5730395

C	-0.95279610	-0.20743618	-0.34105927
C	0.51314599	-0.30899256	-0.13342824
C	1.17151210	-1.50844953	0.38263120
C	1.55531101	0.67343515	-0.37197555
C	2.78143711	0.10355207	-0.02048971

C	2.54653328	-1.26020932	0.38954616
N	-1.66456840	0.80070207	0.05878284
C	-3.10550759	0.53178309	0.29293601
H	3.30068750	-2.00043986	0.56265499
C	-1.81426804	-1.48159596	-0.29086550
C	-3.25433879	-0.93508353	-0.23217704
H	-3.72536889	-0.91928643	-1.20962668
H	-3.87751473	-1.54812426	0.40890563
H	-3.33097509	0.62128697	1.34685035
H	-3.71647972	1.21941886	-0.27308756
H	-1.68739043	-2.13704855	-1.14376481
H	0.73980923	-2.45917151	0.61502842
C	-1.20058981	2.19775666	0.11451767
H	-0.31003816	2.20461377	0.72421126
H	-0.93870874	2.48941809	-0.89636998
H	-1.98002022	2.83162398	0.52233743
H	1.40200531	1.66811357	-0.74026065
C	4.09409908	0.80103516	-0.09655263
H	4.64889424	0.68615228	0.82820455
H	4.68798611	0.46747917	-0.94211534
H	3.94258539	1.87058953	-0.23536210
H	-1.56648223	-2.07507007	0.59156937

Time = 114.00 fs
E(CASSCF/6-31G(d)) = -479.5750611

C	-0.95695102	-0.20966009	-0.34504771
C	0.51950468	-0.30802062	-0.13456446
C	1.16889601	-1.50730500	0.38615405
C	1.55281004	0.67092985	-0.37635741
C	2.78302291	0.10238378	-0.02036066
C	2.54707305	-1.25801608	0.39353830
N	-1.66500832	0.80250672	0.05974141
C	-3.10577470	0.52831256	0.29432071
H	3.29308080	-2.00123423	0.57086915
C	-1.81372331	-1.48014598	-0.29454660
C	-3.25579671	-0.93434814	-0.23186441
H	-3.72611688	-0.91733552	-1.20948734
H	-3.88235236	-1.54492221	0.40767972
H	-3.31920952	0.61913098	1.35258723
H	-3.71300000	1.22489820	-0.26850254
H	-1.68891560	-2.13289414	-1.14876661
H	0.74118290	-2.45654440	0.62191397
C	-1.20013169	2.19742399	0.11712294
H	-0.30561704	2.20184446	0.72031208
H	-0.94190476	2.49121520	-0.89357547
H	-1.98344569	2.82367250	0.53087925

H	1.40894821	1.66666429	-0.74784963
C	4.09440197	0.80212705	-0.09743812
H	4.65297863	0.68409010	0.82574628
H	4.69120308	0.46443526	-0.94041723
H	3.94525905	1.87099327	-0.23695900
H	-1.56237009	-2.07419221	0.58679365

Time = 114.50 fs
E(CASSCF/6-31G(d)) = -479.5769514

C	-0.96113238	-0.21240037	-0.34921519
C	0.52625058	-0.30721005	-0.13565675
C	1.16615196	-1.50586644	0.38964402
C	1.55018499	0.66823845	-0.38072765
C	2.78452319	0.10120826	-0.02026287
C	2.54761275	-1.25573764	0.39754351
N	-1.66576504	0.80466843	0.06092478
C	-3.10587143	0.52462846	0.29560141
H	3.28606206	-2.00224541	0.57917380
C	-1.81321786	-1.47855050	-0.29830733
C	-3.25726343	-0.93353794	-0.23147848
H	-3.72643349	-0.91543128	-1.20938245
H	-3.88729506	-1.54138294	0.40656727
H	-3.30754643	0.61718556	1.35834017
H	-3.70945755	1.23065620	-0.26363960
H	-1.68971856	-2.12855462	-1.15392986
H	0.74171972	-2.45401602	0.62880009
C	-1.19961873	2.19688370	0.11972914
H	-0.30162261	2.20022504	0.71656887
H	-0.94534719	2.49385242	-0.89065203
H	-1.98635384	2.81543795	0.53924015
H	1.41601169	1.66522933	-0.75547126
C	4.09472571	0.80329128	-0.09832886
H	4.65763494	0.68178763	0.82305189
H	4.69491295	0.46112424	-0.93844177
H	3.94793732	1.87131164	-0.23855088
H	-1.55828326	-2.07266635	0.58191804

Time = 115.00 fs
E(CASSCF/6-31G(d)) = -479.5785965

C	-0.96528418	-0.21558642	-0.35354369
C	0.53326627	-0.30658928	-0.13671532
C	1.16332517	-1.50411652	0.39309934
C	1.54751133	0.66538595	-0.38508160
C	2.78591868	0.10004699	-0.02020593
C	2.54811850	-1.25338613	0.40156683

N	-1.66681575	0.80712173	0.06231118
C	-3.10579339	0.52078375	0.29679877
H	3.27984512	-2.00356338	0.58759085
C	-1.81277125	-1.47682370	-0.30213665
C	-3.25873359	-0.93265834	-0.23102777
H	-3.72634114	-0.91355906	-1.20932826
H	-3.89229558	-1.53757849	0.40559150
H	-3.29612349	0.61537958	1.36390936
H	-3.70587788	1.23648555	-0.25837954
H	-1.68981011	-2.12409015	-1.15928425
H	0.74131722	-2.45167325	0.63569412
C	-1.19906298	2.19613769	0.12233479
H	-0.29804936	2.19976195	0.71306897
H	-0.94906982	2.49736296	-0.88763741
H	-1.98863349	2.80703156	0.54740791
H	1.42306882	1.66370104	-0.76310100
C	4.09508780	0.80450645	-0.09922297
H	4.66275363	0.67931071	0.82010644
H	4.69902143	0.45761875	-0.93619773
H	3.95059832	1.87160250	-0.24014572
H	-1.55425204	-2.07053236	0.57693461

Time = 115.50 fs
E(CASSCF/6-31G(d)) = -479.5799033

C	-0.96935444	-0.21913659	-0.35801144
C	0.54043070	-0.30618657	-0.13775099
C	1.16045963	-1.50204690	0.39652097
C	1.54486586	0.66240243	-0.38941650
C	2.78719309	0.09892068	-0.02019887
C	2.54856154	-1.25097827	0.40561445
N	-1.66813103	0.80979276	0.06387461
C	-3.10553852	0.51683489	0.29793572
H	3.27458072	-2.00522281	0.59612945
C	-1.81240330	-1.47498212	-0.30602509
C	-3.26020121	-0.93171558	-0.23052181
H	-3.72587108	-0.91170244	-1.20934331
H	-3.89730715	-1.53358379	0.40477353
H	-3.28507713	0.61363329	1.36909456
H	-3.70228813	1.24217176	-0.25261303
H	-1.68921356	-2.11955550	-1.16484497
H	0.73990907	-2.44954120	0.64258808
C	-1.19847681	2.19519124	0.12493677
H	-0.29487103	2.20043828	0.70990206
H	-0.95310412	2.50176008	-0.88456049
H	-1.99020693	2.79858292	0.55538453
H	1.42998905	1.66196034	-0.77070975

C	4.09550613	0.80574962	-0.10011864
H	4.66821408	0.67672929	0.81689863
H	4.70342340	0.45399689	-0.93369658
H	3.95322372	1.87192907	-0.24175271
H	-1.55030135	-2.06784671	0.57184977

Time = 116.00 fs
E(CASSCF/6-31G(d)) = -479.5808042

C	-0.97329563	-0.22296030	-0.36259358
C	0.54762159	-0.30602911	-0.13877526
C	1.15759698	-1.49965927	0.39991280
C	1.54232528	0.65932199	-0.39373109
C	2.78833347	0.09784884	-0.02025009
C	2.54891958	-1.24853571	0.40969394
N	-1.66967590	0.81260138	0.06558541
C	-3.10510645	0.51284012	0.29903644
H	3.27034700	-2.00719518	0.60478422
C	-1.81213390	-1.47304469	-0.30996512
C	-3.26165992	-0.93071679	-0.22997111
H	-3.72506247	-0.90984380	-1.20944646
H	-3.90228301	-1.52947434	0.40413086
H	-3.27454198	0.61186094	1.37371272
H	-3.69872289	1.24750546	-0.24624922
H	-1.68796473	-2.11499795	-1.17060986
H	0.73746981	-2.44757660	0.64945728
C	-1.19787232	2.19405301	0.12753037
H	-0.29204545	2.20221410	0.70715512
H	-0.95747917	2.50703614	-0.88143770
H	-1.99103167	2.79023665	0.56318694
H	1.43664209	1.65988685	-0.77826772
C	4.09599810	0.80699733	-0.10101426
H	4.67388984	0.67411600	0.81342560
H	4.70800755	0.45033973	-0.93095652
H	3.95579868	1.87235303	-0.24338140
H	-1.54645072	-2.06468090	0.56668365

Time = 116.50 fs
E(CASSCF/6-31G(d)) = -479.5812593

C	-0.97706464	-0.22696055	-0.36726278
C	0.55471765	-0.30614224	-0.13980020
C	1.15477586	-1.49696536	0.40328187
C	1.53996478	0.65618117	-0.39802549
C	2.78933027	0.09684949	-0.02036713
C	2.54917716	-1.24608466	0.41381418
N	-1.67141103	0.81546348	0.06741111

C	-3.10449798	0.50885756	0.30012553
H	3.26714773	-2.00938619	0.61353332
C	-1.81198275	-1.47103265	-0.31395126
C	-3.26310315	-0.92967003	-0.22938704
H	-3.72396110	-0.90796483	-1.20965488
H	-3.90717581	-1.52532417	0.40367568
H	-3.26464934	0.60997369	1.37761370
H	-3.69522902	1.25229427	-0.23922343
H	-1.68611140	-2.11045594	-1.17655850
H	0.73401449	-2.44567094	0.65626056
C	-1.19726074	2.19273507	0.13010956
H	-0.28952036	2.20502713	0.70490704
H	-0.96222100	2.51316177	-0.87827135
H	-1.99109999	2.78214804	0.57084625
H	1.44289917	1.65737062	-0.78574832
C	4.09657978	0.80822659	-0.10190856
H	4.67965370	0.67154391	0.80969735
H	4.71266154	0.44672802	-0.92800589
H	3.95831269	1.87292807	-0.24504080
H	-1.54271453	-2.06111895	0.56146813

Time = 117.00 fs
E(CASSCF/6-31G(d)) = -479.5812610

C	-0.98062276	-0.23103655	-0.37199016
C	0.56160121	-0.30654833	-0.14083853
C	1.15203142	-1.49398625	0.40663814
C	1.53785617	0.65301726	-0.40230083
C	2.79017744	0.09593866	-0.02055652
C	2.54932542	-1.24365484	0.41798509
N	-1.67329384	0.81829336	0.06931732
C	-3.10371471	0.50494347	0.30122710
H	3.26491855	-2.01164289	0.62233955
C	-1.81196909	-1.46896935	-0.31797998
C	-3.26452439	-0.92858421	-0.22878175
H	-3.72261806	-0.90604699	-1.20998206
H	-3.91193721	-1.52120388	0.40341365
H	-3.25552500	0.60788258	1.38069321
H	-3.69186881	1.25637330	-0.23150248
H	-1.68371256	-2.10595904	-1.18265279
H	0.72959538	-2.44366128	0.66294319
C	-1.19665207	2.19125284	0.13266757
H	-0.28724022	2.20879400	0.70322313
H	-0.96735197	2.52008610	-0.87504916
H	-1.99043613	2.77447681	0.57840594
H	1.44863598	1.65432315	-0.79313220
C	4.09726512	0.80941590	-0.10280073

H	4.68538271	0.66908471	0.80574016
H	4.71727684	0.44323914	-0.92488608
H	3.96075977	1.87369373	-0.24673844
H	-1.53910260	-2.05725409	0.55624395

Time = 117.50 fs
E(CASSCF/6-31G(d)) = -479.5808302

C	-0.98393538	-0.23508636	-0.37674584
C	0.56816007	-0.30726582	-0.14190353
C	1.14939540	-1.49075085	0.40999413
C	1.53606653	0.64986647	-0.40655888
C	2.79087226	0.09513026	-0.02082362
C	2.54936110	-1.24127795	0.42221730
N	-1.67527969	0.82100604	0.07126861
C	-3.10275885	0.50115065	0.30236407
H	3.26354037	-2.01376732	0.63115278
C	-1.81211127	-1.46687983	-0.32204945
C	-3.26591730	-0.92746901	-0.22816809
H	-3.72108840	-0.90407195	-1.21043589
H	-3.91651771	-1.51717913	0.40334298
H	-3.24728653	0.60550166	1.38290183
H	-3.68872148	1.25961371	-0.22308827
H	-1.68083732	-2.10152873	-1.18883909
H	0.72429537	-2.44134739	0.66944099
C	-1.19605473	2.18962515	0.13519752
H	-0.28515300	2.21341184	0.70215018
H	-0.97288965	2.52773714	-0.87174542
H	-1.98909138	2.76738038	0.58591862
H	1.45373443	1.65068670	-0.80041044
C	4.09806530	0.81054608	-0.10369050
H	4.69096252	0.66680654	0.80159884
H	4.72175325	0.43994410	-0.92165332
H	3.96313880	1.87467076	-0.24847968
H	-1.53562136	-2.05318490	0.55105707

Time = 118.00 fs
E(CASSCF/6-31G(d)) = -479.5800246

C	-0.98697184	-0.23900938	-0.38149957
C	0.57428958	-0.30830811	-0.14300924
C	1.14689641	-1.48729408	0.41336438
C	1.53465655	0.64676218	-0.41080156
C	2.79141512	0.09443581	-0.02117251
C	2.54928517	-1.23898565	0.42652150
N	-1.67732291	0.82351936	0.07322930
C	-3.10163321	0.49752698	0.30355745

H	3.26285685	-2.01553527	0.63991466
C	-1.81242637	-1.46479037	-0.32615899
C	-3.26727593	-0.92633471	-0.22755954
H	-3.71942967	-0.90202218	-1.21101738
H	-3.92086682	-1.51330952	0.40345390
H	-3.24004047	0.60275063	1.38425036
H	-3.68588296	1.26192874	-0.21401895
H	-1.67756350	-2.09718004	-1.19505190
H	0.71821944	-2.43851312	0.67568639
C	-1.19547557	2.18787419	0.13769332
H	-0.28321615	2.21876008	0.70171235
H	-0.97884563	2.53602334	-0.86832375
H	-1.98713739	2.76100607	0.59344199
H	1.45808534	1.64644139	-0.80758765
C	4.09898820	0.81160090	-0.10457815
H	4.69629094	0.66477218	0.79733744
H	4.72600251	0.43690503	-0.91837925
H	3.96545343	1.87585793	-0.25026708
H	-1.53227514	-2.04901090	0.54595401

Time = 118.50 fs
E(CASSCF/6-31G(d)) = -479.5789334

C	-0.98970560	-0.24270885	-0.38622136
C	0.57989479	-0.30968242	-0.14417045
C	1.14455999	-1.48365474	0.41676480
C	1.53367876	0.64373332	-0.41503059
C	2.79180949	0.09386417	-0.02160589
C	2.54910134	-1.23680737	0.43090789
N	-1.67937779	0.82575600	0.07516431
C	-3.10034122	0.49411428	0.30482591
H	3.26269343	-2.01671821	0.64856434
C	-1.81292970	-1.46272780	-0.33030861
C	-3.26859474	-0.92519203	-0.22697006
H	-3.71770079	-0.89988130	-1.21171960
H	-3.92493364	-1.50964787	0.40372891
H	-3.23387892	0.59955738	1.38481092
H	-3.68346380	1.26327729	-0.20436755
H	-1.67397599	-2.09292405	-1.20121844
H	0.71148568	-2.43494861	0.68161496
C	-1.19491995	2.18602531	0.14015046
H	-0.28140149	2.22470251	0.70190849
H	-0.98522455	2.54483591	-0.86474062
H	-1.98465818	2.75548276	0.60103409
H	1.46159146	1.64160915	-0.81468347
C	4.10003815	0.81256756	-0.10546454
H	4.70128091	0.66303755	0.79303856

H	4.72995132	0.43417332	-0.91515036
H	3.96771142	1.87723097	-0.25210015
H	-1.52906767	-2.04482822	0.54097747

Time = 119.00 fs
E(CASSCF/6-31G(d)) = -479.5776699

C	-0.99211426	-0.24609410	-0.39088200
C	0.58489231	-0.31138882	-0.14540286
C	1.14240885	-1.47987343	0.42021200
C	1.53317589	0.64080292	-0.41924715
C	2.79206171	0.09342126	-0.02212486
C	2.54881467	-1.23476815	0.43538546
N	-1.68139938	0.82764524	0.07703992
C	-3.09888716	0.49094737	0.30618538
H	3.26287578	-2.01710444	0.65704374
C	-1.81363425	-1.46071867	-0.33449827
C	-3.26986867	-0.92405180	-0.22641401
H	-3.71596087	-0.89763450	-1.21252712
H	-3.92866750	-1.50624010	0.40414339
H	-3.22887670	0.59586027	1.38471363
H	-3.68158537	1.26366450	-0.19423908
H	-1.67016499	-2.08877035	-1.20726352
H	0.70421661	-2.43047140	0.68717173
C	-1.19439214	2.18410681	0.14256682
H	-0.27969789	2.23108971	0.70271137
H	-0.99202268	2.55405138	-0.86094991
H	-1.98174186	2.75091336	0.60874789
H	1.46417013	1.63625443	-0.82173265
C	4.10121568	0.81343683	-0.10635106
H	4.70586217	0.66165042	0.78880102
H	4.73354324	0.43178833	-0.91206598
H	3.96992384	1.87874384	-0.25397535
H	-1.52600393	-2.04072523	0.53616206

Time = 119.50 fs
E(CASSCF/6-31G(d)) = -479.5763622

C	-0.99417986	-0.24908255	-0.39545340
C	0.58921176	-0.31341946	-0.14672314
C	1.14046310	-1.47599054	0.42372256
C	1.53317968	0.63798725	-0.42345167
C	2.79218071	0.09311004	-0.02272901
C	2.54843025	-1.23288696	0.43996148
N	-1.68334428	0.82912448	0.07882445
C	-3.09727635	0.48805355	0.30764893
H	3.26324611	-2.01651754	0.66530395

C	-1.81455019	-1.45878843	-0.33872739
C	-3.27109317	-0.92292476	-0.22590596
H	-3.71426850	-0.89526877	-1.21341660
H	-3.93201948	-1.50312513	0.40466672
H	-3.22508838	0.59160928	1.38413955
H	-3.68037475	1.26313924	-0.18376468
H	-1.66622412	-2.08473007	-1.21311542
H	0.69653110	-2.42494428	0.69231712
C	-1.19389586	2.18214950	0.14494337
H	-0.27811137	2.23776164	0.70406793
H	-0.99922760	2.56353587	-0.85690793
H	-1.97847249	2.74736748	0.61662701
H	1.46575581	1.63048078	-0.82878472
C	4.10251777	0.81420297	-0.10723953
H	4.70998196	0.66064952	0.78473606
H	4.73673937	0.42977666	-0.90923513
H	3.97210395	1.88033200	-0.25588645
H	-1.52309128	-2.03677902	0.53153047

Time = 120.00 fs
E(CASSCF/6-31G(d)) = -479.5751438

C	-0.99588837	-0.25160119	-0.39990886
C	0.59279602	-0.31575811	-0.14814894
C	1.13874087	-1.47204480	0.42731241
C	1.53371021	0.63529552	-0.42764389
C	2.79217782	0.09293060	-0.02341636
C	2.54795194	-1.23117558	0.44464115
N	-1.68517140	0.83014066	0.08048883
C	-3.09551548	0.48545259	0.30922672
H	3.26367657	-2.01482979	0.67330975
C	-1.81568449	-1.45696089	-0.34299429
C	-3.27226413	-0.92182120	-0.22546058
H	-3.71268123	-0.89277315	-1.21435792
H	-3.93494405	-1.50033534	0.40526385
H	-3.22254514	0.58676721	1.38330903
H	-3.67995750	1.26178882	-0.17309463
H	-1.66224885	-2.08081867	-1.21871118
H	0.68853691	-2.41828883	0.69703137
C	-1.19343498	2.18018604	0.14728466
H	-0.27666307	2.24455009	0.70590153
H	-1.00681802	2.57314809	-0.85257823
H	-1.97492324	2.74487558	0.62470133
H	1.46630145	1.62442466	-0.83590156
C	4.10393812	0.81486332	-0.10813205
H	4.71360440	0.66006386	0.78096190
H	4.73951795	0.42815221	-0.90677170

H	3.97426624	1.88191747	-0.25782510
H	-1.52034117	-2.03305312	0.52709083

Time = 120.50 fs
E(CASSCF/6-31G(d)) = -479.5741299

C	-0.99723061	-0.25358817	-0.40422345
C	0.59560292	-0.31838011	-0.14969875
C	1.13725781	-1.46807155	0.43099627
C	1.53477487	0.63272985	-0.43182281
C	2.79206653	0.09288011	-0.02418333
C	2.54738195	-1.22963754	0.44942707
N	-1.68684241	0.83065096	0.08200718
C	-3.09361316	0.48315681	0.31092638
H	3.26407799	-2.01197360	0.68104261
C	-1.81704036	-1.45525702	-0.34729588
C	-3.27337779	-0.92075075	-0.22509237
H	-3.71125500	-0.89013892	-1.21531568
H	-3.93740042	-1.49789762	0.40589701
H	-3.22125362	0.58131028	1.38246664
H	-3.68044994	1.25973063	-0.16239047
H	-1.65833415	-2.07705728	-1.22400058
H	0.68032655	-2.41049427	0.70131791
C	-1.19301400	2.17825034	0.14959895
H	-0.27538484	2.25128237	0.70811664
H	-1.01476287	2.58274398	-0.84793608
H	-1.97115176	2.74342654	0.63298386
H	1.46578060	1.61824381	-0.84315283
C	4.10546759	0.81541781	-0.10903085
H	4.71670934	0.65991259	0.77759780
H	4.74187328	0.42691703	-0.90478941
H	3.97642462	1.88341556	-0.25978165
H	-1.51776969	-2.02959510	0.52283555

Time = 121.00 fs
E(CASSCF/6-31G(d)) = -479.5734195

C	-0.99820190	-0.25499397	-0.40837419
C	0.59760484	-0.32125259	-0.15139176
C	1.13602742	-1.46410197	0.43478717
C	1.53636825	0.63028596	-0.43598711
C	2.79186240	0.09295308	-0.02502483
C	2.54672012	-1.22826800	0.45431921
N	-1.68832234	0.83062378	0.08335725
C	-3.09158034	0.48117168	0.31275342
H	3.26440565	-2.00794613	0.68850345
C	-1.81861695	-1.45369436	-0.35162750

C	-3.27443060	-0.91972206	-0.22481551
H	-3.71004387	-0.88735975	-1.21625142
H	-3.93935421	-1.49583406	0.40652760
H	-3.22119368	0.57522795	1.38186267
H	-3.68194992	1.25710138	-0.15181533
H	-1.65457294	-2.07347412	-1.22894967
H	0.67197363	-2.40162084	0.70520501
C	-1.19263865	2.17637664	0.15189814
H	-0.27431305	2.25778456	0.71060380
H	-1.02302198	2.59218063	-0.84297236
H	-1.96719805	2.74296679	0.64146968
H	1.46418843	1.61210393	-0.85061069
C	4.10709480	0.81586822	-0.10993812
H	4.71929011	0.66020522	0.77475700
H	4.74381354	0.42606267	-0.90339565
H	3.97859118	1.88474191	-0.26174603
H	-1.51539826	-2.02643633	0.51874126

Time = 121.50 fs
E(CASSCF/6-31G(d)) = -479.5730811

C	-0.99880232	-0.25578207	-0.41234038
C	0.59878892	-0.32433527	-0.15324747
C	1.13506069	-1.46016225	0.43869609
C	1.53847229	0.62795448	-0.44013561
C	2.79158273	0.09314164	-0.02593436
C	2.54596395	-1.22705388	0.45931475
N	-1.68957997	0.83003906	0.08452082
C	-3.08943097	0.47949666	0.31471196
H	3.26466102	-2.00281008	0.69571316
C	-1.82040916	-1.45228637	-0.35598296
C	-3.27541917	-0.91874261	-0.22464360
H	-3.70909976	-0.88443164	-1.21712585
H	-3.94077888	-1.49416290	0.40711795
H	-3.22231750	0.56852278	1.38173263
H	-3.68452709	1.25404474	-0.14152438
H	-1.65105443	-2.07010475	-1.23354288
H	0.66353171	-2.39179836	0.70874599
C	-1.19231623	2.17459840	0.15419734
H	-0.27348187	2.26388541	0.71324572
H	-1.03154655	2.60132040	-0.83769615
H	-1.96308523	2.74340241	0.65013631
H	1.46154270	1.60616287	-0.85834328
C	4.10880686	0.81621743	-0.11085585
H	4.72135056	0.66094188	0.77253948
H	4.74535776	0.42557206	-0.90268508
H	3.98077486	1.88581937	-0.26370871

H	-1.51325356	-2.02359257	0.51477064

Time = 122.00 fs
E(CASSCF/6-31G(d)) = -479.5731501

C	-0.99903668	-0.25592953	-0.41610393
C	0.59915644	-0.32758162	-0.15528528
C	1.13436587	-1.45627339	0.44273176
C	1.54105684	0.62572254	-0.44426787
C	2.79124639	0.09343593	-0.02690411
C	2.54510870	-1.22597462	0.46440833
N	-1.69058822	0.82888863	0.08548406
C	-3.08718244	0.47812617	0.31680543
H	3.26488988	-1.99668960	0.70271223
C	-1.82240753	-1.45104196	-0.36035474
C	-3.27634011	-0.91781853	-0.22458942
H	-3.70847222	-0.88135299	-1.21790132
H	-3.94165690	-1.49289934	0.40763299
H	-3.22454895	0.56120990	1.38227667
H	-3.68821360	1.25069855	-0.13165543
H	-1.64786278	-2.06699150	-1.23778369
H	0.65503527	-2.38121939	0.71201761
C	-1.19205571	2.17294716	0.15651426
H	-0.27291627	2.26942032	0.71592337
H	-1.04028017	2.61003461	-0.83213638
H	-1.95882273	2.74460383	0.65894620
H	1.45788453	1.60055444	-0.86640762
C	4.11059011	0.81646866	-0.11178562
H	4.72290191	0.66211399	0.77102500
H	4.74653256	0.42542166	-0.90273334
H	3.98298027	1.88658434	-0.26566150
H	-1.51136692	-2.02106572	0.51087534

Time = 122.50 fs
E(CASSCF/6-31G(d)) = -479.5736264

C	-0.99891429	-0.25542722	-0.41964963
C	0.59872209	-0.33094021	-0.15752393
C	1.13394803	-1.45245135	0.44690053
C	1.54408040	0.62357551	-0.44838492
C	2.79087372	0.09382443	-0.02792519
C	2.54414784	-1.22500332	0.46959223
N	-1.69132449	0.82717626	0.08623780
C	-3.08485615	0.47705059	0.31903739
H	3.26517668	-1.98976185	0.70955877
C	-1.82459835	-1.44996523	-0.36473442
C	-3.27719006	-0.91695443	-0.22466470

H	-3.70820807	-0.87812446	-1.21854411
H	-3.94198045	-1.49205611	0.40804153
H	-3.22778357	0.55331670	1.38364094
H	-3.69299595	1.24718301	-0.12232100
H	-1.64507598	-2.06418208	-1.24169408
H	0.64650290	-2.37012741	0.71511692
C	-1.19186771	2.17145136	0.15886833
H	-0.27262561	2.27423531	0.71852185
H	-1.04916013	2.61820681	-0.82634204
H	-1.95441148	2.74641274	0.66785052
H	1.45327897	1.59537369	-0.87484294
C	4.11243084	0.81662481	-0.11272855
H	4.72395964	0.66370503	0.77026699
H	4.74736868	0.42558379	-0.90359149
H	3.98520679	1.88699182	-0.26759829
H	-1.50977339	-2.01884640	0.50700005

Time = 123.00 fs
E(CASSCF/6-31G(d)) = -479.5744784

C	-0.99844863	-0.25427978	-0.42296554
C	0.59751292	-0.33435621	-0.15998099
C	1.13380851	-1.44870762	0.45120649
C	1.54749108	0.62149869	-0.45248991
C	2.79048626	0.09429437	-0.02898774
C	2.54307388	-1.22410825	0.47485684
N	-1.69177087	0.82491760	0.08677778
C	-3.08247767	0.47625713	0.32141203
H	3.26563495	-1.98224397	0.71632529
C	-1.82696390	-1.44905538	-0.36911320
C	-3.27796563	-0.91615339	-0.22487989
H	-3.70835119	-0.87474878	-1.21902641
H	-3.94175185	-1.49164387	0.40831727
H	-3.23188919	0.54488255	1.38590363
H	-3.69880925	1.24359151	-0.11360213
H	-1.64276506	-2.06172742	-1.24531254
H	0.63794291	-2.35880018	0.71815648
C	-1.19176416	2.17013507	0.16127973
H	-0.27259862	2.27819088	0.72093530
H	-1.05811932	2.62573553	-0.82038100
H	-1.94985014	2.74865046	0.67679462
H	1.44781545	1.59066570	-0.88366512
C	4.11431595	0.81668795	-0.11368515
H	4.72454079	0.66569136	0.77028784
H	4.74789775	0.42602875	-0.90528138
H	3.98744798	1.88701901	-0.26951562
H	-1.50851023	-2.01691745	0.50308746

Time = 123.50 fs
E(CASSCF/6-31G(d)) = -479.5756410

C	-0.99765723	-0.25250519	-0.42604338
C	0.59556752	-0.33777337	-0.16267205
C	1.13394397	-1.44505008	0.45565161
C	1.55122804	0.61947870	-0.45658851
C	2.79010643	0.09483223	-0.03008122
C	2.54187952	-1.22325475	0.48019122
N	-1.69191440	0.82213961	0.08710474
C	-3.08007678	0.47573035	0.32393465
H	3.26639380	-1.97437655	0.72309328
C	-1.82948279	-1.44830693	-0.37348248
C	-3.27866349	-0.91541686	-0.22524390
H	-3.70894178	-0.87123083	-1.21932770
H	-3.94098324	-1.49167158	0.40843932
H	-3.23670825	0.53595922	1.38906726
H	-3.70553497	1.23998540	-0.10554576
H	-1.64099336	-2.05967853	-1.24869083
H	0.62936143	-2.34753034	0.72125812
C	-1.19175781	2.16901714	0.16376830
H	-0.27280012	2.28116560	0.72307153
H	-1.06708726	2.63253626	-0.81433789
H	-1.94514170	2.75112760	0.68572290
H	1.44160753	1.58641978	-0.89286327
C	4.11623339	0.81665894	-0.11465537
H	4.72466201	0.66804281	0.77107620
H	4.74814977	0.42672664	-0.90779289
H	3.98969143	1.88666681	-0.27141280
H	-1.50761523	-2.01525677	0.49908261

Time = 124.00 fs
E(CASSCF/6-31G(d)) = -479.5770313

C	-0.99656107	-0.25013428	-0.42887892
C	0.59293484	-0.34113567	-0.16561014
C	1.13434564	-1.44148411	0.46023603
C	1.55522293	0.61750440	-0.46068922
C	2.78975724	0.09542402	-0.03119458
C	2.54055901	-1.22240727	0.48558371
N	-1.69174724	0.81888015	0.08722453
C	-3.07768699	0.47545246	0.32661155
H	3.26758170	-1.96640410	0.72994744
C	-1.83213050	-1.44771005	-0.37783457
C	-3.27928053	-0.91474475	-0.22576398
H	-3.71001577	-0.86757737	-1.21943570

H	-3.93969595	-1.49214629	0.40839224
H	-3.24206063	0.52661105	1.39305909
H	-3.71300262	1.23639319	-0.09816438
H	-1.63981625	-2.05808321	-1.25188997
H	0.62077179	-2.33660297	0.72454604
C	-1.19186164	2.16811030	0.16635255
H	-0.27316965	2.28305908	0.72485468
H	-1.07599226	2.63854262	-0.80831034
H	-1.94029943	2.75365344	0.69458472
H	1.43479226	1.58256998	-0.90239909
C	4.11817250	0.81653733	-0.11563857
H	4.72433858	0.67072341	0.77258637
H	4.74815127	0.42764865	-0.91108266
H	3.99191890	1.88595973	-0.27329199
H	-1.50712478	-2.01384089	0.49493773

Time = 124.50 fs
E(CASSCF/6-31G(d)) = -479.5785496

C	-0.99518403	-0.24720992	-0.43147230
C	0.58967305	-0.34438900	-0.16880518
C	1.13499842	-1.43801390	0.46495842
C	1.55940136	0.61556719	-0.46480330
C	2.78946191	0.09605565	-0.03231650
C	2.53910969	-1.22153146	0.49102265
N	-1.69126682	0.81518730	0.08714816
C	-3.07534472	0.47540329	0.32944969
H	3.26930868	-1.95855482	0.73696890
C	-1.83487998	-1.44725110	-0.38216329
C	-3.27981400	-0.91413546	-0.22644547
H	-3.71160415	-0.86379692	-1.21934665
H	-3.93791940	-1.49307280	0.40816575
H	-3.24774809	0.51691526	1.39773934
H	-3.72099589	1.23281433	-0.09143883
H	-1.63928103	-2.05698253	-1.25497556
H	0.61220487	-2.32627328	0.72813944
C	-1.19208819	2.16742056	0.16904870
H	-0.27362234	2.28379447	0.72622671
H	-1.08476324	2.64370681	-0.80240430
H	-1.93535146	2.75604516	0.70333949
H	1.42752878	1.57900172	-0.91220903
C	4.12012407	0.81632147	-0.11663366
H	4.72358447	0.67369209	0.77473997
H	4.74792449	0.42876772	-0.91507520
H	3.99410662	1.88494358	-0.27515798
H	-1.50707198	-2.01264800	0.49061627

Time = 125.00 fs
E(CASSCF/6-31G(d)) = -479.5800977

C	-0.99355220	-0.24378601	-0.43382843
C	0.58584826	-0.34748277	-0.17226337
C	1.13588022	-1.43464349	0.46981632
C	1.56368502	0.61366079	-0.46894450
C	2.78924325	0.09671331	-0.03343565
C	2.53753353	-1.22059628	0.49649709
N	-1.69047603	0.81111841	0.08689173
C	-3.07308798	0.47556000	0.33245597
H	3.27164770	-1.95102010	0.74422826
C	-1.83770234	-1.44691338	-0.38646455
C	-3.28026173	-0.91358600	-0.22729178
H	-3.71373198	-0.85989949	-1.21906480
H	-3.93568956	-1.49445310	0.40775403
H	-3.25355966	0.50696180	1.40291590
H	-3.72926240	1.22922689	-0.08532340
H	-1.63942695	-2.05640721	-1.25801287
H	0.60371844	-2.31674748	0.73214590
C	-1.19244876	2.16694699	0.17186981
H	-0.27405245	2.28331981	0.72714733
H	-1.09333161	2.64799885	-0.79672807
H	-1.93034398	2.75813563	0.71196084
H	1.41999436	1.57556506	-0.92220980
C	4.12208023	0.81600882	-0.11763919
H	4.72241351	0.67690291	0.77743026
H	4.74748792	0.43005838	-0.91966591
H	3.99622661	1.88368189	-0.27701766
H	-1.50748482	-2.01166039	0.48609615

Time = 125.50 fs
E(CASSCF/6-31G(d)) = -479.5815738

C	-0.99169321	-0.23992636	-0.43595713
C	0.58153353	-0.35037115	-0.17598680
C	1.13696144	-1.43137760	0.47480642
C	1.56799329	0.61178056	-0.47312848
C	2.78912323	0.09738358	-0.03454087
C	2.53583843	-1.21957538	0.50199738
N	-1.68938329	0.80673915	0.08647629
C	-3.07095482	0.47589674	0.33563628
H	3.27461948	-1.94393934	0.75177963
C	-1.84056755	-1.44667787	-0.39073685
C	-3.28062244	-0.91309218	-0.22830426
H	-3.71641757	-0.85589649	-1.21860139
H	-3.93304724	-1.49628568	0.40715472

H	-3.25927837	0.49685259	1.40836387
H	-3.73752684	1.22559766	-0.07975269
H	-1.64028543	-2.05637457	-1.26106219
H	0.59540440	-2.30816858	0.73665574
C	-1.19295268	2.16668180	0.17482520
H	-0.27433867	2.28160908	0.72759264
H	-1.10163296	2.65140562	-0.79138619
H	-1.92534243	2.75978025	0.72043965
H	1.41238096	1.57209075	-0.93230514
C	4.12403414	0.81559646	-0.11865353
H	4.72084122	0.68030576	0.78052795
H	4.74685736	0.43149624	-0.92472604
H	3.99824740	1.88225033	-0.27887928
H	-1.50838472	-2.01086635	0.48137161

Time = 126.00 fs
E(CASSCF/6-31G(d)) = -479.5828991

C	-0.98963551	-0.23570332	-0.43787329
C	0.57680748	-0.35301427	-0.17997314
C	1.13820516	-1.42822191	0.47992470
C	1.57224568	0.60992253	-0.47737199
C	2.78912212	0.09805382	-0.03562155
C	2.53403894	-1.21844837	0.50751564
N	-1.68800266	0.80212224	0.08592767
C	-3.06898147	0.47638433	0.33899451
H	3.27818239	-1.93738934	0.75965628
C	-1.84344517	-1.44652406	-0.39498155
C	-3.28089597	-0.91264878	-0.22948224
H	-3.71967143	-0.85180053	-1.21797313
H	-3.93003612	-1.49856471	0.40636773
H	-3.26468837	0.48669983	1.41384714
H	-3.74550560	1.22189334	-0.07464914
H	-1.64188013	-2.05688589	-1.26417466
H	0.58739134	-2.30060930	0.74173880
C	-1.19360655	2.16661056	0.17791997
H	-0.27435197	2.27866192	0.72755232
H	-1.10960876	2.65392865	-0.78647264
H	-1.92043061	2.76086159	0.72878563
H	1.40488830	1.56840871	-0.94239510
C	4.12597960	0.81508170	-0.11967499
H	4.71888738	0.68384660	0.78388875
H	4.74604818	0.43305679	-0.93010909
H	4.00013598	1.88073051	-0.28075164
H	-1.50978508	-2.01026091	0.47645407

Time = 126.50 fs

E(CASSCF/6-31G(d)) = -479.5840086

C	-0.98740777	-0.23119636	-0.43959680
C	0.57175314	-0.35537908	-0.18421545
C	1.13956763	-1.42518298	0.48516651
C	1.57636376	0.60808242	-0.48169204
C	2.78925791	0.09871234	-0.03666780
C	2.53215643	-1.21720111	0.51304594
N	-1.68635380	0.79734619	0.08527609
C	-3.06720051	0.47699011	0.34253155
H	3.28222948	-1.93138180	0.76786871
C	-1.84630497	-1.44643070	-0.39920302
C	-3.28108350	-0.91224974	-0.23082305
H	-3.72349525	-0.84762533	-1.21720042
H	-3.92670086	-1.50127920	0.40539401
H	-3.26958240	0.47662348	1.41913964
H	-3.75292202	1.21809127	-0.06993069
H	-1.64422697	-2.05792473	-1.26738918
H	0.57984211	-2.29407317	0.74744322
C	-1.19441354	2.16671287	0.18115488
H	-0.27396488	2.27450291	0.72702599
H	-1.11720772	2.65558184	-0.78206455
H	-1.91570801	2.76129237	0.73702726
H	1.39771704	1.56436607	-0.95238528
C	4.12791057	0.81446279	-0.12070209
H	4.71657866	0.68746800	0.78736164
H	4.74507783	0.43471401	-0.93565815
H	4.00185941	1.87920302	-0.28264304
H	-1.51169043	-2.00984579	0.47137130

Time = 127.00 fs
E(CASSCF/6-31G(d)) = -479.5848461

C	-0.98503823	-0.22649051	-0.44115226
C	0.56645696	-0.35743972	-0.18870226
C	1.14099929	-1.42226747	0.49052646
C	1.58027328	0.60625433	-0.48610495
C	2.78954546	0.09934844	-0.03767070
C	2.53021856	-1.21582505	0.51858429
N	-1.68446181	0.79249369	0.08455575
C	-3.06563928	0.47767799	0.34624454
H	3.28659338	-1.92586984	0.77640557
C	-1.84911767	-1.44637660	-0.40340852
C	-3.28118784	-0.91188842	-0.23232214
H	-3.72788123	-0.84338557	-1.21630507
H	-3.92308538	-1.50441245	0.40423441
H	-3.27376893	0.46674790	1.42404424

H	-3.75952107	1.21418843	-0.06551736
H	-1.64733420	-2.05945662	-1.27073071
H	0.57294772	-2.28850295	0.75379587
C	-1.19537271	2.16696322	0.18452637
H	-0.27306163	2.26918071	0.72601891
H	-1.12438716	2.65638979	-0.77821666
H	-1.91128575	2.76101679	0.74521069
H	1.39106320	1.55984202	-0.96219406
C	4.12982059	0.81373964	-0.12173361
H	4.71395092	0.69111010	0.79079715
H	4.74396802	0.43643925	-0.94121382
H	4.00338587	1.87774023	-0.28456037
H	-1.51409624	-2.00962927	0.46616560

Time = 127.50 fs
E(CASSCF/6-31G(d)) = -479.5853947

C	-0.98255428	-0.22167465	-0.44256854
C	0.56100740	-0.35917797	-0.19341774
C	1.14244647	-1.41948116	0.49599832
C	1.58390619	0.60443000	-0.49062551
C	2.78999589	0.09995264	-0.03862251
C	2.52825790	-1.21431635	0.52412834
N	-1.68235691	0.78765000	0.08380418
C	-3.06431831	0.47840873	0.35012617
H	3.29105866	-1.92075954	0.78523665
C	-1.85185546	-1.44634127	-0.40760793
C	-3.28121354	-0.91155775	-0.23397324
H	-3.73281111	-0.83909678	-1.21530811
H	-3.91923108	-1.50794120	0.40288861
H	-3.27707839	0.45719794	1.42840816
H	-3.76508183	1.21020820	-0.06133696
H	-1.65120206	-2.06142963	-1.27420959
H	0.56691516	-2.28379485	0.76080531
C	-1.19647861	2.16733196	0.18802692
H	-0.27154865	2.26276622	0.72453775
H	-1.13111405	2.65638550	-0.77495673
H	-1.90728074	2.76000937	0.75339672
H	1.38511039	1.55476037	-0.97176027
C	4.13170231	0.81291430	-0.12276880
H	4.71105116	0.69471142	0.79405512
H	4.74274714	0.43820001	-0.94662164
H	4.00468662	1.87639987	-0.28650819
H	-1.51698903	-2.00962437	0.46089058

Time = 128.00 fs
E(CASSCF/6-31G(d)) = -479.5856585

C	-0.97998220	-0.21683972	-0.44387817
C	0.55549397	-0.36058332	-0.19834191
C	1.14385317	-1.41682775	0.50157462
C	1.58720258	0.60259803	-0.49526613
C	2.79061557	0.10051661	-0.03951695
C	2.52631025	-1.21267440	0.52967697
N	-1.68007405	0.78290123	0.08306160
C	-3.06325022	0.47914060	0.35416442
H	3.29537923	-1.91592653	0.79431841
C	-1.85449249	-1.44630548	-0.41181319
C	-3.28116697	-0.91125052	-0.23576852
H	-3.73825632	-0.83477521	-1.21422868
H	-3.91517643	-1.51183599	0.40135465
H	-3.27936904	0.44809500	1.43213497
H	-3.76942825	1.20620478	-0.05732969
H	-1.65582235	-2.06377635	-1.27782312
H	0.56195378	-2.27981540	0.76846549
C	-1.19772103	2.16778631	0.19164563
H	-0.26936475	2.25535084	0.72258654
H	-1.13736590	2.65560873	-0.77228292
H	-1.90380799	2.75827223	0.76165609
H	1.38002314	1.54909791	-0.98104888
C	4.13354729	0.81199158	-0.12380742
H	4.70793912	0.69820973	0.79701270
H	4.74145253	0.43995867	-0.95174005
H	4.00573714	1.87522111	-0.28848813
H	-1.52034656	-2.00984689	0.45560688

Time = 128.50 fs
E(CASSCF/6-31G(d)) = -479.5856652

C	-0.97734696	-0.21207680	-0.44511641
C	0.55000578	-0.36165288	-0.20345107
C	1.14516332	-1.41430767	0.50724661
C	1.59011222	0.60074357	-0.50003625
C	2.79140582	0.10103343	-0.04034935
C	2.52441237	-1.21090027	0.53522974
N	-1.67765212	0.77833240	0.08236998
C	-3.06243896	0.47983009	0.35834233
H	3.29929926	-1.91123428	0.80359974
C	-1.85700524	-1.44625165	-0.41603771
C	-3.28105633	-0.91095943	-0.23769877
H	-3.74417736	-0.83043763	-1.21308203
H	-3.91095572	-1.51606079	0.39962845
H	-3.28053063	0.43955283	1.43519138
H	-3.77243614	1.20226448	-0.05345068

H	-1.66117785	-2.06641666	-1.28155728
H	0.55825926	-2.27641982	0.77676016
C	-1.19908506	2.16829152	0.19536892
H	-0.26648847	2.24704454	0.72016428
H	-1.14313148	2.65410495	-0.77016267
H	-1.90097299	2.75583034	0.77006400
H	1.37594137	1.54288812	-0.99005433
C	4.13534567	0.81097916	-0.12484983
H	4.70468730	0.70154343	0.79956919
H	4.74013126	0.44167242	-0.95644563
H	4.00651859	1.87422063	-0.29049843
H	-1.52413901	-2.01031280	0.45037742

Time = 129.00 fs
E(CASSCF/6-31G(d)) = -479.5854630

C	-0.97467235	-0.20747515	-0.44632027
C	0.54463034	-0.36239111	-0.20871833
C	1.14632276	-1.41191708	0.51300404
C	1.59259591	0.59884820	-0.50494182
C	2.79236232	0.10149754	-0.04111684
C	2.52259983	-1.20899519	0.54078635
N	-1.67513305	0.77402544	0.08177208
C	-3.06187953	0.48043284	0.36263820
H	3.30257403	-1.90655195	0.81302848
C	-1.85937280	-1.44616417	-0.42029556
C	-3.28089159	-0.91067731	-0.23975369
H	-3.75052430	-0.82610104	-1.21187900
H	-3.90659912	-1.52057367	0.39770403
H	-3.28048739	0.43167384	1.43761039
H	-3.77403759	1.19850453	-0.04967138
H	-1.66724170	-2.06926145	-1.28539023
H	0.55599921	-2.27346867	0.78566718
C	-1.20055150	2.16881198	0.19918142
H	-0.26294304	2.23797415	0.71726393
H	-1.14841121	2.65192505	-0.76853400
H	-1.89886307	2.75272582	0.77869332
H	1.37297629	1.53622085	-0.99880189
C	4.13708613	0.80988757	-0.12589695
H	4.70138038	0.70465279	0.80165029
H	4.73884053	0.44329320	-0.96063808
H	4.00701862	1.87339198	-0.29253384
H	-1.52833015	-2.01103545	0.44526201

Time = 129.50 fs
E(CASSCF/6-31G(d)) = -479.5851129

C	-0.97198129	-0.20312020	-0.44752725
C	0.53945235	-0.36280945	-0.21411418
C	1.14728124	-1.40964711	0.51883500
C	1.59462634	0.59689034	-0.50998511
C	2.79347493	0.10190476	-0.04181840
C	2.52090494	-1.20695912	0.54634610
N	-1.67256066	0.77005717	0.08131030
C	-3.06155811	0.48090479	0.36702588
H	3.30498887	-1.90176978	0.82255748
C	-1.86157710	-1.44602958	-0.42460063
C	-3.28068421	-0.91039712	-0.24192197
H	-3.75723753	-0.82178241	-1.21062577
H	-3.90213294	-1.52532762	0.39557391
H	-3.27919944	0.42454581	1.43948996
H	-3.77422188	1.19506924	-0.04597922
H	-1.67397691	-2.07221676	-1.28929607
H	0.55530051	-2.27084173	0.79516242
C	-1.20209759	2.16931228	0.20306687
H	-0.25879725	2.22828162	0.71387340
H	-1.15321732	2.64912577	-0.76730882
H	-1.89753926	2.74901136	0.78760749
H	1.37120781	1.52923736	-1.00734655
C	4.13875606	0.80873001	-0.12695019
H	4.69811349	0.70748133	0.80321022
H	4.73764701	0.44476832	-0.96424329
H	4.00723193	1.87270684	-0.29458595
H	-1.53287877	-2.01202271	0.44031200

Time = 130.00 fs
E(CASSCF/6-31G(d)) = -479.5846833

C	-0.96929613	-0.19909149	-0.44877413
C	0.53455237	-0.36292583	-0.21960711
C	1.14799429	-1.40748357	0.52472594
C	1.59618877	0.59484590	-0.51516470
C	2.79472761	0.10225235	-0.04245500
C	2.51935487	-1.20478986	0.55190756
N	-1.66997943	0.76649729	0.08102554
C	-3.06145267	0.48120330	0.37147536
H	3.30637523	-1.89681113	0.83214939
C	-1.86360301	-1.44583672	-0.42896580
C	-3.28044697	-0.91011208	-0.24419154
H	-3.76424862	-0.81749830	-1.20932439
H	-3.89758028	-1.53027150	0.39322982
H	-3.27666275	0.41823937	1.44098721
H	-3.77303281	1.19212361	-0.04237569
H	-1.68133597	-2.07518801	-1.29324895

H	0.55623913	-2.26844852	0.80522269
C	-1.20369812	2.16975806	0.20700905
H	-0.25416246	2.21812212	0.70997850
H	-1.15757367	2.64577028	-0.76637801
H	-1.89702916	2.74474356	0.79685329
H	1.37068363	1.52212153	-1.01576980
C	4.14034181	0.80752178	-0.12801140
H	4.69498965	0.70997717	0.80423196
H	4.73662529	0.44604153	-0.96721485
H	4.00716055	1.87211840	-0.29664373
H	-1.53774023	-2.01327447	0.43556517

Time = 130.50 fs
E(CASSCF/6-31G(d)) = -479.5842411

C	-0.96663917	-0.19546096	-0.45009568
C	0.53000576	-0.36276414	-0.22516406
C	1.14842405	-1.40540660	0.53066155
C	1.59728061	0.59268934	-0.52047593
C	2.79609886	0.10253879	-0.04302943
C	2.51797044	-1.20248224	0.55746817
N	-1.66743300	0.76340651	0.08095596
C	-3.06153410	0.48128842	0.37595358
H	3.30662153	-1.89164089	0.84178104
C	-1.86543831	-1.44557650	-0.43340223
C	-3.28019349	-0.90981559	-0.24654976
H	-3.77148227	-0.81326425	-1.20797382
H	-3.89296196	-1.53535204	0.39066409
H	-3.27290727	0.41280639	1.44230870
H	-3.77056283	1.18984485	-0.03887276
H	-1.68926063	-2.07808392	-1.29722667
H	0.55883518	-2.26623447	0.81582883
C	-1.20532667	2.17011682	0.21099250
H	-0.24918488	2.20766214	0.70556703
H	-1.16151556	2.64192986	-0.76561783
H	-1.89732129	2.73997747	0.80645565
H	1.37142108	1.51508582	-1.02417291
C	4.14182915	0.80627964	-0.12908266
H	4.69211613	0.71209455	0.80472548
H	4.73585537	0.44705474	-0.96953364
H	4.00681356	1.87156644	-0.29869449
H	-1.54286798	-2.01478042	0.43104213

Time = 131.00 fs
E(CASSCF/6-31G(d)) = -479.5838511

C	-0.96403320	-0.19229101	-0.45152341

C	0.52588136	-0.36235357	-0.23075121
C	1.14854075	-1.40339117	0.53662499
C	1.59791129	0.59039520	-0.52591135
C	2.79756214	0.10276366	-0.04354623
C	2.51676501	-1.20002778	0.56302409
N	-1.66496282	0.76083475	0.08113579
C	-3.06176760	0.48112418	0.38042549
H	3.30568065	-1.88626925	0.85144568
C	-1.86707379	-1.44524202	-0.43791867
C	-3.27993788	-0.90950132	-0.24898343
H	-3.77885778	-0.80909441	-1.20657151
H	-3.88829767	-1.54051490	0.38787051
H	-3.26799402	0.40827934	1.44369530
H	-3.76694357	1.18841116	-0.03548779
H	-1.69768224	-2.08082040	-1.30121437
H	0.56304933	-2.26418363	0.82696652
C	-1.20695706	2.17035853	0.21500322
H	-0.24403543	2.19707689	0.70063441
H	-1.16508871	2.63768457	-0.76489739
H	-1.89836245	2.73476184	0.81641247
H	1.37340958	1.50835472	-1.03266901
C	4.14320381	0.80502099	-0.13016614
H	4.68960018	0.71379483	0.80472411
H	4.73541928	0.44774998	-0.97120590
H	4.00620687	1.87098343	-0.30072490
H	-1.54821450	-2.01651915	0.42674334

Time = 131.50 fs
E(CASSCF/6-31G(d)) = -479.5835688

C	-0.96150192	-0.18963298	-0.45308446
C	0.52224045	-0.36172782	-0.23633446
C	1.14832324	-1.40140757	0.54259806
C	1.59810117	0.58793985	-0.53146171
C	2.79908671	0.10292738	-0.04401143
C	2.51574389	-1.19741473	0.56857007
N	-1.66260675	0.75881964	0.08159436
C	-3.06211454	0.48067991	0.38485535
H	3.30357188	-1.88075217	0.86115418
C	-1.86850315	-1.44482837	-0.44252111
C	-3.27969435	-0.90916307	-0.25147899
H	-3.78629135	-0.80500094	-1.20511526
H	-3.88360712	-1.54570651	0.38484555
H	-3.26201053	0.40467171	1.44540313
H	-3.76233375	1.18798906	-0.03223717
H	-1.70652231	-2.08332349	-1.30520706
H	0.56878460	-2.26231722	0.83862680

C	-1.20856472	2.17045605	0.21902911
H	-0.23889669	2.18654734	0.69518902
H	-1.16834828	2.63312444	-0.76408631
H	-1.90005780	2.72913679	0.82669298
H	1.37661528	1.50214467	-1.04137224
C	4.14445220	0.80376296	-0.13126388
H	4.68754405	0.71504768	0.80427944
H	4.73539698	0.44807179	-0.97225973
H	4.00536254	1.87030125	-0.30272219
H	-1.55373215	-2.01845803	0.42264812

Time = 132.00 fs
E(CASSCF/6-31G(d)) = -479.5834474

C	-0.95907019	-0.18752610	-0.45480071
C	0.51913570	-0.36092433	-0.24187990
C	1.14775893	-1.39942236	0.54856140
C	1.59788003	0.58530346	-0.53711701
C	2.80063884	0.10303076	-0.04443224
C	2.51490455	-1.19462831	0.57409948
N	-1.66039789	0.75738541	0.08235501
C	-3.06253448	0.47993149	0.38920827
H	3.30037851	-1.87518753	0.87093534
C	-1.86972294	-1.44433232	-0.44721250
C	-3.27947665	-0.90879484	-0.25402261
H	-3.79369829	-0.80099347	-1.20360532
H	-3.87891128	-1.55087571	0.38158962
H	-3.25506536	0.40197943	1.44768107
H	-3.75690539	1.18871962	-0.02912909
H	-1.71569330	-2.08553138	-1.30921123
H	0.57589145	-2.26068819	0.85080554
C	-1.21012779	2.17038540	0.22306012
H	-0.23394888	2.17625673	0.68925723
H	-1.17135754	2.62834998	-0.76306212
H	-1.90227439	2.72313388	0.83723850
H	1.38098643	1.49664260	-1.05038630
C	4.14556210	0.80252156	-0.13237762
H	4.68603986	0.71583184	0.80345496
H	4.73586174	0.44796986	-0.97274028
H	4.00430807	1.86945757	-0.30467530
H	-1.55937330	-2.02055390	0.41871548

Time = 132.50 fs
E(CASSCF/6-31G(d)) = -479.5835250

C	-0.95676412	-0.18599665	-0.45668810
C	0.51661028	-0.35998331	-0.24735428

C	1.14684381	-1.39739963	0.55449497
C	1.59728548	0.58247182	-0.54286766
C	2.80218293	0.10307474	-0.04481668
C	2.51423692	-1.19165147	0.57960455
N	-1.65836358	0.75654213	0.08343451
C	-3.06298723	0.47886249	0.39345186
H	3.29624051	-1.86970792	0.88083415
C	-1.87073247	-1.44375206	-0.45199282
C	-3.27929772	-0.90839081	-0.25660020
H	-3.80099542	-0.79707857	-1.20204626
H	-3.87423325	-1.55597526	0.37810795
H	-3.24728209	0.40018293	1.45074588
H	-3.75082924	1.19070444	-0.02615633
H	-1.72510004	-2.08739562	-1.31324547
H	0.58417658	-2.25937224	0.86350122
C	-1.21162816	2.17012585	0.22708825
H	-0.22935604	2.16638644	0.68288708
H	-1.17418629	2.62347208	-0.76171686
H	-1.90484761	2.71677793	0.84796594
H	1.38645880	1.49198557	-1.05979271
C	4.14652339	0.80131081	-0.13350862
H	4.68516435	0.71613576	0.80231907
H	4.73687530	0.44740167	-0.97270411
H	4.00307549	1.86840189	-0.30657596
H	-1.56509030	-2.02275523	0.41488655

Time = 133.00 fs
E(CASSCF/6-31G(d)) = -479.5838284

C	-0.95461090	-0.18505751	-0.45875626
C	0.51469711	-0.35894678	-0.25272554
C	1.14558192	-1.39530263	0.56037862
C	1.59636114	0.57943797	-0.54870559
C	2.80368289	0.10306005	-0.04517320
C	2.51372420	-1.18846587	0.58507667
N	-1.65652469	0.75628534	0.08484249
C	-3.06343484	0.47746516	0.39755795
H	3.29134263	-1.86446974	0.89090844
C	-1.87153374	-1.44308702	-0.45685928
C	-3.27916921	-0.90794534	-0.25919763
H	-3.80810330	-0.79325932	-1.20044862
H	-3.86959896	-1.56096300	0.37441113
H	-3.23879329	0.39924949	1.45475796
H	-3.74426150	1.19399308	-0.02328982
H	-1.73464114	-2.08888127	-1.31733999
H	0.59341470	-2.25845544	0.87671138
C	-1.21305192	2.16965995	0.23110735

H	-0.22525368	2.15711155	0.67615047
H	-1.17690927	2.61861120	-0.75996249
H	-1.90759034	2.71009069	0.85877385
H	1.39296012	1.48824367	-1.06964061
C	4.14732876	0.80014198	-0.13465748
H	4.68497421	0.71595795	0.80093807
H	4.73848306	0.44633508	-0.97221323
H	4.00170052	1.86710031	-0.30841960
H	-1.57083506	-2.02500495	0.41108835

Time = 133.50 fs
E(CASSCF/6-31G(d)) = -479.5843668

C	-0.95263837	-0.18470822	-0.46100834
C	0.51341832	-0.35785753	-0.25796313
C	1.14398464	-1.39309550	0.56619284
C	1.59515510	0.57620325	-0.55462516
C	2.80510337	0.10298699	-0.04551044
C	2.51334411	-1.18505328	0.59050694
N	-1.65489527	0.75659610	0.08658123
C	-3.06384313	0.47574108	0.40150412
H	3.28589818	-1.85963901	0.90122391
C	-1.87213140	-1.44233753	-0.46180673
C	-3.27910121	-0.90745302	-0.26180071
H	-3.81494828	-0.78953500	-1.19883011
H	-3.86503760	-1.56580281	0.37051550
H	-3.22973543	0.39913543	1.45980071
H	-3.73733333	1.19857329	-0.02047434
H	-1.74421081	-2.08996606	-1.32153505
H	0.60336244	-2.25801903	0.89042774
C	-1.21438963	2.16897351	0.23511278
H	-0.22173862	2.14859634	0.66914346
H	-1.17960440	2.61389564	-0.75773461
H	-1.91030376	2.70309540	0.86955019
H	1.40041299	1.48540905	-1.07993974
C	4.14797427	0.79902308	-0.13582410
H	4.68550197	0.71530700	0.79936972
H	4.74070981	0.44475057	-0.97132920
H	4.00022163	1.86553898	-0.31020605
H	-1.57655826	-2.02724381	0.40723876

Time = 134.00 fs
E(CASSCF/6-31G(d)) = -479.5851268

C	-0.95087443	-0.18493540	-0.46344110
C	0.51278488	-0.35675818	-0.26303849
C	1.14206963	-1.39074523	0.57191958

C	1.59371847	0.57277768	-0.56062363
C	2.80641091	0.10285537	-0.04583690
C	2.51307046	-1.18139725	0.59588685
N	-1.65348242	0.75744151	0.08864567
C	-3.06418271	0.47370146	0.40527484
H	3.28012952	-1.85537460	0.91184737
C	-1.87253263	-1.44150463	-0.46682829
C	-3.27910193	-0.90690879	-0.26439543
H	-3.82146425	-0.78590079	-1.19721620
H	-3.86058160	-1.57046503	0.36644298
H	-3.22024492	0.39978754	1.46586666
H	-3.73014471	1.20436585	-0.01762710
H	-1.75370075	-2.09063869	-1.32587847
H	0.61377314	-2.25812279	0.90463025
C	-1.21563616	2.16805560	0.23910093
H	-0.21886200	2.14098985	0.66198472
H	-1.18235110	2.60945912	-0.75499446
H	-1.91278898	2.69582161	0.88018127
H	1.40873577	1.48339274	-1.09065768
C	4.14845977	0.79795840	-0.13700760
H	4.68675316	0.71420144	0.79765828
H	4.74355632	0.44264299	-0.97010805
H	3.99867926	1.86372593	-0.31193992
H	-1.58220852	-2.02941417	0.40325207

Time = 134.50 fs
E(CASSCF/6-31G(d)) = -479.5860694

C	-0.94934614	-0.18571356	-0.46604518
C	0.51279643	-0.35569041	-0.26792537
C	1.13985978	-1.38822342	0.57754321
C	1.59210439	0.56917953	-0.56670130
C	2.80757481	0.10266459	-0.04616089
C	2.51287487	-1.17748479	0.60120908
N	-1.65228652	0.75877554	0.09102366
C	-3.06442944	0.47136702	0.40886216
H	3.27424676	-1.85181009	0.92283863
C	-1.87274710	-1.44058998	-0.47191600
C	-3.27917755	-0.90630803	-0.26696803
H	-3.82759395	-0.78234778	-1.19564004
H	-3.85626638	-1.57492652	0.36222065
H	-3.21045588	0.40114366	1.47285309
H	-3.72276335	1.21122464	-0.01463980
H	-1.76300216	-2.09089645	-1.33042241
H	0.62441080	-2.25878881	0.91928078
C	-1.21679023	2.16689853	0.24306873
H	-0.21662593	2.13442200	0.65481217

H	-1.18522865	2.60543749	-0.75172887
H	-1.91485850	2.68830960	0.89056072
H	1.41784162	1.48203025	-1.10172194
C	4.14878909	0.79694837	-0.13820636
H	4.68870489	0.71266931	0.79583127
H	4.74699717	0.44002268	-0.96859656
H	3.99711509	1.86169114	-0.31363065
H	-1.58773165	-2.03146361	0.39904466

Time = 135.00 fs
E(CASSCF/6-31G(d)) = -479.5871309

C	-0.94807875	-0.18700628	-0.46880558
C	0.51344129	-0.35469428	-0.27260012
C	1.13738201	-1.38550781	0.58305123
C	1.59036735	0.56543419	-0.57286111
C	2.80856793	0.10241382	-0.04649040
C	2.51272865	-1.17330815	0.60646835
N	-1.65130170	0.76054033	0.09369645
C	-3.06456420	0.46876738	0.41226569
H	3.26842737	-1.84903635	0.93424209
C	-1.87278689	-1.43959566	-0.47706159
C	-3.27933208	-0.90564669	-0.26950524
H	-3.83328990	-0.77886302	-1.19414176
H	-3.85212966	-1.57917036	0.35787988
H	-3.20049934	0.40313215	1.48056802
H	-3.71522857	1.21894263	-0.01138432
H	-1.77200761	-2.09074230	-1.33521964
H	0.63506145	-2.25998796	0.93431746
C	-1.21785369	2.16549801	0.24701327
H	-0.21498382	2.12900025	0.64777800
H	-1.18831468	2.60196503	-0.74794852
H	-1.91634656	2.68061376	0.90059807
H	1.42763611	1.48109512	-1.11302685
C	4.14897001	0.79598960	-0.13941812
H	4.69130600	0.71074747	0.79389847
H	4.75098010	0.43691567	-0.96683007
H	3.99557143	1.85948485	-0.31529229
H	-1.59307006	-2.03334842	0.39454029

Time = 135.50 fs
E(CASSCF/6-31G(d)) = -479.5882294

C	-0.94709460	-0.18876759	-0.47170224
C	0.51469677	-0.35380770	-0.27704197
C	1.13466630	-1.38258320	0.58843499
C	1.58856273	0.56157248	-0.57910803

C	2.80936717	0.10210225	-0.04683311
C	2.51260443	-1.16886614	0.61166218
N	-1.65051659	0.76266766	0.09663933
C	-3.06457225	0.46594000	0.41549206
H	3.26279922	-1.84708685	0.94607873
C	-1.87266638	-1.43852424	-0.48225716
C	-3.27956744	-0.90492145	-0.27199439
H	-3.83851464	-0.77542981	-1.19276701
H	-3.84821059	-1.58318506	0.35345522
H	-3.19050339	0.40567086	1.48874608
H	-3.70755916	1.22726309	-0.00772119
H	-1.78061301	-2.09018185	-1.34031981
H	0.64554057	-2.26163146	0.94965063
C	-1.21883059	2.16385320	0.25093129
H	-0.21384491	2.12480722	0.64104246
H	-1.19168365	2.59917018	-0.74368425
H	-1.91711786	2.67280467	0.91022590
H	1.43801454	1.48031862	-1.12444398
C	4.14901396	0.79507519	-0.14064012
H	4.69447888	0.70848067	0.79185324
H	4.75542702	0.43336301	-0.96483229
H	3.99409077	1.85717419	-0.31694287
H	-1.59816256	-2.03503645	0.38967483

Time = 136.00 fs
E(CASSCF/6-31G(d)) = -479.5892753

C	-0.94641201	-0.19094355	-0.47471084
C	0.51652958	-0.35306617	-0.28123322
C	1.13174485	-1.37944179	0.59368993
C	1.58674637	0.55762855	-0.58544808
C	2.80995376	0.10172939	-0.04719641
C	2.51247740	-1.16416508	0.61679149
N	-1.64991512	0.76508078	0.09982252
C	-3.06444227	0.46292871	0.41855381
H	3.25742965	-1.84592810	0.95834008
C	-1.87240211	-1.43737878	-0.48749581
C	-3.27988345	-0.90412985	-0.27442371
H	-3.84324066	-0.77202814	-1.19156493
H	-3.84454864	-1.58696347	0.34898294
H	-3.18059378	0.40866570	1.49707184
H	-3.69976368	1.23589485	-0.00350997
H	-1.78871928	-2.08922039	-1.34576575
H	0.65569571	-2.26356942	0.96516146
C	-1.21972613	2.16196696	0.25481896
H	-0.21308257	2.12189920	0.63476654
H	-1.19540551	2.59717103	-0.73898207

H	-1.91707411	2.66496962	0.91940536
H	1.44885965	1.47941289	-1.13583490
C	4.14893547	0.79419522	-0.14186927
H	4.69812283	0.70592043	0.78967591
H	4.76023677	0.42941914	-0.96261696
H	3.99271544	1.85483822	-0.31860352
H	-1.60294456	-2.03650938	0.38439988

Time = 136.50 fs
E(CASSCF/6-31G(d)) = -479.5901841

C	-0.94604431	-0.19347399	-0.47780355
C	0.51889654	-0.35250243	-0.28515930
C	1.12865163	-1.37608276	0.59881565
C	1.58497424	0.55363776	-0.59188731
C	2.81031354	0.10129533	-0.04758744
C	2.51232594	-1.15921903	0.62186087
N	-1.64947746	0.76769637	0.10321201
C	-3.06416534	0.45978202	0.42146788
H	3.25232261	-1.84545695	0.97098521
C	-1.87201266	-1.43616285	-0.49277215
C	-3.28027809	-0.90327044	-0.27678242
H	-3.84744969	-0.76863530	-1.19058571
H	-3.84118241	-1.59050156	0.34449947
H	-3.17089428	0.41200976	1.50520731
H	-3.69185147	1.24452995	0.00137982
H	-1.79623390	-2.08786040	-1.35159051
H	0.66540456	-2.26559686	0.98070385
C	-1.22054542	2.15984595	0.25867166
H	-0.21254598	2.12030555	0.62910412
H	-1.19954432	2.59607078	-0.73389696
H	-1.91615807	2.65721140	0.92812950
H	1.46004052	1.47809482	-1.14706448
C	4.14875140	0.79333751	-0.14310241
H	4.70211863	0.70312370	0.78733871
H	4.76528929	0.42514942	-0.96019104
H	3.99148742	1.85256185	-0.32029727
H	-1.60734872	-2.03776412	0.37868523

Time = 137.00 fs
E(CASSCF/6-31G(d)) = -479.5908898

C	-0.94599902	-0.19629409	-0.48094990
C	0.52174557	-0.35214628	-0.28880891
C	1.12542205	-1.37251125	0.60381551
C	1.58330187	0.54963451	-0.59843086
C	2.81043693	0.10080094	-0.04801306

C	2.51213162	-1.15404935	0.62687857
N	-1.64918099	0.77042654	0.10677062
C	-3.06373401	0.45655118	0.42425411
H	3.24742406	-1.84550544	0.98394148
C	-1.87151841	-1.43488068	-0.49808263
C	-3.28074764	-0.90234282	-0.27906099
H	-3.85113166	-0.76522663	-1.18987776
H	-3.83814834	-1.59379693	0.34003974
H	-3.16152644	0.41558303	1.51282043
H	-3.68384286	1.25286213	0.00705838
H	-1.80307222	-2.08609970	-1.35781514
H	0.67456934	-2.26746674	0.99610963
C	-1.22129235	2.15750095	0.26248396
H	-0.21207459	2.12002901	0.62419388
H	-1.20415693	2.59595325	-0.72848617
H	-1.91435491	2.64964561	0.93642427
H	1.47141289	1.47610824	-1.15801365
C	4.14848005	0.79248849	-0.14433646
H	4.70633414	0.70015125	0.78481188
H	4.77045108	0.42062702	-0.95755903
H	3.99044818	1.85042884	-0.32204774
H	-1.61130605	-2.03881325	0.37251993

Time = 137.50 fs
E(CASSCF/6-31G(d)) = -479.5913549

C	-0.94627728	-0.19933606	-0.48411758
C	0.52501683	-0.35202437	-0.29217402
C	1.12209287	-1.36873683	0.60869606
C	1.58178368	0.54565035	-0.60508202
C	2.81031898	0.10024805	-0.04847990
C	2.51187863	-1.14868353	0.63185603
N	-1.64900113	0.77318086	0.11045888
C	-3.06314152	0.45328816	0.42693356
H	3.24263454	-1.84585260	0.99710899
C	-1.87094131	-1.43353716	-0.50342564
C	-3.28128699	-0.90134776	-0.28125130
H	-3.85428329	-0.76177643	-1.18948475
H	-3.83547951	-1.59684742	0.33563552
H	-3.15260854	0.41925321	1.51961130
H	-3.67577742	1.26060438	0.01360688
H	-1.80915851	-2.08393044	-1.36444753
H	0.68310903	-2.26890819	1.01119655
C	-1.22196850	2.15494694	0.26624976
H	-0.21151403	2.12104659	0.62015167
H	-1.20929180	2.59687885	-0.72280251
H	-1.91169077	2.64239628	0.94434701

H	1.48282120	1.47324287	-1.16859031
C	4.14814013	0.79163409	-0.14556868
H	4.71063040	0.69706609	0.78206998
H	4.77558140	0.41592924	-0.95472781
H	3.98963865	1.84851468	-0.32387771
H	-1.61474737	-2.03968455	0.36591198

Time = 138.00 fs
E(CASSCF/6-31G(d)) = -479.5915759

C	-0.94687357	-0.20253060	-0.48727322
C	0.52864415	-0.35215997	-0.29524987
C	1.11870213	-1.36477174	0.61346621
C	1.58047220	0.54171235	-0.61184153
C	2.80995919	0.09963954	-0.04899428
C	2.51155281	-1.14315364	0.63680725
N	-1.64891216	0.77586840	0.11423604
C	-3.06238140	0.45004371	0.42952706
H	3.23782664	-1.84624141	1.01036813
C	-1.87030459	-1.43213795	-0.50880145
C	-3.28188995	-0.90028717	-0.28334674
H	-3.85690661	-0.75825883	-1.18944276
H	-3.83320457	-1.59964976	0.33131404
H	-3.14425382	0.42287742	1.52533443
H	-3.66771971	1.26750462	0.02107080
H	-1.81442689	-2.08133906	-1.37148246
H	0.69095104	-2.26964739	1.02577821
C	-1.22257219	2.15220337	0.26996262
H	-0.21073195	2.12331096	0.61706368
H	-1.21498776	2.59888104	-0.71688837
H	-1.90822864	2.63559015	0.95198238
H	1.49410174	1.46934905	-1.17873807
C	4.14774980	0.79076079	-0.14679684
H	4.71486780	0.69393165	0.77909799
H	4.78053889	0.41113377	-0.95171151
H	3.98909899	1.84687977	-0.32580766
H	-1.61760519	-2.04041946	0.35888652

Time = 138.50 fs
E(CASSCF/6-31G(d)) = -479.5915812

C	-0.94777575	-0.20580829	-0.49038308
C	0.53255665	-0.35257266	-0.29803494
C	1.11528905	-1.36062911	0.61813632
C	1.57941704	0.53784180	-0.61870702
C	2.80936138	0.09897936	-0.04956223
C	2.51114046	-1.13749436	0.64174783

N	-1.64888790	0.77839961	0.11806093
C	-3.06144717	0.44686557	0.43205397
H	3.23286441	-1.84639874	1.02358918
C	-1.86963242	-1.43068949	-0.51421196
C	-3.28254949	-0.89916409	-0.28534241
H	-3.85900734	-0.75464872	-1.18977777
H	-3.83134691	-1.60219852	0.32709671
H	-3.13656815	0.42630483	1.52981594
H	-3.65976232	1.27335862	0.02945577
H	-1.81882205	-2.07830719	-1.37890274
H	0.69802380	-2.26942874	1.03967484
C	-1.22309805	2.14929422	0.27361625
H	-0.20963200	2.12675198	0.61498134
H	-1.22127297	2.60196382	-0.71077097
H	-1.90406158	2.62934968	0.95943583
H	1.50508692	1.46434782	-1.18844228
C	4.14732567	0.78985644	-0.14801937
H	4.71891205	0.69081006	0.77589642
H	4.78518794	0.40631484	-0.94853567
H	3.98886830	1.84556373	-0.32785459
H	-1.61981570	-2.04107062	0.35148275

Time = 139.00 fs
E(CASSCF/6-31G(d)) = -479.5914246

C	-0.94896537	-0.20910076	-0.49341366
C	0.53668041	-0.35327808	-0.30053096
C	1.11189374	-1.35632129	0.62271728
C	1.57866387	0.53405323	-0.62567266
C	2.80853341	0.09827250	-0.05018936
C	2.51062731	-1.13174105	0.64669402
N	-1.64890227	0.78068811	0.12189274
C	-3.06033234	0.44379680	0.43453109
H	3.22762223	-1.84605567	1.03664285
C	-1.86894954	-1.42919902	-0.51966019
C	-3.28325804	-0.89798258	-0.28723512
H	-3.86059353	-0.75092266	-1.19050358
H	-3.82992409	-1.60448537	0.32299842
H	-3.12964740	0.42937987	1.53296525
H	-3.65202616	1.27801969	0.03872578
H	-1.82229983	-2.07481324	-1.38668134
H	0.70425085	-2.26803390	1.05272374
C	-1.22353686	2.14624813	0.27720513
H	-0.20816414	2.13127835	0.61391808
H	-1.22816396	2.60610010	-0.70445948
H	-1.89930386	2.62378519	0.96682493
H	1.51561002	1.45823624	-1.19773348

C	4.14688204	0.78891100	-0.14923546
H	4.72263949	0.68776042	0.77248524
H	4.78940467	0.40153983	-0.94524051
H	3.98898405	1.84458140	-0.33003104
H	-1.62132106	-2.04169854	0.34374973

Time = 139.50 fs
E(CASSCF/6-31G(d)) = -479.5911734

C	-0.95041822	-0.21234181	-0.49633225
C	0.54094028	-0.35428750	-0.30274282
C	1.10855686	-1.35185856	0.62721971
C	1.57825336	0.53035384	-0.63272907
C	2.80748692	0.09752495	-0.05088084
C	2.50999765	-1.12592783	0.65166172
N	-1.64892975	0.78265238	0.12569184
C	-3.05903057	0.44087445	0.43697186
H	3.22200106	-1.84496631	1.04941037
C	-1.86828078	-1.42767454	-0.52514979
C	-3.28400773	-0.89674763	-0.28902349
H	-3.86167441	-0.74705965	-1.19162050
H	-3.82894758	-1.60649875	0.31902706
H	-3.12357482	0.43194585	1.53478142
H	-3.64465848	1.28140497	0.04880378
H	-1.82482771	-2.07083458	-1.39478425
H	0.70954718	-2.26529748	1.06478870
C	-1.22387612	2.14309828	0.28072531
H	-0.20632983	2.13677916	0.61384855
H	-1.23566484	2.61123148	-0.69794418
H	-1.89408055	2.61898655	0.97426911
H	1.52551021	1.45108787	-1.20668788
C	4.14643024	0.78791704	-0.15044509
H	4.72594162	0.68483728	0.76890601
H	4.79308203	0.39686623	-0.94188275
H	3.98948129	1.84392105	-0.33234445
H	-1.62207173	-2.04236769	0.33574124

Time = 140.00 fs
E(CASSCF/6-31G(d)) = -479.5908972

C	-0.95210512	-0.21546839	-0.49910743
C	0.54526175	-0.35560749	-0.30467856
C	1.10531902	-1.34724813	0.63165326
C	1.57822004	0.52674335	-0.63986339
C	2.80623701	0.09674351	-0.05164125
C	2.50923376	-1.12008592	0.65666554
N	-1.64894580	0.78421733	0.12942038

C	-3.05753591	0.43812855	0.43938578
H	3.21594076	-1.84292398	1.06179272
C	-1.86765062	-1.42612478	-0.53068433
C	-3.28479053	-0.89546494	-0.29070789
H	-3.86225966	-0.74304163	-1.19311476
H	-3.82842287	-1.60822396	0.31518372
H	-3.11841847	0.43384868	1.53535412
H	-3.63782849	1.28349790	0.05957457
H	-1.82638527	-2.06635011	-1.40317370
H	0.71381782	-2.26111827	1.07576765
C	-1.22410096	2.13988223	0.28417513
H	-0.20418139	2.14312553	0.61471015
H	-1.24376692	2.61726925	-0.69119775
H	-1.88851657	2.61501499	0.98187866
H	1.53463755	1.44304805	-1.21542472
C	4.14597822	0.78687003	-0.15164906
H	4.72872846	0.68208931	0.76522235
H	4.79613377	0.39233938	-0.93853613
H	3.99039139	1.84354494	-0.33479703
H	-1.62202878	-2.04314220	0.32751042

Time = 140.50 fs
E(CASSCF/6-31G(d)) = -479.5906579

C	-0.95399269	-0.21842140	-0.50170937
C	0.54957239	-0.35723960	-0.30634927
C	1.10222027	-1.34249355	0.63602609
C	1.57859148	0.52321432	-0.64705961
C	2.80480189	0.09593582	-0.05247458
C	2.50831566	-1.11424242	0.66171813
N	-1.64892717	0.78531562	0.13304297
C	-3.05584322	0.43558141	0.44177811
H	3.20942863	-1.83977266	1.07371775
C	-1.86708269	-1.42455914	-0.53626666
C	-3.28559837	-0.89414079	-0.29229045
H	-3.86235911	-0.73885408	-1.19495904
H	-3.82834984	-1.60964374	0.31146307
H	-3.11422931	0.43494052	1.53485947
H	-3.63172134	1.28434680	0.07088951
H	-1.82696434	-2.06134258	-1.41181170
H	0.71695760	-2.25546626	1.08559837
C	-1.22419556	2.13664146	0.28755599
H	-0.20181524	2.15017234	0.61640694
H	-1.25244849	2.62409697	-0.68417902
H	-1.88272641	2.61189624	0.98974436
H	1.54285684	1.43432438	-1.22410097
C	4.14553044	0.78576828	-0.15284889

H	4.73093063	0.67955797	0.76151836
H	4.79849701	0.38799088	-0.93529005
H	3.99174085	1.84339225	-0.33738600
H	-1.62116585	-2.04408123	0.31910394

Time = 141.00 fs
E(CASSCF/6-31G(d)) = -479.5905055

C	-0.95604436	-0.22114652	-0.50411027
C	0.55380353	-0.35917998	-0.30776907
C	1.09929931	-1.33759456	0.64034454
C	1.57938736	0.51975294	-0.65429927
C	2.80320249	0.09511015	-0.05338413
C	2.50722120	-1.10841936	0.66682953
N	-1.64885212	0.78588894	0.13652717
C	-3.05394863	0.43324747	0.44414990
H	3.20250355	-1.83541558	1.08514579
C	-1.86659925	-1.42298759	-0.54189831
C	-3.28642318	-0.89278183	-0.29377494
H	-3.86198300	-0.73448615	-1.19711367
H	-3.82872340	-1.61073919	0.30785432
H	-3.11103945	0.43508335	1.53355011
H	-3.62652974	1.28405927	0.08257350
H	-1.82656923	-2.05580089	-1.42066305
H	0.71885324	-2.24838441	1.09426172
C	-1.22414479	2.13342067	0.29087289
H	-0.19936051	2.15776053	0.61881527
H	-1.26167521	2.63157413	-0.67683791
H	-1.87680568	2.60961519	0.99792865
H	1.55005161	1.42517112	-1.23290185
C	4.14508788	0.78461265	-0.15404665
H	4.73250025	0.67727679	0.75789529
H	4.80013358	0.38383795	-0.93224695
H	3.99354965	1.84338410	-0.34010420
H	-1.61947109	-2.04523494	0.31055719

Time = 141.50 fs
E(CASSCF/6-31G(d)) = -479.5904792

C	-0.95822130	-0.22359488	-0.50628465
C	0.55789147	-0.36141916	-0.30895498
C	1.09659277	-1.33254733	0.64461304
C	1.58061891	0.51634049	-0.66156243
C	2.80146215	0.09427531	-0.05437245
C	2.50592646	-1.10263307	0.67200682
N	-1.64870073	0.78588910	0.13984405
C	-3.05185014	0.43113352	0.44649833

H	3.19525631	-1.82981919	1.09607294
C	-1.86622066	-1.42142053	-0.54757902
C	-3.28725688	-0.89139485	-0.29516667
H	-3.86114253	-0.72993074	-1.19952867
H	-3.82953437	-1.61149104	0.30434243
H	-3.10886098	0.43415215	1.53173934
H	-3.62244393	1.28279205	0.09443364
H	-1.82521682	-2.04972192	-1.42969805
H	0.71938681	-2.23998552	1.10178225
C	-1.22393582	2.13026668	0.29413476
H	-0.19696411	2.16571989	0.62179076
H	-1.27140075	2.63954104	-0.66912166
H	-1.87082552	2.60811330	1.00645987
H	1.55612691	1.41586853	-1.24202793
C	4.14464837	0.78340611	-0.15524484
H	4.73341052	0.67527072	0.75446653
H	4.80102978	0.37988363	-0.92951812
H	3.99582967	1.84343002	-0.34294110
H	-1.61694857	-2.04664115	0.30189036

Time = 142.00 fs
E(CASSCF/6-31G(d)) = -479.5906090

C	-0.96048345	-0.22572381	-0.50820971
C	0.56177860	-0.36394189	-0.30992687
C	1.09413431	-1.32734511	0.64883433
C	1.58228840	0.51295514	-0.66882897
C	2.79960622	0.09344048	-0.05544124
C	2.50440641	-1.09689407	0.67725386
N	-1.64845496	0.78527895	0.14296861
C	-3.04954833	0.42923940	0.44881750
H	3.18782625	-1.82301321	1.10653191
C	-1.86596490	-1.41986855	-0.55330648
C	-3.28809136	-0.88998666	-0.29647232
H	-3.85985095	-0.72518441	-1.20214649
H	-3.83077043	-1.61188117	0.30090951
H	-3.10768555	0.43203794	1.52977941
H	-3.61964000	1.28073693	0.10626930
H	-1.82293636	-2.04311153	-1.43889421
H	0.71844055	-2.23044424	1.10822607
C	-1.22355956	2.12722707	0.29735441
H	-0.19477427	2.17387250	0.62517611
H	-1.28156771	2.64782452	-0.66098156
H	-1.86483072	2.60728939	1.01533001
H	1.56101111	1.40669741	-1.25167903
C	4.14420714	0.78215307	-0.15644615
H	4.73365416	0.67355590	0.75135117

H	4.80119490	0.37611793	-0.92721842
H	3.99858314	1.84343539	-0.34588395
H	-1.61361917	-2.04832271	0.29310604

Time = 142.50 fs
E(CASSCF/6-31G(d)) = -479.5909344

C	-0.96279034	-0.22749738	-0.50986576
C	0.56541407	-0.36672716	-0.31070725
C	1.09195380	-1.32197937	0.65300976
C	1.58438909	0.50957424	-0.67608016
C	2.79766165	0.09261521	-0.05659139
C	2.50263580	-1.09120730	0.68257154
N	-1.64809892	0.78403324	0.14588032
C	-3.04704708	0.42755921	0.45109969
H	3.18039455	-1.81508600	1.11659079
C	-1.86584710	-1.41834227	-0.55907617
C	-3.28891834	-0.88856385	-0.29769983
H	-3.85812459	-0.72024688	-1.20490504
H	-3.83241705	-1.61189399	0.29753619
H	-3.10748369	0.42865007	1.52803351
H	-3.61826651	1.27810245	0.11788357
H	-1.81976947	-2.03598488	-1.44823749
H	0.71590288	-2.21998394	1.11369587
C	-1.22301185	2.12434843	0.30054821
H	-0.19292429	2.18203696	0.62880824
H	-1.29210981	2.65624443	-0.65237961
H	-1.85884120	2.60700293	1.02449741
H	1.56465699	1.39791156	-1.26203602
C	4.14375749	0.78085864	-0.15765320
H	4.73324049	0.67213976	0.74866635
H	4.80065815	0.37251957	-0.92545975
H	4.00180151	1.84330891	-0.34891909
H	-1.60952115	-2.05028685	0.28418837

Time = 143.00 fs
E(CASSCF/6-31G(d)) = -479.5914883

C	-0.96510183	-0.22888700	-0.51123650
C	0.56875417	-0.36974817	-0.31132121
C	1.09007656	-1.31644129	0.65714008
C	1.58690526	0.50617663	-0.68330019
C	2.79565674	0.09180936	-0.05782305
C	2.50059022	-1.08557290	0.68795806
N	-1.64761893	0.78213908	0.14856341
C	-3.04435448	0.42608275	0.45333691
H	3.17317411	-1.80617562	1.12634890

C	-1.86587917	-1.41685206	-0.56488152
C	-3.28972938	-0.88713271	-0.29885817
H	-3.85598413	-0.71512071	-1.20774116
H	-3.83445855	-1.61151794	0.29420311
H	-3.10820528	0.42391870	1.52684438
H	-3.61843110	1.27509387	0.12909440
H	-1.81576966	-2.02836612	-1.45772230
H	0.71167579	-2.20885851	1.11832273
C	-1.22229405	2.12167449	0.30373538
H	-0.19151809	2.19003291	0.63252566
H	-1.30295346	2.66462012	-0.64329453
H	-1.85285673	2.60708122	1.03389243
H	1.56704202	1.38971009	-1.27324233
C	4.14329166	0.77952784	-0.15886837
H	4.73219207	0.67102123	0.74651920
H	4.79946485	0.36905868	-0.92434421
H	4.00546448	1.84296974	-0.35203319
H	-1.60470980	-2.05252539	0.27510439

Time = 143.50 fs
E(CASSCF/6-31G(d)) = -479.5923076

C	-0.96737878	-0.22987191	-0.51230942
C	0.57176258	-0.37297256	-0.31179625
C	1.08852237	-1.31072362	0.66122633
C	1.58981232	0.50274473	-0.69047763
C	2.79362070	0.09103293	-0.05913549
C	2.49824748	-1.07998723	0.69340943
N	-1.64700358	0.77959635	0.15100721
C	-3.04148368	0.42479704	0.45552276
H	3.16639593	-1.79645692	1.13592984
C	-1.86606949	-1.41540775	-0.57071428
C	-3.29051572	-0.88569910	-0.29995722
H	-3.85345571	-0.70981083	-1.21059387
H	-3.83687882	-1.61074673	0.29089213
H	-3.10977985	0.41779696	1.52650124
H	-3.62018752	1.27189283	0.13974511
H	-1.81100182	-2.02028712	-1.46735044
H	0.70568363	-2.19733018	1.12225537
C	-1.22141330	2.11924426	0.30693713
H	-0.19061848	2.19768590	0.63617481
H	-1.31401986	2.67277689	-0.63372669
H	-1.84686385	2.60732852	1.04342645
H	1.56816834	1.38221421	-1.28538718
C	4.14280166	0.77816488	-0.16009353
H	4.73054075	0.67019131	0.74499869
H	4.79767175	0.36569980	-0.92395703

H	4.00953960	1.84235345	-0.35521437
H	-1.59925657	-2.05501647	0.26580690

Time = 144.00 fs
E(CASSCF/6-31G(d)) = -479.5934207

C	-0.96958350	-0.23043964	-0.51307621
C	0.57441028	-0.37636270	-0.31216206
C	1.08730457	-1.30482271	0.66527095
C	1.59307714	0.49926603	-0.69760656
C	2.79158344	0.09029599	-0.06052718
C	2.49558918	-1.07444430	0.69892032
N	-1.64624388	0.77641783	0.15320646
C	-3.03845359	0.42368773	0.45765420
H	3.16029266	-1.78612438	1.14547178
C	-1.86642267	-1.41401835	-0.57656503
C	-3.29126829	-0.88426837	-0.30100752
H	-3.85057170	-0.70432404	-1.21340741
H	-3.83966191	-1.60958023	0.28758728
H	-3.11211712	0.41026318	1.52720930
H	-3.62352497	1.26863938	0.14971293
H	-1.80554167	-2.01178570	-1.47712924
H	0.69788281	-2.18564463	1.12564747
C	-1.22038230	2.11709013	0.31017552
H	-0.19023889	2.20483218	0.63961522
H	-1.32522735	2.68055217	-0.62370169
H	-1.84084369	2.60753675	1.05300259
H	1.56806260	1.37545195	-1.29849370
C	4.14228015	0.77677256	-0.16132992
H	4.72832363	0.66963377	0.74416830
H	4.79534203	0.36240523	-0.92435993
H	4.01398238	1.84141665	-0.35845306
H	-1.59324770	-2.05772743	0.25623880

Time = 144.50 fs
E(CASSCF/6-31G(d)) = -479.5948334

C	-0.97168037	-0.23058640	-0.51353323
C	0.57667553	-0.37987615	-0.31245016
C	1.08642897	-1.29874046	0.66927876
C	1.59665862	0.49573388	-0.70468726
C	2.78957502	0.08960848	-0.06199571
C	2.49260262	-1.06893734	0.70448512
N	-1.64533320	0.77262906	0.15516153
C	-3.03528935	0.42274008	0.45973309
H	3.15507984	-1.77537268	1.15511531
C	-1.86693939	-1.41269159	-0.58242380

C	-3.29197775	-0.88284529	-0.30202014
H	-3.84737120	-0.69866836	-1.21613365
H	-3.84279219	-1.60802509	0.28427525
H	-3.11510756	0.40132316	1.52906632
H	-3.62836101	1.26541939	0.15891472
H	-1.79947504	-2.00290325	-1.48706892
H	0.68827211	-2.17400621	1.12864432
C	-1.21921883	2.11523628	0.31347242
H	-0.19033896	2.21132318	0.64272358
H	-1.33649372	2.68780090	-0.61327231
H	-1.83477979	2.60749660	1.06252698
H	1.56677547	1.36935376	-1.31251345
C	4.14172116	0.77535173	-0.16257808
H	4.72557944	0.66932594	0.74405999
H	4.79254040	0.35913824	-0.92558535
H	4.01873681	1.84013946	-0.36174266
H	-1.58678233	-2.06061865	0.24633827

Time = 145.00 fs
E(CASSCF/6-31G(d)) = -479.5965201

C	-0.97363566	-0.23031752	-0.51368207
C	0.57854323	-0.38346603	-0.31269376
C	1.08589325	-1.29248614	0.67325807
C	1.60050796	0.49214713	-0.71172605
C	2.78762556	0.08898002	-0.06353775
C	2.48928237	-1.06346063	0.71009902
N	-1.64426749	0.76826825	0.15687869
C	-3.03202233	0.42193944	0.46176702
H	3.15093658	-1.76437505	1.16499068
C	-1.86761639	-1.41143387	-0.58828080
C	-3.29263454	-0.88143402	-0.30300646
H	-3.84390000	-0.69285260	-1.21873425
H	-3.84625465	-1.60609480	0.28094572
H	-3.11862369	0.39101229	1.53205082
H	-3.63453992	1.26225897	0.16730896
H	-1.79289726	-1.99368223	-1.49717986
H	0.67690176	-2.16255617	1.13137123
C	-1.21794493	2.11369742	0.31684840
H	-0.19082436	2.21702949	0.64539581
H	-1.34773871	2.69440017	-0.60251877
H	-1.82866447	2.60700814	1.07191939
H	1.56438171	1.36376008	-1.32732958
C	4.14112074	0.77390110	-0.16383779
H	4.72234583	0.66923953	0.74467032
H	4.78932898	0.35586592	-0.92763237
H	4.02373669	1.83852658	-0.36507988

H	-1.57997024	-2.06364839	0.23604467

Time = 145.50 fs
E(CASSCF/6-31G(d)) = -479.5983936

C	-0.97541810	-0.22964734	-0.51352977
C	0.58000493	-0.38708181	-0.31292715
C	1.08568612	-1.28607716	0.67722098
C	1.60456949	0.48850912	-0.71873452
C	2.78576450	0.08841951	-0.06514898
C	2.48563215	-1.05801089	0.71575896
N	-1.64304521	0.76338525	0.15837002
C	-3.02868976	0.42127103	0.46376939
H	3.14798745	-1.75326519	1.17520433
C	-1.86844652	-1.41024974	-0.59412714
C	-3.29322902	-0.88003807	-0.30397800
H	-3.84021055	-0.68688620	-1.22118160
H	-3.85003421	-1.60381000	0.27759114
H	-3.12252217	0.37939763	1.53602358
H	-3.64183724	1.25912759	0.17489395
H	-1.78591197	-1.98416384	-1.50746890
H	0.66388125	-2.15135842	1.13392446
C	-1.21658581	2.11247814	0.32032189
H	-0.19155031	2.22184376	0.64754899
H	-1.35888522	2.70025267	-0.59154763
H	-1.82250412	2.60589079	1.08112143
H	1.56097898	1.35843861	-1.34276695
C	4.14047722	0.77241712	-0.16510819
H	4.71865767	0.66934140	0.74595924
H	4.78576412	0.35256162	-0.93046483
H	4.02890716	1.83660605	-0.36846451
H	-1.57292908	-2.06677635	0.22530395

Time = 146.00 fs
E(CASSCF/6-31G(d)) = -479.6003598

C	-0.97699826	-0.22859969	-0.51308968
C	0.58105783	-0.39066956	-0.31318551
C	1.08578732	-1.27953963	0.68118383
C	1.60878091	0.48482586	-0.72572850
C	2.78402078	0.08793486	-0.06682391
C	2.48166591	-1.05258853	0.72146464
N	-1.64166760	0.75804147	0.15965384
C	-3.02533354	0.42071931	0.46575856
H	3.14628846	-1.74212110	1.18582883
C	-1.86941891	-1.40914200	-0.59995538
C	-3.29375172	-0.87866032	-0.30494627

H	-3.83636060	-0.68077854	-1.22345951
H	-3.85411578	-1.60119723	0.27420635
H	-3.12664620	0.36657835	1.54074282
H	-3.64996959	1.25594782	0.18170341
H	-1.77863080	-1.97438551	-1.51793733
H	0.64938215	-2.14039361	1.13636787
C	-1.21516876	2.11157239	0.32390838
H	-0.19233011	2.22568296	0.64911966
H	-1.36986224	2.70528866	-0.58048782
H	-1.81632154	2.60399044	1.09010410
H	1.55668696	1.35311029	-1.35860878
C	4.13979138	0.77089428	-0.16638788
H	4.71454660	0.66959438	0.74785130
H	4.78189461	0.34920602	-0.93401127
H	4.03416672	1.83442622	-0.37189929
H	-1.56578184	-2.06996961	0.21407417

Time = 146.50 fs
E(CASSCF/6-31G(d)) = -479.6022590

C	-0.97834880	-0.22720737	-0.51238163
C	0.58170472	-0.39417294	-0.31350421
C	1.08616776	-1.27290722	0.68516641
C	1.61307432	0.48110412	-0.73272626
C	2.78242206	0.08753237	-0.06855566
C	2.47740877	-1.04719799	0.72721879
N	-1.64013870	0.75230827	0.16075438
C	-3.02199882	0.42026696	0.46775639
H	3.14581924	-1.73095852	1.19689570
C	-1.87051921	-1.40811135	-0.60576009
C	-3.29419357	-0.87730299	-0.30592259
H	-3.83241231	-0.67453918	-1.22556240
H	-3.85848282	-1.59828866	0.27078760
H	-3.13083004	0.35268541	1.54588996
H	-3.65861029	1.25261150	0.18779904
H	-1.77117196	-1.96437965	-1.52857778
H	0.63363766	-2.12956543	1.13873402
C	-1.21372161	2.11096393	0.32761993
H	-0.19294662	2.22848927	0.65006311
H	-1.38060505	2.70946712	-0.56948647
H	-1.81015746	2.60118610	1.09887097
H	1.55164452	1.34747722	-1.37461669
C	4.13906621	0.76932543	-0.16767515
H	4.71004157	0.66995794	0.75023947
H	4.77776102	0.34578803	-0.93816768
H	4.03942968	1.83205076	-0.37538894
H	-1.55865419	-2.07320545	0.20232959

Time = 147.00 fs
E(CASSCF/6-31G(d)) = -479.6039662

C	-0.97944370	-0.22551246	-0.51143293
C	0.58195292	-0.39753324	-0.31391829
C	1.08679030	-1.26621993	0.68919122
C	1.61737627	0.47734923	-0.73974723
C	2.78099492	0.08721597	-0.07033544
C	2.47289716	-1.04184744	0.73302719
N	-1.63846575	0.74626661	0.16170229
C	-3.01873176	0.41989383	0.46978610
H	3.14648217	-1.71972997	1.20839506
C	-1.87172992	-1.40715659	-0.61153798
C	-3.29454618	-0.87596768	-0.30691799
H	-3.82843019	-0.66817747	-1.22749468
H	-3.86311715	-1.59512059	0.26733169
H	-3.13490376	0.33787864	1.55110309
H	-3.66740786	1.24899844	0.19326259
H	-1.76365966	-1.95417218	-1.53937374
H	0.61693529	-2.11871480	1.14103135
C	-1.21227149	2.11062669	0.33146477
H	-0.19316861	2.23022977	0.65034892
H	-1.39105714	2.71277458	-0.55870133
H	-1.80406844	2.59739293	1.10746023
H	1.54600649	1.34125131	-1.39055150
C	4.13830660	0.76770259	-0.16896819
H	4.70517102	0.67038879	0.75299222
H	4.77339727	0.34230352	-0.94280288
H	4.04460828	1.82955341	-0.37893946
H	-1.55167194	-2.07647568	0.19006371

Time = 147.50 fs
E(CASSCF/6-31G(d)) = -479.6053442

C	-0.98025800	-0.22356561	-0.51027872
C	0.58181406	-0.40069012	-0.31446182
C	1.08761102	-1.25952167	0.69328172
C	1.62160840	0.47356349	-0.74681014
C	2.77976462	0.08698625	-0.07215196
C	2.46817838	-1.03654757	0.73889788
N	-1.63665940	0.74000549	0.16253433
C	-3.01557749	0.41957614	0.47187023
H	3.14811022	-1.70833375	1.22028020
C	-1.87303074	-1.40627453	-0.61728802
C	-3.29480217	-0.87465539	-0.30794316
H	-3.82447945	-0.66170297	-1.22926863

H	-3.86799750	-1.59173254	0.26383480
H	-3.13869956	0.32234257	1.55601143
H	-3.67600646	1.24499554	0.19818711
H	-1.75622278	-1.94378274	-1.55029868
H	0.59960571	-2.10764268	1.14325445
C	-1.21084324	2.11052605	0.33544713
H	-0.19276884	2.23089526	0.64995774
H	-1.40116960	2.71522356	-0.54829337
H	-1.79812431	2.59256446	1.11594264
H	1.53993901	1.33417813	-1.40619331
C	4.13751868	0.76601760	-0.17026533
H	4.69996506	0.67084152	0.75596123
H	4.76883231	0.33875440	-0.94776540
H	4.04961524	1.82701078	-0.38255684
H	-1.54495905	-2.07978851	0.17729120

Time = 148.00 fs
E(CASSCF/6-31G(d)) = -479.6063135

C	-0.98076706	-0.22142571	-0.50896292
C	0.58130309	-0.40358158	-0.31516719
C	1.08858098	-1.25285795	0.69746059
C	1.62568707	0.46974468	-0.75393190
C	2.77875551	0.08683907	-0.07399027
C	2.46330989	-1.03130971	0.74483995
N	-1.63473401	0.73362095	0.16329357
C	-3.01257766	0.41928595	0.47402851
H	3.15048086	-1.69662746	1.23247782
C	-1.87439899	-1.40546018	-0.62301111
C	-3.29495542	-0.87336654	-0.30900838
H	-3.82062376	-0.65512563	-1.23090229
H	-3.87309869	-1.58816601	0.26029139
H	-3.14205834	0.30628012	1.56026830
H	-3.68406607	1.24051388	0.20266914
H	-1.74899376	-1.93322523	-1.56131722
H	0.58200703	-2.09613575	1.14539653
C	-1.20945812	2.11062008	0.33956729
H	-0.19154391	2.23049827	0.64887643
H	-1.41090081	2.71684955	-0.53841746
H	-1.79240331	2.58669221	1.12441756
H	1.53361435	1.32605644	-1.42135878
C	4.13670925	0.76426302	-0.17156532
H	4.69445802	0.67126916	0.75899037
H	4.76409293	0.33514657	-0.95289168
H	4.05436595	1.82449519	-0.38624582
H	-1.53863636	-2.08317093	0.16404843

Time = 148.50 fs
E(CASSCF/6-31G(d)) = -479.6068412

C	-0.98094554	-0.21915945	-0.50753943
C	0.58043738	-0.40614383	-0.31606434
C	1.08964783	-1.24627361	0.70174737
C	1.62952237	0.46588524	-0.76112664
C	2.77799207	0.08676349	-0.07582994
C	2.45835874	-1.02614323	0.75086168
N	-1.63270825	0.72721525	0.16402964
C	-3.00976810	0.41899115	0.47627619
H	3.15333508	-1.68444481	1.24490260
C	-1.87580992	-1.40470712	-0.62870948
C	-3.29500140	-0.87210101	-0.31012350
H	-3.81692303	-0.64845611	-1.23241711
H	-3.87839094	-1.58446334	0.25669321
H	-3.14483652	0.28990507	1.56357932
H	-3.69128133	1.23550226	0.20680201
H	-1.74210752	-1.92250908	-1.57238729
H	0.56450683	-2.08399149	1.14746186
C	-1.20813290	2.11086104	0.34382148
H	-0.18933349	2.22907071	0.64709399
H	-1.42021503	2.71770765	-0.52921274
H	-1.78698579	2.57980365	1.13300619
H	1.52720522	1.31675128	-1.43591514
C	4.13588504	0.76243298	-0.17286747
H	4.68869086	0.67162380	0.76192480
H	4.75920648	0.33148775	-0.95801517
H	4.05878044	1.82206782	-0.39000861
H	-1.53282119	-2.08667028	0.15039274

Time = 149.00 fs
E(CASSCF/6-31G(d)) = -479.6069437

C	-0.98076587	-0.21684112	-0.50607407
C	0.57923526	-0.40831099	-0.31717959
C	1.09075683	-1.23981108	0.70615546
C	1.63301585	0.46197188	-0.76840524
C	2.77750087	0.08673868	-0.07764197
C	2.45340213	-1.02105201	0.75696816
N	-1.63060592	0.72089667	0.16479939
C	-3.00717652	0.41865594	0.47862254
H	3.15639855	-1.67161198	1.25747435
C	-1.87723688	-1.40400791	-0.63438562
C	-3.29493728	-0.87085812	-0.31129791
H	-3.81343143	-0.64170626	-1.23383551
H	-3.88383952	-1.58066709	0.25302856

H	-3.14691328	0.27343364	1.56572474
H	-3.69739834	1.22995667	0.21067029
H	-1.73570060	-1.91164112	-1.58346315
H	0.54746350	-2.07103954	1.14947676
C	-1.20687945	2.11119666	0.34820193
H	-0.18603657	2.22666174	0.64459880
H	-1.42908000	2.71786985	-0.52079303
H	-1.78194669	2.57195877	1.14184239
H	1.52087911	1.30620085	-1.44979038
C	4.13505222	0.76052387	-0.17417184
H	4.68271293	0.67185711	0.76461937
H	4.75420333	0.32778540	-0.96297570
H	4.06278481	1.81977297	-0.39384374
H	-1.52762843	-2.09035602	0.13640069

Time = 149.50 fs
E(CASSCF/6-31G(d)) = -479.6066755

C	-0.98019571	-0.21455288	-0.50464776
C	0.57771393	-0.41001557	-0.31853356
C	1.09184964	-1.23350899	0.71068751
C	1.63605704	0.45798597	-0.77577515
C	2.77731375	0.08672925	-0.07938400
C	2.44853071	-1.01602956	0.76315807
N	-1.62845757	0.71478045	0.16566830
C	-3.00482013	0.41824182	0.48106993
H	3.15940282	-1.65796077	1.27013581
C	-1.87865125	-1.40335487	-0.64004068
C	-3.29476205	-0.86963664	-0.31254051
H	-3.81019538	-0.63488959	-1.23517889
H	-3.88940502	-1.57681975	0.24928175
H	-3.14819833	0.25707547	1.56657593
H	-3.70222933	1.22392525	0.21434558
H	-1.72991098	-1.90062774	-1.59449902
H	0.53120793	-2.05715677	1.15149821
C	-1.20570530	2.11157106	0.35269651
H	-0.18162246	2.22333654	0.64137813
H	-1.43746340	2.71742416	-0.51323823
H	-1.77734693	2.56324588	1.15106170
H	1.51479269	1.29441757	-1.46297958
C	4.13421599	0.75853485	-0.17547936
H	4.67658284	0.67192101	0.76694563
H	4.74911847	0.32404509	-0.96762840
H	4.06631205	1.81763387	-0.39774495
H	-1.52317316	-2.09432248	0.12216538

Time = 150.00 fs

E(CASSCF/6-31G(d)) = -479.6061160

C	-0.97919444	-0.21238568	-0.50336048
C	0.57588632	-0.41119251	-0.32013672
C	1.09285814	-1.22740073	0.71532784
C	1.63852109	0.45390488	-0.78324045
C	2.77747167	0.08667969	-0.08099405
C	2.44385605	-1.01105192	0.76941977
N	-1.62630317	0.70899120	0.16671269
C	-3.00270305	0.41770907	0.48361310
H	3.16210443	-1.64333840	1.28286776
C	-1.88002200	-1.40274088	-0.64567251
C	-3.29447651	-0.86843472	-0.31385955
H	-3.80725206	-0.62802182	-1.23646627
H	-3.89504425	-1.57296417	0.24543282
H	-3.14864072	0.24102424	1.56610643
H	-3.70566508	1.21750901	0.21788352
H	-1.72487907	-1.88947719	-1.60545273
H	0.51602632	-2.04227517	1.15361965
C	-1.20461511	2.11192513	0.35728820
H	-0.17613553	2.21917666	0.63742080
H	-1.44532853	2.71647681	-0.50658763
H	-1.77322397	2.55377726	1.16078923
H	1.50908675	1.28148443	-1.47554645
C	4.13338059	0.75646806	-0.17679195
H	4.67036838	0.67176861	0.76879700
H	4.74399226	0.32026983	-0.97185171
H	4.06930234	1.81565035	-0.40169999
H	-1.51957424	-2.09869336	0.10779302

Time = 150.50 fs
E(CASSCF/6-31G(d)) = -479.6053671

C	-0.97771050	-0.21043900	-0.50233219
C	0.57375818	-0.41179147	-0.32198147
C	1.09369151	-1.22151119	0.72003255
C	1.64027519	0.44970481	-0.79080062
C	2.77802474	0.08651229	-0.08238760
C	2.43952014	-1.00606987	0.77572779
N	-1.62419453	0.70366465	0.16802070
C	-3.00081454	0.41701831	0.48623910
H	3.16430021	-1.62761905	1.29569449
C	-1.88131548	-1.40216026	-0.65127410
C	-3.29408312	-0.86724980	-0.31526255
H	-3.80462825	-0.62112142	-1.23771322
H	-3.90071123	-1.56914411	0.24145791
H	-3.14823633	0.22544913	1.56439693

H	-3.70768248	1.21085876	0.22132248
H	-1.72074861	-1.87820235	-1.61628868
H	0.50214402	-2.02638299	1.15597186
C	-1.20361251	2.11219695	0.36195457
H	-0.16969618	2.21428124	0.63272272
H	-1.45263104	2.71515421	-0.50083518
H	-1.76958270	2.54368429	1.17112704
H	1.50388086	1.26755026	-1.48761932
C	4.13254958	0.75432839	-0.17811287
H	4.66414691	0.67135648	0.77009301
H	4.73887219	0.31646043	-0.97555307
H	4.07170321	1.81379893	-0.40568900
H	-1.51695346	-2.10362289	0.09339788

Time = 151.00 fs
E(CASSCF/6-31G(d)) = -479.6045847

C	-0.97568620	-0.20881497	-0.50169106
C	0.57132898	-0.41179624	-0.32403448
C	1.09422566	-1.21585067	0.72472500
C	1.64120353	0.44536347	-0.79844735
C	2.77902054	0.08613822	-0.08346902
C	2.43569332	-1.00100831	0.78204438
N	-1.62219293	0.69894121	0.16968626
C	-2.99912994	0.41613171	0.48892809
H	3.16583885	-1.61072705	1.30867160
C	-1.88249687	-1.40160846	-0.65683480
C	-3.29358591	-0.86607865	-0.31675633
H	-3.80233968	-0.61420946	-1.23893080
H	-3.90635691	-1.56540340	0.23732990
H	-3.14702599	0.21048969	1.56162960
H	-3.70833997	1.20416883	0.22468507
H	-1.71766318	-1.86682271	-1.62697932
H	0.48971194	-2.00952978	1.15871845
C	-1.20270012	2.11232476	0.36666938
H	-0.16250093	2.20876499	0.62729059
H	-1.45932233	2.71359443	-0.49592724
H	-1.76638759	2.53310927	1.18214148
H	1.49926829	1.25282919	-1.49938107
C	4.13172628	0.75212309	-0.17944699
H	4.65800814	0.67064864	0.77078415
H	4.73381758	0.31261534	-0.97867016
H	4.07347112	1.81203488	-0.40968354
H	-1.51542274	-2.10928244	0.07909207

Time = 151.50 fs
E(CASSCF/6-31G(d)) = -479.6040099

C	-0.97307424	-0.20760362	-0.50154682
C	0.56860044	-0.41123310	-0.32624020
C	1.09431660	-1.21040999	0.72930912
C	1.64124057	0.44086017	-0.80615979
C	2.78048158	0.08548266	-0.08415959
C	2.43254461	-0.99578203	0.78832811
N	-1.62035671	0.69494582	0.17179240
C	-2.99761709	0.41501289	0.49165531
H	3.16662912	-1.59266544	1.32186805
C	-1.88353434	-1.40108087	-0.66234492
C	-3.29299048	-0.86491761	-0.31834754
H	-3.80039114	-0.60730884	-1.24012315
H	-3.91192589	-1.56178265	0.23301995
H	-3.14507777	0.19625758	1.55806866
H	-3.70775650	1.19766791	0.22798272
H	-1.71575394	-1.85536564	-1.63750469
H	0.47879892	-1.99184096	1.16204797
C	-1.20187682	2.11225359	0.37140719
H	-0.15481932	2.20274849	0.62114052
H	-1.46535993	2.71191967	-0.49176201
H	-1.76356092	2.52219293	1.19385027
H	1.49531356	1.23759839	-1.51105860
C	4.13091348	0.74986145	-0.18080078
H	4.65205908	0.66962046	0.77085823
H	4.72890643	0.30872861	-0.98116886
H	4.07457440	1.81029739	-0.41364819
H	-1.51506132	-2.11582244	0.06497049

Time = 152.00 fs
E(CASSCF/6-31G(d)) = -479.6039304

C	-0.96985371	-0.20686937	-0.50197048
C	0.56558472	-0.41015472	-0.32853552
C	1.09383462	-1.20516239	0.73369226
C	1.64037931	0.43617360	-0.81390374
C	2.78239325	0.08450129	-0.08441729
C	2.43020464	-0.99031626	0.79454195
N	-1.61872724	0.69176816	0.17439624
C	-2.99624332	0.41362727	0.49439287
H	3.16664584	-1.57352357	1.33536658
C	-1.88440254	-1.40057224	-0.66779892
C	-3.29230382	-0.86376271	-0.32004325
H	-3.79877667	-0.60044316	-1.24128563
H	-3.91735336	-1.55831615	0.22849909
H	-3.14246639	0.18284081	1.55403556
H	-3.70608717	1.19160569	0.23122105

H	-1.71512673	-1.84386597	-1.64785198
H	0.46939047	-1.97353132	1.16616942
C	-1.20113570	2.11194077	0.37614791
H	-0.14697622	2.19634656	0.61429486
H	-1.47071609	2.71021110	-0.48819222
H	-1.76098675	2.51106410	1.20621321
H	1.49205466	1.22218173	-1.52291252
C	4.13011273	0.74755446	-0.18218154
H	4.64642524	0.66825941	0.77034298
H	4.72423634	0.30478743	-0.98304190
H	4.07499425	1.80851632	-0.41754503
H	-1.51590675	-2.12333626	0.05109808

Time = 152.50 fs
E(CASSCF/6-31G(d)) = -479.6045643

C	-0.96603616	-0.20664601	-0.50298971
C	0.56230698	-0.40861673	-0.33086366
C	1.09269258	-1.20006973	0.73780034
C	1.63865659	0.43128038	-0.82163506
C	2.78470731	0.08318005	-0.08423831
C	2.42874551	-0.98455798	0.80065628
N	-1.61732180	0.68945480	0.17752316
C	-2.99497955	0.41194333	0.49711108
H	3.16593126	-1.55346195	1.34927700
C	-1.88508369	-1.40007753	-0.67319539
C	-3.29153400	-0.86260979	-0.32185097
H	-3.79748076	-0.59363540	-1.24240553
H	-3.92256582	-1.55503135	0.22373995
H	-3.13926284	0.17030615	1.54988613
H	-3.70350557	1.18623570	0.23440496
H	-1.71585547	-1.83236479	-1.65801722
H	0.46139415	-1.95490051	1.17130842
C	-1.20046498	2.11135735	0.38087843
H	-0.13932095	2.18966248	0.60678258
H	-1.47538031	2.70850309	-0.48503219
H	-1.75851735	2.49983652	1.21913155
H	1.48950894	1.20691712	-1.53522154
C	4.12932406	0.74521439	-0.18359652
H	4.64124562	0.66656348	0.76930191
H	4.71991988	0.30077145	-0.98430829
H	4.07472480	1.80662013	-0.42133792
H	-1.51796092	-2.13184627	0.03750394

Time = 153.00 fs
E(CASSCF/6-31G(d)) = -479.6059899

C	-0.96167366	-0.20693078	-0.50457643
C	0.55881200	-0.40664741	-0.33319505
C	1.09087944	-1.19508943	0.74159901
C	1.63613523	0.42615250	-0.82930337
C	2.78734417	0.08153691	-0.08366116
C	2.42815484	-0.97849096	0.80665417
N	-1.61612380	0.68799721	0.18115618
C	-2.99380638	0.40993340	0.49977998
H	3.16458934	-1.53269543	1.36374546
C	-1.88557002	-1.39959211	-0.67853842
C	-3.29068992	-0.86145476	-0.32377890
H	-3.79647967	-0.58690631	-1.24346236
H	-3.92748088	-1.55194706	0.21871896
H	-3.13551504	0.15870587	1.54598093
H	-3.70017814	1.18179722	0.23754541
H	-1.71797327	-1.82090548	-1.66800481
H	0.45465253	-1.93632978	1.17770145
C	-1.19984691	2.11049093	0.38559585
H	-0.13219742	2.18277708	0.59863487
H	-1.47936590	2.70676908	-0.48206698
H	-1.75598578	2.48860488	1.23245108
H	1.48768225	1.19211944	-1.54826233
C	4.12854539	0.74285421	-0.18505169
H	4.63666749	0.66453952	0.76782865
H	4.71607931	0.29665258	-0.98501142
H	4.07377321	1.80454462	-0.42499814
H	-1.52119070	-2.14128258	0.02417477

Time = 153.50 fs
E(CASSCF/6-31G(d)) = -479.6078697

C	-0.95703801	-0.20753942	-0.50627584
C	0.55528818	-0.40422611	-0.33570119
C	1.08871045	-1.19010472	0.74536739
C	1.63321915	0.42068961	-0.83682652
C	2.78992808	0.07992990	-0.08313291
C	2.42790860	-0.97235676	0.81270270
N	-1.61492293	0.68708176	0.18503095
C	-2.99279198	0.40754643	0.50239290
H	3.16277579	-1.51184404	1.37866377
C	-1.88592226	-1.39908100	-0.68393695
C	-3.28978263	-0.86029570	-0.32585082
H	-3.79574288	-0.58024746	-1.24439626
H	-3.93195799	-1.54901510	0.21341450
H	-3.13096055	0.14813174	1.54251314
H	-3.69600029	1.17852964	0.24071127
H	-1.72134897	-1.80951421	-1.67784159

H	0.44895602	-1.91846807	1.18555393
C	-1.19918324	2.10942749	0.39038540
H	-0.12609719	2.17560964	0.58976796
H	-1.48290224	2.70452137	-0.47904095
H	-1.75324420	2.47730865	1.24595661
H	1.48655872	1.17819584	-1.56228256
C	4.12776178	0.74048332	-0.18655279
H	4.63291033	0.66223009	0.76611029
H	4.71292505	0.29234798	-0.98516147
H	4.07219775	1.80224384	-0.42854056
H	-1.52521437	-2.15106041	0.01091479

Time = 154.00 fs
E(CASSCF/6-31G(d)) = -479.6084395

C	-0.95188185	-0.20867298	-0.50860272
C	0.55152205	-0.40164384	-0.33799242
C	1.08563156	-1.18507598	0.74861867
C	1.62972914	0.41497349	-0.84425158
C	2.79271001	0.07798413	-0.08222901
C	2.42866965	-0.96572370	0.81859065
N	-1.61395207	0.68706493	0.18947609
C	-2.99177705	0.40479401	0.50491441
H	3.16064860	-1.49060280	1.39455754
C	-1.88604905	-1.39857775	-0.68930096
C	-3.28880952	-0.85911323	-0.32806829
H	-3.79523148	-0.57366357	-1.24525712
H	-3.93603925	-1.54633874	0.20777381
H	-3.12612354	0.13840304	1.54004655
H	-3.69149665	1.17653534	0.24387953
H	-1.72622405	-1.79821599	-1.68762408
H	0.44400543	-1.90114233	1.19515421
C	-1.19857422	2.10802139	0.39516243
H	-0.12100746	2.16844893	0.58042169
H	-1.48578460	2.70239350	-0.47578262
H	-1.75000383	2.46617516	1.25959972
H	1.48613229	1.16503276	-1.57743721
C	4.12699284	0.73809692	-0.18812033
H	4.62997318	0.65962951	0.76412813
H	4.71044242	0.28788137	-0.98487409
H	4.06996130	1.79963275	-0.43189766
H	-1.53041652	-2.16180943	-0.00221193

Time = 154.50 fs
E(CASSCF/6-31G(d)) = -479.6089799

C	-0.94675295	-0.21002300	-0.51082556

C	0.54787590	-0.39911395	-0.34018840
C	1.08212688	-1.18000074	0.75161687
C	1.62647527	0.40911090	-0.85123101
C	2.79505224	0.07632120	-0.08165431
C	2.42968746	-0.95916838	0.82437184
N	-1.61298332	0.68751041	0.19397730
C	-2.99088835	0.40173515	0.50726629
H	3.15850487	-1.47071692	1.41042946
C	-1.88603691	-1.39804729	-0.69460256
C	-3.28781631	-0.85794840	-0.33037794
H	-3.79492269	-0.56737689	-1.24595147
H	-3.93942142	-1.54395658	0.20199854
H	-3.12087088	0.12993565	1.53873974
H	-3.68659400	1.17603730	0.24704392
H	-1.73222663	-1.78750553	-1.69705984
H	0.43971958	-1.88558403	1.20626268
C	-1.19791745	2.10645148	0.39986736
H	-0.11762080	2.16147291	0.57090537
H	-1.48827213	2.69984839	-0.47219967
H	-1.74622348	2.45551518	1.27264294
H	1.48636820	1.15345573	-1.59312330
C	4.12625595	0.73578782	-0.18970146
H	4.62818164	0.65692383	0.76216328
H	4.70895398	0.28332604	-0.98414543
H	4.06724733	1.79678444	-0.43497426
H	-1.53599250	-2.17232432	-0.01498242

Time = 155.00 fs
E(CASSCF/6-31G(d)) = -479.6096479

C	-0.94158868	-0.21158436	-0.51295449
C	0.54428726	-0.39658748	-0.34232973
C	1.07816342	-1.17471257	0.75443686
C	1.62341109	0.40295703	-0.85795333
C	2.79696933	0.07489711	-0.08141986
C	2.43091563	-0.95251027	0.83018894
N	-1.61196097	0.68836481	0.19861191
C	-2.99008130	0.39830081	0.50949580
H	3.15644783	-1.45181453	1.42686760
C	-1.88589035	-1.39747760	-0.69998628
C	-3.28678227	-0.85676381	-0.33284472
H	-3.79476985	-0.56122228	-1.24650071
H	-3.94215201	-1.54183645	0.19594044
H	-3.11516150	0.12246768	1.53875995
H	-3.68127768	1.17701915	0.25034474
H	-1.73946837	-1.77714477	-1.70645031
H	0.43579306	-1.87154135	1.21927408

C	-1.19719838	2.10467454	0.40462661
H	-0.11585585	2.15457650	0.56109694
H	-1.49049604	2.69684990	-0.46804244
H	-1.74164723	2.44514520	1.28526010
H	1.48727991	1.14302884	-1.60965527
C	4.12553510	0.73349909	-0.19133790
H	4.62752514	0.65407862	0.76022074
H	4.70846598	0.27855522	-0.98304848
H	4.06403402	1.79362813	-0.43785478
H	-1.54202769	-2.18274690	-0.02781051

Time = 155.50 fs
E(CASSCF/6-31G(d)) = -479.6105088

C	-0.93646628	-0.21331548	-0.51493774
C	0.54079276	-0.39408199	-0.34439888
C	1.07380002	-1.16919225	0.75708011
C	1.62057367	0.39653535	-0.86442700
C	2.79841686	0.07371169	-0.08155024
C	2.43228084	-0.94575112	0.83602931
N	-1.61085614	0.68956159	0.20333382
C	-2.98933980	0.39450971	0.51159116
H	3.15458661	-1.43401985	1.44398497
C	-1.88561545	-1.39687081	-0.70545289
C	-3.28571192	-0.85555371	-0.33547089
H	-3.79474043	-0.55520221	-1.24691668
H	-3.94419285	-1.54002160	0.18961514
H	-3.10908989	0.11598118	1.54023438
H	-3.67562195	1.17941914	0.25385210
H	-1.74789460	-1.76718430	-1.71583730
H	0.43211807	-1.85908255	1.23421779
C	-1.19642520	2.10268759	0.40943826
H	-0.11562242	2.14784170	0.55114473
H	-1.49252900	2.69344947	-0.46319349
H	-1.73614467	2.43518201	1.29729860
H	1.48889263	1.13352905	-1.62681237
C	4.12483708	0.73123402	-0.19302686
H	4.62801242	0.65113691	0.75832492
H	4.70900852	0.27357777	-0.98166399
H	4.06037744	1.79019117	-0.44054701
H	-1.54845467	-2.19293238	-0.04075969

Time = 156.00 fs
E(CASSCF/6-31G(d)) = -479.6115398

C	-0.93146216	-0.21517362	-0.51672580
C	0.53742712	-0.39161486	-0.34637920

C	1.06909360	-1.16343248	0.75955885
C	1.61799505	0.38987927	-0.87067206
C	2.79936052	0.07276044	-0.08206364
C	2.43371154	-0.93889696	0.84188344
N	-1.60964269	0.69102953	0.20809625
C	-2.98865036	0.39038951	0.51354841
H	3.15296191	-1.41739126	1.46182817
C	-1.88522117	-1.39622880	-0.71100368
C	-3.28460844	-0.85431263	-0.33825679
H	-3.79480607	-0.54931307	-1.24722297
H	-3.94552138	-1.53856158	0.18304756
H	-3.10274842	0.11046110	1.54318596
H	-3.66966288	1.18309722	0.25764874
H	-1.75743252	-1.75766465	-1.72525683
H	0.42867506	-1.84814180	1.25102226
C	-1.19561101	2.10048748	0.41429929
H	-0.11671353	2.14134618	0.54120736
H	-1.49445676	2.68972474	-0.45758004
H	-1.72962214	2.42574812	1.30865008
H	1.49123436	1.12464413	-1.64427761
C	4.12417135	0.72899276	-0.19476379
H	4.62960690	0.64814721	0.75646462
H	4.71057882	0.26841878	-0.98006118
H	4.05634385	1.78652387	-0.44306846
H	-1.55519772	-2.20274447	-0.05388540

Time = 156.50 fs
E(CASSCF/6-31G(d)) = -479.6126487

C	-0.92665027	-0.21711624	-0.51827211
C	0.53422299	-0.38920307	-0.34825507
C	1.06409736	-1.15743764	0.76189600
C	1.61570279	0.38302858	-0.87671679
C	2.79977591	0.07203466	-0.08297170
C	2.43514030	-0.93195776	0.84774559
N	-1.60829795	0.69269402	0.21285266
C	-2.98800207	0.38597586	0.51537214
H	3.15153807	-1.40191667	1.48036705
C	-1.88471934	-1.39555279	-0.71664119
C	-3.28347377	-0.85303568	-0.34120082
H	-3.79494311	-0.54354551	-1.24745431
H	-3.94613026	-1.53751031	0.17627017
H	-3.09622561	0.10589681	1.54752986
H	-3.66340333	1.18784242	0.26182560
H	-1.76799197	-1.74861322	-1.73473437
H	0.42553170	-1.83852501	1.26951232
C	-1.19477225	2.09807135	0.41920568

H	-0.11881919	2.13516154	0.53144544
H	-1.49637748	2.68577845	-0.45117451
H	-1.72202604	2.41696960	1.31925866
H	1.49432850	1.11601072	-1.66167483
C	4.12354994	0.72677230	-0.19654292
H	4.63222847	0.64516187	0.75459563
H	4.71314011	0.26312068	-0.97829431
H	4.05200903	1.78269628	-0.44544516
H	-1.56217193	-2.21206215	-0.06723109

Time = 157.00 fs
E(CASSCF/6-31G(d)) = -479.6137306

C	-0.92210069	-0.21910206	-0.51953499
C	0.53121004	-0.38686375	-0.35001145
C	1.05886042	-1.15122211	0.76412437
C	1.61372154	0.37602593	-0.88259417
C	2.79964831	0.07152226	-0.08428007
C	2.43650537	-0.92494703	0.85361429
N	-1.60680384	0.69447896	0.21755836
C	-2.98738547	0.38131114	0.51707532
H	3.15020565	-1.38752011	1.49949121
C	-1.88412438	-1.39484292	-0.72236971
C	-3.28230882	-0.85171883	-0.34429941
H	-3.79513273	-0.53788476	-1.24765466
H	-3.94602615	-1.53692352	0.16932089
H	-3.08960563	0.10228072	1.55308038
H	-3.65682209	1.19338614	0.26647512
H	-1.77946693	-1.74004343	-1.74428110
H	0.42282843	-1.82993055	1.28941544
C	-1.19392707	2.09543662	0.42415216
H	-0.12154879	2.12935193	0.52201428
H	-1.49839908	2.68173485	-0.44399282
H	-1.71334414	2.40897206	1.32912421
H	1.49818758	1.10725480	-1.67860715
C	4.12298688	0.72456694	-0.19835801
H	4.63575811	0.64223587	0.75264741
H	4.71662192	0.25774262	-0.97640150
H	4.04745691	1.77879282	-0.44771043
H	-1.56928381	-2.22078613	-0.08082332

Time = 157.50 fs
E(CASSCF/6-31G(d)) = -479.6146490

C	-0.91787820	-0.22109200	-0.52047712
C	0.52841515	-0.38461300	-0.35163498
C	1.05342887	-1.14480781	0.76628508

C	1.61207244	0.36891329	-0.88833813
C	2.79897225	0.07120761	-0.08598798
C	2.43775077	-0.91788043	0.85949115
N	-1.60514694	0.69630735	0.22217057
C	-2.98679203	0.37644293	0.51867846
H	3.14879436	-1.37407054	1.51901846
C	-1.88345329	-1.39409784	-0.72819558
C	-3.28111368	-0.85035928	-0.34754718
H	-3.79536037	-0.53231195	-1.24787448
H	-3.94522734	-1.53685516	0.16224037
H	-3.08296803	0.09960396	1.55956763
H	-3.64988613	1.19941924	0.27168405
H	-1.79173592	-1.73195265	-1.75389012
H	0.42075049	-1.82198199	1.31038082
C	-1.19309286	2.09258216	0.42913258
H	-0.12446067	2.12397273	0.51305555
H	-1.50063638	2.67773461	-0.43608925
H	-1.70360452	2.40187727	1.33830313
H	1.50280868	1.09802513	-1.69469501
C	4.12249750	0.72236888	-0.20020304
H	4.64004454	0.63942458	0.75053321
H	4.72092313	0.25235816	-0.97440648
H	4.04277825	1.77490661	-0.44990305
H	-1.57643139	-2.22884105	-0.09467083

Time = 158.00 fs
E(CASSCF/6-31G(d)) = -479.6153333

C	-0.91404128	-0.22304962	-0.52106689
C	0.52586144	-0.38246626	-0.35311344
C	1.04784746	-1.13822106	0.76842531
C	1.61077418	0.36172948	-0.89398080
C	2.79775126	0.07107248	-0.08808900
C	2.43882564	-0.91077481	0.86538026
N	-1.60331901	0.69810294	0.22664964
C	-2.98621266	0.37142258	0.52020808
H	3.14709294	-1.36139928	1.53870833
C	-1.88272524	-1.39331454	-0.73412732
C	-3.27988808	-0.84895566	-0.35093698
H	-3.79561549	-0.52680485	-1.24816764
H	-3.94376176	-1.53735417	0.15506933
H	-3.07638816	0.09785078	1.56666168
H	-3.64256315	1.20561271	0.27752579
H	-1.80466347	-1.72432366	-1.76353733
H	0.41949742	-1.81426548	1.33200311
C	-1.19228395	2.08950848	0.43413989
H	-0.12710123	2.11906948	0.50469086

H	-1.50320648	2.67392722	-0.42754848
H	-1.69287192	2.39579710	1.34690315
H	1.50817080	1.08802313	-1.70960795
C	4.12209741	0.72016914	-0.20207303
H	4.64491250	0.63678226	0.74816060
H	4.72591616	0.24705230	-0.97232112
H	4.03806960	1.77113102	-0.45206495
H	-1.58350612	-2.23618066	-0.10876150

Time = 158.50 fs
E(CASSCF/6-31G(d)) = -479.6157504

C	-0.91064104	-0.22494159	-0.52127794
C	0.52356808	-0.38043759	-0.35443644
C	1.04216144	-1.13148961	0.77059571
C	1.60984250	0.35450803	-0.89955002
C	2.79599742	0.07109611	-0.09057090
C	2.43968316	-0.90364651	0.87128630
N	-1.60131683	0.69979099	0.23095922
C	-2.98563700	0.36630354	0.52169479
H	3.14487313	-1.34931642	1.55828359
C	-1.88196133	-1.39248812	-0.74017545
C	-3.27863180	-0.84750832	-0.35446018
H	-3.79589037	-0.52133961	-1.24858741
H	-3.94166375	-1.53846048	0.14784559
H	-3.06993731	0.09699186	1.57400051
H	-3.63483379	1.21163782	0.28405394
H	-1.81810103	-1.71712498	-1.77318190
H	0.41925130	-1.80636669	1.35385171
C	-1.19150884	2.08621871	0.43916637
H	-0.12904772	2.11467712	0.49701612
H	-1.50622371	2.67046252	-0.41847514
H	-1.68124197	2.39082819	1.35507511
H	1.51423441	1.07702231	-1.72308980
C	4.12180141	0.71795856	-0.20396468
H	4.65017185	0.63435984	0.74544363
H	4.73145325	0.24191659	-0.97015067
H	4.03343183	1.76755242	-0.45423881
H	-1.59039475	-2.24278746	-0.12306351

Time = 159.00 fs
E(CASSCF/6-31G(d)) = -479.6159371

C	-0.90772025	-0.22673797	-0.52108894
C	0.52154997	-0.37853931	-0.35559576
C	1.03641794	-1.12463988	0.77284776
C	1.60928983	0.34727581	-0.90506773

C	2.79373089	0.07125543	-0.09341584
C	2.44027925	-0.89650989	0.87721271
N	-1.59914203	0.70129917	0.23506644
C	-2.98505279	0.36113976	0.52317118
H	3.14191390	-1.33762781	1.57745360
C	-1.88118421	-1.39161181	-0.74635188
C	-3.27734507	-0.84601942	-0.35810685
H	-3.79617881	-0.51589238	-1.24918252
H	-3.93897118	-1.54020142	0.14060111
H	-3.06368256	0.09697834	1.58121908
H	-3.62670166	1.21718572	0.29129715
H	-1.83188815	-1.71031206	-1.78276971
H	0.42015045	-1.79790321	1.37549960
C	-1.19076773	2.08271950	0.44420392
H	-0.12995153	2.11081939	0.49009765
H	-1.50979414	2.66748112	-0.40898160
H	-1.66883313	2.38704588	1.36300049
H	1.52094239	1.06488089	-1.73497637
C	4.12162238	0.71572885	-0.20587689
H	4.65562677	0.63220301	0.74231399
H	4.73737377	0.23704380	-0.96789943
H	4.02896868	1.76424272	-0.45646575
H	-1.59698193	-2.24867143	-0.13752779

Time = 159.50 fs
E(CASSCF/6-31G(d)) = -479.6159895

C	-0.90531256	-0.22841259	-0.52048325
C	0.51981742	-0.37678153	-0.35658567
C	1.03066696	-1.11769507	0.77523133
C	1.60912476	0.34005226	-0.91054862
C	2.79097943	0.07152535	-0.09660036
C	2.44057156	-0.88937616	0.88315970
N	-1.59680064	0.70255855	0.23894208
C	-2.98444553	0.35598415	0.52466978
H	3.13802256	-1.32614923	1.59593800
C	-1.88041772	-1.39067701	-0.75266934
C	-3.27602892	-0.84449312	-0.36186608
H	-3.79647499	-0.51044113	-1.24999337
H	-3.93572286	-1.54258845	0.13336014
H	-3.05768618	0.09773739	1.58797562
H	-3.61820041	1.22198411	0.29925553
H	-1.84585384	-1.70383048	-1.79223772
H	0.42227071	-1.78854881	1.39654941
C	-1.19005077	2.07902186	0.44924433
H	-0.12957593	2.10750883	0.48397231
H	-1.51400874	2.66510433	-0.39917545

H	-1.65577804	2.38449780	1.37087469
H	1.52822234	1.05154841	-1.74520733
C	4.12157021	0.71347354	-0.20781114
H	4.66108464	0.63035046	0.73873020
H	4.74351209	0.23252235	-0.96557660
H	4.02478506	1.76125349	-0.45878295
H	-1.60315303	-2.25386676	-0.15209011

Time = 160.00 fs
E(CASSCF/6-31G(d)) = -479.6160206

C	-0.90344180	-0.22994309	-0.51944843
C	0.51837582	-0.37517189	-0.35740327
C	1.02496167	-1.11067354	0.77779246
C	1.60935148	0.33284908	-0.91599952
C	2.78777792	0.07187900	-0.10009555
C	2.44051863	-0.88225242	0.88912279
N	-1.59430271	0.70350441	0.24256058
C	-2.98379846	0.35088705	0.52622099
H	3.13305187	-1.31471841	1.61348740
C	-1.87968637	-1.38967353	-0.75914034
C	-3.27468554	-0.84293548	-0.36572621
H	-3.79677226	-0.50496723	-1.25104876
H	-3.93195650	-1.54561479	0.12613737
H	-3.05200454	0.09916956	1.59397495
H	-3.60939813	1.22581150	0.30789913
H	-1.85981847	-1.69761810	-1.80152044
H	0.42561386	-1.77805146	1.41665547
C	-1.18933745	2.07514205	0.45427976
H	-0.12782115	2.10474655	0.47864837
H	-1.51893774	2.66342460	-0.38914712
H	-1.64221306	2.38319749	1.37888811
H	1.53598928	1.03706713	-1.75383199
C	4.12165084	0.71118875	-0.20977161
H	4.66636390	0.62883248	0.73468465
H	4.74970537	0.22843080	-0.96320154
H	4.02098529	1.75861150	-0.46122197
H	-1.60879747	-2.25842682	-0.16667603

Time = 160.50 fs
E(CASSCF/6-31G(d)) = -479.6161365

C	-0.90212144	-0.23131116	-0.51797569
C	0.51722554	-0.37371525	-0.35804864
C	1.01935824	-1.10358797	0.78057151
C	1.60996920	0.32567030	-0.92141917
C	2.78416791	0.07228793	-0.10386713

C	2.44007946	-0.87514098	0.89509149
N	-1.59166175	0.70407716	0.24590001
C	-2.98309287	0.34589486	0.52785148
H	3.12691168	-1.30320297	1.62990075
C	-1.87901487	-1.38858999	-0.76577617
C	-3.27331847	-0.84135441	-0.36967515
H	-3.79706226	-0.49945682	-1.25236347
H	-3.92770755	-1.54925392	0.11893735
H	-3.04668678	0.10114807	1.59898663
H	-3.60039873	1.22850776	0.31716789
H	-1.87359596	-1.69160826	-1.81055658
H	0.43010281	-1.76624374	1.43554145
C	-1.18859727	2.07110214	0.45930320
H	-0.12473289	2.10252232	0.47410919
H	-1.52462504	2.66249715	-0.37895962
H	-1.62826872	2.38311851	1.38720602
H	1.54414902	1.02156901	-1.76101045
C	4.12186558	0.70887372	-0.21176522
H	4.67130063	0.62766982	0.73020777
H	4.75580092	0.22483307	-0.96080786
H	4.01767117	1.75631670	-0.46380740
H	-1.61381194	-2.26241757	-0.18120565

Time = 161.00 fs
E(CASSCF/6-31G(d)) = -479.6164037

C	-0.90135433	-0.23250261	-0.51605947
C	0.51636160	-0.37241369	-0.35852494
C	1.01391528	-1.09644475	0.78360167
C	1.61097162	0.31851288	-0.92679866
C	2.78019713	0.07272247	-0.10787574
C	2.43921342	-0.86803905	0.90104867
N	-1.58889422	0.70422316	0.24894196
C	-2.98230849	0.34104880	0.52958263
H	3.11957597	-1.29150609	1.64503795
C	-1.87842745	-1.38741431	-0.77258564
C	-3.27193270	-0.83975935	-0.37370050
H	-3.79733424	-0.49390169	-1.25393684
H	-3.92300881	-1.55345966	0.11175426
H	-3.04177325	0.10351988	1.60285855
H	-3.59134082	1.22998132	0.32697331
H	-1.88699641	-1.68573176	-1.81929639
H	0.43558175	-1.75304880	1.45301353
C	-1.18779185	2.06693040	0.46430921
H	-0.12049206	2.10081441	0.47031645
H	-1.53108491	2.66233398	-0.36864129
H	-1.61406051	2.38418945	1.39595003

H	1.55260159	1.00526762	-1.76700780
C	4.12221067	0.70653107	-0.21380135
H	4.67575363	0.62687312	0.72536936
H	4.76166246	0.22177436	-0.95844612
H	4.01493979	1.75434280	-0.46655648
H	-1.61810411	-2.26590999	-0.19560017

Time = 161.50 fs
E(CASSCF/6-31G(d)) = -479.6168388

C	-0.90113249	-0.23350751	-0.51369703
C	0.51577374	-0.37126627	-0.35883844
C	1.00869302	-1.08924396	0.78690766
C	1.61234641	0.31136787	-0.93212233
C	2.77591900	0.07315197	-0.11207719
C	2.43788049	-0.86093881	0.90697019
N	-1.58601906	0.70389564	0.25167144
C	-2.98142414	0.33638389	0.53142945
H	3.11108483	-1.27956821	1.65882861
C	-1.87794735	-1.38613429	-0.77957403
C	-3.27053457	-0.83816107	-0.37778983
H	-3.79757511	-0.48829984	-1.25575255
H	-3.91789122	-1.55816711	0.10457323
H	-3.03729381	0.10610839	1.60552507
H	-3.58239404	1.23021240	0.33720141
H	-1.89982902	-1.67991966	-1.82770763
H	0.44182065	-1.73848085	1.46896954
C	-1.18687789	2.06266121	0.46929457
H	-0.11538902	2.09959013	0.46721487
H	-1.53829962	2.66290052	-0.35818299
H	-1.59968356	2.38629093	1.40518321
H	1.56124505	0.98844475	-1.77218206
C	4.12267715	0.70416666	-0.21589151
H	4.67960772	0.62644260	0.72027683
H	4.76717528	0.21927839	-0.95618470
H	4.01288112	1.75264042	-0.46947915
H	-1.62159567	-2.26897193	-0.20978715

Time = 162.00 fs
E(CASSCF/6-31G(d)) = -479.6174118

C	-0.90143724	-0.23432041	-0.51088811
C	0.51544652	-0.37026897	-0.35899849
C	1.00375221	-1.08197976	0.79050489
C	1.61407468	0.30422208	-0.93736943
C	2.77139217	0.07354520	-0.11642277
C	2.43604180	-0.85382771	0.91282502

N	-1.58305722	0.70305557	0.25407671
C	-2.98041850	0.33192828	0.53339969
H	3.10154231	-1.26736625	1.67127610
C	-1.87759617	-1.38473825	-0.78674204
C	-3.26913166	-0.83657125	-0.38193083
H	-3.79776982	-0.48265542	-1.25777958
H	-3.91238532	-1.56329496	0.09737216
H	-3.03326651	0.10871754	1.60701035
H	-3.57375331	1.22925211	0.34771693
H	-1.91190524	-1.67410518	-1.83578078
H	0.44852309	-1.72264175	1.48340372
C	-1.18581072	2.05833451	0.47425893
H	-0.10978668	2.09880650	0.46473593
H	-1.54621953	2.66411595	-0.34753969
H	-1.58521028	2.38925517	1.41490175
H	1.56997935	0.97143038	-1.77696312
C	4.12325101	0.70178919	-0.21804881
H	4.68277531	0.62636813	0.71507045
H	4.77224983	0.21734592	-0.95410895
H	4.01157538	1.75114233	-0.47257878
H	-1.62422514	-2.27166050	-0.22370581

Time = 162.50 fs
E(CASSCF/6-31G(d)) = -479.6180628

C	-0.90223954	-0.23494054	-0.50763467
C	0.51535960	-0.36941438	-0.35901748
C	0.99915286	-1.07464111	0.79439896
C	1.61613057	0.29706053	-0.94251661
C	2.76667998	0.07387063	-0.12085964
C	2.43366052	-0.84668907	0.91857573
N	-1.58003121	0.70167245	0.25614911
C	-2.97927095	0.32770285	0.53549335
H	3.09111020	-1.25490995	1.68245708
C	-1.87739332	-1.38321563	-0.79408493
C	-3.26773246	-0.83500212	-0.38611138
H	-3.79790230	-0.47697824	-1.25997432
H	-3.90652345	-1.56874884	0.09012428
H	-3.02969630	0.11113696	1.60742651
H	-3.56563100	1.22721732	0.35836855
H	-1.92304179	-1.66822500	-1.84353236
H	0.45533725	-1.70571350	1.49640654
C	-1.18454762	2.05399465	0.47920515
H	-0.10407906	2.09841142	0.46280083
H	-1.55476535	2.66585682	-0.33663638
H	-1.57069183	2.39286863	1.42503472
H	1.57870993	0.95457564	-1.78182185

C	4.12391367	0.69940951	-0.22028729
H	4.68519599	0.62662968	0.70991589
H	4.77682366	0.21595460	-0.95231857
H	4.01109047	1.74977030	-0.47585340
H	-1.62595025	-2.27401551	-0.23731138

Time = 163.00 fs
E(CASSCF/6-31G(d)) = -479.6187292

C	-0.90350047	-0.23537211	-0.50394064
C	0.51548824	-0.36869128	-0.35891084
C	0.99495271	-1.06721328	0.79858567
C	1.61848057	0.28986941	-0.94754108
C	2.76184996	0.07409663	-0.12533116
C	2.43070306	-0.83950294	0.92417950
N	-1.57696476	0.69972515	0.25788266
C	-2.97796254	0.32372128	0.53770255
H	3.07999773	-1.24223507	1.69251644
C	-1.87735549	-1.38155757	-0.80159174
C	-3.26634602	-0.83346605	-0.39031969
H	-3.79795684	-0.47128278	-1.26228351
H	-3.90034225	-1.57442540	0.08280116
H	-3.02657389	0.11314824	1.60696630
H	-3.55824711	1.22428075	0.36899515
H	-1.93306337	-1.66221966	-1.85100644
H	0.46187072	-1.68794587	1.50815833
C	-1.18305069	2.04968877	0.48413943
H	-0.09865064	2.09834510	0.46132248
H	-1.56383225	2.66796385	-0.32537740
H	-1.55616291	2.39687809	1.43545206
H	1.58735105	0.93821934	-1.78722978
C	4.12464275	0.69703982	-0.22262125
H	4.68683433	0.62719812	0.70499407
H	4.78086172	0.21506013	-0.95092332
H	4.01147949	1.74844272	-0.47929717
H	-1.62674969	-2.27605493	-0.25057811

Time = 163.50 fs
E(CASSCF/6-31G(d)) = -479.6193706

C	-0.90517192	-0.23562477	-0.49981166
C	0.51580391	-0.36808332	-0.35869718
C	0.99120552	-1.05968009	0.80305151
C	1.62108256	0.28263966	-0.95242419
C	2.75697354	0.07419129	-0.12977691
C	2.42714078	-0.83224720	0.92958919
N	-1.57388234	0.69720256	0.25927348

C	-2.97647720	0.31999050	0.54001174
H	3.06844707	-1.22939249	1.70165805
C	-1.87749625	-1.37975745	-0.80924462
C	-3.26498166	-0.83197521	-0.39454427
H	-3.79791995	-0.46558678	-1.26464807
H	-3.89388546	-1.58021704	0.07537591
H	-3.02387445	0.11453240	1.60588991
H	-3.55181767	1.22065651	0.37943282
H	-1.94180463	-1.65603292	-1.85827399
H	0.46770915	-1.66963881	1.51891546
C	-1.18128887	2.04546481	0.48907107
H	-0.09384036	2.09854158	0.46020653
H	-1.57329527	2.67025090	-0.31365797
H	-1.54164909	2.40099958	1.44597968
H	1.59582840	0.92265046	-1.79361283
C	4.12541311	0.69469263	-0.22506434
H	4.68767597	0.62803620	0.70048876
H	4.78435485	0.21459909	-0.95003723
H	4.01277859	1.74708203	-0.48290198
H	-1.62662466	-2.27777360	-0.26350097

Time = 164.00 fs
E(CASSCF/6-31G(d)) = -479.6199835

C	-0.90719740	-0.23571475	-0.49525508
C	0.51627533	-0.36756550	-0.35839898
C	0.98795902	-1.05202802	0.80777471
C	1.62388320	0.27537077	-0.95715435
C	2.75212630	0.07412103	-0.13413138
C	2.42295282	-0.82489878	0.93475376
N	-1.57080863	0.69410462	0.26031875
C	-2.97480312	0.31651156	0.54239836
H	3.05671336	-1.21642941	1.71013373
C	-1.87782567	-1.37781169	-0.81701755
C	-3.26364851	-0.83054126	-0.39877404
H	-3.79778183	-0.45990987	-1.26700650
H	-3.88720614	-1.58601609	0.06782617
H	-3.02155599	0.11507831	1.60450486
H	-3.54654218	1.21658027	0.38952232
H	-1.94911066	-1.64960873	-1.86542894
H	0.47243934	-1.65111853	1.52898739
C	-1.17923939	2.04136913	0.49401189
H	-0.08991164	2.09892989	0.45935154
H	-1.58301449	2.67251575	-0.30137607
H	-1.52717561	2.40492959	1.45641930
H	1.60408099	0.90806966	-1.80130642
C	4.12619808	0.69237976	-0.22762888

H	4.68772236	0.62909945	0.69657364
H	4.78731695	0.21449232	-0.94977263
H	4.01500492	1.74562262	-0.48665918
H	-1.62560148	-2.27914428	-0.27609638

Time = 164.50 fs
E(CASSCF/6-31G(d)) = -479.6205841

C	-0.90951316	-0.23566903	-0.49028543
C	0.51686934	-0.36709341	-0.35804150
C	0.98525117	-1.04425477	0.81272365
C	1.62680900	0.26807637	-0.96172760
C	2.74739208	0.07384468	-0.13831679
C	2.41813442	-0.81743479	0.93961503
N	-1.56776779	0.69044598	0.26101666
C	-2.97293399	0.31328106	0.54483331
H	3.04503698	-1.20334780	1.71824147
C	-1.87834919	-1.37572213	-0.82487185
C	-3.26235537	-0.82917525	-0.40299800
H	-3.79753891	-0.45427161	-1.26929914
H	-3.88037020	-1.59171945	0.06013691
H	-3.01955722	0.11459218	1.60314092
H	-3.54259298	1.21228612	0.39911626
H	-1.95483497	-1.64288391	-1.87258316
H	0.47567700	-1.63270765	1.53870501
C	-1.17688965	2.03744343	0.49897476
H	-0.08702274	2.09943409	0.45864638
H	-1.59283552	2.67455658	-0.28844402
H	-1.51277835	2.40835820	1.46657082
H	1.61206403	0.89454737	-1.81052486
C	4.12697071	0.69011133	-0.23032506
H	4.68698330	0.63033682	0.69339758
H	4.78977946	0.21465047	-0.95023369
H	4.01815538	1.74401720	-0.49056094
H	-1.62374468	-2.28012965	-0.28840393

Time = 165.00 fs
E(CASSCF/6-31G(d)) = -479.6210521

C	-0.91207508	-0.23567698	-0.48563980
C	0.51742011	-0.36724652	-0.35699471
C	0.98250664	-1.03615966	0.81750418
C	1.63007003	0.26069416	-0.96628937
C	2.74291150	0.07334124	-0.14224963
C	2.41312037	-0.80948358	0.94420620
N	-1.56499112	0.68664432	0.26196217
C	-2.97064225	0.31026037	0.54735207

H	3.03358916	-1.18993164	1.72614451
C	-1.87892848	-1.37345269	-0.83288152
C	-3.26107082	-0.82784214	-0.40727539
H	-3.79717329	-0.44861618	-1.27161158
H	-3.87342525	-1.59750449	0.05209541
H	-3.01851533	0.11257196	1.60234479
H	-3.54037318	1.20798813	0.40820208
H	-1.95932726	-1.63597310	-1.88014851
H	0.47714772	-1.61400987	1.54839167
C	-1.17435880	2.03346473	0.50391219
H	-0.08505300	2.10043600	0.45820574
H	-1.60257178	2.67736602	-0.27470280
H	-1.49798928	2.41139305	1.47666544
H	1.61986361	0.88176505	-1.82131259
C	4.12774970	0.68785286	-0.23322025
H	4.68539234	0.63171763	0.69095306
H	4.79168690	0.21498692	-0.95146470
H	4.02218947	1.74206457	-0.49460481
H	-1.62159939	-2.28145371	-0.30057032

Time = 165.50 fs
E(CASSCF/6-31G(d)) = -479.6202353

C	-0.91475501	-0.23562802	-0.48088519
C	0.51797441	-0.36782698	-0.35569785
C	0.98008027	-1.02787462	0.82225823
C	1.63349441	0.25332572	-0.97077730
C	2.73872587	0.07263998	-0.14589157
C	2.40768380	-0.80124685	0.94846382
N	-1.56244852	0.68253607	0.26282000
C	-2.96807363	0.30748713	0.54987487
H	3.02281129	-1.17683793	1.73379603
C	-1.87964317	-1.37101735	-0.84092272
C	-3.25983348	-0.82658545	-0.41152267
H	-3.79669989	-0.44305897	-1.27379677
H	-3.86651577	-1.60308427	0.04388574
H	-3.01800382	0.10905679	1.60226838
H	-3.53978985	1.20389422	0.41653660
H	-1.96219317	-1.62886098	-1.88800466
H	0.47651572	-1.59583214	1.55820409
C	-1.17159127	2.02965089	0.50883423
H	-0.08424994	2.10164091	0.45784510
H	-1.61209790	2.68002995	-0.26028248
H	-1.48320860	2.41348501	1.48622148
H	1.62725285	0.87011342	-1.83331781
C	4.12848344	0.68565044	-0.23626092
H	4.68304765	0.63316486	0.68940481

H	4.79315026	0.21538769	-0.95353539
H	4.02703108	1.73986343	-0.49874415
H	-1.61878854	-2.28252099	-0.31244038

Time = 166.00 fs
E(CASSCF/6-31G(d)) = -479.6185789

C	-0.91745172	-0.23551783	-0.47577212
C	0.51855054	-0.36864109	-0.35441529
C	0.97811495	-1.01950312	0.82704409
C	1.63690436	0.24601690	-0.97518760
C	2.73488733	0.07169038	-0.14917497
C	2.40171588	-0.79272546	0.95235723
N	-1.56012589	0.67808870	0.26341290
C	-2.96529222	0.30494863	0.55235804
H	3.01276196	-1.16392872	1.74153181
C	-1.88052230	-1.36843289	-0.84892128
C	-3.25865447	-0.82541751	-0.41573365
H	-3.79614338	-0.43760477	-1.27579456
H	-3.85971077	-1.60834178	0.03552527
H	-3.01770505	0.10403229	1.60303940
H	-3.54078413	1.20004833	0.42407287
H	-1.96316026	-1.62134391	-1.89619914
H	0.47362616	-1.57842617	1.56834638
C	-1.16855906	2.02606987	0.51379630
H	-0.08449901	2.10284738	0.45738008
H	-1.62130500	2.68209985	-0.24510383
H	-1.46856441	2.41427265	1.49507144
H	1.63420530	0.85939650	-1.84633198
C	4.12914798	0.68349814	-0.23945106
H	4.67997628	0.63461426	0.68878047
H	4.79427790	0.21575982	-0.95650646
H	4.03263018	1.73747307	-0.50299708
H	-1.61526065	-2.28310715	-0.32413875

Time = 166.50 fs
E(CASSCF/6-31G(d)) = -479.6167543

C	-0.92005535	-0.23541777	-0.47030083
C	0.51915243	-0.36955367	-0.35331942
C	0.97673638	-1.01123591	0.83186964
C	1.64015487	0.23885710	-0.97950676
C	2.73147378	0.07048744	-0.15209141
C	2.39514387	-0.78395794	0.95591915
N	-1.55808837	0.67344976	0.26372313
C	-2.96231820	0.30264115	0.55475912
H	3.00342396	-1.15085883	1.74965197

C	-1.88153814	-1.36573745	-0.85675236
C	-3.25754113	-0.82434392	-0.41989506
H	-3.79553309	-0.43226803	-1.27758574
H	-3.85312411	-1.61321997	0.02702712
H	-3.01748064	0.09744728	1.60475720
H	-3.54334076	1.19644058	0.43077014
H	-1.96203967	-1.61325323	-1.90479496
H	0.46847239	-1.56188448	1.57882197
C	-1.16529789	2.02272608	0.51880289
H	-0.08549173	2.10397728	0.45666726
H	-1.62998734	2.68351580	-0.22917510
H	-1.45416764	2.41352832	1.50317518
H	1.64069347	0.84929791	-1.85999994
C	4.12972939	0.68139008	-0.24278755
H	4.67618069	0.63599378	0.68903520
H	4.79510489	0.21599810	-0.96038503
H	4.03887933	1.73491762	-0.50737389
H	-1.61118955	-2.28328173	-0.33576914

Time = 167.00 fs
E(CASSCF/6-31G(d)) = -479.6148785

C	-0.92271434	-0.23549927	-0.46520980
C	0.51965101	-0.37064987	-0.35195649
C	0.97607498	-1.00252705	0.83713914
C	1.64363980	0.23153451	-0.98403783
C	2.72837217	0.06928894	-0.15510850
C	2.38762398	-0.77504901	0.95948618
N	-1.55631298	0.66861566	0.26436602
C	-2.95897830	0.30049007	0.55722055
H	2.99484778	-1.13767498	1.75790794
C	-1.88262670	-1.36282902	-0.86481520
C	-3.25643619	-0.82331923	-0.42411448
H	-3.79481828	-0.42692854	-1.27924257
H	-3.84669282	-1.61789749	0.01819008
H	-3.01787546	0.08890861	1.60752802
H	-3.54735292	1.19306668	0.43673130
H	-1.95946979	-1.60485195	-1.91422198
H	0.46099362	-1.54545177	1.58975243
C	-1.16182941	2.01945671	0.52389938
H	-0.08701034	2.10537092	0.45567906
H	-1.63831359	2.68475647	-0.21227433
H	-1.43951535	2.41127935	1.51086125
H	1.64688683	0.83902843	-1.87421767
C	4.13022305	0.67931685	-0.24631133
H	4.67159437	0.63729313	0.69009213
H	4.79543359	0.21587999	-0.96514435

H	4.04579589	1.73206152	-0.51201577
H	-1.60708287	-2.28304036	-0.34766406

Time = 167.50 fs
E(CASSCF/6-31G(d)) = -479.6129259

C	-0.92498720	-0.23593366	-0.46139335
C	0.52003932	-0.37181630	-0.35042604
C	0.97551358	-0.99462418	0.84171827
C	1.64625381	0.22503689	-0.98828920
C	2.72642180	0.06778177	-0.15695168
C	2.38102829	-0.76663025	0.96220219
N	-1.55516628	0.66452063	0.26541215
C	-2.95566882	0.29876549	0.55941023
H	2.98791281	-1.12537316	1.76547553
C	-1.88357699	-1.36010619	-0.87195032
C	-3.25547680	-0.82247319	-0.42785560
H	-3.79414734	-0.42224968	-1.28059872
H	-3.84139283	-1.62181446	0.01013590
H	-3.01890154	0.07950090	1.61094572
H	-3.55227166	1.19020755	0.44126723
H	-1.95578968	-1.59709159	-1.92334755
H	0.45233149	-1.53083760	1.59955629
C	-1.15869638	2.01658188	0.52840269
H	-0.08834397	2.10688398	0.45445328
H	-1.64501652	2.68608154	-0.19663435
H	-1.42651326	2.40785041	1.51736368
H	1.65203026	0.82931124	-1.88688285
C	4.13057834	0.67750063	-0.24959049
H	4.66671537	0.63826061	0.69157712
H	4.79527564	0.21547692	-0.97019232
H	4.05236355	1.72940958	-0.51631403
H	-1.60382769	-2.28282831	-0.35843972

Time = 168.00 fs
E(CASSCF/6-31G(d)) = -479.6118044

C	-0.92706845	-0.23679072	-0.45843952
C	0.52035024	-0.37315146	-0.34861958
C	0.97494172	-0.98669978	0.84613923
C	1.64825232	0.21866699	-0.99275527
C	2.72540129	0.06583825	-0.15784848
C	2.37469297	-0.75782176	0.96442040
N	-1.55452201	0.66077040	0.26698807
C	-2.95208382	0.29725860	0.56157509
H	2.98174127	-1.11258966	1.77300664
C	-1.88447611	-1.35728452	-0.87893251

C	-3.25455374	-0.82170889	-0.43152321
H	-3.79347976	-0.41771442	-1.28181635
H	-3.83671079	-1.62539110	0.00204227
H	-3.02056472	0.06839789	1.61508547
H	-3.55829382	1.18737860	0.44497191
H	-1.95072310	-1.58911936	-1.93307811
H	0.44192673	-1.51626063	1.60903703
C	-1.15558895	2.01378039	0.53280812
H	-0.08934430	2.10863656	0.45277770
H	-1.65084657	2.68759099	-0.18069333
H	-1.41380969	2.40287873	1.52352494
H	1.65668289	0.81888667	-1.89899612
C	4.13083668	0.67573358	-0.25295972
H	4.66108513	0.63896225	0.69345129
H	4.79464975	0.21470095	-0.97593044
H	4.05915294	1.72676043	-0.52073853
H	-1.60113298	-2.28256783	-0.36931691

Time = 168.50 fs
E(CASSCF/6-31G(d)) = -479.6114860

C	-0.92891018	-0.23810117	-0.45637888
C	0.52056826	-0.37463948	-0.34656905
C	0.97430246	-0.97877955	0.85042830
C	1.64960679	0.21243233	-0.99746065
C	2.72532409	0.06347942	-0.15780446
C	2.36867958	-0.74864058	0.96618240
N	-1.55439152	0.65741533	0.26912064
C	-2.94826747	0.29595742	0.56372969
H	2.97621275	-1.09924789	1.78031272
C	-1.88530794	-1.35437160	-0.88576296
C	-3.25366275	-0.82102379	-0.43511503
H	-3.79284771	-0.41331456	-1.28290904
H	-3.83272368	-1.62861012	-0.00604302
H	-3.02278512	0.05574764	1.61961336
H	-3.56511647	1.18437404	0.44792732
H	-1.94433004	-1.58090209	-1.94331084
H	0.43011180	-1.50150373	1.61789585
C	-1.15253155	2.01104496	0.53711914
H	-0.08974571	2.11058958	0.45056180
H	-1.65580901	2.68926350	-0.16457274
H	-1.40146933	2.39633241	1.52949475
H	1.66082380	0.80757196	-1.91025926
C	4.13099993	0.67400541	-0.25640380
H	4.65475033	0.63935486	0.69552437
H	4.79356029	0.21350486	-0.98222746
H	4.06603697	1.72419598	-0.52529848

H	-1.59907479	-2.28221145	-0.38034656

Time = 169.00 fs
E(CASSCF/6-31G(d)) = -479.6119796

C	-0.93047678	-0.23988117	-0.45522728
C	0.52067579	-0.37626678	-0.34429792
C	0.97353230	-0.97088774	0.85460735
C	1.65031711	0.20633513	-1.00240844
C	2.72617945	0.06073520	-0.15685182
C	2.36304959	-0.73911022	0.96753785
N	-1.55477286	0.65449081	0.27182308
C	-2.94427131	0.29484670	0.56589249
H	2.97118648	-1.08526328	1.78719632
C	-1.88605774	-1.35137749	-0.89244859
C	-3.25279894	-0.82041390	-0.43863004
H	-3.79228320	-0.40903803	-1.28389477
H	-3.82949457	-1.63145629	-0.01407045
H	-3.02547258	0.04174324	1.62413947
H	-3.57241504	1.18097335	0.45023598
H	-1.93668694	-1.57240033	-1.95389304
H	0.41725595	-1.48634856	1.62583063
C	-1.14954245	2.00836752	0.54133956
H	-0.08934038	2.11269266	0.44772852
H	-1.65993324	2.69108974	-0.14840419
H	-1.38955037	2.38821818	1.53544836
H	1.66443264	0.79524825	-1.92047281
C	4.13107319	0.67230476	-0.25990734
H	4.64778151	0.63940320	0.69759812
H	4.79200748	0.21185477	-0.98892885
H	4.07288531	1.72179083	-0.52999925
H	-1.59771645	-2.28172502	-0.39156876

Time = 169.50 fs
E(CASSCF/6-31G(d)) = -479.6132386

C	-0.93174628	-0.24213121	-0.45498469
C	0.52065249	-0.37802308	-0.34181733
C	0.97256226	-0.96304515	0.85868955
C	1.65041487	0.20037299	-1.00757910
C	2.72793218	0.05764450	-0.15505136
C	2.35786203	-0.72926003	0.96854235
N	-1.55564965	0.65201573	0.27509367
C	-2.94015275	0.29390694	0.56808322
H	2.96651725	-1.07055147	1.79347439
C	-1.88671254	-1.34831437	-0.89900101
C	-3.25195719	-0.81987384	-0.44206835

H	-3.79181655	-0.40486954	-1.28479349
H	-3.82707030	-1.63391484	-0.02199067
H	-3.02852773	0.02661287	1.62825479
H	-3.57986278	1.17696542	0.45201501
H	-1.92788707	-1.56357114	-1.96463454
H	0.40373856	-1.47059851	1.63257118
C	-1.14663214	2.00574014	0.54547384
H	-0.08800207	2.11488308	0.44421893
H	-1.66327035	2.69307051	-0.13232291
H	-1.37810032	2.37857790	1.54157423
H	1.66749143	0.78186531	-1.92955088
C	4.13106396	0.67062008	-0.26345560
H	4.64027648	0.63908134	0.69947958
H	4.78999086	0.20972886	-0.99586341
H	4.07956823	1.71960386	-0.53484046
H	-1.59710725	-2.28109229	-0.40301126

Time = 170.00 fs
E(CASSCF/6-31G(d)) = -479.6151431

C	-0.93271207	-0.24483579	-0.45563031
C	0.52047093	-0.37990452	-0.33911305
C	0.97131492	-0.95526840	0.86267081
C	1.64998035	0.19454460	-1.01292682
C	2.73051741	0.05425933	-0.15250475
C	2.35316874	-0.71912811	0.96925658
N	-1.55698893	0.64998998	0.27891160
C	-2.93597468	0.29311357	0.57031980
H	2.96206951	-1.05502646	1.79900819
C	-1.88726194	-1.34519706	-0.90543416
C	-3.25113265	-0.81939697	-0.44543123
H	-3.79147494	-0.40079162	-1.28562474
H	-3.82547905	-1.63596928	-0.02975608
H	-3.03183825	0.01061072	1.63156534
H	-3.58714784	1.17216973	0.45338975
H	-1.91803872	-1.55436883	-1.97532263
H	0.38992202	-1.45409285	1.63791174
C	-1.14380188	2.00315645	0.54952813
H	-0.08569836	2.11708214	0.43999557
H	-1.66588863	2.69520881	-0.11646180
H	-1.36715385	2.36748231	1.54805722
H	1.66998766	0.76743195	-1.93753077
C	4.13098167	0.66894041	-0.26703567
H	4.63236352	0.63837456	0.70099403
H	4.78751684	0.20711566	-1.00284986
H	4.08596134	1.71767260	-0.53981337
H	-1.59728063	-2.28031275	-0.41469039

Time = 170.50 fs
E(CASSCF/6-31G(d)) = -479.6174324

C	-0.93338876	-0.24795999	-0.45708559
C	0.52007773	-0.38192269	-0.33609678
C	0.96969849	-0.94758188	0.86650838
C	1.64923754	0.18887784	-1.01835703
C	2.73380106	0.05067570	-0.14943883
C	2.34897559	-0.70878704	0.96976313
N	-1.55873043	0.64838188	0.28320617
C	-2.93181382	0.29243603	0.57261197
H	2.95770861	-1.03855668	1.80376252
C	-1.88770248	-1.34204847	-0.91175272
C	-3.25032234	-0.81897598	-0.44872097
H	-3.79128329	-0.39678433	-1.28640183
H	-3.82472579	-1.63759482	-0.03732047
H	-3.03523677	-0.00597830	1.63370967
H	-3.59398263	1.16644655	0.45448979
H	-1.90723679	-1.54471896	-1.98572768
H	0.37613156	-1.43672016	1.64174829
C	-1.14103739	2.00061858	0.55351689
H	-0.08248448	2.11917671	0.43503860
H	-1.66786519	2.69746716	-0.10094307
H	-1.35674406	2.35500996	1.55505325
H	1.67192764	0.75198800	-1.94459107
C	4.13083783	0.66725564	-0.27063942
H	4.62421233	0.63728693	0.70200594
H	4.78462108	0.20401115	-1.00969254
H	4.09195900	1.71600599	-0.54489499
H	-1.59824316	-2.27938463	-0.42662203

Time = 171.00 fs
E(CASSCF/6-31G(d)) = -479.6188655

C	-0.93373165	-0.25141570	-0.45921611
C	0.51969771	-0.38386877	-0.33352039
C	0.96823243	-0.94004896	0.87066843
C	1.64767509	0.18315042	-1.02391109
C	2.73785319	0.04687182	-0.14578987
C	2.34491337	-0.69819277	0.97029348
N	-1.56090674	0.64720169	0.28794525
C	-2.92768072	0.29183485	0.57498908
H	2.95334383	-1.02119950	1.80758703
C	-1.88802893	-1.33882868	-0.91803011
C	-3.24951728	-0.81859654	-0.45197364
H	-3.79124514	-0.39280231	-1.28715734

H	-3.82478987	-1.63879363	-0.04473999
H	-3.03866865	-0.02310671	1.63445816
H	-3.60022051	1.15965168	0.45545104
H	-1.89534209	-1.53445543	-1.99571273
H	0.36253442	-1.41852042	1.64394547
C	-1.13828856	1.99809648	0.55749856
H	-0.07857665	2.12108987	0.42930226
H	-1.66934933	2.69986891	-0.08567377
H	-1.34672669	2.34111257	1.56279660
H	1.67318410	0.73592583	-1.95082152
C	4.13063090	0.66553649	-0.27427799
H	4.61587196	0.63575908	0.70236804
H	4.78118881	0.20035067	-1.01637789
H	4.09743444	1.71455866	-0.55018340
H	-1.59979884	-2.27845286	-0.43881798

Time = 171.50 fs
E(CASSCF/6-31G(d)) = -479.6183049

C	-0.93377289	-0.25515791	-0.46208987
C	0.51926077	-0.38579234	-0.33119065
C	0.96664900	-0.93264928	0.87495434
C	1.64540843	0.17738804	-1.02948558
C	2.74259783	0.04287797	-0.14157126
C	2.34129665	-0.68740698	0.97078786
N	-1.56343349	0.64643671	0.29312734
C	-2.92365646	0.29127711	0.57744756
H	2.94902743	-1.00312716	1.81043400
C	-1.88822268	-1.33557678	-0.92425573
C	-3.24871787	-0.81825150	-0.45516350
H	-3.79136805	-0.38886502	-1.28790056
H	-3.82565900	-1.63954725	-0.05190688
H	-3.04210735	-0.04038927	1.63363424
H	-3.60563402	1.15186774	0.45638119
H	-1.88276258	-1.52374920	-2.00504981
H	0.34946066	-1.39956007	1.64454773
C	-1.13558469	1.99559549	0.56142535
H	-0.07423531	2.12274338	0.42290340
H	-1.67041313	2.70251674	-0.07090671
H	-1.33714150	2.32610543	1.57132554
H	1.67376585	0.71946583	-1.95653438
C	4.13037427	0.66379624	-0.27790930
H	4.60761655	0.63381915	0.70198388
H	4.77727284	0.19618655	-1.02268834
H	4.10223111	1.71331510	-0.55558669
H	-1.60203378	-2.27759599	-0.45114804

Time = 172.00 fs
E(CASSCF/6-31G(d)) = -479.6169573

C	-0.93359653	-0.25881495	-0.46493870
C	0.51873639	-0.38754180	-0.32915786
C	0.96525150	-0.92601509	0.87915545
C	1.64344440	0.17211138	-1.03444048
C	2.74724946	0.03930979	-0.13810276
C	2.33766000	-0.67736815	0.97161885
N	-1.56599856	0.64594048	0.29799731
C	-2.92013256	0.29074103	0.57979805
H	2.94474983	-0.98489275	1.81270325
C	-1.88832523	-1.33253200	-0.92998701
C	-3.24798166	-0.81795389	-0.45812662
H	-3.79166149	-0.38516010	-1.28855642
H	-3.82717240	-1.63973349	-0.05840694
H	-3.04472001	-0.05649036	1.63106942
H	-3.60974969	1.14350644	0.45735569
H	-1.86988910	-1.51279326	-2.01293641
H	0.33774314	-1.38113035	1.64387675
C	-1.13295860	1.99332600	0.56518779
H	-0.06991815	2.12370238	0.41616644
H	-1.67110241	2.70476465	-0.05743813
H	-1.32859990	2.31072759	1.58008902
H	1.67369600	0.70365947	-1.96191427
C	4.13009498	0.66211373	-0.28135633
H	4.60020938	0.63165610	0.70096694
H	4.77330970	0.19173943	-1.02807683
H	4.10610747	1.71224046	-0.56075939
H	-1.60448412	-2.27675357	-0.46299383

Time = 172.50 fs
E(CASSCF/6-31G(d)) = -479.6153764

C	-0.93315853	-0.26292528	-0.46868188
C	0.51807715	-0.38943215	-0.32716300
C	0.96349237	-0.91895321	0.88371492
C	1.64078806	0.16633127	-1.03963500
C	2.75279393	0.03536160	-0.13400283
C	2.33429646	-0.66638900	0.97256658
N	-1.56898488	0.64574830	0.30360919
C	-2.91655170	0.29011635	0.58241277
H	2.94026664	-0.96458757	1.81439125
C	-1.88828823	-1.32923905	-0.93615557
C	-3.24718988	-0.81764505	-0.46127056
H	-3.79213711	-0.38118390	-1.28926290
H	-3.82953911	-1.63944581	-0.06510594

H	-3.04740938	-0.07359855	1.62661093
H	-3.61318558	1.13368922	0.45858644
H	-1.85568241	-1.50056094	-2.02056497
H	0.32586421	-1.36065016	1.64191545
C	-1.13014114	1.99089343	0.56921079
H	-0.06540454	2.12426590	0.40838138
H	-1.67159424	2.70750292	-0.04352518
H	-1.31968483	2.29331564	1.59037767
H	1.67292196	0.68661988	-1.96796676
C	4.12976002	0.66027158	-0.28505653
H	4.59276174	0.62894614	0.69905136
H	4.76862651	0.18644004	-1.03327359
H	4.10940313	1.71114325	-0.56638609
H	-1.60777988	-2.27609533	-0.47582831

Time = 173.00 fs
E(CASSCF/6-31G(d)) = -479.6129732

C	-0.93251882	-0.26710840	-0.47299233
C	0.51732198	-0.39133920	-0.32535054
C	0.96146351	-0.91196923	0.88824199
C	1.63770908	0.16045061	-1.04459629
C	2.75873501	0.03137405	-0.12968713
C	2.33149259	-0.65530526	0.97360832
N	-1.57210130	0.64583554	0.30949260
C	-2.91323450	0.28939822	0.58508587
H	2.93601950	-0.94380806	1.81557250
C	-1.88810783	-1.32595449	-0.94231493
C	-3.24640210	-0.81733531	-0.46436736
H	-3.79274870	-0.37722451	-1.28996636
H	-3.83253234	-1.63868107	-0.07147917
H	-3.04991957	-0.09027240	1.62066705
H	-3.61568801	1.12334440	0.46007419
H	-1.84138827	-1.48799381	-2.02731653
H	0.31472770	-1.33981213	1.63911582
C	-1.12732900	1.98847923	0.57320964
H	-0.06134048	2.12428080	0.40022522
H	-1.67192368	2.71054534	-0.03022089
H	-1.31096138	2.27531660	1.60136183
H	1.67148021	0.66983102	-1.97461798
C	4.12940091	0.65840566	-0.28873076
H	4.58608533	0.62591604	0.69638891
H	4.76362967	0.18067739	-1.03780573
H	4.11177253	1.71000953	-0.57201092
H	-1.61164004	-2.27577236	-0.48864531

Time = 173.50 fs

E(CASSCF/6-31G(d)) = -479.6097494

C	-0.93171384	-0.27127738	-0.47778787
C	0.51646010	-0.39329520	-0.32369481
C	0.95914044	-0.90503429	0.89267258
C	1.63431499	0.15445327	-1.04925305
C	2.76496599	0.02740002	-0.12528684
C	2.32925126	-0.64413818	0.97478736
N	-1.57527047	0.64614653	0.31557598
C	-2.91022222	0.28853677	0.58779765
H	2.93214552	-0.92268831	1.81652225
C	-1.88778573	-1.32269032	-0.94847479
C	-3.24562017	-0.81701103	-0.46742591
H	-3.79347307	-0.37327564	-1.29066132
H	-3.83606050	-1.63743531	-0.07751897
H	-3.05221016	-0.10639157	1.61346448
H	-3.61735747	1.11275006	0.46189065
H	-1.82720285	-1.47515117	-2.03322471
H	0.30425398	-1.31884157	1.63589845
C	-1.12451277	1.98608593	0.57720388
H	-0.05799883	2.12366077	0.39182431
H	-1.67215618	2.71387142	-0.01748336
H	-1.30230321	2.25691556	1.61288036
H	1.66937512	0.65347070	-1.98216516
C	4.12902541	0.65651650	-0.29237778
H	4.58041972	0.62261359	0.69305196
H	4.75841053	0.17446563	-1.04164130
H	4.11316392	1.70873770	-0.57759443
H	-1.61598876	-2.27585999	-0.50139395

Time = 174.00 fs
E(CASSCF/6-31G(d)) = -479.6056883

C	-0.93077503	-0.27533477	-0.48296668
C	0.51546115	-0.39537461	-0.32212430
C	0.95648861	-0.89810734	0.89692143
C	1.63076811	0.14833864	-1.05355005
C	2.77136653	0.02350484	-0.12096011
C	2.32755693	-0.63290537	0.97614654
N	-1.57841625	0.64661683	0.32177752
C	-2.90755267	0.28748340	0.59052380
H	2.92881118	-0.90140513	1.81753543
C	-1.88732869	-1.31945686	-0.95464429
C	-3.24484722	-0.81665801	-0.47045626
H	-3.79428159	-0.36933249	-1.29133800
H	-3.84001764	-1.63570856	-0.08322411
H	-3.05424501	-0.12187305	1.60530519

H	-3.61834429	1.10222182	0.46409408
H	-1.81330985	-1.46210489	-2.03837550
H	0.29430494	-1.29798487	1.63273186
C	-1.12168585	1.98371929	0.58121790
H	-0.05560349	2.12232865	0.38331106
H	-1.67234889	2.71741986	-0.00523503
H	-1.29356916	2.23829905	1.62471408
H	1.66660984	0.63766797	-1.99079152
C	4.12864225	0.65460447	-0.29599930
H	4.57600028	0.61909634	0.68914000
H	4.75308756	0.16782315	-1.04477487
H	4.11354952	1.70723135	-0.58309483
H	-1.62071958	-2.27639117	-0.51402491

Time = 174.50 fs
E(CASSCF/6-31G(d)) = -479.6008498

C	-0.92970681	-0.27914880	-0.48832640
C	0.51420406	-0.39775682	-0.32044623
C	0.95348737	-0.89107745	0.90092313
C	1.62748424	0.14212478	-1.05745659
C	2.77772655	0.01984529	-0.11707999
C	2.32623894	-0.62166739	0.97779813
N	-1.58144658	0.64711328	0.32796827
C	-2.90527653	0.28618712	0.59324444
H	2.92622563	-0.88030423	1.81885630
C	-1.88676502	-1.31624617	-0.96087022
C	-3.24408644	-0.81626084	-0.47347570
H	-3.79513814	-0.36538366	-1.29197301
H	-3.84426705	-1.63349484	-0.08860105
H	-3.05593157	-0.13665300	1.59650213
H	-3.61878618	1.09208425	0.46673777
H	-1.79983693	-1.44892771	-2.04290981
H	0.28469808	-1.27748812	1.63012980
C	-1.11882025	1.98139987	0.58529949
H	-0.05434174	2.12018358	0.37481156
H	-1.67261515	2.72098089	0.00665087
H	-1.28458996	2.21960321	1.63658823
H	1.66318505	0.62251016	-2.00053983
C	4.12826150	0.65267016	-0.29960660
H	4.57307506	0.61543881	0.68478399
H	4.74783469	0.16076421	-1.04719783
H	4.11295800	1.70540263	-0.58846450
H	-1.62561104	-2.27720795	-0.52649156

Time = 175.00 fs
E(CASSCF/6-31G(d)) = -479.5954708

C	-0.92860410	-0.28280933	-0.49401365
C	0.51295366	-0.39983508	-0.31934592
C	0.95058887	-0.88395754	0.90508850
C	1.62382889	0.13560531	-1.06093105
C	2.78415911	0.01630302	-0.11352586
C	2.32514798	-0.61049867	0.97988242
N	-1.58426740	0.64761997	0.33422941
C	-2.90338345	0.28458704	0.59596023
H	2.92433631	-0.85890681	1.82099293
C	-1.88607738	-1.31307722	-0.96719513
C	-3.24333069	-0.81580176	-0.47652141
H	-3.79601201	-0.36138310	-1.29257103
H	-3.84876731	-1.63079082	-0.09370773
H	-3.05735218	-0.15089628	1.58740229
H	-3.61891049	1.08258849	0.46987830
H	-1.78695306	-1.43557037	-2.04703588
H	0.27517540	-1.25732219	1.62852278
C	-1.11592318	1.97911020	0.58949531
H	-0.05420920	2.11716341	0.36627843
H	-1.67285511	2.72463077	0.01838694
H	-1.27522964	2.20088163	1.64837594
H	1.65910104	0.60759205	-2.01148196
C	4.12787075	0.65070978	-0.30319156
H	4.57177345	0.61165297	0.68001133
H	4.74248713	0.15316651	-1.04906540
H	4.11127668	1.70314184	-0.59382724
H	-1.63094358	-2.27847841	-0.53900578

Time = 175.50 fs
E(CASSCF/6-31G(d)) = -479.5897008

C	-0.92743402	-0.28597988	-0.49935817
C	0.51142501	-0.40191485	-0.31843281
C	0.94793984	-0.87677117	0.90927427
C	1.62093430	0.12910100	-1.06386740
C	2.78999700	0.01338678	-0.11132636
C	2.32377417	-0.60003146	0.98261298
N	-1.58664586	0.64783387	0.34007579
C	-2.90202911	0.28269718	0.59855643
H	2.92347271	-0.83882758	1.82390710
C	-1.88537025	-1.31002304	-0.97350224
C	-3.24261278	-0.81528196	-0.47950148
H	-3.79681929	-0.35746437	-1.29305818
H	-3.85313670	-1.62769550	-0.09835515
H	-3.05822690	-0.16397758	1.57864464
H	-3.61875164	1.07440427	0.47344162

H	-1.77517907	-1.42265226	-2.05083873
H	0.26596261	-1.23848701	1.62847535
C	-1.11303315	1.97699363	0.59372922
H	-0.05530142	2.11324228	0.35815641
H	-1.67332168	2.72769936	0.02969783
H	-1.26565735	2.18291785	1.65926596
H	1.65452930	0.59352042	-2.02291231
C	4.12749943	0.64880549	-0.30663621
H	4.57240122	0.60800244	0.67517932
H	4.73754070	0.14535174	-1.05026393
H	4.10873762	1.70048839	-0.59890687
H	-1.63601050	-2.27955180	-0.55100823

Time = 176.00 fs
E(CASSCF/6-31G(d)) = -479.5844496

C	-0.92619070	-0.28870986	-0.50444989
C	0.50960006	-0.40401530	-0.31772888
C	0.94552061	-0.86931227	0.91361170
C	1.61872904	0.12243839	-1.06636848
C	2.79534151	0.01101977	-0.11047734
C	2.32203466	-0.59000235	0.98609946
N	-1.58859942	0.64772647	0.34562584
C	-2.90117522	0.28044350	0.60107213
H	2.92362776	-0.81956464	1.82787540
C	-1.88463355	-1.30701340	-0.97995744
C	-3.24191684	-0.81467390	-0.48250736
H	-3.79755285	-0.35351117	-1.29345108
H	-3.85739304	-1.62417547	-0.10269345
H	-3.05862529	-0.17633911	1.57044396
H	-3.61852323	1.06756647	0.47754079
H	-1.76434028	-1.40991111	-2.05466347
H	0.25673857	-1.22054950	1.63024167
C	-1.11009606	1.97498731	0.59813351
H	-0.05750618	2.10833706	0.35028266
H	-1.67400561	2.73020957	0.04102570
H	-1.25551237	2.16542723	1.66935251
H	1.64937299	0.57970838	-2.03478480
C	4.12714774	0.64690440	-0.31002733
H	4.57503240	0.60445332	0.67021344
H	4.73293897	0.13713947	-1.05093947
H	4.10531632	1.69736269	-0.60385588
H	-1.64089474	-2.28043977	-0.56284046

Time = 176.50 fs
E(CASSCF/6-31G(d)) = -479.5798920

C	-0.92489762	-0.29096005	-0.50923761
C	0.50749859	-0.40612532	-0.31725050
C	0.94334925	-0.86157651	0.91810285
C	1.61723477	0.11563174	-1.06846425
C	2.80012978	0.00919591	-0.11101273
C	2.31991472	-0.58041885	0.99036001
N	-1.59008952	0.64727078	0.35084123
C	-2.90081986	0.27780952	0.60347296
H	2.92476773	-0.80115278	1.83301089
C	-1.88387561	-1.30406113	-0.98654940
C	-3.24124731	-0.81396373	-0.48554707
H	-3.79818997	-0.34951231	-1.29376085
H	-3.86144442	-1.62029343	-0.10674291
H	-3.05860124	-0.18807335	1.56324685
H	-3.61841338	1.06228758	0.48219539
H	-1.75458243	-1.39744033	-2.05875191
H	0.24753139	-1.20354267	1.63396250
C	-1.10714326	1.97308123	0.60272071
H	-0.06061087	2.10249178	0.34269879
H	-1.67488286	2.73211264	0.05249053
H	-1.24470609	2.14862898	1.67847751
H	1.64366249	0.56594515	-2.04666863
C	4.12682988	0.64500335	-0.31335623
H	4.57968369	0.60107098	0.66513115
H	4.72876408	0.12857650	-1.05120674
H	4.10106850	1.69378660	-0.60869435
H	-1.64553546	-2.28112992	-0.57451133

Time = 177.00 fs
E(CASSCF/6-31G(d)) = -479.5762269

C	-0.92357737	-0.29269696	-0.51367888
C	0.50514228	-0.40824896	-0.31699199
C	0.94141704	-0.85356517	0.92274617
C	1.61647366	0.10870015	-1.07019343
C	2.80429990	0.00789437	-0.11293278
C	2.31742464	-0.57127140	0.99539257
N	-1.59109055	0.64644775	0.35569167
C	-2.90095550	0.27478879	0.60572648
H	2.92678940	-0.78359820	1.83932574
C	-1.88310442	-1.30117673	-0.99326485
C	-3.24060850	-0.81313817	-0.48862733
H	-3.79871549	-0.34545333	-1.29400749
H	-3.86521156	-1.61612732	-0.11052693
H	-3.05821671	-0.19927866	1.55745233
H	-3.61856091	1.05869641	0.48742604
H	-1.74603564	-1.38532712	-2.06333183

H	0.23847181	-1.18743460	1.63963633
C	-1.10420978	1.97125912	0.60749871
H	-0.06433238	2.09577877	0.33542193
H	-1.67592916	2.73338026	0.06419811
H	-1.23316710	2.13273804	1.68654922
H	1.63744106	0.55202116	-2.05810025
C	4.12656354	0.64309650	-0.31661451
H	4.58630431	0.59791748	0.65990955
H	4.72508875	0.11972688	-1.05118414
H	4.09607132	1.68981381	-0.61345344
H	-1.64987255	-2.28161425	-0.58603246

Time = 177.50 fs
E(CASSCF/6-31G(d)) = -479.5735552

C	-0.92225227	-0.29389263	-0.51774022
C	0.50255502	-0.41040403	-0.31692570
C	0.93968947	-0.84528458	0.92753564
C	1.61646777	0.10166539	-1.07159899
C	2.80779105	0.00708075	-0.11620462
C	2.31459957	-0.56253445	1.00117466
N	-1.59158977	0.64524550	0.36015472
C	-2.90157038	0.27138468	0.60780460
H	2.92952398	-0.76688093	1.84673460
C	-1.88232731	-1.29836819	-1.00008998
C	-3.24000460	-0.81218480	-0.49175336
H	-3.79912201	-0.34131673	-1.29421926
H	-3.86862890	-1.61176798	-0.11407229
H	-3.05753569	-0.21004203	1.55337872
H	-3.61904023	1.05682423	0.49325595
H	-1.73881432	-1.37364960	-2.06859844
H	0.22978456	-1.17213716	1.64712945
C	-1.10133187	1.96949900	0.61247034
H	-0.06834021	2.08829501	0.32844640
H	-1.67712336	2.73400737	0.07623539
H	-1.22084223	2.11795842	1.69354793
H	1.63076723	0.53774890	-2.06862820
C	4.12636973	0.64117550	-0.31979490
H	4.59477710	0.59505015	0.65448876
H	4.72197239	0.11067371	-1.05098763
H	4.09042222	1.68552587	-0.61817408
H	-1.65384754	-2.28188985	-0.59741769

Time = 178.00 fs
E(CASSCF/6-31G(d)) = -479.5718762

C	-0.92094510	-0.29452382	-0.52139850

C	0.49976240	-0.41262623	-0.31699534
C	0.93810106	-0.83674610	0.93245189
C	1.61723926	0.09455177	-1.07272513
C	2.81054572	0.00670723	-0.12075826
C	2.31150533	-0.55416379	1.00765910
N	-1.59158829	0.64366025	0.36421591
C	-2.90264921	0.26760970	0.60968563
H	2.93274908	-0.75095841	1.85506820
C	-1.88155045	-1.29564092	-1.00701053
C	-3.23943969	-0.81109242	-0.49492857
H	-3.79940982	-0.33708272	-1.29443176
H	-3.87164552	-1.60731624	-0.11740977
H	-3.05661967	-0.22042352	1.55123645
H	-3.61985599	1.05659929	0.49971045
H	-1.73301678	-1.36247426	-2.07469766
H	0.22176423	-1.15751945	1.65619985
C	-1.09854476	1.96777350	0.61763272
H	-0.07228501	2.08015865	0.32174873
H	-1.67844912	2.73401565	0.08866750
H	-1.20769616	2.10447704	1.69952787
H	1.62371513	0.52298050	-2.07785101
C	4.12627255	0.63922960	-0.32289267
H	4.60492230	0.59251994	0.64877961
H	4.71945991	0.10152087	-1.05072612
H	4.08423763	1.68102630	-0.62290409
H	-1.65740459	-2.28196243	-0.60868214

Time = 178.50 fs
E(CASSCF/6-31G(d)) = -479.5710992

C	-0.91968155	-0.29456997	-0.52464735
C	0.49679240	-0.41498165	-0.31709813
C	0.93653593	-0.82796930	0.93743834
C	1.61880624	0.08738901	-1.07361653
C	2.81251722	0.00670819	-0.12646688
C	2.30826148	-0.54608497	1.01475931
N	-1.59110420	0.64170149	0.36787071
C	-2.90417212	0.26348481	0.61135503
H	2.93620551	-0.73576312	1.86409458
C	-1.88077722	-1.29299865	-1.01400864
C	-3.23891808	-0.80985146	-0.49815423
H	-3.79958707	-0.33273063	-1.29468712
H	-3.87422700	-1.60288001	-0.12057533
H	-3.05552786	-0.23044684	1.55111271
H	-3.62095159	1.05785057	0.50681419
H	-1.72872389	-1.35185320	-2.08171207
H	0.21474110	-1.14342079	1.66652786

C	-1.09588143	1.96604931	0.62297583
H	-0.07583044	2.07150794	0.31529617
H	-1.67989214	2.73346191	0.10153648
H	-1.19370957	2.09245972	1.70461681
H	1.61637229	0.50762257	-2.08544529
C	4.12629881	0.63724535	-0.32590791
H	4.61650176	0.59036866	0.64267392
H	4.71758403	0.09239539	-1.05049995
H	4.07765090	1.67643378	-0.62769398
H	-1.66049420	-2.28185909	-0.61984120

Time = 179.00 fs
E(CASSCF/6-31G(d)) = -479.5710617

C	-0.91850365	-0.29401211	-0.52752853
C	0.49369842	-0.41758211	-0.31706259
C	0.93477446	-0.81900278	0.94234519
C	1.62112644	0.08021907	-1.07431966
C	2.81370243	0.00697330	-0.13305660
C	2.30512532	-0.53815545	1.02230057
N	-1.59018172	0.63941380	0.37113573
C	-2.90610905	0.25903990	0.61280268
H	2.93961143	-0.72115698	1.87355053
C	-1.88000268	-1.29044925	-1.02104688
C	-3.23844508	-0.80845481	-0.50142810
H	-3.79967187	-0.32824194	-1.29503614
H	-3.87636401	-1.59857276	-0.12361180
H	-3.05433213	-0.24010403	1.55297839
H	-3.62224122	1.06031902	0.51458484
H	-1.72599832	-1.34181787	-2.08964345
H	0.20903943	-1.12965286	1.67774067
C	-1.09337720	1.96428308	0.62847752
H	-0.07868141	2.06250874	0.30905875
H	-1.68141949	2.73247138	0.11485912
H	-1.17888004	2.08205582	1.70901828
H	1.60883234	0.49164667	-2.09118340
C	4.12647756	0.63520439	-0.32884835
H	4.62921522	0.58862196	0.63605186
H	4.71636805	0.08345191	-1.05041132
H	4.07080331	1.67187840	-0.63259398
H	-1.66309746	-2.28168169	-0.63091307

Time = 179.50 fs
E(CASSCF/6-31G(d)) = -479.5715240

C	-0.91746971	-0.29294619	-0.53018233
C	0.49077767	-0.41983119	-0.31779019

C	0.93335014	-0.80970466	0.94788855
C	1.62354263	0.07269805	-1.07476838
C	2.81418720	0.00740585	-0.14054335
C	2.30178960	-0.53037974	1.03060910
N	-1.58873916	0.63672691	0.37412752
C	-2.90849371	0.25418504	0.61407718
H	2.94261571	-0.70649015	1.88352433
C	-1.87921143	-1.28795505	-1.02829288
C	-3.23800951	-0.80686482	-0.50482564
H	-3.79967194	-0.32348334	-1.29552460
H	-3.87809724	-1.59438899	-0.12662304
H	-3.05303434	-0.24952921	1.55668571
H	-3.62352468	1.06376836	0.52322853
H	-1.72495631	-1.33216931	-2.09858672
H	0.20485837	-1.11587400	1.68976802
C	-1.09099344	1.96242111	0.63424838
H	-0.08066347	2.05304435	0.30277993
H	-1.68303068	2.73111732	0.12892097
H	-1.16296799	2.07315748	1.71303928
H	1.60111021	0.47450577	-2.09512433
C	4.12681794	0.63305944	-0.33173642
H	4.64307009	0.58727958	0.62863549
H	4.71552292	0.07452418	-1.05059643
H	4.06354195	1.66732743	-0.63788669
H	-1.66543933	-2.28148333	-0.64226678

Time = 180.00 fs
E(CASSCF/6-31G(d)) = -479.5724262

C	-0.91657998	-0.29137454	-0.53245450
C	0.48802191	-0.42178843	-0.31909468
C	0.93226436	-0.80022411	0.95396428
C	1.62625619	0.06501134	-1.07498019
C	2.81382862	0.00802008	-0.14887760
C	2.29823641	-0.52293477	1.03950749
N	-1.58682908	0.63365115	0.37674729
C	-2.91126552	0.24905216	0.61517297
H	2.94494085	-0.69220598	1.89350764
C	-1.87844018	-1.28554629	-1.03562693
C	-3.23762501	-0.80511070	-0.50827357
H	-3.79959558	-0.31853693	-1.29616999
H	-3.87934863	-1.59051548	-0.12959397
H	-3.05163364	-0.25836747	1.56183429
H	-3.62452516	1.06776476	0.53256872
H	-1.72566236	-1.32316048	-2.10827422
H	0.20254986	-1.10227681	1.70205943
C	-1.08876546	1.96048087	0.64015234

H	-0.08163891	2.04343931	0.29659355
H	-1.68477302	2.72941944	0.14340013
H	-1.14632514	2.06601058	1.71681981
H	1.59350896	0.45664086	-2.09717965
C	4.12733932	0.63085100	-0.33452334
H	4.65748522	0.58640893	0.62052905
H	4.71507251	0.06596530	-1.05107354
H	4.05622732	1.66296180	-0.64348554
H	-1.66732786	-2.28111783	-0.65364870

Time = 180.50 fs
E(CASSCF/6-31G(d)) = -479.5736356

C	-0.91601221	-0.28935522	-0.53449092
C	0.48575403	-0.42374822	-0.32030176
C	0.93076549	-0.79129472	0.95951653
C	1.62878280	0.05772782	-1.07504328
C	2.81291176	0.00847571	-0.15656693
C	2.29564814	-0.51574398	1.04786061
N	-1.58472448	0.63057789	0.37895541
C	-2.91417872	0.24399298	0.61601824
H	2.94624768	-0.67870285	1.90271932
C	-1.87769081	-1.28339137	-1.04248068
C	-3.23732461	-0.80329930	-0.51156108
H	-3.79949927	-0.31368719	-1.29700298
H	-3.88014324	-1.58727215	-0.13242842
H	-3.05036357	-0.26610179	1.56784923
H	-3.62519879	1.07168148	0.54202619
H	-1.72798662	-1.31522649	-2.11790072
H	0.20231976	-1.08934698	1.71354046
C	-1.08686298	1.95849281	0.64578597
H	-0.08155455	2.03450188	0.29089670
H	-1.68640474	2.72786710	0.15742487
H	-1.12993507	2.06106559	1.72051557
H	1.58650178	0.43928354	-2.09734642
C	4.12803514	0.62866596	-0.33710268
H	4.67119057	0.58600273	0.61212342
H	4.71510001	0.05849482	-1.05195710
H	4.04942614	1.65915870	-0.64908787
H	-1.66876923	-2.28107772	-0.66444481

Time = 181.00 fs
E(CASSCF/6-31G(d)) = -479.5747033

C	-0.91574632	-0.28680619	-0.53643087
C	0.48390017	-0.42579844	-0.32148953
C	0.92877933	-0.78240543	0.96489709

C	1.63118286	0.05040888	-1.07496993
C	2.81141843	0.00875666	-0.16392630
C	2.29388272	-0.50836512	1.05608579
N	-1.58234905	0.62733960	0.38090600
C	-2.91736305	0.23874854	0.61669725
H	2.94649316	-0.66515338	1.91156393
C	-1.87692680	-1.28135140	-1.04925916
C	-3.23709395	-0.80133302	-0.51487594
H	-3.79939053	-0.30864823	-1.29808407
H	-3.88054304	-1.58451985	-0.13535278
H	-3.04914753	-0.27306405	1.57462306
H	-3.62540594	1.07539201	0.55210118
H	-1.73201139	-1.30788070	-2.12782267
H	0.20414210	-1.07627179	1.72470846
C	-1.08514533	1.95632180	0.65145141
H	-0.08055910	2.02582906	0.28538126
H	-1.68804802	2.72644622	0.17175237
H	-1.11291524	2.05795244	1.72456319
H	1.57979574	0.42155819	-2.09592949
C	4.12894872	0.62637483	-0.33964526
H	4.68470137	0.58602708	0.60296359
H	4.71552325	0.05178512	-1.05333390
H	4.04288419	1.65570607	-0.65504595
H	-1.66984266	-2.28133310	-0.67531386

Time = 181.50 fs
E(CASSCF/6-31G(d)) = -479.5757349

C	-0.91579432	-0.28375083	-0.53829100
C	0.48251086	-0.42792518	-0.32264993
C	0.92631942	-0.77356230	0.97011631
C	1.63338709	0.04304209	-1.07475820
C	2.80937636	0.00882807	-0.17083235
C	2.29294888	-0.50077838	1.06411069
N	-1.57974572	0.62394438	0.38261809
C	-2.92078172	0.23335015	0.61724308
H	2.94561136	-0.65154109	1.91996536
C	-1.87615643	-1.27941537	-1.05596551
C	-3.23693883	-0.79921886	-0.51821382
H	-3.79927696	-0.30342102	-1.29941998
H	-3.88055352	-1.58230330	-0.13843438
H	-3.04798179	-0.27912305	1.58170564
H	-3.62498786	1.07857236	0.56273702
H	-1.73770903	-1.30111222	-2.13782856
H	0.20794875	-1.06303025	1.73546725
C	-1.08357972	1.95394842	0.65712315
H	-0.07887298	2.01754051	0.28010455

H	-1.68972988	2.72522137	0.18632109
H	-1.09533051	2.05660216	1.72913588
H	1.57350274	0.40362848	-2.09327777
C	4.13008258	0.62397686	-0.34217561
H	4.69776464	0.58647708	0.59311845
H	4.71626897	0.04595143	-1.05524213
H	4.03674747	1.65258145	-0.66137251
H	-1.67053329	-2.28184054	-0.68628000

Time = 182.00 fs
E(CASSCF/6-31G(d)) = -479.5766896

C	-0.91615925	-0.28021638	-0.54009019
C	0.48162096	-0.43011901	-0.32376769
C	0.92341537	-0.76476616	0.97517613
C	1.63533749	0.03561430	-1.07440040
C	2.80682438	0.00866434	-0.17717448
C	2.29283404	-0.49296902	1.07186317
N	-1.57696086	0.62040191	0.38411381
C	-2.92439297	0.22782874	0.61769026
H	2.94361721	-0.63786346	1.92791870
C	-1.87538938	-1.27757065	-1.06260305
C	-3.23686575	-0.79696693	-0.52157036
H	-3.79916107	-0.29801491	-1.30100555
H	-3.88018251	-1.58064827	-0.14174557
H	-3.04686348	-0.28417378	1.58865547
H	-3.62382787	1.08094481	0.57385528
H	-1.74502776	-1.29490369	-2.14770415
H	0.21356041	-1.04963702	1.74580590
C	-1.08212705	1.95135764	0.66277838
H	-0.07678550	2.00975104	0.27514320
H	-1.69147281	2.72423954	0.20106686
H	-1.07725324	2.05690454	1.73433703
H	1.56772092	0.38568264	-2.08982688
C	4.13143226	0.62147486	-0.34472373
H	4.71016685	0.58734044	0.58270975
H	4.71726306	0.04108896	-1.05771418
H	4.03115537	1.64972230	-0.66806056
H	-1.67083009	-2.28253779	-0.69736377

Time = 182.50 fs
E(CASSCF/6-31G(d)) = -479.5775177

C	-0.91683572	-0.27623410	-0.54185020
C	0.48125049	-0.43237261	-0.32482355
C	0.92011316	-0.75601319	0.98007145
C	1.63698492	0.02811231	-1.07388476

C	2.80381211	0.00824829	-0.18285420
C	2.29350591	-0.48492741	1.07927205
N	-1.57404290	0.61672378	0.38541844
C	-2.92815028	0.22221437	0.61807262
H	2.94060431	-0.62412748	1.93548780
C	-1.87463681	-1.27580343	-1.06917422
C	-3.23688163	-0.79459019	-0.52494124
H	-3.79904058	-0.29244734	-1.30282416
H	-3.87944076	-1.57956194	-0.14536183
H	-3.04579254	-0.28815066	1.59506763
H	-3.62185969	1.08229200	0.58535697
H	-1.75389145	-1.28923161	-2.15724774
H	0.22069545	-1.03613900	1.75579628
C	-1.08074518	1.94853918	0.66839879
H	-0.07462153	2.00256996	0.27058345
H	-1.69329098	2.72352535	0.21592646
H	-1.05876549	2.05871055	1.74018669
H	1.56253275	0.36791974	-2.08606875
C	4.13298602	0.61887493	-0.34732404
H	4.72173815	0.58859745	0.57190796
H	4.71843414	0.03726753	-1.06077894
H	4.02623703	1.64703342	-0.67508470
H	-1.67072708	-2.28334914	-0.70858037

Time = 183.00 fs
E(CASSCF/6-31G(d)) = -479.5781630

C	-0.91781006	-0.27183908	-0.54359573
C	0.48140503	-0.43467976	-0.32579579
C	0.91647301	-0.74729614	0.98479122
C	1.63828813	0.02052395	-1.07319844
C	2.80039884	0.00757045	-0.18778446
C	2.29491528	-0.47664874	1.08626749
N	-1.57104131	0.61292360	0.38655998
C	-2.93200280	0.21653601	0.61842131
H	2.93673616	-0.61034611	1.94279812
C	-1.87391139	-1.27409859	-1.07567959
C	-3.23699358	-0.79210426	-0.52832254
H	-3.79890901	-0.28674284	-1.30484891
H	-3.87834303	-1.57903433	-0.14935929
H	-3.04477235	-0.29103698	1.60059637
H	-3.61907225	1.08246719	0.59712494
H	-1.76419979	-1.28406672	-2.16628289
H	0.22898704	-1.02260984	1.76558431
C	-1.07939213	1.94548780	0.67397177
H	-0.07270845	1.99609975	0.26651026
H	-1.69518738	2.72307653	0.23084085

H	-1.03996155	2.06183542	1.74661797
H	1.55800649	0.35053432	-2.08251275
C	4.13472498	0.61618577	-0.35001398
H	4.73235482	0.59022145	0.56092508
H	4.71971658	0.03452794	-1.06446294
H	4.02210759	1.64439706	-0.68240367
H	-1.67022323	-2.28419059	-0.71993743

Time = 183.50 fs
E(CASSCF/6-31G(d)) = -479.5785593

C	-0.91906028	-0.26707007	-0.54535416
C	0.48207555	-0.43703459	-0.32666158
C	0.91256666	-0.73860516	0.98932028
C	1.63921351	0.01283901	-1.07233090
C	2.79665202	0.00662816	-0.19188941
C	2.29699883	-0.46813242	1.09278198
N	-1.56800543	0.60901679	0.38756907
C	-2.93589627	0.21082093	0.61876329
H	2.93223203	-0.59653572	1.95002311
C	-1.87322715	-1.27243968	-1.08211729
C	-3.23720851	-0.78952705	-0.53171064
H	-3.79875654	-0.28093188	-1.30704483
H	-3.87690927	-1.57904091	-0.15381258
H	-3.04381035	-0.29287105	1.60497183
H	-3.61551137	1.08140068	0.60902736
H	-1.77582938	-1.27937370	-2.17466879
H	0.23800612	-1.00914237	1.77537240
C	-1.07802880	1.94220373	0.67949097
H	-0.07134595	1.99043451	0.26299526
H	-1.69715184	2.72286205	0.24575843
H	-1.02094841	2.06606275	1.75348276
H	1.55419726	0.33370106	-2.07963852
C	4.13662384	0.61341795	-0.35283250
H	4.74193853	0.59217977	0.55000326
H	4.72105308	0.03287917	-1.06879171
H	4.01886469	1.64168611	-0.68996461
H	-1.66932184	-2.28497471	-0.73143426

Time = 184.00 fs
E(CASSCF/6-31G(d)) = -479.5786695

C	-0.92055683	-0.26196903	-0.54715525
C	0.48323890	-0.43943136	-0.32739762
C	0.90847374	-0.72992909	0.99364165
C	1.63973527	0.00505059	-1.07127766
C	2.79264555	0.00542515	-0.19510507

C	2.29968257	-0.45938206	1.09875263
N	-1.56498370	0.60502048	0.38847841
C	-2.93977397	0.20509445	0.61912001
H	2.92734830	-0.58271159	1.95736351
C	-1.87259938	-1.27080892	-1.08848279
C	-3.23753279	-0.78687833	-0.53510237
H	-3.79857142	-0.27504884	-1.30937126
H	-3.87516595	-1.57954530	-0.15879201
H	-3.04291737	-0.29374858	1.60801207
H	-3.61127814	1.07910254	0.62092175
H	-1.78863600	-1.27511174	-2.18230806
H	0.24729228	-0.99583775	1.78539452
C	-1.07662114	1.93869262	0.68495604
H	-0.07078179	1.98565778	0.26008763
H	-1.69916100	2.72282150	0.26063625
H	-1.00184697	2.07114941	1.76056733
H	1.55115075	0.31755939	-2.07784697
C	4.13865190	0.61058291	-0.35581828
H	4.75045219	0.59443424	0.53940098
H	4.72239641	0.03229743	-1.07378877
H	4.01658525	1.63877708	-0.69770696
H	-1.66803002	-2.28561619	-0.74306009

Time = 184.50 fs
E(CASSCF/6-31G(d)) = -479.5784747

C	-0.92226327	-0.25658074	-0.54903064
C	0.48485832	-0.44186426	-0.32798092
C	0.90427772	-0.72125678	0.99773953
C	1.63983548	-0.00284394	-1.07004369
C	2.78845807	0.00397084	-0.19737958
C	2.30288529	-0.45040522	1.10412347
N	-1.56202289	0.60095328	0.38932225
C	-2.94357777	0.19937966	0.61950649
H	2.92235547	-0.56888429	1.96502136
C	-1.87204440	-1.26918722	-1.09476870
C	-3.23797184	-0.78417932	-0.53849490
H	-3.79834139	-0.26912996	-1.31178472
H	-3.87314737	-1.58050241	-0.16436095
H	-3.04210701	-0.29382229	1.60963041
H	-3.60652442	1.07566118	0.63265947
H	-1.80245728	-1.27123480	-2.18915085
H	0.25638913	-0.98279326	1.79588242
C	-1.07514173	1.93496529	0.69037168
H	-0.07119452	1.98184059	0.25780647
H	-1.70117950	2.72286686	0.27544051
H	-0.98279119	2.07683148	1.76761462

H	1.54890719	0.30220198	-2.07741653
C	4.14077450	0.60769209	-0.35900785
H	4.75789351	0.59694199	0.52937690
H	4.72371034	0.03272664	-1.07947456
H	4.01532264	1.63556259	-0.70556704
H	-1.66635789	-2.28603634	-0.75479331

Time = 185.00 fs
E(CASSCF/6-31G(d)) = -479.5779747

C	-0.92413740	-0.25095216	-0.55101343
C	0.48688445	-0.44432741	-0.32838915
C	0.90006134	-0.71257846	1.00160252
C	1.63950445	-0.01084219	-1.06864557
C	2.78417109	0.00227984	-0.19867423
C	2.30652253	-0.44121342	1.10884873
N	-1.55916763	0.59683491	0.39013581
C	-2.94724931	0.19369748	0.61993059
H	2.91751272	-0.55505543	1.97317019
C	-1.87157925	-1.26755425	-1.10096502
C	-3.23852978	-0.78145225	-0.54188574
H	-3.79805535	-0.26321105	-1.31424182
H	-3.87089659	-1.58186146	-0.17057321
H	-3.04139466	-0.29329914	1.60983898
H	-3.60144569	1.07123781	0.64409052
H	-1.81711644	-1.26769225	-2.19519641
H	0.26488252	-0.97008871	1.80702774
C	-1.07357073	1.93103721	0.69574633
H	-0.07268190	1.97903941	0.25613566
H	-1.70316334	2.72288730	0.29014524
H	-0.96392548	2.08283118	1.77435110
H	1.54750404	0.28766713	-2.07847002
C	4.14295472	0.60475601	-0.36243380
H	4.76428533	0.59965594	0.52017160
H	4.72496974	0.03408035	-1.08586367
H	4.01510427	1.63196195	-0.71348169
H	-1.66431803	-2.28616757	-0.76660086

Time = 185.50 fs
E(CASSCF/6-31G(d)) = -479.5771756

C	-0.92613251	-0.24513175	-0.55313759
C	0.48925652	-0.44681490	-0.32860107
C	0.89590248	-0.70388705	1.00522686
C	1.63874108	-0.01893668	-1.06711234
C	2.77986708	0.00037140	-0.19896398
C	2.31051017	-0.43182189	1.11289576

N	-1.55645980	0.59268576	0.39095456
C	-2.95073130	0.18806678	0.62039273
H	2.91304334	-0.54121512	1.98192501
C	-1.87122126	-1.26588859	-1.10705944
C	-3.23920910	-0.77871986	-0.54527260
H	-3.79770457	-0.25732527	-1.31670195
H	-3.86846592	-1.58356895	-0.17747101
H	-3.04079677	-0.29243475	1.60874730
H	-3.59627068	1.06605558	0.65506818
H	-1.83242615	-1.26442950	-2.20049071
H	0.27243510	-0.95777451	1.81894298
C	-1.07189639	1.92692765	0.70109074
H	-0.07525610	1.97729495	0.25502096
H	-1.70506409	2.72275552	0.30473071
H	-0.94540068	2.08886428	1.78051315
H	1.54697720	0.27393856	-2.08096221
C	4.14515519	0.60178352	-0.36612290
H	4.76966524	0.60252526	0.51198964
H	4.72615959	0.03624483	-1.09296225
H	4.01593012	1.62792941	-0.72139220
H	-1.66192497	-2.28595617	-0.77843855

Time = 186.00 fs
E(CASSCF/6-31G(d)) = -479.5760755

C	-0.92819870	-0.23916873	-0.55543716
C	0.49190380	-0.44932099	-0.32859666
C	0.89187096	-0.69517903	1.00861900
C	1.63755341	-0.02711523	-1.06548428
C	2.77562782	-0.00173125	-0.19823821
C	2.31476723	-0.42224934	1.11624772
N	-1.55393819	0.58852651	0.39181363
C	-2.95396890	0.18250493	0.62088597
H	2.90911478	-0.52734029	1.99131625
C	-1.87098770	-1.26416796	-1.11303797
C	-3.24001045	-0.77600507	-0.54865325
H	-3.79728384	-0.25150115	-1.31912967
H	-3.86591687	-1.58557106	-0.18508327
H	-3.04033015	-0.29152510	1.60655564
H	-3.59124795	1.06038348	0.66545340
H	-1.84819254	-1.26138885	-2.20512115
H	0.27881150	-0.94586290	1.83162971
C	-1.07011508	1.92265865	0.70641644
H	-0.07884491	1.97663140	0.25436963
H	-1.70683417	2.72233566	0.31918113
H	-0.92736857	2.09464766	1.78587102
H	1.54735969	0.26095140	-2.08468872

C	4.14733989	0.59878130	-0.37009474
H	4.77407480	0.60549562	0.50498298
H	4.72727355	0.03908319	-1.10076499
H	4.01777214	1.62345951	-0.72924714
H	-1.65919469	-2.28536412	-0.79025190

Time = 186.50 fs
E(CASSCF/6-31G(d)) = -479.5746526

C	-0.93028436	-0.23311232	-0.55794549
C	0.49474725	-0.45184028	-0.32835723
C	0.88802658	-0.68645486	1.01179689
C	1.63595932	-0.03536181	-1.06381018
C	2.77153266	-0.00400135	-0.19650152
C	2.31921789	-0.41251775	1.11890553
N	-1.55163818	0.58437763	0.39274707
C	-2.95691127	0.17702847	0.62139671
H	2.90582648	-0.51339488	2.00127277
C	-1.87089528	-1.26236948	-1.11888570
C	-3.24093245	-0.77333051	-0.55202550
H	-3.79679216	-0.24576099	-1.32149655
H	-3.86331950	-1.58781550	-0.19342455
H	-3.04001137	-0.29089504	1.60354208
H	-3.58662990	1.05451500	0.67511859
H	-1.86421927	-1.25851059	-2.20920840
H	0.28389021	-0.93432442	1.84496092
C	-1.06823083	1.91825380	0.71173445
H	-0.08329841	1.97705618	0.25405252
H	-1.70843250	2.72149208	0.33348230
H	-0.90997581	2.09990687	1.79024850
H	1.54867811	0.24860312	-2.08931465
C	4.14947574	0.59575349	-0.37436056
H	4.77755024	0.60850923	0.49923781
H	4.72831165	0.04244024	-1.10925190
H	4.02057463	1.61858923	-0.73700440
H	-1.65614446	-2.28436953	-0.80197759

Time = 187.00 fs
E(CASSCF/6-31G(d)) = -479.5728692

C	-0.93233761	-0.22701079	-0.56069434
C	0.49770133	-0.45436802	-0.32786507
C	0.88441836	-0.67771885	1.01478945
C	1.63398720	-0.04365756	-1.06214335
C	2.76765702	-0.00640914	-0.19377455
C	2.32379246	-0.40265190	1.12088883
N	-1.54959137	0.58025885	0.39378706

C	-2.95951316	0.17165402	0.62190617
H	2.90320755	-0.49933169	2.01161762
C	-1.87095979	-1.26047007	-1.12458767
C	-3.24197166	-0.77071822	-0.55538710
H	-3.79623311	-0.24011965	-1.32378236
H	-3.86075111	-1.59025275	-0.20249443
H	-3.03985611	-0.29088331	1.60004208
H	-3.58265487	1.04874256	0.68395073
H	-1.88031127	-1.25573428	-2.21289582
H	0.28766023	-0.92309029	1.85868235
C	-1.06625439	1.91373705	0.71705409
H	-0.08840155	1.97856018	0.25390903
H	-1.70983003	2.72009804	0.34761953
H	-0.89335774	2.10438308	1.79353718
H	1.55094777	0.23676724	-2.09441700
C	4.15153383	0.59270162	-0.37892272
H	4.78011574	0.61150486	0.49476622
H	4.72927769	0.04614790	-1.11838540
H	4.02425574	1.61339675	-0.74463218
H	-1.65279279	-2.28296597	-0.81354538

Time = 187.50 fs
E(CASSCF/6-31G(d)) = -479.5706862

C	-0.93430775	-0.22091066	-0.56371286
C	0.50067587	-0.45690039	-0.32710307
C	0.88108508	-0.66897848	1.01763437
C	1.63167659	-0.05198197	-1.06053725
C	2.76407106	-0.00892229	-0.19009490
C	2.32842738	-0.39267897	1.12223567
N	-1.54782530	0.57618866	0.39496310
C	-2.96173647	0.16639895	0.62239250
H	2.90122415	-0.48509608	2.02207790
C	-1.87119573	-1.25844686	-1.13012995
C	-3.24312257	-0.76818935	-0.55873576
H	-3.79561472	-0.23458397	-1.32597559
H	-3.85829451	-1.59283669	-0.21227748
H	-3.03987853	-0.29182413	1.59642018
H	-3.57952889	1.04332977	0.69185350
H	-1.89627786	-1.25300014	-2.21633719
H	0.29020421	-0.91206017	1.87243276
C	-1.06420190	1.90913152	0.72238194
H	-0.09389245	1.98111817	0.25375515
H	-1.71101468	2.71804384	0.36157617
H	-0.87763246	2.10783945	1.79570548
H	1.55416793	0.22530764	-2.09953326
C	4.15349023	0.58962467	-0.38377481

H	4.78178061	0.61441784	0.49150422
H	4.73017641	0.05003079	-1.12810793
H	4.02871002	1.60799688	-0.75210894
H	-1.64915966	-2.28116039	-0.82488037

Time = 188.00 fs
E(CASSCF/6-31G(d)) = -479.5680857

C	-0.93614651	-0.21485576	-0.56702650
C	0.50357795	-0.45943486	-0.32605392
C	0.87805653	-0.66024339	1.02037447
C	1.62907862	-0.06031384	-1.05904120
C	2.76083854	-0.01150631	-0.18551793
C	2.33306431	-0.38262793	1.12300125
N	-1.54636322	0.57218377	0.39630113
C	-2.96355174	0.16128192	0.62283365
H	2.89979460	-0.47063060	2.03230720
C	-1.87161597	-1.25627770	-1.13550069
C	-3.24437778	-0.76576385	-0.56206920
H	-3.79494894	-0.22915263	-1.32807320
H	-3.85603568	-1.59552453	-0.22274378
H	-3.04009052	-0.29402607	1.59303486
H	-3.57740888	1.03848480	0.69874856
H	-1.91193529	-1.25025052	-2.21968366
H	0.29167276	-0.90111338	1.88577913
C	-1.06209297	1.90445853	0.72772082
H	-0.09948606	1.98468926	0.25339476
H	-1.71199515	2.71524372	0.37533293
H	-0.86289551	2.11006641	1.79680243
H	1.55831803	0.21409143	-2.10420876
C	4.15532620	0.58651950	-0.38890231
H	4.78254073	0.61718049	0.48931616
H	4.73101078	0.05391169	-1.13834046
H	4.03381207	1.60253343	-0.75942224
H	-1.64526679	-2.27897007	-0.83590557

Time = 188.50 fs
E(CASSCF/6-31G(d)) = -479.5650882

C	-0.93780928	-0.20888645	-0.57065568
C	0.50631363	-0.46197055	-0.32469906
C	0.87535510	-0.65152418	1.02305323
C	1.62625619	-0.06863200	-1.05769677
C	2.75801592	-0.01412489	-0.18011766
C	2.33764870	-0.37252911	1.12325578
N	-1.54522383	0.56825855	0.39782257
C	-2.96493919	0.15632289	0.62321058

H	2.89880903	-0.45588013	2.04191693
C	-1.87223156	-1.25394163	-1.14069107
C	-3.24572818	-0.76346029	-0.56538526
H	-3.79425079	-0.22381671	-1.33007968
H	-3.85406112	-1.59827621	-0.23384984
H	-3.04050067	-0.29774968	1.59019950
H	-3.57638957	1.03433828	0.70457629
H	-1.92710856	-1.24743142	-2.22307114
H	0.29225517	-0.89012150	1.89825985
C	-1.05994851	1.89973677	0.73306890
H	-0.10490202	1.98921695	0.25263383
H	-1.71280350	2.71164072	0.38886807
H	-0.84921563	2.11088598	1.79695680
H	1.56335577	0.20299931	-2.10803803
C	4.15702798	0.58338143	-0.39428387
H	4.78238372	0.61972283	0.48800526
H	4.73177960	0.05761668	-1.14898219
H	4.03942103	1.59716851	-0.76656668
H	-1.64113813	-2.27641864	-0.84654464

Time = 189.00 fs
E(CASSCF/6-31G(d)) = -479.5617574

C	-0.93925595	-0.20303885	-0.57461447
C	0.50878944	-0.46450858	-0.32301724
C	0.87299714	-0.64283133	1.02571018
C	1.62328402	-0.07691588	-1.05653509
C	2.75565180	-0.01674029	-0.17398738
C	2.34212820	-0.36241362	1.12308189
N	-1.54442104	0.56442451	0.39954332
C	-2.96588938	0.15154249	0.62351036
H	2.89814932	-0.44079699	2.05051179
C	-1.87305149	-1.25141937	-1.14569620
C	-3.24716324	-0.76129552	-0.56868207
H	-3.79353686	-0.21856085	-1.33200531
H	-3.85245469	-1.60105329	-0.24553982
H	-3.04111311	-0.30318657	1.58814413
H	-3.57649605	1.03092936	0.70929604
H	-1.94163270	-1.24449376	-2.22660925
H	0.29215151	-0.87895998	1.90942919
C	-1.05778834	1.89498180	0.73841891
H	-0.10989420	1.99462871	0.25129673
H	-1.71349598	2.70720966	0.40215826
H	-0.83663163	2.11015486	1.79637175
H	1.56921765	0.19193308	-2.11069761
C	4.15858614	0.58020506	-0.39989299
H	4.78129735	0.62197392	0.48732881

H	4.73247560	0.06097966	-1.15991181
H	4.04538563	1.59206949	-0.77354108
H	-1.63680041	-2.27353145	-0.85672427

Time = 189.50 fs
E(CASSCF/6-31G(d)) = -479.5581780

C	-0.94045122	-0.19734431	-0.57890902
C	0.51091329	-0.46705302	-0.32098203
C	0.87099431	-0.63417396	1.02837628
C	1.62024957	-0.08514551	-1.05557507
C	2.75378610	-0.01931347	-0.16724138
C	2.34645019	-0.35231314	1.12257262
N	-1.54396392	0.56068999	0.40147280
C	-2.96640334	0.14696091	0.62372873
H	2.89770949	-0.42534473	2.05772362
C	-1.87408275	-1.24869374	-1.15051591
C	-3.24867133	-0.75928457	-0.57195815
H	-3.79282421	-0.21336447	-1.33386453
H	-3.85129517	-1.60381801	-0.25774731
H	-3.04192658	-0.31044226	1.58698561
H	-3.57768347	1.02820282	0.71288669
H	-1.95535361	-1.24139491	-2.23037365
H	0.29154899	-0.86751708	1.91889532
C	-1.05562894	1.89020597	0.74375754
H	-0.11427831	2.00083515	0.24924288
H	-1.71415254	2.70195789	0.41518093
H	-0.82515014	2.10776591	1.79531480
H	1.57582001	0.18082023	-2.11196920
C	4.15999466	0.57698521	-0.40569995
H	4.77927836	0.62386327	0.48701659
H	4.73308537	0.06384587	-1.17098959
H	4.05155039	1.58739518	-0.78034461
H	-1.63228317	-2.27033083	-0.86637679

Time = 190.00 fs
E(CASSCF/6-31G(d)) = -479.5544515

C	-0.94136463	-0.19182899	-0.58353531
C	0.51259450	-0.46961163	-0.31855853
C	0.86935402	-0.62555893	1.03106999
C	1.61725442	-0.09330116	-1.05482276
C	2.75244937	-0.02180431	-0.16001638
C	2.35055978	-0.34225974	1.12182899
N	-1.54385665	0.55705956	0.40361254
C	-2.96649204	0.14259644	0.62387136
H	2.89740947	-0.40950229	2.06324278

C	-1.87533025	-1.24574997	-1.15515534
C	-3.25024010	-0.75744032	-0.57521265
H	-3.79212857	-0.20820368	-1.33567334
H	-3.85065299	-1.60653191	-0.27039697
H	-3.04293286	-0.31952617	1.58671022
H	-3.57984305	1.02601658	0.71534694
H	-1.96812892	-1.23809955	-2.23440159
H	0.29060454	-0.85570005	1.92635265
C	-1.05348153	1.88541853	0.74906551
H	-0.11795352	2.00772829	0.24638187
H	-1.71487441	2.69592302	0.42791644
H	-0.81474561	2.10364829	1.79410346
H	1.58306399	0.16961611	-2.11175459
C	4.16124988	0.57371803	-0.41167381
H	4.77634266	0.62532310	0.48678994
H	4.73358900	0.06607501	-1.18206136
H	4.05776123	1.58328096	-0.78697340
H	-1.62761878	-2.26683077	-0.87544176

Time = 190.50 fs
E(CASSCF/6-31G(d)) = -479.5506603

C	-0.94184959	-0.18611290	-0.58841108
C	0.51358438	-0.47215694	-0.31600753
C	0.86867729	-0.61589830	1.03477095
C	1.61459591	-0.10220547	-1.05418725
C	2.75118795	-0.02428481	-0.15271385
C	2.35387194	-0.33164836	1.12149094
N	-1.54394930	0.55300372	0.40592945
C	-2.96623055	0.13803439	0.62399414
H	2.89717715	-0.39203513	2.06726901
C	-1.87697860	-1.24224377	-1.16024586
C	-3.25199522	-0.75559635	-0.57876048
H	-3.79135231	-0.20255135	-1.33757493
H	-3.85042465	-1.60937461	-0.28460868
H	-3.04399800	-0.33118613	1.58709140
H	-3.58285220	1.02395382	0.71689012
H	-1.98119044	-1.23430982	-2.23905197
H	0.28938523	-0.84270621	1.93223323
C	-1.05107203	1.88024897	0.75488649
H	-0.12125958	2.01561880	0.24241966
H	-1.71609117	2.68822276	0.44152205
H	-0.80448048	2.09714154	1.79275984
H	1.59164749	0.15709845	-2.11009763
C	4.16245854	0.57011208	-0.41833659
H	4.77231420	0.62642799	0.48637207
H	4.73390717	0.06767966	-1.19395969

H	4.06445602	1.57941846	-0.79402514
H	-1.62219689	-2.26212197	-0.88470279

Time = 191.00 fs
E(CASSCF/6-31G(d)) = -479.5469701

C	-0.94185038	-0.18074245	-0.59311210
C	0.51376181	-0.47441741	-0.31364865
C	0.86907923	-0.60603229	1.03923346
C	1.61250302	-0.11109593	-1.05370150
C	2.75017104	-0.02651198	-0.14607089
C	2.35603784	-0.32143143	1.12169736
N	-1.54422732	0.54888519	0.40822773
C	-2.96565251	0.13368892	0.62410894
H	2.89699601	-0.37441825	2.06924665
C	-1.87889163	-1.23845953	-1.16534964
C	-3.25377147	-0.75392875	-0.58229338
H	-3.79057767	-0.19687001	-1.33941728
H	-3.85071086	-1.61205330	-0.29906626
H	-3.04501728	-0.34430362	1.58786578
H	-3.58626728	1.02194020	0.71737903
H	-1.99323923	-1.23032406	-2.24390335
H	0.28807408	-0.82966707	1.93583303
C	-1.04861464	1.87515296	0.76067738
H	-0.12396008	2.02368653	0.23769875
H	-1.71783410	2.67962447	0.45478892
H	-0.79521719	2.08874065	1.79182011
H	1.60072245	0.14435971	-2.10722641
C	4.16350519	0.56647719	-0.42508121
H	4.76757105	0.62699453	0.48552282
H	4.73395723	0.06837313	-1.20547888
H	4.07087664	1.57623560	-0.80087341
H	-1.61654724	-2.25663824	-0.89329822

Time = 191.50 fs
E(CASSCF/6-31G(d)) = -479.5438498

C	-0.94138902	-0.17574876	-0.59765891
C	0.51315793	-0.47635605	-0.31157388
C	0.87055395	-0.59597436	1.04447394
C	1.61092070	-0.11999006	-1.05332189
C	2.74945053	-0.02847569	-0.14013584
C	2.35706934	-0.31161245	1.12251116
N	-1.54467478	0.54472211	0.41052051
C	-2.96478499	0.12956329	0.62424327
H	2.89686738	-0.35662269	2.06917796
C	-1.88105957	-1.23439090	-1.17046710

C	-3.25555477	-0.75244374	-0.58581235
H	-3.78981876	-0.19113378	-1.34121393
H	-3.85156549	-1.61452903	-0.31366879
H	-3.04598577	-0.35863700	1.58870654
H	-3.58987118	1.01970002	0.71687923
H	-2.00415562	-1.22612419	-2.24891527
H	0.28670571	-0.81661584	1.93718923
C	-1.04611611	1.87012872	0.76639276
H	-0.12621206	2.03177926	0.23231188
H	-1.72021322	2.67030781	0.46771568
H	-0.78685609	2.07849835	1.79171312
H	1.61015822	0.13144700	-2.10339067
C	4.16438527	0.56281398	-0.43187730
H	4.76219956	0.62696090	0.48401195
H	4.73369430	0.06806224	-1.21646170
H	4.07686417	1.57374865	-0.80750481
H	-1.61075429	-2.25047155	-0.90120731

Time = 192.00 fs
E(CASSCF/6-31G(d)) = -479.5413668

C	-0.94049586	-0.17115493	-0.60207796
C	0.51182048	-0.47794794	-0.30984695
C	0.87307753	-0.58573797	1.05047091
C	1.60979241	-0.12890530	-1.05299621
C	2.74907104	-0.03017088	-0.13494219
C	2.35699402	-0.30218515	1.12396958
N	-1.54527639	0.54053418	0.41282304
C	-2.96365544	0.12565437	0.62442495
H	2.89683936	-0.33863325	2.06719412
C	-1.88346853	-1.23003347	-1.17559731
C	-3.25733190	-0.75114477	-0.58931911
H	-3.78908636	-0.18532095	-1.34297322
H	-3.85303167	-1.61676373	-0.32831514
H	-3.04690262	-0.37389922	1.58925479
H	-3.59345632	1.01697380	0.71547731
H	-2.01383086	-1.22169724	-2.25404126
H	0.28526284	-0.80360415	1.93648023
C	-1.04358243	1.86517032	0.77198481
H	-0.12823027	2.03974417	0.22640413
H	-1.72332373	2.66046916	0.48031938
H	-0.77927914	2.06649739	1.79283622
H	1.61983096	0.11842961	-2.09893071
C	4.16509281	0.55912638	-0.43869948
H	4.75631658	0.62627372	0.48166166
H	4.73307844	0.06667319	-1.22676273
H	4.08228022	1.57192086	-0.81389735

H	-1.60490925	-2.24373021	-0.90841829

Time = 192.50 fs
E(CASSCF/6-31G(d)) = -479.5395411

C	-0.93920149	-0.16697702	-0.60638552
C	0.50979542	-0.47918258	-0.30849066
C	0.87661546	-0.57533147	1.05716887
C	1.60908836	-0.13785404	-1.05266998
C	2.74905925	-0.03159459	-0.13053380
C	2.35583644	-0.29313933	1.12609469
N	-1.54601576	0.53633818	0.41514398
C	-2.96229212	0.12195228	0.62467940
H	2.89701019	-0.32045766	2.06355095
C	-1.88610218	-1.22538428	-1.18074207
C	-3.25909053	-0.75003211	-0.59281621
H	-3.78838595	-0.17941522	-1.34469558
H	-3.85513679	-1.61871870	-0.34290572
H	-3.04776465	-0.38977528	1.58915333
H	-3.59683485	1.01353791	0.71327976
H	-2.02217272	-1.21703546	-2.25923351
H	0.28367772	-0.79070039	1.93402086
C	-1.04101806	1.86027037	0.77740940
H	-0.13026337	2.04742643	0.22016240
H	-1.72724302	2.65029752	0.49263868
H	-0.77236102	2.05283843	1.79550997
H	1.62962805	0.10538692	-2.09425230
C	4.16562002	0.55542210	-0.44552789
H	4.75007369	0.62489061	0.47835570
H	4.73207822	0.06415060	-1.23625248
H	4.08701334	1.57066463	-0.82002060
H	-1.59910278	-2.23651597	-0.91492825

Time = 193.00 fs
E(CASSCF/6-31G(d)) = -479.5383176

C	-0.93753343	-0.16322689	-0.61058031
C	0.50712184	-0.48005901	-0.30749393
C	0.88113114	-0.56475576	1.06449013
C	1.60881045	-0.14684296	-1.05229130
C	2.74941981	-0.03274592	-0.12697119
C	2.35360919	-0.28446441	1.12889823
N	-1.54687379	0.53214617	0.41748222
C	-2.96072378	0.11844051	0.62502735
H	2.89751880	-0.30212429	2.05861477
C	-1.88894246	-1.22044216	-1.18590789
C	-3.26081940	-0.74910247	-0.59630708

H	-3.78771666	-0.17340778	-1.34637210
H	-3.85788907	-1.62035442	-0.35734422
H	-3.04856414	-0.40594234	1.58807926
H	-3.59984867	1.00922187	0.71041109
H	-2.02911203	-1.21213793	-2.26444820
H	0.28184070	-0.77798846	1.93023947
C	-1.03842670	1.85542232	0.78263131
H	-0.13256650	2.05467079	0.21380123
H	-1.73202331	2.63995910	0.50473509
H	-0.76598295	2.03763076	1.79993718
H	1.63945393	0.09239618	-2.08979215
C	4.16595690	0.55171225	-0.45234794
H	4.74365568	0.62278172	0.47404383
H	4.73067056	0.06045660	-1.24482305
H	4.09097908	1.56984807	-0.82583951
H	-1.59342368	-2.22891094	-0.92074389

Time = 193.50 fs
E(CASSCF/6-31G(d)) = -479.5376487

C	-0.93551649	-0.15991336	-0.61464440
C	0.50383321	-0.48058208	-0.30682116
C	0.88658645	-0.55400572	1.07234278
C	1.60898604	-0.15587371	-1.05181423
C	2.75013687	-0.03362725	-0.12432546
C	2.35031560	-0.27614927	1.13238125
N	-1.54782803	0.52796602	0.41982763
C	-2.95897875	0.11509644	0.62548288
H	2.89853503	-0.28367726	2.05284004
C	-1.89196942	-1.21520835	-1.19110500
C	-3.26250844	-0.74834905	-0.59979601
H	-3.78707192	-0.16729764	-1.34798476
H	-3.86127775	-1.62163126	-0.37153864
H	-3.04928970	-0.42208808	1.58576985
H	-3.60237783	1.00392018	0.70701121
H	-2.03460327	-1.20700937	-2.26965053
H	0.27961272	-0.76556345	1.92564563
C	-1.03581327	1.85062137	0.78762768
H	-0.13537556	2.06132756	0.20754637
H	-1.73768276	2.62958979	0.51669246
H	-0.76004498	2.02098819	1.80617505
H	1.64922743	0.07952306	-2.08597242
C	4.16609158	0.54801039	-0.45914968
H	4.73727522	0.61993038	0.46874103
H	4.72884134	0.05557002	-1.25239276
H	4.09411968	1.56930594	-0.83131868
H	-1.58796060	-2.22097474	-0.92588342

Time = 194.00 fs
E(CASSCF/6-31G(d)) = -479.5374764

C	-0.93317503	-0.15704180	-0.61854824
C	0.49996179	-0.48076074	-0.30641847
C	0.89293640	-0.54307365	1.08062538
C	1.60965837	-0.16494347	-1.05120239
C	2.75117761	-0.03424576	-0.12266600
C	2.34595990	-0.26818018	1.13653104
N	-1.54885346	0.52380272	0.42216370
C	-2.95708363	0.11189226	0.62605191
H	2.90024050	-0.26516964	2.04673689
C	-1.89516088	-1.20968716	-1.19634522
C	-3.26414876	-0.74776157	-0.60328793
H	-3.78644067	-0.16109077	-1.34950735
H	-3.86527503	-1.62251072	-0.38540255
H	-3.04992922	-0.43792654	1.58204126
H	-3.60434623	0.99759862	0.70323208
H	-2.03862421	-1.20165865	-2.27481955
H	0.27684857	-0.75352137	1.92078755
C	-1.03318610	1.84586406	0.79238994
H	-0.13888668	2.06726167	0.20161789
H	-1.74419781	2.61929088	0.52861599
H	-0.75447426	2.00302872	1.81412226
H	1.65887841	0.06681313	-2.08315654
C	4.16601084	0.54433134	-0.46592710
H	4.73116518	0.61633324	0.46252281
H	4.72658659	0.04948578	-1.25891028
H	4.09640332	1.56885417	-0.83642805
H	-1.58280363	-2.21274775	-0.93037728

Time = 194.50 fs
E(CASSCF/6-31G(d)) = -479.5376859

C	-0.93053500	-0.15461440	-0.62225684
C	0.49554402	-0.48060653	-0.30621922
C	0.90012320	-0.53195321	1.08923191
C	1.61087659	-0.17404540	-1.05043127
C	2.75249662	-0.03461423	-0.12204830
C	2.34055764	-0.26053918	1.14131854
N	-1.54992370	0.51966020	0.42447101
C	-2.95506213	0.10879621	0.62673145
H	2.90280439	-0.24665643	2.04082393
C	-1.89849197	-1.20388639	-1.20163999
C	-3.26573291	-0.74732643	-0.60678827
H	-3.78580921	-0.15480050	-1.35090773

H	-3.86983781	-1.62295754	-0.39885595
H	-3.05047321	-0.45321284	1.57680215
H	-3.60572716	0.99029774	0.69923419
H	-2.04117532	-1.19609832	-2.27995133
H	0.27342416	-0.74194480	1.91619788
C	-1.03055891	1.84114755	0.79692413
H	-0.14323803	2.07235985	0.19621473
H	-1.75149784	2.60913022	0.54062776
H	-0.74922799	1.98387763	1.82352518
H	1.66834483	0.05428497	-2.08160831
C	4.16570065	0.54068965	-0.47267702
H	4.72556678	0.61199910	0.45551589
H	4.72391173	0.04221420	-1.26435678
H	4.09782146	1.56830403	-0.84114780
H	-1.57804637	-2.20425646	-0.93426759

Time = 195.00 fs
E(CASSCF/6-31G(d)) = -479.5381419

C	-0.92762580	-0.15262960	-0.62573390
C	0.49062435	-0.48013271	-0.30614954
C	0.90807003	-0.52064249	1.09805775
C	1.61268848	-0.18316940	-1.04948998
C	2.75403902	-0.03475119	-0.12250646
C	2.33414507	-0.25320269	1.14669955
N	-1.55101189	0.51554213	0.42672933
C	-2.95293440	0.10577378	0.62750938
H	2.90635418	-0.22819207	2.03557704
C	-1.90193512	-1.19781733	-1.20699891
C	-3.26725484	-0.74702701	-0.61030274
H	-3.78516333	-0.14844567	-1.35215040
H	-3.87491018	-1.62294172	-0.41182633
H	-3.05091706	-0.46775375	1.57005989
H	-3.60654401	0.98213048	0.69518273
H	-2.04227818	-1.19034291	-2.28506064
H	0.26927014	-0.73088969	1.91234141
C	-1.02795210	1.83646851	0.80125002
H	-0.14849917	2.07653758	0.19150132
H	-1.75946540	2.59914443	0.55286206
H	-0.74429048	1.96367189	1.83400044
H	1.67756843	0.04192545	-2.08145952
C	4.16514719	0.53709839	-0.47939795
H	4.72071787	0.60694793	0.44788479
H	4.72083098	0.03378032	-1.26874662
H	4.09838722	1.56747705	-0.84547307
H	-1.57378547	-2.19551911	-0.93760762

Time = 195.50 fs
E(CASSCF/6-31G(d)) = -479.5386516

C	-0.92448145	-0.15108212	-0.62894442
C	0.48525814	-0.47935327	-0.30613388
C	0.91667585	-0.50914638	1.10700596
C	1.61513507	-0.19230296	-1.04838133
C	2.75574295	-0.03468039	-0.12404937
C	2.32678653	-0.24614099	1.15261809
N	-1.55209127	0.51145234	0.42891896
C	-2.95071678	0.10278913	0.62836478
H	2.91094486	-0.20983063	2.03137821
C	-1.90546022	-1.19149455	-1.21242907
C	-3.26870983	-0.74684384	-0.61383724
H	-3.78449045	-0.14204878	-1.35319912
H	-3.88042581	-1.62244050	-0.42424904
H	-3.05126216	-0.48141496	1.56192115
H	-3.60686752	0.97327545	0.69124352
H	-2.04197379	-1.18440775	-2.29018021
H	0.26440388	-0.72037397	1.90956922
C	-1.02539340	1.83182209	0.80539906
H	-0.15466538	2.07974345	0.18759774
H	-1.76794060	2.58934336	0.56545866
H	-0.73966639	1.94256479	1.84507135
H	1.68649147	0.02968912	-2.08269713
C	4.16433797	0.53356803	-0.48608875
H	4.71683987	0.60120927	0.43981532
H	4.71736594	0.02422373	-1.27212612
H	4.09813334	1.56621773	-0.84941728
H	-1.57011888	-2.18655097	-0.94046047

Time = 196.00 fs
E(CASSCF/6-31G(d)) = -479.5389583

C	-0.92114122	-0.14996319	-0.63185578
C	0.47951308	-0.47828216	-0.30609975
C	0.92581166	-0.49747802	1.11599251
C	1.61824711	-0.20143197	-1.04712078
C	2.75754148	-0.03442981	-0.12666044
C	2.31857966	-0.23931805	1.15901139
N	-1.55313552	0.50739507	0.43102135
C	-2.94842213	0.09980656	0.62926867
H	2.91653331	-0.19162894	2.02847464
C	-1.90903508	-1.18493546	-1.21793492
C	-3.27009441	-0.74675496	-0.61739759
H	-3.78378184	-0.13563420	-1.35401961
H	-3.88631005	-1.62144018	-0.43606750

H	-3.05151594	-0.49412504	1.55258751
H	-3.60680878	0.96396543	0.68757912
H	-2.04032069	-1.17830798	-2.29535787
H	0.25895179	-0.71037105	1.90808960
C	-1.02291788	1.82720083	0.80941158
H	-0.16165712	2.08196127	0.18457319
H	-1.77672916	2.57971577	0.57855501
H	-0.73537165	1.92072899	1.85621116
H	1.69505487	0.01750145	-2.08516912
C	4.16326325	0.53010553	-0.49274731
H	4.71412444	0.59482054	0.43149569
H	4.71354364	0.01359882	-1.27457056
H	4.09711039	1.56440392	-0.85301411
H	-1.56714280	-2.17736892	-0.94289724

Time = 196.50 fs
E(CASSCF/6-31G(d)) = -479.5387287

C	-0.91764976	-0.14926067	-0.63443827
C	0.47346981	-0.47693299	-0.30598107
C	0.93531812	-0.48565927	1.12494879
C	1.62204297	-0.21054192	-1.04573399
C	2.75936430	-0.03403063	-0.13029849
C	2.30965926	-0.23269130	1.16581351
N	-1.55411919	0.50337478	0.43301925
C	-2.94606036	0.09679215	0.63018526
H	2.92295932	-0.17365100	2.02695754
C	-1.91262589	-1.17815976	-1.22351859
C	-3.27140620	-0.74673617	-0.62098944
H	-3.78303444	-0.12922540	-1.35458222
H	-3.89248203	-1.61993757	-0.44723400
H	-3.05169182	-0.50587696	1.54234636
H	-3.60650920	0.95447118	0.68434481
H	-2.03739318	-1.17205765	-2.30065183
H	0.25315829	-0.70080842	1.90796319
C	-1.02056728	1.82259404	0.81333374
H	-0.16932425	2.08321144	0.18244247
H	-1.78561421	2.57023814	0.59227977
H	-0.73142207	1.89835706	1.86688905
H	1.70319960	0.00526504	-2.08860603
C	4.16191713	0.52671358	-0.49936929
H	4.71272076	0.58782539	0.42309658
H	4.70939340	0.00197485	-1.27618001
H	4.09538421	1.56195492	-0.85631864
H	-1.56494842	-2.16799555	-0.94499529

Time = 197.00 fs

E(CASSCF/6-31G(d)) = -479.5376136

C	-0.91405670	-0.14895974	-0.63666481
C	0.46722058	-0.47531890	-0.30572042
C	0.94500604	-0.47372046	1.13382208
C	1.62652790	-0.21961847	-1.04425370
C	2.76113959	-0.03351583	-0.13490142
C	2.30019729	-0.22621223	1.17295900
N	-1.55501818	0.49939612	0.43489666
C	-2.94363942	0.09371538	0.63107346
H	2.92994713	-0.15597203	2.02676412
C	-1.91619795	-1.17118888	-1.22918037
C	-3.27264382	-0.74676136	-0.62461809
H	-3.78225241	-0.12284347	-1.35486403
H	-3.89885653	-1.61794109	-0.45770942
H	-3.05180784	-0.51672486	1.53155678
H	-3.60612615	0.94508152	0.68168497
H	-2.03327892	-1.16566891	-2.30612463
H	0.24737183	-0.69157340	1.90911943
C	-1.01838872	1.81798731	0.81721452
H	-0.17745456	2.08354874	0.18116689
H	-1.79436864	2.56088173	0.60674462
H	-0.72782504	1.87565945	1.87661038
H	1.71086844	-0.00713086	-2.09265734
C	4.16029893	0.52339011	-0.50594724
H	4.71272458	0.58027232	0.41475369
H	4.70494458	-0.01056290	-1.27707377
H	4.09303424	1.55883625	-0.85940664
H	-1.56361799	-2.15846214	-0.94683628

Time = 197.50 fs
E(CASSCF/6-31G(d)) = -479.5352824

C	-0.91041398	-0.14904325	-0.63851188
C	0.46086421	-0.47345181	-0.30526967
C	0.95466407	-0.46169269	1.14257822
C	1.63169673	-0.22865340	-1.04271547
C	2.76279675	-0.03291865	-0.14039242
C	2.29039592	-0.21982326	1.18038805
N	-1.55581056	0.49546162	0.43663975
C	-2.94116517	0.09054895	0.63188932
H	2.93712545	-0.13867055	2.02770448
C	-1.91971827	-1.16404144	-1.23492268
C	-3.27380739	-0.74680294	-0.62829036
H	-3.78144784	-0.11650149	-1.35485060
H	-3.90534934	-1.61547000	-0.46746930
H	-3.05188524	-0.52678291	1.52062415

H	-3.60581616	0.93607507	0.67972866
H	-2.02807394	-1.15914792	-2.31183926
H	0.24201158	-0.68251852	1.91139303
C	-1.01643161	1.81336007	0.82110494
H	-0.18579246	2.08305939	0.18065885
H	-1.80277350	2.55161536	0.62204747
H	-0.72456956	1.85284673	1.88495631
H	1.71801434	-0.01980807	-2.09693416
C	4.15841318	0.52012611	-0.51247361
H	4.71417031	0.57220950	0.40654864
H	4.70022086	-0.02391957	-1.27738457
H	4.09014974	1.55505955	-0.86237464
H	-1.56322067	-2.14880604	-0.94850509

Time = 198.00 fs
E(CASSCF/6-31G(d)) = -479.5315033

C	-0.90678095	-0.14949235	-0.63995603
C	0.45451407	-0.47134565	-0.30459050
C	0.96404728	-0.44962638	1.15118348
C	1.63752626	-0.23763004	-1.04115706
C	2.76426555	-0.03227228	-0.14667519
C	2.28049884	-0.21346962	1.18803429
N	-1.55647576	0.49157811	0.43823381
C	-2.93864682	0.08727516	0.63258689
H	2.94404698	-0.12185649	2.02950279
C	-1.92315029	-1.15674458	-1.24074116
C	-3.27489649	-0.74683208	-0.63200838
H	-3.78064276	-0.11021357	-1.35453704
H	-3.91186838	-1.61255829	-0.47648702
H	-3.05194606	-0.53619587	1.50999380
H	-3.60571758	0.92771174	0.67859084
H	-2.02188798	-1.15250493	-2.31784270
H	0.23753445	-0.67349008	1.91456459
C	-1.01474872	1.80869369	0.82504880
H	-0.19404359	2.08185581	0.18079293
H	-1.81061636	2.54242807	0.63824336
H	-0.72162646	1.83016114	1.89159457
H	1.72459245	-0.03286983	-2.10104250
C	4.15627313	0.51691102	-0.51892990
H	4.71702570	0.56369752	0.39851495
H	4.69524503	-0.03796829	-1.27725150
H	4.08683177	1.55068706	-0.86533220
H	-1.56381090	-2.13908852	-0.95008532

Time = 198.50 fs
E(CASSCF/6-31G(d)) = -479.5262078

C	-0.90321680	-0.15028691	-0.64097763
C	0.44828410	-0.46901381	-0.30365359
C	0.97290107	-0.43756919	1.15961577
C	1.64398402	-0.24653892	-1.03961331
C	2.76548162	-0.03160768	-0.15364628
C	2.27076713	-0.20709029	1.19583643
N	-1.55699669	0.48775016	0.43966659
C	-2.93609418	0.08388133	0.63312243
H	2.95024278	-0.10563763	2.03184768
C	-1.92646038	-1.14932174	-1.24663484
C	-3.27591204	-0.74681918	-0.63577556
H	-3.77986754	-0.10398402	-1.35392837
H	-3.91832571	-1.60925027	-0.48475085
H	-3.05201115	-0.54513937	1.50010294
H	-3.60593252	0.92019489	0.67836640
H	-2.01483496	-1.14574333	-2.32416871
H	0.23437819	-0.66432792	1.91840659
C	-1.01338999	1.80396455	0.82908654
H	-0.20191154	2.08006884	0.18141904
H	-1.81771678	2.53331916	0.65536608
H	-0.71893987	1.80783111	1.89631283
H	1.73057565	-0.04641340	-2.10462545
C	4.15389796	0.51372739	-0.52529809
H	4.72119331	0.55479680	0.39062282
H	4.69002941	-0.05257128	-1.27681485
H	4.08318668	1.54582069	-0.86840290
H	-1.56542502	-2.12937981	-0.95166066

Time = 199.00 fs
E(CASSCF/6-31G(d)) = -479.5195393

C	-0.89978100	-0.15140594	-0.64156007
C	0.44228954	-0.46647029	-0.30243741
C	0.98096389	-0.42557060	1.16785593
C	1.65102565	-0.25537335	-1.03811582
C	2.76638810	-0.03095282	-0.16119449
C	2.26147551	-0.20062335	1.20373289
N	-1.55735966	0.48398224	0.44092767
C	-2.93352022	0.08036145	0.63345678
H	2.95526176	-0.09012219	2.03443811
C	-1.92961631	-1.14179630	-1.25260356
C	-3.27685557	-0.74673424	-0.63959442
H	-3.77916123	-0.09780990	-1.35303936
H	-3.92463529	-1.60560070	-0.49225861
H	-3.05209680	-0.55379266	1.49135360
H	-3.60651279	0.91365804	0.67912990

H	-2.00703377	-1.13886344	-2.33082931
H	0.23291990	-0.65488263	1.92272063
C	-1.01240076	1.79914771	0.83325046
H	-0.20911982	2.07783933	0.18238100
H	-1.82393127	2.52430760	0.67342033
H	-0.71642658	1.78607529	1.89902483
H	1.73595432	-0.06051968	-2.10739015
C	4.15131332	0.51055328	-0.53155821
H	4.72651509	0.54557214	0.38278979
H	4.68457644	-0.06757332	-1.27621101
H	4.07932521	1.54059553	-0.87171758
H	-1.56808063	-2.11976372	-0.95331256

Time = 199.50 fs
E(CASSCF/6-31G(d)) = -479.5118213

C	-0.89653151	-0.15282808	-0.64169046
C	0.43664360	-0.46372980	-0.30092731
C	0.98797852	-0.41367938	1.17588498
C	1.65859507	-0.26412994	-1.03669202
C	2.76693865	-0.03033177	-0.16920347
C	2.25289994	-0.19400854	1.21166115
N	-1.55755505	0.48027868	0.44200891
C	-2.93094172	0.07671515	0.63355835
H	2.95871140	-0.07541153	2.03702094
C	-1.93258753	-1.13419145	-1.25864825
C	-3.27772920	-0.74654724	-0.64346652
H	-3.77857026	-0.09168154	-1.35189376
H	-3.93071398	-1.60167322	-0.49901783
H	-3.05221171	-0.56231509	1.48407945
H	-3.60745101	0.90815420	0.68093438
H	-1.99860681	-1.13186208	-2.33781159
H	0.23343981	-0.64502663	1.92736485
C	-1.01181898	1.79421822	0.83756239
H	-0.21543820	2.07530778	0.18353675
H	-1.82916025	2.51543460	0.69238211
H	-0.71397604	1.76509497	1.89977514
H	1.74073730	-0.07524918	-2.10912719
C	4.14855042	0.50736312	-0.53768979
H	4.73278215	0.53609346	0.37489351
H	4.67887848	-0.08280367	-1.27556917
H	4.07536022	1.53516845	-0.87540867
H	-1.57177670	-2.11033638	-0.95511998

Time = 200.00 fs
E(CASSCF/6-31G(d)) = -479.5035245

C	-0.89352307	-0.15453206	-0.64136011
C	0.43145492	-0.46080787	-0.29911442
C	0.99370457	-0.40194093	1.18368189
C	1.66662431	-0.27280875	-1.03536472
C	2.76710015	-0.02976391	-0.17755355
C	2.24530366	-0.18718994	1.21955724
N	-1.55757767	0.47664378	0.44290489
C	-2.92837955	0.07294624	0.63340554
H	2.96029146	-0.06159174	2.03941826
C	-1.93534550	-1.12653007	-1.26477096
C	-3.27853562	-0.74622845	-0.64739241
H	-3.77814699	-0.08558354	-1.35052288
H	-3.93648234	-1.59753852	-0.50504569
H	-3.05235378	-0.57082339	1.47851664
H	-3.60867830	0.90365358	0.68381056
H	-1.98967897	-1.12473264	-2.34507710
H	0.23609378	-0.63466265	1.93227294
C	-1.01167287	1.78915247	0.84203185
H	-0.22070644	2.07260416	0.18477820
H	-1.83335233	2.50676408	0.71220061
H	-0.71145160	1.74506695	1.89873761
H	1.74495175	-0.09064049	-2.10972281
C	4.14564460	0.50412904	-0.54367362
H	4.73974767	0.52643603	0.36678851
H	4.67291881	-0.09807930	-1.27500962
H	4.07140470	1.52970397	-0.87960384
H	-1.57649420	-2.10120346	-0.95715909

Time = 200.50 fs
E(CASSCF/6-31G(d)) = -479.4951872

C	-0.89080583	-0.15649728	-0.64056468
C	0.42682561	-0.45772059	-0.29699423
C	0.99793178	-0.39039456	1.19122156
C	1.67503353	-0.28141357	-1.03415215
C	2.76685474	-0.02926346	-0.18612272
C	2.23892249	-0.18011882	1.22735476
N	-1.55742693	0.47308174	0.44361287
C	-2.92585827	0.06906071	0.63298855
H	2.95981948	-0.04872617	2.04154558
C	-1.93786388	-1.11883478	-1.27097462
C	-3.27927809	-0.74574883	-0.65137161
H	-3.77794782	-0.07949615	-1.34896360
H	-3.94186510	-1.59327192	-0.51036896
H	-3.05250718	-0.57937406	1.47478287
H	-3.61006911	0.90005002	0.68776643
H	-1.98037636	-1.11746485	-2.35256376

H	0.24089579	-0.62373046	1.93746620
C	-1.01197907	1.78392960	0.84665504
H	-0.22485212	2.06983921	0.18604724
H	-1.83650607	2.49838086	0.73280141
H	-0.70869315	1.72613736	1.89620708
H	1.74864196	-0.10671032	-2.10916451
C	4.14263368	0.50082254	-0.54949353
H	4.74714188	0.51668024	0.35832473
H	4.66667381	-0.11320971	-1.27464368
H	4.06757008	1.52435824	-0.88441938
H	-1.58219714	-2.09247605	-0.95950357

Time = 201.00 fs
E(CASSCF/6-31G(d)) = -479.4873310

C	-0.88842426	-0.15870422	-0.63930396
C	0.42284963	-0.45448448	-0.29456495
C	1.00049384	-0.37907096	1.19847324
C	1.68373143	-0.28995166	-1.03306814
C	2.76620097	-0.02883924	-0.19478724
C	2.23394957	-0.17275633	1.23498383
N	-1.55710676	0.46959662	0.44413252
C	-2.92340542	0.06506499	0.63231008
H	2.95724865	-0.03684887	2.04342279
C	-1.94011876	-1.11112800	-1.27726247
C	-3.27996046	-0.74508037	-0.65540271
H	-3.77803105	-0.07339724	-1.34725613
H	-3.94679149	-1.58895082	-0.51502364
H	-3.05264039	-0.58795413	1.47286863
H	-3.61145205	0.89717475	0.69278708
H	-1.97082511	-1.11004482	-2.36019017
H	0.24770961	-0.61221132	1.94306015
C	-1.01274165	1.77853277	0.85141504
H	-0.22789905	2.06709811	0.18734507
H	-1.83866885	2.49038646	0.75409010
H	-0.70552184	1.70841699	1.89258324
H	1.75186737	-0.12345508	-2.10754076
C	4.13955619	0.49741623	-0.55513792
H	4.75468776	0.50691060	0.34936555
H	4.66011629	-0.12800285	-1.27457476
H	4.06396467	1.51926287	-0.88995372
H	-1.58883386	-2.08426505	-0.96222505

Time = 201.50 fs
E(CASSCF/6-31G(d)) = -479.4803799

C	-0.88641567	-0.16113493	-0.63758146

C	0.41961048	-0.45111629	-0.29182675
C	1.00128204	-0.36798976	1.20540001
C	1.69261679	-0.29843380	-1.03212230
C	2.76515417	-0.02849444	-0.20342253
C	2.23052064	-0.16507594	1.24237021
N	-1.55662568	0.46619235	0.44446563
C	-2.92105001	0.06096457	0.63138484
H	2.95267738	-0.02596031	2.04517931
C	-1.94208893	-1.10343203	-1.28363732
C	-3.28058723	-0.74419644	-0.65948354
H	-3.77845454	-0.06726418	-1.34544126
H	-3.95119538	-1.58465201	-0.51905496
H	-3.05270656	-0.59648292	1.47264158
H	-3.61262598	0.89481537	0.69883546
H	-1.96114895	-1.10245448	-2.36786225
H	0.25624873	-0.60013013	1.94926415
C	-1.01395223	1.77294993	0.85628358
H	-0.22996547	2.06443877	0.18873221
H	-1.83993251	2.48289314	0.77595741
H	-0.70174643	1.69197841	1.88834489
H	1.75469866	-0.14085262	-2.10503449
C	4.13644949	0.49388580	-0.56060113
H	4.76211684	0.49721413	0.33980358
H	4.65321928	-0.14227267	-1.27490048
H	4.06069258	1.51450970	-0.89628164
H	-1.59633873	-2.07667595	-0.96539393

Time = 202.00 fs
E(CASSCF/6-31G(d)) = -479.4746090

C	-0.88480867	-0.16377352	-0.63540381
C	0.41717877	-0.44763297	-0.28878092
C	1.00025543	-0.35715823	1.21195883
C	1.70158034	-0.30687329	-1.03132064
C	2.76374609	-0.02822676	-0.21190416
C	2.22870271	-0.15706532	1.24943485
N	-1.55599664	0.46287280	0.44461584
C	-2.91882108	0.05676331	0.63023808
H	2.94635188	-0.01602289	2.04705295
C	-1.94375609	-1.09576921	-1.29010072
C	-3.28116345	-0.74307216	-0.66361130
H	-3.77927314	-0.06107675	-1.34355792
H	-3.95501532	-1.58044903	-0.52251650
H	-3.05264526	-0.60482352	1.47386373
H	-3.61337776	0.89273680	0.70585321
H	-1.95146847	-1.09467202	-2.37548066
H	0.26608809	-0.58755400	1.95637472

C	-1.01559094	1.76717436	0.86122415
H	-0.23125079	2.06189273	0.19032044
H	-1.84042544	2.47601626	0.79828314
H	-0.69717358	1.67685646	1.88401553
H	1.75721391	-0.15886723	-2.10191102
C	4.13334843	0.49021161	-0.56588417
H	4.76918238	0.48767826	0.32957314
H	4.64595989	-0.15584592	-1.27571431
H	4.05785162	1.51013958	-0.90344927
H	-1.60463444	-2.06980157	-0.96908021

Time = 202.50 fs
E(CASSCF/6-31G(d)) = -479.4701878

C	-0.88362334	-0.16660578	-0.63277987
C	0.41561244	-0.44405095	-0.28542786
C	0.99744340	-0.34657103	1.21810028
C	1.71050531	-0.31528602	-1.03066733
C	2.76202416	-0.02802846	-0.22010709
C	2.22848939	-0.14872652	1.25609307
N	-1.55523615	0.45964117	0.44458762
C	-2.91674622	0.05246347	0.62890319
H	2.93865677	-0.00696358	2.04938537
C	-1.94510500	-1.08816223	-1.29665222
C	-3.28169476	-0.74168460	-0.66778279
H	-3.78053650	-0.05481905	-1.34164042
H	-3.95819459	-1.57640970	-0.52546916
H	-3.05238407	-0.61280057	1.47621658
H	-3.61349809	0.89070181	0.71376274
H	-1.94189937	-1.08667145	-2.38294923
H	0.27669307	-0.57458616	1.96476335
C	-1.01762776	1.76120479	0.86619691
H	-0.23201686	2.05946807	0.19225761
H	-1.84030289	2.46986149	0.82094033
H	-0.69162234	1.66305046	1.88011741
H	1.75949727	-0.17745366	-2.09849927
C	4.13028371	0.48637993	-0.57099532
H	4.77567232	0.47838872	0.31865850
H	4.63832301	-0.16856941	-1.27710870
H	4.05553193	1.50613703	-0.91147256
H	-1.61363426	-2.06371644	-0.97335455

Time = 203.00 fs
E(CASSCF/6-31G(d)) = -479.4670441

C	-0.88286809	-0.16962014	-0.62972068
C	0.41495238	-0.44038623	-0.28176919

C	0.99294535	-0.33621145	1.22377282
C	1.71927089	-0.32368962	-1.03016535
C	2.76005006	-0.02788668	-0.22790791
C	2.22980077	-0.14007548	1.26225627
N	-1.55436477	0.45650061	0.44438716
C	-2.91484957	0.04806617	0.62741902
H	2.93009478	0.00132398	2.05260143
C	-1.94612360	-1.08063412	-1.30328845
C	-3.28218732	-0.74001315	-0.67199455
H	-3.78228696	-0.04848163	-1.33971740
H	-3.96068185	-1.57259477	-0.52798111
H	-3.05184634	-0.62022532	1.47933306
H	-3.61280033	0.88849047	0.72246923
H	-1.93255036	-1.07842302	-2.39018370
H	0.28745742	-0.56135475	1.97483820
C	-1.02002501	1.75504502	0.87116388
H	-0.23256099	2.05715803	0.19470657
H	-1.83973253	2.46451713	0.84380002
H	-0.68493864	1.65053130	1.87712267
H	1.76163144	-0.19656235	-2.09516534
C	4.12728120	0.48238360	-0.57594992
H	4.78141791	0.46942677	0.30709824
H	4.63030384	-0.18031608	-1.27917510
H	4.05381357	1.50242935	-0.92033526
H	-1.62324462	-2.05847195	-0.97828872

Time = 203.50 fs
E(CASSCF/6-31G(d)) = -479.4650839

C	-0.88253889	-0.17280751	-0.62623918
C	0.41522136	-0.43665446	-0.27780757
C	0.98692041	-0.32605396	1.22892662
C	1.72775486	-0.33210268	-1.02981789
C	2.75789722	-0.02778401	-0.23518630
C	2.23249236	-0.13114006	1.26783467
N	-1.55340665	0.45345430	0.44402233
C	-2.91315048	0.04357207	0.62582710
H	2.92124737	0.00897143	2.05717611
C	-1.94680325	-1.07320830	-1.31000261
C	-3.28264771	-0.73803974	-0.67624301
H	-3.78455749	-0.04206334	-1.33781022
H	-3.96243129	-1.56905605	-0.53012683
H	-3.05095551	-0.62691865	1.48282820
H	-3.61113358	0.88591535	0.73186364
H	-1.92352109	-1.06989315	-2.39711804
H	0.29776352	-0.54799616	1.98699558
C	-1.02273960	1.74870349	0.87609460

H	-0.23319013	2.05494814	0.19782425
H	-1.83888014	2.46004205	0.86673439
H	-0.67701387	1.63924943	1.87540247
H	1.76369441	-0.21614421	-2.09228106
C	4.12436137	0.47822219	-0.58077006
H	4.78630063	0.46086662	0.29498605
H	4.62190953	-0.19099005	-1.28200420
H	4.05276380	1.49889360	-0.92999053
H	-1.63336789	-2.05409252	-0.98395627

Time = 204.00 fs
E(CASSCF/6-31G(d)) = -479.4641726

C	-0.88261828	-0.17616151	-0.62235000
C	0.41642291	-0.43287087	-0.27354752
C	0.97957175	-0.31606792	1.23351925
C	1.73583654	-0.34054396	-1.02962957
C	2.75564794	-0.02769914	-0.24182688
C	2.23637013	-0.12195692	1.27274184
N	-1.55238930	0.45050547	0.44350288
C	-2.91166242	0.03898218	0.62416896
H	2.91271743	0.01612335	2.06358175
C	-1.94713888	-1.06590857	-1.31678400
C	-3.28308283	-0.73574906	-0.68052460
H	-3.78737038	-0.03557238	-1.33593282
H	-3.96340345	-1.56583567	-0.53198635
H	-3.04964075	-0.63273219	1.48632757
H	-3.60839354	0.88283369	0.74182609
H	-1.91490042	-1.06104464	-2.40371040
H	0.30705404	-0.53463393	2.00155225
C	-1.02572503	1.74219286	0.88097106
H	-0.23419612	2.05282364	0.20174202
H	-1.83789546	2.45645572	0.88961878
H	-0.66780216	1.62914413	1.87518502
H	1.76575626	-0.23615448	-2.09018922
C	4.12153918	0.47390142	-0.58548375
H	4.79025647	0.45277321	0.28246762
H	4.61315972	-0.20052962	-1.28568462
H	4.05243450	1.49536815	-0.94036413
H	-1.64390384	-2.05057363	-0.99043216

Time = 204.50 fs
E(CASSCF/6-31G(d)) = -479.4642105

C	-0.88307510	-0.17967837	-0.61806953
C	0.41854106	-0.42905012	-0.26899584
C	0.97112821	-0.30622163	1.23752194

C	1.74339999	-0.34903169	-1.02960747
C	2.75338972	-0.02760788	-0.24772163
C	2.24120929	-0.11256779	1.27690132
N	-1.55134314	0.44765750	0.44284062
C	-2.91039227	0.03429851	0.62248402
H	2.90505772	0.02292092	2.07221710
C	-1.94712915	-1.05875885	-1.32361795
C	-3.28349969	-0.73312881	-0.68483586
H	-3.79073632	-0.02902653	-1.33409165
H	-3.96356577	-1.56296534	-0.53364404
H	-3.04784066	-0.63756467	1.48949064
H	-3.60452917	0.87915550	0.75222991
H	-1.90676500	-1.05183696	-2.40994631
H	0.31490339	-0.52135658	2.01867062
C	-1.02893329	1.73552963	0.88579106
H	-0.23583544	2.05077523	0.20655047
H	-1.83690032	2.45372913	0.91233265
H	-0.65733461	1.62015079	1.87652976
H	1.76787655	-0.25655575	-2.08916913
C	4.11882430	0.46943223	-0.59012386
H	4.79327730	0.44520028	0.26973420
H	4.60408664	-0.20890864	-1.29030079
H	4.05286026	1.49166908	-0.95135939
H	-1.65475147	-2.04788186	-0.99779181

Time = 205.00 fs
E(CASSCF/6-31G(d)) = -479.4650985

C	-0.88386479	-0.18335682	-0.61341586
C	0.42154061	-0.42520652	-0.26416241
C	0.96182678	-0.29648590	1.24092553
C	1.75033742	-0.35758263	-1.02976143
C	2.75121116	-0.02748435	-0.25277267
C	2.24677410	-0.10301575	1.28025408
N	-1.55030107	0.44491388	0.44204936
C	-2.90934003	0.02952455	0.62080776
H	2.89869612	0.02948463	2.08332682
C	-1.94677662	-1.05178282	-1.33048591
C	-3.28390517	-0.73016997	-0.68917348
H	-3.79465387	-0.02245250	-1.33228676
H	-3.96289373	-1.56046615	-0.53518751
H	-3.04550661	-0.64137328	1.49202880
H	-3.59954635	0.87484835	0.76294554
H	-1.89917754	-1.04222609	-2.41583952
H	0.32107168	-0.50820048	2.03829142
C	-1.03231653	1.72873393	0.89056934
H	-0.23831343	2.04880388	0.21228833

H	-1.83598073	2.45178003	0.93475936
H	-0.64572618	1.61220601	1.87932452
H	1.77010262	-0.27731841	-2.08940566
C	4.11622185	0.46482946	-0.59472676
H	4.79540894	0.43818887	0.25701250
H	4.59473379	-0.21613588	-1.29592925
H	4.05405664	1.48760802	-0.96286315
H	-1.66580967	-2.04595661	-1.00610977

Time = 205.50 fs
E(CASSCF/6-31G(d)) = -479.4666770

C	-0.88493031	-0.18719778	-0.60840819
C	0.42536780	-0.42135441	-0.25906127
C	0.95189742	-0.28683691	1.24374391
C	1.75655391	-0.36621072	-1.03010317
C	2.74919685	-0.02730275	-0.25689568
C	2.25283677	-0.09334146	1.28276505
N	-1.54929800	0.44227804	0.44114478
C	-2.90849885	0.02466569	0.61917026
H	2.89387579	0.03590081	2.09693233
C	-1.94608775	-1.04500340	-1.33736577
C	-3.28430582	-0.72686685	-0.69353424
H	-3.79910974	-0.01588502	-1.33051390
H	-3.96137127	-1.55834906	-0.53670696
H	-3.04260653	-0.64418216	1.49371881
H	-3.59351017	0.86993884	0.77384430
H	-1.89218492	-1.03216460	-2.42143077
H	0.32552812	-0.49514353	2.06009503
C	-1.03582860	1.72182915	0.89533648
H	-0.24177296	2.04692619	0.21893480
H	-1.83518430	2.45047369	0.95678578
H	-0.63317192	1.60525069	1.88330784
H	1.77246841	-0.29842004	-2.09096819
C	4.11373306	0.46011021	-0.59933067
H	4.79674568	0.43176612	0.24455073
H	4.58515268	-0.22225210	-1.30263365
H	4.05601806	1.48300822	-0.97475116
H	-1.67697641	-2.04471321	-1.01545712

Time = 206.00 fs
E(CASSCF/6-31G(d)) = -479.4687623

C	-0.88620391	-0.19120455	-0.60306872
C	0.42995346	-0.41750724	-0.25371123
C	0.94155899	-0.27725636	1.24601532
C	1.76196717	-0.37492694	-1.03064492

C	2.74742495	-0.02703862	-0.26002161
C	2.25918555	-0.08358232	1.28442832
N	-1.54836979	0.43975370	0.44014490
C	-2.90785566	0.01972952	0.61759543
H	2.89063058	0.04221468	2.11277817
C	-1.94507304	-1.03844231	-1.34423278
C	-3.28470760	-0.72321741	-0.69791505
H	-3.80407989	-0.00936476	-1.32876438
H	-3.95899258	-1.55661455	-0.53829188
H	-3.03912275	-0.64608165	1.49440958
H	-3.58653898	0.86450946	0.78480359
H	-1.88582033	-1.02160332	-2.42678476
H	0.32842502	-0.48210969	2.08349782
C	-1.03942642	1.71484235	0.90013622
H	-0.24629179	2.04517103	0.22641228
H	-1.83452052	2.44962347	0.97830104
H	-0.61994100	1.59922457	1.88810161
H	1.77500099	-0.31984080	-2.09380145
C	4.11135686	0.45529264	-0.60397416
H	4.79742073	0.42594491	0.23260414
H	4.57540204	-0.22732776	-1.31046094
H	4.05871935	1.47772464	-0.98689688
H	-1.68815181	-2.04405189	-1.02589908

Time = 206.50 fs
E(CASSCF/6-31G(d)) = -479.4710090

C	-0.88760922	-0.19538186	-0.59742074
C	0.43521419	-0.41367907	-0.24813573
C	0.93101182	-0.26773172	1.24779732
C	1.76651343	-0.38373754	-1.03139657
C	2.74596132	-0.02667095	-0.26209986
C	2.26563567	-0.07377022	1.28526674
N	-1.54755296	0.43734421	0.43906907
C	-2.90739162	0.01472604	0.61610010
H	2.88879077	0.04843188	2.13033965
C	-1.94374670	-1.03211933	-1.35106029
C	-3.28511552	-0.71922339	-0.70231275
H	-3.80953006	-0.00293559	-1.32702947
H	-3.95576219	-1.55525367	-0.54003171
H	-3.03505604	-0.64723063	1.49402811
H	-3.57880192	0.85869391	0.79570895
H	-1.88010010	-1.01048965	-2.43198298
H	0.33005742	-0.46898576	2.10770365
C	-1.04307110	1.70780332	0.90502098
H	-0.25187870	2.04358510	0.23458480
H	-1.83397085	2.44900463	0.99919684

H	-0.60635404	1.59406635	1.89326608
H	1.77771338	-0.34156079	-2.09773328
C	4.10909121	0.45039431	-0.60869411
H	4.79759469	0.42072383	0.22141404
H	4.56554297	-0.23145604	-1.31943453
H	4.06211460	1.47165336	-0.99917513
H	-1.69923544	-2.04386072	-1.03749266

Time = 207.00 fs
E(CASSCF/6-31G(d)) = -479.4731070

C	-0.88906339	-0.19973595	-0.59148956
C	0.44105530	-0.40988402	-0.24236317
C	0.92043992	-0.25825474	1.24916104
C	1.77014838	-0.39264362	-1.03236237
C	2.74485682	-0.02618368	-0.26309966
C	2.27202879	-0.06393204	1.28532855
N	-1.54688384	0.43505270	0.43793810
C	-2.90708336	0.00966803	0.61469388
H	2.88803269	0.05452863	2.14886420
C	-1.94212644	-1.02605160	-1.35782094
C	-3.28553342	-0.71489045	-0.70672401
H	-3.81541755	0.00335805	-1.32530171
H	-3.95169595	-1.55424886	-0.54201335
H	-3.03042405	-0.64785123	1.49257944
H	-3.57050893	0.85266708	0.80645773
H	-1.87502594	-0.99876947	-2.43711603
H	0.33079286	-0.45564329	2.13177964
C	-1.04672881	1.70074429	0.91004731
H	-0.25847863	2.04223018	0.24326603
H	-1.83349868	2.44836554	1.01936808
H	-0.59276248	1.58970771	1.89834811
H	1.78060790	-0.36355567	-2.10249217
C	4.10693480	0.44543108	-0.61352396
H	4.79744026	0.41608781	0.21118898
H	4.55563680	-0.23474802	-1.32954993
H	4.06613921	1.46474313	-1.01146806
H	-1.71012796	-2.04402386	-1.05028382

Time = 207.50 fs
E(CASSCF/6-31G(d)) = -479.4747977

C	-0.89048011	-0.20427398	-0.58530164
C	0.44737398	-0.40613687	-0.23642611
C	0.91001123	-0.24881994	1.25018292
C	1.77284974	-0.40164024	-1.03353755
C	2.74414410	-0.02556718	-0.26301127

C	2.27823231	-0.05408991	1.28468032
N	-1.54639786	0.43288186	0.43677349
C	-2.90690431	0.00457134	0.61337922
H	2.88794291	0.06046559	2.16745433
C	-1.94023306	-1.02025304	-1.36448787
C	-3.28596374	-0.71022820	-0.71114529
H	-3.82169285	0.00947364	-1.32357753
H	-3.94682084	-1.55357511	-0.54432026
H	-3.02526175	-0.64822225	1.49014311
H	-3.56189983	0.84663153	0.81696072
H	-1.87058431	-0.98638700	-2.44227313
H	0.33101073	-0.44196088	2.15474891
C	-1.05037102	1.69369897	0.91527135
H	-0.26598035	2.04118286	0.25222783
H	-1.83306322	2.44744476	1.03871515
H	-0.57952357	1.58607125	1.90292735
H	1.78367327	-0.38579326	-2.10773836
C	4.10488856	0.44041595	-0.61849186
H	4.79712694	0.41200915	0.20208463
H	4.54574159	-0.23732659	-1.34077073
H	4.07071136	1.45700061	-1.02366905
H	-1.72073110	-2.04442738	-1.06430570

Time = 208.00 fs
E(CASSCF/6-31G(d)) = -479.4759868

C	-0.89177169	-0.20900378	-0.57888509
C	0.45406124	-0.40245262	-0.23036117
C	0.89988156	-0.23942310	1.25093837
C	1.77461649	-0.41071623	-1.03490527
C	2.74383702	-0.02481915	-0.26184603
C	2.28413382	-0.04426281	1.28339942
N	-1.54612873	0.43083367	0.43559736
C	-2.90682618	-0.00054473	0.61215209
H	2.88808929	0.06620165	2.18515409
C	-1.93808984	-1.01473371	-1.37103617
C	-3.28640724	-0.70525032	-0.71557267
H	-3.82830116	0.01537406	-1.32185849
H	-3.94117602	-1.55319996	-0.54703051
H	-3.01961868	-0.64866698	1.48686525
H	-3.55322940	0.84080078	0.82714298
H	-1.86674983	-0.97328669	-2.44753294
H	0.33104835	-0.42783948	2.17567085
C	-1.05397428	1.68670201	0.92074475
H	-0.27422959	2.04052797	0.26121461
H	-1.83263202	2.44598620	1.05714616
H	-0.56698041	1.58306602	1.90664967

H	1.78688797	-0.40823085	-2.11309414
C	4.10295708	0.43535869	-0.62361935
H	4.79680490	0.40844889	0.19418833
H	4.53591040	-0.23932110	-1.35302529
H	4.07573527	1.44849288	-1.03568636
H	-1.73094886	-2.04496624	-1.07957610

Time = 208.50 fs
E(CASSCF/6-31G(d)) = -479.4767087

C	-0.89285195	-0.21393329	-0.57226867
C	0.46100508	-0.39884692	-0.22420785
C	0.89019448	-0.23006023	1.25149540
C	1.77546920	-0.41985393	-1.03643481
C	2.74393022	-0.02394523	-0.25963560
C	2.28963859	-0.03446692	1.28156614
N	-1.54610767	0.42890926	0.43443152
C	-2.90682083	-0.00565727	0.61100305
H	2.88807424	0.07170465	2.20103893
C	-1.93572196	-1.00949935	-1.37744399
C	-3.28686280	-0.69997449	-0.72000178
H	-3.83518434	0.02102939	-1.32015325
H	-3.93481215	-1.55308507	-0.55021589
H	-3.01355836	-0.64953916	1.48294515
H	-3.54475250	0.83537957	0.83694308
H	-1.86348549	-0.95941396	-2.45295295
H	0.33117275	-0.41321401	2.19371145
C	-1.05751963	1.67978792	0.92651127
H	-0.28304504	2.04035544	0.26995804
H	-1.83219349	2.44375611	1.07458100
H	-0.55544113	1.58058874	1.90925300
H	1.79021825	-0.43081465	-2.11818004
C	4.10114941	0.43026540	-0.62892023
H	4.79659337	0.40535822	0.18750696
H	4.52618858	-0.24086249	-1.36620614
H	4.08110360	1.43934448	-1.04744465
H	-1.74068855	-2.04554866	-1.09609716

Time = 209.00 fs
E(CASSCF/6-31G(d)) = -479.4771537

C	-0.89363814	-0.21907077	-0.56548158
C	0.46809245	-0.39533561	-0.21800802
C	0.88108340	-0.22072701	1.25191097
C	1.77544762	-0.42902967	-1.03808075
C	2.74440101	-0.02295867	-0.25643049
C	2.29466525	-0.02471662	1.27925784

N	-1.54636290	0.42710915	0.43329725
C	-2.90686181	-0.01073980	0.60991907
H	2.88757558	0.07695950	2.21428267
C	-1.93315584	-1.00455111	-1.38369360
C	-3.28732742	-0.69442220	-0.72442780
H	-3.84228228	0.02641907	-1.31847807
H	-3.92779093	-1.55318701	-0.55394060
H	-3.00715518	-0.65120387	1.47861591
H	-3.53670727	0.83054283	0.84631133
H	-1.86074409	-0.94471616	-2.45856073
H	0.33156115	-0.39805731	2.20818836
C	-1.06099156	1.67299038	0.93260376
H	-0.29223793	2.04075220	0.27819456
H	-1.83176580	2.44055677	1.09095543
H	-0.54516320	1.57852343	1.91058220
H	1.79361819	-0.45348069	-2.12264340
C	4.09947936	0.42513863	-0.63440003
H	4.79657319	0.40267980	0.18196291
H	4.51661265	-0.24208000	-1.38017127
H	4.08670115	1.42973116	-1.05888542
H	-1.74986261	-2.04610024	-1.11385502

Time = 209.50 fs
E(CASSCF/6-31G(d)) = -479.4775862

C	-0.89405346	-0.22442336	-0.55855257
C	0.47521163	-0.39193517	-0.21180471
C	0.87267075	-0.21141840	1.25222780
C	1.77460799	-0.43821435	-1.03978387
C	2.74521168	-0.02187972	-0.25229690
C	2.29914475	-0.01502462	1.27654435
N	-1.54691862	0.42543257	0.43221447
C	-2.90692595	-0.01576255	0.60888568
H	2.88636417	0.08196815	2.22420694
C	-1.93041835	-0.99988516	-1.38977217
C	-3.28779592	-0.68861871	-0.72884546
H	-3.84953363	0.03153343	-1.31685722
H	-3.92018491	-1.55345749	-0.55826003
H	-3.00049165	-0.65401415	1.47412033
H	-3.52930058	0.82641637	0.85520697
H	-1.85847213	-0.92914658	-2.46434790
H	0.33229898	-0.38237984	2.21860291
C	-1.06437709	1.66634138	0.93904172
H	-0.30163284	2.04179335	0.28568603
H	-1.83140365	2.43623704	1.10622372
H	-0.53634287	1.57674354	1.91059626
H	1.79703194	-0.47615758	-2.12618314

C	4.09796494	0.41997794	-0.64005640
H	4.79678527	0.40034943	0.17739794
H	4.50721084	-0.24309823	-1.39474753
H	4.09240825	1.41987016	-1.06996641
H	-1.75838910	-2.04656545	-1.13281882

Time = 210.00 fs
E(CASSCF/6-31G(d)) = -479.4782318

C	-0.89402812	-0.22999752	-0.55150969
C	0.48225351	-0.38866222	-0.20564176
C	0.86506940	-0.20212852	1.25247322
C	1.77301955	-0.44737458	-1.04147242
C	2.74631306	-0.02073441	-0.24731469
C	2.30301756	-0.00540244	1.27348561
N	-1.54779462	0.42387797	0.43120143
C	-2.90699481	-0.02069363	0.60788973
H	2.88431407	0.08675256	2.23030819
C	-1.92753613	-0.99549271	-1.39567208
C	-3.28826115	-0.68259271	-0.73324921
H	-3.85687737	0.03637403	-1.31532276
H	-3.91207641	-1.55384486	-0.56321990
H	-2.99365592	-0.65828716	1.46968286
H	-3.52269723	0.82306133	0.86359571
H	-1.85660955	-0.91266478	-2.47026681
H	0.33337995	-0.36622711	2.22465629
C	-1.06766465	1.65987106	0.94582948
H	-0.31108722	2.04353409	0.29223702
H	-1.83120029	2.43070094	1.12036309
H	-0.52910404	1.57511454	1.90937079
H	1.80039478	-0.49876930	-2.12856738
C	4.09662733	0.41478075	-0.64587971
H	4.79723496	0.39829693	0.17358461
H	4.49800240	-0.24403616	-1.40973618
H	4.09810395	1.41000587	-1.08065838
H	-1.76619464	-2.04690888	-1.15294304

Time = 210.50 fs
E(CASSCF/6-31G(d)) = -479.4792822

C	-0.89350094	-0.23579843	-0.54437967
C	0.48911298	-0.38553354	-0.19956282
C	0.85838303	-0.19285055	1.25265838
C	1.77076114	-0.45647397	-1.04306494
C	2.74764841	-0.01955320	-0.24157418
C	2.30623236	0.00413951	1.27013015
N	-1.54900591	0.42244298	0.43027457

C	-2.90705527	-0.02550048	0.60692189
H	2.88140227	0.09135290	2.23227424
C	-1.92453509	-0.99136017	-1.40139110
C	-3.28871396	-0.67637590	-0.73763324
H	-3.86425289	0.04095344	-1.31391381
H	-3.90355648	-1.55429484	-0.56885556
H	-2.98673930	-0.66427842	1.46548041
H	-3.51701228	0.82046427	0.87144692
H	-1.85509198	-0.89523937	-2.47623157
H	0.33471000	-0.34967655	2.22626056
C	-1.07084358	1.65360726	0.95295488
H	-0.32050700	2.04600168	0.29771028
H	-1.83128728	2.42391131	1.13337545
H	-0.52349257	1.57349730	1.90709541
H	1.80363542	-0.52123860	-2.12964526
C	4.09548930	0.40954355	-0.65185433
H	4.79790050	0.39644750	0.17024318
H	4.48899923	-0.24500655	-1.42492035
H	4.10366934	1.40039329	-1.09094146
H	-1.77321553	-2.04711341	-1.17416868

Time = 211.00 fs
E(CASSCF/6-31G(d)) = -479.4808391

C	-0.89242016	-0.24182963	-0.53718752
C	0.49568987	-0.38256579	-0.19361090
C	0.85270679	-0.18357675	1.25277756
C	1.76791817	-0.46547405	-1.04447220
C	2.74915603	-0.01836955	-0.23517423
C	2.30874468	0.01359171	1.26651427
N	-1.55056211	0.42112448	0.42944810
C	-2.90710000	-0.03015191	0.60597894
H	2.87770643	0.09582568	2.22999413
C	-1.92143962	-0.98746940	-1.40693188
C	-3.28914335	-0.67000263	-0.74199178
H	-3.87160012	0.04529462	-1.31267512
H	-3.89472367	-1.55475128	-0.57519124
H	-2.97983363	-0.67216012	1.46161716
H	-3.51230981	0.81853559	0.87873296
H	-1.85385139	-0.87684943	-2.48212121
H	0.33611055	-0.33283224	2.22354527
C	-1.07390396	1.64757535	0.96038867
H	-0.32985636	2.04918920	0.30203887
H	-1.83183034	2.41589065	1.14528920
H	-0.51947262	1.57175118	1.90406706
H	1.80667828	-0.54349205	-2.12935030
C	4.09457329	0.40426316	-0.65796029

H	4.79874602	0.39472286	0.16706352
H	4.48020709	-0.24611604	-1.44007341
H	4.10898989	1.39127970	-1.10079991
H	-1.77939950	-2.04717760	-1.19642521

Time = 211.50 fs
E(CASSCF/6-31G(d)) = -479.4828534

C	-0.89074494	-0.24809232	-0.52995604
C	0.50189023	-0.37977558	-0.18782772
C	0.84812908	-0.17429845	1.25280841
C	1.76458117	-0.47433499	-1.04559972
C	2.75077160	-0.01721894	-0.22822085
C	2.31051483	0.02294543	1.26266157
N	-1.55246716	0.41991859	0.42873363
C	-2.90712735	-0.03462025	0.60506517
H	2.87339986	0.10024194	2.22356182
C	-1.91827216	-0.98379832	-1.41230124
C	-3.28953678	-0.66350937	-0.74631935
H	-3.87885946	0.04942910	-1.31165560
H	-3.88568301	-1.55515757	-0.58223974
H	-2.97303027	-0.68200543	1.45810764
H	-3.50860713	0.81711615	0.88542941
H	-1.85281679	-0.85748586	-2.48778759
H	0.33732334	-0.31582064	2.21686016
C	-1.07683694	1.64179819	0.96808525
H	-0.33915845	2.05305389	0.30523063
H	-1.83302284	2.40671813	1.15615947
H	-0.51692656	1.56973839	1.90067845
H	1.80944665	-0.56546344	-2.12769735
C	4.09389930	0.39893803	-0.66417489
H	4.79973356	0.39304226	0.16372721
H	4.47162688	-0.24746559	-1.45496794
H	4.11395706	1.38288570	-1.11021632
H	-1.78470721	-2.04711196	-1.21963350

Time = 212.00 fs
E(CASSCF/6-31G(d)) = -479.4852278

C	-0.88844802	-0.25458437	-0.52270492
C	0.50762929	-0.37717910	-0.18225333
C	0.84473236	-0.16500635	1.25271270
C	1.76084267	-0.48301610	-1.04634999
C	2.75242982	-0.01613818	-0.22082515
C	2.31150610	0.03219260	1.25858264
N	-1.55471837	0.41882031	0.42813906
C	-2.90714095	-0.03888312	0.60419229

H	2.86874631	0.10468569	2.21327957
C	-1.91505281	-0.98032155	-1.41750875
C	-3.28988041	-0.65693405	-0.75061086
H	-3.88597052	0.05339495	-1.31090531
H	-3.87654406	-1.55545672	-0.59000185
H	-2.96641677	-0.69377911	1.45486975
H	-3.50588001	0.81598916	0.89151664
H	-1.85191409	-0.83715268	-2.49306415
H	0.33801639	-0.29878567	2.20677000
C	-1.07963563	1.63629589	0.97598443
H	-0.34849092	2.05751679	0.30736944
H	-1.83507513	2.39651862	1.16606591
H	-0.51566367	1.56732739	1.89739631
H	1.81186662	-0.58709681	-2.12477889
C	4.09348353	0.39356959	-0.67047450
H	4.80083718	0.39132419	0.15992907
H	4.46325751	-0.24915046	-1.46938549
H	4.11847092	1.37538862	-1.11916691
H	-1.78911265	-2.04693362	-1.24370759

Time = 212.50 fs
E(CASSCF/6-31G(d)) = -479.4877527

C	-0.88551775	-0.26129973	-0.51545015
C	0.51283414	-0.37479186	-0.17692534
C	0.84259403	-0.15569101	1.25243762
C	1.75679536	-0.49147615	-1.04662480
C	2.75406563	-0.01516486	-0.21310265
C	2.31168273	0.04132565	1.25427551
N	-1.55730570	0.41782306	0.42766788
C	-2.90714895	-0.04292499	0.60337835
H	2.86409458	0.10925301	2.19965410
C	-1.91179909	-0.97701141	-1.42256535
C	-3.29015952	-0.65031549	-0.75486190
H	-3.89287108	0.05723428	-1.31047336
H	-3.86741929	-1.55559252	-0.59846629
H	-2.96007563	-0.70734028	1.45173225
H	-3.50407350	0.81489885	0.89698245
H	-1.85106522	-0.81586692	-2.49777614
H	0.33779393	-0.28188199	2.19403576
C	-1.08229581	1.63108604	0.98401506
H	-0.35797204	2.06246722	0.30860743
H	-1.83820222	2.38545144	1.17510939
H	-0.51543427	1.56439866	1.89473294
H	1.81386899	-0.60834792	-2.12075160
C	4.09333677	0.38816305	-0.67683589
H	4.80205251	0.38948776	0.15539595

H	4.45509764	-0.25126036	-1.48312530
H	4.12244122	1.36890897	-1.12761765
H	-1.79260326	-2.04665999	-1.26855717

Time = 213.00 fs
E(CASSCF/6-31G(d)) = -479.4901799

C	-0.88195883	-0.26822857	-0.50820353
C	0.51744595	-0.37262811	-0.17187838
C	0.84178430	-0.14634358	1.25191894
C	1.75252845	-0.49967445	-1.04632899
C	2.75561701	-0.01433620	-0.20517094
C	2.31101024	0.05033750	1.24972681
N	-1.56021133	0.41691851	0.42731890
C	-2.90716296	-0.04673805	0.60264561
H	2.85986783	0.11405003	2.18338115
C	-1.90852573	-0.97383871	-1.42748226
C	-3.29035888	-0.64369266	-0.75906888
H	-3.89949594	0.06099082	-1.31040567
H	-3.85842274	-1.55551121	-0.60761026
H	-2.95408436	-0.72245527	1.44845384
H	-3.50311416	0.81357206	0.90182539
H	-1.85018846	-0.79365791	-2.50175238
H	0.33621835	-0.26526634	2.17957091
C	-1.08481702	1.62618373	0.99209988
H	-0.36774190	2.06777153	0.30915159
H	-1.84260829	2.37369625	1.18340772
H	-0.51595297	1.56085201	1.89320867
H	1.81539273	-0.62918371	-2.11581704
C	4.09346314	0.38272792	-0.68323714
H	4.80340362	0.38745425	0.14990150
H	4.44714611	-0.25387957	-1.49601205
H	4.12578817	1.36350154	-1.13552301
H	-1.79518006	-2.04630279	-1.29408978

Time = 213.50 fs
E(CASSCF/6-31G(d)) = -479.4923276

C	-0.87779173	-0.27535752	-0.50097298
C	0.52142034	-0.37070082	-0.16714365
C	0.84236200	-0.13695687	1.25108669
C	1.74812473	-0.50757244	-1.04537471
C	2.75702809	-0.01368778	-0.19714798
C	2.30945749	0.05922196	1.24491515
N	-1.56340969	0.41609679	0.42708685
C	-2.90719669	-0.05032257	0.60201790
H	2.85654728	0.11919012	2.16531408

C	-1.90524454	-0.97077372	-1.43227002
C	-3.29046308	-0.63710400	-0.76322919
H	-3.90577564	0.06470749	-1.31074279
H	-3.84966847	-1.55516273	-0.61739996
H	-2.94851666	-0.73881790	1.44474978
H	-3.50292173	0.81173970	0.90605799
H	-1.84919938	-0.77056655	-2.50483503
H	0.33284431	-0.24908702	2.16436726
C	-1.08720328	1.62160159	1.00016119
H	-0.37794258	2.07328331	0.30924702
H	-1.84846883	2.36143708	1.19109036
H	-0.51693020	1.55661437	1.89330670
H	1.81638539	-0.64958154	-2.11019987
C	4.09385928	0.37727815	-0.68965808
H	4.80494666	0.38514880	0.14327705
H	4.43940341	-0.25708649	-1.50790236
H	4.12844358	1.35915310	-1.14282705
H	-1.79685734	-2.04586105	-1.32021219

Time = 214.00 fs
E(CASSCF/6-31G(d)) = -479.4941056

C	-0.87305088	-0.28267043	-0.49376309
C	0.52472615	-0.36902157	-0.16274872
C	0.84436811	-0.12752641	1.24987442
C	1.74365867	-0.51513545	-1.04368594
C	2.75825184	-0.01325227	-0.18915040
C	2.30700062	0.06797433	1.23981617
N	-1.56686831	0.41534717	0.42696351
C	-2.90726452	-0.05368673	0.60151786
H	2.85464170	0.12478888	2.14640620
C	-1.90196431	-0.96778689	-1.43693796
C	-3.29045689	-0.63058680	-0.76734123
H	-3.91163535	0.06842438	-1.31151820
H	-3.84126909	-1.55450250	-0.62779167
H	-2.94344385	-0.75607501	1.44032224
H	-3.50342003	0.80915660	0.90970947
H	-1.84801263	-0.74664384	-2.50688854
H	0.32727263	-0.23347170	2.14938728
C	-1.08946351	1.61734956	1.00812679
H	-0.38869845	2.07885574	0.30915767
H	-1.85591203	2.34884674	1.19829315
H	-0.51810759	1.55164695	1.89542515
H	1.81680403	-0.66952677	-2.10412157
C	4.09451401	0.37183158	-0.69608050
H	4.80677028	0.38250202	0.13541760
H	4.43187154	-0.26095280	-1.51868929

H	4.13035142	1.35578556	-1.14946698
H	-1.79766247	-2.04531567	-1.34683162

Time = 214.50 fs
E(CASSCF/6-31G(d)) = -479.4955733

C	-0.86778329	-0.29014847	-0.48657611
C	0.52734459	-0.36760077	-0.15871755
C	0.84781923	-0.11805149	1.24823050
C	1.73919665	-0.52233349	-1.04120134
C	2.75925079	-0.01305906	-0.18129316
C	2.30362750	0.07659214	1.23440985
N	-1.57054851	0.41465851	0.42693896
C	-2.90738060	-0.05684601	0.60116429
H	2.85464150	0.13095761	2.12762587
C	-1.89869074	-0.96484952	-1.44149376
C	-3.29032562	-0.62417668	-0.77140447
H	-3.91699455	0.07217749	-1.31275710
H	-3.83333473	-1.55349237	-0.63873287
H	-2.93893485	-0.77385232	1.43489001
H	-3.50454548	0.80561871	0.91282768
H	-1.84654386	-0.72194959	-2.50780720
H	0.31921936	-0.21851591	2.13543611
C	-1.09161131	1.61343499	1.01593579
H	-0.40010118	2.08435215	0.30914915
H	-1.86500147	2.33607083	1.20515192
H	-0.51929389	1.54595038	1.89983007
H	1.81661499	-0.68900914	-2.09777577
C	4.09540844	0.36640919	-0.70248793
H	4.80899313	0.37945105	0.12628377
H	4.42455367	-0.26554247	-1.52830489
H	4.13146874	1.35326427	-1.15537841
H	-1.79763483	-2.04462700	-1.37385597

Time = 215.00 fs
E(CASSCF/6-31G(d)) = -479.4968922

C	-0.86204730	-0.29777052	-0.47941298
C	0.52926850	-0.36644782	-0.15507030
C	0.85269990	-0.10853605	1.24612888
C	1.73479741	-0.52914208	-1.03787668
C	2.75999722	-0.01313411	-0.17368921
C	2.29934395	0.08507587	1.22868947
N	-1.57440647	0.41401987	0.42700259
C	-2.90755808	-0.05982234	0.60096998
H	2.85695687	0.13779704	2.10984803
C	-1.89542657	-0.96193428	-1.44594314

C	-3.29005544	-0.61790715	-0.77541946
H	-3.92176697	0.07599772	-1.31447560
H	-3.82597150	-1.55210164	-0.65016350
H	-2.93505541	-0.79177892	1.42821341
H	-3.50625346	0.80097549	0.91548002
H	-1.84471114	-0.69655096	-2.50751943
H	0.30859048	-0.20427517	2.12304020
C	-1.09366464	1.60986265	1.02354327
H	-0.41219760	2.08965635	0.30947252
H	-1.87572272	2.32321627	1.21179711
H	-0.52039346	1.53956799	1.90662021
H	1.81579262	-0.70801865	-2.09130607
C	4.09651658	0.36103383	-0.70886524
H	4.81175909	0.37594081	0.11589995
H	4.41745401	-0.27091043	-1.53672125
H	4.13176652	1.35141083	-1.16050176
H	-1.79682472	-2.04373371	-1.40119376

Time = 215.50 fs
E(CASSCF/6-31G(d)) = -479.4982045

C	-0.85591283	-0.30551368	-0.47227382
C	0.53050326	-0.36557064	-0.15182261
C	0.85895673	-0.09898915	1.24357606
C	1.73051305	-0.53554248	-1.03368587
C	2.76047217	-0.01350022	-0.16644921
C	2.29418026	0.09342983	1.22267033
N	-1.57839407	0.41342065	0.42714336
C	-2.90780859	-0.06264299	0.60093994
H	2.86184641	0.14539188	2.09373949
C	-1.89217163	-0.95901582	-1.45028935
C	-3.28963359	-0.61180923	-0.77938786
H	-3.92586144	0.07991106	-1.31668074
H	-3.81927990	-1.55030826	-0.66201711
H	-2.93186518	-0.80950918	1.42011360
H	-3.50852201	0.79513797	0.91775346
H	-1.84243529	-0.67051825	-2.50599013
H	0.29554330	-0.19076150	2.11236754
C	-1.09564545	1.60663460	1.03092315
H	-0.42498241	2.09468079	0.31035042
H	-1.88797663	2.31034500	1.21834923
H	-0.52142142	1.53258552	1.91570937
H	1.81431859	-0.72654200	-2.08478844
C	4.09780621	0.35572878	-0.71519834
H	4.81522989	0.37192441	0.10434840
H	4.41057615	-0.27710026	-1.54394893
H	4.13123059	1.35001775	-1.16478859

H	-1.79529203	-2.04255376	-1.42875510

Time = 216.00 fs
E(CASSCF/6-31G(d)) = -479.4995034

C	-0.84945966	-0.31335340	-0.46515899
C	0.53106590	-0.36497589	-0.14898510
C	0.86649434	-0.08942463	1.24061181
C	1.72638970	-0.54152217	-1.02862167
C	2.76066513	-0.01417729	-0.15968078
C	2.28819801	0.10166281	1.21639669
N	-1.58246005	0.41285098	0.42735083
C	-2.90814201	-0.06533940	0.60107053
H	2.86935557	0.15380570	2.07966854
C	-1.88892320	-0.95607080	-1.45453326
C	-3.28904862	-0.60591125	-0.78331230
H	-3.92918324	0.08393875	-1.31937088
H	-3.81335329	-1.54809863	-0.67422252
H	-2.92941638	-0.82674018	1.41048524
H	-3.51135372	0.78808241	0.91975326
H	-1.83964130	-0.64392383	-2.50322004
H	0.28051248	-0.17795095	2.10321992
C	-1.09758001	1.60374996	1.03806889
H	-0.43839483	2.09937300	0.31196606
H	-1.90157917	2.29747378	1.22491532
H	-0.52250188	1.52512956	1.92683309
H	1.81218111	-0.74455812	-2.07822396
C	4.09924036	0.35051628	-0.72147335
H	4.81957527	0.36736396	0.09175990
H	4.40392188	-0.28414278	-1.55003460
H	4.12986197	1.34886494	-1.16820889
H	-1.79310448	-2.04098867	-1.45645120

Time = 216.50 fs
E(CASSCF/6-31G(d)) = -479.5005425

C	-0.84277731	-0.32126392	-0.45807002
C	0.53098588	-0.36466875	-0.14656245
C	0.87517535	-0.07986061	1.23730332
C	1.72246838	-0.54707467	-1.02269497
C	2.76057289	-0.01518258	-0.15348752
C	2.28149478	0.10978860	1.20994339
N	-1.58655105	0.41230201	0.42761570
C	-2.90856639	-0.06794592	0.60134909
H	2.87928146	0.16307858	2.06766723
C	-1.88567625	-0.95307791	-1.45867348
C	-3.28829054	-0.60023876	-0.78719645

H	-3.93163596	0.08809847	-1.32253631
H	-3.80827608	-1.54546717	-0.68670515
H	-2.92775147	-0.84322397	1.39930249
H	-3.51477451	0.77984927	0.92159988
H	-1.83625954	-0.61684135	-2.49924268
H	0.26418799	-0.16579429	2.09510137
C	-1.09949932	1.60120444	1.04499177
H	-0.45231951	2.10371920	0.31445533
H	-1.91627000	2.28458135	1.23158657
H	-0.52384990	1.51736186	1.93957750
H	1.80937398	-0.76203609	-2.07154045
C	4.10077876	0.34541609	-0.72767621
H	4.82496033	0.36223081	0.07830098
H	4.39749020	-0.29205317	-1.55505638
H	4.12767780	1.34773509	-1.17075565
H	-1.79033558	-2.03892832	-1.48419403

Time = 217.00 fs
E(CASSCF/6-31G(d)) = -479.5009007

C	-0.83596372	-0.32921886	-0.45101008
C	0.53030457	-0.36465257	-0.14455245
C	0.88482348	-0.07031866	1.23373456
C	1.71878520	-0.55219974	-1.01593362
C	2.76019930	-0.01653075	-0.14796717
C	2.27420608	0.11782635	1.20341194
N	-1.59061283	0.41176605	0.42793021
C	-2.90908793	-0.07049841	0.60175421
H	2.89117622	0.17322621	2.05745623
C	-1.88242387	-0.95001759	-1.46270662
C	-3.28735093	-0.59481448	-0.79104486
H	-3.93312435	0.09240588	-1.32616074
H	-3.80412236	-1.54241602	-0.69938873
H	-2.92690176	-0.85877783	1.38662110
H	-3.51883113	0.77053823	0.92342542
H	-1.83222641	-0.58934392	-2.49412004
H	0.24745243	-0.15423087	2.08733613
C	-1.10143984	1.59898975	1.05171759
H	-0.46659000	2.10774681	0.31790128
H	-1.93172752	2.27162062	1.23843805
H	-0.52573991	1.50947113	1.95342539
H	1.80589788	-0.77893458	-2.06460241
C	4.10237974	0.34044407	-0.73379190
H	4.83153133	0.35650568	0.06415856
H	4.39127515	-0.30082839	-1.55911781
H	4.12471089	1.34642789	-1.17244930
H	-1.78706267	-2.03625846	-1.51189696

Time = 217.50 fs
E(CASSCF/6-31G(d)) = -479.5000899

C	-0.82912373	-0.33719184	-0.44398475
C	0.52907449	-0.36492867	-0.14294520
C	0.89522862	-0.06082303	1.22999480
C	1.71537148	-0.55690330	-1.00838084
C	2.75955475	-0.01823368	-0.14320938
C	2.26650418	0.12580069	1.19692136
N	-1.59459178	0.41123693	0.42828879
C	-2.90971097	-0.07303258	0.60225642
H	2.90439203	0.18424205	2.04852641
C	-1.87915756	-0.94687164	-1.46662767
C	-3.28622293	-0.58965842	-0.79486288
H	-3.93355731	0.09687650	-1.33022300
H	-3.80095468	-1.53895438	-0.71219700
H	-2.92688665	-0.87329086	1.37257593
H	-3.52358592	0.76029871	0.92536838
H	-1.82748578	-0.56150405	-2.48793654
H	0.23129169	-0.14320007	2.07920124
C	-1.10344385	1.59709288	1.05828223
H	-0.48099537	2.11152459	0.32233204
H	-1.94758925	2.25853427	1.24552950
H	-0.52846842	1.50166240	1.96781067
H	1.80176184	-0.79520365	-2.05722821
C	4.10400210	0.33561100	-0.73980407
H	4.83939987	0.35017864	0.04952291
H	4.38526410	-0.31044380	-1.56234031
H	4.12100910	1.34477156	-1.17333950
H	-1.78336491	-2.03286850	-1.53947561

Time = 218.00 fs
E(CASSCF/6-31G(d)) = -479.4977784

C	-0.82236654	-0.34515711	-0.43700242
C	0.52735788	-0.36549621	-0.14172270
C	0.90615172	-0.05140024	1.22616822
C	1.71225278	-0.56119756	-1.00009336
C	2.75865647	-0.02029983	-0.13929261
C	2.25859524	0.13374163	1.19059664
N	-1.59843641	0.41071029	0.42868844
C	-2.91043818	-0.07558251	0.60281950
H	2.91815436	0.19609936	2.04024847
C	-1.87586774	-0.94362257	-1.47043074
C	-3.28490132	-0.58478795	-0.79865653
H	-3.93285085	0.10152700	-1.33469877

H	-3.79882257	-1.53509701	-0.72505567
H	-2.92771459	-0.88672800	1.35737579
H	-3.52910970	0.74931711	0.92756736
H	-1.82199034	-0.53339440	-2.48079196
H	0.21670139	-0.13264893	2.07003976
C	-1.10555926	1.59549566	1.06472694
H	-0.49528947	2.11516026	0.32772228
H	-1.96347519	2.24527120	1.25290698
H	-0.53231789	1.49414597	1.98217188
H	1.79698568	-0.81078851	-2.04921208
C	4.10560723	0.33092168	-0.74569457
H	4.84862831	0.34324914	0.03457181
H	4.37943659	-0.32085138	-1.56485598
H	4.11663386	1.34263320	-1.17350703
H	-1.77932109	-2.02865956	-1.56684830

Time = 218.50 fs
E(CASSCF/6-31G(d)) = -479.4939366

C	-0.81580398	-0.35309011	-0.43007379
C	0.52522584	-0.36635182	-0.14085932
C	0.91732831	-0.04207916	1.22232666
C	1.70944672	-0.56510127	-0.99113942
C	2.75752971	-0.02273322	-0.13628070
C	2.25071616	0.14168464	1.18455689
N	-1.60209887	0.41018370	0.42912835
C	-2.91127045	-0.07817895	0.60340220
H	2.93164749	0.20875564	2.03198332
C	-1.87254407	-0.94025309	-1.47410940
C	-3.28338242	-0.58021790	-0.80243241
H	-3.93092977	0.10637665	-1.33956119
H	-3.79776050	-1.53086190	-0.73789410
H	-2.92938399	-0.89912823	1.34129253
H	-3.53546987	0.73779884	0.93015281
H	-1.81570254	-0.50508842	-2.47279080
H	0.20460052	-0.12253406	2.05934187
C	-1.10783841	1.59417425	1.07109400
H	-0.50920503	2.11879305	0.33399993
H	-1.97901260	2.23179988	1.26060429
H	-0.53752815	1.48712647	1.99599744
H	1.79160190	-0.82563561	-2.04034945
C	4.10716057	0.32637397	-0.75144332
H	4.85921641	0.33572639	0.01945551
H	4.37376401	-0.33197654	-1.56680006
H	4.11165883	1.33992556	-1.17306230
H	-1.77500803	-2.02354939	-1.59393789

Time = 219.00 fs
E(CASSCF/6-31G(d)) = -479.4888095

C	-0.80954835	-0.36096696	-0.42321137
C	0.52275845	-0.36748994	-0.14032130
C	0.92846912	-0.03289179	1.21852443
C	1.70696002	-0.56863979	-0.98159740
C	2.75620881	-0.02553250	-0.13421989
C	2.24313244	0.14967059	1.17890653
N	-1.60553588	0.40965646	0.42960914
C	-2.91220723	-0.08084808	0.60396004
H	2.94408851	0.22215505	2.02316565
C	-1.86917602	-0.93674570	-1.47765704
C	-3.28166409	-0.57596070	-0.80619741
H	-3.92773108	0.11144832	-1.34478321
H	-3.79778770	-1.52627012	-0.75064778
H	-2.93188798	-0.91060116	1.32465114
H	-3.54272103	0.72595144	0.93323878
H	-1.80859417	-0.47666285	-2.46403638
H	0.19577156	-0.11281951	2.04678744
C	-1.11033608	1.59309942	1.07742348
H	-0.52246624	2.12258666	0.34105531
H	-1.99385758	2.21812212	1.26864502
H	-0.54427588	1.48079584	2.00886368
H	1.78565850	-0.83969594	-2.03045882
C	4.10863323	0.32195857	-0.75702854
H	4.87109113	0.32762967	0.00428708
H	4.36820898	-0.34371688	-1.56830342
H	4.10616744	1.33661036	-1.17214308
H	-1.77049838	-2.01748142	-1.62067326

Time = 219.50 fs
E(CASSCF/6-31G(d)) = -479.4829313

C	-0.80372007	-0.36876176	-0.41642700
C	0.52005403	-0.36890167	-0.14006511
C	0.93927411	-0.02387296	1.21479519
C	1.70479110	-0.57184110	-0.97154968
C	2.75473072	-0.02869359	-0.13314086
C	2.23612183	0.15774312	1.17372824
N	-1.60870583	0.40912597	0.43012895
C	-2.91324929	-0.08360976	0.60444657
H	2.95478877	0.23624110	2.01336334
C	-1.86575362	-0.93308383	-1.48106589
C	-3.27974602	-0.57202650	-0.80995879
H	-3.92320524	0.11676843	-1.35033787
H	-3.79890572	-1.52134210	-0.76325887

H	-2.93520578	-0.92131238	1.30781284
H	-3.55088946	0.71396348	0.93691415
H	-1.80064073	-0.44819617	-2.45462071
H	0.19080987	-0.10347136	2.03226887
C	-1.11310589	1.59223922	1.08375164
H	-0.53480949	2.12671164	0.34876056
H	-2.00771955	2.20427580	1.27704125
H	-0.55266483	1.47531994	2.02045140
H	1.77921872	-0.85292846	-2.01940562
C	4.11000286	0.31765858	-0.76242737
H	4.88410499	0.31898834	-0.01086262
H	4.36272668	-0.35594162	-1.56948791
H	4.10025157	1.33269897	-1.17090943
H	-1.76585869	-2.01042682	-1.64699336

Time = 220.00 fs
E(CASSCF/6-31G(d)) = -479.4767862

C	-0.79845361	-0.37644541	-0.40973286
C	0.51723085	-0.37057371	-0.14003676
C	0.94946477	-0.01505714	1.21115392
C	1.70294212	-0.57473072	-0.96107570
C	2.75312309	-0.03221338	-0.13306332
C	2.22994313	0.16594314	1.16907826
N	-1.61156682	0.40858521	0.43068374
C	-2.91440232	-0.08647587	0.60481438
H	2.96319780	0.25097316	2.00231289
C	-1.86226783	-0.92925248	-1.48432660
C	-3.27762991	-0.56842346	-0.81372467
H	-3.91731787	0.12236653	-1.35619664
H	-3.80109610	-1.51609263	-0.77567421
H	-2.93928495	-0.93146429	1.29115528
H	-3.55995718	0.70198603	0.94123543
H	-1.79182063	-0.41976953	-2.44461841
H	0.19008550	-0.09445282	2.01590410
C	-1.11619776	1.59156255	1.09010715
H	-0.54600614	2.13132423	0.35699408
H	-2.02037995	2.19033170	1.28579230
H	-0.56271050	1.47081257	2.03055503
H	1.77235739	-0.86530408	-2.00712292
C	4.11125418	0.31344944	-0.76761720
H	4.89804182	0.30984121	-0.02596320
H	4.35726905	-0.36849249	-1.57046377
H	4.09401136	1.32824931	-1.16953662
H	-1.76114844	-2.00238404	-1.67284796

Time = 220.50 fs

E(CASSCF/6-31G(d)) = -479.4707212

C	-0.79388428	-0.38399004	-0.40314418
C	0.51441007	-0.37248931	-0.14017651
C	0.95879765	-0.00647539	1.20760079
C	1.70141965	-0.57733418	-0.95025389
C	2.75140781	-0.03608663	-0.13399250
C	2.22482665	0.17430697	1.16498526
N	-1.61408186	0.40802619	0.43127243
C	-2.91567564	-0.08945035	0.60501818
H	2.96893128	0.26632772	1.98992851
C	-1.85871020	-0.92523637	-1.48742993
C	-3.27531923	-0.56515761	-0.81750392
H	-3.91005152	0.12827490	-1.36232947
H	-3.80432154	-1.51053126	-0.78784612
H	-2.94404516	-0.94128211	1.27505194
H	-3.56985490	0.69012221	0.94622183
H	-1.78212495	-0.39147200	-2.43408750
H	0.19371948	-0.08572363	1.99803397
C	-1.11965710	1.59103949	1.09650560
H	-0.55587775	2.13655389	0.36565750
H	-2.03169978	2.17639741	1.29488861
H	-0.57432623	1.46732382	2.03910065
H	1.76516046	-0.87681357	-1.99361715
C	4.11237859	0.30929981	-0.77257701
H	4.91262338	0.30023661	-0.04101804
H	4.35178721	-0.38118720	-1.57132759
H	4.08755211	1.32335849	-1.16820849
H	-1.75641897	-1.99338000	-1.69819566

Time = 221.00 fs
E(CASSCF/6-31G(d)) = -479.4651095

C	-0.79013651	-0.39137132	-0.39667887
C	0.51170536	-0.37462953	-0.14042245
C	0.96706587	0.00184534	1.20412057
C	1.70022738	-0.57968026	-0.93916453
C	2.74960915	-0.04030220	-0.13591461
C	2.22097454	0.18286660	1.16145146
N	-1.61622237	0.40744203	0.43189803
C	-2.91708045	-0.09253103	0.60501791
H	2.97178829	0.28229537	1.97630010
C	-1.85507289	-0.92101921	-1.49036773
C	-3.27281917	-0.56223278	-0.82130596
H	-3.90140596	0.13452745	-1.36870542
H	-3.80852752	-1.50466339	-0.79973474
H	-2.94939011	-0.95099297	1.25985005

H	-3.58046032	0.67842365	0.95185235
H	-1.77156147	-0.36340235	-2.42306901
H	0.20156251	-0.07724061	1.97921784
C	-1.12352276	1.59064150	1.10294754
H	-0.56430742	2.14249209	0.37468896
H	-2.04162587	2.16261962	1.30431461
H	-0.58732666	1.46484487	2.04615410
H	1.75772498	-0.88747199	-1.97897058
C	4.11337292	0.30517300	-0.77728849
H	4.92752184	0.29023266	-0.05605333
H	4.34623297	-0.39382530	-1.57216283
H	4.08098045	1.31815250	-1.16711071
H	-1.75171234	-1.98346923	-1.72300434

Time = 221.50 fs
E(CASSCF/6-31G(d)) = -479.4602220

C	-0.78731802	-0.39856870	-0.39035610
C	0.50921865	-0.37697406	-0.14071252
C	0.97410616	0.00988323	1.20068136
C	1.69936164	-0.58180208	-0.92789050
C	2.74775722	-0.04484135	-0.13879479
C	2.21855457	0.19164840	1.15845359
N	-1.61796927	0.40682732	0.43256693
C	-2.91862898	-0.09571208	0.60478106
H	2.97176353	0.29888291	1.96168890
C	-1.85134891	-0.91658377	-1.49313285
C	-3.27013653	-0.55965041	-0.82514054
H	-3.89139727	0.14115740	-1.37529175
H	-3.81364288	-1.49849031	-0.81130973
H	-2.95521308	-0.96079881	1.24584850
H	-3.59160004	0.66689229	0.95806560
H	-1.76015493	-0.33566918	-2.41158704
H	0.21317653	-0.06895977	1.96023090
C	-1.12782492	1.59034250	1.10941844
H	-0.57124808	2.14918093	0.38407402
H	-2.05019356	2.14918028	1.31404903
H	-0.60143974	1.46331333	2.05191530
H	1.75015709	-0.89731983	-1.96334219
C	4.11423800	0.30102886	-0.78173755
H	4.94237812	0.27989674	-0.07110367
H	4.34056253	-0.40619643	-1.57304352
H	4.07440226	1.31277374	-1.16642310
H	-1.74706045	-1.97272998	-1.74725098

Time = 222.00 fs
E(CASSCF/6-31G(d)) = -479.4562272

C	-0.78551629	-0.40556531	-0.38419475
C	0.50703703	-0.37950173	-0.14098574
C	0.97981144	0.01762317	1.19723378
C	1.69881046	-0.58373664	-0.91651666
C	2.74588878	-0.04967766	-0.14257801
C	2.21768775	0.20067114	1.15594469
N	-1.61931289	0.40617747	0.43328786
C	-2.92033402	-0.09898642	0.60428395
H	2.96905665	0.31611902	1.94652292
C	-1.84753254	-0.91191272	-1.49571874
C	-3.26727965	-0.55740942	-0.82901770
H	-3.88005684	0.14819519	-1.38205251
H	-3.81958150	-1.49200920	-0.82255086
H	-2.96139839	-0.97084888	1.23327855
H	-3.60305648	0.65548865	0.96476291
H	-1.74794687	-0.30838772	-2.39965232
H	0.22782727	-0.06083982	1.94205843
C	-1.13258365	1.59012006	1.11588981
H	-0.57672626	2.15660585	0.39385146
H	-2.05752535	2.13628881	1.32406475
H	-0.61632320	1.46261886	2.05670177
H	1.74256972	-0.90642264	-1.94696240
C	4.11497704	0.29682592	-0.78591565
H	4.95682285	0.26930387	-0.08619776
H	4.33473953	-0.41808934	-1.57403882
H	4.06792111	1.30736541	-1.16631067
H	-1.74248536	-1.96125895	-1.77092193

Time = 222.50 fs
E(CASSCF/6-31G(d)) = -479.4530971

C	-0.78479592	-0.41234687	-0.37821252
C	0.50522936	-0.38219098	-0.14118254
C	0.98413940	0.02505806	1.19371193
C	1.69855307	-0.58552483	-0.90513018
C	2.74404797	-0.05477672	-0.14718992
C	2.21843812	0.20994360	1.15385531
N	-1.62025272	0.40548829	0.43407127
C	-2.92220791	-0.10234850	0.60351236
H	2.96407574	0.33405926	1.93139094
C	-1.84361928	-0.90698960	-1.49811915
C	-3.26425844	-0.55550579	-0.83294762
H	-3.86743053	0.15566552	-1.38894782
H	-3.82624380	-1.48521577	-0.83344879
H	-2.96782250	-0.98121962	1.22229192
H	-3.61458078	0.64414512	0.97181404

H	-1.73499374	-0.28167790	-2.38726716
H	0.24450347	-0.05284828	1.92587259
C	-1.13780801	1.58995596	1.12232160
H	-0.58083683	2.16469602	0.40410705
H	-2.06382521	2.12417520	1.33432853
H	-0.63158375	1.46261179	2.06092611
H	1.73507871	-0.91486730	-1.93012229
C	4.11559386	0.29252371	-0.78982085
H	4.97049571	0.25853451	-0.10134611
H	4.32873751	-0.42930094	-1.57521683
H	4.06163674	1.30205418	-1.16691518
H	-1.73799810	-1.94916541	-1.79401014

Time = 223.00 fs
E(CASSCF/6-31G(d)) = -479.4507549

C	-0.78519602	-0.41890133	-0.37242496
C	0.50384316	-0.38502053	-0.14124557
C	0.98711581	0.03218981	1.19003783
C	1.69856004	-0.58721130	-0.89382082
C	2.74228691	-0.06009617	-0.15253828
C	2.22080515	0.21946288	1.15209571
N	-1.62079713	0.40475575	0.43492875
C	-2.92426152	-0.10579629	0.60246141
H	2.95743141	0.35279152	1.91703055
C	-1.83960615	-0.90179997	-1.50032776
C	-3.26108423	-0.55393234	-0.83694051
H	-3.85357665	0.16358478	-1.39593223
H	-3.83351896	-1.47810480	-0.84400547
H	-2.97435466	-0.99190120	1.21295398
H	-3.62590730	0.63278084	0.97906476
H	-1.72136552	-0.25565845	-2.37443066
H	0.26197920	-0.04496463	1.91297524
C	-1.14349601	1.58983628	1.12866616
H	-0.58373496	2.17332923	0.41496370
H	-2.06936623	2.11307743	1.34479986
H	-0.64680434	1.46311464	2.06505855
H	1.72779951	-0.92275823	-1.91315980
C	4.11609133	0.28808514	-0.79345862
H	4.98306532	0.24767160	-0.11653111
H	4.32254219	-0.43964593	-1.57664959
H	4.05564471	1.29693483	-1.16834780
H	-1.73359976	-1.93656229	-1.81651410

Time = 223.50 fs
E(CASSCF/6-31G(d)) = -479.4491156

C	-0.78672774	-0.42521911	-0.36684524
C	0.50290168	-0.38796953	-0.14112096
C	0.98882867	0.03902914	1.18613068
C	1.69879399	-0.58884399	-0.88268034
C	2.74066568	-0.06558635	-0.15851611
C	2.22472136	0.22921331	1.15055987
N	-1.62096325	0.40397610	0.43587324
C	-2.92650324	-0.10933273	0.60113485
H	2.94991160	0.37243912	1.90429696
C	-1.83549143	-0.89633218	-1.50233822
C	-3.25776971	-0.55267840	-0.84100666
H	-3.83856595	0.17195782	-1.40295297
H	-3.84128730	-1.47067320	-0.85423377
H	-2.98085880	-1.00279768	1.20524703
H	-3.63677146	0.62131763	0.98634661
H	-1.70714655	-0.23044414	-2.36114738
H	0.27894267	-0.03717795	1.90468371
C	-1.14963487	1.58975137	1.13487342
H	-0.58562173	2.18234207	0.42656525
H	-2.07447062	2.10323031	1.35543042
H	-0.66157569	1.46393599	2.06957935
H	1.72084197	-0.93021279	-1.89643809
C	4.11647006	0.28347854	-0.79684225
H	4.99424545	0.23679773	-0.13169888
H	4.31615350	-0.44896554	-1.57841631
H	4.05003564	1.29205860	-1.17068299
H	-1.72928149	-1.92355991	-1.83843214

Time = 224.00 fs
E(CASSCF/6-31G(d)) = -479.4482595

C	-0.78937408	-0.43129208	-0.36148353
C	0.50240305	-0.39101845	-0.14075843
C	0.98940955	0.04559420	1.18192212
C	1.69920974	-0.59047461	-0.87180393
C	2.73925296	-0.07119053	-0.16500303
C	2.23005971	0.23916689	1.14913054
N	-1.62077639	0.40314578	0.43691832
C	-2.92893775	-0.11296586	0.59954352
H	2.94242238	0.39315704	1.89410910
C	-1.83127500	-0.89057880	-1.50414408
C	-3.25432866	-0.55172963	-0.84515619
H	-3.82247935	0.18077610	-1.40994951
H	-3.84942302	-1.46292135	-0.86415817
H	-2.98719562	-1.01373608	1.19907876
H	-3.64692524	0.60969344	0.99348535
H	-1.69243195	-0.20613842	-2.34743134

H	0.29419159	-0.02947864	1.90215592
C	-1.15620203	1.58969489	1.14089723
H	-0.58672874	2.19154392	0.43905703
H	-2.07948573	2.09484925	1.36616146
H	-0.67553246	1.46488701	2.07492349
H	1.71430810	-0.93735302	-1.88032425
C	4.11672740	0.27867930	-0.79999290
H	5.00380865	0.22599152	-0.14675684
H	4.30958566	-0.45713437	-1.58060568
H	4.04489517	1.28742510	-1.17395539
H	-1.72502597	-1.91025678	-1.85976109

Time = 224.50 fs
E(CASSCF/6-31G(d)) = -479.4484397

C	-0.79308994	-0.43711376	-0.35634683
C	0.50232011	-0.39414952	-0.14011240
C	0.98900951	0.05190843	1.17737537
C	1.69975528	-0.59215843	-0.86129004
C	2.73812559	-0.07684589	-0.17186853
C	2.23664698	0.24928522	1.14768813
N	-1.62026968	0.40226166	0.43807816
C	-2.93156494	-0.11670833	0.59770356
H	2.93588958	0.41511861	1.88735527
C	-1.82695840	-0.88453746	-1.50573889
C	-3.25077573	-0.55106785	-0.84939890
H	-3.80540712	0.19001641	-1.41685311
H	-3.85779768	-1.45485515	-0.87381456
H	-2.99322579	-1.02448661	1.19429695
H	-3.65615245	0.59787326	1.00031020
H	-1.67732603	-0.18282735	-2.33331114
H	0.30686199	-0.02184398	1.90617051
C	-1.16316662	1.58966332	1.14670187
H	-0.58730097	2.20073247	0.45256731
H	-2.08475385	2.08811453	1.37692157
H	-0.68838765	1.46579653	2.08142408
H	1.70828934	-0.94429817	-1.86516441
C	4.11685680	0.27367084	-0.80293930
H	5.01159592	0.21532431	-0.16157322
H	4.30286700	-0.46406541	-1.58331646
H	4.04030336	1.28298137	-1.17815870
H	-1.72080842	-1.89673326	-1.88049316

Time = 225.00 fs
E(CASSCF/6-31G(d)) = -479.4499742

C	-0.79780378	-0.44267891	-0.35143914

C	0.50260220	-0.39734704	-0.13914262
C	0.98777491	0.05799783	1.17250256
C	1.70037329	-0.59395361	-0.85123995
C	2.73736675	-0.08248501	-0.17897568
C	2.24428363	0.25952287	1.14612247
N	-1.61948334	0.40132111	0.43936733
C	-2.93437922	-0.12057604	0.59563450
H	2.93112642	0.43848874	1.88476539
C	-1.82254470	-0.87821144	-1.50711655
C	-3.24712616	-0.55067094	-0.85374416
H	-3.78744932	0.19963945	-1.42358707
H	-3.86628269	-1.44648886	-0.88324963
H	-2.99881379	-1.03478767	1.19070774
H	-3.66428132	0.58585787	1.00666173
H	-1.66194054	-0.16057650	-2.31883495
H	0.31661382	-0.01422451	1.91692735
C	-1.17049143	1.58965539	1.15226750
H	-0.58758091	2.20970744	0.46719081
H	-2.09057998	2.08315689	1.38762422
H	-0.69996075	1.46652371	2.08926494
H	1.70286331	-0.95115605	-1.85125904
C	4.11684770	0.26844478	-0.80571708
H	5.01752275	0.20485763	-0.17597874
H	4.29603929	-0.46971262	-1.58665545
H	4.03633381	1.27862736	-1.18324711
H	-1.71659742	-1.88304820	-1.90061098

Time = 225.50 fs
E(CASSCF/6-31G(d)) = -479.4529927

C	-0.80342040	-0.44798339	-0.34676139
C	0.50317810	-0.40059780	-0.13781509
C	0.98583286	0.06388956	1.16737404
C	1.70100292	-0.59592043	-0.84175681
C	2.73706369	-0.08803755	-0.18618496
C	2.25276517	0.26983237	1.14434598
N	-1.61846396	0.40032217	0.44080054
C	-2.93736888	-0.12458654	0.59335734
H	2.92869085	0.46338406	1.88676821
C	-1.81803870	-0.87161017	-1.50827165
C	-3.24339544	-0.55051297	-0.85820067
H	-3.76871550	0.20959043	-1.43006806
H	-3.87475255	-1.43784664	-0.89252060
H	-3.00383202	-1.04437290	1.18809355
H	-3.67119387	0.57368833	1.01239859
H	-1.64639182	-0.13942637	-2.30407198
H	0.32369272	-0.00654152	1.93394547

C	-1.17813554	1.58967162	1.15759389
H	-0.58779445	2.21828383	0.48297602
H	-2.09720188	2.08004388	1.39816517
H	-0.71019399	1.46696593	2.09845280
H	1.69809287	-0.95801555	-1.83884309
C	4.11668574	0.26300052	-0.80836785
H	5.02158136	0.19464053	-0.18977194
H	4.28915608	-0.47407099	-1.59073403
H	4.03305253	1.27422676	-1.18913930
H	-1.71235610	-1.86923671	-1.92008598

Time = 226.00 fs
E(CASSCF/6-31G(d)) = -479.4572284

C	-0.80982472	-0.45302350	-0.34231172
C	0.50396018	-0.40389149	-0.13610223
C	0.98328894	0.06961139	1.16211444
C	1.70158268	-0.59811988	-0.83294390
C	2.73730394	-0.09343260	-0.19335854
C	2.26190117	0.28016947	1.14230483
N	-1.61726354	0.39926342	0.44239223
C	-2.94051577	-0.12875727	0.59089285
H	2.92877816	0.48982866	1.89337546
C	-1.81344703	-0.86474923	-1.50920002
C	-3.23959895	-0.55056427	-0.86277616
H	-3.74932549	0.21979953	-1.43620819
H	-3.88308684	-1.42896473	-0.90169435
H	-3.00816509	-1.05299588	1.18623017
H	-3.67683210	0.56144837	1.01740246
H	-1.63079825	-0.11939148	-2.28911356
H	0.32883651	0.00130237	1.95611221
C	-1.18605724	1.58971400	1.16270153
H	-0.58813962	2.22630243	0.49991611
H	-2.10476660	2.07877114	1.40842115
H	-0.71915345	1.46706145	2.10881589
H	1.69402396	-0.96494016	-1.82807226
C	4.11635348	0.25734415	-0.81093812
H	5.02383908	0.18470816	-0.20272632
H	4.28228075	-0.47717408	-1.59566194
H	4.03051641	1.26962168	-1.19572402
H	-1.70804291	-1.85531129	-1.93887566

Time = 226.50 fs
E(CASSCF/6-31G(d)) = -479.4620246

C	-0.81688595	-0.45779679	-0.33808528
C	0.50484910	-0.40722077	-0.13398450

C	0.98023771	0.07519303	1.15688732
C	1.70205282	-0.60061185	-0.82490116
C	2.73817135	-0.09860119	-0.20036444
C	2.27152474	0.29049781	1.13998462
N	-1.61593843	0.39814448	0.44415645
C	-2.94379560	-0.13310404	0.58826029
H	2.93118829	0.51772020	1.90413135
C	-1.80877802	-0.85765001	-1.50989894
C	-3.23575158	-0.55079203	-0.86747723
H	-3.72940904	0.23018620	-1.44191745
H	-3.89117415	-1.41989028	-0.91084655
H	-3.01171280	-1.06045245	1.18490073
H	-3.68120051	0.54926140	1.02158240
H	-1.61527754	-0.10045608	-2.27407018
H	0.33306808	0.00940258	1.98186366
C	-1.19421704	1.58978589	1.16762998
H	-0.58877761	2.23363787	0.51794705
H	-2.11331692	2.07925795	1.41825003
H	-0.72701591	1.46678493	2.12002822
H	1.69068786	-0.97196724	-1.81901788
C	4.11583163	0.25148703	-0.81347787
H	5.02443122	0.17507996	-0.21460115
H	4.27548391	-0.47908976	-1.60154209
H	4.02877187	1.26465078	-1.20286758
H	-1.70361301	-1.84126461	-1.95692689

Time = 227.00 fs
E(CASSCF/6-31G(d)) = -479.4665709

C	-0.82446174	-0.46230177	-0.33407444
C	0.50573917	-0.41058116	-0.13145053
C	0.97677478	0.08066677	1.15187410
C	1.70235886	-0.60345269	-0.81772129
C	2.73974057	-0.10347896	-0.20708063
C	2.28149422	0.30079089	1.13740783
N	-1.61454829	0.39696589	0.44610664
C	-2.94717838	-0.13763968	0.58547645
H	2.93538497	0.54681958	1.91815776
C	-1.80404169	-0.85033901	-1.51036766
C	-3.23186749	-0.55116081	-0.87230911
H	-3.70910708	0.24066193	-1.44710698
H	-3.89891324	-1.41068165	-0.92006005
H	-3.01439329	-1.06659625	1.18390753
H	-3.68436543	0.53728715	1.02487670
H	-1.59994480	-0.08257592	-2.25906959
H	0.33746694	0.01783345	2.00942710
C	-1.20258006	1.58989176	1.17243392

H	-0.58982747	2.24020285	0.53694816
H	-2.12279117	2.08135083	1.42749400
H	-0.73404416	1.46614026	2.13165438
H	1.68810026	-0.97910759	-1.81166947
C	4.11510061	0.24544409	-0.81603917
H	5.02354995	0.16575978	-0.22515310
H	4.26884108	-0.47991416	-1.60846225
H	4.02785304	1.25916678	-1.21042139
H	-1.69901919	-1.82707573	-1.97417616

Time = 227.50 fs
E(CASSCF/6-31G(d)) = -479.4701844

C	-0.83240366	-0.46653707	-0.33026918
C	0.50652383	-0.41397128	-0.12849581
C	0.97300289	0.08606739	1.14725404
C	1.70245558	-0.60669240	-0.81148633
C	2.74207132	-0.10800923	-0.21339853
C	2.29169069	0.31103214	1.13462434
N	-1.61315471	0.39572894	0.44825522
C	-2.95062890	-0.14237278	0.58255491
H	2.94061767	0.57676900	1.93427927
C	-1.79924967	-0.84284678	-1.51060781
C	-3.22795964	-0.55163306	-0.87727522
H	-3.68857133	0.25113397	-1.45169318
H	-3.90621448	-1.40140892	-0.92942412
H	-3.01614596	-1.07135071	1.18308175
H	-3.68645086	0.52571534	1.02725231
H	-1.58490997	-0.06568294	-2.24425123
H	0.34299714	0.02663885	2.03704574
C	-1.21111774	1.59003632	1.17717779
H	-0.59136253	2.24595264	0.55674352
H	-2.13303603	2.08483409	1.43598555
H	-0.74055514	1.46515122	2.14320856
H	1.68625992	-0.98634853	-1.80594598
C	4.11414271	0.23923214	-0.81867468
H	5.02142816	0.15673679	-0.23415064
H	4.26243014	-0.47976386	-1.61648549
H	4.02778103	1.25305136	-1.21822911
H	-1.69421123	-1.81271697	-1.99055203

Time = 228.00 fs
E(CASSCF/6-31G(d)) = -479.4725561

C	-0.84055957	-0.47050214	-0.32665687
C	0.50710032	-0.41739194	-0.12512431
C	0.96903912	0.09143197	1.14319011

C	1.70230963	-0.61037212	-0.80626241
C	2.74520322	-0.11214493	-0.21922561
C	2.30200722	0.32121204	1.13169895
N	-1.61182045	0.39443586	0.45061364
C	-2.95410819	-0.14730680	0.57950610
H	2.94606698	0.60713011	1.95117785
C	-1.79441497	-0.83520671	-1.51062375
C	-3.22403986	-0.55217013	-0.88237722
H	-3.66796554	0.26151006	-1.45560064
H	-3.91300007	-1.39215091	-0.93903114
H	-3.01693284	-1.07471351	1.18228889
H	-3.68763015	0.51475587	1.02870353
H	-1.57027760	-0.04968728	-2.22976296
H	0.35040016	0.03582862	2.06313523
C	-1.21980825	1.59022428	1.18193009
H	-0.59341168	2.25088195	0.57711223
H	-2.14382840	2.08944403	1.44355551
H	-0.74688959	1.46385090	2.15420568
H	1.68515117	-0.99365962	-1.80171134
C	4.11294431	0.23286822	-0.82143608
H	5.01831944	0.14798729	-0.24138896
H	4.25632905	-0.47876958	-1.62564344
H	4.02856247	1.24622939	-1.22613452
H	-1.68913887	-1.79816257	-2.00597906

Time = 228.50 fs
E(CASSCF/6-31G(d)) = -479.4737501

C	-0.84877905	-0.47419652	-0.32322247
C	0.50737422	-0.42084629	-0.12134606
C	0.96500987	0.09679845	1.13981967
C	1.70190259	-0.61452169	-0.80209715
C	2.74915155	-0.11585107	-0.22448732
C	2.31234527	0.33132564	1.12869917
N	-1.61060781	0.39308945	0.45319188
C	-2.95757461	-0.15243915	0.57633704
H	2.95096240	0.63743214	1.96754501
C	-1.78955171	-0.82745354	-1.51042274
C	-3.22011839	-0.55273309	-0.88761452
H	-3.64746309	0.27170386	-1.45876633
H	-3.91920497	-1.38299358	-0.94897627
H	-3.01673994	-1.07675971	1.18143403
H	-3.68811405	0.50462653	1.02924644
H	-1.55614509	-0.03448476	-2.21575239
H	0.36016800	0.04538619	2.08637698
C	-1.22863641	1.59045918	1.18675766
H	-0.59596070	2.25502554	0.59779505

H	-2.15490511	2.09488783	1.45004262
H	-0.75338217	1.46227272	2.16421541
H	1.68474289	-1.00099997	-1.79879482
C	4.11149856	0.22636825	-0.82437256
H	5.01447306	0.13947722	-0.24670791
H	4.25061319	-0.47706746	-1.63592769
H	4.03019004	1.23867668	-1.23398689
H	-1.68375050	-1.78339565	-2.02038344

Time = 229.00 fs
E(CASSCF/6-31G(d)) = -479.4741427

C	-0.85691592	-0.47761972	-0.31994855
C	0.50726337	-0.42433905	-0.11717721
C	0.96104890	0.10220428	1.13725015
C	1.70123214	-0.61915791	-0.79901637
C	2.75390386	-0.11910576	-0.22912784
C	2.32260909	0.34136922	1.12568601
N	-1.60957778	0.39169317	0.45599835
C	-2.96098570	-0.15776049	0.57305202
H	2.95466297	0.66721368	1.98219184
C	-1.78467465	-0.81962192	-1.51001522
C	-3.21620404	-0.55328367	-0.89298431
H	-3.62724636	0.28163840	-1.46114224
H	-3.92477492	-1.37402686	-0.95935410
H	-3.01557754	-1.07763831	1.18046317
H	-3.68813554	0.49553829	1.02891281
H	-1.54260280	-0.01996383	-2.20236109
H	0.37254534	0.05527606	2.10575447
C	-1.23759237	1.59074288	1.19172107
H	-0.59895903	2.25845058	0.61850711
H	-2.16599210	2.10086211	1.45530305
H	-0.76033883	1.46044310	2.17289611
H	1.68498976	-1.00832532	-1.79701080
C	4.10980778	0.21974599	-0.82752921
H	5.01011166	0.13116575	-0.25000652
H	4.24535351	-0.47479452	-1.64728578
H	4.03264242	1.23042629	-1.24164697
H	-1.67799512	-1.76841533	-2.03369655

Time = 229.50 fs
E(CASSCF/6-31G(d)) = -479.4742504

C	-0.86483096	-0.48077119	-0.31681531
C	0.50670047	-0.42787594	-0.11263867
C	0.95729221	0.10768490	1.13555696
C	1.70031219	-0.62428364	-0.79702309

C	2.75941872	-0.12190087	-0.23310961
C	2.33270489	0.35133825	1.12270823
N	-1.60878900	0.39025095	0.45903970
C	-2.96429991	-0.16325412	0.56965319
H	2.95669555	0.69605591	1.99411920
C	-1.77979888	-0.81174500	-1.50941488
C	-3.21230417	-0.55378530	-0.89848141
H	-3.60750391	0.29125116	-1.46269780
H	-3.92966577	-1.36534059	-0.97025684
H	-3.01348017	-1.07756545	1.17936305
H	-3.68793152	0.48767890	1.02774095
H	-1.52973415	-0.00601259	-2.18971745
H	0.38755532	0.06545321	2.12056372
C	-1.24667018	1.59107490	1.19687055
H	-0.60232804	2.26124896	0.63895117
H	-2.17683231	2.10707050	1.45921875
H	-0.76801951	1.45837620	2.18002138
H	1.68583387	-1.01559527	-1.79617639
C	4.10788560	0.21301250	-0.83094578
H	5.00540918	0.12300857	-0.25125682
H	4.24061429	-0.47208411	-1.65961879
H	4.03588577	1.22156921	-1.24899058
H	-1.67182294	-1.75324217	-2.04586125

Time = 230.00 fs
E(CASSCF/6-31G(d)) = -479.4745315

C	-0.87239396	-0.48364996	-0.31380124
C	0.50563536	-0.43146300	-0.10775532
C	0.95387474	0.11327229	1.13478285
C	1.69917165	-0.62988786	-0.79609682
C	2.76562595	-0.12424147	-0.23641264
C	2.34254053	0.36122569	1.11979953
N	-1.60829703	0.38876757	0.46232093
C	-2.96747845	-0.16889582	0.56614181
H	2.95676291	0.72360092	2.00254648
C	-1.77493897	-0.80385292	-1.50863876
C	-3.20842499	-0.55420383	-0.90409846
H	-3.58842879	0.30049521	-1.46342273
H	-3.93384031	-1.35702376	-0.98177133
H	-3.01050684	-1.07681675	1.17815871
H	-3.68772663	0.48119587	1.02576785
H	-1.51761573	0.00747134	-2.17793134
H	0.40502668	0.07586917	2.13041021
C	-1.25586598	1.59145245	1.20224314
H	-0.60597066	2.26352818	0.65883039
H	-2.18720860	2.11324142	1.46170594

H	-0.77662351	1.45607175	2.18549618
H	1.68720565	-1.02277760	-1.79612613
C	4.10575820	0.20617593	-0.83465542
H	5.00047645	0.11496185	-0.25051215
H	4.23645086	-0.46906406	-1.67278125
H	4.03987410	1.21225044	-1.25591119
H	-1.66518617	-1.73792292	-2.05683306

Time = 230.50 fs
E(CASSCF/6-31G(d)) = -479.4754062

C	-0.87948550	-0.48625481	-0.31088207
C	0.50403513	-0.43510641	-0.10255467
C	0.95092709	0.11899439	1.13493652
C	1.69785217	-0.63594615	-0.79619534
C	2.77242911	-0.12614525	-0.23903198
C	2.35202721	0.37102139	1.11697736
N	-1.60815312	0.38724734	0.46584479
C	-2.97048731	-0.17465374	0.56251902
H	2.95473849	0.74957362	2.00693206
C	-1.77010917	-0.79597219	-1.50770691
C	-3.20457134	-0.55450884	-0.90982573
H	-3.57021244	0.30934577	-1.46332493
H	-3.93726907	-1.34915419	-0.99397791
H	-3.00673784	-1.07570379	1.17690657
H	-3.68771046	0.47618189	1.02301693
H	-1.50631931	0.02057871	-2.16708882
H	0.42461640	0.08647971	2.13521550
C	-1.26517639	1.59187035	1.20786031
H	-0.60978664	2.26539529	0.67786599
H	-2.19696068	2.11913592	1.46271595
H	-0.78628756	1.45351211	2.18936022
H	1.68902821	-1.02985509	-1.79672337
C	4.10346490	0.19924202	-0.83868409
H	4.99535442	0.10698334	-0.24791163
H	4.23290865	-0.46585637	-1.68658871
H	4.04455402	1.20266337	-1.26232123
H	-1.65804065	-1.72252918	-2.06658994

Time = 231.00 fs
E(CASSCF/6-31G(d)) = -479.4769510

C	-0.88599817	-0.48858395	-0.30803222
C	0.50188489	-0.43881153	-0.09706645
C	0.94857542	0.12487390	1.13599200
C	1.69640512	-0.64242222	-0.79725556
C	2.77970841	-0.12764071	-0.24097654

C	2.36107898	0.38071104	1.11424311
N	-1.60840400	0.38569503	0.46961220
C	-2.97329845	-0.18048947	0.55878752
H	2.95065170	0.77378302	2.00696380
C	-1.76532213	-0.78812447	-1.50664238
C	-3.20074738	-0.55467385	-0.91565170
H	-3.55304312	0.31779673	-1.46243283
H	-3.93992497	-1.34180132	-1.00694634
H	-3.00227448	-1.07456199	1.17568594
H	-3.68802801	0.47266172	1.01949432
H	-1.49591111	0.03337870	-2.15724960
H	0.44582553	0.09724588	2.13522307
C	-1.27459683	1.59232167	1.21372684
H	-0.61368273	2.26694730	0.69580781
H	-2.20599664	2.12456138	1.46224116
H	-0.79707676	1.45066435	2.19178945
H	1.69121756	-1.03682552	-1.79786665
C	4.10105725	0.19221483	-0.84305008
H	4.99001904	0.09903535	-0.24367517
H	4.23002107	-0.46257861	-1.70082276
H	4.04986471	1.19303680	-1.26815123
H	-1.65034653	-1.70715905	-2.07512781

Time = 231.50 fs
E(CASSCF/6-31G(d)) = -479.4790841

C	-0.89183782	-0.49063488	-0.30522484
C	0.49918713	-0.44258250	-0.09132143
C	0.94694096	0.13092787	1.13788713
C	1.69488823	-0.64926974	-0.79919650
C	2.78732518	-0.12876527	-0.24226686
C	2.36961310	0.39027568	1.11158212
N	-1.60909095	0.38411533	0.47362211
C	-2.97589089	-0.18635954	0.55495260
H	2.94467550	0.79613180	2.00255904
C	-1.76058862	-0.78032629	-1.50547071
C	-3.19695669	-0.55467684	-0.92156333
H	-3.53710107	0.32586115	-1.46079368
H	-3.94178200	-1.33502193	-1.02073371
H	-2.99723624	-1.07372534	1.17458629
H	-3.68876982	0.47058527	1.01518440
H	-1.48645191	0.04591678	-2.14844601
H	0.46801173	0.10813732	2.13101760
C	-1.28412040	1.59279831	1.21983030
H	-0.61758499	2.26825845	0.71244685
H	-2.21429888	2.12937687	1.46031418
H	-0.80898619	1.44748195	2.19308766

H	1.69368611	-1.04370317	-1.79949225
C	4.09859727	0.18509797	-0.84776416
H	4.98439431	0.09108491	-0.23809367
H	4.22780860	-0.45934606	-1.71524245
H	4.05574138	1.18362055	-1.27334739
H	-1.64206948	-1.69193407	-2.08246308

Time = 232.00 fs
E(CASSCF/6-31G(d)) = -479.4815118

C	-0.89692514	-0.49240412	-0.30243210
C	0.49596172	-0.44642183	-0.08535108
C	0.94614093	0.13716748	1.14052246
C	1.69336276	-0.65643390	-0.80192158
C	2.79512589	-0.12956372	-0.24293340
C	2.37754890	0.39969054	1.10896371
N	-1.61024904	0.38251231	0.47787134
C	-2.97825142	-0.19221744	0.55102314
H	2.93711629	0.81662491	1.99386408
C	-1.75591731	-0.77258919	-1.50421943
C	-3.19320251	-0.55450054	-0.92754642
H	-3.52255402	0.33356860	-1.45847162
H	-3.94281285	-1.32885724	-1.03538177
H	-2.99175732	-1.07350004	1.17369222
H	-3.68996860	0.46982671	1.01005013
H	-1.47799573	0.05821310	-2.14068331
H	0.49040091	0.11913387	2.12354310
C	-1.29373675	1.59329164	1.22614180
H	-0.62144894	2.26936953	0.72762516
H	-2.22192539	2.13349514	1.45700543
H	-0.82194605	1.44390825	2.19366980
H	1.69634547	-1.05051749	-1.80157304
C	4.09615486	0.17789607	-0.85283009
H	4.97837298	0.08310300	-0.23151364
H	4.22627814	-0.45627439	-1.72959622
H	4.06211761	1.17466804	-1.27786879
H	-1.63318209	-1.67699260	-2.08863076

Time = 232.50 fs
E(CASSCF/6-31G(d)) = -479.4838261

C	-0.90119797	-0.49388647	-0.29962545
C	0.49224603	-0.45033010	-0.07918674
C	0.94628907	0.14359798	1.14376202
C	1.69189112	-0.66385278	-0.80532137
C	2.80294610	-0.13008673	-0.24301585
C	2.38480587	0.40892416	1.10634118

N	-1.61190574	0.38088842	0.48235413
C	-2.98037478	-0.19801646	0.54701217
H	2.92840574	0.83537879	1.98125580
C	-1.75131460	-0.76492035	-1.50291722
C	-3.18948800	-0.55413233	-0.93358624
H	-3.50955263	0.34096183	-1.45554467
H	-3.94298787	-1.32333155	-1.05091464
H	-2.98598243	-1.07413825	1.17306742
H	-3.69160326	0.47019058	1.00403777
H	-1.47058689	0.07026427	-2.13394149
H	0.51210857	0.13022969	2.11410198
C	-1.30343147	1.59379317	1.23261791
H	-0.62526477	2.27028071	0.74124224
H	-2.22900662	2.13688233	1.45241879
H	-0.83583175	1.43988115	2.19403657
H	1.69910908	-1.05731048	-1.80411318
C	4.09380436	0.17061624	-0.85824528
H	4.97184084	0.07506301	-0.22431790
H	4.22542314	-0.45348156	-1.74363532
H	4.06892813	1.16641710	-1.28168239
H	-1.62366459	-1.66248270	-2.09368175

Time = 233.00 fs
E(CASSCF/6-31G(d)) = -479.4856615

C	-0.90461360	-0.49507320	-0.29677568
C	0.48809667	-0.45430569	-0.07285875
C	0.94749347	0.15021831	1.14743754
C	1.69053259	-0.67145937	-0.80927691
C	2.81061595	-0.13038909	-0.24256197
C	2.39130247	0.41793733	1.10365244
N	-1.61407957	0.37924314	0.48706116
C	-2.98226321	-0.20371261	0.54293693
H	2.91909269	0.85262961	1.96533945
C	-1.74678473	-0.75732395	-1.50159296
C	-3.18581632	-0.55356396	-0.93966799
H	-3.49822679	0.34809159	-1.45210191
H	-3.94227420	-1.31845236	-1.06733708
H	-2.98006462	-1.07581609	1.17274027
H	-3.69360877	0.47142404	0.99708501
H	-1.46425517	0.08204633	-2.12817763
H	0.53218009	0.14143692	2.10430692
C	-1.31318563	1.59429482	1.23920444
H	-0.62905746	2.27094923	0.75325628
H	-2.23573808	2.13955486	1.44668561
H	-0.85048075	1.43534176	2.19473917
H	1.70189528	-1.06413213	-1.80713903

C	4.09162091	0.16326946	-0.86400163
H	4.96470021	0.06693930	-0.21690469
H	4.22522385	-0.45108920	-1.75712642
H	4.07611028	1.15907121	-1.28475917
H	-1.61350609	-1.64855363	-2.09767881

Time = 233.50 fs
E(CASSCF/6-31G(d)) = -479.4868064

C	-0.90714784	-0.49595136	-0.29385355
C	0.48358751	-0.45834511	-0.06639585
C	0.94984968	0.15702075	1.15135987
C	1.68933805	-0.67918520	-0.81366517
C	2.81796863	-0.13052689	-0.24162535
C	2.39695811	0.42668333	1.10082167
N	-1.61678036	0.37757320	0.49197940
C	-2.98392475	-0.20926746	0.53881891
H	2.90982991	0.86873659	1.94693537
C	-1.74232969	-0.74980219	-1.50027504
C	-3.18219083	-0.55279102	-0.94577723
H	-3.48868159	0.35501075	-1.44823963
H	-3.94063656	-1.31421180	-1.08463417
H	-2.97416619	-1.07862019	1.17269276
H	-3.69589101	0.47323357	0.98913142
H	-1.45901363	0.09351905	-2.12332915
H	0.54966016	0.15278801	2.09595183
C	-1.32297583	1.59478811	1.24584106
H	-0.63288242	2.27129422	0.76368125
H	-2.24236674	2.14157472	1.43995793
H	-0.86571594	1.43024643	2.19633646
H	1.70462947	-1.07103463	-1.81068607
C	4.08967654	0.15587175	-0.87008619
H	4.95688999	0.05870566	-0.20966660
H	4.22564824	-0.44922265	-1.76986322
H	4.08360495	1.15278328	-1.28707143
H	-1.60270618	-1.63534691	-2.10069230

Time = 234.00 fs
E(CASSCF/6-31G(d)) = -479.4873098

C	-0.90879042	-0.49650527	-0.29083116
C	0.47880202	-0.46244355	-0.05982523
C	0.95342981	0.16399079	1.15534006
C	1.68834592	-0.68696543	-0.81836583
C	2.82485068	-0.13055397	-0.24026231
C	2.40169854	0.43510969	1.09776239
N	-1.62001150	0.37587483	0.49709356

C	-2.98537031	-0.21465024	0.53468362
H	2.90134833	0.88417356	1.92704043
C	-1.73794883	-0.74235581	-1.49899116
C	-3.17861509	-0.55181253	-0.95190012
H	-3.48099409	0.36176841	-1.44405747
H	-3.93803968	-1.31059008	-1.10277244
H	-2.96846234	-1.08254706	1.17285518
H	-3.69834482	0.47530465	0.98012981
H	-1.45486025	0.10463180	-2.11932055
H	0.56369665	0.16433271	2.09078144
C	-1.33277493	1.59526258	1.25246601
H	-0.63681697	2.27120977	0.77258008
H	-2.24917064	2.14304389	1.43240169
H	-0.88137139	1.42458055	2.19934885
H	1.70724619	-1.07806543	-1.81478107
C	4.08803645	0.14844486	-0.87648166
H	4.94839913	0.05033423	-0.20297164
H	4.22665257	-0.44800965	-1.78167498
H	4.09135593	1.14764228	-1.28859148
H	-1.59127721	-1.62298912	-2.10279669

Time = 234.50 fs
E(CASSCF/6-31G(d)) = -479.4868822

C	-0.90953886	-0.49672258	-0.28770441
C	0.47379423	-0.46658565	-0.05317636
C	0.95824866	0.17107787	1.15922258
C	1.68759358	-0.69473746	-0.82327454
C	2.83113014	-0.13051961	-0.23854417
C	2.40546164	0.44316425	1.09439717
N	-1.62376556	0.37415068	0.50238104
C	-2.98659039	-0.21982240	0.53055845
H	2.89439696	0.89932281	1.90677424
C	-1.73364919	-0.73500308	-1.49775576
C	-3.17509304	-0.55063389	-0.95800744
H	-3.47526438	0.36842720	-1.43970774
H	-3.93452842	-1.30766247	-1.12168712
H	-2.96318074	-1.08760944	1.17316545
H	-3.70098628	0.47737744	0.97012050
H	-1.45176933	0.11538797	-2.11619508
H	0.57376561	0.17614879	2.09004883
C	-1.34253235	1.59570478	1.25900159
H	-0.64084294	2.27067637	0.77999276
H	-2.25641958	2.14419271	1.42424257
H	-0.89722369	1.41839012	2.20433873
H	1.70969897	-1.08511345	-1.81925371
C	4.08676261	0.14103667	-0.88315667

H	4.93925582	0.04180443	-0.19719941
H	4.22813594	-0.44753065	-1.79232587
H	4.09925890	1.14358858	-1.28922376
H	-1.57923929	-1.61175600	-2.10411643

Time = 235.00 fs
E(CASSCF/6-31G(d)) = -479.4877981

C	-0.90941402	-0.49657966	-0.28442490
C	0.46868396	-0.47078058	-0.04646222
C	0.96433941	0.17829833	1.16290066
C	1.68708844	-0.70246054	-0.82827954
C	2.83669121	-0.13047191	-0.23651704
C	2.40819861	0.45079858	1.09061866
N	-1.62804467	0.37238901	0.50783813
C	-2.98762301	-0.22479559	0.52646965
H	2.88972265	0.91498540	1.88719417
C	-1.72940733	-0.72771609	-1.49660622
C	-3.17162429	-0.54925356	-0.96411318
H	-3.47144285	0.37498896	-1.43522284
H	-3.92998154	-1.30527306	-1.14134372
H	-2.95845805	-1.09354321	1.17339621
H	-3.70360649	0.47909190	0.95902650
H	-1.44971329	0.12567723	-2.11372984
H	0.57947069	0.18830486	2.09465191
C	-1.35225059	1.59610060	1.26542861
H	-0.64517519	2.26945965	0.78613979
H	-2.26440171	2.14505484	1.41558137
H	-0.91332436	1.41171589	2.21145081
H	1.71193368	-1.09237127	-1.82428289
C	4.08589003	0.13364950	-0.89010464
H	4.92958740	0.03305875	-0.19256151
H	4.23009063	-0.44796581	-1.80186798
H	4.10732663	1.14064876	-1.28901548
H	-1.56662620	-1.60151498	-2.10465678

Time = 235.50 fs
E(CASSCF/6-31G(d)) = -479.4887069

C	-0.90844573	-0.49605687	-0.28097811
C	0.46357450	-0.47501525	-0.03971587
C	0.97165071	0.18561563	1.16633410
C	1.68684210	-0.71007518	-0.83327424
C	2.84142503	-0.13045483	-0.23424986
C	2.40987375	0.45795474	1.08636091
N	-1.63282821	0.37058879	0.51343946
C	-2.98848152	-0.22955942	0.52245263

H	2.88795247	0.93168554	1.86927158
C	-1.72521975	-0.72050678	-1.49556831
C	-3.16821811	-0.54767846	-0.97019962
H	-3.46951401	0.38144096	-1.43069542
H	-3.92437497	-1.30337523	-1.16162414
H	-2.95448591	-1.10012002	1.17334973
H	-3.70613555	0.48017292	0.94690142
H	-1.44864269	0.13542804	-2.11184145
H	0.58090219	0.20084684	2.10450383
C	-1.36188568	1.59643188	1.27170359
H	-0.64989824	2.26743723	0.79118204
H	-2.27332193	2.14575605	1.40658758
H	-0.92965685	1.40468944	2.22077309
H	1.71390919	-1.09984233	-1.82981566
C	4.08544981	0.12633093	-0.89728605
H	4.91959074	0.02406901	-0.18927485
H	4.23245070	-0.44944206	-1.81026637
H	4.11550110	1.13871128	-1.28795023
H	-1.55351500	-1.59230225	-2.10446193

Time = 236.00 fs
E(CASSCF/6-31G(d)) = -479.4903496

C	-0.90667867	-0.49513376	-0.27734917
C	0.45856569	-0.47928111	-0.03295893
C	0.98009513	0.19300171	1.16955825
C	1.68685858	-0.71753858	-0.83816898
C	2.84525033	-0.13050908	-0.23181410
C	2.41048349	0.46460274	1.08158359
N	-1.63809426	0.36874626	0.51916666
C	-2.98918113	-0.23411830	0.51853420
H	2.88949409	0.94981195	1.85363555
C	-1.72107469	-0.71337903	-1.49466309
C	-3.16487875	-0.54591447	-0.97625921
H	-3.46942349	0.38775747	-1.42620585
H	-3.91769026	-1.30191818	-1.18242035
H	-2.95144116	-1.10709261	1.17281820
H	-3.70854310	0.48039549	0.93382433
H	-1.44848191	0.14459779	-2.11045317
H	0.57855064	0.21382050	2.11870473
C	-1.37141115	1.59667908	1.27780771
H	-0.65508297	2.26450408	0.79530436
H	-2.28332304	2.14639882	1.39740520
H	-0.94629808	1.39748416	2.23221427
H	1.71559636	-1.10751895	-1.83576016
C	4.08545721	0.11912145	-0.90466658
H	4.90950951	0.01479038	-0.18748225

H	4.23514914	-0.45207778	-1.81754935
H	4.12373771	1.13759940	-1.28602259
H	-1.53998097	-1.58410295	-2.10356573

Time = 236.50 fs
E(CASSCF/6-31G(d)) = -479.4924336

C	-0.90417161	-0.49378983	-0.27352783
C	0.45375979	-0.48356834	-0.02620982
C	0.98954845	0.20042784	1.17266022
C	1.68713601	-0.72481439	-0.84288483
C	2.84810621	-0.13067213	-0.22928657
C	2.41005847	0.47073793	1.07628655
N	-1.64381460	0.36685772	0.52500124
C	-2.98973719	-0.23848338	0.51473618
H	2.89446956	0.96950590	1.84048454
C	-1.71695843	-0.70633861	-1.49390761
C	-3.16160945	-0.54396767	-0.98228608
H	-3.47108354	0.39388905	-1.42182606
H	-3.90992113	-1.30085323	-1.20361064
H	-2.94948648	-1.11421108	1.17159980
H	-3.71083946	0.47959108	0.91991341
H	-1.44913468	0.15316033	-2.10950012
H	0.57321038	0.22724782	2.13576070
C	-1.38080802	1.59682116	1.28373756
H	-0.66077044	2.26058407	0.79869628
H	-2.29445579	2.14704884	1.38816120
H	-0.96338532	1.39031762	2.24551073
H	1.71697539	-1.11537196	-1.84197784
C	4.08591286	0.11206288	-0.91220775
H	4.89963770	0.00517290	-0.18725779
H	4.23811567	-0.45597664	-1.82378868
H	4.13199474	1.13708970	-1.28324343
H	-1.52611516	-1.57686596	-2.10198870

Time = 237.00 fs
E(CASSCF/6-31G(d)) = -479.4943555

C	-0.90099625	-0.49200463	-0.26950868
C	0.44926104	-0.48786554	-0.01948320
C	0.99985928	0.20786447	1.17575239
C	1.68766736	-0.73187211	-0.84735340
C	2.84995173	-0.13097844	-0.22674915
C	2.40866643	0.47638500	1.07051173
N	-1.64995464	0.36491942	0.53092505
C	-2.99016535	-0.24267088	0.51107434
H	2.90269154	0.99061421	1.82956279

C	-1.71285559	-0.69939346	-1.49331475
C	-3.15841274	-0.54184430	-0.98827584
H	-3.47437707	0.39976614	-1.41762201
H	-3.90107723	-1.30013642	-1.22506151
H	-2.94876614	-1.12124449	1.16951532
H	-3.71307706	0.47765378	0.90532438
H	-1.45048303	0.16111109	-2.10893424
H	0.56585067	0.24112671	2.15389989
C	-1.39006734	1.59683661	1.28950315
H	-0.66696851	2.25563543	0.80154342
H	-2.30666870	2.14772604	1.37896626
H	-0.98109433	1.38343992	2.26025791
H	1.71803412	-1.12335335	-1.84828987
C	4.08680363	0.10519635	-0.91986709
H	4.89030638	-0.00483683	-0.18860913
H	4.24127822	-0.46122369	-1.82909348
H	4.14023350	1.13692924	-1.27964575
H	-1.51202148	-1.57050955	-2.09974260

Time = 237.50 fs
E(CASSCF/6-31G(d)) = -479.4954537

C	-0.89723557	-0.48975881	-0.26529139
C	0.44517438	-0.49215961	-0.01278945
C	1.01085882	0.21528067	1.17894821
C	1.68844111	-0.73868685	-0.85151627
C	2.85076560	-0.13145946	-0.22428788
C	2.40641048	0.48159677	1.06433642
N	-1.65647415	0.36292814	0.53692084
C	-2.99048185	-0.24670071	0.50755741
H	2.91369188	1.01270617	1.82023065
C	-1.70874949	-0.69255301	-1.49289272
C	-3.15529013	-0.53955080	-0.99422535
H	-3.47916044	0.40530327	-1.41365471
H	-3.89118574	-1.29973110	-1.24663116
H	-2.94940168	-1.12799964	1.16642148
H	-3.71535011	0.47454209	0.89024603
H	-1.45238996	0.16846745	-2.10872614
H	0.55749156	0.25543770	2.17136246
C	-1.39919234	1.59670415	1.29512422
H	-0.67364738	2.24965625	0.80402012
H	-2.31980892	2.14840500	1.36991688
H	-0.99961287	1.37711725	2.27595943
H	1.71876826	-1.13140030	-1.85448592
C	4.08810396	0.09856032	-0.92759870
H	4.88186690	-0.01529477	-0.19148343
H	4.24456218	-0.46787880	-1.83359913

H	4.14841941	1.13685351	-1.27528916
H	-1.49781477	-1.56492877	-2.09683412

Time = 238.00 fs
E(CASSCF/6-31G(d)) = -479.4952251

C	-0.89298277	-0.48703406	-0.26088058
C	0.44160404	-0.49643587	-0.00613435
C	1.02236791	0.22264348	1.18234543
C	1.68944193	-0.74523851	-0.85532375
C	2.85054493	-0.13214361	-0.22199163
C	2.40342363	0.48644822	1.05786018
N	-1.66332751	0.36088106	0.54297229
C	-2.99070386	-0.25059518	0.50418651
H	2.92679902	1.03514528	1.81161050
C	-1.70462255	-0.68582757	-1.49264464
C	-3.15224173	-0.53709386	-1.00013241
H	-3.48526629	0.41040412	-1.40998116
H	-3.88029078	-1.29960822	-1.26817162
H	-2.95148778	-1.13433196	1.16221880
H	-3.71778815	0.47027612	0.87489298
H	-1.45470212	0.17526707	-2.10886256
H	0.54910615	0.27015280	2.18660316
C	-1.40819909	1.59640345	1.30062603
H	-0.68074020	2.24268481	0.80628372
H	-2.33363580	2.14901993	1.36109776
H	-1.01911485	1.37161333	2.29207781
H	1.71918050	-1.13944155	-1.86033956
C	4.08977812	0.09218830	-0.93535409
H	4.87467280	-0.02625746	-0.19577695
H	4.24789028	-0.47597071	-1.83745562
H	4.15652312	1.13660489	-1.27026280
H	-1.48361780	-1.56000134	-2.09326808

Time = 238.50 fs
E(CASSCF/6-31G(d)) = -479.4934431

C	-0.88833776	-0.48381439	-0.25628571
C	0.43865168	-0.50067812	0.00048034
C	1.03420257	0.22991744	1.18601865
C	1.69065126	-0.75151120	-0.85873378
C	2.84930413	-0.13305622	-0.21995027
C	2.39986070	0.49102756	1.05119002
N	-1.67046479	0.35877635	0.54906486
C	-2.99085012	-0.25437750	0.50095544
H	2.94123347	1.05719075	1.80274457
C	-1.70045686	-0.67922734	-1.49256874

C	-3.14926635	-0.53448091	-1.00599551
H	-3.49250935	0.41496954	-1.40665746
H	-3.86845459	-1.29974794	-1.28953241
H	-2.95509045	-1.14015667	1.15685891
H	-3.72055058	0.46493205	0.85949839
H	-1.45725378	0.18156624	-2.10934825
H	0.54155867	0.28524405	2.19839030
C	-1.41711586	1.59591549	1.30603539
H	-0.68814455	2.23480001	0.80847062
H	-2.34784282	2.14947749	1.35258545
H	-1.03973727	1.36717221	2.30808420
H	1.71928059	-1.14740309	-1.86562065
C	4.09178271	0.08610696	-0.94308310
H	4.86905750	-0.03777936	-0.20134884
H	4.25118117	-0.48549048	-1.84081297
H	4.16452130	1.13594864	-1.26468622
H	-1.46955751	-1.55559915	-2.08905409

Time = 239.00 fs
E(CASSCF/6-31G(d)) = -479.4901975

C	-0.88340548	-0.48008590	-0.25152048
C	0.43641438	-0.50486900	0.00705669
C	1.04617661	0.23706441	1.19001710
C	1.69204772	-0.75749252	-0.86171036
C	2.84707303	-0.13421921	-0.21825272
C	2.39589091	0.49542772	1.04442749
N	-1.67783221	0.35661288	0.55518562
C	-2.99094160	-0.25807053	0.49785101
H	2.95620012	1.07809531	1.79273383
C	-1.69623457	-0.67276137	-1.49265829
C	-3.14636104	-0.53172036	-1.01181355
H	-3.50069036	0.41890417	-1.40373908
H	-3.85575488	-1.30013847	-1.31056281
H	-2.96024487	-1.14545064	1.15034534
H	-3.72381335	0.45863118	0.84430303
H	-1.45987069	0.18743400	-2.11019981
H	0.53557032	0.30069077	2.20583894
C	-1.42598120	1.59522281	1.31137718
H	-0.69572792	2.22611558	0.81069479
H	-2.36208653	2.14967089	1.34444888
H	-1.06156569	1.36400262	2.32349790
H	1.71908429	-1.15521531	-1.87011315
C	4.09406976	0.08033411	-0.95073491
H	4.86531321	-0.04990979	-0.20803779
H	4.25434868	-0.49638547	-1.84380843
H	4.17239815	1.13468788	-1.25870822

H	-1.45576112	-1.55159475	-2.08420931

Time = 239.50 fs
E(CASSCF/6-31G(d)) = -479.4858131

C	-0.87829169	-0.47583762	-0.24660232
C	0.43498202	-0.50899046	0.01359962
C	1.05810473	0.24404370	1.19436637
C	1.69360760	-0.76317279	-0.86422244
C	2.84389526	-0.13565085	-0.21698475
C	2.39169088	0.49973906	1.03765973
N	-1.68537318	0.35439053	0.56132379
C	-2.99100243	-0.26169530	0.49485405
H	2.97095847	1.09718174	1.78082555
C	-1.69193846	-0.66643662	-1.49290221
C	-3.14352141	-0.52882210	-1.01758564
H	-3.50960248	0.42212436	-1.40128382
H	-3.84228446	-1.30077579	-1.33111436
H	-2.96695589	-1.15025512	1.14273369
H	-3.72775706	0.45152708	0.82954589
H	-1.46237497	0.19294771	-2.11144490
H	0.53170299	0.31648408	2.20840127
C	-1.43484109	1.59430979	1.31667190
H	-0.70333522	2.21677533	0.81304792
H	-2.37601644	2.14949601	1.33675117
H	-1.08462376	1.36226712	2.33791720
H	1.71861279	-1.16281844	-1.87362622
C	4.09658959	0.07487717	-0.95825894
H	4.86366889	-0.06268848	-0.21568014
H	4.25730152	-0.50855505	-1.84655423
H	4.18014540	1.13267456	-1.25250406
H	-1.44235173	-1.54787139	-2.07876397

Time = 240.00 fs
E(CASSCF/6-31G(d)) = -479.4807562

C	-0.87310005	-0.47106171	-0.24155153
C	0.43443467	-0.51302394	0.02011611
C	1.06980542	0.25081251	1.19907048
C	1.69530503	-0.76854456	-0.86624274
C	2.83982658	-0.13736531	-0.21622688
C	2.38743908	0.50404445	1.03095449
N	-1.69302906	0.35210990	0.56747075
C	-2.99106074	-0.26526979	0.49194052
H	2.98486906	1.11389197	1.76645895
C	-1.68755232	-0.66025705	-1.49328547
C	-3.14074146	-0.52579785	-1.02331095

H	-3.51903689	0.42456482	-1.39935254
H	-3.82814853	-1.30166261	-1.35104314
H	-2.97519795	-1.15467129	1.13412721
H	-3.73254989	0.44378909	0.81545323
H	-1.46458929	0.19818569	-2.11311608
H	0.53035053	0.33263081	2.20584779
C	-1.44374560	1.59316291	1.32193380
H	-0.71079890	2.20694315	0.81560249
H	-2.38930372	2.14886561	1.32954806
H	-1.10887080	1.36207375	2.35103982
H	1.71789158	-1.17016756	-1.87600695
C	4.09929372	0.06973199	-0.96560555
H	4.86427219	-0.07614151	-0.22412720
H	4.25994291	-0.52184822	-1.84912961
H	4.18776298	1.12981850	-1.24627114
H	-1.42944416	-1.54432963	-2.07276342

Time = 240.50 fs
E(CASSCF/6-31G(d)) = -479.4755112

C	-0.86792864	-0.46575384	-0.23639044
C	0.43483980	-0.51695068	0.02661439
C	1.08110437	0.25732640	1.20411327
C	1.69711210	-0.77360230	-0.86774715
C	2.83493324	-0.13937204	-0.21605240
C	2.38331155	0.50841605	1.02435797
N	-1.70074000	0.34977221	0.57362009
C	-2.99114954	-0.26880785	0.48908294
H	2.99742029	1.12781342	1.74927890
C	-1.68306131	-0.65422265	-1.49378979
C	-3.13801364	-0.52266142	-1.02898859
H	-3.52878817	0.42618368	-1.39800940
H	-3.81346181	-1.30280655	-1.37021155
H	-2.98491686	-1.15885291	1.12466970
H	-3.73833014	0.43558498	0.80222816
H	-1.46634161	0.20321976	-2.11524575
H	0.53173073	0.34915645	2.19825284
C	-1.45274542	1.59177116	1.32716962
H	-0.71795085	2.19679269	0.81841668
H	-2.40166615	2.14772138	1.32288569
H	-1.13420381	1.36347234	2.36267572
H	1.71694920	-1.17723604	-1.87714729
C	4.10213704	0.06488202	-0.97272661
H	4.86717489	-0.09027658	-0.23326075
H	4.26217140	-0.53606435	-1.85157697
H	4.19525890	1.12609156	-1.24022309
H	-1.41714107	-1.54089304	-2.06626947

Time = 241.00 fs
E(CASSCF/6-31G(d)) = -479.4704862

C	-0.86286610	-0.45991305	-0.23114252
C	0.43624871	-0.52075248	0.03310283
C	1.09184207	0.26354018	1.20945915
C	1.69899916	-0.77834214	-0.86871418
C	2.82929029	-0.14167543	-0.21652532
C	2.37947538	0.51291288	1.01789488
N	-1.70844643	0.34737897	0.57976763
C	-2.99130713	-0.27231855	0.48625203
H	3.00824381	1.13869148	1.72912822
C	-1.67845238	-0.64832882	-1.49439456
C	-3.13532887	-0.51942877	-1.03461754
H	-3.53865879	0.42696618	-1.39732146
H	-3.79834481	-1.30421747	-1.38848964
H	-2.99603058	-1.16299358	1.11453443
H	-3.74518845	0.42706427	0.79004186
H	-1.46747044	0.20810764	-2.11785929
H	0.53586967	0.36611038	2.18598789
C	-1.46188846	1.59012645	1.33237795
H	-0.72463616	2.18649434	0.82154069
H	-2.41288702	2.14604132	1.31679693
H	-1.16046330	1.36645300	2.37275122
H	1.71581521	-1.18401901	-1.87699136
C	4.10507978	0.06029838	-0.97957598
H	4.87232706	-0.10507892	-0.24300114
H	4.26388410	-0.55095778	-1.85390435
H	4.20264835	1.12153046	-1.23458330
H	-1.40553032	-1.53751015	-2.05935945

Time = 241.50 fs
E(CASSCF/6-31G(d)) = -479.4659439

C	-0.85798900	-0.45354268	-0.22583130
C	0.43869497	-0.52441101	0.03958960
C	1.10187839	0.26940797	1.21505120
C	1.70093425	-0.78276206	-0.86912493
C	2.82298064	-0.14427392	-0.21769890
C	2.37608526	0.51757927	1.01156913
N	-1.71608932	0.34493190	0.58591125
C	-2.99157773	-0.27580614	0.48341826
H	3.01712111	1.14643410	1.70603865
C	-1.67371440	-0.64256600	-1.49507759
C	-3.13267671	-0.51611816	-1.04019664
H	-3.54846416	0.42692651	-1.39735792

H	-3.78292119	-1.30590568	-1.40575640
H	-3.00843263	-1.16731085	1.10391201
H	-3.75315460	0.41834562	0.77902733
H	-1.46782799	0.21288701	-2.12097132
H	0.54258755	0.38357174	2.16973152
C	-1.47121688	1.58822413	1.33754888
H	-0.73072575	2.17620465	0.82502427
H	-2.42282888	2.14384600	1.31129825
H	-1.18744217	1.37094901	2.38130994
H	1.71451895	-1.19053380	-1.87553590
C	4.10808797	0.05594047	-0.98610993
H	4.87957670	-0.12050817	-0.25331215
H	4.26497886	-0.56624537	-1.85609143
H	4.20995361	1.11623160	-1.22957709
H	-1.39468230	-1.53415704	-2.05212540

Time = 242.00 fs
E(CASSCF/6-31G(d)) = -479.4619709

C	-0.85335897	-0.44665011	-0.22047944
C	0.44219135	-0.52790834	0.04608176
C	1.11110302	0.27488408	1.22080980
C	1.70288389	-0.78686178	-0.86896326
C	2.81609347	-0.14715993	-0.21961463
C	2.37327522	0.52244298	1.00536512
N	-1.72361151	0.34243225	0.59205071
C	-2.99201108	-0.27927085	0.48055338
H	3.02399453	1.15111612	1.68021849
C	-1.66883831	-0.63691967	-1.49581596
C	-3.13004546	-0.51274986	-1.04572466
H	-3.55803621	0.42610784	-1.39818894
H	-3.76731428	-1.30787996	-1.42190047
H	-3.02199375	-1.17202538	1.09299432
H	-3.76218954	0.40950901	0.76927622
H	-1.46728391	0.21757104	-2.12458064
H	0.55147576	0.40165852	2.15048024
C	-1.48076448	1.58606355	1.34266450
H	-0.73612834	2.16605544	0.82892364
H	-2.43144166	2.14120014	1.30638608
H	-1.21489727	1.37684129	2.38850553
H	1.71308661	-1.19682015	-1.87282905
C	4.11113371	0.05175755	-0.99228785
H	4.88867877	-0.13649693	-0.26419579
H	4.26535862	-0.58161787	-1.85809939
H	4.21720216	1.11034300	-1.22542355
H	-1.38464851	-1.53083591	-2.04467067

Time = 242.50 fs
E(CASSCF/6-31G(d)) = -479.4585032

C	-0.84902249	-0.43924611	-0.21510826
C	0.44673025	-0.53122649	0.05258555
C	1.11944372	0.27992308	1.22662988
C	1.70481318	-0.79064279	-0.86821587
C	2.80872250	-0.15031958	-0.22230191
C	2.37115104	0.52751286	0.99925014
N	-1.73095768	0.33988006	0.59818706
C	-2.99266216	-0.28271013	0.47763164
H	3.02897645	1.15298378	1.65204431
C	-1.66381726	-0.63137099	-1.49658648
C	-3.12742240	-0.50934585	-1.05120022
H	-3.56722643	0.42458041	-1.39988388
H	-3.75164440	-1.31014549	-1.43682087
H	-3.03656316	-1.17733805	1.08195964
H	-3.77218271	0.40059373	0.76083849
H	-1.46572609	0.22214672	-2.12866823
H	0.56188236	0.42054085	2.12955150
C	-1.49055474	1.58364823	1.34769996
H	-0.74079817	2.15614594	0.83330630
H	-2.43876566	2.13821167	1.30203352
H	-1.24256229	1.38396514	2.39458935
H	1.71154044	-1.20293895	-1.86896443
C	4.11419450	0.04769102	-0.99807285
H	4.89931147	-0.15295154	-0.27568259
H	4.26493499	-0.59675444	-1.85988426
H	4.22442457	1.10405253	-1.22232839
H	-1.37546071	-1.52757266	-2.03710610

Time = 243.00 fs
E(CASSCF/6-31G(d)) = -479.4559200

C	-0.84502199	-0.43134232	-0.20973624
C	0.45229466	-0.53434341	0.05910839
C	1.12690173	0.28448466	1.23237473
C	1.70670145	-0.79409818	-0.86686368
C	2.80094786	-0.15374283	-0.22579069
C	2.36975215	0.53277166	0.99318482
N	-1.73806928	0.33726779	0.60431976
C	-2.99359440	-0.28611976	0.47462944
H	3.03236626	1.15245676	1.62206561
C	-1.65864706	-0.62589888	-1.49736610
C	-3.12479389	-0.50592874	-1.05662236
H	-3.57590885	0.42243650	-1.40250910
H	-3.73602535	-1.31270054	-1.45042024

H	-3.05195032	-1.18339476	1.07096131
H	-3.78295340	0.39160164	0.75372466
H	-1.46305880	0.22657290	-2.13319492
H	0.57290943	0.44046993	2.10855541
C	-1.50059859	1.58098870	1.35262662
H	-0.74474056	2.14652478	0.83825577
H	-2.44493409	2.13501767	1.29818565
H	-1.27016501	1.39210889	2.39988245
H	1.70989560	-1.20898293	-1.86407184
C	4.11725141	0.04367686	-1.00343258
H	4.91110133	-0.16975491	-0.28781549
H	4.26363274	-0.61133755	-1.86141078
H	4.23165426	1.09757313	-1.22047443
H	-1.36713256	-1.52440767	-2.02953890

Time = 243.50 fs
E(CASSCF/6-31G(d)) = -479.4614347

C	-0.84141270	-0.42292300	-0.20437212
C	0.45880321	-0.53724852	0.06558701
C	1.13323650	0.28845993	1.23788943
C	1.70838946	-0.79726560	-0.86497061
C	2.79302488	-0.15732024	-0.22997394
C	2.36932036	0.53817956	0.98710083
N	-1.74483684	0.33461164	0.61036624
C	-2.99483101	-0.28947757	0.47155456
H	3.03459853	1.15019758	1.59108902
C	-1.65337065	-0.62054610	-1.49812922
C	-3.12217093	-0.50255166	-1.06193967
H	-3.58388747	0.41981306	-1.40610521
H	-3.72066703	-1.31553613	-1.46260926
H	-3.06809062	-1.19029850	1.06016790
H	-3.79423210	0.38257427	0.74793402
H	-1.45914502	0.23075209	-2.13792390
H	0.58345652	0.46144467	2.08942499
C	-1.51082124	1.57807098	1.35738436
H	-0.74795381	2.13739204	0.84379750
H	-2.45010822	2.13188390	1.29479168
H	-1.29722000	1.40117036	2.40480783
H	1.70820186	-1.21495255	-1.85834260
C	4.12026420	0.03968036	-1.00830505
H	4.92350659	-0.18663599	-0.30052868
H	4.26139364	-0.62496217	-1.86263826
H	4.23885156	1.09115804	-1.22001310
H	-1.35973982	-1.52144722	-2.02220627

Time = 244.00 fs

E(CASSCF/6-31G(d)) = -479.4602468

C	-0.83816521	-0.41396863	-0.19899767
C	0.46622687	-0.53993946	0.07207536
C	1.13858950	0.29184273	1.24306113
C	1.70986763	-0.80016960	-0.86251597
C	2.78498773	-0.16105352	-0.23488267
C	2.36980083	0.54371600	0.98088960
N	-1.75126989	0.33188894	0.61638226
C	-2.99644351	-0.29280786	0.46837177
H	3.03637902	1.14696037	1.55972133
C	-1.64794738	-0.61524188	-1.49886172
C	-3.11951902	-0.49921304	-1.06719206
H	-3.59114411	0.41681593	-1.41074907
H	-3.70555606	-1.31865604	-1.47340565
H	-3.08485231	-1.19811173	1.04955956
H	-3.80582767	0.37340773	0.74337020
H	-1.45392893	0.23464356	-2.14284868
H	0.59245662	0.48400031	2.07380049
C	-1.52128588	1.57489552	1.36197261
H	-0.75057986	2.12865753	0.85008693
H	-2.45460224	2.12898264	1.29172343
H	-1.32367855	1.41094094	2.40978824
H	1.70644017	-1.22100424	-1.85190875
C	4.12324127	0.03561091	-1.01270838
H	4.93620664	-0.20357377	-0.31394012
H	4.25816545	-0.63746844	-1.86358988
H	4.24609815	1.08494615	-1.22105776
H	-1.35319933	-1.51872456	-2.01513915

Time = 244.50 fs
E(CASSCF/6-31G(d)) = -479.4588619

C	-0.83525566	-0.40452065	-0.19363104
C	0.47446841	-0.54239382	0.07857658
C	1.14305029	0.29459948	1.24775937
C	1.71109791	-0.80282262	-0.85950632
C	2.77694491	-0.16490369	-0.24049635
C	2.37112684	0.54930501	0.97447557
N	-1.75733544	0.32909755	0.62237869
C	-2.99848960	-0.29611484	0.46507398
H	3.03856181	1.14365801	1.52887725
C	-1.64237714	-0.60995725	-1.49954507
C	-3.11682357	-0.49593616	-1.07237939
H	-3.59761706	0.41357426	-1.41648327
H	-3.69078324	-1.32203749	-1.48275196
H	-3.10198940	-1.20677312	1.03914143

H	-3.81745052	0.36406327	0.73996104
H	-1.44739767	0.23816669	-2.14791086
H	0.59889396	0.50850394	2.06318896
C	-1.53197123	1.57148255	1.36635909
H	-0.75277129	2.12028208	0.85724223
H	-2.45873138	2.12652782	1.28885903
H	-1.34932415	1.42116030	2.41518877
H	1.70459681	-1.22724675	-1.84494361
C	4.12616890	0.03141378	-1.01663041
H	4.94880186	-0.22041682	-0.32804572
H	4.25392775	-0.64863634	-1.86431259
H	4.25341158	1.07907690	-1.22369007
H	-1.34747451	-1.51629272	-2.00841409

Time = 245.00 fs
E(CASSCF/6-31G(d)) = -479.4577693

C	-0.83264360	-0.39462363	-0.18828889
C	0.48341218	-0.54458990	0.08509278
C	1.14670021	0.29671025	1.25190171
C	1.71203412	-0.80524596	-0.85596066
C	2.76901409	-0.16882133	-0.24677755
C	2.37320650	0.55485852	0.96778124
N	-1.76300874	0.32623523	0.62836715
C	-3.00102029	-0.29940778	0.46165872
H	3.04210505	1.14128088	1.49948824
C	-1.63666125	-0.60466563	-1.50016210
C	-3.11407059	-0.49274296	-1.07750156
H	-3.60326140	0.41021483	-1.42333756
H	-3.67642958	-1.32565219	-1.49060891
H	-3.11924883	-1.21614893	1.02887016
H	-3.82881484	0.35451327	0.73761885
H	-1.43955816	0.24123989	-2.15304483
H	0.60210707	0.53522162	2.05850001
C	-1.54284866	1.56785331	1.37051919
H	-0.75470564	2.11221279	0.86537528
H	-2.46282915	2.12473395	1.28606159
H	-1.37398399	1.43158153	2.42130947
H	1.70264843	-1.23378216	-1.83760545
C	4.12903264	0.02704361	-1.02006698
H	4.96092395	-0.23701564	-0.34280184
H	4.24867930	-0.65829963	-1.86489133
H	4.26079604	1.07362499	-1.22794744
H	-1.34251782	-1.51419347	-2.00208936

Time = 245.50 fs
E(CASSCF/6-31G(d)) = -479.4575599

C	-0.83027655	-0.38432577	-0.18298685
C	0.49292831	-0.54650625	0.09162560
C	1.14959974	0.29817832	1.25547186
C	1.71262445	-0.80746893	-0.85191008
C	2.76131953	-0.17274837	-0.25367504
C	2.37593023	0.56028533	0.96073746
N	-1.76827300	0.32329895	0.63435951
C	-3.00407811	-0.30270016	0.45812698
H	3.04795439	1.14078779	1.47240410
C	-1.63080213	-0.59934405	-1.50069713
C	-3.11124703	-0.48965317	-1.08255870
H	-3.60804596	0.40685408	-1.43132596
H	-3.66256610	-1.32946641	-1.49695518
H	-3.13637027	-1.22604433	1.01866412
H	-3.83965581	0.34475089	0.73624664
H	-1.43043668	0.24378772	-2.15818385
H	0.60196162	0.56421109	2.05982453
C	-1.55388531	1.56403047	1.37443932
H	-0.75657208	2.10439046	0.87458236
H	-2.46721805	2.12379638	1.28318617
H	-1.39753567	1.44197557	2.42834729
H	1.70056254	-1.24070123	-1.83002432
C	4.13181651	0.02246660	-1.02302227
H	4.97225228	-0.25323036	-0.35812048
H	4.24243741	-0.66635096	-1.86544841
H	4.26824024	1.06858878	-1.23382122
H	-1.33827370	-1.51244943	-1.99620013

Time = 246.00 fs
E(CASSCF/6-31G(d)) = -479.4585405

C	-0.82809395	-0.37367867	-0.17773983
C	0.50287666	-0.54812180	0.09817651
C	1.15178401	0.29903692	1.25852159
C	1.71281325	-0.80952825	-0.84739733
C	2.75398995	-0.17661957	-0.26112612
C	2.37918540	0.56550364	0.95329431
N	-1.77311948	0.32028458	0.64036745
C	-3.00769519	-0.30600825	0.45448204
H	3.05688232	1.14296412	1.44827497
C	-1.62480310	-0.59397420	-1.50113598
C	-3.10834113	-0.48668363	-1.08755126
H	-3.61194994	0.40359110	-1.44044438
H	-3.64925427	-1.33344205	-1.50178847
H	-3.15309037	-1.23622268	1.00841709
H	-3.84974552	0.33479741	0.73574381

H	-1.42007796	0.24574777	-2.16326542
H	0.59888791	0.59524101	2.06641412
C	-1.56504770	1.56003876	1.37811907
H	-0.75855796	2.09675784	0.88493524
H	-2.47217796	2.12387121	1.28008873
H	-1.41990947	1.45213389	2.43636963
H	1.69829739	-1.24807847	-1.82229122
C	4.13450249	0.01766174	-1.02550897
H	4.98252608	-0.26893776	-0.37386746
H	4.23523674	-0.67274398	-1.86613982
H	4.27571616	1.06388752	-1.24125619
H	-1.33468079	-1.51105890	-1.99075627

Time = 246.50 fs
E(CASSCF/6-31G(d)) = -479.4605399

C	-0.82603053	-0.36273706	-0.17256270
C	0.51310998	-0.54941636	0.10474689
C	1.15327003	0.29934945	1.26115480
C	1.71254231	-0.81146766	-0.84247618
C	2.74715683	-0.18036386	-0.26905886
C	2.38287282	0.57045391	0.94543058
N	-1.77754750	0.31718696	0.64640221
C	-3.01189154	-0.30934981	0.45072813
H	3.06931932	1.14828151	1.42744739
C	-1.61866806	-0.58854322	-1.50146573
C	-3.10534277	-0.48384763	-1.09248001
H	-3.61495855	0.40050109	-1.45066877
H	-3.63654545	-1.33753779	-1.50512658
H	-3.16914926	-1.24642890	0.99801208
H	-3.85890640	0.32470536	0.73601030
H	-1.40854380	0.24707479	-2.16823361
H	0.59376342	0.62778223	2.07687361
C	-1.57630525	1.55590584	1.38157209
H	-0.76083477	2.08926823	0.89647306
H	-2.47791743	2.12505820	1.27663589
H	-1.44108579	1.46186900	2.44530619
H	1.69580333	-1.25596911	-1.81445354
C	4.13707011	0.01262072	-1.02754825
H	4.99155342	-0.28403715	-0.38986440
H	4.22712762	-0.67749110	-1.86714783
H	4.28317910	1.05936792	-1.25015194
H	-1.33167370	-1.50999171	-1.98574058

Time = 247.00 fs
E(CASSCF/6-31G(d)) = -479.4631257

C	-0.82401937	-0.35155752	-0.16747057
C	0.52347750	-0.55037134	0.11133829
C	1.15407254	0.29920378	1.26350116
C	1.71175291	-0.81333646	-0.83720958
C	2.74095150	-0.18390641	-0.27739459
C	2.38691856	0.57510754	0.93715888
N	-1.78156403	0.31399946	0.65247440
C	-3.01667360	-0.31274186	0.44686937
H	3.08523577	1.15680596	1.40990987
C	-1.61240140	-0.58304411	-1.50167463
C	-3.10224373	-0.48115438	-1.09734627
H	-3.61706154	0.39763122	-1.46195421
H	-3.62448150	-1.34171174	-1.50700830
H	-3.18429879	-1.25641319	0.98733549
H	-3.86702002	0.31456037	0.73695132
H	-1.39591079	0.24774523	-2.17304369
H	0.58769732	0.66107182	2.08947738
C	-1.58763343	1.55166296	1.38482486
H	-0.76354722	2.08188965	0.90919824
H	-2.48455002	2.12738599	1.27271257
H	-1.46109225	1.47101257	2.45495764
H	1.69302283	-1.26440814	-1.80651321
C	4.13949640	0.00734709	-1.02916957
H	4.99921571	-0.29845682	-0.40589310
H	4.21817320	-0.68065887	-1.86867182
H	4.29057036	1.05481952	-1.26036639
H	-1.32918392	-1.50919083	-1.98111049

Time = 247.50 fs
E(CASSCF/6-31G(d)) = -479.4656977

C	-0.82199455	-0.34019801	-0.16247885
C	0.53382769	-0.55096999	0.11795224
C	1.15421901	0.29870273	1.26569062
C	1.71038813	-0.81518786	-0.83166731
C	2.73550213	-0.18717070	-0.28605035
C	2.39127789	0.57946819	0.92852376
N	-1.78518332	0.31071425	0.65859358
C	-3.02203344	-0.31619915	0.44290895
H	3.10412430	1.16819215	1.39531726
C	-1.60600790	-0.57747576	-1.50175179
C	-3.09903789	-0.47860894	-1.10215185
H	-3.61825013	0.39499913	-1.47423442
H	-3.61309485	-1.34592181	-1.50749404
H	-3.19831028	-1.26595116	0.97628869
H	-3.87403265	0.30447969	0.73847991
H	-1.38226909	0.24775938	-2.17766314

H	0.58181697	0.69422100	2.10247497
C	-1.59901583	1.54734537	1.38791441
H	-0.76680548	2.07460941	0.92307345
H	-2.49208342	2.13080361	1.26823043
H	-1.47999539	1.47941203	2.46502094
H	1.68989131	-1.27341103	-1.79843264
C	4.14175583	0.00185411	-1.03041049
H	5.00546921	-0.31215701	-0.42170251
H	4.20844833	-0.68235893	-1.87091474
H	4.29782007	1.04999633	-1.27172054
H	-1.32714010	-1.50857431	-1.97680089

Time = 248.00 fs
E(CASSCF/6-31G(d)) = -479.4677559

C	-0.81989361	-0.32871674	-0.15760322
C	0.54401127	-0.55119794	0.12459028
C	1.15375891	0.29795431	1.26783596
C	1.70839621	-0.81707643	-0.82592270
C	2.73092967	-0.19008135	-0.29494195
C	2.39593052	0.58356592	0.91959410
N	-1.78842596	0.30732231	0.66476823
C	-3.02794852	-0.31973257	0.43884843
H	3.12509029	1.18176668	1.38307949
C	-1.59949277	-0.57184264	-1.50168719
C	-3.09572139	-0.47621208	-1.10689910
H	-3.61851663	0.39259285	-1.48742311
H	-3.60240824	-1.35012812	-1.50666619
H	-3.21098308	-1.27486224	0.96479666
H	-3.87995648	0.29460810	0.74051927
H	-1.36771998	0.24714108	-2.18207289
H	0.57711620	0.72632138	2.11430427
C	-1.61044495	1.54299202	1.39088479
H	-0.77067835	2.06743628	0.93802229
H	-2.50042065	2.13518215	1.26313172
H	-1.49789419	1.48692851	2.47512679
H	1.68633803	-1.28297617	-1.79014149
C	4.14382051	-0.00383785	-1.03131626
H	5.01034093	-0.32513270	-0.43701837
H	4.19803647	-0.68273658	-1.87406775
H	4.30485189	1.04464121	-1.28400445
H	-1.32546737	-1.50804220	-1.97272879

Time = 248.50 fs
E(CASSCF/6-31G(d)) = -479.4690496

C	-0.81765908	-0.31717166	-0.15285949

C	0.55388325	-0.55104334	0.13125376
C	1.15276729	0.29706455	1.27002397
C	1.70573394	-0.81905527	-0.82004899
C	2.72734349	-0.19256687	-0.30398659
C	2.40086971	0.58744644	0.91045261
N	-1.79131822	0.30381369	0.67100539
C	-3.03438205	-0.32334807	0.43468748
H	3.14701036	1.19666101	1.37248249
C	-1.59286157	-0.56615406	-1.50147121
C	-3.09229249	-0.47396076	-1.11159080
H	-3.61785393	0.39037457	-1.50141512
H	-3.59243473	-1.35429241	-1.50462864
H	-3.22215023	-1.28302028	0.95281434
H	-3.88486705	0.28511261	0.74300359
H	-1.35237485	0.24593720	-2.18626621
H	0.57437629	0.75652764	2.12369928
C	-1.62192170	1.53864489	1.39378347
H	-0.77519239	2.06040054	0.95393134
H	-2.50937462	2.14032150	1.25739249
H	-1.51491103	1.49343434	2.48487913
H	1.68228692	-1.29308990	-1.78155169
C	4.14566062	-0.00970433	-1.03193936
H	5.01392353	-0.33741310	-0.45155398
H	4.18703029	-0.68195750	-1.87829317
H	4.31158764	1.03851133	-1.29698477
H	-1.32408682	-1.50748263	-1.96879850

Time = 249.00 fs
E(CASSCF/6-31G(d)) = -479.4696012

C	-0.81523999	-0.30561955	-0.14826315
C	0.56330510	-0.55049694	0.13794385
C	1.15134288	0.29613233	1.27231335
C	1.70237059	-0.82117273	-0.81411562
C	2.72483644	-0.19456293	-0.31310551
C	2.40609070	0.59116009	0.90118568
N	-1.79389081	0.30017772	0.67731059
C	-3.04128399	-0.32704570	0.43042384
H	3.16870335	1.21195284	1.36279979
C	-1.58612031	-0.56042319	-1.50109464
C	-3.08875146	-0.47184859	-1.11623020
H	-3.61625751	0.38828530	-1.51608963
H	-3.58317713	-1.35837920	-1.50150541
H	-3.23168419	-1.29036240	0.94032941
H	-3.88889579	0.27617359	0.74587836
H	-1.33635316	0.24421470	-2.19024786
H	0.57414430	0.78410767	2.12972012

C	-1.63345374	1.53434746	1.39665759
H	-0.78033181	2.05355204	0.97065580
H	-2.51869214	2.14596767	1.25102081
H	-1.53118590	1.49881318	2.49389457
H	1.67765742	-1.30373139	-1.77256874
C	4.14724520	-0.01571979	-1.03233890
H	5.01636314	-0.34906003	-0.46502343
H	4.17553009	-0.68019488	-1.88370847
H	4.31795309	1.03140095	-1.31041263
H	-1.32291464	-1.50678188	-1.96490720

Time = 249.50 fs
E(CASSCF/6-31G(d)) = -479.4696840

C	-0.81259290	-0.29411532	-0.14382912
C	0.57214663	-0.54955209	0.14466128
C	1.14960328	0.29524645	1.27473641
C	1.69829151	-0.82346932	-0.80818485
C	2.72348013	-0.19601537	-0.32222654
C	2.41158276	0.59475344	0.89187576
N	-1.79617777	0.29640340	0.68368783
C	-3.04859269	-0.33081900	0.42605340
H	3.18906611	1.22677469	1.35337400
C	-1.57927531	-0.55466592	-1.50054847
C	-3.08510034	-0.46986651	-1.12082082
H	-3.61372722	0.38625159	-1.53131371
H	-3.57462776	-1.36235569	-1.49743966
H	-3.23950060	-1.29689237	0.92736388
H	-3.89221920	0.26797483	0.74909847
H	-1.31978116	0.24205646	-2.19403104
H	0.57674162	0.80846966	2.13174056
C	-1.64505315	1.53014295	1.39955109
H	-0.78604269	2.04695758	0.98802553
H	-2.52808363	2.15183267	1.24405380
H	-1.54686978	1.50296105	2.50183619
H	1.67236557	-1.31487845	-1.76310597
C	4.14854325	-0.02186038	-1.03257962
H	5.01784421	-0.36016283	-0.47715579
H	4.16364489	-0.67761707	-1.89037075
H	4.32388254	1.02315992	-1.32403197
H	-1.32186105	-1.50583263	-1.96095108

Time = 250.00 fs
E(CASSCF/6-31G(d)) = -479.4697248

C	-0.80968242	-0.28271161	-0.13957115
C	0.58028756	-0.54820463	0.15140618

C	1.14767957	0.29448345	1.27730169
C	1.69350117	-0.82597493	-0.80230902
C	2.72332063	-0.19688296	-0.33128581
C	2.41732489	0.59826420	0.88259663
N	-1.79821540	0.29247985	0.69013957
C	-3.05623706	-0.33465442	0.42157076
H	3.20716083	1.24038428	1.34366248
C	-1.57233321	-0.54889969	-1.49982408
C	-3.08134259	-0.46800368	-1.12536632
H	-3.61027006	0.38419303	-1.54694627
H	-3.56676800	-1.36619111	-1.49259131
H	-3.24556043	-1.30267957	0.91397297
H	-3.89504267	0.26069099	0.75262510
H	-1.30279119	0.23955665	-2.19763474
H	0.58228312	0.82917630	2.12942326
C	-1.65673369	1.52607251	1.40250209
H	-0.79224037	2.04069568	1.00585341
H	-2.53725314	2.15761663	1.23655248
H	-1.56212008	1.50578811	2.50843868
H	1.66632534	-1.32651233	-1.75309570
C	4.14952522	-0.02810631	-1.03273055
H	5.01857012	-0.37083116	-0.48770975
H	4.15149323	-0.67437921	-1.89826664
H	4.32932348	1.01370785	-1.33758789
H	-1.32083035	-1.50454268	-1.95683086

Time = 250.50 fs
E(CASSCF/6-31G(d)) = -479.4701355

C	-0.80648143	-0.27145820	-0.13550159
C	0.58761840	-0.54645278	0.15817794
C	1.14571146	0.29390576	1.27999636
C	1.68802494	-0.82870697	-0.79652907
C	2.72437557	-0.19713918	-0.34022877
C	2.42328496	0.60171906	0.87341102
N	-1.80004112	0.28839667	0.69666705
C	-3.06413900	-0.33853122	0.41696983
H	3.22225822	1.25219883	1.33325522
C	-1.56530072	-0.54314212	-1.49891318
C	-3.07748278	-0.46624824	-1.12987032
H	-3.60590350	0.38203021	-1.56284351
H	-3.55956805	-1.36985714	-1.48713503
H	-3.24987057	-1.30785567	0.90024211
H	-3.89758283	0.25447365	0.75642129
H	-1.28552039	0.23681492	-2.20108024
H	0.59069535	0.84595623	2.12269729
C	-1.66850813	1.52217339	1.40554099

H	-0.79881806	2.03485114	1.02394415
H	-2.54592674	2.16303075	1.22859547
H	-1.57709659	1.50722134	2.51352783
H	1.65945056	-1.33862143	-1.74249616
C	4.15016487	-0.03444359	-1.03286329
H	5.01873983	-0.38118535	-0.49648896
H	4.13920328	-0.67061870	-1.90730700
H	4.33423998	1.00304204	-1.35083428
H	-1.31972039	-1.50284277	-1.95245613

Time = 251.00 fs
E(CASSCF/6-31G(d)) = -479.4712328

C	-0.80297062	-0.26040189	-0.13163100
C	0.59404118	-0.54429701	0.16497492
C	1.14384321	0.29355962	1.28278773
C	1.68190950	-0.83166959	-0.79087408
C	2.72663250	-0.19677331	-0.34901032
C	2.42942133	0.60513348	0.86436965
N	-1.80169280	0.28414464	0.70327070
C	-3.07221626	-0.34242140	0.41224488
H	3.23385082	1.26180707	1.32187664
C	-1.55818442	-0.53740987	-1.49780820
C	-3.07352613	-0.46458827	-1.13433619
H	-3.60065803	0.37969212	-1.57886343
H	-3.55298683	-1.37332686	-1.48125730
H	-3.25248273	-1.31260527	0.88628193
H	-3.90004686	0.24943685	0.76044596
H	-1.26811088	0.23393101	-2.20438805
H	0.60172752	0.85871834	2.11174848
C	-1.68038589	1.51847758	1.40868897
H	-0.80565892	2.02950802	1.04210372
H	-2.55387453	2.16781592	1.22027294
H	-1.59195845	1.50720687	2.51703114
H	1.65165684	-1.35120342	-1.73129625
C	4.15044127	-0.04086512	-1.03304987
H	5.01852499	-0.39134542	-0.50335495
H	4.12691322	-0.66645546	-1.91732949
H	4.33861551	0.99124096	-1.36354111
H	-1.31842375	-1.50069209	-1.94774932

Time = 251.50 fs
E(CASSCF/6-31G(d)) = -479.4731085

C	-0.79913730	-0.24958644	-0.12796817
C	0.59946931	-0.54173999	0.17179412
C	1.14222108	0.29347256	1.28562378

C	1.67522197	-0.83485397	-0.78536201
C	2.73004904	-0.19579030	-0.35759429
C	2.43568501	0.60851224	0.85551093
N	-1.80320846	0.27971636	0.70995098
C	-3.08038507	-0.34629033	0.40739174
H	3.24165120	1.26896966	1.30937988
C	-1.55099045	-0.53171751	-1.49650268
C	-3.06947821	-0.46301257	-1.13876692
H	-3.59457946	0.37712283	-1.59487057
H	-3.54697238	-1.37657438	-1.47515355
H	-3.25349084	-1.31715293	0.87222119
H	-3.90261320	0.24564375	0.76464744
H	-1.25070986	0.23099954	-2.20757524
H	0.61495498	0.86757282	2.09702248
C	-1.69237112	1.51501071	1.41195681
H	-0.81264825	2.02474275	1.06014813
H	-2.56092737	2.17175887	1.21168082
H	-1.60686131	1.50571209	2.51898332
H	1.64286434	-1.36426529	-1.71951556
C	4.15034086	-0.04737096	-1.03336034
H	5.01804851	-0.40141993	-0.50823758
H	4.11477001	-0.66199611	-1.92810758
H	4.34245447	0.97846236	-1.37550055
H	-1.31682896	-1.49808192	-1.94264815

Time = 252.00 fs
E(CASSCF/6-31G(d)) = -479.4755711

C	-0.79497449	-0.23905271	-0.12452088
C	0.60382594	-0.53878661	0.17863080
C	1.14099210	0.29365093	1.28843430
C	1.66804846	-0.83824019	-0.78000177
C	2.73455448	-0.19420982	-0.36595255
C	2.44202164	0.61185021	0.84686084
N	-1.80462610	0.27510669	0.71670987
C	-3.08856262	-0.35009800	0.40240902
H	3.24558460	1.27361572	1.29573862
C	-1.54372406	-0.52607626	-1.49499143
C	-3.06534427	-0.46151104	-1.14316477
H	-3.58773053	0.37428615	-1.61074198
H	-3.54146207	-1.37957607	-1.46902491
H	-3.25302643	-1.32174628	0.85819603
H	-3.90541634	0.24309533	0.76895847
H	-1.23346955	0.22810482	-2.21065459
H	0.62977820	0.87285862	2.07923280
C	-1.70446145	1.51179130	1.41534387
H	-0.81968288	2.02061916	1.07791161

H	-2.56698708	2.17470726	1.20291500
H	-1.62195677	1.50272755	2.51952781
H	1.63299977	-1.37782182	-1.70719933
C	4.14985943	-0.05396793	-1.03386031
H	5.01736781	-0.41149665	-0.51114188
H	4.10292602	-0.65734157	-1.93936739
H	4.34578327	0.96493624	-1.38653146
H	-1.31482207	-1.49503847	-1.93710586

Time = 252.50 fs
E(CASSCF/6-31G(d)) = -479.4783566

C	-0.79048445	-0.22883472	-0.12129616
C	0.60704703	-0.53544235	0.18547823
C	1.14031234	0.29407923	1.29112883
C	1.66050440	-0.84179274	-0.77479071
C	2.74003828	-0.19207135	-0.37406701
C	2.44836431	0.61512829	0.83843433
N	-1.80598100	0.27031129	0.72354843
C	-3.09667225	-0.35380092	0.39729984
H	3.24578573	1.27585402	1.28104905
C	-1.53638979	-0.52049510	-1.49327089
C	-3.06112940	-0.46007552	-1.14753191
H	-3.58019180	0.37116968	-1.62636561
H	-3.53638487	-1.38230509	-1.46306965
H	-3.25124402	-1.32662725	0.84434523
H	-3.90853050	0.24172466	0.77329108
H	-1.21654377	0.22532024	-2.21363059
H	0.64542165	0.87517143	2.05938585
C	-1.71664574	1.50883241	1.41883912
H	-0.82669149	2.01717071	1.09525558
H	-2.57203493	2.17656137	1.19406594
H	-1.63739142	1.49826126	2.51890170
H	1.62200214	-1.39190013	-1.69441607
C	4.14900318	-0.06066822	-1.03460905
H	5.01647086	-0.42163632	-0.51214772
H	4.09153715	-0.65259475	-1.95080638
H	4.34865224	0.95095435	-1.39648626
H	-1.31229175	-1.49161605	-1.93109132

Time = 253.00 fs
E(CASSCF/6-31G(d)) = -479.4811440

C	-0.78569883	-0.21895497	-0.11829841
C	0.60909782	-0.53170941	0.19232817
C	1.14036249	0.29472556	1.29359204
C	1.65274902	-0.84545008	-0.76970789

C	2.74632778	-0.18944501	-0.38193238
C	2.45461504	0.61830434	0.83024001
N	-1.80729525	0.26532190	0.73045581
C	-3.10465030	-0.35735455	0.39207080
H	3.24259621	1.27598044	1.26552489
C	-1.52899308	-0.51498328	-1.49133638
C	-3.05683752	-0.45869898	-1.15187084
H	-3.57206132	0.36778386	-1.64164211
H	-3.53165873	-1.38473001	-1.45747103
H	-3.24829379	-1.33199992	0.83080313
H	-3.91196285	0.24139463	0.77753612
H	-1.20006959	0.22270926	-2.21648686
H	0.66095014	0.87537647	2.03877932
C	-1.72889992	1.50614448	1.42242730
H	-0.83364308	2.01438164	1.11207745
H	-2.57613793	2.17725888	1.18520441
H	-1.65331193	1.49233170	2.51741520
H	1.60982677	-1.40654394	-1.68124876
C	4.14778960	-0.06748644	-1.03565841
H	5.01527696	-0.43186985	-0.51140506
H	4.08075831	-0.64786992	-1.96212007
H	4.35114003	0.93685212	-1.40525215
H	-1.30912818	-1.48788854	-1.92458207

Time = 253.50 fs
E(CASSCF/6-31G(d)) = -479.4840263

C	-0.78066851	-0.20943053	-0.11552439
C	0.60997585	-0.52759097	0.19917016
C	1.14131687	0.29553452	1.29568690
C	1.64493623	-0.84914420	-0.76472705
C	2.75323249	-0.18641031	-0.38954302
C	2.46066772	0.62132450	0.82227653
N	-1.80858195	0.26013144	0.73741324
C	-3.11243978	-0.36071516	0.38673204
H	3.23656266	1.27446491	1.24948990
C	-1.52153963	-0.50954909	-1.48918353
C	-3.05247171	-0.45737654	-1.15618324
H	-3.56344981	0.36416483	-1.65649023
H	-3.52719225	-1.38682032	-1.45240864
H	-3.24433922	-1.33801336	0.81768305
H	-3.91566660	0.24190865	0.78156127
H	-1.18417246	0.22032408	-2.21919061
H	0.67531130	0.87458276	2.01893869
C	-1.74119025	1.50373047	1.42608902
H	-0.84054435	2.01220820	1.12830761
H	-2.57944101	2.17679664	1.17638246

H	-1.66985421	1.48498538	2.51544618
H	1.59645033	-1.42178787	-1.66778298
C	4.14624626	-0.07443858	-1.03705187
H	5.01364563	-0.44219902	-0.50912225
H	4.07073779	-0.64329668	-1.97301906
H	4.35334501	0.92299064	-1.41274673
H	-1.30522251	-1.48395046	-1.91757086

Time = 254.00 fs
E(CASSCF/6-31G(d)) = -479.4866692

C	-0.77544241	-0.20027747	-0.11296645
C	0.60968970	-0.52309579	0.20598996
C	1.14332288	0.29643053	1.29727421
C	1.63719579	-0.85281657	-0.75982902
C	2.76057322	-0.18304170	-0.39688805
C	2.46642561	0.62413193	0.81452880
N	-1.80985575	0.25473929	0.74440618
C	-3.11998479	-0.36384338	0.38129926
H	3.22842578	1.27193708	1.23336835
C	-1.51403362	-0.50419762	-1.48681125
C	-3.04803444	-0.45610592	-1.16046911
H	-3.55447746	0.36037466	-1.67084962
H	-3.52288978	-1.38855089	-1.44806305
H	-3.23956946	-1.34474698	0.80506203
H	-3.91955720	0.24302392	0.78521528
H	-1.16898051	0.21820217	-2.22170371
H	0.68741970	0.87404628	2.00147646
C	-1.75347822	1.50158286	1.42979701
H	-0.84743334	2.01059701	1.14390453
H	-2.58215506	2.17525059	1.16764005
H	-1.68713828	1.47630323	2.51342596
H	1.58187048	-1.43763805	-1.65409156
C	4.14440975	-0.08154084	-1.03882365
H	5.01139343	-0.45259966	-0.50555209
H	4.06161175	-0.63902046	-1.98324396
H	4.35537519	0.90973617	-1.41891202
H	-1.30047417	-1.47991467	-1.91006735

Time = 254.50 fs
E(CASSCF/6-31G(d)) = -479.4886455

C	-0.77007211	-0.19150915	-0.11061385
C	0.60825970	-0.51823676	0.21276889
C	1.14650050	0.29733692	1.29823169
C	1.62964457	-0.85641407	-0.75499916
C	2.76817144	-0.17941303	-0.40395498

C	2.47179349	0.62666365	0.80697079
N	-1.81113007	0.24914929	0.75142186
C	-3.12723318	-0.36670748	0.37579311
H	3.21909352	1.26917177	1.21767308
C	-1.50647825	-0.49893225	-1.48422087
C	-3.04352720	-0.45488663	-1.16472749
H	-3.54527088	0.35649618	-1.68467904
H	-3.51865316	-1.38990182	-1.44460729
H	-3.23418403	-1.35219153	0.79297754
H	-3.92352446	0.24446760	0.78833778
H	-1.15461734	0.21636831	-2.22398252
H	0.69629927	0.87499313	1.98787783
C	-1.76572048	1.49968609	1.43352029
H	-0.85437777	2.00948015	1.15885624
H	-2.58454763	2.17275893	1.15900032
H	-1.70527314	1.46639287	2.51179989
H	1.56610863	-1.45407242	-1.64022330
C	4.14232445	-0.08880663	-1.04099902
H	5.00831733	-0.46302993	-0.50097710
H	4.05350001	-0.63520300	-1.99258232
H	4.35734583	0.89743374	-1.42371041
H	-1.29479378	-1.47590019	-1.90209071

Time = 255.00 fs
E(CASSCF/6-31G(d)) = -479.4901016

C	-0.76461541	-0.18313506	-0.10845303
C	0.60572182	-0.51303080	0.21948382
C	1.15093156	0.29819829	1.29847625
C	1.62238703	-0.85988836	-0.75022645
C	2.77584790	-0.17559858	-0.41073070
C	2.47667713	0.62885198	0.79956969
N	-1.81241560	0.24336874	0.75844748
C	-3.13413679	-0.36928543	0.37023817
H	3.20958409	1.26704717	1.20297391
C	-1.49887617	-0.49375612	-1.48141579
C	-3.03895057	-0.45371952	-1.16895673
H	-3.53595935	0.35262917	-1.69795355
H	-3.51438518	-1.39085983	-1.44220279
H	-3.22838531	-1.36024211	0.78142881
H	-3.92744898	0.24595581	0.79076785
H	-1.14119530	0.21483969	-2.22598091
H	0.70126561	0.87837513	1.97922576
C	-1.77787025	1.49801805	1.43722847
H	-0.86146831	2.00877702	1.17317756
H	-2.58692977	2.16950642	1.15046955
H	-1.72435660	1.45538508	2.51098658

H	1.54921423	-1.47103567	-1.62619480
C	4.14004097	-0.09624379	-1.04359508
H	5.00421911	-0.47343948	-0.49569198
H	4.04650206	-0.63202099	-2.00088140
H	4.35937358	0.88638229	-1.42711940
H	-1.28810478	-1.47202260	-1.89366716

Time = 255.50 fs
E(CASSCF/6-31G(d)) = -479.4915388

C	-0.75913624	-0.17516094	-0.10646817
C	0.60212788	-0.50749858	0.22610738
C	1.15664703	0.29900005	1.29798466
C	1.61551304	-0.86319733	-0.74550391
C	2.78342592	-0.17167171	-0.41720219
C	2.48099037	0.63063121	0.79229073
N	-1.81372033	0.23740828	0.76547031
C	-3.14065068	-0.37156617	0.36466148
H	3.20092827	1.26646447	1.18984363
C	-1.49122969	-0.48867284	-1.47840133
C	-3.03430422	-0.45260603	-1.17315482
H	-3.52666880	0.34888277	-1.71066107
H	-3.50999087	-1.39141886	-1.44099545
H	-3.22237496	-1.36870338	0.77037917
H	-3.93121749	0.24721207	0.79235374
H	-1.12881271	0.21362726	-2.22765321
H	0.70208945	0.88463832	1.97596090
C	-1.78987980	1.49655101	1.44089538
H	-0.86880903	2.00840026	1.18690691
H	-2.58963655	2.16570849	1.14203902
H	-1.74447621	1.44343227	2.51133434
H	1.53126722	-1.48843566	-1.61198735
C	4.13761427	-0.10385241	-1.04662184
H	4.99892615	-0.48377847	-0.48998778
H	4.04069441	-0.62966111	-2.00805506
H	4.36156959	0.87680942	-1.42912465
H	-1.28034661	-1.46838269	-1.88482481

Time = 256.00 fs
E(CASSCF/6-31G(d)) = -479.4933545

C	-0.75370431	-0.16758886	-0.10464149
C	0.59754547	-0.50166436	0.23260852
C	1.16362114	0.29977787	1.29680150
C	1.60909623	-0.86630609	-0.74082860
C	2.79073458	-0.16770385	-0.42335714
C	2.48466529	0.63194708	0.78510356

N	-1.81504983	0.23128183	0.77247752
C	-3.14673232	-0.37354965	0.35909097
H	3.19403720	1.26823256	1.17878446
C	-1.48354104	-0.48368744	-1.47518463
C	-3.02958705	-0.45154758	-1.17731976
H	-3.51751617	0.34536877	-1.72279740
H	-3.50537991	-1.39158078	-1.44111316
H	-3.21635236	-1.37730628	0.75976419
H	-3.93473666	0.24798490	0.79296110
H	-1.11755027	0.21273898	-2.22895700
H	0.69907198	0.89360902	1.97778610
C	-1.80170340	1.49525287	1.44450252
H	-0.87650720	2.00826186	1.20010130
H	-2.59300382	2.16159103	1.13369048
H	-1.76570731	1.43070822	2.51308138
H	1.51237922	-1.50614246	-1.59754901
C	4.13510161	-0.11162314	-1.05008312
H	4.99230971	-0.49400537	-0.48413732
H	4.03612938	-0.62831491	-2.01408780
H	4.36403281	0.86885248	-1.42971556
H	-1.27147685	-1.46505689	-1.87559115

Time = 256.50 fs
E(CASSCF/6-31G(d)) = -479.4955453

C	-0.74839528	-0.16041679	-0.10295437
C	0.59205777	-0.49555624	0.23895436
C	1.17177573	0.30061226	1.29502983
C	1.60319379	-0.86918680	-0.73620123
C	2.79761125	-0.16376419	-0.42918532
C	2.48766407	0.63276841	0.77798996
N	-1.81640756	0.22500617	0.77945678
C	-3.15234058	-0.37524566	0.35355375
H	3.18955714	1.27293398	1.17014471
C	-1.47581269	-0.47880696	-1.47177452
C	-3.02479730	-0.45054480	-1.18144985
H	-3.50860430	0.34219428	-1.73436309
H	-3.50046914	-1.39135760	-1.44266369
H	-3.21051263	-1.38573421	0.74950132
H	-3.93794527	0.24806275	0.79248230
H	-1.10746819	0.21218282	-2.22985879
H	0.69299551	0.90455611	1.98376360
C	-1.81330066	1.49408828	1.44804099
H	-0.88466071	2.00828028	1.21283324
H	-2.59734062	2.15736998	1.12540274
H	-1.78811296	1.41740699	2.51632531
H	1.49269323	-1.52399021	-1.58279854

C	4.13256050	-0.11953634	-1.05397729
H	4.98429759	-0.50409457	-0.47838294
H	4.03283481	-0.62817107	-2.01903289
H	4.36684510	0.86254525	-1.42888169
H	-1.26147288	-1.46209188	-1.86599029

Time = 257.00 fs
E(CASSCF/6-31G(d)) = -479.4977464

C	-0.74328947	-0.15363907	-0.10138751
C	0.58576284	-0.48920487	0.24511153
C	1.18099298	0.30161015	1.29280951
C	1.59784583	-0.87181879	-0.73162523
C	2.80390466	-0.15991866	-0.43467927
C	2.48998833	0.63309661	0.77094921
N	-1.81779543	0.21860083	0.78639627
C	-3.15743553	-0.37667245	0.34807439
H	3.18775448	1.28080966	1.16405160
C	-1.46804759	-0.47404090	-1.46818144
C	-3.01993277	-0.44959707	-1.18554414
H	-3.50001673	0.33945479	-1.74535706
H	-3.49518413	-1.39077025	-1.44573259
H	-3.20504514	-1.39365231	0.73950164
H	-3.94082100	0.24728513	0.79084091
H	-1.09860396	0.21196844	-2.23033494
H	0.68496943	0.91639606	1.99256171
C	-1.82463978	1.49302044	1.45151262
H	-0.89334817	2.00838589	1.22518505
H	-2.60290303	2.15322773	1.11716179
H	-1.81173818	1.40374306	2.52100846
H	1.47238336	-1.54178345	-1.56763645
C	4.13004659	-0.12756221	-1.05829774
H	4.97488257	-0.51404036	-0.47292714
H	4.03081468	-0.62940919	-2.02300893
H	4.37006647	0.85781503	-1.42661453
H	-1.25033324	-1.45949855	-1.85604259

Time = 257.50 fs
E(CASSCF/6-31G(d)) = -479.4994463

C	-0.73847130	-0.14724634	-0.09992163
C	0.57877297	-0.48264281	0.25104843
C	1.19113079	0.30288360	1.29029256
C	1.59307615	-0.87418765	-0.72710573
C	2.80947707	-0.15622966	-0.43983550
C	2.49168251	0.63297043	0.76400072
N	-1.81921415	0.21208775	0.79328486

C	-3.16197826	-0.37785469	0.34267358
H	3.18847416	1.29170296	1.16038208
C	-1.46024950	-0.46940134	-1.46441723
C	-3.01499101	-0.44870207	-1.18960269
H	-3.49181390	0.33722834	-1.75577240
H	-3.48946051	-1.38984816	-1.45038140
H	-3.20013048	-1.40073760	0.72968037
H	-3.94338444	0.24555006	0.78799598
H	-1.09097084	0.21210858	-2.23037393
H	0.67624196	0.92793847	2.00273086
C	-1.83570054	1.49201254	1.45492914
H	-0.90261932	2.00852565	1.23724508
H	-2.60986973	2.14929319	1.10897003
H	-1.83660566	1.38994909	2.52692173
H	1.45165223	-1.55930583	-1.55195718
C	4.12761163	-0.13566186	-1.06303308
H	4.96412564	-0.52386028	-0.46792581
H	4.03005076	-0.63219207	-2.02619100
H	4.37373354	0.85448825	-1.42291162
H	-1.23807819	-1.45725097	-1.84576458

Time = 258.00 fs
E(CASSCF/6-31G(d)) = -479.5002136

C	-0.73402834	-0.14122575	-0.09853795
C	0.57121370	-0.47590350	0.25673716
C	1.20203591	0.30453224	1.28762362
C	1.58889333	-0.87628425	-0.72264833
C	2.81420636	-0.15275580	-0.44465538
C	2.49282952	0.63246351	0.75718190
N	-1.82066377	0.20549090	0.80011228
C	-3.16593110	-0.37882131	0.33736706
H	3.19118860	1.30508558	1.15878404
C	-1.45242329	-0.46490260	-1.46049503
C	-3.00996934	-0.44785555	-1.19362686
H	-3.48403062	0.33557137	-1.76559283
H	-3.48324487	-1.38862779	-1.45664638
H	-3.19593724	-1.40670642	0.71996612
H	-3.94569967	0.24281770	0.78394359
H	-1.08455716	0.21261962	-2.22997729
H	0.66803921	0.93808489	2.01292060
C	-1.84647654	1.49102915	1.45830988
H	-0.91248785	2.00866659	1.24910395
H	-2.61832433	2.14562736	1.10085459
H	-1.86271039	1.37627212	2.53372708
H	1.43072742	-1.57633115	-1.53566246
C	4.12530146	-0.14378914	-1.06816730

H	4.95215476	-0.53359617	-0.46348425
H	4.03050494	-0.63665792	-2.02879790
H	4.37785999	0.85230449	-1.41778352
H	-1.22474944	-1.45528845	-1.83517034

Time = 258.50 fs
E(CASSCF/6-31G(d)) = -479.4998111

C	-0.73005023	-0.13556109	-0.09721851
C	0.56322278	-0.46902045	0.26215518
C	1.21355228	0.30663319	1.28492848
C	1.58529228	-0.87810343	-0.71825799
C	2.81798769	-0.14955174	-0.44914597
C	2.49354052	0.63167583	0.75054254
N	-1.82214398	0.19883588	0.80686924
C	-3.16925816	-0.37960342	0.33216500
H	3.19511826	1.32015440	1.15873928
C	-1.44457517	-0.46056082	-1.45642904
C	-3.00486501	-0.44705136	-1.19761943
H	-3.47667508	0.33451660	-1.77479068
H	-3.47649508	-1.38715052	-1.46453749
H	-3.19261810	-1.41133708	0.71030764
H	-3.94787122	0.23910996	0.77871611
H	-1.07932651	0.21352115	-2.22915918
H	0.66145984	0.94594517	2.02200523
C	-1.85697628	1.49003724	1.46167872
H	-0.92292738	2.00879746	1.26085174
H	-2.62824697	2.14221724	1.09287189
H	-1.89001574	1.36296716	2.54099369
H	1.40985627	-1.59263557	-1.51867459
C	4.12315411	-0.15189326	-1.07367993
H	4.93915907	-0.54331364	-0.45965610
H	4.03212237	-0.64291210	-2.03107518
H	4.38243934	0.85093708	-1.41126164
H	-1.21040906	-1.45352048	-1.82427355

Time = 259.00 fs
E(CASSCF/6-31G(d)) = -479.4982227

C	-0.72662713	-0.13023321	-0.09594640
C	0.55494836	-0.46202644	0.26728637
C	1.22552557	0.30923717	1.28230951
C	1.58225590	-0.87964277	-0.71393805
C	2.82073498	-0.14666795	-0.45332055
C	2.49394213	0.63072197	0.74413744
N	-1.82365454	0.19214957	0.81354749
C	-3.17192612	-0.38023238	0.32707186

H	3.19937878	1.33596335	1.15964518
C	-1.43671295	-0.45639307	-1.45223448
C	-2.99967514	-0.44628169	-1.20158460
H	-3.46972962	0.33407323	-1.78332645
H	-3.46918016	-1.38545967	-1.47403783
H	-3.19030621	-1.41448677	0.70067861
H	-3.95003785	0.23450645	0.77237891
H	-1.07521875	0.21483489	-2.22794486
H	0.65742197	0.95088227	2.02913198
C	-1.86722295	1.48900672	1.46506059
H	-0.93387082	2.00892895	1.27257551
H	-2.63951683	2.13897924	1.08510790
H	-1.91845096	1.35028888	2.54824163
H	1.38929998	-1.60801010	-1.50094786
C	4.12119842	-0.15992179	-1.07954647
H	4.92537919	-0.55309932	-0.45644541
H	4.03483486	-0.65101873	-2.03327447
H	4.38744910	0.85001759	-1.40340608
H	-1.19513781	-1.45183448	-1.81309056

Time = 259.50 fs
E(CASSCF/6-31G(d)) = -479.4956348

C	-0.72384783	-0.12522038	-0.09470561
C	0.54654670	-0.45495287	0.27212147
C	1.23780500	0.31236877	1.27984548
C	1.57975696	-0.88090142	-0.70968956
C	2.82238183	-0.14415066	-0.45719895
C	2.49416489	0.62971981	0.73801943
N	-1.82519558	0.18545963	0.82013978
C	-3.17390529	-0.38073799	0.32208663
H	3.20311239	1.35155018	1.16089115
C	-1.42884620	-0.45241645	-1.44792749
C	-2.99439669	-0.44553762	-1.20552792
H	-3.46315303	0.33422929	-1.79115028
H	-3.46127982	-1.38359731	-1.48510357
H	-3.18911193	-1.41610271	0.69108003
H	-3.95236263	0.22913647	0.76502445
H	-1.07215190	0.21658286	-2.22636815
H	0.65664540	0.95251237	2.03372257
C	-1.87725281	1.48791058	1.46847839
H	-0.94521343	2.00909193	1.28435772
H	-2.65192501	2.13577156	1.07767318
H	-1.94791154	1.33848194	2.55498623
H	1.36932665	-1.62227212	-1.48247751
C	4.11945302	-0.16782396	-1.08573893
H	4.91109350	-0.56305647	-0.45381202

H	4.03856453	-0.66099249	-2.03563072
H	4.39285591	0.84916170	-1.39431172
H	-1.17903256	-1.45010508	-1.80164359

Time = 260.00 fs
E(CASSCF/6-31G(d)) = -479.4923778

C	-0.72179764	-0.12049880	-0.09348095
C	0.53817917	-0.44782945	0.27665802
C	1.25024414	0.31602878	1.27759320
C	1.57775990	-0.88187906	-0.70551087
C	2.82288219	-0.14204173	-0.46080754
C	2.49433505	0.62878168	0.73223421
N	-1.82676788	0.17879405	0.82663986
C	-3.17517086	-0.38114686	0.31720356
H	3.20558053	1.36603326	1.16191598
C	-1.42098644	-0.44864696	-1.44352511
C	-2.98902629	-0.44480974	-1.20945600
H	-3.45688388	0.33495582	-1.79820491
H	-3.45278353	-1.38160059	-1.49766415
H	-3.18911996	-1.41622738	0.68154048
H	-3.95501945	0.22316733	0.75676372
H	-1.07002484	0.21878482	-2.22446748
H	0.65965128	0.95068556	2.03545589
C	-1.88711244	1.48672473	1.47195060
H	-0.95682014	2.00933345	1.29627564
H	-2.66519525	2.13241313	1.07069281
H	-1.97826270	1.32777088	2.56077774
H	1.35020372	-1.63527557	-1.46330552
C	4.11792608	-0.17555393	-1.09222657
H	4.89660080	-0.57329875	-0.45168059
H	4.04322708	-0.67279073	-2.03833909
H	4.39862115	0.84799418	-1.38411149
H	-1.16220324	-1.44820414	-1.78996337

Time = 260.50 fs
E(CASSCF/6-31G(d)) = -479.4888428

C	-0.72055558	-0.11604344	-0.09225721
C	0.53000878	-0.44068344	0.28089962
C	1.26270276	0.32019631	1.27558935
C	1.57622279	-0.88257502	-0.70139728
C	2.82221012	-0.14037845	-0.46417875
C	2.49456880	0.62800842	0.72681688
N	-1.82837312	0.17218091	0.83304235
C	-3.17570452	-0.38148090	0.31241324
H	3.20621557	1.37867223	1.16224351

C	-1.41314707	-0.44509848	-1.43904567
C	-2.98356027	-0.44408897	-1.21337628
H	-3.45084597	0.33621213	-1.80443005
H	-3.44369005	-1.37949910	-1.51162359
H	-3.19038857	-1.41499877	0.67211536
H	-3.95817689	0.21679039	0.74771642
H	-1.06872023	0.22145590	-2.22228366
H	0.66676589	0.94546565	2.03424919
C	-1.89685526	1.48542764	1.47548969
H	-0.96853657	2.00971144	1.30840130
H	-2.67900928	2.12870503	1.06429511
H	-2.00934401	1.31835120	2.56523343
H	1.33219044	-1.64691745	-1.44352312
C	4.11661574	-0.18307384	-1.09897660
H	4.88220089	-0.58394206	-0.44995246
H	4.04873456	-0.68630857	-2.04153561
H	4.40470644	0.84617172	-1.37297738
H	-1.14476944	-1.44601199	-1.77809291

Time = 261.00 fs
E(CASSCF/6-31G(d)) = -479.4853887

C	-0.72019113	-0.11182896	-0.09101854
C	0.52219505	-0.43353960	0.28485455
C	1.27504873	0.32483155	1.27385199
C	1.57509878	-0.88298790	-0.69734144
C	2.82035984	-0.13919335	-0.46735045
C	2.49496992	0.62748598	0.72179065
N	-1.83001409	0.16564781	0.83934248
C	-3.17549583	-0.38175634	0.30770387
H	3.20464186	1.38890039	1.16149849
C	-1.40534353	-0.44178175	-1.43450865
C	-2.97799435	-0.44336732	-1.21729658
H	-3.44495242	0.33795179	-1.80976685
H	-3.43400604	-1.37731101	-1.52686114
H	-3.19294803	-1.41264333	0.66288319
H	-3.96197908	0.21020420	0.73799869
H	-1.06810835	0.22460338	-2.21985317
H	0.67811947	0.93711346	2.03024817
C	-1.90653755	1.48400018	1.47910136
H	-0.98020181	2.01028703	1.32080087
H	-2.69303285	2.12445278	1.05859764
H	-2.04097630	1.31038136	2.56806173
H	1.31553038	-1.65714542	-1.42327066
C	4.11551125	-0.19035637	-1.10595459
H	4.86817330	-0.59509519	-0.44852146
H	4.05499698	-0.70137562	-2.04528291

H	4.41107767	0.84340151	-1.36111810
H	-1.12685684	-1.44342477	-1.76608827

Time = 261.50 fs
E(CASSCF/6-31G(d)) = -479.4822649

C	-0.72076100	-0.10783063	-0.08974754
C	0.51488918	-0.42641986	0.28853435
C	1.28716133	0.32987738	1.27238050
C	1.57433752	-0.88311523	-0.69333372
C	2.81734500	-0.13851383	-0.47036495
C	2.49562812	0.62728363	0.71716642
N	-1.83169481	0.15922149	0.84553589
C	-3.17454373	-0.38198315	0.30306280
H	3.20067852	1.39633751	1.15941280
C	-1.39759331	-0.43870353	-1.42993486
C	-2.97232345	-0.44263855	-1.22122470
H	-3.43911081	0.34012849	-1.81416286
H	-3.42374543	-1.37504127	-1.54323302
H	-3.19680082	-1.40946317	0.65394070
H	-3.96652630	0.20359793	0.72771177
H	-1.06805057	0.22822403	-2.21720406
H	0.69363889	0.92607890	2.02382993
C	-1.91621441	1.48242556	1.48278415
H	-0.99166411	2.01111566	1.33353525
H	-2.70693993	2.11948537	1.05369444
H	-2.07296963	1.30397640	2.56907827
H	1.30044406	-1.66596062	-1.40273411
C	4.11459465	-0.19738678	-1.11312446
H	4.85475723	-0.60684987	-0.44729028
H	4.06192326	-0.71775726	-2.04956534
H	4.41770895	0.83945652	-1.34877429
H	-1.10859352	-1.44036222	-1.75401946

Time = 262.00 fs
E(CASSCF/6-31G(d)) = -479.4795577

C	-0.72230623	-0.10402510	-0.08842403
C	0.50822987	-0.41934275	0.29195246
C	1.29893591	0.33525994	1.27115424
C	1.57388645	-0.88295346	-0.68936245
C	2.81319739	-0.13836162	-0.47326757
C	2.49661760	0.62745271	0.71294239
N	-1.83342051	0.15292764	0.85161853
C	-3.17285805	-0.38216510	0.29847809
H	3.19433527	1.40079356	1.15582873
C	-1.38991573	-0.43586606	-1.42534702

C	-2.96654174	-0.44189882	-1.22516814
H	-3.43322915	0.34270133	-1.81757742
H	-3.41292938	-1.37268149	-1.56057543
H	-3.20192392	-1.40581701	0.64539638
H	-3.97185801	0.19713717	0.71693306
H	-1.06840260	0.23230241	-2.21435465
H	0.71303433	0.91300213	2.01561722
C	-1.92593602	1.48068946	1.48652971
H	-1.00279864	2.01223802	1.34666159
H	-2.72043383	2.11367002	1.04964544
H	-2.10513205	1.29920367	2.56821507
H	1.28712342	-1.67341509	-1.38213893
C	4.11384256	-0.20416432	-1.12044811
H	4.84213440	-0.61927156	-0.44618609
H	4.06942097	-0.73516110	-2.05429350
H	4.42458600	0.83418668	-1.33621198
H	-1.09010639	-1.43677511	-1.74197170

Time = 262.50 fs
E(CASSCF/6-31G(d)) = -479.4771580

C	-0.72484896	-0.10039119	-0.08702430
C	0.50233806	-0.41232313	0.29512268
C	1.31028811	0.34088963	1.27013093
C	1.57369131	-0.88249852	-0.68541488
C	2.80796616	-0.13875197	-0.47610522
C	2.49799610	0.62802556	0.70910446
N	-1.83519757	0.14679038	0.85758654
C	-3.17046023	-0.38230091	0.29393981
H	3.18580760	1.40227275	1.15069893
C	-1.38233189	-0.43326677	-1.42076958
C	-2.96064234	-0.44114699	-1.22913372
H	-3.42721939	0.34563936	-1.81998519
H	-3.40158631	-1.37021017	-1.57870721
H	-3.20826981	-1.40209258	0.63736127
H	-3.97793982	0.19095303	0.70570926
H	-1.06901755	0.23680978	-2.21131000
H	0.73578352	0.89871327	2.00649688
C	-1.93574433	1.47878031	1.49032305
H	-1.01352133	2.01367142	1.36023308
H	-2.73326320	2.10692342	1.04646707
H	-2.13727851	1.29608004	2.56552456
H	1.27572677	-1.67961015	-1.36174161
C	4.11322810	-0.21070273	-1.12788474
H	4.83041701	-0.63239153	-0.44517379
H	4.07739616	-0.75324725	-2.05931784
H	4.43170777	0.82752463	-1.32371382

H	-1.07151758	-1.43264830	-1.73004356

Time = 263.00 fs
E(CASSCF/6-31G(d)) = -479.4747874

C	-0.72839007	-0.09691057	-0.08552019
C	0.49731281	-0.40537173	0.29805788
C	1.32115774	0.34666234	1.26924538
C	1.57369659	-0.88174663	-0.68147801
C	2.80171661	-0.13969280	-0.47892476
C	2.49980329	0.62901446	0.70562638
N	-1.83703357	0.14083208	0.86343611
C	-3.16738360	-0.38238649	0.28944123
H	3.17547436	1.40097825	1.14408837
C	-1.37486455	-0.43089827	-1.41622881
C	-2.95461728	-0.44038473	-1.23312721
H	-3.42100092	0.34892445	-1.82137923
H	-3.38975182	-1.36759421	-1.59743378
H	-3.21576857	-1.39867284	0.62993857
H	-3.98465737	0.18513703	0.69405468
H	-1.06974886	0.24170342	-2.20805999
H	0.76112617	0.88422423	1.99762937
C	-1.94567052	1.47668962	1.49414336
H	-1.02380024	2.01540315	1.37429864
H	-2.74523420	2.09921797	1.04412573
H	-2.16923945	1.29457088	2.56117752
H	1.26637560	-1.68468981	-1.34182009
C	4.11272242	-0.21702962	-1.13539029
H	4.81964320	-0.64620267	-0.44426357
H	4.08575224	-0.77164350	-2.06444882
H	4.43908706	0.81948782	-1.31156995
H	-1.05294211	-1.42800075	-1.71834512

Time = 263.50 fs
E(CASSCF/6-31G(d)) = -479.4720751

C	-0.73290748	-0.09356838	-0.08387858
C	0.49322818	-0.39849482	0.30076917
C	1.33150948	0.35246302	1.26841079
C	1.57384554	-0.88069540	-0.67753963
C	2.79452917	-0.14118374	-0.48177138
C	2.50205936	0.63041027	0.70246975
N	-1.83893709	0.13507323	0.86916334
C	-3.16367300	-0.38241787	0.28497957
H	3.16389630	1.39731920	1.13617740
C	-1.36753778	-0.42874867	-1.41175272
C	-2.94845748	-0.43961632	-1.23715308

H	-3.41450322	0.35255279	-1.82177266
H	-3.37746985	-1.36479219	-1.61655124
H	-3.22432946	-1.39589905	0.62321257
H	-3.99181867	0.17974183	0.68195436
H	-1.07045225	0.24692820	-2.20458053
H	0.78808957	0.87069141	1.99042573
C	-1.95573338	1.47441208	1.49796499
H	-1.03366086	2.01738699	1.38890109
H	-2.75621788	2.09058480	1.04253479
H	-2.20086791	1.29459143	2.55545599
H	1.25915322	-1.68883177	-1.32266206
C	4.11229582	-0.22318513	-1.14291709
H	4.80978032	-0.66065811	-0.44351237
H	4.09438968	-0.78996302	-2.06948085
H	4.44674984	0.81017518	-1.30006821
H	-1.03448553	-1.42288472	-1.70699544

Time = 264.00 fs
E(CASSCF/6-31G(d)) = -479.4687140

C	-0.73835533	-0.09035348	-0.08206118
C	0.49013099	-0.39169414	0.30326540
C	1.34132881	0.35817317	1.26752565
C	1.57407997	-0.87934518	-0.67358933
C	2.78649831	-0.14321521	-0.48468722
C	2.50476380	0.63218154	0.69958437
N	-1.84091762	0.12953233	0.87476429
C	-3.15938381	-0.38239465	0.28055633
H	3.15181179	1.39191404	1.12726249
C	-1.36037671	-0.42680215	-1.40737069
C	-2.94215283	-0.43884836	-1.24121435
H	-3.40766768	0.35653478	-1.82119922
H	-3.36479353	-1.36175847	-1.63585130
H	-3.23384158	-1.39403464	0.61723793
H	-3.99916491	0.17478731	0.66937134
H	-1.07098718	0.25241914	-2.20083598
H	0.81556925	0.85933080	1.98645769
C	-1.96593871	1.47194543	1.50175885
H	-1.04318524	2.01954340	1.40407482
H	-2.76615420	2.08111366	1.04155388
H	-2.23204513	1.29601045	2.54873978
H	1.25410541	-1.69223493	-1.30455202
C	4.11191839	-0.22921962	-1.15041405
H	4.80073485	-0.67567393	-0.44301847
H	4.10320586	-0.80782313	-2.07421617
H	4.45473285	0.79975853	-1.28948420
H	-1.01624256	-1.41738220	-1.69611941

Time = 264.50 fs
E(CASSCF/6-31G(d)) = -479.4646470

C	-0.74466455	-0.08725848	-0.08002502
C	0.48804033	-0.38496707	0.30555316
C	1.35061080	0.36368311	1.26648883
C	1.57433965	-0.87770073	-0.66961973
C	2.77773169	-0.14576755	-0.48771008
C	2.50789584	0.63427486	0.69690878
N	-1.84298534	0.12422590	0.88023497
C	-3.15458041	-0.38232318	0.27617715
H	3.14012084	1.38558135	1.11775076
C	-1.35340699	-0.42503987	-1.40311306
C	-2.93569233	-0.43808906	-1.24531237
H	-3.40044804	0.36089339	-1.81971239
H	-3.35178623	-1.35844786	-1.65512644
H	-3.24417330	-1.39323546	0.61203133
H	-4.00638764	0.17027038	0.65625677
H	-1.07121819	0.25810476	-2.19678259
H	0.84248036	0.85127389	1.98727271
C	-1.97627962	1.46929002	1.50549432
H	-1.05250472	2.02176317	1.41984279
H	-2.77505188	2.07094973	1.04099076
H	-2.26268402	1.29865606	2.54148491
H	1.25124268	-1.69510544	-1.28775846
C	4.11156030	-0.23519064	-1.15782745
H	4.79236770	-0.69113537	-0.44291035
H	4.11209552	-0.82486364	-2.07848648
H	4.46307973	0.78846868	-1.28007185
H	-0.99829663	-1.41159879	-1.68584362

Time = 265.00 fs
E(CASSCF/6-31G(d)) = -479.4601738

C	-0.75174436	-0.08427956	-0.07772336
C	0.48694837	-0.37830702	0.30763702
C	1.35934382	0.36890822	1.26522278
C	1.57456141	-0.87577313	-0.66562760
C	2.76834944	-0.14881016	-0.49087238
C	2.51141712	0.63661765	0.69437200
N	-1.84515089	0.11916851	0.88557151
C	-3.14933440	-0.38221895	0.27185127
H	3.12984659	1.37930357	1.10813949
C	-1.34665422	-0.42344095	-1.39901041
C	-2.92906429	-0.43734754	-1.24944675
H	-3.39281014	0.36566097	-1.81738324

H	-3.33852239	-1.35482052	-1.67417525
H	-3.25517117	-1.39353224	0.60756700
H	-4.01315070	0.16617646	0.64256095
H	-1.07101581	0.26391132	-2.19237304
H	0.86796222	0.84737739	1.99411659
C	-1.98673801	1.46644805	1.50914165
H	-1.06178720	2.02391444	1.43621370
H	-2.78298440	2.06028769	1.04060627
H	-2.29272924	1.30232425	2.53419332
H	1.25054418	-1.69764111	-1.27252121
C	4.11119198	-0.24115938	-1.16510215
H	4.78451194	-0.70690559	-0.44333175
H	4.12095159	-0.84076353	-2.08217057
H	4.47183526	0.77657604	-1.27205492
H	-0.98072042	-1.40565578	-1.67629198

Time = 265.50 fs
E(CASSCF/6-31G(d)) = -479.4558494

C	-0.75948509	-0.08141602	-0.07510719
C	0.48682231	-0.37170432	0.30952015
C	1.36749410	0.37380453	1.26369881
C	1.57467849	-0.87358145	-0.66161504
C	2.75848315	-0.15230129	-0.49420052
C	2.51527725	0.63912470	0.69189747
N	-1.84742512	0.11437291	0.89077028
C	-3.14372254	-0.38210758	0.26759049
H	3.12206314	1.37414820	1.09897262
C	-1.34014322	-0.42198380	-1.39509279
C	-2.92225666	-0.43663293	-1.25361536
H	-3.38473048	0.37087491	-1.81429748
H	-3.32508778	-1.35084679	-1.69280796
H	-3.26665888	-1.39482913	0.60377737
H	-4.01911364	0.16249068	0.62824424
H	-1.07025740	0.26976711	-2.18756086
H	0.89156981	0.84804153	2.00763083
C	-1.99728701	1.46342271	1.51267476
H	-1.07122075	2.02585158	1.45317928
H	-2.79008124	2.04936232	1.04012289
H	-2.32215292	1.30678926	2.52737414
H	1.25196192	-1.70001505	-1.25904005
C	4.11078425	-0.24718668	-1.17218309
H	4.77699090	-0.72283618	-0.44442511
H	4.12966606	-0.85525498	-2.08520690
H	4.48103774	0.76436687	-1.26561991
H	-0.96357739	-1.39968020	-1.66758094

Time = 266.00 fs
E(CASSCF/6-31G(d)) = -479.4521946

C	-0.76776091	-0.07867003	-0.07212693
C	0.48760733	-0.36514700	0.31120465
C	1.37499868	0.37837694	1.26195445
C	1.57461924	-0.87115443	-0.65759000
C	2.74827547	-0.15618741	-0.49771428
C	2.51942265	0.64170888	0.68940903
N	-1.84981895	0.10984996	0.89582816
C	-3.13782479	-0.38202432	0.26340793
H	3.11778417	1.37114045	1.09077134
C	-1.33389716	-0.42064719	-1.39138900
C	-2.91525745	-0.43595349	-1.25781432
H	-3.37619488	0.37657217	-1.81055131
H	-3.31158014	-1.34651110	-1.71085138
H	-3.27843850	-1.39691807	0.60055814
H	-4.02395453	0.15920747	0.61328538
H	-1.06882814	0.27560792	-2.18230708
H	0.91336140	0.85311514	2.02764739
C	-2.00789434	1.46021744	1.51607416
H	-1.08099502	2.02742645	1.47071249
H	-2.79651545	2.03843681	1.03923740
H	-2.35094631	1.31181605	2.52150043
H	1.25542550	-1.70236263	-1.24746555
C	4.11030864	-0.25332915	-1.17901685
H	4.76963516	-0.73877665	-0.44631642
H	4.13813144	-0.86813427	-2.08760140
H	4.49071034	0.75211679	-1.26091136
H	-0.94692411	-1.39379637	-1.65981566

Time = 266.50 fs
E(CASSCF/6-31G(d)) = -479.4494254

C	-0.77643435	-0.07604613	-0.06873475
C	0.48922927	-0.35862187	0.31269237
C	1.38177248	0.38267616	1.26009289
C	1.57430720	-0.86853150	-0.65356719
C	2.73787863	-0.16040413	-0.50142707
C	2.52380720	0.64429575	0.68684002
N	-1.85234297	0.10560897	0.90074268
C	-3.13172191	-0.38201220	0.25931649
H	3.11782616	1.37109798	1.08394426
C	-1.32793672	-0.41941143	-1.38792523
C	-2.90805512	-0.43531568	-1.26203816
H	-3.36719510	0.38278320	-1.80624630
H	-3.29810784	-1.34181492	-1.72815353

H	-3.29029210	-1.39950699	0.59777918
H	-4.02738978	0.15633782	0.59768790
H	-1.06662210	0.28137944	-2.17658158
H	0.93382816	0.86193594	2.05318637
C	-2.01852646	1.45683535	1.51932953
H	-1.09128316	2.02849681	1.48876742
H	-2.80248271	2.02778519	1.03763690
H	-2.37910793	1.31717092	2.51696103
H	1.26084555	-1.70476969	-1.23789225
C	4.10973784	-0.25963534	-1.18555310
H	4.76229550	-0.75458561	-0.44910104
H	4.14624209	-0.87926864	-2.08943013
H	4.50085147	0.74006438	-1.25802801
H	-0.93081284	-1.38811369	-1.65308424

Time = 267.00 fs
E(CASSCF/6-31G(d)) = -479.4474514

C	-0.78535935	-0.07355109	-0.06488666
C	0.49159876	-0.35211549	0.31398471
C	1.38772426	0.38678492	1.25826641
C	1.57365902	-0.86576355	-0.64956776
C	2.72745455	-0.16487560	-0.50534552
C	2.52840339	0.64683760	0.68414065
N	-1.85500747	0.10165797	0.90551270
C	-3.12549354	-0.38211921	0.25532746
H	3.12267234	1.37446703	1.07869973
C	-1.32227905	-0.41825966	-1.38472458
C	-2.90063917	-0.43472346	-1.26627997
H	-3.35772723	0.38952593	-1.80148546
H	-3.28478958	-1.33677869	-1.74458759
H	-3.30198849	-1.40225585	0.59529598
H	-4.02919087	0.15391387	0.58148390
H	-1.06354380	0.28704190	-2.17036837
H	0.95370572	0.87349153	2.08266015
C	-2.02915309	1.45327917	1.52244127
H	-1.10222569	2.02893695	1.50727963
H	-2.80818005	2.01767533	1.03502042
H	-2.40662987	1.32263251	2.51402226
H	1.26811760	-1.70726633	-1.23035609
C	4.10904632	-0.26614284	-1.19174574
H	4.75485502	-0.77013839	-0.45283471
H	4.15389468	-0.88860005	-2.09083799
H	4.51142601	0.72838837	-1.25702255
H	-0.91529565	-1.38271964	-1.64745607

Time = 267.50 fs

E(CASSCF/6-31G(d)) = -479.4521408

C	-0.79427158	-0.07122066	-0.06052370
C	0.49448338	-0.34566350	0.31504637
C	1.39266772	0.39077047	1.25668968
C	1.57254580	-0.86292840	-0.64564654
C	2.71727814	-0.16945860	-0.50942415
C	2.53335884	0.64934253	0.68121450
N	-1.85785972	0.09797796	0.91024401
C	-3.11919099	-0.38238364	0.25145606
H	3.13242228	1.38114491	1.07496904
C	-1.31693982	-0.41712818	-1.38183840
C	-2.89301832	-0.43417903	-1.27052118
H	-3.34777833	0.39679923	-1.79642258
H	-3.27175250	-1.33145993	-1.76011303
H	-3.31336916	-1.40483030	0.59290398
H	-4.02925352	0.15201004	0.56470190
H	-1.05960693	0.29249253	-2.16376751
H	0.97352914	0.88660673	2.11393597
C	-2.03976330	1.44954128	1.52534612
H	-1.11387593	2.02876759	1.52617070
H	-2.81377373	2.00862088	1.03123046
H	-2.43340156	1.32797828	2.51283100
H	1.27698395	-1.70995173	-1.22454870
C	4.10820532	-0.27285441	-1.19756094
H	4.74718731	-0.78533463	-0.45748212
H	4.16097827	-0.89613492	-2.09201443
H	4.52235388	0.71719118	-1.25788636
H	-0.90034679	-1.37770836	-1.64290310

Time = 268.00 fs
E(CASSCF/6-31G(d)) = -479.4540042

C	-0.80310594	-0.06904716	-0.05562104
C	0.49784711	-0.33921893	0.31591075
C	1.39666009	0.39476937	1.25547364
C	1.57094181	-0.86007200	-0.64182065
C	2.70742362	-0.17412190	-0.51368944
C	2.53857392	0.65181629	0.67812638
N	-1.86088454	0.09457166	0.91488070
C	-3.11289500	-0.38285895	0.24770164
H	3.14671534	1.39066021	1.07248544
C	-1.31192174	-0.41602595	-1.37925858
C	-2.88517302	-0.43367696	-1.27475999
H	-3.33734026	0.40458125	-1.79112187
H	-3.25910921	-1.32590317	-1.77464135
H	-3.32417307	-1.40689675	0.59049229

H	-4.02746222	0.15069030	0.54743067
H	-1.05467386	0.29776713	-2.15675608
H	0.99404091	0.90016674	2.14527586
C	-2.05034345	1.44563104	1.52810675
H	-1.12631124	2.02785493	1.54535854
H	-2.81939103	2.00066995	1.02599821
H	-2.45942261	1.33304212	2.51331674
H	1.28734672	-1.71269596	-1.22052708
C	4.10719383	-0.27979311	-1.20296832
H	4.73926659	-0.80011297	-0.46300885
H	4.16740938	-0.90197246	-2.09322790
H	4.53353169	0.70648705	-1.26059089
H	-0.88602239	-1.37308038	-1.63946973

Time = 268.50 fs
E(CASSCF/6-31G(d)) = -479.4549400

C	-0.81173743	-0.06703773	-0.05016223
C	0.50159944	-0.33276390	0.31659359
C	1.39971697	0.39888025	1.25474682
C	1.56878008	-0.85726018	-0.63812229
C	2.69803467	-0.17878842	-0.51814084
C	2.54404442	0.65430379	0.67491870
N	-1.86408137	0.09144578	0.91940435
C	-3.10667701	-0.38359150	0.24406455
H	3.16484372	1.40213495	1.07077268
C	-1.30722732	-0.41495393	-1.37698666
C	-2.87709734	-0.43321024	-1.27898759
H	-3.32641252	0.41281968	-1.78565830
H	-3.24699514	-1.32017836	-1.78811550
H	-3.33416447	-1.40817753	0.58795483
H	-4.02379785	0.15004797	0.52979330
H	-1.04866891	0.30289046	-2.14935840
H	1.01569207	0.91319519	2.17501134
C	-2.06088977	1.44155816	1.53076473
H	-1.13952606	2.02613019	1.56473312
H	-2.82508253	1.99390400	1.01917689
H	-2.48462240	1.33767173	2.51527497
H	1.29905519	-1.71536837	-1.21821646
C	4.10599434	-0.28695592	-1.20794028
H	4.73110688	-0.81443113	-0.46932193
H	4.17309811	-0.90625989	-2.09477722
H	4.54480232	0.69621856	-1.26506385
H	-0.87239825	-1.36881969	-1.63716631

Time = 269.00 fs
E(CASSCF/6-31G(d)) = -479.4551527

C	-0.82003655	-0.06520240	-0.04413970
C	0.50563645	-0.32628646	0.31711116
C	1.40187903	0.40318622	1.25461140
C	1.56599329	-0.85456317	-0.63458820
C	2.68925938	-0.18337245	-0.52276844
C	2.54978674	0.65686634	0.67163854
N	-1.86745207	0.08860421	0.92380672
C	-3.10059865	-0.38462120	0.24054056
H	3.18588135	1.41446347	1.06925143
C	-1.30285252	-0.41391201	-1.37502090
C	-2.86878868	-0.43276842	-1.28319439
H	-3.31499218	0.42143620	-1.78010042
H	-3.23554421	-1.31436802	-1.80051131
H	-3.34313818	-1.40846984	0.58520821
H	-4.01832536	0.15019526	0.51193180
H	-1.04154016	0.30789470	-2.14161719
H	1.03869704	0.92490475	2.20174569
C	-2.07141405	1.43733427	1.53335997
H	-1.15345413	2.02356574	1.58418043
H	-2.83081557	1.98834870	1.01072158
H	-2.50890766	1.34173516	2.51837948
H	1.31192237	-1.71781729	-1.21748428
C	4.10459207	-0.29432709	-1.21245854
H	4.72276101	-0.82828188	-0.47628172
H	4.17795920	-0.90919012	-2.09697697
H	4.55597221	0.68625910	-1.27119620
H	-0.85955151	-1.36488253	-1.63597361

Time = 269.50 fs
E(CASSCF/6-31G(d)) = -479.4547835

C	-0.82787756	-0.06355273	-0.03755646
C	0.50984938	-0.31977812	0.31748248
C	1.40321604	0.40775605	1.25513640
C	1.56252291	-0.85204999	-0.63125697
C	2.68123878	-0.18778728	-0.52755608
C	2.55581728	0.65956635	0.66833551
N	-1.87099839	0.08604838	0.92808427
C	-3.09471213	-0.38598000	0.23712080
H	3.20881747	1.42648469	1.06733181
C	-1.29878696	-0.41290033	-1.37335450
C	-2.86024791	-0.43233807	-1.28737035
H	-3.30307541	0.43032747	-1.77450789
H	-3.22488482	-1.30856081	-1.81183149
H	-3.35092468	-1.40765757	0.58219875
H	-4.01118629	0.15125666	0.49400287

H	-1.03325677	0.31281864	-2.13358686
H	1.06306621	0.93470206	2.22439702
C	-2.08194118	1.43297415	1.53592970
H	-1.16797257	2.02017020	1.60358621
H	-2.83647414	1.98396212	1.00069065
H	-2.53217654	1.34511762	2.52222097
H	1.32572684	-1.71987918	-1.21816267
C	4.10297484	-0.30188137	-1.21651500
H	4.71431931	-0.84169269	-0.48370813
H	4.18191126	-0.91098451	-2.10013271
H	4.56682100	0.67643352	-1.27884620
H	-0.84756009	-1.36120308	-1.63584770

Time = 270.00 fs
E(CASSCF/6-31G(d)) = -479.4540909

C	-0.83514276	-0.06210145	-0.03042642
C	0.51412934	-0.31323298	0.31773013
C	1.40382353	0.41264502	1.25635739
C	1.55832455	-0.84978404	-0.62816734
C	2.67410026	-0.19194930	-0.53248244
C	2.56214210	0.66245596	0.66505686
N	-1.87472117	0.08377731	0.93223657
C	-3.08906025	-0.38769061	0.23379211
H	3.23266837	1.43711852	1.06448335
C	-1.29501384	-0.41191875	-1.37197528
C	-2.85147957	-0.43190348	-1.29150519
H	-3.29065949	0.43936953	-1.76893331
H	-3.21513521	-1.30284573	-1.82210156
H	-3.35739722	-1.40571993	0.57890581
H	-4.00259108	0.15336243	0.47617219
H	-1.02380891	0.31770311	-2.12532614
H	1.08864406	0.94217645	2.24219740
C	-2.09250595	1.42849575	1.53850573
H	-1.18291030	2.01598438	1.62284007
H	-2.84186983	1.98063913	0.98924124
H	-2.55433353	1.34771875	2.52634922
H	1.34021183	-1.72139409	-1.22006479
C	4.10113284	-0.30958789	-1.22011257
H	4.70590445	-0.85472284	-0.49139127
H	4.18487593	-0.91187176	-2.10451299
H	4.57711343	0.66654161	-1.28784360
H	-0.83649974	-1.35770130	-1.63672341

Time = 270.50 fs
E(CASSCF/6-31G(d)) = -479.4534090

C	-0.84172460	-0.06086218	-0.02277400
C	0.51837025	-0.30664706	0.31788056
C	1.40381771	0.41789527	1.25827789
C	1.55337290	-0.84781872	-0.62535650
C	2.66795168	-0.19578191	-0.53752213
C	2.56875161	0.66557070	0.66184388
N	-1.87861978	0.08178792	0.93626541
C	-3.08367669	-0.38976536	0.23053758
H	3.25655309	1.44545226	1.06027779
C	-1.29151031	-0.41096630	-1.37086601
C	-2.84249203	-0.43144771	-1.29558867
H	-3.27774593	0.44842381	-1.76342336
H	-3.20639923	-1.29730475	-1.83136424
H	-3.36247763	-1.40273310	0.57534313
H	-3.99280427	0.15663887	0.45860717
H	-1.01320936	0.32258645	-2.11689280
H	1.11514583	0.94708396	2.25467854
C	-2.10314887	1.42391978	1.54111213
H	-1.19806044	2.01107509	1.64183916
H	-2.84676279	1.97822384	0.97661627
H	-2.57530520	1.34945004	2.53031458
H	1.35508701	-1.72221981	-1.22299781
C	4.09905844	-0.31741416	-1.22326600
H	4.69766316	-0.86745851	-0.49910409
H	4.18677641	-0.91206771	-2.11032072
H	4.58661335	0.65638256	-1.29799375
H	-0.82644048	-1.35429342	-1.63851904

Time = 271.00 fs
E(CASSCF/6-31G(d)) = -479.4530761

C	-0.84752751	-0.05984905	-0.01463337
C	0.52247145	-0.30001801	0.31796398
C	1.40332944	0.42353583	1.26087108
C	1.54766509	-0.84619382	-0.62285884
C	2.66287684	-0.19921853	-0.54264621
C	2.57561982	0.66892672	0.65872954
N	-1.88269193	0.08007482	0.94017424
C	-3.07858669	-0.39220513	0.22733732
H	3.27973534	1.45078280	1.05440784
C	-1.28824769	-0.41004023	-1.37000474
C	-2.83329776	-0.43095373	-1.29961068
H	-3.26434312	0.45734449	-1.75802174
H	-3.19876132	-1.29200583	-1.83967388
H	-3.36614047	-1.39886833	0.57155772
H	-3.98212662	0.16119634	0.44146939
H	-1.00149484	0.32749872	-2.10833832

H	1.14218672	0.94933578	2.26166084
C	-2.11391158	1.41926889	1.54376380
H	-1.21319506	2.00552775	1.66049019
H	-2.85088869	1.97652768	0.96312559
H	-2.59505221	1.35023348	2.53370352
H	1.37003126	-1.72224513	-1.22677405
C	4.09674616	-0.32532977	-1.22600215
H	4.68975617	-0.88000526	-0.50661799
H	4.18753588	-0.91175796	-2.11766777
H	4.59509754	0.64577714	-1.30908269
H	-0.81744186	-1.35090129	-1.64114018

Time = 271.50 fs
E(CASSCF/6-31G(d)) = -479.4533441

C	-0.85246894	-0.05907667	-0.00604699
C	0.52633989	-0.29334479	0.31801410
C	1.40249827	0.42958152	1.26408081
C	1.54122232	-0.84493283	-0.62070480
C	2.65893235	-0.20220479	-0.54782277
C	2.58270588	0.67252101	0.65573707
N	-1.88693336	0.07863059	0.94396778
C	-3.07380825	-0.39499861	0.22416932
H	3.30164290	1.45262938	1.04669048
C	-1.28519194	-0.40913540	-1.36936575
C	-2.82391340	-0.43040571	-1.30356149
H	-3.25046952	0.46598726	-1.75277118
H	-3.19228249	-1.28699600	-1.84709075
H	-3.36841576	-1.39438535	0.56762778
H	-3.97087240	0.16711536	0.42490697
H	-0.98872735	0.33245705	-2.09970296
H	1.16930497	0.94899479	2.26325533
C	-2.12483214	1.41456685	1.54646604
H	-1.22808290	1.99943658	1.67871150
H	-2.85398676	1.97534907	0.94912231
H	-2.61358018	1.35000125	2.53616636
H	1.38469858	-1.72139938	-1.23122013
C	4.09419276	-0.33330964	-1.22835934
H	4.68234503	-0.89247787	-0.51372042
H	4.18707685	-0.91108609	-2.12655725
H	4.60236858	0.63458578	-1.32088217
H	-0.80954883	-1.34746098	-1.64448395

Time = 272.00 fs
E(CASSCF/6-31G(d)) = -479.4542879

C	-0.85647987	-0.05856002	0.00293580

C	0.52989214	-0.28662784	0.31806741
C	1.40146631	0.43603150	1.26782138
C	1.53408883	-0.84404166	-0.61891994
C	2.65614730	-0.20469927	-0.55301730
C	2.58995761	0.67633280	0.65287917
N	-1.89133803	0.07744566	0.94765226
C	-3.06935355	-0.39812170	0.22101045
H	3.32187440	1.45072970	1.03706160
C	-1.28230400	-0.40824353	-1.36892045
C	-2.81435989	-0.42979013	-1.30743194
H	-3.23615610	0.47421789	-1.74771619
H	-3.18699636	-1.28229601	-1.85367573
H	-3.36938882	-1.38962140	0.56365786
H	-3.95934640	0.17443124	0.40904811
H	-0.97499637	0.33746103	-2.09101349
H	1.19597875	0.94628031	2.25987927
C	-2.13594092	1.40983774	1.54921458
H	-1.24250759	1.99289468	1.69643284
H	-2.85582556	1.97449332	0.93497963
H	-2.63094560	1.34869630	2.53743792
H	1.39872527	-1.71965902	-1.23618348
C	4.09139746	-0.34133632	-1.23038581
H	4.67557874	-0.90498852	-0.52023293
H	4.18532124	-0.91014977	-2.13687615
H	4.60826546	0.62272189	-1.33315551
H	-0.80278726	-1.34393033	-1.64844279

Time = 272.50 fs
E(CASSCF/6-31G(d)) = -479.4557475

C	-0.85950412	-0.05831465	0.01226039
C	0.53305664	-0.27987026	0.31816221
C	1.40036912	0.44286638	1.27197695
C	1.52632520	-0.84350963	-0.61752443
C	2.65452792	-0.20667182	-0.55819275
C	2.59731816	0.68032625	0.65015647
N	-1.89589914	0.07650844	0.95123749
C	-3.06523014	-0.40153738	0.21783762
H	3.34020235	1.44503140	1.02556804
C	-1.27954028	-0.40735212	-1.36863900
C	-2.80466250	-0.42909677	-1.31121344
H	-3.22144832	0.48192033	-1.74290460
H	-3.18290648	-1.27789726	-1.85948749
H	-3.36919821	-1.38497342	0.55977135
H	-3.94782141	0.18311944	0.39399475
H	-0.96042181	0.34248900	-2.08228494
H	1.22164418	0.94157923	2.25228339

C	-2.14725749	1.40510476	1.55199522
H	-1.25628484	1.98598582	1.71359633
H	-2.85622457	1.97379323	0.92106939
H	-2.64725970	1.34627307	2.53735092
H	1.41174045	-1.71705021	-1.24153394
C	4.08836223	-0.34940114	-1.23213732
H	4.66957888	-0.91763415	-0.52602782
H	4.18219171	-0.90900450	-2.14840017
H	4.61267159	0.61015922	-1.34566285
H	-0.79715914	-1.34029572	-1.65290780

Time = 273.00 fs
E(CASSCF/6-31G(d)) = -479.4572413

C	-0.86149229	-0.05835880	0.02186636
C	0.53577735	-0.27308338	0.31833504
C	1.39931114	0.45004144	1.27640490
C	1.51797870	-0.84331683	-0.61653164
C	2.65408540	-0.20809272	-0.56331138
C	2.60474789	0.68445695	0.64755256
N	-1.90061534	0.07580598	0.95474986
C	-3.06143974	-0.40519551	0.21462974
H	3.35657266	1.43568327	1.01234866
C	-1.27685175	-0.40644038	-1.36849385
C	-2.79485088	-0.42831955	-1.31489797
H	-3.20640594	0.48900474	-1.73839220
H	-3.17998440	-1.27376157	-1.86458422
H	-3.36803296	-1.38087780	0.55609451
H	-3.93652652	0.19308454	0.37981631
H	-0.94517289	0.34748803	-2.07353449
H	1.24571450	0.93545587	2.24157524
C	-2.15879015	1.40038741	1.55477805
H	-1.26927512	1.97878345	1.73015641
H	-2.85507487	1.97314489	0.90775131
H	-2.66268724	1.34270059	2.53584876
H	1.42338284	-1.71365315	-1.24715024
C	4.08509159	-0.35750401	-1.23367442
H	4.66442708	-0.93048626	-0.53103834
H	4.17761242	-0.90767345	-2.16080791
H	4.61551874	0.59693436	-1.35816472
H	-0.79263681	-1.33658206	-1.65777004

Time = 273.50 fs
E(CASSCF/6-31G(d)) = -479.4541771

C	-0.86248091	-0.05867161	0.03171247
C	0.53803588	-0.26621723	0.31864828

C	1.39849568	0.45753592	1.28094249
C	1.50922027	-0.84341942	-0.61594539
C	2.65467312	-0.20899948	-0.56836402
C	2.61212174	0.68866853	0.64505779
N	-1.90547432	0.07531626	0.95817034
C	-3.05798360	-0.40906089	0.21135249
H	3.37116132	1.42300540	0.99764709
C	-1.27417662	-0.40551063	-1.36844259
C	-2.78491992	-0.42745164	-1.31849199
H	-3.19105585	0.49543189	-1.73420800
H	-3.17816447	-1.26980618	-1.86900511
H	-3.36608568	-1.37774450	0.55277241
H	-3.92555209	0.20417593	0.36653580
H	-0.92927781	0.35246800	-2.06468865
H	1.26768950	0.92863089	2.22922316
C	-2.17056490	1.39569753	1.55757333
H	-1.28144872	1.97127131	1.74612286
H	-2.85229893	1.97235528	0.89524570
H	-2.67751366	1.33794198	2.53294953
H	1.43329397	-1.70949246	-1.25307057
C	4.08158648	-0.36569115	-1.23505720
H	4.66015412	-0.94360906	-0.53532017
H	4.17150441	-0.90615872	-2.17376748
H	4.61679784	0.58310282	-1.37050091
H	-0.78925055	-1.33279847	-1.66298602

Time = 274.00 fs
E(CASSCF/6-31G(d)) = -479.4576661

C	-0.86245949	-0.05926103	0.04170285
C	0.53978239	-0.25929505	0.31914225
C	1.39805697	0.46525089	1.28536282
C	1.50022458	-0.84375722	-0.61577442
C	2.65619266	-0.20940861	-0.57329185
C	2.61931470	0.69286726	0.64265929
N	-1.91043879	0.07502223	0.96148429
C	-3.05488209	-0.41305591	0.20800076
H	3.38422670	1.40757162	0.98186998
C	-1.27147202	-0.40455180	-1.36845038
C	-2.77494522	-0.42649732	-1.32197446
H	-3.17554966	0.50114648	-1.73042697
H	-3.17736174	-1.26595468	-1.87276487
H	-3.36359302	-1.37598398	0.54994079
H	-3.91503001	0.21608814	0.35423142
H	-0.91296447	0.35734994	-2.05575768
H	1.28692731	0.92202233	2.21711009
C	-2.18251084	1.39107508	1.56034410

H	-1.29274161	1.96348309	1.76139936
H	-2.84793347	1.97136795	0.88385280
H	-2.69194727	1.33200630	2.52880001
H	1.44108010	-1.70472849	-1.25923601
C	4.07787123	-0.37394787	-1.23633848
H	4.65676392	-0.95694302	-0.53893497
H	4.16383738	-0.90447165	-2.18680675
H	4.61654320	0.56887542	-1.38241631
H	-0.78695712	-1.32902240	-1.66844695

Time = 274.50 fs
E(CASSCF/6-31G(d)) = -479.4602685

C	-0.86143830	-0.06013371	0.05178048
C	0.54098992	-0.25231543	0.31985522
C	1.39810894	0.47310486	1.28944248
C	1.49113945	-0.84426101	-0.61601939
C	2.65852800	-0.20935012	-0.57805486
C	2.62622490	0.69696610	0.64032590
N	-1.91548679	0.07490286	0.96468798
C	-3.05214268	-0.41711546	0.20456140
H	3.39626818	1.39007428	0.96545100
C	-1.26868349	-0.40354934	-1.36848230
C	-2.76496828	-0.42546111	-1.32533830
H	-3.15999587	0.50615401	-1.72711012
H	-3.17745426	-1.26209946	-1.87589185
H	-3.36078430	-1.37591527	0.54769182
H	-3.90499589	0.22848218	0.34290766
H	-0.89641389	0.36206620	-2.04671514
H	1.30294880	0.91660349	2.20727286
C	-2.19458661	1.38653992	1.56306210
H	-1.30319654	1.95539942	1.77596131
H	-2.84208322	1.97017455	0.87376068
H	-2.70629339	1.32490198	2.52362779
H	1.44642020	-1.69953031	-1.26563138
C	4.07395986	-0.38229547	-1.23757134
H	4.65419416	-0.97043728	-0.54202730
H	4.15457692	-0.90262966	-2.19949696
H	4.61484747	0.55445936	-1.39372300
H	-0.78567534	-1.32534144	-1.67407217

Time = 275.00 fs
E(CASSCF/6-31G(d)) = -479.4619301

C	-0.85944319	-0.06129412	0.06189132
C	0.54164561	-0.24527877	0.32082125
C	1.39874564	0.48101722	1.29296709

C	1.48211264	-0.84485610	-0.61667604
C	2.66155014	-0.20886121	-0.58261343
C	2.63274732	0.70087233	0.63801999
N	-1.92059357	0.07493547	0.96777493
C	-3.04977364	-0.42117746	0.20102639
H	3.40794820	1.37133028	0.94888518
C	-1.26575599	-0.40248992	-1.36850392
C	-2.75503358	-0.42435009	-1.32857633
H	-3.14451458	0.51049316	-1.72431884
H	-3.17829549	-1.25812525	-1.87841284
H	-3.35786843	-1.37774046	0.54607218
H	-3.89544627	0.24096043	0.33255173
H	-0.87981418	0.36654682	-2.03753591
H	1.31538738	0.91329793	2.20168753
C	-2.20673810	1.38210916	1.56569922
H	-1.31290905	1.94698076	1.78979520
H	-2.83492412	1.96881750	0.86508825
H	-2.72088384	1.31665332	2.51773058
H	1.44903222	-1.69407674	-1.27225847
C	4.06986839	-0.39075796	-1.23880164
H	4.65234079	-0.98400612	-0.54479401
H	4.14370801	-0.90067189	-2.21143430
H	4.61184848	0.54009725	-1.40425695
H	-0.78530480	-1.32185987	-1.67978972

Time = 275.50 fs
E(CASSCF/6-31G(d)) = -479.4630328

C	-0.85651580	-0.06274420	0.07198501
C	0.54175139	-0.23818783	0.32206886
C	1.40003884	0.48892322	1.29576290
C	1.47328640	-0.84546566	-0.61773559
C	2.66512192	-0.20798445	-0.58692925
C	2.63877791	0.70449285	0.63570060
N	-1.92573192	0.07509597	0.97073589
C	-3.04778346	-0.42518728	0.19739358
H	3.42004371	1.35222070	0.93269817
C	-1.26263484	-0.40136269	-1.36848143
C	-2.74518781	-0.42317275	-1.33168099
H	-3.12923382	0.51423215	-1.72211220
H	-3.17971958	-1.25391861	-1.88035709
H	-3.35501765	-1.38151544	0.54507142
H	-3.88635816	0.25308817	0.32313792
H	-0.86335408	0.37072588	-2.02820145
H	1.32401684	0.91280941	2.20190701
C	-2.21890142	1.37779634	1.56822985
H	-1.32201614	1.93817504	1.80290146

H	-2.82669578	1.96738834	0.85788187
H	-2.73606014	1.30730106	2.51144882
H	1.44867985	-1.68854249	-1.27912898
C	4.06561416	-0.39935935	-1.24006704
H	4.65106803	-0.99753892	-0.54746684
H	4.13123673	-0.89866529	-2.22226522
H	4.60772120	0.52605135	-1.41388050
H	-0.78572998	-1.31869035	-1.68553572

Time = 276.00 fs
E(CASSCF/6-31G(d)) = -479.4642167

C	-0.85271340	-0.06448321	0.08201527
C	0.54132448	-0.23104815	0.32362005
C	1.40203707	0.49678775	1.29772798
C	1.46479269	-0.84601371	-0.61918491
C	2.66910267	-0.20676608	-0.59096631
C	2.64422084	0.70774169	0.63332732
N	-1.93087260	0.07535907	0.97355894
C	-3.04617982	-0.42910124	0.19366696
H	3.43335887	1.33360353	0.91740422
C	-1.25926673	-0.40016043	-1.36838185
C	-2.73547908	-0.42193785	-1.33464425
H	-3.11428599	0.51746378	-1.72054429
H	-3.18154805	-1.24937809	-1.88176131
H	-3.35235726	-1.38713758	0.54461671
H	-3.87770966	0.26441941	0.31463168
H	-0.84721569	0.37454863	-2.01870586
H	1.32876307	0.91546332	2.20870037
C	-2.23100669	1.37361062	1.57063337
H	-1.33068281	1.92892673	1.81529779
H	-2.81769072	1.96602368	0.85211597
H	-2.75215252	1.29690323	2.50513021
H	1.44517799	-1.68308423	-1.28625627
C	4.06121539	-0.40812090	-1.24139675
H	4.65022063	-1.01091246	-0.55029328
H	4.11719105	-0.89670640	-2.23170537
H	4.60266712	0.51258456	-1.42248334
H	-0.78682456	-1.31594569	-1.69125332

Time = 276.50 fs
E(CASSCF/6-31G(d)) = -479.4659925

C	-0.84810872	-0.06650767	0.09194002
C	0.54039784	-0.22386817	0.32548986
C	1.40476623	0.50461202	1.29885005
C	1.45674996	-0.84642748	-0.62100716

C	2.67335164	-0.20525497	-0.59469178
C	2.64899853	0.71054873	0.63086502
N	-1.93598483	0.07569858	0.97622980
C	-3.04496823	-0.43288832	0.18985633
H	3.44860156	1.31620972	0.90345590
C	-1.25560089	-0.39888064	-1.36817323
C	-2.72595585	-0.42065314	-1.33745750
H	-3.09980273	0.52029739	-1.71966072
H	-3.18359637	-1.24442287	-1.88267347
H	-3.34996212	-1.39435289	0.54457449
H	-3.86949764	0.27452230	0.30699271
H	-0.83156779	0.37797666	-2.00905985
H	1.32969927	0.92113132	2.22184473
C	-2.24298256	1.36955614	1.57289685
H	-1.33908705	1.91918612	1.82702256
H	-2.80824102	1.96489855	0.84769782
H	-2.76945540	1.28553643	2.49908910
H	1.43839635	-1.67782788	-1.29364630
C	4.05669065	-0.41705843	-1.24281186
H	4.64963659	-1.02400410	-0.55351707
H	4.10162060	-0.89492016	-2.23955311
H	4.59690155	0.49994015	-1.42998210
H	-0.78845676	-1.31372875	-1.69689057

Time = 277.00 fs
E(CASSCF/6-31G(d)) = -479.4684227

C	-0.84278939	-0.06881136	0.10172110
C	0.53902033	-0.21665918	0.32768665
C	1.40823082	0.51242965	1.29920208
C	1.44926073	-0.84663935	-0.62318238
C	2.67773121	-0.20350166	-0.59807678
C	2.65306324	0.71286823	0.62828820
N	-1.94103693	0.07608774	0.97873218
C	-3.04415053	-0.43653032	0.18597639
H	3.46624301	1.30054259	0.89119766
C	-1.25159024	-0.39752644	-1.36782480
C	-2.71666597	-0.41932410	-1.34011159
H	-3.08590981	0.52284996	-1.71949540
H	-3.18568065	-1.23900023	-1.88315603
H	-3.34786013	-1.40278224	0.54475983
H	-3.86175130	0.28300303	0.30017749
H	-0.81655952	0.38099229	-1.99929317
H	1.32703112	0.92927937	2.24017705
C	-2.25476086	1.36563175	1.57501734
H	-1.34740491	1.90891779	1.83813743
H	-2.79870226	1.96421639	0.84447703

H	-2.78820197	1.27329755	2.49356543
H	1.42826198	-1.67285715	-1.30128957
C	4.05205861	-0.42617983	-1.24432615
H	4.64915938	-1.03670524	-0.55736052
H	4.08459504	-0.89345629	-2.24569773
H	4.59064044	0.48832158	-1.43631917
H	-0.79049466	-1.31212273	-1.70239795

Time = 277.50 fs
E(CASSCF/6-31G(d)) = -479.4711062

C	-0.83685716	-0.07138542	0.11132402
C	0.53725670	-0.20943477	0.33021267
C	1.41241549	0.52029412	1.29891814
C	1.44241030	-0.84658826	-0.62568808
C	2.68210936	-0.20155760	-0.60109693
C	2.65640653	0.71468403	0.62558377
N	-1.94599709	0.07649960	0.98104832
C	-3.04372349	-0.44002042	0.18204543
H	3.48639897	1.28681295	0.88083486
C	-1.24719261	-0.39610715	-1.36730718
C	-2.70765554	-0.41795289	-1.34259699
H	-3.07272206	0.52523633	-1.72006715
H	-3.18762423	-1.23309112	-1.88328827
H	-3.34604115	-1.41196116	0.54495142
H	-3.85454066	0.28952620	0.29414130
H	-0.80231488	0.38360182	-1.98945639
H	1.32107840	0.93911417	2.26187571
C	-2.26628125	1.36183097	1.57700318
H	-1.35579755	1.89810744	1.84872901
H	-2.78943478	1.96419502	0.84226115
H	-2.80854080	1.26030566	2.48868969
H	1.41476116	-1.66820481	-1.30915341
C	4.04733779	-0.43548314	-1.24594695
H	4.64864824	-1.04893187	-0.56201029
H	4.06620229	-0.89248367	-2.25012227
H	4.58408695	0.47787337	-1.44146068
H	-0.79281082	-1.31118309	-1.70772619

Time = 278.00 fs
E(CASSCF/6-31G(d)) = -479.4734385

C	-0.83042665	-0.07421868	0.12071745
C	0.53518714	-0.20221010	0.33306515
C	1.41728674	0.52826380	1.29816123
C	1.43626628	-0.84622053	-0.62849992
C	2.68636162	-0.19947449	-0.60373278

C	2.65906350	0.71600986	0.62275145
N	-1.95083413	0.07690749	0.98315954
C	-3.04367758	-0.44336095	0.17808373
H	3.50877428	1.27493216	0.87242811
C	-1.24237199	-0.39463846	-1.36659261
C	-2.69896780	-0.41653753	-1.34490407
H	-3.06033861	0.52755987	-1.72137682
H	-3.18926352	-1.22671348	-1.88316716
H	-3.34446865	-1.42138612	0.54490959
H	-3.84798027	0.29383055	0.28883978
H	-0.78892842	0.38583678	-1.97962044
H	1.31225294	0.94975878	2.28483548
C	-2.27749516	1.35814258	1.57887385
H	-1.36440021	1.88676681	1.85890951
H	-2.78078338	1.96504920	0.84083631
H	-2.83051916	1.24670324	2.48446115
H	1.39794030	-1.66384720	-1.31717526
C	4.04254637	-0.44495525	-1.24767623
H	4.64798664	-1.06063338	-0.56760646
H	4.04654591	-0.89218352	-2.25290185
H	4.57742012	0.46866615	-1.44539462
H	-0.79528629	-1.31093117	-1.71282403

Time = 278.50 fs
E(CASSCF/6-31G(d)) = -479.4749109

C	-0.82362416	-0.07729767	0.12987274
C	0.53290697	-0.19500124	0.33623756
C	1.42279533	0.53638985	1.29709498
C	1.43087880	-0.84549047	-0.63159239
C	2.69037258	-0.19730403	-0.60597004
C	2.66110931	0.71688432	0.61980231
N	-1.95551830	0.07728551	0.98504686
C	-3.04399620	-0.44656074	0.17411191
H	3.53269267	1.26456613	0.86591300
C	-1.23709975	-0.39314237	-1.36565520
C	-2.69064218	-0.41507129	-1.34702331
H	-3.04883865	0.52990342	-1.72340513
H	-3.19045244	-1.21992292	-1.88290634
H	-3.34309113	-1.43056009	0.54439466
H	-3.84222850	0.29573923	0.28423027
H	-0.77646207	0.38775341	-1.96987495
H	1.30103365	0.96039482	2.30699400
C	-2.28836885	1.35455168	1.58065798
H	-1.37331411	1.87493649	1.86881559
H	-2.77305692	1.96696991	0.83999233
H	-2.85407537	1.23265630	2.48074460

H	1.37790667	-1.65970072	-1.32525954
C	4.03770186	-0.45457133	-1.24951159
H	4.64708818	-1.07179848	-0.57423560
H	4.02574247	-0.89274158	-2.25419717
H	4.57078641	0.46068772	-1.44812967
H	-0.79781317	-1.31135068	-1.71763710

Time = 279.00 fs
E(CASSCF/6-31G(d)) = -479.4752570

C	-0.81658582	-0.08060714	0.13876334
C	0.53052554	-0.18782446	0.33972064
C	1.42887840	0.54470928	1.29586533
C	1.42628094	-0.84436018	-0.63493927
C	2.69403725	-0.19509728	-0.60779970
C	2.66265004	0.71736245	0.61675533
N	-1.96002209	0.07760894	0.98669162
C	-3.04465543	-0.44963258	0.17014956
H	3.55719996	1.25522904	0.86113598
C	-1.23135572	-0.39164638	-1.36447108
C	-2.68271322	-0.41354262	-1.34894579
H	-3.03827864	0.53232372	-1.72611170
H	-3.19106605	-1.21281119	-1.88263461
H	-3.34185294	-1.43903268	0.54318347
H	-3.83748384	0.29516604	0.28027304
H	-0.76494408	0.38943003	-1.96032489
H	1.28794081	0.97034553	2.32654506
C	-2.29888482	1.35104117	1.58238973
H	-1.38260168	1.86268663	1.87860664
H	-2.76650980	1.97010338	0.83954882
H	-2.87904336	1.21835176	2.47728606
H	1.35482895	-1.65562479	-1.33327551
C	4.03282066	-0.46429540	-1.25144736
H	4.64590066	-1.08245832	-0.58192698
H	4.00391838	-0.89433933	-2.25424306
H	4.56429595	0.45384133	-1.44969498
H	-0.80029581	-1.31238745	-1.72210721

Time = 279.50 fs
E(CASSCF/6-31G(d)) = -479.4744976

C	-0.80945433	-0.08413063	0.14736468
C	0.52816337	-0.18069490	0.34350295
C	1.43546245	0.55324262	1.29459179
C	1.42248916	-0.84280014	-0.63851368
C	2.69726296	-0.19290392	-0.60921794
C	2.66380953	0.71750655	0.61363364

N	-1.96432114	0.07785478	0.98807621
C	-3.04562430	-0.45259118	0.16621388
H	3.58120495	1.24638420	0.85789568
C	-1.22512905	-0.39018242	-1.36301858
C	-2.67520991	-0.41193540	-1.35066348
H	-3.02869057	0.53484774	-1.72943525
H	-3.19100271	-1.20550184	-1.88249226
H	-3.34070175	-1.44643147	0.54108316
H	-3.83397771	0.29211705	0.27693235
H	-0.75437043	0.39096333	-1.95108662
H	1.27351273	0.97910586	2.34203721
C	-2.30904188	1.34759361	1.58410420
H	-1.39228468	1.85011583	1.88845943
H	-2.76132800	1.97453067	0.83937913
H	-2.90516614	1.20399275	2.47374498
H	1.32893927	-1.65142794	-1.34106302
C	4.02791716	-0.47408166	-1.25347583
H	4.64440624	-1.09268620	-0.59065247
H	3.98120739	-0.89714482	-2.25332955
H	4.55802194	0.44795211	-1.45014151
H	-0.80265071	-1.31395239	-1.72617342

Time = 280.00 fs
E(CASSCF/6-31G(d)) = -479.4728468

C	-0.80237673	-0.08785074	0.15565369
C	0.52595039	-0.17362664	0.34757188
C	1.44246541	0.56199436	1.29336537
C	1.41950447	-0.84078789	-0.64228868
C	2.69996950	-0.19077229	-0.61022601
C	2.66471728	0.71737872	0.61046175
N	-1.96839477	0.07800213	0.98918438
C	-3.04686550	-0.45545109	0.16231895
H	3.60361482	1.23752682	0.85597504
C	-1.21841892	-0.38878529	-1.36127848
C	-2.66815495	-0.41022973	-1.35216970
H	-3.02008180	0.53747235	-1.73329504
H	-3.19018369	-1.19814365	-1.88262613
H	-3.33959392	-1.45248447	0.53794064
H	-3.83196391	0.28668497	0.27417695
H	-0.74470702	0.39246083	-1.94228091
H	1.25828535	0.98634182	2.35239551
C	-2.31885370	1.34419302	1.58583275
H	-1.40234680	1.83734730	1.89856496
H	-2.75762047	1.98025430	0.83942699
H	-2.93211959	1.18979034	2.46973467
H	1.30053129	-1.64687667	-1.34843429

C	4.02300321	-0.48387685	-1.25558859
H	4.64262133	-1.10259593	-0.60032942
H	3.95774793	-0.90130206	-2.25178018
H	4.55200561	0.44278025	-1.44954324
H	-0.80480515	-1.31592754	-1.72977308

Time = 280.50 fs
E(CASSCF/6-31G(d)) = -479.4706620

C	-0.79550101	-0.09174946	0.16360874
C	0.52402284	-0.16663219	0.35191394
C	1.44979890	0.57095489	1.29224969
C	1.41731326	-0.83830730	-0.64623719
C	2.70209000	-0.18874910	-0.61083017
C	2.66549844	0.71703580	0.60726366
N	-1.97222649	0.07803259	0.99000161
C	-3.04833668	-0.45822488	0.15847511
H	3.62344188	1.22823759	0.85516482
C	-1.21123490	-0.38749074	-1.35923429
C	-2.66156446	-0.40840313	-1.35345955
H	-3.01243659	0.54016745	-1.73759397
H	-3.18855141	-1.19090204	-1.88318350
H	-3.33849731	-1.45703473	0.53364939
H	-3.83170493	0.27903909	0.27198038
H	-0.73589398	0.39403263	-1.93402579
H	1.24277654	0.99187489	2.35690587
C	-2.32834598	1.34082639	1.58759916
H	-1.41274028	1.82452285	1.90912245
H	-2.75541746	1.98719172	0.83971357
H	-2.95953942	1.17595367	2.46486451
H	1.26995876	-1.64170840	-1.35518327
C	4.01808730	-0.49362323	-1.25777799
H	4.64059288	-1.11233639	-0.61082713
H	3.93368168	-0.90692027	-2.24992383
H	4.54626324	0.43803945	-1.44799895
H	-0.80669514	-1.31817487	-1.73284482

Time = 281.00 fs
E(CASSCF/6-31G(d)) = -479.4683496

C	-0.78897055	-0.09580921	0.17120983
C	0.52251735	-0.15972161	0.35651435
C	1.45737158	0.58010253	1.29128287
C	1.41588834	-0.83534817	-0.65033130
C	2.70357189	-0.18687868	-0.61104140
C	2.66626637	0.71652647	0.60406159
N	-1.97580460	0.07793057	0.99051568

C	-3.04999226	-0.46092183	0.15468858
H	3.63987332	1.21820978	0.85527803
C	-1.20359682	-0.38633345	-1.35687233
C	-2.65544790	-0.40643196	-1.35453021
H	-3.00571766	0.54288092	-1.74222157
H	-3.18606647	-1.18394786	-1.88430477
H	-3.33739221	-1.46004759	0.52815301
H	-3.83345453	0.26941047	0.27031959
H	-0.72785351	0.39578233	-1.92642888
H	1.22747277	0.99566433	2.35518923
C	-2.33755302	1.33748480	1.58941733
H	-1.42339384	1.81179671	1.92033013
H	-2.75467687	1.99517775	0.84033215
H	-2.98704724	1.16268098	2.45877903
H	1.23763399	-1.63564822	-1.36109902
C	4.01317357	-0.50326200	-1.26003857
H	4.63839109	-1.12208270	-0.62197569
H	3.90915461	-0.91406431	-2.24806323
H	4.54079407	0.43341922	-1.44563374
H	-0.80826350	-1.32054539	-1.73533089

Time = 281.50 fs
E(CASSCF/6-31G(d)) = -479.4662737

C	-0.78291986	-0.10001285	0.17843894
C	0.52156640	-0.15290265	0.36135700
C	1.46509155	0.58940524	1.29047895
C	1.41519006	-0.83190462	-0.65454215
C	2.70437706	-0.18520297	-0.61087513
C	2.66711852	0.71589019	0.60087575
N	-1.97912239	0.07768359	0.99071668
C	-3.05178555	-0.46354603	0.15096150
H	3.65230920	1.20725759	0.85615621
C	-1.19553433	-0.38534482	-1.35418210
C	-2.64980821	-0.40429312	-1.35538140
H	-2.99987029	0.54554672	-1.74705941
H	-3.18270293	-1.17744658	-1.88611605
H	-3.33627096	-1.46161296	0.52144647
H	-3.83743716	0.25807001	0.26917389
H	-0.72049650	0.39779565	-1.91957903
H	1.21281605	0.99779478	2.34718299
C	-2.34651358	1.33416371	1.59129051
H	-1.43422658	1.79932569	1.93237911
H	-2.75529396	2.00397713	0.84143326
H	-3.01427719	1.15014868	2.45118697
H	1.20402076	-1.62842592	-1.36597659
C	4.00826138	-0.51273712	-1.26236837

H	4.63610052	-1.13202512	-0.63357846
H	3.88431805	-0.92274563	-2.24644607
H	4.53559097	0.42860787	-1.44259922
H	-0.80945751	-1.32289081	-1.73718074

Time = 282.00 fs
E(CASSCF/6-31G(d)) = -479.4647045

C	-0.77746983	-0.10434393	0.18528028
C	0.52129321	-0.14618096	0.36642426
C	1.47286918	0.59882174	1.28982804
C	1.41516772	-0.82797408	-0.65883941
C	2.70448181	-0.18376135	-0.61035112
C	2.66813398	0.71515743	0.59772458
N	-1.98217813	0.07728229	0.99059719
C	-3.05367134	-0.46609474	0.14729221
H	3.66037939	1.19531138	0.85767253
C	-1.18708621	-0.38455092	-1.35115650
C	-2.64464225	-0.40196599	-1.35601576
H	-2.99482614	0.54809291	-1.75198608
H	-3.17844278	-1.17154622	-1.88872099
H	-3.33513636	-1.46194124	0.51357476
H	-3.84382443	0.24530369	0.26852232
H	-0.71373022	0.40013069	-1.91353889
H	1.19918804	0.99847046	2.33313499
C	-2.35526668	1.33086295	1.59321228
H	-1.44516287	1.78725949	1.94544350
H	-2.75711557	2.01330290	0.84320264
H	-3.04089637	1.13850346	2.44188160
H	1.16962669	-1.61979142	-1.36963076
C	4.00334525	-0.52199873	-1.26476954
H	4.63380778	-1.14235528	-0.64542685
H	3.85933199	-0.93291623	-2.24524081
H	4.53064752	0.42331300	-1.43907160
H	-0.81022717	-1.32507394	-1.73835445

Time = 282.50 fs
E(CASSCF/6-31G(d)) = -479.4637642

C	-0.77272212	-0.10878701	0.19172096
C	0.52180517	-0.13956009	0.37169632
C	1.48062085	0.60830194	1.28929522
C	1.41576096	-0.82355620	-0.66319067
C	2.70387704	-0.18259029	-0.60949297
C	2.66937175	0.71434986	0.59462477
N	-1.98497505	0.07672059	0.99015238
C	-3.05560863	-0.46855733	0.14367574

H	3.66395132	1.18241164	0.85973425
C	-1.17829924	-0.38397067	-1.34779209
C	-2.63994138	-0.39943400	-1.35643907
H	-2.99050742	0.55045059	-1.75688226
H	-3.17327070	-1.16636630	-1.89219357
H	-3.33399911	-1.46135321	0.50462902
H	-3.85271091	0.23138653	0.26834035
H	-0.70746597	0.40280905	-1.90833869
H	1.18688980	0.99801585	2.31361333
C	-2.36384773	1.32758628	1.59516831
H	-1.45614770	1.77573071	1.95967124
H	-2.75995512	2.02283902	0.84583549
H	-3.06662218	1.12785532	2.43075280
H	1.13499221	-1.60953192	-1.37190907
C	3.99841495	-0.53100634	-1.26724842
H	4.63158731	-1.15325026	-0.65731597
H	3.83436818	-0.94446698	-2.24452202
H	4.52596571	0.41728065	-1.43524898
H	-0.81052404	-1.32697830	-1.73882499

Time = 283.00 fs
E(CASSCF/6-31G(d)) = -479.4633893

C	-0.76875502	-0.11332734	0.19775143
C	0.52318925	-0.13304206	0.37715058
C	1.48827228	0.61778692	1.28881861
C	1.41690107	-0.81865194	-0.66756082
C	2.70256821	-0.18172282	-0.60832780
C	2.67086960	0.71348088	0.59159133
N	-1.98752107	0.07599550	0.98937990
C	-3.05756326	-0.47091464	0.14010472
H	3.66313366	1.16870081	0.86228569
C	-1.16922706	-0.38361465	-1.34408908
C	-2.63569218	-0.39668624	-1.35666053
H	-2.98683113	0.55256217	-1.76163602
H	-3.16716945	-1.16198862	-1.89657167
H	-3.33287331	-1.46026240	0.49473943
H	-3.86409183	0.21655838	0.26859640
H	-0.70162465	0.40580982	-1.90397090
H	1.17611668	0.99688102	2.28952565
C	-2.37228511	1.32434063	1.59713887
H	-1.46716082	1.76484568	1.97517631
H	-2.76360600	2.03226444	0.84951139
H	-3.09123635	1.11827244	2.41779130
H	1.10067681	-1.59748672	-1.37270427
C	3.99345618	-0.53973130	-1.26981445
H	4.62948718	-1.16485688	-0.66906087

H	3.80961219	-0.95723023	-2.24426861
H	4.52156136	0.41030983	-1.43134707
H	-0.81030162	-1.32851603	-1.73858117

Time = 283.50 fs
E(CASSCF/6-31G(d)) = -479.4633150

C	-0.76561938	-0.11795075	0.20336627
C	0.52550680	-0.12662786	0.38276119
C	1.49576268	0.62720880	1.28830758
C	1.41851261	-0.81326278	-0.67191175
C	2.70057517	-0.18118809	-0.60688569
C	2.67264291	0.71255587	0.58863738
N	-1.98982841	0.07510708	0.98827990
C	-3.05951023	-0.47313992	0.13657031
H	3.65828154	1.15441775	0.86531254
C	-1.15992891	-0.38348426	-1.34005137
C	-2.63187707	-0.39371842	-1.35669279
H	-2.98371284	0.55438828	-1.76614728
H	-3.16011718	-1.15845084	-1.90185293
H	-3.33177113	-1.45914838	0.48406506
H	-3.87784735	0.20100676	0.26924880
H	-0.69614046	0.40906731	-1.90038894
H	1.16692795	0.99564764	2.26213763
C	-2.38059759	1.32113534	1.59910133
H	-1.47822738	1.75467721	1.99203096
H	-2.76785401	2.04127482	0.85437287
H	-3.11459426	1.10977787	2.40308770
H	1.06724355	-1.58355858	-1.37196378
C	3.98845157	-0.54815847	-1.27247856
H	4.62751565	-1.17727665	-0.68051000
H	3.78526330	-0.97098779	-2.24437441
H	4.51746772	0.40226312	-1.42759383
H	-0.80951548	-1.32963383	-1.73762867

Time = 284.00 fs
E(CASSCF/6-31G(d)) = -479.4631111

C	-0.76333612	-0.12264322	0.20856464
C	0.52879025	-0.12031822	0.38849867
C	1.50304806	0.63649115	1.28764295
C	1.42051497	-0.80739015	-0.67620211
C	2.69793199	-0.18101093	-0.60519909
C	2.67468387	0.71157242	0.58577358
N	-1.99191315	0.07405832	0.98685495
C	-3.06143530	-0.47520116	0.13306357
H	3.64999989	1.13989228	0.86884734

C	-1.15046837	-0.38357170	-1.33568641
C	-2.62847493	-0.39053356	-1.35655192
H	-2.98107018	0.55591340	-1.77033167
H	-3.15208705	-1.15574365	-1.90799308
H	-3.33069601	-1.45852050	0.47278072
H	-3.89373634	0.18485938	0.27024397
H	-0.69096167	0.41247372	-1.89750885
H	1.15921940	0.99502751	2.23306876
C	-2.38879265	1.31798121	1.60103249
H	-1.48942344	1.74526020	2.01026003
H	-2.77248721	2.04960107	0.86050916
H	-3.13663082	1.10234813	2.38682670
H	1.03524203	-1.56772295	-1.36969736
C	3.98338180	-0.55628679	-1.27525107
H	4.62563150	-1.19055416	-0.69155473
H	3.76153191	-0.98548285	-2.24466908
H	4.51373675	0.39307217	-1.42422315
H	-0.80812416	-1.33031628	-1.73599035

Time = 284.50 fs
E(CASSCF/6-31G(d)) = -479.4622881

C	-0.76189579	-0.12739044	0.21335066
C	0.53304143	-0.11411423	0.39432976
C	1.51010277	0.64555079	1.28668198
C	1.42282389	-0.80103526	-0.68038716
C	2.69468671	-0.18121111	-0.60330234
C	2.67696179	0.71052105	0.58300731
N	-1.99379467	0.07285492	0.98511002
C	-3.06333556	-0.47706503	0.12957663
H	3.63913988	1.12553670	0.87297526
C	-1.14091230	-0.38386047	-1.33100492
C	-2.62546152	-0.38714210	-1.35625711
H	-2.97882538	0.55714884	-1.77412367
H	-3.14304884	-1.15381165	-1.91490671
H	-3.32963427	-1.45887299	0.46106112
H	-3.91140179	0.16818655	0.27151627
H	-0.68604964	0.41588489	-1.89521347
H	1.15271195	0.99584254	2.20423455
C	-2.39686586	1.31488959	1.60291055
H	-1.50087434	1.73659063	2.02983868
H	-2.77730378	2.05702492	0.86794454
H	-3.15736339	1.09591292	2.36927765
H	1.00519075	-1.55003365	-1.36598249
C	3.97822688	-0.56412899	-1.27813956
H	4.62374015	-1.20467138	-0.70213324
H	3.73863317	-1.00043501	-2.24494593

H	4.51043916	0.38273953	-1.42146875
H	-0.80609108	-1.33058645	-1.73370534

Time = 285.00 fs
E(CASSCF/6-31G(d)) = -479.4604753

C	-0.76125945	-0.13217734	0.21773362
C	0.53823161	-0.10801812	0.40021764
C	1.51691730	0.65430245	1.28527105
C	1.42535295	-0.79419885	-0.68441903
C	2.69090102	-0.18180293	-0.60123090
C	2.67942527	0.70938602	0.58034187
N	-1.99549503	0.07150513	0.98305231
C	-3.06521911	-0.47870203	0.12610396
H	3.62677704	1.11183202	0.87783573
C	-1.13132966	-0.38432666	-1.32602046
C	-2.62280984	-0.38356145	-1.35583021
H	-2.97690730	0.55813454	-1.77747832
H	-3.13297327	-1.15255763	-1.92247137
H	-3.32854698	-1.46063540	0.44906459
H	-3.93038827	0.15101350	0.27298980
H	-0.68137515	0.41913104	-1.89335958
H	1.14697788	0.99897209	2.17769897
C	-2.40480134	1.31187124	1.60471686
H	-1.51274763	1.72862762	2.05069235
H	-2.78211772	2.06338978	0.87663225
H	-3.17688958	1.09035742	2.35078051
H	0.97755903	-1.53062604	-1.36096670
C	3.97296726	-0.57171013	-1.28114726
H	4.62169601	-1.21954746	-0.71222847
H	3.71677886	-1.01555618	-2.24499123
H	4.50766179	0.37133465	-1.41955729
H	-0.80338634	-1.33050339	-1.73082685

Time = 285.50 fs
E(CASSCF/6-31G(d)) = -479.4576234

C	-0.76136131	-0.13698725	0.22172803
C	0.54430336	-0.10203408	0.40612199
C	1.52349120	0.66266668	1.28326758
C	1.42801476	-0.78688148	-0.68824691
C	2.68665011	-0.18279437	-0.59902084
C	2.68200709	0.70814733	0.57777603
N	-1.99703834	0.07001949	0.98069127
C	-3.06710382	-0.48009185	0.12264327
H	3.61415800	1.09930167	0.88361665
C	-1.12179070	-0.38494076	-1.32074908

C	-2.62049035	-0.37981518	-1.35529520
H	-2.97525271	0.55893816	-1.78037245
H	-3.12183770	-1.15185029	-1.93053377
H	-3.32736290	-1.46412457	0.43691842
H	-3.95016903	0.13333841	0.27458200
H	-0.67691368	0.42202957	-1.89178658
H	1.14152385	1.00525996	2.15542058
C	-2.41257328	1.30893525	1.60643732
H	-1.52523998	1.72129839	2.07270067
H	-2.78676272	2.06860859	0.88645280
H	-3.19537970	1.08552682	2.33172703
H	0.95275039	-1.50971727	-1.35486836
C	3.96758457	-0.57906537	-1.28427186
H	4.61931128	-1.23504497	-0.72186084
H	3.69616765	-1.03056625	-2.24461191
H	4.50550312	0.35898552	-1.41870199
H	-0.79998901	-1.33015669	-1.72741930

Time = 286.00 fs
E(CASSCF/6-31G(d)) = -479.4540826

C	-0.76211349	-0.14180211	0.22535286
C	0.55117417	-0.09616800	0.41200065
C	1.52981990	0.67058075	1.28056833
C	1.43072285	-0.77908345	-0.69181818
C	2.68202122	-0.18418726	-0.59670785
C	2.68463389	0.70678400	0.57530488
N	-1.99845014	0.06841072	0.97803841
C	-3.06901490	-0.48122817	0.11919588
H	3.60260118	1.08846963	0.89053274
C	-1.11236568	-0.38566981	-1.31520882
C	-2.61847114	-0.37593173	-1.35467737
H	-2.97380646	0.55965253	-1.78280370
H	-3.10963277	-1.15153393	-1.93891883
H	-3.32597473	-1.46950671	0.42470906
H	-3.97017875	0.11515251	0.27620820
H	-0.67263997	0.42439941	-1.89032590
H	1.13593460	1.01538578	2.13891909
C	-2.42014841	1.30608757	1.60806369
H	-1.53856022	1.71450530	2.09570333
H	-2.79109566	2.07266787	0.89721565
H	-3.21306244	1.08123327	2.31253798
H	0.93108542	-1.48760200	-1.34797282
C	3.96206250	-0.58623689	-1.28750543
H	4.61637065	-1.25098229	-0.73107628
H	3.67697503	-1.04520741	-2.24365866
H	4.50406658	0.34586568	-1.41909597

H	-0.79588950	-1.32965853	-1.72355351

Time = 286.50 fs
E(CASSCF/6-31G(d)) = -479.4504967

C	-0.76341015	-0.14660153	0.22863172
C	0.55874014	-0.09042863	0.41780916
C	1.53588134	0.67800969	1.27713613
C	1.43339199	-0.77080573	-0.69507852
C	2.67711406	-0.18597614	-0.59432700
C	2.68723835	0.70527858	0.57292195
N	-1.99975667	0.06669338	0.97510723
C	-3.07098244	-0.48212179	0.11576661
H	3.59335331	1.07980255	0.89878754
C	-1.10312427	-0.38647988	-1.30941932
C	-2.61671824	-0.37194293	-1.35400265
H	-2.97252128	0.56039047	-1.78478953
H	-3.09636884	-1.15143930	-1.94743887
H	-3.32423999	-1.47677636	0.41247863
H	-3.98984795	0.09645825	0.27778702
H	-0.66852377	0.42607513	-1.88881126
H	1.13003345	1.02973643	2.12896943
C	-2.42748939	1.30332987	1.60959475
H	-1.55291164	1.70813363	2.11950973
H	-2.79499671	2.07562831	0.90866579
H	-3.23020296	1.07726622	2.29363404
H	0.91279107	-1.46464812	-1.34062935
C	3.95638696	-0.59327047	-1.29083452
H	4.61264776	-1.26714774	-0.73993390
H	3.65934409	-1.05925608	-2.24204251
H	4.50345171	0.33217621	-1.42090696
H	-0.79109225	-1.32913287	-1.71930200

Time = 287.00 fs
E(CASSCF/6-31G(d)) = -479.4474290

C	-0.76513445	-0.15136328	0.23159178
C	0.56688201	-0.08482646	0.42350297
C	1.54162600	0.68495286	1.27301204
C	1.43594005	-0.76204988	-0.69797332
C	2.67203817	-0.18814903	-0.59191188
C	2.68977431	0.70362274	0.57062391
N	-2.00098407	0.06488359	0.97191276
C	-3.07303795	-0.48280139	0.11236293
H	3.58741646	1.07364260	0.90851606
C	-1.09413449	-0.38733856	-1.30340171
C	-2.61519568	-0.36788242	-1.35329687

H	-2.97135666	0.56127850	-1.78636375
H	-3.08208194	-1.15139503	-1.95590439
H	-3.32198866	-1.48575738	0.40022961
H	-4.00863486	0.07728444	0.27924487
H	-0.66452468	0.42691927	-1.88708669
H	1.12399581	1.04832229	2.12544451
C	-2.43455872	1.30065821	1.61103748
H	-1.56847398	1.70206020	2.14390677
H	-2.79837186	2.07762058	0.92049261
H	-3.24707329	1.07340537	2.27540695
H	0.89799043	-1.44128502	-1.33324049
C	3.95054676	-0.60021175	-1.29424104
H	4.60792412	-1.28331656	-0.74849168
H	3.64337809	-1.07253260	-2.23974678
H	4.50374336	0.31812621	-1.42427099
H	-0.78561732	-1.32870356	-1.71473199

Time = 287.50 fs
E(CASSCF/6-31G(d)) = -479.4451011

C	-0.76716304	-0.15606258	0.23426358
C	0.57546859	-0.07937436	0.42903736
C	1.54697878	0.69144478	1.26830927
C	1.43828848	-0.75281914	-0.70044875
C	2.66691199	-0.19068692	-0.58949376
C	2.69222898	0.70182141	0.56841564
N	-2.00215746	0.06299861	0.96847108
C	-3.07521148	-0.48331232	0.10899357
H	3.58538713	1.07014961	0.91972339
C	-1.08546189	-0.38821747	-1.29717812
C	-2.61386614	-0.36378370	-1.35258505
H	-2.97027672	0.56244764	-1.78757291
H	-3.06683760	-1.15123810	-1.96413406
H	-3.31903383	-1.49612542	0.38793462
H	-4.02605378	0.05769454	0.28052080
H	-0.66058994	0.42683288	-1.88501355
H	1.11834933	1.07077566	2.12738902
C	-2.44132313	1.29806224	1.61240792
H	-1.58538780	1.69616073	2.16867195
H	-2.80114949	2.07883691	0.93234343
H	-3.26391979	1.06943507	2.25818719
H	0.88670207	-1.41799067	-1.32625034
C	3.94453403	-0.60710241	-1.29770342
H	4.60200520	-1.29926824	-0.75679577
H	3.62913355	-1.08490785	-2.23683153
H	4.50500032	0.30390955	-1.42928863
H	-0.77950352	-1.32848196	-1.70990149

Time = 288.00 fs
E(CASSCF/6-31G(d)) = -479.4433471

C	-0.76937228	-0.16067229	0.23668004
C	0.58436142	-0.07408707	0.43436906
C	1.55185167	0.69754820	1.26318860
C	1.44036478	-0.74311839	-0.70245341
C	2.66185854	-0.19356497	-0.58710071
C	2.69462769	0.69989507	0.56631575
N	-2.00329965	0.06105666	0.96479816
C	-3.07752842	-0.48371342	0.10566688
H	3.58736378	1.06926841	0.93223854
C	-1.07716907	-0.38909436	-1.29077148
C	-2.61269133	-0.35967844	-1.35189081
H	-2.96924745	0.56402346	-1.78847103
H	-3.05073309	-1.15082314	-1.97196362
H	-3.31518619	-1.50744633	0.37555108
H	-4.04169695	0.03779145	0.28157023
H	-0.65665134	0.42576310	-1.88247534
H	1.11386402	1.09643152	2.13328055
C	-2.44775768	1.29552484	1.61373162
H	-1.60374177	1.69031602	2.19358286
H	-2.80327805	2.07951613	0.94383996
H	-3.28092910	1.06515890	2.24221778
H	0.87884429	-1.39527305	-1.32012689
C	3.93834486	-0.61397686	-1.30119788
H	4.59473533	-1.31480126	-0.76487071
H	3.61661655	-1.09630828	-2.23343517
H	4.50724461	0.28968458	-1.43602120
H	-0.77280867	-1.32855452	-1.70485217

Time = 288.50 fs
E(CASSCF/6-31G(d)) = -479.4420108

C	-0.77164392	-0.16516214	0.23887618
C	0.59341584	-0.06898030	0.43945686
C	1.55616578	0.70334459	1.25782739
C	1.44210848	-0.73295429	-0.70394095
C	2.65699757	-0.19675472	-0.58475610
C	2.69702658	0.69787815	0.56436089
N	-2.00442921	0.05907651	0.96090747
C	-3.08000713	-0.48407298	0.10238920
H	3.59295385	1.07074195	0.94570151
C	-1.06931508	-0.38995561	-1.28420473
C	-2.61163262	-0.35559464	-1.35123575
H	-2.96823438	0.56611619	-1.78911349

H	-3.03389773	-1.15002995	-1.97925265
H	-3.31026783	-1.51922147	0.36303688
H	-4.05524982	0.01771772	0.28236854
H	-0.65262135	0.42370967	-1.87937838
H	1.11139059	1.12444578	2.14135838
C	-2.45384920	1.29302234	1.61504394
H	-1.62356477	1.68441599	2.21842513
H	-2.80471935	2.07991915	0.95459771
H	-3.29819894	1.06041321	2.22763457
H	0.87424965	-1.37364730	-1.31533847
C	3.93197996	-0.62085953	-1.30470010
H	4.58600791	-1.32974534	-0.77271333
H	3.60578198	-1.10671712	-2.22976971
H	4.51045178	0.27555655	-1.44448851
H	-0.76561032	-1.32897281	-1.69960595

Time = 289.00 fs
E(CASSCF/6-31G(d)) = -479.4438781

C	-0.77378623	-0.16953913	0.24089245
C	0.60250115	-0.06410384	0.44424404
C	1.55963844	0.70889325	1.25250202
C	1.44324982	-0.72237461	-0.70485333
C	2.65265557	-0.20014120	-0.58248773
C	2.69970940	0.69584764	0.56247776
N	-2.00562730	0.05708614	0.95690217
C	-3.08263056	-0.48445463	0.09917283
H	3.60138938	1.07397334	0.95962473
C	-1.06194043	-0.39077006	-1.27752789
C	-2.61065319	-0.35156096	-1.35063971
H	-2.96719042	0.56882585	-1.78956684
H	-3.01649276	-1.14877527	-1.98595772
H	-3.30418014	-1.53097548	0.35029679
H	-4.06655449	-0.00233939	0.28286865
H	-0.64858341	0.42064974	-1.87577408
H	1.11174557	1.15395032	2.14981751
C	-2.45960104	1.29052044	1.61632108
H	-1.64480258	1.67838586	2.24305832
H	-2.80550710	2.08047922	0.96437985
H	-3.31572592	1.05507943	2.21449486
H	0.87270034	-1.35355971	-1.31234005
C	3.92544310	-0.62776883	-1.30817551
H	4.57579722	-1.34399234	-0.78030726
H	3.59651400	-1.11616022	-2.22608488
H	4.51452500	0.26157022	-1.45462787
H	-0.75803489	-1.32979563	-1.69416918

Time = 289.50 fs
E(CASSCF/6-31G(d)) = -479.4443174

C	-0.77572779	-0.17375928	0.24276236
C	0.61147040	-0.05946298	0.44870364
C	1.56230973	0.71429269	1.24732511
C	1.44381399	-0.71138619	-0.70516543
C	2.64887873	-0.20371930	-0.58031292
C	2.70269984	0.69384879	0.56077279
N	-2.00688725	0.05509847	0.95277218
C	-3.08540198	-0.48493469	0.09601534
H	3.61166740	1.07849913	0.97339058
C	-1.05509865	-0.39154570	-1.27075704
C	-2.60971555	-0.34759287	-1.35011966
H	-2.96607276	0.57220113	-1.78987252
H	-2.99870375	-1.14700426	-1.99199091
H	-3.29679836	-1.54221180	0.33733360
H	-4.07550675	-0.02216971	0.28309809
H	-0.64436530	0.41671128	-1.87161191
H	1.11553196	1.18406812	2.15716844
C	-2.46502836	1.28798908	1.61762458
H	-1.66736280	1.67214185	2.26729855
H	-2.80559670	2.08139431	0.97283792
H	-3.33339415	1.04908625	2.20270593
H	0.87392851	-1.33545272	-1.31151039
C	3.91874570	-0.63470439	-1.31160628
H	4.56410094	-1.35745055	-0.78758045
H	3.58867498	-1.12472751	-2.22270981
H	4.51933682	0.24769813	-1.46638229
H	-0.75018665	-1.33098521	-1.68851902

Time = 290.00 fs
E(CASSCF/6-31G(d)) = -479.4439253

C	-0.77738232	-0.17779306	0.24451906
C	0.62018136	-0.05507056	0.45280701
C	1.56418072	0.71962645	1.24241719
C	1.44376351	-0.70001668	-0.70486063
C	2.64577223	-0.20745289	-0.57824508
C	2.70608206	0.69193461	0.55931462
N	-2.00821978	0.05313230	0.94853493
C	-3.08830503	-0.48558442	0.09291238
H	3.62272844	1.08378590	0.98635949
C	-1.04883317	-0.39229239	-1.26391633
C	-2.60878406	-0.34370071	-1.34969012
H	-2.96482950	0.57625695	-1.79006333
H	-2.98074210	-1.14469957	-1.99732093

H	-3.28805359	-1.55250231	0.32415845
H	-4.08213548	-0.04153317	0.28309575
H	-0.63984104	0.41204582	-1.86690566
H	1.12316416	1.21401135	2.16219771
C	-2.47016203	1.28539552	1.61899574
H	-1.69108508	1.66562181	2.29100451
H	-2.80495528	2.08285508	0.97973253
H	-3.35099503	1.04241360	2.19209327
H	0.87767719	-1.31966184	-1.31314204
C	3.91190402	-0.64165761	-1.31497494
H	4.55098363	-1.37008340	-0.79443942
H	3.58208220	-1.13254439	-2.21999505
H	4.52470242	0.23385437	-1.47963975
H	-0.74220262	-1.33248072	-1.68261864

Time = 290.50 fs
E(CASSCF/6-31G(d)) = -479.4433658

C	-0.77867124	-0.18161466	0.24619522
C	0.62849961	-0.05094065	0.45652806
C	1.56526903	0.72496831	1.23787544
C	1.44305042	-0.68830515	-0.70393560
C	2.64344493	-0.21129783	-0.57629230
C	2.70993694	0.69015400	0.55816171
N	-2.00963643	0.05120705	0.94421407
C	-3.09130902	-0.48646877	0.08985551
H	3.63358000	1.08931847	0.99793042
C	-1.04318072	-0.39302473	-1.25703215
C	-2.60782550	-0.33988838	-1.34936306
H	-2.96340070	0.58096924	-1.79015940
H	-2.96283757	-1.14188027	-2.00196078
H	-3.27792640	-1.56150544	0.31080548
H	-4.08659269	-0.06016974	0.28291919
H	-0.63489601	0.40683270	-1.86170207
H	1.13485326	1.24310606	2.16399432
C	-2.47504553	1.28270876	1.62046652
H	-1.71575276	1.65878260	2.31407152
H	-2.80354140	2.08498444	0.98492433
H	-3.36825334	1.03508739	2.18241849
H	0.88373577	-1.30639447	-1.31740101
C	3.90493807	-0.64861177	-1.31826883
H	4.53656777	-1.38190610	-0.80076811
H	3.57652308	-1.13976205	-2.21829149
H	4.53039433	0.21990629	-1.49424483
H	-0.73423613	-1.33419664	-1.67641736

Time = 291.00 fs

E(CASSCF/6-31G(d)) = -479.4431978

C	-0.77952712	-0.18519760	0.24782134
C	0.63629328	-0.04708957	0.45984193
C	1.56561643	0.73037807	1.23377256
C	1.44162808	-0.67630988	-0.70240401
C	2.64199737	-0.21520435	-0.57445881
C	2.71432250	0.68854878	0.55736497
N	-2.01114569	0.04934323	0.93983909
C	-3.09436795	-0.48764358	0.08683441
H	3.64337446	1.09464182	1.00757805
C	-1.03817550	-0.39376065	-1.25013684
C	-2.60681011	-0.33615614	-1.34914803
H	-2.96172095	0.58627273	-1.79016726
H	-2.94524422	-1.13860078	-2.00596060
H	-3.26645295	-1.56897523	0.29734352
H	-4.08914264	-0.07779595	0.28264492
H	-0.62943019	0.40127319	-1.85607359
H	1.15060953	1.27078102	2.16194207
C	-2.47972779	1.27990413	1.62205747
H	-1.74108570	1.65160287	2.33641328
H	-2.80130047	2.08781277	0.98837845
H	-3.38484800	1.02717697	2.17341130
H	0.89195877	-1.29571620	-1.32429538
C	3.89787545	-0.65553990	-1.32148007
H	4.52103792	-1.39297333	-0.80642921
H	3.57177278	-1.14653698	-2.21792149
H	4.53615273	0.20570357	-1.50999329
H	-0.72645595	-1.33603102	-1.66986164

Time = 291.50 fs
E(CASSCF/6-31G(d)) = -479.4438517

C	-0.77989761	-0.18852264	0.24942888
C	0.64345176	-0.04352559	0.46273371
C	1.56529061	0.73591227	1.23014719
C	1.43945578	-0.66408119	-0.70029511
C	2.64150934	-0.21912832	-0.57274076
C	2.71927663	0.68714787	0.55696461
N	-2.01275527	0.04755772	0.93543477
C	-3.09742880	-0.48915602	0.08383059
H	3.65147731	1.09939687	1.01490432
C	-1.03383733	-0.39452096	-1.24325334
C	-2.60570984	-0.33249304	-1.34905166
H	-2.95971818	0.59207668	-1.79008085
H	-2.92819428	-1.13493878	-2.00941254
H	-3.25369947	-1.57478741	0.28384526

H	-4.09015465	-0.09415548	0.28236484
H	-0.62335177	0.39556423	-1.85009705
H	1.17029147	1.29663634	2.15570035
C	-2.48426967	1.27695908	1.62377960
H	-1.76680747	1.64406545	2.35800787
H	-2.79816170	2.09127603	0.99017424
H	-3.40048288	1.01876891	2.16477541
H	0.90228918	-1.28751532	-1.33367334
C	3.89073449	-0.66242353	-1.32461441
H	4.50459869	-1.40339903	-0.81128520
H	3.56759728	-1.15302899	-2.21914503
H	4.54171538	0.19106948	-1.52667208
H	-0.71902188	-1.33788035	-1.66288679

Time = 292.00 fs
E(CASSCF/6-31G(d)) = -479.4455866

C	-0.77974355	-0.19156974	0.25104660
C	0.64986891	-0.04025656	0.46519156
C	1.56438142	0.74161116	1.22701392
C	1.43650740	-0.65168590	-0.69765620
C	2.64203834	-0.22302428	-0.57113059
C	2.72480619	0.68597000	0.55699126
N	-2.01446843	0.04586754	0.93103010
C	-3.10042724	-0.49104081	0.08082487
H	3.65746198	1.10332039	1.01962872
C	-1.03018319	-0.39532553	-1.23641015
C	-2.60450071	-0.32888655	-1.34907845
H	-2.95732020	0.59826157	-1.78988406
H	-2.91192951	-1.13099397	-2.01243447
H	-3.23978210	-1.57892484	0.27041345
H	-4.09007168	-0.10899299	0.28218177
H	-0.61659561	0.38989437	-1.84385519
H	1.19359411	1.32038878	2.14519153
C	-2.48872917	1.27385997	1.62563078
H	-1.79260476	1.63616954	2.37884271
H	-2.79404653	2.09521684	0.99048687
H	-3.41488163	1.00997377	2.15623098
H	0.91472996	-1.28153631	-1.34521427
C	3.88353844	-0.66924133	-1.32768546
H	4.48749578	-1.41332145	-0.81519556
H	3.56377859	-1.15936902	-2.22213204
H	4.54682256	0.17585217	-1.54403332
H	-0.71209550	-1.33964796	-1.65543812

Time = 292.50 fs
E(CASSCF/6-31G(d)) = -479.4484467

C	-0.77903950	-0.19432049	0.25270133
C	0.65544923	-0.03728587	0.46721045
C	1.56299826	0.74750063	1.22436053
C	1.43277750	-0.63919589	-0.69455037
C	2.64361323	-0.22684960	-0.56961593
C	2.73089055	0.68502213	0.55746439
N	-2.01628479	0.04428826	0.92665469
C	-3.10329341	-0.49331968	0.07779616
H	3.66112164	1.10625483	1.02160192
C	-1.02722309	-0.39619176	-1.22963631
C	-2.60316232	-0.32532130	-1.34923094
H	-2.95445739	0.60469266	-1.78955411
H	-2.89668068	-1.12687602	-2.01516431
H	-3.22485426	-1.58148320	0.25716752
H	-4.08937907	-0.12208249	0.28220209
H	-0.60912583	0.38442497	-1.83742265
H	1.22007472	1.34190646	2.13059739
C	-2.49316071	1.27059996	1.62759778
H	-1.81816080	1.62792140	2.39892229
H	-2.78887557	2.09940292	0.98957338
H	-3.42782523	1.00091105	2.14752515
H	0.92931967	-1.27739480	-1.35845312
C	3.87630954	-0.67597781	-1.33071745
H	4.46999060	-1.42289793	-0.81803358
H	3.56012254	-1.16565076	-2.22693800
H	4.55123736	0.15993167	-1.56181378
H	-0.70582706	-1.34125652	-1.64747217

Time = 293.00 fs
E(CASSCF/6-31G(d)) = -479.4522646

C	-0.77777405	-0.19675851	0.25441776
C	0.66010903	-0.03461127	0.46879309
C	1.56126672	0.75359005	1.22214757
C	1.42828761	-0.62668489	-0.69105360
C	2.64623087	-0.23056509	-0.56818007
C	2.73748301	0.68429929	0.55839157
N	-2.01820006	0.04283370	0.92233843
C	-3.10595619	-0.49600034	0.07472277
H	3.66246724	1.10815059	1.02080773
C	-1.02495993	-0.39713270	-1.22296130
C	-2.60167791	-0.32178094	-1.34951030
H	-2.95106641	0.61123062	-1.78906539
H	-2.88266014	-1.12269310	-2.01774977
H	-3.20910106	-1.58266627	0.24423883
H	-4.08856065	-0.13324430	0.28252604

H	-0.60094187	0.37927718	-1.83085714
H	1.24916586	1.36122755	2.11236369
C	-2.49761138	1.26717829	1.62965832
H	-1.84316990	1.61932957	2.41825824
H	-2.78257873	2.10355343	0.98774959
H	-3.43917155	0.99170064	2.13844490
H	0.94609188	-1.27461288	-1.37281425
C	3.86906861	-0.68262635	-1.33374454
H	4.45234495	-1.43228975	-0.81969960
H	3.55646537	-1.17192323	-2.23349224
H	4.55476046	0.14323386	-1.57974399
H	-0.70034849	-1.34265714	-1.63896181

Time = 293.50 fs
E(CASSCF/6-31G(d)) = -479.4566461

C	-0.77594897	-0.19886885	0.25621839
C	0.66377648	-0.03222425	0.46995082
C	1.55932428	0.75986931	1.22030865
C	1.42309078	-0.61422561	-0.68725173
C	2.64985439	-0.23413497	-0.56680187
C	2.74451295	0.68378458	0.55976761
N	-2.02020626	0.04151587	0.91811074
C	-3.10834827	-0.49907561	0.07158392
H	3.66172612	1.10907034	1.01736272
C	-1.02338944	-0.39815596	-1.21641499
C	-2.60003437	-0.31825027	-1.34991671
H	-2.94709432	0.61774259	-1.78839312
H	-2.87005568	-1.11853962	-2.02033629
H	-3.19273242	-1.58277495	0.23176630
H	-4.08804766	-0.14236215	0.28323692
H	-0.59208354	0.37452202	-1.82419261
H	1.28019272	1.37858541	2.09120851
C	-2.50211787	1.26359883	1.63178374
H	-1.86735142	1.61040027	2.43685777
H	-2.77510467	2.10736577	0.98536466
H	-3.44886716	0.98245555	2.12882468
H	0.96503449	-1.27266298	-1.38765683
C	3.86183564	-0.68919117	-1.33680908
H	4.43480500	-1.44164465	-0.82013414
H	3.55267606	-1.17819068	-2.24159946
H	4.55724201	0.12573967	-1.59755758
H	-0.69576852	-1.34383536	-1.62990035

Time = 294.00 fs
E(CASSCF/6-31G(d)) = -479.4610132

C	-0.77357899	-0.20063842	0.25812317
C	0.66639271	-0.03010930	0.47070417
C	1.55731512	0.76630687	1.21875147
C	1.41727287	-0.60188717	-0.68323683
C	2.65441419	-0.23752642	-0.56545613
C	2.75188824	0.68344928	0.56157385
N	-2.02229164	0.04034473	0.91400058
C	-3.11041089	-0.50252341	0.06836124
H	3.65933742	1.10919043	1.01151440
C	-1.02250025	-0.39926284	-1.21002738
C	-2.59822222	-0.31471708	-1.35044999
H	-2.94250174	0.62411267	-1.78751732
H	-2.85902520	-1.11448556	-2.02305695
H	-3.17597667	-1.58219310	0.21989052
H	-4.08817098	-0.14939774	0.28438878
H	-0.58263192	0.37017680	-1.81743733
H	1.31239862	1.39442742	2.06811772
C	-2.50670425	1.25986883	1.63394180
H	-1.89045946	1.60113424	2.45471513
H	-2.76642959	2.11054558	0.98277633
H	-3.45695487	0.97327688	2.11855220
H	0.98605783	-1.27100946	-1.40231953
C	3.85463042	-0.69568849	-1.33995867
H	4.41758680	-1.45108018	-0.81932979
H	3.54865269	-1.18441890	-2.25095457
H	4.55858962	0.10748951	-1.61500004
H	-0.69216693	-1.34481572	-1.62030404

Time = 294.50 fs
E(CASSCF/6-31G(d)) = -479.4647583

C	-0.77069140	-0.20205557	0.26014911
C	0.66791237	-0.02824373	0.47108294
C	1.55538468	0.77284938	1.21736136
C	1.41095201	-0.58973328	-0.67910386
C	2.65981001	-0.24070872	-0.56411376
C	2.75949785	0.68325307	0.56377815
N	-2.02444098	0.03932816	0.91003565
C	-3.11209818	-0.50630776	0.06504023
H	3.65594440	1.10880176	1.00363516
C	-1.02227408	-0.40044864	-1.20382795
C	-2.59623549	-0.31117381	-1.35111010
H	-2.93726540	0.63024994	-1.78642491
H	-2.84969274	-1.11056632	-2.02602163
H	-3.15907269	-1.58136325	0.20874848
H	-4.08911983	-0.15440089	0.28599690
H	-0.57270900	0.36620560	-1.81057397

H	1.34498095	1.40941734	2.04431559
C	-2.51138071	1.25599748	1.63609948
H	-1.91229044	1.59152339	2.47180245
H	-2.75656230	2.11282904	0.98032874
H	-3.46357388	0.96424924	2.10756947
H	1.00897130	-1.26914877	-1.41616557
C	3.84747346	-0.70214643	-1.34324261
H	4.40086637	-1.46067026	-0.81734003
H	3.54431638	-1.19054751	-2.26117011
H	4.55877404	0.08858417	-1.63183739
H	-0.68959309	-1.34566100	-1.61021315

Time = 295.00 fs
E(CASSCF/6-31G(d)) = -479.4673130

C	-0.76732440	-0.20311125	0.26231147
C	0.66830506	-0.02659692	0.47112581
C	1.55367214	0.77942917	1.21600844
C	1.40427090	-0.57781337	-0.67494494
C	2.66591909	-0.24365454	-0.56274149
C	2.76722031	0.68314484	0.56633742
N	-2.02663702	0.03847140	0.90623958
C	-3.11338055	-0.51038342	0.06160940
H	3.65236746	1.10830180	0.99420143
C	-1.02268623	-0.40170412	-1.19784116
C	-2.59407055	-0.30761649	-1.35189798
H	-2.93137659	0.63609895	-1.78511068
H	-2.84214061	-1.10677424	-2.02931180
H	-3.14225141	-1.58075508	0.19846014
H	-4.09092075	-0.15751563	0.28803234
H	-0.56246578	0.36252480	-1.80356182
H	1.37716366	1.42440737	2.02116770
C	-2.51614605	1.25199224	1.63822663
H	-1.93270036	1.58154318	2.48807743
H	-2.74553991	2.11400581	0.97833194
H	-3.46896139	0.95543285	2.09586405
H	1.03349199	-1.26663989	-1.42862784
C	3.84038207	-0.70860778	-1.34670993
H	4.38476259	-1.47044240	-0.81428172
H	3.53959960	-1.19650833	-2.27181560
H	4.55783066	0.06916926	-1.64787331
H	-0.68806386	-1.34647134	-1.59968528

Time = 295.50 fs
E(CASSCF/6-31G(d)) = -479.4683459

C	-0.76353246	-0.20379652	0.26461936

C	0.66755471	-0.02513403	0.47087886
C	1.55230702	0.78596031	1.21456319
C	1.39740286	-0.56618401	-0.67085411
C	2.67259090	-0.24633353	-0.56130480
C	2.77491524	0.68306521	0.56919575
N	-2.02885679	0.03777863	0.90263837
C	-3.11424268	-0.51469233	0.05806704
H	3.64956362	1.10817625	0.98378021
C	-1.02370598	-0.40301476	-1.19209656
C	-2.59172961	-0.30405191	-1.35281439
H	-2.92485179	0.64163256	-1.78358009
H	-2.83642371	-1.10306312	-2.03297031
H	-3.12576023	-1.58082830	0.18914215
H	-4.09343565	-0.15896713	0.29041721
H	-0.55209392	0.35901927	-1.79635582
H	1.40819695	1.44031910	2.00008224
C	-2.52097990	1.24786492	1.64029406
H	-1.95156614	1.57117084	2.50345210
H	-2.73344676	2.11392821	0.97705195
H	-3.47343119	0.94687927	2.08348631
H	1.05920359	-1.26313209	-1.43921508
C	3.83338356	-0.71511629	-1.35039995
H	4.36936771	-1.48035879	-0.81033825
H	3.53444647	-1.20222899	-2.28243445
H	4.55585915	0.04946428	-1.66292536
H	-0.68756966	-1.34737471	-1.58881111

Time = 296.00 fs
E(CASSCF/6-31G(d)) = -479.4678598

C	-0.75938085	-0.20410515	0.26707917
C	0.66566255	-0.02381387	0.47039423
C	1.55139998	0.79236430	1.21291184
C	1.39053256	-0.55488668	-0.66691717
C	2.67966431	-0.24871769	-0.55976652
C	2.78244579	0.68295019	0.57229325
N	-2.03107607	0.03725126	0.89925306
C	-3.11468454	-0.51917536	0.05441527
H	3.64853352	1.10895830	0.97296538
C	-1.02529748	-0.40436473	-1.18661687
C	-2.58921702	-0.30049114	-1.35386113
H	-2.91772230	0.64686443	-1.78184665
H	-2.83255458	-1.09934364	-2.03700675
H	-3.10982636	-1.58198782	0.18088178
H	-4.09638805	-0.15905546	0.29303218
H	-0.54179677	0.35554974	-1.78890507
H	1.43747103	1.45801469	1.98226635

C	-2.52585295	1.24362350	1.64227877
H	-1.96882170	1.56036906	2.51782877
H	-2.72039247	2.11252505	0.97669575
H	-3.47736958	0.93861695	2.07052142
H	1.08561495	-1.25837708	-1.44755737
C	3.82650188	-0.72172553	-1.35434540
H	4.35472215	-1.49033987	-0.80574767
H	3.52879732	-1.20765662	-2.29259610
H	4.55301788	0.02971776	-1.67685902
H	-0.68807428	-1.34852002	-1.57769144

Time = 296.50 fs
E(CASSCF/6-31G(d)) = -479.4662416

C	-0.75494763	-0.20403290	0.26969288
C	0.66264832	-0.02259089	0.46972795
C	1.55104037	0.79858214	1.21097527
C	1.38385157	-0.54395515	-0.66321143
C	2.68697049	-0.25077953	-0.55808905
C	2.78968523	0.68273771	0.57557105
N	-2.03326833	0.03688840	0.89610143
C	-3.11471734	-0.52377490	0.05066176
H	3.65020108	1.11116667	0.96231796
C	-1.02742048	-0.40573815	-1.18142070
C	-2.58654027	-0.29695128	-1.35504025
H	-2.91003675	0.65184460	-1.77993179
H	-2.83051032	-1.09549259	-2.04139874
H	-3.09466394	-1.58454288	0.17373893
H	-4.09940313	-0.15813238	0.29572416
H	-0.53178471	0.35196903	-1.78116661
H	1.46452506	1.47809933	1.96853398
C	-2.53072658	1.23927342	1.64416369
H	-1.98443972	1.54909516	2.53109696
H	-2.70651477	2.10980479	0.97740540
H	-3.48120886	0.93066086	2.05708813
H	1.11216508	-1.25223888	-1.45341305
C	3.81976153	-0.72849178	-1.35856857
H	4.34083398	-1.50026983	-0.80079261
H	3.52258723	-1.21276277	-2.30191730
H	4.54951569	0.01020731	-1.68957959
H	-0.68952135	-1.35006389	-1.56643744

Time = 297.00 fs
E(CASSCF/6-31G(d)) = -479.4640676

C	-0.75032380	-0.20357762	0.27245795
C	0.65855252	-0.02141697	0.46893757

C	1.55129581	0.80458844	1.20872218
C	1.37755025	-0.53341463	-0.65980303
C	2.69434019	-0.25249236	-0.55623568
C	2.79653052	0.68237425	0.57897760
N	-2.03540544	0.03668691	0.89319770
C	-3.11435959	-0.52843994	0.04681904
H	3.65526306	1.11522913	0.95229246
C	-1.03003101	-0.40712149	-1.17652229
C	-2.58371025	-0.29345421	-1.35635377
H	-2.90185830	0.65665370	-1.77786116
H	-2.83023473	-1.09136088	-2.04609723
H	-3.08047098	-1.58866913	0.16774207
H	-4.10205500	-0.15657839	0.29831909
H	-0.52226515	0.34813428	-1.77311437
H	1.48904028	1.50076394	1.95915962
C	-2.53555634	1.23481650	1.64593836
H	-1.99842756	1.53730349	2.54314474
H	-2.69197164	2.10585579	0.97925469
H	-3.48539670	0.92301885	2.04332295
H	1.13824796	-1.24470058	-1.45667885
C	3.81318593	-0.73546991	-1.36307998
H	4.32768889	-1.51001346	-0.79578315
H	3.51574264	-1.21754975	-2.31008477
H	4.54559944	-0.00877654	-1.70103119
H	-0.69184265	-1.35215660	-1.55516425

Time = 297.50 fs
E(CASSCF/6-31G(d)) = -479.4617812

C	-0.74561222	-0.20273963	0.27536703
C	0.65343711	-0.02024224	0.46808009
C	1.55221401	0.81039713	1.20617127
C	1.37180957	-0.52328204	-0.65674486
C	2.70161022	-0.25383137	-0.55417272
C	2.80291502	0.68182298	0.58247498
N	-2.03745872	0.03664126	0.89055280
C	-3.11363258	-0.53312950	0.04290359
H	3.66405010	1.12140272	0.94317742
C	-1.03308201	-0.40850523	-1.17193134
C	-2.58074114	-0.29002515	-1.35780305
H	-2.89326180	0.66139717	-1.77566209
H	-2.83164192	-1.08678653	-2.05103577
H	-3.06742923	-1.59439024	0.16288607
H	-4.10391791	-0.15477794	0.30063472
H	-0.51343211	0.34391950	-1.76474881
H	1.51080513	1.52571535	1.95385323
C	-2.54029585	1.23024995	1.64759895

H	-2.01082005	1.52494975	2.55387118
H	-2.67693611	2.10084600	0.98224924
H	-3.49036101	0.91570161	2.02936776
H	1.16323842	-1.23586112	-1.45739084
C	3.80679676	-0.74270975	-1.36787816
H	4.31526193	-1.51943363	-0.79104002
H	3.50818082	-1.22205228	-2.31686920
H	4.54153744	-0.02694745	-1.71119351
H	-0.69496476	-1.35492859	-1.54398326

Time = 298.00 fs
E(CASSCF/6-31G(d)) = -479.4595497

C	-0.74092583	-0.20152224	0.27840798
C	0.64738530	-0.01901581	0.46721021
C	1.55382517	0.81605682	1.20338264
C	1.36679417	-0.51356623	-0.65407486
C	2.70862911	-0.25477499	-0.55187166
C	2.80881693	0.68106973	0.58604223
N	-2.03940023	0.03674362	0.88817499
C	-3.11255634	-0.53781448	0.03893473
H	3.67644160	1.12971767	0.93506841
C	-1.03652391	-0.40988538	-1.16765331
C	-2.57765021	-0.28669082	-1.35938836
H	-2.88433003	0.66619659	-1.77336012
H	-2.83462028	-1.08160835	-2.05614051
H	-3.05570332	-1.60157753	0.15913178
H	-4.10461097	-0.15309732	0.30249286
H	-0.50545708	0.33922583	-1.75610539
H	1.52967328	1.55222850	1.95186508
C	-2.54490098	1.22556548	1.64914768
H	-2.02167261	1.51199496	2.56319937
H	-2.66159006	2.09501973	0.98633015
H	-3.49647167	0.90873398	2.01535648
H	1.18651754	-1.22592793	-1.45572034
C	3.80061355	-0.75025254	-1.37295004
H	4.30352743	-1.52840869	-0.78687765
H	3.49981042	-1.22633660	-2.32213372
H	4.53759959	-0.04404219	-1.72007656
H	-0.69881593	-1.35847785	-1.53299423

Time = 298.50 fs
E(CASSCF/6-31G(d)) = -479.4572915

C	-0.73638543	-0.19993161	0.28156411
C	0.64050135	-0.01768684	0.46637858
C	1.55614413	0.82163852	1.20044235

C	1.36264685	-0.50426735	-0.65181478
C	2.71526209	-0.25530556	-0.54931021
C	2.81425954	0.68012363	0.58967380
N	-2.04120425	0.03698376	0.88607023
C	-3.11114712	-0.54247789	0.03493374
H	3.69186969	1.13996758	0.92788143
C	-1.04030485	-0.41126495	-1.16368987
C	-2.57445799	-0.28347746	-1.36110853
H	-2.87514973	0.67117801	-1.77097340
H	-2.83903698	-1.07567646	-2.06133945
H	-3.04543997	-1.60996462	0.15640917
H	-4.10382926	-0.15186577	0.30373128
H	-0.49848365	0.33398657	-1.74725734
H	1.54553338	1.57929582	1.95217119
C	-2.54933409	1.22074895	1.65059227
H	-2.03105732	1.49840725	2.57108461
H	-2.64611606	2.08868337	0.99138358
H	-3.50399958	0.90216545	2.00139956
H	1.20749522	-1.21521053	-1.45196703
C	3.79465305	-0.75812749	-1.37827179
H	4.29246749	-1.53684745	-0.78358694
H	3.49053400	-1.23049828	-2.32583865
H	4.53403610	-0.05984028	-1.72771291
H	-0.70333421	-1.36285737	-1.52228080

Time = 299.00 fs
E(CASSCF/6-31G(d)) = -479.4547704

C	-0.73211782	-0.19797721	0.28481443
C	0.63290892	-0.01620532	0.46563094
C	1.55917099	0.82722108	1.19744611
C	1.35948494	-0.49537733	-0.64997020
C	2.72139439	-0.25541076	-0.54647392
C	2.81930428	0.67901476	0.59337579
N	-2.04284845	0.03734925	0.88424253
C	-3.10941526	-0.54711328	0.03092272
H	3.70940794	1.15174250	0.92140397
C	-1.04437130	-0.41265452	-1.16003933
C	-2.57118816	-0.28040882	-1.36296072
H	-2.86580712	0.67646026	-1.76850897
H	-2.84474237	-1.06886466	-2.06657088
H	-3.03676388	-1.61918156	0.15462071
H	-4.10136918	-0.15136165	0.30421207
H	-0.49261997	0.32817299	-1.73831896
H	1.55830041	1.60580099	1.95366531
C	-2.55356727	1.21578069	1.65194444
H	-2.03905613	1.48416485	2.57752446

H	-2.63069143	2.08219292	0.99725211
H	-3.51308806	0.89607806	1.98757784
H	1.22563165	-1.20410317	-1.44654219
C	3.78892945	-0.76634930	-1.38380996
H	4.28207681	-1.54470014	-0.78142268
H	3.48025029	-1.23465692	-2.32803903
H	4.53105490	-0.07418150	-1.73415022
H	-0.70847078	-1.36806984	-1.51190532

Time = 299.50 fs
E(CASSCF/6-31G(d)) = -479.4518268

C	-0.72825356	-0.19567161	0.28813424
C	0.62474951	-0.01452249	0.46500715
C	1.56289155	0.83287874	1.19448662
C	1.35739834	-0.48688055	-0.64853120
C	2.72693286	-0.25508439	-0.54335684
C	2.82403775	0.67778795	0.59716016
N	-2.04431468	0.03782543	0.88269401
C	-3.10736455	-0.55172229	0.02692367
H	3.72791764	1.16449680	0.91536068
C	-1.04866872	-0.41407250	-1.15669696
C	-2.56786741	-0.27750434	-1.36494028
H	-2.85638394	0.68214287	-1.76595835
H	-2.85157480	-1.06107838	-2.07179005
H	-3.02977380	-1.62879558	0.15364726
H	-4.09714206	-0.15180188	0.30382870
H	-0.48793339	0.32179606	-1.72944403
H	1.56791989	1.63067006	1.95530778
C	-2.55758467	1.21063676	1.65321828
H	-2.04575702	1.46925751	2.58256327
H	-2.61548157	2.07593030	1.00375177
H	-3.52373423	0.89058957	1.97393917
H	1.24045542	-1.19306450	-1.43994528
C	3.78345463	-0.77491642	-1.38952304
H	4.27236600	-1.55196604	-0.78059253
H	3.46885809	-1.23894994	-2.32887841
H	4.52880248	-0.08698037	-1.73944431
H	-0.71419185	-1.37406513	-1.50190783

Time = 300.00 fs
E(CASSCF/6-31G(d)) = -479.4484901

C	-0.72492492	-0.19303029	0.29149573
C	0.61618030	-0.01259127	0.46454058
C	1.56727770	0.83867309	1.19164566
C	1.35644890	-0.47875537	-0.64747428

C	2.73180652	-0.25432702	-0.53996199
C	2.82855779	0.67649567	0.60103947
N	-2.04558929	0.03839563	0.88142457
C	-3.10499225	-0.55631180	0.02295756
H	3.74620308	1.17762689	0.90947442
C	-1.05314243	-0.41554475	-1.15365538
C	-2.56452519	-0.27477759	-1.36704079
H	-2.84695421	0.68829569	-1.76329435
H	-2.85936556	-1.05226088	-2.07697398
H	-3.02453720	-1.63835418	0.15335443
H	-4.09118092	-0.15333524	0.30251112
H	-0.48444680	0.31490560	-1.72082149
H	1.57437658	1.65297222	1.95621547
C	-2.56138311	1.20529137	1.65442809
H	-2.05125086	1.45368695	2.58629419
H	-2.60063435	2.07027488	1.01069247
H	-3.53578802	0.88585026	1.96050148
H	1.25157850	-1.18258783	-1.43273063
C	3.77823843	-0.78381038	-1.39536318
H	4.26336268	-1.55869720	-0.78124962
H	3.45626062	-1.24352423	-2.32857669
H	4.52734990	-0.09823496	-1.74365452
H	-0.72047776	-1.38074372	-1.49230647

Time = 300.50 fs
E(CASSCF/6-31G(d)) = -479.4449826

C	-0.72226353	-0.19007132	0.29486853
C	0.60737156	-0.01036660	0.46425787
C	1.57228802	0.84464897	1.18899039
C	1.35667049	-0.47097679	-0.64676534
C	2.73596599	-0.25314662	-0.53630160
C	2.83296159	0.67519194	0.60502335
N	-2.04666331	0.03904124	0.88043167
C	-3.10228967	-0.56089117	0.01904356
H	3.76314112	1.19053921	0.90350929
C	-1.05773857	-0.41710329	-1.15090475
C	-2.56119334	-0.27223523	-1.36925401
H	-2.83758189	0.69495104	-1.76046973
H	-2.86794340	-1.04239654	-2.08212381
H	-3.02108540	-1.64742456	0.15359937
H	-4.08364072	-0.15603871	0.30022890
H	-0.48213750	0.30758671	-1.71266849
H	1.57770119	1.67197266	1.95570226
C	-2.56497110	1.19971992	1.65558592
H	-2.05562877	1.43746716	2.58885732
H	-2.58627714	2.06557109	1.01790036

H	-3.54896775	0.88203288	1.94726138
H	1.25870746	-1.17316253	-1.42546370
C	3.77328872	-0.79299646	-1.40127828
H	4.25511108	-1.56499869	-0.78348803
H	3.44237144	-1.24852602	-2.32741171
H	4.52668615	-0.10802835	-1.74684205
H	-0.72731949	-1.38796546	-1.48310048

Time = 301.00 fs
E(CASSCF/6-31G(d)) = -479.4416582

C	-0.72039748	-0.18681481	0.29822083
C	0.59850412	-0.00780581	0.46417875
C	1.57786940	0.85083306	1.18657167
C	1.35807044	-0.46351954	-0.64636379
C	2.73938197	-0.25155921	-0.53239699
C	2.83733667	0.67392764	0.60911661
N	-2.04753223	0.03974172	0.87971017
C	-3.09924364	-0.56546986	0.01519833
H	3.77777013	1.20270074	0.89729467
C	-1.06240451	-0.41878456	-1.14843371
C	-2.55790578	-0.26987662	-1.37157017
H	-2.82832020	0.70209985	-1.75741797
H	-2.87713942	-1.03151117	-2.08726583
H	-3.01941074	-1.65562708	0.15423721
H	-4.07479310	-0.15991669	0.29699288
H	-0.48093914	0.29995183	-1.70522146
H	1.57797221	1.68715337	1.95328912
C	-2.56836654	1.19390320	1.65669960
H	-2.05898255	1.42062311	2.59043373
H	-2.57251538	2.06209624	1.02524098
H	-3.56289002	0.87931840	1.93420435
H	1.26165055	-1.16522769	-1.41866929
C	3.76861077	-0.80242539	-1.40721414
H	4.24767020	-1.57102615	-0.78734078
H	3.42712118	-1.25408896	-2.32569505
H	4.52672121	-0.11652206	-1.74907189
H	-0.73471373	-1.39556131	-1.47427419

Time = 301.50 fs
E(CASSCF/6-31G(d)) = -479.4388945

C	-0.71944847	-0.18328509	0.30151701
C	0.58977129	-0.00487180	0.46431598
C	1.58395337	0.85722885	1.18442680
C	1.36062786	-0.45636907	-0.64622829
C	2.74204230	-0.24959014	-0.52828165

C	2.84175246	0.67274879	0.61331529
N	-2.04819573	0.04047452	0.87925253
C	-3.09584009	-0.57005149	0.01143899
H	3.78932645	1.21365943	0.89073692
C	-1.06708607	-0.42062559	-1.14623153
C	-2.55470032	-0.26769575	-1.37397571
H	-2.81921861	0.70968519	-1.75405719
H	-2.88678190	-1.01967941	-2.09244477
H	-3.01946378	-1.66265298	0.15512560
H	-4.06502230	-0.16490030	0.29285670
H	-0.48074805	0.29213795	-1.69873086
H	1.57531597	1.69820428	1.94870135
C	-2.57159017	1.18783546	1.65776963
H	-2.06140159	1.40320468	2.59123823
H	-2.55944503	2.06003605	1.03262956
H	-3.57709697	0.87788051	1.92132732
H	1.26032490	-1.15912512	-1.41277748
C	3.76421058	-0.81202822	-1.41311209
H	4.24111568	-1.57697385	-0.79277769
H	3.41048045	-1.26031430	-2.32374144
H	4.52729532	-0.12393708	-1.75041509
H	-0.74265197	-1.40334278	-1.46580880

Time = 302.00 fs
E(CASSCF/6-31G(d)) = -479.4370043

C	-0.71952345	-0.17950120	0.30472716
C	0.58135062	-0.00152623	0.46467553
C	1.59047361	0.86383337	1.18257370
C	1.36430022	-0.44950681	-0.64632086
C	2.74395806	-0.24726932	-0.52399002
C	2.84626874	0.67169189	0.61761775
N	-2.04865934	0.04121687	0.87904734
C	-3.09205799	-0.57464417	0.00777317
H	3.79730102	1.22309143	0.88380110
C	-1.07173981	-0.42266594	-1.14428311
C	-2.55160974	-0.26567614	-1.37645972
H	-2.81030101	0.71762618	-1.75028710
H	-2.89672458	-1.00699091	-2.09773439
H	-3.02115189	-1.66830189	0.15613245
H	-4.05478175	-0.17086493	0.28790393
H	-0.48142987	0.28427404	-1.69342971
H	1.56989780	1.70505632	1.94185323
C	-2.57467058	1.18151219	1.65879161
H	-2.06298271	1.38524065	2.59150866
H	-2.54712101	2.05945757	1.04006133
H	-3.59113220	0.87786343	1.90861412

H	1.25476101	-1.15503371	-1.40805647
C	3.76008185	-0.82174492	-1.41892692
H	4.23551750	-1.58308116	-0.79972195
H	3.39242884	-1.26727278	-2.32183168
H	4.52819735	-0.13055512	-1.75095902
H	-0.75113395	-1.41113712	-1.45766549

Time = 302.50 fs
E(CASSCF/6-31G(d)) = -479.4361467

C	-0.72071301	-0.17548476	0.30781936
C	0.57342047	0.00226258	0.46525665
C	1.59735284	0.87062124	1.18101706
C	1.36901883	-0.44293249	-0.64661165
C	2.74515475	-0.24463528	-0.51956670
C	2.85092302	0.67078623	0.62201471
N	-2.04893240	0.04194419	0.87908079
C	-3.08788011	-0.57924713	0.00420902
H	3.80141023	1.23077924	0.87652077
C	-1.07632117	-0.42494052	-1.14257591
C	-2.54866938	-0.26379794	-1.37900785
H	-2.80159150	0.72580664	-1.74600241
H	-2.90682405	-0.99357299	-2.10321885
H	-3.02434256	-1.67247225	0.15713787
H	-4.04459061	-0.17762984	0.28225044
H	-0.48283281	0.27648687	-1.68953819
H	1.56191837	1.70784662	1.93285184
C	-2.57763185	1.17494711	1.65975401
H	-2.06382597	1.36678148	2.59148967
H	-2.53559512	2.06031072	1.04759283
H	-3.60454219	0.87937325	1.89607528
H	1.24510571	-1.15295187	-1.40459223
C	3.75621657	-0.83150401	-1.42461390
H	4.23095144	-1.58960434	-0.80804607
H	3.37300732	-1.27497627	-2.32018492
H	4.52918479	-0.13667685	-1.75080813
H	-0.76014348	-1.41878250	-1.44981224

Time = 303.00 fs
E(CASSCF/6-31G(d)) = -479.4362810

C	-0.72308443	-0.17125628	0.31076314
C	0.56614561	0.00652136	0.46605209
C	1.60451028	0.87755007	1.17974629
C	1.37469119	-0.43665945	-0.64708103
C	2.74567392	-0.24173258	-0.51506145
C	2.85573362	0.67005229	0.62649344

N	-2.04902891	0.04263101	0.87933640
C	-3.08329342	-0.58385342	0.00075256
H	3.80160747	1.23662751	0.86898532
C	-1.08078836	-0.42747850	-1.14109899
C	-2.54591356	-0.26203755	-1.38160466
H	-2.79310890	0.73409200	-1.74109596
H	-2.91694905	-0.97957213	-2.10899034
H	-3.02886560	-1.67517219	0.15803823
H	-4.03498771	-0.18497806	0.27603082
H	-0.48480062	0.26887897	-1.68724367
H	1.55160296	1.70693267	1.92199321
C	-2.58049330	1.16816726	1.66064099
H	-2.06404155	1.34788219	2.59141799
H	-2.52490688	2.06243330	1.05533941
H	-3.61692042	0.88246576	1.88373928
H	1.23162635	-1.15267881	-1.40227233
C	3.75259991	-0.84123535	-1.43013559
H	4.22748493	-1.59680594	-0.81758744
H	3.35231396	-1.28337413	-2.31893160
H	4.53000646	-0.14260649	-1.75008979
H	-0.76965172	-1.42614755	-1.44221970

Time = 303.50 fs
E(CASSCF/6-31G(d)) = -479.4371573

C	-0.72667614	-0.16683411	0.31353000
C	0.55967004	0.01127087	0.46704861
C	1.61186435	0.88456057	1.17873511
C	1.38120196	-0.43071423	-0.64771945
C	2.74557222	-0.23861079	-0.51052765
C	2.86069899	0.66950125	0.63103866
N	-2.04896735	0.04325125	0.87979510
C	-3.07829271	-0.58844878	-0.00259155
H	3.79808322	1.24066910	0.86133227
C	-1.08510247	-0.43030120	-1.13984377
C	-2.54337488	-0.26036925	-1.38423376
H	-2.78486913	0.74233939	-1.73546551
H	-2.92697980	-0.96514522	-2.11514048
H	-3.03451716	-1.67651796	0.15874882
H	-4.02648539	-0.19267732	0.26938493
H	-0.48718647	0.26151602	-1.68668682
H	1.53918889	1.70289404	1.90976269
C	-2.58326705	1.16121191	1.66143502
H	-2.06375280	1.32860225	2.59150705
H	-2.51508580	2.06556888	1.06345702
H	-3.62793204	0.88713944	1.87165382
H	1.21471213	-1.15382815	-1.40080426

C	3.74920980	-0.85087422	-1.43546257
H	4.22516815	-1.60493633	-0.82816273
H	3.33051142	-1.29235064	-2.31809926
H	4.53042309	-0.14863091	-1.74895586
H	-0.77961486	-1.43314102	-1.43486452

Time = 304.00 fs
E(CASSCF/6-31G(d)) = -479.4383383

C	-0.73149375	-0.16223388	0.31609402
C	0.55411216	0.01652541	0.46822708
C	1.61933490	0.89157706	1.17794194
C	1.38841405	-0.42513359	-0.64852499
C	2.74492054	-0.23532295	-0.50602033
C	2.86579708	0.66913422	0.63563292
N	-2.04877069	0.04377862	0.88043542
C	-3.07288445	-0.59301063	-0.00581901
H	3.79126563	1.24307137	0.85374174
C	-1.08922725	-0.43342035	-1.13880501
C	-2.54108342	-0.25876722	-1.38687831
H	-2.77688763	0.75041070	-1.72901893
H	-2.93680755	-0.95044908	-2.12175219
H	-3.04106592	-1.67672700	0.15920523
H	-4.01951879	-0.20050294	0.26244298
H	-0.48986438	0.25441537	-1.68795180
H	1.52491122	1.69652521	1.89682695
C	-2.58595638	1.15413032	1.66212012
H	-2.06310099	1.30900300	2.59193394
H	-2.50614950	2.06939526	1.07212123
H	-3.63733282	0.89333406	1.85988278
H	1.19487031	-1.15586848	-1.39976075
C	3.74601701	-0.86036501	-1.44057289
H	4.22402301	-1.61421277	-0.83958482
H	3.30782716	-1.30172875	-2.31761265
H	4.53022610	-0.15500173	-1.74758269
H	-0.78997322	-1.43971986	-1.42773018

Time = 304.50 fs
E(CASSCF/6-31G(d)) = -479.4392805

C	-0.73750707	-0.15746791	0.31843187
C	0.54956094	0.02229242	0.46956281
C	1.62684530	0.89850965	1.17731185
C	1.39617052	-0.41996043	-0.64949954
C	2.74380312	-0.23192393	-0.50159417
C	2.87098529	0.66894158	0.64025666
N	-2.04846618	0.04418679	0.88123403

C	-3.06709060	-0.59750778	-0.00892493
H	3.78181957	1.24414218	0.84643203
C	-1.09312881	-0.43683702	-1.13798139
C	-2.53906636	-0.25720732	-1.38952150
H	-2.76918157	0.75818361	-1.72167986
H	-2.94633323	-0.93563112	-2.12889158
H	-3.04826070	-1.67610477	0.15936382
H	-4.01439871	-0.20826048	0.25531188
H	-0.49273724	0.24754025	-1.69105990
H	1.50898746	1.68880791	1.88400981
C	-2.58855501	1.14697922	1.66268512
H	-2.06224761	1.28914680	2.59282906
H	-2.49810146	2.07355512	1.08150562
H	-3.64498031	0.90093260	1.84850290
H	1.17271413	-1.15818221	-1.39864183
C	3.74298610	-0.86966385	-1.44545028
H	4.22403269	-1.62479932	-0.85168028
H	3.28454718	-1.31128092	-2.31730807
H	4.52925214	-0.16192152	-1.74616968
H	-0.80065108	-1.44589359	-1.42080746

Time = 305.00 fs
E(CASSCF/6-31G(d)) = -479.4394633

C	-0.74465380	-0.15254166	0.32052424
C	0.54607024	0.02857629	0.47102750
C	1.63433034	0.90526473	1.17678019
C	1.40430472	-0.41523511	-0.65064550
C	2.74231421	-0.22846703	-0.49729934
C	2.87620390	0.66890263	0.64489273
N	-2.04808489	0.04445030	0.88216621
C	-3.06094664	-0.60190432	-0.01190522
H	3.77062986	1.24433238	0.83964457
C	-1.09677904	-0.44054421	-1.13737487
C	-2.53734590	-0.25566718	-1.39214866
H	-2.76176525	0.76556334	-1.71338599
H	-2.95547090	-0.92081206	-2.13660380
H	-3.05584222	-1.67502115	0.15920068
H	-4.01126952	-0.21580968	0.24806039
H	-0.49574385	0.24079570	-1.69596791
H	1.49159312	1.68084097	1.87222990
C	-2.59104888	1.13981448	1.66312617
H	-2.06137225	1.26908446	2.59427118
H	-2.49092388	2.07768697	1.09176644
H	-3.65084472	0.90976953	1.83759289
H	1.14892216	-1.16013071	-1.39693805
C	3.74007509	-0.87874656	-1.45008518

H	4.22513496	-1.63679723	-0.86431127
H	3.26098878	-1.32075423	-2.31696041
H	4.52739228	-0.16953821	-1.74493268
H	-0.81156633	-1.45172585	-1.41408972

Time = 305.50 fs
E(CASSCF/6-31G(d)) = -479.4384977

C	-0.75282690	-0.14746404	0.32235097
C	0.54366844	0.03536572	0.47258645
C	1.64172049	0.91173944	1.17628192
C	1.41262723	-0.41100138	-0.65195960
C	2.74055925	-0.22500969	-0.49318906
C	2.88136759	0.66898658	0.64951671
N	-2.04766209	0.04454418	0.88320469
C	-3.05451531	-0.60615217	-0.01474982
H	3.75878967	1.24422007	0.83365039
C	-1.10014778	-0.44451982	-1.13699214
C	-2.53594291	-0.25413077	-1.39474221
H	-2.75466715	0.77247534	-1.70410848
H	-2.96412733	-0.90611075	-2.14489295
H	-3.06353632	-1.67388470	0.15870893
H	-4.01010202	-0.22305749	0.24073079
H	-0.49884927	0.23404882	-1.70256478
H	1.47289893	1.67374878	1.86243119
C	-2.59341156	1.13270340	1.66344756
H	-2.06067269	1.24889666	2.59628608
H	-2.48458914	2.08144436	1.10300770
H	-3.65499501	0.91962181	1.82725158
H	1.12426054	-1.16111005	-1.39419102
C	3.73724373	-0.88758974	-1.45446192
H	4.22721463	-1.65020771	-0.87736095
H	3.23753125	-1.32986839	-2.31631438
H	4.52460102	-0.17792516	-1.74410312
H	-0.82260955	-1.45732029	-1.40758565

Time = 306.00 fs
E(CASSCF/6-31G(d)) = -479.4362697

C	-0.76189302	-0.14223754	0.32389492
C	0.54235259	0.04264729	0.47420253
C	1.64895803	0.91784848	1.17576080
C	1.42095021	-0.40729230	-0.65343225
C	2.73864743	-0.22160553	-0.48930978
C	2.88638047	0.66915521	0.65410874
N	-2.04723554	0.04444524	0.88432137
C	-3.04787276	-0.61020400	-0.01744943

H	3.74751567	1.24448012	0.82871761
C	-1.10321172	-0.44873582	-1.13684239
C	-2.53487255	-0.25258569	-1.39728745
H	-2.74791347	0.77888424	-1.69383710
H	-2.97222038	-0.89160970	-2.15373290
H	-3.07107639	-1.67310335	0.15789504
H	-4.01068840	-0.22997532	0.23332734
H	-0.50204524	0.22712551	-1.71068493
H	1.45305259	1.66851551	1.85542869
C	-2.59561126	1.12570674	1.66366381
H	-2.06035621	1.22865242	2.59885411
H	-2.47904047	2.08452482	1.11528968
H	-3.65760757	0.93023431	1.81756966
H	1.09949752	-1.16060551	-1.39003488
C	3.73444952	-0.89618973	-1.45856662
H	4.23010476	-1.66495122	-0.89076318
H	3.21454461	-1.33835642	-2.31511728
H	4.52089528	-0.18709462	-1.74391893
H	-0.83367033	-1.46282318	-1.40130197

Time = 306.50 fs
E(CASSCF/6-31G(d)) = -479.4329836

C	-0.77169562	-0.13686231	0.32513945
C	0.54209718	0.05040265	0.47583621
C	1.65599183	0.92353198	1.17518037
C	1.42909205	-0.40413094	-0.65504401
C	2.73668523	-0.21830665	-0.48570443
C	2.89113765	0.66936916	0.65865308
N	-2.04684343	0.04413257	0.88548604
C	-3.04111036	-0.61401473	-0.01999436
H	3.73804386	1.24581622	0.82509578
C	-1.10595294	-0.45315811	-1.13693653
C	-2.53414520	-0.25102443	-1.39977074
H	-2.74153296	0.78478999	-1.68258691
H	-2.97967301	-0.87736519	-2.16306083
H	-3.07820325	-1.67304492	0.15677446
H	-4.01267233	-0.23658510	0.22583135
H	-0.50533499	0.21983148	-1.72011464
H	1.43223300	1.66583285	1.85177998
C	-2.59761162	1.11888121	1.66380012
H	-2.06063138	1.20842401	2.60191887
H	-2.47419379	2.08668109	1.12861441
H	-3.65894956	0.94132449	1.80863269
H	1.07538505	-1.15822500	-1.38422811
C	3.73165180	-0.90455452	-1.46238237
H	4.23359162	-1.68086258	-0.90449101

H	3.19238434	-1.34597588	-2.31314784
H	4.51635326	-0.19699591	-1.74461967
H	-0.84463244	-1.46840700	-1.39524567

Time = 307.00 fs
E(CASSCF/6-31G(d)) = -479.4291997

C	-0.78207619	-0.13133924	0.32606664
C	0.54286937	0.05861401	0.47744888
C	1.66279157	0.92876230	1.17452446
C	1.43690744	-0.40152799	-0.65676088
C	2.73474876	-0.21516919	-0.48241798
C	2.89551712	0.66960191	0.66314939
N	-2.04651594	0.04358993	0.88666145
C	-3.03433302	-0.61754721	-0.02237683
H	3.73148097	1.24886656	0.82299767
C	-1.10836113	-0.45774981	-1.13728452
C	-2.53376597	-0.24944357	-1.40217929
H	-2.73555601	0.79022544	-1.67039871
H	-2.98641635	-0.86340930	-2.17277210
H	-3.08466225	-1.67399441	0.15537308
H	-4.01558817	-0.24294428	0.21821773
H	-0.50870738	0.21197260	-1.73059231
H	1.41069997	1.66597723	1.85168919
C	-2.59937626	1.11227690	1.66389588
H	-2.06169610	1.18828686	2.60539251
H	-2.46992823	2.08771743	1.14291612
H	-3.65935484	0.95259244	1.80051397
H	1.05262100	-1.15372115	-1.37667743
C	3.72881349	-0.91270125	-1.46588842
H	4.23742829	-1.69770427	-0.91854950
H	3.17137140	-1.35252490	-2.31024067
H	4.51111101	-0.20752201	-1.74644403
H	-0.85537276	-1.47424991	-1.38941821

Time = 307.50 fs
E(CASSCF/6-31G(d)) = -479.4295671

C	-0.79268778	-0.12564701	0.32668289
C	0.54452589	0.06719263	0.47897432
C	1.66906193	0.93357129	1.17390157
C	1.44395477	-0.39948126	-0.65856835
C	2.73320382	-0.21217449	-0.47941937
C	2.89968369	0.66976066	0.66748131
N	-2.04637135	0.04277649	0.88786767
C	-3.02762367	-0.62075589	-0.02456529
H	3.72862911	1.25405285	0.82243112

C	-1.11040859	-0.46245427	-1.13792400
C	-2.53373836	-0.24785324	-1.40450069
H	-2.73001802	0.79525231	-1.65733708
H	-2.99235087	-0.84977646	-2.18281682
H	-3.09030556	-1.67617803	0.15361197
H	-4.01896726	-0.24912676	0.21043122
H	-0.51236895	0.20329969	-1.74197074
H	1.38892343	1.66886882	1.85479896
C	-2.60084150	1.10595110	1.66394412
H	-2.06377476	1.16834792	2.60925614
H	-2.46622557	2.08769108	1.15816870
H	-3.65923356	0.96373417	1.79331020
H	1.03187752	-1.14700807	-1.36735890
C	3.72590388	-0.92064197	-1.46906059
H	4.24130011	-1.71514546	-0.93291057
H	3.15180362	-1.35782994	-2.30628700
H	4.50532172	-0.21850534	-1.74954944
H	-0.86584844	-1.48057794	-1.38381981

Time = 308.00 fs
E(CASSCF/6-31G(d)) = -479.4285611

C	-0.80337722	-0.11978702	0.32696495
C	0.54700925	0.07613591	0.48038596
C	1.67480343	0.93800414	1.17333927
C	1.45018512	-0.39796898	-0.66041458
C	2.73206910	-0.20937854	-0.47675096
C	2.90354409	0.66986500	0.67168497
N	-2.04643834	0.04168762	0.88906861
C	-3.02108833	-0.62363607	-0.02655890
H	3.72986611	1.26156796	0.82344945
C	-1.11210211	-0.46724544	-1.13885897
C	-2.53405566	-0.24625330	-1.40672707
H	-2.72494030	0.79996315	-1.64347036
H	-2.99744186	-0.83644297	-2.19304248
H	-3.09490183	-1.67963827	0.15151466
H	-4.02228481	-0.25522056	0.20246134
H	-0.51623657	0.19367140	-1.75398958
H	1.36739089	1.67396621	1.86060516
C	-2.60198360	1.09992557	1.66400210
H	-2.06701182	1.14865699	2.61340706
H	-2.46289180	2.08655198	1.17420234
H	-3.65896899	0.97447313	1.78704648
H	1.01361339	-1.13813720	-1.35647749
C	3.72289405	-0.92841480	-1.47187684
H	4.24494289	-1.73288478	-0.94762427
H	3.13385289	-1.36180524	-2.30126780

H	4.49918800	-0.22978008	-1.75415167
H	-0.87596433	-1.48753563	-1.37843481

Time = 308.50 fs
E(CASSCF/6-31G(d)) = -479.4272908

C	-0.81393453	-0.11376138	0.32689288
C	0.55022892	0.08541392	0.48164913
C	1.67994752	0.94212881	1.17290752
C	1.45549598	-0.39695757	-0.66224483
C	2.73141939	-0.20682048	-0.47443668
C	2.90709993	0.66994203	0.67577014
N	-2.04676439	0.04031644	0.89024163
C	-3.01483135	-0.62619100	-0.02835159
H	3.73508814	1.27125521	0.82598972
C	-1.11344795	-0.47209319	-1.14009710
C	-2.53470667	-0.24464958	-1.40884812
H	-2.72034776	0.80445931	-1.62889944
H	-3.00165456	-0.82339539	-2.20329096
H	-3.09824971	-1.68429278	0.14907491
H	-4.02506203	-0.26129648	0.19432282
H	-0.52024499	0.18297994	-1.76641929
H	1.34673729	1.68050460	1.86834223
C	-2.60277989	1.09422292	1.66411969
H	-2.07152568	1.12929093	2.61776696
H	-2.45974529	2.08436707	1.19080888
H	-3.65891936	0.98454030	1.78174147
H	0.99818435	-1.12733861	-1.34436798
C	3.71976627	-0.93604569	-1.47431081
H	4.24808787	-1.75058484	-0.96270323
H	3.11764088	-1.36441433	-2.29524786
H	4.49292265	-0.24115483	-1.76042106
H	-0.88564404	-1.49523001	-1.37324217

Time = 309.00 fs
E(CASSCF/6-31G(d)) = -479.4259776

C	-0.82414600	-0.10757130	0.32644692
C	0.55408695	0.09499253	0.48272943
C	1.68441442	0.94602362	1.17268698
C	1.45980110	-0.39639739	-0.66399617
C	2.73132717	-0.20453186	-0.47249174
C	2.91040255	0.67003879	0.67974633
N	-2.04739810	0.03865773	0.89136873
C	-3.00894838	-0.62843865	-0.02993941
H	3.74374217	1.28263656	0.82990352
C	-1.11445773	-0.47697338	-1.14164490

C	-2.53567487	-0.24304895	-1.41085356
H	-2.71625881	0.80884672	-1.61373887
H	-3.00497155	-0.81061688	-2.21340644
H	-3.10017068	-1.68994193	0.14627277
H	-4.02689594	-0.26740729	0.18605699
H	-0.52431019	0.17115798	-1.77906112
H	1.32764933	1.68763531	1.87715087
C	-2.60321874	1.08885601	1.66434693
H	-2.07739692	1.11032384	2.62228305
H	-2.45659505	2.08128786	1.20775168
H	-3.65937652	0.99369632	1.77738915
H	0.98578490	-1.11501010	-1.33149809
C	3.71651114	-0.94355595	-1.47633723
H	4.25048966	-1.76792031	-0.97813542
H	3.10320333	-1.36568170	-2.28837292
H	4.48672427	-0.25244863	-1.76848679
H	-0.89483369	-1.50371271	-1.36820547

Time = 309.50 fs
E(CASSCF/6-31G(d)) = -479.4245453

C	-0.83380577	-0.10121786	0.32560725
C	0.55848431	0.10483575	0.48359415
C	1.68812791	0.94976520	1.17275521
C	1.46304455	-0.39622290	-0.66559628
C	2.73185062	-0.20253880	-0.47092516
C	2.91353297	0.67021796	0.68362420
N	-2.04838470	0.03670844	0.89243500
C	-3.00352454	-0.63040991	-0.03132159
H	3.75494638	1.29499326	0.83498509
C	-1.11514865	-0.48186932	-1.14350613
C	-2.53693869	-0.24145773	-1.41273272
H	-2.71268376	0.81322426	-1.59811433
H	-3.00739672	-0.79809211	-2.22324198
H	-3.10050911	-1.69629441	0.14308122
H	-4.02748189	-0.27358280	0.17773498
H	-0.52832150	0.15818407	-1.79175177
H	1.31079967	1.69454731	1.88619893
C	-2.60330266	1.08382787	1.66473362
H	-2.08466353	1.09182486	2.62693164
H	-2.45324355	2.07753604	1.22477314
H	-3.66053365	1.00173744	1.77395661
H	0.97643273	-1.10171131	-1.31846289
C	3.71312847	-0.95095902	-1.47793345
H	4.25193950	-1.78459767	-0.99387647
H	3.09048743	-1.36569064	-2.28086434
H	4.48075654	-0.26351178	-1.77842967

H	-0.90350081	-1.51297106	-1.36327386

Time = 310.00 fs
E(CASSCF/6-31G(d)) = -479.4229869

C	-0.84272199	-0.09470283	0.32435445
C	0.56332356	0.11490675	0.48421266
C	1.69102414	0.95342056	1.17317795
C	1.46520619	-0.39635317	-0.66696364
C	2.73302854	-0.20086301	-0.46974079
C	2.91658432	0.67054917	0.68741482
N	-2.04976309	0.03446798	0.89342781
C	-2.99863245	-0.63214581	-0.03250121
H	3.76764838	1.30748064	0.84100163
C	-1.11554303	-0.48677215	-1.14568090
C	-2.53847174	-0.23988044	-1.41447425
H	-2.70962344	0.81767323	-1.58215974
H	-3.00895844	-0.78581206	-2.23266756
H	-3.09913539	-1.70300211	0.13947214
H	-4.02663026	-0.27982742	0.16945710
H	-0.53214115	0.14408382	-1.80436730
H	1.29679116	1.70054853	1.89476388
C	-2.60304976	1.07913265	1.66532805
H	-2.09331882	1.07385628	2.63172067
H	-2.44949264	2.07338763	1.24160676
H	-3.66246685	1.00849993	1.77138436
H	0.96996287	-1.08814730	-1.30597030
C	3.70962788	-0.95825817	-1.47908117
H	4.25227560	-1.80037136	-1.00984569
H	3.07935348	-1.36457816	-2.27300914
H	4.47512932	-0.27424382	-1.79027486
H	-0.91163326	-1.52292570	-1.35838399

Time = 310.50 fs
E(CASSCF/6-31G(d)) = -479.4214238

C	-0.85072233	-0.08802884	0.32267040
C	0.56851090	0.12516855	0.48455672
C	1.69305774	0.95704285	1.17400404
C	1.46630359	-0.39669436	-0.66800957
C	2.73487787	-0.19952221	-0.46893779
C	2.91964630	0.67110009	0.69112831
N	-2.05156322	0.03193824	0.89433629
C	-2.99433057	-0.63369419	-0.03348561
H	3.78077576	1.31924136	0.84772000
C	-1.11566768	-0.49168040	-1.14816523
C	-2.54024336	-0.23831873	-1.41606593

H	-2.70706813	0.82224818	-1.56601391
H	-3.00971096	-0.77377596	-2.24157628
H	-3.09595042	-1.70969710	0.13542248
H	-4.02427558	-0.28612010	0.16135057
H	-0.53560814	0.12892616	-1.81682522
H	1.28611730	1.70510786	1.90227781
C	-2.60249331	1.07475739	1.66617528
H	-2.10331284	1.05647050	2.63668915
H	-2.44515067	2.06915031	1.25799191
H	-3.66513109	1.01386243	1.76958744
H	0.96603867	-1.07513660	-1.29481160
C	3.70602854	-0.96544506	-1.47976810
H	4.25138971	-1.81505603	-1.02592502
H	3.06958192	-1.36252614	-2.26514443
H	4.46988528	-0.28460621	-1.80398593
H	-0.91923796	-1.53343652	-1.35346330

Time = 311.00 fs
E(CASSCF/6-31G(d)) = -479.4201204

C	-0.85765743	-0.08119943	0.32053917
C	0.57395652	0.13558378	0.48460036
C	1.69420503	0.96066966	1.17526375
C	1.46638854	-0.39714434	-0.66864173
C	2.73739382	-0.19853003	-0.46851095
C	2.92279421	0.67192941	0.69477253
N	-2.05380382	0.02912351	0.89515065
C	-2.99066175	-0.63510618	-0.03428638
H	3.79334842	1.32949383	0.85492654
C	-1.11555331	-0.49659835	-1.15095040
C	-2.54221905	-0.23677064	-1.41749440
H	-2.70499755	0.82696887	-1.54982060
H	-3.00973400	-0.76199609	-2.24988915
H	-3.09089064	-1.71602472	0.13091824
H	-4.02048089	-0.29241513	0.15356571
H	-0.53854533	0.11281975	-1.82908222
H	1.27914103	1.70786908	1.90834435
C	-2.60167952	1.07068384	1.66731552
H	-2.11455487	1.03971143	2.64190171
H	-2.44004310	2.06513732	1.27368685
H	-3.66837147	1.01774526	1.76846041
H	0.96418809	-1.06355732	-1.28581220
C	3.70235805	-0.97249970	-1.47998975
H	4.24923074	-1.82853477	-1.04196005
H	3.06088976	-1.35975061	-2.25763815
H	4.46499578	-0.29462584	-1.81946043
H	-0.92633671	-1.54431247	-1.34843524

Time = 311.50 fs
E(CASSCF/6-31G(d)) = -479.4194402

C	-0.86340432	-0.07421929	0.31794829
C	0.57957551	0.14611534	0.48431992
C	1.69446607	0.96432268	1.17696774
C	1.46554103	-0.39760018	-0.66877122
C	2.74055096	-0.19789600	-0.46845035
C	2.92608201	0.67308254	0.69835260
N	-2.05649061	0.02603046	0.89586206
C	-2.98765250	-0.63643338	-0.03491935
H	3.80454948	1.33758878	0.86243890
C	-1.11523341	-0.50153432	-1.15402339
C	-2.54436154	-0.23523027	-1.41874537
H	-2.70338091	0.83181832	-1.53372503
H	-3.00913153	-0.75049531	-2.25755763
H	-3.08393214	-1.72167330	0.12595842
H	-4.01543682	-0.29864535	0.14627109
H	-0.54076948	0.09590190	-1.84113098
H	1.27608262	1.70865195	1.91274378
C	-2.60066417	1.06689130	1.66878254
H	-2.12691959	1.02361102	2.64744383
H	-2.43401977	2.06163575	1.28848569
H	-3.67194827	1.02011171	1.76788044
H	0.96386964	-1.05426191	-1.27975485
C	3.69864983	-0.97939164	-1.47975121
H	4.24580532	-1.84076356	-1.05776414
H	3.05295105	-1.35648502	-2.25086027
H	4.46036450	-0.30439210	-1.83653022
H	-0.93296484	-1.55533002	-1.34322378

Time = 312.00 fs
E(CASSCF/6-31G(d)) = -479.4197785

C	-0.86786778	-0.06709409	0.31488968
C	0.58528697	0.15672617	0.48369391
C	1.69386482	0.96800779	1.17910758
C	1.46385856	-0.39796795	-0.66832126
C	2.74430616	-0.19762521	-0.46874115
C	2.92953951	0.67458947	0.70187032
N	-2.05961494	0.02266822	0.89646217
C	-2.98531263	-0.63772525	-0.03540419
H	3.81375992	1.34303855	0.87011241
C	-1.11474337	-0.50649830	-1.15736698
C	-2.54663135	-0.23368794	-1.41980385
H	-2.70217809	0.83674368	-1.51787349

H	-3.00802880	-0.73930722	-2.26456386
H	-3.07509352	-1.72639535	0.12055687
H	-4.00945539	-0.30472597	0.13964772
H	-0.54210313	0.07832914	-1.85299208
H	1.27701616	1.70744788	1.91543214
C	-2.59950819	1.06335914	1.67060237
H	-2.14025361	1.00819031	2.65341179
H	-2.42696421	2.05887470	1.30223092
H	-3.67556932	1.02096695	1.76771335
H	0.96456529	-1.04796993	-1.27728161
C	3.69493974	-0.98608238	-1.47906903
H	4.24117596	-1.85177218	-1.07312414
H	3.04542664	-1.35296316	-2.24515102
H	4.45584085	-0.31404438	-1.85496521
H	-0.93916738	-1.56625170	-1.33775833

Time = 312.50 fs
E(CASSCF/6-31G(d)) = -479.4214576

C	-0.87098090	-0.05983079	0.31136038
C	0.59101369	0.16737944	0.48270307
C	1.69244853	0.97171541	1.18165609
C	1.46144471	-0.39817311	-0.66723695
C	2.74860170	-0.19771790	-0.46936339
C	2.93317201	0.67646406	0.70532404
N	-2.06315339	0.01904849	0.89694329
C	-2.98363574	-0.63902708	-0.03576388
H	3.82057104	1.34552781	0.87784276
C	-1.11411917	-0.51149947	-1.16096050
C	-2.54898790	-0.23213072	-1.42065452
H	-2.70134131	0.84166103	-1.50241231
H	-3.00656786	-0.72847408	-2.27091800
H	-3.06443742	-1.73002205	0.11474225
H	-4.00295629	-0.31056043	0.13388223
H	-0.54238835	0.06026532	-1.86470435
H	1.28186840	1.70441697	1.91654089
C	-2.59827318	1.06006902	1.67279254
H	-2.15438318	0.99346053	2.65990196
H	-2.41880112	2.05699832	1.31482322
H	-3.67892547	1.02035703	1.76781930
H	0.96588143	-1.04515438	-1.27878859
C	3.69126192	-0.99252861	-1.47797269
H	4.23545611	-1.86166085	-1.08780738
H	3.03799802	-1.34940089	-2.24078609
H	4.45123928	-0.32375442	-1.87448101
H	-0.94499629	-1.57684510	-1.33197827

Time = 313.00 fs
E(CASSCF/6-31G(d)) = -479.4246081

C	-0.87270421	-0.05243796	0.30736292
C	0.59668142	0.17803868	0.48133075
C	1.69028641	0.97542083	1.18456764
C	1.45839944	-0.39816896	-0.66549358
C	2.75336843	-0.19816923	-0.47029184
C	2.93696130	0.67870391	0.70870857
N	-2.06706828	0.01518574	0.89729882
C	-2.98260069	-0.64037834	-0.03602396
H	3.82478700	1.34491536	0.88556683
C	-1.11339607	-0.51654403	-1.16478081
C	-2.55139039	-0.23054332	-1.42128246
H	-2.70081689	0.84646291	-1.48748703
H	-3.00490156	-0.71804365	-2.27665280
H	-3.05207037	-1.73247118	0.10855692
H	-3.99644392	-0.31604851	0.12915891
H	-0.54150126	0.04186973	-1.87631251
H	1.29041865	1.69988755	1.91637877
C	-2.59701724	1.05700660	1.67536194
H	-2.16912190	0.97942553	2.66699851
H	-2.40950234	2.05604694	1.32622623
H	-3.68172346	1.01836703	1.76805877
H	0.96762795	-1.04595238	-1.28433981
C	3.68764464	-0.99868589	-1.47650557
H	4.22880311	-1.87059306	-1.10156967
H	3.03040112	-1.34598091	-2.23794496
H	4.44636210	-0.33370552	-1.89474998
H	-0.95050770	-1.58689895	-1.32583649

Time = 313.50 fs
E(CASSCF/6-31G(d)) = -479.4290470

C	-0.87302458	-0.04492566	0.30290535
C	0.60221763	0.18866820	0.47956324
C	1.68746653	0.97908466	1.18777962
C	1.45481233	-0.39794098	-0.66310155
C	2.75853009	-0.19896901	-0.47149605
C	2.94086847	0.68129070	0.71201458
N	-2.07130901	0.01109743	0.89752406
C	-2.98217400	-0.64181075	-0.03621125
H	3.82642282	1.34123260	0.89326489
C	-1.11260722	-0.52163307	-1.16880352
C	-2.55379888	-0.22890946	-1.42167390
H	-2.70054799	0.85103152	-1.47324053
H	-3.00318772	-0.70806520	-2.28181829

H	-3.03814098	-1.73375111	0.10205549
H	-3.99047290	-0.32109739	0.12565092
H	-0.53936258	0.02328639	-1.88785758
H	1.30230647	1.69435290	1.91543207
C	-2.59579106	1.05416180	1.67831067
H	-2.18427908	0.96608253	2.67476135
H	-2.39908908	2.05594994	1.33646698
H	-3.68371481	1.01511861	1.76829684
H	0.96985087	-1.05012987	-1.29362812
C	3.68410705	-1.00451247	-1.47472493
H	4.22140604	-1.87878141	-1.11416720
H	3.02245471	-1.34284051	-2.23668359
H	4.44102056	-0.34407319	-1.91541537
H	-0.95575852	-1.59624149	-1.31930165

Time = 314.00 fs
E(CASSCF/6-31G(d)) = -479.4342732

C	-0.87195201	-0.03730584	0.29800049
C	0.60754989	0.19923326	0.47739066
C	1.68409354	0.98265465	1.19121382
C	1.45076366	-0.39750484	-0.66010568
C	2.76400540	-0.20010175	-0.47294076
C	2.94483534	0.68419028	0.71522846
N	-2.07581450	0.00680427	0.89761747
C	-2.98231357	-0.64334733	-0.03635283
H	3.82570474	1.33468002	0.90096220
C	-1.11178228	-0.52676137	-1.17300397
C	-2.55617526	-0.22721308	-1.42181724
H	-2.70047624	0.85524930	-1.45981082
H	-3.00158161	-0.69858476	-2.28647181
H	-3.02283630	-1.73395742	0.09530183
H	-3.98560220	-0.32563510	0.12351048
H	-0.53594877	0.00463542	-1.89936488
H	1.31704337	1.68846068	1.91435632
C	-2.59463536	1.05152842	1.68163091
H	-2.19966618	0.95342552	2.68321501
H	-2.38763213	2.05653273	1.34563018
H	-3.68471731	1.01076675	1.76840798
H	0.97279819	-1.05711550	-1.30600143
C	3.68065729	-1.00997268	-1.47270057
H	4.21347265	-1.88647013	-1.12536639
H	3.01407717	-1.34006858	-2.23692440
H	4.43505529	-0.35500647	-1.93610631
H	-0.96080432	-1.60474877	-1.31235933

Time = 314.50 fs

E(CASSCF/6-31G(d)) = -479.4395191

C	-0.86951718	-0.02959201	0.29266454
C	0.61260228	0.20970003	0.47480818
C	1.68028764	0.98606937	1.19478008
C	1.44633214	-0.39690024	-0.65658030
C	2.76970847	-0.20154721	-0.47458665
C	2.94878457	0.68735376	0.71833209
N	-2.08051557	0.00233016	0.89758154
C	-2.98297326	-0.64500125	-0.03647492
H	3.82306720	1.32562093	0.90873388
C	-1.11094659	-0.53191655	-1.17735751
C	-2.55848418	-0.22543981	-1.42170401
H	-2.70054371	0.85901185	-1.44732856
H	-3.00022981	-0.68964109	-2.29066924
H	-3.00637609	-1.73326589	0.08836588
H	-3.98234135	-0.32962608	0.12285841
H	-0.53129350	-0.01399081	-1.91083754
H	1.33404307	1.68298052	1.91394764
C	-2.59357930	1.04910265	1.68530808
H	-2.21510184	0.94144775	2.69234268
H	-2.37524685	2.05753734	1.35384833
H	-3.68463222	1.00549607	1.76827972
H	0.97684228	-1.06609116	-1.32054520
C	3.67729254	-1.01503991	-1.47051211
H	4.20521121	-1.89390944	-1.13495700
H	3.00528929	-1.33770766	-2.23845819
H	4.42834981	-0.36661521	-1.95645338
H	-0.96569535	-1.61235382	-1.30501252

Time = 315.00 fs
E(CASSCF/6-31G(d)) = -479.4441863

C	-0.86578422	-0.02180023	0.28691705
C	0.61730304	0.22003768	0.47181834
C	1.67619439	0.98926202	1.19837611
C	1.44161960	-0.39618244	-0.65261826
C	2.77553187	-0.20328128	-0.47638955
C	2.95260477	0.69072074	0.72130414
N	-2.08532769	-0.00229572	0.89741609
C	-2.98411088	-0.64677693	-0.03660351
H	3.81915146	1.31456576	0.91672302
C	-1.11012180	-0.53707925	-1.18183866
C	-2.56069406	-0.22357788	-1.42132933
H	-2.70069518	0.86223772	-1.43591676
H	-2.99926490	-0.68126238	-2.29444923
H	-2.98900190	-1.73191466	0.08132758

H	-3.98108935	-0.33308454	0.12377887
H	-0.52547521	-0.03252637	-1.92223558
H	1.35266079	1.67874320	1.91508994
C	-2.59264142	1.04688143	1.68932688
H	-2.23041376	0.93014259	2.70207501
H	-2.36208066	2.05863517	1.36128315
H	-3.68344470	0.99951061	1.76781316
H	0.98237770	-1.07611231	-1.33620279
C	3.67400062	-1.01969813	-1.46824545
H	4.19681662	-1.90133260	-1.14276163
H	2.99620156	-1.33576694	-2.24097002
H	4.42084602	-0.37896116	-1.97610872
H	-0.97046588	-1.61904514	-1.29727783

Time = 315.50 fs
E(CASSCF/6-31G(d)) = -479.4482484

C	-0.86086779	-0.01395038	0.28078444
C	0.62160935	0.23022400	0.46842850
C	1.67198025	0.99216218	1.20188957
C	1.43675875	-0.39541142	-0.64831102
C	2.78133679	-0.20527764	-0.47830040
C	2.95614147	0.69422426	0.72412178
N	-2.09013817	-0.00703739	0.89710582
C	-2.98569643	-0.64867179	-0.03676490
H	3.81478403	1.30214295	0.92515084
C	-1.10932684	-0.54222342	-1.18642192
C	-2.56277788	-0.22161901	-1.42069211
H	-2.70088138	0.86487712	-1.42569027
H	-2.99880165	-0.67346443	-2.29781989
H	-2.97096578	-1.73018091	0.07427976
H	-3.98208226	-0.33608522	0.12631455
H	-0.51861182	-0.05093386	-1.93345235
H	1.37225739	1.67654194	1.91865540
C	-2.59183101	1.04486133	1.69367916
H	-2.24544119	0.91950285	2.71228608
H	-2.34829748	2.05944126	1.36810697
H	-3.68122022	0.99302143	1.76692093
H	0.98974169	-1.08622977	-1.35189635
C	3.67076244	-1.02394216	-1.46598798
H	4.18845500	-1.90893028	-1.14864611
H	2.98698983	-1.33423653	-2.24407249
H	4.41255501	-0.39205561	-1.99476531
H	-0.97512866	-1.62486374	-1.28917359

Time = 316.00 fs
E(CASSCF/6-31G(d)) = -479.4519450

C	-0.85487848	-0.00606446	0.27429504
C	0.62548114	0.24023976	0.46464701
C	1.66777671	0.99471460	1.20522928
C	1.43186121	-0.39464196	-0.64375030
C	2.78701069	-0.20750510	-0.48026981
C	2.95925696	0.69778520	0.72675599
N	-2.09484303	-0.01185743	0.89664701
C	-2.98770547	-0.65067327	-0.03697906
H	3.81091248	1.28907360	0.93425983
C	-1.10857281	-0.54731712	-1.19108652
C	-2.56471320	-0.21955997	-1.41979728
H	-2.70105712	0.86691869	-1.41674539
H	-2.99892769	-0.66624637	-2.30077144
H	-2.95254048	-1.72837124	0.06730209
H	-3.98537621	-0.33876946	0.13045074
H	-0.51089858	-0.06920765	-1.94436887
H	1.39227747	1.67700210	1.92533992
C	-2.59114433	1.04303496	1.69834913
H	-2.26003918	0.90952471	2.72281686
H	-2.33409002	2.05959131	1.37450676
H	-3.67811512	0.98625598	1.76553578
H	0.99915599	-1.09557236	-1.36660276
C	3.66755564	-1.02778081	-1.46382273
H	4.18024457	-1.91682150	-1.15252772
H	2.97786742	-1.33309686	-2.24733580
H	4.40354956	-0.40585173	-2.01215128
H	-0.97970186	-1.62990365	-1.28072837

Time = 316.50 fs
E(CASSCF/6-31G(d)) = -479.4549141

C	-0.84792247	0.00183392	0.26747700
C	0.62887229	0.25006661	0.46048562
C	1.66368962	0.99689327	1.20833776
C	1.42703440	-0.39392098	-0.63902650
C	2.79245803	-0.20992800	-0.48224770
C	2.96182887	0.70131946	0.72917282
N	-2.09934850	-0.01671672	0.89604716
C	-2.99012097	-0.65276129	-0.03725977
H	3.80851765	1.27609648	0.94430582
C	-1.10786410	-0.55232474	-1.19581445
C	-2.56648259	-0.21740296	-1.41865575
H	-2.70118262	0.86839404	-1.40915867
H	-2.99970322	-0.65959253	-2.30327358
H	-2.93401168	-1.72679374	0.06046132
H	-3.99083945	-0.34134043	0.13611620

H	-0.50257980	-0.08735914	-1.95486371
H	1.41230108	1.68044443	1.93552141
C	-2.59056706	1.04138918	1.70331513
H	-2.27407609	0.90020659	2.73348491
H	-2.31966620	2.05876960	1.38066961
H	-3.67436670	0.97945137	1.76361111
H	1.01069749	-1.10340172	-1.37942266
C	3.66435768	-1.03123716	-1.46182396
H	4.17224774	-1.92503710	-1.15437767
H	2.96905546	-1.33233062	-2.25032617
H	4.39396305	-0.42024499	-2.02804075
H	-0.98420264	-1.63430310	-1.27198138

Time = 317.00 fs
E(CASSCF/6-31G(d)) = -479.4571099

C	-0.84011052	0.00971927	0.26035813
C	0.63173836	0.25968872	0.45595961
C	1.65980630	0.99870738	1.21119588
C	1.42239077	-0.39328546	-0.63422230
C	2.79758985	-0.21250737	-0.48418329
C	2.96374997	0.70474795	0.73133766
N	-2.10356596	-0.02157404	0.89531878
C	-2.99293397	-0.65491231	-0.03761547
H	3.80849262	1.26387311	0.95553951
C	-1.10720038	-0.55720910	-1.20058831
C	-2.56807376	-0.21515540	-1.41728387
H	-2.70122360	0.86937612	-1.40298538
H	-3.00116111	-0.65347306	-2.30527136
H	-2.91566964	-1.72572387	0.05381280
H	-3.99816560	-0.34404992	0.14318817
H	-0.49392503	-0.10540667	-1.96481389
H	1.43205792	1.68679867	1.94916387
C	-2.59007742	1.03990443	1.70855329
H	-2.28743304	0.89154616	2.74409221
H	-2.30523425	2.05672145	1.38676960
H	-3.67027129	0.97284484	1.76111772
H	1.02429091	-1.10914638	-1.38962863
C	3.66114835	-1.03434745	-1.46005373
H	4.16447121	-1.93351398	-1.15422147
H	2.96075566	-1.33193314	-2.25264248
H	4.38398848	-0.43507840	-2.04226543
H	-0.98864322	-1.63822963	-1.26297632

Time = 317.50 fs
E(CASSCF/6-31G(d)) = -479.4586595

C	-0.83155818	0.01756430	0.25296539
C	0.63404038	0.26909318	0.45108704
C	1.65619768	1.00020113	1.21382352
C	1.41804629	-0.39276111	-0.62940925
C	2.80232314	-0.21520186	-0.48602593
C	2.96493897	0.70800608	0.73321958
N	-2.10741219	-0.02638691	0.89447776
C	-2.99614098	-0.65710308	-0.03804955
H	3.81149359	1.25288758	0.96816675
C	-1.10657790	-0.56193346	-1.20539035
C	-2.56947947	-0.21282967	-1.41570283
H	-2.70115150	0.86997655	-1.39825892
H	-3.00330917	-0.64784775	-2.30669016
H	-2.89780565	-1.72537721	0.04739882
H	-4.00691358	-0.34717851	0.15149898
H	-0.48520990	-0.12336618	-1.97410794
H	1.45140481	1.69559548	1.96579830
C	-2.58964912	1.03855449	1.71403867
H	-2.30000335	0.88353838	2.75444077
H	-2.29099160	2.05327100	1.39295902
H	-3.66616238	0.96666879	1.75804281
H	1.03971966	-1.11241506	-1.39668815
C	3.65791119	-1.03715920	-1.45855947
H	4.15687129	-1.94209979	-1.15213469
H	2.95312980	-1.33191737	-2.25394191
H	4.37387226	-0.45014937	-2.05471942
H	-0.99303085	-1.64186591	-1.25375848

Time = 318.00 fs
E(CASSCF/6-31G(d)) = -479.4596448

C	-0.82238435	0.02534024	0.24532436
C	0.63574718	0.27826998	0.44588814
C	1.65292154	1.00144661	1.21627298
C	1.41411747	-0.39236152	-0.62464560
C	2.80658170	-0.21796860	-0.48772586
C	2.96535178	0.71105090	0.73479555
N	-2.11081110	-0.03111257	0.89354275
C	-2.99973984	-0.65931378	-0.03856050
H	3.81780844	1.24336852	0.98230479
C	-1.10599053	-0.56646357	-1.21020170
C	-2.57069721	-0.21044238	-1.41393785
H	-2.70094317	0.87033937	-1.39499010
H	-3.00613313	-0.64267026	-2.30744337
H	-2.88070988	-1.72588784	0.04124814
H	-4.01655882	-0.35101221	0.16084630
H	-0.47669999	-0.14124422	-1.98265825

H	1.47027378	1.70604577	1.98459704
C	-2.58925439	1.03730642	1.71974514
H	-2.31169277	0.87617264	2.76434503
H	-2.27711602	2.04833194	1.39936291
H	-3.66238080	0.96114591	1.75438830
H	1.05664140	-1.11300164	-1.40027700
C	3.65463405	-1.03972854	-1.45737248
H	4.14936608	-1.95056897	-1.14823585
H	2.94628561	-1.33231658	-2.25395932
H	4.36390579	-0.46521836	-2.06536055
H	-0.99736958	-1.64539267	-1.24437234

Time = 318.50 fs
E(CASSCF/6-31G(d)) = -479.4600133

C	-0.81270979	0.03301728	0.23745866
C	0.63683681	0.28721210	0.44038496
C	1.65002643	1.00253173	1.21861684
C	1.41071838	-0.39208781	-0.61997488
C	2.81029711	-0.22076392	-0.48923494
C	2.96498782	0.71386459	0.73605411
N	-2.11369509	-0.03570910	0.89253445
C	-3.00372532	-0.66153041	-0.03914249
H	3.82728135	1.23525776	0.99794667
C	-1.10543094	-0.57076980	-1.21500154
C	-2.57172882	-0.20801340	-1.41201711
H	-2.70058006	0.87063089	-1.39316693
H	-3.00960035	-0.63789248	-2.30744140
H	-2.86466960	-1.72729571	0.03537600
H	-4.02654680	-0.35581961	0.17100424
H	-0.46863647	-0.15903252	-1.99041234
H	1.48861707	1.71717525	2.00451153
C	-2.58886712	1.03612108	1.72564543
H	-2.32242003	0.86943032	2.77364512
H	-2.26375733	2.04191418	1.40607496
H	-3.65924386	0.95648607	1.75016929
H	1.07460638	-1.11088612	-1.40028765
C	3.65130910	-1.04211686	-1.45650760
H	4.14185071	-1.95864659	-1.14267737
H	2.94026844	-1.33318414	-2.25251899
H	4.35441335	-0.48001998	-2.07420772
H	-1.00166256	-1.64897078	-1.23486046

Time = 319.00 fs
E(CASSCF/6-31G(d)) = -479.4596188

C	-0.80265607	0.04056487	0.22938994

C	0.63729838	0.29591548	0.43460101
C	1.64755432	1.00354749	1.22093432
C	1.40795809	-0.39192788	-0.61542432
C	2.81340956	-0.22354414	-0.49050740
C	2.96388871	0.71645243	0.73699702
N	-2.11600601	-0.04013654	0.89147486
C	-3.00808551	-0.66374593	-0.03978605
H	3.83932492	1.22823973	1.01494639
C	-1.10489153	-0.57482872	-1.21976658
C	-2.57258002	-0.20556459	-1.40997076
H	-2.70004741	0.87102724	-1.39275562
H	-3.01366415	-0.63346914	-2.30660175
H	-2.84996726	-1.72954651	0.02978581
H	-4.03634237	-0.36183197	0.18173520
H	-0.46122393	-0.17670445	-1.99736163
H	1.50636727	1.72796844	2.02442512
C	-2.58846603	1.03495386	1.73171102
H	-2.33211756	0.86328276	2.78221692
H	-2.25103116	2.03412488	1.41315551
H	-3.65701345	0.95288346	1.74541339
H	1.09307685	-1.10623532	-1.39683553
C	3.64793262	-1.04438704	-1.45596375
H	4.13421413	-1.96603620	-1.13563548
H	2.93505839	-1.33459165	-2.24954064
H	4.34573669	-0.49427627	-2.08133366
H	-1.00591358	-1.65272515	-1.22526377

Time = 319.50 fs
E(CASSCF/6-31G(d)) = -479.4583056

C	-0.79234503	0.04795197	0.22113751
C	0.63713314	0.30437930	0.42856151
C	1.64554131	1.00457747	1.22329975
C	1.40593819	-0.39185597	-0.61100379
C	2.81586886	-0.22626629	-0.49150049
C	2.96213039	0.71883653	0.73763858
N	-2.11769574	-0.04435767	0.89038622
C	-3.01279887	-0.66596030	-0.04047886
H	3.85301488	1.22182047	1.03302996
C	-1.10436550	-0.57862455	-1.22447062
C	-2.57325984	-0.20311822	-1.40782947
H	-2.69933272	0.87169880	-1.39370097
H	-3.01826817	-0.62936150	-2.30485802
H	-2.83687597	-1.73250151	0.02447090
H	-4.04546826	-0.36922820	0.19280129
H	-0.45462238	-0.19421426	-2.00354473
H	1.52341990	1.73748619	2.04328201

C	-2.58803708	1.03375569	1.73791200
H	-2.34073251	0.85768911	2.78998013
H	-2.23901351	2.02516465	1.42063074
H	-3.65586887	0.95051448	1.74015982
H	1.11144519	-1.09940375	-1.39026400
C	3.64450475	-1.04659945	-1.45572538
H	4.12635477	-1.97244801	-1.12729989
H	2.93057143	-1.33662651	-2.24504062
H	4.33821588	-0.50771275	-2.08685261
H	-1.01012871	-1.65673031	-1.21562056

Time = 320.00 fs
E(CASSCF/6-31G(d)) = -479.4559827

C	-0.78189813	0.05514740	0.21271835
C	0.63635573	0.31260592	0.42229339
C	1.64401724	1.00569107	1.22577470
C	1.40475057	-0.39183244	-0.60670507
C	2.81763512	-0.22888888	-0.49217522
C	2.95981043	0.72104767	0.73800299
N	-2.11872657	-0.04833898	0.88929048
C	-3.01783271	-0.66817923	-0.04120662
H	3.86723410	1.21542623	1.05182824
C	-1.10384760	-0.58214964	-1.22908448
C	-2.57378012	-0.20069546	-1.40562349
H	-2.69842466	0.87279496	-1.39592850
H	-3.02335063	-0.62554103	-2.30216870
H	-2.82565380	-1.73595868	0.01941716
H	-4.05353495	-0.37812475	0.20397456
H	-0.44894162	-0.21149741	-2.00904940
H	1.53963386	1.74494088	2.06017394
C	-2.58757522	1.03247438	1.74421680
H	-2.34822763	0.85259557	2.79690335
H	-2.22773832	2.01531868	1.42849434
H	-3.65588710	0.94953562	1.73446028
H	1.12905609	-1.09092896	-1.38114212
C	3.64102949	-1.04880829	-1.45576458
H	4.11819349	-1.97762336	-1.11786412
H	2.92666482	-1.33938793	-2.23912807
H	4.33216679	-0.52007580	-2.09090349
H	-1.01431648	-1.66100266	-1.20596839

Time = 320.50 fs
E(CASSCF/6-31G(d)) = -479.4526945

C	-0.77143638	0.06212020	0.20414743
C	0.63499514	0.32060084	0.41582540

C	1.64300463	1.00694000	1.22840351
C	1.40447492	-0.39180461	-0.60250243
C	2.81867941	-0.23137256	-0.49249693
C	2.95703508	0.72311785	0.73812082
N	-2.11907110	-0.05205107	0.88820834
C	-3.02314269	-0.67041215	-0.04195379
H	3.88082238	1.20849443	1.07091934
C	-1.10333481	-0.58540448	-1.23357620
C	-2.57415502	-0.19831497	-1.40338159
H	-2.69731199	0.87442911	-1.39934615
H	-3.02884785	-0.62199158	-2.29852326
H	-2.81653577	-1.73967815	0.01460589
H	-4.06025948	-0.38857021	0.21504642
H	-0.44423996	-0.22847276	-2.01400951
H	1.55484059	1.74973497	2.07438787
C	-2.58708493	1.03105633	1.75059198
H	-2.35458223	0.84793460	2.80300453
H	-2.21719778	2.00494016	1.43671126
H	-3.65703286	0.95008044	1.72837950
H	1.14523095	-1.08151613	-1.37024837
C	3.63751502	-1.05105833	-1.45604384
H	4.10968321	-1.98135630	-1.10751678
H	2.92314526	-1.34298237	-2.23199753
H	4.32785727	-0.53115025	-2.09363093
H	-1.01848727	-1.66549838	-1.19634534

Time = 321.00 fs
E(CASSCF/6-31G(d)) = -479.4486258

C	-0.76107954	0.06884000	0.19543776
C	0.63309476	0.32837254	0.40918819
C	1.64251780	1.00835729	1.23121142
C	1.40517626	-0.39170941	-0.59835557
C	2.81898432	-0.23368097	-0.49243611
C	2.95390832	0.72507479	0.73802541
N	-2.11871203	-0.05546927	0.88715892
C	-3.02867324	-0.67267004	-0.04270444
H	3.89269776	1.20053733	1.08987042
C	-1.10282666	-0.58839695	-1.23791151
C	-2.57440065	-0.19599172	-1.40113049
H	-2.69598294	0.87666643	-1.40384661
H	-3.03469727	-0.61871098	-2.29394654
H	-2.80972486	-1.74341055	0.01001591
H	-4.06547790	-0.40054380	0.22583515
H	-0.44052705	-0.24504631	-2.01859917
H	1.56885788	1.75147284	2.08542555
C	-2.58657948	1.02944878	1.75700196

H	-2.35979248	0.84362577	2.80835064
H	-2.20734615	1.99442970	1.44522386
H	-3.65916334	0.95225623	1.72199667
H	1.15929964	-1.07200103	-1.35852890
C	3.63397370	-1.05338228	-1.45651906
H	4.10081502	-1.98350692	-1.09643376
H	2.91978282	-1.34751782	-2.22391580
H	4.32548304	-0.54077606	-2.09516376
H	-1.02265240	-1.67011823	-1.18679225

Time = 321.50 fs
E(CASSCF/6-31G(d)) = -479.4440944

C	-0.75094634	0.07527710	0.18660059
C	0.63071262	0.33593252	0.40241464
C	1.64256176	1.00995734	1.23420400
C	1.40690169	-0.39147885	-0.59421566
C	2.81854439	-0.23578141	-0.49196907
C	2.95052471	0.72693888	0.73775001
N	-2.11764160	-0.05857372	0.88615909
C	-3.03435851	-0.67496328	-0.04344298
H	3.90193954	1.19117887	1.10827160
C	-1.10232541	-0.59114091	-1.24205474
C	-2.57453457	-0.19373610	-1.39889423
H	-2.69442470	0.87951542	-1.40930897
H	-3.04083939	-0.61571081	-2.28849975
H	-2.80538223	-1.74692231	0.00562514
H	-4.06914957	-0.41395689	0.23619249
H	-0.43777114	-0.26111654	-2.02302205
H	1.58150157	1.74996092	2.09300990
C	-2.58607888	1.02760244	1.76340887
H	-2.36387230	0.83957683	2.81305220
H	-2.19810719	1.98420641	1.45396063
H	-3.66204328	0.95613975	1.71540520
H	1.17063697	-1.06328258	-1.34701944
C	3.63042203	-1.05579915	-1.45714293
H	4.09162128	-1.98401125	-1.08477179
H	2.91632791	-1.35309729	-2.21520423
H	4.32514755	-0.54886132	-2.09559756
H	-1.02682267	-1.67471760	-1.17735282

Time = 322.00 fs
E(CASSCF/6-31G(d)) = -479.4395005

C	-0.74115442	0.08140277	0.17764598
C	0.62792086	0.34329492	0.39554023
C	1.64313205	1.01173666	1.23736704

C	1.40967668	-0.39104830	-0.59003515
C	2.81736677	-0.23764537	-0.49107846
C	2.94696533	0.72872215	0.73732599
N	-2.11586102	-0.06134933	0.88522301
C	-3.04012369	-0.67729956	-0.04415466
H	3.90783074	1.18016655	1.12575796
C	-1.10183590	-0.59365433	-1.24597001
C	-2.57457556	-0.19155384	-1.39669401
H	-2.69262461	0.88292581	-1.41560067
H	-3.04721924	-0.61301575	-2.28227998
H	-2.80361911	-1.75001717	0.00141292
H	-4.07135667	-0.42865617	0.24600910
H	-0.43590902	-0.27657956	-2.02749928
H	1.59259614	1.74520322	2.09708543
C	-2.58560681	1.02547419	1.76977237
H	-2.36685298	0.83568562	2.81725488
H	-2.18938650	1.97467496	1.46284649
H	-3.66536821	0.96177155	1.70871207
H	1.17870637	-1.05622108	-1.33672638
C	3.62688010	-1.05831378	-1.45786816
H	4.08217583	-1.98288532	-1.07266372
H	2.91253319	-1.35981051	-2.20621570
H	4.32684857	-0.55538901	-2.09498263
H	-1.03100633	-1.67912261	-1.16807566

Time = 322.50 fs
E(CASSCF/6-31G(d)) = -479.4352562

C	-0.73181829	0.08718977	0.16858294
C	0.62480307	0.35047595	0.38860183
C	1.64421515	1.01367461	1.24066682
C	1.41350143	-0.39036738	-0.58577968
C	2.81547125	-0.23924922	-0.48975414
C	2.94329527	0.73042832	0.73678119
N	-2.11337969	-0.06378622	0.88436229
C	-3.04588735	-0.67968281	-0.04482656
H	3.90988379	1.16736997	1.14202830
C	-1.10136489	-0.59595741	-1.24962289
C	-2.57454318	-0.18944594	-1.39454807
H	-2.69056981	0.88679121	-1.42257922
H	-3.05378603	-0.61066033	-2.27541590
H	-2.80448846	-1.75255457	-0.00264079
H	-4.07229841	-0.44443041	0.25521683
H	-0.43486061	-0.29133547	-2.03225150
H	1.60197950	1.73739956	2.09782348
C	-2.58518708	1.02303087	1.77605035
H	-2.36878311	0.83184429	2.82112878

H	-2.18108476	1.96619070	1.47181460
H	-3.66879521	0.96915359	1.70203576
H	1.18310747	-1.05150958	-1.32847564
C	3.62336998	-1.06091736	-1.45865041
H	4.07259108	-1.98022373	-1.06021421
H	2.90817987	-1.36772381	-2.19730489
H	4.33047254	-0.56041858	-2.09331920
H	-1.03520791	-1.68314601	-1.15901203

Time = 323.00 fs
E(CASSCF/6-31G(d)) = -479.4316719

C	-0.72304998	0.09261230	0.15941985
C	0.62145373	0.35749378	0.38163862
C	1.64578818	1.01573371	1.24404977
C	1.41834654	-0.38941009	-0.58144040
C	2.81289128	-0.24057438	-0.48799327
C	2.93956371	0.73205360	0.73613928
N	-2.11021436	-0.06587892	0.88358528
C	-3.05156325	-0.68211150	-0.04544749
H	3.90784146	1.15277232	1.15685446
C	-1.10092063	-0.59807030	-1.25298200
C	-2.57445752	-0.18740967	-1.39247195
H	-2.68824968	0.89095789	-1.43009423
H	-3.06049265	-0.60868673	-2.26806149
H	-2.80797985	-1.75445972	-0.00655418
H	-4.07227609	-0.46101819	0.26379050
H	-0.43454369	-0.30529521	-2.03748315
H	1.60951079	1.72694416	2.09562707
C	-2.58483971	1.02025112	1.78219907
H	-2.36972937	0.82794167	2.82485327
H	-2.17311332	1.95902470	1.48081786
H	-3.67197215	0.97824495	1.69550161
H	1.18361723	-1.04955226	-1.32277060
C	3.61991364	-1.06358919	-1.45945049
H	4.06301359	-1.97619411	-1.04749872
H	2.90310464	-1.37687070	-2.18879952
H	4.33580117	-0.56407922	-2.09056265
H	-1.03942714	-1.68660628	-1.15021496

Time = 323.50 fs
E(CASSCF/6-31G(d)) = -479.4288158

C	-0.71495731	0.09764631	0.15016494
C	0.61797584	0.36436785	0.37469114
C	1.64781939	1.01786027	1.24744245
C	1.42414971	-0.38818164	-0.57704167

C	2.80967464	-0.24160735	-0.48580031
C	2.93580361	0.73358748	0.73541889
N	-2.10638819	-0.06762631	0.88289724
C	-3.05706336	-0.68457796	-0.04600817
H	3.90167814	1.13646013	1.17009127
C	-1.10051192	-0.60001112	-1.25602083
C	-2.57433891	-0.18543988	-1.39047883
H	-2.68565747	0.89523856	-1.43798999
H	-3.06729382	-0.60714051	-2.26038863
H	-2.81401687	-1.75573190	-0.01034411
H	-4.07167281	-0.47811785	0.27174514
H	-0.43488862	-0.31838552	-2.04336456
H	1.61507334	1.71442400	2.09113679
C	-2.58457756	1.01712760	1.78817449
H	-2.36977634	0.82386847	2.82860124
H	-2.16540781	1.95333821	1.48983786
H	-3.67456578	0.98896114	1.68923757
H	1.18022069	-1.05038253	-1.31969482
C	3.61653041	-1.06629954	-1.46023587
H	4.05361727	-1.97102711	-1.03456401
H	2.89722435	-1.38724220	-2.18097171
H	4.34252730	-0.56655716	-2.08663670
H	-1.04365787	-1.68934408	-1.14173755

Time = 324.00 fs
E(CASSCF/6-31G(d)) = -479.4264367

C	-0.70764195	0.10227006	0.14082698
C	0.61447727	0.37111786	0.36780020
C	1.65026927	1.01998581	1.25075249
C	1.43081431	-0.38671906	-0.57264178
C	2.80588404	-0.24233980	-0.48318707
C	2.93203157	0.73501368	0.73463298
N	-2.10193003	-0.06903133	0.88230023
C	-3.06230126	-0.68706780	-0.04650101
H	3.89160012	1.11861241	1.18168555
C	-1.10014731	-0.60179459	-1.25871935
C	-2.57420775	-0.18353041	-1.38858003
H	-2.68279239	0.89942857	-1.44610780
H	-3.07414401	-0.60606621	-2.25257776
H	-2.82245668	-1.75644606	-0.01402609
H	-4.07092256	-0.49539932	0.27912967
H	-0.43585117	-0.33055276	-2.05001689
H	1.61857693	1.70060846	2.08522657
C	-2.58440372	1.01366768	1.79393367
H	-2.36902608	0.81952180	2.83252204
H	-2.15793749	1.94916736	1.49888967

H	-3.67628539	1.00117465	1.68336872
H	1.17312275	-1.05365461	-1.31890082
C	3.61323442	-1.06901305	-1.46098098
H	4.04459379	-1.96500066	-1.02143144
H	2.89055553	-1.39877867	-2.17401432
H	4.35027786	-0.56807882	-2.08145044
H	-1.04788809	-1.69123676	-1.13363061

Time = 324.50 fs
E(CASSCF/6-31G(d)) = -479.4240119

C	-0.70119757	0.10646481	0.13141586
C	0.61106775	0.37776247	0.36100601
C	1.65309098	1.02202992	1.25387117
C	1.43820958	-0.38508533	-0.56832618
C	2.80159780	-0.24276836	-0.48017232
C	2.92824790	0.73631120	0.73378790
N	-2.09687338	-0.07010068	0.88179309
C	-3.06719582	-0.68955973	-0.04691951
H	3.87804724	1.09948928	1.19168688
C	-1.09983439	-0.60343076	-1.26106534
C	-2.57408433	-0.18167578	-1.38678549
H	-2.67966096	0.90332235	-1.45428916
H	-3.08099368	-0.60550260	-2.24480699
H	-2.83309306	-1.75674935	-0.01761544
H	-4.07047056	-0.51251754	0.28601579
H	-0.43742160	-0.34176505	-2.05749928
H	1.61995757	1.68641906	2.07897617
C	-2.58430987	1.00989289	1.79943681
H	-2.36759775	0.81480953	2.83672590
H	-2.15070822	1.94642236	1.50802148
H	-3.67690129	1.01471806	1.67801164
H	1.16273439	-1.05871152	-1.31968868
C	3.61003297	-1.07169250	-1.46166663
H	4.03614066	-1.95842017	-1.00810313
H	2.88322276	-1.41136522	-2.16802696
H	4.35864098	-0.56889177	-2.07491835
H	-1.05210026	-1.69220833	-1.12594030

Time = 325.00 fs
E(CASSCF/6-31G(d)) = -479.4209407

C	-0.69570656	0.11021479	0.12194403
C	0.60785381	0.38431819	0.35434607
C	1.65623155	1.02390642	1.25668062
C	1.44617341	-0.38335881	-0.56419564
C	2.79691028	-0.24289392	-0.47678120

C	2.92443718	0.73745426	0.73288252
N	-2.09125603	-0.07084392	0.88137139
C	-3.07167531	-0.69202563	-0.04725711
H	3.86168965	1.07942585	1.20025433
C	-1.09957956	-0.60492449	-1.26305515
C	-2.57398867	-0.17987276	-1.38510418
H	-2.67627948	0.90673153	-1.46237873
H	-3.08778789	-0.60547906	-2.23724490
H	-2.84566297	-1.75685240	-0.02112572
H	-4.07072666	-0.52912498	0.29248455
H	-0.43962623	-0.35201232	-2.06580524
H	1.61917846	1.67286560	2.07359972
C	-2.58427572	1.00583679	1.80464887
H	-2.36562608	0.80965317	2.84127061
H	-2.14375968	1.94490125	1.51730931
H	-3.67625626	1.02938727	1.67326895
H	1.14963695	-1.06470799	-1.32114627
C	3.60692572	-1.07430251	-1.46227785
H	4.02844853	-1.95159571	-0.99457212
H	2.87545357	-1.42483256	-2.16301487
H	4.36719453	-0.56924551	-2.06697752
H	-1.05627160	-1.69223851	-1.11870592

Time = 325.50 fs
E(CASSCF/6-31G(d)) = -479.4167923

C	-0.69123764	0.11350830	0.11242688
C	0.60493562	0.39079812	0.34785410
C	1.65963263	1.02553229	1.25906415
C	1.45451654	-0.38162208	-0.56035319
C	2.79193124	-0.24272162	-0.47304459
C	2.92056871	0.73841424	0.73190786
N	-2.08511879	-0.07127339	0.88102752
C	-3.07568136	-0.69443180	-0.04750615
H	3.84341920	1.05881395	1.20766578
C	-1.09938800	-0.60627543	-1.26469394
C	-2.57394038	-0.17812174	-1.38354470
H	-2.67267402	0.90949796	-1.47022789
H	-3.09446279	-0.60601098	-2.23003957
H	-2.85985612	-1.75701380	-0.02457098
H	-4.07201895	-0.54488443	0.29860838
H	-0.44252528	-0.36130379	-2.07486082
H	1.61622932	1.66094117	2.07032102
C	-2.58426967	1.00154227	1.80954176
H	-2.36326014	0.80399130	2.84615481
H	-2.13715522	1.94431463	1.52684805
H	-3.67427387	1.04494940	1.66922491

H	1.13453059	-1.07074113	-1.32230124
C	3.60390522	-1.07681274	-1.46280137
H	4.02168745	-1.94481698	-0.98083396
H	2.86756170	-1.43896355	-2.15890048
H	4.37553374	-0.56937503	-2.05760290
H	-1.06037513	-1.69136436	-1.11195818

Time = 326.00 fs
E(CASSCF/6-31G(d)) = -479.4114941

C	-0.68784358	0.11633839	0.10288368
C	0.60240326	0.39721096	0.34155888
C	1.66323052	1.02683981	1.26092179
C	1.46302851	-0.37995237	-0.55689362
C	2.78678477	-0.24226008	-0.46899810
C	2.91659969	0.73916104	0.73084707
N	-2.07850487	-0.07140379	0.88075070
C	-3.07917197	-0.69674061	-0.04765678
H	3.82431654	1.03808205	1.21431291
C	-1.09926409	-0.60747833	-1.26599531
C	-2.57395840	-0.17642786	-1.38211543
H	-2.66888124	0.91150628	-1.47769827
H	-3.10094458	-0.60709736	-2.22331298
H	-2.87532838	-1.75751733	-0.02796502
H	-4.07455268	-0.55947822	0.30443585
H	-0.44620408	-0.36966464	-2.08453165
H	1.61113054	1.65148054	2.07019575
C	-2.58425005	0.99705820	1.81409569
H	-2.36066065	0.79777977	2.85131716
H	-2.13096858	1.94431803	1.53674005
H	-3.67096102	1.06114936	1.66594115
H	1.11817834	-1.07596378	-1.32225059
C	3.60095869	-1.07920046	-1.46322244
H	4.01599359	-1.93833012	-0.96689940
H	2.85991916	-1.45350379	-2.15554161
H	4.38329376	-0.56948609	-2.04681860
H	-1.06438087	-1.68968051	-1.10571844

Time = 326.50 fs
E(CASSCF/6-31G(d)) = -479.4053744

C	-0.68555861	0.11870320	0.09333856
C	0.60033258	0.40356061	0.33548320
C	1.66695588	1.02778907	1.26218721
C	1.47148492	-0.37841508	-0.55389636
C	2.78160639	-0.24152108	-0.46468116
C	2.91248058	0.73966540	0.72967713

N	-2.07145909	-0.07125142	0.88052702
C	-3.08212305	-0.69891311	-0.04769611
H	3.80558900	1.01766578	1.22067982
C	-1.09921206	-0.60852395	-1.26698035
C	-2.57406087	-0.17480161	-1.38082465
H	-2.66494792	0.91269196	-1.48466460
H	-3.10714955	-0.60871933	-2.21715626
H	-2.89171671	-1.75864225	-0.03132141
H	-4.07838225	-0.57261539	0.30997950
H	-0.45076054	-0.37713187	-2.09463363
H	1.60394258	1.64501457	2.07391740
C	-2.58416728	0.99243583	1.81829998
H	-2.35799637	0.79099109	2.85664070
H	-2.12526913	1.94454420	1.54708409
H	-3.66640391	1.07771790	1.66345395
H	1.10135022	-1.07966223	-1.32024971
C	3.59807027	-1.08145225	-1.46352176
H	4.01146023	-1.93231851	-0.95280716
H	2.85292339	-1.46817755	-2.15275833
H	4.39016938	-0.56974435	-2.03470367
H	-1.06825689	-1.68733356	-1.09999772

Time = 327.00 fs
E(CASSCF/6-31G(d)) = -479.3990951

C	-0.68439522	0.12060716	0.08382187
C	0.59877951	0.40984539	0.32964313
C	1.67073093	1.02837711	1.26284204
C	1.47965248	-0.37706057	-0.55142357
C	2.77654283	-0.24051864	-0.46013549
C	2.90816692	0.73990161	0.72837137
N	-2.06402847	-0.07083411	0.88034053
C	-3.08452774	-0.70091173	-0.04760818
H	3.78846707	0.99796938	1.22729620
C	-1.09923679	-0.60940068	-1.26767636
C	-2.57426491	-0.17325927	-1.37968022
H	-2.66093053	0.91304672	-1.49101694
H	-3.11298448	-0.61083952	-2.21163017
H	-2.90865685	-1.76063026	-0.03465176
H	-4.08340322	-0.58403490	0.31520985
H	-0.45628900	-0.38374835	-2.10495148
H	1.59478316	1.64166358	2.08166218
C	-2.58396595	0.98772566	1.82215210
H	-2.35543879	0.78361227	2.86196495
H	-2.12011018	1.94463691	1.55796550
H	-3.66076180	1.09437969	1.66177337
H	1.08477977	-1.08129861	-1.31575719

C	3.59522358	-1.08356456	-1.46367288
H	4.00812862	-1.92689175	-0.93863317
H	2.84696331	-1.48270161	-2.15035649
H	4.39592753	-0.57026764	-2.02139364
H	-1.07197102	-1.68451481	-1.09479888

Time = 327.50 fs
E(CASSCF/6-31G(d)) = -479.3943573

C	-0.68428475	0.12204577	0.07437258
C	0.59774341	0.41609291	0.32398300
C	1.67430394	1.02865854	1.26300420
C	1.48706675	-0.37593213	-0.54954631
C	2.77198533	-0.23924082	-0.45534190
C	2.90381104	0.73981850	0.72681381
N	-2.05627835	-0.07020716	0.88023795
C	-3.08637114	-0.70268640	-0.04738615
H	3.77401428	0.97918839	1.23464030
C	-1.09933549	-0.61009868	-1.26812451
C	-2.57460386	-0.17182777	-1.37868325
H	-2.65688231	0.91263462	-1.49667068
H	-3.11829965	-0.61339566	-2.20686609
H	-2.92593535	-1.76367172	-0.03799596
H	-4.08941864	-0.59351468	0.32002337
H	-0.46289839	-0.38958971	-2.11538394
H	1.58384044	1.64110385	2.09302011
C	-2.58357411	0.98296566	1.82562775
H	-2.35317973	0.77563763	2.86715140
H	-2.11560743	1.94442861	1.56955002
H	-3.65428786	1.11099697	1.66090955
H	1.06907771	-1.08053183	-1.30832450
C	3.59239216	-1.08553610	-1.46365208
H	4.00591561	-1.92205796	-0.92440073
H	2.84238586	-1.49683314	-2.14814571
H	4.40042030	-0.57112827	-2.00700052
H	-1.07554050	-1.68140530	-1.09006980

Time = 328.00 fs
E(CASSCF/6-31G(d)) = -479.3950139

C	-0.68536453	0.12299766	0.06514529
C	0.59737208	0.42226059	0.31852223
C	1.67755333	1.02871186	1.26273898
C	1.49343186	-0.37507481	-0.54825848
C	2.76808548	-0.23771464	-0.45036915
C	2.89942982	0.73940212	0.72500512
N	-2.04822875	-0.06942305	0.88015764

C	-3.08764564	-0.70418296	-0.04701682
H	3.76316648	0.96165429	1.24307913
C	-1.09952053	-0.61057035	-1.26838208
C	-2.57510470	-0.17055248	-1.37784625
H	-2.65290978	0.91155996	-1.50151346
H	-3.12288707	-0.61629370	-2.20295754
H	-2.94313812	-1.76784525	-0.04134605
H	-4.09602119	-0.60078387	0.32425132
H	-0.47056466	-0.39469112	-2.12558014
H	1.57143067	1.64275313	2.10698310
C	-2.58291746	0.97823651	1.82872688
H	-2.35145562	0.76715609	2.87196426
H	-2.11185521	1.94369232	1.58185276
H	-3.64726813	1.12718842	1.66078566
H	1.05490272	-1.07720551	-1.29794792
C	3.58958326	-1.08735754	-1.46342219
H	4.00475714	-1.91780645	-0.91034136
H	2.83950623	-1.51023103	-2.14598471
H	4.40355674	-0.57234801	-1.99188486
H	-1.07884357	-1.67824066	-1.08565582

Time = 328.50 fs
E(CASSCF/6-31G(d)) = -479.3931931

C	-0.68759302	0.12351330	0.05612951
C	0.59764534	0.42835312	0.31325773
C	1.68047151	1.02861309	1.26211530
C	1.49870313	-0.37447958	-0.54754990
C	2.76485071	-0.23596801	-0.44526439
C	2.89495881	0.73867223	0.72298081
N	-2.03988589	-0.06848468	0.88005718
C	-3.08840179	-0.70540311	-0.04647168
H	3.75632494	0.94541476	1.25285283
C	-1.09980682	-0.61081126	-1.26848084
C	-2.57577296	-0.16945329	-1.37717164
H	-2.64906933	0.90995670	-1.50552195
H	-3.12670640	-0.61947485	-2.19981514
H	-2.96000950	-1.77310956	-0.04470589
H	-4.10277854	-0.60572901	0.32781356
H	-0.47932104	-0.39910908	-2.13533810
H	1.55783965	1.64574391	2.12246405
C	-2.58194657	0.97354834	1.83149572
H	-2.35038863	0.75813125	2.87622128
H	-2.10872576	1.94216851	1.59490148
H	-3.63993450	1.14274413	1.66133074
H	1.04259553	-1.07137754	-1.28476533
C	3.58677927	-1.08906416	-1.46292442

H	4.00456844	-1.91402441	-0.89660521
H	2.83850526	-1.52274129	-2.14374363
H	4.40537978	-0.57388809	-1.97627770
H	-1.08185785	-1.67526174	-1.08156154

Time = 329.00 fs
E(CASSCF/6-31G(d)) = -479.3914726

C	-0.69088943	0.12363759	0.04737022
C	0.59852504	0.43434566	0.30819343
C	1.68298609	1.02844948	1.26123495
C	1.50276850	-0.37412566	-0.54740347
C	2.76234940	-0.23403317	-0.44008577
C	2.89044210	0.73765139	0.72078527
N	-2.03130311	-0.06740874	0.87990999
C	-3.08868049	-0.70634711	-0.04572875
H	3.75348434	0.93046472	1.26389676
C	-1.10021004	-0.61081694	-1.26845381
C	-2.57661401	-0.16856119	-1.37666342
H	-2.64543909	0.90800203	-1.50866693
H	-3.12968796	-0.62285723	-2.19738158
H	-2.97627406	-1.77928338	-0.04806026
H	-4.10920339	-0.60822474	0.33059680
H	-0.48912316	-0.40286980	-2.14445628
H	1.54346820	1.64914771	2.13817845
C	-2.58061688	0.96892830	1.83395932
H	-2.35009766	0.74856791	2.87974859
H	-2.10609237	1.93971562	1.60865591
H	-3.63256130	1.15741184	1.66246286
H	1.03241083	-1.06331181	-1.26915724
C	3.58398024	-1.09068189	-1.46209635
H	4.00522607	-1.91058253	-0.88341706
H	2.83949039	-1.53418551	-2.14135095
H	4.40599275	-0.57566111	-1.96050521
H	-1.08455665	-1.67269040	-1.07779919

Time = 329.50 fs
E(CASSCF/6-31G(d)) = -479.3900896

C	-0.69514820	0.12342008	0.03891778
C	0.59995436	0.44021153	0.30332485
C	1.68501276	1.02830539	1.26020260
C	1.50553930	-0.37397322	-0.54778349
C	2.76063899	-0.23194465	-0.43488842
C	2.88597410	0.73636802	0.71847957
N	-2.02253734	-0.06621470	0.87969437
C	-3.08852229	-0.70702426	-0.04477047

[truncated: 190,940 more chars]
